# The impact of OsASR5 on rice microbiome: Implications for nutrient cycling and stress response

**DOI:** 10.1590/1678-4685-GMB-2025-0064

**Published:** 2026-10-26

**Authors:** Isabel Cristina Cadavid Sánchez, Marcia Margis-Pinheiro, Mateus Dias-Oliveira, Lucas Roani Ponte, Felipe Klein Ricachenevsky, Rogerio Margis

**Affiliations:** 1 Universidade Federal do Rio Grande do Sul, Instituto de Biociências, Departamento de Genética, Porto Alegre, RS, Brazil; 2 Universidade Federal do Rio Grande do Sul, Centro de Biotecnologia, Programa de Pós-Graduação em Biologia Celular e Molecular, Porto Alegre, RS, Brazil; 3 Universidade Federal do Rio Grande do Sul, Instituto de Biociências, Departamento de Botânica, Porto Alegre, RS, Brazil; 4 Universidade Federal do Rio Grande do Sul, Departamento de Biofísica, Porto Alegre, RS, Brazil

**Keywords:** Bacterium, microbiome, OsASR5, phyllosphere, rhizosphere

## Abstract

Plant-microorganism interactions contribute to plant health by enhancing nutrient acquisition. The transcription factor OsASR5 regulates detoxification gene expression under aluminum stress in rice and is implicated as a positive regulator of responses to iron and phosphorus deficiency. Given its role in nutrient regulation, we investigated whether OsASR5 influences the assembly of the rice microbiome. In this study, we characterized the bacteria communities associated with the leaves and roots of OsASR5-RNAi rice plants compared to wild-type *Oryza sativa* cv. Nipponbare. Notably, OsASR5-RNAi plants showed altered bacterial beta diversity compared with wild type. The bacterial composition of OsASR5-RNAi plants differed in both organs, with decreased Desulfobacteriota and increased in Acidobacteria abundance in leaves. At the genus level, *Methylobacterium*, a known plant growth promoter, was less prevalent in leaves, while *Magnetospirillum*, associated with iron cycling, was less abundant in roots. Conversely, several bacteria were more abundant in OsASR5-RNAi plants, including *Aneurinibacillus*, *Anaeromyxobacter*, and *Methylobacter*, which are associated with nitrogen cycling and phosphorus solubilization. Additionally, *Geobacter* and *Geothrix*, both known for their role in metal reduction, particularly iron, were more abundant in OsASR5-RNAi plants. These suggest that OsASR5 affects plant-microbe interactions, potentially influencing nutrient cycling and plant stress responses through microbiome modulation.

## Introduction

Plants establish beneficial associations with microorganisms that play a central role in nutrient acquisition and overall plant performance. By secretion of secondary metabolites and organic compounds from both roots and aerial tissues, plants selectively recruit microbial communities that enhance access to essential nutrients such as nitrogen (N), phosphorus (P), and iron (Fe) (Parasar et al., 2024). In the rhizosphere, bacteria directly contribute to plant nutrition by fixing atmospheric nitrogen, solubilizing inorganic phosphate, and producing siderophores that increase iron bioavailability. These processes improve nutrient uptake under limiting soil conditions and are widely recognized as key mechanisms of plant growth promotion (Timofeeva et al., 2023). 

However, nutrient acquisition is not exclusively mediated by root-associated microbes. Increasing evidence shows that leaf-associated microbial communities also influence plant nutrient status. Phyllosphere bacteria participate in nitrogen cycling through atmospheric nitrogen fixation and nitrate metabolism, contribute to phosphorus mobilization, and produce siderophores that can enhance iron redistribution within plant tissues (Ruinen, 1965; Abadi et al., 2020; Kumar et al., 2023; Zhu et al., 2023). In addition, both root and leaf microbiota synthesize phytohormones that regulate root architecture, stomatal behavior, and nutrient transport pathways, thereby indirectly modulating nutrient uptake and allocation (Patel and Chaudhari, 2026). Given the beneficial effects of both rhizosphere and phyllosphere microorganisms, they serve as a source of bioinoculants to enhance soil fertility and contribute to the biocontrol of pathogens and pests, thereby exerting a significant economic impact on agriculture (dos Reis et al., 2024; Arora and Mishra, 2024).

Rice (*Oryza sativa* L.) is a critical crop worldwide. Brazil ranks as the leading producer outside Asia, with an estimated production of 11 million tons of grain in 2024 (CONAB, 2024). Rice production is often affected by nutrient imbalances, which result from various agronomic practices and soil degradation (Jin et al., 2022). Understanding the mechanisms plants use to maintain nutrient homeostasis is crucial for developing solutions to these challenges. Specifically, investigating bacterial recruitment mechanisms can provide valuable insights into plant-microbe interactions and offer potential sources for bioinoculants that could improve crop health and sustainability.

Proteins from the ABA, stress, and ripening (ASR) family are involved in plant adaptive responses to environmental stress and developmental processes (Hu et al., 2013; Nunes, 2018; Zhang et al., 2024). Their molecular functions include molecular chaperone activity and transcriptional regulation of stress-responsive pathways. In rice (*Oryza sativa* L.), six ASR family members have been identified, among which OsASR5 has emerged as a key transcription factor associated with stress responses (Philippe et al., 2010; Arenhart et al., 2014). OsASR5 is best characterized for its role in aluminium stress tolerance (Arenhart *et al*., 2012, 2013, 2014), where it regulates detoxification pathways that mitigate the detrimental effects of Al toxicity on root function. One such effect is the disruption of nutrient uptake, leading to deficiencies in essential elements such as iron (Fe), phosphorus (P), calcium (Ca), and nitrogen (N) (Ur Rahman et al., 2024). Rice manages aluminium stress in part through the efflux of organic acids (OAs) into the rhizosphere, preventing the entry of toxic Al³⁺ ions into root cells, a process that is under OsASR5 regulation. Beyond its role in aluminium detoxification, OsASR5 has also been implicated in the regulation of nutrient homeostasis, acting as a positive regulator of plant responses to iron deficiency (Ponte et al., 2025) and phosphorus starvation (Barros et al., 2025). Therefore, given its role in nutrient regulation, OsASR5 might act as a molecular integrator linking environmental nutrient availability to plant responses. By regulating pathways involved in iron and phosphorus homeostasis, OsASR5 may provide a mechanistic link between microbiome-driven nutrient dynamics and plant stress tolerance. While previous studies have established the role of OsASR5 in aluminum detoxification and nutrient homeostasis, it remains unknown whether this transcription factor also influences the assembly of plant-associated microbial communities. This represents a critical knowledge gap, as nutrient availability is a major driver of microbiome composition, and reciprocal interactions between host regulatory pathways and microbial communities may ultimately shape plant resilience under stress conditions.

Therefore, we hypothesized that OsASR5-mediated nutrient homeostasis, even under non-stress conditions, contributes to shaping plant-associated microbial community assembly in roots and leaves. To address this question, we characterized bacterial communities associated with leaves and roots of OsASR5-RNAi silenced rice plants and wild-type *Oryza sativa* cv. Nipponbare, under basal conditions, using 16S rRNA gene amplification and Illumina sequencing.

## Material and Methods

### Plant sample collection

The comparison of the microbiomes of ASR5-silenced plants and wild-type *Oryza sativa cv. Nipponbare* plants were performed under standard conditions. ASR-silenced rice plants were generated as described by Arenhart et al. (2013) via *Agrobacterium tumefaciens*-mediated transformation of embryogenic calli (cv. Nipponbare) using an RNAi construct targeting a conserved ASR coding region. This approach induced post-transcriptional gene silencing and resulted in reduced expression of ASR transcripts, including ASR5. (Arenhart *et al*., 2013). For microbiome analysis, seeds were germinated in Petri dishes and after 7 days transferred to vermiculite and irrigated every two to three days with nutrient solution as described (Ricachenevsky et al., 2011). At the three-leaf stage, plants were transferred to soil. We used soil derived from an experimental rice field located at IRGA (Instituto Rio Grandense do Arroz; Rio Grandense Rice Institute; 29º 56’ 41.546’’ S; 51º 7’ 17.147’’ W). Plants were maintained in greenhouse conditions until sample harvest, which occurred when plants were 60 days old. Six individual plants per genotype were sampled in November 2023. Roots and leaves were collected and processed separately for each plant (no bulk sampling). The entire root system was harvested, while leaf material corresponding to the volume fitting into a 50 mL Falcon tube was collected from each individual plant.

### Collection of microbiome community 

Leaf and root material from each individual plant was transported to the laboratory in sterile plastic tubes and soil was removed from the roots prior to processing. To isolate the total phyllosphere and rhizosphere microbiome, tissues were cut into small pieces (~ 0.5 mm * 0.5 mm) using sterile tweezers and a scalpel on a sterilized glass Petri dish under laminar flow. Samples were transferred to an Erlenmeyer flask containing 50 ml of sterilized and pre-chilled PBST buffer (Dong et al., 2019) solution. Tissue samples were shaken for 2 h at 180 rpm at 4 °C to release microbial cells. Subsequently, 40 ml of suspension was placed into Falcon tubes and centrifuged for 30 min at 3000 rpm. The resulting pellets were stored frozen until DNA extraction. 

### DNA isolation and sequencing

Total DNA was isolated by using ZymoBIOMICS DNA MiniPrep™kits (Zymo Research Corporation). DNA quality was evaluated by electrophoresis agarose gel and NanoDrop^®^. The V3-V4 region of 16S rDNA was amplified using specific primer pairs (341F 5’CCTAYGGGRBGCASCAG and 806R 5’GGACTACNNGGGTATCTAAT) (Wang and Qian, 2009; Caporaso et al., 2011). The PCR was carried out according to Christoff *et al*. (2017) (Christoff *et al*., 2017). The PCR products were analyzed on 1.5 % agarose gel. Samples with a bright band detected between 400 and 450 bp were chosen for sequencing. Amplicon library preparation, including indexing and pooling of PCR products, was performed by Neoprospecta (Florianópolis, Brazil). Sequencing was carried out on an Illumina MiSeq platform as part of a complete NGS workflow provided by the company.

### Sequence analysis

Raw sequences were evaluated for quality with FastQC and filtered by removing reads with low quality (Q<30) and less than 200 nt, using Trim Galore (Edgar, 2010). Bowtie2 mapping analysis was done to filter chloroplastic (cp) and mitochondrial (mt) reads by comparison against cp16S rRNA and mt18S rRNA genes from *Oryza sativa* and finally, these reads were removed from the dataset. DADA2 was used to assess the quality of the reads, which included filtering, trimming, denoising, and removing chimeras (Callahan et al., 2016). Reads were clustered into Operational Taxonomic Units (OTU) using a 97% sequence similarity using *q2-vsearch* plugin and *cluster-features-de-novo* command in QIIME2 (Caporaso et al., 2011). 

Multiple sequence alignment of the representative sequences for each OTU was aligned using the *mafft* program in QIIME2 and *FastTree* program created an unrooted and rooted tree to calculate phylogenetic diversity metrics.

Taxonomic classification of the representative sequence for each OTU was made using QIIME2 pipeline classifier against Silva 16S rRNA database (Quast et al., 2013) with a minimum confidence of 0.8 to record an assignment. An OTU table was generated describing the occurrence of bacterial phylotypes within the samples. *Taxa barplot* were produced by the *ggplot2* package in R, at phylum, family and genus levels.

Amplicons of plant-based origin that were identified as chloroplasts (where taxa = Cyanobacteria/Chloroplast) and mitochondria (where taxa = Proteobacteria/Mitochondria) were discarded from further analysis using Qiime *taxa filter-table* option (Taxonomy-based feature table filter). Twenty-four libraries belonging to two genotypes were obtained. After applying all quality analyses and the exclusion of chloroplast and mitochondrial reads, a total of 213 to 2003 and 2184 to 79558 of sequencing depth for leaf and root, respectively, per sample was obtained. Detailed information on sequence and OTUs numbers obtained for each plant are reported in Table S1. These sequence data have been submitted to the GenBank databases under BioProject accession No.PRJNA1240251

### Statistical analyses

The OTUs relative abundance was calculated. Samples were rarefied to the least sequences per sample. After that, various diversity indices and statistics were estimated using q2-diversity (QIIME2 plugin).

Richness and Alpha-diversity were calculated with Observed features, Shannon and Faith’s Phylogenetic diversity (PD) indexes. Beta-diversity was calculated using Bray Curtis and Jaccard. Indexes were measured in different plant genus using OTU. Principal Coordinates Analysis (PCoA) based on indexes was visualized in Emperor (http://emperor.colorado.edu/). Pairwise Kruskal-Wallis test was used for assessing statistical significance of alpha diversity between several groups. Bray Curtis and Jaccard distance metrics were subjected to permutational multivariate analysis of variance (PERMANOVA) to assess statistical significance of diversity between several groups with a permutation number of 999. Frequency tables were collapsed to the sixth taxonomic level (genus) for subsequent analysis (Venn diagram). Besides, the presence of bacteria genus in plants were represented in Venn diagrams to display the shared and unique microbiota using the online platform *Interactivenn* (http://www.interactivenn.net/). 

## Results

### Library sequencing depth and quality

After quality filtering and chimera removal, 1,242,603 high-quality reads were retained across all samples. Sequencing depth ranged from 21,310 to 118,126 reads per sample, with an average of approximately 51,800 reads per sample after filtering (Table 1). Detailed sequencing metrics for each sample are provided in Table S1.

**Table 1 -:** Summary of sequencing depth and reads retained after quality filtering according to plant genotype and compartment. Values represent mean ± standard deviation, with the range of raw sequencing reads indicated.

Compartment	Genotype	n	Sequencing depth (mean ± SD)	Range	Reads after filtering (mean ± SD)
F	ASR5i	6	72,642 ± 31,817	21,310-104,564	56,865 ± 27,437
F	WT	6	50,231 ± 19,137	26,835-73,032	41,076 ± 14,578
R	ASR5i	6	59,291 ± 28,776	29,379-113,760	45,778 ± 22,171
R	WT	6	86,149 ± 26,752	42,993-118,126	61,482 ± 15,657

### Alpha diversity of leaf and root bacterial communities

Leaf and root bacteria communities consisted of 416 and 19234 OTUs, respectively, and this data was used to assess diversity (Table S2 and S3). Among the plant organs, the root microbiome was the most diverse in terms of alpha and beta diversity (Figure 1 and 2). A genotype comparison was made for each organ. In leaves, alpha diversity of *ASR5-RNAi* plants was significantly higher compared to wild type (Figure 1 A , p-value < 0,037), while in roots, there was no significant difference between genotypes (Figure 1 B ). To ensure that the amount of sequence data generated did not affect the diversity inferences, dilution curves were constructed. The alpha rarefaction analysis confirmed that sequencing depth did not affect the diversity inferences. 

**Figure 1 -:**
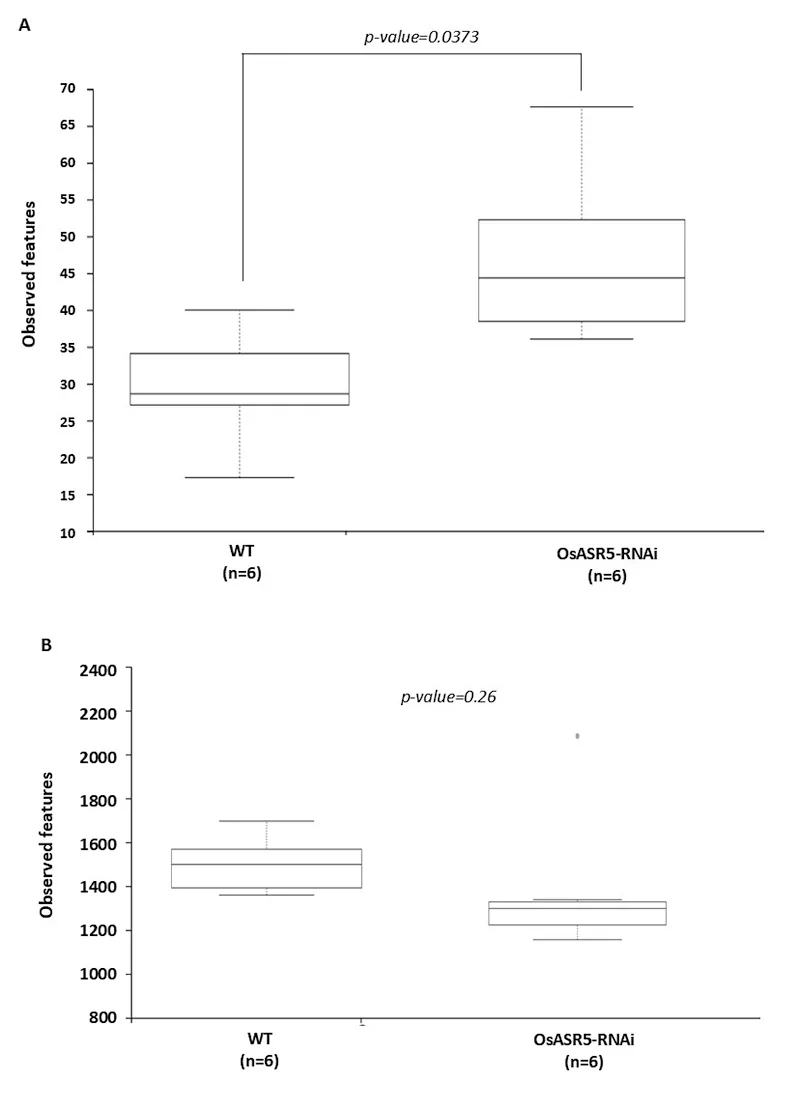
Alpha diversity based on observed feature index in rice-associated microbial communities (A) leaf microbiome. (B) Root microbiome. Statistical differences between groups were evaluated using the Kruskal-Wallis test, and p-values < 0.05 were considered statistically significant. Data are presented as boxplots showing median and interquartile range.

### Beta diversity and community structure

The bacterial communities of ASR5 RNAi and wild-type plants showed significant differences in terms of beta diversity, as assessed by PERMANOVA analysis. For leaf tissue, Jaccard distance was used (Figure 2 A ), while Bray-Curtis distance was applied for root (Figure 2 B ). PCoA analysis revealed a clear separation between genotypes for both organs. For leaf, the PCoA ordination (Figure 2 C, based on Jaccard distance) showed that axis 1 explained 11.71% of the variation, with the bacterial communities of ASR5-RNAi and wild-type plants being distant in the ordination space. A similar pattern was observed for root, with axis 1 explaining 22.58% of the variation (Figure 2 D).

**Figure 2 -:**
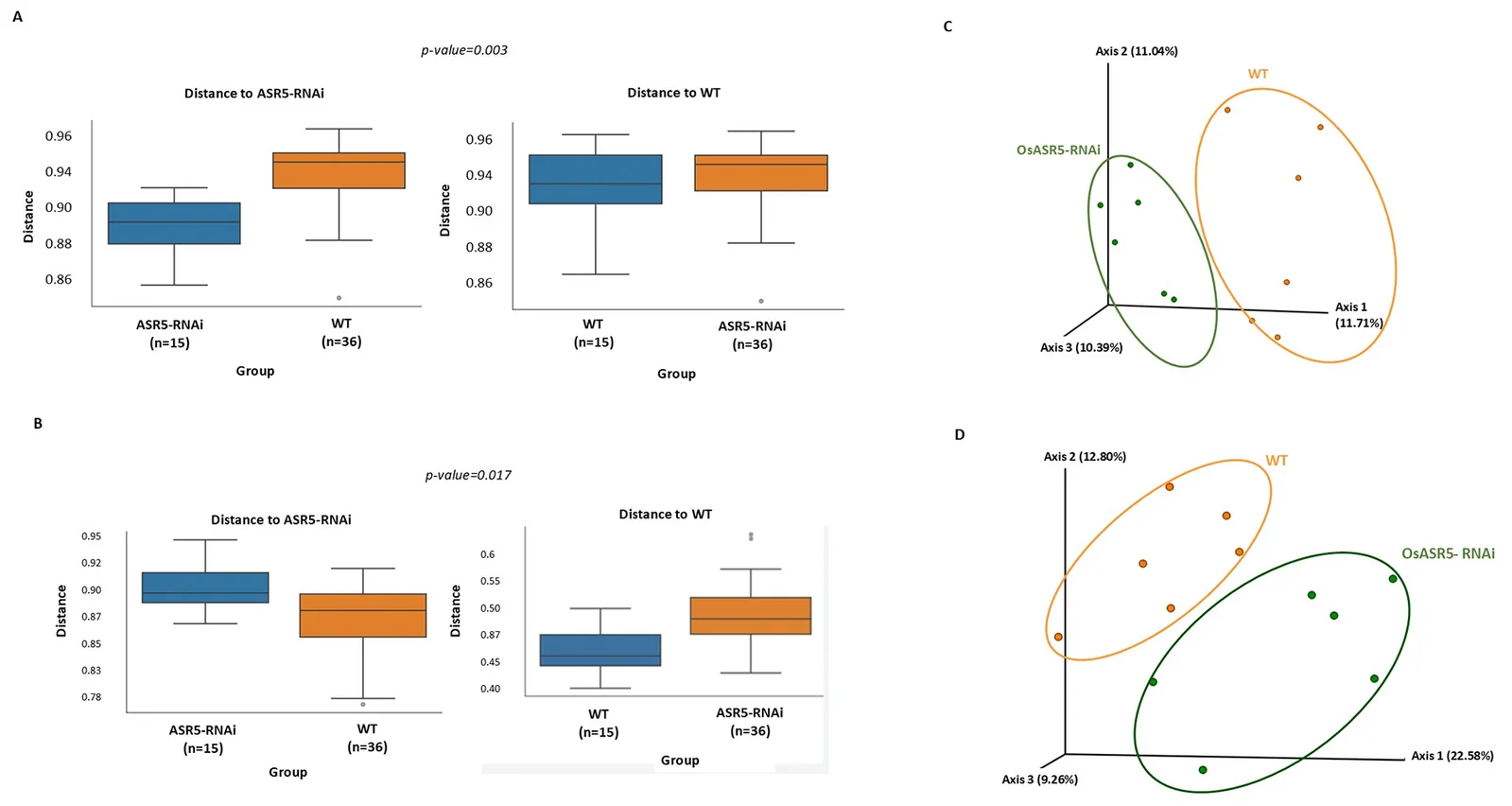
Beta diversity of rice-associated microbial communities based on Jaccard distance. (A) Leaf microbiome. (B) Root microbiome. Data are presented as boxplots showing median and interquartile range. Principal Coordinates Analysis (PCoA) based on Jaccard distance for (C) leaf and (D) root microbiomes. Differences in community composition between groups were assessed using PERMANOVA, with p < 0.05 considered statistically significant.

### Taxonomic shifts associated with ASR5 silencing

Leaf taxonomic analysis (Figure 3 A ) at phylum level revealed that, as a result of OsASR5 silencing, Acidobacteriota and Desulfobacteriota became more abundant in the ASR5-silenced plants, with a significant difference observed between genotypes (Table S4). Statistical analysis at family level also showed that *Rhodanobacteraceae*, *Oxobacteraceae*, *Hungateiclostridiaceae*, *Acidobacteriaceae*, *Rhodocyclaceae*, and *Geobacteraceae* were differentially more abundant in the ASR5-silenced genotype (Table S5). In roots, no significant differences were observed at the phylum level (Figure 3 B , Table S6). However, taxonomic analysis revealed that the most dominant phyla in the root samples were Proteobacteria (47.2%), Acidobacteriota (9.4%), Firmicutes (9.4%), and Desulfobacteria (3.9%) (Table S6). At the family level, differences included an increase in *Desulfobulbaceae* and *Aneurinibacillaceae*, as well as a reduction in the abundance of *Caloramatoraceae*, *Paludibacteraceae*, *Gracilibacteraceae*, *Microbacteriaceae*, *Rhodobacteraceae*, and *Sandaracinaceae* (Table S7).

**Figure 3 -:**
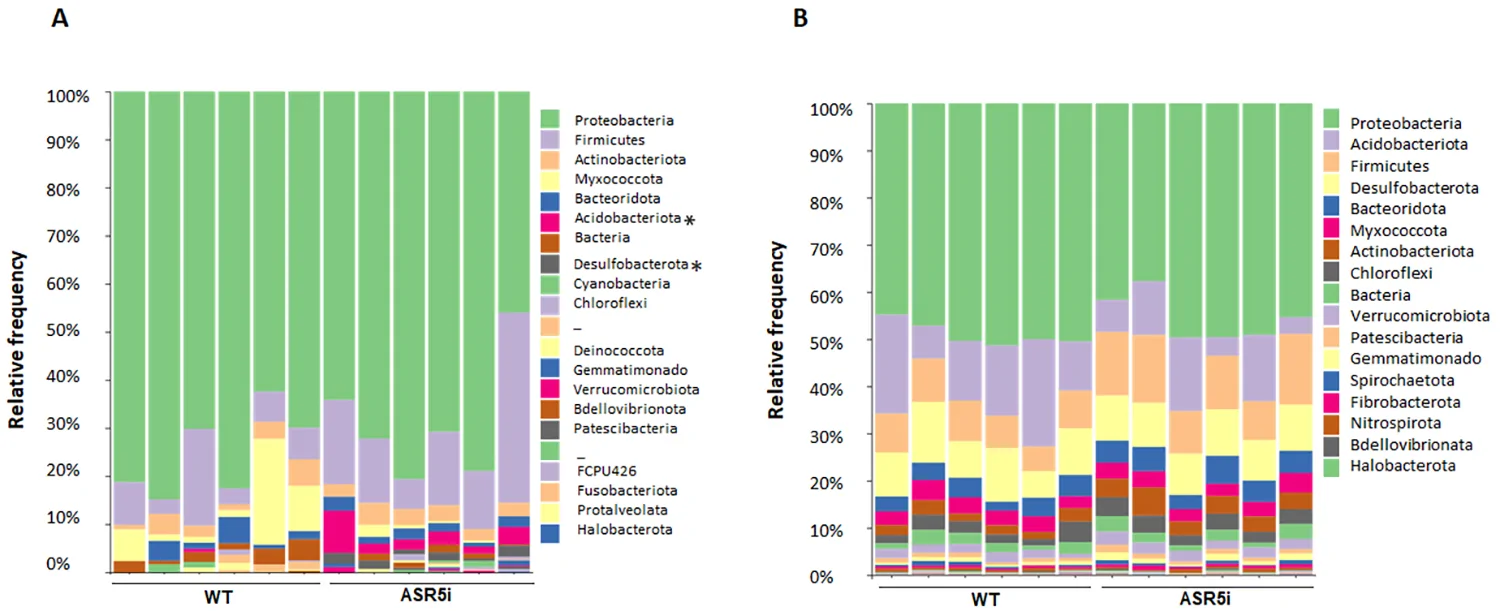
Relative abundance of bacterial taxa at the phylum level in rice-associated microbial communities. Taxa boxplots represent the distribution of relative abundance in (A) the leaf microbiome and (B) the root microbiome.

### Genotype-specific bacterial genera

The number of shared and unique bacteria genus between genotypes was investigated. For this purpose, Venn diagrams were constructed considering the presence of bacteria genus in at least one sample per plant group. For leaf tissue, 60 bacterial genera are shared by both genotypes, while various bacteria are unique to each genotype (Figure 4 A , Table S8). In this respect, *ASR5* silenced plants had the highest number of unique bacteria (140 genus), while wild-type plants had 42 unique bacteria. Interestingly, *Escherichia*, *Aneurinibacillus*, and *Geobacteria* are more abundant in OsASR5-RNAi plants, while *Methylobacterim* is less abundant (Figure 4 B ). 

For root tissue, 534 bacterial genera were shared by both genotypes (Figure 4 C , Table S9). *ASR5* silenced plants had 133 of unique genus and wild-type plants had 128 unique bacteria. *Fonticella, Lutispora, Magnetospirillum,* were negatively affected, and *Geobacter*, *Anaeromyxobacter*, *Kapabacteriales, Geothrix, Longivirga, Desulfobulbus, Bordetella, Methylobacter, Rhizobium, Azospira, Aneurinibacillus, Taonella* were positively affected by ASR5 silencing. (Figure 4 D )

**Figure 4 -:**
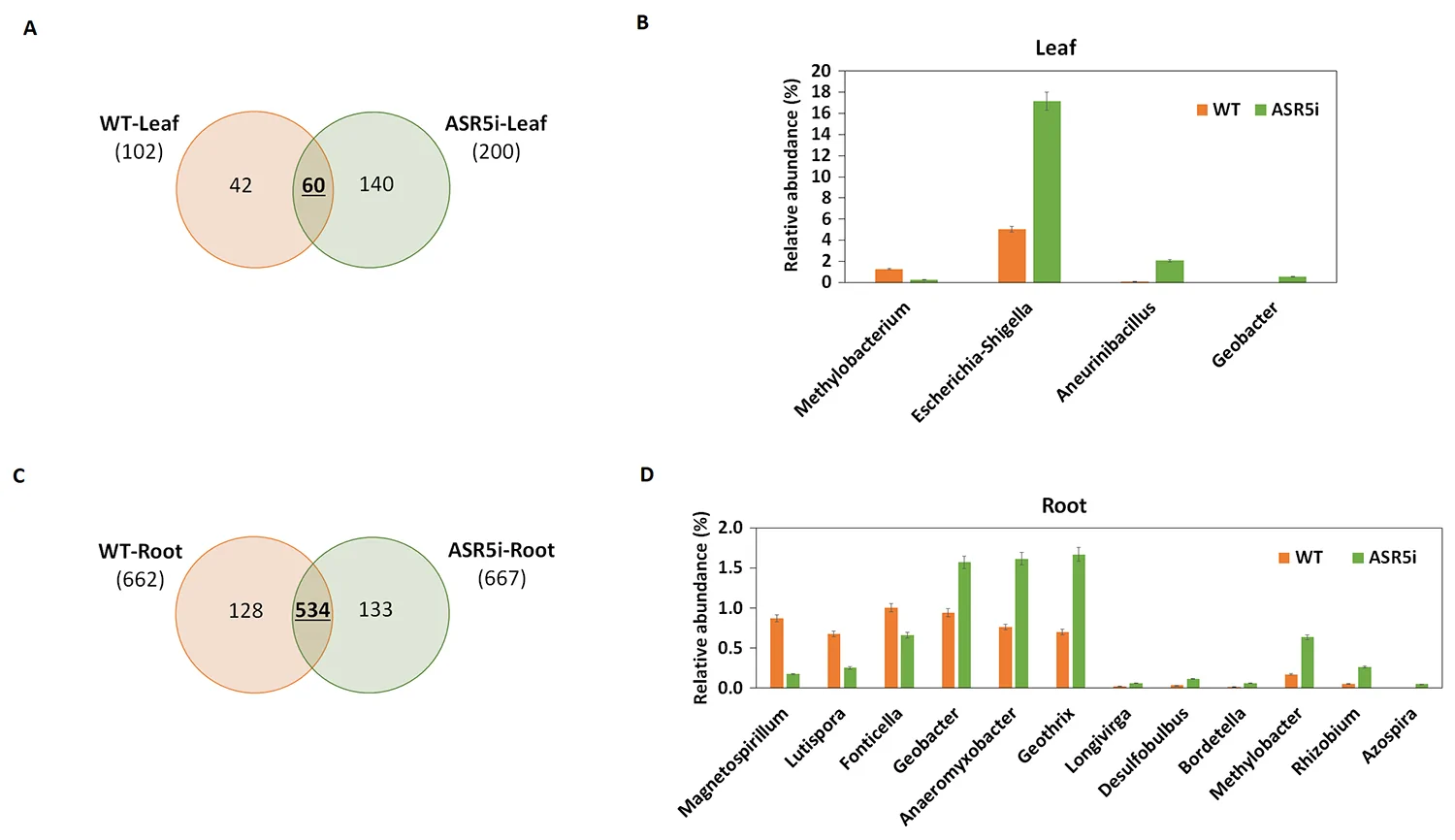
Bacterial community composition and differentially abundant genera in rice-associated microbiomes. (A) Venn diagram shows the number of unique and shared bacterial genera between wild type (WT) and knockdown plants (ASR5-RNAi) in the leaf microbiome. (B) Relative abundance of the most abundant bacterial genera in the leaf microbiome that were significantly different between genotypes (t-test, p < 0.05). (C) Venn diagram showing the number of unique and shared bacterial genera between WT and ASR5-RNAi plants in the root microbiome. (D) Relative abundance of the most abundant bacterial genera in the root microbiome that was significantly different between genotypes (t-test, p < 0.05). Bars represent mean relative abundance and error bars indicate standard error.

## Discussion

OsASR5 is a transcription factor involved in aluminum stress tolerance in rice (Arenhart et al., 2013, 2014) and also has a key role in nutrient acquisition. Reanalysis of the root transcriptome of ASR5-RNAi plants compared to wild-type Nipponbare rice, based on data from Arenhart *et al*. (2013), revealed the downregulation of several genes associated with the transport of essential ions, including iron, aluminum, phosphate, urea, ammonia, and potassium, while transcripts related to sodium and sulfate transport were upregulated (Figure 5 A ). Previous studies have shown that silencing *OsASR5* represses phosphate starvation responses and affects root architecture, reinforcing its role in nutrient homeostasis (Barros et al., 2025). Likewise, Ponte et al. (2025) reported that OsASR5-RNAi plants exhibit altered ion profiles and attenuated expression of Fe deficiency-responsive genes under Fe limitation compared to wild-type (WT) plants, suggesting that ASR5 contributes to regulating iron homeostasis. In this context, we investigated whether reduced ASR5 expression affects plant microbiome recruitment. Our results revealed that OsASR5 silencing led to noticeable changes in both phyllosphere and rhizosphere bacterial communities, including shifts in beta diversity and taxonomic composition. These findings align with previous reports demonstrating that plant genotype influences microbiome assembly (Singh et al., 2022; Su et al., 2024).

**Figure 5 -:**
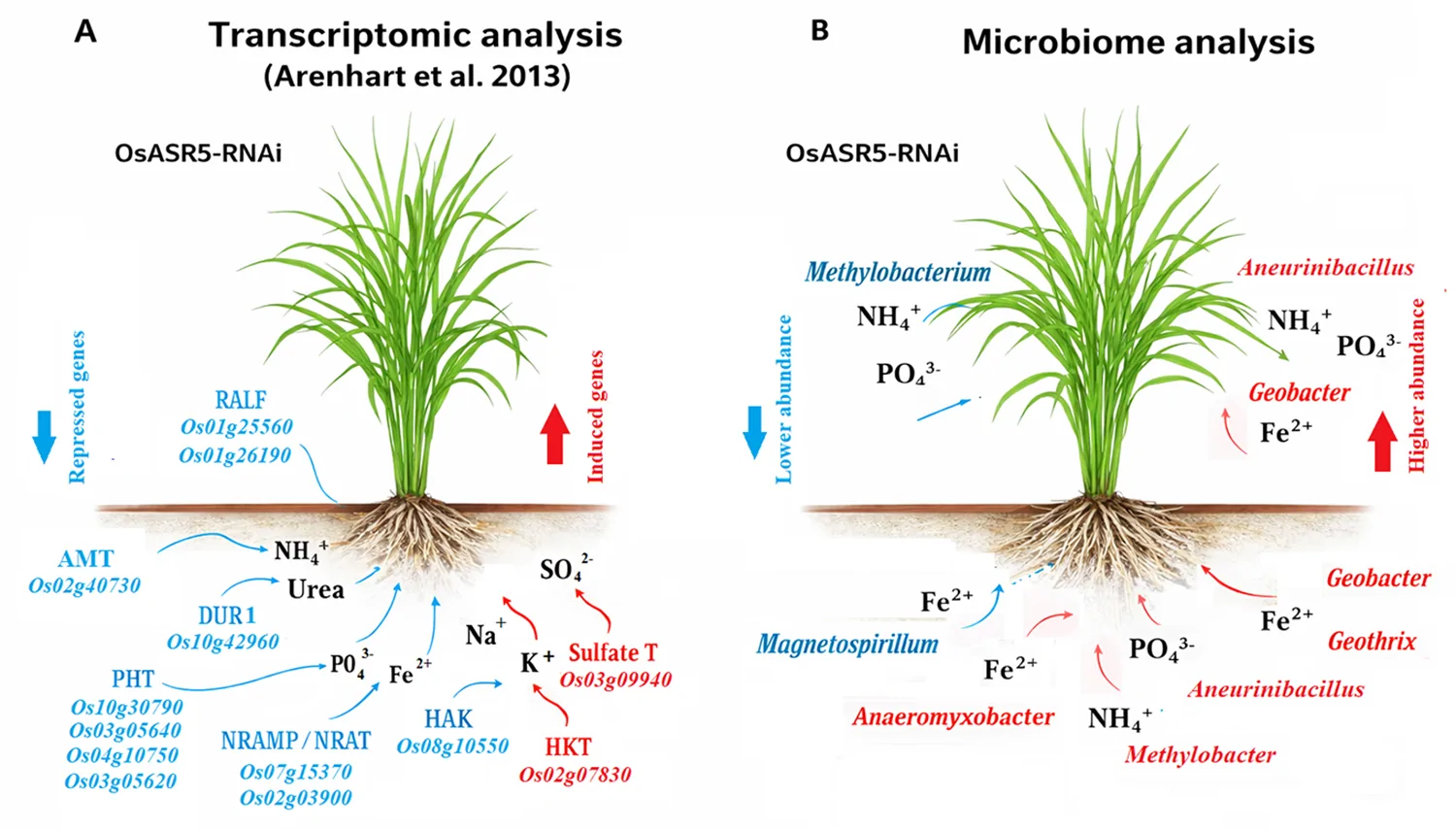
(A) Transcriptomic comparison between OsASR5-RNAi and wild-type Nipponbare plants using root tissue data from Arenhart et al. (2013). Genes associated with metal and ion transport are shown, with increased (red) or decreased (blue) expression in OsASR5-RNAi plants. (B) Bacteria associated with OsASR5-RNAi plants in leaves and roots under basal conditions. Blue indicates taxa with reduced relative abundance, while red represents taxa with increased relative abundance compared with wild-type plants. Data from this study.

Importantly, although several OsASR5-mediated mechanisms have been described under nutrient stress conditions, such as aluminium toxicity or iron and phosphorus deficiency, our study was conducted under standard growth conditions. Therefore, the microbial shifts observed here likely reflect altered basal regulation of nutrient homeostasis rather than responses to imposed stress. It is well known that nutrient homeostasis affect each other, and molecular mechanisms of crosstalk are being described (Wairich et al., 2025). Previously described stress-related roles of OsASR5 provide a useful mechanistic framework, suggesting that pathways involved in nutrient balance may also influence microbiome assembly even in non-stress environments. 

In our study, the abundance of plant growth-promoting and nutrient-cycling bacteria was modulated by genotype (Figure 5 B ). For instance, *Methylobacterium*, known for nitrogen fixation (Palberg et al., 2022; Valente et al., 2024), and phosphorus solubilization (Lu et al., 2023) was less abundant in the OsASR5-silenced leaves. Conversely, genera such as *Aneurinibacillus* (found in both leaf and root), *Anaeromyxobacter* (in root), and *Methylobacter* (in root), were enriched in the OsASR5i plants and are linked to nitrogen cycling and phosphorus solubilization (Chauhan et al., 2017; Wang et al., 2020; Lu *et al*., 2023). 

Notably, iron-reducing bacteria were also affected. *Magnetospirillum* was less abundant in ASR5-silenced roots, whereas *Geobacter and Geothrix,* known Fe(III)-reducing genera (Amor et al., 2018; Han et al., 2023; Wang et al., 2023; Masó-Martínez et al., 2024)*,* were enriched. These shifts suggest that OsASR5 influences microbial groups involved in nutrient redox processes and cycling*.* Such microbiome restructuring may represent a compensatory response to altered nutrient regulation in ASR5-RNAi plants. Since OsASR5 is known to coordinate responses related to aluminium detoxification and nutrient balance, its silencing may indirectly shape the phyllosphere and rhizosphere environment-potentially affecting microbial recruitment through changes in tissue exudation patterns.

Interactions among iron, phosphorus, and aluminium availability are well documented in soils, particularly under acidic conditions (Qiu et al., 2019; Nam et al., 2021; Alves et al., 2025). Rice mitigates aluminium toxicity through organic acid efflux into the rhizosphere, a process regulated by OsASR5. Organic acids such as citrate and acetate can also serve as electron donors for Fe(III)-reducing microbes, including *Anaeromyxobacter* and *Geobacter*, facilitating Fe³⁺ reduction to Fe²⁺ (Ding et al., 2008; Hori et al., 2010). Thus, OsASR5-dependent modulation of root exudation may influence microbial-mediated nutrient transformations. In conclusion, our findings indicate that OsASR5 contributes to shaping plant-associated microbial communities, likely through its role in regulating nutrient homeostasis and rhizosphere-phyllosphere interactions. This influence on microbiome assembly may affect nutrient cycling and plant adaptive responses, extending the functional relevance of OsASR5 beyond stress-specific contexts. Together, these results provide new insights into the multifaceted role of OsASR5 in coordinating nutrient regulation and plant-microbe interactions, with potential implications for improving crop resilience and nutrient management strategies.

## Supplementary material

The following online material is available for this article:

Table S1 - Summary of sequencing depth, read quality, and filtered reads for each sample according to plant genotype and compartment.

Table S2 - OTU abundance per sample from leaf microbiome.

Table S3 - OTU abundance per sample from root microbiome.

Table S4 - Bacterial phylum abundance per sample from leaf microbiome.

Table S5 - Bacterial family abundance per sample from leaf microbiome.

Table S6 - Bacterial phylum abundance per sample from root microbiome.

Table S7 - Bacterial family abundance per sample from root microbiome

Table S8 - Bacterial genera abundance per sample from leaf microbiome.

Table S9 - Bacterial genera abundance per sample from root microbiome.

## Data Availability

 The data supporting the findings of this study are available from the corresponding author, Rogerio Margis, upon request.
