# Supplementary material for: The impact of OsASR5 on rice microbiome: Implications for nutrient cycling and stress response
Source: Genet Mol Biol. 2026 Oct 26;49(Suppl 3):e20250064. doi: 10.1590/1678-4685-GMB-2025-0064 (PMC13720868; doi:10.1590/1678-4685-GMB-2025-0064)
Supplement: Supplementary file 1 [file 1415-4757-GMB-49-s3-e20250064-s1.pdf]

Genetics and Molecular Biology

https://doi.org/10.1590/1678-4685-GMB-2025-0064

Supplementary Material to "The impact of OsASR5 on rice microbiome: Implications for nutrient cycling and stress response"

Table S1 - Summary of sequencing depth, read quality, and filtered reads for each sample according to plant genotype and compartment.

Sample ID	Compartment	Genotype	Sequencing Depth	% Good Quality Reads	Reads Passed Filtering
F1_ASR5i	F	ASR5i	68537	80.33	55058
F2_ASR5i	F	ASR5i	94181	80.58	75888
F3_ASR5i	F	ASR5i	104564	80.93	84620
F4_ASR5i	F	ASR5i	95136	81.05	77107
F5_ASR5i	F	ASR5i	21310	81.94	17461
F6_ASR5i	F	ASR5i	52125	59.58	31056
F1_WT	F	WT	73032	79.92	58368
F2_WT	F	WT	71956	79.55	57241
F3_WT	F	WT	26835	84.88	22778
F4_WT	F	WT	35315	84.30	29770
F5_WT	F	WT	42122	83.42	35140
F6_WT	F	WT	52125	82.80	43161
R1_ASR5i	R	ASR5i	29379	78.81	23154
R2_ASR5i	R	ASR5i	61626	75.50	46527
R3_ASR5i	R	ASR5i	51319	80.46	41291
R4_ASR5i	R	ASR5i	53749	71.75	38564
R5_ASR5i	R	ASR5i	45913	80.53	36976
R6_ASR5i	R	ASR5i	113760	77.49	88153
R1_WT	R	WT	42993	81.24	34928
R2_WT	R	WT	89377	67.21	60067
R3_WT	R	WT	109455	67.64	74039
R4_WT	R	WT	81788	73.60	60192
R5_WT	R	WT	75154	78.97	59349
R6_WT	R	WT	118126	67.99	80316
