# Supplementary material for: The impact of OsASR5 on rice microbiome: Implications for nutrient cycling and stress response
Source: Genet Mol Biol. 2026 Oct 26;49(Suppl 3):e20250064. doi: 10.1590/1678-4685-GMB-2025-0064 (PMC13720868; doi:10.1590/1678-4685-GMB-2025-0064)
Supplement: Supplementary file 2 [file 1415-4757-GMB-49-s3-e20250064-s2.pdf]

Genetics and Molecular Biology

https://doi.org/10.1590/1678-4685-GMB-2025-0064

Supplementary Material to "The impact of OsASR5 on rice microbiome: Implications for nutrient cycling and stress response"

Table S2 - OTU abundance per sample from leaf microbiome.

#OTU ID	L1_ASR5i	L1_WT	L2_ASR5i	L2_WT	L3_ASR5i	L3_WT	L4_ASR5i	L4_WT	L5_ASR5i	L5_WT	L6_ASR5i	L6_WT
6b132e267e31a04cdb4919c38ed95d88	16.0	18.0	0.0	0.0	11.0	3.0	0.0	43.0	3.0	6.0	6.0	4.0
eda9d8f034d9ab57ec7c5a0dc356634e	0.0	0.0	21.0	0.0	0.0	0.0	0.0	0.0	0.0	0.0	0.0	0.0
1b9af6ea4c6046e61eec82ad8318bb2d	3.0	3.0	0.0	0.0	0.0	0.0	0.0	0.0	0.0	0.0	0.0	0.0
771c04242b0a935ac07b2aeeef005f07	0.0	0.0	0.0	0.0	0.0	0.0	0.0	37.0	0.0	0.0	0.0	0.0
5ddf7571629a1dd2a57106f7a6a57e8a	45.0	0.0	0.0	0.0	0.0	0.0	12.0	0.0	0.0	0.0	6.0	0.0
5cf8078712e0e0c61c91da8e9fe5d2fe	0.0	0.0	4.0	0.0	0.0	0.0	0.0	0.0	0.0	4.0	0.0	0.0
38e1f0fa5f42998d8e00e78a39229896	0.0	2.0	0.0	0.0	0.0	0.0	0.0	0.0	0.0	0.0	0.0	0.0
aa108b32328e8ad7c3c1cca242ab4e52	0.0	0.0	2.0	0.0	0.0	0.0	0.0	0.0	0.0	0.0	0.0	0.0
f1fa069fd7ffd7452f03c9ebee9bf503	3.0	0.0	0.0	0.0	0.0	0.0	14.0	0.0	0.0	0.0	0.0	0.0
caa2591653bf2305dd2b2641533ca0fd	30.0	0.0	80.0	46.0	351.0	42.0	363.0	11.0	94.0	54.0	52.0	0.0
eb394fbde90ee2e5b23b8db7cb3a92d9	41.0	0.0	0.0	4.0	6.0	0.0	19.0	0.0	9.0	0.0	10.0	0.0
cf90188b9bd6e105b09868336c855c42	0.0	0.0	9.0	0.0	0.0	0.0	0.0	0.0	0.0	0.0	0.0	0.0
616d5dcd765b60cdc5e93eabf47de30c	0.0	0.0	19.0	0.0	0.0	0.0	0.0	0.0	0.0	0.0	0.0	0.0
e883c5ed19ecbc5613011381fbe4e68e	0.0	0.0	0.0	4.0	0.0	0.0	0.0	0.0	0.0	0.0	0.0	0.0
995ef38682bb208fb33238a5fec30e43	0.0	0.0	0.0	5.0	0.0	10.0	0.0	28.0	0.0	0.0	0.0	0.0
01434f1f96d343412375f34c807560e1	0.0	0.0	0.0	0.0	5.0	0.0	0.0	0.0	0.0	9.0	0.0	0.0
6736ea039cbe05de6d2be949ea474617	0.0	19.0	0.0	12.0	8.0	0.0	0.0	0.0	0.0	0.0	0.0	0.0

#OTU ID	L1_ASR5i	L1_WT	L2_ASR5i	L2_WT	L3_ASR5i	L3_WT	L4_ASR5i	L4_WT	L5_ASR5i	L5_WT	L6_ASR5i	L6_WT
68f41afd948c4033105bb49e59388ab7	0.0	0.0	0.0	0.0	0.0	0.0	0.0	0.0	4.0	0.0	0.0	0.0
ccbc5f8778fb4aac36fac232a0ef6335	0.0	0.0	0.0	0.0	0.0	0.0	0.0	0.0	0.0	14.0	0.0	0.0
f55bbad68f06560c29e45fd27516dcc5	0.0	0.0	10.0	3.0	22.0	0.0	0.0	0.0	0.0	0.0	0.0	0.0
20c6e6ff93e0e439e7f21e4023fe61ac	0.0	8.0	0.0	0.0	0.0	0.0	0.0	0.0	0.0	0.0	0.0	0.0
7d90c7e399700a5e04f4b53aff4eacff	3.0	0.0	0.0	0.0	0.0	0.0	0.0	0.0	0.0	0.0	0.0	0.0
3aa2ed0f80ec98ad7e04b38230837e5d	0.0	0.0	0.0	0.0	11.0	0.0	0.0	0.0	0.0	0.0	3.0	0.0
ff10a3f70d93fffd82380bb5348b98c9	0.0	0.0	0.0	0.0	0.0	0.0	0.0	0.0	3.0	0.0	0.0	0.0
d99d4febfc3b15e2c499d060967bf761	0.0	0.0	0.0	0.0	0.0	13.0	12.0	4.0	0.0	0.0	0.0	0.0
c5e152cb43440ab15b610fcf744bf99f	0.0	0.0	0.0	0.0	0.0	0.0	0.0	5.0	0.0	0.0	0.0	0.0
a6045db18891e4b07752710449944fd3	0.0	0.0	0.0	0.0	0.0	0.0	12.0	0.0	0.0	0.0	0.0	0.0
3b1c6a2d73bdc2ea162ac7b4ddb91d28	0.0	0.0	0.0	0.0	11.0	0.0	0.0	0.0	0.0	0.0	0.0	0.0
6b3a72462e8f406bd94434f25e8dbafb	0.0	0.0	0.0	0.0	3.0	0.0	0.0	0.0	0.0	0.0	0.0	0.0
423fec6d1841fded71346e4e14fc6d4b	9.0	0.0	20.0	0.0	0.0	130.0	22.0	3.0	0.0	4.0	0.0	0.0
09e005324c7318e29864a91da2f3ad3f	0.0	0.0	0.0	0.0	4.0	0.0	0.0	0.0	0.0	0.0	0.0	0.0
821862745e4b0d2f22ca9bdfc90c3c95	3.0	0.0	4.0	0.0	0.0	0.0	0.0	0.0	0.0	0.0	0.0	0.0
fb363ebeb1b8df1abfb2c45ee4cc21e8	0.0	0.0	0.0	5.0	0.0	0.0	0.0	12.0	0.0	0.0	0.0	0.0
f4eabb05bd9788542d83673d22f06429	2.0	0.0	0.0	0.0	0.0	0.0	13.0	0.0	0.0	0.0	0.0	0.0
9395a8438ba71a178cca6fc51620d28f	0.0	2.0	0.0	11.0	0.0	0.0	6.0	26.0	5.0	0.0	0.0	0.0
947b7abd721fa840389574d3186e2892	7.0	0.0	0.0	0.0	0.0	0.0	0.0	29.0	0.0	5.0	0.0	0.0
932c1a96f7455df97f3185e6b9f76536	0.0	0.0	0.0	0.0	0.0	0.0	0.0	9.0	0.0	0.0	0.0	0.0
94ccb2053e41d525207ae33769cf5db0	0.0	0.0	0.0	0.0	0.0	3.0	0.0	0.0	0.0	0.0	2.0	0.0
d650ff4ecf72293b011b0c0192490f4a	26.0	8.0	37.0	0.0	44.0	0.0	0.0	17.0	2.0	9.0	4.0	4.0
a4c281cd48ae8a9eaf83577833395c11	0.0	0.0	11.0	0.0	24.0	2.0	0.0	0.0	0.0	0.0	0.0	0.0
ee50c6156c43d0a332c408445adf06a8	0.0	0.0	0.0	0.0	0.0	10.0	0.0	0.0	0.0	0.0	0.0	0.0
c1a475ef6843189b546d7e46bee19dd7	4.0	0.0	0.0	0.0	0.0	0.0	0.0	0.0	0.0	0.0	0.0	0.0
b6ba440a073ba27eea423172b3691990	0.0	0.0	4.0	0.0	0.0	0.0	0.0	0.0	0.0	0.0	0.0	0.0
2d2c7554fd6f5beb272ba798e7b807d7	0.0	0.0	0.0	0.0	34.0	0.0	13.0	0.0	0.0	0.0	0.0	0.0
0569d3bfc998b1a92e9c1a7613433777	5.0	0.0	0.0	4.0	0.0	0.0	0.0	0.0	0.0	0.0	0.0	0.0
7cea5709b1dd69c15a56388fe9846bf4	0.0	0.0	11.0	0.0	4.0	0.0	0.0	0.0	0.0	0.0	0.0	0.0

#OTU ID	L1_ASR5i	L1_WT	L2_ASR5i	L2_WT	L3_ASR5i	L3_WT	L4_ASR5i	L4_WT	L5_ASR5i	L5_WT	L6_ASR5i	L6_WT
591edb972aca7223d709711cf0cce770	0.0	3.0	0.0	0.0	0.0	0.0	0.0	0.0	0.0	0.0	0.0	0.0
e045542ff52d61816d8f8ef1e3b03b49	0.0	0.0	0.0	0.0	0.0	0.0	0.0	7.0	0.0	0.0	0.0	0.0
152c2aef73f046b9b9a5546ec288d1bb	0.0	0.0	0.0	0.0	0.0	0.0	0.0	4.0	0.0	0.0	4.0	0.0
6c069025b4d7654f52fb219bb037a268	0.0	0.0	0.0	0.0	0.0	0.0	0.0	0.0	0.0	6.0	0.0	0.0
21afbd1df09d16caf084e68c6dcbcfd6	2.0	0.0	0.0	0.0	0.0	0.0	0.0	0.0	0.0	0.0	0.0	0.0
6da964ec38cc11acaf6225e97f653101	10.0	0.0	0.0	0.0	8.0	0.0	0.0	0.0	0.0	0.0	4.0	0.0
393e1e79854e0e671b97c68f1a1d12e4	8.0	0.0	0.0	0.0	0.0	0.0	0.0	0.0	0.0	0.0	0.0	0.0
3ac222277f5a2adfb0c585621c7fc313	6.0	0.0	0.0	0.0	0.0	0.0	0.0	0.0	0.0	0.0	0.0	0.0
d4789017fdec6acdbe2559113b0d8272	4.0	0.0	0.0	0.0	0.0	0.0	0.0	0.0	0.0	0.0	0.0	0.0
9ba4c57f7e8f223fe3e4f5c600df47b3	0.0	0.0	0.0	0.0	0.0	0.0	15.0	0.0	0.0	0.0	0.0	0.0
72e56b924b36e361ca28d27878e8a62b	0.0	4.0	0.0	0.0	0.0	0.0	0.0	0.0	0.0	0.0	0.0	0.0
94fdeb23ba8c6a0879cfb85ba1e6fed8	0.0	0.0	0.0	0.0	0.0	0.0	0.0	0.0	4.0	0.0	0.0	0.0
6df488822dde2a9d6b7e51c68fa43654	11.0	0.0	0.0	0.0	0.0	0.0	0.0	0.0	0.0	0.0	4.0	0.0
a46019cb4192a94dc08380c1d3e01cdc	0.0	0.0	3.0	0.0	0.0	0.0	0.0	0.0	0.0	0.0	0.0	0.0
6b8b4626dddf2b5fedde27d0b9ab9480	0.0	0.0	0.0	0.0	10.0	0.0	0.0	0.0	0.0	0.0	0.0	0.0
d296a61229aec8e4647e0bc1f5d74cb0	0.0	0.0	2.0	0.0	0.0	0.0	0.0	0.0	0.0	0.0	0.0	0.0
da9d5ea39d75a8af111d56f90920d9dd	0.0	0.0	0.0	0.0	0.0	0.0	0.0	0.0	2.0	0.0	0.0	0.0
64d78f32b3207d6da2187755865c3d34	0.0	0.0	0.0	0.0	0.0	0.0	15.0	0.0	0.0	0.0	0.0	0.0
d21ddd1304159ae6309fdaff6580aa1a	2.0	0.0	0.0	0.0	0.0	0.0	0.0	0.0	0.0	0.0	0.0	0.0
2ec04c6a51e69598d6533e7d81e5cd97	0.0	0.0	0.0	0.0	9.0	0.0	0.0	0.0	0.0	0.0	0.0	0.0
e958218947e6d2e2c7ee1482fa1d6d43	0.0	0.0	0.0	0.0	4.0	0.0	0.0	0.0	0.0	0.0	0.0	0.0
9224d1302394c0ffcd2ff6cf4cd03582	0.0	2.0	0.0	0.0	0.0	0.0	0.0	0.0	0.0	0.0	0.0	0.0
ac6342d19b53605e0b3e5046efd1bcf3	0.0	0.0	0.0	0.0	0.0	0.0	0.0	0.0	2.0	0.0	0.0	0.0
c862fd22cb60bd5c99b2f357b3f3b8f2	12.0	0.0	6.0	0.0	11.0	0.0	0.0	0.0	0.0	0.0	0.0	0.0
582233271ee735b9681aaa228f24aa3a	0.0	16.0	0.0	0.0	21.0	27.0	0.0	0.0	0.0	3.0	0.0	0.0
7069150b50bb66fc96ca59d6051ef10c	13.0	0.0	2.0	0.0	17.0	0.0	0.0	0.0	0.0	0.0	8.0	0.0
3f7440c3663343ac2704638e4b37c483	0.0	2.0	0.0	0.0	0.0	0.0	0.0	0.0	0.0	0.0	0.0	0.0
a8cc598a3761a602e159d456daff5b50	0.0	0.0	12.0	0.0	0.0	0.0	0.0	0.0	0.0	0.0	0.0	0.0
4f06522c5ea6c8b6c1e3bd22743948a4	7.0	0.0	0.0	0.0	0.0	0.0	0.0	0.0	2.0	0.0	0.0	0.0

#OTU ID	L1_ASR5i	L1_WT	L2_ASR5i	L2_WT	L3_ASR5i	L3_WT	L4_ASR5i	L4_WT	L5_ASR5i	L5_WT	L6_ASR5i	L6_WT
9ee25053cb74bd563e33744ad42c0084	0.0	0.0	4.0	0.0	0.0	0.0	0.0	0.0	0.0	0.0	0.0	0.0
edd8c7402c51d38878f6789bf47fe8ee	0.0	0.0	0.0	0.0	0.0	0.0	0.0	2.0	0.0	0.0	0.0	0.0
7f191c8ba69132e1e8f01cd9ee88bd87	0.0	0.0	0.0	0.0	0.0	0.0	0.0	3.0	0.0	0.0	0.0	0.0
e2967d558637db97799961c3f3acbb69	0.0	0.0	0.0	0.0	0.0	0.0	0.0	0.0	3.0	0.0	0.0	0.0
49d205c67eb35f08d9eee00e7fa8f0d4	3.0	0.0	0.0	0.0	0.0	0.0	0.0	0.0	0.0	0.0	0.0	0.0
ff1821a92fa1aee30a09856f5dbc0854	2.0	0.0	0.0	0.0	0.0	0.0	0.0	0.0	0.0	0.0	0.0	0.0
8323dfaee501b342797f7a48c758d0e6	0.0	0.0	0.0	0.0	25.0	0.0	0.0	0.0	0.0	0.0	0.0	0.0
287e4a90ad50937927a8ef25d6648d86	8.0	3.0	0.0	0.0	11.0	0.0	0.0	5.0	0.0	0.0	0.0	0.0
793f38b450d183c7ef46439a4a7facdd	0.0	0.0	0.0	0.0	0.0	0.0	0.0	0.0	0.0	2.0	0.0	0.0
c36e37062680907e461387b207f09e01	0.0	0.0	6.0	0.0	0.0	0.0	0.0	0.0	0.0	0.0	0.0	0.0
8280499fec35fa1fd1650d2100e92dd5	0.0	0.0	0.0	0.0	0.0	0.0	8.0	0.0	0.0	0.0	0.0	0.0
209c49e8932f08abfca7a1c8efb4dcb5	11.0	0.0	6.0	0.0	0.0	0.0	0.0	0.0	0.0	0.0	0.0	0.0
208c4d1626b30253d698652e8acb55c2	0.0	0.0	5.0	0.0	0.0	0.0	0.0	0.0	0.0	0.0	0.0	0.0
6c587e6a9cafc0c7cbde521b7a7bd9e7	0.0	0.0	0.0	0.0	0.0	0.0	0.0	0.0	0.0	8.0	0.0	0.0
eb7580bdd8381194c64f70300008ce6d	0.0	0.0	0.0	0.0	21.0	0.0	0.0	0.0	0.0	0.0	0.0	0.0
58b9c5cceed1ef86bea29236caf012de	0.0	0.0	0.0	0.0	0.0	0.0	0.0	0.0	0.0	0.0	2.0	0.0
622a2e098e2788407e3b7c4974dacf80	27.0	0.0	0.0	0.0	11.0	0.0	0.0	0.0	0.0	0.0	0.0	0.0
90e35cd2808533e68d1f66dec5f005c8	0.0	0.0	0.0	0.0	10.0	0.0	0.0	0.0	0.0	0.0	0.0	0.0
99baa034688e2c8bef38ab5c8d1fa7ae	0.0	0.0	0.0	0.0	0.0	0.0	0.0	10.0	0.0	0.0	0.0	0.0
b809df1a5834eec09691921f6e043287	0.0	0.0	0.0	0.0	0.0	0.0	5.0	0.0	0.0	0.0	0.0	0.0
10a2f0e6c6e29e2b08103f2d0313d227	0.0	0.0	0.0	0.0	0.0	0.0	11.0	0.0	0.0	0.0	0.0	0.0
3a0fef955ffa3ac46fe6e1ca33fc2fbb	0.0	0.0	10.0	0.0	0.0	0.0	0.0	0.0	0.0	0.0	3.0	0.0
d5758f5755662fa107f118de9eafcfd5	0.0	0.0	0.0	0.0	10.0	0.0	0.0	0.0	0.0	0.0	0.0	0.0
a41e12b93bed4431009e25202abdaba8	0.0	0.0	0.0	0.0	7.0	0.0	0.0	0.0	0.0	0.0	0.0	0.0
c447a4a7694df57145ec4bff87f96b66	6.0	0.0	0.0	0.0	0.0	0.0	7.0	0.0	0.0	0.0	0.0	0.0
96a86bd0f8aa935988f314b2caf913cf	0.0	0.0	0.0	0.0	9.0	0.0	0.0	0.0	0.0	0.0	0.0	0.0
3128f7fd3c8b5fb372ccd63be53405cd	130.0	0.0	32.0	0.0	0.0	0.0	0.0	0.0	0.0	0.0	0.0	0.0
b06b09453128846be6186bf208b1240e	0.0	0.0	0.0	0.0	0.0	9.0	0.0	19.0	0.0	0.0	0.0	0.0
e4a0e32a96d6b6d2e9712bbf30e206fc	0.0	2.0	0.0	0.0	0.0	0.0	0.0	0.0	0.0	0.0	0.0	0.0

#OTU ID	L1_ASR5i	L1_WT	L2_ASR5i	L2_WT	L3_ASR5i	L3_WT	L4_ASR5i	L4_WT	L5_ASR5i	L5_WT	L6_ASR5i	L6_WT
873f822c31a9b7281ba9ba20e8bc25bc	0.0	0.0	11.0	0.0	19.0	0.0	6.0	0.0	5.0	0.0	0.0	4.0
1f6c334c4189f68606a493f8d4948805	14.0	0.0	0.0	0.0	5.0	0.0	0.0	0.0	0.0	0.0	2.0	0.0
51a2eca5c0adc16bf317085c4eb9a3a5	3.0	0.0	0.0	0.0	0.0	0.0	0.0	0.0	0.0	0.0	0.0	0.0
77173f04e990cce0914055aaa092e069	0.0	0.0	0.0	4.0	0.0	0.0	0.0	0.0	0.0	0.0	0.0	0.0
d8cc43ed76c543be363f368aae876a53	0.0	0.0	0.0	4.0	3.0	0.0	0.0	0.0	0.0	0.0	0.0	0.0
48deb0e8d0b759679a62bada686b9de1	0.0	0.0	0.0	0.0	0.0	0.0	0.0	0.0	0.0	0.0	2.0	0.0
8a946d0081af8af2c4685d5742a9498b	0.0	0.0	0.0	0.0	0.0	4.0	0.0	0.0	0.0	0.0	0.0	0.0
a248ccdeb7436acc4aa388a7617fe2e4	0.0	0.0	3.0	0.0	0.0	0.0	19.0	0.0	0.0	0.0	0.0	0.0
f9d000176b4681fa670887ee0f122634	0.0	0.0	6.0	0.0	0.0	0.0	0.0	0.0	0.0	0.0	0.0	0.0
fc1a70d9ca6196b80060b285cddce702	5.0	0.0	0.0	0.0	0.0	0.0	0.0	0.0	0.0	0.0	0.0	0.0
9f23d2a1a881be4c00378f8a53c5b9cd	0.0	0.0	0.0	5.0	0.0	0.0	0.0	0.0	0.0	0.0	0.0	0.0
131f6a7f9f0309819dca2d9ca421624b	40.0	0.0	0.0	0.0	19.0	0.0	21.0	0.0	13.0	0.0	21.0	0.0
4430c1d79ceeb77a18ed0855fc089878	0.0	0.0	5.0	0.0	0.0	0.0	0.0	0.0	0.0	0.0	0.0	0.0
c920054469eb9966c8f7191d84e93cd1	0.0	0.0	15.0	0.0	0.0	0.0	0.0	0.0	0.0	0.0	0.0	0.0
8d2ac18003c8ac5561903d3a0193d400	0.0	0.0	8.0	0.0	0.0	0.0	0.0	0.0	0.0	0.0	0.0	0.0
a37f0f3735011010141201e8eaa81a65	0.0	0.0	0.0	0.0	0.0	0.0	0.0	0.0	0.0	0.0	2.0	0.0
d5faf7a67432463efc4ea2f9e8b139e5	0.0	0.0	0.0	0.0	11.0	0.0	4.0	0.0	0.0	0.0	0.0	0.0
99a31dbdd68d175cc9fb3cbb67859750	4.0	0.0	0.0	0.0	0.0	0.0	0.0	0.0	0.0	0.0	0.0	0.0
b6f29a505347a477668f0b4fe135accf	9.0	0.0	0.0	0.0	0.0	0.0	0.0	0.0	6.0	0.0	0.0	0.0
d337f78f021ef017abbc9b27f7a65181	0.0	0.0	3.0	0.0	19.0	0.0	0.0	0.0	0.0	0.0	0.0	0.0
d31121d6733c4e4bfc1ee20e9524466e	9.0	30.0	18.0	8.0	38.0	0.0	39.0	0.0	9.0	0.0	0.0	9.0
3232c716f79ea8bf2f6f0bb0b1f27248	0.0	0.0	0.0	0.0	0.0	58.0	0.0	0.0	0.0	0.0	0.0	10.0
efe1011bab3127b436579fbdc45ab313	0.0	0.0	11.0	0.0	0.0	0.0	0.0	0.0	0.0	0.0	0.0	0.0
32f737e4186c17684cac890a6f07db74	0.0	0.0	0.0	0.0	0.0	0.0	16.0	0.0	0.0	0.0	0.0	0.0
f6e503e97d1810d7948ee8a0e5ccac57	0.0	0.0	0.0	0.0	0.0	0.0	0.0	0.0	5.0	0.0	0.0	0.0
e296a7313e77369ca665aeaa8e40ce81	0.0	0.0	0.0	0.0	0.0	0.0	0.0	0.0	0.0	6.0	0.0	0.0
3ddfe5fbddb2c2b40df6fc793ce6bd7d	0.0	0.0	0.0	0.0	0.0	0.0	0.0	0.0	0.0	4.0	2.0	0.0
2ab08ec39a4a3572c7339697a3e3a5ac	0.0	19.0	0.0	0.0	0.0	0.0	0.0	0.0	0.0	0.0	0.0	0.0
8aa4ba14b52d06cc891fc66f3b071ced	0.0	0.0	0.0	0.0	39.0	0.0	0.0	0.0	0.0	0.0	0.0	0.0

#OTU ID	L1_ASR5i	L1_WT	L2_ASR5i	L2_WT	L3_ASR5i	L3_WT	L4_ASR5i	L4_WT	L5_ASR5i	L5_WT	L6_ASR5i	L6_WT
1e53c14b23c67096f6f8d1235f061874	0.0	0.0	0.0	0.0	0.0	0.0	0.0	0.0	4.0	0.0	0.0	0.0
4cfd1653562e380a744c52a940c34a13	0.0	0.0	0.0	0.0	5.0	0.0	0.0	0.0	0.0	0.0	0.0	0.0
b783ceadb0f059e20cacacda1696c733	0.0	0.0	2.0	0.0	0.0	0.0	0.0	0.0	0.0	0.0	0.0	0.0
180d78df892ab06f0c41ce900cda1146	11.0	0.0	0.0	0.0	10.0	0.0	0.0	0.0	0.0	0.0	5.0	0.0
107c11b1ef1bf3da0618f994c17b4788	7.0	0.0	0.0	0.0	0.0	21.0	0.0	0.0	0.0	0.0	0.0	0.0
9ff9d5d3ca6406f1d0e9e20e418dcd60	82.0	0.0	22.0	0.0	17.0	0.0	32.0	5.0	3.0	0.0	4.0	0.0
a2aca8e3eb27105a2d1823fa35e6529d	0.0	234.0	7.0	0.0	8.0	0.0	2.0	0.0	0.0	0.0	0.0	0.0
c7d75e9be7b2b99262b2fe8c8d1d9b10	0.0	0.0	11.0	0.0	0.0	0.0	0.0	13.0	0.0	0.0	0.0	0.0
7a70b2d33a650c2a65cf14c44e4db9eb	0.0	0.0	5.0	0.0	0.0	0.0	0.0	0.0	0.0	0.0	0.0	0.0
2b125bc4f464e30d8ded1bb014064de7	0.0	0.0	0.0	0.0	0.0	0.0	0.0	0.0	0.0	0.0	2.0	0.0
11440cbdc404e5ef95e0a64385c6dafd	95.0	0.0	0.0	0.0	99.0	0.0	0.0	22.0	0.0	0.0	19.0	0.0
e944296aa600a1f7de737dca618bab51	8.0	0.0	0.0	0.0	0.0	0.0	0.0	0.0	0.0	0.0	0.0	0.0
47f775f713e15ac26ccf3c6f17e741c4	0.0	0.0	0.0	0.0	0.0	7.0	0.0	0.0	0.0	0.0	0.0	0.0
886103dbbb06c72b25d03076599bf55c	0.0	0.0	0.0	0.0	0.0	0.0	0.0	2.0	0.0	0.0	0.0	0.0
374dbcdc4fad33d79ec275ade52e1607	0.0	0.0	0.0	0.0	0.0	12.0	0.0	0.0	0.0	0.0	0.0	0.0
0ef3970eed04b66708057b8d7f73b52d	315.0	62.0	359.0	117.0	404.0	188.0	570.0	457.0	62.0	299.0	48.0	144.0
ea415fd351e80409ffc80337ade96ace	0.0	0.0	0.0	0.0	0.0	0.0	0.0	0.0	0.0	7.0	0.0	0.0
5939bf468a4c192790877fe5aaba5b70	0.0	0.0	0.0	0.0	0.0	0.0	0.0	0.0	0.0	5.0	0.0	0.0
02e1407e50c87e5118e6bec8e71a567d	4.0	0.0	0.0	0.0	0.0	0.0	0.0	0.0	0.0	0.0	0.0	0.0
1ddea4c9b5236999682be6134f57b9b3	4.0	0.0	0.0	0.0	0.0	0.0	0.0	0.0	0.0	0.0	0.0	0.0
508bdc8fca7bc4f7ce8fc081bb747f2d	0.0	0.0	19.0	0.0	0.0	0.0	0.0	0.0	0.0	0.0	0.0	0.0
6995743b09b32c3ee5e4266d63c38b07	0.0	0.0	0.0	0.0	0.0	0.0	10.0	0.0	4.0	0.0	0.0	0.0
c36a67b450ad256a123820881c49cff4	22.0	0.0	7.0	0.0	0.0	0.0	0.0	0.0	0.0	0.0	0.0	0.0
4e0ef4ad2ce89368d9d476dc8be0875c	0.0	0.0	5.0	0.0	0.0	3.0	0.0	14.0	0.0	0.0	0.0	0.0
3459249b3b927540a2a0d5524993da8b	0.0	0.0	0.0	0.0	5.0	0.0	0.0	0.0	2.0	0.0	0.0	0.0
36b8f752a95e54de01760e34b2015a84	0.0	0.0	0.0	0.0	0.0	0.0	0.0	20.0	0.0	0.0	0.0	0.0
7657724b91330e48098e6549e7c5cc97	0.0	3.0	0.0	0.0	0.0	0.0	0.0	0.0	0.0	0.0	0.0	0.0
b972960247410ef94a07db21cca8693b	0.0	5.0	0.0	0.0	0.0	0.0	0.0	0.0	0.0	0.0	0.0	0.0
e61ccc6bda8a0c2e46217c6d1b7618a9	0.0	0.0	0.0	3.0	0.0	0.0	0.0	0.0	0.0	0.0	0.0	0.0

#OTU ID	L1_ASR5i	L1_WT	L2_ASR5i	L2_WT	L3_ASR5i	L3_WT	L4_ASR5i	L4_WT	L5_ASR5i	L5_WT	L6_ASR5i	L6_WT
24c1f8ed1f70e1bef8f37cc55abbe5c1	0.0	0.0	0.0	0.0	0.0	0.0	0.0	0.0	0.0	3.0	0.0	0.0
f4e157223289693a26efb7a9098cf13d	0.0	0.0	0.0	0.0	0.0	0.0	0.0	19.0	0.0	0.0	0.0	0.0
37566a284b18cd409744d4ad6d5c3ad8	0.0	0.0	0.0	0.0	0.0	0.0	0.0	0.0	0.0	0.0	0.0	3.0
4794eb8e5a6009a80cfd131672f8e09f	0.0	0.0	0.0	0.0	0.0	0.0	9.0	0.0	0.0	0.0	0.0	0.0
9512d7d9bc1ac7df910847ec1afb10aa	0.0	0.0	888.0	0.0	0.0	0.0	0.0	0.0	0.0	0.0	0.0	0.0
36468d25f18e907430be71de85c62b89	0.0	0.0	5.0	0.0	0.0	0.0	0.0	4.0	0.0	0.0	0.0	0.0
4eef2b0f21d87364115fc49a9691cf2a	7.0	0.0	7.0	0.0	0.0	0.0	0.0	0.0	0.0	0.0	0.0	0.0
ad8d5a8f60aecc975a0b299d27cc1b0c	0.0	0.0	2.0	0.0	0.0	0.0	0.0	0.0	0.0	0.0	0.0	0.0
a2132d33fbe2b6d113bcd3f0532cc152	0.0	0.0	0.0	0.0	7.0	0.0	0.0	0.0	0.0	0.0	0.0	0.0
6d618695af894f56953ef22243d6b6c9	0.0	0.0	0.0	0.0	0.0	0.0	2.0	0.0	0.0	0.0	0.0	0.0
c74ce4bc7ca262a9cc7f34739addf890	0.0	0.0	0.0	0.0	0.0	16.0	0.0	24.0	0.0	0.0	0.0	0.0
9b4d0041ae3b7e0d691ae117d633b4b3	4.0	0.0	0.0	0.0	0.0	0.0	0.0	0.0	7.0	0.0	0.0	0.0
8bc4fbf031223a588edeee4eb57a183b	0.0	0.0	0.0	3.0	0.0	0.0	0.0	0.0	0.0	0.0	0.0	0.0
450431393a605b03c7bf1e66263e4814	0.0	0.0	0.0	0.0	0.0	0.0	6.0	0.0	0.0	0.0	0.0	0.0
68f092b457b129beb0ba4cc81ba97fc5	0.0	0.0	0.0	0.0	2.0	0.0	18.0	0.0	0.0	0.0	0.0	0.0
b55611eeff70c0ea3a1cd600db61ccf7	0.0	0.0	0.0	0.0	0.0	0.0	14.0	0.0	0.0	0.0	0.0	0.0
9e87bee48d8e450cb03fd2bcf4bb32b5	0.0	0.0	0.0	0.0	0.0	0.0	0.0	44.0	0.0	0.0	0.0	0.0
c54682d6d0929616c74715062390c067	6.0	0.0	0.0	0.0	0.0	0.0	0.0	0.0	0.0	0.0	0.0	0.0
74c73ae2bd3401bbc6981283dc94573c	0.0	0.0	0.0	0.0	2.0	0.0	0.0	0.0	0.0	0.0	0.0	0.0
d41e5631816777d8ed0f81f2e742b097	0.0	0.0	6.0	0.0	0.0	0.0	0.0	0.0	0.0	0.0	0.0	0.0
92a482c8fa131c8f3b9a9c30bff22d16	0.0	0.0	0.0	0.0	0.0	0.0	0.0	8.0	0.0	0.0	0.0	0.0
794adc9c2810f6172c5be7c0c60c3e6a	0.0	0.0	0.0	0.0	0.0	0.0	0.0	0.0	0.0	0.0	2.0	0.0
e893266fb2a57cf06cd59aa8e9db7103	2.0	0.0	0.0	0.0	0.0	0.0	0.0	0.0	0.0	0.0	0.0	0.0
f97b1acaa9c241b364ad4d93c1f4b3bb	4.0	0.0	0.0	0.0	0.0	0.0	0.0	0.0	0.0	0.0	0.0	0.0
d355995048adb8b0c7202e24bbc859d5	0.0	0.0	8.0	0.0	0.0	0.0	0.0	0.0	0.0	0.0	0.0	0.0
d6576f8bd76f9487cc13c535d086509e	0.0	0.0	0.0	0.0	3.0	0.0	0.0	0.0	0.0	0.0	0.0	0.0
fa63e6f474357ca8dcb45f7070db60a5	0.0	0.0	0.0	0.0	0.0	0.0	0.0	0.0	3.0	0.0	0.0	0.0
99932cfdc7933cb661cab9f6877e8dfa	0.0	0.0	0.0	8.0	0.0	0.0	0.0	0.0	0.0	0.0	0.0	0.0
a8ae5d0776b3c45dd13e0213204e9b99	0.0	0.0	4.0	0.0	0.0	0.0	0.0	0.0	0.0	0.0	0.0	0.0

#OTU ID	L1_ASR5i	L1_WT	L2_ASR5i	L2_WT	L3_ASR5i	L3_WT	L4_ASR5i	L4_WT	L5_ASR5i	L5_WT	L6_ASR5i	L6_WT
cd7d19e86872b636ac4bd31edd86df67	0.0	0.0	0.0	0.0	0.0	5.0	0.0	0.0	0.0	0.0	0.0	0.0
57333daf85d0ad336104bb3844a3d10a	6.0	0.0	0.0	0.0	0.0	0.0	0.0	0.0	0.0	0.0	0.0	0.0
beb8bfabe0ae66134b9d7fefd9477a19	0.0	0.0	0.0	0.0	0.0	0.0	0.0	0.0	0.0	2.0	0.0	0.0
b3ae392748f9bc92a82b71255f6da7fa	0.0	0.0	0.0	0.0	0.0	0.0	0.0	0.0	0.0	7.0	0.0	0.0
204c201f0a40ed5c1b0fced3bf251de1	0.0	0.0	0.0	0.0	0.0	0.0	0.0	0.0	3.0	0.0	0.0	0.0
7d9312bc86b8d8ab32b29e5fcdac3064	0.0	0.0	0.0	11.0	0.0	0.0	0.0	0.0	0.0	0.0	0.0	0.0
37dc9491775e22ca4cb0070420094319	0.0	0.0	2.0	0.0	0.0	0.0	4.0	0.0	0.0	0.0	0.0	0.0
74b383e1d36a6525d381685d2437c3e4	0.0	0.0	0.0	0.0	0.0	0.0	0.0	0.0	2.0	0.0	0.0	0.0
7f60934fdbe831eb4f70e8cb9bf7951c	0.0	5.0	0.0	0.0	0.0	0.0	0.0	0.0	0.0	0.0	0.0	0.0
10f48f2dd6ab91ef173df1dbd4070c15	4.0	6.0	0.0	0.0	9.0	0.0	0.0	0.0	4.0	0.0	0.0	3.0
c81c3321482d6fd0601be5a37a3b150f	0.0	0.0	0.0	0.0	0.0	0.0	0.0	10.0	0.0	0.0	0.0	0.0
3878cf48e581dba0e8a27cfa9c190bf2	0.0	3.0	0.0	3.0	5.0	0.0	0.0	0.0	0.0	0.0	0.0	0.0
a6b4752f21deddd1c69131a8c6807af1	0.0	0.0	0.0	74.0	0.0	9.0	8.0	0.0	0.0	0.0	0.0	0.0
8551ab46faedb5ee73cdc6f2f9e3cdcb	0.0	0.0	0.0	0.0	0.0	0.0	10.0	0.0	0.0	0.0	0.0	0.0
5b7f29e3669f30debd3a5aeef17ec198	0.0	0.0	0.0	0.0	14.0	0.0	0.0	0.0	0.0	0.0	0.0	0.0
02c8e747312684dedf53f83971dbcad9	0.0	0.0	0.0	0.0	8.0	0.0	0.0	0.0	0.0	0.0	0.0	0.0
d883eb4b5890ff7609808dc251f0136c	0.0	0.0	0.0	0.0	19.0	0.0	0.0	0.0	0.0	0.0	0.0	0.0
e4ae6c2f010d1bfa31515ad8a8ca6cd2	11.0	0.0	0.0	0.0	8.0	0.0	0.0	0.0	0.0	0.0	0.0	0.0
168690d74595afc30d401ee5d2706aae	2.0	0.0	0.0	0.0	0.0	0.0	0.0	0.0	0.0	0.0	0.0	0.0
8d0dcb47ae9585f4a9430d029aab6355	0.0	0.0	0.0	0.0	0.0	0.0	9.0	0.0	0.0	0.0	0.0	0.0
666ae6331d058d655cf416751313779b	0.0	0.0	0.0	0.0	0.0	0.0	0.0	0.0	0.0	31.0	4.0	0.0
52932e2c7963f191df70c53a5e8eab3c	0.0	0.0	0.0	0.0	0.0	0.0	0.0	0.0	0.0	0.0	3.0	0.0
860df414cf1bd336f6fcad5b593beea2	0.0	0.0	0.0	0.0	0.0	0.0	4.0	0.0	0.0	0.0	0.0	0.0
f02492e769292cbb03bd8d931a8498d6	0.0	0.0	0.0	0.0	0.0	0.0	0.0	0.0	2.0	0.0	0.0	0.0
589e0ab5ef28174e7aa8cb88e9c8f601	0.0	0.0	5.0	0.0	0.0	0.0	0.0	0.0	0.0	0.0	0.0	0.0
52a9a1ce4fa220361b8521f3a4dc33e1	0.0	0.0	0.0	0.0	4.0	0.0	0.0	0.0	0.0	0.0	0.0	0.0
0ebd1a038fed6d4e587b6a9bf052f850	0.0	0.0	0.0	3.0	0.0	0.0	0.0	0.0	0.0	0.0	0.0	0.0
1cd49abe3fd42f51eb392f482d3e910a	0.0	0.0	0.0	0.0	4.0	0.0	7.0	0.0	0.0	0.0	0.0	0.0
c2d31f25e1d39a5b9f434765da275af8	0.0	0.0	0.0	0.0	2.0	0.0	0.0	0.0	0.0	0.0	0.0	0.0

#OTU ID	L1_ASR5i	L1_WT	L2_ASR5i	L2_WT	L3_ASR5i	L3_WT	L4_ASR5i	L4_WT	L5_ASR5i	L5_WT	L6_ASR5i	L6_WT
963be4626671dc240867c3f9873f7ad0	0.0	0.0	0.0	0.0	5.0	28.0	0.0	0.0	0.0	0.0	0.0	0.0
74c8395991988dd0a0af3574760757bd	2.0	0.0	0.0	0.0	0.0	0.0	0.0	0.0	0.0	0.0	0.0	0.0
2a5ade4be3f3f3b1be8fbb6e281447da	0.0	7.0	10.0	0.0	0.0	0.0	0.0	0.0	0.0	5.0	0.0	0.0
7ade28ab70d20e826c92e9212107d409	0.0	0.0	5.0	0.0	0.0	0.0	0.0	0.0	0.0	0.0	0.0	0.0
6f867a6dc6c08ec22235904fd9dc649f	0.0	0.0	0.0	0.0	0.0	0.0	5.0	0.0	0.0	0.0	0.0	0.0
c7e221f40a593312e7de4a5d2ccae049	0.0	0.0	0.0	0.0	5.0	0.0	0.0	0.0	0.0	0.0	0.0	0.0
8b98ddd9be89173ef537f445afb3cc76	0.0	0.0	0.0	0.0	0.0	0.0	0.0	8.0	0.0	0.0	0.0	0.0
61a7192bb65b52af06900d57d42f3142	0.0	0.0	7.0	0.0	0.0	0.0	0.0	0.0	0.0	0.0	0.0	0.0
a60e98d57ebf7a42a5332d9c20dec3a5	8.0	0.0	0.0	0.0	0.0	0.0	0.0	0.0	0.0	0.0	0.0	0.0
6845f026563c3f0b7c2ff0509adde2e6	0.0	0.0	0.0	0.0	0.0	0.0	0.0	5.0	0.0	0.0	0.0	0.0
c64aaa81354595b3e423be6939c243bb	0.0	0.0	0.0	0.0	0.0	0.0	7.0	0.0	0.0	0.0	0.0	0.0
243b7e872a49f50889f76a6ea60f722a	0.0	0.0	5.0	0.0	0.0	0.0	0.0	0.0	0.0	0.0	0.0	0.0
8a9d9f7411938425481c1976cd96c26f	3.0	0.0	0.0	0.0	0.0	0.0	0.0	0.0	0.0	5.0	0.0	0.0
6d33ea3187249185f4f10e5df2fed12f	0.0	0.0	0.0	0.0	8.0	0.0	0.0	0.0	0.0	0.0	0.0	0.0
49575752047bc3f1efe5299201a3c50e	3.0	0.0	0.0	0.0	0.0	0.0	0.0	0.0	0.0	0.0	0.0	0.0
cfc623dc92bec30bf4db1955d5f9dc74	3.0	0.0	0.0	0.0	0.0	0.0	0.0	0.0	0.0	0.0	0.0	0.0
ede4d6afc6ccdebf6aaf66a8ec347f4a	0.0	4.0	0.0	0.0	13.0	0.0	0.0	5.0	0.0	0.0	0.0	0.0
5861e907d4cf7e6b6f65f3f898095858	0.0	0.0	0.0	0.0	11.0	0.0	0.0	0.0	0.0	0.0	0.0	0.0
8011f1bd954f3f94f918190082880061	0.0	0.0	0.0	0.0	0.0	0.0	0.0	14.0	0.0	0.0	0.0	0.0
8d8211845960bd8e05d83a710e00e37a	0.0	0.0	0.0	7.0	0.0	0.0	0.0	0.0	0.0	0.0	0.0	0.0
1e2fe5b38c2c7ff24a268e4bde29d9b7	0.0	0.0	5.0	0.0	0.0	0.0	0.0	0.0	0.0	0.0	0.0	0.0
66d9195b5fc8c0e0534a4bce0e9c2902	0.0	0.0	0.0	0.0	0.0	0.0	0.0	0.0	0.0	0.0	7.0	0.0
bd64b401a0370ff8ff2c1f5eb82b7e31	0.0	0.0	0.0	0.0	0.0	0.0	0.0	0.0	2.0	0.0	0.0	0.0
1038e6679186bf5150678e00464f23f8	2.0	0.0	0.0	0.0	0.0	0.0	0.0	0.0	0.0	0.0	0.0	0.0
7a043cb7877c4917e8382384d43425e9	3.0	0.0	0.0	0.0	0.0	0.0	0.0	0.0	0.0	0.0	0.0	0.0
739559b8733ccf6e027fc28c5d0f4b0e	3.0	0.0	0.0	0.0	0.0	0.0	0.0	0.0	0.0	0.0	0.0	0.0
83ebd76ff5e22c790f65805a7dfe587c	2.0	0.0	12.0	0.0	0.0	0.0	0.0	0.0	0.0	0.0	0.0	0.0
2ff9cdeec44c83a252cc4fc2eceaf346	0.0	0.0	0.0	0.0	0.0	0.0	0.0	0.0	0.0	4.0	0.0	0.0
8e86548f11e10a95f5ca8f635027db1a	0.0	0.0	25.0	0.0	15.0	0.0	0.0	0.0	0.0	0.0	0.0	0.0

#OTU ID	L1_ASR5i	L1_WT	L2_ASR5i	L2_WT	L3_ASR5i	L3_WT	L4_ASR5i	L4_WT	L5_ASR5i	L5_WT	L6_ASR5i	L6_WT
519c2004082f623d11f4e8488bcac5ee	5.0	0.0	0.0	0.0	2.0	0.0	0.0	0.0	0.0	0.0	0.0	0.0
05d1148297e42e552f99f9625e6e6f2b	0.0	0.0	4.0	0.0	0.0	0.0	0.0	0.0	0.0	0.0	0.0	0.0
12671e7a7dc07385bd8a738664f1f117	12.0	0.0	0.0	0.0	0.0	0.0	0.0	0.0	0.0	0.0	0.0	0.0
bdfb2ea4cb3e7b981042bc1f55c11806	7.0	0.0	0.0	0.0	0.0	0.0	0.0	0.0	0.0	0.0	0.0	0.0
21126bb02587d5eda21cc4a8e5b461b4	7.0	0.0	0.0	0.0	0.0	0.0	0.0	0.0	0.0	0.0	0.0	0.0
41097a50d17ab6862bb245d990e6e616	0.0	4.0	3.0	0.0	0.0	0.0	0.0	0.0	0.0	0.0	0.0	0.0
23bed36498aff1a75b8a84ee37d39eaa	0.0	0.0	0.0	0.0	2.0	0.0	0.0	0.0	0.0	0.0	0.0	0.0
f3d99027f1db7cf8b662eb3ebb2d833e	0.0	0.0	0.0	0.0	0.0	0.0	0.0	0.0	0.0	0.0	0.0	11.0
db0d503b3f69e1de0853907923df9d99	0.0	0.0	0.0	0.0	0.0	0.0	0.0	0.0	0.0	0.0	2.0	0.0
1262df956aa878717eee609b5001828b	0.0	0.0	0.0	0.0	0.0	4.0	0.0	0.0	0.0	3.0	0.0	0.0
39aad405a145d5e45b5f61e6a914c392	0.0	0.0	0.0	2.0	0.0	0.0	0.0	0.0	0.0	0.0	0.0	0.0
e3430a64b5213ecb3972356f12617307	0.0	11.0	0.0	0.0	0.0	0.0	0.0	0.0	0.0	0.0	0.0	0.0
c0b839d7b688201d42f47dba60941890	0.0	4.0	0.0	3.0	0.0	0.0	0.0	0.0	0.0	0.0	0.0	0.0
c1fb84a1c5664e6b6b5513317303ab0c	0.0	0.0	0.0	0.0	2.0	0.0	0.0	0.0	0.0	0.0	0.0	0.0
9da0ecdcdb2bcd6661ae09db58254358	8.0	0.0	5.0	0.0	0.0	0.0	0.0	0.0	0.0	0.0	0.0	0.0
74347c829d2cb346721dd4197aae9d40	0.0	0.0	0.0	0.0	0.0	0.0	0.0	0.0	0.0	8.0	0.0	0.0
00a3425c63f224ad666d56cacf0b5903	0.0	2.0	0.0	0.0	0.0	0.0	0.0	0.0	0.0	0.0	0.0	0.0
cdba69264af69a3d035fb380d8b3b813	0.0	0.0	0.0	0.0	10.0	0.0	0.0	0.0	0.0	0.0	0.0	0.0
7eee74dffa9735b1c10944f4ed91ef27	0.0	0.0	0.0	0.0	0.0	11.0	0.0	0.0	0.0	0.0	0.0	0.0
d0b108b9fce55986814978665ec6b747	0.0	0.0	0.0	0.0	15.0	0.0	0.0	0.0	0.0	0.0	0.0	0.0
c437699b2197638185d93d1fdacf79c2	0.0	0.0	0.0	0.0	0.0	0.0	0.0	0.0	0.0	0.0	0.0	6.0
ed2f9b886bcccf27fc002a2a64a83433	0.0	0.0	0.0	0.0	0.0	0.0	0.0	0.0	0.0	0.0	4.0	0.0
9d9c8654bdafa9ccbaeccb9210fa4824	0.0	0.0	0.0	0.0	0.0	0.0	4.0	0.0	0.0	0.0	0.0	0.0
5f3049043071e42c4f8ddd9a5189e3fa	0.0	0.0	4.0	0.0	9.0	0.0	0.0	0.0	0.0	0.0	0.0	0.0
e3498665592003d4fce5b1fbabf9accb	0.0	0.0	2.0	0.0	0.0	0.0	0.0	0.0	0.0	0.0	0.0	0.0
b837ee603bcaa5ca070e6461e2f4afbf	0.0	0.0	0.0	0.0	0.0	0.0	5.0	0.0	0.0	0.0	0.0	0.0
18a1bd2b70de9fcf246fd510813e6e04	0.0	3.0	0.0	0.0	0.0	0.0	0.0	0.0	0.0	0.0	0.0	0.0
9eb17e6894a27bfa2338e18c4b2275a9	0.0	0.0	0.0	0.0	0.0	0.0	0.0	0.0	0.0	2.0	0.0	0.0
d586507a646392586bad913768e074c6	0.0	0.0	0.0	0.0	6.0	0.0	0.0	0.0	0.0	0.0	0.0	0.0

#OTU ID	L1_ASR5i	L1_WT	L2_ASR5i	L2_WT	L3_ASR5i	L3_WT	L4_ASR5i	L4_WT	L5_ASR5i	L5_WT	L6_ASR5i	L6_WT
d06fa41247a6ba9fe50a1f2be1340d8a	0.0	0.0	0.0	0.0	0.0	0.0	0.0	0.0	0.0	0.0	2.0	0.0
bf44309c5a465fff43f10b26d729576a	0.0	8.0	0.0	0.0	0.0	0.0	0.0	0.0	0.0	8.0	0.0	0.0
9a1d9d6b080e7257c60890d25dc2e4f7	0.0	13.0	0.0	0.0	0.0	0.0	0.0	0.0	0.0	0.0	0.0	0.0
c5ade7be62d27c100e1f4d0e03b6efd6	0.0	0.0	0.0	0.0	0.0	0.0	0.0	0.0	0.0	0.0	0.0	2.0
acc8892b8078de57dddc853b59de4cc0	0.0	0.0	3.0	0.0	0.0	0.0	0.0	0.0	0.0	0.0	0.0	0.0
58ed7259ed490012bb107188a95e5cd1	0.0	0.0	0.0	0.0	0.0	0.0	0.0	15.0	0.0	0.0	0.0	0.0
cb1c19ce6ccc5c7f2c690275bbc6f61d	0.0	0.0	6.0	0.0	0.0	0.0	0.0	0.0	0.0	0.0	0.0	0.0
8297790565be533410b5e3148cb10ee9	14.0	0.0	0.0	0.0	0.0	0.0	0.0	0.0	0.0	0.0	0.0	0.0
01cc171fd99f4fa33eb865ae33406d04	0.0	0.0	0.0	0.0	0.0	0.0	0.0	0.0	0.0	7.0	0.0	0.0
42c54ca826296dd98c7a6e5cbb11f734	0.0	0.0	0.0	0.0	0.0	0.0	8.0	0.0	0.0	0.0	0.0	0.0
2adb09f346f7493e0ed9963bc03475f5	4.0	0.0	0.0	0.0	0.0	0.0	0.0	0.0	0.0	0.0	0.0	0.0
3c46e2b3702000e769f55fee7bf95421	0.0	0.0	0.0	0.0	0.0	0.0	0.0	7.0	0.0	0.0	0.0	0.0
ca5cd76f1b795b1e7d2eeadfd7fc5d98	0.0	0.0	0.0	0.0	0.0	0.0	0.0	0.0	0.0	0.0	2.0	0.0
bdb0a8bd5d73122c2a7cd89c57bae9e6	0.0	7.0	0.0	0.0	0.0	0.0	0.0	0.0	0.0	0.0	0.0	0.0
d5170e42023e42f9f52309aa7f5a2028	0.0	0.0	0.0	0.0	3.0	0.0	0.0	0.0	0.0	0.0	0.0	0.0
db077c825f235b11f034f0de2701b3bf	0.0	4.0	0.0	0.0	0.0	0.0	0.0	0.0	0.0	0.0	0.0	0.0
84a09ca661c705d6d0c847d9d4b015d5	22.0	0.0	0.0	0.0	0.0	0.0	0.0	0.0	0.0	0.0	0.0	0.0
4142213994e9fe299577403cf10fbfa7	0.0	4.0	0.0	0.0	0.0	0.0	0.0	0.0	0.0	0.0	0.0	0.0
bfddf853ef9d6482cf64b19f9c2e96fd	0.0	0.0	0.0	0.0	0.0	0.0	2.0	0.0	0.0	0.0	0.0	0.0
b68b07b290b2610982017fe8f9eb2b8f	0.0	0.0	0.0	0.0	0.0	0.0	5.0	0.0	0.0	0.0	0.0	0.0
7fa38ac50d9b450fca54a58ff92713fb	0.0	0.0	0.0	0.0	6.0	0.0	0.0	0.0	0.0	0.0	0.0	0.0
ea9c7d0d565244719ca7537af6e8fa27	0.0	0.0	0.0	0.0	6.0	0.0	0.0	0.0	0.0	0.0	0.0	0.0
c3a39830e9a1c8245708ea1ad0bf46d1	0.0	7.0	0.0	0.0	0.0	0.0	0.0	0.0	0.0	0.0	0.0	0.0
9f7d26f9013f8484829719cf44a4acab	0.0	0.0	0.0	0.0	0.0	0.0	0.0	13.0	0.0	0.0	0.0	0.0
84ee18e0fbbdd6d7102c91a4a2362d16	0.0	0.0	0.0	0.0	13.0	0.0	0.0	0.0	0.0	0.0	0.0	0.0
c84e381e28fa8e0d4f63f38ba9af56e1	0.0	0.0	0.0	0.0	9.0	0.0	0.0	0.0	0.0	0.0	0.0	0.0
21642f4c6ba839ff05aab540b8223a43	0.0	0.0	0.0	0.0	0.0	0.0	0.0	4.0	0.0	0.0	0.0	0.0
8b64957caea558c1c554c3264e4f6860	0.0	0.0	0.0	0.0	0.0	0.0	0.0	0.0	0.0	0.0	0.0	6.0
14bdd5c7e17a0f654126e6ebdb3bdafa	2.0	0.0	0.0	0.0	0.0	0.0	0.0	0.0	0.0	0.0	0.0	0.0

#OTU ID	L1_ASR5i	L1_WT	L2_ASR5i	L2_WT	L3_ASR5i	L3_WT	L4_ASR5i	L4_WT	L5_ASR5i	L5_WT	L6_ASR5i	L6_WT
e3ffc886f657b0003e9493c2cb04ceb1	0.0	0.0	0.0	0.0	3.0	0.0	0.0	0.0	0.0	0.0	0.0	0.0
c8d44c711ab2fc7e88562dff9f037e1a	2.0	0.0	0.0	0.0	0.0	0.0	0.0	0.0	0.0	0.0	0.0	0.0
ecd8ce8c50ab47f35ba375fa5bf5e5e2	0.0	0.0	4.0	0.0	0.0	0.0	0.0	0.0	0.0	0.0	0.0	0.0
876ae5139aea40b6f4fa857fca69c56a	5.0	0.0	0.0	0.0	0.0	0.0	0.0	0.0	0.0	0.0	0.0	0.0
878939b3ee1a961122c8353fccf7b044	2.0	0.0	0.0	0.0	0.0	0.0	0.0	0.0	0.0	0.0	0.0	0.0
250b7a46ab1afeb60ea5dfa8ad7bd1f8	0.0	0.0	0.0	0.0	0.0	0.0	5.0	0.0	0.0	0.0	0.0	0.0
43b29f0724c6a15c04e331497f343623	0.0	0.0	2.0	0.0	4.0	0.0	0.0	0.0	0.0	0.0	0.0	0.0
34354581336885281 66aec5c75d23efa	0.0	0.0	0.0	0.0	9.0	0.0	0.0	0.0	0.0	0.0	0.0	0.0
440ee2918db69400c962ab2b54e80045	6.0	0.0	0.0	0.0	0.0	0.0	0.0	0.0	0.0	0.0	0.0	0.0
53fbbf4000bf1315b2c436120c3fd4c3	0.0	0.0	0.0	3.0	4.0	0.0	0.0	0.0	0.0	0.0	0.0	0.0
79f5f6ee44e1421a9f9cf6878697cd9a	0.0	0.0	0.0	0.0	0.0	0.0	0.0	0.0	0.0	0.0	0.0	2.0
74fa2d385f58c2fa83c2a2dc6ac264c6	3.0	0.0	0.0	0.0	0.0	0.0	0.0	0.0	0.0	0.0	0.0	0.0
8d4f1bb808993449a5d46fcdcce94d19	3.0	0.0	0.0	0.0	0.0	0.0	0.0	0.0	0.0	0.0	0.0	0.0
3fda336b7731172724cc86cc0822f3bd	4.0	0.0	0.0	0.0	0.0	0.0	0.0	0.0	0.0	0.0	0.0	0.0
cc5ba89697fcad5368d1e599d09c5070	0.0	0.0	0.0	0.0	0.0	0.0	7.0	0.0	0.0	0.0	0.0	0.0
d25a221b925762c100a702adaed3842b	2.0	0.0	0.0	0.0	0.0	11.0	0.0	4.0	4.0	0.0	0.0	0.0
5a8d944f0adc92088d6c0cf9181f417a	0.0	8.0	0.0	0.0	0.0	0.0	0.0	0.0	0.0	0.0	0.0	0.0
535178ed1d55b8b448c2aebaa083481a	0.0	0.0	0.0	0.0	0.0	0.0	4.0	0.0	0.0	0.0	0.0	0.0
4d509fb6a93e03e99a7bead306d468a5	9.0	0.0	0.0	0.0	0.0	0.0	0.0	0.0	0.0	0.0	0.0	0.0
c05b79c493e59e725b1ef1b14e83f896	0.0	61.0	8.0	0.0	0.0	0.0	0.0	0.0	0.0	0.0	0.0	0.0
aadd481277481c9c35e2c988f7798fce	0.0	0.0	0.0	0.0	0.0	0.0	10.0	0.0	0.0	0.0	0.0	0.0
8d08614160e4c018ef9c6015611e6e85	0.0	0.0	0.0	0.0	0.0	0.0	7.0	0.0	0.0	0.0	0.0	0.0
5fbae463e264b482be83496006d5d34e	0.0	0.0	0.0	0.0	0.0	0.0	0.0	0.0	0.0	0.0	2.0	0.0
dd363efefad571478564b1b2a5ec06a5	0.0	2.0	0.0	0.0	0.0	0.0	0.0	0.0	0.0	0.0	0.0	0.0
4a771be3e1fa74bde3263c6a429f7aaf	4.0	0.0	0.0	0.0	0.0	0.0	0.0	0.0	0.0	0.0	0.0	0.0
1e9fccbfc9ade04cb0bfc25fcfe131db	0.0	0.0	2.0	0.0	0.0	0.0	0.0	0.0	0.0	0.0	0.0	0.0
be6d75c8ea6106b7dff5c4b75d7c6dae	0.0	0.0	0.0	0.0	6.0	0.0	0.0	0.0	0.0	0.0	0.0	0.0
839f2509337a14ed399c33b5c001cd63	19.0	0.0	55.0	2.0	0.0	0.0	0.0	0.0	0.0	0.0	0.0	0.0
ac825e99b482dcce38b77c23f868524b	0.0	0.0	4.0	0.0	0.0	0.0	0.0	0.0	0.0	0.0	0.0	0.0

#OTU ID	L1_ASR5i	L1_WT	L2_ASR5i	L2_WT	L3_ASR5i	L3_WT	L4_ASR5i	L4_WT	L5_ASR5i	L5_WT	L6_ASR5i	L6_WT
23ba0112d42f6ebe5f84013ea4b9e0bb	10.0	0.0	0.0	0.0	0.0	0.0	0.0	0.0	0.0	0.0	0.0	0.0
a792390bfd926d4656eae5375a0f9f47	2.0	0.0	0.0	0.0	0.0	0.0	0.0	0.0	0.0	0.0	0.0	0.0
ef122ebc71d03754dddd60a8836cd25f	0.0	3.0	0.0	0.0	0.0	0.0	0.0	16.0	0.0	0.0	0.0	0.0
a9a34ea04a580e573f00e445cc96e925	0.0	0.0	0.0	0.0	0.0	0.0	3.0	0.0	0.0	0.0	0.0	0.0
310fd5f7a6b7a349c05fe21c3a398e65	0.0	0.0	0.0	0.0	0.0	0.0	0.0	0.0	2.0	0.0	0.0	0.0
64c3e14efcd170f424e271f2318081d7	0.0	0.0	8.0	0.0	7.0	0.0	0.0	0.0	5.0	0.0	0.0	0.0
248385e5d9c90bd00334a543546644de	0.0	2.0	0.0	0.0	0.0	0.0	0.0	0.0	0.0	0.0	0.0	0.0
c69e6ade02a0398efad9ea57ca010640	0.0	0.0	0.0	0.0	0.0	0.0	11.0	0.0	0.0	0.0	0.0	0.0
0189b5da357aa4e5b7c5e9a22e226dae	0.0	0.0	0.0	0.0	15.0	0.0	0.0	0.0	0.0	0.0	0.0	0.0
bbf7ef629b5ec650f98b0d360978e794	6.0	0.0	0.0	0.0	0.0	0.0	0.0	0.0	0.0	0.0	0.0	0.0
c0ef672caa81191ecb103d93608400dc	4.0	0.0	0.0	0.0	0.0	0.0	0.0	0.0	0.0	0.0	0.0	0.0
25d792e507bc5296e6e493b5d6c0ac00	2.0	0.0	0.0	0.0	0.0	0.0	0.0	0.0	0.0	0.0	0.0	0.0
6432bfb1f853122a4696874e2ee7deb0	0.0	0.0	0.0	0.0	14.0	0.0	0.0	0.0	0.0	0.0	0.0	0.0
772022362e1695deb5c179d43151125b	0.0	0.0	0.0	0.0	0.0	0.0	0.0	0.0	0.0	0.0	2.0	0.0
0be9aafa096f709fa40abaf6650c65aa	4.0	0.0	0.0	0.0	0.0	0.0	0.0	0.0	0.0	0.0	0.0	0.0
92b7d8acacb3b5938c266df5346e2aec	0.0	0.0	0.0	0.0	6.0	0.0	0.0	0.0	0.0	0.0	0.0	0.0
86f3e4ac4f7b1464bd12e9a4f516d2d9	0.0	0.0	0.0	0.0	0.0	0.0	2.0	0.0	0.0	0.0	0.0	0.0
9af4880c8370d67607ba72eb3d8bc47d	0.0	0.0	6.0	0.0	0.0	0.0	0.0	0.0	0.0	0.0	0.0	0.0
cc377de58067c3f32b5d9a6c6b6b0ba6	0.0	0.0	0.0	0.0	0.0	0.0	0.0	0.0	0.0	0.0	3.0	0.0
ef809c2a506e25c894f2030f836d61c7	6.0	0.0	0.0	0.0	0.0	0.0	0.0	0.0	0.0	0.0	0.0	0.0
0387dd393860e63cf3a465f40462a9ff	5.0	0.0	0.0	0.0	0.0	0.0	0.0	0.0	0.0	0.0	0.0	0.0
4ef1e96eaa25006deea2f9a1fe3fe146	0.0	0.0	0.0	0.0	3.0	0.0	0.0	0.0	0.0	0.0	0.0	0.0
95d8d6cd90e87dd0be34be64761d99f3	0.0	0.0	0.0	0.0	0.0	0.0	0.0	0.0	0.0	0.0	3.0	0.0
69f8ef7bbb222b51afa97d7cf679f97f	0.0	0.0	0.0	0.0	0.0	0.0	0.0	0.0	0.0	0.0	17.0	0.0
1b279b693f910d8bb6eadfd71854254e	0.0	8.0	0.0	0.0	0.0	0.0	0.0	0.0	0.0	0.0	0.0	0.0
680ab3ae874dc5bdd81178f0117ac983	4.0	0.0	0.0	0.0	0.0	0.0	0.0	0.0	0.0	0.0	0.0	0.0
cb0ad063df637dc120b0650c71dbbdf7	7.0	0.0	0.0	0.0	0.0	0.0	0.0	0.0	0.0	0.0	0.0	0.0
c3f4a8b19d3df6c59bf360b35c4bc387	0.0	0.0	8.0	0.0	0.0	0.0	0.0	0.0	0.0	0.0	0.0	0.0
a6db7172c1f6ad27fe95fd7201c6ae23	0.0	0.0	0.0	0.0	0.0	0.0	0.0	0.0	0.0	0.0	0.0	5.0

#OTU ID	L1_ASR5i	L1_WT	L2_ASR5i	L2_WT	L3_ASR5i	L3_WT	L4_ASR5i	L4_WT	L5_ASR5i	L5_WT	L6_ASR5i	L6_WT
a319d6e233a46c4c916f85152e2c029c	0.0	0.0	0.0	0.0	0.0	0.0	20.0	0.0	0.0	0.0	0.0	0.0
b3baa664febb98dbb205bf0b4445b8df	0.0	0.0	0.0	0.0	0.0	0.0	3.0	0.0	0.0	0.0	0.0	0.0
00f6010b35687db6b50ff3043c02c7d0	7.0	0.0	0.0	0.0	0.0	0.0	0.0	0.0	0.0	0.0	0.0	0.0
8b55c8d9b5537765229a11d74e9b9f2d	0.0	0.0	3.0	0.0	0.0	0.0	0.0	0.0	0.0	0.0	0.0	0.0
605fc6a8edf7a389f09e7002c9c269fd	12.0	0.0	0.0	0.0	0.0	0.0	0.0	0.0	0.0	0.0	0.0	0.0
27a444015587d09b597f25953c2493ea	0.0	0.0	6.0	0.0	0.0	0.0	0.0	0.0	0.0	0.0	0.0	0.0
cbf69a84f62fd0624cd640da74adbaf6	0.0	0.0	0.0	0.0	0.0	0.0	0.0	0.0	0.0	0.0	3.0	0.0
417fceede5cdeab1e33cacf68ca7ad78	8.0	0.0	0.0	0.0	0.0	0.0	0.0	0.0	0.0	0.0	0.0	0.0
31b3a9f72a46a21405f60ff08e7a3792	0.0	0.0	0.0	0.0	0.0	0.0	10.0	0.0	0.0	0.0	0.0	0.0
006bffdc09747b4b810247293425c82b	0.0	0.0	0.0	0.0	11.0	0.0	0.0	0.0	0.0	0.0	0.0	0.0
123064e9091a843baeebfb9675719765	0.0	0.0	5.0	0.0	0.0	0.0	0.0	0.0	0.0	0.0	0.0	0.0
9a01df1ebbaca692ec7c7e75ecd99e4a	0.0	0.0	0.0	0.0	4.0	0.0	0.0	0.0	0.0	0.0	0.0	0.0
22e9975344b10cbb348b887338f839b8	0.0	0.0	0.0	0.0	0.0	0.0	0.0	0.0	0.0	4.0	0.0	0.0
f44462f039383280a4927da782209bec	4.0	0.0	0.0	0.0	0.0	0.0	0.0	0.0	0.0	0.0	0.0	0.0
9839e222a3f49a703242d5bde554a77f	0.0	0.0	0.0	0.0	0.0	12.0	0.0	0.0	0.0	0.0	0.0	0.0
fa503c4fa31e98bdd875f25c276dc0d1	3.0	0.0	0.0	0.0	0.0	0.0	0.0	0.0	0.0	0.0	0.0	0.0
7a408ced4b1144ed6d2799f275f005aa	0.0	2.0	0.0	0.0	0.0	0.0	0.0	0.0	0.0	0.0	0.0	0.0
7afeac5e397a6e9ea84c01a6b067a30b	0.0	0.0	0.0	0.0	0.0	0.0	9.0	0.0	0.0	0.0	0.0	0.0
996ee94b7295f641e20965184350ca47	8.0	0.0	0.0	0.0	0.0	0.0	0.0	0.0	0.0	0.0	0.0	0.0
2f1e3dd5f8327944a0d19561c419f809	0.0	0.0	0.0	3.0	0.0	0.0	0.0	0.0	0.0	0.0	0.0	0.0
76ef1dbe389af4357aae53b69c05b2b0	0.0	0.0	0.0	0.0	0.0	0.0	14.0	0.0	0.0	0.0	0.0	0.0
b4402abb3b332577ac7b0e0a94d945ec	0.0	0.0	0.0	0.0	11.0	0.0	0.0	0.0	0.0	0.0	0.0	0.0
10ec83d7c91e0454377dedc50ae3703e	2.0	0.0	0.0	0.0	0.0	0.0	0.0	0.0	0.0	0.0	0.0	0.0
b64c778bc2363d0223b3144d75165148	0.0	0.0	0.0	0.0	0.0	0.0	10.0	0.0	0.0	0.0	0.0	0.0
72c4f27adf1b0b78d5f4d97f909debe6	0.0	0.0	0.0	0.0	0.0	0.0	5.0	0.0	0.0	0.0	0.0	0.0
a9c1f9ce8e5f5c1bd15bab50abd2ee95	0.0	0.0	9.0	0.0	0.0	0.0	0.0	0.0	0.0	0.0	0.0	0.0
b300c85aec83fb77763d4b2e421fe4f4	0.0	0.0	0.0	0.0	0.0	0.0	8.0	0.0	0.0	0.0	0.0	0.0
ef62b7f7f41ab9828ec1d0c2685504e4	0.0	6.0	0.0	0.0	0.0	0.0	0.0	0.0	0.0	0.0	0.0	0.0
d361b91f249066f303cfa659af123874	0.0	0.0	0.0	3.0	0.0	0.0	0.0	0.0	0.0	0.0	0.0	0.0

**#OTU ID**	**L1_ASR5i**	**L1_WT**	**L2_ASR5i**	**L2_WT**	**L3_ASR5i**	**L3_WT**	**L4_ASR5i**	**L4_WT**	**L5_ASR5i**	**L5_WT**	**L6_ASR5i**	**L6_WT**
24270ee166c6eccbac23de22f53236a4	0.0	4.0	0.0	0.0	0.0	0.0	0.0	0.0	0.0	0.0	0.0	0.0
0506b4b62c977af4ce39f0fccddb1c30	8.0	0.0	0.0	0.0	0.0	0.0	0.0	0.0	0.0	0.0	0.0	0.0
8bfb6a62f5db557fa69e06abbee83fb1	0.0	0.0	2.0	0.0	0.0	0.0	0.0	0.0	0.0	0.0	0.0	0.0
a5705a4ad67e5030c145deaf1059b2d7	0.0	3.0	0.0	0.0	0.0	0.0	0.0	0.0	0.0	0.0	0.0	0.0
3fb5bd114359eef54b9daf0a382943e9	0.0	0.0	0.0	0.0	0.0	3.0	0.0	0.0	0.0	0.0	0.0	0.0
7bc0ec2c15aa2d58ac9e5b9773671a34	0.0	0.0	0.0	0.0	0.0	0.0	0.0	0.0	4.0	0.0	0.0	0.0
141211298ebdd6d28941e81126979aa7	0.0	10.0	0.0	0.0	0.0	0.0	0.0	0.0	0.0	0.0	0.0	0.0
e15471c2bacb95080b4e39f75e73ac88	9.0	0.0	0.0	0.0	0.0	0.0	0.0	0.0	0.0	0.0	0.0	0.0
a750ba1be9e9612251447becb084748f	3.0	0.0	0.0	0.0	0.0	0.0	0.0	0.0	0.0	0.0	0.0	0.0
9639df550884cd0a97442114f40c9b59	0.0	0.0	0.0	0.0	0.0	0.0	7.0	0.0	0.0	0.0	0.0	0.0
67215ac3ef6adf437562d75a060bb003	0.0	0.0	0.0	4.0	0.0	0.0	0.0	0.0	0.0	0.0	0.0	0.0
49370a775e675254f7334a1b73a8ebad	0.0	5.0	0.0	0.0	0.0	0.0	0.0	0.0	0.0	0.0	0.0	0.0
00cf82528d288243ee7f9934dc49a3a7	0.0	0.0	12.0	0.0	0.0	0.0	0.0	0.0	0.0	0.0	0.0	0.0
9dfa5ee1c03fa966cdbde6636211b646	5.0	0.0	0.0	0.0	0.0	0.0	0.0	0.0	0.0	0.0	0.0	0.0
b56006bed9aed5f997294ab9eaca8dab	0.0	0.0	0.0	0.0	0.0	0.0	0.0	0.0	0.0	0.0	2.0	0.0
9d9a13d6b700b3c00487784d809b436c	0.0	0.0	0.0	0.0	0.0	0.0	15.0	0.0	0.0	0.0	0.0	0.0
3e025546446de7127f6633526df74211	0.0	0.0	0.0	0.0	0.0	0.0	11.0	0.0	0.0	0.0	0.0	0.0
04f3e2ff174560d4a5b5e7fc98a83b90	0.0	0.0	0.0	0.0	0.0	13.0	0.0	0.0	0.0	0.0	0.0	0.0
0147bf3bec40ae84e685145bac95ef82	0.0	0.0	0.0	0.0	16.0	0.0	0.0	0.0	0.0	0.0	0.0	0.0
5d7db9523d22a014cd405674c4f8e60e	0.0	0.0	9.0	0.0	0.0	0.0	0.0	0.0	0.0	0.0	0.0	0.0
00c03747204af72f1ead18eb1217b099	0.0	0.0	0.0	0.0	0.0	4.0	0.0	0.0	0.0	0.0	0.0	0.0
25c919a961b73751bfd3a3d267885bae	0.0	0.0	0.0	0.0	0.0	0.0	0.0	12.0	0.0	0.0	0.0	0.0
