# Supplementary material for: The impact of OsASR5 on rice microbiome: Implications for nutrient cycling and stress response
Source: Genet Mol Biol. 2026 Oct 26;49(Suppl 3):e20250064. doi: 10.1590/1678-4685-GMB-2025-0064 (PMC13720868; doi:10.1590/1678-4685-GMB-2025-0064)
Supplement: Supplementary file 3 [file 1415-4757-GMB-49-s3-e20250064-s3.pdf]

Genetics and Molecular Biology

https://doi.org/10.1590/1678-4685-GMB-2025-0064

Supplementary Material to "The impact of OsASR5 on rice microbiome: Implications for nutrient cycling and stress response"

Table S3 - OTU abundance per sample from root microbiome**.**

#OTU ID	R1_ASR5i	R1_WT	R2_ASR5i	R2_WT	R3_ASR5i	R3_WT	R4_ASR5i	R4_WT	R5_ASR5i	R5_WT	R6_ASR5i	R6_WT
69dde4568b4c5e3a6aabd3450f55bad1	0	0	0	0	28	0	0	0	0	0	0	0
736cd90e6c106e27260ebc67030fdc30	0	0	0	0	0	0	3	0	0	0	0	0
800add5d706da3980fcc888d8b38fc3e	0	0	0	0	0	0	0	0	0	0	0	2
2ca0a940f4a6b748eb9a35d9cb7e216b	0	0	0	0	0	4	0	0	0	0	0	0
c60d3932e8b4a6cf6fa873461f07b07a	0	0	0	0	0	0	0	0	0	0	48	0
9f8f65b0b829f06ed50d7ddaa9a44e5b	2	0	0	0	0	0	0	0	0	0	0	0
408c1dba7c924dfa0e68c52f881f1bca	0	0	0	0	0	5	0	0	0	0	0	0

9ad2b516dd20881001480cb3eb1dbf47	0	0	0	0	2	0	0	0	0	0	0	0
1955c047c873c95b4e6ce599eb56af35	0	0	0	0	0	0	0	0	0	0	64	0
d71387c421a2c41944939e9822386887	16	0	0	0	0	0	0	0	0	0	0	0
176b1a636c55c4ff1df5243fc4b108c8	0	0	2	0	0	0	0	0	0	0	0	0
998ee957c1e3645099f600fee7518358	0	0	0	0	0	0	0	0	0	29	0	0
0a7a45bffab0d408a3d3cea244dd5869	0	0	0	0	0	0	0	0	0	2	0	0
0b338515acd829476df0e47ee5ef5c84	0	0	0	5	0	0	0	0	0	0	0	0
8a3185b1c00b8f50fbebb40bdd50b415	0	0	0	3	0	0	0	0	0	0	0	0
a94c018172709f1bd1169092a17bce43	0	0	0	0	0	2	0	0	0	0	0	0
071fa7487b2d873935e65687b3cb63ef	0	0	2	0	0	0	0	0	0	0	0	0
662029bb2e3b171955f3f046bd15436e	0	0	0	0	0	0	0	0	0	2	0	0
397a6cd7de40eaa47b036bf5ce02bc24	46	0	0	0	0	0	0	0	0	0	0	0
da458880c87de6e31422d8771cbcfbcf	0	0	0	0	0	0	0	0	0	3	0	0
96325a3a9c3c6972f36f7e962ca33913	0	0	0	0	0	0	0	0	0	0	9	0

8e569856f7db057e677eabcf7af51740	0	0	0	0	0	25	0	0	0	0	0	0
b9a4308f020ae7936e7cad8d12817e96	0	0	14	0	0	0	0	0	0	0	0	0
642b3b8d81d3ffbd0345c0ffe883b9a4	0	0	0	0	0	0	0	0	0	0	0	4
742deeced90ad118433b803097939058	0	0	0	0	0	0	0	0	7	0	0	0
4f5adfba5b1d6438e006f283632a3842	0	0	0	0	0	8	0	0	0	0	0	0
6be07f9f894b07aed7b74f41993c3cf7	0	0	0	0	0	0	0	0	2	0	0	0
ab33d271c2ce2314be0453ee706f683f	0	0	0	0	0	17	0	0	0	0	0	0
ce54902d7be51f6082540a2734d693e6	0	0	0	2	0	0	0	0	0	0	0	0
03ea9fa35e70b17e8155a5bbb4e4b185	0	0	0	0	0	0	0	17	0	0	0	0
24144bb6cf77ea5f4710f382fc6c9d9d	0	0	0	0	0	0	0	0	0	0	35	0
c31e9cbff4629a48102ac82721db7355	0	0	0	0	0	0	0	0	0	0	3	0
61e92b2e40b35c915d81f2320885b48e	0	0	0	0	0	3	0	0	0	0	0	0
4e42cdc25efb7f20f86aed48c5a5b413	0	0	0	14	0	0	0	0	0	0	0	0
55df397b8c5c5fcbbbf518b9e34696ca	0	0	0	7	0	0	0	0	0	0	0	0

50bfdb91486ab107a70ee757 0836ab28	0	0	0	0	0	0	0	0	0	23	0	0
a42dbdc9425809d3d7f0ce16 fa4f42fc	0	0	0	0	0	0	0	4	0	0	0	0
78ef2e9531739db8b5669b1 58dba3704	0	0	0	0	0	0	0	0	0	0	23	0
d6534c1ea8f5c48dbe6de21b 940b3341	0	0	0	0	0	9	0	0	0	0	0	0
dc8a7a99e1777cba2e8fb3d3 23996736	0	0	0	0	0	0	0	30	0	0	0	0
a9cd0a7b87d547e712a9f36c af377710	0	7	0	0	0	0	0	0	0	0	0	0
e6df2b2b6664f0f376ce0124 14aa82ea	0	0	0	0	0	0	3	0	0	0	0	0
445124946352e2c92f41f575 3210d476	0	0	0	0	0	0	0	0	0	0	0	3
445b0efcd1ad9594a35c0a83 143db402	0	148	0	0	0	0	0	0	0	0	0	0
db0856ff2238dfe44e23170b 9d50141c	0	0	0	0	0	0	0	0	0	0	0	7
0d806a94ce54454d3b8c01d b885ca77a	0	0	0	0	0	0	0	0	0	0	5	0
b637e9500d7aa87dff1fab67 85650010	0	0	0	0	0	0	0	0	0	2	0	0
596b623a417d6feb37b72d0c 416b429d	0	0	0	0	0	0	0	0	0	0	0	24
bb1d370fc7f84bfb11e27b90 8b88fbec	0	0	0	0	0	0	7	0	0	0	0	0

ac27bb6d5a2e37e318cf1af9b568641c	0	0	0	0	27	0	0	0	0	0	0	0
764250b7c30fed7a240ecc6ff7882402	0	19	0	0	0	0	0	0	0	0	0	0
683cf5948066f4b78aebea440d18fdce	0	0	0	0	0	9	0	0	0	0	0	0
7c84430cccedbc38968c193cab9391f7	0	0	7	0	0	0	0	0	0	0	0	0
059a44713e7398b8908ea7402c98f4d6	0	0	0	0	0	0	0	0	0	0	0	2
60cf2a27b309260019565846dd6cb35d	0	0	0	0	0	0	0	0	0	0	6	0
cbbc5b1cf7a2114a80d7709f96fbf9d0	0	0	0	0	2	0	0	0	0	0	0	0
d350eb64bac3c581d5b88c89744140ac	0	0	0	3	0	0	0	0	0	0	0	0
a085d86d8af450659cac9023f7b7893b	0	0	0	0	0	0	0	0	0	46	0	0
9ef1775f8c68529ccf2c96d6643d6ed6	0	0	0	3	0	0	0	0	0	0	0	0
8ba303eb6911d591e47f5c31406411e7	0	0	0	0	0	4	0	0	0	0	0	0
302ce89f04177aa642093ed4ae6f90cb	0	0	0	13	0	0	0	0	0	0	0	0
215e2fa285ca0fa3dd447179b99178a5	0	0	0	0	0	0	0	0	0	0	17	0
9b6bac3007a34a1a7d1d3c65aecc0b60	0	0	0	0	0	0	0	0	0	0	15	0

3ad5178fe06a180c7e6bb19093f8b885	0	0	0	0	0	0	0	0	0	0	0	8
284de95adc0ea440dfebb52fe8711218	0	0	0	0	0	0	0	0	0	0	3	0
9f248c20300345306cd87773610f0063	0	0	0	0	0	0	0	0	0	0	2	0
5a164d8528b902c803e499ed4a47bf6d	0	17	0	0	0	0	0	0	0	0	0	0
1dff8071b78ccae4d841a11acf4f62ab	0	0	0	0	0	0	0	3	0	0	0	0
d95cef8520157e5aebd59f7fcb971963	4	0	0	0	0	0	0	0	0	0	0	0
c028a86c4797cfb58225a9a71f189a07	0	0	0	0	0	0	0	30	0	0	0	0
de656483bdff8d55a81f0676b0505ff8	0	0	0	0	0	0	0	0	0	16	0	0
6b3b8f9d73ff89c1b7e7ee5a1242a079	0	0	0	0	0	0	0	0	33	0	0	0
210e7de2a1a0e6b875c1df0d443b0aa3	0	0	0	0	20	0	0	0	0	0	0	0
c946d777dbfcd515840d2920720a8610	0	0	0	0	11	0	0	0	0	0	0	0
8013441c076e561d9a36bcd079822bc7	0	21	0	0	0	0	0	0	0	0	0	0
908840f8a074afbeb39ba61984be2c7b	0	0	0	0	0	0	0	20	0	0	0	0
31901d3f0e35d308de1ea778ad1f9952	0	0	0	0	0	0	29	0	0	0	0	0

7c9457d458628940c66bc1c59a7fc176	0	0	0	0	0	0	0	0	0	2	0	0
bbb1c74f30a7c5d6bef44d1e24edcf0d	0	0	0	0	0	0	0	0	3	0	0	0
bad2e3510423e809fb3a287299f4ce9a	0	0	0	0	0	0	20	0	0	0	0	0
76b308c6af07d3bf195e810baa7e462c	0	0	0	0	0	0	0	0	0	0	0	5
73b480aabf50d3d061239af8a211bee9	3	0	0	0	0	0	0	0	0	0	0	0
18bf52f71a3fb575f970752885d6ed48	0	0	0	0	0	0	0	0	2	0	0	0
fe867e41951cd2b953f8ac66bd5e2176	0	0	0	0	0	4	0	0	0	0	0	0
e1170d6ebd5c552c79b806c6f9768b3a	0	0	0	0	0	0	0	0	0	0	0	36
c3f0e0fc447fce9ba4d51d8ecbb5d778	6	0	0	0	0	0	0	0	0	0	0	0
17e59dcd9ba358d5023062bc33cfe0d7	0	0	0	0	0	0	0	2	0	0	0	0
f6884d64b99500e3b4215676b7ef2a4b	0	0	0	0	0	0	0	0	0	0	4	0
a630ef26c2f9035880d7ff23fb6b0024	0	0	8	0	0	0	0	0	0	0	0	0
b0f7f8b04adc40fdd0ed64558ac10858	0	0	0	0	0	0	0	0	0	0	0	16
626cdd363bdcd87d37818876021c21aa	0	0	0	0	0	0	0	0	2	0	0	0

f7c79595eebd9d84d5cc0a5030d9b86c	0	0	0	0	29	0	0	0	0	0	0	0
57413460d6dfdff589fb09d1c16abaca	0	0	0	0	0	0	0	0	37	0	0	0
fd3f78bef6097533a73880a802c11133	0	0	0	0	0	0	0	0	0	33	0	0
f650932bd63ff7c520264ba460613239	0	0	0	0	0	0	0	0	0	0	2	0
eca8181a195edb107ffb68347ef541a3	0	0	0	0	8	0	0	0	0	0	0	0
b81bd4e6e78da4c79b8b5c61f564e5bb	0	0	0	0	0	0	0	0	61	0	0	0
b443d467986ca88052d1f7bbe7447a64	0	0	0	0	0	0	0	0	0	0	0	9
57b6ae52da896ab8e90ed4ef5b714008	0	5	0	0	0	0	0	0	0	0	0	0
decf6ba011f2faed79ec233726b676a1	0	0	0	0	0	0	0	0	23	0	0	0
8aa0c2c0829e3d170785cbc51afccf6c	0	0	0	0	0	0	0	0	7	0	0	0
5c5c36e6356abb1c6a90a08b132518c8	0	0	0	0	0	0	0	0	0	0	2	0
f364a848ddc54b4395d95dbc20b900ad	0	0	0	0	0	0	0	0	0	0	2	0
c58f3b0842ca1733be4ebe5f1cbbde94	0	0	0	0	0	0	0	2	0	0	0	0
970d36e33bce4b6fa2f114d5560281da	0	0	0	0	0	0	0	3	0	0	0	0

1fa73ba312b3aaedbe9a5d498764fd3f	0	0	0	0	0	0	0	0	0	0	2	0
dc0bbbf775e439e6cbf19bf80b723634	0	0	24	0	0	0	0	0	0	0	0	0
d361c74f73475f2d67081bfafffda76c	0	0	0	0	0	0	0	2	0	0	0	0
5d1ff0e2e365cff11ad0387bad6938bf	3	0	0	0	0	0	0	0	0	0	0	0
8d845ab4d5211adc4b343dd977bcda94	0	0	0	0	0	0	0	0	0	0	2	0
f22ad7370d2f589e3928530a52487458	0	0	0	0	0	0	0	2	0	0	0	0
33020ab9a86d7c4261a0c4c3e1b54e67	0	0	0	0	18	0	0	0	0	0	0	0
cdcbf27fa11ea110a8b705a9135c42a7	11	0	0	0	0	0	0	0	0	0	0	0
ddb043a188350092bcccb41c4d55f051	0	0	0	0	0	0	0	26	0	0	0	0
93d0076bb7202b0bdf4bfb896ae5bcbc	0	0	0	0	0	0	0	2	0	0	0	0
5905b192ba6916be2ba4ef2297457385	0	0	0	0	0	0	0	0	17	0	0	0
674fb65df00d49534363a0ea5734d92c	65	47	0	0	0	0	82	0	0	0	0	0
7c3c2f9ba5d98ba72c5c024de6ba4239	0	0	0	0	0	0	0	0	0	2	0	0
b966aea4933164890e9e37f57b54a0bf	0	0	0	0	0	0	0	0	0	0	2	0

5fe1a89d3ba23629e7c645e309935b76	0	0	3	0	0	0	0	0	0	0	0	0
fb9261c6bdb92c850969bca574a43620	0	0	0	0	0	0	0	0	2	0	0	0
4d3c3c65c451d48881306aa5d40a70ca	0	0	0	0	0	0	11	0	0	0	0	0
aad28331bd6540a9fd829c481c236096	0	0	0	0	0	0	0	0	17	0	0	0
9f776052d724a23a94ec572c0cf80008	0	0	0	0	13	0	0	0	0	0	0	0
1866da7072d7f7dfd4d49a13b8e41f6d	0	0	0	0	0	6	0	0	0	0	0	0
e2f3bc35ac879d2f14e2721ae4218469	0	0	0	0	0	0	0	0	0	0	49	0
53820c95ce37374241c44bee87ec5679	0	0	0	0	0	0	0	0	3	0	0	0
b6324e5adcd0f0d5884c46071bcead6c	0	0	0	0	0	0	7	0	0	0	0	0
db74e83d2cd051bf53fd478457156cdc	0	0	0	0	0	0	0	0	7	0	0	0
2bc8c4c9ffecd79514b12fcdb4a56064	0	0	0	0	0	3	0	0	0	0	0	0
f1fe8d410ec59dda42ac37d8e48c7c78	0	0	0	0	0	0	0	0	0	0	5	0
7d26abef6dba6d7ac5e446c2e77f3aa8	0	0	0	0	2	0	0	0	0	0	0	0
a29fa20cb5f2b45ce189b923da0e9f98	0	0	0	0	0	2	0	0	0	0	0	0

502408f8a3191457aea28c6b519937eb	0	0	0	0	0	0	0	0	0	8	0	0
d1fea8126ad7bfd5e8b45a6e5bbfba4b	0	0	0	5	0	0	0	0	0	0	0	0
7b643932e09568c11bfc72a93bae15df	0	0	0	0	0	0	0	0	0	0	2	0
9ad344a5f542ba299aba0144bf84efa3	0	0	0	0	0	0	0	0	0	0	0	4
b611eba085ba13f98c605883ffb7e9f9	0	0	0	0	3	0	0	0	0	0	0	0
08be435e25d26443563f03d48bff78a4	0	0	0	0	0	0	0	0	0	0	2	0
1e28910e6e716cbb80822e2278a9303d	0	25	0	0	0	0	0	0	0	0	0	0
8d478e346e1cf2c4dcc07ed088108db8	79	0	0	0	0	0	0	0	0	0	0	0
0b247266c52f2353297773f040f2e749	0	0	0	4	0	0	0	0	0	0	0	0
a4b3fa157c417aa6fa36b5173b451fbf	0	0	0	0	0	0	0	0	4	0	0	0
d937622ad41fb32480ec9ba6fd434b68	0	0	0	0	0	0	0	0	0	0	151	0
2afa433eda7d3ff8b0bee6c6d3569672	0	2	0	0	0	0	0	0	0	0	0	0
636112648d148134608f5d80dd9b1f0d	0	0	0	0	89	0	0	0	0	0	0	0
949d862c4fabe000f6ae6477988ad92e	0	0	0	0	0	0	0	0	0	2	0	0

877167ad37d3ddb5d75c4f291f36e8c2	0	0	0	0	0	4	0	0	0	0	0	0
a6f0c512a62c9656cdfc7828a569d69c	0	0	0	0	0	31	0	0	0	0	0	0
3d24512368b94440efb0a133611f8d2a	0	0	0	0	0	8	0	0	0	0	0	0
920dc5d79bdd40f782c8843e98227825	0	0	0	0	0	0	0	0	0	36	0	0
3dbaa416f8d62395fee7cb8663e7fc0d	0	0	0	0	0	0	0	11	0	0	0	0
1d7a9db5568868c04d0112ae8994be8f	0	0	0	0	0	3	0	0	0	0	0	0
ca757ae723aa1e50b184a393af6f0a49	0	6	0	0	0	0	0	0	0	0	0	0
f7451f20720c197f0f096a3bad38868e	0	0	0	4	0	0	0	0	0	0	0	0
795618fc61a65f1c043c0d5de13c3983	0	0	0	0	0	0	0	4	0	0	0	0
84f98076208a7d01b328dd5eba63039a	0	0	0	0	0	0	0	0	0	0	7	0
c7b42040586f6b59e79769c251500c72	0	0	0	0	0	0	0	3	0	0	0	0
de58684f9172eab975a1d6289a18212b	0	0	0	0	0	0	0	0	0	0	4	0
14ecf9e85b846d442c9c7be6cc72c260	0	0	0	0	0	0	0	0	0	0	2	0
032c14236b6baf62ad5c3221dfbf4b03	0	0	7	0	0	0	0	0	0	0	0	0

d0b5eb59387f2b31ae96d519dd838cdd	0	0	0	0	0	0	0	0	0	0	3	0
11fa1d57a560f466f60825eecc82f6bb	0	0	0	0	0	0	0	0	0	0	30	0
00c013ba119d95de724da1fcaa6837b8	0	0	0	0	0	0	0	0	0	3	0	0
6c4a9d30414dfd745cb19529ade07468	0	0	1684	0	0	0	0	0	0	0	0	0
cbbbfa175c84f55b0eac3a4e59579946	0	0	0	0	0	0	6	0	0	0	0	0
1887bdf2f2e0eba8a099c664c60cbccf	0	0	0	0	3	0	0	0	0	0	0	0
aa1814c07979231d4866cf20a39b01e8	0	0	0	0	0	0	0	0	0	0	5	0
a673b62a7cf20fc12d919585d1669a25	0	0	0	0	0	40	0	0	0	0	0	0
9c3792000d94213b4bc72b42f80e88e3	0	0	0	0	0	0	0	0	0	0	0	12
a6e57856cea08f98b987fbd276ddd689	0	0	0	155	0	0	0	0	203	0	0	91
ba8a95c388a7ee42fcfc2defe31fa022	0	3	0	0	0	0	0	0	0	0	0	0
02c5ec932f50025c70fefbd25f4467b2	0	0	0	0	0	0	2	0	0	0	0	0
16b1dbb8ad97519bd5c81c01ad827d8d	3	0	0	0	0	0	0	0	0	0	0	0
e0eed618c3be360ca07103e039aac081	0	0	3	0	0	0	0	0	0	0	0	0

160e5dbb5341c338e139b28 2e161b956	0	0	0	0	0	0	0	0	0	0	0	5
c0da26252faa5c834242dec9 a08a1bd0	0	0	0	0	0	0	0	0	0	0	0	3
44d541419626bc9af48b81e1 1572c95e	0	0	0	0	0	0	0	0	5	0	0	0
60fb2659038f6dd659079c47 400e21a9	0	0	0	0	2	0	0	0	0	0	0	0
9266386880ac2470cdae998b 9e8d3e3b	0	0	0	0	10	0	0	0	0	0	0	0
d0e0f05d4f383c8c99102c7b d99de9c0	0	0	0	0	0	0	0	0	0	2	0	0
f897c8a4c8baa5cbca77f1c54 867b93e	0	0	0	0	0	0	0	0	2	0	0	0
b2299213071eeec36f4b96b8 2a87f7c8	0	0	0	0	0	0	0	0	0	15	0	0
8e1643b80de95dd43cfaaa2b a7e89bf4	0	0	0	0	0	0	2	0	0	0	0	0
2f36bbf166500332c9d0e054 ee065591	0	0	7	0	0	0	0	0	0	0	0	0
61cbc85128f04300d11dde5c d2744493	11	0	0	0	0	0	0	0	0	0	0	0
68d1394e6544478880a37b7 1f8f0cb97	0	0	0	0	0	0	19	0	0	0	0	0
83a25fac581a6a54b2d0243ff 8e242a4	0	0	0	0	0	0	0	0	2	0	0	0
31385d1e6a99a64b0d09562 62ee06756	0	0	0	0	0	0	0	0	7	0	0	0

1f7523e7b48ca31c9cfd7ee5ad0000a8	0	0	0	0	0	0	0	0	0	2	0	0
6a74eb91e2571d3544f0b15816d2f5d9	0	0	0	0	0	0	23	0	0	0	0	0
e6b722259135f2975cc8222c839543bf	0	0	0	0	0	0	0	13	0	0	0	0
443ce46d06caff8d555f9e689be092c4	0	0	0	8	0	0	0	0	0	0	0	0
9b6628c91fb3c4d246d6da460e17ba33	0	0	0	0	0	0	0	0	0	0	0	4
d7a0777dcea666991b032b01a6fdb74f	13	0	0	0	0	0	0	0	0	0	0	0
fed5a05ce1664399874827790db0c707	0	0	0	0	0	0	0	4	0	0	0	0
8ced702b1832d6b9f55bc5516501b5c4	0	0	0	0	0	0	0	0	0	0	7	0
e8fa0284f3be072df25470bc38cbc434	0	0	0	0	0	0	0	0	0	0	9	0
f569daccf8078e400dd38f4790cfd207	0	0	0	0	0	15	0	0	0	0	0	0
856493feb7c4a8262e9621b79fbc0f77	0	0	0	0	0	0	3	0	0	0	0	0
c06af4671a19b01411cbb8cf5588506b	0	0	0	0	0	0	0	0	0	0	19	0
4214b914ae80d8881c76bc43541d24f3	0	0	0	0	0	0	0	0	0	0	4	0
b9e8fdb5c707f17da341fc5ad7ace1f6	20	0	0	0	0	0	0	0	0	0	0	0

15d6f8892100545b7d55bbb3a978ae6e	0	0	0	0	0	0	0	0	0	0	7	0
6b6a0b7425b65b67cc00491a80f87f0b	0	0	0	0	0	0	0	0	0	0	3	0
9c9f1044622ac08341cd8a98ede4b0ba	0	0	0	0	0	2	0	0	0	0	0	0
8443c3e7dd6588b840511ef41c98feff	0	0	0	0	0	0	0	0	0	78	0	0
128592b473a0fca91cac9d711480799e	0	0	0	0	0	0	0	0	0	0	5	0
c909bd8b1cb63f1556a192826aaf639d	0	0	0	0	0	0	0	0	0	16	0	0
92eef7d75972823071f5ce3573aad6ad	0	0	0	0	0	0	20	0	0	0	0	0
14912e5f4322d803bcffe636fd68c560	0	0	0	0	0	4	0	0	0	0	0	0
4a2ca090408fde4a40dfeff1453a8fd5	0	0	0	0	13	0	0	0	0	0	0	0
eebddab5a09a6f9df628f1ae5d76ad05	0	0	0	0	0	0	0	30	0	0	0	0
2fc227faec646ae58f2c7f1de458490e	0	0	0	0	0	0	0	0	0	0	0	3
2f3e83202310d05062ab70d50f232ea8	0	0	0	0	0	0	0	0	2	0	0	0
abbe2a939d42ec48d393f3e423986d5d	0	0	0	0	0	0	0	0	0	0	2	0
15dba14d433d5369feadee60e460c2f6	0	30	0	0	0	0	0	0	0	0	0	0

fca4c0e3f437f0253e58a8a9f88fce56	0	0	0	12	0	0	0	0	0	0	0	0
3fa73f10a10aa9f3486a2b0e47e1bc90	0	0	0	0	0	0	0	0	0	17	0	0
4110409b0fdb634cc12de895ff7505ae	0	0	0	0	0	0	0	0	0	0	17	0
92193757f13b3d1dc020372837d4a17e	0	0	0	0	0	0	0	0	0	0	3	0
ef2e8f24b6c55be2330ece0cf7aad91f	0	0	0	0	0	0	0	24	0	0	137	0
a346c775a11319a749d853a13aecd7de	0	0	0	0	0	0	0	0	25	0	0	0
7b92b09125b2a232922202539be862fb	0	0	0	0	0	0	0	0	0	12	0	0
e537524310ab1e4103d908a68d550e71	3	0	0	0	0	0	0	0	0	0	0	0
963d2f77020f9d9ad6c974f08add8943	3	0	0	0	0	0	0	0	0	0	0	0
63c53929838b683a8c165e7848ed5bd8	0	0	0	5	0	0	0	0	0	0	0	0
861cac34cc04906970f2cdea9c815023	0	0	0	0	0	0	0	0	0	13	0	0
ce11379f26904a7fa592002bca7c3971	0	0	0	0	0	0	0	0	0	32	0	0
3aa6270be9a589a4b5d0731c54b29b29	0	0	0	0	0	0	0	0	187	0	0	0
2e686397a15c55f3e1aebfd63c4493af	0	0	0	0	0	0	0	0	0	3	0	0

2bb7cbfae6b5f434578cb31cc34f7489	0	0	0	0	0	0	0	0	0	0	8	0
361db2ca1598e6b0cb81204d6f198f42	0	2	0	0	0	0	0	0	0	0	0	0
7fb012851ba03cbf8277666ddd829fa6	0	15	0	0	0	0	0	0	0	0	0	0
ccd46bf334bf91f0e6918c5bcfa5bf25	0	3	0	0	0	0	0	0	0	0	0	0
0b905dcc5ebdb383dca9935146429c1e	0	0	0	12	0	0	0	0	0	0	0	0
f4cbe401a9cb2d9d7505b79fa1663e92	0	0	0	0	0	0	0	0	0	0	7	0
9af26f1a6765b06e0603883914ab8928	0	0	0	0	0	0	0	4	0	0	0	0
6d712a88d06e81d313d489e701e146a7	10	0	0	0	0	0	0	0	0	0	0	0
896b02ef5d60601cd90d64211ee531f5	3	0	0	0	0	0	0	0	0	0	0	0
dfa579746c890482103738386419f1c2	0	0	0	0	0	0	0	0	0	0	0	4
18e9c40dc6660c65a479adf7decab2ea	0	0	0	0	0	0	0	0	0	0	0	141
d2104253f5314771792345c1af39ebe9	0	0	0	0	0	2	0	0	0	0	0	0
9e533baeff721c26a9fc9dc9ad6f8f42	0	0	0	0	0	0	10	0	0	0	0	0
9bc75891e031165a827de7ed9c3b5e42	2	0	0	0	0	0	0	0	0	0	0	0

d0ca44003bcaac9efaa0a5b197561dc9	0	0	0	0	0	0	2	0	0	0	0	0
0d5cff4c2ff096e37cdc0ef4ccaee2eb	0	0	0	0	0	0	0	0	0	0	18	0
de888feb0aa74b4bbccd4be5f138b3dc	0	0	44	56	0	0	0	0	0	0	0	0
f9a61cf98ba0634f531377324c4774e4	0	0	0	2	0	0	0	0	0	0	0	0
e0c0d8a0eaf4d8d8cafd2d8f6c8291ec	0	0	0	0	0	0	0	0	0	3	0	0
64f2a327fb1429cf47fe7cb435dc62bb	0	0	0	0	0	0	0	5	0	0	0	0
ff71a27ba41323210a0910afc90f14ea	0	10	0	0	0	0	0	0	0	0	0	0
4d57e4684fbaf465a4c2e8fb38a33fbd	0	0	0	7	0	0	0	0	0	0	0	0
6e9956dee8fcf33b47c2f64bcb74c139	0	0	0	0	0	0	0	0	0	0	4	0
eeb90947ee58bf04da756adc840a605c	0	0	0	0	0	0	3	0	0	0	0	0
22d36880e0d4af9d9670de9fb21ae2a9	0	0	0	0	0	0	0	0	0	0	8	0
cbb8459dd294611e6cfd737ac5649a73	0	0	0	0	0	0	0	149	0	0	0	0
df4a1d76da47fe3a379001fecfa9cecc	0	0	0	0	0	0	0	0	4	0	0	0
b25d77f20f012d278b2bfa6513f012a5	0	0	0	0	0	0	0	0	0	0	0	3

e5e4923c22f144c06504f8d4055de619	0	0	0	0	0	0	0	0	0	0	0	30
376979dc8237c1600a037d64c910f5bb	0	0	0	0	0	0	0	0	2	0	0	0
0ea5127c8bd2a0a1d5edcbf148c12525	0	0	0	0	0	0	4	0	0	0	0	0
f9eb9685da7f7a7d955092f198182059	0	0	0	0	0	0	0	0	0	0	0	3
2e5088acedf2d0a24155023da6f03a93	0	0	2	0	0	0	0	0	0	0	0	0
c9f0c9e831cfa97bc0e0dfe9efa1d818	0	0	0	0	0	0	0	0	0	0	0	6
333c3746c3eca9e9a197cc5cfe261ace	0	0	0	0	0	0	0	0	0	0	8	0
96fc4b130f00b0ca216077d68482214b	0	0	0	0	0	0	0	0	0	0	0	17
d62435be41942ecb425b793addc96dff	0	0	0	0	0	3	0	0	0	0	0	0
810025827bd5250688542963671bdcbf	0	0	0	0	0	0	0	0	0	3	0	0
fe76c4a77f8ed6f11d5c12e4bd3c5ce2	0	4	0	0	0	0	0	0	0	0	0	0
97d3a82f115322fb742203d32f80ef2a	0	0	0	5	0	0	0	0	0	0	0	0
86f95a1b692a383d49764b7bccaa079f	0	0	0	2	0	0	0	0	0	0	0	0
73033c13c1dea6f51f731ac4e265f160	0	0	0	0	0	0	0	0	0	0	4	0

66a13d9a84d9ef6af6cf26bff66467be	0	0	0	0	71	0	0	0	0	0	0	0
f602ca79102bf676cc47257009dab305	0	0	0	0	0	0	0	0	0	0	38	0
24103507b819510a4d904a475af8b8fe	0	0	0	0	0	0	0	0	25	0	0	0
34191f8a66ef0f318ce290ffa01aa3fe	0	0	0	0	0	0	0	0	0	0	0	20
ee65982c3e53524fbb18ed53f410ff9e	0	0	0	0	0	0	0	0	0	0	10	0
e7f7b27890784cc179b9e1503627fcad	0	0	4	0	0	0	0	0	0	0	0	0
cb63e07cfc7ebed7f4b4a88ae0f9d0b8	0	0	0	22	0	0	0	0	0	0	0	0
ac90d61e4c848bc2437066ad9438e45f	0	0	0	0	0	0	0	0	0	0	0	5
abc95d2737a2cee199afb052bb550fc0	0	0	0	0	0	0	0	0	14	0	0	0
25af9782186c5030ac93f7284779b108	0	0	0	0	0	2	0	0	0	0	0	0
8dd1eef5e96513dad6ca283c5af98c7c	0	0	0	28	0	0	0	0	0	0	0	0
c8509897c802382fd509d195c22dce36	0	0	0	0	0	0	0	0	0	2	0	0
75c70e18433081de2a3cb28b16030f74	7	0	0	0	0	0	0	0	0	0	0	0
3838d9a211df8b4e40c0b655223f3167	0	0	0	0	0	0	5	0	0	0	0	0

2b52a7bf9adf228d90d0a4c4efa27b4b	0	0	0	0	0	0	0	0	0	0	2	0
dcc6808432bd8ad4baac0887aadc0291	26	0	0	0	0	0	0	0	0	0	0	0
5ede598d78174eaa48548a7ce77e547c	3	0	0	0	0	0	0	0	0	0	0	0
b919bdbc539149008cdec625b6d0b669	0	0	0	0	0	0	0	0	0	0	0	11
4933f592082adce87d40390a091c93b9	0	0	0	0	0	25	0	0	0	0	0	0
801bf3192a3d0e9dbc652da0f65ac544	0	0	0	3	0	0	0	0	0	0	0	0
893a1ceab6b2578cbbf2045653e20aec	0	0	0	0	0	0	0	0	0	0	0	3
16b61cda1bed85c575c8fa2ca29634ea	0	0	0	7	0	0	0	0	0	0	0	0
4be66442e9369513b96bbd9947c5cbb4	0	0	0	0	0	0	0	0	0	0	43	0
ccca7f985e9f0974a332a5d9337bad58	0	0	0	2	0	0	0	0	0	0	0	0
7b4d2f90adb9e5fb14c85b3f322824d3	0	0	0	0	0	0	0	0	0	0	15	0
35bf7135413694b399ffb274519f96a5	0	0	0	0	0	0	0	2	0	0	0	0
ce809e5962548071ecd936406cdc54e1	0	0	0	0	0	0	0	0	0	3	0	0
c952c41449edfa8df09d66211c616196	0	0	0	0	0	0	0	0	0	0	10	0

098b010c1eef5b1e109fada86945ba58	0	0	0	0	0	0	0	0	0	0	0	7
bb7e8718e4e840ce6092ba8e9b6d7885	0	0	0	0	0	0	0	4	0	0	0	0
ec68df14bcda7de53ac2264d338ec7d8	0	0	0	0	0	0	0	0	0	0	3	0
ff3e71c4044c45d0ac2fc7cdce1bf36b	0	0	0	0	0	0	0	0	0	6	0	0
41127466fcb610eb8adc548a2988e895	0	0	0	0	0	0	0	3	0	0	0	0
cd6e2f4965a587d737023adbf34faa2e	0	0	0	0	0	0	0	0	0	0	0	34
38a056e7c05deb4209b51d3d570b1ff0	0	0	0	0	0	0	0	0	0	0	13	0
ee9121450ee402191490efbf1625818a	11	0	0	0	0	0	0	0	0	0	0	0
d1fc58504fd60a4adf7a6b093aba2383	0	0	0	4	0	0	0	0	0	0	0	0
b32bd8ff20d871f2a0016c9380d598ae	0	0	0	0	0	0	0	0	3	0	0	0
e390180d4c7fa27b9eac751b8ffd8f39	0	0	0	0	0	0	0	3	0	0	0	0
414acd261a2c90c84b66008729e5e189	4	0	0	0	0	0	0	0	0	0	0	0
7e1153ba186c5aa9d5d2ef69c7c5d5c2	0	0	0	0	0	0	0	9	0	0	0	0
a60fed9b478a7cd847701620e82491ee	0	0	0	0	0	0	0	0	0	0	0	58

f3a1aeb4901034d30d46564244a1503f	0	0	0	0	4	0	0	0	0	0	0	0
21d4fe487f1887f6840a4a5f9d26b6ac	0	0	0	0	0	0	0	0	0	0	2	0
06269e04d463b0e1912e58d1bcc3e1f7	0	0	0	0	0	0	32	0	0	0	0	0
58ceb39a63da93fde4423aab43747278	0	0	0	0	0	2	0	0	0	0	0	0
65e7fc3a8641ca39f59e02b237945b6a	0	0	0	0	0	0	0	0	0	0	0	3
58d68c9edd053c20ca2e28012cfc8da4	2	0	0	0	0	0	0	0	0	0	0	0
816f8d663137c8c413e92b1522428986	0	0	0	0	0	26	0	0	0	0	0	0
628a03f14c853a357bf9c61a3ea7afc8	0	0	23	0	0	0	0	0	0	0	0	0
8760a2367bdab526463fced449d6bb37	0	0	0	0	0	0	0	3	0	0	0	0
f0d0e02888d05a07f9da5028f045d7e8	0	0	3	0	0	0	0	0	0	0	0	0
939d15b6f1f2ac547c4f8ce2aa5955a8	0	9	0	0	0	0	0	0	0	0	0	0
f949b1e3c2670df17f3fac0d8da002f7	0	0	0	0	0	18	0	0	0	0	0	0
b19a419a62f93759379178516b08c8df	0	0	0	0	0	0	0	0	0	0	0	12
3c5766df97b6214e532a69cbd091d53d	0	0	0	0	0	0	0	0	0	0	5	0

439331d1001a31e6accc3275 9bf4a05b	0	0	0	0	0	90	0	0	0	116	0	76
41e5683218649a082350502 bcd1284a5	0	0	0	0	0	0	0	0	0	0	0	5
9fe7b697e63de51be13dbcb2 e4e854de	0	0	0	0	0	0	0	0	0	0	4	0
e93d106da0db7c82f716cf18 5cdd8808	0	0	0	0	0	0	0	11	0	0	0	0
8244a61381f6e3fc32d3955d 02c96940	3	0	0	0	0	0	0	0	0	0	0	0
ac5aca6d71fbe2d5e230abfcf c034c71	0	0	0	0	0	0	0	0	4	0	0	0
ac109e358e33c0d4e927b12 bc75e5243	0	0	0	0	0	0	0	0	0	0	10	0
b6b7a9151f3307329c615110 fa91bfe9	0	0	0	0	0	0	0	0	0	0	0	10
a47a008572b42a77e62adaa b995187f0	0	0	0	0	0	0	0	0	0	0	24	0
90267bc221aa0e9c4316d6ed 550c76ab	0	0	0	0	0	0	0	0	0	4	0	0
68cb344cd1a5db59bc347f19 6d4e6e17	0	0	0	0	3	0	0	0	0	0	0	0
ea4fdec6fad7dca8e16459e2 ddbd32de	0	0	0	0	6	0	0	0	0	0	0	0
0747309687c6b2c39c4cd6c6 881d489d	0	0	0	0	0	0	4	0	0	0	0	0
59b2c7180f6373c00e90e02d 0e6dc50d	0	0	0	0	0	0	0	4	0	0	0	0

007f12a0a37ac505fd83ba21c13a8686	0	0	0	0	0	0	0	0	0	0	0	3
d4046903fed1cfac7c945655f8a44935	0	0	0	0	0	0	0	0	2	0	0	0
f590e4c9b3174f34bc5057ebcb1ff3e0	0	0	0	0	0	0	0	0	0	0	0	2
204d6015f27fbde10199ee36344fb32c	0	0	0	0	5	0	0	0	0	0	0	0
a2af52384a4e48b9b532cc5e623a6221	0	0	3	0	0	0	0	0	0	0	0	0
478ac3e969257009a5d4a8b25e4aa2e5	0	0	0	0	0	9	0	0	0	0	0	0
f0a01b4bbd9b9b59b4695aa473bca4be	0	0	0	0	0	0	0	3	0	0	0	0
1e5bb5e2cee3465e2ffba6d7e839e440	0	0	0	0	0	14	0	0	0	0	0	0
322768e4696277f7d35aafeec853f430	0	0	0	0	0	0	0	0	0	0	0	6
28ff176000d05c094fcebffd86cc62d4	0	0	0	0	0	0	0	0	0	17	0	0
4d2679fffc2eab715d5dd6b4d1ed2add	0	0	7	0	0	0	0	0	0	0	0	0
44e1f1b47a7eed2c4e26aa8b4c629e95	0	0	0	0	0	0	0	0	0	0	8	0
3bf1ba86e6bf206d9948606e98a9b1bb	0	0	0	0	0	0	0	59	0	0	0	0
355c78d4d4ab03497c3ec04d5cb74ed2	0	0	0	0	0	0	0	0	0	0	0	6

3c6ba16e01f6cc4bc812e689 26c9b5aa	0	0	0	0	0	2	0	0	0	0	0	0
78a0b2516f4eb4e9728c86db 1a7d8286	6	0	0	0	0	0	0	0	0	0	0	0
3f4b74a59e94dabec71c9d8c 05476bc3	0	0	0	0	0	0	0	0	0	0	0	6
257046928037390ac838825 ec23d89f6	0	0	2	0	0	0	0	0	0	0	0	0
d6c10a88641e8c5f036ce5c6 3976ca95	0	0	0	17	0	0	0	0	0	0	0	0
acfe345677773e8795d7b13d d5701997	0	0	0	0	0	0	0	0	0	0	0	2
6ff984405ebfc1cd78a5bbe83 9232ac2	0	0	0	0	0	0	0	0	0	0	4	0
1acb6042401a1ef934af80da 3e669d09	0	0	0	0	0	0	0	0	0	0	12	0
94e80d1049dff110d9442e7d 875672b6	207	669	0	0	0	0	0	0	0	0	0	0
4f7a1c40fecca29ff20f066805 b9ca05	0	0	0	0	0	0	0	4	0	0	0	0
bbcc461e49cc5ddc69ae5441 e9f7a845	0	0	0	0	0	0	0	0	0	0	0	5
f296e07ee41453ec7f160452 e06bec4b	0	0	0	0	0	0	3	0	0	0	0	0
ad6880497922c2aefd96f055 457f4e65	0	0	5	0	0	0	0	0	0	0	0	0
095ef28d2201d3d16f062662 2cfacd45	0	0	0	0	172	0	0	0	81	215	0	0

5f2bf09f271be8907cdd8a3e d92ef426	0	0	0	0	0	0	0	0	0	0	5	0
7144045d7b11a03e85c851ef c5703a21	0	0	0	0	0	0	0	0	20	0	0	0
fba921a9900b350d44e56ce5 5276afd8	0	0	0	0	0	2	0	0	0	0	0	0
ed56e97cf9578035ca1a3eb8 b96e1e44	0	0	0	0	0	0	0	0	0	0	0	2
6b62637014d778a950075d3 0d1874600	0	0	0	0	0	0	0	0	14	0	0	0
a6b580e1cc74221486783f58 412f06be	0	0	0	0	0	0	0	0	0	0	34	0
f90c164fbb1e6e8dd5fe2cb82 dfb331a	0	0	0	0	0	0	0	0	13	0	0	0
07cc89fbcaf3a58859d613d3 d29869b0	0	0	0	0	0	0	0	5	0	0	0	0
73d233a5040dfe23b30dc912 86ec7206	0	0	0	0	0	7	0	0	0	0	0	0
246b0085adb5ba6c17f1ca8a b7c56187	0	0	0	0	0	0	0	0	0	0	15	0
c4aa1ed3b2fc470587182eda 2257e674	0	0	0	0	0	0	0	0	0	0	0	5
60a8b4f9ce5139e322b53836 bb667cc7	0	0	0	0	0	0	0	0	0	0	3	0
0e238c621d07a575e1f41a0a d115a27d	0	0	0	0	0	0	0	0	4	0	0	0
c0a1cc78a0ff76780897fed81 ec5fc36	3	0	0	0	0	0	0	0	0	0	0	0

30904ea75056c5ae045ec2c90070c88e	0	0	0	0	0	0	2	0	0	0	0	0
8436536cc581ff7da7183110780654a8	0	0	2	0	0	0	0	0	0	0	0	0
07600bc9262d9dea7a27069dd9b8d230	0	0	0	0	0	0	0	0	0	0	0	5
3c74ca14f195970c09733a784f2ee327	0	0	0	12	0	0	0	0	0	0	0	0
cb95ed574ce87be452221a072a2e1709	0	0	0	0	0	0	0	0	0	0	0	2
9e35e8e0193412d8644fbe795181e6bf	0	0	0	0	0	0	0	0	15	0	0	0
272b745d4f3885c64aa5ebc6dc5b553f	0	0	3	0	0	0	0	0	0	0	0	0
87b820e030e1d626cb3cd8bc7e93bdb6	0	0	0	0	0	0	0	0	0	0	5	0
c72af4354efc591708c3be9ce81ce1f8	0	0	0	0	0	0	0	0	0	0	0	6
45010409ba6d94683ef6395668d6b51b	0	0	0	0	0	0	0	13	0	0	0	0
ae56bd9bdf1d54bb585cf6435cf22bd8	0	0	0	0	0	0	0	0	0	8	0	0
8ba7ccbe6866f45b0b2c9480b02acb42	6	0	0	0	0	0	0	0	0	0	0	0
5600058df21603e0ef49e27d2a7e86a8	0	0	84	0	0	0	0	0	0	0	0	0
c38c7a7637d0d1ac281e909b048ad73a	0	0	0	0	0	0	0	0	0	8	0	0

42459dd5b037e73d3a594d1085d27456	0	0	24	0	0	0	0	0	0	0	0	0
a47ce41ed5e433515dd9880bb8c1203f	0	0	0	0	0	0	12	0	0	0	0	0
b99c4a14d4367e99b856d9171427c7b2	0	0	0	0	0	6	0	0	0	0	0	0
969ab63381ab86c7fa41621aa7d56637	92	0	0	0	0	298	0	214	0	0	0	0
a3c2ec813a6ef5332eff10f356bb710e	0	0	0	0	0	0	0	0	17	0	0	0
fc0e81e67e9c05a6f4af3e89f868fc08	0	0	0	0	0	0	0	9	0	0	0	0
48726c306f684b10ed78224daf51a89f	0	0	0	2	0	0	0	0	0	0	0	0
bd543c652b54d2d123737fc73261bf67	0	0	0	0	0	0	0	0	0	33	0	0
6bf08aacac7786edd66642f8efef22af	0	0	0	0	0	0	0	0	0	0	3	0
f6d88643e2038a46f051dec46a0b65d6	0	0	0	0	0	0	0	0	0	0	12	0
d4ec00f08b5b7f957b41d309e5c4a166	0	0	0	0	0	6	0	0	0	0	0	0
675065d207bcaab834024242dea3d06f	0	0	0	0	0	7	0	0	0	0	0	0
16f2827fa7a635ada5ceb8fc5fc5382e	0	0	0	0	2	0	0	0	0	0	0	0
0549ce471c7f8076e97c072d7f8d2631	0	0	0	0	64	0	0	0	0	0	0	0

a297bedc4797cafe9d40a317074dedda	0	0	0	93	0	0	0	0	0	0	0	0
e330b1f7a65df60513594aeae48d59c9	0	0	0	0	0	0	2	0	0	0	0	0
680bd69f79024d655d4ac7cd9b9dfaf9	0	0	0	0	0	0	0	0	0	2	0	0
627789b418aad83e2e1118119c3ba996	0	0	0	0	0	0	0	0	0	0	23	0
18dcd604f8c85506516de55f7a0622de	0	0	0	0	0	0	0	0	2	0	0	0
d5b4f5fb52658a456c80b03cb075221e	0	0	0	0	0	0	0	5	0	0	0	0
7e66b67cfdfa7df1eb20a349a48a44b3	0	0	0	0	0	0	2	0	0	0	0	0
d006d59a7e23f2eb863eddb05ddcd1b7	105	0	0	0	0	0	0	0	0	0	0	0
e2072b48f4821cde94bccb7766c7371e	0	7	0	0	0	0	0	0	0	0	0	0
1edef9d743ef7649fad08f9e8a89ffe4	0	0	0	0	0	0	0	0	0	0	59	0
587501eb79b01d5397ad8d7cc6c8439b	0	0	0	0	0	0	0	0	0	0	3	0
d0f774d9ebfe00fcaea782ea86a861a5	0	0	0	3	0	0	0	0	0	0	0	0
20e94f16d3ed13cd41060bd1ab42ae18	0	0	0	0	0	0	0	0	0	0	0	29
6362d7702097a6037bc57d0f79054ff2	0	0	0	0	0	19	0	0	0	0	0	0

27b7644e1106b30b05cc831531aebd44	0	0	0	0	0	8	0	0	0	0	0	0
989ac889af27d980e242a4fd21d1f211	0	0	0	23	0	0	0	0	0	0	0	0
826dbf332672ab827b25902c57a347fb	0	0	0	2	0	0	0	0	0	0	0	0
2e5c5ba26b88eb95fa3f2730fc22800e	0	0	0	0	0	0	0	0	0	2	0	0
195a661ca80d50873071bd9f024cabfb	0	0	0	0	0	11	0	0	0	0	0	0
c15de4f3ace48c932f14588af847e841	0	0	0	7	0	0	0	0	0	0	0	0
eafefbcdd074965292232f1882c0068d	0	0	0	0	8	0	0	0	0	0	0	0
bbb04149e2a0ea952c54b26f5f9d1aaf	0	0	0	2	0	0	0	0	0	0	0	0
9758f3a355137e9edac179e216a5faf0	0	0	0	0	0	0	0	0	0	0	0	2
071529b9af3e129653dabaa75c8bd6d4	0	6	0	0	0	0	0	0	0	0	0	0
7c807291937c8cd42b31be8e024796f9	0	0	0	0	0	0	0	0	0	0	0	6
be12b7d18852a8d2a1febfa64d7f8859	0	0	0	0	0	0	4	0	0	0	0	0
c99d807870d6d98485560ae4b550bac3	0	0	0	0	0	8	0	0	0	0	0	0
117df51e5743b6e9af81cf5629a36914	0	214	0	1131	0	0	0	0	0	0	0	0

9250d3d809b95b9cafee994315cc5a51	0	0	0	2	0	0	0	0	0	0	0	0
6733a6fad014c5ea18d95f125a7e5a24	0	0	0	0	0	0	0	0	3	0	0	0
e2958d581489298a73d9bde019f0e206	10	0	0	0	0	0	0	0	0	0	0	0
09ec0a5fe4e63151afa048b2b2bb0578	0	0	0	0	0	5	0	0	0	0	0	0
eeee31d66bfc5fd060788c201841c806	0	0	0	0	0	0	0	0	0	4	0	0
fa0663a54c99a402d801f3d0aece1018	0	0	0	0	0	0	0	0	0	0	0	3
b9c3d1bedd2ee873e53df01011e0ae79	0	0	0	0	0	2	0	0	0	0	0	0
d93193aed50d774fd6f596801837ff51	0	0	7	0	0	0	0	0	0	0	0	0
95e06bfb22b802747ec0c2b3750b39c3	0	0	0	0	87	0	0	0	0	0	0	0
bb9e86990a31449e96d127fbdcb03c47	0	0	0	0	0	0	0	0	0	0	0	4
4f0c5d46cf44bcab5112ecabe2449092	0	0	3	0	0	0	0	0	0	0	0	0
0d209c773626671cd7e7c1ba3a26cbcc	0	0	7	0	0	0	0	0	0	0	0	0
51ae895ebc7825885feda21e87e030b7	0	0	0	0	0	0	0	0	0	4	0	0
9883dcb0a1adba29f6c209c64afbadf0	0	0	0	0	0	2	0	0	0	0	0	0

5163ef5fb09b901dfa852b2df8e81fa7	0	0	0	0	0	0	0	0	0	0	2	0
5c9b98e63e5652578d2145a731c35ea4	0	0	0	0	109	0	0	0	0	0	0	0
42e9545b9f430f202042fbad2f61fbe0	0	0	0	0	0	0	0	16	0	0	0	0
0ffba2e3657301f703795412c3ede0cb	0	0	0	0	0	0	0	0	0	0	0	7
4d35ed790b980e649de5dbd49d7f49b3	0	0	0	0	0	0	0	0	0	0	8	0
8dce22ecbb061431781ffc51c9165c62	0	0	0	4	0	0	0	0	0	0	0	0
6b0d06f60fe3d6e3f63842bdb0caaf42	0	0	58	0	0	0	0	0	0	0	0	0
45e3d105b0ad35e62ff0d03319d4b6af	0	0	7	0	0	0	0	0	0	0	0	0
30891379f940801de4779fcdb04ce1ff	0	0	0	0	0	0	0	0	0	5	0	0
439348452ab6dde935b7769eff6eb304	0	0	3	0	0	0	0	0	0	0	0	0
2bdd39b8664ed44f446e244d44476249	0	0	0	0	0	0	2	0	0	0	0	0
6b404c6700059cccd89f1071498dd196	0	0	0	2	0	0	0	0	0	0	0	0
384d62b1421660cd0b1b4b8bfa319571	0	0	0	0	0	0	0	3	0	0	0	0
8f7388d36e708739f67983fa3c3dc26b	0	30	0	0	44	13	0	0	56	0	95	12

3214cec74118ca8cd45ceaf653585b6b	0	0	2	0	0	0	0	0	0	0	0	0
733181655ae0a02697c0643b5ac5f484	0	0	0	0	0	0	0	92	0	0	0	0
0a8ac58fb0312414c8640c760fba65b9	0	0	0	0	15	0	0	0	0	0	0	0
1e3b8a0707c5935bedcbaa5b7b3c57b8	0	0	0	0	0	0	0	0	14	0	0	0
1e46553486fd8e03df8b844af4010132	0	0	0	0	0	0	0	0	4	0	0	0
ac1dc8e817995f2c7d65f551688f70ea	0	0	0	0	0	0	3	0	0	0	0	0
fc51f900d43a6ccdb7b73bee20f5c8b3	0	0	0	6	0	0	0	0	0	0	0	0
93471a539678f6c83c4d8f4e6f268271	0	0	0	0	0	0	0	0	0	0	0	2
43ce2678c41a4c78d0a1f6b33bfaba79	0	0	0	0	0	0	0	0	0	0	0	6
d381e92ff1d9da605eb971fa39ad9f2e	0	0	0	0	0	0	0	0	0	0	0	2
82d60b75b6e74fbfc3a583b29f580874	0	0	0	0	0	0	0	0	0	0	0	10
ff9cf9de7038d1334e98a0bcdaf41875	0	0	0	0	0	0	0	0	0	0	2	0
473a6d0b153ff9da044944cf3c2cbfeb	0	0	0	0	0	2	0	0	0	0	0	0
b9955da383dad6140ea93bd90975685a	0	0	0	0	4	0	0	0	0	0	0	0

b0c3e79c17b00363a062a072 73b67be8	0	0	0	0	0	0	0	0	0	0	0	24
a8e5463af0f57cbcab4e9800 79828355	0	0	4	0	0	0	0	0	0	0	0	0
113c7db8368cf2f75bbd9083 05577a33	0	0	0	0	0	20	0	0	0	0	0	0
9f554155a3b93405bbed2ab1 fc48b11d	0	0	0	0	0	0	38	0	0	0	0	0
4ec0b789e959a970b63435c2 c211a74a	0	0	0	0	0	8	0	0	0	0	0	0
83f66290b9b26761af04b385 40ab5d5e	0	0	0	0	0	0	0	0	0	0	3	0
5102d6f302d770d608d6733 a3caeed46	0	0	0	0	0	0	0	47	0	0	0	0
a4ce3f7a8045f290a82218fd0 b61a940	0	0	0	0	0	0	0	0	0	0	10	0
ba3239c9c90d30fc2ad9a91d c0a24a51	0	0	0	0	0	0	0	0	0	0	3	0
f9da25d4b3b831c04fd50a66 ca65d212	0	0	0	0	0	0	0	0	2	0	0	0
ff0021589397dcd4090ef6b4 9e634a39	0	0	0	0	0	0	0	0	8	0	0	0
646e503f4e2afc536fe14de8c e8090da	0	0	0	0	0	0	0	0	0	4	0	0
5267cabcd5c0d5dc53df4e0c 6f7a548b	0	0	0	0	0	0	0	0	0	0	0	5
eb18fe2f967bcd938b6704a3 88021fd0	0	0	0	0	0	0	0	12	0	0	0	0

320d3c02c1d92cfe17fe97b6bd05dcc6	0	0	0	0	0	55	0	0	0	0	0	0
bda5fa00a7ffdb48b29113ffbd6dd964	0	0	0	49	0	0	0	0	0	0	0	0
56d9f50163f31cbdd3def9f14d8d8e91	0	0	0	0	0	0	0	0	0	0	15	0
cd04ae81a6fdf8d1cca1d8d7811a7655	0	0	0	0	0	0	0	0	0	0	0	12
1896849e9748748354b84910b4064ca5	0	0	0	0	0	0	0	0	0	0	0	21
5ea376b0846e09393b4889c9860958aa	0	0	0	0	0	0	0	0	0	58	0	0
6d2dee73f60fb4b59443ee60e852a779	0	0	0	4	0	0	0	0	0	0	0	0
3ea407d68e929de0f9a7ab52a85b4db2	0	0	0	0	0	0	0	0	0	0	0	2
f5da904bc0c77e4eab9aef75f4ed8b76	0	13	0	0	0	0	0	0	0	0	0	0
6da4712d61d3e5d5e3cac98d0a6f312c	0	0	0	0	0	0	0	0	0	59	0	0
28b9979b3e40a2196ebc0c2ea374b5fe	0	0	0	0	0	0	0	0	0	0	0	3
0afb1101d60272bf30ce27788bb13cd9	0	0	0	0	24	0	0	0	0	0	0	0
8e536b7d199306d37eeb6ed9c3ce16bc	0	0	0	0	0	0	0	0	0	30	0	0
c74cd2a073894f18811893842d23b549	0	0	0	0	0	0	0	0	2	0	0	0

e22645f6b8721c1ddc0fdcc449dd9276	0	0	0	3	0	0	0	0	0	0	0	0
c336b3d9a9fcd983b9cfc43c39096721	42	0	0	0	0	0	0	0	0	0	0	0
887e53ec94826e70815b8cc10f978cbe	0	0	0	0	0	0	0	0	0	0	0	9
11082e00d95aa072783c28f603dfc780	0	3	0	0	0	0	0	0	0	0	0	0
1c9424e7b73ab604560264324084e0be	0	0	0	0	0	0	0	0	0	4	0	0
c4f209567ca860b3a921ee24dfdc8d49	0	0	0	0	10	0	0	0	0	0	0	0
4469c07347eb2d1321c68f49b2d9fd04	0	0	0	0	0	0	0	0	0	0	12	0
0f92a420415dacc281106a6cba2be39f	0	0	0	3	0	0	0	0	0	0	0	0
72c216cbad71e244dc98825a23b95593	0	0	0	3	0	0	0	0	0	0	0	0
6b8ba460a5f62dc4a8f1de5ba1030b21	0	0	0	16	0	0	0	0	0	0	0	0
d1bf84e857ff9a0cf363269a494831a3	0	0	0	0	58	0	0	0	0	0	0	0
8dbb2297322cd289dc63577f7947cc35	0	0	0	0	0	0	0	0	0	20	0	0
0e145317bbb0be712ade6bdf24ece947	0	0	0	2	0	0	0	0	0	0	0	0
7eb9e427ab5b50dee504145da62ea4ab	19	0	0	0	0	0	0	0	0	0	0	0

d758ed4c8110bd956317792a84d0dcb4	0	0	0	0	0	4	0	0	0	0	0	0
c9a1549ae103d999d1b6770437fb00f0	0	0	0	0	0	3	0	0	0	0	0	0
467e1290773bb08efd490781fa6fd4ac	0	0	0	0	0	0	0	0	0	0	0	2
5b309ecb9b857e8df1656f159b0faa72	0	0	0	0	0	0	0	0	0	8	0	0
f0cdf758618e66e0f48ec962ccaac82d	0	0	0	0	0	0	0	0	0	0	4	0
8a2100e8bb7eb3c2991cbc8c41c2fe0c	0	0	0	0	0	0	10	0	0	0	0	0
653ec3c5fd2c6f829559a900eddcf2bd	0	0	0	0	0	0	28	0	0	0	0	0
eb9716816c028e519a37be540b26000c	0	0	0	0	0	0	0	0	23	0	26	0
45fc377e92fde3c5ea9bf41b564af0d3	0	0	0	0	0	0	0	0	0	0	0	8
39150ae2196ec3a6df3bcf7383152ad8	0	0	0	0	0	0	0	0	0	2	0	0
be5d7e90936c77889e54ef20ee36d9ba	0	0	0	0	0	0	0	3	0	0	0	0
173792fe1fce10e371474787880270e4	0	0	0	0	0	0	0	0	2	0	0	0
f175069596b93ae3ec1d1aadae67ceff	0	0	42	0	0	0	0	0	0	0	0	0
6c14b05118428b3f711ef1ed6c897351	0	0	0	2	0	0	0	0	0	0	0	0

4571f6d92412e96c827565ddd556a321	0	0	0	0	0	2	0	0	0	0	0	0
2ae11c8166a340d9338b818374f67511	0	0	0	0	0	0	0	0	0	2	0	0
9d7d16a03b104aa07fba929016956387	0	0	0	0	0	0	0	0	0	0	0	4
57b5e395002cd478c186f6042a823fe1	0	0	0	0	0	0	0	0	3	0	0	0
7cc7f8f85ffc39788ca3a976cd466354	0	0	0	0	4	0	0	0	0	0	0	0
8719cb41593d735258f188e9820ad697	0	0	0	0	0	21	0	0	0	0	0	0
d7c394cfdea08c7179dcb9f5175f9904	0	4	0	0	0	0	0	0	0	0	0	0
2b0a72e9abe689b592a352fb0e1a5687	0	0	0	0	0	0	0	0	0	0	7	0
28389b880874f07e2dc011389a3a895e	0	0	0	2	0	0	0	0	0	0	0	0
c15d6ac714d84a4d655cd4a24316a5ea	0	0	0	0	89	0	0	0	0	0	0	0
32f4e29063bd08418253950dd0547eb5	0	0	8	0	0	0	0	0	0	0	0	0
e39f297e2321bfc53f090675893ea2a4	0	0	0	0	0	0	0	0	21	0	0	0
47ac69d9841c61e681b6f33abbc7ae20	0	0	0	0	0	0	0	0	0	0	25	0
54d136a0af6f08d7424348efa9d7bd12	0	0	0	0	0	0	0	0	29	0	0	0

3dff7aed12fbf8f6b2c6f3e73b3e9514	0	0	0	0	3	0	0	0	0	0	0	0
e28b3e5daa12f486afdc0c2e2bb205b6	0	0	0	0	0	0	0	0	0	0	0	6
645a0510350462fd6905362db35995b2	0	0	0	0	0	0	0	0	0	0	0	3
ed3aa5f06b390f04b542b825ef748121	0	0	0	0	0	0	0	0	38	0	0	0
dd990c15a12306c3951442b3efcc475f	0	0	0	0	0	4	0	0	0	0	0	0
b92644e7ff63eb93bc3a29b75168a2e6	0	0	0	0	0	45	0	0	0	0	0	0
9d53ccc2c2ee73350d6ce4ff69be728d	0	0	0	0	0	0	0	0	0	5	0	0
06d170c2c7fdde164d3cade6dfb6b9af	0	0	7	0	0	0	0	0	0	0	0	0
9ef984d1669c4d9ec149d48d9f78710a	0	0	0	0	0	0	0	0	0	0	5	0
eff1022f5f5a20bdd2af1ffae306f3ce	0	0	0	0	0	3	0	0	0	0	0	0
998b08ef29c00b157a93481fa3739620	4	0	0	0	0	0	0	0	0	0	0	0
df2573c3d92f1e52dfffb75b208ddf5b	0	0	0	0	0	3	0	0	0	0	0	0
649f107f3fd8b4409117240e5ba3e4ef	0	0	0	0	0	0	0	0	38	0	0	0
978cdb6e60ab29de699f14132fab1ef2	0	0	0	0	0	2	0	0	0	0	0	0

45064cd43cfd6d45201de3cd05980fa5	0	0	0	0	0	0	0	0	0	0	7	0
104037922d0b91b9f517f842414aafca	0	0	0	0	3	0	0	0	0	0	0	0
c5205afb04f9666417b5012e20a899da	0	0	0	0	0	0	0	0	4	0	0	0
5c6e43c4ccdeff2f1f50485e0c89dac2	0	0	0	0	49	0	0	0	0	0	0	0
f36c4853c25c64ca17adabdf99f5bfc8	0	0	54	0	0	0	0	0	0	0	0	0
84d27a6395672280c97f9d1462958bcc	0	0	0	0	0	0	0	2	0	0	0	0
e2efdbc5e1a91103a1d6086cd6126e1e	0	0	0	0	0	0	0	0	0	0	0	66
5fd345f1d7a1b4eb433d73c4b00f56e0	0	0	0	0	43	0	0	0	0	0	0	0
d2556633c36065dfaa3e09e3a6aaee7f	0	0	0	0	0	0	0	10	0	0	0	0
badf6218e83e8df99b2490eab26eb3c7	0	0	0	0	0	0	0	0	0	60	0	0
c541ea53d68c49fefa1b7fdcbfb5ee2c	0	0	0	0	0	0	0	2	0	0	0	0
c62371cea35d20f4b60ebf0d34ad9c2f	0	0	0	0	6	0	0	0	0	0	0	0
25bc4f9deb5b790f072fef2a639368f1	2	0	0	0	0	0	0	0	0	0	0	0
207447ef4d6b0343941e3bb79e17e15a	0	0	0	0	0	0	0	0	0	0	71	0

fb386bb4e0ac31fe35f34d1cf2677202	0	0	0	0	0	0	0	0	0	0	6	0
cec907f448de031623c0c0ff846f2757	0	0	0	0	0	0	0	0	0	0	0	15
15b020e40c5cc81967cd8cd79590ff7a	0	0	0	4	0	0	0	0	0	0	0	0
0ce53f7ec10934220b538aa21c55925e	0	0	0	0	0	4	0	0	0	0	0	0
896b450a7e1ae7ee080d2edaf0cbc2b5	0	4	0	0	0	0	0	0	0	0	0	0
99b27d4ca807ec6f2c36219c345512fa	0	0	3	0	0	0	0	0	0	0	0	0
8a945978e752525c66ec7d0b80d7d103	0	0	0	20	0	0	0	0	0	0	0	0
f800d2d0c25dd985e58f1f483dae5995	0	0	0	0	0	0	0	0	0	0	8	0
6686b87df9de867e5424607c259ee328	2	0	0	0	0	0	0	0	0	0	0	0
93ee2a9c0a7bd13a393bb66ae3d36499	0	0	0	0	0	0	0	0	0	0	4	0
71288c55a259077d23797ab1c2f47dc2	0	3	0	0	0	0	0	0	0	0	0	0
8b7e7479e9524efbf071cd73c6fa8563	11	0	0	0	0	0	0	0	0	0	0	0
ab851078ee37173252fe54438b6d9817	13	0	0	0	0	0	0	0	0	0	0	0
ceeb614e112fd1cb93230a0c72643c83	0	0	7	0	0	0	0	0	0	0	0	0

62916c76cc9cbdfaa8f6749dceb369cf	0	3	0	0	0	0	0	0	0	0	0	0
0513afefa7e4d1e1f6f1fddc0b2de421	0	0	0	0	0	0	0	0	0	0	0	2
ad28fe03cc774a1c5450450d99a341a3	0	0	0	116	134	0	0	0	0	106	0	208
afd820d0457fdf35e8e8545f84d78502	0	0	0	0	0	0	0	0	0	2	0	0
a7a4066a00fb46966cc96b43b21ca5a9	0	0	0	0	0	43	0	0	0	0	0	0
4e6c56dd25fdefb260b31bf4e66d6f0b	0	0	0	0	0	0	0	58	0	0	0	0
687fec0e6fa3f58c887e2361238f27e8	0	0	0	0	0	5	0	0	0	0	0	0
661ccd2d00ba3f7c8ee2d5b37378d65b	0	0	0	0	0	0	23	0	0	0	0	0
849427a4b18f7c34644be9f0d2302180	4	0	0	0	0	0	0	0	0	0	0	0
a33439dbab87740b52b581837895a5e8	0	0	0	0	0	0	0	0	0	11	0	0
8f5ba16e49c19aba7fea4eb5e24a2254	0	0	0	0	0	20	0	0	0	0	0	0
7ef43e2d0916be0319e36ac3af518848	0	0	0	0	0	0	0	0	3	0	0	0
40b5fb512d41b54766ef7b5ca4b84945	0	0	0	0	0	0	0	0	0	0	0	2
cf69b5eff1580b8223302858cebbcf8c	0	0	0	0	0	0	0	3	0	0	0	0

4a9a535698febbf63bb66bb4758e72ff	0	0	0	0	0	0	0	19	0	0	0	0
0c694301f723a0e7a4719c60ba299c70	0	0	0	0	0	0	0	0	0	0	13	0
8c3e3d4b4fe2f07a390e7c97b1b67db9	0	0	0	0	0	0	0	0	0	0	0	3
af9c56986c2260685661e7d61d347c00	0	0	0	0	0	0	0	0	0	0	3	0
dc7bd520cfbc043eaeb7292347548ffe	0	0	0	0	0	0	0	0	0	0	40	0
667c8a057fc3ba47f2c45ae21285d1bb	0	0	0	0	0	0	7	0	0	0	0	0
4559eba9628114ad34e63c981fc09901	0	0	0	0	99	0	0	0	0	0	0	0
0c3f98d9c70d85a968ca011f01c46ff6	0	0	0	0	6	0	0	0	0	0	0	0
51185357f56b69bdeeab9d00c6b1ea95	0	0	0	0	0	0	0	18	0	0	0	0
6a948ee6ffa2af85b5d7db771675947b	0	0	13	0	0	0	0	0	0	0	0	0
7efe78e4d6810db82524237287ee10c7	0	0	0	0	0	0	0	0	0	0	11	0
3efa37d6d705b38c9845be6d11f98ac6	0	0	0	0	0	0	0	0	5	0	0	0
cfb92cb49aca6bf57e62b7436260aa3c	0	0	0	0	0	0	0	0	0	0	0	3
9094024b7155d06fb4363b203f9e403f	0	0	0	0	0	0	0	2	0	0	0	0

9b8a4835300ee977daa53fcea5ebe7b3	0	0	0	0	6	0	0	0	0	0	0	0
075a1d6b3c7383a70f612c0ccfb80ef0	27	0	0	0	0	0	0	0	0	0	0	0
84f60f53687b500855e88b336746497f	0	0	0	0	2	0	0	0	0	0	0	0
6146cf5a81c54fe99ed2d955c11ed692	0	0	0	0	0	0	0	0	3	0	0	0
ba20518441434f59737b60cef7777c45	0	0	0	0	0	0	0	0	16	0	0	0
e052f74f1b4f6271729746cc58cc9600	0	0	0	0	0	0	0	0	0	10	0	0
3a9c130fe4eaf3e1af6e35ed1862d7d0	0	0	0	2	0	0	0	0	0	0	0	0
1ea06b3ec5cdddc7920e2135bae66388	0	0	0	0	0	0	0	4	0	0	0	0
30574c0e9d519847bf3d26dcc20a062f	0	0	0	0	0	0	0	0	0	0	2	0
e257d7b19b0ef005e65fefe0054fbda3	0	6	0	0	0	0	0	0	0	0	0	0
e9a4be86a222e946fd0d64d6aa95db96	0	0	0	0	0	0	0	0	0	7	0	0
b351de0ca25ff4a9a5aa836caa3666ff	0	0	0	0	0	12	0	0	0	0	0	0
9ecdf240adfef6e4653f76d51218fcae	0	0	0	0	0	0	0	7	0	0	0	0
a0d22d5f1e7689905f9101e0bd0836dc	0	0	0	0	11	0	0	0	0	0	0	0

a865de74afefd6c4132ed7b2216e664a	0	0	0	0	0	0	0	0	0	0	0	4
0bf4859e23196ab2faa1fa29e19d3e64	0	3	0	0	0	0	0	0	0	0	0	0
d3bc7c4c9a7903c7059a979c4236ba7b	0	0	0	0	0	0	0	2	0	0	0	0
baa500dce3b9981d7a6fde70389fc98e	6	0	0	0	0	0	0	0	0	0	0	0
73cc1b8e159295b32e7353b52233b44c	0	0	0	0	27	0	0	0	0	0	0	0
666046b5fbd3a5b27e76a606f65d1ca7	0	0	0	0	0	0	0	0	0	0	2	0
91ffbce6f0a1945abddfbc525bb54896	0	0	0	0	0	0	0	0	0	0	28	0
980af5b35e81c680776dc46d4274a134	0	0	0	0	0	0	0	0	10	0	0	0
63885d9bd074429fe2de355c20c9430e	0	0	0	0	0	0	0	0	0	0	21	0
3bc64095279a3d30c2ca8b93d9bc4dfc	0	0	0	0	0	0	0	7	0	0	0	0
0c79ca810436cabcc5a7f0a0ebc5ef0f	0	0	0	0	0	11	0	0	0	0	0	0
01b67398e1f3866053c05160ef1fb32e	0	0	0	0	3	0	0	0	0	0	0	0
671de0b38e1f6d4d66d9ec10a38b8481	0	0	0	0	0	5	0	0	0	0	0	0
6c06db05c4f1644527b92acb55abe88e	0	0	7	0	0	0	0	0	0	0	0	0

9df14ffacad65189965fe507ec8db698	0	0	0	0	0	0	0	0	0	3	0	0
b182fa8e7564e5f761ba72254209973e	0	0	0	0	30	0	0	0	0	0	0	0
c934966df908cb90df6c2dd04629e6a9	0	0	0	7	0	0	0	0	0	0	0	0
fb84904227740457a853858c61530651	0	0	2	0	0	0	0	0	0	0	0	0
19642534666fb60c09542aed6dd4084e	0	0	0	0	0	0	0	16	0	0	0	0
786f5317ce26dff2917909b90d90e1f2	0	7	0	0	0	0	0	0	0	0	0	0
26709ff7abced2d07d6917d1816a6205	0	0	0	0	0	0	0	0	24	0	0	0
355f10bd0b64be78a256df613a644ef7	0	0	0	0	0	0	7	0	0	0	0	0
01ef50da5e5505335feddd876727f3fd	0	72	0	0	0	0	0	0	0	0	0	0
02455d80e45e8cdc2a5de252e732a3fd	4	0	0	0	0	0	0	0	0	0	0	0
033cdc39b404aa5cca7e5181b934b34d	0	0	0	0	0	0	0	11	0	0	0	0
f46cab2a33c65df2bf619684723c2414	0	0	0	0	0	0	3	0	0	0	0	0
bda979a2fb57cf5882f469bf5a9850d9	0	0	0	0	0	0	0	0	0	0	3	0
7d65b8dc5fe6d75ce1ce621cf8c2b0f1	0	0	0	2	0	0	0	0	0	0	0	0

370c15bb06d153b0365bf04762511025	0	0	0	0	0	0	0	0	0	0	2	0
ee17b7da376771bcd52be1acf8cc9b09	0	0	0	9	0	0	0	0	0	0	0	0
6f7b1fe5de78c6814dc18430ad808f1b	0	0	0	0	0	7	0	0	0	0	0	0
d18321647990960 4b051bf367c52283a	0	0	0	3	0	0	0	0	0	0	0	0
aa87134097cab9640a49121501159f1a	0	0	0	0	0	0	0	0	3	0	0	0
851f5c33fd4966fc30b728ea7202f1a9	0	0	0	0	0	0	0	0	0	0	9	0
65b9b8fdd326fe5a43138a453ca72229	0	0	0	0	0	0	0	0	0	0	18	0
086e7cd01d7c3caea21c69bdea6e5b96	0	0	0	0	0	0	0	0	0	4	0	0
bf2f18444642cb147d5d2ebb13b6c554	0	0	0	0	0	0	0	17	0	0	0	0
1585f3a7c96e989fed1e9ab486ceea0e	0	0	0	0	0	0	0	0	0	0	7	0
25ffc8639004aaf224d263725725a007	0	0	0	0	0	0	0	12	0	0	0	0
ba46e57e3c146d6833bbfba47301299d	0	0	0	0	0	0	0	0	0	0	3	0
0ac3189fce112d5448869a4617280d1a	0	0	0	0	5	0	0	0	0	0	0	0
3ac8340c84d2858474e313401698a21d	0	0	0	0	0	0	0	3	0	0	0	0

1bcbcbc6f745fdbd6c1aa8332 9d4ed37	0	0	0	0	0	0	0	0	0	0	0	5
8c039332d9bc1c624939a1a8 5e336073	0	0	0	6	0	0	0	0	0	0	0	0
7eec8a029177425c4dddf8e5 d167915b	0	0	0	0	0	0	13	0	0	0	0	0
8f34acce9519137e54e4690b 35def0eb	0	0	0	0	0	0	0	0	0	0	0	77
39f7e26b6b857a7d0eaf369fc dbdf5c8	0	0	0	0	0	0	0	0	0	13	0	0
ac20bd5b53ad1b097e989e1f 710c609f	0	0	0	0	0	0	0	0	0	5	0	0
bea8f34e300a86fcace46c744 4f69757	0	0	0	0	0	0	0	0	0	0	2	0
d6ef3bf02775b101eceb1b99 ea3000e9	0	0	0	0	0	0	0	0	0	0	5	0
b21df85d2c7c4b744128d987 61924611	0	0	0	0	0	0	0	0	0	0	0	5
a950a98c821f98b38674a964 e20e2f31	0	0	0	0	0	0	0	0	0	0	3	0
e407923a702c1bbaed2cf378 b45aab9d	4	0	0	0	0	0	0	0	0	0	0	0
aa000c02e1ea8078816e78b0 8700c58d	0	0	0	0	0	0	0	4	0	0	0	0
3eec328362abd058bb8274fa c6cce741	0	0	0	0	0	0	0	0	0	0	3	0
c92d08b6dbac200febf6c053 86ee3d45	0	0	0	0	0	0	0	0	2	0	0	0

54521ad11662bd497840b30fd6a4205c	0	0	0	0	0	3	0	0	0	0	0	0
9644955d0c54f3c511ba3686b5317473	0	0	0	2	0	0	0	0	0	0	0	0
28dcac38bdbe5578e452799ef918a4b3	0	0	0	0	0	0	0	3	0	0	0	0
e57cf6d5d842de01fa8cbd1acf2c3f11	0	0	0	0	0	2	0	0	0	0	0	0
9fb5420f92f89c2f54fd96cf455c5e3a	2	0	0	0	0	0	0	0	0	0	0	0
23a192e1cf1f18a78adfc50700bb1492	0	0	0	0	0	0	0	0	6	0	0	0
ae3de36bee0436e3bb8ef04e8f2bb158	2	0	0	0	0	0	0	0	0	0	0	0
88bae8d18642cc22274d579a85d4fa1d	0	0	0	0	20	0	0	0	0	0	0	0
3c62962198e8dc77431728f0043e1d1b	0	0	0	0	0	0	0	0	0	0	0	131
0e87dccf801723492bc70d94c4cdd5fc	0	0	0	0	0	3	0	0	0	0	0	0
94cd5b014aae5aeb12e18f3b81a63fe0	5	0	0	0	0	0	0	0	0	0	0	0
ee7881879a70efec6aa96d2ba8b397ae	0	0	0	0	0	13	0	0	0	0	0	0
1250e69b74f74453108c80fe5e441cdf	0	0	0	0	0	0	0	3	0	0	0	0
a7c81dfe328b420a710cc4c9b7ed3ac3	0	0	0	0	0	0	0	36	0	0	0	0

1d2494d47ae0b639fc0c7680bfbc5c85	0	0	0	0	0	0	23	0	0	0	0	0
283fd5c5c0ee7024ca65b83375f67978	0	0	0	0	0	0	0	3	0	0	0	0
becfb84f311600568188c906dcc0da7a	0	0	0	0	0	5	0	0	0	0	0	0
863c17f4c5a47953ce134a0d3cd46396	0	0	0	0	0	0	0	0	13	0	0	0
a618ed74eb1a59ae809329668664d310	0	0	0	0	0	0	0	0	0	8	0	0
87996ce09788edd71274a6ee997cf70d	0	0	0	0	0	0	41	0	0	0	0	0
8945aab0887ee3a661b1f4b069520e78	0	0	0	0	0	0	0	2	0	0	0	0
ccdefe4e8157b1256ce7b99dce5922a0	0	0	0	0	0	0	0	0	0	3	0	0
bd5f221490d08bcf60bb2bdb76eb3f26	0	6	0	0	0	0	0	0	0	0	0	0
5297ac1c79e5a9134a6c0387aed334a8	0	0	0	0	0	0	0	0	0	0	0	2
2ab5347db5f5ecf37b70be424e41e117	0	0	0	0	18	0	0	0	0	0	0	0
af9bca2c18c9c8e8ca8810f866a7e5eb	0	0	0	0	0	6	0	0	0	0	0	0
2e56f17d8d07787253c247ba7eeb33be	0	0	0	0	0	0	0	0	0	0	11	0
bd8dc0cee4d02c68d2402d0276453364	0	0	62	0	0	0	0	0	0	0	0	0

df36d4e947c6d5ba1a2d28c033cfb107	0	0	0	0	0	0	0	0	0	0	0	3
c2f625c8bfc44bafa678274584b196ec	0	0	0	0	0	0	0	0	0	3	0	0
e5190d95f75fa5fc8ab444c872700e7f	0	0	0	0	0	0	0	0	0	0	2	0
66912e0b5af538b22fa56fd0787f5143	0	30	0	0	0	0	0	0	0	0	0	0
50778ccb6d9ae12a068a152a4cb414ce	0	0	0	0	0	0	0	0	0	0	3	0
726ac67288c4862af623c995bb1b3f90	0	0	0	0	0	0	0	0	0	0	105	0
082cccb3efa69d3b0e8d302e5b9630be	0	0	0	0	0	0	0	0	0	0	0	13
68cb144740e48db78bd4ef4a7bf3d7f6	0	0	0	0	0	0	5	0	0	0	0	0
9fcb5a98b5f8f6689d0b105132ecd5bc	0	0	0	0	0	0	0	0	0	7	0	0
c228404d2ee32af65d7625412becc27a	0	0	0	0	0	4	0	0	0	0	0	0
5b80d3d61dcc32782256edd0708fe275	0	0	0	0	0	0	0	0	0	0	2	0
ea1f99013d3348c4634c42683ded8553	0	0	0	0	0	0	156	0	0	0	0	0
85d77cca32243043dd19b48c894a7d14	0	0	0	0	0	0	2	0	0	0	0	0
c5955afe6a632eb144453e694a357a60	0	0	0	0	0	0	0	0	2	0	0	0

de8625ee85d2f69f65404f09 64ad55cd	0	0	0	0	0	0	0	0	0	0	3	0
26506249906032 4327d18afc 9e8cc64e	15	0	0	0	0	0	0	0	0	0	0	0
15f1d95a019504186d1dc8e3 7287c9c4	0	0	0	0	0	0	0	2	0	0	0	0
e8422dc972ca563758ba67f2 14c78d5a	0	0	0	0	0	0	0	0	0	4	0	0
9a88e48298bda1598774418 6af08a861	0	0	0	0	0	0	0	0	0	0	0	7
22adaba5e5f86fcd6dab35b1 d6fa652f	0	0	0	0	0	0	0	0	0	3	0	0
74b4ebd7219bf02220e4f029 cfc374f1	0	0	0	0	0	0	0	0	0	0	0	6
9776c7dc8f3cb23972063215 3e5c5379	0	0	0	0	0	0	0	0	4	0	0	0
94d3300fcf17840111f8e0bcf e4a7960	0	0	0	0	0	0	0	0	0	0	51	0
806ec13168d999f0b65ee0e8 b1effc3d	0	0	0	0	0	0	0	0	0	0	0	6
d6aa30558722fab0d4786121 5e0d0bf7	0	0	0	0	0	0	0	0	95	0	0	0
c4dccea3c832c80b6352bb97 dba1a46e	0	0	0	0	0	0	0	10	0	0	0	0
77e223ad6db5b03b1016568 c8afb1ca3	0	0	0	0	0	3	0	0	0	0	0	0
3f6b236dd57b3ed90844a78 583e07248	0	0	0	0	0	0	0	0	0	0	4	0

7b6996145817120b9a23e0d c49c0d3a9	0	0	0	8	0	0	0	0	0	0	0	0
7be481ac4ea58ebaf9a54506 8c8fa7fc	0	0	0	3	0	0	0	0	0	0	0	0
0b059a23708e0561f75aef9c 5b3325df	0	0	0	0	0	0	0	5	0	0	0	0
d2da3ebdffd5ace69b01f574a 215971e	0	0	0	3	0	0	0	0	0	0	0	0
a87ce9b5cfda5d7ac112b68d d6967547	0	0	0	0	0	0	0	0	0	5	0	0
362ed408a9b064fe522e22df 65b98055	0	0	0	0	0	0	0	0	0	0	2	0
a01ecfb87995bba239457bcf a7ec6951	0	3	0	0	0	0	0	0	0	0	0	0
e11e7ba85f64f0f5689171cfa b398719	0	0	0	0	0	0	0	0	3	0	0	0
b08f1afdff31e177758bd6b02 23677cb	0	0	0	0	12	0	0	0	0	0	0	0
60caabedf2c79a81f4201221 7063c571	0	0	40	0	0	0	0	0	0	0	0	0
ecb712716107fb2b8eecd724 2674472d	0	0	0	0	0	111	0	0	0	0	0	0
5c6df489461090baff3331a39 4608e69	5	0	0	0	0	0	0	0	0	0	0	0
2d2ac7ea03766512950c8298 b58ad58e	0	0	0	0	0	2	0	0	0	0	0	0
7ed9ba254c3df675d7d065ea 86728e26	6	0	0	0	0	0	0	0	0	0	0	0

1856c5e95d617a404caa5146 9d552f6c	12	0	0	0	0	0	0	0	0	0	0	0
344713537c58811806d99da c2d02e635	0	0	0	0	0	0	0	0	0	0	0	2
9d2d54c0f144d9aed2380ff9a 0a7410e	0	0	0	0	0	0	0	0	0	0	0	25
e96a8c00ac25290bfc64260fc b4a60e6	0	0	0	0	0	0	0	0	23	0	0	0
7710c0dcee3564e11d26c1af b7aa2911	0	0	0	0	0	0	0	0	0	0	8	0
31d5e850cb7f7eda124844ec 801d4817	0	11	0	0	0	0	0	0	0	0	0	0
b373e174a3114c225d5690a d7cc8195b	0	0	0	0	0	0	0	0	0	0	3	0
1a13e4a9d602b06b4895a62 a55ecb96f	0	0	0	0	3	0	0	0	0	0	0	0
533abff1de31f408437eee13 5cd3a028	0	0	0	0	0	0	0	9	0	0	0	0
592c3b749442960d2d1ffc49 b44185f4	0	0	0	0	0	0	0	0	0	0	5	0
ea6afee5ecfa7fce0bef249af9 72efd4	0	0	0	0	0	0	0	2	0	0	0	0
b9212fd3bb70f215752fd73e 5469aa9a	0	0	0	0	0	0	0	11	0	0	0	0
608f89884ab22b86aa9e335b edffef0c	0	0	0	0	15	0	0	0	0	0	0	0
c7857df8f8102f333e7e0e189 3c0acff	0	0	0	0	0	3	0	0	0	0	0	0

1ee227c4c2fe13ef2f1a48e2e3f8c76d	0	0	0	0	0	0	0	0	0	0	6	0
6fbf76ad19f29f7bdb8a0145762ec2f0	0	0	0	0	0	0	0	0	0	0	20	0
c17aa037b972af8c63e8d2dba067fccb	0	0	0	39	0	0	0	0	0	0	0	0
4387328544b614edcddc76397db8a464	0	0	0	0	0	0	0	0	0	0	0	3
592c55f51e8dd89fc0327d54b65ce10e	0	10	0	0	0	0	0	0	0	0	0	0
e22c0138babda81ef9b36325c353b84a	0	0	0	0	0	0	0	0	0	0	50	0
f114e976bba6feb0b49aa93729a25e3e	0	0	0	0	0	0	0	0	0	0	3	0
8f84ed4748e29f961fc0bf902bbd6d21	0	0	0	0	0	0	3	0	0	0	0	0
8701064aa91ebdd59fc8aee19ef43545	0	0	0	0	21	0	0	0	0	0	0	0
d84f91cd0d62e7ac3d84c6c11fde6b1b	0	0	0	0	0	0	0	0	0	0	9	0
b1797eb3776b3fcc1e00c786112c29c1	0	0	0	0	0	0	14	0	0	0	0	0
e374002bd93194c00ed50cf19973f9da	23	0	0	0	0	0	0	0	0	0	0	0
52cdc1ceb840985ccec3181cb4827a71	0	0	0	0	0	0	0	0	0	0	0	7
88951fe756fc6426c47580da78101e3a	0	3	0	0	0	0	0	0	0	0	0	0

3a348fbab7d9399a7672fdfe58f11984	0	0	0	0	0	0	0	0	0	9	0	0
d67ed6db7627cbcdb49744e15b2603bf	0	0	0	0	0	0	0	0	3	0	0	0
d93bda00316158b518c37c04057dfcda	0	0	0	0	0	0	0	2	0	0	0	0
abd0a624b3d5932151c253a38d7acad6	0	0	0	0	0	0	0	0	0	0	0	5
0f2226cba0809ae0c5b1fa4bade256e0	0	0	11	0	0	0	0	0	0	0	0	0
5e57d92ac6588f9e6cc65cde429808f3	3	0	0	0	0	0	0	0	0	0	0	0
f283e80abd679ba5598f62764b572c4c	0	0	0	0	0	0	0	0	0	0	3	0
43cbe2b5752814f3a0cf46b4f7a3a740	0	0	0	0	6	0	0	0	0	0	0	0
8eddd2623c767f044e0ef2c78ba7a152	0	0	19	0	0	0	0	0	0	0	0	0
4c1650d9a9b166039cf78841b194b424	0	0	0	0	0	0	0	0	0	11	0	0
6566a419bbbd8b8ed0af578be9841a41	0	92	0	0	0	0	0	0	0	0	0	0
4c59f03045a4388fc77ff361b1dab452	0	0	0	0	0	0	0	0	0	0	0	14
12340ab5705c482d8c3e95f6d3240e73	0	0	0	0	0	0	0	0	0	26	0	0
9dfbc633c5e49aaba3c46301c1a3daf3	0	0	0	3	0	0	0	0	0	0	0	0

efa96ac724e03cf1481b6bc12e9a0295	0	0	0	0	0	0	0	0	0	0	0	4
142d22f213fc08dc9975ecb5dc16ce5e	0	0	0	0	0	0	0	0	0	4	0	0
e1cc9fe1fe82f62660d5062fe74b449f	0	0	0	0	0	5	0	0	0	0	0	0
ef9fb157f3dcc8c4c541928fba6ec412	0	0	0	0	0	0	0	0	0	0	0	7
f75dd4743a290fc0326cfab692ea3ddb	0	0	0	0	0	0	0	0	0	0	154	0
0ab491f1638e096a377ea1d8b03423a5	0	0	0	0	6	0	0	0	0	0	0	0
08c69b51c8804306e6fd306461d8f9dc	0	0	0	0	0	0	0	0	0	0	269	0
9c20ec8961b8cd97e33bb157a11113bc	0	0	0	0	0	0	0	0	0	0	2	0
f47e4251c64b71c010255b95a898435a	0	0	0	0	0	13	0	0	0	0	0	0
b19572a737445e628a1b02e5caaf2496	0	0	0	0	31	0	0	0	0	0	0	0
ff140bcf75b267e727ecdfc7cfbcbe06	0	0	0	0	0	0	0	3	0	0	0	0
fd14b888aa61694302cb87cba65a2b36	0	0	0	5	0	0	0	0	0	0	0	0
5304ad30d36c18ee396c72fbd423df50	0	0	0	0	0	0	0	45	0	0	0	0
b7721d34d984102cf9bad85b53d36041	0	0	0	111	0	0	0	0	0	0	0	0

529ad20d2e213512c022b6a3ebf65b6c	0	0	0	0	0	0	22	0	0	0	0	0
438d8514c949df37adbc6e50a31de3cd	0	0	0	0	0	0	0	0	0	0	0	5
dc4fe248b61d0d8d37ab0668634cd94f	0	0	0	0	0	0	0	141	0	0	0	0
6873bfc754181961ee16dfd495f01f0a	0	0	0	0	23	0	0	0	0	0	0	0
a22d12a3317ae2c0ba88c050064b86b7	0	3	0	0	0	0	0	0	0	0	0	0
2319f391d93c2d2506e5d4114b8242f3	0	0	0	0	0	0	0	3	0	0	0	0
f75bf3440ee9628da402bcc01dee9cac	0	0	0	0	0	0	0	0	0	0	0	3
441509a090974c8b12042a2da2b0011e	0	0	0	0	0	0	0	0	0	0	0	17
68d3b7bf2d3fa5342a127d35441e8b94	0	0	4	0	0	0	0	0	0	0	0	0
eb7a40cc1c96a2d4a04047a9970fd1e0	0	0	0	0	0	0	0	0	24	0	0	0
680a18288d9159e5de2635a3b8fe729d	0	0	0	0	0	0	0	0	0	77	0	0
d90c30d863b3c8024b452fb40043f0cf	0	0	0	0	0	0	0	0	4	0	0	0
69716ee84a92c87536770adea03f367f	0	0	62	0	0	0	0	0	0	0	0	0
eec99004013f043f60b1d09cf0cc6f7b	0	0	0	0	0	0	0	0	0	0	0	56

f1ee4802207ff42f676e33751 0884bd3	0	7	0	0	0	0	0	0	0	0	0	0
ff845da9fd00d939cd757e21d 185d2b4	0	0	3	0	0	0	0	0	0	0	0	0
a927e22353f048a4d00bc79e e81c5556	0	0	0	0	0	0	0	4	0	0	0	0
e06724e1d03c2340519bfa47 65ef9d51	0	0	0	0	0	0	0	6	0	0	0	0
3c2e8f18994844644fe80a85 3d0cdd5b	0	0	0	8	0	0	0	0	0	0	0	0
709639cbfde17b279e1a645f df9f1953	0	0	0	0	0	0	0	0	0	0	0	29
6dba624c0264d4b36a53e10f 37a2d106	0	0	0	0	471	0	0	0	0	0	0	0
495475a6f04b0ecc40402c0ff 09fb002	0	0	0	0	0	0	0	0	0	0	0	2
53f5e555eda424616f8ea6d8 17304821	0	0	0	0	0	0	0	0	0	3	0	0
dc5e18a65fbea145349f2b90 050fb1b1	0	0	0	21	0	0	0	0	0	0	0	0
d4332d43bf3bbd921f88279c 1d56b99b	0	0	0	0	0	0	0	15	0	0	0	0
4058a2b68c77811d8f0c69dd 43df01ae	0	0	0	0	0	0	0	0	0	0	57	0
55f1d3ad1bf13c2aeef4b490a 8281f4f	0	0	3	0	0	0	0	0	0	0	0	0
39df271f1155027a2193c009 b85a21ef	0	0	0	0	0	0	0	0	0	198	0	0

031360b2a25a105d518f63cf1ba520cc	0	0	0	0	0	10	0	0	0	0	0	0
a1fdcd4dc9e26a88605f2b6cfd41fbc8	0	0	0	0	0	0	0	0	0	0	13	0
aff251c1a88f34bdf3ac4d61a9a8e309	0	0	0	0	0	0	0	0	0	0	13	0
c494213f8d3e358e0088d7f1e6b3ebf7	0	0	0	0	0	0	0	0	0	0	0	7
cbfcdc74ea20e684481b234d36539f0a	0	0	0	0	0	0	0	0	0	0	2	0
87061be848324f5b4d4445d1d61902cc	0	0	4	0	0	0	0	0	0	0	0	0
b6c55a335f4727feea741a556ff25df7	0	0	0	0	0	0	0	0	0	0	15	0
c5054ddb0fdc35aab99bf09728514f2d	0	0	0	0	0	0	0	18	0	0	0	0
07b03932fc9664590070783cc6a54da7	0	0	0	0	0	0	0	0	5	0	0	0
1523195fb28a919392b1332e2b2e9f9b	0	0	0	73	0	0	0	0	0	0	0	0
5bec43a33157b9503a40d294aa228965	0	0	0	46	0	0	0	0	0	0	0	0
9d8f94163f8bf60bd22afd0fe213734c	0	0	0	0	0	0	0	0	0	154	0	0
82b69fff1ee71c6876e3086b178fa808	0	0	0	0	0	0	0	0	0	0	38	0
a6d280257d7bf73d48aed26eb311111f	6	0	0	0	0	0	0	0	0	0	0	0

3fe3bf3d87493e6df7090e972a90d9d7	0	0	3	0	0	0	0	0	0	0	0	0
f3a5108821b5a8e9a5d3604c6b79d6c2	0	0	0	0	0	0	17	0	0	0	0	0
2df7ea33f341b1c2beda4c2da45dd9cd	3	0	0	0	0	0	0	0	0	0	0	0
867c00477573e34304bcbf8ef0a44b8a	0	0	0	0	0	0	84	0	0	0	0	0
491729cc5c4d822485b9cf40b59b8339	0	0	0	0	0	0	0	0	0	0	2	0
3ae36fdfab4fc065851be06d591edad2	0	0	0	0	0	0	0	0	3	0	0	0
e1f1257c8fb44730e9fd401fab317bde	0	0	0	0	0	0	0	0	745	0	0	0
b6af030b432ce06dc8d568033ed7221e	0	0	0	0	0	0	0	0	0	0	2	0
2c1f2047e170fd32f220a5bc62251dcc	0	0	0	0	0	22	0	0	0	0	0	0
3b2f9b8955e5264d2402042a97806e58	0	0	0	0	0	0	15	0	0	0	0	0
599cf02aa9eb6b53d64c4b9259639010	0	0	0	0	0	13	0	0	0	0	0	0
403b8dbe608f120222db40c2dba9a7ac	0	0	0	0	0	0	200	0	0	0	0	0
81d3d5c459a249fd0dc1423ed0c37a43	0	0	0	0	0	0	0	0	0	0	0	3
cf4c5eb7ed4d7f216fd3b03df1949d75	0	0	0	0	3	0	0	0	0	0	0	0

34d40a6e1643b2fc3613b91cf7d2242d	0	0	0	0	0	0	0	0	0	2	0	0
7db70c0bf77cef89c8c0fd79896b5c6b	0	0	0	0	0	0	0	0	0	0	7	0
6f054ecc1cb2a3b2a84517e7d84d80e2	0	11	0	0	0	0	0	0	0	0	0	0
fad5024c817094d161e3ec707ec9363b	0	0	0	0	0	0	3	0	0	0	0	0
091ef6e776b2c4bdf98a7efeefa0f95e	0	0	0	0	0	0	0	10	0	0	0	0
2d65783ecfaa348e6be3e1e3ceefcb04	0	0	0	0	0	0	0	0	0	0	0	3
85768a26913def369ca6e6dcdff3fda1	0	0	0	0	0	0	3	0	0	0	0	0
d2a83edf829ae2b80548e2d949353673	0	0	22	0	0	0	0	0	0	0	0	0
7a7b01317dfcd35d7982cb7ca826ba49	0	0	0	0	0	0	0	0	0	0	2	0
861f633e9be6264bd4a0de30736be6c1	0	0	163	0	0	0	0	0	0	0	0	0
daef599a5ebc1d28e409422bcb9d1813	10	0	0	0	0	0	0	0	0	0	0	0
d4de23163fa50df3e15e50eff0de98bc	0	0	0	0	0	0	0	3	0	0	0	0
08256c96b1f72183d55ab6713b7bdd3a	0	0	0	0	0	0	0	0	6	0	0	0
251876e40ff0812a695deda5b27dd293	0	0	0	0	0	0	0	95	0	0	0	90

b6cf8e53bead6bee6809c5525ae0d234	0	0	0	0	0	66	0	0	0	0	0	0
8238fed3ec305d34f34f0c3df7c68bfe	0	0	0	0	0	0	0	0	5	0	0	0
111ec01a232121189fdfd6e9bf7744d8	0	0	0	0	4	0	0	0	0	0	0	0
db43f4973419705d7fff0ac8ab5443a4	0	0	0	0	0	0	42	0	0	84	0	123
dd1565f69df8df04c67bc45c23967866	2	0	0	0	0	0	0	0	0	0	0	0
3933eb1a09600e7ed56c6e1cc20790f6	0	0	0	0	0	0	0	0	0	0	0	14
ee2cbc310f0dcaf0bcaa599cebf02ce1	0	0	0	0	0	0	0	0	3	0	0	0
164df03c3835fe33cdb3f84e0f04721c	0	0	0	0	0	0	0	0	0	0	0	2
0ddfe4704408bd9fe722e3cc0b7a4632	0	0	0	0	0	0	0	2	0	0	0	0
1a2bd36a76a3b7dfee80eedbed67caa2	0	0	0	0	0	0	0	0	2	0	0	0
16e9b31084235ad56efde8acb689c7a5	3	0	0	0	0	0	0	0	0	0	0	0
53d17c44868e30e64c049e7c9ae23970	0	0	0	0	0	0	0	0	0	0	3	0
b3b85837dcf15c16a52b1153916dbe19	0	0	0	0	0	0	0	0	16	0	0	0
f2e02d50993ac4ef370df08645553e45	0	12	0	0	0	0	0	0	0	0	0	0

d9945fc04c607a3109719f37 10e5c9e4	0	0	0	0	0	0	0	0	2	0	0	0
3114e62c7a5ba3e0d8e61c25 dee57e26	0	0	0	0	0	4	0	0	0	0	0	0
81b22b231da394398903f16f a7e0882d	0	0	0	0	0	0	0	0	4	0	0	0
86ea64ffbc302a1e04135b68 9c1654c8	0	0	0	0	0	0	0	0	0	0	8	0
36e87cee465480e2851c23c1 fbbf945e	0	0	0	0	0	0	0	4	0	0	0	0
eb7ce75d07bc5ca0f22570b3 cac57496	0	0	0	0	0	0	0	0	0	0	0	15
5ab64544824b9820868a435 05b1203c9	0	0	0	0	0	0	0	0	0	8	0	0
10183464a6e00704b22acbb 90d95f259	0	0	0	0	0	0	0	0	0	0	4	0
968e8ed15297ca3803915fda c5bc17b7	0	0	0	0	0	0	0	0	4	0	0	0
7eca4562d994593823b1f462 fdac6fa8	0	0	0	0	0	15	0	0	0	0	0	0
cba0e08443dad9f2cb42d110 c6835997	0	0	105	0	0	0	0	0	0	0	0	0
f13799b244fa0b4c48a0443e e2706ef5	0	0	0	0	0	0	0	0	0	0	5	0
ca79b1babb90113c005828c1 59edb0cb	0	0	0	0	0	5	0	0	0	0	0	0
881dc003f0f41d289ed23ba1 2240f367	0	0	0	0	0	0	0	0	0	0	3	0

25305ac94e510aa2515bcf18b89242c5	0	0	0	0	0	0	0	9	0	0	0	0
d94e8547ea792eaade232f6c5c9cb7b1	0	0	0	5	0	0	0	0	0	0	0	0
abcc063666379e719fc23fb0b6e0bc30	3	0	0	0	0	0	0	0	0	0	0	0
f3a1be881915c8b92b5a6df268d871f2	0	0	0	0	0	0	0	0	0	0	53	0
f9b71959c70f37202a212305ff2f9934	0	0	0	0	0	0	0	0	0	0	6	0
1cf110dca86a3d0336cd1099c18c1fcb	0	0	0	0	0	0	0	0	0	0	2	0
ffb9f2970dd248d80d7ba0cc6374c7e5	0	0	0	11	0	0	0	0	0	0	0	0
1ce75e359bbd169ca6c8954583c15b25	0	0	0	0	0	0	0	0	0	0	2	0
c1c4f270097ba7c7d8cd36a721914670	0	0	0	0	0	0	0	0	0	2	0	0
a7b09284542dbf6e4a869e01eafd8afe	0	0	0	0	0	3	0	0	0	0	0	0
d75e37357b5ff3b0882cdc7d6d01d5a4	0	0	0	0	0	0	0	76	0	0	0	0
89bc0d9d4fd571f872cb6fb7e65ce285	0	0	0	0	0	0	0	0	0	0	26	0
651708f25bcd00f1661d0b0b304699c3	0	0	0	0	0	0	0	0	0	8	0	0
493c4636f37c6da59a8b5520e7215041	6	0	0	0	0	0	0	0	0	0	0	0

65b706b7851c10b2f85431756d49878c	2	0	0	0	0	0	0	0	0	0	0	0
b539387c554f632a8fc8dee1ef936a44	0	0	0	0	0	0	0	0	0	0	3	0
d63bdf7d037621766272a46d23d78c3e	0	0	0	0	0	0	0	0	0	19	0	0
aaebbd0f071892e83f41fd997fdd556e	0	0	0	0	0	0	0	0	9	0	0	0
02e3e8fc3a059b59114232748cd8d1cf	0	0	0	0	2	0	0	0	0	0	0	0
4ecea36aa99df555996e07fcbb088b8a	0	0	5	0	0	0	0	0	0	0	0	0
9c527a3fdd28d50c94baf8dfb5c15c42	0	0	0	0	0	0	0	0	0	0	4	0
7fd8b793990c4c62201e119fa73ff171	0	0	0	0	3	0	0	0	0	0	0	0
b6297f55b1ae431434a8e2aca1377f0e	0	0	0	0	15	0	0	0	0	0	0	0
06cb36d54e384eaac3554bfa5dc32d98	0	0	0	0	31	0	0	0	0	0	0	0
a68e63c099eebc9207603357d21bbbe2	0	0	0	0	0	30	0	0	0	0	0	0
f6ebc06662ed1f058ac6ed09b05ed0ad	0	0	4	0	0	0	0	0	0	0	0	0
0448629d3bd115e0e015eb0494536210	0	0	0	0	0	0	0	0	0	0	0	3
5a028674affbd7933d97f484b78eaf62	0	0	0	0	0	0	0	0	0	5	0	0

4c75a551b1064faa67587b8f3bcdd7f1	0	0	0	0	0	0	0	0	0	0	14	0
8599731d06e09ad937aeec614f810d17	0	0	0	0	0	0	0	0	0	0	0	13
4e4c8c92c67790557c9b3b74d13ecc0e	0	0	0	0	0	0	0	0	2	0	0	0
dec0882111eea14153892ce1e024b03e	0	0	0	0	0	0	0	0	0	0	4	0
953884e33a372d0ba766b71c18626765	0	0	0	0	0	0	0	0	0	0	0	4
43051a414a0766ae91bfe5edb13008de	0	0	0	4	0	0	0	0	0	0	0	0
2447af19b6f2b67e30cfc7cbd9fc17db	0	0	0	0	0	0	0	0	0	0	0	8
dd3ad8ec0a0489e700b65a7eeb6082e7	0	0	0	0	0	0	0	7	0	0	0	0
c7b79b0b4ed3d8c072d229c593f09c15	0	0	0	0	0	4	0	0	0	0	0	0
d575f6ef2237acf4f760d030de98fd68	0	0	0	0	0	3	0	0	0	0	0	0
67480845d2a4960eceb8977b0e5f0dfd	123	0	0	0	0	0	251	0	0	0	0	283
1c19868942a8e80e4397b735aed8c4a0	0	0	0	12	0	0	0	0	0	0	0	0
42bcac0c41c688a4a571db53d7889da1	0	0	0	0	0	0	0	0	0	0	0	3
271e548d94e2172182e9f117a178648c	0	0	0	0	0	0	0	0	0	0	3	0

471558b23d30c153d108a36beeb8b5ef	0	0	0	0	3	0	0	0	0	0	0	0
b47c5a8a97f1288e3aba8429b256091c	0	0	0	0	0	0	0	0	0	0	2	0
fdf58da5caf6bec7f8654469c35004da	0	0	0	0	0	0	0	0	2	0	0	0
920e9ec4bcae4f3f91d7e9c9a9711d8a	0	0	0	0	0	0	0	0	0	0	0	8
9bc46f05ced401e94135a3b958684d6d	0	0	0	0	0	0	0	0	0	0	31	0
17949e36b3d274b3bcb6945da1e025d9	0	0	0	0	0	0	0	251	0	0	0	0
ca553b2da1dacb19d3be4e2de271edc5	0	0	0	0	0	0	0	0	0	0	0	10
7860ef4312c9b5e18ad5530b7c7d2dbd	0	0	0	0	0	0	0	0	3	0	0	0
dce6d7ed31d2ab61fc2c4d84a681d57f	0	0	0	0	19	0	0	0	0	0	0	0
c273a7508d9288de860578f571580507	0	0	0	0	0	3	0	0	0	0	0	0
83701769291f9fc5a44bc5e97507bec7	0	0	0	0	0	0	5	0	0	0	0	0
d0770e66b58f24f0a97fe6893c2c337f	2	0	0	0	0	0	0	0	0	0	0	0
e75dc2871e6bfe378997c220423015d9	0	0	0	0	0	0	0	0	0	0	40	0
36875ddc4447274b6aca17afd7d25095	0	0	0	0	0	0	5	0	0	0	0	0

83cb484ba1d61d964bd64b37003fabe9	2	0	0	0	0	0	0	0	0	0	0	0
bd4c81d225027f05a3a34ec70d8ad147	0	0	0	0	0	0	0	0	0	0	22	0
bdf07b18cea9d44b95d806f72e684123	0	0	0	0	3	0	0	0	0	0	0	0
8ef06f2bbc1c023f48586ac9dfb86cba	0	0	0	0	0	16	0	0	0	0	0	0
8a76567bfcf77649b75db74303893859	0	0	0	0	2	0	0	0	0	0	0	0
a8491563fc0b27e0d5a8777d7c6a5ea8	0	33	0	0	0	0	0	0	0	0	0	0
9d18a353e492108bb052eed4216c2a52	46	0	0	0	0	0	0	0	0	0	0	0
d7d3d732ac98844c7cfa522dd45c03c5	0	0	0	0	0	0	0	0	0	0	4	0
dc42d6a19f9f80db55b964969f41e2a5	0	0	0	0	0	0	0	0	14	0	0	28
e5a87d864e3bab43f32c40b98be3312d	0	0	0	0	0	0	0	0	0	3	0	0
2f9a599719dd200c0fc6cdbd9041193d	0	0	0	0	0	0	0	0	0	0	3	0
120abd7befeaf008560231d8c0767ddd	0	0	0	0	2	0	0	0	0	0	0	0
938300283d94559fb64f21611c25912c	0	0	0	0	0	0	0	0	0	0	5	0
6a54351eb11f4dfc187635be27004d7a	0	0	0	0	0	0	2	0	0	0	0	0

d1fe13ce4958942a961e8bbb b742b953	0	0	0	0	0	0	0	0	0	0	0	3
8d56ea7846d558add0a7350 c91b9d390	15	0	0	0	0	0	0	0	0	0	0	0
29685383301afa05b1596bde 383113f6	0	0	0	0	0	41	0	0	0	0	0	0
6e204f6aee62dba3badb309c 47e023f6	14	0	0	0	0	0	0	0	0	0	0	0
5b1139958332b8de0a1f122f 5204abbf	0	0	0	0	0	0	0	0	0	29	0	0
10a44cfff9904cb63d137dd4d aa2a9e5	0	0	0	0	0	0	0	0	0	0	3	0
0720be94c7b7b6edfd2f8368 65d0aa72	0	0	0	0	3	0	0	0	0	0	0	0
bd49aebe45780d95f7030608 47f37552	0	49	0	0	0	203	0	0	0	0	0	155
80f7592f6d781f7a3ce57bcfb 7ecae1a	0	0	0	0	0	0	0	0	0	0	5	0
03bbef674f3303daf64a25e9 37aaed43	0	0	0	0	0	0	3	0	0	0	0	0
f7e44d564917166bac8673ba c916d3a0	0	0	0	0	9	0	0	0	0	0	0	0
dc3bf1753cc103fd19f0f8c25 85b6ca4	0	0	0	0	0	0	0	0	0	0	0	2
ee6dbf8ebab3a7e6b1e2f2ec 6b8907d1	0	0	0	0	0	0	0	0	0	47	0	0
55b7c194b0f85fc7eeb3b4c3 decb5235	0	0	0	0	0	0	11	0	0	0	0	0

756f05adf8585db4b2542547 7a77e1fb	0	0	0	0	0	0	0	0	15	0	0	0
91ecc24824882576e20791de 8006fde5	0	0	0	0	0	0	0	0	3	0	0	0
e06527c668e8c88fb5172559 4ab6d477	0	0	0	0	0	0	5	0	0	0	0	0
6941253dfd9bc581869c688d 6a1aaedf	0	0	0	0	0	0	0	0	0	0	10	0
f76d6998b2fcd50381d6983f dd8f9abb	0	0	0	0	0	0	0	0	8	0	0	0
2ea68685cc774bac239c8879 75319329	0	0	0	0	0	0	0	0	0	14	0	0
cfd39bd3b528b2680959de4a 77503f88	0	0	0	0	0	0	0	0	6	0	0	0
3d006c9571f17618fd79d3fc6 28c3529	0	0	0	0	0	0	0	0	0	0	2	0
8a89c2ef1c3883c3b30bdbab 9dfb82f3	16	0	0	0	0	0	0	0	0	0	0	0
9ebd60e094deb8d852e7482 787a31419	0	0	0	0	0	0	0	0	0	2	0	0
13e6096939acd780008e83a 9c5e8364e	0	0	0	0	0	3	0	0	0	0	0	0
c23931343eae25e53d7c266c 66f1b098	0	0	0	4	0	0	0	0	0	0	0	0
37da6fea70990787c8ac37f6a 616179b	0	0	0	0	7	0	0	0	0	0	0	0
3348638c0b2e7a493059018 42e5e4be7	0	0	0	0	0	0	0	0	0	0	3	0

8f669493417f71474a366b3b7417f2e0	0	0	0	0	0	0	0	3	0	0	0	0
384bcd91b7072bdec76a8e642eac3194	0	0	0	0	0	0	0	0	0	0	4	0
12ebf4eafd11193dcfa2a427757f0105	0	0	0	0	0	0	0	0	3	0	0	0
321c6c0b622e49da2661aaa8c8e24c93	3	0	0	0	0	0	0	0	0	0	0	0
9bbedbf0db757103ce2021c09bcc2c52	0	0	0	0	0	0	0	5	0	0	0	0
a8b13e382a0872fd51e55c16bfceb42b	0	0	0	0	0	0	0	3	0	0	0	0
035c2e473010158c9b20d0d76dd13fbe	0	0	0	3	0	0	0	0	0	0	0	0
18b0dbae13ac7570f789e75cb770bf33	0	0	0	0	0	0	0	0	0	0	0	21
b39b2aa774c5198f653e412366459863	0	0	0	0	0	0	2	0	0	0	0	0
8240775118dec12d739b5905ef4db2b8	0	0	0	0	0	0	0	0	0	0	3	0
1a7e1da791ec26c539d62744f3b6c10c	0	0	0	0	0	57	0	0	0	0	0	0
6c46c533f3c3ca5360beaf2b10cc1460	0	0	0	2	0	0	0	0	0	0	0	0
a1087c9db93ed4fc4080f3bafc6ce632	6	0	0	0	0	0	0	0	0	0	0	0
7ef2d91d4018389f67cdb145b5a2f9d5	0	0	0	0	0	0	0	0	0	0	0	34

7d69fbafbddb8351590fd491 3b9d2ae8	0	0	0	0	0	0	0	0	0	3	0	0
be83a903854e5d26f8665960 03b2a3fb	0	0	0	10	0	0	0	0	0	0	0	0
6be35e2adaca348281cf9078 d3d387a7	0	0	0	0	0	0	0	0	15	0	0	0
8fd8b69208123f07997d274b 2608a3f9	0	0	0	0	0	0	0	0	0	0	0	2
ecbbede8e59e1fef261b0e94 297e178c	0	0	0	0	0	0	0	0	0	4	0	0
ba04649a35c1678bc0eb7e6 b13f47a6d	0	0	0	0	0	0	0	2	0	0	0	0
e28b405d5fdeac4d921f0973 c012b066	268	0	492	0	0	0	0	0	0	0	0	0
898926d3e7d3718c40e0653 2a331399b	25	0	0	0	0	0	0	0	0	0	0	0
71d9155dc3580d52ae390a8c 8a193c53	0	0	0	0	0	0	4	0	0	0	0	0
cc51bd037c27faeefc1389b47 3e1d2cf	0	0	3	0	0	0	0	0	0	0	0	0
53a6df383b2199fc452ffc132 d8a49a5	0	0	0	0	0	3	0	0	0	0	0	0
97f905e6711c5c64f23b59de 6fe76c58	0	0	0	0	0	0	0	0	0	10	0	0
731eb200c9d196b31dd4e26 21205499d	0	0	0	0	0	0	0	2	0	0	0	0
87ff7e7505146cfcbdb359947 266d680	0	0	0	0	0	0	0	0	0	75	0	0

85fb5ecc283b75a7c87ed02bf864a04f	0	0	0	0	0	0	0	0	0	0	0	3
2478fd277bccac0613e5041f6e90d342	0	0	0	0	0	0	0	0	0	0	143	0
89b85bbc5007f041d7a0b8d3e101045a	0	0	0	0	0	0	0	0	2	0	0	0
e8cc048083e6cd4fe57350b15b3947b8	0	0	0	0	0	0	0	0	0	7	0	0
54119c0cc39e777419b0c1348d16e05d	0	0	4	0	0	0	0	0	0	0	0	0
4b5267b3a4a3bb245744f79d5336afba	0	0	0	0	0	0	0	0	0	0	131	0
dd39c07a36044e424c639da513f107c8	0	0	0	0	0	0	0	0	0	0	17	0
5410186d74a3e561126192844dad4bda	0	0	0	0	0	0	0	0	0	0	2	0
90f82a2d4fec293851a9e2c4b9099c8f	0	0	0	0	0	0	0	0	0	0	4	0
9c07df87420a80768d29db4f7322b8e3	0	0	0	0	3	0	0	0	0	0	0	0
be6fd48012a498a3ac78406b7655d068	5	0	0	0	0	0	0	0	0	0	0	0
fe5e5bd25a93f7a2ab87a106206f832d	0	8	0	0	0	0	0	0	0	0	0	0
a4d3dead9ee48652b10155869c1c9c23	0	0	0	0	0	0	0	0	0	0	0	20
093f1c46375c81a634dfe4c99bbc401f	0	0	0	0	0	0	0	0	2	0	0	0

25bf9d39bcd1b84c80b7c3b374be7a92	0	0	0	0	0	0	0	0	0	0	0	15
0b6107a5e8834f6c98f1002897e162e1	0	0	0	0	0	0	0	0	0	0	49	0
0bb208016cbc39610224250f0a4aa0fe	0	0	0	0	0	0	0	5	0	0	0	0
125089e6218b1fc1e04f97bf5fe8f148	0	0	0	0	5	0	0	0	0	0	0	0
481e4e239e30be504016a0fc80eea469	0	0	0	0	0	0	4	0	0	0	0	0
50b684cd67fef50dbc16bdb5767c505c	0	0	0	0	0	0	0	0	0	0	36	0
03b5b2349fb5038a0726c571a5302a00	0	0	0	0	0	0	0	6	0	0	0	0
4288da134ab593a92df80c262740fa76	0	0	0	0	0	9	0	0	0	0	0	0
463cfe00bc50064c4ac59945992e162e	0	0	0	0	0	2	0	0	0	0	0	0
7efecf27ba5d2cd9e600210518a0f61f	0	0	0	0	0	0	41	0	0	0	0	0
85944975b36a9b80135e4b5ed969775d	0	0	8	0	0	0	0	0	0	0	0	0
4e8dd2bd50a6c9b251dfda716e6452af	0	0	0	0	0	0	0	0	0	0	0	2
f94b42774fcffe2445e5fec1ec8edf88	0	0	0	0	0	0	0	0	0	0	0	11
bf5c2698a0dbda4c371fa978eadc525f	0	0	0	0	0	0	0	0	0	0	11	0

896feb270196eee8907ee151 5ed7ee91	0	0	0	0	0	11	0	0	0	0	0	0
f198effbe5c34a9a5f7a2503b 07acd0f	0	0	0	0	0	0	0	3	0	0	0	0
848a1983efe3136e0bd4d25d 003dc7ec	0	0	0	0	0	2	0	0	0	0	0	0
6cf1e935e7d74544dd880704 8784ba32	0	0	0	0	0	7	0	0	0	0	0	0
de1d94b5571a7ef88be206cb 26356c4d	0	0	0	0	0	19	0	0	0	0	0	0
8d6f9949f664f8b43f9370df7 9ae9829	0	0	0	0	0	0	0	0	0	0	0	2
6bcd7c266db775239c82df7c 8061f982	3	0	0	0	0	0	0	0	0	0	0	0
84fdc15b089b676fed32ba55 46367b75	0	0	0	0	0	0	0	0	0	9	0	0
0610576199cbb6fe33cf13a8 2fd3c972	0	0	0	72	0	0	0	0	0	0	0	0
15a954485ed645240169b53 10d206eae	0	0	0	0	0	0	0	0	0	0	0	2
d229c4e9c13704d974c85c29 104b09b8	0	0	0	0	0	0	0	0	0	0	9	0
98b74d7964601c036834c7c8 07b7f55b	0	0	0	0	0	0	0	0	0	0	0	138
a3ab575797681a15016c7c6a 28bd681f	0	0	0	6	0	0	0	0	0	0	0	0
9fa0913def540386bd6037f8 a039a405	2	0	0	0	0	0	0	0	0	0	0	0

589658c829ec84d469cf931cc997d201	0	0	0	0	0	0	0	6	0	0	0	0
68e19619cdec31000910117241bce4c4	0	0	0	0	0	0	56	0	0	0	0	0
3fffb174b807eec62f9b593157353af4	0	0	0	0	0	0	0	0	0	0	3	0
8e0ac085c5f86d8cac18488d739803fb	0	0	9	0	0	0	0	0	0	0	0	0
d116988899e47e793cb281069c1d3eef	2	0	0	0	0	0	0	0	0	0	0	0
0d436b900076f76e6690d4ef8690a905	0	0	0	0	0	0	0	0	0	2	0	0
4481ed6cb66b444354919a8adae86a47	0	0	0	0	0	0	0	0	0	0	3	0
6dcd79a1df0f966105345d99117c617b	0	0	0	0	2	0	0	0	0	0	0	0
a7d7ee0330f02f7708ce05efd63b4456	0	0	0	0	0	0	0	0	0	0	56	0
06b2883fbb60072950e61ac6d61d5bb4	0	0	0	0	13	0	0	0	0	0	0	0
c7ae1eb30510410608913e3faadb816e	0	0	0	0	0	0	8	0	0	0	0	0
6bfaecedc6efe48228c5cffcc494e1bd	0	0	0	0	0	2	0	0	0	0	0	0
3ca19b69b04c5cd1cc28789886559655	0	0	76	0	0	0	0	0	0	0	0	0
ed5f80e14934283dc705c7b75db82c19	0	0	0	0	0	7	0	0	0	0	0	0

e91560e900c879fc1b0f3d49d1d40df2	0	0	0	0	0	0	0	0	0	0	4	0
2dc525950596061d97bd4e99b5fe2af0	0	0	0	0	0	0	0	0	0	0	4	0
99d95e4c10d578eee7e7af81ec6d86b9	0	0	0	0	0	0	0	2	0	0	0	0
4e461e714ff1bd8f0e1ee94b9b36b2c9	0	0	0	0	0	0	0	0	0	3	0	0
b933b15238bca8e1b8378b4ab56fda3c	0	0	0	0	0	0	0	160	0	0	0	0
344505aea16ea1054a0376a49223dc95	0	0	0	0	0	0	0	5	0	0	0	0
a0a37cd7e270ba1fcf754c5525e6a4f0	0	0	0	0	0	0	0	0	0	2	0	0
4e8a948889384291a54ade562cb93674	0	0	0	0	0	0	0	0	0	0	0	24
0fff3c05a2f365d932e22c24eee77cad	0	0	0	0	3	0	0	0	0	0	0	0
2d1f78efeb6c0fb0d956b23ddfedde8d	0	0	0	0	2	0	0	0	0	0	0	0
56289c45d4a302ee792b8c9558be8ab8	0	0	0	0	0	0	0	2	0	0	0	0
b2304317b103e5cc436a0f9911472540	0	0	0	0	0	10	0	0	0	0	0	0
1c81071e2b4c7deaa58f5f011ce45db2	0	0	0	7	0	0	0	0	0	0	0	0
fbf54e49e2175c154b4d3bd5fd2bd2d8	0	0	0	0	0	3	0	0	0	0	0	0

f8fe02676bc5cec61d478f0e5 3e74bd5	0	0	0	0	16	0	0	0	0	0	0	0
9f6b2150079691d5783af768 18e2cd6f	0	0	0	0	3	0	0	0	0	0	0	0
3b459ac7a3c83a9eed0837b1 d1daa11d	0	0	0	0	0	0	0	0	0	0	2	0
2bec39d85b08a9f8ae5f5dbf b3b7adb4	0	0	0	0	0	0	0	0	0	0	0	49
4bbb8d921c4e00737c8acfa1 94a6d4b9	3	0	0	0	0	0	0	0	0	0	0	0
0c252b154cd25f8ed2410dfa ecc7b88d	0	0	0	7	0	0	0	0	0	0	0	0
9135a66bb06c7ba500b33e3 144c73417	0	0	0	0	39	0	0	0	0	0	0	0
4b73646ab950729828e8c3c 775736012	0	0	0	0	0	2	0	0	0	0	0	0
477b0e173f017a0c06aa2fb9 50eb4d27	0	0	0	0	0	0	0	0	0	0	0	7
3d76bafe03de6c884b223211 cccbc218	0	0	0	0	0	0	0	0	0	0	4	0
c11b0f00fe8f8e7bd3777950 0d913897	0	0	0	0	0	0	0	0	0	5	0	0
705dc40027ae99a13150897 94e3f6b23	0	0	0	0	0	0	0	0	0	0	69	0
de8f3131b7f55a6fc615f30da 5df9bd7	0	0	0	0	0	0	0	0	40	0	0	0
081e14918f0a520620051820 975ae16b	0	0	0	0	0	0	0	0	4	0	0	0

34a404ddc5d6bd129f23a0b09509623b	0	0	0	0	0	0	0	0	0	0	0	88
4929e0c55a23661ae0f6a1939d41edb5	0	0	0	0	0	0	0	21	0	0	0	0
edf0dc63a9cd39ddc610a17f9c62f51b	0	0	0	0	0	0	0	5	0	0	0	0
2c68fd4efbda6ecb597f59cacdc8797c	0	0	0	0	0	11	0	0	0	0	0	0
8574453dbcb73a0cd56aaf2dd285c081	0	0	0	0	0	0	2	0	0	0	0	0
ff4eaf797625c0637463a05c9a8cd6a4	0	0	0	0	0	0	0	0	0	16	0	0
1c80e13268727d90e1f1ab122fcb96dd	0	0	0	0	0	0	0	0	0	0	3	0
bad365439a1369f744ff732ea5aed859	0	0	0	0	0	0	0	15	0	0	0	0
404003f1cef457c92d2dac86f6fab379	0	0	0	0	0	0	0	0	0	0	0	14
611f8e1046deef0a583ee8bac84100fe	0	0	0	0	0	13	0	0	0	0	0	0
4ca4078b6a353b55c0ca8124ca6ff123	0	0	2	0	0	0	0	0	0	0	0	0
2e3f324684385f3a0b3e0276177d874b	2	0	0	0	0	0	0	0	0	0	0	0
e1dfbbd57396f73e3e03859030881139	0	0	0	0	0	0	0	0	0	0	0	5
242d9f14398ad007d2701a3d044f116e	0	0	0	0	0	0	0	0	0	0	2	0

0d098ea3f85563c1a6f37ea5c7e36f76	0	0	0	0	0	0	0	0	0	0	32	0
80273c69187f2dbb790a9500bed1a5bb	0	0	0	3	0	0	0	0	0	0	0	0
914bf364f361ff11dccc730a533854f5	0	0	0	0	0	0	10	0	0	0	0	0
078ac3b93ffa444d7ddcdc036e32e2a0	0	0	0	0	0	0	0	0	0	0	3	0
950da8314ba692dfbc06e44d75adbca1	0	0	0	0	0	0	0	0	0	0	19	0
a295ea2479929c9fb2884b56776258d0	2	0	0	0	0	0	0	0	0	0	0	0
463531747839532734e650ab03ed26b9	0	0	19	0	0	0	0	0	0	0	0	0
0eac9c2d8a71cf867dfd685c1679e5b0	0	0	0	0	0	7	0	0	0	0	0	0
d92b7c6700e3fb0a1ffb1baed306416f	0	0	0	0	0	13	0	0	0	0	0	0
c20f725f3c09dee85ac94b7cd0a97edf	0	0	9	0	0	0	0	0	0	0	0	0
19e3385ad47d17b0b8c6f3177ac65d3e	0	0	10	0	0	0	0	0	0	0	0	0
dcadde79def9ec2395e05b18293e41fd	0	0	0	0	0	2	0	0	0	0	0	0
866667264e68a5afc3b862e1b786ebad	11	0	0	0	0	0	0	0	0	0	0	0
ee9d9ad33dcf807198b98fa03bd4ac19	0	0	0	0	0	0	0	0	0	0	5	0

2165c6e8c8317e977ea146e1ff6fa406	0	0	0	0	0	0	0	0	17	0	0	0
b76b325545263cb82474293c913bd5f0	0	0	0	0	0	0	5	0	0	0	0	0
9f95471d22af772e8942c6f86c443b42	0	0	0	0	0	0	0	0	37	0	0	0
29384e9c1ae3f2371910c1c6547164b2	0	0	0	0	0	0	0	0	0	0	23	0
4bfbc4267237505ab2adb2701d725bdc	0	0	0	0	0	0	0	0	5	0	0	0
27212271768774b68219c5663e0df170	0	0	0	0	0	4	0	0	0	0	0	0
4463859b3e596a509ecbf2a7728e086a	0	0	0	0	0	0	2	0	0	0	0	0
a8f96c943f8e77b60a3b9f521a30bc7c	0	0	0	0	0	0	0	0	0	0	0	2
6ba0e2dccac04ae6bc80dea44eb09b13	14	0	0	0	0	0	0	0	0	0	0	0
37b3defacbf6d26fe70310b176978757	0	0	0	0	5	0	0	0	0	0	0	0
eac0a6eae5198265e791faa47237ca24	0	0	0	0	0	0	0	0	0	4	0	0
286512ecf3d1338dd30466d3ef5afe7f	8	0	0	0	0	0	0	0	0	0	0	0
ccb2340022b101006cf51d6f821f894a	42	0	0	112	0	0	0	0	0	0	0	0
5cf168f89c06ae8e8d21083789cebc5f	0	0	0	0	2	0	0	0	0	0	0	0

736b5752960427cd9536cdcaa630b328	0	0	0	0	0	0	0	18	0	0	0	0
0408385e76dfabf5c1a0907533798151	0	0	2	0	0	0	0	0	0	0	0	0
703364f768175babbd5d2b07ef1f5bdc	0	0	0	0	0	0	4	0	0	0	0	0
2d59b6aa12fc456dc8d3d2bee12ba8f5	0	0	0	0	0	0	0	0	0	0	8	0
29e229c7a64dff4775f4fe1fa5c87387	0	0	0	0	0	0	0	0	57	0	0	0
512cfa996460f944e39e42d168ff2de2	0	0	0	0	186	0	0	0	0	0	0	0
c1992a6194ca36c3480649dbf6faaba3	0	0	0	0	0	0	0	0	2	0	0	0
4b32086f40269e81ae8032d44e173e73	3	0	0	0	0	0	0	0	0	0	0	0
7ec711f6461f115627e77b34bb0f40fe	0	0	0	0	0	0	0	0	0	0	0	35
3bdf1e17cdea81cea354564c9fa1dd87	0	0	0	0	0	0	0	0	0	20	0	0
16329f053354a698f6ebd07673660979	3	0	0	0	0	0	0	0	0	0	0	0
f0242f65f0763ca03d3b659a32efeb36	0	0	0	0	0	0	0	21	0	0	0	0
0b553c3f7f271b2f83d120ab692a3c86	0	0	2	0	0	0	0	0	0	0	0	0
c734614e5e70c7c60e0ec20efb541ab8	0	0	0	0	0	0	8	0	0	0	0	0

ea5e0e3c1bfa9fe34ae4f5a5aecfffc7	0	0	0	0	0	0	0	0	2	0	0	0
2a24d0ad6bb4d2dd0a59cb8782112d46	0	0	0	0	0	0	0	0	0	77	0	0
722f74d8db8541c44cffb5603609f5e0	0	0	0	0	0	0	9	0	0	0	0	0
ca4257e988a926eccf1202aafe5fc87c	0	0	0	0	0	0	6	0	0	0	0	0
caf178d044510e7dc715bfbb9afef61d	0	0	0	0	0	3	0	0	0	0	0	0
6798a613575138f49d80b1698ebca081	0	0	0	0	0	0	0	0	0	0	0	2
6f6ffce6170a8bb480a6e5eff5227330	0	9	0	0	0	0	0	0	0	0	0	0
8de90d11c43861ef81f4357d637c9430	0	0	0	0	2	0	0	0	0	0	0	0
d19befa4367c3ccfc4ef8452d7f129c0	0	0	0	0	0	0	0	2	0	0	0	0
3bd6d26db812eca8a2c22467b4ace840	0	0	0	0	0	0	0	0	0	0	0	2
fd67a411ceee03924e4dfb59886b169c	0	0	0	0	0	0	0	0	72	0	0	0
a2b32bc65f4fd9d061ebf93e27f325a9	0	0	153	0	0	0	0	0	0	0	0	0
39847998cb5401bbf5720a12a0d6082b	3	0	0	0	0	0	0	0	0	0	0	0
9a37e1653e490bc0149e1ff2c3d0fa4e	0	0	0	0	0	0	0	0	2	0	0	0

20dc7300667b0581b9c47ccb83601d39	0	0	0	0	0	0	0	0	0	0	0	8
bd73a6518f2de29e17574c3ffd5c43f2	0	0	0	0	0	0	0	85	0	0	0	0
8a23722208d0c8a65b4936575d109efe	0	0	0	0	0	0	0	0	0	0	35	0
8d1393462b96835fa0c73807c989c855	0	0	0	0	0	0	0	0	0	0	5	0
08696d27d30424d7599b60ba9b59208e	0	0	0	0	0	0	0	0	0	0	0	2
1981cc5886ef544799ffcc2f91394310	0	0	0	0	0	56	0	0	0	0	0	0
e1601cd856616cc73a2d8ccf11aab956	0	0	0	0	0	0	0	0	0	0	12	0
c923da9c20e75f6a33e3b12ca61aa842	0	0	0	0	0	0	2	0	0	0	0	0
ef4c3703c3e5b1eec0d94db6e0717868	0	0	0	0	0	0	0	0	0	93	0	0
e6da7a5658dd11552c7016ef55fa25f6	0	0	3	0	0	0	0	0	0	0	0	0
d64b839a522bcf9df381414a7eafd99d	0	0	0	2	0	0	0	0	0	0	0	0
a0c9c474bb8dca22c642089e903bccde	0	0	0	0	0	0	2	0	0	0	0	0
36b103e34a04ab0e97012183b49e380e	0	0	0	0	0	0	0	8	0	0	0	0
2df89377d9c2b9717e387976f34d6ef5	52	0	0	0	0	0	0	0	0	0	0	0

7b7461989f956a4c039ed06c344b2c67	0	0	0	0	0	0	0	2	0	0	0	0
8071ec00a41f5e1ed17f4f0639ff06b3	0	0	0	0	0	0	0	0	13	0	0	0
982e53d9dc9239902fa05eba63567d72	0	0	0	0	0	5	0	0	0	0	0	0
69d110d7252528f00a7a23333085f1d5	0	0	0	0	0	0	0	0	0	0	18	0
4d1281992a1b85436c5d01e884d1e9f1	0	0	0	0	0	0	0	0	0	0	0	2
014bb5738aa4c9adcde3da847316641b	0	0	0	0	14	0	0	18	0	0	0	0
51773ee2d2842ebdbded83afd37de5c4	0	0	0	0	0	2	0	0	0	0	0	0
bd6cd5d4375d3d2e2bec4e71a4b521f2	0	0	0	0	0	28	0	0	0	0	0	0
d79896871c350aece1a637d20a218afc	0	0	0	0	0	0	0	30	0	0	0	0
42685d20d9edd4c81c4bfb930702c985	0	4	0	0	0	0	0	0	0	0	0	0
eefc8843e71e84b4141790b65973cfe2	0	0	0	26	0	0	0	0	0	0	0	0
489cac9a5434dbe83069a721cc20bbfb	0	0	0	0	0	0	0	0	0	0	6	0
c9d8635cc17c731dabe6c6727e418ea1	0	0	0	0	0	0	0	0	0	6	0	0
564a7caff06b15194cca2b770903df26	0	0	0	0	4	0	0	0	0	0	0	0

7b89e668eebd45705c773ee6a7bb35af	0	0	0	0	0	0	0	4	0	0	0	0
6e1b5345ae004b3b18086b67c4a71913	0	0	0	0	0	0	0	27	0	0	0	0
f1151fa6ef445ab92471c985020b5c71	0	0	0	0	0	0	0	0	0	0	4	0
271d5c2fc2ee91614ffac7ae64586dfb	0	0	104	0	0	0	0	0	0	0	0	0
8f0af465c61444d3a7a1f34c2216785f	0	0	0	0	0	0	0	0	0	0	2	0
130524b01f7555e87a9068e27236999a	0	0	0	20	0	0	0	0	0	0	272	0
ca54b31cda2cce95d63ce3040a610174	0	0	0	5	0	0	0	0	0	0	0	0
f9af5d2d3f64e7bd129ef7377779dcbe	0	0	0	0	0	0	0	0	0	0	15	0
f9d5f80e2221e70b688c19035437fd9e	0	0	66	0	0	64	89	0	0	0	0	0
56b7b506a4ef6688f93ddc7eef592f1e	0	0	0	0	0	0	0	0	0	8	0	0
60239020b772f3d76774a6c2f149195f	0	0	0	0	0	0	0	0	0	0	0	2
12a6843d068a50235a02c31220923b12	0	0	7	0	0	0	0	0	0	0	0	0
dc84d3a8c7062306663ad70edb557d19	0	0	0	0	0	0	0	0	0	4	0	0
54df1b6b1093b9b722ef078332df82a2	0	0	0	0	0	0	5	0	0	0	0	0

35298930afe6c29ba3a28612 40208397	0	0	0	0	0	0	0	0	0	0	7	0
09485f48e3046e0d0bc9df5b 0edafc32	0	0	0	0	0	0	0	16	0	0	0	0
2b9b5d12a42ded472522745 b39bb445c	0	70	0	0	0	0	0	0	0	0	0	0
ca99ffdcccee1e5dfad448252 c27e26f	0	0	0	0	0	51	0	0	0	0	0	0
1f3a7fb686fab92e38a19192c 8baf1d7	0	0	0	0	0	0	18	0	0	0	0	0
5dffae351478018df1dd9b68 db16f14d	0	0	0	0	6	0	0	0	0	0	0	0
769e93e11119dc9ecaabae28 086c4bad	0	0	0	0	0	0	0	0	0	0	0	6
46352c080a5bc0698625dcf0 811b3e8b	0	0	0	0	0	3	0	0	0	0	0	0
fad3db2c69acc3fc19216c7d7 70cd7be	0	0	0	0	0	0	0	0	0	0	14	0
e9f7b1cd03503eead090ed70 737b89ce	2	0	0	0	0	0	0	0	0	0	0	0
0143653562142e89e905e4b b3cbd7ef5	0	0	0	0	0	0	0	0	0	0	237	0
2619383f1e31453d964e4422 c0abd099	0	0	0	0	0	0	4	0	0	0	0	0
6237d37ecdd57add8902874 cdf65de7e	0	0	0	0	0	0	0	0	0	0	9	0
abaa6f36d3e0030527128fee 95fdb868	0	0	0	0	5	0	0	0	0	0	0	0

fc6e5579ef39fc0735101a4685d900bb	0	0	0	0	0	0	0	0	0	0	0	3
9208a343bbf93a0773afd1d90b86ba09	0	0	0	0	0	0	0	0	0	5	0	0
f45860c792167b8ac74c9a0ae9abed76	0	0	0	0	0	0	0	0	0	0	7	0
a252c15a1b4c873e0bacf5419db35e2c	0	0	0	0	0	0	0	4	0	0	0	0
bd3f720735b7080eed7c2a99f7caa548	0	0	0	0	0	0	0	0	0	0	0	20
47392475dded054d383c171cf20901de	0	0	0	0	0	0	0	0	0	0	0	13
5a7994b89df9c311939ad8167ee79934	0	0	3	0	0	0	0	0	0	0	0	0
6265d42287254553fb356b71202d8716	0	0	0	0	0	0	0	0	0	0	8	0
8ce6e3d2d5cb7be0ce408d261a0ab0a7	0	0	0	0	0	0	0	0	0	211	254	0
8fd966eda56d038451b50b2e8fde00b2	0	0	0	0	0	3	0	0	0	0	0	0
9d37e322920b4d5df9317a43f2aad04b	0	0	0	0	0	0	0	0	0	0	69	0
130df00b3d0fd2464cf40b08e7e373b6	0	0	0	0	26	0	0	0	0	0	0	0
178d35369e07007adbb412c89018e575	0	0	0	0	3	0	0	0	0	0	0	0
6a8ae6183e5b5c1ef7bd7f8ec8ee08e0	0	0	0	0	11	0	16	0	0	0	0	0

64f411cc0a146fa6da0c537d7da30f4d	0	0	0	0	0	0	0	0	0	0	12	0
59241c4c15501feedae2cf16b1821ba5	0	0	0	0	0	0	0	12	0	0	0	0
4f199886d24bc2a6ae238cf6caa808cb	0	0	0	0	0	0	0	0	17	0	0	0
df9254494dd41434473d75a49f80c558	0	0	0	0	0	0	10	0	0	0	0	0
9d3d234358a3bc9b15611af161de5372	0	0	0	0	0	0	0	0	23	0	0	0
d469920e4101c4759e24231562952eb2	0	0	0	0	0	3	0	0	0	0	0	0
e67d72b164046bcdc617ef939836b260	0	0	0	0	0	0	0	17	0	0	0	0
195e2d6f02d51477d9ff3fec3d8bfdda	0	0	0	0	0	0	0	10	0	0	0	0
a2b18cf17431627c905ed4c776c6df4d	0	0	0	0	0	0	0	0	0	7	0	0
78dacb69b5c9b850e7dc333fbe16e227	0	0	0	0	0	0	0	0	0	4	0	0
94f461d2512664198ddd41d71d48fb90	0	0	0	0	0	0	0	0	0	0	4	0
24aa14272590a9869502b67b16a789f5	0	0	0	0	0	0	0	5	0	0	0	0
5023896d85c9ab18535bc29ca973d367	0	0	0	0	0	0	0	0	0	0	13	0
f3b46ac2787f26ca1ff5ca380460b595	0	0	0	0	0	0	3	0	0	0	0	0

5ecc513d085a7f125afbef1e00781a37	0	0	0	0	2	0	0	0	0	0	0	0
fe585b790c30d0eb7dded361c905fa34	0	0	0	0	0	0	0	0	0	0	6	0
76324f8dc40846e71fd7e10fec663541	0	0	0	0	0	0	0	0	0	0	5	0
12eeac961233794ce4dbc662d2834a97	0	0	0	0	0	17	0	0	0	0	0	0
cd4a363e2de86c1f9d04deaa082d9bc0	0	0	0	0	0	0	0	0	0	0	0	3
ffcad2eaf5d86c11b973a51deec04d1b	0	0	0	0	0	0	0	0	0	0	0	12
b12e299a24033339e5acaec09a28743e	0	0	0	8	0	16	0	0	0	0	0	0
89582881fddeec79b6770cdeb0b05af6	0	0	0	0	0	0	0	0	0	0	0	3
cc515922989f82adc2ae8afed6d223f4	0	0	0	0	0	0	0	0	0	0	0	21
21ad6e1d55e1426b07ce09863906a401	0	0	0	0	0	5	0	0	0	0	0	0
d731a95af1a2924c6ffd33300a4f093e	0	0	0	0	0	0	0	0	0	2	0	0
82da1f0679aedab0b16100fb50f239fa	0	0	0	0	0	0	0	0	0	0	2	0
42082087f3d72a65e260ce80f81d22d4	0	0	0	2	0	0	0	0	0	0	0	0
30dddd4952aa3e72a44e78b6a2cef07e	0	0	0	0	0	0	0	8	0	0	0	0

b1b3763dc267cc4dc1cf4e18 30880e29	0	0	0	0	0	0	0	0	3	0	0	0
33d460f5e53bed6fedfb7bd2 e50b4bce	0	0	0	0	0	0	0	0	0	117	0	0
cc096eb3dce3819084399130 bbf84804	0	0	0	0	0	2	0	0	0	0	0	0
46d209e694c59dee2e573d0 02caad35d	0	0	0	0	0	0	0	0	0	0	2	0
c058ec44c84c33b543a36f9bf a8b01b8	0	0	0	0	0	0	0	0	0	0	0	9
7cf9f106d53c89fb57cf170e3 ebe968a	0	0	3	0	0	0	0	0	0	0	0	0
74622a2cf4b26a534a00e869 855b6ac7	0	0	0	0	0	2	0	0	0	0	0	0
9bffb2360794ec778be81b3e b2e39a5d	0	3	0	0	0	0	0	0	0	0	0	0
3f7340bd38ce1a91752fdb2e 3ea17554	0	0	0	75	0	0	0	0	0	0	0	0
9b5820c0a6513ef517cb896f 2b197787	0	0	0	0	0	0	3	0	0	0	0	0
8e100df40af4eb5c5a8158e2 3f6d6a4a	0	0	0	127	0	0	0	0	76	0	0	0
245a07f68494be0c0d7bc61f 71b7ef46	0	0	0	2	0	0	0	0	0	0	0	0
a4cde8c90f6c3d5a87a69c60 576b96d9	0	0	0	0	0	0	0	0	0	0	13	0
90e64b34b87d259f78da66f1 3d18248e	0	0	0	0	0	0	0	0	0	0	0	11

c0984209765b2c3a2550fd963af49190	0	0	7	0	0	0	0	0	0	0	0	0
ef67250f0666e9e2a6be87cabae41fcf	0	0	0	0	25	0	0	0	0	0	0	0
16cef1f2a647253775924e6e84c62849	0	0	15	0	0	0	0	0	0	0	0	0
9083859b314f5b01b1b561c62f08844c	0	0	0	0	0	3	0	0	0	0	0	0
22792ca771cdc19bf8e70db5a05b0717	0	0	0	0	0	3	0	0	0	0	0	0
c3bae11f0243ab4cbd1935b086c79414	0	0	0	0	0	0	0	0	0	0	2	0
b91898c55ea07cd1c360f6f9b148e7a5	0	0	0	0	259	0	0	0	0	0	0	0
f8a0dd87e067791388378aa9923fd035	0	0	0	0	0	0	0	0	0	0	40	0
85075b90a4d3d02c68d9151a71fb5856	0	0	0	0	0	0	0	0	0	0	0	26
02ee0134beb64b4afc696674bb9b9612	0	0	0	0	0	0	0	0	0	5	0	0
086d9a71e698155fcd0c397ec279d260	0	0	0	0	0	5	0	0	0	0	0	0
9f3d71712f4b4844a15c70a325940741	0	0	0	0	0	0	0	0	0	0	0	299
166b54037f98143913545cd82ce5b5ab	0	2	0	0	0	0	0	0	0	0	0	0
d4ef8eaebf743728b7413a4e2c814868	0	0	0	0	0	0	0	0	14	0	0	0

47628a3991c975b29c3b3ad80757e34a	0	0	0	23	0	0	0	0	0	0	0	0
d9ddae106864b5c107265772742e25e4	7	0	0	0	0	0	0	0	0	0	0	0
cd8fb88ac0b6569c0c7f67a9c6c09f38	0	0	0	0	0	0	0	0	7	0	0	0
7b5a00ba54ec9bf5e8b58990c4952473	0	0	0	0	0	0	0	4	0	0	0	0
de04ac241d6081bb172777f4abc8cb02	0	0	0	0	0	0	0	0	0	0	3	0
e0a067e74df4907f484c75eac3dd97e0	0	0	0	0	0	0	0	0	0	0	0	48
8dee9f10595797085b7969eb60fb3d6e	0	0	0	0	0	0	0	3	0	0	0	0
17703c6a400e1b4d3900211ae10d29b8	110	0	0	0	0	0	0	0	0	0	0	0
4d2627b6c8f14030f24167ad105fdde7	0	0	0	0	0	0	0	0	0	27	0	0
079ae68aff122bec47cb60f73416aef7	0	0	0	4	0	0	0	0	0	0	0	0
bcab6503e09445c077bff07e3d0d48f3	0	0	0	0	0	0	6	0	0	0	0	0
e510cfbed76d9b08c6b8d0dd30b1f5c2	0	0	0	0	0	0	0	0	0	7	0	0
58c7c24382211f56aa6e385b4198ae57	0	8	0	0	0	0	0	0	0	0	0	0
ae709201a84ef8aa16845e48474931d2	0	0	0	0	0	0	0	0	0	0	2	0

d777e5bacd0534cc744f55f991002981	0	0	2	0	0	0	0	0	0	0	0	0
21d58593b2d56ac01d3065434f529651	0	0	0	0	0	0	0	2	0	0	0	0
2dab201d1a4ac22820df8d12951668c5	0	0	0	0	0	33	0	0	0	0	0	0
066283a4e1577a20bea0440ee597c82b	0	21	9	0	0	0	0	0	0	0	0	0
88b667f8720551c7d265b4ed3116c9b3	0	0	0	0	3	0	0	0	0	0	0	0
cfe676cf3442fb1f77776366d0019546	0	0	0	0	0	0	0	0	0	0	0	149
77abf81d5d2a87b1c4b6f8aee7180234	3	0	0	0	0	0	0	0	0	0	0	0
e86ba2500f1b14d94bc3b8a2f0c7f3ed	0	0	0	0	0	0	0	0	6	0	0	0
04339138526472b9388d15edfc52744d	0	0	0	0	0	9	0	0	0	0	0	0
ee0fcc6c06c52c3ea7c967c51d15146f	0	32	0	0	0	0	0	0	0	0	0	0
e1becda1c9567e1ca214a7066c42b007	0	0	0	0	0	0	0	0	0	263	0	0
a944e53eeec45df684c7c223f9fa5c14	0	0	0	0	0	3	0	0	0	0	0	0
5005c589b1fe199c57bf28b3fd32990f	0	2	0	0	0	0	0	0	0	0	0	0
785a1fc286bc9de7c7d15fd121c9d2a4	0	0	0	7	0	0	0	0	0	0	0	0

6aa99f0821a0a9bf7126019d96514550	0	0	0	0	0	0	0	4	0	0	0	0
e5717243d97142c333ca56f0a5301c77	0	0	0	0	0	5	0	0	0	0	0	0
11d31b871229d9e4cfaa1c7ec1e69789	0	0	0	0	0	21	0	0	0	0	0	0
26f196f0f2b7fb4b7f2a3115e9a01cf3	0	0	0	0	0	0	0	0	0	0	2	0
2342a4edaaceab5c4046beb6b6c02d0b	0	0	0	0	0	0	0	0	0	7	0	0
5f7be267dd47d8a408b19268c1c20ff9	0	0	0	0	0	0	2	0	0	0	0	0
a948bd200b94738438e530c4a2e73147	0	0	0	0	0	0	0	0	0	0	2	0
de308ccf94a9248263b8c29100b4b466	0	0	0	2	0	0	0	0	0	0	0	0
a5ab7ce97cab8f3cc3501c347cd0efc6	0	0	0	0	0	2	0	0	0	0	0	0
7ddbae4f3fcc4461141bf0c713c47b60	0	0	0	0	0	0	0	0	0	0	10	0
5bb98edcedcc09bf2c66cb679492f282	0	0	0	0	0	0	0	0	0	0	3	0
2ce505bf74f9c69e4f442afd5e154486	0	0	0	0	0	0	0	2	0	0	0	0
699040d237c65d2561ca1a62824e8791	0	0	0	3	0	0	0	0	0	0	0	0
53508bf3c5462ba6af6aa25b254eb33b	0	0	0	0	0	0	0	3	0	0	0	0

f830daa497cb8b866c729fe015254b37	0	0	0	0	0	0	10	0	0	0	0	0
ba33feae1b6ef9090175f272c85829c5	0	0	0	0	0	0	0	0	0	0	0	3
04b384d404e68a0a1a4fafdfc781ec23	0	0	0	0	0	0	0	0	0	0	0	3
0c0cc5c770d4b251a0a35315d5026595	0	0	0	0	0	0	0	0	39	0	0	0
3239332421a7cdcd21fa76dc196d1f1d	0	0	0	0	0	0	0	0	0	0	0	4
2278a6bc82cd4ed980c6764197e90c61	0	0	0	0	0	0	0	0	0	0	3	0
b602164240c0014d4f8c445b24f79ccc	0	0	0	0	0	0	0	0	0	0	0	2
70b8db23eb7fabfab7ef1c0517d553b0	0	0	0	0	0	0	0	0	0	27	0	37
ed4b80f2e876872abd6e124463aa9ced	0	0	0	0	0	5	0	0	0	0	0	0
ed22355fe5f32d599c09dc6176913eca	0	0	0	0	0	0	0	0	0	9	0	0
bff33a0d9e13e9ec242fe3ac1d4427d2	0	0	0	18	0	0	0	0	0	0	0	0
5f04fd0e02b84c6657ad4e92817c7d86	0	0	0	0	0	0	0	0	0	0	5	0
6ae7162258c57cf0c389f198fc107338	0	0	0	0	0	0	0	0	62	0	0	0
01420f81717a798bce23c5c76e695ca4	0	0	0	0	0	0	0	2	0	0	0	0

88bc68d3ff15d0e4eeee4ed81fbf0511	0	0	0	7	0	0	0	0	0	0	0	0
e431e26779f2cdd911f7970bc6a4bfc3	0	0	0	0	0	0	0	0	0	0	0	17
ce88a625a6e007da1d921e4f9dcd756a	0	0	0	0	0	0	0	0	0	2	0	0
22592c43ff3201a99567baf133064dbe	37	0	0	0	0	0	0	0	0	0	0	0
8b021e0f0eaa07c5ff5f45e0d029d9ae	0	0	0	0	0	0	0	0	38	0	0	0
e6e3f1eb717c8cf06be914ce511f8737	0	0	0	0	0	0	0	0	0	9	0	0
812d9cd6b2144106df63ec442c649b48	0	0	0	0	0	0	0	4	0	0	0	0
caa700b2ce5b7554682813885a464c26	0	0	0	0	0	2	0	0	0	0	0	0
e26820a92b0c088a7066f2f8e6b40b36	0	0	0	0	0	0	0	0	0	0	0	5
91d4dd05f55edf56be55f79c10fde0f3	0	0	0	0	0	0	0	2	0	0	0	0
719400ab5fc4921c87130b918ba6b46a	0	0	0	3	0	0	0	0	0	0	0	0
c8ebbd9a7c803ef94e27a3eb846176e8	0	0	0	0	0	0	0	7	0	0	0	0
2a32735ce5d1aa60af80c153bafabb5c	0	0	0	0	0	0	0	0	0	0	0	4
e4a8dc0fa2dea8bb80e254b9d8aecc7c	0	0	0	0	4	0	0	0	0	0	0	0

8243c517e39e1381804aebb7fac3d04a	0	0	0	0	0	3	0	0	0	0	0	0
41e582bb3a1189d3f9e69bcaa4cedd95	0	0	0	0	0	0	0	0	0	3	0	0
21e207abd3b592bc43433a5a6c443ff4	0	0	0	0	0	0	8	0	0	0	0	0
960d34db2f080982dbb496b88fca16be	0	0	0	0	0	0	0	3	0	0	0	0
72de20805be244d9afe4519f3b1f3777	50	0	0	0	0	0	0	0	0	0	0	0
3458d3696d6a1c40091f821752a64ed1	0	0	0	0	5	0	0	0	0	0	0	0
04d09e2e543c046a76fd588ebf79d98b	45	0	0	0	0	0	0	0	0	0	0	0
a62ef37f802e2da1eb98258db3890116	0	21	0	0	0	0	0	0	0	0	0	0
8f29735e5fcdf43540efa370a33bd87a	0	10	0	0	0	0	0	0	0	0	0	0
1eef6a3e8eeebb6babe1a811698a725b	0	0	0	0	0	0	0	0	0	0	199	0
e659b05da361456b6cf8b32354f21e5c	0	0	5	0	0	0	0	0	0	0	0	0
797034255a1a510b911af0d229c96c33	0	0	0	0	0	0	0	0	3	0	0	0
fd64d58aec0a368e73cb832c57184ee1	0	0	0	0	0	0	35	0	0	0	0	0
e6c35c2ef674f5db7eacd969fc830ac6	14	0	0	0	0	0	0	0	0	0	0	0

317610ab2f87228e7d3f48c461fa7d30	0	0	0	3	0	0	0	0	0	0	0	0
d47d2e2e294fca83b0890a100758cfa7	0	0	0	0	0	0	0	0	0	2	0	0
aa26f4e4d98e72520be794077efba48d	0	0	0	0	4	0	0	0	0	0	0	0
838d6a47e1359d896c4da0016dd0fcf5	0	0	0	0	0	0	0	0	0	0	0	3
d1de2b82545d97e8318ae4a63cb2e460	0	0	0	0	0	0	0	4	0	0	0	0
ce2c5e1353b4d09d30d10435008a5e41	0	0	0	0	0	0	0	0	0	0	0	4
d295c83ce0b8940272571a684bf1a9c7	0	0	2	0	0	0	0	0	0	0	0	0
d1cf02880b78313c76e84ac171293098	0	0	0	0	0	0	0	0	0	0	12	0
aecce7fa1948c87adcf06afbd6885fd3	0	0	0	0	26	0	0	0	0	0	0	0
7aafd931d8afa6ca39cc8a7d242dd317	0	6	0	0	0	0	0	0	0	0	0	0
763410331293d8c24bbc9390d3b4822d	0	0	57	0	0	0	0	0	0	0	0	0
a04c31f4ad5086c78aacf9acb1194d52	0	0	0	0	0	3	0	0	0	0	0	0
877cd8044585d426ae673292f6eabf5a	0	0	0	0	0	2	0	0	0	0	0	0
b712864e2d7c7bc46653d74438daf168	0	0	0	0	0	4	0	0	0	0	0	0

d30dc93f1b9fc127493bcbd5a2fbf52f	0	0	0	4	0	0	0	0	0	0	0	0
bde223a5928419329b2e883ee8585578	0	0	0	0	0	102	0	0	0	0	0	0
d2c3f8c7b1e492c2fd7ca2be609bb0ea	2	0	0	0	0	0	0	0	0	0	0	0
78812afc4b1168095d109fbecfd53be0	0	0	0	0	0	0	0	0	0	0	19	0
4a8a3ba9f9953cfa4fae7a14d93fac58	0	0	0	0	0	0	0	0	0	0	0	143
cdbbc8843ecebae4785267a8001c4d6f	0	0	0	0	0	0	0	0	0	54	0	0
c9d06b41c9b47b074dc61571544cb25c	0	0	0	0	0	0	14	0	0	0	0	0
b5e55fd2af0d9497828963f740b6bea3	56	0	0	0	0	0	0	0	0	0	0	0
564e853cb72fceba1d4f3ccb5871953a	3	0	0	0	0	0	0	0	0	0	0	0
4df170cfd75ac51e7de4cdc36ddadc49	0	0	0	0	41	0	0	0	0	0	0	0
7af99b31150c21d558fa22b89339ba41	0	0	0	0	0	0	0	0	0	74	0	0
e6352cc67c74543fc22b450e95cde418	0	0	0	0	0	0	0	0	0	0	0	2
fb954073e6671e578e9cabec0ffd9780	0	5	0	0	0	0	0	0	0	0	0	0
04e0668d5b8e6b722ead604d70ddd6d5	0	0	0	0	0	0	0	5	0	0	0	0

fd6df4df3eb10cf3e52eb25d48934108	0	0	5	0	0	0	0	0	0	0	0	0
46e24e8b247862dc6f05941d93cbadd9	0	0	0	0	56	0	0	0	0	0	0	0
ba7a346216330394a50d5ada65cc5550	0	0	0	0	0	0	4	0	0	0	0	0
cff32d2b9daa0776c614916e5f63443a	0	0	0	0	0	0	0	0	11	0	0	0
aa1f086035381302a4b42b137ef52060	0	0	0	0	0	0	0	0	0	17	0	0
f4d51bd8ada13ed786704807fa69fb8d	0	0	0	0	0	0	0	0	0	0	2	0
423f054bbb07910c49a367db368d3b17	0	0	0	0	25	0	0	0	0	0	0	0
548c4d9163617245ade6d1853d3ba375	0	0	0	6	0	0	0	0	0	0	0	0
eeac74a1bf9a533e1d989ab39fe0af74	0	0	0	0	88	0	0	0	0	0	0	0
9d1c50838057a4e0e5e23e1e625436c7	3	0	0	0	0	0	0	0	0	0	0	0
d97e14d769efd609f45d0932e9096499	0	0	0	0	0	0	0	0	4	0	0	0
fab7a619f3e3472631b1d8ef4cc3a9ea	0	0	0	0	0	0	0	0	0	0	2	0
52e5e421d23fa11b7b1c86cf5b80c18b	0	0	0	0	0	0	0	0	0	0	0	39
70ab1f5dc1badf03cb7e314d3927e2af	0	0	0	0	0	0	0	3	0	0	0	0

6145ee3f14f2ac32ef7dd1c28866aa8f	0	0	0	0	0	0	0	0	96	0	0	0
2eec542332bfa25e433d61f98e18f5c0	0	0	0	0	49	0	0	0	0	115	0	0
30b968481d73cf502068d1805601b078	0	0	29	0	0	0	0	0	0	0	0	0
9c4d8326247f0b59a985cc614790c72b	0	0	0	0	0	0	0	0	0	0	6	0
a8fd6e7ff7f6d4fd5bebf153bbdccc94	0	0	0	0	0	0	5	0	0	0	0	0
b297d004cdea5b63f67ab9abb252eabf	0	0	0	0	0	0	124	0	0	0	0	0
a0dba2633a06671b1d38632c23e57914	0	0	3	0	0	0	0	0	0	0	0	0
5157c97df0c36d5092f8e1836013ffea	0	0	0	0	0	0	0	0	0	0	13	0
03957015b23c9818f94a48071ac66ea9	0	0	0	0	0	0	0	0	0	0	2	0
8de4008e866335d5d5fb37351f57069d	0	0	0	0	0	0	0	0	0	3	0	0
8da57014fd933bc8f3a5df74f8e1c815	0	16	0	0	0	0	0	0	0	0	0	0
3d7625ea671ab13c9f0139f34502d831	0	0	0	0	0	0	0	7	0	0	0	0
5fda3a464d869d119f8d22b8239f075d	0	0	0	0	0	0	0	2	0	0	0	0
4e95e0e35ae28722e369b810dc6e4dca	0	0	0	0	0	0	0	0	0	23	0	0

eed4f275edef2136c3dea65a34e21de6	0	0	0	0	0	0	0	0	0	2	0	0
4d0b0b0e442b8b59def41f4ae6fd9e37	0	0	0	0	0	0	0	0	0	0	2	0
957feb8f8a13e12a354823aa01b8dc45	0	0	6	0	0	0	0	0	0	0	0	0
637db2ccffa5f6b7ecf066acf47203fe	0	0	4	0	0	0	0	0	0	0	0	0
cd15c0192f47a584ba8f86661287dd8e	0	0	0	0	0	0	0	0	0	0	3	0
b46a291e240aff5586e20ad203ac0871	0	0	0	0	0	15	0	0	0	0	0	0
38394c4272fc7d73430f454797cedfe2	0	0	0	0	0	0	0	0	49	0	0	0
8cb1802757ea884d816fabdbbc76ff92	0	0	0	0	0	0	0	0	0	0	10	0
b8131cecf73cba55564987cd24c608ad	0	0	8	0	0	0	0	0	0	0	0	0
39af91a6092992c04a998d5d8961b0e6	0	0	0	0	0	216	0	0	0	0	0	0
347ce2b469f3ff7b6b802afd8e22210a	0	0	0	0	0	0	0	0	14	0	0	0
c909a9221fc09e38ecc4a054f2e71bbf	0	0	0	0	0	0	0	0	0	0	161	0
f8c926048087324f2344375570565a57	0	0	0	0	0	2	0	0	0	0	0	0
8e38db17b31c67d258ae4708070a981d	0	0	0	0	0	0	0	0	0	0	3	0

fc4d21bb273aa1d5aa29c0fa3c293aad	0	0	5	0	0	0	0	0	0	0	0	0
fcd09bb265c3e7a692e4e115057101ee	0	0	0	0	0	0	0	0	0	9	0	0
99f8ab8b83956a818dafb663f307d462	0	0	0	0	0	0	0	0	20	0	0	0
d9a4953b928c90f19e6a1f10c9e2901a	19	0	0	0	0	0	0	0	0	0	0	0
c060f3a2ceda85b3e8ed11a0104e2387	0	6	0	0	0	0	0	0	0	0	0	0
9432c316a2abe8b1faef8daa7e7550b0	0	0	3	0	0	0	0	0	0	0	0	0
31567d3e828d8286d5d7f1b19b56ff82	0	0	0	0	0	0	0	0	2	0	0	0
2bc45beb5db955a8df4889c32708a2f8	0	0	0	14	0	0	0	0	0	0	0	0
446f53c4434ed9c253f24ee1d5b4a8ba	0	0	0	0	0	0	0	0	0	0	0	4
6041c189f036fabf3e79e0540f320af4	0	0	0	0	0	0	0	0	0	0	5	0
1865c0d8bc07f72ccc6a2fe0ac6c710a	0	0	0	0	0	0	5	0	0	0	0	0
c5ea1739e06aa67607aca3487e3dd882	0	0	0	0	0	0	0	0	0	0	7	0
94861329e65ff835eaf9f6e51d72c3cb	0	0	0	0	0	10	0	0	0	0	0	0
85acd770880d2fc078fb4002920a979b	0	0	0	0	0	0	0	0	0	9	0	0

050e5006419b506367e30ae97b8bcadb	0	0	0	0	0	0	0	0	3	0	0	0
3c3f147ee27bca2821ebdc45485786bd	0	0	0	0	0	0	2	0	0	0	0	0
7bf18017838a223389b674bde1e7feba	0	0	0	0	0	0	5	0	0	0	0	0
e00c75a406b5809dee35104c677e7fbf	0	0	4	0	0	0	0	0	0	0	0	0
38b9c7908a668505aae1c8717593ba02	0	0	0	0	0	0	4	0	0	0	0	0
f3b55fd2dc98aa3d12f2e9c05e8c9629	0	0	0	0	0	0	0	0	0	4	0	0
bccfc7d87398348774aed3377edb1fc1	0	0	0	0	0	0	0	0	0	0	0	5
7a84d7db73f989ffa7a81bd60f6bcaa6	0	0	0	0	0	0	0	0	0	98	0	0
21613daf915679051f288e47f477e6c7	0	0	0	0	0	0	0	0	0	0	21	0
567cd06b1eb349325ec3e67eb41da843	7	0	0	0	0	0	0	0	0	0	0	0
d5e58884bb372f2ba5d3a5b337af4735	2	0	0	0	0	0	0	0	0	0	0	0
2e8d7ecd2b5f1be3efe9b3a54294255f	0	0	0	13	0	0	0	0	0	0	0	0
d69285560befe0c8ac330c5de36e6847	0	14	0	0	0	0	0	0	0	0	0	0
61f092e08f401d30df79ac44556d4158	6	0	0	0	0	0	0	0	0	0	0	0

1c3ade50c578cfe0c84ef4915 5039d9f	32	0	0	0	0	0	0	0	0	0	0	0
337d0fbeedd10960b1a7427 5351adc62	0	0	0	0	0	0	5	0	0	0	0	0
bd09169c2047f62ec86a1a01 ee0c923f	0	0	0	0	0	6	0	0	0	0	0	0
967d06c4fa0cd2018ba48e48 521a3a9b	0	0	0	0	0	0	0	0	0	13	0	0
b385fc5b3924271d7751a648 b47b72ba	0	0	0	0	0	0	0	0	0	0	0	2
1bbe527cf2584d17cb295638 491e8af8	0	0	0	0	0	0	0	0	2	0	0	0
e0791c069974d6444809a1a e0e80f056	0	0	0	0	0	0	0	0	0	0	3	0
7b39dfe4a59a02ea0ec9506c 89ae8f72	0	0	0	0	0	0	0	0	5	0	0	0
2037454216c6dd0fb05ec0a8 a18ad772	0	0	0	0	33	0	0	0	0	0	0	0
a7d441c12cef366d16723f2a 152d0886	0	0	0	0	0	0	0	0	0	3	0	0
1d77e80d27ba8609a66f100e feec7213	0	0	0	0	0	0	0	21	0	0	0	0
b4f4096c4c98d58feb06d119 1dd86d55	0	0	0	4	0	0	0	0	0	0	0	0
4b83457696cc7ee32967a8e8 a001d797	0	0	0	0	0	0	0	2	0	0	0	0
4ada4e483cf1995b86f60fb65 15cbd45	0	13	0	0	0	0	0	0	0	0	0	0

eae23b50c08d67ea88a956b e6760e647	0	0	0	0	0	0	0	0	0	0	6	0
b353d79ba1fdae9708a8b699 58846990	0	0	0	0	0	0	0	0	0	0	0	36
ce575669e2cee95ba1cb7a04 ae748de2	0	0	0	0	0	0	0	4	0	0	0	0
55e7f6460133f960261f205c2 9f08b7e	0	0	0	0	0	0	0	0	5	0	0	0
031cb8c4310b62f69d35a63b 62e92bd6	0	0	0	0	0	0	4	0	0	0	0	0
3241c69c47d1b52e91b741b 516023e1c	0	0	0	0	0	0	0	0	0	3	0	0
97dea36e3e8c5704a299231 b9b93b865	0	0	0	0	0	0	0	0	0	0	8	0
436a9edcaf52fe208533f9a5b 15442c6	0	0	0	0	0	0	0	0	0	12	0	0
0d4d78e58a75d2c7c1b47f01 93a6ad88	0	0	0	0	0	0	0	0	5	0	0	0
ccb25ce50193204bab1031c5 a8b67cfb	0	0	0	0	0	0	0	0	0	4	0	0
5c96c8decb38a3b4bc01fd18 42fbda64	0	0	0	0	0	0	0	0	3	0	0	0
b74ee6ba7e0d0ded57071f1f 6ed17dd4	0	0	0	0	0	3	0	0	0	0	0	0
7dff3cb782452a546ace4a98 0dc92113	0	0	0	0	0	0	0	0	0	10	0	0
7f4cb70f62e7e1311699fef29 7389202	0	0	0	0	0	0	0	0	0	0	9	0

e315935ee59c549ab532576 8bbec552f	0	0	0	0	0	0	2	0	0	0	0	0
1a9c9ee9267356d83d2ea06 9537b61ef	0	0	4	0	0	0	0	0	0	0	0	0
a9b99e8b6db0b4844b05ad6 67324ab04	0	0	0	0	0	0	0	0	0	0	9	0
c7c4f99e1aa166ff4a3e30c10 6a90a2f	2	0	0	0	0	0	0	0	0	0	0	0
60d51290178f7293da140f12 584861eb	0	0	0	0	0	0	0	0	0	8	0	0
e884bd08c61730bbd402d56 4459d034e	34	0	0	0	0	0	0	0	0	0	0	0
e94bc0b44ed2b59295db6a5 e92ae55fa	0	0	0	0	0	0	0	0	0	0	2	0
28714ce79be93b5ea11a7b5 48da25286	0	0	0	0	0	2	0	0	0	0	0	0
f6fcdce71e9a82af4e85b6b25 173cdfa	0	0	0	0	0	0	0	0	0	0	0	24
d219f645c932adc68c59fc1f7 c874915	0	6	0	0	0	0	0	0	0	0	0	0
38ff7461a8c04e9de1995c3fc a5283e1	10	0	0	0	0	0	0	0	0	0	0	0
a8bdfc4d379bc16906883c29 1a5cbdef	0	0	0	0	0	0	0	0	0	2	0	0
26405d469d47b63de555c4d ce327c4fd	0	0	0	0	0	0	0	20	0	0	0	0
419d2339eedd1fd8547e16f5 992886e4	0	0	0	9	0	0	0	0	0	0	0	0

f934c30934cd878c70e6b0d18802450c	0	0	0	3	0	0	0	0	0	0	0	0
fd9e012bf091776f21455812e585d266	0	0	0	0	0	3	0	0	0	0	0	0
b110a4e65d6423f3b91d22ecf964dc49	0	0	0	0	0	0	0	0	0	0	17	0
f7c012f9dbe2b81be50ffa42dfff03cd	0	0	0	0	0	0	3	0	0	0	0	0
1c1f85dcf775b47a4da4b65bccefac74	0	0	0	0	0	0	0	0	13	0	0	0
3e0950aabd27ebe31e4da0fc379801f7	22	0	0	0	0	0	0	0	0	0	0	0
07253ea8aedbec8b3a38bb3f2a04fca5	0	0	0	0	0	0	0	12	0	0	0	0
9d1dcaf272348fc493ccedc25179c667	0	3	0	0	0	0	0	0	0	0	0	0
e530afb4fe6e6c5d0574865d06e35c14	0	0	0	0	0	0	0	0	0	2	0	0
4d5e6cfc7b4fbe5b5590a41775ebc1c0	0	0	0	0	0	0	0	0	0	8	0	0
9bfffce89e74911437b71a85024018bd	0	0	0	25	0	0	0	0	0	0	0	0
7c47ebfe2df011abf2bd13e572c35045	0	0	0	0	0	0	0	0	0	0	0	3
616e4f0ee6ca90f86321cccdcd084a0b	2	0	0	0	0	0	0	0	0	0	0	0
a8c24accccafc2200a160d8783f622b0	0	0	0	0	0	0	6	0	0	0	0	0

ccfc0e5443d7c8cb5cede6aac529eaa2	0	0	0	25	0	0	0	0	0	0	0	0
ae04af7d3da8950d18625ae620638d0c	0	0	0	0	0	12	0	0	0	0	0	0
f5c19a020a63ac5fa446692975c07232	0	0	0	0	4	0	0	0	0	0	0	0
aa56f4fcc36bb56599cca6b8aef3228e	0	0	0	0	0	0	0	0	0	0	100	0
ccaa90d0083947ce6a8552d7e8062b73	0	0	4	0	0	0	0	0	0	0	0	0
432cc87ec11b31f5caa7facae8ece919	0	0	7	0	0	0	0	0	0	0	0	0
555a337e28a0b5d33181102cd021ae1f	0	2	0	0	0	0	0	0	0	0	0	0
2e30e66d8fff284af134ba0a55fa3df2	0	0	158	0	0	0	0	0	0	0	0	0
7597bb2b77c4347e8a60b856bcfc0186	3	0	0	0	0	0	0	0	0	0	0	0
d79b43ba7f2d9bbf8e117e17a86e2b42	0	0	0	0	0	0	0	0	6	0	0	0
9fa2b266a743c1d7ad70a42b2c6e53c2	0	0	0	0	0	0	0	0	0	0	0	3
c5283c3ff106cc78f445a22f4c58d3f4	0	0	0	0	0	0	0	0	0	4	0	0
50590ad968d9e5908e52ac19f94beacd	0	15	0	0	0	0	0	0	0	0	0	0
17b091ac6cace5f69c53cb4929330931	0	0	0	0	0	5	0	0	0	0	0	0

c8986a6dced2acd4aea7c866e138c159	0	0	0	0	0	0	0	0	0	0	0	3
7b48d4c545fa48e5c94b7e1fb6b379f6	0	0	0	0	0	0	85	0	0	0	0	0
47bb581a45802c1abad2059e146e0843	0	0	0	0	0	0	0	0	0	0	36	0
be5f7d3187aec7cd0682e452d21f8637	0	0	0	0	0	0	0	21	0	0	0	0
78e93121475df9daab024cbfa50a1669	0	0	0	0	0	0	0	0	5	0	0	0
1d011b1dbb1b22e3ebc3ceba13da854c	0	0	0	0	0	0	0	0	0	0	3	0
d9464eedcce45a6663a3f56ffba6864d	0	6	0	0	0	0	0	0	0	0	0	0
ee15a3c49e336c343cea87c07f1982dc	0	0	0	0	0	0	0	0	0	0	11	0
1109267496d9dc5dc753cc6c7961d863	0	0	0	0	0	0	0	0	22	0	0	0
001f455db0545ba175729198320cb31c	0	0	0	0	2	0	0	0	0	0	0	0
2241616780956706944 5de796af8963f	0	0	0	4	0	0	0	0	0	0	0	0
e5928006ff69f9549b95820336472fc1	0	0	0	0	0	0	0	0	0	57	0	0
b3e1988478141c51d07de86ba98c4f45	0	0	0	0	0	0	0	2	0	0	0	0
dc29116b1445fa12b2c815a4ffa9647e	0	0	0	0	0	0	0	0	121	0	0	0

7fb988c85e545e8dc3fdea90 5dba55f0	0	0	0	6	0	0	0	0	0	0	0	0
f2136d8bc4b317083d010483 d1ab04c6	0	0	0	0	0	0	0	0	0	0	4	0
90ca707bb17fb4fd4987dbb0 9a6290f3	0	0	28	0	0	0	0	0	0	0	0	0
87bd1b662b499204eb9fa8c1 1e704e3e	0	5	0	0	0	0	0	0	0	0	0	0
823333cc7c7079e8c246330e f0408d39	0	0	0	0	0	3	0	0	0	0	0	0
f844358f2f133f2631f2bf7d7 436f897	0	0	0	0	0	0	0	0	0	0	5	0
66abd9480094f383a32ea29b 389e6f1e	0	0	0	0	0	0	0	0	0	4	0	0
b65d794f0c0d3452c95fac97 d4d2467c	0	0	0	0	0	0	0	0	0	4	0	0
a1e2c0e160b3a776afc1a7d9 b5fbcf98	0	0	0	2	0	0	0	0	0	0	0	0
c95b2943733ca4259b6f4ecef 0cf8d16	0	0	0	0	0	0	0	0	0	0	0	3
23df16c7f4758234f32bda29 2822997c	0	0	0	6	0	0	0	0	0	0	0	0
5164a3992fddb35568708739 8e1b9f9f	0	29	0	0	0	0	0	0	0	0	0	0
cf916378847067d8feab1650 cf6c2035	0	0	0	0	0	3	0	0	0	0	0	0
2930447ecec4d3b7380ca0c0 c7953074	0	0	0	14	0	0	0	0	0	0	0	0

6543ef71c6aefc95235c2b0e4d4c75f3	0	0	0	0	0	0	0	4	0	0	0	0
fd5cc9ebad476e390d35f0a6d2c2b89b	0	0	0	0	0	0	69	0	0	0	0	0
61318baa5d8242b10f742460489b396a	0	0	2	0	0	0	0	0	0	0	0	0
7ec1362fa70263253b262e6a8351e664	0	0	0	0	0	0	0	0	0	0	12	0
bfc7f943e323f0ca3f5b33662d00153b	0	0	0	0	0	0	3	0	0	0	0	0
8c99ba93bd44b830891343775f3a39b5	0	0	0	0	0	0	0	0	0	0	2	0
44387393af70bf9c22972ca8d9ac981b	0	4	0	0	0	0	0	0	0	0	0	0
ff6eeec1cdb8bf4e6173ba31722b66cb	0	0	7	0	0	0	0	0	0	0	0	0
89697f65b56325c95513b6ba5ea86451	0	0	0	0	0	2	0	0	0	0	0	0
0ca7a9e926ce70c2a6f327ff7d7b808f	0	0	0	0	0	0	3	0	0	0	0	0
fa02daa1ed09d49f9c662526d85f75d2	0	6	0	0	0	0	0	0	0	0	0	0
dfb12ffdffeb53ab5a046fb5c799297b	3	0	0	0	0	0	0	0	0	0	0	0
3908724747f60ce0434b843cf2453d6f	0	0	0	0	0	0	0	0	0	0	0	5
2095c585ffbcc666b90a9d021cc8deae	3	0	0	0	0	0	0	0	0	0	0	0

f1a77f4a554686b337181d36 46382d62	0	0	0	0	0	0	0	0	0	0	0	7
1c09407f2b666e6d1a94284f d9ce07c2	0	0	0	0	0	0	0	0	0	0	5	0
ca04a1fd42347f9b5bfb9f4d3 a14d3ef	0	0	0	0	0	0	0	0	0	0	0	29
87f6a6ba5109a5f34f5137e8 bd4def74	0	0	0	5	0	0	0	0	0	0	0	0
fafb2b1579447ef20145df340 550917d	0	0	0	0	64	0	0	0	0	0	0	0
a1a1458eba0d3cb481c9d6fc 9a30877c	0	0	0	0	28	0	0	0	0	0	0	0
7d8e2b6163cb609acaea47f8 dc41852f	0	0	0	2	0	0	0	0	0	0	0	0
1f66a27622fda5c8d9b55066 e34b1dff	0	0	0	0	0	0	0	0	0	0	6	0
6add7deb3bf3f068597f8c1d e74f5b63	0	0	45	0	0	0	0	0	0	0	0	0
680df9eccbc58a4b59464ce7 160926c2	0	0	0	0	0	0	0	0	0	0	2	0
a6562d4dd466592061527b2 dc650f6b3	0	0	3	0	0	0	0	0	0	0	0	0
8db99727d53346722eb264b d885a0eb0	0	0	5	0	0	0	0	0	0	0	0	0
b20d5f09e012b68e67a8c02d 0f4cceab	0	0	0	0	0	0	0	3	0	0	0	0
358762f6bcb616d32955c087 9ffe6bc8	0	0	0	0	0	0	6	0	0	0	0	0

31829db8d0de714dd6f1e64 29b90972e	0	0	0	0	88	0	0	0	0	0	0	0
2ee01b866aa9742cf7254502 dee0293b	32	0	0	0	0	0	0	0	0	0	0	0
894cc087dc33f2f75115a72a3 6af26bd	0	0	0	0	0	3	0	0	0	0	0	0
370abe3d83a03eb3c053c0be cc3e02cf	0	0	53	0	0	0	0	0	0	0	0	0
4e3d9e0a2ed6ccbbe821dbb ef3905f87	0	0	0	0	0	0	0	3	0	0	0	0
3938f32b41a4c4c3a52e5e0c a695b8b0	0	0	0	0	2	0	0	0	0	0	0	0
7d6e534e76a1556696d45ee e42f67ec1	0	0	28	0	0	0	0	0	0	0	0	0
e788afa84ed47a00b17f9580 9a8a0009	0	0	0	0	0	0	0	0	0	34	0	0
7e6d783c87a905b8ffeb9e30 10f63351	0	0	0	0	0	136	0	0	0	112	13	0
ff0350372f52ae8f12069d722 274348c	0	0	0	0	0	4	0	0	0	0	0	0
2277f7a0d55c6bdfe50dac76 e5e8d9fb	0	0	0	0	0	0	0	0	0	0	4	0
032f173348c943ad477a0acd bcf543b3	8	0	0	0	0	0	0	0	0	0	0	0
50e1fa593bf5ecd3dc01a68a 4b9b029f	0	0	0	0	0	0	0	0	2	0	0	0
a91e286c5b47ea20664b4f04 770704a3	0	0	0	0	0	0	0	0	0	6	0	0

a5f42be0d980f319f2653d0239c056d0	2	0	0	0	0	0	0	0	0	0	0	0
d17c2913dac2d3ec024b40b9e2df943f	0	0	0	0	10	0	0	0	0	0	0	0
edf7fdaba41a2a781e636f89935f6b8e	0	0	0	0	0	0	0	0	0	0	5	0
caa678a4600f3dc6f3225e4bcd76d3a7	0	2	0	0	0	0	0	0	0	0	0	0
c06772ff892ef18f0e401f2b361a9467	0	0	0	0	0	0	0	0	0	0	84	0
c5e5c73b5724aefffb92b3472b0bee79	0	14	0	0	0	0	0	0	0	0	0	0
97a24a3c0092a8ff3888164a84e846d1	0	0	0	0	0	0	0	0	10	0	0	0
525cddcf4b38d8c4b5fc18159f43944f	0	0	0	0	0	0	0	0	0	48	0	0
46f50a08434264c7df57e85c45c755f9	0	0	0	0	0	0	0	0	6	0	0	0
932cb2f1f0de4e6be0e6d8e50262c035	0	0	2	0	0	0	0	0	0	0	0	0
66b1f073252d3ddcfd01d40820afd282	0	0	0	0	0	0	0	0	0	0	0	21
1fe92d75ac7fbc6771d85f5544432a34	0	0	0	0	0	30	0	0	0	0	0	0
78617c2fe785f243327ac15406832160	0	0	0	0	0	0	0	0	0	0	8	0
816ca73372d3796e9696709f389da141	0	0	0	0	0	9	0	0	0	0	0	0

42349f5c6b6963767fd7854d74a00c03	0	0	7	0	0	0	0	0	0	0	0	0
8d6c5e42596364bba6fd50bc34e97137	0	0	6	0	0	0	0	0	0	0	0	0
a231df8147e23b1c7969549accb620b3	0	0	0	0	0	0	9	0	0	0	0	0
843bf302128f2145ad21bafbe150898a	0	0	0	0	0	0	4	0	0	0	0	0
03fdebe5f10d35d5fb2f9d22ba782fdc	0	0	0	0	0	0	0	0	16	0	0	0
56b388b7093e7c048a17eb91c5dcd30a	4	0	0	0	0	0	0	0	0	0	0	0
44ce7f66b0151e66047ea9db523ea0e4	0	5	0	0	0	0	0	0	0	0	0	0
8998531281d0194c8720f93f724b3ca2	0	0	0	0	0	0	0	0	77	0	262	0
b8ad0aa9d5f065a461c0d3614c2692b4	0	0	0	0	0	0	0	4	0	0	0	0
61ebe9502e08ddc4de2c2e1fb58bc198	0	0	0	0	8	0	0	0	0	0	0	0
f8999cd2812730377b627a4f7cd49cf1	0	0	0	0	0	0	0	34	0	0	0	0
116680350132550e26072025fa23d1f9	0	0	0	0	0	0	0	7	0	0	0	0
4495e8da91704b762da90ae0c6d3def9	13	0	0	0	0	0	0	0	0	0	0	0
6e897335a8cb1ea647ce035a64a52740	0	0	0	2	0	0	0	0	0	0	0	0

add92469373921643e6ecbeb662555f0	0	0	0	0	0	0	0	0	0	4	0	0
a90c7bbc561657333cf649bd154e8ec1	0	0	0	0	0	0	0	0	0	0	15	0
d236aa2eb1a2fb8439e33ce177703eb5	0	0	0	0	0	0	0	0	0	0	46	0
4ed9a33078dfd25442a260bfecd7ad9d	0	0	0	0	0	0	0	2	0	0	0	0
914d158d287dedc90b71bfa239624b01	0	0	0	0	4	0	0	0	0	0	0	0
d61aa2688f643d1b810424e8da081cb3	18	0	0	0	0	0	0	0	0	0	0	0
09dbd0daab2394a72f2763f271594237	0	0	0	0	0	0	11	0	0	0	0	0
887a4a85ab333ea6ae1588cd3e2c25f7	0	0	0	0	0	0	0	0	0	7	0	0
a49457d2fed95f43c637f02cac02e77f	0	0	0	0	0	0	0	0	4	0	0	0
84f06564bf2ed628fdf8827563f94cc1	0	0	0	0	0	0	0	0	0	0	16	0
d96435ded1057f54214a72f75e90aef9	0	0	0	0	0	0	0	0	0	0	5	0
ae7d9bb7f963c0a44bb25d114bc0d016	0	0	0	0	0	2	0	0	0	0	0	0
537fd7bf883eb6aa7db9ec50a73a8294	0	0	0	0	0	0	0	0	0	0	0	15
d516acb2fb86bd496c9eca1d3899f2aa	2	0	0	0	0	0	0	0	0	0	0	0

c7ec9fcce2a71d9abebb67b0ee019109	0	0	0	0	0	0	5	0	0	0	0	0
e423e3c0089d69546079893c090a53c8	0	0	0	0	0	0	3	0	0	0	0	0
6793e52e357cd62b80e3609ac671f7d6	0	0	0	0	0	3	0	0	0	0	0	0
7c5c3d5ae3c6129bd5c2fbdc58e35bfc	0	0	0	0	0	0	0	0	0	0	0	43
33a3f932f0359fe689b236eba105ca85	0	0	0	0	0	0	3	0	0	0	0	0
7b88a82efaca9a20e7badc7570ebc83c	0	0	4	0	0	0	0	0	0	0	0	0
514cb80ffbb862f45f13b01cc9ab4a1f	0	0	0	0	0	0	0	0	0	0	5	0
cfec87237ebcee265fb89a7323dd32ea	0	0	0	0	0	0	0	0	5	0	0	0
f34fdbb47295b053ce889303fb128128	0	0	0	0	0	0	0	0	0	0	4	0
2ceb854e75b71ae9297b50a7c2ff1fe7	0	0	0	7	0	0	0	0	0	0	0	0
b3cdfe02a4d7eb3a39f78ca3d6fd8413	0	0	0	0	0	0	0	23	0	0	0	0
39c45dcfc9ac00e0c757d69f7bddd2d8	0	0	0	0	0	0	2	0	0	0	0	0
ca2345ca1266b044256109d4b80e9c23	0	0	0	0	0	0	0	3	0	0	0	0
f03e44e68a1f3841ea6ac6f0b463da03	0	0	0	0	0	0	0	0	0	0	2	0

cfdeb05485f63d1bb05ed025e0a41c7f	0	0	0	0	0	0	0	4	0	0	0	0
af0e9e262e04b0893c3fe850af13aff1	0	0	0	6	0	0	0	0	0	0	0	0
2813d4c6d86e15c6868794223e8dbc63	0	0	21	0	0	0	0	0	0	0	0	0
c80a61754068aa1b04a910394b5fe3a8	0	0	0	0	0	0	0	0	0	2	0	0
983f14f7b48b201862c33ce0eca9d649	0	0	0	0	0	0	0	0	0	16	0	0
f56dc51595f819a41860de76b241e8eb	0	0	0	0	0	0	0	0	4	0	0	0
919bcd8ced1c42b9ea901fb54d2dc8dc	0	0	2	0	0	0	0	0	0	0	0	0
dc03bbf19061cef6e9e9e9d5889701cd	0	0	0	0	0	0	0	0	0	0	0	6
b56d7852f07e8f858f917ba93f11bdbb	0	0	0	0	0	0	0	0	0	0	3	0
1a863396683187987e10f17e5722d14e	0	0	0	0	0	0	0	0	0	136	0	0
df650682cff03cfd34efeeb515e39b88	0	0	0	0	0	3	0	0	0	0	0	0
3f71655c883b48d965af0138b2cb576a	0	0	0	0	0	17	0	0	0	0	0	0
d4817ca5d441ea42c505a5af831f04f7	0	0	0	0	3	0	0	0	0	0	0	0
5859b04e0f7c5bd66f170a03f6166007	0	0	0	0	0	0	0	0	0	0	0	2

24c28359ad134cb26c644c53 4423cb72	0	0	0	0	0	0	0	0	0	0	8	0
fe70e0dd45790ce0991aa56d da16d4fc	0	0	0	0	0	3	0	0	0	0	0	0
01ee4deb075b7f25f26ef2b2 08ee5c2b	0	0	0	0	0	0	0	4	0	0	0	0
fb7ad300dfc41a7b6e8eb210 9f4f88a8	0	0	0	20	0	0	0	0	0	0	0	0
2f99fd9f63fcedabfc5c702b90 d758ce	0	0	0	0	0	0	0	0	0	0	2	0
0121248d61e073233c2d32e 9a8f6c489	0	0	7	0	0	0	0	0	0	0	0	0
10edd855b0bbb8721051cef4 790c69a4	4	0	0	0	0	0	0	0	0	0	0	0
775600142f107194c752b3d2 7dcb794b	3	0	0	0	0	0	0	0	0	0	0	0
1e1b65d406e9b6c26de100b b54e6a4f7	0	0	0	0	0	0	0	0	0	0	12	0
6d42388291aac64efeaf5170 aef87dd1	0	0	0	20	0	0	0	0	0	0	0	0
73c9cc704d2754b79a54e051 22fbcd29	0	0	0	0	0	0	0	0	0	3	0	0
43a8664fc38238496ff61ec6e 6e04114	0	0	7	0	0	0	0	0	0	0	0	0
7ceb5ef7b65fe701051db3e6 e1d9b37c	0	0	0	0	0	0	0	0	0	2	0	0
e72c4c8590948c3d219da997 6035e3a8	0	0	0	0	0	0	0	0	0	0	12	0

329991a934fc8781e6586038 02b9fb2b	0	0	0	0	0	0	3	0	0	0	0	0
8b1ea4ad4f25aab4b5bf1c2a 9e6ee00e	0	0	0	0	0	0	0	0	0	3	0	0
3f28bd82e940746636493e38 c4f16e60	0	0	13	0	0	0	0	0	0	0	0	0
7de79718d7fbdee6b90a1c1b c957548c	0	0	0	0	0	0	0	0	4	0	0	0
bbed43b41bcdc8f6ad83b854 9401c2ce	0	2	0	0	0	0	0	0	0	0	0	0
872116c1f99013aae89fc1f5d 197eccb	0	0	0	0	5	0	0	0	0	0	0	0
c937dd758881f4270f793913 9b252bb8	0	0	0	17	0	0	0	0	0	0	0	0
6ab3e85e60a869ca0cb39790 46473feb	0	0	0	0	8	0	0	0	0	0	0	0
c8514c0aa2fca9ed6bff8b6e7 97745e7	0	0	0	4	0	0	0	0	0	0	0	0
d80e853125bc32dd5e1a2c4 0b50234f5	0	0	0	0	5	0	0	0	0	0	0	0
df06d75c0206ace306e92ac9 994516a6	0	0	0	0	0	0	0	0	0	0	0	5
030350b226e31836688ed31 e274aca1e	0	2	0	0	0	0	0	0	0	0	0	0
b7e9b884dd8be897efdd653 dfce11454	0	0	0	0	0	0	0	8	0	0	0	0
1d91f801bffe6c9eb700653c4 32a3f7c	0	3	0	0	0	0	0	0	0	0	0	0

6af590617691fdb3d20c27de9345aa5c	0	0	0	0	0	0	71	0	86	0	0	0
3b1033148aefc69ff5255b4f24affd25	0	0	0	2	0	0	0	0	0	0	0	0
67e42466c8e1ce1d305dbd658d332982	0	0	0	0	0	0	0	0	0	18	0	0
700ed7b395c72f4aa226456ed6e7492e	0	0	0	0	0	0	0	0	5	0	0	0
c6d4f1a238b1d80703372d23ba8cc07a	0	0	0	0	0	0	111	0	0	0	0	0
4488d60d0d11f32849b68a1a74bcc15c	2	0	0	0	0	0	0	0	0	0	0	0
3dc4018db29c35cb3178f2dd02eea450	0	0	0	30	0	0	0	0	0	0	0	0
451a3844784f5c5f5e2418a518ef423b	0	0	0	0	0	0	0	0	0	23	0	0
58ee2a017e2cdbc5f50abeb67b2079bb	0	0	0	0	2	0	0	0	0	0	0	0
6e3c652ad40e44aab530b74569ef6637	0	0	0	0	0	0	0	3	0	0	0	0
f88830c3cd3e45c0aa98cef5aa9b1521	0	0	0	0	0	0	0	0	0	0	3	0
04504862fc76df1d0fa5356ee19dca9f	0	0	0	0	0	0	0	0	0	0	3	0
cdfb52f670a6f6bd9be04d3ab4fc2f10	0	0	0	0	0	0	0	0	0	10	0	0
2a58e33b4e2e66935ae089cdf026884c	0	0	0	0	0	0	0	0	0	0	4	0

53515a0a4389590543b100b 8c5fcb1af	0	0	0	0	0	0	0	0	0	0	35	0
92ea2e69857127ded07e939 4ee9e02ca	0	0	0	0	0	2	0	0	0	0	0	0
8a4131fa466f1ee9bd98e511 3ab4e656	0	0	10	0	0	0	0	0	0	0	0	0
894c8433cfa431859b9e3751 e308f85c	13	0	0	0	0	0	0	0	0	0	0	0
96105648bd3c9dbba392c54 9aa7def6d	0	0	0	0	0	0	0	0	0	0	0	28
53abfeeda72f1f543b94958ae 974e298	0	0	0	0	0	0	0	0	0	0	0	6
90e35cd2808533e68d1f66de c5f005c8	0	0	0	0	0	0	0	0	0	0	132	0
51e9382dd0dde1b3a31f156 98e0d89b6	0	0	0	0	3	0	0	0	0	0	0	0
6c5abee20054730acdc27de6 b4e16e37	0	0	0	0	0	0	0	0	0	0	0	3
f5623d2055a3fec31895767b 66f5253e	0	0	0	0	0	0	0	9	0	0	0	0
e44b2ad77ff979dc315c7dc9 60177439	0	0	0	0	0	20	0	0	0	0	0	0
4e6fc09a2366403a1779dc18 b0f8f67a	0	0	0	0	6	0	0	0	26	0	0	0
4da70fec86505b8c5c3f698d db008d15	0	0	0	0	0	0	0	2	0	0	0	0
b94e343730efbb68ab1e789d a28744b5	0	0	0	0	0	0	0	0	0	0	6	0

8f34edc983261d9df1595246fdd1e4fa	0	0	0	0	0	0	51	0	0	0	0	0
fc3ddc5f5cb888965f7898fcc0329abd	0	0	0	0	0	8	0	0	0	0	0	0
1f4b2ec115e86ba3fa1ff0ef3bec43cb	0	0	0	0	0	0	46	0	0	0	0	0
19c6b72f2141f70ba2bb585506490250	0	0	0	0	0	0	0	0	0	0	0	3
67d30d947800d3d4994dc1664f643e66	24	0	0	0	0	0	0	0	0	0	0	0
8c7ab0699153a19103b381bd990b9fa9	0	0	0	0	0	0	0	0	3	0	0	0
36654d503d5c0bcd23f58aa41799b23c	0	0	0	0	0	0	0	0	0	0	6	0
0333258067cbe68c06d92ec2a478ed15	0	0	0	0	2	0	0	0	0	0	0	0
2c2590d43a9b37b730ff0d3def57a46c	0	0	0	0	0	0	0	0	0	0	0	5
8104c56d568e623140ee1d2c93b1b4d8	0	0	0	0	0	0	0	0	0	0	2	0
b800114e6a4649b1f1a16fd7d1b11c0d	0	0	0	0	0	0	0	9	0	0	0	0
8d6d1c5cadd94e3a8e10af0c26c3438f	0	0	0	0	0	0	0	0	0	0	3	0
93b6c3f0a9945135d18c2047f0cd4c90	0	0	0	0	0	0	0	0	0	0	5	0
6a6b66093b42042124dde2697441571f	0	0	0	0	0	0	0	0	0	10	0	0

4c38dd0f72c5cc2b4cb2fdc89715ff15	0	0	0	0	0	0	0	0	0	0	2	0
b7d0bcb17e756801d89acfb1d939591e	0	0	0	0	0	0	0	0	0	0	2	0
a08a574f7e77682dd3f4d4f58103b54a	0	0	0	0	0	0	0	0	0	0	29	0
8931b29ef40dcd94b07216a438c4e1b4	0	0	3	0	0	0	0	0	0	0	0	0
c972306b4044e37f0b8d4ffad0da4020	0	0	0	0	0	0	0	0	0	0	7	0
724b8a3c7b8edd0e63f6ee9a1272ff85	0	0	0	0	0	0	0	0	7	0	0	0
04aa6726d92eb67ed52e3b78d4a99dc3	0	0	0	0	0	9	0	0	0	0	0	0
d8935413a6c7ad365754ed1a428550b2	0	0	0	0	0	6	0	0	0	0	0	0
599496bdfc5cccaf345ce155747124e6	0	0	0	0	0	0	0	0	20	0	0	0
93d49d647b793980317a2db1333ba706	0	0	0	0	0	0	0	3	0	0	0	0
920a5883371d0174b7c6016d72624fdc	0	0	0	0	0	0	0	0	0	0	0	35
59fbc3207b482938b30625f5000e74a0	0	0	0	0	0	0	5	0	0	0	0	0
f0013deebed6b8147076e28453ecad22	0	0	0	0	0	0	0	0	0	0	0	3
5b71f26fdb769d7efb37ab151d492168	11	0	0	0	0	0	0	0	0	0	0	0

2d8391422579f569f6980c782cdeaf36	0	0	0	0	3	0	0	0	0	0	0	0
16d6a3ecdbd75041917106b8b0cb720a	0	0	0	4	0	0	0	0	0	0	0	0
f77bcab0332ad5d70a6625d687c23980	0	0	4	0	0	0	0	0	0	0	0	0
7fe152e53bb5c6e5dbd7aed28c389b01	0	0	0	0	0	0	0	0	0	0	4	0
54062b1cf901b7c8c05cfe2b777ca124	0	0	0	0	7	0	0	0	0	0	0	0
2667a70a4b6840b6d6d068e8e86042f2	4	0	0	0	0	0	0	0	0	0	0	0
26922f3a6bf96ad35d82a867702707ae	0	0	0	0	0	0	0	3	0	0	0	0
a490bb095f9d9137935bb90c8fee6c86	0	0	0	0	0	0	0	0	0	0	4	0
197c00ed53a0e30278efcb034c6f08d3	3	0	0	0	0	0	0	0	0	0	0	0
451b80c007b662f6d8e6af3564821ff5	0	6	0	0	0	0	0	0	0	0	0	0
2be3d7b98756cb66592bea5888d62c7c	0	0	0	0	0	2	0	0	0	0	0	0
4b52cd5ff4ad0b0d6aaac83ee61246fe	0	0	0	0	0	0	0	0	0	0	9	0
d2a47551e06de4e048d21f6be682bbae	0	0	0	0	0	0	0	0	0	4	0	0
aa27547b25519ad66d1c7dd41e4535f6	0	0	0	0	0	0	0	0	6	0	0	0

913cd0e71a8e66d2f0e03617d79f02a5	0	0	0	0	0	0	3	0	0	0	0	0
c90f8be5fc39e15ee967c090522861ca	0	0	15	0	0	0	0	0	0	0	0	0
4a5482295eaba14cb6ad9be94df218db	0	0	0	0	23	0	0	0	0	0	0	0
345f2249e6569c2396775ac7de02ab6e	0	0	0	0	0	0	0	0	0	0	0	11
7e45a1e67255423bb8b70a2c6342f896	0	0	0	0	0	25	0	0	0	0	0	0
e099ffd8dd25160fe048d1bd6183bad5	0	0	0	0	0	0	3	0	0	0	0	0
0868dae2258b99e402882f97ef0cfd6c	0	0	8	0	0	0	0	0	0	0	0	0
2aabf79050946ccf1af56382ab7adf86	0	0	0	0	2	0	0	0	0	0	0	0
b8fcac80e476f1bd09f999fc5d984bd7	0	0	0	3	0	0	0	0	0	0	0	0
1e897727d2b8da00f622e1261b9f4baf	0	0	0	0	0	0	0	0	28	0	0	0
98df4596ef0a9e917eca6ddd3c0d4db6	0	0	0	0	0	0	0	0	0	0	5	0
566419b342e5fa6783f51932ff4a41fe	0	0	0	10	0	0	0	0	0	0	0	0
3d61e27fdf2c50399f391be29b79e350	0	0	0	0	0	0	0	0	0	0	11	0
677d60397218bf6a9de6637b7d5d0a6e	0	0	0	0	0	32	0	0	0	0	0	0

2d2774c3d63cd26693a4930dfa3af3de	0	0	0	0	0	0	0	5	0	0	0	0
90193b784d67139e39fceafec241592d	0	0	3	0	0	0	0	0	0	0	0	0
e226a4fbdf298916f5dda8a8ec8ac1d8	0	0	0	0	0	0	0	0	0	0	2	0
1f45bf66dcd208180e6ea7ba3f216cfa	0	0	0	5	0	0	0	0	0	0	0	0
8274449866982441599edfca023e9173	0	0	0	0	0	0	0	0	6	0	0	0
7a74e7639b4cfa27b8d3208598f58d4c	0	0	2	0	0	0	0	0	0	0	0	0
c5717b7eff3f8dcf542e73b377e769d8	0	0	0	0	0	3	0	0	0	0	0	0
2617aa15189f3bfb7bb50400726d133f	0	0	0	0	0	0	0	0	0	6	0	0
2f8099ce57c5b0dac605efea0f20a243	0	0	0	0	0	4	0	0	0	0	0	0
a013593c0681c85647c41c794da0a5c9	0	0	0	0	0	0	0	0	0	3	0	0
aadf02418e68b33324d947a183043cc3	0	0	0	0	0	0	3	0	0	0	0	0
22dd91f0e9fb83195e0aa8608d60535a	0	0	0	0	0	0	0	0	0	0	0	38
820dfebf169797742a2f7641954d9c25	0	0	0	0	0	0	3	0	0	0	0	0
611b831046e47bf774c48e93ca97fd05	0	0	0	0	0	0	0	0	0	0	232	0

770d4a31199b5996b00ed68a3864e854	0	0	0	4	0	0	0	0	0	0	0	0
5b5121ec3705eedb03306cfb553f693c	0	0	0	0	0	19	0	0	0	0	0	0
61ef9245a76f5c1b7abc3c619de187df	0	0	0	0	0	0	0	0	0	0	13	0
f184ae3cd3fdafe18441130a4fe0e959	0	0	0	0	0	0	0	0	0	6	0	0
3fad1283b6c1aa7ab3e0e9b0eb387ed1	0	0	0	0	0	0	0	0	12	0	0	0
f34e252e667f3f0d08db62353a653f70	87	0	0	0	0	0	0	0	0	0	0	0
109319b38c4c78440c55d25c3564289c	0	2	0	0	0	0	0	0	0	0	0	0
41f80d2bea26090727b33f6c84484229	0	0	0	0	0	0	0	0	12	0	0	0
fe23bb24e66a02069d19c292b364ebc4	0	0	0	0	0	0	0	0	0	18	0	0
2b7ff9d54d1c4efa1f6352597485b202	4	0	0	0	0	0	0	0	0	0	0	0
c7465a3ace2e278f130b4c7787e188cd	0	0	0	5	0	0	0	0	0	0	0	0
63e4b8ae78c1c898f0e5a241ea78fe7d	0	0	0	0	0	0	0	0	0	21	0	0
75cf92ed5cc13ee11ed85ef89d1745bb	0	0	0	0	146	0	0	0	0	0	0	0
fa85d8e1aa7809fa24fb2e8ec4af81b4	0	0	0	0	0	0	0	0	2	0	0	0

96b9db2cfd980e6fa08696c4b6c9d6e0	0	0	0	0	0	0	0	0	0	0	0	53
97988d88898ac80359d584dc35970fdf	0	0	0	0	0	0	0	0	0	0	26	0
e91a64d9da7c1557d18d7b2baf3cffa9	0	0	0	0	0	0	0	0	0	0	72	0
2a99e8f65cc38bb1188e0c8abdfbb257	0	0	0	0	0	0	0	0	0	0	85	0
0ad787bd4e306f41cbaab4e7c30af0e7	0	0	0	0	0	0	0	19	0	0	0	0
26adfff64ab379540bee33f9a4fdda04	2	0	0	0	0	0	0	0	0	0	0	0
71def61bfd73f1e81009d3eeaa9be6e1	0	0	2	0	0	0	0	0	0	0	0	0
a780bc955b081499c8f256843696f137	0	0	0	0	0	0	0	0	0	46	0	0
8d81a9ff8529e5b5ccbc8711ffc022af	0	0	0	0	0	0	0	2	0	0	0	0
907a8e9fea72120336efac2e7c74692b	0	0	0	0	0	0	0	8	0	0	0	0
a729ff7e556f4ac3930c1cfbbb4f62ad	0	8	0	0	0	0	0	0	0	0	0	0
3392a848c177e5a08cc34a1fa11de244	0	0	0	0	0	3	0	0	0	0	0	0
60e3d05b8b1761219c1fbcd21d2e2253	0	0	10	0	0	0	0	0	0	0	0	0
352bdb55db80599a3e10735846c0f2fb	0	0	0	0	3	0	0	0	0	0	0	0

82000ce419b0856f0994135c02e79798	0	13	0	0	0	0	0	0	0	0	0	0
5380378f9b8be71b77547d1a461dbc29	0	0	0	0	0	0	0	0	0	0	19	0
6ab54fe4b391398532260dc0997d370c	0	0	0	0	0	0	0	0	0	0	3	0
50b5d02f69b64839991c1bfbef32e0b6	0	0	0	0	2	0	0	0	0	0	0	0
f3050819de7619a87d9188b7b97f68cd	0	0	0	0	0	0	5	0	0	0	0	0
df02f9c7a9278f796f67999535e2207b	0	0	0	0	0	0	0	0	0	0	9	0
39cd68d8687a1310b7c117f6107c8683	6	0	0	0	0	0	0	0	0	0	0	0
14ed6d511d36dd69c6d4e88f2ec84394	0	0	0	0	0	0	0	0	0	0	0	5
053df11705cb2bfc5cf8c0fd54554973	0	0	0	0	6	0	0	0	0	0	0	0
ebc4c097fa28213df0031169f053e27f	0	0	0	4	0	0	0	0	0	0	0	0
1419c6ea2269509a3821df1b9fc952ff	0	0	0	0	0	2	0	0	0	0	0	0
756369027cd82b0534cc3b924fdbc008	0	0	0	0	0	7	0	0	0	0	0	0
ae0c226ce1a16b7f2ab78da58cb01689	0	6	0	0	0	0	0	0	0	0	0	0
15c5eb6b49cd47b85987ef04e27c325c	0	0	0	0	0	0	0	0	0	0	0	2

1d0d989f02e4f2e0304dd2b48a5c5402	0	0	0	4	0	0	0	0	0	0	0	0
783640e9bf76e6fabe16d91b5a4241f8	0	0	0	0	0	0	0	0	0	0	2	0
faf63ae839f49428c65d67b611d63514	0	0	0	0	0	0	0	6	0	0	0	0
3089200557c04d4236805806e960d389	0	0	0	0	3	0	0	0	0	0	0	0
0b7e4d112b19b4fa32df7febf85809af	0	0	0	0	0	0	0	0	0	4	0	0
aa1a29e8a51347d361c160b960fb6a57	0	0	0	0	0	0	0	0	0	3	0	0
e318c21565a8ce819698746f7e7d429e	0	0	0	3	0	0	0	0	0	0	0	0
b6cc8f90709cc7616d908577f5e10f0f	0	0	0	0	0	0	0	0	0	0	9	0
8c80f93741d7e92793ff7020fa226694	0	0	33	0	0	0	0	0	0	0	0	0
a71657aa30acd59db1243ceb7e89c9be	0	0	0	0	0	0	50	0	0	0	0	0
0c14b54af0be6971406e0b7ab1ab84dd	0	0	0	0	0	0	2	0	0	0	0	0
9b989c263cd5fea6df5b16382fa5c437	0	0	2	0	0	0	0	0	0	0	0	0
ed8ae4b67dcd1f3c4e04d58bd95f3d14	0	0	0	0	0	0	0	0	0	40	0	0
ca88794de2799f724302beb1e834448f	0	0	0	0	0	0	0	0	0	0	5	0

d3153fbc47b1461c0affd9ea680773df	0	0	0	0	0	0	0	0	6	0	0	0
909d538e1651e9c95a57ea994e06575c	0	0	0	35	0	0	0	0	0	0	0	0
8c13f37c1841816fa3041449572298bf	0	0	4	0	0	0	0	0	0	0	0	0
eb947e3a418d6d2f7caabe1aaeaef65f	0	0	0	0	0	0	0	0	0	0	11	0
2001b87ec45a0217aa02ee01b71434cd	0	0	0	0	0	0	0	0	0	13	0	0
da0541d0c24579a968314f0652db3951	0	0	0	0	5	0	0	0	0	0	0	0
0bba0931f4cee75d393bc0553ec0c044	0	0	339	0	0	0	343	0	0	0	0	0
ad1ed83481616679260afbb7457b29f0	0	0	0	0	0	0	0	0	0	0	2	0
ef63775cd934a24a6312de207eeae640	0	0	0	0	0	0	0	0	0	0	12	0
b46b6d507153ea3acd5f5bffe7120367	0	27	0	0	0	0	0	0	0	0	0	0
12b0c8e9463c77858a0899de9088fa75	0	0	0	0	0	5	0	0	0	0	0	0
b6dbd2023f73d0263797f0996a413bda	0	0	0	0	0	0	0	0	0	0	8	0
9324b845545c0620546d2c728de8aa12	0	0	0	0	0	0	0	0	0	0	0	14
e45407195e18074030613d7e73f6a909	0	0	0	0	18	0	0	0	0	0	0	0

e84a056646143b3e6fe300798660a9c7	0	0	0	0	8	0	0	0	0	0	0	0
4a1a17f6a3142806b377ec77bd1d627a	0	0	0	0	0	0	0	0	0	9	0	0
b4deb8efeecd537a9af70f43d74912a9	0	0	2	0	0	0	0	0	0	0	0	0
3fac783775d79fef50e381350624ef1b	0	0	0	211	0	0	0	0	262	0	0	0
f37e2f0e66f7e92f09e7ed4ed49b97a7	0	0	3	0	0	0	0	0	0	0	0	0
bb999011cbea00b556d00fa31af6d999	0	0	0	23	0	0	0	0	0	0	0	0
6a5b21635305ed2b3e882660e67af594	0	0	0	0	0	0	3	0	0	0	0	0
4f81880667ed25167eceb340ab0c144c	0	0	0	0	0	0	0	0	0	0	0	8
34cad6661d2b5f66f9263f944de6bd92	17	0	0	0	0	0	0	0	0	0	0	0
fd0bd718070caa0c9e9fd94cb826dfdf	0	0	0	0	0	0	0	0	0	27	0	0
4c00b7cf3612316fc359712753b124d5	0	0	110	0	0	0	0	0	0	0	0	0
0aacd0c132e23ed2d4a8cf0364c3e8ba	0	0	0	0	0	2	0	0	0	0	0	0
2d0f220b702d37ff6e7efb82f805b2cf	0	0	0	0	0	2	0	0	0	0	0	0
33c967ffd80bb4d2166bc303a1a9d93f	0	0	0	0	0	0	0	33	0	0	0	0

00c8035ba8ba8899360855c5e8411a3b	0	0	0	0	0	0	0	0	0	2	0	0
fec4fb4efe302407478e25dbf3d1c342	0	0	0	0	0	0	0	3	0	0	0	0
0ff10b1a96300a9b206c856b3c476c0d	0	0	0	0	0	0	0	0	0	0	2	0
f1b9aa8b9f3270b133ddb7254a0ee223	0	0	0	0	0	0	0	0	0	58	0	0
e1ed880fd87fb1acb537a8c001e754c6	0	0	5	0	0	0	0	0	0	0	0	0
0357df42ed329568de0b51909efc5f78	0	0	2	0	0	0	0	0	0	0	0	0
5f38a6a2de58b49a68bb093499bca27c	0	0	0	0	0	2	0	0	0	0	0	0
bbb89a76792e5cdd0977f957ac9f7c60	0	0	0	0	0	0	0	0	0	0	0	18
0b3be5639f9c912b6ff75451ec917fa9	0	0	0	0	0	4	0	0	0	0	0	0
20ba10edb9d8ce2f243dfed7d2ae268c	0	0	0	0	0	0	0	0	0	0	3	0
231cbd691560e798aa2d8ba521c634f6	0	0	0	0	0	0	0	0	0	0	3	0
db8956b0a6bd8e944c0a797b2c0d0c37	0	0	0	0	0	0	0	0	2	0	0	0
7648eba53786033006abe8c365c5055c	0	2	0	0	0	0	0	0	0	0	0	0
e3208ef12c4347d6c7b83e71ecdc8a08	0	0	0	0	0	0	0	0	30	0	0	0

c990421cb6d3166146a1f5318449f616	0	0	0	0	0	0	0	0	0	0	0	4
1d0ee0c64e2d52b07e6dcb3da5e83645	0	0	0	0	0	0	0	0	0	0	4	0
69b9da7feb6875d19118fe147782b9d4	0	0	0	0	0	4	0	0	0	0	0	0
3c5219e8d56d84023915da7a5f72172c	0	0	4	0	0	0	0	0	0	0	0	0
aa6fadc8e926b53ac5d29cb6c365490b	0	0	0	0	0	38	0	0	0	0	0	0
d385c6a8f6897c09bb4bc26e1f064e74	0	0	0	0	0	0	0	4	0	0	0	0
a126fe0c64f896d182a7354c310eb586	0	0	0	0	0	0	0	0	0	0	0	9
0a4875bc4f64a2d43b36d3f9df0ae5d8	0	0	0	0	0	0	0	0	0	0	3	0
5bbe2ed6ee143735b993a99e8314d54c	0	0	0	0	0	0	0	3	0	0	0	0
1f03c211fda730a64f026bb9e57ebb0b	0	0	0	0	0	0	0	0	7	0	0	0
e0431b7603807ac10bbc5d8f45691ec9	0	0	0	0	0	0	0	0	8	0	0	0
c2d119bb817bbb988fbc23163a51b999	0	0	0	7	0	0	0	0	0	0	0	0
0864a80b100bc156c35a5eae7ef32aea	0	0	0	0	0	0	0	301	0	0	0	0
a98290350591a8d2081ac55826a9fa7d	0	0	0	0	0	0	0	2	0	0	0	0

5883e32f084ed6bdb00f439bc31e6bf1	0	0	0	0	0	0	0	0	122	0	0	0
42528848422a83f79a70f759ee93cb5d	0	0	0	0	0	0	0	0	0	0	3	0
16fb8625ec1e62c568a4810f5a6fd9e9	0	0	0	0	0	0	0	0	0	0	22	0
b619d3bf6d904c2ceb4e7a7b9ad3b1ee	0	0	0	0	0	0	15	0	0	0	0	0
a7a663b66e92dba23ec0ef969bb27a6f	0	0	0	0	0	3	0	0	0	0	0	0
fc3820760b3a6af3d52a333bdb464ddd	0	0	0	0	0	0	0	0	0	0	7	0
af08e8e5238dda3ba1536a7d84d41687	0	0	0	0	0	0	0	0	4	0	0	0
eb26b7f422db71f568a9728b0d295f87	0	0	0	0	0	0	16	0	0	0	0	0
1ec5cf2d587983aac73eddd46fe69a70	0	0	0	0	0	0	0	0	0	15	0	0
7a4a54575b791bb16d22d5fb8a6fbfda	26	0	0	0	0	0	0	0	0	0	0	0
2016b31ce2ed6fb90cab27792193be0c	0	0	0	0	0	0	0	0	0	0	4	0
f34ba0adfefd9240f1d18bd20ddc7426	0	0	0	0	0	0	0	0	0	0	13	0
33de6ec1162efa69bf729a860ec8d177	0	0	0	0	0	0	0	0	0	0	2	0
838dfc5b80fa6cae580317dc57bbea7c	0	0	0	0	0	0	0	0	0	6	0	0

30e9c341ae1020413fe7ac65f10a0f57	0	0	0	0	0	0	0	0	0	0	8	0
14a92e1ce2fe1ab4f98d21c47fe05a47	0	0	0	0	0	0	0	0	7	0	0	0
937afad7ef5cc2af4d8f8c917738ad67	0	0	0	0	0	0	0	0	0	0	0	5
b18093f019970ac1d2a5991886d89713	0	0	0	0	0	0	0	6	0	0	0	0
ea36bbcb3d90c01d2ba66225d979db0a	0	0	0	0	0	0	8	0	0	0	0	0
3f02157cc9d5211ac56df9f634a74acd	0	0	0	0	0	0	0	0	0	0	4	0
85f10743c538e6bf7b0b23dc1830b828	0	0	0	0	0	0	0	0	0	26	0	0
2d772844dbbf49834faa9b89d44f9166	0	0	0	0	0	0	0	0	33	0	0	0
882af5a259027bdbe24d69994dc8b5dc	0	0	0	0	0	0	0	0	30	0	0	0
9321e77e124dec2400fa6a6689cc188e	0	0	0	0	0	0	0	5	0	0	0	0
cbddba60ba342daf54242945fa171b0e	0	0	0	0	12	0	0	0	0	0	0	0
cf9f48d7861ff397900af6d6d78f4de2	0	0	0	0	0	0	0	0	0	0	0	8
9ef697441990cb835e530485950ea5aa	0	0	0	0	0	45	0	0	0	0	0	0
4c2476f4073c22ccc804618b7326664d	0	0	0	0	0	10	0	0	0	0	0	0

19dfef8c88cf901f4268e5769 b49f978	0	0	0	0	0	0	0	0	0	4	0	0
bd6e3361687f96fc18840ee5 1a8a73ff	0	2	0	0	0	0	0	0	0	0	0	0
c8b8d2167a862f52e6a7a76a cc832913	0	0	0	0	0	0	7	0	0	0	0	0
77a797c5e99a181b2fea5d1e 5ab6722c	0	0	0	0	0	0	0	13	0	0	0	0
f1c1b504db8d24b9741ae958 4ccbd268	0	0	0	0	0	0	0	0	0	0	26	0
6134d2714494ec048735d36 4b5582449	0	0	0	0	0	0	0	0	0	0	4	0
f74b674edc908c52e03befe2 4cd23fcf	35	0	0	0	0	0	0	0	0	117	0	0
7539199493663b115723d14 f94d67ce0	0	0	0	0	0	0	0	0	0	4	0	0
595b57d8bd81fd665b374c0f 60fbfa76	0	0	0	0	0	0	0	0	0	0	0	13
ae0174b5a664202d620b8d5 1f9b7375a	0	0	15	0	0	0	0	0	0	0	0	0
be9ec6d07d1e834c0abdb9b ecedbfcbd	0	0	3	0	0	0	0	0	0	0	0	0
ba93fa15b9c6256941cc91ea a7ee57f4	0	0	0	0	0	7	0	0	0	0	0	0
d10d6bdc62437723fa0a4f3a 56262daa	0	0	0	0	0	0	0	0	0	0	7	0
da295796e74f7dbc7c68b348 840cc893	0	0	8	0	0	0	0	0	0	0	0	0

d9d21af8bfcaa805ac501a62cd031b63	0	0	0	0	0	0	0	0	0	0	0	5
e94535fb79406b8cad12400f2e1db423	9	0	0	0	0	0	0	0	0	0	0	0
44eb7d7404a4c3dbd066a4201540d980	0	0	0	6	0	0	0	0	0	0	0	0
97b3b9cd1514ef315ca47ec5c6b29cd0	0	0	0	0	3	0	0	0	0	0	0	0
90950678710f4de3c1b7f11ffe25de5e	0	0	0	0	0	0	0	0	0	0	0	3
8a515c566c6abd727dce0e35cffc0c00	0	0	0	0	0	0	0	0	0	0	16	0
b3954e636e28d5bcc21110816416aff8	0	0	0	0	0	0	0	0	0	0	0	8
d1c235bb293e91e6527f525d456d7c9f	0	0	0	0	0	3	0	0	0	0	0	0
afd2dbc2b5239e4e955af0015d3ace8f	0	0	0	0	0	0	0	15	0	0	0	0
d2b128dc248bd107d8b2abf9a85877ae	0	0	0	0	18	0	0	0	0	0	0	0
f11c6cf5c2a61a8b429772c3322a0284	0	0	0	0	0	0	3	0	0	0	0	0
65575147558193b7eba8aee6e7b091a6	0	0	0	0	0	0	0	0	0	0	6	0
2dadb8733f0086a6d3a09cc05c0bb753	0	3	0	0	0	0	0	0	0	0	0	0
af62381b5e29e6727a2b90442f7ca22b	0	0	0	0	0	0	0	0	0	0	0	8

2310d666fc2fd7ef7765a202b142c50a	0	0	0	0	0	10	0	0	0	0	0	0
5e357ad3a3e9283971ff4440fb10e5ba	0	0	0	0	0	0	0	0	0	3	0	0
6dbfe8b5cabc08d67060c663c9cee864	0	0	0	0	9	0	0	0	0	0	0	0
9e3a01d145bd9a7945b4f70496724249	0	0	0	0	0	0	0	0	4	0	0	0
3de65b1c3998ab0c7402d5754f08261a	0	0	0	0	0	0	0	0	2	0	0	0
e4d472ad2fc5869fc5d325a14e6dbb71	0	0	0	0	0	0	0	23	0	0	0	0
82a6d2d072be417c9a2636a9cb0d7a81	0	0	0	0	4	0	0	0	0	0	0	0
da76327b43b2e9ea9b7739ecfb227cd2	0	0	2	0	0	0	0	0	0	0	0	0
a58ff9a8634dd72d85112794025e1591	0	0	0	0	0	0	0	0	20	0	0	0
5a0f408b588d943c0243bc25da85621c	0	0	0	0	0	0	0	23	0	0	0	0
e08f89ae31f6d06e2982e0bc2fad7b15	0	0	0	0	0	0	0	0	0	0	5	0
12d120141941d3983ac7d8466e095846	0	0	0	0	27	0	0	0	0	0	0	0
61daebae5e98e09073b48ebcf01f921f	0	0	0	5	0	0	0	0	0	0	0	0
00de81aa21552580d6c491fe6a3cee8c	0	0	0	0	0	0	0	0	0	0	31	0

c1bba19a0ac53a81bd8f5e17 54403a89	0	0	24	0	0	0	0	0	0	0	0	0
806b1dd2c24391425a2fc911 f890bfed	0	0	0	0	0	0	72	0	0	0	0	0
b844b383cd884595bca7511 3a6c82037	0	0	0	0	0	0	0	0	0	0	39	0
2919b8802391ddc02e09330 0efb6fab2	0	0	0	0	0	0	6	0	0	0	0	0
99bd7444e74659c0479a112 2707fe2c4	0	0	0	0	0	7	0	0	0	0	0	0
cea6882f51eb868203d521ae e4508985	0	0	0	0	0	27	0	0	0	0	0	0
8b40d4ac1736e0ab8168ea3 a51b0dda4	0	0	0	0	0	0	0	0	0	0	4	0
e5e4139ff9fb7f1107162cb08 027b162	12	0	0	0	0	0	0	0	0	0	0	0
99f2199c08e005e46bac035c 47ad8bf1	0	0	44	0	0	0	0	0	0	0	188	0
9d2155ea1861e8820964bfe6 365f3871	0	119	0	0	0	0	0	14	0	154	0	0
a65d0c55f5cdc69690b2111c e0f9ecba	0	0	0	0	4	0	0	0	0	0	0	0
f11911977938175d2544205 ddef9fbba	0	0	0	13	0	0	0	0	0	0	0	0
6c6729d0312a9a594111160 2eb959226	0	0	0	0	0	0	0	0	0	0	0	44
47f72a350bf7dfffe437f65bc7 ce0f1c	0	0	0	0	0	0	0	3	0	0	0	0

e587491ecdfc77ec8dc72221 844453b2	0	0	0	0	0	0	0	9	0	0	0	0
61c2445ab5dc91761a4b800 7d2750d4f	0	0	0	0	3	0	0	0	0	0	0	0
73efe85f5fc0d7cb905bfb2e3 cd45f79	0	0	0	0	0	0	0	0	0	0	0	7
6dcac22c6550fe4ad147a4e9f 6598f8d	0	0	0	0	0	0	0	0	0	0	15	0
a409fc13e56412bc7743f5ee 44abea42	0	0	0	0	0	0	0	0	0	7	0	0
2e9d15a2223bde6cb7b46f7d 670f1d62	0	0	0	0	0	3	0	0	0	0	0	0
7da6e74fa49616eb18d95c8f 3c565365	0	0	0	0	0	2	0	0	0	0	0	0
0e803664ba02164a2548a61 9fe2b2a92	0	0	0	0	0	0	0	0	0	0	0	3
eb32cd1e65a5a02479b0b97 a2d287607	0	0	3	0	0	0	0	0	0	0	0	0
fbff53d7a8ec3d35847b4087a d3be062	0	0	0	0	0	0	0	0	0	0	0	3
4fd28b9e4f38299f000ea65cc cd5d545	0	0	0	0	0	0	4	0	0	0	0	0
5ae50749ae4b65582ba9f27e 76861620	0	0	0	0	0	0	0	5	0	0	0	0
fec40e78ab8a9663131c841f 1b5e795b	0	0	0	0	15	0	0	0	0	0	0	0
a102c0effcb79a5bd7b833a8 14951fa0	0	0	0	0	0	19	0	0	0	0	0	0

89271c4755b131512650f81fc8351d17	0	0	0	0	0	0	0	7	0	0	0	0
9d867acfdc95d89a2ab867ec34126cce	0	0	0	3	0	0	0	0	0	0	0	0
3490dfb87dc3d28a38fb3421d62be873	0	0	0	0	0	3	0	0	0	0	0	0
b11ebdef423127d3b27c5e6c1fd31337	0	0	0	0	0	0	0	0	0	12	0	0
3f2604a202a09f2f59c09e3b1927440e	0	0	0	0	0	3	0	0	0	0	0	0
934984a399683c8ff71b93fb079025f6	0	0	0	0	0	0	0	0	3	0	0	0
dd01c6328c0e4dc47b0657c55dcb8a0d	0	0	0	0	0	0	0	0	0	0	4	0
58c576d162b5e430694fff6f4870f0dd	0	0	0	0	0	0	0	0	0	0	0	2
a5e359f245b23b4a9558e5de07aa1ae6	0	0	0	2	0	0	0	0	0	0	0	0
8b3b0aa9bb629a9721538612d4bc0316	0	0	0	3	0	0	0	0	0	0	0	0
e63eb2715b75f3d4fdbbe25006e46fc8	0	0	0	0	0	0	0	4	0	0	0	0
25496f19ee2fc9667283d30db65e4416	0	0	0	0	0	2	0	0	0	0	0	0
3b6da6722dafabc83eb9a8769932d495	0	0	0	0	2	0	0	0	0	0	0	0
7fd9a2f4860a46145afa822d715e348b	0	0	0	0	0	3	0	0	0	0	0	0

3d4353f3871646cf1a813f3dc531d706	0	0	0	0	0	0	0	0	0	0	12	0
03461bab2c5e84e315dc950a988d1a82	0	0	27	0	0	0	0	0	0	0	0	0
ea555c51d40795c999277dde8e012782	0	0	0	0	0	670	0	0	0	0	0	292
f53f5ec820b882d439c13044d1f6a7ee	0	0	0	0	0	0	0	0	2	0	0	0
e3dff01f429b3b7a043619e3635b3e6b	0	0	0	0	0	0	0	0	0	0	0	2
311a825d994baae546d6ff7d3879a804	0	0	0	0	0	0	0	0	0	0	4	0
1c9c6f03f886c090032fb00977d06feb	0	0	0	0	0	0	0	78	0	0	0	0
e9841ab8144a8bf428ee7b025519b80d	0	0	0	0	0	0	0	0	0	32	0	0
a1345018b67844b62965002bce6789c4	0	0	0	0	0	0	0	0	2	0	0	0
7ce18201f9e2350f3646b1548242e035	0	0	0	3	0	0	0	0	0	0	0	0
f81a730e0dba895fcd3a92f3ee00eb66	0	0	0	0	0	0	0	0	0	2	0	0
b5baa9fe1dedbef3bf54812926e3ec55	0	4	0	0	0	0	0	0	0	0	0	0
44d386e103f7be4537ea7260fab4d6cc	0	0	0	0	0	0	0	20	0	0	0	0
fbcc08c620f2e14c916e0706f0148734	0	0	0	0	0	0	0	0	13	0	0	0

dce50286198cdefbae4cf0f2f57aec0c	0	0	0	0	0	40	0	0	0	0	0	0
faaa414c8aea0723dfaacff7d8869b8c	0	0	0	0	0	0	0	0	3	0	0	0
d10c6804949f54738a9b5abf0888efff	0	0	0	7	0	0	0	0	0	0	0	0
8a2b6e248a4f8838145cfff3bfc40e8e	0	0	0	0	0	0	0	2	0	0	0	0
caff0fcc1fc42df9087351f513a92dd3	0	0	0	0	0	0	0	0	0	0	0	14
e3ea17c14c8a9b3679873b36885febc6	0	0	0	0	0	0	0	0	0	0	86	0
6c56aeebba0315f7c4107e45ff25e21a	0	0	0	0	0	0	0	8	0	0	0	0
2158c09daf482cd6089ceee919a3a028	0	0	0	0	0	0	0	0	0	0	0	4
9ebb7284894323a0ad3be896a942de9a	0	0	0	0	0	0	0	0	3	0	0	0
7dafc2b50bb9249232309e90d38d6ae0	0	0	0	0	0	0	0	0	0	0	0	2
446464312b6641ea7ad833b33d93e316	0	0	0	0	0	0	0	0	0	0	7	0
ee00efb72c40a52ed9bf4c538804cedd	0	0	0	0	0	0	0	0	0	22	0	0
01b8b6982777aee7d921f63ab162cc6e	0	0	0	0	0	2	0	0	0	0	0	0
112778fdc9395be4514d84c7e65065c0	0	0	0	0	0	0	0	0	0	6	0	0

5a18239ff61e80c753408fa7fbc7350d	0	0	0	2	0	0	0	0	0	0	0	0
fe4eaa2b2fff9b369036eccd63e6ed0b	0	0	0	0	0	0	0	0	0	26	0	0
54eb7395f5154ef3d295944553611e4f	0	0	0	0	0	4	0	0	0	0	0	0
1318901a5231368895ee7025b15cde49	0	0	11	0	0	0	0	0	0	0	0	0
ff04cea3648258460543e99cfad6894a	7	0	0	0	0	0	0	0	0	0	0	0
ad25b27cf102cc1767d973b5316d7abe	0	0	0	0	0	7	0	0	0	0	0	0
be521ea8e0a7a55c795cb48802a1608a	0	0	0	0	0	0	0	0	0	0	0	3
f273b7c126701961d9e01fcb7d2f8e31	0	0	0	0	0	0	0	11	0	0	0	0
26828ebfaf4c5d195eac622dd3d2fe5c	0	0	0	0	0	0	0	0	0	0	0	21
8574adbe8db5fa7fe1fd902b5e677ea2	53	0	0	0	0	0	0	0	0	0	69	0
ca77da8dd11d53954bb9eae82f21e75a	0	0	77	0	0	0	0	0	0	0	0	0
44a518aeba0f71313c4c9c870d1274cc	2	0	0	0	0	0	0	0	0	0	0	0
8b2941d237b2fee086ddc441b87a5809	0	0	0	0	0	0	0	0	7	0	0	0
e3a367c89fe1098eeba7424475aad04b	0	0	0	0	0	0	0	0	3	0	0	0

4e84be4ca4c2efcb336e04e22315ca99	0	0	0	0	0	0	0	0	0	0	0	6
b10464917aab561f414f139d38261892	0	0	0	0	0	0	0	0	7	0	0	0
057d152698598b0843963e7bd48719f1	0	0	22	0	0	0	0	0	0	0	0	0
0a3bea74b33b6fe22168f8116c437e35	0	0	4	0	0	0	0	0	0	0	0	0
d58ceb3d46f49559d4f0f538ecbd18b1	0	0	0	0	0	0	0	0	0	0	5	0
a0807f5c68c97708ebc0d877c4b57931	0	0	2	0	0	0	0	0	0	0	0	0
f9c09f0684c2c532830301510393650e	7	0	0	0	0	0	0	0	0	0	0	0
dc8e57831b1cacfe229d14659c7dc945	0	0	0	0	10	0	0	0	0	0	0	0
fbd4e413aa48e6dd1571c86926fff14f	42	0	0	0	0	0	0	0	0	142	0	0
ab8c1d09673953fdf753b9fc7d3ad496	0	0	0	0	0	0	0	0	0	9	0	0
26d32d2c9093e8ad0972b11ac85fdfad	0	0	0	0	0	3	0	0	0	0	0	0
876a0922381456283d76bb59b97e51c9	0	0	0	0	0	0	0	0	0	0	20	0
22609a8eedcd92ce9d1a05969b6096b7	0	0	28	0	0	0	0	0	0	0	0	0
3e5b5f74bc9d0fa1733ee78ae8bc2f70	0	0	0	0	4	0	0	0	0	0	0	0

4ac876b4342f22fe11a8c2c5def6dd03	0	0	0	0	0	0	3	0	0	0	0	0
872885b8d67c6efee37344bce2246f0f	0	0	0	0	0	8	0	0	0	0	0	0
a639bc565401b7b8a94e6e8b59fa2a3c	0	0	0	0	0	0	5	0	0	0	0	0
4b3c7006f85a82232728a19174a468d7	0	0	0	0	3	0	0	0	0	0	0	0
f4dba2b4098ee5220c514f750ebc3e09	0	0	0	0	0	0	0	0	0	0	330	0
514fefd513c8959afcf799a132117b79	0	0	0	0	0	0	0	0	0	0	23	0
d5e51fcf2915577d65ce3e4cd72e2161	0	0	0	0	10	0	0	0	0	0	0	0
955d96a97e694a2dfd6621a684b41c31	0	0	0	0	0	0	0	0	0	0	0	5
2613b09e0cbcf10db3b923e610ecad45	0	0	6	0	0	0	0	0	0	0	0	0
e4f83bf13404e2654f8f6db5457c1e28	0	0	0	0	3	0	0	0	0	0	0	0
43f23cf56d1b8cf0c0f822710e44b2a3	0	0	0	0	0	0	0	5	0	0	0	0
8d3731897dc1b9dced8f40e42651b990	0	0	0	0	0	0	0	0	0	0	6	0
59b6f3d2fae9be0c20878a697387b25e	0	0	0	0	0	0	0	0	0	0	0	4
5bfbb86293397d11ad5395853dee6e96	0	0	0	0	0	0	0	0	0	0	5	0

1203fde85128927a5c5d6e3da8b67f62	0	0	0	0	0	2	0	0	0	0	0	0
9e18e61d0fe66fd1d85bcd38fd22d384	0	0	0	0	0	21	0	0	0	0	0	0
4a410d63f27fdf5c1883ec1f9dde1c97	0	0	0	0	0	0	0	0	6	0	0	0
060749def58bf0b0366137559aeee9fe	0	0	12	0	0	0	0	0	0	0	0	0
4791e13fc531a255e843fb15d7941d6d	0	0	0	0	0	0	0	0	0	0	0	2
bbfef1676b600afab431e54d78e8b73c	0	0	0	0	0	0	0	0	0	0	0	32
3f96f07a2e630de7a1ee4e609f9043d4	0	0	0	5	0	0	0	0	0	0	0	0
5f703535328ddad47916d21c2aed927b	0	0	0	79	0	0	0	0	0	0	0	0
732b81827400b8f6ed6f104956d39913	0	0	0	0	0	0	12	0	0	0	0	0
7fb45a0d27371106fb95e156628415a2	0	0	0	0	0	0	0	26	0	0	0	0
d5b4f54041923105b5a8eea9b11d9f1a	0	0	0	0	32	38	0	0	0	0	0	0
131015450294eb3277c026bf9e8221a9	0	0	0	0	0	2	0	0	0	0	0	0
4e9e9a78fc21011778f2f225d54a05fa	0	0	0	0	0	0	0	3	0	0	0	0
a504355a3d5426ab597431c26e212a6e	0	0	0	0	0	0	0	0	3	0	0	0

849a2ee99cd020b149abd02 afcba86a9	0	0	0	0	0	0	0	0	0	0	4	0
b4e56bc4e1fe5170183a3c8f e61f323b	0	0	0	0	0	0	0	0	6	0	0	0
d9ed749ccc3cfad0f790c63c4 ba3b10d	0	0	0	0	0	0	0	0	0	0	0	3
2e85593f7bb8c46627306bf0 f87dd54d	0	0	0	0	17	0	0	0	0	0	0	0
71acd50793e04d7a7cdf3b02 f56779cf	0	0	0	0	0	0	0	0	0	0	0	9
e4262404b539990f679f17d9 5de9ebcc	0	0	0	0	0	2	0	0	0	0	0	0
8db4cd179ff370ed2cc127c90 61482e6	0	0	0	0	0	0	0	2	0	0	0	0
65cfc2e4ab89652685a51302 05c4bca2	0	0	0	0	0	0	0	0	0	3	0	0
b00828ee24a2b010a4e50f8f 6f307ff4	0	0	0	0	0	0	0	5	0	0	0	0
43fc7928d5a6a5112b5972f5 be999982	0	0	0	0	23	0	0	0	0	0	0	0
e42949f69c87d54df7a8e11a 4ffc0ba7	0	0	0	0	0	0	0	0	0	0	0	25
f09eb6fb66bd652a7373b932 f53f5eaf	0	0	0	0	0	0	0	0	0	0	0	4
6eeddb7fba38d95a58118f9b 5752c019	0	0	0	0	0	0	0	0	0	0	5	0
7925c3cff5ab0f3e5d8c98e85 41099ed	0	0	0	0	0	0	0	0	0	0	12	0

a7cf1844deb452e31c577b87e24ccd4a	0	0	0	0	0	0	0	0	0	13	0	0
4bf8bdb3c52337a1ab09a43a1041e5df	0	0	0	0	0	0	0	0	3	0	0	0
9aba15300dee8930ae0e0d0f114e6886	0	0	0	0	0	0	0	0	0	0	3	0
81e78421d38bc0f8307adffecaa1b963	0	0	0	0	0	0	0	0	0	0	2	0
d7ca76fcb66476cfd7149e7402fa5cc3	0	0	0	0	0	0	0	3	0	0	0	0
edd67f38d9f40969829f26c97c9e839c	0	0	0	0	0	0	0	0	14	0	0	0
9593a6a73418dfa52b56f1ee8dd88806	0	0	0	0	0	0	2	0	0	0	0	0
4867ab98e163db59b1a656c7445dc887	0	0	0	0	0	0	0	0	0	8	0	0
6fcfd518340783649f43bf68fe5fa776	0	0	0	0	0	0	0	0	0	0	0	3
dd7c8f844e2fc0907c296f7422385d28	39	0	0	0	0	0	0	0	0	0	0	0
0343178154c999207ff81d04d9c10a26	0	0	0	0	0	0	0	0	0	0	2	0
cd2f94aac4cc3cf52906be709f084b83	0	0	0	0	5	0	0	0	0	0	0	0
9b295ab39040cadc4c3aa0bee1b015f9	0	0	0	0	0	0	14	0	0	0	0	0
15f3c76f66059bd3ad056449d4855f81	0	0	0	0	0	0	0	0	0	0	0	6

3670a0ce441369d87709d57e0d82407b	3	0	0	0	0	0	0	0	0	0	0	0
991cbfa37507e6561b233139549282d0	0	0	0	0	0	14	0	0	0	0	0	0
b0cf654f27342f3ff197ecc1fbf1eb16	0	0	0	0	0	0	0	0	0	40	0	0
543637a7a9177885b893a0eca8eb2047	0	0	0	0	0	0	9	0	0	0	0	0
ef1e8bed95f16931e5aa1d32e31bf21e	0	0	0	0	0	0	0	0	0	2	0	0
0e740cbb5a9861f4128f63648a4f53f8	2	0	0	0	0	0	0	0	0	0	0	0
d0bb75ffe987d1fc8891ca501dfe46b2	12	0	0	0	0	0	0	0	0	0	0	0
97f59f4cd538362d42e7c4d0338fc537	0	0	0	0	0	0	0	0	0	0	0	3
f9b4b35dc0ae3bce43c3ee064ea1795d	0	0	0	0	0	0	0	0	0	2	0	0
8138c8be91bfe01424c6ddc0ce1dad50	0	0	0	0	0	11	0	0	0	0	0	0
6d76da1cfaf61913c6eba81a88fb061a	0	0	0	0	0	0	0	0	0	0	27	0
68af9b97b91211dabc355159a56fc28c	0	0	0	0	0	0	0	0	0	0	0	3
2592407524d52c0dfff96742ca8d0bff	0	0	0	0	0	0	0	0	0	0	0	2
aa9b760e0489019fc1499fac85ebd2b8	0	0	0	0	26	0	0	0	0	0	0	0

0f43ebe08cbfe3502b4a00bc c1eea3e4	0	0	0	0	0	19	0	0	0	0	0	0
6074a146da5698f75210b89c ed4d98c0	0	0	0	0	0	0	0	0	0	0	5	0
4710afaac242f21dc1f80fe91 2ed1365	0	0	0	0	0	0	0	0	4	0	0	0
6965b273a7c3b7cb306559e 76aee189d	0	0	0	0	0	0	0	3	0	0	0	0
c82459bef77fe987ec49975ca 7e9d340	0	0	0	0	0	0	0	0	3	0	0	0
e08da76e00e5e96c8fe37092 52beb9a0	0	0	0	0	0	0	0	0	0	0	113	0
2d056b0d452934d317e91bd dce66e10c	0	0	0	0	3	0	0	0	0	0	0	0
ae95005548cacc7820cc18b3 637e1d92	0	0	0	0	0	0	3	0	0	0	0	0
91fc9f04b43d61b4a262a3fd5 2090f9c	0	0	0	0	0	0	0	0	0	0	4	0
01d268c0c8f578319a3e1aef 6d8dfa5f	0	0	0	0	0	0	8	0	0	0	0	0
399441282fc8d9b9f6a1aad5 7042f74c	0	0	0	0	0	0	0	0	0	88	0	130
8e30b295d04a4208f7e7a292 ef48e80b	0	0	0	3	0	0	0	0	0	0	0	0
cb2d1087ce0c1a632229cdb7 af2f832a	0	0	5	0	0	0	0	0	0	0	0	0
6a90deb7ef7dc16f7e965ce8 1d291334	0	9	0	0	0	0	0	0	0	0	0	0

a0d0ba865e72cd7f6fba5492ca8bac68	0	23	0	0	0	0	0	0	0	0	0	0
7c3a0cf1e438ef072f53ea3bf1cc8d9a	0	0	0	0	0	0	0	0	3	0	0	0
f7ea1bba1149edfc9db542219be797d6	0	0	0	0	0	0	0	0	0	0	15	0
dbccebe3cadbba2d6f8aeb36cc18077c	0	0	0	0	0	0	2	0	0	0	0	0
f57bb956277d08869ca99ffd45c9ffae	7	0	0	0	0	0	0	0	0	0	0	0
524c782b76153590f55c6f9d887d15b2	3	0	0	0	0	0	0	0	0	0	0	0
f0ed808e858f23839a1680ff6c5b7398	0	2	0	0	0	0	0	0	0	0	0	0
6e1cdbe445ba7182fee45ff711f87654	0	0	22	0	0	0	0	0	0	0	0	0
790a4cefc766712ab1eb9770f4f227df	0	0	0	0	0	0	0	0	0	9	0	0
bf141f1b0a3c1911abd8ee050e9d4441	0	0	0	0	0	0	0	0	0	0	0	2
c2e53caef01096791f388607d941c88d	0	0	0	0	0	0	0	0	0	0	0	5
cb12f7e63bd923b6d40b933feb5c5e9a	0	0	0	0	70	0	0	0	0	0	0	0
20c2f9c9c48a0a95dbf4b87cf1e92c73	2	0	0	0	0	0	0	0	0	0	0	0
95eed8c995b2bfaa04860446947d5e7e	0	0	0	0	0	0	0	0	0	33	0	0

0c07560606fd1155123c49a157759efd	0	0	0	0	6	0	0	0	0	0	0	0
bc841200bd6e86348312d2d833ec5515	0	0	0	0	0	0	0	0	0	0	20	0
6e715e5344a48b7d30e79dd0ef632ab8	0	0	0	0	0	0	0	12	0	0	0	0
96c38a5e4f9e80ee9ee5e0992659426a	0	0	0	0	0	0	0	0	6	0	0	0
2c9a3c0ee435963a5a4cbe5ef329bbdd	0	0	0	0	0	0	0	0	0	0	61	0
4135e8dcca065dd1994fab4c6424611d	0	179	0	0	0	0	0	167	0	0	389	0
2b6adee17410da7f0ef536580869dfff	0	0	0	0	0	0	0	0	0	0	5	0
3c3cc9c48bf34d96a1a11a60c9a4cff0	0	0	0	0	2	0	0	0	0	0	0	0
61f82ed04eff8d2fa9ca85c23a3e2a3b	0	0	0	2	0	0	0	0	0	0	0	0
518c0e4c1782261dcdb6b614fd169d94	0	0	12	0	0	0	0	0	0	0	0	0
8500b9f22003d61830b42c3b8e00e17b	2	0	0	0	0	0	0	0	0	0	0	0
84a524745a13695597952e9a8a3367f4	0	0	0	0	3	0	0	0	0	0	0	0
e99ee88a5a2eff71753fac6fa60abcf0	0	0	0	0	0	0	2	0	0	0	0	0
16df9911615152cfb870beeee0ee9dfc	0	0	0	0	0	0	0	0	0	0	0	5

a6c7fbeef419e55cc0ecbb3bafa8cef0	0	0	0	0	0	0	0	0	0	0	35	0
5df54718fab87c02553d684df4ca1724	0	0	0	0	0	4	0	0	0	0	0	0
936563e0c7eeb7bc816c86da88b1006c	0	0	0	0	0	0	0	0	0	0	141	0
57fea3f8220c90e5c75b9ecdd3c8d3b3	0	0	0	0	0	0	0	2	0	0	0	0
add04507720926042621126088a20e7b	0	0	0	0	0	0	0	0	0	0	0	30
3b46f29e2874e0f88276facfee918bc7	0	0	0	0	0	0	0	0	0	0	6	0
92e3fdb4113340f343532173ac88606c	0	0	0	0	0	20	0	0	0	0	0	0
6a7abd6484f06b08c28ad4f6339ceee1	0	0	0	0	0	0	0	0	0	3	0	0
64bf4872ca2c53a57fc6513b37bca9db	0	0	0	0	0	0	0	0	3	0	0	0
5c584a78e66d48b819aba70c6eb8b6b2	0	0	0	0	0	0	0	0	0	6	0	0
003de3d4200c5c960ba814db92ed5ade	0	0	0	0	4	0	0	0	0	0	0	0
43f4ca8235496c1d2dc75a5abd78499f	0	0	0	0	0	3	0	0	0	0	0	0
45d3bd9038d91b92001595ecee14c93e	0	0	0	0	0	0	0	0	0	56	0	0
20e72920777fd5a2f3187fecd5ae88a4	0	0	0	0	0	2	0	0	0	0	0	0

a7a8b6761b50359f767e80a8a8db955b	0	0	0	0	0	0	0	0	0	3	0	0
5a56da1b18a7d2cd0de5f7c7f8bb2057	0	0	0	5	0	0	0	0	0	0	0	0
e2c628d0c5feec796551b934844e9302	0	0	0	0	4	0	0	0	0	0	0	0
16a3b15ce2d6b81996ad380c6ba994ae	0	0	2	0	0	0	0	0	0	0	0	0
e878b552ea41700d2ade6213bbb14aaf	0	0	0	6	0	0	0	0	0	0	0	0
bf2d08164a011af076f103bb780de3c0	0	0	0	0	0	0	0	0	2	0	0	0
7f97e18bde8019381788fee5b7d495e5	0	0	0	0	0	0	0	0	7	0	0	0
d0f2de0da62b60e76806f4eb169a6bb4	0	0	0	0	28	0	0	0	0	0	0	0
6adc7bff9ab9c3076e366bd8cf0de061	0	7	0	0	0	0	0	0	0	0	0	0
8f84620f847cf348326d4a6bf93a7e96	0	0	0	0	0	0	0	0	0	0	0	6
c9863c387d6d7c9c1b115a7272495432	0	3	0	0	0	0	0	0	0	0	0	0
ffe3d07d244b0f6a224bfe432c9b4f55	5	0	0	0	0	0	0	0	0	0	0	0
414a2ebba8042b4f0e9090257fe1fd59	0	0	0	0	3	0	0	0	0	0	0	0
f5bad680359866993a5aa9dec79e087c	0	0	0	0	0	0	0	0	0	0	2	0

8e9b85329a78ecf606462fe70d06e218	0	0	0	0	0	0	8	0	0	0	0	0
cf04b009d05f63d82af38f777b2f1e46	0	0	0	0	0	0	0	0	0	0	11	0
2e698b1ac703bb6942a314fb116484f1	0	0	0	0	0	0	0	0	11	0	0	0
02bb8f8e20959adf057ebc2b1393fb50	0	0	0	0	0	0	0	0	0	0	6	0
dd453673c473d37ea9dc9742bf620c50	0	0	3	0	0	0	0	0	0	0	0	0
b8bc1b7438481d2818ed3dd588c1d583	3	0	0	0	0	0	0	0	0	0	0	0
a6e25de777ff8c73e14877b85da307a4	0	0	0	0	0	0	0	0	0	10	0	0
8256dc3dd669dca598d4a3c171fe6323	0	0	0	0	0	2	0	0	0	0	0	0
21a160793a780b376258afb76cddd01c	0	0	0	0	0	0	0	0	0	6	0	0
6019027911bd7410887aa2c7bcc65d0f	0	0	0	0	0	0	0	6	0	0	0	0
944616a195c5f10eb33b70df3d06ad44	0	0	0	0	0	0	0	0	6	0	0	0
f102bcfb693c2bb5c958e72a6404721c	0	0	0	3	0	0	0	0	0	0	0	0
8b18e2db74075c93c27c6837da4ca3a2	0	0	0	44	0	0	0	0	0	0	0	0
b87d8220333d31a8a15580a60f4042f7	3	0	0	0	0	0	0	0	0	0	0	0

191889ef10055063b85264c6477393d0	0	0	0	0	0	0	0	0	0	0	0	8
b286e59caf38e5ddfd3ad069fdceb8e8	0	0	0	2	0	0	0	0	0	0	0	0
8ee79a54d153ba7aa2f8e349bda0f6b0	0	0	0	0	0	4	0	0	0	0	0	0
9b1c88774b1f9a4c07ad89f15cc83495	0	0	0	0	0	0	0	2	0	0	0	0
7eedfc6dcb469b40c1bc88da683d9f46	0	0	0	0	0	4	0	0	0	0	0	0
f6861a94941ab950f5aa503f2bab0619	0	0	0	0	0	0	0	0	0	0	23	0
ed4725494a9c2cb77e00c0856175f14b	0	0	0	0	0	0	0	0	0	0	0	6
795847db73a93b3d57d017db6e4c75b9	0	0	0	0	7	0	0	0	0	0	0	0
366adebc3668b5500b90c3b645bcd1d9	0	0	0	0	3	0	0	0	0	0	0	0
1a7d1c06e8760306227c79335c71ffe8	2	0	0	0	0	0	0	0	0	0	0	0
03b5d2683e1ddea91f12197054809cf1	0	0	0	0	0	0	0	22	0	0	0	0
7fb76ab94c3a3843b47163c34372ddc3	0	0	0	0	0	0	0	0	7	0	0	0
d9af59a49f235bac160858a1c6a30944	0	0	0	0	0	0	48	0	0	0	0	0
47651cfb706734d1e5d7f1e9d4c49ba8	0	0	0	0	0	8	0	0	0	0	0	0

6c16711270f9d45330e81c61b954272f	0	0	0	0	0	0	0	0	0	0	15	0
3e5413f84db0ee08bde642f775e431ef	0	0	0	0	0	0	0	0	0	17	0	0
f9889318b44eee05192a56dff25fed71	0	0	0	0	39	0	0	0	0	0	0	0
5ab0d42973ddb13fed0d07e961f371bb	0	0	0	0	0	0	0	46	0	0	0	0
9351de82c4277e6253583c2b02bce1ed	0	0	0	8	0	0	0	0	0	0	0	0
74edc38f2ac46cb64126fa0d7482acec	0	0	0	0	0	0	0	0	0	0	72	0
15fdae5fbe10fd8057e31e613c2801ae	0	0	0	9	0	0	0	0	0	0	0	0
32c509392663301a862447e48b42f12b	0	0	0	0	0	0	0	0	0	0	4	0
78ca762b5b6af1d5d7e9e3a459d29d71	0	0	0	0	3	0	0	0	0	0	0	0
9e9b71bb70bb55937fe67f77efcca49a	0	0	0	50	0	0	0	0	0	0	0	0
32d8fdcb694e710fe6ee22380a6655d9	0	0	0	0	0	2	0	0	0	0	0	0
05c34be4c3e96b245d87607ca6b2c3a9	0	0	0	0	0	0	5	0	0	0	0	0
7ed226f738ad2eb458efac87b7f3719b	0	0	0	0	12	0	0	0	0	0	0	0
03aada43ba31a30a4786557745fa3863	0	0	0	0	0	0	0	49	0	0	0	57

fb78509d0be1c89a65789847738963ca	0	0	0	0	0	0	0	0	0	0	0	4
ac8f7dd5d22f76e0bb9987d0199afec7	0	0	0	0	0	0	0	0	0	0	3	0
bf616ad97a5aedc80284e63eb01a3251	0	0	0	0	0	0	0	0	0	0	0	5
2862e205a6d40fbf6a52b4737b00c925	0	0	0	0	0	0	0	0	3	0	0	0
5fc210ef3074f6b7b583a83de65f17bf	2	0	0	0	0	0	0	0	0	0	0	0
4a57a7a67a86d9b027cdc5fa41d4a688	0	0	0	0	0	0	0	0	0	0	0	2
783bf1e06dca93d82c80a9a6d16f65ba	0	0	0	0	0	0	0	0	0	2	0	0
b04c48b19fc8cde89c37cf368ddd7b26	0	0	0	0	0	0	0	0	0	0	0	6
5b60f79e4dd3ea7f37c26b5cc076cad8	0	0	62	0	0	0	0	0	0	0	678	0
1d784ba853985ae73993cfc09498dd34	0	0	0	0	0	0	0	0	0	0	0	2
66c61881ad1dc4036168e9f4567805f8	0	0	15	0	0	0	0	0	0	0	0	0
cf42d9a82fb1f472fe9087e4a50f80ef	0	0	0	2	0	0	0	0	0	0	0	0
154398c759414481ae8b05eec5e3c957	0	0	16	0	0	0	0	0	0	0	0	0
c06147a7c7b522065717c6bb33ef1503	0	0	0	2	0	0	0	0	0	0	0	0

aead02c8f6916d6228c5865c98a21c0a	0	0	0	0	0	0	0	0	0	0	5	0
15d44c545623cc6b74c3c33790cf3cb6	0	0	0	0	5	0	0	0	0	0	0	0
15a1365e4523f2a09f93b85436f6706d	0	0	0	0	3	0	0	0	0	0	0	0
d77293ec68d78f2c3a6ead61da252767	0	0	0	0	0	0	0	24	0	0	0	0
9bf7e93cbd6967c5f38c578029b5d79b	0	0	4	0	0	0	0	0	0	0	0	0
6a29ae621f9a851591a42a070c37e89c	0	0	0	0	0	0	0	0	0	2	0	0
a246dab5a8ad611cbc7791140cd13e5e	0	0	0	0	0	2	0	0	0	0	0	0
d4ff4fdddaffd4e6223dedc80c1d8d2d	0	0	0	0	0	2	0	0	0	0	0	0
f747dde532b48fe7da5db87e56492ab2	0	0	0	0	0	0	0	0	4	0	0	0
2f6d709e497d1ea7557e001951199747	0	0	0	0	0	0	30	0	0	0	0	0
60b44c0487df4124d271028d7c5442ac	0	0	0	0	0	0	0	0	0	0	4	0
d244a9e7d350864e057551c5e0a0c4b6	0	0	0	0	0	13	0	0	0	0	0	0
ab5df144112313ba98f3b0d94eedc220	0	0	6	0	0	0	0	0	0	0	0	0
e5496cb853f3e42e053debea7258dfad	0	0	0	0	2	0	0	0	0	0	0	0

6dab8eef33dd140393e95ee3f62f1bd6	0	0	0	0	0	0	0	0	0	0	3	0
7b851667e747dad8db0ccc49ced2216b	0	0	0	0	2	0	0	0	0	0	0	0
c24ee394984c335580e4353bb6473698	0	0	0	0	0	0	0	0	0	0	8	0
edf2337d91a1c7d6d01a5514ff23095c	0	0	6	0	0	0	0	0	0	0	0	0
b9581153ef04e0ca020414d75c423e56	0	0	0	0	0	0	0	0	0	0	0	6
ab6e1ff071a0f8ddac08621dab9b89ff	0	0	0	0	0	0	0	0	0	4	0	0
d47055b976a2fed757f0d07bbf475126	0	0	0	0	0	0	0	0	0	0	0	4
c0fae55bb3a4ea7c9c857d3b0270fb76	0	0	0	0	16	0	0	0	0	0	0	0
7649e735f673e614a7a1a2828e3e62a4	0	0	0	0	0	0	0	0	0	0	6	0
3e58c4bb633abb7e034caf3251e3a04e	0	0	0	0	0	0	0	0	0	0	0	4
dc20b85ec07b766028bc1b5b80b73d55	5	0	0	0	0	0	0	0	0	0	0	0
7cb7eb28b12f8b3299def0c6d3100b74	0	0	0	0	0	0	0	0	0	0	2	0
4ed564b91dec90f34e2be43c9d73f814	24	0	0	0	0	0	0	0	0	0	0	0
48f29cd562144bf1b53d650c34c86d13	0	0	0	0	0	0	0	0	0	0	0	2

275ec986242be9be0cd6ac25906925bf	0	0	0	28	0	0	0	0	0	0	0	0
9b300a5887ff4e4c0b700668d2767370	0	0	0	10	0	0	0	0	0	0	0	0
097c82bcb3cbb4c969692a14474b0be3	0	0	0	0	0	0	0	0	0	9	0	0
ecedec7f423539c5f016f75980472667	0	0	0	0	0	3	0	0	0	0	0	0
13b9534b34ea370eeeab74feca770b27	0	0	0	0	0	0	0	0	0	0	10	0
1d644fc79d3537e383326b88ec26ad78	0	0	0	0	3	0	0	0	0	0	0	0
9e826f53b9b45dcd422b22791f80af25	226	0	0	0	0	0	0	0	0	0	61	0
c3a34acf9202beb978b06c91a4f70a07	0	0	0	0	0	25	0	0	0	0	0	0
79a4146e0c1d6133bb9a04176f86597b	0	0	0	8	0	0	0	0	0	0	0	0
b72957787b2f1bf1139d1a1e95340079	0	0	0	0	0	5	0	0	0	0	0	0
b963064224aa7d67b9490b1ed34ef058	0	0	0	0	0	0	0	2	0	0	0	0
bf43b4881f7adbb226d2b5607e93760c	0	0	0	0	0	0	0	0	0	9	0	0
fba96c3ebf0d1b76c14cd89f520425e7	0	0	0	0	0	0	0	0	0	2	0	0
844e3db14635163cb1841a8085b8fde9	0	0	0	12	0	0	0	0	0	0	0	0

a3baa6e8ee8421a10bb30d09ce9ae1a9	0	0	0	0	0	0	0	0	0	0	0	7
1caa6740dfeb4e44bfca86cd62a0dad5	0	0	0	0	0	0	0	0	11	0	0	0
38416f1a9250b62aaf75012a500cfbdd	0	4	0	0	0	0	0	0	0	0	0	0
1fc45e6770e9466221bf86466a152678	0	0	0	0	0	0	0	0	0	0	9	0
9e5b06cc1e8003645f997bab54e23fe3	0	0	0	0	0	0	0	8	0	0	0	0
3532667ad766b800b88be11919d323da	0	0	0	0	0	10	0	0	0	0	0	0
0803ed6f960fff5a0e607350caa12e85	0	0	0	0	21	0	0	0	0	0	0	0
d790a7061330c972e77164d31e3fa985	7	0	0	0	0	0	0	0	0	0	0	0
95e8dad76c5de482f4900ae621b7033e	0	0	0	4	0	0	0	0	0	0	0	0
d82ddcfe30ac6af583c9fbc36680a37a	0	0	0	0	2	0	0	0	0	0	0	0
66db3ac9ceaf08e1a05671f151717fa5	0	0	0	0	0	0	0	20	0	0	0	0
7d3b1c684c6cf41ad0109996802711ee	0	0	0	0	0	68	0	0	0	0	0	0
ee10fac6a1b8c98b04ac9753b94f2314	0	0	0	0	27	0	0	0	0	0	0	0
83cfe59929440dcadca851e73cc54351	0	0	0	0	0	0	0	0	4	0	0	0

e9333cd6d7a0d1491a6eb45772abe67d	0	0	0	0	0	0	0	0	0	0	64	0
87ceb0ce3e6d5783d9a0f916759c53a9	0	0	2	0	0	0	0	0	0	0	0	0
b9957c554e3797bc1fc9c73e665a2328	0	0	0	0	0	0	0	0	0	0	0	4
c387ea687833ab03b5c326b84ec33f11	0	0	0	0	0	0	0	32	0	0	0	0
a541a8542f7144d8cff82038bd6f8740	0	0	0	0	0	0	0	0	0	0	54	0
b31aa07e887e107f73cb51f07d1398f5	0	0	0	0	0	0	0	0	0	0	18	0
5b2aaeeb6684ae70756e03400c93b5cd	0	0	0	0	0	6	0	0	0	0	0	0
9866914ad86629e23f6823e3ee39b1ca	0	8	0	0	0	0	0	0	0	0	0	0
d7365b5599a0731e541bfd27c7de6012	0	0	0	0	0	0	0	0	0	0	5	0
a8fbb5bd327e9798b8a06de1813edaeb	0	0	0	0	0	0	0	0	0	0	67	0
dc1ad2619ed4b8073cea974d05867c4f	0	0	0	0	0	3	0	0	0	0	0	0
98d10eca0b883327fa80389340bd2b6f	0	0	0	0	0	8	0	0	0	0	0	0
06576ab04532ef33f1fd37dbdefc9e91	0	0	0	0	0	0	0	0	3	0	0	0
775b3203c341b42d08368ed61eafb62e	0	0	0	13	0	0	0	0	0	0	0	0

31077c22b77c08d77a918be42f92a080	0	0	0	0	4	0	0	0	0	0	0	0
fc220a3793e994cdb4ce143d571cdb81	0	5	0	0	0	0	0	0	0	0	0	0
3a5eaaa82a07ae1a1dea1b9eef4606e0	0	0	2	0	0	0	0	0	0	0	0	0
780663ee0701b7ab76d8b4839fdb099c	0	36	0	0	0	0	0	0	0	0	0	0
cf62093c1f2ff74ec4ac5938a03507f1	0	0	0	53	0	0	0	0	0	0	0	0
83874b598dcaa5934a602e9f761eed63	0	0	0	0	0	0	0	0	0	0	10	0
75a1e0fe70a1769d2c5b3d7482a0a6c1	0	0	0	2	0	0	0	0	0	0	0	0
09fcd38aaa571147a2e94f0368d59253	0	0	0	0	0	0	0	0	0	8	0	0
15bd6c2b00f4caa40b96b6e8bd7d660d	0	0	0	0	0	0	0	0	0	0	0	26
9f088e9d8f0b620d266ee61405c17b34	0	0	0	0	0	0	0	5	0	0	0	0
f02e61bb524acf231b5fdf09da12b7ec	0	0	0	4	0	0	0	0	0	0	0	0
daa01a2c5293ca0a6bb1c9ca4d659db4	0	0	0	0	3	0	0	0	0	0	0	0
b06011f857cc173ae60512ddf5ededf2	0	0	0	0	0	0	0	0	106	0	0	0
299409711f23019b80aabaaedfe7c093	0	0	0	0	0	0	0	2	0	0	0	0

8422daaf1d7f4ad24bee071d6fc26842	0	19	0	0	0	0	0	0	0	0	0	0
f367ef6488eb6a9b9a02f2eb7c7e2b80	0	0	0	0	0	4	0	0	0	0	0	0
7accc433bb529f269b0f35c15c930a58	2	0	0	0	0	0	0	0	0	0	0	0
abdea60194e55267e16344d4022e8fd0	0	0	0	0	0	2	0	0	0	0	0	0
0e0c880358395ef26899e98554fcaa8a	0	0	0	0	11	0	0	0	0	0	0	0
d66ae564da0e8c2b79efd387b222b379	0	0	0	0	0	0	0	0	0	0	3	0
b7eca94cbaa4e6c94c4b6d0614606463	0	0	0	0	0	0	0	0	0	0	22	0
df27991760a58055acbca7064bc5add4	0	0	0	0	0	0	0	2	0	0	0	0
62d669a6dbc7e432887a93148b723ab4	0	0	0	0	0	0	0	0	2	0	0	0
1d677ae81df39b164489be3b453bd94a	0	0	0	0	0	2	0	0	0	0	0	0
45f86aa53428d206af245df5009f6c7b	0	0	0	0	0	3	0	0	0	0	0	0
dba81d7679b67e1b67698d9f7e5adacb	0	0	0	0	0	0	0	0	0	2	0	0
53f5476cde65dd8f1e17c139a135476f	0	0	0	0	2	0	0	0	0	0	0	0
c552f880d3ef1775617f51de38ab7571	0	0	23	0	0	0	0	0	0	0	0	0

e9fd9b8a9f89d830a8caf5d258bc9b63	0	0	0	0	0	0	0	0	0	0	17	0
5349336784f977fa0ac90570a9db91b9	0	0	0	0	0	0	0	0	0	4	0	0
09b37f850f704c9a7478525aec64bd64	0	0	36	0	0	0	0	0	0	0	0	0
af3cb25b7f0e71086b6585cd667b6b44	0	0	0	0	0	0	0	0	0	0	6	0
bea13d3bb7dcba912d50f49f3045bc92	0	0	0	0	0	0	0	0	0	2	0	0
84c766881aa54154e919bf313607e691	0	0	0	0	0	0	0	0	0	0	10	0
e5fa03e44d3ecfd9b734dd7b02274957	0	0	0	0	0	0	0	0	0	0	0	2
f677f52f91cf2b89c05052d98845aa03	0	0	0	0	0	0	0	0	0	0	11	0
64c2815ba5ace7e2e1417bb4965cbb59	0	0	0	0	0	0	0	0	0	0	0	2
f996084e3252e24a56a63cd58be75b2a	0	0	0	0	0	0	0	4	0	0	0	0
39c20863984f4baf2afdab7d7ca2ef4c	3	0	0	0	0	0	0	0	0	0	0	0
51ee7ac91c1cbb25752ebc7a223adbf6	0	0	0	4	0	0	0	0	0	0	0	0
c8baf0a525139a0394932c4467fadd33	0	0	133	0	0	0	63	0	77	0	0	0
847a995bdabb285bc5c27bef77f98cf3	0	0	0	0	0	0	0	19	0	0	0	0

bdafa92da950df5991487cb3a7d58924	3	0	0	0	0	0	0	0	0	0	0	0
2070fbbac8c376668d265f02a52d8800	0	0	0	0	0	0	0	0	0	0	7	0
4d4f8b3bcca59e9ca3968e5618b30a38	0	0	0	0	0	0	0	0	0	0	0	21
f4640e576971a3e56490ae1c9213436a	0	0	0	0	0	0	0	0	0	0	0	20
c4da249b6898ae89160db87497efce2c	0	0	0	0	0	0	0	0	2	0	0	0
214639b0dcfdb70b53cc40ca11d721d6	0	0	0	0	0	0	0	0	0	0	50	0
d1ce08ec54265d2d3db3951a2250d656	0	0	0	0	0	0	59	0	0	0	0	0
47b30ff61c1600c398954d4e2b6addd9	0	0	0	0	0	0	0	0	0	0	2	0
c292af38440efe4ac11e0807b12864a3	0	0	0	0	0	0	0	0	3	0	0	0
33ef0b853bf0bafd79f9a6b7baba158b	0	0	0	0	0	0	0	0	0	0	11	0
ba3238dcbc809dc856b434dfd4442538	0	0	0	0	0	0	0	0	0	0	4	0
0f6b84b5566fa2b25e7169cb73d9ece8	0	0	0	0	0	0	0	0	0	0	0	2
84a3de7a04c475ae1fa556d169927019	0	0	0	0	0	0	0	0	0	0	3	0
4978e57c3ff7d020457070c2bb468918	0	0	0	0	0	0	0	0	7	0	0	0

f2f68b1194ba9a0d6187cf77ec6fcd95	0	0	7	0	0	0	0	0	0	0	0	0
ba9a844c27d69d7d42402f1cdbe2142d	0	0	0	71	0	0	0	0	0	0	0	0
ce0d89b2736d2b59edb1c245adbc21ce	0	0	0	0	0	2	0	0	0	0	0	0
5f5a5c999dd8923f87021750dbb1db2f	0	0	0	0	0	0	0	2	0	0	0	0
59880e4631ec9269a34f2935700c9f8f	0	0	0	0	0	0	0	0	2	0	0	0
09eb7a5c81926097ba34bc33f38bd3cc	0	0	0	0	0	0	0	0	0	0	2	0
331705a5f11099bc383ff9aee9980708	0	0	0	0	132	0	72	0	92	119	0	0
99a9363d7fa7ccec78bed3ee3c523a18	0	0	0	15	0	0	0	0	0	0	0	0
488bcdaeab90fb227a2cb8c0cca0805e	0	0	0	0	0	0	0	0	15	0	0	0
17b504ed6aa7fe5317315e022e94ac94	0	0	0	0	0	7	0	0	0	0	0	0
6637c38e673e387bae879cbbad92e88e	0	0	0	0	13	0	0	0	0	0	0	0
b344145b0732b8c077d7d8f0f3daa91b	0	0	0	0	0	3	0	0	0	0	0	0
0eb3395143af5a366786b966bb0c2d13	0	0	0	0	0	0	0	0	0	0	0	9
ae50a1eba4b785f08f8e2d94a7a5de7b	0	0	0	0	0	0	0	3	0	0	0	0

03195b58fba5288205e23432b9275eac	0	0	0	0	0	0	8	0	0	0	0	0
c25ff5035bcbe14fe1cf617f5bea100a	0	0	0	0	0	0	0	0	0	0	5	0
c5d0ca327f3e94cbbec2a7e1d2c8ff97	0	0	6	0	0	0	0	0	0	0	0	0
c306cbc813abbb0597f3a5610e394997	0	0	0	0	0	0	0	0	3	0	0	0
b473df7629470335c46fc90aad80cb7b	0	144	0	0	0	0	0	0	0	0	0	0
63a4ef749fd7f9dea5ca4a629330d211	0	0	0	0	0	6	0	0	0	0	0	0
f3618654f916781e8348143a2bc0a359	0	21	0	0	0	0	0	0	0	0	0	0
18909a5d8f4cc2ad81051d02aea26654	0	0	0	0	3	0	0	0	0	0	0	0
527f4b65ed68cb59d12d139aebefc928	0	0	5	0	0	0	0	0	0	0	0	0
c2b0bc0b5e8be44ec4e7e13c397fae71	0	0	0	0	0	0	0	0	0	0	3	0
72c93bcd764443ea2248deb0378f36b4	0	0	0	0	63	0	0	0	0	0	0	0
88dc64112b95344fe8ee663ee866fc93	0	0	0	0	0	0	0	0	0	0	3	0
b1635db58fe34d20e5d2dd7891668801	0	0	0	0	41	0	0	0	0	0	0	0
b79aa25e5bba8ae64848c89be2cb1715	0	0	0	0	0	0	0	9	0	0	0	0

ba44342f91c0032c15783746538f5f97	0	0	0	0	0	0	0	0	0	2	0	0
9f8e86675954f7200bd98d4c21bf9b1c	0	0	0	10	0	0	0	0	0	0	0	0
4a663eb3f774763a55944f7d1f1fced3	0	0	0	0	0	0	0	3	0	0	0	0
a96910fb2c87104f52d4e0f8adbb9a12	0	0	0	0	2	0	0	0	0	0	0	0
131cad73cffe2795d0120eca8aa50b78	0	0	0	0	0	0	0	0	5	0	0	0
e47e73d051b9f3642162a296bdf2b6f0	0	0	0	0	0	28	0	0	0	0	0	0
7d62a531c117d3628565029f28e98e5e	0	0	0	0	0	0	0	0	27	0	0	0
4e296285059db4e1429507061497f6b5	0	0	24	0	0	0	0	0	0	0	0	0
479f3e4c61ad45a96d7afdd0555a035e	0	0	0	0	0	0	0	0	0	0	3	0
1877ec59b224a21e1176af2a7317778f	0	0	0	0	0	0	0	0	0	0	0	4
11f19c45365dafcd8c964206a4b8d537	0	0	0	0	0	0	0	0	0	6	0	0
89a758af45a7d41e7828b550910d9eaf	0	6	0	0	0	0	0	0	0	0	0	0
4f9b989649c85d72cde2f486afcf41c2	0	0	0	0	0	0	0	0	0	0	0	10
316260b245cb1d5867355ca93811b0c7	0	0	0	0	0	0	2	0	0	0	0	0

8a2dd31af84769cf84ff546e67db6733	0	0	0	0	0	9	0	0	0	0	0	0
f202fbc8614ceb2c7661827eaf62a76f	0	0	0	0	0	0	0	0	0	0	0	2
5fba4e8e6ad08779ae9de3f81f8b346c	2	0	0	0	0	0	0	0	0	0	0	0
138f4d7a648306ee1066d18dd91d559f	0	0	5	0	0	0	0	0	0	0	0	0
e7077482973c6c37ba5c9f76e70fab51	0	0	0	0	0	8	0	0	0	0	0	0
d3104f49c2094e6495fed19fb00a27e9	3	0	0	0	0	0	0	0	0	0	0	0
0dae8f18b63c59c2c52286931c2a7215	0	0	0	0	0	0	0	0	0	0	0	11
f999c5b7b6156df9eb2c1e8648df8a26	0	0	0	224	0	133	0	0	0	186	80	0
d89a2a0c304c65c83b9ba9be686cc2d1	0	0	0	0	0	4	0	0	0	0	0	0
2355a46985662b7a7e56440adeec628a	0	0	0	0	0	0	0	0	0	0	0	17
c6586d475a6e30a65016bff14ca87962	0	0	0	0	0	0	0	0	0	5	0	0
30d80a558f9e0da96c5e1a2618840915	0	0	0	0	6	0	0	0	0	0	0	0
c4fc6ab616e422710512982c53952661	0	0	0	0	0	0	0	0	0	0	0	3
3835311823953cd5f3e7c8f279922a07	0	0	0	0	0	0	0	0	0	0	0	12

8478715f404efc45932d9c4df af1f9c9	0	0	2	0	0	0	0	0	0	0	0	0
a7aaf84f7270941c0d011f523 c96f3c8	0	0	11	0	0	0	0	0	0	0	0	0
a9ee9ca615eb02e2bb4927fc 878edd98	0	0	0	0	0	0	0	0	19	0	0	0
3029048804ea9eb81900701 31c112220	0	0	0	0	0	0	0	0	2	0	0	0
321f0d6b5012f0603506203d 4f4a9a24	0	0	0	0	0	0	0	0	48	0	0	0
061bf9c67c698d12eb8f1dad 12dbfa95	0	0	0	0	0	11	0	0	0	0	0	0
a50f773ad9f00add9aebe0d9 36e424a2	0	0	0	6	0	0	0	0	0	0	0	0
92e5341f5c54ee0e39bb0ea8 53b2fba1	0	0	12	0	0	0	0	0	0	0	0	0
fedb4850c1312c3791aeca68 173005a1	0	0	2	0	0	0	0	0	0	0	0	0
e52c46c1ebd8c339927a6eb5 83b70850	0	0	0	0	0	0	0	0	0	0	4	0
9488185cf56acba445e971c3 108bba91	0	0	0	10	0	0	0	0	0	0	0	0
0b9627a0b8ca764b0065f052 77dbeed1	0	0	0	0	0	0	0	0	0	0	0	2
02aa99fa978e3217a31eba39 f42052a6	0	0	0	0	0	0	0	0	0	36	0	0
6ac7c22cbe7774c14c071548 278475df	0	0	0	0	0	33	0	0	0	0	0	0

bfa0c8857a5f740e58b12989cabfdc21	0	0	0	0	0	0	0	0	0	0	346	0
e9451923d87d8812c975c13da519ca6a	0	0	0	6	0	0	0	0	0	0	0	0
1faadb9070bfc6d063660ae9cf5dee8a	175	176	0	1196	182	389	0	707	0	395	0	470
ef9a8f2a8378c7d4b691bd88a0fb467d	0	0	0	0	0	6	0	0	0	0	0	0
cd7be18906bc26b0ded757dc57f94d31	0	0	0	0	0	0	0	0	0	5	0	0
dd9d8db5ebacafa0e172758cdffb146e	0	4	0	0	0	0	0	0	0	0	0	0
9389c667b144c8159e46cc5fc8487202	0	0	0	3	0	0	0	0	0	0	0	0
2bb3493ffb1c7c41b7672fc6d941d73d	0	50	0	0	0	0	0	0	0	0	0	0
0a0ba9832ff2db7cdc22c2b1fd58a6c6	0	2	0	0	0	0	0	0	0	0	0	0
d7b092537293f33d9cbc8ad07a221085	4	0	0	0	0	0	0	0	0	0	0	0
14c78471734b7837809ca27c8c2b1e34	0	0	4	0	0	0	0	0	0	0	0	0
79624bbaeb51acbeb4bebff5645bd23d	0	0	0	0	0	0	2	0	0	0	0	0
b6ebc63aaf003e4a6df8b0ca31b0704e	0	0	0	0	0	0	0	0	0	3	0	0
fe567942bee4d07afdd45960678a097a	0	0	0	0	0	0	0	0	0	8	0	0

272cdd2530c7467c2fea5080 18dba081	0	12	0	0	0	0	0	0	0	0	0	0
bd92dea0d6ab8834f408a9c2 f0af0c43	0	0	0	0	2	0	0	0	0	0	0	0
ce7909165ba5913192ab962 61b004aee	0	0	0	4	0	0	0	0	0	0	0	0
6593a332bddc10cdb0d0472 bdb18c402	0	0	0	0	0	0	3	0	0	0	0	0
1e597e675932d907dff52157 fcfecd9f	0	0	0	0	5	0	0	0	0	0	0	0
6fff1483f08b2c7c3cf395bd2f bdfcf2	0	0	11	0	0	0	0	0	0	0	0	0
1326a4dae92b32fc47aac71d b3cffc28	0	0	0	0	0	0	0	0	0	0	4	0
cea9fe7475602da637b186a2 294a06d1	0	0	0	2	0	0	0	0	0	0	0	0
2d7aa3bc6d1f15c139a49c55 8f8bd650	0	0	0	0	0	0	0	0	0	0	0	3
e6cad6cba63d4c02216f9b6d a8d1fde9	0	0	0	0	0	0	0	0	0	0	6	0
ea25bb942c2f5e689b3193f5 1cdf4c0d	0	0	0	0	0	0	0	0	43	0	0	0
f9879147e0a8ff2ff796a2acc0 480845	0	0	0	0	0	0	0	0	0	0	48	0
4d94ab0f43efa62453563860 4a62afc5	0	0	0	0	0	0	0	26	0	0	0	0
79b03b3f2836454ed358e7c8 95253852	0	0	0	0	0	0	0	0	0	0	2	0

f2fde4b9c8743280f664dadd8331e63c	0	0	7	0	0	0	0	0	0	0	0	0
b779d60bd839a3097c7cd6f2994812cf	0	0	39	0	0	0	0	0	0	0	0	0
4fe28d453ea8c250af1751637be88360	0	0	0	16	0	0	0	0	0	0	0	0
dd57f290495982aa415e30e2d299058d	0	0	0	0	0	2	0	0	0	0	0	0
d267c124775fd22efe65587308902bb6	27	0	0	0	0	0	0	0	0	0	0	0
c43da6389b596f604ee42b07995ca987	8	0	0	0	0	0	0	0	0	0	0	0
49babb0487561d1ac81b3b2e307e9132	0	0	0	13	0	0	0	0	0	0	0	0
24bd501aa9882f902b32114d0f09f4af	0	0	0	0	0	0	5	0	0	0	0	0
ecad61e27cc4e2966468ce62ff5768ee	0	0	0	0	0	0	0	6	0	0	0	0
12ebddd3357f98c22c6d64b37e3d27fe	0	0	0	0	0	0	0	0	0	0	4	0
129d0a7e589c8f14d9f9d473103ef5c8	0	0	0	0	0	0	0	0	42	0	0	0
68f9f0822c44b7e0d2913ff41e5b9e89	0	0	0	0	0	0	0	0	0	0	3	0
b8e1b81d226a0af1e9dcba213d16ae49	0	0	0	0	0	0	0	0	0	3	0	0
8b8219de40dd979b59764cf366fde1be	0	0	0	0	0	0	3	0	0	0	0	0

a5ac5d10cda18cd012cff0143615a8f1	0	0	0	0	0	0	0	0	0	0	0	3
7e8cbaf0b90cb3f19eb54a714e6dab18	0	0	0	0	42	0	0	0	0	0	0	0
30725b6a55f59857f934316aa582b3c5	0	0	5	0	0	0	0	0	0	0	0	0
0c6f579265dc40ac0be1ce43e2daa0af	0	0	0	0	0	0	2	0	0	0	0	0
55b46da2f16420e5fcd78477dd85fb85	0	0	0	0	0	0	0	2	0	0	0	0
ebf5597995ead444b62ae579e28c4bac	0	0	0	0	0	0	2	0	0	0	0	0
b9eac19240c967e7143aad0a7ea5ed89	0	0	0	0	0	10	0	0	0	0	0	0
c9d5b64d10aef4e50f8116fa0b0e2276	0	0	0	2	0	0	0	0	0	0	0	0
2b23955c096bb8b577e0d55624d52b2b	0	0	13	0	0	0	0	0	0	0	0	0
903d3750f9c5279a0f0944b10e95b5dd	0	0	0	0	0	6	0	0	0	0	0	0
3e6389b5553b25df51545f123ff0f74b	0	0	0	81	0	0	0	0	0	0	0	0
78356a266a9a7db1366866755eb7e0c0	0	0	0	0	0	3	0	0	0	0	0	0
6e63b607b4fd9f15b49fe0d43a5e058a	0	0	0	4	0	0	0	0	0	0	0	0
925c0ae1e85e78c52ee9863f660015a4	0	0	0	16	0	0	0	0	0	0	0	0

3328e1b54556546d76760c095da588d4	0	0	0	0	0	0	0	0	0	0	3	0
fe00d828add755a6113520cd757e820f	0	0	0	0	0	0	0	0	0	0	4	0
f4d99aef6779a3475b8b12e5c64d5e07	0	0	7	0	0	0	0	0	0	0	0	0
b16e27251de30dee2ad0049489491a18	0	15	0	0	0	0	0	0	0	0	0	0
77bb720aa3c960b090dd3014ab08fd30	0	0	0	0	0	0	0	0	0	0	2	0
c51e920b0e80e2ce547f3aa3ac9efd91	0	0	0	3	0	0	0	0	0	0	0	0
1bbc4d076f6ab9d30c1b9471fb6182d2	0	0	0	0	0	0	0	0	0	0	7	0
d717ad536efa7dda01033435b7b436da	0	0	0	150	0	0	0	0	0	0	0	0
337324a414f61263392edac5d670b71a	0	0	0	0	0	0	2	0	0	0	0	0
3327749e2485a7e35d60c64264f4589c	0	0	15	0	0	0	0	0	0	0	0	0
bbcafeb9bfcd975578efc898b0c7cbe6	0	0	0	0	0	0	0	0	0	4	0	0
7e43984da5be968c5fe4fd2e8b315d8d	0	0	0	0	0	0	0	0	0	0	0	7
893b3ba60798fd80d2ee01bad19e4677	0	0	0	0	0	0	0	0	0	0	0	11
6cbb67ab654e3f6fc5ac4755049f3a83	0	0	0	0	0	0	0	0	30	0	0	0

fc67a0a7cf212d61df0fd4ec8d0082a3	0	0	18	0	0	0	0	0	0	0	0	0
681746b3f4506d9fba3855379d501c35	0	0	0	0	0	0	0	0	0	0	0	39
2fd7573be055f1d3688d1e509f471b04	0	0	0	0	0	0	0	0	0	0	0	3
ec970e4e3046fd816221f505079400aa	2	0	0	0	0	0	0	0	0	0	0	0
2234b69ad8d8c238513d1c79e5b1efc2	0	0	0	0	0	0	0	0	0	3	0	0
a0ca34b45b0fd0aaee102036de634f93	0	0	0	0	0	5	0	0	0	0	0	0
861642df2365e0f48abd32ea9b38e42d	0	0	0	0	0	0	0	6	0	0	0	0
738dc99ca0e4bea5f9e4420424a850be	0	212	0	0	164	0	0	0	0	0	0	0
db7bc3d03279c6644ec513d47036f0c1	0	0	0	10	0	0	0	0	0	0	0	0
4514c7fda893e615e2944f5979fd807b	0	0	0	3	0	0	0	0	0	0	0	0
2beb21f751c77ae8066a9188ed88a494	0	0	0	0	0	3	0	0	0	0	0	0
3d17bf0bfe1c2803031a16608db6d849	0	0	0	0	0	2	0	0	0	0	0	0
5622386806c0b47c30ae80aa39a04e45	0	0	0	0	0	0	0	4	0	0	0	0
501af292d0c39964ba369b851df20523	14	0	0	0	0	0	0	0	0	0	0	0

e1579499ab304547c1a6e87 50998e3c0	0	17	0	0	0	0	0	0	0	0	0	0
2554e9550596192410ee83b e8557acb6	18	0	0	0	0	0	0	0	0	0	0	0
339940607b5c2c392b93cfa2 798919ad	0	0	90	0	0	0	0	0	0	0	0	0
69a06c4efb277e9aefe0da67 29ff02dd	0	0	0	0	0	0	0	0	0	0	43	0
e334fb52c999b74315e8de67 93864087	0	0	0	0	0	0	0	0	0	0	0	3
43d6d2894ddd6eb22ee8041 604bc0f15	0	0	0	5	0	0	0	0	0	0	0	0
4fe47bcb5ed099f8fd6572fdd ea97a13	0	0	0	0	0	0	0	0	0	0	0	12
58a2a457ac01dce0f4370f60 7cdc28b1	0	0	0	0	0	3	0	0	0	0	0	0
1eab54937f11dc0918f309c2 2b37497d	0	0	0	0	0	0	0	0	0	0	0	2
74c0e03d7cdbf3c2df5d2014 a86692e6	0	0	0	0	0	0	0	0	0	0	4	0
ed52ce931f564a08dbd48317 dd369c43	0	0	2	0	0	0	0	0	0	0	0	0
c05f6495477578adfd0aa65b 1e0bb910	0	0	0	0	0	0	0	0	19	0	0	0
de7dee440bbfeb5550fb7980 ed4d8419	0	0	0	0	0	2	0	0	0	0	0	0
7aa7fe406cb91dbed0e7cd61 91485d2d	0	0	0	0	0	0	0	0	5	0	0	0

632bf58fc12405e8077454b3cbb7b947	0	0	0	3	0	0	0	0	0	0	0	0
1ad0ef57cee68efbb5178e60abdffc09	0	0	0	0	0	0	0	0	0	70	0	0
c3fd69cad633dd5ff323f3b535a6781e	0	0	0	0	0	0	0	0	0	0	0	5
581fe8bb12d080461f232b8a31e40760	0	0	0	0	0	0	0	0	0	23	0	0
e79c52fefb7131f56eb8d9ad9a66fd8e	0	0	0	0	0	0	2	0	0	0	0	0
04011e59a401a55dcaeac19be710d2d3	0	0	5	0	0	0	0	0	0	0	0	0
c8fcd88c5dfebd2bbc2832091dd853f8	0	0	0	0	0	4	0	0	0	0	0	0
e09a0eeab030df8b39f2491dc9b694d0	0	0	0	0	0	0	0	4	0	0	0	0
adeb60503d4c2fbeae108c154bc346a8	0	0	0	0	0	0	0	0	0	0	0	2
4e19d7ac32c823efde67fe75c91a8709	0	0	0	0	0	0	5	0	0	0	0	0
431fd9b409ea0e97cc8dc880ec2480d6	0	115	0	0	0	0	0	164	0	265	0	0
cca2057179369a291b3f1c604198a69a	0	0	2	0	0	0	0	0	0	0	0	0
060bd6d2b030a6a7ee76d66b8b9954ee	0	0	0	0	0	0	0	0	0	0	2	0
114f92ff6a855ab7207b4bd1d6d53ac4	0	78	0	0	0	0	0	0	0	0	0	0

a9682beab01fb978f635e70a10645535	0	0	0	0	0	2	0	0	0	0	0	0
fddbb24adedea91cbf0ad4ec76c0b30a	0	0	21	0	0	0	0	0	0	0	0	0
a3692344b9a74f10caa148915acbfd19	0	0	0	0	0	0	0	0	0	4	0	0
20b82bf6ffe24935c34bd5d95d0cec15	0	0	0	0	5	0	0	0	0	0	0	0
afd94522e2f1d98deae658318f50c7a2	0	0	0	0	0	0	2	0	0	0	0	0
77d548ea1aab42943011937b79b21dbb	0	0	0	0	0	0	0	0	0	4	0	0
8064cee5187c488eaa4774058e96a86e	0	2	0	0	0	0	0	0	0	0	0	0
17f5b0904233aa8ee8fba1a13f535c1d	0	0	0	0	0	18	0	0	0	0	0	0
ee41bbf0d056051b514d0d480c3344d7	0	0	0	0	0	0	0	0	0	0	2	0
3f5f90747da78bd89d0d845294d26c6d	0	0	0	0	0	0	3	0	0	0	0	0
1e0a5b9f2ee36ddf374081ca5d5d4481	0	0	0	0	0	2	0	0	0	0	0	0
3139e9726d798899d0103cb71d71ccbc	3	0	0	0	0	0	0	0	0	0	0	0
df2011a43050565fda89dbdb546ddffb	0	0	2	0	0	0	0	0	0	0	0	0
7a08073b1a3fb7e85dcbfd91f2e13d8c	0	0	0	0	0	0	0	0	0	0	3	0

09d81b5cd5d150fd57f6a849afebc9ef	0	0	0	0	0	0	0	0	0	0	5	0
b4455eb2846170d2e8ba9267d8949f8f	0	0	0	0	0	0	0	0	7	0	0	0
84a1cd5a30aa5e76768275fbf78ac4fa	0	0	0	0	0	0	0	16	0	0	0	0
32aa3a01495aa8bdd353500cfa157364	0	0	0	0	0	0	0	0	2	0	0	0
9ba6922da8e11a4fdf84872558ab7aec	0	0	0	0	0	0	0	0	0	0	15	0
f40ad94462043f31d282d39b3dbe3898	0	5	0	0	0	0	0	0	0	0	0	0
8baf3c9bbe6fa17f3ae738d2648a81cb	0	0	0	0	0	0	0	0	2	0	0	0
85b4cb6f08c97dacc93d7f966cb87179	0	0	2	0	0	0	0	0	0	0	0	0
2480fba006fb83da2d2790aadf79211a	0	0	0	0	0	4	0	0	0	0	0	0
04bd41539fe41757c886f5ab4168a84b	0	0	0	0	0	0	0	0	3	0	0	0
b12a0baea93080c4dd4e5c6465ace01d	0	5	0	0	0	0	0	0	0	0	0	0
2e5354765da21b8637625847736239c9	0	0	0	0	0	0	0	0	0	0	4	0
1c61c8ddc8e660eb2d90c7d75d6dec11	0	0	0	0	0	0	0	0	2	0	0	0
df9821f806c779dc7cdce86c2596afba	0	0	0	0	0	0	0	0	2	0	0	0

06d0b6924955c1ff6afa81bc3ce93986	0	0	0	0	0	2	0	0	0	0	0	0
3d9eeab228cde3fbbf2a06bc013c58c8	0	0	0	0	0	0	0	0	0	0	9	0
0450d8c91364b1083efbf881ac5dfa86	0	0	0	0	0	0	0	0	0	2	0	0
f56f7484469c901d906e3383406d52c9	0	0	0	0	0	2	0	0	0	0	0	0
2b61c859ba1d29c1ce62d11d9e16dd1e	0	0	0	0	0	0	0	0	0	22	0	0
b67827beab6b228e22f4e6cc7d041061	0	0	0	0	0	0	0	0	0	0	0	23
012682d1d37033812d6770794be90c76	0	0	0	2	0	0	0	0	0	0	0	0
e3d79aa82e81eaa40890517cbec48eca	0	0	0	0	0	0	0	10	0	0	0	0
453521dcb7b2cc515ed029fdf6e532e4	0	0	0	0	0	0	0	0	0	0	0	35
a87f0f907dbfeec51863972487861c15	0	0	0	0	0	0	0	0	0	0	5	0
bb8bb12dfd751ad25f2ca24013fd136c	0	0	0	0	0	0	0	0	0	5	0	0
24263681d2afe3a58744a0da6e07ce92	0	0	0	0	0	0	0	0	0	12	0	0
8bfedc285117f368443144ca4bd91ef3	0	0	0	0	0	0	0	0	0	0	0	14
49f36e637a23b22a92f50b3f63ec6e4b	0	0	0	0	0	0	0	0	0	0	12	0

a19518b9a1913d417e31d30 50a19cc96	0	0	0	0	0	0	0	8	0	0	0	0
1801da8c0af00d6c9e552f76 19cdaf88	0	0	0	0	0	0	0	0	0	0	8	0
8462c4a7f3f028d4b49993eb 909c1560	0	0	3	0	0	0	0	0	0	0	0	0
af312a698ccabbf5ed5c37d25 ff5c52d	31	0	0	0	0	0	0	0	0	0	0	0
b03bd84548a40ee6200f761f 63320ba7	0	0	0	0	0	0	0	0	0	4	0	0
350d4428481d4806520cd5c 46782ed08	0	0	0	5	0	0	0	0	0	0	0	0
2249b480726f71cec6493648 8aa9079b	0	0	3	0	0	0	0	0	0	0	0	0
7d2363337f946ca1603e39c0 611cb636	0	0	0	0	0	0	0	0	0	0	0	28
4a3e43fd8383742451ed7275 bf775073	0	0	0	2	0	0	0	0	0	0	0	0
eb1be22032f62c43d4d0af6c 1774b083	0	0	0	0	0	0	0	0	7	0	0	0
eb461f2dc0e7f96fbb935b8cc 6bc9a33	0	0	0	0	0	0	0	0	0	0	0	4
e53f17e8415ec20d94472eae c4379131	0	0	0	0	0	4	0	0	0	0	0	0
b5b5eed4ea7b3619844ea84 9cc780ff8	0	0	65	0	0	0	0	0	0	0	0	0
b87ab1baf0bdc9eeaf70d1e0 c6343f0f	0	0	0	0	0	0	0	0	15	0	0	0

0d90caae782681a44c088182d573c151	0	0	0	0	0	0	0	0	0	0	2	0
387046d0dae7e7a8393fcbbaf843f8c6	0	0	0	54	0	0	0	0	0	0	0	0
a1b6373818d43f888d29cd380cc18b71	0	0	0	0	0	0	0	0	0	0	9	0
5bd5c8c7571a75bb8b40528feb45838e	0	0	0	3	0	0	0	0	0	0	0	0
f354bf5761ccc37e8cfa73310f824248	0	0	0	0	0	0	0	3	0	0	0	0
ed1f394aca2b653fd6e8f91fad56c0b1	0	0	0	0	6	0	0	0	0	0	0	0
47bc8dbc0bceea532b6aa2436c2de2b9	0	0	0	0	0	0	0	0	0	0	9	0
21b1c194ffeaa56932732f948fdbccd7	0	0	0	0	0	0	0	0	2	0	0	0
fcbbbb3bd83b6bb5ff7863c33c0baa0a	0	39	0	0	0	0	0	0	0	0	0	0
a5663d8eb088e67027371a3f17d3c1ef	0	0	0	0	0	0	0	5	0	0	0	0
ecef6f1bc8c883eaa320b7edeb0d9338	0	0	0	0	3	0	0	0	0	0	0	0
17458bad073c436899c97e31e56ebf91	0	0	0	0	0	0	0	0	0	0	0	3
dabc58235104f2b2ef344a335cc53a06	0	0	0	0	0	0	0	28	0	0	0	0
0e5a0ebea1421c4705ce85742aa1cdc2	0	0	0	0	12	0	0	0	0	0	0	0

5e08bda6450fabb16fc9485fe9291ce2	0	0	0	0	0	0	0	0	0	5	0	0
f21aa43535ec0a3e51962ba5a167f56b	0	0	0	0	0	0	0	0	0	0	0	8
bb61491871a354a9fb42e5f5ba8c7a30	0	0	0	0	153	0	0	0	0	0	0	0
ef16d861d653c6054b6893c7cc1baf0e	0	6	0	0	0	0	0	0	0	0	0	0
dfbdd384b9c0975ef74670c6472872b3	0	0	0	0	0	0	0	0	3	0	0	0
70ed7eda95c6258a0c85c1303e89ce6d	0	0	0	0	0	0	0	0	0	0	2	0
5e041442eba9c9ce33bff397ccec9379	0	0	0	0	0	0	10	0	0	0	0	0
b603b1d5005a38ceb8e16b5276c6c5ee	0	0	0	0	2	0	0	0	0	0	0	0
94f812b3f018d5b6507900f8dcdba012	0	30	0	0	0	0	0	0	0	0	0	0
c685c91ec6a0caf8fe4b9b59f91befc3	0	0	0	0	0	0	0	0	0	0	3	0
fbcc885e67602abba0b451091da073d1	0	0	0	0	0	0	0	0	4	0	0	0
a2497c90388a2f9a29c4dc4a9f51a466	0	0	0	0	0	0	0	0	0	0	0	3
b07209d47c32169f8e970cd48b2cbb2d	0	0	0	0	0	0	0	0	0	0	0	12
859fadd4d27bd60ccb1dcfb63404179c	0	0	0	0	2	0	0	0	0	0	0	0

c810a5775d23890a653185c0629eed4b	0	0	12	0	0	0	0	0	0	0	0	0
90a9a9627649f8635394bd2fedb9fee9	0	0	0	0	0	0	0	0	0	0	0	3
e7a63904c5137a2a1b16b5185c267679	0	0	0	0	0	0	0	0	0	16	0	0
849c34d70c6272e0a1082e5546f92962	0	0	0	2	0	0	0	0	0	0	0	0
08393148213ffe9371f453315d4eb94a	0	0	0	3	0	0	0	0	0	0	0	0
fc9889810e656bcea1effe661b4d8492	0	0	0	0	0	0	0	0	0	0	0	4
ea9dbabc11e2ca3e4461a178cf383f46	0	0	0	0	0	0	0	0	0	0	9	0
305eade000eaea853522c982905e341c	0	4	0	0	0	0	0	0	0	0	0	0
ab81d437510278ab39f11b83824fdc7f	0	0	25	0	0	0	0	0	0	0	0	0
b0eeba608b3306c13e1ea7f4af10d0f4	0	0	0	0	0	0	0	0	0	3	0	0
754625db46a183000b30382db42916a2	0	0	0	0	0	0	0	0	0	0	0	5
c336c306af022346f5df2f7fa1fc0dda	0	0	0	0	0	0	0	0	0	0	5	0
3c05f075e84ff763dcf2f83481e9016a	0	0	0	0	0	0	0	0	0	4	0	0
2f42576f2e6ee708edb10a062b8f1deb	0	0	0	0	0	0	741	0	0	0	0	0

ab714e3ea46eb53cda6eb2628e1c4de1	0	0	0	0	0	0	0	0	0	25	0	0
4ab8203fd656449618be0ff7eb983d3d	0	0	0	0	0	0	0	0	0	0	3	0
a91aa98bc583775687128bfa9360aec8	0	0	0	0	0	0	0	0	0	3	0	0
8891338e1457b0b04ae0a27194299dbd	0	0	0	0	4	0	0	0	0	0	0	0
6f7ade7f7c3dc05cf0074be164d0f3b5	0	0	0	0	0	0	0	0	0	0	3	0
ac5336f0441ab77f92fd2a4e4d1e1596	0	0	0	0	0	0	47	0	0	0	0	0
83a2fc52d8df97fde5771d3125bd1f6b	0	0	0	0	0	0	0	3	0	0	0	0
a698776b6246dc573b214c4634e5bf75	0	0	0	0	0	0	26	0	0	0	0	0
201ae917c4c9991331c2e05719158a05	0	0	0	0	0	0	0	0	0	2	0	0
8db6700d67a0641990693cbc0bac7a96	0	8	0	0	0	0	0	0	0	0	0	0
96832d1cd63e7433b01d1540181ccd24	0	0	0	0	0	0	0	0	0	68	0	0
3633a3174682c124a00b6517893e7834	0	0	0	0	0	0	0	0	6	0	0	0
f63f56ad59333978edb6358426312b6b	0	0	0	0	0	0	0	5	0	0	0	0
9c58f0facde62f9704674fa7120dbe64	0	0	0	0	11	0	0	0	0	0	0	0

d9bd934ecfbd77744752662cec5b6dbc	0	0	0	0	0	0	0	0	0	0	0	17
be34a0eefd8354b861e1dcc046e5aa45	0	0	0	0	0	30	0	0	0	0	0	0
48a2c7f445249da08a5222d0018a866b	0	78	0	0	0	0	0	0	0	0	0	0
36c14bca86b6da145ba3d96007f0ddb1	0	0	0	64	0	0	0	0	0	0	0	0
522e73912cbe1514e72ab77e28992c2e	0	0	0	0	0	0	0	13	0	0	0	0
ac0551604451a74a762233db186e8df9	0	0	0	0	0	0	0	0	0	0	5	0
59585856a9b45e80069468f445594027	0	0	80	0	0	0	0	0	0	0	0	0
e2b05b871e9d21b4a875c47d3d1f489a	0	0	0	0	0	0	0	0	5	0	0	0
c41e7574cca1abea0002a03b8f70a3bc	0	0	0	0	0	0	0	0	4	0	0	0
11ef53876f3893f4a5bbda7fce1bb4e6	0	0	0	0	0	0	0	0	0	0	79	0
1e3837c8d36e03a835331c0ddd904c37	0	0	11	0	0	0	0	0	0	0	0	0
b677b575b8ecd6f170f149e0c75873f5	0	0	0	0	0	0	0	0	0	0	0	81
0d532388fb9b31296d4c48e81203d5fb	0	0	0	0	0	0	3	0	0	0	0	0
fff93b971a3702fc9896aafd9a610da7	16	0	0	0	0	0	0	0	0	0	0	0

96dede5746b5dc7ba9c33c8a6e132465	0	0	0	0	0	0	0	0	0	18	0	0
50347241d08cb48f34dba387d3a5aaa3	0	0	0	12	0	0	0	0	0	0	0	0
853ac305fd08e9a0e93965ecddcd761c	0	0	0	0	0	0	0	0	0	0	28	0
aa02c98bf35f65b7f35f3911952e5d7a	0	0	0	0	0	0	0	0	0	0	3	0
b6044c5a8f9e999fa622836f752f3fad	0	0	0	0	0	0	0	0	0	0	22	0
759551548ceb9794ede402d0d1b42728	0	0	0	0	53	0	0	0	0	0	0	0
c74519cefe3fb403023f033b57ade260	0	0	0	0	0	0	6	0	0	0	0	0
68d23e2a9054f9cf3962fb438e59022c	0	0	0	0	0	27	0	0	0	0	0	0
63b33c2cc4124c8ec9b578ff0dda4d63	0	0	0	0	0	0	0	7	0	0	0	0
9e0764717aa0c851ce52a78f71bd1d20	0	0	0	0	0	0	0	8	0	0	0	0
6cc166204081bca01b6586dca398ee7f	0	0	0	0	0	0	0	0	10	0	0	0
a909108f807aaa8d8f4a2b7260e9dfc3	0	0	0	0	0	0	0	0	0	0	0	4
bba968db918e229f867752ce9ec679c9	0	0	0	0	0	0	0	0	11	0	0	0
f3154288ec2a284ac45668c71d13b491	0	0	0	0	0	0	0	0	0	0	4	0

12bc24b5a413d36f31c743f5f283dde9	0	0	0	0	0	0	0	3	0	0	0	0
2821f245d0ad0d9aa369178532960baf	0	0	0	0	0	0	0	0	0	0	0	4
674d5dabda5d981493f5ee995155ea18	0	0	0	0	0	2	0	0	0	0	0	0
fc5f26085c5ace3502c34f1c3de6e493	0	0	0	0	0	0	0	0	0	4	0	0
45e7302cd826616d1854b9b91a72cd30	0	0	0	0	0	0	0	0	16	0	0	0
519d0ef67b63e1bd693084631e401301	0	0	0	0	0	0	0	0	0	0	3	0
96c4fc10364be127e73a30f6fc342c58	0	0	0	0	0	0	0	0	0	0	0	2
8f0460cdd7ac30975ad322c0e9a8bfd1	2	0	0	0	0	0	0	0	0	0	0	0
4d66ce1c52430a28f98ac7f73bf872d3	0	0	0	0	0	0	0	0	0	0	0	3
cd5b4fea872c335d2e58dc109cf0bed7	0	0	0	22	0	0	0	0	0	0	0	0
ab8ea1042d92cfa3f45d2628b3164030	0	0	0	0	0	0	0	0	0	0	8	0
c94366ee0efc9d29c2b5d34e6176ca4d	0	0	0	0	0	0	3	0	0	0	0	0
26f6b877308f3fb7b243c19e9842b598	0	0	0	0	0	0	0	0	0	0	22	0
67884bddc45ebc6298545e24fc4ef788	0	0	0	2	0	0	0	0	0	0	0	0

830ff87e65598ace71158985 3fc44a26	0	0	0	0	0	0	0	44	0	0	0	0
8c65669751b065419bb1757 58a87e085	0	0	0	0	0	0	0	6	0	0	0	0
871ef2132192909c8c405d55 02423a14	0	0	0	0	0	0	0	0	0	0	2	0
a99deb78c464866a4723c87b 5ae9d5c0	0	0	0	0	0	0	0	0	0	0	3	0
4489635f4800dd429e253a4e 9d483e23	0	0	0	0	0	0	0	0	0	0	0	23
d06bce5a0fb591124d1957ab 26d5344a	0	0	0	0	0	0	0	0	0	0	3	0
d6da5ad3da38be8563c99a5 2df1d4f1d	0	0	0	0	0	0	2	0	0	0	0	0
92013f18c8a3141f74d36e66 740c324d	0	0	0	0	0	0	0	0	0	0	3	0
d91645950eac2382c2020f14 2ada7b5e	0	0	0	0	0	0	0	16	0	0	0	0
ee70f9ace27c3d567e6f1c23e 7d95156	4	0	0	0	0	0	0	0	0	0	0	0
f0dde1d5aa6a3f4347bf78f36 1411131	0	0	0	0	0	0	0	0	0	0	0	19
85907db82d11a1da0781d38 61a239776	0	3	0	0	0	0	0	0	0	0	0	0
12e612701a48380fb958060f 6cb7e7d4	0	0	0	0	0	0	21	0	0	0	0	0
899d430850ef1c0f015ea655 9eb6bfeb	0	0	16	0	0	0	0	0	0	0	0	0

1f17a9625180e80db66f7c2eb18ed40e	0	0	0	0	0	0	2	0	0	0	0	0
49af3d40e11487ac5a6d4006ada04891	0	0	0	0	0	0	0	0	0	0	2	0
c5cdab2fd2fb6fa4ff3a26f8c6473ec9	0	0	0	0	0	0	0	0	0	0	40	0
1729b9e42fcf252e82fa6779cca2eb8d	0	0	0	0	0	4	0	0	0	0	0	0
e9a2259923b1e5e308b753ee5c57b6fb	0	0	0	0	0	0	0	0	0	6	0	0
9263fbcb486e24da4d201102c6392b3f	0	0	0	0	0	0	0	2	0	0	0	0
29cce3722eccfdd52a78f292f03bf8e9	0	0	0	3	0	0	0	0	0	0	0	0
7c04c1824466e14d7dae73d2f99444c9	0	0	0	0	3	0	0	0	0	0	0	0
7c031667f8953e2bf1ce7eb850ce09c5	0	7	0	0	0	0	0	0	0	0	0	0
a391a1d5104103f94dbe224ced8d6fcd	0	0	0	23	0	0	0	0	0	0	0	0
43985e7d1c1ead2b00fa9b5ad7d893a2	0	0	0	0	0	0	0	0	0	0	2	0
dab89ae4513e7afe9c45eb360f882c4d	0	0	0	0	33	0	0	0	0	0	0	0
c862720a21f6936662eef054e07c62b0	0	0	0	0	0	0	0	0	0	0	0	9
f6f9f2f7c030bf5649b7190fb91eca20	0	0	0	0	0	0	2	0	0	0	0	0

e662bfba79601e0c385a4675fd7b1ab7	0	0	0	0	0	3	0	0	0	0	0	0
8f0b736f141794e9d062823f24e4fb9b	0	27	0	0	0	0	0	0	0	0	0	0
236ddc78838634fac1ad7d03fed2b57c	0	0	2	0	0	0	0	0	0	0	0	0
0e23db340ffbcb0566896bcc8e4cf4f5	0	0	0	0	0	0	0	0	0	0	5	0
aed10b7def392c01358643e38f00463b	0	0	0	0	0	0	0	0	0	0	0	26
618cbbaef095139520a6a10625444ea5	0	0	0	0	0	0	0	0	0	3	0	0
8d07136b91aa749ceea5e8c707c0b65d	0	0	0	0	11	0	0	0	0	0	0	0
8a4304ccb599f468280b92d957bb57ab	0	0	0	0	0	0	0	0	0	0	2	0
2db9239d7b30753d0c3595d5b0663ca7	0	0	5	0	0	0	0	0	0	0	0	0
d469eb64bcc9fb8f3a32499707fbf7d2	0	0	0	0	0	0	0	0	4	0	0	0
a889d36b536ba1fa3c5b0c3cf14760d3	0	0	0	0	0	0	0	0	0	0	14	0
18e16a2d5964aa3501661cfa3bad009d	0	0	0	0	2	0	0	0	0	0	0	0
16359d9dbc680667bcecd3dc0cb25966	4	0	0	0	0	0	0	0	0	0	0	0
6c6ecdfd7494b7cc639be3cc0416ab86	0	0	0	0	0	0	0	0	0	9	0	0

954b16457e05243fb8eba7d6c097646e	0	0	0	0	0	0	3	0	0	0	0	0
0621c83bd4644c6fbfb794864ecb3a24	0	0	0	0	0	0	0	0	0	0	0	10
b2fa14cec2170302f731b7ed286781fd	0	4	0	0	0	0	0	0	0	0	0	0
4dcc1a202d5c1d65f6224b4df9d3043d	0	0	0	0	0	3	0	0	0	0	0	0
4b188935ffdee41a8b3d1a03bccc06ff	0	0	0	0	0	18	0	0	0	0	0	0
51280c9e8bb052723b8ceed3c71b225a	2	0	0	0	0	0	0	0	0	0	0	0
fb66de9c6f532055d4c67016042e65f4	0	0	0	0	0	0	0	0	0	0	0	3
188f372d2ac045be64e2fce1316017e1	0	0	0	0	0	0	0	3	0	0	0	0
7ff616cec60fba93cf5e0776ea19b06f	0	0	0	0	11	0	0	0	0	0	0	0
d6bffef790a9159dfe762f5f624af98a	0	0	0	0	0	23	0	0	0	0	0	0
c51c6adbe64c059e2933f8e4526669b5	0	0	0	0	3	0	0	0	0	0	0	0
181b896f5bc0e278cd0b59af2ea79623	0	0	0	0	0	0	0	0	0	0	6	0
40f39837d70cfdcd0f3122b3db40af17	0	0	0	0	0	0	0	9	0	0	0	0
dafab8eb7d0dcd79f51c77e4eae06556	0	0	0	0	0	0	0	0	4	0	0	0

98fd210eeb9e9ca1722e4e17e9177285	0	0	0	0	0	0	0	0	0	60	0	0
5c912e8a67e58d31ee1e543e34dbac7a	0	0	0	0	0	0	9	0	0	0	0	0
c72104f767c3e94d953f2c75e865d9b2	0	0	0	0	0	3	0	0	0	0	0	0
acd1536fe843d43ac2f4618df05ece86	0	0	0	0	0	0	2	0	0	0	0	0
f2669cc6650b1335af50ccd13d62085a	4	0	0	0	0	0	0	0	0	0	0	0
121ffec297fe354da794b13c3ad7d2e0	0	0	0	0	0	0	0	0	0	0	0	6
2a8fd9e4b55332e541b0373a5d4f463a	0	0	0	26	0	0	0	0	0	0	0	0
f0e01fb940b1879e898dd7df9f92f3f7	0	0	0	0	0	0	0	7	0	0	0	0
815f624fd1df44c74fe6f1ede58b860a	0	21	0	0	0	0	0	0	0	0	0	0
564c4b36b2661340c1749aa03c197b49	0	0	0	0	0	0	0	0	2	0	0	0
a0d3cc33160e61ca8d4b1c169b04e725	0	0	0	0	0	0	0	0	0	0	0	9
35f2c517ef8ddc1015a57bd205aef1b3	0	0	7	0	0	0	0	0	0	0	0	0
426f17d1081052167f1682c94ffb07e5	0	0	0	0	0	2	0	0	0	0	0	0
db93f98f02c477303e6b2f23e99956b5	0	0	0	0	0	51	0	0	0	0	0	0

e9217008b38f90b9df26313f d35fdfaa	0	0	0	0	0	0	0	0	0	0	2	0
1ea8ec9e69c091d9aa596849 9f269475	0	0	0	0	0	0	0	0	0	0	234	0
14be182aefd087541335d864 bc5410d2	0	0	4	0	0	0	0	0	0	0	0	0
4cc5a03d870c6a7e1b9563eb 293cb96c	0	0	0	0	0	0	0	0	2	0	0	0
55fde244d04877a31999d1a7 02c3850a	0	0	0	0	0	0	0	0	0	359	0	0
4e21a087384010afc2cb1649 442a35ba	0	0	0	0	0	0	0	0	0	0	17	0
e78bc7a4288efb1f98949843 31ba0615	0	0	0	0	0	0	0	0	3	0	0	0
bc3c46ee4272e9bb5b1589e 5b5da72de	0	0	0	0	0	2	0	0	0	0	0	0
1f8c132a639a4e7309dede2c acbccbd7	0	0	3	0	0	0	0	0	0	0	0	0
cb06510dac1fb68828878f22 987b5329	0	0	0	0	0	0	0	0	0	0	2	0
e7120d35819d9cc444875d6 9f9c23384	0	0	0	0	0	2	0	0	0	0	0	0
b30264d85e6cede33f775256 d73e16d4	0	0	2	0	0	0	0	0	0	0	0	0
15026720b4201406977bedff 20b5c1bd	0	0	0	0	0	0	0	3	0	0	0	0
a842cca8b3c7177ce790508e 4974b8e7	9	0	0	0	0	0	0	0	0	0	0	0

fe0b1b29ea88d663b511a142d386ac24	0	0	0	0	0	0	0	0	0	0	0	2
07b50e672eeeb0a682ffd8a6d431be3a	0	0	0	0	0	0	0	0	5	0	0	0
4e521753b8c69e7057529c48c9d0f179	0	0	0	0	0	0	5	0	0	0	0	0
260df70469f542333619a5d331a9dc48	0	0	0	0	0	0	0	0	0	0	2	0
66117069d11dafd0bc0265c576307066	0	0	0	5	0	0	0	0	0	0	0	0
94be33e6a5d32abf3d888656d7d45e39	0	0	0	0	0	0	0	0	0	0	185	0
a7ed664aaa34e30384ef5d487bec51ab	0	0	0	0	0	0	0	0	0	14	0	0
5696d24caad8194214b557fea405150b	0	96	0	0	0	0	0	0	0	0	0	0
4624b83d972804f00989e3de94d8d6f4	0	0	0	0	0	0	0	0	3	0	0	0
82b24a6c141e6be3fce80905eda7092e	0	0	0	0	0	0	0	0	0	0	0	11
c211bf4e906731ca8dfec0ac64a055b0	0	0	0	0	0	0	0	0	0	5	0	0
43f4132bad9b8ae496c3012c4c9be047	2	0	0	0	0	0	0	0	0	0	0	0
d126f42f846511921af0bddaa2d6244a	2	0	0	0	0	0	0	0	0	0	0	0
d9c8bc9050895d43ef550798267f8328	0	0	3	0	0	0	0	0	0	0	0	0

f8e12ec715ac1fb468b853a9e96d8023	0	0	0	0	0	0	0	2	0	0	0	0
60566a1b0c1c7251be4386031d7fbc16	0	0	0	0	0	0	0	0	0	0	3	0
e0dd9e33971b14e7c3b2217ee187616d	0	0	0	0	0	0	0	0	0	25	0	0
5c0be4a3a953d3427c0cbad149e56567	0	0	0	0	0	0	0	39	0	0	0	0
3fccfbf3dd27c071de4b1b0c901b5ff0	0	0	0	0	3	0	0	0	0	0	0	0
c853685f362b5825ebf30b921450973d	0	2	0	0	0	0	0	0	0	0	0	0
cca5386aa14c0ae982a99bd5943076fc	0	0	0	0	0	0	0	0	0	0	0	2
8c51bfd1ceb2c5e119c75f1fff6085bc	0	0	0	0	0	0	0	0	0	3	0	0
ce35a17751be74a06055796eb3415799	0	0	0	0	0	0	0	6	0	0	0	0
ce760f7560f6e82b212465eb0ff632f1	0	0	0	0	0	3	0	0	0	0	0	0
d2611867f8805aa70d47d5380adef184	0	0	0	0	0	0	0	0	0	0	0	21
0e6b7784997f7a4865fc993fdbf763bc	0	0	0	0	0	0	0	2	0	0	0	0
759e9ef78b6a1315e8ddac210c8c51ee	0	3	0	0	0	0	0	0	0	0	0	0
693b6708820a27919d1c1a0386c57e0f	0	0	0	0	0	0	0	0	0	6	0	0

cd47626c56c03365434a389185acb80a	0	0	0	0	0	0	0	5	0	0	0	0
635a587b55a81c9100c5f91cbe35a3c2	0	0	0	0	0	0	0	0	0	0	8	0
f49c43418d4256adbf83ebe07ceba8df	0	2	0	0	0	0	0	0	0	0	0	0
9f5411d5febe0f836f6e2a35aca62eed	0	0	0	0	0	0	0	0	0	0	2	0
6b8ae6e18c396847caebe3d5e0cdb4f9	0	0	0	0	0	0	0	0	0	0	0	3
cd32d07c82ed82a8f0067adc7853a921	0	0	0	0	0	0	0	0	12	0	0	0
36cc1748cf44bebdf6435fb85e4f3003	0	0	0	19	0	0	0	0	0	0	0	0
8e1e8313a9d2f5503e676f25c0f5818e	0	0	0	0	0	2	0	0	0	0	0	0
2c7ace42d829f49b4c495b6b4c9a6f7e	0	0	0	4	0	0	0	0	0	0	0	0
1ca59193c937d21d475f3cbdcb7eb89f	0	0	0	0	0	0	0	0	0	0	0	2
eb1e39d299f51f036315a6cca7420003	0	0	0	0	0	0	0	0	0	2	0	0
678bfadefb86af83e5a6e78f77b4a846	0	2	0	0	0	0	0	0	0	0	0	0
3a96b6776d9801f5bfca8787195da751	4	0	0	0	0	0	0	0	0	0	0	0
4c97c67830a26108fb1aa7a875b36a9b	0	0	0	0	0	0	0	3	0	0	0	0

18250f97e5c9eb3ab176b7de3a0ec7e9	0	0	0	327	0	0	0	0	0	345	0	475
16bcdb91972caa3084aa42d29443d3ed	0	0	0	0	0	0	0	0	0	0	4	0
068ed37bba238c4a66ea32d1ac0b5381	0	0	0	0	0	0	0	0	0	0	0	30
29d94ee7996a0c523269b9c011f7b5aa	0	0	6	0	0	0	0	0	0	0	0	0
c3c3140112da458d51d97288cee8ea34	0	0	0	0	0	0	6	0	0	0	0	0
e236476931a5da48b21d02e40fa79b0c	0	0	0	0	0	0	0	4	0	0	0	0
2d4235f89244d733523347efd64360b6	0	0	0	0	0	0	0	0	0	18	0	0
da0a147ef7a15523a2dd4c4274b19fb5	0	0	0	0	0	0	0	0	0	5	0	0
9bc3c1528818bd6060dbed5e49323f33	0	0	2	0	0	0	0	0	0	0	0	0
a87e4b972dd0145f0058b871ca67ceef	0	0	0	0	0	0	0	0	0	0	0	4
c294a2acfde1b79c8b7ad503c73c982d	0	0	0	0	0	0	0	0	0	0	0	2
8a846e752d59b99d88bf12c457224a5d	0	0	0	0	0	0	0	3	0	0	0	0
1a0d13da77f0339b54e135e807625853	0	0	0	0	0	2	0	0	0	0	0	0
5962843085af0df9602d71fd66a8467f	0	0	0	0	0	19	0	0	0	0	0	0

c8c0d5a3b506e04e2748b4c06f6c00fd	0	0	0	0	0	0	0	0	0	207	0	0
6ddb6ec939841e067c1d410d843dac9a	0	0	0	0	0	0	0	2	0	0	0	0
ffddc63275938d605f7652ff7a7a2449	0	0	0	0	0	0	0	0	0	4	0	0
4d3483c1c6316fb199ad9dba95a5fbb1	0	0	0	0	0	3	0	0	0	0	0	0
ce10aac2678ad2e8ab310de46f59915c	0	0	0	0	0	0	2	0	0	0	0	0
d914637c611aa085364f34528dc4ec51	0	0	0	0	0	0	0	0	2	0	0	0
53ab1dd536bab03e515383791c728583	0	0	0	0	0	0	0	0	0	16	0	0
5f2412a9d5d6b43ee125b1e59c8a83bf	0	0	2	0	0	0	0	0	0	0	0	0
f0340241ceabb1ae3c090ffd49211f2a	0	0	0	0	0	18	0	0	0	0	0	0
c97b3bf422fb39516f676449825cd408	0	0	0	0	0	0	0	0	0	0	0	2
9f5a376a8c396476541697f5eb359aa7	0	0	0	0	0	0	0	0	0	8	0	0
e7ccc24a7227c56394413e9bcc3fbc2f	0	0	72	0	0	0	0	0	0	0	0	0
b2b0dafb372062b8be10999d8dca1ce7	0	0	0	0	0	0	0	0	3	0	0	0
76e0dc5d0719059f916dc3c4bd2c6a81	0	0	0	0	0	0	0	54	0	0	0	0

a529e1769ab207a133f28f77e96d411b	0	0	0	0	0	7	0	0	0	0	0	0
7f9d8ac0032a350c53dd13176e60f29e	0	0	0	0	0	0	0	0	0	222	0	0
14433e2f7283a41f83305d651a223b95	0	0	0	0	0	0	0	0	0	0	0	7
32d122479dadb281a8b11debdb1c2699	0	4	0	0	0	0	0	0	0	0	0	0
35de57726b8d32a7c3c6520895f47c75	0	0	0	0	0	10	0	0	0	0	0	0
d1a8da805f7965bcff1b049c55de2af1	0	0	0	0	0	0	0	0	9	0	0	0
165904a9b2b3019156f7c7412a9597f6	0	0	0	0	0	0	0	0	0	8	0	0
50fa6b82505846858c43957c7fb4484a	0	0	0	0	0	55	0	0	0	0	0	0
230dad8dd3ec0f7a714843cd6ce925f5	0	0	3	0	0	0	0	0	0	0	0	0
42d344deaff9d5dbd7534b91c10097c8	0	0	0	0	0	0	39	0	0	0	0	0
86c9daa48c6120adbff6931a734053bd	0	0	0	0	0	0	0	11	0	0	0	0
ed64c006ba477c756b0139ae65d35d1f	0	0	0	0	0	0	0	0	0	0	4	0
aeea705a2ce1b77145e9646e9b16ab30	0	0	0	4	0	0	0	0	0	0	0	0
0d6ceb6fcf01f5663b679802192e600d	0	0	2	0	0	0	0	0	0	0	0	0

199e16636f245d212954c47af9f6c755	0	0	0	0	0	3	0	0	0	0	0	0
1c4e70bb58d5cbd2f78713f49690771f	0	0	0	0	0	0	0	0	0	0	6	0
10915a15595a870744f984c426c99757	0	0	0	0	2	0	0	0	0	0	0	0
c424dd30b94e6dee1131a50407e8f14a	0	0	0	0	0	0	0	0	3	0	0	0
a1efe6497eb88fe6fe451d7d12da5807	0	0	0	0	0	0	0	0	0	0	4	0
cba9963288d32e0418f88eb31ed4822c	0	0	0	0	0	0	0	0	0	0	22	0
988ce45b95b48dd9dfc05f017511c6b7	0	0	0	0	0	0	0	0	0	0	0	4
97927a3551d3b7d6379f17b13ae7d40e	56	0	0	0	0	0	0	0	0	0	0	0
f5fcc04faaed45ddf72881617e54aaa8	0	0	0	2	0	0	0	0	0	0	0	0
559730080ff441098f62394861f5aa64	0	0	0	0	0	0	0	0	0	0	6	0
a2645b0df9005e930bfaf40e4aced080	0	0	0	2	0	0	0	0	0	0	0	0
12d332e7b59b1ceeabbfbd8dedf6b438	0	0	0	0	0	0	0	2	0	0	0	0
aae5d8baeebb2e873cc672ede276a9c3	0	0	0	0	0	0	0	0	0	4	0	0
a1f07bac8181d39b429a9955fa1dc276	0	0	0	0	3	0	0	0	0	0	0	0

8dfb7f98205f90f7eab30e73b5276113	0	0	0	0	0	0	0	0	0	0	4	0
b084549a8c61d0a9c78f48fa2b4fd897	0	0	0	0	0	5	0	0	0	0	0	0
f67b361a7ddcfaba4318c8c2d054599a	0	0	0	0	5	0	0	0	0	0	0	0
41b6f3a5b9d5f444b06c12a2c1549461	0	0	0	0	0	7	0	0	0	0	0	0
6b7e74b9da0035cefc7ba51f3e7800d2	0	0	0	0	0	8	0	0	0	0	0	0
07a6e8202e7a19bb9b4c0d4704021e00	0	0	0	0	0	0	0	2	0	0	0	0
7073fce8cfdcef461be9e3b2113a16f1	0	0	6	0	0	0	0	0	0	0	0	0
810340a9942d43e2a9c7506f4303e625	0	0	20	0	0	0	0	0	0	0	0	0
daeecbeeb931abf80d7b0b9c503a35bd	0	0	0	0	0	4	0	0	0	0	0	0
1560b3e08de39235be502e4bee1ed4e8	0	46	0	0	0	0	0	0	0	0	0	0
f19c38193240dc360f45dc8c0eb3791e	0	0	0	0	50	0	0	0	0	0	0	0
17823c821a15aad82e57b62518dda410	0	0	7	0	0	0	0	0	0	0	0	0
04feed0297c61872411123a46b839050	0	0	0	0	0	0	0	0	0	0	0	3
e781d0de8f2f17866422a53c91aa6edf	0	0	0	0	0	0	0	0	0	0	0	3

22bd4352323b1df900321d538ec6e31b	0	0	0	0	0	0	0	0	3	0	0	0
022b14ea8373031eaadef7816881d53c	0	0	2	0	0	0	0	0	0	0	0	0
41c014a9a35d4647258ee718b5d368d2	0	0	0	0	0	0	0	2	0	0	0	0
3fdd28107a8a7e082d48a678ab195899	0	0	0	0	0	0	0	0	0	0	0	3
87f20f6fd060f92896fbdc0d25d5c089	0	0	0	0	3	0	0	0	0	0	0	0
7926eb6a803974a571ed978669016938	18	0	0	0	0	0	0	0	0	0	0	0
13601102085968ae62397dde491ef76d	3	0	0	0	0	0	0	0	0	0	0	0
eababa2ad6d03060a634364c05379aec	5	0	0	0	0	0	0	0	0	0	0	0
3abbb7f3ff0b8fba48f17c7c88755f4e	0	0	0	0	0	0	3	0	0	0	0	0
b3c82a0fa45d1470b12c9b2674ec48fd	0	0	0	0	0	0	0	0	0	0	68	0
59bc9db03386d69c1b4b3adb698996f9	0	0	0	0	0	0	0	0	0	0	0	4
6ea68feea94fd2d0cdc788564899cef6	0	0	0	0	0	0	0	0	2	0	0	0
6c3651c31f876b028beb138a6edb6c03	0	0	0	0	0	0	0	0	0	0	4	0
9bfe890d47fcc10ee527f98a1ea82e03	0	0	0	0	0	0	4	0	0	0	0	0

76f827f637eca2aa214af3354 8f288e8	0	0	0	0	0	0	0	61	0	0	0	0
8e5067deb95b5b7323c83e7 9f845c394	0	0	0	0	0	0	7	0	0	0	0	0
571d098297b68493be21fcb5 effe8a8f	0	0	0	0	0	25	0	0	0	0	0	0
54b1015f4571d62fc9ff92e4a fa1c7cd	0	0	0	0	3	0	0	0	0	0	0	0
a80d7db77b6f958d76b0cd9e 014f8922	0	0	0	0	0	0	0	0	0	0	0	2
59453ae32a7e39254498866 a4b56a9ca	0	0	0	4	0	0	0	0	0	0	0	0
bc2697563e46d80cc8ca2d95 725b639c	0	55	0	0	0	0	0	0	0	0	0	0
310040f21ea23c06c518f4ea a7d5635e	0	0	0	0	2	0	0	0	0	0	0	0
6f8c4f0cc8e169ec9f3a5a540 463ae67	0	0	0	0	102	0	0	0	0	0	0	0
e10566a7468aa1b5b35242cc b078f333	0	0	0	0	0	0	0	0	0	0	2	0
5ab1a89fe52ea2d0c95da954 3f68c906	0	0	0	0	0	0	0	0	0	0	0	25
1fa2e7f1f277244245d5f25f5 d3d1414	0	0	0	0	0	0	0	5	0	0	0	0
6d6a6ff8390b61478453d3b4 777c7ea1	0	0	0	0	0	0	0	2	0	0	0	0
8b17d1fe131b79b2fbf4b81f6 ae6f726	0	0	0	0	0	0	0	0	0	0	3	0

b5606b638f98d6faa95f95ad 1a5b789a	0	0	0	0	0	0	0	0	0	0	3	0
ffe5de942bbd2265b299b162 ce7b44eb	0	0	0	0	16	0	0	0	0	0	0	0
1b56eb83a08fab3bd1685b34 7c6c7761	0	0	0	0	0	15	0	0	0	0	0	0
e104d0b89c5ef0d5d3307081 cf348c59	0	0	0	0	0	0	0	0	0	0	0	11
fccf0bce7aeac63299b1d5afd bd25968	0	0	0	0	0	0	0	0	6	0	0	0
5013d7640d1cfa4cfd6afc61c 642336d	0	0	0	0	0	0	0	9	0	0	0	0
eb2fd994f7d606969c0de7ec 4b04099a	0	0	0	0	0	0	0	0	0	0	0	40
c86b011cb92d6ed01f9366ac ec8bb564	0	0	0	0	0	0	0	0	3	0	0	0
93c9ef0ac213bd398e0b9bd4 6a18931a	0	0	0	0	0	2	0	0	0	0	0	0
c39185983ac146b68cddb91e c8da50a6	0	0	0	0	77	199	0	0	22	0	201	0
fda493c93ad80deb75df64c4 85d7722d	0	0	0	0	2	0	0	0	0	0	0	0
7adc02661e69e40d54f37b3c 4ac4f25d	0	0	0	0	0	0	0	0	0	0	61	0
9580b5c21d45912166b57cb aa338ad9e	0	0	0	0	0	0	0	0	5	0	0	0
083f09304a18f4036ede60e4 9b86d56c	0	0	0	0	0	0	0	0	0	0	3	0

cb74403643e27bed7602180 54c3f7636	0	0	62	0	0	0	0	0	0	0	0	0
d182bcc5f18366b9537fedcd 59731d23	0	0	6	0	0	0	0	0	0	0	0	0
0d50747b4add724c5bca73c1 d0693f44	0	0	0	0	0	0	0	0	20	0	0	0
b45d6fdb71ea66f73cdc01dff bf15245	0	30	0	0	0	0	0	0	0	0	0	0
b8dd2505243aaf33f4131fd1f 508503d	0	0	0	0	0	2	0	0	0	0	0	0
a4e467e0eb1d4ae12856798 a2507f561	0	0	0	12	0	0	0	0	0	0	0	0
1a6bb15aba9b2c9bdf87906f 7df42bc3	0	0	0	0	0	0	0	0	0	34	0	0
74394dfb066c5e8fadccaff97c 83cb6a	0	0	15	0	0	0	0	0	0	0	0	0
0f77dfdccbe5871ac57e029ac d5cb41a	0	0	0	0	0	0	0	0	0	0	0	12
7c9e4061e377b985bb8f0d00 a40f7836	0	0	0	11	0	0	0	0	0	0	0	0
5620165ae9bd21b58ee36ff7 efc89ba3	0	0	0	0	0	0	0	0	0	11	0	0
1dbb69cace572d9aeba1cfd9 854e0f0e	0	0	0	0	0	0	0	0	0	0	15	0
a270fa2eacc3892c4a1f39301 e0d9eb8	3	0	0	0	0	0	0	0	0	0	0	0
de61251a6368451de08892a 4516d1a9b	0	0	0	0	0	0	0	5	0	0	0	0

d93ede214eff357068568a25355c42a9	0	0	0	0	0	0	3	0	0	0	0	0
3dc4849ff003371ab8c585cda666c868	0	0	0	0	4	0	0	0	0	0	0	0
cbe9edf6b2949082376e0060de915ff6	0	0	0	16	0	0	0	0	0	0	0	0
dedee646ce9f8679f6daa2aac2255f6c	0	0	0	5	0	0	0	0	0	0	0	0
394e7594d703100137c5dab43eac3032	0	0	0	0	0	0	0	0	0	0	2	0
e963ca247d91cf41079a74ac0e536710	0	0	0	0	0	3	0	0	0	0	0	0
f2622109a330c7f40665a83ccaf15878	0	0	0	0	0	0	0	0	0	0	23	0
d2b09b52acea439a491d8e224711fdb8	0	0	0	0	0	0	0	0	0	0	0	11
6a121ec48e3a9d55d16330f59f6d3e37	15	0	0	0	0	0	0	0	0	0	0	0
430a909e88bd666acce7d92dcefca441	0	0	0	0	2	0	0	0	0	0	0	0
f65d1e0553d52cea1b7d7c91bed644bb	0	0	0	0	0	0	0	0	0	11	0	0
a51ad70c41280956bdad8fda88bd6c77	0	0	0	0	0	0	31	0	0	0	0	0
ba6f5f6044c1a1d09d7610fd9f061470	0	0	0	0	10	0	0	0	0	0	0	0
4294e0e040c9862308909d5608e27557	0	0	0	0	5	0	0	0	0	0	0	0

82dedf0f3076dae28013a99f573baad9	0	0	0	0	0	0	0	0	0	0	3	0
e34fc7d3f14312d3b2e4bfd670ffd8df	0	0	0	3	0	0	0	0	0	0	0	0
e132a156c636e9ae5e0f23c6c4e11a6f	0	0	0	0	0	0	0	0	3	0	0	0
a7f4383dc66923cb0d5c4250145c3d70	0	0	0	0	0	0	0	0	0	0	2	0
0f00a010d9c8357c6f0d5f4a3ddc2dc7	0	0	0	9	0	0	0	0	0	0	0	0
e7c3598b96bd1d6b05b516b43a435698	0	0	0	0	0	0	0	0	4	0	0	0
8dc5f645b105aa46f1ca8e937db40e1e	0	0	0	0	0	0	0	0	8	0	0	0
164698705effcbf007105ca1b97303f4	0	0	0	0	0	0	0	0	0	83	0	0
f2b108e63d7922c57095e1cd393ad0e5	0	3	0	0	0	0	0	0	0	0	0	0
69195695696379bf373a00101f5fdf39	0	0	58	0	0	0	0	0	0	0	0	0
e5fd1037d3a8d76311a1647734f9a2c8	0	0	0	0	0	0	0	0	0	0	0	7
d2d1b6ff3617ed117abb9448c0273f4a	0	38	0	0	0	0	0	0	0	0	0	0
8e56abd404230dade3592fb6c7729cab	0	0	0	13	0	0	0	0	0	0	0	0
138cef163077b4379de94bf528f434a0	4	0	0	0	0	0	0	0	0	0	0	0

995ff59b97cd9b86a0ae8521 96a43dad	0	0	0	0	0	0	0	2	0	0	0	0
6e8fb87c4d3dfbe878691252 37b2b2e7	0	0	0	0	0	0	0	0	0	0	0	2
c8a65f6e79523f6cf149d737d 278bed0	0	0	0	0	0	0	2	0	0	0	0	0
eeb942848ce206063bdc052 b7a0b82f9	0	0	0	0	0	8	0	0	0	0	0	0
6c3c197d12dbca34a1c40097 c756580e	0	0	2	0	0	0	0	0	0	0	0	0
7f8733a86e8b94f9bcc4faf66 5290059	0	0	0	4	0	0	0	0	0	0	0	0
9e08168413a4436ccb68008c 51fd8607	0	0	0	2	0	0	0	0	0	0	0	0
e89f5226904dd2104f32ef13 89d0b3e6	0	0	0	0	0	0	0	0	0	0	0	4
30a9017f195e54ac0de4610a 77c35be4	0	0	0	0	41	0	0	0	0	0	0	0
95a21f3d9da79cefbd5ed14a 03846039	0	0	0	0	0	0	0	0	4	0	0	0
584a5755cea357b85727976 a5d5ce71c	0	0	0	0	0	3	0	0	0	0	0	0
990ca7a8d7be3cba45175c55 70834434	0	18	0	0	0	0	0	0	0	0	1108	0
5429ce25c2f1e521ce609315 9a2e2fd9	0	0	0	0	0	7	0	0	0	0	0	0
43f0d1b80905e293cb2f4c86 09521918	0	4	0	0	0	0	0	0	0	0	0	0

53e6c668308e553d8d9a1bd77d154f6e	0	0	0	0	10	0	0	0	0	0	0	0
294a3a02c9cb19a400a228504e09b186	0	0	0	0	0	0	0	38	0	0	0	0
33f3388c0fe4b184f1249e955dc0ea6d	0	0	0	0	0	0	0	0	3	0	0	0
2d321580679e40d2996898220294ce12	0	0	0	0	0	5	0	0	0	0	0	0
1fccdf2215e703a4b731d961750d9d0f	0	0	0	0	0	0	0	0	0	0	4	0
e10502cd13b1eff7fe5ede3a85d192af	0	0	0	28	0	0	0	0	0	0	0	0
11d76147544c00440a21d15d68aaa034	0	0	0	0	0	0	0	3	0	0	0	0
3d8a99e8689f9e14803ee25e8d9a5fb9	0	0	0	0	0	198	0	0	0	0	0	0
26d4ab24fc0728637628ed9ba91202b5	0	0	0	0	4	0	0	0	0	0	0	0
ecd1f7067467418881e83b0f7371c03e	0	0	0	0	0	3	0	0	0	0	0	0
ce4fd4f246f7a2463c00016c6785170a	0	0	0	0	0	0	0	0	0	0	7	0
7087cb8ae46d0aefaa3ce3e2dd74aee7	0	0	0	0	0	0	2	0	0	0	0	0
e6487d559123641a4b120245980fc53c	0	0	0	0	0	0	0	0	0	2	0	0
f2d51edf6e92fd4c556ba0cea9bea322	4	0	0	0	0	0	0	0	0	0	0	0

153aafa196fb08eba12f4a711fbe2eef	0	0	0	0	2	0	0	0	0	0	0	0
8f59965baa36ecb12dc8b5e1029d19ca	0	0	0	0	0	0	0	0	0	3	0	0
bf496b8acdd14ea59c4b9e786cf645bd	0	0	0	0	0	0	0	0	0	31	0	0
cc649aec3673a7ec4dcce7d636ecfbd8	0	0	0	0	0	0	0	0	0	0	3	0
ec13dc60096754068bb87b7696757007	0	0	0	0	0	0	0	0	0	0	0	3
4be46024b962f1d6b6cb396f27cd0f64	0	0	0	0	0	32	0	0	0	0	0	0
ffa95f48d75ff51eb0129c6b5ef11085	0	0	0	0	0	14	0	0	0	0	0	0
dffd00890331f703ae8a369783b8b433	0	0	0	0	2	0	0	0	0	0	0	0
7942ca1efc66b1b2fd638db8ced99737	0	0	0	0	0	0	0	0	39	0	0	0
02b93d63b8c86efd02b696c60d216015	37	0	0	0	0	0	0	0	0	0	0	0
237cab677c82e74844e2ca5574e56bf0	0	0	0	0	21	0	0	0	0	0	0	0
f1fc3b739bd86072598890f117df1be5	0	0	0	15	0	0	0	0	0	0	36	0
d78bfd515e0d75e29840055948d8e73b	0	0	0	0	0	0	0	0	0	0	3	0
f0b6047cf0643ef3617deaec0de6ee2d	0	0	0	0	0	0	0	3	0	0	0	0

57e1542f3427ae9e4b08e796 25850817	0	0	0	0	0	0	0	0	2	0	0	0
f9b120a1046344529d1cd688 240e5f8d	0	0	0	0	0	0	0	0	0	3	0	0
be3479e6808afc5453f3d585 20e6bf09	0	0	0	0	0	0	0	15	0	0	0	0
8c317d41e1192d3a75a424a e2d9e4ee0	0	0	0	0	0	0	0	0	0	3	0	0
5245f76e3f69bab83071760d 9a33be54	0	0	0	0	0	0	0	0	0	0	0	2
2e3c8f276b2576d8ab112025 bcc6f126	3	0	0	0	0	0	0	0	0	0	0	0
7bbe3546d1cef6d1e9b3d2c9 2f6c6c9f	0	0	0	0	0	0	0	6	0	0	0	0
5dbf428cdd7d7ccfa2da1b71 d66d9912	0	0	0	0	0	0	0	0	0	3	0	0
344d0e8f50bdfa8538bcceb7 c69d3920	0	0	0	0	0	0	0	0	0	0	0	29
a4a3a91a0c79ea13ef8c02fe1 045a915	0	0	0	0	16	0	0	0	0	0	0	0
3a0ba49b247afadeeaf78b5fd f369a0d	0	0	0	0	0	0	0	16	0	0	0	0
2ec46c993290a669f8f95801 6b545b97	0	0	0	0	0	0	0	0	0	0	10	0
09a47a078b4c125e1085c349 92a54462	0	0	0	0	0	0	0	0	0	0	7	0
bc811f0f04d6db9e87faf51b7 d51a3e5	0	0	0	0	0	0	0	0	0	0	2	0

def74a3d4c04f809338b5958eb5f2ddf	0	0	0	0	0	0	0	0	0	0	15	0
4390076dcf901c79ff8bb27e0712928f	0	0	0	0	0	0	0	0	0	2	0	0
7b0cfa3a1e5aebb3796bd31bd06c9f03	0	0	0	0	22	0	0	0	0	0	0	0
c614a6f32ac1a3da808a2fa41e389f78	0	0	0	0	0	0	0	6	0	0	0	0
5300577018a74ee03498d9f190ed7372	0	0	0	0	0	28	0	0	0	0	0	0
fa3aea010c73aee6a0dd79e37e5564df	0	0	33	0	0	0	0	0	0	0	0	0
6ac19cd4db7c7daa53003e4feda7a4ba	0	0	0	0	0	43	0	0	0	0	0	0
68eec25e633d6c10498e196a31457772	0	0	0	0	10	0	0	0	0	0	0	0
2a4c23d22e3fae8bc6f25b019c9e6a5d	0	0	0	0	0	0	0	0	0	13	0	0
3eb99c3d231533a8f7fca6774b7f3c0c	16	0	0	0	0	0	0	0	0	0	0	0
b25ed63c4303f19798ff8d65556348d4	0	0	0	0	0	0	2	0	0	0	0	0
c66fec42f9216189257283dd0bc7a07a	0	0	852	1223	522	1088	1738	1102	463	1099	0	1578
9d3af3463b2e90a5bd2d2105c11a6a8b	0	0	0	0	0	0	0	0	8	0	0	0
8f1119c143c1225bd3f8a20205fb0bf1	0	0	0	0	0	3	0	0	0	0	0	0

0d8cf1ec01d3fb32d43b55cbdc2c7257	0	0	0	16	0	0	0	0	0	0	0	0
d0502721b8225b3cfddccc2d9b89b592	0	0	0	0	0	0	8	0	0	0	0	0
25840a78b6aa6c612469055c2c2c6635	0	27	0	0	0	0	0	0	0	12	0	0
11fb558cb57ce435ece31aca61b78b88	0	0	0	0	0	0	0	0	0	0	5	0
5fb7c5bd19f5c4f60ac9e5e63b3ff1ab	0	0	0	0	16	0	0	0	0	0	0	0
b4b834fdc2fad771fe364c31b2ca48d7	0	0	0	0	0	0	0	20	0	0	0	0
e2d982da0b50c952bb5f8db55a157aac	0	0	0	0	0	6	0	0	0	0	0	0
939ce90cecd29c4b468567d4833dbe90	0	0	0	0	0	0	0	0	4	0	0	0
419f5580c0b6a54a2d819cea618c9117	0	0	0	0	2	0	0	0	0	0	0	0
a69758e01dcfe2f5d89583ee991f11f1	0	0	19	0	0	0	0	0	0	0	0	0
0ddb875227341779fb616c09e02b7369	0	0	0	0	0	0	3	0	0	0	0	0
b06b7752ea8b31ecde03b23d8a454c51	0	0	0	0	0	0	0	0	241	0	0	0
5b8f8495f5c877c577b227f68c5119b3	0	0	0	0	0	0	0	0	0	0	5	0
ff2a374027b48e2a4484225395ced258	2	0	0	0	0	0	0	0	0	0	0	0

1e43bd6f56290688539e14dc41704e89	0	0	0	0	0	0	0	0	0	0	15	0
e669dbdf32ef941922b16f9a84b716b7	0	0	0	0	0	0	0	0	0	0	11	0
654a953b0c548a577b809bd3c4c7ef92	0	0	0	0	0	0	0	0	0	0	141	0
15a3a283c2524257134339b2133eaf75	0	0	0	0	0	0	0	0	0	2	0	0
6d348a927e548491bd5fac54569e8162	0	28	0	0	61	0	0	0	0	0	101	0
1d3c66f69ed7094a3ed8837c4b6ca641	0	0	0	0	0	0	0	0	0	0	2	0
6c8c7d614d12153206e4da3bd8d394cc	4	0	0	0	0	0	0	0	0	0	28	0
78316714b3b24257fe68506725a973b0	2	0	0	0	0	0	0	0	0	0	0	0
32d1279a02e5a0c95c8855b77783f0a0	0	0	0	0	6	0	0	0	0	0	0	0
2ad33a3a5840d888d5ccdcd15659982f	0	0	0	0	6	0	0	0	0	0	0	0
02b1b23b7d62aa95294b8a39457a2cc8	0	0	0	0	0	0	0	0	0	12	0	0
e1fdc155bc77874bf6ffe708ae43ed13	22	0	0	0	0	0	0	0	0	0	0	0
fd2ebf0ff6c8832846fd5e9c7bda2452	0	0	0	0	0	3	0	0	0	0	0	0
c8d41a095fd4ea70daad2a5f2a1ac2cf	0	0	0	0	0	3	0	0	0	0	0	0

322b92fcb8dd251324b6f0a7a5a70639	0	0	18	0	0	0	0	0	0	0	0	0
656e1045531d51bd94e19f727c1ebec1	0	0	0	0	0	0	0	86	61	0	0	0
def04943456a20196cbb433130c1e18e	0	0	0	0	3	0	0	0	0	0	0	0
a271ddd2624423f326739c52fcd9834c	0	0	0	0	0	0	0	0	0	0	2	0
31765ce6032b83f535dfabfb053fbe5c	0	0	0	0	0	0	0	0	0	0	0	4
86d01fa059c7cd1c4795a193756ff1f3	0	0	0	5	0	0	0	0	0	0	0	0
116ea1ddd92af956e19c569ccbca19a8	0	0	0	0	0	0	0	0	0	0	0	3
20f9ab8b2abd3729ee2688f9064d8f99	0	0	0	0	0	0	0	0	0	0	0	18
e9aa82c7484feb02068fe1cbac5a021e	0	0	0	0	0	0	0	17	0	0	0	0
add2388c5216d88e5149fcd061dcb2d3	0	0	69	0	0	0	0	0	0	0	0	0
6619e643fcb495b6e705cf5b2c6a6b54	0	0	0	0	0	0	0	0	2	0	0	0
298031fd09a1e0d85f385bae52839bed	0	0	0	0	2	0	0	0	0	0	0	0
ba78d8ec5ab756b666aa5da3980cab93	0	0	0	0	0	0	0	0	0	0	4	0
93e27e04433f39f114eb565547fdba11	0	0	0	0	0	0	0	0	0	0	5	0

3ec6f5185b21216148bf2beed9d990ea	0	0	0	0	0	0	0	0	3	0	0	0
e77c0c06353dd42d00d51248e2120a1e	0	0	0	0	0	0	0	0	0	0	81	0
5a764ebc1a81fee7fedac182798f4a92	0	0	0	0	183	0	0	0	0	0	0	0
458c8acb7bbeff48cbf7bd375f8d9edf	0	0	0	0	0	0	0	0	0	0	0	14
05f060bbc22e3b15129c72d7c11bc451	0	0	0	0	0	0	0	0	0	0	6	0
57c27f90be1f3e0dbc178a600d8b9f0a	0	0	0	6	0	0	0	0	0	0	0	0
fcf528b07b83f38b3b2a2d9d07569625	0	0	0	0	0	0	0	62	0	0	0	99
c1d6d630dbbdd3ddc783308e3461b035	0	0	0	0	0	0	0	3	0	0	0	0
e8d968091dfc56c85c80082196b87322	0	0	0	0	0	0	0	8	0	0	0	0
b9bc9ea6d2e46fcdbc1e2561273be9ab	0	0	0	0	0	29	0	0	0	0	0	0
f59ce30645f454c543bf0efcfac8b650	0	0	0	0	0	0	0	0	0	16	0	0
31ae451688d2bbf82ce1edc473194c07	0	0	0	0	87	0	0	0	0	0	0	0
482ca67cbd9a40cec915ff207ba30bc5	0	0	0	0	0	0	0	0	0	0	4	0
2bfd3145ae003954bccd75b4027fe248	0	0	0	0	0	12	0	0	0	0	0	0

ae91194263451c63cc83adb960889fd0	0	0	0	0	0	3	0	0	0	0	0	0
94b075bd6b0e46161a4bcd30597adf1b	0	0	0	0	0	0	0	0	0	2	0	0
523c3ea5b67e497b94b0c03eaff4c0f0	49	0	0	0	0	0	0	0	0	0	0	0
e2db14b921962294e2e7bd71113d433f	0	0	0	3	0	0	0	0	0	0	0	0
0ae946969c1a173469aba9093822e57a	0	0	0	0	0	3	0	0	0	0	0	0
9924768cb6a61ba78a932e56ee20212c	0	0	0	0	0	0	0	0	0	0	0	3
6ba8ad5a374d3b4b35288f7089f93cd7	0	0	0	0	0	0	0	0	0	0	0	6
cb5515f3b3a6c80820b2ec48c8dc6223	22	0	0	0	0	0	0	0	0	0	0	0
bbfd5b32a4c9734c5613964cdebd52b4	0	0	0	0	0	0	0	0	11	0	0	0
7da4e9b8612dabbd62d7fce31d8b615e	0	35	0	0	0	0	0	0	0	0	0	0
35a523adeb30ac2e5cd5096206eee178	0	0	3	0	0	0	0	0	0	0	0	0
4cfe5f270f249b7e12593fa0c8696ae5	0	0	0	0	0	0	0	0	0	0	4	0
12699771eb2757f97b08b9d868479b9c	0	0	0	0	0	0	0	0	6	0	0	0
5b2794a96c2e1919fd2e70f3915a2768	0	0	0	0	0	0	0	0	2	0	0	0

5f5b76adf1e3f03f93474e7ff4ff6288	0	2	0	0	0	0	0	0	0	0	0	0
bc9c60595ff933a5fbc0d02d0b4529b9	0	22	0	0	0	0	0	0	0	0	0	0
2fd574fad3b2a466cdd7980d7c25494d	0	0	0	0	0	0	0	0	0	0	0	30
a808b84a237f8b9c6e985171e9a7a40c	0	0	0	0	0	0	0	0	12	0	0	0
0e8e42431535ce6a660a1ef161872910	0	0	0	0	0	0	0	0	0	0	0	2
25b7fdbf36d0bab1849f34f69bc16edc	0	0	0	0	0	0	0	0	0	0	11	0
bb1fafc6c68e3b7741d27835598b26c8	0	0	0	0	0	4	0	0	0	0	0	0
d5ea3726dd788774090f72cd55fc1942	0	0	0	0	0	0	0	0	0	2	0	0
e39780be17824736adb7a6886aee6603	0	0	0	0	0	0	0	0	65	0	0	0
2012076712f843ad1dd97397b6381864	0	0	0	0	0	0	0	8	0	0	0	0
b3ed86a01d64449a8a7bb5473e14cc7a	0	0	0	0	0	0	0	0	0	0	0	10
140d7f53b0723d9796c7ee4d9e02f63b	0	0	0	0	0	0	0	8	0	0	0	0
27416ac5ea7a5135553feb94371aaa32	0	0	0	0	23	0	0	0	0	0	0	0
c4bffd5cebcdcf9ebf037c267ae3d36e	0	0	0	0	0	0	0	0	0	0	0	36

6b2b0e4d81985abd2c19968d1ac07c62	0	0	0	0	0	0	0	0	0	0	2	0
511969bdfed6d882102ee035fd69f241	0	0	0	31	0	0	0	0	0	0	0	0
3e8ae4486c4b7c831689f7967d5b44a5	0	0	0	0	0	0	0	0	0	0	0	6
0f926e9705c9941d995fc56a36e94c1c	0	0	0	0	0	0	23	0	0	0	0	0
b7bbc7260a624b7875a2ea5215cab372	0	0	0	0	0	20	0	0	0	0	0	0
7897c7ae1bf7b440a6b4475277afbf2b	2	0	0	0	0	0	0	0	0	0	0	0
2ecc0ac1012a2dbf222c64f907deee8c	0	0	0	0	0	0	0	5	0	0	0	0
db11c34ff7836e83b5d4c1d5e839444b	0	0	0	0	0	8	0	0	0	0	0	0
282d8f8ac41ccaf04cca23a0d82f121a	0	0	0	0	0	0	0	0	0	0	3	0
072b0a05a8ff280c11e42b323823acca	0	0	0	0	0	2	0	0	0	0	0	0
e558ffa73948365b005052b4bfbbcdad	0	0	5	0	0	0	0	0	0	0	0	0
4fbfad07307db2dc76d4d4295f9bd4a3	0	0	0	0	0	0	0	0	0	0	5	0
13b85125c36bf29348f8c8fd92b7d171	3	0	0	0	0	0	0	0	0	0	0	0
215e70cd20c1fd15f1f4934d1b6f3fbf	0	0	0	0	0	0	0	0	0	0	2	0

ca9280ffee1e554138bfa630c97d861a	0	3	0	0	0	0	0	0	0	0	0	0
411a6cf86064ef215e78ddeb62cdf287	0	0	0	11	0	0	0	0	0	0	0	0
074b5fc4218e79643e0190f75bf2f2f0	0	0	0	0	0	0	0	0	0	0	5	0
19d010d1fa81b53d77151c63ea943187	0	0	0	0	0	0	0	0	0	0	5	0
4918c3234acefe7264a50b0f27f441a6	0	79	0	0	0	46	0	0	0	0	0	0
8617a3fdb95d63f62db7db2d2b5484df	0	0	0	0	0	0	0	0	0	0	4	0
ab430f24667eeed9c86e18a84b85d222	0	0	0	0	0	0	0	0	0	0	8	0
d7580a6f2e88b1b8b72c2409117c45a7	0	0	0	0	0	0	0	0	5	0	0	0
bed5a2360f48953f161f955f8c4507cd	0	0	0	0	0	0	0	11	0	0	0	0
57bc7f94460d6886fe8cdf5b64e88c7a	0	0	0	0	0	0	0	0	121	0	0	0
d71cebefd5df4ed65c39c480990797f5	0	0	0	6	0	0	0	0	0	0	0	0
944b8ff49b12e3973e9e676cad15222a	0	0	0	0	0	0	0	5	0	0	0	0
d0e6fa3644806db3843ea34dcba80769	0	0	0	0	0	4	0	0	0	0	0	0
b6a96a64c3c231fbbd005c09d2585f6f	0	0	0	0	0	0	0	0	0	0	33	0

a69e0ed9b5edfe9addeb3d712d675582	10	0	0	0	0	0	0	0	0	0	0	0
84df060e6d3b533d9629ac3f92de3dfa	0	0	0	0	0	0	0	0	0	0	75	0
1275e03c6ada2f9a06c6166896d9ebb2	0	0	0	0	0	0	0	0	0	0	9	0
c7590317c29d4f3b6003c7420de8c591	0	0	0	0	0	0	0	14	0	0	0	0
ef280dcb859ab55c3fac535103cefead	0	0	0	0	0	0	0	0	0	7	0	0
4234fa29be73b671d64c72d757147e0f	0	0	0	0	0	0	0	0	0	0	0	6
dc8d3e79537f89ff95afc8d45216bf78	0	0	0	0	0	0	0	0	0	0	11	0
b8e0c388702ed38e8392daa3d2c05dc2	0	0	0	58	0	0	0	0	0	0	0	0
96d7ebaad38083edbf3775602ba7b000	0	0	0	0	0	0	0	0	0	4	0	0
7b8051fb08126fdd0ec63c32faca5e7a	0	0	0	0	0	0	0	0	0	0	0	3
05c12483b597ed5624e981b2d999360e	0	0	0	0	0	0	0	0	0	37	0	0
b1fa7e2fc55fe5f5e18b936cebe8e0a9	0	0	0	0	0	0	0	24	0	0	0	0
1ae3d4c7b8575cb7cb63fde887dba16e	0	0	0	0	0	0	12	0	0	0	0	0
300d0ee5972851564f51e7d235c86c17	0	0	0	0	9	0	0	0	0	0	0	0

56c68ba863a353247425ddc1e49859a5	0	0	0	0	41	0	0	0	0	0	0	0
12917f174b75da0bdbb1331829ec3d57	0	0	0	0	0	0	0	2	0	0	0	0
b4f87156bb5b60e5f08a04f2ef7478de	86	0	0	0	0	0	0	0	0	0	0	0
b31713ad3fd27204803dbeedb6ec5cf3	0	0	0	0	0	0	0	0	2	0	0	0
036987f3e32ba355606ebff983b44333	0	0	0	2	0	0	0	0	0	0	0	0
df8dff8a1ad25837f42438762779a57a	0	0	0	0	0	5	0	0	0	0	0	0
4a0c0ad9d61d07b14ffb8b37894beae2	0	0	0	0	0	0	0	20	0	0	0	0
3ca968798ffca320bf2cc2faf2ab4e68	0	0	0	0	0	0	0	2	0	0	0	0
9e360de02256e1f64bff5387eeafd052	0	0	0	0	0	0	2	0	0	0	0	0
5ebaec786b838fcd6fc783f12791a15c	0	0	0	0	0	0	0	0	0	5	0	0
9d29a98b48f7a32acc1ee7151d3d36dc	0	0	0	0	0	0	0	0	0	0	5	0
d343c243eb8dbd814147101af6202a31	0	0	0	0	0	0	0	0	0	0	9	0
b7421a20716c11e8421916c5aade42be	0	0	0	0	0	0	0	0	0	0	3	0
edfb7669f27e734fdf846edb57800807	0	0	0	0	0	0	2	0	0	0	0	0

a89a114336237961f0bc851551f730a5	0	0	0	0	0	0	0	0	0	11	0	0
ddc0c7119657ef728d6111b4fba1eedb	0	0	0	3	0	0	0	0	0	0	0	0
d670278be5044bfb6881c54391f5df8a	0	0	0	0	0	0	0	0	0	0	0	2
20b41800e7313ff2657ed5ec14ce955a	0	0	0	0	0	0	0	0	0	0	0	4
13a09e5ff369e206c069af193cc03074	2	0	0	0	0	0	0	0	0	0	0	0
b151e141ba8539dacf1c41fb3ac83871	0	0	0	0	0	0	0	0	0	242	0	0
8a9800a7223bae99e46def76e8389d92	0	0	0	0	0	0	0	9	0	0	0	0
98f63073ddf4aacd736310314d087251	0	0	0	0	0	0	0	0	0	4	0	0
c76937eedfaa8b3a6e0b97309159acd0	0	0	0	0	0	2	0	0	0	0	0	0
ffd9d160bae73b1e3b790b05a1094b5a	0	0	0	0	0	0	0	0	4	0	0	0
fa9f7b5c43d948943f8b87392531021d	0	0	0	4	0	0	0	0	0	0	0	0
d9ba6e4321e832cbceba91e91c20fe3a	0	0	0	0	0	0	0	0	0	0	5	0
f2b669e0ff1a2ad062c2a76918f39596	0	0	0	0	0	0	4	0	0	0	0	0
eff1e7a7b6e063b3265b2dbfdc2a1992	0	0	0	0	0	0	0	0	0	7	0	0

43a48a8c47567c98fb616e11c0b6064e	0	0	0	0	0	0	0	0	0	4	0	0
20db3c716f2c7a7da3b77556bfc0db47	0	0	0	0	4	0	0	0	0	0	0	0
a34fbad128627a94d0c05519c61759a7	0	0	0	0	0	0	0	0	0	149	0	0
ef2fdc50795dacf07a0297a04b0e72a7	0	0	0	0	0	0	0	0	0	2	0	0
a31b3c8ca2784548062de27f8b6c1381	0	0	0	0	0	0	0	0	0	0	0	2
cc80c6f7db61a2e4cf28d73d2d724e66	0	0	0	0	0	0	0	0	0	21	0	0
aa73c452149b34368378a1902d002f2c	0	0	0	0	0	0	0	0	0	0	0	3
da90ac9fa2cbb1e1a915e23e505efe03	0	0	0	0	0	0	0	0	0	0	197	0
5aaed011ecf5c76c6a707595a1a5309b	0	0	0	0	0	0	3	0	0	0	0	0
cbb5a142921f1c8e757464b22db0f0a0	0	0	0	0	0	0	0	0	0	9	0	0
f102e2b540b6230e73bf9478463c966e	0	0	35	0	0	0	0	0	0	0	0	0
b23a9e8aab05adf258728f817092df9b	0	0	0	0	0	0	4	0	0	0	0	0
e7b7bfcb05007b05707ca90c5463ad41	0	0	0	0	0	0	0	0	0	0	2	0
6bece814e8d7011d9a4f153a49301613	0	0	4	0	0	0	0	0	0	0	0	0

6796e5aae6ca5e08c6230244357437a6	0	0	0	0	0	0	0	3	0	0	0	0
e4f1687ba5464dfe1b779c4f0edde277	0	14	0	0	0	0	0	0	50	0	0	0
3199a8235222c6e433d7d6f267b2ee5e	0	0	0	2	0	0	0	0	0	0	0	0
8e3e0c01cd0acf78b6324c12b02c019b	0	0	0	0	0	2	0	0	0	0	0	0
99e979c63eebab9c60ea6c027b0feed7	0	0	0	0	0	0	0	0	30	0	0	0
f5776a7067ff1437838d3cb23b1c69a6	0	5	0	0	0	0	0	0	0	0	0	0
2f07852993d455796912231e35772f48	3	0	0	0	0	0	0	0	0	0	0	0
0f4c62bffdcd74adf63029d70fe9a1f1	215	0	0	0	311	0	0	0	0	0	0	0
f1d3b7b17b13c68a03c05c45b9cabb98	0	0	0	0	0	8	0	0	0	0	0	0
c904eba98f4b070d5be50a14a531e84c	0	0	0	0	0	6	0	0	0	0	0	0
fde7910d80891dd103b0a9aa806bd1a1	0	2	0	0	0	0	0	0	0	0	0	0
5656239af31286f4682e7373b31fe9e0	0	0	0	0	0	0	0	0	0	0	16	0
fc7d30a8ccf0567b9d030b64694caab3	0	0	0	0	0	0	2	0	0	0	0	0
e210a82a05697c5cacebd4a040360930	0	6	0	0	0	0	0	0	0	0	0	0

e00e26e59a0bf15f785f5dd6772f42a5	0	0	0	0	0	0	0	8	0	0	0	0
a835db557f7893acfb92fca12f07c7b1	0	0	0	0	0	0	0	0	25	0	0	0
cd888c0426b048bb836cee12e78c379f	0	5	0	0	0	0	0	0	0	0	0	0
2c332f23890a30876095d2d856493857	0	0	0	0	0	0	0	0	0	0	44	0
c4345d63db94d142467f6a7fbf0fa1d4	0	0	0	0	0	0	2	0	0	0	0	0
5f3700e28cf9df07b878f6de4472c79c	5	0	0	0	0	0	0	0	0	0	0	0
35faccc4d05b9372d65d1a629cb73eef	0	0	0	0	0	0	0	0	0	6	0	0
df552b42a82b99b097d42e8f0e858dfc	0	0	0	0	0	0	2	0	0	0	0	0
c6251c54b50d461338bd3a539d13707c	0	0	0	0	0	0	0	0	0	0	0	2
8a5230f2e2e93155655cf37f354e49c1	0	0	34	0	0	0	0	0	0	0	0	0
f420096992407d1fe504e31f84052829	0	0	0	0	0	0	0	0	0	0	18	0
d7261a57cd02ef5baa5731b329915d91	0	0	0	0	2	0	0	0	0	0	0	0
a3234673f0724a1dbc5f1ed0d9d7395d	0	0	0	0	0	0	0	0	0	0	0	6
b7f164b9108aab6340f0014229baee3b	0	0	0	0	0	0	0	0	8	0	0	0

b46c493e9aab89482ba51fef1dadcdcd	0	0	0	0	0	0	0	0	251	0	0	0
6c1b0dc68a65711405dffbd607fa904e	0	0	0	0	3	0	0	0	0	0	0	0
31bbec542e4fd7894ac88c13122d7ff7	0	0	3	0	0	0	0	0	0	0	0	0
d0d0063d436c5f0e04fbd21324764703	11	0	0	0	0	0	0	0	0	0	0	0
bc3c3a0ff278cbdbc3b23f89e7811b29	0	0	0	0	0	0	0	0	0	0	3	0
1878c03d196c331036dc1bb8ae4a66ee	0	0	0	0	0	63	0	0	0	0	0	0
169cfd868b88df572a3896f44754a89e	0	0	2	0	0	0	0	0	0	0	0	0
ff2510209e335dbf515f57e460bdd062	0	0	0	0	0	0	0	4	0	0	0	0
610dffda511f331d18b36f6c3ecca2e2	0	0	0	12	0	0	0	0	0	0	0	0
a507ee372518a8cf6df75eed877dea22	0	0	0	0	0	0	0	0	0	22	0	0
c599bda0b1387aa30844b32cfb9ff95f	0	0	0	0	0	0	0	0	0	0	0	14
c6c06d49540571c132dbaf6502f74766	0	0	0	0	0	0	2	0	0	0	0	0
a9b3809c99fc387644976cd949a6cc77	0	0	0	0	0	0	0	0	0	0	4	0
cbd913610225100193f6fa7c430e7cd1	0	0	0	0	2	0	0	0	0	0	0	0

191092d3f7e05fbe12c41ff14078ece9	0	0	0	0	0	0	0	2	0	0	0	0
0a24942f13d7e5bea6e51d5274d36bbc	0	0	0	0	0	0	0	0	2	0	0	0
c810bae53c60cfb1a067a25047e8d6f5	0	0	0	0	0	0	0	0	0	2	0	0
12f0d54c126996fdfcdaf524435f6dee	0	0	0	0	5	0	0	0	0	0	0	0
9a67afe674984968a128893a0378af21	0	0	0	0	0	0	10	0	0	0	0	0
259d48e084353569523b5b0c9fe745db	0	0	0	55	0	0	0	0	0	0	0	0
46e834aa7f75de136de6991273f73bfc	2	0	0	0	0	0	0	0	0	0	0	0
587299c6147a82353de7a8a1b0e231ac	0	0	0	0	0	0	0	0	0	0	0	3
2c7bb97e7782e9c0e01ba0d2bee93ec9	0	0	6	0	0	0	0	0	0	0	0	0
330a5683cf2a72795de5f92d19973c7d	0	0	0	0	0	0	0	5	0	0	0	0
f2a5b789bd63e1b322eba00de4cb9721	0	0	0	0	0	0	0	0	0	0	0	3
d4ae62ebfeb41167d645d1f95377a895	2	0	0	0	0	0	0	0	0	0	0	0
b2d1158775be1a60822b6068fdebf386	0	0	0	25	0	0	0	0	0	0	0	0
1d3b939fdb272e7f24b9bbafc6f8b5dd	0	0	0	0	0	0	0	0	0	0	13	0

41b823506ed85546e47b671872b02a03	0	0	2	0	0	0	0	0	0	0	0	0
9ceb8792a708eedba4ceec0ca833d99f	0	0	0	0	0	59	27	0	0	0	0	0
5a52e4345dba3dadd900c5567efce4f6	0	0	0	0	0	0	0	0	0	40	0	0
2d7ac972198edd7af5160760d97f581e	0	0	0	0	0	9	0	0	0	0	0	0
cb7888056fc761ba9233accae6e9fa18	0	0	0	35	0	0	0	0	0	0	0	0
32eb2b151a44363c74be2fa127787704	0	0	0	0	0	0	0	0	0	0	0	8
088ab957e9bb239c5334c667ffe8add8	0	0	0	0	7	0	0	0	0	0	0	0
5b3301f8085a24a323a4262fdccf5ea7	0	0	0	0	0	0	0	0	0	7	0	0
6dfe8967032099f9ee800c3e076b8c47	0	0	0	0	0	0	0	0	0	0	0	17
b7b18234baa840501d2234655b5c8a59	0	0	0	0	0	0	0	0	0	0	0	3
6b448f4cb777bed10676acc6cc0ce39c	0	0	0	0	0	0	0	0	4	0	0	0
a49b3dffbe06bfa09005f3be84a6c037	11	0	0	0	0	0	0	0	0	0	0	0
ddc1ac294489b30e2df22a78a56aec59	0	4	0	0	0	0	0	0	0	0	0	0
3ce1bf7a374cf3cee139d2834974ee2f	0	0	8	0	0	0	0	0	0	0	0	0

31b0b2c19ff908ac5bede0cf5 b6822c5	0	0	0	0	13	0	0	0	0	0	0	0
c2f3237f6bd9381508ec5045 13d37035	0	0	0	0	0	16	0	0	0	0	0	0
4c704bccff6c4892e68946a6d 2af5241	0	0	0	0	0	0	0	0	0	0	3	0
5bf24a815fafe8c436b9c9fcb e6fa530	0	0	0	0	0	0	0	30	0	0	0	0
bec7ed7b620901cec586cdeb 52c77206	0	0	0	0	0	0	0	0	0	0	0	4
5bfeed2cba284914daa0ecec 082dc2a6	0	0	0	0	0	0	0	0	0	0	0	2
c040cbd819eeeedfb49d7ad5 a433d5ea	0	0	0	0	0	0	0	0	0	0	0	24
9c2eba7022092d14bb3d3efc 1c18f694	0	0	0	0	0	0	0	0	0	0	24	0
02b9ef1c910f1ea965adf89c5 354f81b	0	0	0	0	0	0	0	0	0	0	71	0
d6b03ac38a781edbd649ee3 4fd0563cf	0	0	0	0	0	0	0	0	7	0	0	0
348c74ee774a8da7699d63e 4adc36fae	0	0	0	0	0	0	0	0	0	0	90	0
a57e903bfbb0b2ca8d10d654 b813344a	0	0	0	0	0	35	0	0	0	0	0	0
bbd2968def8b03e9db0829e b80210d25	0	0	0	0	0	0	0	0	0	3	0	0
28c98b7a0d47ea5cddf0cfb4 d605be64	0	0	0	0	0	2	0	0	0	0	0	0

e71179e77529dab414c68ea b77f9f621	4	0	0	0	0	0	0	0	0	0	0	0
c9f4436727e6c49cde5d2d70 04537868	0	0	0	0	0	0	0	0	0	2	0	0
39777c13ba0c1198765bc2a8 2d1bba26	0	0	0	0	0	3	0	0	0	0	0	0
e33ae9f8759ccddd01257424 54d4bf59	6	0	0	0	0	0	0	0	0	0	0	0
e08d86b486bd1396f4842f8c a95f88e3	0	0	0	0	0	0	0	0	0	0	4	0
7cc4de7d3409dab09de83bd 9cb7c3531	0	0	0	0	0	0	2	0	0	0	0	0
90e6da9e1a8f6ad8f9773453 50d2e9c7	0	0	0	0	0	0	0	0	0	0	0	8
ae1b3a472b6e30ca83a0180 df641a4ef	0	0	0	0	0	0	0	0	0	0	0	2
cb8ed38b653c1a1bd585e1bc 2736682a	0	0	0	0	0	58	0	0	0	0	0	0
7b1f1f5b0b226f10848310c9 50ee56ab	0	0	0	0	0	0	0	0	22	0	0	0
57fcf3080d357744113268f38 141b0a0	0	0	0	6	0	0	0	0	0	0	0	0
6c438275d2b4a4c295b9519 977fad6b7	0	0	0	0	0	0	0	0	0	0	6	0
1614ea85dddbf321fd1a877e 9075ce58	0	0	0	11	0	0	0	0	0	0	0	0
758e9a552d8934024c5c8773 d84407aa	0	0	0	0	0	0	41	0	0	0	0	0

f8cf604ae76867d4e3440b71f7a6ca69	0	0	0	0	0	0	0	0	5	0	0	0
3c73b51bb411e03f7e73441171732b8c	0	0	0	0	0	0	0	0	13	0	0	0
3fd213ec806a8ed11c679d50a343efd1	0	0	0	44	0	0	0	0	0	0	0	0
20ed63be29bb780219e8d0df8209fef6	0	0	0	0	0	0	0	0	0	7	0	0
3b8846ed0d21984ab378593cb4a0ef3f	0	0	0	0	0	0	0	0	0	0	4	0
dc14d20bc1bc9ff4afe0094fe47ce07c	0	0	0	0	0	2	0	0	0	0	0	0
82668141b643ba328ea5295f2e9b4a2d	0	0	0	0	0	0	0	0	46	0	0	0
b94d99464f623e53cfe16a9f0cde9f64	0	0	0	0	0	83	0	0	0	0	0	0
9090360e40cc6ceaffd9c86d25143f0f	0	0	0	0	0	17	0	0	0	0	0	0
7706a68dd09f582dd702727554198cde	0	15	0	0	0	0	0	0	0	0	0	0
d2e5e11ffade4e0f9c8e343c6a35238b	0	3	0	0	0	0	0	0	0	0	0	0
0ce08d9e49675d52878e6e347c246b9b	0	0	0	0	0	0	0	0	0	0	6	0
6983f1844fcf3c818483d42d9d15445d	0	0	0	0	0	0	0	0	20	0	0	0
af93a8a1714719fe867e91aad3abf524	0	0	0	0	0	6	0	0	0	0	0	0

22fa6f224fca5f290853350f41ff0da3	0	0	0	0	0	0	2	0	0	0	0	0
6d6cac370125dbbb893667c1a1e21459	0	0	0	0	6	0	0	0	0	0	0	0
59d8fab490a039f55a912c6ea74e1a9f	0	0	0	0	0	0	2	0	0	0	0	0
a557edcfe9c3162d6a1eda1f91a2486d	0	0	0	0	0	0	0	0	0	0	5	0
c11a85ec5aed5f7f7c420149eaa66333	0	0	0	0	0	0	0	0	0	0	5	0
e087ea25d15771492b1126af96f5b997	0	0	0	0	0	0	0	0	20	0	0	0
cbca0fc94b49d29762558cc50dac9b5d	0	0	0	0	0	0	0	0	0	0	7	0
8b9e6bb263b3de7286b829f7b21bee83	0	0	0	0	0	5	0	0	0	0	0	0
7293fc6956fc0481d15f4b2752ca5cb4	0	0	0	0	0	0	0	0	0	0	4	0
4e46c29ab6e02ea9a5cb0e8d554e5f22	0	0	0	0	0	0	0	0	0	5	0	0
4f9f4c601d623651993ccb71bb5fc71f	0	0	0	0	0	0	0	0	0	5	0	0
52228ca3f2fb286472f8860c213908a3	0	0	0	0	0	0	0	0	48	0	143	0
95bf70419e6a6b09a7fefb320cfecd2f	0	11	0	0	0	0	0	0	0	0	0	0
c9c4dd0866f349adfb58ecb5a057a988	0	0	0	0	0	2	0	0	0	0	0	0

0b055f18d1368480151585e69a6ae89e	0	0	0	0	0	0	4	0	0	0	0	0
52176a2d8064b289cd308353ebcdccdd	0	0	0	0	0	0	0	0	0	0	2	0
ce8e3c5fbb31aab1cb5ff96e976fe814	0	0	0	12	0	0	0	0	0	0	0	0
8d47fb2fdc1c2355677897b6d581cd77	0	0	0	0	0	0	0	0	0	4	0	0
8092a36efb1d00854c7c1315e9a1fe7d	0	0	0	22	0	0	0	0	0	0	0	0
6d3cd2bd0bf01916b0c1773d1792eaa5	0	0	0	0	0	0	0	0	0	0	3	0
1d3a7b3b8b64f58672a93d5fc3fb2786	0	0	0	0	0	0	0	3	0	0	0	0
e11ea166c4c904339455418ce9aea7ea	0	0	0	0	0	0	0	0	0	0	27	0
2d9cad67102c233f308cc5ea74b14505	0	0	0	5	0	0	0	0	0	0	0	0
d3dd7f121939246f623ab145cc730dfa	0	2	0	0	0	0	0	0	0	0	0	0
730712a7df6172e462362ec8f9b1ca2b	0	0	0	0	0	0	0	0	0	66	0	0
6366a2772ce663f4c8ee0f9f336679f5	0	0	0	0	0	0	0	0	0	0	0	10
c0097b30c1a8562343b816b78826596e	0	0	0	0	0	0	0	0	0	0	0	4
5d25750e114f918b17e15e5859b376e4	0	0	0	0	0	0	0	0	0	0	4	0

7b6247ef3dc8005192bde232 8b2e0546	0	0	4	0	0	0	0	0	0	0	0	0
be27e5578fa8ed235d1d6db 4751f12b1	0	0	0	5	0	0	0	0	0	0	0	0
29d984dc1de0e72d5c0aac8b dd96f80e	0	0	5	0	0	0	0	0	0	0	0	0
fe7896c7dde0bc538776a16b 71a923d2	0	0	0	0	0	0	0	0	0	0	0	24
5784474296accff173189904 24f44a10	0	0	0	2	0	0	0	0	0	0	0	0
e8ecd0054441d7a6186254d d779145b6	0	25	0	0	0	0	0	0	0	0	0	0
8201c9b9c442cb726ff26ce16 1786551	0	0	0	0	0	3	0	0	0	0	0	0
99ff92afb0a5567138bd1e6d 078f7bbc	0	0	0	0	0	4	0	0	0	0	0	0
8b005a431d8cdffc39361eb0 4211c5df	0	0	0	0	0	0	0	0	2	0	0	0
bbda2c4f80e5ab6e8c1393b6 60bf36ae	0	0	0	0	0	0	0	7	0	0	0	0
bce2713e0eb37db43250a48 968929728	0	0	0	0	0	0	0	0	0	0	11	0
517155c42756a69d5bae201c 1d7c0106	4	0	0	0	0	0	0	0	0	0	0	0
9fb2321a92d2025264b4b12 b0aa91c44	0	0	0	55	0	0	0	0	0	0	0	0
4c23f7f34f4f894932d83bc20 17e24d6	0	0	0	0	0	7	0	0	0	0	0	0

add76ce5d7112f19fc7070ee8c4dffaf	0	0	0	35	0	0	0	0	0	0	0	0
308856e7fb391aabb63420d9e9bd0d21	0	0	0	3	0	0	0	0	0	0	0	0
6c4695d801d13582a3320dcd058a5225	0	0	0	4	0	0	0	0	0	0	0	0
eb87cd1e69cbb4f3d5a51b2e9929fcd2	0	34	0	0	0	0	0	0	0	0	0	0
da3e83dce2934e91b42bd2724fd2bc5d	0	0	0	0	0	0	0	0	0	42	0	0
94322b2a8a5ddfe69d3cdd79016143af	0	9	0	0	0	0	0	0	0	0	0	0
9b766bea2b4a5365892e355973af80eb	0	0	0	0	0	0	0	0	0	0	0	2
120e89dd4382c0ebe4766c049c9296ce	0	0	9	0	0	0	0	0	0	0	0	0
78f766285bbdfa13d3479fd2cab1b51d	0	0	0	0	0	9	0	0	0	0	0	0
234df7dd9bc27917e6a25e6c83ec5274	0	0	0	0	0	0	0	9	0	0	0	0
994055efaef98d7b3a495bc08904fbf5	0	0	0	0	0	0	0	0	0	0	0	8
d067e5266d4fd638e917f4f0783801ac	0	0	0	0	0	0	0	0	0	0	0	9
29f365d93a6ec67febfb461a6d1377e1	0	83	0	0	0	0	0	0	0	0	0	0
d85da9cd12a32de7c93a28b4ba059f6a	0	0	0	0	0	0	0	0	0	0	0	2

26f7789a7cdf4aea2698030974507557	0	0	0	0	0	2	0	0	0	0	0	0
e784bab55bd1451cd47e1a7b94c289f9	0	0	0	0	0	0	0	0	0	0	8	0
66b178631af63181bf077e4d5425d757	0	0	0	0	0	0	0	0	0	0	0	4
7143900d3ac2984cd218424806825b63	0	0	0	0	2	0	0	0	0	0	0	0
11d101d921590fe36c28d6c9e2098cb4	0	0	0	0	0	0	0	0	0	0	8	0
bbf42f2fb625018f4c8a06813adb18e4	0	41	0	0	0	0	0	0	0	0	0	0
454dd00872f5001115503dc8190be60b	0	0	0	0	0	0	0	0	0	0	156	0
f4191ae67fb8f48ce5b27227f49165e2	0	0	3	0	0	0	0	0	0	0	0	0
6c002edc5403324a811b9a0e351002bd	0	0	0	0	0	0	0	0	0	0	0	2
c1c07dd3979f273fa6a1764b89048423	0	0	0	0	79	0	0	0	0	0	0	0
61211c59e6a525e9f988eec21f5a5d11	0	0	2	0	0	0	0	0	0	0	0	0
862aab3f266eca782478da5a461b0f60	0	0	5	0	0	0	0	0	0	0	0	0
11aa0c93281aabf0343ba5702dd4f03a	0	0	0	0	0	0	0	10	0	0	0	0
fc863bb658cc5e5ad56e19484ecb9d28	0	0	0	35	0	0	0	0	0	0	0	0

ae06f9f0021552de5fb1b390 8a15260d	0	0	0	0	0	0	0	0	0	8	0	0
7d81a8b367706d221363746 75ef8320a	0	0	0	0	3	0	0	0	0	0	0	0
1bb354e8746d3b5b084b404 ed8356615	0	0	0	0	0	0	0	0	0	0	7	0
ac3b93d8b0b6b5d4b590eca 6a6aa4fbf	0	0	0	0	0	0	0	0	18	0	0	0
03dab1e1eaa82da74746598 32f86b8a3	0	0	0	0	0	0	4	0	0	0	0	0
794445b2e9e95e9c4ee8efc6 d3ccbe09	0	0	0	0	9	0	0	0	0	0	0	0
8312b63305e0c3d6f1db626b b1b3de08	0	0	0	0	0	0	0	0	0	5	0	0
1e00407e5e2948748bf9459b 4d977b96	0	0	0	0	0	0	0	0	0	3	0	0
6fcb0542197a78f4e99ed13ef d4adddb	0	0	0	0	0	12	0	0	0	0	0	0
38bb9668467c02e30f56709d 2e03c3fb	0	0	0	0	0	0	0	0	0	0	18	0
efb0af4cc31bc347fe33a9a5d 1f952d7	0	0	0	0	36	3	0	0	0	0	0	0
f70b840167be6e6fe515e41e 0336f636	0	0	0	0	0	0	0	0	0	0	3	0
1547cf2aec936bb1bd5a8e2c 9722d7cf	0	0	0	0	142	0	0	0	96	0	258	47
ba211ad3d90a5b8e29ef3c22 32c6c5f0	0	0	0	0	0	5	0	0	0	0	0	0

1fea5e799c7764a45f6b729e06b33b52	0	0	0	0	8	0	0	0	0	0	0	0
e9acf0def4f33dd3e735b068b6f29880	0	0	0	0	0	0	0	0	0	21	0	0
d5529592a022b5fbaa727b90bf816f23	0	0	0	0	0	0	0	0	0	0	2	0
a2e04977f00ff59dc90ea4f281edcd9b	0	0	0	0	0	4	0	0	0	0	0	0
1972c1755be1d197d0dfcef37c548a98	0	0	0	2	0	0	0	0	0	0	0	0
600163d240c6cfcf8ed89fc069c14530	0	0	0	0	0	0	0	0	0	0	2	0
fa1eb529f50c0dd12788c04547fb5ed4	0	0	0	3	0	0	0	0	0	0	0	0
0563965648467bf38984f56e85ac82e9	0	0	0	0	0	0	0	0	0	0	0	7
907c5631b9ed90a6f49944c9892175c4	0	0	0	0	0	0	0	0	0	9	0	0
be3c46abbfd8d92eef226ff89dbc9767	2	0	0	0	0	0	0	0	0	0	0	0
b3b0b46b19ee05413ddacbdb9cd71620	0	0	0	0	0	0	3	0	0	0	0	0
278dd12ff1b36b71f676446c53a08291	0	0	0	0	0	0	0	14	0	0	0	0
ff3313b6980bcf07e77e99e033e10653	0	0	6	0	0	0	0	0	0	0	0	0
d4f449e618ac09234feb1213847391b1	0	2	0	0	0	0	0	0	0	0	0	0

e89fbb5354b924b7ea8194e3a242a5b9	2	0	0	0	0	0	0	0	0	0	0	0
4ffb5c402b2caaacad2d5c421fe9af78	0	0	0	0	0	0	0	0	0	0	0	3
aed56d972ef15b5b517b7624f9fc07dc	0	0	0	0	0	0	0	0	3	0	0	0
ae939f863995b7c85ea8f1b74612205c	0	0	0	0	0	0	0	0	0	0	17	0
2656e70832354fb494dbb57dc58ae1fb	0	0	0	0	0	0	0	0	0	0	0	2
3efcec4b29a599e18ce4317a01d58a72	0	0	0	0	0	0	0	0	0	0	9	0
fb80561f3fd8ef17105b6d8e9e854dae	0	0	0	0	0	0	0	0	0	0	0	37
88db287fc3da379f8dc5d4fad67ad39b	0	0	0	0	0	0	0	0	0	0	0	3
837efda86df83c0e30c7cb66aa2478c6	0	0	210	0	0	0	0	0	0	0	177	0
807b7aaea165f11e624f3a64cd0a925d	0	0	0	0	0	0	0	0	0	3	0	0
fc6cfa4b831df3b55cde4586d402c73e	69	0	0	0	0	0	0	0	0	0	0	0
3655d8b68c57d49a2778ad3a3c19e1bf	0	0	0	0	0	5	0	0	0	0	0	0
c442a706bea318e83ce47bc9fb6f2878	0	0	0	0	0	0	0	0	0	187	0	0
1674513e015691e3a1d5d3c36b1ab057	0	3	0	0	0	0	0	0	0	0	0	0

fc7f9c1fcf3238d81ea1e95d9eb99d79	0	0	0	0	0	0	0	0	0	27	0	0
f1cb897234b63df0612e00ed6e25b062	0	0	0	2	0	0	0	0	0	0	0	0
7998799d87afd676c97e5500cb5180ac	0	0	0	0	0	5	0	0	0	0	0	0
8ee409aad7b90f432b57856531c036e8	0	0	0	0	0	0	0	0	0	3	0	0
72ab9bb6b0bef1517a10710983ceff64	0	0	0	0	0	0	0	0	0	0	0	14
f9713178ed75768163171ae59ac35346	0	0	0	0	0	0	7	0	0	0	0	0
1f796779f92703f936c5ff0c898e9aa9	0	0	0	0	0	0	0	0	0	20	0	0
1a650ec14fa333c3653e6f8f88e0c9ee	0	0	0	0	0	0	0	0	0	54	0	0
19d6e6faad1309c79a79cec623dacc7a	0	0	0	0	12	0	0	0	0	0	0	0
88519fdbf2179144b952bb4d1a278dec	0	0	5	0	0	0	0	0	0	0	0	0
c36e0da9ba40e164ae74a2c28bb00281	0	0	0	0	8	0	0	0	0	0	0	0
af964d9d1ca815f03dc13312794d1cc4	0	0	0	0	0	11	0	0	0	0	0	0
8f651e56ec8d42c076cecdec4d55fe0e	0	0	0	0	0	0	0	0	3	0	0	0
a97913889fe311d9d1c72550d427e05b	0	0	0	0	0	0	0	0	0	5	0	0

fb7ffe84851f816d8d7ff99cb5c1009e	0	0	0	0	0	0	0	0	0	0	0	26
bf27daa73ea3f5a2035d45b113ab58b4	0	0	0	0	0	0	0	0	3	0	0	0
4316b521de5c59ab59a28601dd44491f	0	0	0	0	0	15	0	0	0	0	0	0
08dca2f3157a02d408f7b9b18858b3da	0	0	0	0	0	0	0	0	0	0	9	0
3b5194a19d304689de6c813b4d5e2988	22	0	0	0	0	0	0	0	0	0	0	0
94e19629e7660ae8b2e86ebdcedb1cf8	0	0	0	0	0	0	0	0	0	0	30	0
4ba5d06555dd48c1de2a42b797906e2f	0	0	0	0	2	0	0	0	0	0	0	0
d4ddf45f7265d06b3c2138b5c3e1f223	0	0	6	0	0	0	0	0	0	0	0	0
3f37118efd56860ff76b6286bd81148b	8	0	0	0	0	0	0	0	0	0	0	0
48966310a78db53fb0f183b2e8ae2ab4	0	0	0	0	0	0	0	5	0	0	0	0
af5849b8a1a1c8f83b889ff3dcc7de8d	0	0	0	0	0	0	0	0	0	0	0	11
d3adcebf554c04eb8f344d23deb4e166	0	0	0	0	0	0	0	3	0	0	0	0
e2a1ea474812f1fe5d7df55602778a5c	0	0	0	0	0	115	0	0	0	0	0	0
e002dd70746e193a002a6a741c6b06c0	0	0	0	0	0	0	0	0	0	0	46	0

75e52b77926d9aefea339991d5188ceb	0	0	0	0	7	0	0	0	0	0	0	0
a8dfa1e50c559039801f9496b331e7f8	4	0	0	0	0	0	0	0	0	0	0	0
5599b7c0db95ee79e262a661983d4a67	0	0	0	0	0	0	0	0	0	2	0	0
5e3f2ae84e01021588ae022cc26ffaa4	0	2	0	0	0	0	0	0	0	0	0	0
a71f26203a4ce9d36df2c841d99120b3	0	0	0	0	0	0	0	0	10	0	0	0
f04270d0a133754fbc02b1dd9696792a	0	0	0	0	0	0	0	0	0	0	0	2
f4a92b8b7a2a5075b32483e8995b1355	0	0	0	0	0	0	0	0	2	0	0	0
2d4a1f9858aafa9b8fe8e5e7d9fdbc39	0	0	0	0	0	0	0	0	0	11	0	0
7c7516f0a368f2141f4e2f5160d5650b	0	0	0	0	0	0	0	0	0	0	7	0
938ae804c6a82f8a863a89e94047428d	0	0	0	0	0	0	5	0	0	0	0	0
05acab8c81eb03721c28eccd0d9de645	0	0	0	0	0	99	0	62	137	107	169	0
ea35fe94f0291908a6eebda93e9eb583	0	0	0	0	5	0	0	0	0	0	0	0
511df1d114f975025c48fcfcc61459bf	0	38	0	0	0	0	0	0	0	0	0	0
d33087cccca5b9be3397945d4e98f10b	0	0	0	0	0	3	0	0	0	0	0	0

bbf29fd64e701419130b3a33 92c8d567	0	5	0	0	0	0	0	0	0	0	0	0
3206499d34ad8e7f5eaca019 19e4269e	0	0	0	0	0	7	0	0	0	0	0	0
1f04984cd2e0cc3884f48f5da 6ef852f	0	0	0	0	0	0	0	0	0	0	0	11
1f81d6f39f47d77a1e280a78 38f41f39	0	0	12	0	0	0	0	0	0	0	0	0
37fa6a17c06ca670f43139344 32add28	0	0	0	0	0	0	0	71	0	0	0	0
a67ceb9c6dc1db62d39b4d3 0c03fe4cd	4	0	0	0	0	0	0	0	0	0	0	0
e3b110171ff181b11cf7b41b bdeaa983	0	0	0	2	0	0	0	0	0	0	0	0
2a21a89203470f568be193df 7bf2b7a0	0	0	0	0	0	0	0	3	0	0	0	0
d2dfd1522b10aa8f3a15ff481 a84c2b9	0	0	0	0	0	0	0	0	76	0	0	0
d9fbcdb3fe45d625acc58de3 61ce6aff	0	0	0	0	0	0	0	0	0	0	8	0
1296c70e3734f877a423910a 21f170ac	71	0	0	0	0	0	0	0	0	0	0	0
48c92689de1b346b0282e69 8f973b931	0	0	0	0	0	10	0	0	0	0	0	0
d163c8dee1aa90143add9c44 d27a6333	0	0	0	0	3	0	0	0	0	0	0	0
dbe2ebf589679f34a4b0c39ef f64802a	0	0	0	0	0	0	0	0	0	2	0	0

a5ddda38f744db0b0f80337dc6007c09	0	0	0	0	0	0	0	5	0	0	0	0
3d933bdd6a4bde67da2cd3e01aac150c	53	0	0	0	0	0	0	0	0	0	0	0
ff7e69ed183f760c68a0b7476983f5c5	0	0	0	0	0	0	0	0	0	0	0	2
566e17f3b57eb0b84c54d7077c2aa98b	0	0	0	0	0	22	0	0	0	0	0	0
88923afa1c556b8a78248fcc1205dbce	0	0	0	0	0	0	0	0	0	0	0	15
6ef54eb900024df1450d00fa54dc207c	0	7	0	0	0	0	0	0	0	0	0	0
52d59c4d9f383663e39477d01f47d48b	0	0	0	0	0	2	0	0	0	0	0	0
eab1bdff7409b316545aad609a4f09b9	0	0	0	0	0	5	0	0	0	0	0	0
85b205ff3caf483847231e0297c107bd	0	0	0	0	0	0	0	0	0	0	0	2
b4b7afb6cfcc1c17ff77b58108c0880d	0	0	0	0	0	0	0	0	0	0	4	0
0b3a2a33af8db174f32da930717e6c37	0	0	0	0	0	0	0	0	0	2	0	0
122ee0590ecd357d7847858a808e8f52	0	0	0	0	0	6	0	0	0	0	0	0
09c1756af953662e6a31c8c9e6277fdf	0	0	0	0	0	0	0	0	0	14	0	0
5d006986fe6dc8f485069b18108011b3	0	0	0	0	0	0	0	0	0	0	0	3

fce2e09eaf2d5d0fa0691a3f2e331452	0	0	0	5	0	0	0	0	0	0	0	0
f0c271145ed50100b5e886091609a336	0	0	0	0	0	41	0	0	0	0	0	0
8bdf68f7745c4e90d946486361933120	31	0	0	0	0	0	0	0	0	0	0	0
b78410dd47d1f303ec927c974fa322cb	5	0	0	0	0	0	0	0	0	0	0	0
b11ccafba1bd5064680a065967adfe1e	0	0	0	0	0	0	0	0	0	6	0	0
6a016c658f51d8c94c6111845265e4bb	0	0	0	0	0	0	2	0	0	0	0	0
0ab50c34751eae11808847d4af1d1608	0	0	0	0	0	0	0	0	9	0	0	0
6e4f1f49927349234fdb4affdf3dcf91	0	0	0	0	0	0	0	0	0	0	40	0
64fc8289d9940517840173e3b7bb2fa4	4	0	0	0	0	0	0	0	0	0	0	0
389024d49a2eb2dd85c4f48b1a6793aa	0	0	0	0	0	0	0	0	0	0	0	5
2192bddacaafc5b428e18417c4f16bf8	0	0	0	0	0	0	0	0	0	3	0	0
057512704f3d30e55d16671cc95023a4	0	0	0	0	0	0	0	35	0	0	0	0
abe3c7f50ca06506959245d35d38c430	0	0	0	0	0	0	0	0	0	0	11	0
ea2124e935bfb92577abc6b32f25027c	0	47	0	0	0	0	0	0	0	0	0	0

81ce964808b6e86e0274bc04b93b3a5e	0	0	0	0	0	0	0	0	0	3	0	0
e68ff417e61314ff270c0106f3b2fefe	0	0	0	0	0	0	0	0	0	0	6	0
31e51bdc8a6e648a14dad1fea2fd863e	0	0	0	0	25	0	0	0	0	0	0	0
e99372b04834b12a2d8a03bf9171bc53	0	0	0	0	0	47	0	0	0	0	0	0
518b85fe3de0a951f8a879d17ae098c8	0	0	0	0	0	0	0	0	0	0	6	0
41ce895ae3c39389674d1a1848999505	7	0	0	0	0	0	0	0	0	0	0	0
29666c6d0669e3aba785cf2c8353d75e	0	0	0	4	0	0	0	0	0	0	0	0
ef21d1b62fd99f719a04973437f9c817	0	0	0	0	0	2	0	0	0	0	0	0
9d1578572f8890a36f8ac7241662fc5c	0	0	0	0	3	0	0	0	0	0	0	0
b946154f4531266ac736af159b5d21cc	0	0	0	0	0	0	0	11	0	0	0	0
e30ba15d7b0d937c875750bf01417f25	0	12	0	0	0	0	0	0	0	0	0	0
73eaa5a3a87f09966d507929cceff471	0	0	0	0	0	0	0	0	0	3	0	0
1d29871a90c7fd52cd01c3dfe463a10d	0	0	4	0	0	0	0	0	0	0	0	0
12ef6da4fd40f53b792d84537a8be3ed	0	0	0	0	4	0	0	0	0	0	0	0

b844af37834e12affa89ce7c95bbd0e7	0	0	0	0	0	3	0	0	0	0	0	0
8b3d9745245247e61ebcfd67c791c190	0	0	0	0	0	0	0	0	0	0	10	0
32656312784a07308452cff12f62ecec	0	0	0	0	0	2	0	0	0	0	0	0
c483e6be3e1fcf1932e4798587843f1d	0	0	0	0	0	0	0	0	0	0	7	0
2358d68e174c493623038d889c44ee1d	0	0	0	0	0	0	0	0	0	0	54	0
f045e8a88f1b2cbfc942628f34aade8e	0	0	0	0	0	0	2	0	0	0	0	0
e587820e918bdcd38ebcdc6b96daab63	0	0	0	0	0	0	0	23	0	0	0	0
f0b93386752569795ae358a3a47d6557	0	0	0	0	0	0	0	0	0	33	0	0
bc1733ef1251a371b52d11fd2b92214c	0	0	0	0	0	0	0	0	0	0	3	0
111e18179a8698261c733ad5e810781e	0	0	0	0	0	0	0	0	0	0	16	0
28639a575bd874570c06c9fdb281d751	2	0	0	0	0	0	0	0	0	0	0	0
b0fa4bb73320588589c43d7df4a8396f	0	0	0	0	0	0	0	0	4	0	0	0
926bb888d63c7c0ddbef95beaba93b53	0	0	0	0	0	0	0	0	0	0	0	19
5e33a5871b8418034e431e39b140525d	0	0	0	0	0	0	0	0	0	0	43	0

08445d61c094f548d43e1960bb4cab8f	0	0	0	0	0	0	0	0	0	0	0	4
1af09b54601cc55e83fcd55c40aae372	0	0	0	0	0	0	0	0	0	0	2	0
96c7eace90dfbade7d18b8211115e047	0	0	0	0	0	0	0	10	0	0	0	0
511bba4695a56cf83d78ce4ba370a16d	0	0	0	0	0	0	0	0	0	0	0	28
27b405375a4f0cf5b989cfcacc163c9e	0	0	0	0	3	0	0	0	0	0	0	0
e37ff0e2ced5073062c506e5de5d6746	0	0	0	0	0	13	0	0	0	0	0	0
ab57a92d6aa16daebb59074c0797d74c	0	0	0	0	0	0	0	0	2	0	0	0
15f1e292832ffae85e8155387605b175	0	0	0	0	0	0	0	0	0	0	17	0
aeba45902e4be57212d2a1cd0a5f2384	0	0	0	0	0	0	0	0	0	0	25	0
c51e3f8bbc8875580e235d73f9f6945b	0	0	0	0	0	0	0	0	0	3	0	0
100f2d1e743dcfa638b4da625ce4a330	0	0	0	0	0	0	0	0	0	0	2	0
8f0b42dc71cec917c2f6146d594e35f1	0	0	0	0	0	2	0	0	0	0	0	0
dfec70a3b71fceff987a44fe1251de58	0	2	0	0	0	0	0	0	0	0	0	0
e835c1e4344692648385d431aaffba2e	0	0	0	0	0	0	37	0	0	0	0	0

0cae494ba14ce20d37fdcf978daeb280	0	0	0	0	0	0	0	0	0	0	12	0
3e6328e628a60d3a6424870a4ee3ca27	0	0	18	0	0	0	0	0	0	0	0	0
afc7cd70fdbdeadf5ad8c4071ae1fcb6	0	0	0	0	0	0	0	0	0	0	5	0
d494b3daf771157f0e8abbf34022d9fe	0	0	0	7	0	0	0	0	0	0	0	0
29943994a7733e5180ffe505ad23c82b	0	2	0	0	0	0	0	0	0	0	0	0
992b96a0fe130b27f676485786083376	0	0	0	0	0	0	0	0	0	2	0	0
0719dbebcae5e75ed2e8926c6e0140a0	0	9	0	0	0	0	0	0	0	0	0	0
1fc1409a461c45d4874fe4baf3ad111a	0	0	3	0	0	0	0	0	0	0	0	0
95b5547f022ffe799cf70fa667a99da5	0	0	0	0	0	0	0	0	0	0	0	7
b43d29f6a8ee976a5b989dcd4416a2d2	0	0	4	0	0	0	0	0	0	0	0	0
c279aaa13ddfa8b16ed6423fda281ee7	0	0	0	0	0	0	0	0	0	0	0	12
9fbe1dd62bd337fd32248c9da10c62c5	0	0	2	0	0	0	0	0	0	0	0	0
8302c133504d1309025cd00eb22abbad	0	9	0	0	0	0	0	0	0	0	0	0
5cf0780426adf3b6a6247dc9674719b4	0	0	0	0	0	0	0	0	0	0	0	4

10a4f8ccc8b9f90708ce41aa5526585e	0	0	0	0	0	0	0	0	0	0	0	25
69961a47a5065cd7d5d58891f2da0f8c	0	0	0	0	0	0	0	0	0	0	2	0
c04a7511f143333e7ecd8500d64f3b83	0	0	0	0	0	0	0	0	0	0	0	4
8ae094fd01a1026bb66f22b90d3bb0e5	0	0	0	0	0	0	0	0	0	0	0	11
e74064addf4d0ddaa6a17c10270225b7	0	0	0	0	5	0	0	0	0	0	0	0
dacbae522af71be3e66ecdba2df6da3d	0	0	0	0	0	0	0	0	3	0	0	0
60c7f5bf3a926de088e900ccd4a9ac9a	0	19	0	0	0	0	0	0	0	0	0	0
fe1792ff7c4d87060d62f9ce7769116f	0	0	0	0	0	0	0	0	0	0	0	5
b8256c0ed9c2ea334038bae57244ac44	0	0	0	0	0	0	0	0	0	0	0	5
2289df38d132c730bc72f37a6b5a461d	0	0	0	0	0	0	0	0	0	5	0	0
a6be302f831b86f2619bfd8e6dfb409d	0	0	0	3	0	0	0	0	0	0	0	0
4da6d6a0bacc5173f10bc998abeca124	0	0	0	0	0	0	0	7	0	0	0	0
a965b6e75568c739cd0cfa8a691d2542	0	0	0	0	0	0	0	0	0	126	0	0
f7f34d778c641efe777512acc04dc5f2	0	0	0	0	3	0	0	0	0	0	0	0

73f57840d411f8e8f5e36a338c9b1746	0	114	0	0	328	0	0	0	0	0	0	0
c2fcf2b19b262c0240c5765ed5d27933	0	0	0	0	0	0	0	0	0	0	3	0
7176bf9762af37fc9a25c234636327a6	0	0	0	0	0	0	0	2	0	0	0	0
15fe250a99119a88a5410c1f54a5923b	0	0	4	0	0	0	0	0	0	0	0	0
b696faf0a8f6fba218929da445486cae	0	0	0	0	0	0	0	0	16	0	0	0
c977c55b33d36255bd5883dd506a1258	0	0	2	0	0	0	0	0	0	0	0	0
23ef812a31698e0568060d58cb5e6632	0	0	0	0	0	0	0	0	0	0	17	0
21231a53fab5eb68e617a19bad11a2d7	0	6	0	0	0	0	0	0	0	0	0	0
f76bb2fc08d594edbc232f325163803c	0	30	0	0	0	0	0	0	0	0	0	0
0842013987bb526c006c754fa7c571f8	0	0	0	0	0	2	0	0	0	0	0	0
59d23999b3bf08d2fd9fd582bf5e59bc	0	0	0	0	0	2	0	0	0	0	0	0
45363049ae7452ea693ef551aae938f0	0	0	0	0	0	0	0	0	3	0	0	0
c5ead289a89f5be94ebfe3ed02783e77	0	70	0	0	0	0	0	0	0	0	0	0
01fbea1af5d2521872465dc780cb8d8a	0	0	0	0	18	0	0	0	0	0	0	0

9b926cc49127eee2a602e8dc1a9ea87c	0	0	0	2	0	0	0	0	0	0	0	0
f7aa31b0d9b1d0f7c8d8a6ba6cdade11	0	8	0	0	0	0	0	0	0	0	0	0
4828b41d5b35e66f0a56a5864fad4769	0	0	0	0	0	0	0	0	0	0	43	0
333a58ea699550d1b3c4b389f0a26ba2	0	0	0	0	0	0	2	0	0	0	0	0
5a8d05f93c53003dcd1ded3f08a03876	0	0	0	0	0	3	0	0	0	0	0	0
25e14143f2e26609480890ca305ac9d7	0	0	0	22	0	0	0	0	0	0	0	0
88a4c23cabcc3c9f65c2904c23351a75	0	0	0	0	4	0	0	0	0	0	0	0
bb3c580fd99c7fecb11b1c47b2e2f46b	0	0	0	0	0	0	0	0	4	0	0	0
6f54332bf9732334c299d9c71c68fcb4	0	0	0	17	0	0	0	0	0	0	0	0
885dfba6aec662425bdcddc04537fb34	0	0	0	0	0	3	0	0	0	0	0	0
588b570cc0ae7252d4114dc5043875f0	0	0	0	2	0	0	0	0	0	0	0	0
e3167092c6639b11e268551438a1b8ad	0	0	0	0	0	0	0	0	6	0	0	0
b41fdd1c8b8225dd96626a9896b65ea8	0	0	0	0	0	0	0	0	0	0	3	0
4345f52886b393c8babe09ea19e2f7b2	0	21	0	0	0	0	0	0	0	0	0	0

3a41b27a0d99fcb17974da68375f507a	0	0	0	0	7	0	0	0	0	0	0	0
dbb20968c029d1a70c60d345c19a6bf8	0	0	0	0	0	0	0	0	0	10	0	0
b0f902fd07197748f47d79788cdef305	0	0	0	0	2	0	0	0	0	0	0	0
6167349e897e1a0f2d5f4ca712c9ef50	0	0	0	11	0	0	0	0	0	0	0	0
e5fb353a6ff1eebfa2c7ed27b9c48400	0	0	0	0	0	0	0	0	3	0	0	0
f758abeca32c97d316d9fb7822d1deae	0	0	57	0	0	0	0	0	0	0	0	0
36f013a3e4d54521e3fee39cc78e3aa3	32	0	0	0	0	0	0	0	0	0	0	0
2ebd4526506736809cca588a829d5ea6	0	0	0	0	0	0	0	0	6	0	0	0
513b6f11a400d647fd2ec8df0a55ec6b	0	0	0	0	0	0	0	0	0	9	0	0
dcf9ba532d68ed68d5e35d9652a82503	0	0	0	0	0	0	0	0	0	0	11	0
2e189ef1d8c802e98fae3ddcc0c3544a	0	0	5	0	0	0	0	0	0	0	0	0
eba749a96e17c31d7907b09f542253ca	0	0	0	0	0	2	0	0	0	0	0	0
2a5bdcb4c965c2e9463b73a6fc6b944e	0	0	16	0	0	0	0	0	0	0	0	0
0c184703a25e21aba4f180c10be09d1c	0	0	15	0	0	0	0	0	0	0	0	0

f07db61e220e9ee66772c539 46beaf4a	0	0	0	0	0	18	0	0	0	0	0	0
f529d3e42721a27f37922286 7b371224	0	0	0	0	0	0	0	0	2	0	0	0
d035861f6e98e4c14a05cc55 3d9b3e92	0	0	0	0	0	0	0	0	0	0	2	0
179accfd658975e7003ea0d0 cd2c16de	0	0	0	0	0	0	0	19	0	0	0	0
46183dc2bf30e73133539b03 4702f0ee	0	0	0	0	0	7	0	0	0	0	0	0
bda2e3654827b25ef9c5a1bd 7ab048c5	0	0	4	0	0	0	0	0	0	0	0	0
6a01403c8edb5576da1b31ac b0e5f063	0	0	0	0	0	0	0	0	0	2	0	0
724cb86a652d37eb8cede7e 1d1e5b8b3	0	0	0	0	0	0	0	0	0	0	5	0
7dd49a6d639b348d04a8398 9fe36a7a6	0	0	0	0	0	0	23	0	0	0	0	0
b40cc28f0ffe8107b94f61d14 6a4e227	0	0	52	0	0	79	0	0	0	0	0	133
9a6ac47d045c48b48c749699 1a9235ad	0	0	38	0	0	0	0	0	0	0	0	0
7716b1da7a6802c1e49c8115 a66a2797	0	0	0	0	28	0	0	0	0	0	0	0
4aaae00032a78d086318c48f 54e9b451	0	0	0	0	0	0	0	0	0	0	5	0
6387fbe92fe764ca4a0f9aec1 ad639c8	0	0	0	0	0	0	0	0	0	4	0	0

fb001d2e0029a1f1233a7504 8bfc37b5	0	0	0	2	0	0	0	0	0	0	0	0
e7fbe01faa406c03492f0867b 2bba9ad	0	0	0	0	0	0	5	0	0	0	0	0
f38654c6b126247066a31f49 d9bfd253	0	0	0	0	0	0	0	0	0	0	91	0
d4cc8f43d0a3bff1eaf4603b4 21e183b	0	0	0	0	0	0	0	0	0	0	4	0
e3260918711ac0b51c4f9405 a69072c2	0	0	0	0	0	0	0	0	0	0	0	16
61f124ffc837f6b6ffe20f2a02 525191	0	0	0	0	0	0	0	0	0	0	0	5
2d8e0e8426ccf195cbd42671 dcb63b5d	0	0	0	0	0	0	4	0	0	0	0	0
abd4351e75d74a988774ccb 9cbdfde29	0	0	0	5	0	0	0	0	0	0	0	0
6f87c7a9533ca79035a37307 0148ac5d	0	0	0	0	0	5	0	0	0	0	0	0
f2b95102297b74ccecc8da38 c1b4ac92	0	0	6	0	0	0	0	0	0	0	0	0
979728340a87a6b4cf963aa5 2eafa0a3	0	0	0	0	0	0	0	0	0	0	2	0
69b5fd911987faded62da3ce 2af39ba9	0	0	0	0	0	0	17	0	0	0	0	0
c0172dcdd7f28128a28b9480 90caf237	0	11	0	0	0	0	0	0	0	0	0	0
839d3a534cdb323712614c5 6363e2751	0	0	0	0	0	0	0	8	0	0	0	0

95300f630bcfd99e81006fb301c1fe1d	0	0	9	0	0	0	0	0	0	0	0	0
b1437d9e748858cc3d912c3ae3d1dfd9	0	0	0	0	0	0	0	0	0	0	0	5
6f1565cf16e6f711f06b8c3adfe3ba10	0	0	0	0	0	0	0	2	0	0	0	0
8f1cf51f1fdea32f58f607107089e00d	0	0	0	0	0	0	10	0	0	0	0	0
8681c0304ae7589b7cd25b5a7d6a2e85	0	0	0	0	0	0	0	0	0	0	0	13
94b38829b07bd4f0f7543b836344e57c	0	0	0	0	0	0	2	0	0	0	0	0
39eee8be7e6a73abf348e5acb97938a8	0	0	0	0	0	0	0	0	0	0	4	0
4c1089c8235cd68bc59ea92562854faf	0	0	0	0	0	0	0	5	0	0	0	0
4b13c1b9dea14181b6c640b1d38c4b79	0	0	0	0	0	0	0	6	0	0	0	0
5f5a2a4f894c4a8fd8624ec1a4ec0e99	3	0	0	0	0	0	0	0	0	0	0	0
3233fdbda6592a8ad1be0be85b37c65f	0	0	17	0	0	0	0	0	0	0	0	0
ef88881cafaf91a3eb6678aa030bcd41	0	0	0	0	0	0	0	0	0	0	0	11
52fe6693004a908079ff4eaa92f193a2	0	0	0	0	3	0	0	0	0	0	0	0
c67ad7ec8eeafb6ded15286f15d64d3c	0	0	0	0	5	0	0	0	0	0	0	0

bbf82120898506dc05eeae6ca6172090	0	0	0	0	0	0	0	0	0	24	0	0
9dc3300408e5d2f6b4009257cb1f5d28	0	0	0	0	0	0	0	0	0	21	0	0
673ec12d1828700186e0790012a46580	0	0	0	0	0	0	0	9	0	0	0	0
78e580cd62b88d801ea6d70992a23993	0	0	0	0	0	0	0	0	0	0	0	19
d735d0179f251ad857f2d41727587c5a	0	0	0	0	0	0	6	0	0	0	0	0
dd46ee965545d0d34e01c1bc0dea0e09	0	0	0	0	0	0	0	0	26	0	0	0
b0219e77319b9217b602e013585f294b	5	0	0	0	0	0	0	0	0	0	0	0
d78a5a0b80d99886fd4ec1d993b75677	0	0	0	0	0	0	0	0	0	0	3	0
63d8e466310cb8d558d00e65893139dd	0	0	0	0	4	0	0	0	0	0	0	0
281bb83d3ba223acf0ecc1d0d575c703	2	0	0	0	0	0	0	0	0	0	0	0
c8aaa1d6d2a46423defdeff8d8813f08	0	0	0	0	0	0	2	0	0	0	0	0
7e8f7f8345ceab76dcf10a6cdced2de9	0	0	0	0	0	0	0	22	0	0	0	0
dc4dd7c3a2ca7cdafe1a475236456cc6	0	0	0	0	0	0	0	0	0	0	0	2
b91ecbee45e7f23e7f952738d4be45e3	0	0	0	0	0	0	0	0	0	0	25	0

e809630ddc4d5510a2b8d71d9ec85485	0	0	0	0	0	0	0	4	0	0	0	0
7422ae96635fa3cc82a578eea20e1f21	2	0	0	0	0	0	0	0	0	0	0	0
a35598ba6943c49d7b09b3d084c40db8	0	0	0	0	0	0	0	4	0	0	0	0
c215ff0680c4976fe4dbc05ad061441a	0	0	0	0	0	0	0	0	2	0	0	0
abe102f29fee2556359db06e386f0507	0	0	0	0	0	0	0	4	0	0	0	0
617d8b1f5c3388a0e834586d773e5ebf	0	0	0	0	0	0	0	0	0	0	8	0
d7723c517791a95b36d4adc658e86ef9	0	0	0	0	0	0	0	0	2	0	0	0
8249adc7d1b81e55c60000a57ef48806	0	0	0	0	0	0	0	0	0	0	4	0
592bda907989736f30a3059816f81de5	0	0	0	0	0	8	0	0	0	0	0	0
0de9624552ed3023ee6fe9912645d9f9	0	0	0	0	0	0	0	0	0	0	2	0
0d36d4c37a24a84760103bc341ab0e87	0	0	0	0	0	0	0	0	0	0	0	21
0338c6183669f07db99618f627ed51a4	0	0	0	0	0	0	0	0	0	44	0	0
24e8a0fa27aeb1a77da3d0caa8d44717	0	0	0	0	17	0	0	0	0	0	0	0
33f400362835fe4f83d60d02e10492b5	0	0	0	0	0	4	0	0	0	0	0	0

0037d64d284f6e2c6aa24245 4704c9cc	0	0	0	0	0	0	70	0	0	0	0	0
01f5a353b05dabe555eba4d9 78e00d17	0	0	0	0	0	0	0	0	0	0	33	0
70ac86dfb1e503bd0dd44b99 1f221c75	0	0	37	0	0	0	0	0	0	0	0	0
aa35ca1b942c440a3d5bf755 669d5ee6	0	0	0	0	88	0	0	0	0	0	0	0
6df067e1b6231a2054fed806 7d8b5654	0	0	0	0	0	0	0	0	0	0	7	0
d993875235bfc1b81da3bd13 9e6c5664	0	123	0	0	0	0	0	0	0	0	0	0
8689af87232818f53ade4f85 eb999851	0	0	0	0	0	0	0	0	0	0	0	5
32c5e7465351e4e2434b3fe5 4508458f	0	0	0	0	0	0	0	0	0	0	3	0
18ee1653d6c9b60f4e2ca382 ab129cfb	0	0	0	0	0	0	0	0	10	0	0	0
c1c8cbedf0a0973c0f951962c 677d40c	0	0	0	0	0	0	0	0	0	0	3	0
b893a9c3ae2b6745353d898 a3bf210a1	0	0	0	0	0	0	0	0	0	0	0	4
814f03a23bd5e16b81739ed bf5ef62ba	0	0	0	2	0	0	0	0	0	0	0	0
0f18f067139365e752faca20f 03a6b7f	0	0	0	0	0	0	0	0	2	0	0	0
23cc205246f998bc46f724f1c d06c993	0	0	0	0	0	2	0	0	0	0	0	0

bdba8cc1ff02591c18e62bb02efad37f	119	0	793	0	199	0	303	497	0	380	0	0
f77e7a196185b567a17656328121d850	0	9	0	0	0	0	0	0	0	0	0	0
41764b441e41029a5760d023390e1341	0	0	13	0	0	0	0	0	0	0	0	0
809f867d5bbd2af33e5c1d58a606fddf	0	0	0	22	0	0	0	0	0	20	0	0
3c4d90f7e57f59cc06c52ebdc58b4085	0	0	0	0	0	0	0	0	0	0	0	61
6ab006cc72f29fe0a9e8830c5797940a	0	0	0	0	0	0	0	0	0	9	0	0
a031533f84d3dd9ec9a5ac3302e7d28d	0	0	0	2	0	0	0	0	0	0	0	0
e09036e701fc6bd5fa1da263cd8a2546	0	0	0	0	0	0	0	0	0	0	0	4
33c468a15bb1e5b61438c008e6d2e8d2	0	0	6	0	0	0	0	0	0	0	0	0
cd8ffeeeaf4d624376d44451b8710da3	0	0	0	0	0	0	0	0	58	0	0	0
5a4d8fa1a1ffd7f55ccfc8406b57fc9d	0	0	0	0	0	4	0	0	0	0	0	0
169c44ca20b04b7993dfc3496ac1e17b	0	0	0	0	0	0	0	0	0	0	91	0
1baeaa2027aa6534c4291ef2b9286b9c	0	0	0	0	0	0	0	0	0	0	0	2
fcbacfa33d833662eb539abd2a2568c2	0	0	0	0	0	6	0	0	0	0	0	0

756b411ba3a0daa83d62af8b90fa9791	0	0	0	0	0	9	0	0	0	0	0	0
ba739797b9d93e97f82b83c89942e8e1	0	0	0	0	0	0	0	0	61	0	0	0
e3a377ec5f4ae2d2dbca70805e304f28	0	0	0	9	0	0	0	0	0	0	0	0
8a9daa2911628adcf03c2bff2cb7c0ce	0	0	0	0	0	0	0	0	0	0	0	3
6f17dc82b40a194b94c45a6e81676e8a	0	0	0	0	0	0	0	0	0	0	8	0
27d513202de3d21c17282e93ff650be6	0	8	0	0	0	0	0	0	0	0	0	0
ab9d3fc53f00dcb42db67b6ae380f48b	0	0	0	0	0	0	0	11	0	21	0	0
c46bd5b5443873a788775b4a4f584f19	0	0	0	0	0	0	0	16	0	0	0	0
0959c4997485c59aa734619e4ae02379	0	0	0	0	0	0	0	0	4	0	0	0
9a3c4c324184388794a38a8922afd9e4	0	0	0	0	0	0	0	0	0	0	0	13
3fc0ab2863d618f6c51ba64faf8730fa	0	0	0	0	0	0	0	0	0	0	0	4
e884bb8c98ae2f0ba997efde85cba27f	0	0	0	0	0	0	0	0	0	3	0	0
b293eb17cc3957278626c9be2ba02520	0	0	0	0	0	0	0	0	6	0	0	0
2ab354ffa11290bc9243d6ea79ca8041	0	0	0	0	2	0	0	0	0	0	0	0

5a1a7a6ac86ee9bc97237931a183e1d1	0	0	0	0	0	26	0	0	0	0	0	0
377d8a1a53b975dd6a8a92968b0f5d5e	0	0	0	0	0	0	0	0	0	2	0	0
2c8dde43521f8a0cbb628e69b13e326f	0	0	0	0	0	0	0	0	0	0	0	3
1206b6eebad5941c0a54c82459464b21	0	2	0	0	0	0	0	0	0	0	0	0
def31d2a95f9e9457aba550bebfd6685	0	0	0	0	0	0	0	0	0	0	0	13
c3376e77146b1d8776f46793c6dc254a	0	0	0	0	0	2	0	0	0	0	0	0
624fc46490d61c44e5be413c1646125e	0	0	0	0	0	2	0	0	0	0	0	0
63c895540744956fd0811f20b9c1ac24	0	0	0	0	0	0	0	0	0	0	4	0
c85172a142089490ae2b1bf936c99421	0	0	0	0	0	0	0	0	0	0	0	2
b2c8c72447c402bb812f5c3dc0ceca6a	0	0	0	0	0	0	0	0	2	0	0	0
bd98aacca5f0f198d5e636bdee2305cf	0	0	0	12	0	0	0	0	0	0	0	0
99952a9445ab5d6c6fbb7cabc36582e8	0	0	0	0	0	14	0	0	0	0	0	0
173d08584017b3c9e76a131f5845d0ec	0	0	0	0	0	0	0	7	0	0	0	0
8847cc40daaa8e4798edf092854e6ce5	0	0	0	0	0	0	0	0	35	0	0	0

534bd0828f4630ddaff55af82 76d156c	0	0	0	0	0	0	0	0	0	0	3	0
f2224e406a79e92009742553 3df6df29	0	0	0	0	0	6	0	0	0	0	0	0
1452adcd8a6fed6af5af2c0ef e27a623	0	0	4	0	0	0	0	0	0	0	0	0
87960875c60ae2b60641b85 cea00f3dc	0	0	0	0	0	0	0	0	0	2	0	0
ec7d5ab5fe833a928c5ce0ef9 b5b39de	0	0	0	0	0	0	0	0	0	0	0	2
03742c2958d5ce51170f9a71 64858910	0	0	0	3	0	0	0	0	0	0	0	0
37338940c11d6244d9dd5ad 8d137a73d	0	0	0	0	0	0	0	0	0	0	0	2
d17259d1f569f7cf954d8af71 7da435f	0	0	110	0	0	0	119	101	0	0	0	0
332f31c3baa284aa53dc5cf15 8b52f75	0	0	0	0	0	0	0	0	0	0	0	19
ee2a2d9ad7e7b379d084416 449ee68f4	0	0	0	0	0	0	0	2	0	0	0	0
17640c4ff494e826bb984073 af39767f	0	0	0	3	0	0	0	0	0	0	0	0
92f8fdacbd3658188907f5788 fe47b80	3	0	0	0	0	0	0	0	0	0	0	0
b55a341c1712d3336f00fe29 8cf37e8e	0	0	0	0	0	0	0	0	16	0	0	0
4c98ced8205c9bf5720090a7 38a3dcf4	0	0	0	0	3	0	0	0	0	0	0	0

b8a234cd342c3332f71ab483488c136d	0	0	0	0	0	0	0	0	0	0	3	0
3b8bf948adfa0cbebf614c9ed6b04593	0	0	0	0	0	0	0	0	0	0	39	0
10e58a0cc0bc5bc49f0a318c9b19b541	0	0	0	0	0	3	0	0	0	0	0	0
e19070d88b087b03451636e823beb091	0	0	0	0	0	0	0	2	0	0	0	0
38b2795db2fa499671b57eebce2e0711	0	0	0	0	0	0	0	0	0	0	3	0
64aa73006b16c9421f6d5440ff9e1c3c	0	0	0	0	0	0	0	3	0	0	0	0
5b6c34b7efe477dae33c81afe612c3f4	0	0	0	0	0	0	0	0	0	0	144	0
a2b26749861c9a854f434ae9af150545	0	0	0	0	0	0	0	0	0	0	0	4
403ca756a7764f9d090cad1a2e5aa948	0	0	0	0	0	0	0	0	0	0	4	0
57056c83aa6149cff082fe058d4d4c19	0	0	0	0	0	0	0	0	0	3	0	0
975659373f160cba0d388c0779295536	0	0	0	3	0	0	0	0	0	0	0	0
113b992ee754e2bf6387869132d263c6	0	0	0	0	0	0	115	0	0	0	0	0
834824e4076bac72b450b7bcc12d0a4f	0	0	0	0	0	0	0	0	3	0	0	0
bea57b327b0b39e08af953e293c89a79	0	0	0	0	0	3	0	0	0	0	0	0

93760e2c285c08fd97fe6449a62b8e2f	3	0	0	0	0	0	0	0	0	0	0	0
5fe18568198ddd668c13fa6ad1dfb5b8	0	0	0	0	0	2	0	0	0	0	0	0
af60985d53538ceab22f4681845ac501	0	0	0	0	0	0	0	0	3	0	0	0
c59f07817669850ad106ab46dd52077c	0	0	0	0	0	0	0	0	0	0	37	0
4627f7cb8dd4c201073826dd12a39bee	0	0	0	0	0	0	0	0	0	0	5	0
56fbd8dab8bc07766ca8942596bcac9e	0	0	0	0	0	0	0	4	0	0	0	0
50b9d383c8c633a8b04f801ca0ef2769	0	0	0	0	0	0	0	0	0	0	0	2
9440b68f9f75412424ddf48491b6759d	0	0	0	0	0	5	0	0	0	0	0	0
d43b11738653208f5b55616759cd127f	0	0	0	0	0	0	0	0	0	0	2	0
7dafbc28c6d09bb4366cc4a519767cf1	0	0	0	0	0	0	2	0	0	0	0	0
9b5b7d5521f53a6e71bb3207ba37d586	0	0	2	0	0	0	0	0	0	0	0	0
4e39837de012199dc7035bff8a53247b	0	0	0	0	6	0	0	0	0	0	0	0
c3e3bcba1dafc57d3118bf5344bc50da	0	0	0	13	0	0	0	0	0	0	0	0
3acff2c86eb8da948d9232710e070aa3	0	0	0	0	0	0	0	0	2	0	0	0

62bb74a1b4bb7eb555f250e e5b00a968	0	0	0	0	0	0	0	0	0	0	2	0
e8bda1a47b269540b588df32 b598da96	0	0	0	0	0	0	3	0	0	0	0	0
7c04fac4a419d8b4cae58985 ad9bb066	0	0	3	0	0	0	0	0	0	0	0	0
90177e5173d03d677cfc7c84 7adefbe8	0	0	0	0	2	0	0	0	0	0	0	0
3809cff75825ba6998927ca9 844787c7	0	0	0	0	0	0	0	0	0	0	0	3
69d00abe4fea84f040a6b38b 90bda331	0	2	0	0	0	0	0	0	0	0	0	0
1b4c8bbdf095900ffe1984a72 696d1de	0	0	0	0	0	0	0	0	0	0	22	0
8c1e49ecf3893860230c8d3e 6d717c46	0	0	0	0	0	7	0	0	0	0	0	0
4efb2dd66d75d793e1a047a2 4e5d1ba7	0	0	0	0	0	0	0	0	64	0	0	0
0d78e5fb18e95d2ac10f02bc 571c1989	0	0	0	0	0	3	0	0	0	0	0	0
7b22e01bfe5b3a4141dfd976 37a0262e	0	0	0	0	0	3	0	0	0	0	0	0
61de2a9f953298536718e899 3d1d778e	0	0	3	0	0	0	0	0	0	0	0	0
bd8debbeaa9709e5afe6b514 686549e9	0	0	0	0	0	0	0	0	0	22	0	0
bc48e438318939948fd843e7 5ee941f4	2	0	0	0	0	0	0	0	0	0	0	0

70ebb5774dc50da945cad65 4761d7862	0	0	0	46	0	0	0	0	0	0	0	0
7ddbddadc082402f0e1215d7 5716bd4c	0	0	0	0	0	0	3	0	0	0	0	0
9f9f686e472359f5d18aa038 efad980b	0	0	0	0	0	3	0	0	0	0	0	0
76c4e759d5a9d1f81eeb9ac3 eb0c740e	0	0	0	0	0	0	0	3	0	0	0	0
0f69a6077797537d4472dee6 91c8c962	0	0	0	0	0	2	0	0	0	0	0	0
502b041b62089015550d6c9 a2e5acda2	53	0	0	0	0	0	0	0	0	0	0	0
3b5972b872ef2c9a93a1d2d0 3c50bae5	0	0	0	5	0	0	0	0	0	0	0	0
30ae5f13c2b12fe682c55216 94777f72	0	4	0	0	0	0	0	0	0	0	0	0
7e5bdb79776fb0572df1faa1 a7f582e6	0	0	0	0	0	0	0	0	0	0	0	2
76bc20e2615cac8af6b62b5b 4d80262d	0	0	15	0	0	0	0	0	0	0	0	0
07263b465d1ba08ad965af96 28b24b58	0	0	0	0	0	0	0	0	0	4	0	0
e860c7940b27770e69975ed 48e0629ce	0	0	0	0	0	0	0	0	0	2	0	0
64cd2a16e0f08286c008294c aa42324f	0	0	0	0	2	0	0	0	0	0	0	0
9e27306d43606d6784b80b6 f2c4af96d	0	0	0	0	0	0	0	0	17	0	0	0

ae71ca1fdd0d3b85af74095d909b3c4e	0	0	9	0	0	0	0	0	0	0	0	0
93aa254a312ef327d28e23922a8e8dcb	0	0	0	5	0	0	0	0	0	0	0	0
c8418e98480e917acf42d253580f3580	0	0	0	0	0	5	0	0	0	0	0	0
ac0d1c77c219bbb91972f776585a0a24	0	0	0	0	0	0	0	0	0	4	0	0
9d5db935562ae53d182cddb038c18fca	0	0	0	0	0	0	0	0	2	0	0	0
8dd40ee9e220772792877e0b087707b2	10	0	0	0	0	0	0	0	0	0	0	0
f0759483b518c320e123b712f5dc4221	0	0	0	0	4	0	0	0	0	0	0	0
5e7cbf54d19b032dd63fe3bdb5df0e9a	0	0	0	0	0	0	0	0	3	0	0	0
650c5236c21be4bbae5c50a3402a00a1	0	0	0	0	14	0	0	0	0	0	0	0
4b89c7459ce816e27d0b8184569ae132	0	16	0	0	0	0	0	0	0	0	0	0
085af409c4a7716a37412f19ef332dda	4	0	0	0	0	0	0	0	0	0	0	0
246afa75f7f26eb40899f86d77845946	0	0	0	0	0	0	0	0	14	0	0	0
aa921ebcced871cb80495a44e960b044	0	24	0	0	0	0	0	0	0	0	0	0
e02d0388a6b0836c32cb2b823023bcae	0	0	0	0	0	0	0	0	0	0	8	0

f2cf0099e94c11967c4ed5136c707254	0	0	0	0	0	0	0	0	0	0	18	0
344e8a53cdc777a85feb1698cba1ec10	0	0	0	0	0	0	0	0	0	0	0	18
ebe82d22d0144b1ec143612ea192ecdf	0	0	0	0	0	5	0	0	0	0	0	0
bae25d138fd80527e7f6db8015c962b0	0	0	5	0	0	0	0	0	0	0	0	0
c9f00afafc349b6a7bedca2e24c4bbdc	0	0	0	0	0	0	16	0	0	0	0	0
d50deb5208e49739011be49c085d2c98	0	0	0	0	0	0	0	0	0	0	7	0
d0ab69e2a4ee50a9332414b5d7a903b5	0	0	0	0	0	0	0	0	0	0	0	2
ab9f996c521a600435ca81879db1220c	0	0	0	0	0	0	0	0	12	0	0	0
eea35729b5a3a8224374ad2511dd28b7	0	0	0	0	0	0	0	0	0	0	4	0
ce4ed4e157fb4f1711900595d9aa07be	0	0	0	0	0	0	0	0	45	0	0	0
5c2d0f7a6323b00cec8e399c2571a6ec	0	0	2	0	0	0	0	0	0	0	0	0
eee674c767b47b82f5f6c5a2c60fd5e4	0	0	0	0	0	0	0	0	0	0	51	0
c7f29cc0edf2285dbc5ff23c4ababb94	0	0	0	13	0	0	0	0	0	0	0	0
7710806a2fee06e607eed74196bfbc03	0	0	0	95	0	0	0	0	0	0	0	0

62c9970a151748e3ec61025b b04b9e3b	0	0	0	0	0	0	0	0	0	6	0	0
5c776202832d41802dd5415 956bee5c9	0	0	8	0	0	0	0	0	0	0	0	0
244caf1b9ba062a82a9aa7ee 506077cf	0	0	0	0	0	0	0	7	0	0	0	0
7a4585ae041a45122e4f4d73 691f613f	0	0	0	0	0	0	0	4	0	0	0	0
76f84898c50025d3a0ee04c5 35932efb	0	0	0	0	0	0	0	0	0	0	20	0
32aee56e29e5c160817bc84a fc29e3e1	0	0	0	0	0	2	0	0	0	0	0	0
7ba2dd2f35e0c9fd5c462efc6 6e94056	0	62	0	0	0	0	0	0	0	0	0	0
4ff94c409af573a3cb8afa491 d3fcfee	0	0	0	110	0	0	0	0	0	0	0	0
dff34917c17b7fffabf37e0ebd a4f751	0	0	0	0	0	0	0	0	0	0	0	48
3737d142ae4e72c8cb9bb3c4 42b36abc	0	0	0	19	0	0	0	0	0	0	0	0
05e8058189b6281b5efc8703 f71fa787	0	0	0	0	0	0	0	48	0	0	0	0
b7196f514f8dfeff023cc0a88f 6c4530	0	0	0	0	0	0	8	0	0	0	0	0
d7559726b638a2adc62e18d 92cc62090	0	0	0	0	0	0	0	0	0	70	0	0
a6d7ec8c2c10b43d02324d0a 28e39920	0	0	0	0	4	0	0	0	0	0	0	0

bec73de74b7e61fe9683e43b7a8499a3	0	0	0	0	0	0	0	0	0	5	0	0
c966eab1fc989bfd9b2e23d9b8bf1244	0	0	0	0	0	0	0	0	0	8	0	0
7cd2b5ce775e9a49f4bb303e89feaf9b	0	0	0	0	0	0	0	0	0	0	0	2
56c8a30adda009a6ae52b6ec4ca67724	0	0	0	0	0	0	0	0	0	0	12	0
e0c20458b2c634ab730ddc66ddbd71e9	0	0	0	3	0	0	0	0	0	0	0	0
398c0b064a1d280b6ce739ca671d4d86	0	0	2	0	0	0	0	0	0	0	0	0
01ee69c0e692cb0f48b015b169494a59	0	0	0	0	0	0	0	0	0	8	0	0
74a17fb2c0b70d0c119d414383f19504	0	0	0	0	0	0	0	0	0	0	2	0
4a406c5113e7e9ff26d091f17b334191	0	0	0	0	49	0	0	0	0	0	0	0
99219cfa5261ed310221d4b69250ea1d	0	0	0	3	0	0	0	0	0	0	0	0
7350a8e8df5d6125ec55ced2e559450b	0	0	0	0	0	0	0	3	0	0	0	0
f43bf930f829aa652fef97224393731a	0	0	2	0	0	0	0	0	0	0	0	0
abd9eb641581e4ab387f51857f79cab9	0	0	0	2	0	0	0	0	0	0	0	0
2b1b6332893f9f853bf572684c0dc423	0	0	0	0	0	0	0	0	2	0	0	0

b32fdab44a3fb3e438907c9df2a6115d	0	0	0	0	0	0	0	3	0	0	0	0
2d2102070402caeee90991a2211da8e9	0	0	0	0	0	0	0	0	0	0	0	3
dc7d98d8b89988ed0737100655effdb1	0	0	0	0	0	0	0	0	0	5	0	0
bd5c7b61d30a35b69cbbf64a960b3277	0	0	16	0	0	0	0	0	0	0	0	0
4664d7b8d270771b57822c56e9e7c319	0	0	0	0	0	0	0	65	0	0	0	0
7bd01bf263651e54c1429eba60db831d	0	0	0	0	0	0	0	28	0	0	0	0
3428329bc1bddf0a567271eee0f110e8	0	0	0	0	0	0	0	6	0	0	0	0
ab57b3586c24709c894a13f7ac41d376	0	0	0	0	0	16	0	0	0	0	0	0
351a97b176dc404c9e50b1e5a7ee7bd8	0	0	0	0	0	38	0	0	0	0	0	0
70d2a7d827e84e21a254139dac5d456a	0	0	0	0	0	0	4	0	0	0	0	0
6e6177ca9f5f43c4b98588f533e2c602	0	0	0	0	0	0	0	0	0	0	0	5
0d58d3f2c14cb50ae1440a05e87689a7	0	0	0	0	0	0	0	5	0	0	0	0
52840a6ed539ba1846415b09ee81f893	0	0	0	0	0	0	0	0	0	5	0	0
eadd50a972282a564b41c919d11091e5	0	0	0	3	0	0	0	0	0	0	0	0

8263bcef2e2f0783f15bc9c949e1e37b	0	0	0	0	0	5	0	0	0	0	0	0
b99b81b08c80320c930607f0803a4d17	0	0	0	0	0	0	23	0	0	0	0	0
143144c2c863383a8d47c8b04b68e648	0	98	0	0	0	0	0	0	0	0	0	0
70f8c8bb64cfbc588b56b95cff2026dd	0	0	0	0	0	0	0	0	23	0	0	0
4ef4011eada4ca33283f0b28cb1cd768	0	0	0	0	0	0	0	0	0	0	0	10
80075da3e284bca8e57f7050b1ea22c8	0	0	0	0	4	0	0	0	0	0	0	0
55b956851f10d8bc7f968364c585102d	0	0	0	0	0	0	0	0	0	0	9	0
b7a35820980e0c15fafc2b46f5f7c2b7	0	0	0	0	0	0	0	0	0	0	0	3
8ac1c8bacc8a67acaa910ad01c1a934c	0	0	0	0	0	0	0	0	2	0	0	0
a6f931c9f42d4cbbcd8fbeb360e0c355	0	0	0	0	0	0	0	0	0	3	0	0
eb0b474a2c55c9e3c670f17f4909ffb8	0	0	0	0	0	5	0	0	0	0	0	0
078545eb4ed08f9a700c7448d578ee95	0	0	0	0	4	0	0	0	0	0	0	0
dc808e57682c811daeacfe65fe0afb5b	0	0	0	0	0	0	0	0	0	0	4	0
1c227cd8cf1b05627840eec72c3602ef	0	0	0	0	2	0	0	0	0	0	0	0

b1861272d0b5e9212ed6564ce63bf39c	0	0	0	0	0	0	0	17	0	0	0	0
7bcab214ea4551f48a0a34023958cddc	0	0	26	0	0	0	0	0	0	0	0	0
2f5c8093f1c81f8d9d80bec566ff5937	0	0	0	0	0	0	0	0	0	0	4	0
f5705f0bfbbd34ccf3a555eaaedca994	0	0	0	0	0	0	0	0	0	46	0	0
0ce9f460c507c08eec8dd762066b36e1	0	0	0	0	0	0	0	0	5	0	0	0
c6e70493a6cecc5b9fdde02a496f5590	0	0	0	0	2	0	0	0	0	0	0	0
8949d5c3c96cd659cc21b8b592f81b9c	0	0	0	0	0	6	0	0	0	0	0	0
4972fe17619e16b823053c91b021e40f	0	0	0	0	0	0	0	0	0	0	0	9
0b3c08d43d6aa924051aa8326b32f691	0	0	0	0	0	0	0	0	0	2	0	0
184f27525b0091159febde98c0ae9430	0	0	106	0	0	0	222	0	201	0	242	0
3033d21a3183f6d9a0484ac7727379f2	0	0	0	0	0	0	0	0	0	0	11	0
e9ff79758f115d0848e2b0c772bf9fef	0	0	0	0	0	0	0	0	0	0	2	0
592264f0a2ba0180c0dbc342b961e723	0	0	0	0	0	0	0	0	0	0	11	0
7563c552da3f1a4b17d06cc4bc93630c	0	0	0	0	0	0	0	2	0	0	0	0

436c19b1a87003f0885e60fd9399fe60	0	0	0	0	0	0	0	2	0	0	0	0
977ac02ea09502b51954f54be80f1bd2	0	0	0	0	0	3	0	0	0	0	0	0
b8c6425abaad0e789f3b13e0b3c1a094	0	0	0	0	0	0	0	0	0	0	3	0
94357e22ded3135d2c92cacd5416ee9e	0	0	0	0	0	0	0	0	0	8	0	0
bf5e2186bfd76c06846e8bade04efa50	0	0	0	0	0	0	0	0	0	0	16	0
8dee1ed77ee0608a641a38793e202cfd	0	0	0	0	0	0	3	0	0	0	0	0
8d1dbcd85f9b24f2d09062ca72316a18	0	0	0	0	0	0	0	0	2	0	0	0
3cec78dafef42fc80ef11aa82419e3ad	0	0	0	0	0	0	0	0	0	0	6	0
34b9a9b6da0916aa87a2927785459b94	0	0	0	0	0	0	0	0	0	0	2	0
abb11188a45ccf746d41bac776da6a0b	0	0	0	0	0	0	0	0	0	0	2	0
8973395ae6e56fa3f5cad9bc76329dc3	3	0	0	0	0	0	0	0	0	0	0	0
d9443cdb04018b082c907fd310d24292	0	0	0	0	0	3	0	0	0	0	0	0
dcae099f46d2d3d1342eee76730062a7	0	0	0	0	0	0	0	8	0	0	0	0
e8100ce4ddc36b488cf2837ab8242c91	0	0	0	0	0	0	0	4	0	0	0	0

a6c1a3c92dcbf25f87956bda52574389	0	0	0	10	0	0	0	0	0	0	0	0
5a3872ca8b8c84c8f155265e6023e350	0	0	0	0	7	0	0	0	0	0	0	0
17b37f40cf1b466fb1d895ab0d2906c5	0	0	0	0	0	0	0	4	0	0	0	0
339c20e66e1fb01b80c256897a1bcb13	0	0	0	0	0	0	0	0	0	0	0	6
ecbc1690a761f096b180c7203a7dbbc7	0	0	0	0	0	3	0	0	0	0	0	0
600a8e69678182c4af05f7b4b1f01bef	0	0	0	408	0	0	0	0	0	44	0	0
4cc617ab10781d418426e5a70eff3e71	0	0	23	0	0	0	0	0	0	0	0	0
2c006e507481735a14bcef4017e00412	0	0	0	0	0	0	0	0	0	4	0	0
c9d73fad5d209103640a97d6e5914cf7	0	0	0	0	0	0	0	0	0	0	32	0
84a09ca661c705d6d0c847d9d4b015d5	0	0	0	0	150	0	63	0	0	0	0	165
f6d163f8206a8276bb526f99accd9a58	0	0	0	0	0	10	0	0	0	0	0	0
5a2726e9f68e4aff3bd3ba73775cbf80	0	0	0	0	0	0	0	0	4	0	0	0
cc472963e671a5fe23e246aeacf66889	0	0	0	4	0	0	0	0	0	0	0	0
13a14e6c037fdf5a75d2bbeb674b7697	0	0	2	0	0	0	0	0	0	0	0	0

13866b5e8927dd588fe3fb08 8353d409	2	0	0	0	0	0	0	0	0	0	0	0
2b63eeed32e7d42a63d0443 1597a51cd	0	0	0	0	0	0	0	0	0	0	2	0
2fef7a85ededdbc4cf3e572f9 b4d6dc4	0	0	0	0	0	0	0	0	9	0	0	0
536a8f13e8933eb42b88b781 5c2fcd3b	0	0	0	0	0	0	0	4	0	0	0	0
c1ebba4f62ce9b52e3683b69 a3144a80	0	0	0	0	0	0	0	0	0	0	0	137
b1bdedbf3f7c8e982b7c07d4 efaec599	0	0	0	0	0	0	0	0	0	0	0	2
426ebac515fcdf6aca1441301 6c78618	0	0	0	0	0	3	0	0	0	0	0	0
10f2570c2020e02c723b8a9d df85eab3	0	0	0	0	0	0	0	0	0	0	9	0
c5c532f1977b40373b26ebd6 6dfe8155	0	0	0	0	0	0	0	0	0	0	0	46
9dd62eef4693452c5b6388b5 aefb7465	0	0	0	0	0	0	0	0	16	0	0	0
95f85912eb148930fbb277fa d16ff9f2	0	0	0	0	0	0	0	0	0	0	0	5
09961bdc3cf3e3343d98aa2e 722b0000	0	0	0	0	5	0	0	0	0	0	0	0
b9b2f48d0e0d8aa3eade87a0 3ae58a85	0	0	0	4	0	0	0	0	0	0	0	0
39103b0280239897e0d74ed 87aeb93e0	0	0	4	0	0	0	0	0	0	0	0	0

eb2f322c0f9b3ee5b671c97ad9adc9f9	0	0	0	0	0	0	0	0	0	4	0	0
106c593d343ad6bf095cf198bdca662a	0	0	0	0	0	0	0	0	0	3	0	0
e5b421efd965723382eb856f532250ed	0	0	0	0	0	0	6	0	0	0	0	0
4e43fc0ce8510a719cd304c291b77fc8	0	0	0	0	0	0	0	5	0	0	0	0
29bf16d83823dfc1a72007ecba0307e0	0	0	0	0	0	0	0	52	0	79	0	0
fd4fc5eeb52694b51060c9fe3098876e	0	0	0	0	0	0	0	0	0	0	9	0
b00aece3a0c253936983e2faa1cc20dc	0	0	0	0	2	0	0	0	0	0	0	0
67a950b0c51f4350256add871b309640	0	0	0	0	0	0	0	0	5	0	0	0
a46a719e46e70bdd92f282171789b69f	0	0	0	71	0	0	0	0	0	0	0	0
e5aac9437802a916bdcd3ef1eaad2356	0	0	0	0	0	0	0	0	0	0	0	18
0082b68a03561a9e6e831ff48e4a91ee	0	0	0	0	0	0	3	0	0	0	0	0
9e6ce8adf5e95865026dc803d073b79b	0	4	0	0	0	0	0	0	0	0	0	0
b28b8231fb60206f1c5338eda29ecbe2	0	0	0	29	0	0	0	0	0	0	0	0
39159552fb314658ec96ada27547abb6	0	0	18	0	0	0	0	0	0	0	0	0

1a02c23c7b1d8286827f8cc3dba6a5c9	0	0	0	0	5	0	0	0	0	0	0	0
c71a8604ef77761974bc34b043162dd8	0	0	0	0	4	0	0	0	0	0	0	0
3f8a603b03870f730c7bd845e030b73b	0	0	0	0	0	0	0	0	0	27	0	0
6888e980cee36deb997176c206138e88	0	0	0	0	0	2	0	0	0	0	0	0
0edd301094daf2de82ed68891c5dc285	0	0	0	0	0	0	0	0	3	0	0	0
8e926e98c1e58b13ba908bc7e0d79988	0	0	0	0	0	0	0	0	0	0	0	2
ff805d7197b29e5d46bcabe4f73c7654	0	0	0	9	0	0	0	0	0	0	0	0
6c6f4511e0206cbe20ecb33d1583db86	0	0	0	0	0	0	204	0	0	0	0	0
2e21a9a3ee740c3193c393b87c505d97	0	0	0	12	0	0	0	0	0	0	0	0
1a9d8b8842bb1632e219ab289bd8fcda	0	0	0	0	0	0	11	0	0	0	0	0
31411756ae391cb542363c1842b65e8e	0	0	0	0	0	0	0	0	0	0	0	3
9f4ea175e5c487d1356bf516bab48ed0	0	0	0	0	0	0	0	0	0	0	6	0
374f37af30d62a84261d4ef2b1e1eb8d	0	0	5	0	0	0	0	0	0	0	0	0
8aeae6d11ec2f59f957b857a56ba5ad8	0	2	0	0	0	0	0	0	0	0	0	0

09a063ec104ad235c7025ad5b0231693	2	0	0	0	0	0	0	0	0	0	0	0
a517f9d82115805890365f9a63d4ff7f	0	0	0	0	0	0	0	0	0	4	0	0
76089eb26b56620e1911dcfdf5e600be	0	0	0	0	4	0	0	0	0	0	0	0
791ec15fa11029cc0c4844854ced8756	0	0	0	0	0	2	0	0	0	0	0	0
69d05c1dbaea10bda0327aeeaef9de1e	0	0	0	0	0	0	0	0	0	3	0	0
975a6ce96055c00cf9b48c6613ad90d2	0	0	0	0	0	0	0	0	10	0	0	0
4248f25b865306eaf9cfc3b388a2d46d	0	0	0	0	0	0	0	0	0	4	0	0
82b76feeb3d31354ae74ada2054fcdb1	0	0	10	0	0	0	0	0	0	0	0	0
e0f5ad5f7e369bd5e0d07bb42b214608	0	5	0	0	0	0	0	0	0	0	0	0
5f926bb2d13f82a56e5f09a1cf8c91cf	0	0	0	0	0	0	0	0	0	14	0	0
0cc3089af2d4a739fceb4c8e8e765c10	0	0	0	0	27	0	0	0	0	0	0	0
57eae30b131ceb33d225761d02da708a	0	0	0	0	0	0	0	0	0	11	0	0
b0e20d3284e658274416d494e5647716	0	0	0	0	0	14	0	0	0	0	0	0
a7bb3ece0b242ab78bb045758ef28a25	3	0	0	0	0	0	0	0	0	0	0	0

388b4af38f7223036694af0a78331455	0	0	0	0	0	0	0	0	0	134	0	0
60442addc9dd5233440686f80127cb83	0	0	0	0	0	0	0	0	0	0	0	2
8d17a628d3c5fd3ba7f75d1f1cb63e7f	0	0	0	0	0	4	0	0	0	0	0	0
139572855cada202c51003f3c2795148	0	0	0	0	0	0	0	0	0	35	0	0
9c0a5eb279a585fba9f8ecd38b8bce65	0	0	0	0	0	0	0	0	0	0	14	0
73404629cad586f6810b0aa7bea22b95	0	0	0	0	0	0	0	0	0	9	0	0
f0c176236a21d3d7487ce1b35407435a	0	0	0	0	0	0	0	0	0	3	0	0
e99e78fccfb5a9a37700c22a8cb98116	0	83	0	67	0	0	0	831	0	0	0	0
f8557e12a87d2f03d50dd4c825ef9342	0	0	0	0	0	0	0	0	0	3	0	0
877d7b3087de408d1b4ecbf0b13e3baf	0	0	2	0	0	0	0	0	0	0	0	0
ec13c47cfb025deca2d09a204aa0256c	0	0	0	0	2	0	0	0	0	0	0	0
255c43ecd5ce7da9920065355cdf6578	0	0	0	0	0	0	0	0	0	0	5	0
83706e0751d1ad7e005418eaccd321df	0	0	0	0	0	0	5	0	0	0	0	0
1d0a1f4e49c66d97af7f703eefcd215a	0	0	0	0	0	0	0	0	301	0	0	86

ccf2e695ebdbf325a4d901fc1bfec9af	0	0	0	0	0	0	0	0	0	0	0	9
5553f507b95a79304cffdb3a287abae1	0	0	0	0	0	3	0	0	0	0	0	0
e1b85fd70c628c2629129d76a4fc15cb	0	5	0	0	0	0	0	0	0	0	0	0
3fcd128aebc375217eff43397e2aeb4e	0	0	0	0	0	0	0	0	0	0	8	0
6cf6340403b7d0c578c63d76b8eefa38	9	0	0	0	0	0	0	0	0	0	0	0
3957375d042ebc2be8845e75037ec91b	0	0	0	0	0	0	0	37	0	0	0	0
b71b0d7b231ee087dd68d26e32a929e5	0	0	0	0	6	0	0	0	0	0	0	0
bab263edcc324aad76179ec4a6b8b159	0	0	0	0	0	0	0	0	0	7	0	0
bda74d77dc5e6e677b92fe8039ff846a	0	0	0	0	0	0	4	0	0	0	0	0
31dd68a006f9b01cc300debae779b255	0	0	0	0	0	0	0	0	12	0	0	0
2cf5d18e750567cade8fb1bcbd02e2d4	0	0	0	0	0	0	9	0	0	0	0	0
ce7932e6cadeba944e43f1a9da1dfa7c	0	0	0	0	0	0	0	0	0	0	4	0
8452cfd2b9a7f016fb79fc3dcfe85445	0	0	0	0	0	3	0	0	0	0	0	0
6e51b81e1fd2af7ec12076ed6101133c	0	0	0	0	0	0	3	0	0	0	0	0

b6073ef006c5dbed18205ed92f74f438	0	0	0	0	0	0	2	0	0	0	0	0
b81e133c232ea57c00dcedcedfb9fdbc	0	0	0	0	0	0	0	2	0	0	0	0
811e452f6d3460367d66d3b9dc0adfd6	0	0	0	0	30	0	0	0	0	0	0	0
afcaab045012a737454af4fb3c8008fa	0	0	0	0	0	0	0	0	2	0	0	0
c821029c188ac5a0ce2f039c1e6fd2be	9	0	0	0	0	0	0	0	0	0	0	0
b1dd3e69c722548c79d2ebbeea899245	0	34	0	0	0	0	0	0	0	0	0	0
1e6170d403a1f43adfa61dca5d88b702	4	0	0	0	0	0	0	0	0	0	0	0
e52f7e87a1439fcf559c02a7466cadaa	0	0	0	0	0	0	0	0	0	0	7	0
c884db5def799d09a233a544dc87195c	0	0	2	0	0	0	0	0	0	0	0	0
4fc41b09cd2f452e5034153ae991552a	0	0	0	0	0	0	0	0	0	0	10	0
46dd1bad6ae431c7fd3b1b5b34d0f3de	0	0	0	0	0	0	4	0	0	0	0	0
5dac31c473ec986c44965f279adda76b	0	0	2	0	0	0	0	0	0	0	0	0
c1dea0a29e6fb1fd4014e696aa4d2442	0	0	0	0	0	2	0	0	0	0	0	0
261e6e9ca230c992cda7386622ada823	0	0	2	0	0	0	0	0	0	0	0	0

08432c6a1d2a169bb679da97e47ca243	0	0	0	0	0	0	0	0	0	0	0	4
757beb31483316b1bd9bcb879d3645f9	0	0	0	6	0	0	0	0	0	0	0	0
154534e6073031daf6b2925343b344e4	0	0	0	0	3	0	0	0	0	0	0	0
650cfdb26ec7a2d8b09a8f6d2f9c336b	0	0	0	0	0	0	0	0	0	14	0	0
d0e283a7c2034375c94650d274b9eb01	0	0	0	0	0	0	17	0	0	0	0	0
8b2e39c0dc2748a4de05a75009ab672f	0	0	0	0	0	0	0	0	0	0	3	0
b4aef30cf0e2b26124cac1b1d22ec0af	0	0	0	0	0	0	16	0	0	0	0	0
ff65e6e07821cc450cae1d7c6a970bbb	0	0	0	0	0	4	0	0	0	0	0	0
2dac3dbbaf6d943b6aa5f3cbdaabd4e7	0	0	0	0	0	0	0	14	0	0	0	0
caea8344418076c7d28a85a4e49f0c00	0	0	0	0	0	0	0	0	0	0	5	0
f6f34d19145d3eedd734adfe0a569806	0	2	0	0	0	0	0	0	0	0	0	0
55fb7682906d41548f72deef6562afdc	0	0	0	0	0	0	0	0	0	24	0	0
6a407509d0671f6a9389049c8278f841	0	0	0	0	0	10	0	0	0	0	0	0
cb598a23362183acc0907b5f6512e095	0	0	0	0	0	0	0	2	0	0	0	0

1dbfbe2bd207e9b6327f33384003854d	0	0	60	0	0	0	0	0	0	0	0	0
9fb56989f13b2f7f31124075bef57c57	0	0	0	0	0	0	0	89	0	0	0	0
f505046f47963e3003cd9ba7fef842e3	0	0	0	0	0	0	0	0	0	0	0	24
24f11ac732a52ebf36731300b0666b82	0	0	0	0	0	0	17	0	0	0	0	0
8400fbab29e042ac498db05c644cb053	0	0	0	0	0	0	0	0	0	0	4	0
d27a586244fafa64bbb5065c2e6e55d7	0	12	0	0	0	0	0	0	0	0	0	0
0190c0ad5a5544f6a38cfa3aec1b143c	0	0	0	0	0	0	0	0	0	0	0	2
cd616069d02f2e7f4ee8b4fce4cd36f5	0	0	0	0	0	5	0	0	0	0	0	0
448abdcfb930c56df8d91afc89f38e63	0	0	0	0	0	0	0	0	0	0	38	0
19b9ff923605f158416c9ccd9fa955b7	0	0	0	0	0	0	0	0	0	0	0	20
499abba86cdb8661c86af15bb239c046	0	0	0	0	0	5	0	0	0	0	0	0
70791d7a7ce07b888cc60ac8f6af0f3c	0	0	0	0	0	0	0	3	0	0	0	0
86a7c0926a6216a24eba9986788dc758	0	0	0	3	0	0	0	0	0	0	0	0
156e9c711bd63279c701910b0d431734	7	0	0	0	0	0	0	0	0	0	0	0

fe82981ccfe3038f5f9ecc137e9507a7	0	0	0	0	0	0	0	0	0	3	0	0
ee225706ec62d9aaf73bc32a984095c3	0	0	0	0	0	0	0	8	0	0	0	0
69094d853d6299eea94f3d4bc91fbf72	0	0	0	0	0	0	0	0	86	0	0	0
938e189f96bec58b18e96a355988b9b3	0	0	0	0	0	0	5	0	0	0	0	0
eb82d73227b5e8088dc7b7f6eec9924b	0	0	0	0	0	0	0	0	2	0	0	0
32d860daf07b0891ae8b122e1812809e	0	0	0	0	0	0	0	0	38	0	0	0
49cbb199eb2a6227c7c173214384e531	0	0	0	0	0	8	0	0	0	0	0	0
5d9bdc959ba9055fa5b8e153303f2159	0	0	0	0	0	0	0	0	0	0	0	3
a2a5940df50ecc4113a591fb22fb3574	4	0	0	0	0	0	0	0	0	0	0	0
5217cf18c7830d68908763ab0a163506	0	0	28	0	0	0	0	0	0	0	0	0
11eece86e8675feb80efa45ff2dd5c9b	0	0	0	0	0	0	0	0	0	0	7	0
40fa610e51bb717e3cc2b3a9304a43df	0	0	0	0	0	0	0	0	0	0	3	0
051383393176fa1c067c9eeda01b2ec7	0	0	0	0	0	0	0	4	0	0	0	0
182fe436a413d9d6223b407babeb68a4	0	5	0	0	0	0	0	0	0	0	0	0

f546afe09e2cc048a6a4fe237d51252c	0	0	0	0	0	2	0	0	0	0	0	0
9627885a4a3a4eaabec1f183aabab62d	0	0	0	0	0	0	0	6	0	0	0	0
9b46ac15e80c880d36deb23c43831945	0	0	0	15	0	0	0	0	0	0	0	0
adb2b6f9f64fb04432854b72a1ebe021	0	0	0	0	0	0	0	0	0	0	22	0
fe4130aa28ced351efc6b8aa2c4c89d3	0	0	0	0	0	0	0	0	0	0	10	0
e7f9c85050083684658f951ced5ec1d3	0	0	0	0	0	0	0	0	0	0	5	0
aec7c88463d0be25a8b5e66692923b33	0	0	0	0	0	0	0	191	0	0	0	0
bec8baa43d8680231d73e848cfb73ae4	0	30	0	0	0	0	0	0	0	0	0	0
0c83f236ce65782be26693eed24b2859	0	0	0	0	0	0	4	0	0	0	0	0
9e8a335128e2685d509d55ed82131a3f	0	4	0	0	0	0	0	0	0	0	0	0
9a33961e4e198738249de4028e15e602	0	0	0	0	0	0	0	31	0	0	0	0
00e00fc500d9836e899ebc497bc0628f	0	0	0	0	0	0	0	0	0	6	0	0
a81d04a82888dd466ddc9ae6a39eeeae	0	0	0	0	0	8	0	0	0	0	0	0
30feb5a60d91b8a84abc363b63a441a8	0	0	0	0	3	0	0	0	0	0	0	0

fab47d568f1acf57f8fe46125b00fb6c	0	0	0	0	0	0	0	0	0	0	2	0
cb7e40cc7bc665df32fce343a365376d	31	0	0	0	0	0	0	0	0	0	0	0
37588975f3b9eb0ed3669cd424b09d34	87	0	0	0	197	0	0	0	0	0	0	0
6c7af0335b0e7e75f72f9c6908912325	0	0	0	0	0	4	0	0	0	0	0	0
292d3cab8447ff2c59f5dc3924f1d779	0	0	0	0	108	0	0	0	0	0	0	0
73af3784c970785ec8e0a95deecfad16	0	0	0	0	0	0	2	0	0	0	0	0
1e619b7b3c195dd4de4fbfec596f9734	0	0	0	0	0	0	0	4	0	0	0	0
f982bad696737f8adc8b7fbbf6e89935	0	0	0	0	0	0	0	0	0	0	2	0
ee19a5e2cc879e0d11729aaf29d02e91	0	0	0	0	0	0	0	3	0	0	0	0
01bb63d221595fc52cb90488ad4dfdff	0	0	0	0	0	0	0	0	0	0	0	3
1a53004dea48a675815a23c712283b44	0	0	10	0	0	0	0	0	0	0	0	0
abc620a3783706d6c5560d2ae4721d1d	0	0	0	0	0	45	0	0	0	0	0	0
5b1a696f60e81b56563c9446bf69c32a	0	0	0	0	0	0	0	0	0	0	0	2
328a1df640f4a1a512220936449e2693	0	0	0	0	0	0	0	0	0	0	14	0

21a45822f1333c185168b9529aaecf9b	0	0	0	0	0	0	0	4	0	0	0	0
4c47318a67d51bba5ea36f6fdaa95d87	0	0	0	3	0	0	0	0	0	0	0	0
3c73ba2001ec75a5eefe3d69fee96212	0	0	0	0	0	0	0	0	0	0	12	0
66652898175ee41b8add6b9820c4768c	0	0	0	0	0	43	0	0	0	0	0	0
371a4a6fb7113ad6e07d39345c389e2a	0	0	0	0	0	0	0	280	0	0	0	0
35089474e46338b61c5391bce8797469	0	0	0	0	0	0	0	0	0	0	112	0
331d9acf81d43425fc55d0716d86ffc9	0	0	0	18	0	0	0	0	0	0	0	0
ca3172203a6679f4c7368a8c0ffe9811	0	0	0	0	0	0	0	0	0	0	0	8
808b3cde2cb61686bfd38d779f22ab53	0	0	0	0	0	0	0	3	0	0	0	0
11874a5d6dd67a47b4d162a2e348da65	0	0	0	0	0	0	0	0	0	0	0	8
4740490ee7c2e27f6632036a98a3f488	0	0	0	0	0	0	0	3	0	0	0	0
1a7698f9871eb160a6a7c8ea8ba72874	0	0	0	0	0	0	0	0	0	0	13	0
c7edba5f53632f5568b07c4ca436cbe9	0	0	29	0	0	0	0	0	0	0	0	0
18fc97729b5b0731b1d965b7469fdb04	0	0	0	0	0	0	0	0	0	0	0	2

c2836111d5fc5ff68e0aa0b102a9eec7	0	0	0	0	0	0	0	0	0	0	0	14
e991797ef85a414858c9d5e480ca4c76	0	0	0	0	0	0	0	4	0	0	0	0
a30979484554b27c7e3dbd175bdec154	0	0	0	0	0	0	0	0	0	0	0	3
a382b9ac77e3d59f132095cdd205f378	0	5	0	0	0	0	0	0	0	0	0	0
4e6d4dbe17226378ccab162520c76ce0	0	0	0	0	0	0	0	0	0	2	0	0
3b387f85ec62f972b4402976d3460894	0	0	0	0	0	0	0	0	0	0	0	3
df842f1cdf5f611150ed194611f02392	0	0	0	36	0	0	0	0	0	0	0	0
05056e1eb109c3279888d912f5e03be6	0	0	0	0	0	0	0	0	0	0	0	22
4095f8c51bb49ca48025a3140edd6ec2	0	0	0	0	0	0	3	0	0	0	0	0
e0d75cbd529ba79fe934cd42a483188b	0	0	0	0	0	0	0	4	0	0	0	0
a0d1f8a38cf74f5117e3ac67f07ba099	0	56	0	0	0	0	0	0	0	0	0	0
b70df1dfd2d88d592dcda2d52cbac1eb	0	0	0	0	0	0	37	0	0	0	0	0
cbfb10a628b7223052b4393d62438e2d	0	0	0	0	0	4	0	0	0	0	0	0
3716a92af3dfebbcf60e910b86f6193b	0	0	2	0	0	0	0	0	0	0	0	0

d74724237a956a17f58ba420db826367	0	0	11	0	0	0	0	0	0	0	0	0
0be2a582fa4634c914252729fbea310f	0	0	0	0	0	0	0	0	0	0	4	0
87761db470f228f1015222f11a8672d8	4	0	0	0	0	0	0	0	0	0	0	0
f5d26668cc97bfa6ac96cb22a7d57495	0	0	0	0	0	135	0	0	0	0	0	0
a511ab9e9c02a4877ec8acb95b2fdd94	0	0	0	0	0	0	0	0	0	4	0	0
ccda0c8ff5419f4fcc013273acfe15b1	0	0	0	0	0	0	0	0	0	0	2	0
1699776eb1684bcbb458b9b77d375502	0	0	0	0	0	0	0	0	3	0	0	0
fc3e57f52b7b87eedeb0c86783462e3e	0	0	0	0	0	0	0	0	0	0	4	0
55c0f0f27a05e228e8821a6435a2d4df	0	0	0	0	0	0	17	0	0	0	0	0
6de85cfd1fbe7b7c54f5d3e4761a91ba	0	0	0	0	0	0	4	0	0	0	0	0
558c811cdcc680dc07d9a3f00b4697a5	0	0	0	0	0	0	0	0	0	0	45	0
6b2730cf91739f18d13ef641412b4579	0	0	0	0	0	0	6	0	0	0	0	0
d85685c8d22a72f9e3d0cd270d843dfb	10	0	0	0	0	0	0	0	0	0	0	0
2615e5f30fe681497921f296fc8360a6	0	0	0	0	0	7	0	0	0	0	0	0

c0a1146d122f37bd2ba1c8127f1f861e	0	0	0	0	0	0	0	0	17	0	0	0
633fe5e306ff04f669de351b7d25e44d	0	0	0	0	0	0	0	0	0	0	35	0
5ab443644542263c0f132d436a6b1f87	2	0	0	0	0	0	0	0	0	0	0	0
71055cafee9661c40611b7d8b9199b8d	0	0	0	0	0	0	0	2	0	0	0	0
d7a66d5966b8fa34bf8d099c414dd6c0	6	0	0	0	0	0	0	0	0	0	0	0
a96ecdad84bedf65df866ac3e308eb2d	0	0	0	0	0	0	0	0	0	3	0	0
96a7cc3f7087b77a838278dcb4c96737	0	0	0	0	0	0	0	0	0	0	0	11
1e7fa3a55d87a14f197afa16867f8403	0	0	0	0	18	0	0	0	0	0	0	0
1c5c17cd91c75954bbd5933fa0f23a8e	0	0	0	0	0	0	0	0	0	3	0	0
06dee67432ef950cd361a86e406dd961	0	0	0	0	0	0	10	0	0	0	0	0
eb4ec83503b5a694ae41ea1ed203bc18	0	0	0	0	0	0	36	0	0	0	0	0
0cbf04ff68f8a30af38107f8908b2f73	0	0	0	0	0	0	0	45	0	0	0	0
e5c8213e436544e61929e680373100c0	0	0	0	0	0	0	0	0	6	0	0	0
0017a4a9c47ceee4a307a7fa30970f22	0	0	0	0	0	0	0	10	0	0	0	0

10d7dceb23bba0963d8cfa4eea9f954a	0	0	0	0	0	2	0	0	0	0	0	0
2d91d29b2ae8bf48dfd6e54b6702b0e8	0	0	0	0	0	0	0	104	0	0	0	0
b3a50741ff7eb204ed0b6fe5c80c6361	0	0	0	0	0	0	0	0	0	0	30	0
3bb7814c7bae1df601dd56c2bf479314	0	0	0	0	0	0	0	0	0	0	8	0
e2425192c5526b44a4f9cbdd03277bfe	0	0	0	0	12	0	0	0	0	0	0	0
2eb46ac60994372b6dbe5ac7b8928b57	0	0	0	0	0	4	0	0	0	0	0	0
ecc510c47a677e4e87a4bba1a69e8e79	0	0	0	0	0	0	0	0	0	0	7	0
629521bd44b8ae77e4b3d7c3d374da32	0	0	0	0	0	0	0	0	3	0	0	0
74c38e23e9659f9affe62deb5fda8886	0	0	0	0	0	0	0	0	0	0	0	9
2caa948e00a09f28a80ddcb86a7dd168	0	0	0	0	6	0	0	0	0	0	0	0
caa152db7981b89f8374931892b7b496	0	0	0	0	0	0	0	0	0	38	0	0
bedb6690418f002435dfc7ce234c57b8	0	0	0	0	0	0	0	0	0	0	0	35
e57d2213a4b068748cdc27eca4e263c1	0	0	10	0	0	0	0	0	0	0	0	0
5d108826b4b81a4530c9fba6ab230a54	0	0	0	0	0	0	0	0	0	0	3	0

1f80ae598b115f302a2c0b68 6f1e7303	0	0	0	0	0	0	2	0	0	0	0	0
c22f5c433b166b3c01fce2b79 957f44d	0	0	0	0	0	0	0	13	0	0	0	0
e91e73a1642187ff75e39bf6 8498c8f3	0	0	0	0	0	0	0	0	0	0	0	2
2db9caa86ffb26e6e4eca036 ba0251c7	0	0	0	0	0	0	0	0	0	0	8	0
2d80523cb9aeac6d0092bcf1 1083e74c	0	0	0	0	0	0	0	0	0	0	3	0
1ce4fdc88b3251630e4abeda c03a9082	0	0	26	0	0	0	0	0	0	0	0	0
c757984cf86afa890c6e1f907 49ca71a	0	0	2	0	0	0	0	0	0	0	0	0
045bcdb71fcf09b8762d9ba9 c4f175dc	0	0	0	0	0	0	0	0	10	0	0	0
7e22d284ea2fafd7054a9b09 ccd700a3	0	0	0	0	83	0	0	0	0	0	0	0
cc487b9b5f4e8842a5f36e70 34f69137	0	0	0	0	0	0	0	0	0	0	9	0
cb6d261cb5fb2ed94f0ef2f80 96a5c35	0	0	0	0	0	0	0	0	2	0	0	0
fbb890a8a75f0b65d70f9d5a 38e26ad2	0	0	0	0	0	0	0	0	0	0	0	4
e7539515c7036d171af7c355 5b3dc489	0	0	0	0	0	0	0	11	0	0	0	0
a7340e42523206aeccd6e322 d02a89fa	0	0	3	0	0	0	0	0	0	0	0	0

62a63b526e28a2251302a57757a9a841	0	0	0	0	0	0	0	0	0	6	0	0
d7dad2d561970aba14f4a0daefaecba1	0	0	0	0	0	2	0	0	0	0	0	0
11f3aad75c3a266e692423d3f54ed3a3	0	0	0	0	64	0	0	0	0	0	0	0
d782379e5e419a0d80495b1682a8e1c9	0	0	0	0	6	0	0	0	0	0	0	0
2b5fcafb001419deb05dc090965d02f2	0	0	0	0	0	0	0	0	2	0	0	0
ce50897141eff7d22d0d7b5955f839d7	0	0	0	3	0	0	0	0	0	0	0	0
833f63fcb49c71a0e402e8c9bdf39974	0	0	0	0	0	0	0	0	0	0	36	0
5b1388667f1eddbd3b6bac2b6bf4b5d1	0	0	0	0	0	0	0	4	0	0	0	0
974e8975500e9a0af3e652a002cddbb3	0	0	0	0	0	0	0	0	0	0	0	31
d302df94d395a89239704676b4e3331b	0	0	0	0	0	0	0	0	9	0	0	0
ad0185d7cb8bfa73ea8dbefce71ca8e9	4	0	0	0	0	0	0	0	0	0	0	0
f2ca4c4a93ba2f6893b58c91504d6c4e	0	0	0	0	0	4	0	0	0	0	0	0
f9a2f416893d64e42689c41775ae57f1	59	0	0	0	0	0	64	0	0	0	0	0
fb6ecc3e43022d31743325348ee466d6	0	0	0	0	0	0	0	0	0	0	4	0

4ac7e156c1ee78d5c931ed30 3903e1db	0	0	0	0	0	0	0	0	0	0	4	0
f4da558da9f693cc35611153 0d797d66	0	0	0	2	0	0	0	0	0	0	0	0
227e0dacfb93e78fe01f1203e eebb98f	0	0	0	0	0	0	0	0	5	0	0	0
ce70b371fc11dee6c0d32646 fa649cbd	0	0	0	0	0	0	0	7	0	0	0	0
722e8abb520a16cd3d7cbb5 309a076e8	0	0	0	0	11	0	0	0	0	0	0	0
0023a02e3dcc96704d7d5de ddd900267	0	0	0	0	0	0	0	0	0	0	0	7
7e875558717401b6117fac6d f146a5c3	0	9	0	0	0	0	0	0	0	0	0	0
573f910a49c1f689211050b7 b53bb592	0	0	0	0	0	0	0	22	0	0	0	0
886d7b8ace60c2caae968377 e19e0bd9	0	0	0	0	0	0	0	14	0	0	0	0
8d5e2918b5bdb4e20a24407 7a63ec706	0	0	0	0	0	0	0	29	0	0	0	0
c3f606763bee3156d26af032 4c42fc88	0	0	0	0	0	0	0	0	0	0	3	0
9d191789ae601952b4c5fb47 a750f08d	0	0	0	2	0	0	0	0	0	0	0	0
858cce98932c2edf101f0891 7f09b686	0	0	0	0	0	0	0	0	0	8	0	0
c3ae6a78a1e1f5de81d43ba9 90d4ee1f	0	0	0	0	2	0	0	0	0	0	0	0

ff368fafbb99ba664f66373bf6707ecb	0	0	0	0	0	4	0	0	0	0	0	0
586f3e9e5caf27cd3405e5767613bf77	0	0	0	0	0	0	0	0	0	2	0	0
d1dd7a6049a25263d992fa727bc8da90	0	7	0	0	0	0	0	0	0	0	0	0
51f5c0b21f87553bfa143e43a07febd6	0	0	0	0	5	0	0	0	0	0	0	0
df71edec2e1e15c68c60a1f3177e9691	0	0	0	33	0	0	0	0	0	0	0	0
de636017c8d0c1d58689c43662dd470b	0	0	0	0	9	0	0	0	0	0	0	0
a7d23e4b8dae66289563bfecb45f0c46	0	0	0	0	0	0	0	2	0	0	0	0
e01f6f777b909fa535ef90f38ba37a4b	0	0	0	0	46	0	0	0	0	0	0	0
b21aaadde353e8239d076a0bdb6c5b5b	0	0	0	0	0	0	0	0	0	0	0	2
537b0cc847c4987b23ae841c04c6093a	0	0	0	0	0	0	0	0	0	9	0	0
3e8300bcc0f3c41e132a03c3a8a00659	0	0	0	0	0	0	0	0	2	0	0	0
db45c70bb2bb14e340e83e010eb5ffd7	0	0	0	0	7	0	0	0	0	0	0	0
b3636b036e916bf03fb59edbc7073e00	0	0	0	0	2	0	0	0	0	0	0	0
14e44e07d0917999d35406692681ef76	0	0	0	0	0	0	0	0	0	0	0	6

d797eb6c3e7e87d87d89774a8b1df224	0	0	0	0	0	0	0	5	0	0	0	0
18859dbf0af3a8d210cf9c0fc02a5235	0	0	0	0	0	0	0	0	0	0	11	0
4af6bf3e009bd46578de779ca3f666d3	0	0	116	0	0	0	0	0	0	0	0	0
d73e45faeeff08f0c06fe00c4a2596d6	0	0	0	0	0	0	0	0	0	0	8	0
ab6b7a59e5cee12efca497999fe0e836	0	0	0	0	5	0	0	0	0	0	0	0
7f143ebe1a399755ca1f3aea4f99f74d	0	0	0	0	0	0	0	0	0	3	0	0
f9f66b0f0a60e544bfd1a9ba44d32964	0	0	11	0	0	0	0	0	0	0	0	0
3a8255028c144b29aa3befa2bb080353	0	4	0	0	0	0	0	0	0	0	0	0
0d37e6ec3c8bf493cc354d098bf614c0	0	6	0	0	0	0	0	0	0	0	0	0
9ef7cdfedccffa24480bc8b69fed5962	0	0	0	0	0	0	0	11	0	0	0	0
1a021d6ca69c94064bad5ddfaa07828e	0	0	0	0	0	0	2	0	0	0	0	0
cb13ab80e6f5a550ec689612596bae0a	0	0	16	0	0	0	0	0	0	0	0	0
248e9b30f1390fbd7b9981a270a9afc5	0	0	0	0	0	0	0	0	0	0	3	0
68471941528a1a927cf703ecac538f11	0	0	0	5	0	0	0	0	0	0	0	0

3b57e1a504b94915cd3d2c0b8249532d	0	0	0	2	0	0	0	0	0	0	0	0
0832070cb4bfc572527648ea895c75fa	0	0	0	0	0	0	0	0	0	0	9	0
2512b0369bd388a24afe2d17508e4ff0	0	0	0	0	0	0	0	0	0	0	6	0
2c753714f329d3a9bdc21664e421bbf8	0	0	0	0	0	0	0	0	0	0	12	0
d766fe206f8acf953ccdd408cc2e0717	0	0	0	0	0	0	0	0	0	0	5	0
4d82ee6f60ee2344e013bd31c9367eb8	0	0	0	0	0	10	0	0	0	0	0	0
13828fd76826a7941ddfeef96842486f	0	2	0	0	0	0	0	0	0	0	0	0
0a14c5dde29974597551807f5d43313e	2	0	0	0	0	0	0	0	0	0	0	0
7846bc9023077c1806f15e4ee743e632	0	0	6	0	0	0	0	0	0	0	0	0
330262c0eefc5490c0b04cc5d1808589	0	0	0	0	0	0	0	0	0	0	0	9
467da0be8427703e67eea1b6c1bd2b91	0	0	0	0	0	0	2	0	0	0	0	0
da2e9ed7b2855a438bd0cc8405100f15	0	0	0	0	0	0	0	12	0	0	0	0
d8f4027b0c930629b0c9a809020e5323	0	0	0	0	0	0	0	0	0	108	0	0
2183ef588a8ba1b5d9e5960da82bb04b	2	0	0	0	0	0	0	0	0	0	0	0

e9785ffddc71abd792280c3ce06dbbfd	0	0	0	0	0	0	0	0	0	6	0	0
97161db3435108d7fd6a96d20397e438	0	0	0	0	0	0	0	0	96	0	162	0
be47e893b86d8964c245f17df4fcf5de	0	0	0	0	0	0	0	2	0	0	0	0
5895f2687fb977a627a8c2eabf9465c7	36	0	0	0	0	0	0	0	0	0	0	0
18c2308735803d7edc2e754f119e9482	3	0	0	0	0	0	0	0	0	0	0	0
4c35558692eccab46c27954c5183f247	0	0	0	0	0	0	0	0	0	0	3	0
55391a725a6a3b09007aefb3e537db0b	0	0	0	0	0	0	0	0	0	4	0	0
a882baa1183b3a575310534ef3ec40ef	0	0	0	0	0	0	0	0	0	0	8	0
97b0788cfb96ca8e124ff1324a24a3ba	0	0	0	6	0	0	0	0	0	0	0	0
90aef149e896a4a4430ad5120cc50e94	0	0	0	0	0	0	0	0	3	0	0	0
1901731351bde71cc3049aa887483835	0	0	0	0	0	0	0	0	0	0	3	0
76a5d3d105a7820e5131fdfecbf6ce63	0	4	0	0	0	0	0	0	0	0	0	0
da5320705f2f8aef146face65f2e03a7	0	0	0	0	4	0	0	0	0	0	0	0
bd125368bac5f709937c6d25f1fe9b6b	0	0	0	0	0	0	0	2	0	0	0	0

03b569c060e7e76cc0fe5717 6ea0ce25	0	0	0	0	2	0	0	0	0	0	0	0
25d0333fa2b1a3cbc5049291 50366859	0	0	0	0	0	0	0	0	2	0	0	0
3ca1ed2a2db67629a313d22 2ca950ffa	0	0	0	0	0	0	0	0	3	0	0	0
505e99e5e9c8e1f9f70fd77cf 4be4fd8	0	3	0	0	0	0	0	0	0	0	0	0
d4036d52138e2b42ec16775 e91fc4dd5	0	0	0	0	0	0	0	0	0	0	46	0
bdc965c465b3fb2266b462d2 e3074395	0	0	0	0	0	0	0	0	0	7	0	0
23b9234ac4f3fb9f69056d75f 45a1bcc	0	0	0	16	0	0	0	0	0	0	0	0
64ca0cf81e42fb96d5f0baaab 4ea0fe2	0	6	0	0	0	0	0	0	0	0	0	0
6c7178be0fa20ea84bd962c5 1718be57	0	0	0	0	15	0	0	0	0	0	0	0
2d749a4ef7f6b951e119e530 b2e06cd6	0	0	0	0	0	0	0	0	0	0	0	4
fb27dc49f51f9fd8a478de6c0 5bbb62f	0	0	22	0	0	0	0	0	0	0	0	0
79a4beb25dae43cc3c535b0f 9337823e	0	0	0	4	0	0	0	0	0	0	0	0
5be3b6703102164711e320b a5104c626	0	0	0	7	0	0	0	0	0	0	0	0
420469619808edcb59b89f59 16ab184d	0	0	0	0	0	0	0	0	0	0	3	0

7f53fa12181e917d8168dc2b ab3ae4bd	0	0	0	0	0	0	0	0	0	3	0	0
f1dfce368b604d9858c324efb 5597a3d	0	0	0	3	0	0	0	0	0	0	0	0
c39bd14db29bf6ab3fcd275e 56a97e54	0	0	0	4	0	0	0	0	0	0	0	0
76d063d0ba03d63318f456bc 2d5d7959	0	10	0	0	0	0	0	0	0	0	0	0
d797486c0723c6225e017b5 470713479	0	0	0	2	0	0	0	0	0	0	0	0
f6a216baeb9c6608043a2134 6e926045	0	0	0	0	0	0	0	0	0	0	0	57
8cae8e7ba488648ed50744bc 85f39942	0	0	0	0	0	0	0	0	0	0	0	2
49efa45f3e2580c2fa45108dc f41b7ed	0	0	0	0	0	0	17	0	0	0	0	0
c2bfbc48d8fa159fa7e3c32a2 d70e9f5	0	6	0	0	0	0	0	0	0	0	0	0
98fcb9d0441965f0f731ccd3a b39f3d8	0	0	0	0	0	0	0	8	0	0	0	0
fe4fd0ba386633bc1b7e515b d69bd6af	0	0	0	0	8	0	0	0	0	0	0	0
7f013da1b1e87b98d69c72b2 b0ccd741	0	0	0	0	0	0	0	0	0	0	3	0
5b15a9bb6c852c54f3d1dd50 722946d6	0	3	0	0	0	0	0	0	0	0	0	0
ef3bc0a141fe2db8fd0c9b34b 80f0ef9	0	0	0	0	0	0	0	5	0	0	0	0

51a17a3a4f4645ce8910ce32ec2c74de	0	0	0	0	0	0	0	30	0	0	0	0
91aa74930f1aab7c37f15baa19736a8b	0	3	0	0	0	0	0	0	0	0	0	0
cb33f730132dc23bce7e4e13652e162c	2	0	0	0	0	0	0	0	0	0	0	0
9fb2d842caf73de4e1673e5834184a3f	0	0	0	0	0	0	3	0	0	0	0	0
8189a6b0569de9d61ea1a8c2182baa3a	14	0	0	0	0	0	0	0	0	0	0	0
47c62ffd50033907b3f7f5e54f43128d	0	0	0	0	0	0	0	2	0	0	0	0
ef1f5f11319e2da9c42d2d8337d35184	0	0	0	0	0	0	0	0	33	0	0	0
87f482027840fd5dd2ecad0ec881fed6	0	0	0	0	0	3	0	0	0	0	0	0
c2a8ce15d92678c961d60f7ceef30cae	0	0	0	0	0	5	0	0	0	0	0	0
ac7e0192bea25395c97c163584b98641	0	0	0	0	0	0	0	0	0	0	2	0
6be71fc2ab8224d8ea90e3697759d17c	0	0	0	7	0	0	0	0	0	0	0	0
4d20da8cb17e55fdd94476d907500279	0	0	0	0	0	8	0	0	0	0	0	0
20621a92117c5b69f94f8ba7a19516ec	0	0	0	0	0	0	0	0	0	0	59	0
b5b169792bfdc23da8d2dac7ec888ece	0	0	0	0	0	0	0	31	0	0	0	0

37a22576f2a8a7f822fe62dd5420407d	0	0	0	0	0	0	0	0	0	0	6	0
e20f6fe7d2756e8e303c5350acbb6634	0	0	0	7	0	0	0	0	0	0	0	0
ac11fb3b31640eae22928dc8a36b286e	0	0	0	0	0	0	0	0	2	0	0	0
18608c2cb03f16d0673d245a0b1da074	0	46	0	0	0	55	0	0	0	0	0	0
41854f1cb147a2d1a1dd2dd4368bc84c	0	0	0	6	0	0	0	0	0	0	0	0
aec2dbf313b4574ae0639c8e8a37dcb2	0	0	0	0	0	0	0	0	4	0	0	0
b176f44f52367ecde80f0fc7eba27616	0	62	0	0	0	0	0	0	0	0	0	0
c98e1202d5d0619f2fb0634568d21bf8	0	0	0	0	0	0	0	0	0	0	0	14
557042bcddc37de92490393171162d58	0	0	0	0	3	0	0	0	0	0	0	0
2b314d40177068b956891b52ac1ecce1	0	0	0	0	0	2	0	0	0	0	0	0
e1563bc52c00856616620c4f4ede4684	0	0	0	0	0	0	0	0	0	8	0	0
135dd8cd0eb028caec64c5ba418752ed	0	0	0	0	0	0	31	0	0	0	0	0
f27236b3cd7e71a81095f472a24165e6	0	0	0	0	0	2	0	0	0	0	0	0
a65f5f7a7e8773a1a106a69f35931387	0	0	0	0	0	0	0	0	0	0	6	0

3d5c519af49c3c9a1e5e7245d44083a8	0	0	0	0	0	0	0	17	0	0	0	0
7712c2468cd857e62c15569f270d568a	0	0	0	0	0	0	0	0	0	0	13	0
c69e13ed17b991aff9af898a799fb443	0	0	0	0	0	0	0	0	0	0	0	20
75f6720ad9927e05d179e026f0f2d9ce	0	0	0	0	0	0	0	0	0	0	0	24
dc340e85dc2502aeb27bf269cf6cbe83	0	0	0	0	0	0	0	0	0	0	3	0
8002172c4ccd1162f7221372ae05eb79	0	0	0	0	0	0	0	0	0	0	10	0
4668d4c9c25ca4ba3453adb09b71f08d	0	0	0	0	0	0	0	0	0	2	0	0
e1c7bcc2298ed5bc6e636ad56d7c4461	0	0	0	0	0	4	0	0	0	0	0	0
84e7efa64f402b32969506457edc52cb	0	0	0	0	5	0	0	0	0	0	0	0
134cd19230156472d504af0d91f79ee9	0	0	0	0	0	0	0	0	0	9	0	0
44d0dcf43b1456443c15ceac68478b65	0	0	0	0	0	6	0	0	0	0	0	0
69abd6c346892ec1c444dcec817b69d4	0	0	0	0	13	0	0	0	0	0	0	0
d5537df0517f576409f6e969ffbb5817	0	0	0	0	29	0	0	0	0	0	0	0
b75ff1f7ec910b4dfcaa632028022e44	0	0	0	2	0	0	0	0	0	0	0	0

133682e0c422cca28df76d09c87d7ce8	0	0	0	0	0	0	0	0	0	0	5	0
ba03564219009e713c26b7d24ac2d5b9	0	0	0	0	0	0	0	0	15	0	0	0
7760d4ae02bd599f273ca96738f45098	0	0	0	0	0	0	0	0	0	2	0	0
f2614f176dd1578c4efb6fcbaf1004cc	0	0	0	0	0	0	0	0	0	0	0	12
9b1bd5f24470576c7f8ac668b22d100f	0	0	0	0	3	0	0	0	0	0	0	0
5baec554100f2d655930c1e896c74497	0	6	0	0	0	0	0	0	0	0	0	0
a1b0c521cb3f837746990f5bc86d8584	0	0	0	0	22	0	0	0	0	0	0	0
9cf5211654df537ec1eca1710f4dd7ff	0	55	0	0	0	0	0	0	0	0	0	0
97052eb690febd7cc72ff358bf1b7742	0	0	0	3	0	0	0	0	0	0	0	0
750508dfd4fb37096158ddf528fdbd78	0	0	0	0	0	0	0	0	0	90	0	0
5b9cfb5f5d88f0bfadabe69621e51c06	0	0	0	2	0	0	0	0	0	0	0	0
53ca80863efac5b5a1329b7b69fcfe80	0	0	5	0	0	0	0	0	0	0	0	0
b67c41deff432105dd17b0a31522299c	0	0	0	3	0	0	0	0	0	0	0	0
a611a8f6efa50f46267035b9dbbe17a1	0	0	0	0	0	0	0	0	0	3	0	0

f007b19624de62c912637f3bff54b075	0	0	0	0	0	0	4	0	0	0	0	0
3cac178f16c72339c54bd19caf2729a8	0	0	0	0	6	0	0	0	0	0	0	0
2e59a2c9ec0b531c4e2250a5c3506d72	0	0	0	0	0	0	0	0	0	2	0	0
06f8596b0b3c0b32fb55570d74a6158e	0	0	0	0	0	0	0	0	0	4	0	0
27460d5176c6ab198a1c1ffa00bb5390	0	13	0	0	0	0	0	0	0	0	0	0
e49d8af0da5f25b47b614215ccb78965	0	0	0	0	0	0	0	0	0	0	3	0
4d8e12ca6a0d2f7ffe1faa25531fa652	0	0	0	0	0	0	0	0	0	0	0	132
8e201832045da7e5edb14688b8de1173	0	0	0	0	5	0	0	0	0	0	0	0
ae6be72ffca654a5ddbfcc36368341ca	0	0	0	0	0	0	0	0	0	4	0	0
d37e58a3fd0d678ff0d672ca72c46b64	0	0	0	0	0	0	0	0	0	0	5	0
e9c95b2f158c5d88a3b8137ebb9d47e8	0	2	0	0	0	0	0	0	0	0	0	0
69cec0585c9b09a27b29402a29da4958	0	0	0	0	0	0	2	0	0	0	0	0
b257527eb988e2f4b65680308b7f397c	0	0	0	0	0	2	0	0	0	0	0	0
bb1bc2845379834c2fb1187ec6d00583	0	0	0	0	0	0	0	4	0	0	0	0

aa56dfdba7dc0dde03d7ee2d2d92686d	0	0	0	0	0	0	0	0	0	0	0	2
b3eeee5fceb6aa833532d8e147a40c67	0	0	0	0	9	0	0	0	0	0	0	0
55cb041f6a71c12af2d5b1a714a37508	0	0	0	0	0	0	0	0	59	0	0	0
d468b89c85278895434e4332122481a6	0	0	15	0	0	0	0	0	0	0	0	0
310a7ade01d8335e58ed0095df15f4dd	0	0	0	0	2	0	0	0	0	0	0	0
56281b97b8f613e0c356262bdf1a8d41	0	0	16	0	0	0	0	0	0	0	0	0
008e3755292beddee65e86f369f04f31	0	12	0	0	0	0	0	0	0	0	0	0
967162171e4089137b55e95c073b7464	0	0	0	0	0	0	0	0	0	4	0	0
50d42d114954b6418328a43adf115b46	0	0	0	0	0	0	0	0	0	0	146	0
e3ec14bce415705c426baa1cc94831f3	0	0	0	0	229	0	0	0	0	0	0	0
f7ea0ac4d0248a41c1a9da32b356d728	0	0	0	0	0	0	0	4	0	0	0	0
ebcbfd38b90d7b7aede1f30e57481b19	0	0	0	0	0	0	0	0	18	0	0	0
53be592a94a23e88af5f3a772ecbb932	0	11	0	0	0	0	0	0	0	0	0	0
d96def83af675e47434f08b374708014	0	0	0	0	0	0	0	4	0	0	0	0

8872840e31bc3f4ff0036fcd730a2089	0	0	0	0	0	0	3	0	0	0	0	0
80b1c496d139c2fadba86a0f5a0935b6	0	2	0	0	0	0	0	0	0	0	0	0
8f1bc711294f1c826f39fa1603690683	0	0	0	0	0	2	0	0	0	0	0	0
db92a542a7f4c9399ee61d871ad24da1	0	0	0	0	0	0	0	0	12	0	0	0
60be2f49ae042867c855877b19cfd524	0	10	0	0	0	0	0	0	0	0	0	0
0baa5714016eb078a76dae00e642efd8	0	0	0	0	0	0	0	0	0	5	0	0
48064258e39458773f3c89cbdc3fe4a3	0	0	0	0	0	0	36	0	0	0	0	0
93b3775a6c104e0acad6644b5f8cd97e	0	0	0	0	0	0	0	0	0	0	4	0
b40f24338aff6ee835d9863c222d1f4d	0	0	9	0	0	0	0	0	0	0	0	0
813a7fa4429eb7e00d6c98fe5ed4daba	0	0	0	0	0	0	0	0	26	0	0	0
9d4fb17f098e9dfe5fd865896a7e6c6c	0	0	0	0	0	0	0	0	0	6	0	0
d103b57d8fe1b8d760eef4a0d93fb48c	0	0	0	0	0	0	2	0	0	0	0	0
75ba20428851b0911b2dc0493b13a47f	0	0	0	0	0	0	0	57	0	0	0	0
664ad32bbe07c0fc9b5ef5d1fedd04cf	0	0	0	0	0	0	0	0	0	2	0	0

7f9888b196dd6c5aabb62c36b5262fc1	0	0	0	0	0	0	0	0	0	0	9	0
bb1f09126e256841433c386217c87532	123	0	0	0	0	0	0	0	0	0	0	0
1e343a1c0d91e510fba7431cb41d572b	0	0	0	0	0	0	0	0	0	0	16	0
5bf628d5837df184081c52b689cdfbd9	0	0	0	0	0	0	0	0	0	0	0	2
9443536df16f8e4678f3bcf134d8eb9f	0	0	3	0	0	0	0	0	0	0	0	0
e311b1e6133ab154061cded6440b97dd	0	0	0	0	0	0	0	2	0	0	0	0
c0ab2b8b5f43c80e93b81e7fedec3e6c	0	0	0	0	0	0	0	0	22	0	0	0
28f6760585779fe4ee3cd086555e018b	0	0	0	0	0	0	0	0	0	0	16	0
53cbbdaa645323e0bc2beeadeb5b22a4	0	0	0	0	0	0	0	0	0	0	28	0
0c903935d9a8cb3d84eb030a9d17433c	0	0	4	0	0	0	0	0	0	0	0	0
c362876550bae5986655e7b350bc5278	0	0	0	0	0	0	0	0	0	0	62	0
646ac62a9a9544d99a943ae4d5ad3224	0	0	0	0	5	0	0	0	0	0	0	0
0216e1c44ae21c0e18ad004454d0efd1	0	0	0	0	0	0	0	0	0	2	0	0
681421f7976d2e50cac494dc42f3279e	0	0	0	0	0	0	0	0	0	0	8	0

6cd64a4cce80aa9f730128fc8c3783c1	0	0	0	0	0	0	0	0	3	0	0	0
ee30c2cd787d21a6eeb9f765ac5317c3	0	0	0	0	27	0	0	0	0	0	0	0
9aedb1c8a0e9b62ee86ae925e419fe80	0	0	0	0	0	0	0	0	0	0	6	0
2fc2b57450e983e8ae6eb9bd78b9d398	0	0	0	0	0	2	0	0	0	0	0	0
44d015911d4e8263d83fb471c32655f6	0	0	0	0	0	0	0	0	0	6	0	0
6dbeb29c1d9591185e8f860c99d2fccd	0	0	0	0	0	0	0	0	0	0	0	2
a7ec7b202abcb0fd527fbc202cf8ec7d	0	0	0	0	0	0	0	0	2	0	0	0
0614bb5a91d85abb04f05936e3fea1a4	0	0	0	0	6	0	0	0	0	0	0	0
417135a6f91be0735fe690969a4ee263	0	0	0	0	0	0	0	3	0	0	0	0
b7841ec1094413ffad3967e8fab3aa50	0	0	0	0	0	0	0	0	9	0	0	0
8206ad373bd984dda152d8c8f30fcc34	0	0	0	0	0	0	7	0	0	0	0	0
44385ac198902fea7b71325d1c93cddb	0	0	4	0	0	0	0	0	0	0	0	0
954f214f9c4ed8cebc4ec70d3a3269e0	0	0	0	0	0	4	0	0	0	0	0	0
bbe9eeba0e583e1bde44a734bababd02	0	0	0	0	0	0	0	19	0	0	0	0

085e2d8a97078abcaec0c3587c8a9518	0	0	0	0	0	0	36	0	0	0	0	0
2cec3f1066fa3bfc9ee81f2e5cfb75c0	0	0	4	0	0	0	0	0	0	0	0	0
c650633d927d2035dc6cbef38823a054	0	0	0	0	4	0	0	0	0	0	0	0
cb6e362f79fb14c9af483604c1e9501d	0	0	0	0	0	0	0	0	0	5	0	0
526d7854720dbdf1694e7aa48c1125c6	15	0	0	0	0	0	0	0	0	0	0	0
cb5d8563bfe360ac2863fcd303c17c33	0	0	0	0	0	0	0	0	0	6	0	0
d934bd1fee2dbd8dc94bfdcaf6e9c6c2	0	0	0	0	0	0	0	0	14	0	0	0
8354c273bcd99576ffb707aa180e5534	0	0	0	0	0	0	0	0	0	20	0	0
36013b7f70a908eb2c73a728da592118	0	23	0	0	0	0	0	0	0	0	0	0
d8bb942101a2fe611eba7b2bbcf79993	0	0	0	0	0	0	0	0	0	4	0	0
ee19c277de23bdf42a9711680acda11d	0	0	0	0	0	0	0	0	0	2	0	0
6b577375cb7352f56bbab182d2f91fb6	0	0	0	0	0	0	0	0	19	0	48	0
98c56949ba3765ed4425d59f6f849673	0	0	0	0	0	0	0	0	0	0	0	55
daf88bb67ef1bd139c189e29182d282c	0	0	0	0	0	0	2	0	0	0	0	0

e7c4696d0ee9f7a2aca930d927934240	0	0	0	0	0	0	0	9	0	0	0	0
666ad8a4d8fc879d9301ae0a939d4ee2	0	0	0	19	0	0	0	0	0	0	0	0
cd401f8127f04d3f8d6b4abdce0004f6	0	0	0	6	0	0	0	0	0	0	0	0
7847e32922e10a6a5cc307376de82154	0	0	0	0	0	2	0	0	0	0	0	0
cff87d84fb8c2c88aad67f6d350c7c11	2	0	0	0	0	0	0	0	0	0	0	0
7ee07e1875476675e6cea8831dd8ffdd	0	0	0	0	0	2	0	0	0	0	0	0
de61a45db7c9b2e4099ad1e52fbb7d42	0	0	0	0	0	12	0	0	0	0	0	0
f1eb703488e0379fc15c9f975c77ce81	0	0	0	0	0	0	0	0	0	0	0	4
7fdf0cd38b6961bc4c19eadc3de2c660	0	0	0	0	0	31	0	0	0	0	0	0
3f11e21fbe50b42100b44b03a6c69e80	0	0	3	0	0	0	0	0	0	0	0	0
7551916a53e94f73dcff3fd95e47b404	0	0	8	0	0	0	0	0	0	0	0	0
1bf0ecd8488393edc6dc39aea721949a	0	0	0	0	0	0	0	0	2	0	0	0
e554c2031488acbc4c9667e5ad3d8916	0	73	0	0	0	0	0	0	0	0	0	0
3ee9e7ffb64cd2b3b8326eb5349c1598	0	0	2	0	0	0	0	0	0	0	0	0

4f5719c5a5b22583b727a1b2f063ea17	0	0	0	0	0	0	5	0	0	0	0	0
36c102f3cebee4088a42a5aadf5df106	0	0	3	0	0	0	0	0	0	0	0	0
fe37b23f240106e7d2b5a5473eacf7ec	0	0	0	0	0	3	0	0	0	0	0	0
6c9af14fa368068fab6e44b598dc3d1b	2	0	0	0	0	0	0	0	0	0	0	0
fdfe06325ddeec6ca4428163843494ef	0	0	0	0	0	0	0	0	0	0	4	0
ddc5fbd7f494ff6b9918eb3204d03699	0	0	0	0	22	0	0	0	0	0	0	0
fb3a3d3a023763f6945d9fbc658d274f	0	0	0	0	54	0	0	0	0	0	0	0
bacf83751277bfa060fdd2ba45a53443	0	0	0	0	0	0	0	0	5	0	0	0
579258620308442369f1b7bbee22d675	0	0	0	0	0	0	0	0	0	0	4	0
90d8a1656ee0d8612c3ffc0ae2d6dd95	0	0	0	0	0	0	0	0	0	0	7	0
bef9543b48cd247cf3736e64acf70843	0	0	0	0	0	4	0	0	0	0	0	0
b90c08ad9d8048cf500adad7bdbd1d23	0	0	0	0	0	0	0	20	0	0	0	0
2c9880e55bcfe0c0f7708d3563acc7fc	4	0	0	0	0	0	0	0	0	0	0	0
ed4d979232dd878e228548716f610d42	0	0	0	0	0	0	0	23	0	0	0	0

1f4ac86774bf5e491e09bf4125f5512f	0	0	0	0	0	0	0	0	0	59	0	0
a71b76069cae6cec55d2f89339b27e9e	0	0	0	0	0	0	0	0	0	0	0	24
57fc2f54697499c6b9691524add5e413	0	0	0	0	0	0	0	0	0	0	0	2
dc7c73c0ea524c98bc1508aafc5ca0bc	0	0	0	0	0	0	0	0	25	0	0	0
0405b03ae1da36f78996f26665841368	0	0	0	0	0	0	0	0	0	0	0	3
b439b66612d7f60f4970a8c82877be4f	0	0	0	0	0	0	0	0	30	0	0	0
264b407ae3394cdf4d4e214b224c4f09	0	0	0	0	0	0	10	0	0	0	0	0
05fad5b7976d895cfa3ea127c7d4f84d	0	0	0	0	0	0	0	0	0	0	0	2
4f47248d00fe787add5f9014fd435f3c	0	0	0	0	0	0	0	8	0	0	0	0
4f0728f978a98ae946916e0af3ef5396	0	0	0	0	0	3	0	0	0	0	0	0
9b2353a6834ae3276983918830bca281	0	2	0	0	0	0	0	0	0	0	0	0
93d0dcaab7d9219916bcf241e1abdfd8	0	0	0	0	2	0	0	0	0	0	0	0
785e42168b023a2d19e5d0f29b0662ba	0	0	0	0	0	0	0	2	0	0	0	0
1f739746678a1a2b9c9935ac4d69ff72	0	0	8	0	0	0	0	0	0	0	0	0

90f3f19f54585e57eace85700 8ab1eff	0	0	0	0	0	8	0	0	0	0	0	0
faa87c6179b40cfb93a35421 38a23d7e	0	0	0	0	0	0	2	0	0	0	0	0
a4051c76baa6504d9f86182a 48ebd376	0	0	0	0	0	0	0	0	0	0	5	0
b481f72090c51270653ceb2a c4f2e1e8	0	0	0	0	0	0	0	0	0	2	0	0
95723bfdca5d553697c8e845 afaf640b	0	0	0	0	0	0	0	0	0	3	0	0
7eee6cb65483a3c6ebcc4f3b edc3246e	0	0	0	0	0	0	0	2	0	0	0	0
a72985c516ea04eca73e0952 774ab64b	0	0	0	0	0	0	0	0	0	0	10	0
b0585b453f10d268da1753d e88839500	0	0	4	0	0	0	0	0	0	0	0	0
17decf17c3a37eb7d9639a00 9f0ebe54	0	0	0	0	0	0	0	0	0	0	0	17
6676db03f4e93c0b6152954a f3328ffe	0	0	0	0	3	0	0	0	0	0	0	0
73ca884479a5a62076679856 971e698f	0	2	0	0	0	0	0	0	0	0	0	0
e58d99fc6b0eeba330d1bdd0 5e2b3314	0	0	0	0	0	0	0	20	0	0	0	0
43c17b1bc8eb120d6a8542d 6def304d0	0	0	0	0	166	0	0	0	0	0	0	0
1a5ae9bd3ea3c1b7e0e9b2d 1475e5b8c	0	0	0	0	0	0	0	0	0	0	0	4

b1d083bd96db50c7f4a6b0f327d97d9d	0	0	0	0	0	6	0	0	0	0	0	0
ac7f92bca960f1bc79595cd42e73e93b	0	0	0	0	0	0	0	0	0	12	0	0
076fe268e66f310f307e72f1c466b4d5	0	0	0	0	0	0	0	5	0	0	0	0
2161483c81b99ebb435811325d9af9cd	0	0	0	0	0	20	0	0	0	0	0	0
174477d1745b8437a2aea4d03d32f8a9	0	0	0	0	0	0	0	0	0	2	0	0
6e093c50482ee03dd6999bb564fc71cc	0	0	0	0	2	0	0	0	0	0	0	0
653fa0cc5c269c062b7bc688228b7663	0	0	0	0	33	0	0	0	0	0	0	0
c737841e21273f1ef277e66cd45a6171	0	0	0	14	0	0	0	0	0	0	0	0
cc0baceeb43e59a4692382e73e15e26a	0	0	0	3	0	0	0	0	0	0	0	0
cf90dac1a5fd0a42f3c374cd8a9e9ebf	0	0	0	0	14	0	0	0	0	0	0	0
82500450e7ce74df00446b5fe6e4698e	0	0	0	0	0	0	0	2	0	0	0	0
f0186270a3051613a9ecf313e7d45179	0	0	0	0	0	2	0	0	0	0	0	0
eae25fa5dd0787199d36fc0911539ea7	0	0	0	0	0	0	0	0	0	0	0	6
abec643565ccb55248beea86fa27ea42	0	0	0	0	0	0	0	0	2	0	0	0

f028704f91b3455607b57400 e698ad57	0	0	0	0	0	2	0	0	0	0	0	0
9c88d3448f7ca884149c043a ecdaae58	0	0	0	0	0	2	0	0	0	0	0	0
75d191c93a81c09d066b960 8ab1a3d0b	0	0	0	0	17	0	0	0	0	0	0	0
c20fad4de3db83cbf5f29d144 b9e3b0a	0	0	0	0	0	0	0	0	0	0	0	13
4dabc85415dde9f2644bf288 051df25b	0	0	0	11	0	0	0	0	0	0	0	0
467f09c08c00c4e694bc7ed0 9b997fdd	0	0	0	0	0	14	0	0	0	0	0	0
2a481ada20e93d29aa10ebcf a7520b35	0	0	0	0	0	4	0	0	0	0	0	0
3566de740d4d078276a1a98 efc370843	0	0	0	0	0	16	0	0	0	0	0	0
c02c5d689cfb437145fa1eb3 d44db049	0	0	0	0	0	0	37	0	0	0	0	0
d1ec6b7b466b3f7ae263e65b df9b8997	0	0	0	2	0	0	0	0	0	0	0	0
da7f13e79a408d23b0c9c5ac 88570d93	0	0	0	0	0	0	0	0	0	0	2	0
c6bf7cc6c913c4ee76bb4215 4e22f7ca	0	0	0	0	0	0	0	0	0	0	2	0
bc1d0620c65bbce2286e2bc1 a41686db	0	0	0	0	0	0	0	0	0	0	8	0
8a128a9d73a3c822436a6517 d01f9cef	0	0	0	0	0	0	0	0	0	5	0	0

70947330d25058445aea10f2f9cd2d08	0	0	0	0	0	0	0	0	0	0	8	0
456f4de12a8c67c8308777703fbd2460	0	0	0	0	0	0	29	0	0	0	0	0
ec0dd9fcb575ae69e1465c13d8d5f366	0	0	0	0	0	0	0	0	0	0	2	0
2d1551015f9a0b12ac010e6ee2e9ecc7	0	0	0	18	0	0	0	0	0	0	0	0
3b470e16369d8a88445aeb411eee439b	0	0	0	0	0	0	0	0	0	0	0	3
6a0f3efe89afa6079b8b2469c88b5c32	0	0	0	0	0	0	3	0	0	0	0	0
da39824ccbdef029812c4586f5213704	0	0	0	0	0	0	0	0	0	0	0	27
044efd9244781a139d78668ec33b8f78	0	0	0	0	0	0	0	3	0	0	0	0
1af1a8e342bab6962251d21b67d28607	0	0	0	0	0	0	0	0	0	2	0	0
084f91624c1da1281b8e20c96008cc16	0	0	0	0	0	0	0	0	4	0	0	0
9f201fc2de691765fd57e8581734b7d0	0	0	0	0	0	0	0	0	4	0	0	0
a3fe5ac1dc255fdfc3bd5ae229b4da8b	0	0	0	0	0	5	0	0	0	0	0	0
ad6856feec238ddd87dd5d9ada01e083	0	0	2	0	0	0	0	0	0	0	0	0
72f56888e62b76066af37603c2eba680	0	0	0	0	0	0	0	0	0	0	17	0

a88b2e1773aff7bd0627eb47133f8701	0	0	0	0	0	0	0	5	0	0	0	0
5a050e987e9d1971ecd5104605808653	0	0	0	0	0	0	0	0	0	0	5	0
370c79e0184bbd24c3e0d9f467af0ebc	70	0	0	0	0	0	0	124	0	0	0	0
e8c9c4326ab59e041931c8db5cfeb354	0	0	0	0	0	0	0	0	0	0	0	4
bae1e014ac43dcaa575931dc942ae24c	0	0	0	3	0	0	0	0	0	0	0	0
cdc4389cd90ccb6a67765206fcaa9ec4	10	0	0	0	0	0	0	0	0	0	0	0
c4d59059199732d149d48883a73b8a6a	0	0	0	0	0	0	0	42	0	0	0	0
abd83d1cd7bd5b7794eaf5176c3c500d	0	0	0	4	0	0	0	0	0	0	0	0
ad2db21a17333007c7f5b1223ab29e3d	0	0	0	0	0	3	0	0	0	0	0	0
ef82f8d829a57785d1b713911077c984	0	0	0	0	0	6	0	0	0	0	0	0
9f5042c1b58a8fdb2a1bced02abe833b	0	0	5	0	0	0	0	0	0	0	0	0
cb1a1d606af8cb468d65bb58b15b578c	0	0	0	0	52	0	0	0	0	0	0	0
f17f88ee8cf6d954e40aece49b8a7a5a	0	0	0	0	0	166	0	0	0	0	148	36
0ae9a68489929ea4556e55eea8017c59	0	0	0	0	0	21	0	0	0	0	0	0

cc98dab911e982a840f12192e405139f	0	0	0	0	0	0	0	0	0	0	0	19
c3b12b1951a353b88527842b282ea9ac	0	0	0	0	0	0	0	0	0	15	0	0
2e476d77918ae8d0856b60caa65c7cf3	0	0	0	0	5	0	0	0	0	0	0	0
418795b76d5cbb15dfc15eb8fe4ece62	0	0	0	0	0	0	0	0	0	0	0	2
c8121ad5903d732cc5c09b219cb232d8	0	0	0	0	0	0	0	0	2	0	0	0
8a299705e451555f3ffa6f155e1cc8e5	0	0	0	0	0	4	0	0	0	0	0	0
248382e4c4b99f7fad880ce0a86d268f	0	0	0	0	0	83	0	0	0	0	0	0
6ccd1035e7b968b84aa319c356548ca1	0	85	0	0	0	0	0	0	0	0	0	0
0be9c46810e1432e83f3348a29e3f9c8	0	0	0	0	0	0	0	0	0	0	0	8
49880359337786426b0f8a4c5b36e6ae	0	0	0	0	0	0	0	0	4	0	0	0
1ea76ca62b731ed2017b58bf0e88817d	0	0	0	0	0	0	8	0	0	0	0	0
c48358abdf8abe5635a73c08effb88ab	0	0	46	0	0	0	0	0	0	0	0	0
1b1384c8c38bb53160c1706ce18cf405	0	0	66	0	0	0	0	0	0	0	0	0
590baadd2d8d9b3fae2a480292064eef	0	0	0	0	0	0	0	0	0	0	2	0

57adb4ca09d4cc1209321502a25c0d2b	0	0	0	0	0	0	0	0	0	26	0	0
603e77dd4909a853f167ef2fb4d270e6	0	0	0	0	0	0	0	13	0	0	0	0
6873700a0e957c6426663f1744a80671	2	0	0	0	0	0	0	0	0	0	0	0
e08f2ff8603b98033b6cef9c6367d0ec	0	0	0	0	0	0	0	0	0	25	0	0
19181a03ad4a814a74f413a99bf1d290	0	0	0	0	0	0	2	0	0	0	0	0
1ba7b9c5b19dad68b467b241dcbbd389	0	0	0	3	0	0	0	0	0	0	0	0
2bc9d8f0d5ec3c2810649f6c1d7b2a45	0	0	0	0	0	0	0	0	0	0	2	0
787a78af869e4dfb6676a317860ecda5	0	0	0	0	0	0	4	0	0	0	0	0
dcac496cc2e428fa6fe4ee517d9972f4	0	0	0	0	0	0	0	0	0	2	0	0
9b6fafc6860ef55526d8acfd5f289ea8	0	0	0	0	0	9	0	0	0	0	0	0
1d799ba91d57274bad5b9dde070d1bb0	0	0	0	0	0	9	0	0	0	0	0	0
13fa7dfc634898d033dfb9e14fb5dcc9	0	0	0	0	0	0	0	0	0	0	2	0
033dfae20b9d81a391fa6704e0a47491	0	0	0	0	0	0	0	0	0	3	0	0
0dc5ee83af7a88d816cc39bc9ea58a0f	0	0	0	0	0	0	0	0	0	0	0	16

67cd07a6bee6a9a88972313 9cbe2ef20	0	0	0	0	2	0	0	0	0	0	0	0
3b7c83057ac0692ba2ecdfc6 2e118d41	0	0	0	0	0	5	0	0	0	0	0	0
421e4f4f1f5549927953559a e10c6216	0	0	0	0	0	0	0	0	0	0	3	0
cf3087656cb849b20e677817 84159b9f	0	0	0	0	0	0	0	0	0	0	4	0
164237c53bfc8500d02b766a 2b06999a	0	0	0	4	0	0	0	0	0	0	0	0
870d65f72b507f09f4053098 96429d3b	0	0	0	3	0	0	0	0	0	0	0	0
0722a7b7fac501d6b79d3802 16466a27	0	0	0	0	0	0	0	0	0	0	29	8
69169a1605fe65f1f1fdd3d62 336c507	0	0	0	0	0	0	0	71	0	0	0	0
de15756acf80d24fbcd32636 a06474ee	0	0	0	0	6	0	0	0	0	0	0	0
7de5503d03ac5a2f0638b20b 4c14a3f5	0	0	0	0	0	0	0	0	0	0	2	0
8da2768ca4d86944c1d5fd43 0dc69b43	0	0	0	0	0	0	2	0	0	0	0	0
a2b0b2d3553639da1846126 b46494a28	0	0	0	0	0	0	0	0	0	0	2	0
3174cc1eec02623ffee9e31d7 8c1d112	0	0	0	0	0	0	0	0	0	25	0	0
a8b0b8073ec10b4365d3496f 6ae10aad	0	0	0	0	0	0	3	0	0	0	0	0

b6f725985570e5705c8f6711de593b10	0	0	0	0	0	0	2	0	0	0	0	0
8f3db738f9d34d32fda671e7f899ab52	0	0	0	0	0	0	0	0	0	0	0	6
4b49863abc553e42caf5a157bee02ea7	0	0	0	0	0	0	0	4	0	0	0	0
f3f5f53376d4e07791e7e988333ab5cd	0	0	0	0	0	0	0	0	0	0	0	3
2b9a4a1cda22a77c66a5a8ecd14f93fe	0	0	4	0	0	0	0	0	0	0	0	0
37ba5fe750a7e1e0c3f89e2fb2051cba	0	0	0	0	0	0	0	0	0	0	6	0
90805289c711aa8cbc03fe9ce796cc11	0	0	0	0	0	0	0	3	0	0	0	0
0bc09e21ab7fb461292a3aa34164fb23	0	0	3	0	0	0	0	0	0	0	0	0
7ca8204480115b36a2ec019f10c7b7d0	0	0	0	0	0	0	0	0	0	2	0	0
6c6ddf2e00b82101b4ce7cafd7036dac	0	0	0	0	0	0	0	7	0	0	0	0
fa596f7a10796a7994489bd07a88b201	0	0	0	0	0	8	0	0	0	0	0	0
9a31189970e75e8170efdae72c772c43	0	0	4	0	0	0	0	0	0	0	0	0
6e5bf1b8f2deb23fcfb81e0cfe00327d	0	0	0	0	0	0	0	0	0	0	5	0
ee4356d5ae974bc1c7362cb5d0d0f1c8	0	0	0	94	0	0	0	0	0	0	0	0

dd171a054008a45efc00efd56d266681	0	0	0	0	0	0	0	9	0	0	0	0
1d3600b799a91fae2b518c2d901eac38	0	0	0	0	0	0	0	0	0	0	3	0
56cfbe32506fb65ce8590049fd0b3e72	0	0	0	0	0	0	0	0	0	18	0	0
eaf71ce9118a5333a5b26f7151fadf52	0	0	0	0	0	0	0	0	0	0	18	0
10607741bb43bca01214886e8e4f4bfa	3	0	0	0	0	0	0	0	0	0	0	0
780cd7d7cd4b673ba7c398b03f66becf	0	0	34	0	0	0	0	0	0	0	0	0
a397113e9907a63dc1e9aca226393449	0	0	0	0	0	4	0	0	0	0	0	0
2f63488b11a71ad921e63b0c25353244	0	0	0	0	0	0	0	0	0	0	7	0
8e03a6645e6b993cfb69daefeff3a5d0	0	0	0	0	0	3	0	0	0	0	0	0
17dac0adf46097a918292cf74c515931	5	0	0	0	0	0	0	0	0	0	0	0
7329242a5ab7ba61d0bccce8301e3601	0	0	0	0	0	0	0	0	0	0	0	2
f25d774fe3478aafe47ede337b7ffdae	0	0	0	0	0	0	0	0	0	3	0	0
8e7018c2f4281ba61f8f042de132967f	0	0	3	0	0	0	0	0	0	0	0	0
8a6ad9ff6e3e361de11cf58bbd7d5c52	0	0	0	0	0	5	0	0	0	0	0	0

06f2419edc9b5d0ff561fce01 47018e5	0	0	0	0	0	0	0	0	0	0	3	0
ae0a06d2c8c619927ff36760 86aa74c0	0	0	0	0	0	0	0	0	0	0	0	7
87fe09bfc0fc2bd3816885695 97a1caa	0	0	0	0	0	17	0	0	0	0	0	0
dee05f6ad738359c17dec81a 828474c7	0	0	0	0	0	0	0	0	2	0	0	0
c75b22675c689e2675fcc22ef d416571	0	0	0	0	0	0	10	0	0	0	0	0
c6f0ec19d073f5fcb218f4b12f f74a28	0	0	0	0	0	0	0	0	2	0	0	0
b5956bd5fb132d1f33248beb 69ba73ca	0	0	0	0	0	0	0	0	0	0	0	5
648e831236f94195fb28acfda 3c3abd4	0	0	0	0	0	173	0	0	0	0	332	0
57b239bc5d6a23a020610f3e 9d554790	0	0	24	0	0	0	0	0	0	0	0	0
d04ee6fcc95cb9a9898ff2863 396179b	0	0	0	0	0	2	0	0	0	0	0	0
221f09822828370bbe954bea 02f93ca3	0	0	0	0	0	0	0	0	4	0	0	0
dce1ca5096b2186d7af5402c 7710a09f	0	0	3	0	0	0	0	0	0	0	0	0
da8d861a0981f05ce711bd18 35d656bf	12	0	0	0	0	0	0	0	0	0	0	0
860677de651f2c1bb9ee0d2b 473263f3	0	0	0	0	0	0	0	0	0	0	21	0

431dd60d9d760e16011e8bd b133ac0e0	0	0	0	0	0	42	0	0	0	0	0	0
aeed0bbf80c41c1eefb28f77e 151ab71	0	0	0	0	0	0	0	0	0	0	6	0
e026225c1d5410adda20485 034309aac	0	0	0	0	0	0	0	0	46	0	0	0
180a01b83c28819a9374080 3109aa90e	0	0	0	0	0	0	3	0	0	0	0	0
834bf4a77555b3adad687392 a54ac3da	0	0	0	2	0	0	0	0	0	0	0	0
b95f0d34e0e08212c93ff8afd e8e0173	0	0	0	0	0	0	3	0	0	0	0	0
3ceba3134731e9374b3303ff 843fafee	0	0	0	0	0	3	0	0	0	0	0	0
1741606ac5fbd45664019bbb 079a4b93	0	0	0	0	0	0	0	0	0	3	0	0
66131cfe21d4699f1edc4e47 4b8ead59	0	0	0	0	0	0	0	0	6	0	0	0
dbdde077a599f01f74c7bef0 251399a2	0	0	0	0	0	0	0	0	0	0	7	0
76ba708df890302518bcea32 2feb8992	0	0	0	0	0	4	0	0	0	0	0	0
32ccbf979ca66587d7108de1 130ce6c7	0	0	0	0	0	0	0	0	0	0	8	0
5d3cd504883ffe54a9c3ae18 50b41ca7	0	0	0	10	0	0	0	0	0	0	0	0
9f70c192646f531bc63ec8ced 265dd34	0	0	0	0	0	0	0	0	8	0	0	0

0b0964bd51a87ae2649007b16087cf97	0	0	0	0	0	0	33	0	0	0	0	0
7bfa61abb982d19c9f96b39fb7f3bd21	0	0	0	0	0	0	0	0	0	0	2	0
2690b7ba96f88a512c28c953eea18483	0	0	0	0	0	0	0	0	0	0	0	57
0e42c89e9af22604a13a66cc5676a918	0	0	0	0	0	0	0	13	0	31	0	0
38c8cfbabd82a8ac036a03389dd1d511	0	0	0	0	0	0	2	0	0	0	0	0
e0c6de02c488edec4ede236fcddac916	0	0	0	0	0	0	0	0	0	0	5	0
afbc32763fcc6d65356e3386de3be10e	0	0	0	0	0	0	0	0	0	0	2	0
7f0e1e84c6af2db03bb7ba69356e331d	79	0	0	0	0	0	0	0	0	0	0	0
5e6a151b6dbc8fa51e87a290ef2acf37	0	0	0	20	0	0	0	0	0	0	0	0
3a630194dc9daeaa45b9e06559c7ed18	0	0	0	0	0	0	0	0	0	0	0	2
a6bbd93b0346b159c8575517d9196bbe	0	0	0	4	0	0	0	0	0	0	0	0
5994bd8c1d8708ca767489d049352395	0	0	0	0	0	0	0	0	0	0	3	0
1a0329402c828a02ac2253479d017859	0	7	0	0	0	0	0	0	0	0	0	0
eda1f895db2081c2d9e2d657c680dad4	2	0	0	0	0	0	0	0	0	0	0	0

6d0ba1eb4c1fdb7d39e26d3b47b2bd94	0	0	0	0	0	0	0	0	2	0	0	0
88bbdc5a7bc967642b8e727a1b93237c	0	242	0	0	0	0	0	0	0	0	0	0
166450a2f8f22dc1c78a372df6ff05f4	0	0	0	6	0	0	0	0	0	0	0	0
7282617371b079731b024514fb7108d8	0	3	0	0	0	0	0	0	0	0	0	0
4c73dc7648353829ed6a1de0fd17ae5a	0	0	0	0	0	0	0	0	63	0	224	0
b869ddc2068f963f725538df0089f88a	0	0	0	0	0	0	0	0	0	0	0	4
2d3562c4632fda5d45177b61cf12defb	0	0	4	0	0	0	0	0	0	0	0	0
01219182939fcf2e7d8feb5ab1f91363	0	0	0	0	4	0	0	0	0	0	0	0
b1bbbab4bc80a8a1eab12e505fd0d83f	0	0	0	0	0	0	0	0	0	0	8	0
21d4368ef4801062fde4279286f68009	0	0	0	0	0	0	0	27	0	0	0	0
6efb400b313cf0debd8aa7f9d0ab7df6	0	0	29	0	0	0	0	0	0	0	0	0
56c18816582bc4c946687faf21577adf	0	0	0	0	0	0	0	4	0	0	0	0
d66fa79c942f1e7a15fa0c1d79f6fb44	4	0	0	0	0	0	0	0	0	0	0	0
fd39e4eca301bb001167a9f3311ba6bf	0	0	0	0	0	0	0	0	21	0	0	0

d256d22b13c1b66fb76527f3a3e242c9	0	0	0	7	0	0	0	0	0	0	0	0
874b0291a7ee09591c5a6637ae756b30	0	0	0	0	0	0	0	0	3	0	0	0
1c072affbec49fbb943e4de3701b0223	0	0	0	0	0	0	0	0	0	3	0	0
e2a6e4e355509e00e8e42228b17a8667	0	0	0	0	0	0	4	0	0	0	0	0
80bdaddd9dbee0e9248527fed0f199b9	0	0	0	0	0	0	0	0	0	0	2	0
fb82c13ebe7ed80cd23bb507cd0d7458	0	0	0	0	0	0	0	0	0	0	0	9
e9087bd440e9b87d4a99e11df023b67b	0	0	0	0	0	0	0	0	0	0	0	59
152c1ceb736e5121181abcd3ae1d5892	0	0	0	0	0	5	0	0	0	0	0	0
e10d08af76f806a1f2158c4940d11a6e	0	2	0	0	0	0	0	0	0	0	0	0
c46d48924dbe18d820bac96c98769f96	0	0	0	0	0	0	0	0	3	0	0	0
ecd959648c850e24ee8043a6eea03a6c	0	0	0	0	31	0	0	0	0	0	0	0
cd449cbb4978d99ceb068862319266cc	0	0	0	0	0	0	0	0	0	0	3	0
de13bf8bededba6054129efaf6a044b7	0	0	0	0	0	0	0	0	0	0	26	0
a7f3a35eb28ccfbdcf2fdcf1cee46880	0	0	0	6	0	0	0	0	0	0	0	0

ba17a237a8e52cbf9c4e940c31364f5c	0	0	0	0	0	0	0	0	5	0	0	0
0bda3157e6a3c55b4510226d075a27eb	0	0	0	3	0	0	0	0	0	0	0	0
0a1616806c4a43b9e0f3fe33d88d3c30	0	0	0	0	0	5	0	0	0	0	0	0
13472050bcdceb7d236e3e644714875d	0	0	59	0	0	0	0	0	0	0	0	0
7d3d0c872cc516800ddd5b103444ab14	3	0	0	0	0	0	0	0	0	0	0	0
dbb906ed5427a956e95a003ead45729b	0	39	0	0	0	0	0	0	0	0	0	0
baee423a0940257bdac0e25d0c4261a0	0	4	0	0	0	0	0	0	0	0	0	0
1bdd6aae154d5c5dbeb1bfa56c7e554b	0	0	0	0	0	0	0	4	0	0	0	0
6821cebca51a4b2aedf98eaaf8a61f01	0	0	0	0	0	6	0	0	0	0	0	0
64eb1a91f1dafda96edb538be8eab57a	0	0	0	0	0	0	17	0	0	0	0	0
e162eb788ed71cd33f1d5d3376761d0c	0	0	0	0	0	0	0	0	0	0	0	2
578ce71b5c738b61e71004443f8244eb	24	0	0	0	0	0	0	0	0	0	0	0
bc57d478858570b2c3d7422bb9a88e16	0	0	0	0	0	0	0	0	0	15	0	0
45a2022b772e64eadf707e555ca12c09	0	0	0	0	0	2	0	0	0	0	0	0

a83c88ac1cbe7b92a83aef086b559618	0	0	0	0	0	0	0	0	0	0	0	41
a6f8bb73e6aa70368ec2857bfdbee676	0	0	0	0	18	0	0	0	0	0	0	0
4c12f72c57bfa384b2f731913f89ffc7	0	0	0	0	0	0	0	0	0	0	21	0
9a04911f6edc89bd4b3c568f2a016ada	0	0	0	0	0	0	0	0	3	0	0	0
fada41caebc0588a26f4c7b1fb5f7639	0	0	0	0	2	0	0	0	0	0	0	0
b7b138a71873667a955feccc080910dc	0	0	0	0	0	0	0	0	0	0	169	0
62a8e16dec9c92f689319873e9e5256f	0	0	0	0	0	2	0	0	0	0	0	0
e450f810076e657e95644b53f2923a5e	0	0	0	23	0	0	0	0	0	0	0	0
910d9c692eb9374a72f4d7e27ea09d8f	0	0	0	0	0	0	0	0	26	0	0	0
17ba9b8adf97fb27d82dfeeadfa84d1c	0	0	0	8	0	0	0	0	0	0	0	0
96b82fe6a7de7cef7dffe0ddd50254f4	0	5	0	0	0	0	0	0	0	0	0	0
fa872b7dec75ee3634777c9b88acc9fa	0	0	0	0	0	4	0	0	0	0	0	0
7bc48abbf7389dbb41e32f90b10c4ba7	0	9	0	0	0	0	0	0	0	0	0	0
7dcbb8c37245aef664b8a020528a4886	0	0	3	0	0	0	0	0	0	0	0	0

fd2fe83dc18c96a5523d18cea d5bef5e	0	0	0	0	0	0	0	0	0	0	33	0
70b5265417642103a940ca7 b131e98e3	0	0	0	0	0	0	0	0	0	0	0	10
5054784b68dabc1c6331a14 00d0b97d1	0	0	5	0	0	0	0	0	0	0	0	0
c912c07ea14b5d325ea9c323 1f31b7b9	0	0	84	0	0	0	0	0	0	0	0	0
42a44ffebf1a7e61a6e17a31b 4afe1ab	0	0	0	16	0	0	0	0	0	0	0	0
69dc45886713fb6192927fc8 a9aeec04	0	0	0	0	0	0	0	0	0	0	0	16
66563a280bab09c8dc5731dc e3b01710	0	0	0	0	0	0	0	0	0	17	0	0
7dfde6f2cc58697bac0f855e3 d825824	0	0	0	0	0	0	8	0	0	0	0	0
4067662ce13592a5b3bd0cef 7b007ceb	0	0	26	0	0	0	0	0	0	0	0	0
359ff4080e6a2d869f4e97ad d49cd302	0	0	0	0	122	0	0	0	0	0	0	0
d5692b986218545c47924ce b5c592cf9	0	0	0	0	0	0	0	0	4	0	0	0
c31b14091b3fb886f8ce2715 268c4190	0	0	0	0	0	0	0	0	0	0	13	0
044037ea22cc0a71bce40c36 19dce4a6	0	0	0	0	0	0	0	0	0	0	0	3
3417b1c7b4940ecc4e513e5a fa984fea	0	0	0	0	11	0	0	0	0	0	0	0

3b01d4cc031c03736b991b42d8d5ff08	0	0	0	0	0	0	70	0	0	0	0	0
cee79e66f0637abd245c929401950c3b	0	90	0	0	0	0	0	0	0	105	0	0
45340d51ff2de130c2e0cdc929eacd4b	0	0	0	0	0	0	0	0	0	0	0	157
56b59558712df9baeb510c2b11195f63	0	0	6	0	0	0	0	0	0	0	0	0
147bd2de903b72e39bfaad290a900cfc	0	0	0	0	0	0	0	0	0	0	12	0
6ecfa2c9d2d3000f30324e0b00b1ed3d	0	0	10	0	0	0	0	0	0	0	0	0
792a1fdf77c8f1768a8cdc6f4bb467f8	0	0	0	0	0	0	0	0	10	0	0	0
3e382d80d9f5301592cb5043383c43c4	0	0	0	0	0	0	0	0	0	0	0	7
8519c00e66afa3fdb3ec14b6777f17c7	0	0	0	0	0	0	0	0	0	21	0	0
253404fb42563e664c3f69a1d1c2e517	0	0	0	0	0	0	0	0	0	17	0	0
ace3f84340716ff36b95e86409f28e74	0	0	0	0	0	0	0	0	0	0	47	0
dcc3969896ab599dfc0a264bc1af1135	0	0	0	296	0	160	0	0	0	0	0	0
b8a18d11969e8f77eb8b163b5fed9755	0	0	0	0	0	0	0	0	0	3	0	0
62a4cc620cea7babd5f1b1e42d6c8a2f	0	0	0	7	0	0	0	0	0	0	0	0

44b7831a1f1096f9c5666c7815d1864c	0	0	0	0	0	0	12	0	0	0	0	0
060fe5fdfb729d52c9074db449f14630	0	0	0	0	0	0	2	0	0	0	0	0
a5b77a94bc32ac43effd51c1c2331621	0	0	0	0	0	3	0	0	0	0	0	0
c1284d65e9d66be3c69460ab812a7889	0	0	0	2	0	0	0	0	0	0	0	0
bf11ea0050ed26814f64f874955712d1	0	0	0	0	0	2	0	0	0	0	0	0
b8c2290c1246350b0111f322ada3926a	0	0	3	0	0	0	0	0	0	0	0	0
31a1a03a34d7e7f9a95934386d390d30	0	18	0	0	0	0	0	0	0	0	0	0
e9d59ab8a50e8214b642298f255ed846	0	0	0	0	0	0	17	0	0	0	0	0
2d0fb4ff9e5ad83fb081b502fa8931db	0	0	0	0	0	0	0	0	0	2	0	0
64c00dc146b2f5f0144d1140f7db7539	0	0	0	0	0	0	0	0	0	0	0	6
876496328964bc72a8754efec1a2a355	0	0	0	7	0	0	0	0	0	0	0	0
cd2b301f0ecea7533898c4cbeaee618c	0	0	0	0	0	0	0	0	0	0	0	9
5746a067ebbd48825932f4f76d0b6b87	0	0	0	0	0	0	0	0	0	0	3	0
e3d14dbdc96ff20cd56ab03535b55d46	0	3	0	0	0	0	0	0	0	0	0	0

6f21e24dc06baa090fd90907f482ec08	0	0	0	0	0	0	0	8	0	0	0	0
01fd607e4be0d790b6d8025835e322c6	0	0	0	0	0	4	0	0	0	0	0	0
38fee607d66ad006939990c7c0bbd9ec	0	0	0	0	0	0	0	0	0	0	4	0
a08eaaa9fe039356cd2230044b27e15a	0	0	0	0	0	0	0	0	0	0	10	0
336541c8dea235f4e91380cc57cabf9f	0	7	0	0	0	0	0	0	0	0	0	0
a9f8526b1a5ad2f15c5fa7ad3322099a	0	0	16	0	0	0	0	0	0	0	0	0
36c9ec9fa12a68354de5564e9e9090e2	0	0	0	0	0	0	0	0	0	0	0	3
f58b8c7ebae0eda898cbe9a5db516b0b	0	0	0	0	0	0	0	0	0	0	0	7
7638c3a1343bc712d7ac68ef8f7d8fe4	4	0	0	0	0	0	0	0	0	0	0	0
187fdb1eb3645de3fc171efef9718ab5	0	0	0	0	4	0	0	0	0	0	0	0
c54eea81abf23def49060a4bf5b2059c	0	0	0	0	0	0	0	0	0	3	0	0
64814af30de63f9e57028eddd8b9be9c	0	0	7	0	0	0	0	0	0	0	0	0
91d04c739d21bb1047bff78bdcf6fa7a	0	0	4	0	0	0	0	0	0	0	0	0
70f351e77f5d1fee9395e87aa87125c7	0	0	0	0	0	0	0	0	0	0	5	0

a4d790d2c844bdbc0a624c2bfe0d09b4	0	0	0	0	0	0	0	0	0	3	0	0
fd51f360795928ad97db56989f9e3d07	0	3	0	0	0	0	0	0	0	0	0	0
06b9f39e1d345b4623cca4f5ac4c0a54	0	0	0	0	0	0	0	0	0	0	3	0
8369800f48346d8a801e718863a6e655	0	0	0	0	0	0	0	0	0	6	0	0
112a1b331a8375691f87e338d0ae3d33	0	0	0	0	4	0	0	0	0	0	0	0
baadb1cb2a64a6edcefcb190845508e0	0	0	0	0	0	0	0	0	0	34	0	0
d8234bb0e2bb545f9e749d224f06ab6f	0	0	0	0	0	0	0	0	12	0	0	0
8426a0b7f4de8340e7958fb91c698efe	0	0	0	0	0	0	0	0	0	0	3	0
42c96ef4387c39b038dea05e6ff913c0	0	0	0	0	0	0	0	0	0	0	6	0
3b64c6c560fac2f583e160f151b8ddf8	0	0	0	0	0	0	0	13	0	0	0	0
3035c0a5249c74bd3b2ff821b636d343	0	0	0	0	0	0	0	0	0	0	0	2
f2faa9bc0ff9aa7a7473208ced58ac29	0	0	0	0	0	0	0	0	0	7	0	0
6f5fd09dad79dfe7da9190fe640247b9	0	0	0	0	0	0	0	0	0	36	0	0
f3b4fe21a7646346f5190fde1d6d43e4	0	0	0	0	0	0	0	0	0	0	0	4

5300ac3d18cf1e4757c25241 6b263ec2	0	0	0	0	6	0	0	0	0	0	0	0
a286f834aebfd5634d9eddf8 64f41662	0	0	0	0	0	0	0	0	0	0	4	0
9e435a81606713304e607dc ac52152a3	0	21	0	0	0	0	0	0	0	0	0	24
2e85cb2d6328ef3ffc40fdaca 8a76b4f	0	0	0	0	0	0	0	45	0	0	0	0
b9d07e97f64940c0a035c28a da209397	0	0	16	0	0	0	0	0	0	0	0	0
57823c317ef2c4872765f6cef 385fa1e	0	0	0	0	0	0	0	0	0	0	5	0
89fd6fa26ff0b2aeda1a8508d 549399d	0	0	0	0	0	0	0	12	0	0	0	0
10c80cb68ef31502be132f4f1 808681a	0	0	0	0	0	0	0	0	37	0	0	0
3ee11a4380efd224fe8df0f83 3a58db8	0	0	0	0	0	22	0	0	0	0	0	0
6cb80e4c8e7825348fa71c5a 2fe58742	0	0	0	0	0	0	0	0	0	74	0	0
9b33c62f9d1ebf327c89c897 2b65b8cb	0	0	0	0	0	0	0	0	0	0	4	0
37cd334b96cdc87ed47ef14b 43ce3f9f	0	0	30	0	0	0	0	0	0	0	0	0
53a332455dd7027a2102f516 e0a7072f	0	0	0	0	0	8	0	0	0	0	0	0
e8e31f11973b49e50b399a24 a43c317d	0	0	0	12	0	0	0	0	0	0	0	0

c841ff944b48361c26c14f30b1da9f56	0	0	0	0	0	0	0	0	0	0	7	0
18b3dd928f08ef83c6cec1c4bd81777f	0	0	0	0	0	0	0	0	0	2	0	0
92d492c93725aed1219590c3b8f89877	0	0	0	0	0	0	0	2	0	0	0	0
07b82acbd6ca5a36e401b90e118b4290	0	0	2	0	0	0	0	0	0	0	0	0
cbb4fb5b86f6b481dfb4b4e17696e027	0	0	0	0	0	0	0	0	3	0	0	0
e63e3c25d7735c384af7cb3239d8c4c7	0	2	0	0	0	0	0	0	0	0	0	0
b20ebec0f7ece4d78aee4bf2366479ce	0	0	0	0	0	0	14	0	0	0	0	0
e7c2fa7d52b18c2a122883ffd0207b21	0	0	0	0	0	0	0	0	0	0	0	21
a2b1aaeda9626d8eb7ae8ab5fa3d4bc8	0	0	0	0	0	14	0	0	0	0	0	0
27891fd648e9264c4d7d6fde636b6b25	0	0	0	0	0	0	0	14	0	0	0	0
a899f710550a760b59c189cfc4e06a1f	0	33	0	0	0	0	0	0	0	0	0	0
3a0caa5f3a56e59a5e896a188f967724	0	0	0	0	0	0	0	6	0	0	0	0
f29bcee4af548523573a3dd7c98dab9e	0	0	0	0	0	0	10	0	0	0	0	0
b475103b375289cf2d7c156420285ec1	0	0	0	0	0	0	0	0	0	0	0	6

8860ee82290af34dba0b76c3b02cb282	8	0	0	0	0	0	0	0	0	0	0	0
8b4cc88c578de71e0bfc592b90bc1548	0	0	0	4	0	0	0	0	0	0	0	0
c6d7d07073652ea799adfecbb2898bf5	0	88	0	0	0	185	0	0	215	0	0	0
89845092f293dd9c3f22df95dd2c275a	0	0	0	0	0	0	0	0	0	0	3	0
ae36ced7bb7b47c1b2e652d26e83c58a	0	0	0	0	0	0	0	12	0	0	0	0
076713a1d28434db007c8496c4f29eda	2	0	0	0	0	0	0	0	0	0	0	0
2297b509b5bf8dcd6252089a846fe4f8	0	0	0	0	0	0	0	31	0	0	0	0
269e927546b9d6d65e83c9f0861edec8	2	0	0	0	0	0	0	0	0	0	0	0
b1cd829950477820248704812c73ca4e	0	0	0	2	0	0	0	0	0	0	0	0
cf8bd68fb8a144425285d7e8c4c1b515	0	0	0	0	0	0	0	0	26	0	0	0
27f5e01b6f944e7e04b93093a8eab35d	0	0	0	0	0	0	0	0	0	0	4	0
f4c96a4a1ba5539a9150081b2953c892	0	0	0	0	0	2	0	0	0	0	0	0
89cd47142d49ea1d39f75dcda6370953	0	0	0	0	0	0	0	0	0	0	0	5
e2e77c797e7b86f572edfc19152548ca	0	0	0	0	0	0	0	0	0	0	40	0

b8b9eff2f65280f99958bc1d7fc923a7	17	0	0	0	0	0	0	0	0	0	0	0
fabcebddbd4cbeb566944c6c5e50ac39	0	0	0	0	0	0	0	0	0	0	0	15
7a83f21b948ed8fd06555ac59cde73bb	0	0	0	0	0	0	0	0	0	0	0	19
1db0e0580690357c8884c5223b35e754	0	0	0	145	0	0	0	0	0	0	318	0
9b6c8b66082a3ea9415dce1b309f7817	0	0	2	0	0	0	0	0	0	0	0	0
87991a335b8e6ee6d8a4017af324ea7f	0	0	5	0	0	0	0	0	0	0	0	0
ac0e4473f2c8fe934798e45879f1bbc5	0	0	0	0	0	0	6	0	0	0	0	0
a3b1ad272d0040ba1e5d76802a86801e	0	0	0	0	2	0	0	0	0	0	0	0
de219d98f89a53c21f485aab18737a66	0	0	0	0	0	0	0	0	0	4	0	0
c711d4915ab2a53c12a048ce0b2ac11d	0	0	0	0	0	0	0	0	0	31	0	0
75ea0a5475e0c4742d61bb52a5efa8dd	0	0	0	0	0	0	0	0	0	0	0	2
26371400be4b01594111db588a887395	0	0	4	0	0	0	0	0	0	0	0	0
05feaca37a26b2f38ed035ab8e76e2cd	0	0	0	0	0	0	0	0	0	0	5	0
c7389f08ae9b4f79d578a63002c03e2d	0	0	0	0	0	0	0	0	2	0	0	0

e2b9501ca519c095eb295e5113573ac0	0	0	0	0	0	0	0	0	10	0	0	0
edfbd25090a0c79bd5c3bd6894a53ea5	0	0	0	0	0	0	0	0	2	0	0	0
3f489e9b9278219570f1f5c80902f16e	0	0	0	0	0	0	0	0	0	12	0	0
bbe3ed822efcbc1ef404dcb281ccfb70	0	0	0	0	0	0	0	0	0	0	0	7
1c32d075da5c65e1c62aec0ad887a15c	0	0	0	0	0	0	0	0	0	0	2	0
c3efe33d5d29c1b779bd0e3bb6e87b98	0	0	103	0	0	285	0	0	0	0	0	0
310261c1f56ed487ddafdd201f1a6f73	0	0	0	0	0	0	0	0	7	0	0	0
a717417170d0423912c874a3c60996a5	0	0	0	0	0	0	0	0	0	0	0	3
8da90f5e534e18b8f3553b4c407efea7	0	0	0	0	0	0	3	0	0	0	0	0
347850ff595b7b1d71947953de7bb9a2	0	0	0	0	0	0	4	0	0	0	0	0
053fcfdfe0511ec9e5de5529f7f0dfdc	0	0	0	0	0	0	0	3	0	0	0	0
ef2b531a3b5cc0c1acf86aa803715037	0	0	0	0	0	2	0	0	0	0	0	0
5b047a97e11cb9f313b38220f142bc4c	0	0	8	0	0	0	0	0	0	0	0	0
5ab03367636149a5ee1b66df88f99f27	0	0	0	0	0	0	0	115	0	0	0	103

b6cf5bb5a49f7513de4af2ed61a0e067	0	0	0	0	0	0	0	0	0	29	0	0
981e6f4e5913a4c26fba77a24f54fe89	0	0	0	0	0	0	0	0	0	0	0	4
f03315e6e688c5d6784302f6c7d9d3b4	0	0	0	0	3	0	0	0	0	0	0	0
81d0910553e85d307c8dea9f7af79a68	0	0	0	112	0	0	0	96	0	0	0	0
46ee57d2e0841338173d35adef2c6325	0	0	0	0	0	0	0	0	0	0	2	0
ebcdb7a6b38666de1b81850adcf551cb	0	0	0	0	0	0	0	0	0	0	2	0
cf22ed0a2f1e63fdcc8f43375ebbf4bb	0	0	0	0	0	3	0	0	0	0	0	0
f1ca1ffeb4d24414c21289965847d15e	0	0	0	0	0	0	0	0	3	0	0	0
45b2334d62ca46a8a1609f0e5cf35760	0	0	0	0	0	0	0	0	0	0	0	5
729e95a65a8688bd3c5a78eec22b1b11	0	0	0	0	0	0	0	0	0	0	36	0
367a3ac9c203a186148df5989c6a1024	0	0	0	0	0	0	0	0	0	0	0	2
dbc1501717527167bdbc6b102cf3466f	0	0	0	11	0	0	0	0	0	0	0	0
05cdb50e0033b964ca1918eae68c7ea3	0	0	0	0	0	20	0	0	0	0	0	0
cebae4dd2507629672c2dca1afc07a2d	0	0	0	5	0	0	0	0	0	0	0	0

b792cb3514bf7c7325c3a1ed 5ba9829c	6	0	0	0	0	0	0	0	0	0	0	0
a1615a0e51003d43c0f8f5a9 d381b44d	0	0	0	0	0	0	8	0	0	0	0	0
089f9b9c8231fee2ede9ffd82 b196356	0	0	0	0	0	0	0	0	0	0	6	0
2e8c17b06f409c3abf71d018 c7fccdba	0	0	0	0	0	0	0	0	43	0	0	0
4a5d7167b6205ce24aa3848 420a76067	0	0	0	0	4	0	0	0	0	0	0	0
c764c9da6bc51f3fdba609f53 4c5981d	0	0	0	0	0	3	0	0	0	0	0	0
98dcd5b9782847bc7612c978 a62fa4e7	0	0	0	0	0	0	0	0	0	0	99	0
07f667b3a14d1ae8da5405f3 8eed7c6a	0	15	0	0	0	0	0	0	0	0	0	0
9920569301e520d9ce92e5f5 463f21b1	0	0	0	0	0	0	0	9	0	0	0	0
25eec8201e735531479240d 97cfedd06	0	0	0	0	0	0	0	0	0	0	0	16
f1aff926a91aafc5136742f2ef 13bcb4	5	0	0	0	0	0	0	0	0	0	0	0
1e12e45f5e1139214e05ef07 dcdfd00b	0	0	0	0	0	0	0	0	0	0	4	0
e87f48494d449cd738a2ce78 f21fb888	0	0	0	0	0	0	0	0	0	0	39	0
88903a32a8da0f9de27e3249 72487097	0	0	0	0	0	0	0	0	0	0	0	22

3168d2086b63c2b9e5eabee2ab20ff7b	0	0	0	0	0	0	0	0	0	0	2	0
d627bccc072a62a678dcaf4c0d5b1f9b	0	0	0	0	0	0	0	0	0	0	7	0
c3f4e6bc0a963eb6af68cf69b86ee00f	0	0	9	0	0	0	0	0	0	0	0	0
cf476f797974c7bd83022a44467ab7ee	0	0	0	0	0	0	0	3	0	0	0	0
2358a427569c46e83d5966a0ebdfd540	0	0	0	0	8	0	0	0	0	0	0	0
d8e17988c5020f815d8aa19b09c40794	0	0	0	0	0	0	0	0	24	0	0	0
af83b1067913f51ede01d2a3e42af70b	0	0	0	0	0	53	0	0	0	0	0	0
437382496ea4ef4aebe50426dfb26678	0	0	0	0	0	0	0	0	0	0	3	0
935250c8e1db4e1d068783a66d52e2cd	0	0	0	0	0	0	0	0	3	0	0	0
250b1acc39c799e31d4aa6fb2770d779	0	0	0	0	0	0	4	0	0	0	0	0
d59bc4da7188235de014afa010055269	0	0	0	0	0	139	0	0	0	0	0	0
283297d4352a5dbfd8a2431544093fd0	0	0	44	0	0	0	0	0	0	0	0	0
5b91a82c6ca92f5ea2f7870117bd6ff0	0	0	0	0	0	0	0	0	0	0	10	0
c8b3e8d8cc806a885808d8f682d0a071	0	0	0	0	0	0	7	0	0	0	0	0

5d7c0ded33e044ba0d5795c d3e167e58	0	0	0	0	0	0	0	4	0	0	0	0
6b3be9e412bc90c711037dbc 66643723	0	0	0	0	0	0	0	4	0	0	0	0
3155274e55ef04bc33557a84 f5ecef34	0	0	0	0	0	0	0	2	0	0	0	0
b29a2998d2550e5a16c930d 38229581f	0	0	0	0	0	0	0	0	0	0	0	4
35e047fdfdbf3b1134368ae0f c8dbe63	0	0	0	0	0	0	28	0	0	0	0	0
fe2b27fade8450ed9129bc8c 3f962485	0	0	0	0	0	0	5	0	0	0	0	0
943aacdedbf8826dbfafb4897 c657b1a	0	0	0	0	0	0	0	0	0	42	0	0
a928aa47a003a80c4f4c4c55 7541d481	0	0	0	0	0	0	0	2	0	0	0	0
f4efe48c0826aee4aaba9f9f4 7f1914a	0	0	0	0	28	0	0	0	0	0	77	0
d78a7b793615f8ddda15d08 1d0482a3f	0	0	0	0	0	0	0	0	3	0	0	0
8703cfa263b15fa1f757db73e cd41bf3	0	0	0	0	0	0	0	0	0	0	22	0
d1e9d4181fe3c4ce529cd0c4 9624f024	0	2	0	0	0	0	0	0	0	0	0	0
d42f5f71b43c11618c0330ed 657f2251	0	0	0	9	0	0	0	0	0	0	0	0
b939c92710a50e0b9407ff55 59df3e71	0	0	0	0	0	0	0	0	0	0	2	0

d27b3fdf8f47b72957c13272c0f74e49	0	0	0	25	0	0	0	0	0	0	0	0
11c623b1f8ac3aad6da824cdd9841289	0	0	0	0	0	0	0	3	0	0	0	0
cba6cf2cf06a2a5e18d99b26ee5a3dca	0	0	11	0	0	0	0	0	0	0	0	0
43e878c99aa860430aae88f2d584a5a7	0	0	7	0	0	0	0	0	0	0	0	0
53f582aa316988a740f2418043acac38	0	0	0	29	0	0	0	0	0	0	0	0
efaf2aa8f20af1bb57fe6c3a86d74875	2	0	0	0	0	0	0	0	0	0	0	0
5b935406b72d6c0aa15fef1ced53e460	0	0	0	0	0	3	0	0	0	0	0	0
3d6982b0d162515f3d883aa1bce06d62	0	0	4	0	0	0	0	0	0	0	0	0
0a77e09c4761e560a85c48e5117cdfbe	0	0	0	0	0	0	0	0	0	0	238	0
ab5b93f4c8e6c4be589738b74e760405	0	0	0	0	0	3	0	0	0	0	0	0
22617dd3ea3095c5dd725a2a8f524924	0	0	0	2	0	0	0	0	0	0	0	0
1b7f3f8386e6852eeb23e349ba2b60d6	0	0	0	0	0	0	0	52	0	0	0	0
f2ba45983e2e0350f876c807a7f616cc	0	0	0	0	0	0	0	0	0	5	0	0
6b101824e778e00db98624c40d3279f0	0	0	0	0	2	0	0	0	0	0	0	0

ffbcd4e8387ab07755aead23ae7928e3	0	0	0	0	0	0	0	0	0	0	2	0
f716b8977c2aea6e7b8fe7c965d62f92	0	0	0	0	0	232	0	0	0	0	0	0
73c09daf42c58d5c184a4ca811f0db95	0	0	0	0	0	0	0	0	62	0	0	0
e3c15568b637282c9f37e2c0e7b82b24	0	0	0	0	0	0	0	0	0	0	0	33
7cf4c71ce0021e069cb0a62a907ae462	0	0	0	0	0	0	0	0	0	0	0	5
ffba2e39ed8a1ae8965f8c98ee545926	0	0	0	0	0	0	0	169	0	0	0	0
e31273e9c7500a411e317d33861e6b4c	0	0	0	0	0	0	0	0	0	0	0	2
dd2980345d5731a75ae9445910ef0626	0	0	0	0	0	0	0	0	0	0	16	0
a724a5a49b76c203264e0a2ebdc69343	0	0	0	0	0	0	18	0	0	0	0	0
4bd38a423e6eaeca4548008ccf6cb5ab	0	0	0	0	0	0	0	0	0	0	26	0
bdd86d44d6e20a706db49005ce7435cc	0	0	0	0	0	0	5	0	0	0	0	0
2907dfee5c6d1f943eddb0e7dd229850	4	0	0	0	0	0	0	0	0	0	0	0
b67a277fe345f5b123a18ca7a5152934	0	0	6	0	0	0	0	0	0	0	0	0
c375836de9f9ca76fde8efb40e7a4eac	0	0	0	0	0	0	0	0	3	0	0	0

4a5c327656450eb78f1e7c53b290655a	0	0	0	0	31	0	0	0	0	0	0	0
09ab40667854ccfb99348101c1bcd830	0	0	0	0	0	3	0	0	0	0	0	0
fe8e4c2ae4994df9bb9c769ccbbfda0d	0	0	0	0	5	0	0	0	0	0	0	0
f65ada06c5f4ae48ccaa98453df24b0c	0	0	0	0	0	0	0	0	0	0	38	0
f71b45496b6e26466e3041f7ddc96d0e	0	0	0	0	0	0	0	0	0	2	0	0
2ea69fb276c3369a4ebdc7b459b3aed7	0	0	0	0	0	3	0	0	0	0	0	0
92645a2d045d8d53b03f411e2c8ef90e	0	2	0	0	0	0	0	0	0	0	0	0
3d2a564810e6a2268bbab1f4b09ba8e8	0	0	0	0	0	0	0	0	0	0	6	0
fc0c24b99c2f2327385bd99ababd592b	0	0	0	0	0	0	0	0	0	0	3	0
7da407cb96234d362c2dbbcd73d435d9	0	0	0	0	0	0	0	4	0	0	0	0
455724e496648603997d4223127c5361	0	0	0	0	0	0	0	0	0	0	0	15
aa76fab909b6e188f0312222db062c3c	0	0	0	0	0	0	0	0	0	0	3	0
cad307b429bd86f652e4b0273c1bcffc	0	23	0	0	0	0	0	0	0	0	0	0
465bc9b4fe6fba1605c5f39a0fd2f176	0	0	0	4	0	0	0	0	0	0	0	0

084b01484d66f2fa509a5b65 059cb198	0	0	0	8	0	0	0	0	0	0	0	0
e2c31d5d1ea8bc99173e3fc9 a7ab9bcf	0	0	0	0	5	0	0	0	0	0	0	0
94bf5d162e40c664a8341b51 8fd97457	0	0	0	21	0	0	0	0	0	0	0	0
26a3926b6641b29fec7bd3b2 d37d208c	0	0	0	4	0	0	0	0	0	0	0	0
4855d559cf872b6a7154f0ab 5ee0b10d	0	0	0	0	0	0	0	95	0	0	0	0
bcb8008c68df9de33083e0f4 1c76c229	0	0	0	0	0	5	0	0	0	0	0	0
4ebadf102b9a3202b4484671 01d70312	0	0	0	0	0	0	0	0	0	0	0	11
7f11801ac712fd9465aac57a8 a7710d0	0	0	0	0	0	34	0	0	0	0	0	0
109499e7aa73e50301a9879 9d8a11315	0	0	3	0	0	0	0	0	0	0	0	0
31bbfd82b3edc9bb351eced0 9b5b8e8c	0	0	0	0	0	0	0	0	0	24	0	0
4320be511b4f66ac0205d9a6 b11f242e	0	0	0	0	0	0	0	0	0	0	5	0
ea07f7a80c2f43bd1c9e4eab e22c4914	0	0	0	0	0	0	0	3	0	0	0	0
5b01124cb00f1dc6a7c33163 b4927452	0	48	0	0	0	0	0	0	0	0	0	0
fec3f99944e7bf80dd582e4f7 197d614	6	0	0	0	0	0	0	0	0	0	0	0

63d870e1244a8f23627bea0f4e051ab4	0	0	0	0	0	0	0	0	4	0	0	0
d962c79db60c8f196fc7e41e28edfed4	0	0	0	0	0	5	0	0	0	0	0	0
5fccb937762a10f3fcd1ea042b0c5cde	0	0	6	0	0	0	0	0	0	0	0	0
a20495db760385fdfbecaafe2c46b678	0	0	0	3	0	0	0	0	0	0	0	0
9d97200a14a94eb62371aefe9a5a6be8	0	0	0	0	0	0	0	0	0	0	11	0
561686b092d90669a8482ec311958e41	0	0	0	0	0	0	2	0	0	0	0	0
d57ae8f810e60694982cfbd602ee85e2	0	0	0	0	0	0	0	10	0	0	0	0
e61bb46003b72979fc1b966bf197589f	0	0	0	41	0	0	0	0	0	0	0	0
a3e88bde4e8211cecfb15ffacd17daeb	0	0	0	0	0	3	0	0	0	0	0	0
63dc143bdf2867f10c852c008d7ddbf9	0	0	9	0	0	0	0	0	0	0	0	0
21b1e6949547d9a64a82a86b696a77d6	0	0	0	0	0	0	0	5	0	0	0	0
68d818acff1af49b33c7c0734cbe37ac	0	0	0	0	0	0	3	0	0	0	0	0
d715c8e50518b0584090ed2e158205bd	0	0	0	0	0	0	0	0	0	0	4	0
55514c191d5663f6a171aa67b94f1c61	0	0	0	0	0	0	0	0	0	0	0	59

f7103ce00ba7a233c2c37dc2 9091460e	0	0	0	0	0	0	0	0	0	30	0	0
85f86cbdd2df05624a45197e 231c7a82	0	0	0	0	0	0	0	0	0	0	3	0
e1c5cdff241d2fe202bc10cde e29295c	0	0	0	0	0	0	0	0	0	0	7	0
f924bc6949d0fbc8184fb6e2c e37f20a	0	0	0	0	0	0	0	0	0	12	0	0
b52218c0c17db8cd6ae129f4 9c3a66d8	0	0	0	0	0	0	0	0	0	0	0	53
1664dc3dd06651afacd4bbbd 940c0eab	0	0	0	34	0	0	0	0	0	0	0	0
80bdff2c7751c72de0edec9fd 3634020	27	0	0	0	0	0	0	0	0	0	0	0
a9e15dadaef7989f738ad260 b2c1ec30	5	0	0	0	0	0	0	0	0	0	0	0
616fc9d8e3b41ac1c2cc51ca2 914700f	0	0	0	0	0	0	0	0	0	79	0	0
4134073073644e154c26e72 254dc2a69	0	0	0	0	0	0	0	0	0	0	5	0
d6b61251a12bcdb12b55905 3fc17e8de	0	0	61	0	0	0	0	0	0	0	0	0
daa3476551b1c6b494d38d7 776512859	0	0	0	0	0	0	0	2	0	0	0	0
d8a39d4c0800f9df8c2e0873 cb0e23a6	0	0	0	0	0	0	0	0	4	0	0	0
2c550db5e2bc763eb2f578c1 c328c0c6	0	0	0	0	0	0	0	0	0	0	18	0

d2af74815e8fba633926167ec2953678	0	0	0	0	0	0	0	0	0	0	2	0
669cc531a70973084037cf854d0b3dd3	0	2	0	0	0	0	0	0	0	0	0	0
10604c1fb66dfe2f6ac8d4fecdb96bb4	0	0	0	0	0	0	0	0	4	0	0	0
de1868d3205981819f3c1d20fb88bdfa	0	0	0	0	0	0	0	0	0	0	4	0
acc98576a61b0d6dad0c77b7e114f7f1	0	0	0	0	0	0	0	0	2	0	0	0
cfadc010e5cdec997d106c8b26b1dc45	0	0	0	0	0	0	0	0	0	73	0	0
9d7fb4235fdef162c5494f4a7b7f67c2	0	0	0	0	0	0	0	0	0	0	23	0
cdc6d308b0b7c01252a2e0193fcb36b0	0	0	0	0	40	0	0	0	0	0	0	0
c079d0df63f1e1482cb3647423b47824	0	0	0	0	10	0	0	0	0	0	0	0
2f113b19c3da6adc4017e935947802f9	0	0	0	0	7	0	0	0	0	0	0	0
48fc8a77ef282341b81efe442dff3b5a	0	0	0	0	0	0	0	0	0	0	2	0
db49f0aa6e230c613267989bd4cfe88b	0	0	0	0	0	0	0	0	0	0	16	0
fcae5788bf8c2163ef14660402a78f12	0	0	2	0	0	0	0	0	0	0	0	0
e66b90fc1541349eaf603d43cb1df287	0	0	0	6	0	0	0	0	0	0	0	0

eed8ce3e9804200a9230182b223ea28e	0	0	0	0	0	12	0	0	0	0	0	0
e5d6ddecdd1b1f50158eb21ed174565c	13	0	0	0	0	0	0	0	0	0	0	0
964613b50742f71a60510092415e0b92	0	0	0	0	0	0	0	0	0	5	0	0
51aac0adca96d70eede9dad009da1bf1	0	9	0	0	0	0	0	0	0	0	0	0
bc2320a319d36ec2b5479a421879deb5	0	0	0	0	0	0	0	0	0	0	2	0
666384888d9dc155a6f18f31d96cadf5	6	0	0	0	0	0	0	0	0	0	0	0
03072993162d169c9b40b90c0f37ada7	0	0	0	0	0	0	0	0	0	8	0	0
c512fd95810a23e4ab357deebb244104	2	0	0	0	0	0	0	0	0	0	0	0
8a904fc0adf85ab10a1480a536bbb5ae	2	0	0	0	0	0	0	0	0	0	0	0
80f4927330f727ec2c72ade5c52f8b7e	0	0	12	0	0	0	0	0	0	0	0	0
8accd911e860747b2741a549bbdf6a92	0	0	0	0	0	0	0	0	0	0	3	0
42a3834d79f6739fa2f8eba349860260	0	0	0	0	0	0	0	0	2	0	0	0
8a046734dbf60f47a0aad6fab50e7972	0	0	0	0	0	0	0	0	0	5	0	0
9bda92e2c4dab53ca54860ade8ca1df8	0	0	0	7	0	0	0	0	0	0	0	0

3c5d22e2be16428074c9461cc93c87fe	0	0	0	0	0	0	0	0	0	11	0	0
f7a615d834aa09955a346e9b964b74af	0	0	0	7	0	0	0	0	0	0	0	0
358148bd1bba964913879eec1b201dab	0	0	0	0	0	0	10	0	0	0	0	0
d3e4d684de2783a94e5ca1c07b1bc367	0	0	0	0	0	0	5	0	0	0	0	0
42b0ad4ac9bf879cb390daf6068ede78	0	0	0	0	2	0	0	0	0	0	0	0
bf51de3bfcd0b2dcba6b57a4c1dfd559	0	0	0	0	0	0	0	0	0	2	0	0
1e161eef9764b6a8fc9290fdca4ccdf9	0	0	0	0	0	3	0	0	0	0	0	0
5842a00801a818358c9371a8c346a93c	0	0	0	0	6	0	0	0	0	0	0	0
2b46a9fc5686a535378cece9ee7e0c8b	0	0	0	0	0	0	0	0	0	4	0	0
12c1297626e44ee0412a427ae428fa36	0	0	0	0	0	0	0	0	0	0	0	2
1173265c89b0b9ba4e71e2083ad4eec0	0	0	0	0	0	0	0	0	4	0	0	0
bc427995bc76c0e9bf21d434a6471230	4	0	0	0	0	0	0	0	0	0	0	0
42f93f19807d9a28cf4313e3b9804bc6	0	0	0	0	0	0	0	4	0	0	0	0
23e7707452b34f9c7d3fb85bd0e43d7f	0	0	0	0	0	0	4	0	0	0	0	0

83b50e51bccf50bdf42b4f520701fc19	0	0	0	0	0	0	0	0	3	0	0	0
9e7a79e4a03e6b071c41159d4a682c3a	0	2	0	0	0	0	0	0	0	0	0	0
0ea5b3567b8e087a03dffad253ce3058	0	0	0	0	0	0	0	0	0	0	0	3
5d224b7b4e84ff0646bfe10ae4f2bce6	0	0	0	0	0	0	0	0	0	0	0	5
0fe000e1d53b70c1908477070eb89536	0	0	0	0	0	0	0	0	0	0	0	3
910e914c8aa309ba472e2fd892c30b5a	0	0	0	24	0	0	0	0	0	0	0	0
1e2fff5b9c809f592b3799cdc083463d	0	0	0	0	7	0	0	0	0	0	0	0
392fd8db8a243902adff960ea4ec9604	0	0	0	0	0	0	0	0	4	0	0	0
8e340889ce4cfe65ce19661b81f77cb7	0	0	0	0	0	0	0	4	0	0	0	0
7bc8c7540554bd6a17279b6be4ea3d9d	0	0	16	0	0	0	0	0	0	0	0	0
0ce25a1026a8365dfa5c462bcd4f0b8d	0	0	0	0	0	0	0	0	0	6	0	0
7b7971f15a081c2d273105e78cbc1b71	0	0	0	0	0	0	0	0	0	0	52	0
2c46ddac0715d5990e1ec842404508f4	0	0	0	0	0	0	0	0	0	0	0	54
12894f9c10d061ad7c6c0174cccd8a66	3	0	0	0	0	0	0	0	0	0	0	0

3799b00df008663d323586086dff2240	0	0	0	0	0	0	0	0	0	0	4	0
097b31a0ec5f4d5c76a3b4e093620058	0	0	0	0	0	0	0	0	2	0	0	0
f0fbabdfb874050aa2720a1b821f5fd6	0	0	0	0	0	0	0	0	32	0	0	0
7dafc74db551a9759bba23e9ae610b72	0	0	3	0	0	0	0	0	0	0	0	0
2292478ace1f62817e2cd9f62bdbe972	0	0	0	0	0	0	0	4	0	0	0	0
32a5db6566e730a85a12cf93516fbfde	0	0	3	0	0	0	0	0	0	0	0	0
235a85f3fd063a56d5ca5379552460d9	3	0	0	0	0	0	0	0	0	0	0	0
389c3d4c2bbaf412802e367883d3b52d	0	0	0	0	0	0	0	0	0	2	0	0
b71ab5199f8950ad76936a45a2d45d6f	0	0	0	0	0	0	0	0	0	12	0	0
43a994d4ed80b78b88d901b25661279a	0	0	0	0	0	0	0	0	0	0	2	0
1f83cbe4e36a82ee7ca9eeaaa44f1f75	0	3	0	0	0	0	0	0	0	0	0	0
032f443616fcb7bbb5e731d933379c07	0	0	0	0	0	0	0	0	0	0	0	24
fa283fb55ccf6b5b954a7516cd255175	0	4	0	0	0	0	0	0	0	0	0	0
bfa3819f6769f73620424b06222f0297	0	0	0	0	0	0	0	0	0	4	0	0

5ff6cf3c5a20a602dc4d66aa0b957b09	0	0	0	0	0	0	0	0	10	0	0	0
0d1c73dcb2615315defe27ac1621a1a8	0	0	2	0	0	0	0	0	0	0	0	0
ddd4e3da36bfa610f9468c0e7e520b8c	2	0	0	0	0	0	0	0	0	0	0	0
0e6436c7a7c109af6934bace5e2eea19	0	0	0	0	0	0	0	0	0	0	0	27
3d05260d7fd5c3ee0f6179187c167e0b	0	0	0	0	2	0	0	0	0	0	0	0
188117fb1c6aa216fca883256148a053	0	0	0	0	0	0	0	0	0	0	5	0
a47c2ea103a67c250c33ff4581ce4b88	0	0	0	0	0	0	0	0	0	0	0	10
5eb1285c4edb75919440161b37476151	0	0	0	4	0	0	0	0	0	0	0	0
b191021e7c85082ac5a51afe7a06da13	0	0	0	0	0	0	0	0	17	0	0	0
9d7749cb2a6ae93e2f77f38d583d8bca	0	0	0	0	0	0	0	0	0	3	0	0
855e2bdef24e8e773b73475f41c38f75	0	0	0	0	0	4	0	0	0	0	0	0
2eee6c5789d60721980c61e38da8b121	0	0	0	0	0	0	0	0	0	51	0	0
2b345d857c32db347dbc53bc9a291edd	0	0	0	0	3	0	0	0	0	0	0	0
cbd84b0d69c1571745b402c924b10ee1	0	0	0	0	2	0	0	0	0	0	0	0

fa6d6defdbe548ab258f7484bf447b8f	0	0	0	0	0	0	0	0	0	6	0	0
da3d9ae9a76dfea72a3f7f49ee1d1b90	0	0	0	0	0	0	0	0	0	0	0	2
cbc17ddd817f6af1c5d34571fd822dfe	0	0	0	0	0	11	0	0	0	0	0	0
f453ec7751ccbf568a6a7aaf04c908ef	0	0	20	0	0	0	0	0	0	0	0	0
fe289e5f03cf055f0c97f7dfa444200a	0	0	0	0	0	0	0	0	0	0	6	0
fe6115c283a831534bf21eb55dbe82a1	0	0	0	0	0	0	0	0	0	0	0	8
bfe73faa514d491581a04ed80dcf9215	0	0	0	0	0	0	0	0	0	0	2	0
4dc5dcd0d16eacddf08215edc570e59d	2	0	0	0	0	0	0	0	0	0	0	0
4c1390e4c5fc0f6940583826a2f13bc1	0	0	0	6	0	0	0	0	0	0	0	0
d9cc4fd1eb74d1d66f869d62ff70d1d5	0	0	0	0	0	0	0	0	7	0	0	0
2ff5b8d95ffb3abc1673e0b00db7538f	0	0	0	0	4	0	0	0	0	0	0	0
5f113ed23125c18f53c5e98c12b292b4	0	0	0	3	0	0	0	0	0	0	0	0
32a74a478a8f8b5f8a79940507b025bb	0	0	0	0	0	0	3	0	0	0	0	0
df3edba176a9abc109ba76ed07747cbc	0	0	0	0	0	0	0	0	0	0	15	0

51983039167014d4e59671acb46673f1	0	0	0	0	0	0	0	0	0	0	5	0
307bebe0f06d52ba8813b90f6fa82de2	0	0	0	0	0	11	0	0	0	0	0	0
633ca03b2f99e0fe8b0df16435a75d28	0	0	0	0	0	0	0	0	0	0	0	3
dc56491b65a32d2bfe5d12084e834ff7	0	0	0	0	0	0	0	3	0	0	0	0
e7c255935580c526d7ccf32c082e52a6	0	0	0	0	0	0	0	0	57	0	0	0
7ee324c7271875781abf3191eb6102e4	0	0	0	0	0	0	0	35	0	0	0	0
042ebd34e81205a483deaae79073fcaf	0	0	0	0	0	0	0	6	0	0	0	0
d4aa4e7e8a93d35656b4faa0a5671ca7	0	0	0	0	0	0	0	0	0	0	0	44
787223c52e2dc10741167d21cb705a03	0	0	0	0	0	0	0	0	9	0	0	0
e57af5e2933d42fe1ed66f3c75a0ac6a	0	0	0	0	0	0	0	0	0	0	3	0
1dbd26cb8de7e6a80e2d0e628b64e07d	0	0	0	0	17	0	0	0	0	0	0	0
125337330e7fbc25c6928f49d97dc6ca	0	0	0	0	0	0	0	0	0	31	0	0
1c232ec9e5f0a71b31967468e8f7d5d9	0	0	14	0	0	0	0	0	0	0	0	0
75daa5040dc0c7726cebdb786bc6ee04	0	0	2	0	0	0	0	0	0	0	0	0

6626d38bd7e366d9bc8d99e0b8da6310	0	0	0	0	0	2	0	0	0	0	0	0
34c234140a72d9d70fd69b3aec06c3ed	0	0	0	0	0	0	0	0	2	0	0	0
38457befaff0cebff160c63118766043	0	0	0	0	0	0	0	2	0	0	0	0
197e5bb4cda540cf96032f33f319fd9b	0	0	0	0	0	0	0	0	0	0	5	0
99634ff4d8221e112aadfff6fc98f79a	0	0	0	0	0	0	3	0	0	0	0	0
a738ad878481c7e256cd528aba5f0459	0	0	0	0	0	0	0	0	0	0	0	13
d7e18e14008315e380b6ad048f0dc2c8	0	0	0	0	6	0	0	0	0	0	0	0
eb26d4780c8655ba26f159860bb97d98	0	0	0	0	0	0	0	0	3	0	0	0
a4ff1152b873deacf6e7f9c597e53717	0	0	0	0	0	0	0	0	0	9	0	0
541ccdf6a53bf58c5aa9641ce811b59a	0	0	39	0	0	0	0	0	0	0	0	0
ac69aa7b13ab62f2df877bbf9ad072ee	28	0	0	0	0	0	0	0	0	0	0	0
3b238ef76fab59c676e75b779404bb09	0	0	0	9	0	0	0	0	0	0	0	0
0e974c6f348b586887efdb9f56dd5111	0	0	0	0	0	0	2	0	0	0	0	0
e0895834823a855f0360df0b5764f194	0	0	0	0	0	0	0	16	0	0	0	0

d500d9a5d4fc4f72d092bcddceeb69a4	0	0	0	0	0	4	0	0	0	0	0	0
8d2a45cc60ad794732ae9d5fa43d810e	0	0	34	0	0	0	0	0	0	0	0	0
0d0dd7745446b460a13a40d0fc5b78e0	0	0	0	0	4	0	0	0	0	0	0	0
365b8cbf66fbcf81f5810007058db74b	0	0	0	0	0	0	0	0	3	0	0	0
b9a3c5e153928d03648105dbfad9ea12	0	0	0	0	0	0	0	2	0	0	0	0
bb63ec609fa0decf7b001717e1cd3a00	0	0	0	0	0	12	0	0	0	0	0	0
61d5f95e131c0245fcee1d590a0441e7	0	0	0	0	0	0	0	0	0	0	14	0
ed5112bb80f84ee7e97f137605714c2b	0	24	0	0	0	0	0	0	0	0	0	0
e44fde619c76f7d85a0f9d889c4d1b71	0	0	6	0	0	0	0	0	0	0	0	0
d9bb333a39306a48f5595c105b7be6ae	0	0	0	0	0	53	0	0	0	0	0	0
b7d452c8f826150060b5b3a12801fc58	0	0	0	0	0	5	0	0	0	0	0	0
d8857409dfddd607848eb18a9ff6a1ab	0	0	0	0	0	0	0	6	0	0	0	0
36d3a70ff5c7cfa6b2688c7b63d20276	0	0	0	0	0	0	0	0	0	0	2	0
28e61ad22868d652b0f305090ab34989	0	0	0	0	0	0	0	0	0	20	0	0

9f407084ab62cac8b7a1ff769 8a1172d	0	0	0	0	0	0	0	0	0	15	0	0
fa545844233557ebb8b46663 ea074afa	3	0	0	0	0	0	0	0	0	0	0	0
4fe13b04383bf6cb0dd42557 2ca3b883	0	0	0	5	0	0	0	0	0	0	0	0
6bcea54f3a47869d34fc2b73 3b73d442	0	0	0	0	0	0	0	0	0	0	3	0
a09c245bd053617cc2be83bf 9f5ac87a	0	0	0	5	0	0	0	0	0	0	0	0
56ca717a7fabbd4891514bdd 79a01c51	0	0	0	0	0	0	0	0	13	0	0	0
02c08df2286d447dc7898a13 1c2b000e	0	0	0	2	0	0	0	0	0	0	0	0
20415836549998c8d58aa99 36ff39711	0	0	0	0	0	0	45	0	0	0	0	0
e87d8f4e0a7bcad86bec5e44 024f041a	0	0	0	0	0	0	4	0	0	0	0	0
1089facedb2901876f7b8b80 49cf4dac	0	0	0	0	0	0	0	0	0	6	0	0
9622c86c3f4422832cf8af5f7f b8d5bb	0	0	0	0	0	0	0	0	3	0	0	0
95e49459b339af746db8acaa 622d336a	0	0	0	0	0	0	0	0	0	5	0	0
ab82bcf14d80cd678c92c4a1 42a43082	0	0	0	0	0	0	0	4	0	0	0	0
e96ff4e617a0fe414bf209ae9 d7731d1	0	0	0	0	0	0	0	0	0	0	2	0

a361d01be130b1e42f01cbff60f2d451	0	86	0	0	0	0	0	0	27	0	0	0
e3d19f2ec5f03f2ae9f73f0d1a4df442	0	0	0	0	5	0	0	0	0	0	0	0
a29ab9cc0025a405acc00fda9df28af2	0	0	0	0	0	0	0	0	0	0	3	0
6802c1107aecfd3d4231384a2ddc0749	0	0	0	0	0	0	0	0	5	0	0	0
c2ed5dfe5bc9e4bcf74780ecd3a80f20	0	0	0	0	0	8	0	0	0	0	0	0
ce987bebc9122fe2146db05ad6db557d	0	0	0	0	0	0	0	0	0	0	2	0
34ff1b8ca59b04bd62f078d467a54c7d	0	0	0	4	0	0	0	0	0	0	0	0
6658509bc73ef9fcc072363c6277e8e1	0	0	0	0	0	0	0	10	0	0	0	0
07513eca6def6faf39d3f27673382488	0	0	0	0	3	0	0	0	0	0	0	0
a2cdbebf92922d5ae4eb67b03ea000ec	0	0	0	0	0	0	0	0	0	6	0	0
4b42203d4a4a4d9927aaf7229165969c	0	0	0	0	0	0	0	0	0	0	0	9
1b58e84842bd9822d40d22a52af0f0e7	0	6	0	0	0	0	0	0	0	0	0	0
9ff12fdd063adb4a3971a7e6b9723cb0	0	0	0	0	0	0	0	0	3	0	0	0
8a9f60fe90173db5d5c4cdbdbde0c22b	0	0	0	0	0	0	0	0	0	0	3	0

2750591b855a4fc393e20e54 17f2a39f	0	0	0	0	0	0	0	0	0	0	0	12
397eccd3d2541649202effb2 be32ec66	0	0	0	0	0	0	4	0	0	0	0	0
efb8e14bdc44a6b375d5372c 1807229e	0	0	0	0	0	31	0	0	0	0	0	0
e7194d5dce2d12047c70176c 74554764	0	0	0	0	0	0	0	0	0	0	0	3
af004fb6f1659e17e51cd81a0 8c1d8b8	0	0	0	0	0	0	0	0	0	0	0	10
d63860ed222224d461d8601 1d47fb1d4	0	0	0	0	0	0	0	0	0	0	0	15
636a8138a7061191c3bc13f8 50f266e3	0	0	0	0	0	0	0	0	0	0	97	0
7bc1f49d19a2f707014fa900b 78a76e0	0	0	0	0	0	0	0	0	5	0	0	0
cb35bc703585627fb45bccf9 d1f91fbd	0	13	0	0	0	0	0	0	0	0	0	0
f11c0986e4ade2861aa64dfb 6ced60eb	0	0	0	4	0	0	0	0	0	0	0	0
c32b8393666da7fc6d94e5b6 a57d67c9	0	0	0	10	0	0	0	0	0	0	0	0
0306c858d7fd0ece87e9b6e6 5672e083	0	0	0	0	0	0	0	0	0	60	0	0
ed20c3caf1593090ad1daa7d ca388ecc	0	0	0	0	0	0	0	0	0	2	0	0
248171c1ac64a4b697caab53 ddf2df84	0	0	0	0	0	0	0	0	0	0	2	0

ea40952b3a1e426899582070312b1869	0	0	0	6	0	0	0	0	0	0	0	0
38a59fe4de783f89c2fcc9ddcedc2fb1	0	0	0	0	0	0	0	0	0	0	13	0
9a98af8463262ed6a4044041a9cbb3b8	0	0	0	0	0	0	0	0	5	0	0	0
ec1e18caf8c2806b7e4bfd99b8cddddc	0	0	0	0	0	126	0	0	0	0	0	0
a15738b21d48e527a59aadec2f2d4910	0	0	0	0	0	0	2	0	0	0	0	0
c8a95e68a542db97fd41043ebf3548cf	0	0	0	0	0	0	0	0	0	0	21	0
16b2322854021df6eb6e1f154d91a87b	0	0	0	0	0	0	0	0	0	0	0	8
3ed58aec6fc918536ee2e319185613f6	0	0	0	0	0	0	0	0	0	0	3	0
da40f526801243da30f34bd05cd033af	0	0	0	0	0	0	0	0	0	49	0	0
2d2139b1b4b1e17c8016cf3c5e3a3585	0	0	0	0	0	0	0	0	0	0	19	0
3f6a42234fe9377c2a8c415979724653	0	0	0	0	0	0	0	0	0	0	0	3
0728d1edda7737525d6579d3cf957577	0	0	0	0	0	0	0	0	0	0	4	0
da0a9d6c073829ecd5da4b8855f662fe	0	0	61	0	0	0	0	0	0	0	0	0
7d79bf957bd7dc98a712653fd28b13cc	0	0	14	0	0	0	0	0	0	0	0	0

f6ee305d92d34b45985af5d2c0628913	0	0	0	0	0	0	0	0	0	0	2	0
7f377183c55fc2a396e4f9952d494b6b	0	0	0	0	0	0	0	0	2	0	0	0
be373085b8d6cef9155712bedaca07a8	0	0	0	0	0	0	0	0	0	0	0	5
049a5426d306f9a328db96890c6346cd	0	0	0	0	0	0	0	8	0	0	0	0
59526854924772a5eea7017decd30216	0	0	3	0	0	0	0	0	0	0	0	0
4b849d8f321427d7d2bf50958bd005f8	0	0	0	0	0	0	0	0	0	0	2	0
575aa5f2dcf24b26f026ad335e48fc63	0	0	0	0	0	0	0	0	0	0	0	154
a8b898ab110b0b1f8bb3f991c0d4bda6	0	0	0	0	0	0	7	0	0	0	0	0
c268a40ed69966623c168514f4b271bb	0	0	0	0	0	0	0	0	0	0	28	0
458c1c78f37403e569c84c4e7377c31a	3	0	0	0	0	0	0	0	0	0	0	0
ecbe279633af414e3ca540841e61ed0e	0	0	0	0	0	0	0	12	0	0	0	0
42fcdadd33eadde031233abd844eb762	0	0	0	0	0	0	0	0	0	0	0	3
fcd54a5f6e2c0521d6e066ad9dad73cd	0	0	0	0	0	0	0	2	0	0	0	0
052826c9279eb3ad75ac7a42f9ac7c84	0	0	0	0	0	0	0	0	0	0	0	7

eda30a3fbc2f5bcc6ec194133 a838c0d	0	0	0	0	112	0	0	0	0	0	0	0
30aa48400a492fafd9dfdfad1 06de697	0	5	0	0	0	0	0	0	0	0	0	0
ad012d8ac8e05f8ee56fdaeb 1f97c22c	0	0	0	0	0	0	0	2	0	0	0	0
a932e51a7db50e29456db8b b762eddb5	0	0	0	0	0	0	0	0	0	6	0	0
079298017626249802fc263b 5a7ad0c6	0	0	0	0	0	0	0	0	2	0	0	0
3a2834a2bd02d000641d4f86 f3666d38	0	0	0	5	0	0	0	0	0	0	0	0
256ed4b91dfe7a085ad7ca42 603e9293	0	0	0	88	0	0	0	0	0	0	0	0
c4aa2ad66c1caffab15d226c0 ef62463	0	3	0	0	0	0	0	0	0	0	0	0
26cfd954478d05bed2e137f8 441de942	0	0	0	0	0	0	0	0	0	0	5	0
30dbcaaf80b5eb84a1560c70 affba149	0	0	0	0	0	0	2	0	0	0	0	0
2d32b56f8e4fa19ea5e162ce 92f2ba66	0	0	0	0	0	0	0	0	0	0	4	0
f3ca7783a670085d0d1b553a 2e721143	0	0	0	0	0	0	0	0	16	0	0	0
765c83fb193bea0e0d773ebc f55bee52	0	0	0	0	0	0	0	0	0	8	0	0
244b610d7ed8bae386d5c31 28e3ac7f9	0	0	0	0	0	0	0	0	0	0	8	0

c91beaa9c6037a216b323514140cf34a	0	7	0	0	0	0	0	0	0	0	0	0
13afeb7ed8b368396503285cb33383d8	0	0	2	0	0	0	0	0	0	0	0	0
d9baf871dc795edd792286ab41f717b0	0	0	0	0	0	0	0	7	0	0	0	0
8cc7b093ef745f8502770e5bf7a66db1	2	0	0	0	0	0	0	0	0	0	0	0
a42e3be06a9137fec4e61fc2a73dead1	12	0	0	0	0	0	0	0	0	0	0	0
255591b08b8997a1e812d2fa35966346	0	0	0	0	0	0	0	0	0	0	0	2
e54b5540508e147ac439cb4df5910e67	0	0	0	0	0	0	0	0	0	0	0	6
5ce1d0c1a282bf40932c39b44a95b0d3	0	0	0	0	0	4	0	0	0	0	0	0
2269842ebf8006c48e7a116df38a9a23	0	0	0	0	0	0	0	0	0	0	0	255
1f177f30fa987f0abbed628dd27bd705	0	0	0	0	0	0	0	0	0	0	0	6
824a97cbb8c1c776c26827857f057f19	0	0	0	0	0	0	0	0	0	0	3	0
4213433077285b0fbb05a232bd2e1e1a	0	0	0	0	0	0	0	0	0	0	2	0
eb35284719ef2c0f0988557040ae5b85	0	0	0	0	0	0	0	0	0	0	0	10
9da80d15deace534614ce31f69cd3e2d	0	0	0	0	0	0	0	0	0	0	0	11

d11e30a1fdf99e442aca82c1f4f69c02	0	0	0	0	63	0	0	39	0	0	0	0
2cb579ae2d126dcedf3a3cb7d2ccce2b	0	0	0	0	0	0	0	0	0	0	0	7
7cc6149b7a414c92ac2a68fb6b69f1da	0	0	0	0	0	0	0	0	5	0	0	0
9ef7c5ba3b413a3594655cff94d019b3	0	0	0	0	0	0	0	0	0	0	0	25
740073a339bbe54d59ead0e311bbbaa7	0	0	0	0	26	0	0	0	0	0	0	0
75c2177eebda9f807e54874ba7a30a68	0	0	0	0	0	0	0	0	0	0	3	0
f7043771cb38517c83c402e50553e0fe	0	15	0	0	0	0	0	0	0	0	0	0
36f0ddda09fb83365e23777fb37ad71a	0	0	0	0	0	0	55	0	0	0	0	0
a4830d6476b461611618a2bd28ae3f46	0	0	0	0	0	0	0	0	0	30	0	0
cb0ceeeea5dcbc06410aa65de44c2bf2	0	0	0	0	0	0	0	0	4	0	0	0
810eaa0bdc733a56a34fd3e18f77de6a	0	0	0	0	0	0	4	0	0	0	0	0
9022e7173f9e8f67cf007bf54789d8d5	0	0	0	0	0	0	0	0	0	0	29	0
13d7fa5d9894db05ddbcfe2e41c8d0ed	0	0	0	0	0	0	13	0	0	0	0	0
1f25f310d9375fcdbcd17a73575b22f6	0	0	0	0	0	0	0	0	0	0	4	0

288566e0dff5a9c0a4278041e07337ce	0	0	0	0	0	3	0	0	0	0	0	0
74d7344c950c975219aff2825087f7f5	0	0	0	0	0	0	0	0	0	0	3	0
30e6cff089a8a1632f22f2e36e83d8b7	0	0	0	0	0	0	0	0	0	0	0	6
443f05ea46aebc1bcea9a87a475f7aeb	0	0	0	0	64	0	0	0	0	0	0	0
0640728b8bc24471737efe27322e4e2b	0	0	0	0	0	0	0	4	0	0	0	0
69e07dadc91f35e48b6790f6dadfb2e9	18	0	0	0	0	0	0	0	0	0	0	0
c660b6240347fbf9e160ce2c0e4c0c08	0	0	0	0	0	0	0	0	0	0	3	0
8fc3825e5950f2a65885cf47e6476716	0	0	0	0	2	0	0	0	0	0	0	0
e8c79f3e989339657367c29c756170cf	0	0	0	13	0	0	0	0	0	0	0	0
60fb2314f481c3fc945439bf375b9e62	0	0	0	0	0	8	0	0	0	0	0	0
62b26a662e8a32da72ce43e0646f771c	0	0	0	0	0	0	0	0	0	0	19	0
27189adad1b5a2d870d82bc1c95cf66d	0	0	0	0	0	0	3	0	0	0	0	0
6bd4811c583bf7ac0cd40b08c6776fae	0	0	0	0	0	0	0	0	0	0	15	0
c5343e2a55ca676c23b00448f6128862	17	0	0	0	0	0	0	0	0	0	0	0

ce396c3520cdeefe5e441633 9ac0e44a	0	0	4	0	0	0	0	0	0	0	0	0
0b6b4f56703bc4441e84db34 f28276e9	0	0	0	0	2	0	0	0	0	0	0	0
129a5509b2287811351c4ca8 107979b3	0	0	0	0	0	0	0	0	0	0	3	0
60bfcc4b9db74ec85b2b3d05 9b7ec9c8	0	0	0	0	0	0	0	2	0	0	0	0
0183a147bebd232d15a0e0e 7dc4f821a	0	0	0	0	3	0	0	0	0	0	0	0
7d84fc4bf3b74188d2d5a24e 2ad0d166	0	0	0	0	0	0	0	0	0	0	0	4
66597578a5d27f9496cabdfc 7cdd53e1	0	0	0	0	0	4	0	0	0	0	0	0
69da6bd119c0b66512ec2d1 014b19d63	0	0	0	3	0	0	0	0	0	0	0	0
85dcc3a3ed9cc25a6f26cb5fa 949d35f	0	0	0	0	11	0	0	0	0	0	0	0
3ed0566d22a3b4e2185d8e1 3d55caee6	0	0	0	0	0	0	0	0	0	0	0	36
2261e6383b9bf5138c84a9f0 903dbb5d	0	0	0	0	0	0	3	0	0	0	0	0
a236c071aa3a394312af0f0ff 01cee67	0	0	0	0	0	0	0	0	0	3	0	0
cf629706baeb8f1d2c246208 b8ea2825	0	0	0	0	0	0	0	0	0	0	0	5
46be4083c3f1f493346f1ed5f d1cbfb7	0	0	0	0	0	0	0	0	20	0	0	0

7982f2d3da6e230668b175f255e2fce1	0	0	0	0	0	0	0	0	0	0	3	0
c7484846a224aa23c4afde65579bd59e	0	0	0	0	0	0	0	0	0	0	0	9
2e309c0156879ada663721e6f1684935	0	0	0	0	0	0	0	38	0	0	0	0
3ba29f235efb4b17152eece80926d171	0	0	0	3	0	0	0	0	0	0	0	0
c11064a3c1911ec4f35acee1ae73f34d	0	0	0	0	0	0	0	8	0	0	0	0
d9f236cdd285e8375b85af60ec1c93cc	0	0	0	0	0	2	0	0	0	0	0	0
393860f9ba04b12fd477f5beab468ba6	0	0	0	0	0	0	0	0	5	0	0	0
de005dc1117dfee3e03f060d6344ef63	0	0	0	0	0	0	0	0	124	0	0	0
c64f7ce14347229abb09a15d9e0b8b42	0	0	0	0	0	0	0	0	0	0	0	63
d275127af4b0f02a84b9d3ff02ad07cc	0	0	0	0	0	0	0	0	0	0	0	5
865183552a52769e71ce95cf785bc6ae	0	0	2	0	0	0	0	0	0	0	0	0
75bd65cad9968c057512c897c570b9c5	0	0	0	0	0	0	0	0	0	0	2	0
b84f39e660907d4f48a07d87bc559fda	0	0	4	0	0	0	0	0	0	0	0	0
3810fbd726bb70958a8f50a5dd4fc5af	0	0	0	0	0	0	0	0	120	0	0	0

8ccbb8c3a56aa18f75d86253 5168d82a	0	0	0	6	0	0	0	0	0	0	0	0
8aeed96f3061d59d65761c45 28b39b3d	0	0	0	0	0	33	0	0	0	0	0	0
46fca44b1a4cdf84a5ffdb5b2 5681be8	0	0	0	0	34	0	0	0	0	0	0	0
878c982f2ea00a8bb335e958 4f224ac2	0	0	0	0	0	0	0	2	0	0	0	0
d7a6db4e2ac13c4564a90695 3eb655cf	0	0	22	0	0	0	0	0	0	0	0	0
ae06ddaa17bd730346d0709 700941213	8	0	0	0	0	0	0	0	0	0	0	0
d760acc0d51235eae29ecf70f d417c75	9	0	0	0	0	0	0	0	0	0	0	0
ffd19b2289ed1f7111c5a6e3b 78886d9	0	0	0	0	4	0	0	0	0	0	0	0
161535124f8f4c89cafb8a0bf 2647429	0	0	0	0	0	0	0	0	0	5	0	0
117f486f1249958eca784557 5939bffe	0	0	0	0	0	0	0	0	0	0	0	67
0d02d4060621a6ba07253d6f af9c25c4	0	0	0	0	0	0	0	0	0	6	0	0
5ce2b0c044f3285e4fdbc966 e210db0b	0	0	0	0	0	0	0	0	0	0	4	0
4df1736b7e35adbe67cca847 70ee1d84	0	0	25	0	0	0	0	0	0	0	0	0
b6725112e1fd7cb40a871fae 2bb5721d	0	0	0	0	2	0	0	0	0	0	0	0

98cfce64db878d8b38ab939f0a376335	0	0	0	0	0	0	8	0	0	0	0	0
d377a5adbeae38bc10b01117d8a84dca	0	0	0	0	0	9	0	0	0	0	0	0
0d403ed49566738ad3fc805975f313c1	0	0	0	0	0	0	0	0	0	0	0	4
96f1d5e60fc8db6b3f814a4d0dc8aa58	0	0	0	0	0	0	0	0	2	0	0	0
2f7db7ae33dc60d50881ee1e22b096d7	0	0	0	0	0	0	0	0	0	0	6	0
14702743d054951e1f5c9ef7eead5b1f	0	0	0	0	0	0	0	0	0	0	0	4
1dadafe4f8c67cf797ae1d21fb4de2c1	0	0	4	0	0	0	0	0	0	0	0	0
e2cab65b95295a6efea0167662992593	0	0	0	0	0	0	0	0	0	0	10	0
5c256b5c164c0328d5cf240a6b41bf68	0	0	0	0	0	0	0	0	3	0	0	0
ebf85267305ceff982e4a039dd2d062c	0	0	0	0	0	0	0	6	0	0	0	0
ed42950aa6378477ae6f099e1b95270c	0	0	0	0	7	0	0	0	0	0	0	0
6bf995658a8d2fb8c26e18530dfad9a0	0	0	5	0	0	0	0	0	0	0	0	0
b5aa11b671ca9dd3491c859d64a99c9d	0	2	0	0	0	0	0	0	0	0	0	0
daa281c004d023cc152b7f87855962fb	0	0	0	0	0	0	0	0	0	0	2	0

92b0a90a10025e001147733 d55833e38	0	0	0	0	0	0	0	0	0	3	0	0
66428ac0f2717cfbeea53f81ff c64bb3	0	0	0	0	0	0	0	0	0	0	4	0
0a38d54e6445620e84284cf6 64c67c56	0	0	0	0	0	0	0	0	0	0	9	0
4116c889506ac8b98cb4b36b 8c474c39	0	0	9	0	0	0	0	0	0	0	0	0
5fc2c16e08ac89de69e9294c a62303d1	0	7	0	0	0	0	0	0	0	0	0	0
c2d5af0702cad54d27b8c897 f1a7e908	0	0	0	0	0	3	0	0	0	0	0	0
82ec235f63d7f3860ca2ddcb 745fe12f	0	0	0	76	0	0	0	0	0	0	0	0
4ba53f0cf6ed66ea888cf538d e00f500	0	0	0	0	0	0	0	0	0	12	0	0
834073c3ea52ca4f6df54e2ff 4c89666	0	0	0	3	0	0	0	0	0	0	0	0
994e26cc0a88504309382d5f 41a4d02c	0	0	0	3	0	0	0	0	0	0	0	0
b85f9b14a6fa5487d0d4a707 84f9614d	0	0	0	0	0	0	0	0	0	79	0	0
2e00861cbd0331cd3a63839 b8712ead0	0	0	0	0	0	0	0	3	0	0	0	0
b6ca0fe9d88e59edf596a042f acf2280	0	0	0	0	0	0	0	32	0	0	0	0
67053648c88586c8dd7d180 0d4f663a0	0	0	0	0	0	0	0	0	0	0	2	0

e1ca89105694950ee1b44d87e2553862	0	0	0	0	0	0	0	5	0	0	0	0
5f7caf863c7468bffd4e4c1a0f9188bd	0	0	0	0	0	0	0	0	0	0	0	5
b196873f936417f60754935812504820	0	0	0	0	0	0	0	0	0	0	3	0
2d4a2b8d86b0e9ab86891cb1f3fac178	0	0	0	0	0	0	0	0	0	100	0	0
36a87ef50ce38c81b8686e8336cebe0e	0	0	0	0	0	0	0	0	0	0	50	0
27248a9eb6724eb45f6972ee45af8e61	0	0	0	0	0	0	0	0	18	0	0	0
6df9ddad612d43e82090fd58c5bf5dc2	0	0	0	0	0	0	0	0	0	0	0	5
35dc03fe63d6bce11cf82a2b70aec703	0	0	0	0	0	0	0	0	0	2	0	0
f1678b9d75baca04504d86cd50dd256a	0	0	0	0	0	0	0	0	0	0	4	0
75b36aca659aa224f6cfd9846e1da00f	0	0	0	0	2	0	0	0	0	0	0	0
3d200013a1234ee112e2ee12ebac3bcf	45	0	0	0	0	0	0	0	0	0	0	0
f0d019d6fc37b4ec5b2a9076fc31028f	0	0	0	0	0	0	0	0	0	92	0	0
3f54bc51e50ff34e1ab438177c79eae2	0	0	0	20	0	0	0	0	0	0	0	0
a6c14b97e35a19030bd7e131e406baf9	3	0	0	0	0	0	0	0	0	0	0	0

bb01c70cff2a0c9d465ec2dcb082e688	0	0	0	0	0	0	0	0	2	0	0	0
eb4e5591ff9d5dc20b65d13a0539a836	0	0	0	0	0	0	5	0	0	0	0	0
09653dfafd8691cec10fdee455d49adb	0	3	0	0	0	0	0	0	0	0	0	0
22f7857cfa641567e7c3f2cd545cb247	0	0	0	0	0	0	0	0	0	0	16	0
d5c43ee7a385f5fb195be17a8668b31f	0	0	0	12	0	0	0	0	0	0	0	0
e211a071baab3d7d593dad5d94e4274a	0	0	0	0	0	0	0	0	3	0	0	0
16a7717029ef9d54dd1867ad8de7feb1	0	0	0	0	0	22	0	0	0	0	0	0
ecd79b308e1b2f92ad4d1fe5665269b0	0	0	0	0	0	0	0	0	12	0	0	0
d3cad6c74c08e0c112cc74e0f43bc470	0	0	0	0	0	0	0	2	0	0	0	0
dc297c70e88d85ebe0a98df6691a0a30	0	0	0	0	0	0	3	0	0	0	0	0
a2fb3ee367273fc918a7095a135e8da2	0	11	0	0	0	0	0	0	0	0	0	0
2fa51e71b3ec4062fea6b88c8a98441a	8	0	0	0	0	0	0	0	0	0	0	0
d4d978d791d93d16cd4b4eb90c8feb80	0	0	0	4	0	0	0	0	0	0	0	0
cfe5aa3fd72ff7856be7fce5707b1071	0	0	4	0	0	0	0	0	0	0	0	0

363b9f6b7a942fa3c214ab65 67b028a4	0	0	0	0	0	0	0	0	0	0	3	0
ffe8891d4ed9b3c44229b227 a2f643df	0	0	0	0	0	0	0	0	0	0	0	3
f3e390f3af3391319064846b 2ced5b27	0	0	13	0	0	0	0	0	0	0	0	0
d323fd0530bad63c655536ac 5c408195	0	0	0	0	0	2	0	0	0	0	0	0
002d5807c5b528977e2339fb b8dc8c62	0	0	49	0	0	0	0	0	0	0	0	0
14e0537703ef02739e54b335 3ae6a3ea	0	0	0	0	0	9	0	0	0	0	0	0
dc3d1cddee8731cee68cfafd0 5ee8ddb	0	0	0	0	0	0	0	0	2	0	0	0
4f7f28e4a64ef05b7ab5d1fb8 1fb2e93	0	0	0	0	3	0	0	0	0	0	0	0
44475fc058a21d51f278b132 577d82ca	0	0	0	0	0	2	0	0	0	0	0	0
970d4585e2812537415ad54f 2855c2f9	0	0	0	0	0	0	0	0	0	0	68	109
79ec88a7602fe0d90356de3e 622e2534	0	9	0	0	0	0	0	0	0	0	0	0
64cda662b8f0b7f35285ad3e 499626b2	0	0	0	0	7	0	0	0	0	0	0	0
08afcb1037bf4252cf1782e49 5495298	0	0	0	0	0	0	0	0	0	0	144	0
92069a2290b9df9bb5f020b2 12f08b32	0	0	0	0	0	0	0	0	0	0	5	0

20c2465fb4eb3bc8a8c6a9ca48e877b5	0	0	0	0	0	0	0	0	0	4	0	0
0bcd35bb476980db61151af424b045f4	0	0	0	0	0	0	0	0	0	0	0	2
1ac1989a6a7877c3ae17afb5e08d60b3	0	0	0	17	0	0	0	0	0	0	0	0
bcd3705c7045438b4fddc0fbab77578b	0	0	0	0	0	0	0	0	0	0	0	107
19e89b08027ee0781eefc87039964d00	0	0	0	4	0	0	0	0	0	0	0	0
7c05422a85fcb6eccd8fd37ddaa62aa3	0	0	51	0	0	0	0	0	0	0	0	0
2fd0675788961ec2de5b3a18af03f95e	0	0	0	0	0	0	0	0	0	0	0	25
f3116488ef9261ac6b3f1a0d1c519b23	0	0	3	0	0	0	0	0	0	0	0	0
c8797a597860cfefa144871b102ecce1	0	0	0	0	0	3	0	0	0	0	0	0
687a489fc843b702374acebe4847dc1c	0	0	0	0	0	15	0	0	0	0	0	19
3a3328cb5dca0c8a1b338b50a221848f	0	0	0	0	0	25	0	0	0	0	0	0
6b1c864276c079a9f3c9832a9cf610c9	0	0	0	0	0	0	7	0	0	0	0	0
a517a8275e16b5dedee01aeca7955d9f	0	0	0	0	0	7	0	0	0	0	0	0
002238a58aacf6b4aa0daf2ad6c1df8c	0	0	0	92	0	0	0	0	0	0	0	0

4a0aabf08d65cf1e4d7f48cea5f03eb9	0	0	252	0	0	0	0	0	0	0	0	0
836a5768fc288dc77fcf905db8bd4728	0	0	0	0	0	42	0	0	0	0	0	0
9a82e2e54d810c2657a806d6c51ca726	0	0	0	0	0	0	0	2	0	0	0	0
91d5d885998424393fe449d06a6674f8	0	0	0	0	0	8	0	0	0	0	0	0
afbaa410fa5ad3395d3fb7b1c095d10f	0	0	0	3	0	0	0	0	0	0	0	0
dce0cb9b2692842d196c36f9e3cd1041	0	0	0	0	0	0	0	2	0	0	0	0
dc8800d353648328501abbf82a3045fb	0	0	0	0	2	0	0	0	0	0	0	0
95f91e062a4ab2e16581b349f2de744e	0	0	0	0	0	0	0	0	0	0	3	0
a7777089e76f01b4693aa3d245431ac6	0	0	0	0	0	0	0	32	0	0	0	0
d61879f3dada19fb3bcdeac08fdff628	0	0	0	0	0	0	0	0	0	0	0	3
85871dbb6b52bf3653242d9c2c0b9eae	0	0	0	3	0	0	0	0	0	0	0	0
158e99083e5f122f190e5f4a1d5886bd	0	0	0	0	0	0	0	12	0	0	0	0
7a894ba41ca84f26a2742e977e10a03e	0	0	0	0	0	0	0	0	2	0	0	0
bdb93c73b684f98ecfd6e2fe2d2065e8	0	0	0	0	0	0	0	0	0	0	0	4

7976010d1055ebe2e300dfb 4da4d5ae6	0	0	0	0	0	0	0	0	0	0	0	6
9b424b86f7f06af7568939bd 34fe36a2	0	0	0	0	4	0	0	0	0	0	0	0
612f691d76a02a4b125ec41b 7f4a4bd7	0	4	0	0	0	0	0	0	0	0	0	0
dd1cc00b9d99b9378b42469 b2c7230be	0	0	0	0	0	0	0	0	0	0	0	16
73f71c43e36694be5ebd3e14 a390d848	0	0	36	0	0	0	0	0	0	0	0	0
6ccfc33ef6f81a741d1b223c6 74243dc	0	0	0	0	0	0	0	18	0	0	0	0
1a767fea06bd1efb38be1e68 31baed88	0	0	0	0	0	6	0	0	0	0	0	0
ba01086ae24c2831eb333c17 86878b41	0	0	0	0	2	0	0	0	0	0	0	0
f007370e6e0b1de7228a858c c735ed84	0	0	0	0	30	0	0	0	0	0	0	0
84c9c2043c8c32ffae0cfb59c 9fbc215	0	0	0	0	0	0	0	0	0	0	0	10
cda5da1bfe93fae6d73e8459 56abdcea	0	0	0	0	0	0	0	0	0	0	0	3
5a44c9f56dc86e7fcd47e9493 a8a7323	0	0	0	0	0	0	3	0	0	0	0	0
851a7626ade7e5c342892b0 6682c5a6a	0	0	0	0	16	0	0	0	0	0	0	0
bcac2ac8f8cdac303f4b3cdb8 d81185d	0	0	0	0	0	0	0	0	0	0	4	0

9dbb49f8872db6378fbc4f3ef64de7de	0	0	6	0	0	0	0	0	0	0	0	0
c87a0d2d05c8b72dbb5cfcba8a9f2c85	0	0	0	0	0	0	0	0	0	0	2	0
d5d335348136abc84717d1c7769b727b	0	0	0	0	0	0	0	0	0	10	0	0
6128032a5415515c4426ea4377b58b89	0	0	0	0	15	0	0	0	0	0	0	0
f3acd4bb51d5da8417e305b4617c27ee	5	0	0	0	0	0	0	0	0	0	0	0
9375d7c84a8f4960ce294b6a994b2c6a	0	7	0	0	0	0	0	0	0	0	0	0
7775b3062d2b81784d8a7d732db253d3	0	0	4	0	0	0	0	0	0	0	0	0
ab7e0c746b5cc22d0a811856e244b1c3	0	0	0	10	0	0	0	0	0	0	0	0
a54798dfc20ee47124de07e0e6bb8ec9	0	239	0	0	0	0	0	0	0	0	0	0
f898f1a43e1cfcd909045318c316cd12	0	0	0	0	2	0	0	0	0	0	0	0
0ba86a3c2b2b891fd7f7608e1c84e998	0	0	0	0	0	0	0	28	0	0	0	0
3d9e4a71bee56d905209546af31e2227	0	0	0	0	0	0	0	0	0	0	5	0
855084e38e8f37f2aef9561b4935d154	0	0	0	0	0	0	0	0	0	0	3	0
a8d0519d1987974dc64f673524756923	0	0	0	0	8	0	0	0	0	0	0	0

5bb949f91d0359e8eca36c8e4cc053c7	0	0	0	0	0	0	0	0	0	0	0	2
c0b68732ba0bc9c519c5be0a3ab92a58	0	0	0	0	0	26	0	0	0	0	0	0
d178835c0713c7cf691bb04614d9dc90	0	0	0	0	0	0	0	0	0	201	0	0
725503ba84bf9b7a4885ce9c3a0c5a8f	0	0	0	0	0	0	0	0	0	0	0	6
99977165e7b7eaf5a776edf6d31318f1	0	15	0	0	0	17	0	0	0	0	0	0
c1cb896dcbb402a48f181ddab89e1d7b	0	0	0	0	0	0	0	7	0	0	0	0
850e872c3099c9f38d3728ffc2f1c2f4	0	0	0	0	0	0	0	0	0	5	0	0
096840b82aa1bb85464d379fe4845dbb	0	0	0	0	0	49	0	0	0	0	0	0
bf484a4cca96de26f635b79e9aea53bd	0	0	0	0	0	0	0	0	0	0	3	0
3fa60a27fab51c02c8067a2a867d5288	9	0	0	0	0	671	0	0	0	0	0	0
cf38bc17d34518b150c813268c4ae2d2	0	0	0	0	0	5	0	0	0	0	0	0
a650fb654ff6bc349f66c0ff1300ac9b	0	0	0	31	0	0	0	0	0	0	0	0
612be89a0ca4bcf7c4e273887a7c5f95	0	0	0	0	0	0	2	0	0	0	0	0
5c65098f3a659747ecbc7f52a679a61f	0	0	0	0	0	0	0	9	0	0	0	0

87e32c8fb1c60b106d26c817e7318a52	0	0	0	0	0	0	0	0	3	0	0	0
a79fcebd395c7c7c78fb3fca725ab2e4	0	0	0	0	0	0	0	0	0	12	0	0
a8e275416aabe32c74616d9d5ada6f0d	0	0	0	0	0	0	5	0	0	0	0	0
621cbf8ab10c8eb72f74ff7dad3fdd7d	0	0	0	0	0	0	0	11	0	0	0	0
c4481ec2b91bbdf61620ac0eff851ca8	0	0	2	0	0	0	0	0	0	0	0	0
545ec3fc626530d1c2ca32114e955520	0	4	0	0	0	0	0	0	0	0	0	0
aba0142e7ad13b859c026d6a8fd8efca	0	0	0	0	0	0	0	0	0	0	32	0
1d371c540299a4dbb4b025dfb33ba5cc	3	0	0	0	0	0	0	0	0	0	0	0
fc36a54443c3fb53da172dede334f91d	0	0	0	0	0	0	0	0	0	0	4	0
ff4fa0b1b17e30e1a2c8468ae40a9021	0	0	0	0	0	10	0	0	0	0	0	0
210b37ec18c02af75c3921ae46f4f350	0	0	0	2	0	0	0	0	0	0	0	0
452aa9dbc2e58f033e6b11d31411bc6c	0	0	0	0	0	0	0	16	0	0	0	0
a04cb3d52f3d5d43af4798a584cc53b4	0	0	4	0	0	0	0	0	0	0	0	0
8b0e7e2d55ab3d601c60f5ac04d082e5	0	0	0	0	0	0	0	0	0	8	0	0

5d150be7b359c601e3b1dd330ac471f2	0	0	0	0	0	0	0	0	0	12	0	0
2ebc5b206fbfa9591c6b14a244fc1269	0	0	0	0	0	0	0	72	0	0	0	0
32c89151d6edb177cda693ff7a928e54	0	0	0	0	0	0	0	0	0	0	44	0
f7fa0a6c54ad70d287bb7b1e35599988	0	0	0	0	0	0	0	0	132	0	0	0
778110749af304c4fd108112583b4a99	0	0	0	0	0	0	0	0	0	57	0	0
ca6be25d6d9c4e144887111b30c4dc04	0	0	0	0	0	0	0	0	0	0	3	0
cdc67052eb7c12839edf8ae82a943ca9	0	0	0	8	0	0	0	0	0	0	0	0
f3781f1889b1af93c1fe67c70c38131f	0	0	0	0	0	0	0	0	0	0	0	5
8ed6a59a4044433f6391aab9c676a6da	0	0	0	6	0	0	0	0	0	0	0	0
bc04551535a78da2a91604729a3abf5c	0	0	0	0	0	0	0	0	17	0	0	0
7079e23dfcbb6211ce1fcc0fa4ccc158	0	2	0	0	0	0	0	0	0	0	0	0
d0c9125e9c06d9f5e7edff9aae388e6f	0	0	0	0	0	0	0	0	0	0	15	0
34209dba0bb85f79403d9d4ffb349ce6	0	0	6	0	0	0	0	0	0	0	0	0
0f6d981601555945756ae9078ae83a5d	0	0	0	0	0	13	0	0	0	0	0	0

d8deb949639d16c0ec40237 2fcb9b55b	104	0	0	0	151	214	0	133	174	329	0	0
c0cba8aedbee37051e4da36e 9a0aa9a7	0	0	0	0	0	2	0	0	0	0	0	0
8231caf82052a978099d912b 3322c908	0	0	0	0	0	0	0	0	4	0	0	0
b832359cf33d2d803145b76e 148de096	0	6	0	0	0	0	0	0	0	0	0	0
dc8197770d2685899f825c6b 0771134a	0	0	0	0	0	0	0	0	0	4	0	0
750e17f1b94b894988dc651e e807e8a3	0	0	0	0	0	0	0	0	0	0	0	5
0ce14a5980e9e7314d9e520f afd838a5	0	0	0	0	0	0	0	0	0	4	0	0
47c8fe1b85827600257a7c3e e06197df	0	0	0	0	0	0	0	8	0	0	0	0
77784439a217b6d9a224d76f ec71e8d0	0	4	0	0	0	0	0	0	0	0	0	0
9b401f442950b255ddc231e5 46dd6458	0	0	0	0	0	0	0	0	0	0	2	0
2385afafd69b489b963bf5d0 72355f46	0	0	0	0	0	0	0	0	0	0	11	0
35e7b86b812f60f8589e697c 27f4dce2	5	0	0	0	0	0	0	0	0	0	0	0
a8909f5d35c35921f226b983 908f0eb7	0	0	0	0	0	0	3	0	0	0	0	0
414c4afeec9d01f1fc5332c61 15a6093	0	0	0	0	0	0	4	0	0	0	0	0

e1f93a77777ba6ec83e41d13df3de8f5	0	0	0	0	2	0	0	0	0	0	0	0
a6240a61deb90dec0a078bdbadf06a7d	0	0	0	0	0	0	0	0	0	3	0	0
41d3ef9215835d8bdfdf9f711d3f83dc	0	0	0	0	0	0	0	0	0	0	3	0
a38e9e98e6ca6521c9234c09abea6b97	0	0	0	0	0	0	0	0	0	183	0	0
3a9ef8e33f768b7a35825d1f66a69489	0	0	0	0	0	0	0	0	0	2	0	0
9f0d066c0cda10f81f7de96b0c5ac281	0	0	0	0	0	0	23	0	0	0	0	0
ba6fed80fccfe0a21a1d8d758a0c91c9	0	0	0	0	0	0	0	0	3	0	0	0
47cb8083a0cd29c89229de0fb62c4cb4	0	0	0	0	0	0	5	0	0	0	0	0
5cba0b3fc7c5a06d8509e64f8a4bade4	0	3	0	0	0	0	0	0	0	0	0	0
7211752dd0fc1a9cf2e49f640a2e11c3	0	0	0	0	0	0	0	0	2	0	0	0
3f401252e911586cd7f9f0b492d4c708	0	0	0	0	4	0	0	0	0	0	0	0
ca14be5197cd8f431f4ac09875e224d1	25	0	0	0	0	0	35	0	0	116	0	0
67a0fc9f2b86684cc84e78caaa94da09	0	0	7	0	0	0	0	0	0	0	0	0
7aa1b3304d3e6b227a8e91a9dea676de	0	0	2	0	0	0	0	0	0	0	0	0

761381b4f853c99c4800484d4e9314a2	0	0	0	0	0	0	0	4	0	0	0	0
809101817bf68fe3f0af141fb3a40121	0	0	0	0	0	0	0	0	2	0	0	0
7abc1c51992f8863296b6c5ea4f5af4b	0	0	0	0	0	0	4	0	0	0	0	0
d023882b1036c65a005bc4b709c68187	0	0	0	0	0	0	0	0	0	0	0	13
4cf93ffab9e23fd5440ea6c215409dfd	0	0	0	0	0	0	0	0	0	8	0	0
d8e79621b4d39df79ccd408151377728	0	27	0	0	0	0	0	0	0	0	0	0
9ec885765ab2386673b032ac0be33485	0	0	79	0	0	0	0	95	0	0	0	0
b73c3bbe25af170434cbf564b56a228c	0	0	0	0	0	0	0	0	0	0	4	0
7154ce4466d643dbf895cbe488486d54	0	0	0	0	0	0	0	0	10	0	0	0
7f7faaf30e9248a71cad6bb1e7a500c3	0	0	0	0	3	0	0	0	0	0	0	0
c5b46dd4de9877fe9d346d5e5cec09d2	0	0	0	0	0	0	0	0	0	0	2	0
d8dcb4cb2c5f5ac399d67d3b47606fa8	0	0	4	0	0	0	0	0	0	0	0	0
3e7796a93ac4d8602fbc79f67d3222ca	0	5	0	0	0	0	0	0	0	0	0	0
1028f353a5f0359455d420626c105f9a	0	0	0	0	0	0	0	2	0	0	0	0

039ac622bccb53718a7c0c681dad52c6	0	0	0	0	0	36	0	0	0	0	0	0
a0f25d791f192613b01bf60ffbc3a94c	0	0	0	0	0	0	0	4	0	0	0	0
999f61144e5b8d1bb6aa6dddeedea231	0	0	0	0	5	0	0	0	0	0	0	0
a1c58d71e4da2f6f3dccfe9e99bbf70f	0	0	0	0	0	8	0	0	0	0	0	0
6d9c49bbdcfd42bf0f1e45ebb6b9a17c	0	0	0	0	0	0	0	0	0	0	560	0
9e619a929be7be59586871e62094d9d7	0	0	0	0	0	0	0	0	0	0	0	7
f123d1a69c88244847b509456225f1ea	0	0	0	0	0	2	0	0	0	0	0	0
d764cffac5068c0ad372e73a2e7a6edd	0	3	0	0	0	0	0	0	0	0	0	0
926bee65a1e2ff539f4b49f459868d43	0	0	0	0	0	3	0	0	0	0	0	0
5288ff28ab890ec95e1fb13d2af947db	0	0	0	0	0	0	0	0	0	3	0	0
4b54c860c53eeb900778507e2ca91967	0	0	0	0	0	0	0	0	0	0	2	0
ab827aabfdf15d4332da355d4d7a251f	0	0	0	0	0	0	0	0	0	0	7	0
5fa885ec1117a92397373f174cf8137b	0	0	0	0	0	0	0	0	0	0	0	3
65210a20315d8b7a661f0c5288a622ba	0	0	2	0	0	0	0	0	0	0	0	0

3bb7d77cb5195afd892b8b4205c439fd	0	0	0	0	0	0	0	0	0	0	211	0
cda13c5cd59ff4c43fe5168f94ae83b7	0	0	0	0	0	0	0	0	0	0	0	4
0f436f51989b34f4ecba4e9cac209263	0	0	0	0	0	2	0	0	0	0	0	0
2aaf9548981dd76f5effc48fd2617f16	0	0	0	3	0	0	0	0	0	0	0	0
6bee0b915e06c212e1b5a72df232d7e9	0	0	7	0	0	0	0	0	0	0	0	0
f2d49a0f7461a588d50fefcf365b25d4	0	0	0	0	0	0	0	0	0	0	0	3
b17c37a0ffbd69caa1c3b47d83daa4cd	0	0	0	0	0	0	0	0	0	0	0	2
29702c793f9710c9bea5003ef6d26f92	0	0	0	7	0	0	0	0	0	0	0	0
6feb020bed3fca347a3de5061ff17df6	0	0	0	2	0	0	0	0	0	0	0	0
e4efd3c1f338eae32728579ae2af19b2	0	0	0	0	0	31	0	0	0	0	0	0
8bd81e503199942c1f268c746f6df3f3	18	0	0	0	0	0	0	0	0	0	0	0
132fb9c1f09ca67cb690f8a198447576	0	0	0	12	0	0	0	0	0	0	0	0
551f69d2ff20e8d6ad76d581283b00ee	0	0	0	0	2	0	0	0	0	0	0	0
2624eb8e3eb2d45dc8dd9380b87dde99	0	0	0	0	0	0	0	0	3	0	0	0

b74ac2771fe4c3124443a7498c821131	0	0	0	0	13	0	0	0	0	0	0	0
5231ddcc879ac22df261c1540939e9f1	0	0	0	0	0	0	0	0	0	0	0	6
d9e546bb246e74214c52a6dda742f472	0	0	0	0	0	0	0	0	0	0	0	24
ff4fe7b87c394b2a52af885adc20ee1d	0	0	0	0	0	0	0	8	0	0	0	0
94adba62bed2fd9f7b185cce674dd857	0	0	0	0	0	0	0	8	0	0	0	0
6df64c49f30592ded1da2ee4529145c3	0	0	0	0	0	0	0	0	2	0	0	0
ef4376e37e6f3e38833b88b77b68de61	0	0	0	0	0	0	0	5	0	0	0	0
bacac93d4f0b2be31747956c5b11d956	0	0	0	3	0	0	0	0	0	0	0	0
6a567c341ab77e4d0058f33756dc46fd	0	0	0	0	0	0	0	0	0	0	11	0
a1129ae0d1c9dbfe99205b7f6dfc5500	0	0	0	0	0	0	2	0	0	0	0	0
a0a9e7af49c3628ad9a1eeffe291e0e3	0	8	0	0	0	0	0	0	0	0	0	0
6b54f3bcafef9df8669473d166a6cd23	0	0	0	0	0	0	0	0	0	0	83	0
6bdab24e99e53ad48bc4430070553c18	0	0	0	0	0	0	0	0	0	7	0	0
4ff19a544fdceeb9a984d79c8bdb891c	0	0	0	0	7	0	0	0	0	0	0	0

91e8d93cd2d0e5a3e15e6f82 2a3817e7	4	0	0	0	0	0	0	0	0	0	0	0
1a4a5754e6b2526ddc56a67 33be01f9e	0	0	0	0	0	0	0	0	0	4	0	0
f651966bc3c2a26958ee78e3 44a227f8	0	0	0	0	10	0	0	0	0	0	0	0
fe95fcdb99f7fe948c5e01f2cf e944d3	0	0	0	0	0	0	0	0	0	0	2	0
6417e7c7c9eacfc68a68ccc19 2ac982f	0	0	0	16	0	0	0	0	0	0	0	0
c87acf7d04cba2c4ec972884 dae0d440	0	0	6	0	0	0	0	0	0	0	0	0
984116bf3a7fa771a3125024 35f0e3f4	0	0	0	11	0	0	0	0	0	0	0	0
d5065674639ad7d64c43dd8 01a545d5a	0	0	0	0	0	0	0	0	0	0	3	0
2cab5c231c72b8d92f255791 194e629d	0	0	54	0	0	0	0	0	0	0	0	0
ec91ea97ab24e1c6b6e907d0 0a6882ac	0	0	5	0	0	0	0	0	0	0	0	0
ec38e9980e1bb40f6834fa11 9aeacf82	0	0	0	0	0	5	0	0	0	0	0	0
da112e7ef94dfbdfc596d631c a27a3ce	0	0	0	2	0	0	0	0	0	0	0	0
25580a29c93853a316e7c1d1 075b041d	0	0	0	0	0	0	0	19	0	0	0	0
0d9f46fd9750fb9671a6c4dc6 9410691	0	0	0	0	0	0	6	0	0	0	0	0

1e36d5dc3c8fcc94b0e129935884c87f	0	0	0	5	0	0	0	0	0	0	0	0
36aaefd220c9708b251bdf8dbb85daa2	0	0	0	9	0	0	0	0	0	0	0	0
d869081a8c65040c7c9a258609b4f01b	0	0	0	0	0	45	0	0	0	0	0	0
814a6690dc2d76edb2942b893e87ca8e	0	0	0	0	0	3	0	0	0	0	0	0
4929609dea707d98dac109d8c24eb36f	0	0	0	0	0	0	0	0	0	0	0	7
2985c16cb3b71c844ebdf247944314e1	0	0	0	0	0	0	0	0	0	2	0	0
c9f0a34541b877ea3de220a29ada8d9c	0	0	0	0	0	0	0	0	0	0	0	4
68c2bf71d3bdae74a18291e6244a5ee0	0	0	0	0	0	0	2	0	0	0	0	0
4505654da28a9892ce03b19baeb9a0b3	0	0	0	0	0	0	0	0	0	0	2	0
d3239d41da5c18ac19f8a7d07cf69192	0	0	0	0	0	0	0	0	0	0	0	5
b96051ba3a050f079c4ef929e751af76	0	0	0	0	0	0	0	0	3	0	0	0
5724ef268208d7af54b9d3103b53a335	0	141	0	0	0	0	0	0	0	0	0	0
2defcbd0336ce0a29c8cbba67f20ef65	0	0	0	0	0	0	0	0	0	0	25	0
3dcd1a2cf28f88e215e6b62493315096	0	0	0	0	0	0	0	10	0	0	0	0

6ac6b321451e974416524e5 0139d10d2	0	0	0	4	0	0	0	0	0	0	0	0
571b4575f9143dcd0a0fd332 37b8ab38	0	0	0	0	0	8	0	0	0	0	0	0
910e2f6bfa95a6012c6e2a72 45f441fa	0	0	0	0	0	0	0	0	0	0	5	0
9cbd3bb57c25035b7fe35013 0165bd16	0	0	0	0	0	2	0	0	0	0	0	0
4df8157dfa78d1ee2db5e99f 9e8d6590	0	0	2	0	0	0	0	0	0	0	0	0
61e2cd358768880840464e0 4665c88a3	0	0	2	0	0	0	0	0	0	0	0	0
116081e09fe745a4e75c9e86 c19af9e2	0	0	0	0	0	0	0	0	2	0	0	0
903e9be9a09a995608b9ca8 e05d4a818	0	0	0	0	30	0	0	0	0	0	0	0
39c40c232e02c1d23b44dce8 cab44fbc	0	0	0	0	0	0	0	0	37	0	0	0
07cc2e265ca140f093e3d161 9b556e46	0	0	0	0	0	0	0	0	0	0	2	0
5ac05765fec9d4724fde9a3d 08df5288	2	0	0	0	0	0	0	0	0	0	0	0
03aa58c0f2cceb2c4aedfc4a5 4edaafb	0	0	0	0	0	0	0	11	0	0	0	0
c42825914c1a7f6f26f6840b5 c8d5faf	0	0	0	0	0	0	4	0	0	0	0	0
f66abb9a5cc0cf6162807ae18 f79e91c	0	3	0	0	0	0	0	0	0	0	0	0

89b6cf6ace83f4f1311169d6f a4135f7	0	110	0	0	0	0	0	0	0	0	0	0
dd7f0e990d4b9309487b7a7 8217ea9e0	0	0	0	0	0	0	0	0	0	3	0	0
70261641b05b2e167c17cc1f d00d9602	0	0	0	0	0	0	0	0	0	0	0	72
9a23a4fa1dfdb9d48f464279 6e1558e9	0	0	0	2	0	0	0	0	0	0	0	0
28dabebfbc8c51a5f13ba7f16 2e1a829	0	0	0	0	0	0	0	0	0	2	0	0
3763938af42898f692560c5cf b954487	0	0	0	0	0	0	0	0	0	0	3	0
5812b3d2fe46463002baa211 18643b49	0	5	0	0	0	0	0	0	0	0	0	0
51a50314f57020243c1b748d 48a13d66	0	0	3	0	0	0	0	0	0	0	0	0
ab0351e9e81295a2c3971e0f 64ab00b9	2	0	0	0	0	0	0	0	0	0	0	0
7ee43d214f34e0ecd6bde454 c20ebf18	0	0	0	0	0	0	0	0	0	0	0	39
46e8d1b5267cd1ce4235431 3d0bada40	0	0	0	0	0	0	0	0	0	4	0	0
b0884038ec3b0928226b942 5569e59a5	0	0	0	0	0	0	0	0	0	0	4	0
7982398d2b4823785e0fdced 313f7a87	0	0	0	0	0	0	0	0	0	0	0	16
24c7c5374474155d6ce8431a 9a2b7ac3	13	0	0	0	0	0	0	0	0	0	0	0

429181385c15cc22414f36b814a23e17	0	0	0	0	0	0	0	0	0	3	0	0
517d208168b5f931f221eb5896906744	0	0	0	0	0	0	0	0	5	0	0	0
1649acc9166107c25f4b43b90d65fd09	0	0	7	0	0	0	0	0	0	0	0	0
f42d6e3a6dc95ada6d5adb90fc6eb487	0	0	0	0	0	0	0	0	0	0	0	7
9460e7bb947711e703f19c316593974d	0	0	0	0	0	2	0	0	0	0	0	0
b63c5fa3f43cfdbf08e88f1d663cebfc	0	0	0	0	0	98	0	0	0	0	0	0
1bdab99e8ca68a097d6f421be3fddebb	0	0	0	0	0	0	0	0	0	6	0	0
eadbf85b821256d75b687032676f0b1a	0	0	0	0	0	2	0	0	0	0	0	0
900e8807cbba24d6e418c6ea17dc1331	0	0	0	0	0	0	0	0	0	0	4	0
321766d73a9b97ccf85f8d7107227518	0	0	0	2	0	0	0	0	0	0	0	0
d767ac197ebee3a705e9e116702e72eb	0	0	0	0	0	0	0	0	0	0	4	0
efa8c8a10d15fb381ed0d4c6bf312eb8	0	0	0	0	6	0	0	0	0	0	0	0
50cd624e7267573cc28385cff078b18b	0	0	0	0	0	0	0	0	0	4	0	0
627c47b5a78e5b475813d6439cdcafee	0	0	0	0	0	0	0	3	0	0	0	0

13dded89b55ada1bf26f0fea97dd938d	0	0	0	0	0	0	3	0	0	0	0	0
10fd696dec7e69a71bdee3ea06485bad	0	0	0	0	0	0	0	0	0	0	0	2
e967e700c516645ff442c12013839744	0	6	0	0	0	0	0	0	0	0	0	0
257d88eece2f1c325972e828af60dd30	0	0	0	0	0	0	0	145	0	0	0	0
8f5593a6bafdc324c2d3bddcf4d52091	0	0	0	0	0	0	0	2	0	0	0	0
ff998a848446e48acfaf68c02c630896	0	0	0	0	0	0	0	0	0	0	0	24
fd80900cf6262ebef676643ff5142e1e	9	0	0	0	0	0	0	0	0	0	0	0
1a0a3eba194b567b72b2ad4e79fb0dfd	0	0	0	0	0	0	0	0	0	0	2	0
e9c4c550ef802d4cd8a739872d9304ae	0	0	0	0	0	0	0	0	0	63	0	0
a646b0532e9d05133099fd432f2daf93	0	0	0	0	0	0	0	5	0	0	0	0
7a04aeaf6cbe5a97854813a77ba9e0f6	0	0	0	0	0	0	0	0	2	0	0	0
d4339ae05a856d0a330ead48678ae951	0	0	37	0	0	0	0	0	0	0	0	0
8e467166eb3f6010c3f0dd7141c72a3a	0	0	0	0	0	0	0	0	0	26	0	0
05d2c4287652ceb06d8e089161d1241b	0	0	0	0	0	3	0	0	0	0	0	0

88bffe7e4c27ec6581621241 038316de	0	0	0	0	0	4	0	0	0	0	0	0
3f2d32cb9f0ab3dd86f748d2 da5ee4d3	0	0	0	0	0	0	0	0	0	9	0	0
e288a3e9c39f666fe1c649d4 47c1f0da	0	0	0	0	0	0	0	2	0	0	0	0
1cb4cc3b2d37c8076cb04266 dd7a61f3	0	0	0	0	0	0	23	0	0	0	0	0
5568d3fbe60d14194926d57 8a9f3a749	0	0	0	0	3	0	0	0	0	0	0	0
4b3f1229957771c9d4f625ad 846d0608	0	0	0	0	0	3	0	0	0	0	0	0
3b97afb3001f334e438f021f6 ecbcf8d	0	0	0	0	0	4	0	0	0	0	0	0
73b83340a0a3ad49cac4860a 490da7d0	0	0	0	0	0	4	0	0	0	0	0	0
a63725776df870d2670b12c2 7d3107e0	0	0	0	4	0	0	0	0	0	0	0	0
d91e9fdad3f0a3ee4472e934 21180269	0	0	0	0	2	0	0	0	0	0	0	0
65cdfed3115ff33ca91e462e7 91b96c2	0	0	0	0	0	0	0	0	0	0	77	0
c88ac2095be1358e3e9f1702 4ef31cfa	0	0	0	0	0	13	0	0	0	0	0	0
973780f3501e72c87d8003c1 769149df	0	0	0	0	0	0	0	0	0	0	6	0
f29af515bd654ebaf7443d52 dad4d42c	0	0	0	0	0	0	0	4	0	0	0	0

68f085e633c150a8a10f8f8fff c2c051	0	0	0	0	18	0	0	0	0	0	0	0
c6c7ed949af62169b4014928 aca3b1d6	0	0	0	0	0	0	0	0	10	0	0	0
d9f79699dfea8c67546f14e95 28b146f	0	0	9	0	0	0	0	0	0	0	0	0
4f2a30df07fedbf34055cd4af 6024e3e	0	0	0	11	0	0	0	0	0	0	0	0
191060bb44db87d47bbfed3 70f23a651	0	0	0	0	0	0	0	0	0	0	4	0
f456c061814b4bacc6a0e90b cada0c65	0	0	0	0	0	0	0	0	0	0	34	0
84d1a3d9cdc1a6ffe7081c247 1b16a39	0	0	0	0	0	0	0	4	0	0	0	0
ca730a3339b74d33618e40d e8894d46c	0	0	0	0	0	0	0	0	0	0	4	0
fdded3dac8afd8543c7657f78 3d05844	0	0	21	0	0	0	0	0	0	0	0	0
aef0f414788139d4038d7a77 62773db9	0	0	0	4	0	0	0	0	0	0	0	0
150946f262ae4cb081e33630 fe5cacf5	0	0	0	0	0	0	0	0	0	9	0	0
3e0e0d133caefa5a91bdbee6 03e2a563	0	0	0	3	0	0	0	0	0	0	0	0
601bb1cb4467e7adcc6c9517 02876d81	0	0	0	0	0	0	0	0	0	0	8	0
9d5afc33b61ba66085d09095 e5990b06	0	0	0	32	0	0	0	0	0	0	0	0

98454651c59223e2487dd8b8eb03c2df	0	0	0	0	0	0	0	0	0	0	13	0
8db145cb498977b7418be4f03b998077	103	0	0	0	187	0	0	300	0	81	0	0
611a46fa33994d13446341808ac1b411	0	0	0	0	0	0	0	0	0	4	0	0
fce35ef79f7761828ba904c7aff6ef28	0	0	0	0	0	0	0	0	0	0	2	0
b882acf46766963e53fdda7cea3a9f46	0	0	0	0	0	0	0	0	0	0	0	5
326cfcc2607a662e04db362f4ae34b99	2	0	0	0	0	0	0	0	0	0	0	0
f27c843bee9c0a73a05adc3d568401c4	0	0	0	0	0	0	0	0	6	0	0	0
fb8c02c4bcd38c750830562c1ba58648	0	0	0	0	0	0	0	0	0	3	0	0
ef8c1b87a5f5fe1389bc3b8f270664ea	0	0	0	0	0	0	0	0	0	0	3	0
3f4cadf8e6735cf5756108538312847b	0	0	0	0	4	0	0	0	0	0	0	0
41890acc8f8d7bb3a3b37d234d8db428	5	0	0	0	0	0	0	0	0	0	0	0
96dc629f49077c0624100575a6b8215b	0	0	0	0	0	0	0	8	0	0	0	0
fc3251eedcdc8cf22507598948eefafc	0	0	0	0	0	0	0	0	0	0	2	0
587fee4406b7e839fac684c3cbf39869	12	0	0	0	0	0	0	0	0	0	0	0

063463975d5770e76b456e66587dc754	0	0	0	0	0	0	0	2	0	0	0	0
7b6237e435b3fcc0a6b7cca4b48a6f7f	0	0	0	5	0	0	0	0	0	0	0	0
101429b07087255285d26cd0c26d74f3	0	0	0	0	0	0	0	0	0	0	126	0
d9b63c0e0f317d31da05c3f86a498a76	0	2	0	0	0	0	0	0	0	0	0	0
92c23f2d0dcaeef82d9d0c2a2875bf5c	7	0	0	0	0	0	0	0	0	0	0	0
4bc21826004d80873adaf967a1719cc0	0	0	0	0	0	0	0	0	0	0	5	0
e5cda6a94f548d4b7e334bfc69130e6a	110	0	0	0	0	0	0	0	0	0	0	0
a31a67de823b9714abceb7e000a598de	0	0	0	0	0	0	0	6	0	0	0	0
8907d688e1833402b6fd7060b048783a	0	0	0	0	0	0	0	0	6	0	0	0
c3c729e6d92ceb71174812f3d5be1657	0	0	0	0	0	0	0	0	4	0	0	0
5c6c173751f8f8c0b747499b24ec59c3	0	10	0	0	0	0	0	0	0	0	0	0
74352aea104a9091d116faf679d7b1cf	0	0	0	0	0	0	0	0	0	0	0	6
4b6295f8a449abbce05934768205033b	0	0	0	0	0	0	0	0	0	0	0	62
bf38f1ba9dec051a2ffe5b6c807e1564	20	0	0	0	0	0	0	0	0	0	0	0

6fc399ccf6dd3bc26504ea2c94c327ab	0	0	0	0	0	0	0	131	0	0	149	0
e178366cd77f15ef017a3c570ac35c27	0	0	0	0	0	62	0	0	0	0	0	0
34a6b33f35188364a8aa26e0f7fea5d5	0	0	0	0	0	0	0	0	0	0	3	0
8c9478bf57efe50362e58b37f0768bf9	0	0	0	0	0	0	0	0	0	23	0	0
923992a9ef0c26e70ec2edc5c1ccde9b	0	0	0	0	0	0	12	0	0	0	0	0
51e2291228c5481c6ba088b1e037fc7b	0	0	0	0	0	2	0	0	0	0	0	0
870603cf6cacbb9edc3f36f45f99e35b	0	0	0	0	0	0	0	0	0	0	107	0
a0e486fe08e9775e9c60985b38ff4a02	0	0	0	0	0	0	0	0	0	0	0	102
5ac9485eb62a48fd34c54079f0fd9bfe	0	0	0	0	0	0	0	5	0	0	0	0
721836366d88abc489af06c911bec7aa	0	0	0	0	0	0	0	0	0	0	3	0
7ab18e90014f62bbfd884e8d22ff852b	0	0	0	0	0	0	0	0	0	0	0	2
2dfd3b2604277e6b5fe09db6e7f5993e	0	0	0	0	0	4	0	0	0	0	0	0
f0ce3bedff6498f42c7d4325df13ed3d	0	0	26	0	0	0	0	0	0	0	0	0
e71d0dfec72495af9aae6a4d496487d8	0	0	0	0	0	0	0	0	4	0	0	0

106dd7a43db0542a6803a34547880f83	0	0	0	0	0	0	0	0	0	0	0	2
c193a18820b03006f159b91e34b9b763	0	0	0	0	0	0	0	5	0	0	0	0
fd3665297ad5543bc15a8aff233ed478	0	0	0	29	0	0	0	0	0	0	0	0
43865507b6260f6f1b786e233b3aaeed	0	0	0	0	2	0	0	0	0	0	0	0
71681c550652fa66cc42053268441a82	0	0	10	0	0	0	0	0	0	0	0	0
c280c28a2fa732a4ebecc1141fd1ca64	0	0	0	0	0	47	0	0	0	0	0	0
4269ed22099edbe70c1fae98fc3f93f0	0	0	0	0	0	0	0	0	0	0	3	0
99e19d55da3e902f2f4b4ec407e9c759	0	0	0	11	0	0	0	0	0	0	0	0
e726168d7cbcd72b1e734bfbe360dd58	0	0	0	0	0	0	0	0	0	0	3	0
8fa35a03c95cb6c139aa80d586251bf9	0	0	0	0	0	3	0	0	0	0	0	0
1546e5ab04fb433ac96ba784f8be0343	0	0	0	0	0	0	0	0	0	0	3	0
7e4cbef950ada68162d6b8422ea7b7dd	8	0	0	0	0	0	0	0	0	0	0	0
24e5635a99e567be6fbc1c948aa5d1f3	0	0	2	0	0	0	0	0	0	0	0	0
482f09f3ead6b86882ec20cb8512365a	0	0	0	0	0	0	0	0	0	0	11	0

9c5b8de66317f566c3178f81a8a4b7c2	0	0	0	0	0	0	0	0	0	0	0	5
1761164e65d5baec3794d1038458b006	0	0	0	0	6	0	0	0	0	0	0	0
65cb7fecbc868792e55a82e12eac917c	0	0	0	0	0	0	0	0	0	0	2	0
a5da379ddb5f060d7413b71ff0ea97bf	0	0	0	0	0	0	0	0	0	2	0	0
a1f73885dedccfbbc45934bc6c542598	0	0	0	0	0	0	0	0	0	0	0	6
243afea7ff0548967826394a07160b68	0	0	0	0	0	0	0	0	0	0	4	0
01dabf0afbc9331ee7de92c8e157052b	0	0	0	0	106	111	0	48	0	0	251	92
c340c93850af48cf225dc2d715f00c50	0	0	0	0	0	0	0	0	0	0	0	87
dc5cbe86c9b7dd6140ad5d60dd35af1b	0	0	0	0	0	0	0	0	0	2	0	0
ab692e0f240cf5ad5d5902380ef6fd49	0	0	0	0	0	0	0	0	0	0	0	3
bfd61681cd82809c99d11276cb36c00e	0	0	0	0	0	0	0	0	0	0	7	0
1a2386db4557425f6d00e65792f83167	4	0	0	0	0	0	0	0	0	0	0	0
ba01cb3c436ce2d773d7ee0c1d7a5be2	0	0	0	0	0	0	0	0	0	0	53	0
d1ebc9730fda989005affaa33d080035	0	0	0	0	0	0	0	0	0	8	0	0

26f027fa8a96bc7dd99879322ae74b51	0	0	0	0	0	0	0	3	0	0	0	0
5454ab5b93510ea8475612482ec0b34f	0	0	0	0	0	0	0	5	0	0	0	0
7a3552a3b2191b689f4f6c566987b0f3	0	0	0	0	0	40	0	0	0	0	0	0
f859e2b99d4519f90066fd2ff44316e3	0	0	0	0	2	0	0	0	0	0	0	0
a72fb3275b2a629eafde5197d6861c93	0	0	0	0	0	7	0	0	0	0	0	0
4851ca3c248cf3e4e8d6182332df1f87	0	0	0	0	0	5	0	0	0	0	0	0
fa06ef7df2e42c3c39b7718090f5b0a9	0	0	0	0	0	8	0	0	0	0	0	0
c1d0b0778b4de5ab664cdfd41d58e563	0	0	0	0	0	0	0	0	0	0	0	3
702989a65710c539bd710971258b14d8	0	0	0	0	0	0	0	0	0	0	36	0
b6083de8de75d624a6c9b3154b793ff5	0	0	0	0	0	0	0	2	0	0	0	0
e3a1feb6e4366e97e94c89e0911e3921	0	0	0	0	0	0	0	0	0	2	0	0
452e278e09a98b91316770c1c31bd1c8	0	0	19	0	0	0	0	0	0	0	0	0
21c0be79ba7c01cb67f707b16ef26651	0	0	0	0	0	0	29	0	0	0	0	0
ac95aec29f19ba297abcb06ee641e71e	0	0	0	0	0	3	0	0	0	0	0	0

250c95c14ca6dc19df9cf3863 7448ac5	0	0	0	2	0	0	0	0	0	0	0	0
f44462f039383280a4927da7 82209bec	0	0	0	0	0	0	0	0	0	0	0	17
594d9b90e6f8cffa6991f80ad 5f3fbb5	3	0	0	0	0	0	0	0	0	0	0	0
f7e2d653e155591da42fb458 12fccbeb	0	0	0	0	0	0	5	0	0	0	0	0
085c457af190f67b30810e04 1f1d9ed0	0	0	0	9	0	0	0	0	0	0	0	0
98bb4fb6cbb7af78cbc603ddf ef9d539	0	0	0	0	0	0	0	0	0	0	9	0
f3df1cc29b07c665eda0223d a220a444	0	0	0	0	0	0	0	0	0	0	0	14
c06d66a5102366fde0d3c1d0 46b02375	2	0	0	0	0	0	0	0	0	0	0	0
328f778dc1fe43444449379e 5990accd	0	0	0	0	0	0	0	0	0	0	13	0
dc73968b9654d1635b36bb6 278cf2d08	0	0	0	0	27	0	0	0	0	0	0	0
36ad884ad77812d5189a800 5cda2e5bd	0	0	0	0	0	0	0	0	0	2	0	0
3fd7688e5a9853c6da21a4ac ac79e839	0	0	0	0	0	0	0	0	0	0	2	0
c4b7a70413ea3ce2f2bfdd19 ecaa0e1c	0	0	0	0	0	4	0	0	0	0	0	0
5f3f8d7c9d49f272872573d6 81723f60	0	0	0	0	0	0	0	2	0	0	0	0

d60d916435454498a204fdfd22484f22	0	0	0	15	0	0	0	0	0	0	0	0
73c9f96122f96cf6cb840e1a5f454c9b	0	0	0	0	0	0	0	43	0	0	0	0
30ec3da383c8416fad1d80129578f814	0	0	0	0	28	0	0	0	0	0	0	0
6f8d4949f5b734a130c52082c2972515	0	0	0	5	0	0	0	0	0	0	0	0
c72ee041ce00e4653c4a44dddfdfc151	0	0	0	0	0	0	0	0	0	0	14	0
e15bc21cf31b2ebaaf3b57d94273d96f	0	0	0	0	5	0	0	0	0	0	0	0
be29b7d76e101a26ff0c7e26dfbc58c6	0	0	0	0	25	0	0	0	0	0	0	0
2b4ce43149debd80ee752e8d39490e71	0	0	0	0	0	0	0	0	0	0	18	0
eda3ae5556c19ae19ccc5f91ea1d712d	0	0	0	0	0	0	0	0	0	0	2	0
66e648f5a68149ab85674d92d9bc6ac8	0	0	0	0	14	0	0	0	0	0	0	0
2dcbb25075015a4e823564f54768e3db	0	0	0	0	0	0	0	0	0	0	6	0
3cd2ac8b866e7ddbfb1c8b40fd918381	0	0	0	0	0	4	0	0	0	0	0	0
709778bf175b66202a886de2093ba6d0	0	0	0	0	0	2	0	0	0	0	0	0
110c5162238ad895443b5eaafc8894ff	0	0	0	0	0	0	0	0	2	0	0	0

f8536f12a11cee00eb5c3b96bd2ce935	0	0	0	0	0	0	0	0	0	0	7	0
efa683ae5aa3ce7e476d05591a55ed97	2	0	0	0	0	0	0	0	0	0	0	0
ceeac9d183ac38e79a4660204dba253a	0	0	0	0	0	0	0	0	0	7	0	0
87fa22081a2c9240ce5635d07c05a446	0	0	0	0	0	3	0	0	0	0	0	0
4199a1556307620488aec69e111db57d	0	0	0	0	0	0	0	8	0	0	0	0
a82a99bbf0c3149f32347133f2283990	0	0	77	0	0	0	0	0	0	0	0	0
4f1c19e87746b9ed2087a5412d992379	0	0	0	0	0	0	0	0	2	0	0	0
eda9de62921e24eb1cf0a6c4fa9e088e	0	0	0	0	0	0	0	0	0	0	0	6
d8981bf2e05dfef47f00ae183a61242a	0	0	0	0	0	0	0	0	0	0	3	0
7ad21f2e11c81db793eddafacbd880cc	0	0	0	0	0	7	0	0	0	0	0	0
6f50d548956c4fd816842fbce3c8eb2d	0	0	0	0	0	0	0	0	0	0	0	8
4e73f3ecd97a43ac3c2b32c2565fdc5a	0	0	0	0	0	13	0	0	0	0	0	0
53904ed63586378e627c108cf1c6bc8c	0	0	0	0	0	0	2	0	0	0	0	0
c38a73e840fcf977061f3f35448fea73	0	4	0	0	0	0	0	0	0	0	0	0

b8f921085bdd6c300e611f232e81b2e0	0	0	0	0	0	0	0	73	0	0	0	0
552ff013602de9b117eb6894985580ee	0	0	0	0	0	0	0	0	0	0	0	3
7791abf288e394ecaa0e9f64ecd2a16b	0	0	0	0	0	42	0	0	0	0	0	0
d777457a48b155fbcda28f2ccf6dc515	0	0	0	0	0	0	0	0	0	0	11	0
c29e8cc0f994284ec2cc4823186d04dd	0	0	0	0	0	0	0	0	0	0	2	0
e62a1855fcf15d2eedf2348910e02244	0	0	0	0	0	0	0	0	0	3	0	0
8ed9dc73f7017b5dda2e366aa4dddf43	0	0	0	6	0	0	0	0	0	0	0	0
a07061f546332ea1fa8e3f980f431c37	0	0	0	0	0	0	0	2	0	0	0	0
482bb11694fcac7969ba861cca44ed74	0	0	0	0	0	0	0	0	0	3	0	0
c41005bde906cf8c2bfbad41d9302050	0	0	0	0	0	0	0	2	0	0	0	0
9266e1ffd6e6a5b1b45a9aa467096ab4	0	0	0	0	0	0	5	0	0	0	0	0
e1ae45354497471088139763a5e5c12e	0	0	0	0	0	0	0	0	0	0	3	0
39757b9be2df27a24541fa48b402b8e1	0	0	0	0	0	4	0	0	0	0	0	0
6d5c037978072fe7fdbf236fce30d81c	0	0	0	0	0	0	0	9	0	0	0	0

72271fd31b241255c03436cc f88a2aec	0	0	0	0	0	5	0	0	0	0	0	0
1610775b0818732390e6de8 c4b80e27c	0	0	0	0	0	0	0	20	0	0	0	0
c5c4ef558d0d12c37b396ceb 619d0899	0	0	0	0	2	0	0	0	0	0	0	0
deda2b006191314da4dba13 c3450ce05	0	0	0	0	0	0	0	0	0	0	0	89
a0c0338930076bb479ef9583 fd23f778	0	0	0	0	0	0	0	0	0	0	0	2
c4b0340b471da799d7e2410 0e60b61fc	0	0	0	8	0	0	0	0	0	0	0	0
59dfd53b43765798dfa6ce97 d9223807	0	0	0	0	0	379	0	0	0	0	0	0
690caa6449bfe5c390e3b14f 79de0ff0	0	0	0	180	0	367	0	0	133	301	617	280
f40369cf48ccb2ab10771d43 196485eb	0	0	0	0	4	0	0	0	0	0	0	0
0b77c4fdbe3297eda3733ec0 a2178f60	0	0	0	0	0	0	0	7	0	0	0	0
8b691689da1b06a4acf56af0 5f2460d9	0	0	0	0	0	0	0	0	0	0	9	0
6b9b50a084444bf963356449 a37757e8	0	0	0	0	0	9	0	0	0	0	0	0
25cad1d28b56128178061b5 aa5f32294	0	0	0	0	0	0	0	0	5	0	0	0
0e61f10f5068b5c5ddc283d2 23b0d12a	0	0	5	0	0	0	0	0	0	0	0	0

4a3d5c7871e2fd3a38931d46c553b0b2	0	16	0	0	0	0	0	0	0	0	0	0
ff6494a002c00a23cfe19654cb5ba269	15	0	0	0	0	0	0	0	0	0	0	0
b3a61e0b597c46848a0f359749bec5b5	0	0	0	0	0	0	0	0	5	0	0	0
31423219d58febc09a7e6af3494cf487	0	0	0	0	6	0	0	0	0	0	0	0
8b0ab295212f77c5ddae6c8f9bc39306	0	0	0	0	0	0	0	0	0	0	26	0
6cce0a6f1adabf836f4dc701968c6b3e	0	0	0	0	0	16	0	0	0	0	0	0
62103f9bd3d52e13a07f6a83cbc12651	0	0	8	0	0	0	0	0	0	0	0	0
fd2c2c1749397b468b2d33a1a42ab198	0	0	0	0	0	0	0	0	0	3	0	0
eef26abe61e99b40f113de1415d917f3	0	0	0	0	0	0	8	0	0	0	0	0
9950001a7c8954754b23876d6d27660a	0	0	0	0	0	0	0	0	0	0	4	0
c942ababc2ec43534af31d719550d68b	0	0	0	0	0	0	0	0	0	0	5	0
f3b62280c7d135539d306ea444738b69	0	0	0	0	0	2	0	0	0	0	0	0
de15727c014a7fed794e8d2dcb6e15f0	0	0	0	0	0	0	0	9	0	0	0	0
ed3bf119d77d8f32b36b34cf062f93b5	0	0	0	0	0	0	0	0	0	0	11	0

1d49d9e8c28bee1e45a2905d4b0a4732	0	0	0	0	0	0	0	0	0	0	24	0
f01462c56c862915a365f97ee0fce142	2	0	0	0	0	0	0	0	0	0	0	0
99774c2a0363e259895b316332509bec	0	0	0	0	0	0	0	0	14	0	0	0
ed582769c2cc83afe5cf2e8d2c55096b	0	0	0	16	0	0	0	0	0	0	0	0
3f809d83d9bc86fdbd0b26f4cdfc7dd3	0	0	7	0	0	0	0	0	0	0	0	0
b41acd3794fc0e6a2b363cf91dcf8211	0	0	0	0	14	0	0	0	0	0	0	0
67c85a2968d1fa75a6553232bd99b584	0	0	0	0	34	0	0	0	0	0	0	0
47dbf8027b57643188d9726bf1aeba32	0	0	0	0	0	0	0	0	0	2	0	0
98a247884097dbe9705b52b67987e9fe	0	0	0	0	0	0	0	0	0	0	2	0
d08b731c4b303fcf9325c1bacec908fe	0	0	0	0	0	0	0	0	0	13	0	0
3f27edb5c6ded5b2281572689aeda6d8	0	0	0	0	0	0	0	0	16	0	0	0
c85edafd0d6088f0f996103c96e0586e	0	0	0	18	0	0	0	0	0	0	0	0
e04a5918b9d9d16717b3d49725796d44	0	0	0	0	0	3	0	0	0	0	0	0
d3cc3d2333b98cd159a23b09b1636922	0	0	0	0	0	0	0	2	0	0	0	0

5d8c83b0ded93943a8d72ec0e5014754	0	0	0	0	0	0	0	0	0	0	0	45
0aae55e43b587116a16bc96ed44036c1	0	0	21	0	0	0	0	0	0	0	0	0
51b924ab1a0a9cb0c63e237dd3e05237	0	0	0	0	0	0	0	9	0	0	0	0
66d8b03a0747d0a33f2b3d755f33bae0	0	0	0	0	0	0	0	0	0	0	0	3
21b70b9a8a0b16ae36178b4191577633	0	0	0	0	0	0	0	0	0	0	0	4
5abd963c1ba59c5902844e45c1360881	0	0	0	0	0	0	0	0	0	0	0	3
e803c5356a09c5c652ebad1f498d5833	0	0	0	0	0	0	0	0	0	6	0	0
a36cc5ae3d2a57975925c7852f51d86e	0	0	0	0	0	0	0	0	0	0	8	0
77e24fdf3df927230d8e138b2afe1c5e	0	0	0	0	0	0	0	0	3	0	0	0
283cbd07c5319c5f435fe5babd5b9058	0	0	6	0	0	0	0	0	0	0	0	0
623c2c0f9a88e4c7bcef80f83a37c5bd	0	0	0	0	0	0	0	0	0	3	0	0
2821d30ca325bf17149be11f8fc3cec4	0	0	0	0	0	0	0	27	0	0	0	0
b3ae31995a16435db25286c651a61bc8	0	0	0	0	0	0	0	0	0	0	4	0
9a4ae49adcb2e99b9aef5d6bf9a3acf7	0	0	0	0	0	0	0	0	0	0	0	25

3b5e1435b05d3d9869868efc2552caa2	0	0	0	0	0	0	0	0	0	0	2	0
c02c2d1b0e4c37a8e0e19e9117b4212f	5	0	0	0	0	0	0	0	0	0	0	0
fe1fc5ec4aa7a2e704e1efb56b4778d5	0	0	0	0	0	0	0	0	0	0	0	2
34aed48ac26411439bda6399301a4296	0	0	0	0	0	0	0	0	44	0	0	0
03c9b515a34b37095860ecd4f88be55a	0	0	0	0	0	41	0	0	0	0	0	0
e50766d8de0d55c3c2a6fb4346cc9c44	0	0	0	0	0	3	0	0	0	0	0	0
5b33c1881ad27f48d83e3e8284d2f5b4	0	0	0	0	0	0	0	0	0	0	6	0
c25404cb6440dd93d50fd50050fc3fca	0	0	0	67	0	0	0	0	0	0	0	0
168a838ab7197a9959cf01b5be8f7b1d	0	0	0	0	0	0	0	0	0	0	5	0
665fc15a873400bff79b8cdbbc05fc09	0	0	0	0	52	0	0	0	0	0	0	0
123cd7c1f474735aa1ef34c3d722af06	0	0	0	0	0	0	0	0	0	0	2	0
026e962f8daa8d47c3e88f8a7ffadddc	0	0	0	0	0	0	0	3	0	0	0	0
2174917f7390ee74bc8cdd3d7ac8992b	0	0	0	0	0	16	0	0	0	0	0	0
111f173623340926570496471fd9a50b	0	0	0	0	0	0	0	4	0	0	0	0

194cb3210d6e5b2c034cf0b79106c755	0	0	0	0	0	0	0	0	5	0	0	0
1729d68d6c42248d83497025f967625c	0	0	0	0	0	0	0	0	0	0	0	6
c3075560d77e1f8bb01fa7b9f2f35eb7	4	0	0	0	0	0	0	0	0	0	0	0
c1d745b4e0a8acf2dec2630c94647142	0	7	0	0	0	0	0	0	0	0	0	0
214dff324e3a84e6911b3bf47e200833	0	0	0	0	0	0	0	0	0	0	19	0
cb9fef647b2c479ef310f6476a94faae	0	0	0	0	0	0	0	5	0	0	0	0
9c6c5146ca6263e9f32d62f64f65a328	0	0	0	0	0	0	0	0	0	0	12	0
b08ace1b4676f9da8aaf3d5205a1fc0e	0	0	0	0	0	0	0	0	0	0	2	0
b600ed7ae77cf96983cef7124632575a	0	0	0	0	0	0	0	0	0	0	6	0
fdcc96c7e4418500077a1e45b305a835	0	0	0	0	0	0	0	0	0	0	0	34
e871c9c19def7c1d224994cbf6b180ea	0	0	0	0	0	0	0	0	4	0	0	0
19a8c5c5b72a2ec606f7e0e6e930b3d1	0	0	0	0	0	0	0	0	0	66	0	0
c04180a5f3a9f27b61ef5d12b0520bbf	0	0	0	0	0	0	0	0	0	0	2	0
cf69331474dac16d794adb105402a62c	3	0	0	0	0	0	0	0	0	0	0	0

78416d025c4006725786a1218499fb19	0	0	0	0	0	0	0	0	0	0	3	0
c67d19b30c4fa95605d8ac15ec7cb15f	0	0	0	0	0	2	0	0	0	0	0	0
1074b77143681211fc340f6b18c895ce	0	0	5	0	0	0	0	0	0	0	0	0
28c5fad2c4ab58ba4ef1a9b9259a1b72	0	0	0	0	61	0	0	0	0	0	0	0
c737fd48f3d1052e52e8d882b98c0a30	0	0	0	0	0	0	13	0	0	0	0	0
5425fd4f7124d93a26ca49412c00e74f	0	0	0	0	0	4	0	0	0	0	0	0
aedbc5fc9de29ceb2848c37e67387105	0	0	0	0	0	0	0	0	0	3	0	0
c739bd7b0cfa400e6bb4e962b65eca41	0	0	0	3	0	0	0	0	0	0	0	0
9357a37d5dcdb79c72a540f2f5ed18e8	0	0	0	0	0	0	0	6	0	0	0	0
42a20b19754eb57df4e785a9a3c7ef04	0	0	0	0	0	0	0	40	0	0	0	0
95b7d463776051b1b65180d969e1e9b1	0	0	0	0	0	0	0	7	0	0	0	0
008bc2db201876d6d970e854ffa7185f	16	0	0	0	0	0	0	0	0	0	0	0
a018220148e082fd96437cb904b8f85e	0	0	0	2	0	0	0	0	0	0	0	0
b422f1b766eb95743d976e2a018f5cb7	0	3	0	0	0	0	0	0	0	0	0	0

31cab6435b39a373da30e61da69766a9	0	0	0	0	0	0	0	0	0	0	0	7
c68afcbe94f669f67dba5df2e62d07ec	0	0	0	0	0	0	0	0	0	0	0	5
a35429a29c92898dab2adcceeb37c99a	4	0	0	0	0	0	0	0	0	0	0	0
efb6b693ee6631456d1d4e644ccdee48	0	0	0	0	0	0	0	0	0	0	0	13
40f0ed7cb1faa94cfa4b9fa74beefe7d	0	0	0	0	0	0	0	0	3	0	0	0
63990159f0b4586d7e9a27023be1f849	0	0	0	0	0	0	0	2	0	0	0	0
d90803ae50997c79448d473b986a9f0a	0	0	0	0	0	2	0	0	0	0	0	0
6725b821fb83fbe6ce9419476fdd69d9	0	0	0	0	0	2	0	0	0	0	0	0
a3f761b2865b5609465add9a9a622bc2	0	0	0	0	0	0	0	0	3	0	0	0
7c7443776c9e952f6fa82c31a992d02d	0	0	0	0	0	0	0	6	0	0	0	0
339cdcf5f1dd858014320f46300e925d	0	0	0	0	0	0	0	0	0	0	0	20
5c9dd2b1f74dd68b17dfe2c46d36d453	0	0	0	0	0	0	0	0	0	0	0	3
57ef6f348795088c319e1180e0f146e2	0	0	0	0	0	0	0	0	0	3	0	0
713325dde7be9635e4315792a753496f	0	0	0	0	0	3	0	0	0	0	0	0

6d0b84201c16eb2620afb343663bcaf8	0	0	0	0	7	0	0	0	0	0	0	0
a68e048e935e4513f8df9fd8cea26518	0	0	0	0	0	0	0	0	0	0	0	4
60428b60173807318f90046d36f74aae	0	0	0	0	0	0	0	0	4	0	0	0
005350b051458f7af7fe471ed1aced13	3	0	0	0	0	0	0	0	0	0	0	0
a25ed3bd01e9e3b4d1cec2a2e7869647	0	0	0	7	0	0	0	0	0	0	0	0
35a7237d34969309a82e36a21d8aed5c	0	0	57	0	0	0	0	0	0	0	0	0
d3fd2dc13efc6c936b090f9e8908759b	0	0	0	12	0	0	0	0	0	0	0	0
7e3d063a43f014286dff3346f434ad5e	0	0	0	0	0	0	0	0	0	4	0	0
779a73365733799a16474333970a793c	0	0	0	2	0	0	0	0	0	0	0	0
702b11fbe4e17069bc25d852462fedaf	0	0	0	0	0	0	0	0	0	0	0	6
7a51ffc80f4d65bcdcd0eee41f5c71bc	0	0	0	2	0	0	0	0	0	0	0	0
60e258f25f7ccedfed70eaf4cea3162d	0	0	0	0	0	0	0	0	37	0	0	0
fdbd396ef760b24a6d7c59e902dc25f1	0	0	0	0	0	0	2	0	0	0	0	0
f21cc572a07ea67b638f0f9c12994669	0	0	0	0	0	2	0	0	0	0	0	0

aec1a384b130083e93165e2e0ed7f03c	0	0	0	0	0	0	0	0	0	0	4	0
6f0b5351e6039a5395c885d208efd870	0	0	0	0	0	3	0	0	0	0	0	0
267f2d9847ebdb45f468c9294897c393	0	0	0	0	3	0	0	0	0	0	0	0
cd48650eef17fe610a95153130950029	0	0	0	0	0	0	39	0	0	0	0	0
be65208697aae0cb545c8e865a71fbea	0	0	7	0	0	0	0	0	0	0	0	0
c15e6d8a588224b3ad2f8ced650f6a5a	0	0	0	0	10	0	0	0	0	0	0	0
561542de4b241c4adc554b0607f893b5	0	0	0	0	0	0	0	0	2	0	0	0
e0396dd121b105cf73bd70cda01a01ed	0	0	0	0	0	0	0	0	0	0	2	0
36c9db8ce3e02da7cbaddbde71de16ef	0	0	0	0	0	0	0	0	0	0	3	0
55528deaa0443b9210af96e56477ea3e	0	0	0	0	0	0	0	0	0	0	47	0
b63bbffd3d25e7856b0a4babbeec3a3f	0	0	0	0	0	0	0	0	0	0	0	5
9594df9daa72388411e1b6fb95a1203c	0	0	0	0	0	0	0	0	0	0	0	5
76d389cd0d074cc68df9dff476a12320	0	0	4	0	0	0	0	0	0	0	0	0
64cfc3e9cb4006cb78604c20a857e7a7	0	0	0	0	7	0	0	0	0	0	0	0

5a8908d6121c89aa8e65718ffffb4546	0	0	0	0	0	3	0	0	0	0	0	0
c3e24e2f3cf59451a2910d074e1264f4	0	12	0	0	0	0	0	0	0	0	0	0
012ff05281729fb53a0c2363a1359cbb	0	0	0	0	0	0	0	0	0	0	2	0
8a6b4df021fafa2608bc1e15d5222863	0	0	0	0	0	0	0	0	0	0	10	0
622f21e3f7048a22bd7bc98ef58f3efd	0	0	0	0	0	0	0	6	0	0	0	0
b1e6de8d162d46e03d0d5993f9fd229b	0	0	0	4	0	0	0	0	0	0	0	0
8d81c407f8568345ff406c528e8cb5ed	0	0	0	0	0	0	0	0	0	7	0	0
b573d23b4eec4abbf86d9217b192cea0	0	2	0	0	0	0	0	0	0	0	0	0
afb7b10dd44ca776bc3bf10cea18c4d6	0	0	260	0	346	1009	0	1106	137	508	481	1173
e845f37cb6377270d3c8b27c830ebb19	0	0	0	0	0	0	0	0	0	0	9	0
9299673c33ac54fc34bb1953503092e0	0	0	0	0	0	0	3	0	0	0	0	0
e8811d5e53c65f71cbf4f1bfcbbf5ef8	0	0	0	0	0	0	0	216	0	0	0	0
b71c9f3e56a8fc6f45e80cd86af2c382	0	0	2	0	0	0	0	0	0	0	0	0
7a8cdeef93fd53c17dc346e24cd0a400	0	0	0	0	0	0	0	0	0	0	4	0

67e22950bdb7f1795ea35a968f530bdf	0	0	0	2	0	0	0	0	0	0	0	0
5fae0371b44dee2e9a169ece61888460	0	0	2	0	0	0	0	0	0	0	0	0
eae98c8f0b644eaee203a436911a494a	0	0	0	0	0	0	0	10	0	0	0	0
80322df6944f2c026208ecd1eab3d7ea	0	0	0	0	2	0	0	0	0	0	0	0
af6354f76666ad54d44620ac4b62aa64	0	0	0	6	0	0	0	0	0	0	0	0
635b0acdc66c696ba28512866955c66a	0	0	0	0	0	0	0	0	5	0	0	0
9b3be328259345694810774821cd5da7	0	0	0	0	0	11	0	0	0	0	0	0
0787aaf472ab0606eade141604a4da87	0	0	0	0	0	0	0	0	0	0	36	0
7616318f7bb1f792a78c566f17cfa5dd	0	0	0	0	0	0	0	0	0	0	0	4
9f81a77f8a532b6eda2127f5e824c2db	0	0	0	13	0	0	0	0	0	0	0	0
a59d0a0e44f913c9c5b61ccf419e883f	0	0	0	0	9	0	0	0	0	0	0	0
b8d04fe9e017133f58e573792d41347d	0	0	0	0	0	0	0	0	0	0	0	16
a169fbae1e305e9666a8ad5dd64c762f	0	0	0	0	0	15	0	0	0	0	0	0
01329f47c0fa5b1085edd1635abd8013	0	0	0	4	0	0	0	0	0	0	0	0

5a68c0ca8a890153dc4a1074eda6a192	0	0	0	0	0	0	0	0	0	4	0	0
98a26710687fb708fd9b0af1d2cee6d8	0	0	0	0	0	0	0	7	0	0	0	0
95712f7add8a6ee002f034a175810bba	0	0	0	0	0	9	0	0	0	0	0	0
636dce20a658650cb9e7b70724949f19	0	0	0	0	0	0	0	0	0	5	0	0
39c835b53576963caec6f3b8d284e8b7	0	0	0	0	0	0	0	0	0	0	3	0
672a0101ba513afa66b5c0abf7c72c65	0	0	0	0	0	0	0	0	3	0	0	0
ced4105926b54b0521cd69291d97c330	0	0	3	0	0	0	0	0	0	0	0	0
c588640aa04f771d156c18d96c5d9404	0	0	2	0	0	0	0	0	0	0	0	0
c4d68816ed1a27f573eb9b720d5bd267	0	147	0	0	0	0	0	0	0	0	0	0
1043209e1468ddee9c09cbdd6abf8ee9	0	0	0	0	0	0	0	7	0	0	0	0
29f1f4eff45ad9deffa6e609dd6b3747	9	0	0	0	0	0	0	0	0	0	0	0
400b874db8a45f8dd5532bd6e3d177b5	0	0	0	0	14	0	0	0	0	0	0	0
aab4ffe79535d12d17e9d8cf45fc32d8	0	0	0	0	0	0	0	0	0	16	0	0
299bee75ac5cea4e61e70742c918d108	0	0	0	0	0	0	0	0	0	0	5	0

93458910db1b5e34a23320aab95a4cae	0	0	0	0	0	7	0	0	0	0	0	0
8309e1995c89d9d3fc34dba392de9657	0	0	0	0	0	0	0	0	0	0	0	12
1f7849e528ad32d16d09d07430f1b367	0	0	0	0	0	0	0	0	0	0	0	2
b5b7550c6294a3e1052cc09e4f951fe9	0	0	0	3	0	0	0	0	0	0	0	0
a6d918f5a6dd59ebc5ae56820b1e0d40	0	0	0	0	0	0	0	0	0	0	2	0
1c6b8081e2693954dff7d23b71e057ad	0	0	0	0	0	0	0	0	0	0	12	0
6f37dacfb168bd4fda3b8b627cf4a49a	0	0	0	0	0	0	0	0	0	0	4	0
0c4cba5bf024b9ee573109b126ad24ef	0	0	0	0	0	0	0	0	0	22	0	0
17812c684c2498c4ad54b4b4b5f30550	0	0	0	0	0	0	0	0	0	0	4	0
8058bbc0d651a5ec2b24a5ab0fdc1f64	0	0	0	19	0	0	0	0	0	0	0	0
b195a5af67cc6730ec3add9eb783ae81	0	0	0	0	0	0	0	0	0	0	52	0
332700bc43bdf28ae8752004c7f0e835	0	0	0	0	2	0	0	0	0	0	0	0
73d3715a1b9583233c15bf189fb45899	0	0	0	0	5	0	0	0	0	0	0	0
f37c94ecff9ad06f1b291a554ccb4162	0	0	0	19	0	0	0	0	0	0	0	0

dab84076c0557f26f6ad5e00fb011620	0	0	0	0	0	50	0	0	0	0	0	0
03f5da56b5cf0635748e7a8ec3bd0725	0	0	52	0	0	0	0	0	0	0	0	0
8db4f6000b8f757a99d2a87f1cd538e4	0	16	0	0	0	0	0	0	0	0	0	0
ae492945bb742acb7861f454fbf4c836	0	0	0	0	0	0	0	0	0	0	2	0
81d02d5718988ce9508a2dcf8ba46d57	0	0	0	0	0	0	0	0	0	97	0	0
8e02ef8cc79995f79aab8b7aeab06e94	0	0	0	0	0	0	0	2	0	0	0	0
c0ec8c9dd87674b3dcc0d8b57dfd0ffc	0	0	14	0	0	0	0	0	0	0	0	0
74f967623750e3d819a9ed0d5a632749	0	0	0	0	0	0	0	0	2	0	0	0
94f99b236f93860200715e09e88dba64	0	0	0	0	0	2	0	0	0	0	0	0
6d62f24256c17b5e044a6cc51a1251b4	0	0	0	0	3	0	0	0	0	0	0	0
6733aadd17cac0ccc85ba7021d35db95	0	0	0	0	0	0	3	0	0	0	0	0
11a725ced162003f3bf91cde3e166c8f	0	0	0	0	0	0	0	0	0	0	2	0
215d44fd3867e6331a068a9fa27955f7	0	0	0	0	0	0	0	0	0	0	0	75
d4267de5929670bc2d2e07e5861853f2	0	0	0	25	0	0	0	0	0	0	0	0

cd38f07c38c586f9c21114bd4a260b0b	0	0	0	0	0	0	0	0	0	52	0	0
04a3249025a9c3d06f002f6ce37a4239	0	0	0	0	0	0	0	0	0	0	6	0
bfb8edc973bcd5e87680c660f967d556	0	0	0	0	0	0	0	0	0	0	6	0
16f6dd28d89447ffabb48a7edb49bd22	0	0	0	0	0	57	0	0	0	0	0	0
2b801d3b709269e8c9095e6b9a2f9788	0	0	3	0	0	0	0	0	0	0	0	0
56d7733c7c0b380b70af4870616186ae	0	0	0	0	0	0	0	6	0	0	0	0
320f316c0a4f327656c6d0be509f70d9	0	0	0	0	0	0	0	0	0	8	0	0
1e980a0cd32437713fc8a233629014c2	0	0	3	0	0	0	0	0	0	0	0	0
65ce9b1343387080acb40232190341d7	0	0	0	0	0	0	0	0	0	0	3	0
293781550508321c3ca06e295bd22064	0	0	0	0	0	0	0	0	0	0	2	0
74fe0d68b9ebccae8054b1dd4903be28	0	120	0	0	0	0	0	0	0	0	0	0
62dbd17e33957a2290efd0e29c6f716d	0	0	0	0	0	0	0	0	0	38	0	0
b3a265fe062e1b4cf15e30695abfe029	0	0	0	0	0	0	0	0	0	0	4	0
931ee393b46874f2ae22f98eaec16f6d	4	0	0	0	0	0	0	0	0	0	0	0

38f5cebfad865ec765f2989aaac89d76	0	0	0	0	7	0	0	0	0	0	0	0
01888607db93850ebd8c3d6a94bdcbab	0	0	0	0	2	0	0	0	0	0	0	0
3bc3c10c6d754c5b6b6f778eb910d537	0	0	0	0	0	87	0	0	0	0	0	0
edb510d53d2ea4d0d23a4e10d219b410	0	0	0	0	0	0	0	0	0	0	0	4
dabda61b6ff81a9be79a23b4b0f7ec81	0	0	0	0	0	0	0	0	0	0	0	4
9fdc0733ac1196b75bdf3a98603f06a1	0	3	0	0	0	0	0	0	0	0	0	0
64ad9398586974bc6dc352ac88b5ab25	0	0	0	0	0	0	0	0	0	0	11	0
65b62a9691f6f603be8f32857b3ee417	0	0	0	0	0	0	0	0	5	0	0	0
e08fa741047d85136e15ee4f06285e6e	0	0	0	0	0	0	0	0	6	0	0	0
e85c33c2ca1f65b817a91a82c50577f7	0	0	0	0	13	0	0	0	0	0	0	0
ca6c739634ee510aa148c2bb879223ef	0	0	0	0	0	0	0	0	0	10	0	0
64f8f2c98b5e571e1a6993b3f0d8cfcd	0	0	4	0	0	0	0	0	0	0	0	0
df3ab774447564672c0df944e4dfa43a	0	0	0	0	0	0	0	0	0	0	3	0
84186ff0654ea9a7a6e83d70c62d56ae	0	0	6	0	0	0	0	0	0	0	0	0

ff6a9270bc9e8dffd25a584d1072fafa	0	0	0	0	0	0	0	0	0	0	5	0
3862295044f0b823a117529d114ac0eb	0	0	0	0	0	0	0	0	2	0	0	0
6a615ef46a476480082a29a0e4158af2	0	0	0	0	0	0	9	0	0	0	0	0
77261e0ef63057d32ce2da760223c47b	0	0	0	0	0	0	0	0	0	4	0	0
5f73e3c6c0021a4fedc1c8b88b8ced01	0	0	21	0	0	0	0	0	0	0	0	0
cb5a503d5efe65eeba588dd6e035ad76	2	0	0	0	0	0	0	0	0	0	0	0
26757356dbec85ad408767db1dc584bd	0	0	0	0	0	0	0	0	0	0	2	0
557367961b4782cfb7684e71a16058fd	0	6	0	0	0	0	0	0	0	0	0	0
6749cc4e4f9dc5e9f3ecb2f4cbbeed4d	0	0	0	0	0	0	0	2	0	0	0	0
04a79599afe694140e13f7979a675f6b	0	0	15	0	0	0	0	0	0	0	0	0
7fa9018ab52d3ec76048a4f697be25d6	0	0	0	0	13	0	0	0	0	0	0	0
e3cfbeacda6753bfa27e553286d30c28	0	0	0	0	0	0	0	0	0	0	12	0
67723f9b6eafa9031b9d6b7e5260302e	0	0	0	0	0	0	0	3	0	0	0	0
f8329e0db34037b3c9dc6c9ec0bb9cc4	0	0	0	0	0	0	0	0	8	0	0	0

35912e5bb7836d0cced61d6494b69184	0	0	0	0	6	0	0	0	0	0	0	0
6464e32ba9741bc76989cb34a938428e	0	0	0	0	0	5	0	0	0	0	0	0
3bcab0c05f68ece05e4223209e98d5d6	0	0	0	0	0	0	0	0	0	0	114	0
e04c7d9f5baef11ab19d0f49aa567a42	5	0	0	0	0	0	0	0	0	0	0	0
1ffcec95f12ec591fe48498d775b735b	0	0	0	2	0	0	0	0	0	0	0	0
328d70984bd0dab7b791db53c64a5d9c	0	9	0	0	0	0	0	0	0	0	0	0
6a1c0982ad9f717767616bbfcefa66be	0	0	57	0	0	0	0	0	28	0	43	311
5a3bef8b5a60273d0a6f7f466207ea27	0	0	0	0	10	0	0	0	0	0	0	0
7d1d8879d268f8d1fac966152bdd567b	0	0	0	0	0	0	0	0	2	0	0	0
cad5c112b08b712845530158db64e585	0	0	0	0	0	0	0	0	0	12	0	0
48fb22845dd99dcdbbf5c035e29d39ae	0	0	0	0	3	0	0	0	0	0	0	0
01afb9aec05fc74964b821e8aae2958e	0	0	0	0	20	0	0	0	0	0	0	0
b4dddaea8203d4c8efa458b7d32a1136	6	0	0	0	0	0	0	0	0	0	0	0
c1cf79845125a552acfd4a116473a555	0	0	0	4	0	0	0	0	0	0	0	0

c3fa37c80ab23d2472c47a04 36d54fc4	0	0	0	0	0	0	0	0	0	0	7	0
ac154eb72e4893ac425a9897 d49c1c92	0	0	0	0	2	0	0	0	0	0	0	0
00a48b0ae9143de17fb04dde d9e7f019	0	0	0	0	0	0	0	6	0	0	0	0
1e727146be95c759ddba762 3a416c950	0	0	0	0	0	0	3	0	0	0	0	0
6ca02938057c4e10db364b9 541d9b014	0	0	0	0	0	0	0	8	0	0	0	0
9e76f24787720fa730df8c4df 51fbb33	0	0	0	44	0	0	0	0	0	0	0	0
082d45ae34c9f9a5f975976c e4009569	0	0	0	0	0	0	0	17	0	0	0	0
7cf7d370afa7326f2780f510a 5b521a5	0	0	0	2	0	0	0	0	0	0	0	0
84609068f7d9c9cfee71eadef b2610e7	0	0	0	0	0	7	0	0	0	0	0	0
0ec82a48db9c520abb95d92 7ff48d931	0	0	0	0	0	0	0	0	0	28	0	0
1b58d176952ee088a5f14d20 2a7f866a	0	0	0	0	0	0	0	0	0	0	0	15
4d989b7f31c9b91156a5a96b 12132f76	0	0	0	3	0	0	0	0	0	0	0	0
dd3a21199b00e984bddbbfd ddd0c4417	0	12	0	0	0	0	0	0	0	0	0	0
703bd01816cf5412186fd95f 237ed287	0	0	0	0	0	0	0	0	0	0	2	0

32368f43f5d08129c8e7d7d1a40806f3	0	0	0	0	0	0	0	0	2	0	0	0
22ebec912643f67732f0bc820553c0fc	0	0	0	0	0	0	2	0	0	0	0	0
a381bd53410494af406cf6467859f4ea	0	0	0	0	0	12	0	0	0	0	0	0
cf7a7d04ec9cac3de242120a620f9b77	0	0	0	14	0	0	0	0	0	0	0	0
cc1c7a5702447be4eef6b4d0ec5417e5	0	0	0	0	0	0	0	26	0	0	0	0
4c975a5a0011fc2e15bdcee1d5ac1b2f	0	0	0	34	0	0	0	0	0	0	0	0
dfc807907611cc7f6b04e16af9838eac	0	0	0	0	0	0	0	0	0	2	0	0
d47093199f31e520c88267036427eff4	0	0	0	0	0	0	0	0	0	0	116	0
d65f300bc5a9d8d45285a336d1a7586f	0	0	9	0	0	0	0	0	0	0	0	0
58edaf16e277189972028f50fd0743b7	0	0	0	0	0	0	0	0	0	0	22	0
f0c73a44254314fc9b3aa9af4e328fff	0	0	0	0	0	0	7	0	0	0	0	0
3201a2af99af755ba5d192e1979dbc1e	16	0	0	0	0	0	0	0	0	0	0	0
3f6e5946520b66292b34ee4b26225829	53	0	0	0	0	0	0	0	0	0	0	0
1ef9740d81d4835b05864eab63208e2a	0	0	0	0	0	3	0	0	0	0	0	0

c6263544abaa5dfc866c56ad3dd5ce7f	0	0	0	0	0	0	0	0	0	0	0	3
320901055a28f42e34a0f6e78944da5a	0	0	0	0	0	0	0	0	0	0	0	32
26c6b74f4d1105a984505133436a9cb9	0	0	0	0	0	0	0	0	0	0	12	0
69ac6c3ec94a92cd4f7b617522091dfb	0	0	0	2	0	0	0	0	0	0	0	0
f819afd9a6738ea95691ea790ca9cc57	0	0	0	0	0	0	0	35	0	0	0	0
eab32a795789e23e8a36da8551ea2d1c	0	0	0	4	0	0	0	0	0	0	0	0
085f2ab66a2d5bfd84bee14f28daae56	0	0	0	2	0	0	0	0	0	0	0	0
557590e82f7518805914ab7d6b3ab234	0	0	0	2	0	0	0	0	0	0	0	0
4e7640d7023cd54d8ea89b46a43999b3	0	0	0	0	0	0	0	0	0	11	0	0
d2972b582d17ef9a7a0f6b7f8e98a860	0	0	0	0	9	0	0	0	0	0	0	0
6206603a63565c454277b0dd180c502d	0	0	0	0	0	0	0	0	0	5	0	0
f2a4763a54eb7e18db61f7f0a782baab	0	0	7	0	0	0	0	0	0	0	0	0
f142d0fe83c439b7764be752ed5fead3	0	76	0	0	0	0	0	233	0	0	0	0
be766e22f34f75c29f908735595f3fe0	0	0	0	0	0	0	0	0	0	11	0	0

6a4d86777e34e255bdb66e51df88182c	0	0	0	0	0	0	0	4	0	0	0	0
32d50ff131ae098ff68edc3d45ea75fc	0	0	0	0	0	0	0	0	0	4	0	0
f7b59c18acf9c9f5fdc6ecb29ac50872	0	0	8	0	0	0	0	0	0	0	0	0
a59cfd529a95684d30337aa45e5fb5bd	0	0	0	0	0	0	5	0	0	0	0	0
52dbe6c2022a125366e42e1c2ca3951f	0	0	0	0	0	0	0	0	0	0	0	4
2cfc260721ca1ec02ee1441ce017f24d	0	0	0	0	0	2	0	0	0	0	0	0
75a3b8d9aca49332e4c179a6d436159c	0	0	0	0	0	0	0	0	0	15	0	0
667e7f43901c200410e7cefbc0745545	0	0	0	0	0	0	0	0	0	0	0	3
db0a2dc532d47c3d52e7deed0365c861	2	0	0	0	0	0	0	0	0	0	0	0
ee4a8fb63426f24d0abd5f132a1aabd9	0	0	0	0	6	0	0	0	0	0	0	0
3e7b37f9f69981964a76901ddd09b7ec	0	0	0	0	0	0	0	0	0	2	0	0
ea967909ec7f49aeee4df36e9f56dbe1	0	0	0	0	0	0	2	0	0	0	0	0
a83e14e72aaa60d1d5c42dcbb289da79	0	0	0	0	0	0	0	0	0	0	2	0
0b467eca655629eac526f91f7c684fa4	0	22	0	0	0	0	0	0	0	0	0	0

2b8dddcd8d4f337ae176bdbdd6c7cf25	0	0	0	0	0	0	7	0	0	0	0	0
80cd7db08a99db52d7d64af8b2ec16bd	0	0	0	0	0	0	0	0	0	0	0	11
21b3c840d45e3850a9fd977211b2b1e4	0	0	0	0	0	0	0	0	0	0	0	16
c162ec9cf3db4d51dc909a643368e9ac	0	0	0	0	0	0	0	0	5	0	0	0
a92f83053a6d26177870bf583e5d28d4	0	0	12	0	0	0	0	0	0	0	0	0
f5b3a35aa7b950adf71bae8c4c4840b0	0	0	0	0	0	0	0	0	0	109	0	0
4777ec4c4ad124a418d2e64ecb804734	0	0	0	0	0	0	0	0	0	0	0	6
4b92660e243c0683dae1f0c32017b92d	3	0	0	0	0	0	0	0	0	0	0	0
3b145c77785ff3e567aa3e77c69d5c56	0	0	0	0	0	0	0	0	42	0	0	0
b9c994f2636236e2ded57d503bf8eb2b	0	0	0	0	5	0	0	0	0	0	0	0
375f343f93ca38765d611d28dbcd002e	0	0	0	0	0	0	0	0	5	0	0	0
673916d7788c160182bdf709bfd6a368	0	0	0	0	0	3	0	0	0	0	0	0
d558f53eb56ed37a1338b1d3c827462f	0	0	0	0	0	0	0	0	0	2	0	0
f63cf07cdc519cc8e315c390b2fa5e74	0	0	0	0	0	0	5	0	0	0	0	0

bee1700335a366d32bb8253 86421c315	0	0	0	0	0	0	0	32	0	36	0	0
0d68ca45deea2cf566a19731 f6b42182	0	0	0	0	0	0	0	0	57	0	0	0
e74c0f9db8911968e3881070 0e898b6c	0	0	0	0	0	0	0	0	0	0	0	2
eb7ea5feaa84d594f6762b1f3 b3a4221	0	0	0	0	0	0	0	0	4	0	0	0
0789b4de19bdff3916c22252 7eb14dbf	0	993	0	0	0	0	0	576	0	0	0	0
cacf92b29b4a9be4627f2fb53 402ae3d	0	0	0	0	0	0	0	0	0	3	0	0
f96a81eada7de5851a0f7bb2 302a54b9	0	9	0	0	0	0	0	0	0	0	0	0
bd50bd3e442ae1b46405933 4b8bd839f	0	5	0	0	0	0	0	0	0	0	0	0
754d7fb9e69f77107135363c 39646d54	0	0	0	0	0	0	0	0	15	0	0	0
1c16f7a552f6b43ce4905f86e 1fa452d	0	0	0	0	0	0	0	0	0	3	0	0
e38f53bf5a10befb4a37aa43e de574b7	0	0	0	0	0	0	0	0	0	0	7	0
1c8c4886bd566b645550f4e8 fcdda5f1	0	0	0	9	0	0	0	0	0	0	0	0
692791e8db380c315073d2c 9ef2f05dd	0	0	0	2	0	0	0	0	0	0	0	0
2b6ca11b55d86039cf23dbfb ca0fc03b	0	0	0	0	0	3	0	0	0	0	0	0

87aa4872b4eed223dd7dc8b3f507f5bf	0	0	0	0	0	0	0	0	0	0	2	0
d9c535f7512d34c89169b12073e8ab2d	0	0	0	0	0	0	0	0	3	0	0	0
67edd5201f6c52a1a6318b0b1286d2b6	0	0	0	0	0	0	0	0	0	0	0	2
86d5adf9d3193029dada845d1d3eee2b	0	0	0	0	0	0	0	0	0	0	0	14
57cfb630d8d06e135471ea3457f15465	0	0	0	0	0	0	0	0	2	0	0	0
ebcd5396195358058c3b347b4625a4e1	0	0	0	0	0	0	0	0	0	0	3	0
2d6b8d31328a7e0cc5efc9f1cfccc7d1	0	0	0	0	0	0	0	0	2	0	0	0
b83a55e8f42748a447be999ea7487a2c	0	0	3	0	0	0	0	0	0	0	0	0
040b1a1ae2ed9f2931b6ee0b3764734f	0	0	0	0	0	10	0	0	0	0	0	0
953b229efa99bde8b1ac5c6c57ebea98	0	0	0	0	0	0	0	0	4	0	0	0
07af3d2d9e729ca0a4a8a12ea23aff41	0	0	0	0	0	0	0	6	0	0	0	0
f298503c1f7168c4a7c71e7613df37f1	0	0	0	2	0	0	0	0	0	0	0	0
932de56ec796a9bd55b56284205599dc	0	0	0	0	0	0	0	0	0	0	4	0
fc6082c5a9c47be5481651e82fcb61cf	0	0	0	0	0	0	0	4	0	0	0	0

763fd58d08c79c8d40ec97d9054a0eba	0	0	0	0	0	0	0	0	0	8	0	0
60a25524b7ee23ba8e942a65df1fc7c6	0	0	4	0	0	0	0	0	0	0	0	0
6a9a3cc6367d2825500b5dd2c5a28bf1	0	0	0	0	4	0	0	0	0	0	0	0
df5eda5abb83f3685ae277b3c8e2cacc	0	8	0	0	0	0	0	0	0	0	0	0
adff547ad047bb7a41808300d492e0d5	0	0	0	0	0	0	18	0	0	0	0	0
3db7a78796c4c9eb7a07fff13dda3852	0	0	0	0	0	7	0	0	0	0	0	0
2fded6e023ccbefa8f63dc8a81adcfec	2	0	0	0	0	0	0	0	0	0	0	0
cb17f0844aa5a1bd674612ddccd28c4e	0	0	0	2	0	0	0	0	0	0	0	0
0ade9c367123b6f35829640576b9bfc4	0	0	0	0	0	2	0	0	0	0	0	0
211271fe176e3f90b059fdc6f249fcc1	0	0	0	0	0	0	0	0	0	21	0	0
29c35428b12f39a3cc6df980d78b0d90	0	0	0	0	8	0	0	0	0	0	0	0
16574e77aa39b386c2eba65e32910365	0	0	0	0	0	0	0	0	0	0	0	2
5f0aaaef48df1f5f4807553133b88051	0	0	0	11	0	0	0	0	0	0	0	0
81786eb6493c94a45ece70c8a2c26a65	0	0	0	0	0	0	0	0	3	0	0	0

a6ccbbd3f525ad0b4fed041a16ff62c1	0	4	0	0	0	0	0	0	0	0	0	0
52310e239ed6ce54830c4e45d5245903	0	0	0	0	0	0	0	3	0	0	0	0
fd236e4789d15c08e7397c0aeb233ea8	0	0	0	0	0	0	0	0	0	0	5	0
32d60db72b2dbcdd810d9b5b1f819671	0	0	0	3	0	0	0	0	0	0	0	0
c4e951a6370f6a8836ca8edaf41b3325	0	0	0	0	0	0	0	0	0	0	6	0
a025810980e79548ad84e362f7a81412	0	0	0	0	0	0	0	0	0	0	0	7
acb4493f719f61c8fd95283aff9db1cb	0	2	0	0	0	0	0	0	0	0	0	0
8e453cdd6969dc2b5f5fd19b1a7b3fec	0	0	0	0	0	0	0	5	0	0	0	0
3b71b9b6c0fd15037d5515838589210a	0	0	0	0	0	0	0	0	0	0	20	0
c8c41cc06c864a81ca30e2d3b693ae9f	0	0	0	0	0	4	0	0	0	0	0	0
ba935f4532f5002e4d061447cfecb0b2	0	0	0	0	0	0	0	0	0	0	5	0
71b8d97ae21961ed3685eb27cccb941e	0	0	0	0	0	0	0	39	0	0	0	0
4f256475b45fb0080bb6f34ab702b0f1	0	0	3	0	0	0	0	0	0	0	0	0
d6c2b52b1a491a766c65c8d57d991665	0	0	0	0	0	0	0	0	0	4	0	0

047371a7326397e15f166dff9d8339cd	0	0	0	0	0	0	0	0	10	0	0	0
e37354908838ace579bef23d1dfa25df	0	0	0	0	0	0	0	68	0	0	0	0
b96b1a9fcce674d0d0be0c537c912a4c	0	0	0	0	10	0	0	0	0	0	0	0
51074d562f82e9c55660285301ee56d2	0	0	0	0	0	0	0	0	0	0	6	0
f5107b2a508c1c5aedb2738249cd1ac7	0	0	0	0	0	0	3	0	0	0	0	0
3ee559ba8eb75b04619c7887373aef45	0	0	0	0	0	0	0	0	0	8	0	0
206fa0da1c034f34c6dff399ce25d19f	0	0	0	0	0	28	0	0	0	0	0	0
246605a8ef62a9bed5e262d0018383c2	0	0	0	0	0	3	0	0	0	0	0	0
8bd05df8593a64e8e056202029fcdb88	0	0	0	0	0	0	0	0	0	0	0	33
11b4a45ac23c1bcfad561f9fd54a890b	0	0	0	0	0	3	0	0	0	0	0	0
97b2d9b9aa429087411a68edb5c68036	0	0	0	0	0	0	0	0	0	3	0	0
3741be2de8c9dd3c33a4f75207438082	0	0	10	0	0	0	0	0	0	0	0	0
bb59fe8644557a7a3a8b0ecacc474e0a	0	0	0	0	0	2	0	0	0	0	0	0
39fedf6fda47c9dba2d998fd2b6ff372	0	0	0	0	0	0	0	0	9	0	0	0

652324d787f5e34ea407ba12987c968c	0	0	0	0	9	0	0	0	0	0	0	0
e2b6b7987a053f6ad5fc8f5734a493da	0	0	0	127	0	0	0	0	0	0	0	0
d8688ceceadfef80895c339286e15a2e	0	0	12	0	0	0	0	0	0	0	0	0
f7c7ad16279faf798eca997d1151d383	0	0	3	0	0	0	0	0	0	0	0	0
8e3846a82aee8b6448ac6c79cdda68d1	0	0	0	0	0	0	0	5	0	0	0	0
5b21e6b83071e25f9d28b283c3a1ae51	0	0	0	0	0	0	0	0	0	0	0	3
c1662737ea280dd69ced25bc0a021bc7	0	0	0	0	0	6	0	0	0	0	0	0
f9d2ed9b765c9c15c133a4e54820c30c	0	0	0	0	0	0	0	0	40	0	0	0
fd07f7d43d8319ba28d54353e5883cfd	0	0	0	0	0	0	0	0	3	0	0	0
69aa2087e2b64b31d7b0ac81bb38ca3e	0	0	0	0	0	0	0	0	0	0	6	0
8edeb65cfcee1d007de21f16e4773ac8	0	0	0	0	4	0	0	0	0	0	0	0
bfbaffeedb063bc7efbc14b4576c0304	0	0	0	0	0	0	0	0	0	0	0	48
291d7a04c695a48c87ed7d95e4fafc8f	0	0	0	16	0	0	0	0	0	0	0	0
0ec3f3be9f21e6658374e02fdca598ac	0	0	0	2	0	0	0	0	0	0	0	0

f8b81d0b6592f920c694edaf83fefeda	0	0	0	0	0	0	13	0	0	0	0	0
19ce1b09624f1776dd00e4a42233c3dc	0	0	0	0	0	0	0	0	0	0	3	0
fb4b8a88e3395f2186cd3e502bada3ef	0	0	0	0	0	0	0	0	0	0	23	0
f16a58e510625843b26872ae7519e037	0	0	0	0	0	0	0	0	0	43	0	0
73e5144dd021b7c76a480f9cdbd98de6	0	0	0	0	0	0	0	0	0	0	11	0
242622044f7776c3abe24a2a89a61e36	0	0	0	0	0	10	0	0	0	0	0	0
bc86bff88ef4a755fbbde7ef0b955e81	0	0	0	0	0	0	0	0	0	0	50	0
7bdd779aae714a9c218d06e430005fe6	0	0	0	0	0	0	0	0	3	0	0	0
b3153653ecf56fa60ce4296b4a6d350e	0	0	0	0	0	0	0	0	2	0	0	0
5dd78837a5965d01bf374a17cfc8eaaf	0	0	0	0	0	0	0	0	0	222	0	0
fd198ff48e5976e57dcdf158b2b926b1	0	0	5	0	0	0	0	0	0	0	0	0
65a28a20176ce0d0b78fc9ba02c03e0f	0	0	0	0	53	0	0	0	0	0	0	0
ab1651d170c3ac338e827e303496921d	0	0	0	0	0	0	0	4	0	0	0	0
fe808a0aa090125aaafb67e8f2736b07	0	0	0	0	0	0	0	3	0	0	0	0

255374a3ef27b30f47822f8fd df741a2	0	0	3	0	0	0	0	0	0	0	0	0
bae43ecacfc5e05382555f290 d575b8c	0	0	2	0	0	0	0	0	0	0	0	0
21fb0545d5008c763cc52a2f 2b6936ad	0	0	0	0	0	0	0	0	0	0	3	0
929940db9aa299b70a57e76 3ef606f3f	0	0	0	0	0	0	0	0	0	0	21	0
31cef469d01b5553ae50d2a7 d420b6c9	0	0	0	0	0	0	0	0	0	0	7	0
021fa0d1ea525e46f38af6c6b 8964fd5	0	0	0	0	0	0	0	0	0	2	0	0
638ee9d940c1dcac61776df7 7dc0aebd	3	0	0	0	0	0	0	0	0	0	0	0
b4d6904a3c277763992e02c 45b8793a0	0	0	0	0	0	0	0	0	0	7	0	0
1ec5359f36cd176a2cc60c0f8 6810615	0	0	0	0	0	0	0	0	0	0	123	0
4be43b8de92c0a3523dab4e 2ebd32566	0	0	0	0	0	0	0	0	0	42	0	0
80fb52e57a3220c1eb543ce4 1687cf37	0	0	0	0	0	0	0	0	0	0	3	0
0a8548a18f129e03d47eb00f 7ab63da1	0	0	0	0	0	0	0	0	0	0	0	8
96caaca73dd1a49aeb84da4c 9ef13954	0	0	0	0	0	0	0	0	0	0	3	0
0dd179f8c072fe18d0091d09 b79add1f	0	0	0	0	0	0	4	0	0	0	0	0

2ef5c292212d5f6aa672ff4cbe15cba9	0	8	0	0	0	0	0	0	0	0	0	0
b01ae731e5c72700ab141fdeb52c10e9	0	0	0	5	0	0	0	0	0	0	0	0
3d18552dcb2e28a7f23ab41c81e44b2d	0	0	0	0	0	12	0	0	0	0	0	0
f9b8cf6916eeed5659edca9533d8f1bb	0	0	0	0	0	0	0	0	0	0	143	0
8dfa3667d4d3fddef46b02bab3366c6a	6	0	0	0	0	0	0	0	0	0	0	0
0d319da3a2f3237b8d08137784dbb1e9	0	0	0	0	0	0	0	0	0	0	27	0
f36a88fb2c0cfc1eee2ea70dfff2d537	0	0	46	0	0	0	0	0	0	0	0	0
44f627b9df26c1ca70ef784d74d195f8	0	0	0	0	0	0	0	0	0	17	0	0
fbade2e8c638dda37b4d95a7f2aeabc0	0	0	0	0	0	0	0	0	0	0	0	4
4d2d3d4a34a1b94926dfeb49e90603c1	0	0	0	0	0	4	0	0	0	0	0	0
615531425f89d98c813bb75676a6cb85	0	0	0	0	0	98	0	0	0	0	0	0
a6e8bcbbbeef41e97630a5deea254d71	57	0	0	0	0	0	0	0	0	0	0	0
524adef6a5e1133e49b724ccbab58fcb	0	0	0	0	0	2	0	0	0	0	0	0
5a698d943ec6200eb8b69b4c4acf2a37	0	0	0	0	0	0	0	0	0	0	0	6

a501b3bc89d723bdbebe496abe9f7fdf	0	11	0	0	0	0	0	0	0	0	0	0
7ffdab0d0163f77a9d13366a84641203	0	0	0	35	0	0	0	0	0	0	0	0
4a1cfbf87df4cd5ef65242031b57cb8b	0	0	0	26	0	0	0	0	0	0	0	0
a60bb4cd335d865ed74b4f38a69bbbfb	8	0	0	0	0	0	0	0	0	0	0	0
a484f216ff5bf03a65d473326f8763c0	0	0	0	0	0	0	0	0	0	3	0	0
ed7495eb5c7925758bc92f356cdf7586	0	2	0	0	0	0	0	0	0	0	0	0
1ceef397ca0587338422c70a183de157	0	0	141	0	0	0	0	0	0	0	0	0
f885b154545f2503ebb7398656753a50	5	0	0	0	0	0	0	0	0	0	0	0
72ff2d02729e66f3cd88f2cdf9e64148	0	0	0	0	0	5	0	0	0	0	0	0
3990a19f3c94022d7f4da4ee33e73017	0	0	0	0	0	0	0	2	0	0	0	0
872103cecbe39e6f667aea775a200289	0	0	0	0	0	0	0	0	0	0	7	0
521a94c6524de8eb5936a185ec37ea2b	0	0	0	0	0	0	0	0	0	0	61	0
4c3c08aba10bb0d0f1f6957d237c92d2	55	0	0	0	0	0	0	0	57	0	0	0
3d0ab4c6f32ff748c8145aefb574eb5e	0	0	0	0	0	0	0	0	0	3	0	0

a835f690535bcf5baa0d708d a0fbcbb9	7	0	0	0	0	0	0	0	0	0	0	0
b0b3e78363f3552fea1c3d3ff 7835c4d	0	0	0	0	0	0	23	0	0	0	0	0
53fe283f5db0009a79bb1a70 a4614513	0	0	0	0	0	0	0	24	0	0	0	0
058979d7d9775fca421ec16e 0162d244	0	0	0	4	0	0	0	0	0	0	0	0
2700d69af317ad8aed088abb dc077bd2	4	0	0	0	0	0	0	0	0	0	0	0
09e1a182d6f2756dc119de75 8874e217	0	0	0	0	0	0	0	40	0	0	0	0
6a7dbf478b0820739b57ecdb b604c7e3	0	0	0	0	3	0	0	0	0	0	0	0
f6694fc6b2b70fe3c4fde7ccee 1919ad	0	0	0	0	0	0	60	0	0	0	0	0
17a9c8416845618e099ea9e 08b93d974	0	0	0	0	0	0	0	3	0	0	0	0
a3e9c640d74ddf272d2bd60b 1072251c	0	0	0	9	0	0	0	0	0	0	0	0
b70cbf757ff5eb63cfcdb9143 8557431	0	0	0	0	0	0	0	0	0	0	3	0
8eb8fa0ef33e17d5ac44ccca4 12972eb	0	0	0	0	56	0	0	0	0	0	0	0
2c3e436ba2247448be3b8fd1 4075630a	0	0	0	2	0	0	0	0	0	0	0	0
fb64c07ffa63abbcd5a2aedf5 790db80	0	0	0	0	0	131	0	0	0	0	0	0

926310e3be1d3ed10370ecc174d98ab0	0	0	0	56	0	0	0	0	0	0	0	0
ddeecc720b75804126c3783361a58a18	0	0	17	0	0	0	0	0	0	0	0	0
996be0102bf72b28bbd48f10c96c9bbc	0	0	0	0	22	0	0	0	0	0	0	0
427a2ab172b77842942c9fcd134d847e	0	0	0	0	0	3	0	0	0	0	0	0
0affdc4f0d53f9376d52c55af3e4b172	0	0	0	0	32	0	0	0	0	0	0	0
e89ac6155f8384b7dd9485241ca95474	0	9	0	0	0	0	0	0	0	0	0	0
62a294e67ce3c20ae0bc13ab22d952fc	0	0	0	0	0	0	30	0	0	0	0	0
c91baf75514973d056e58f191bc57fe4	0	0	0	0	12	0	0	0	0	0	0	0
bda80b1a9bfe8e9632a0c1b8b5b03ee0	6	0	0	0	0	0	0	0	0	0	0	0
3e898f9f2d7c4964e3796ceadd9e9411	0	0	0	0	0	0	0	5	0	0	0	0
eba6e5040569007b5a024f6e28e07988	0	0	2	0	0	0	0	0	0	0	0	0
613b94ac38590f47cbda9a3c9bf1421e	0	0	0	0	0	10	0	0	0	0	0	0
3ffe94a1a369f3217b9118c55d39a087	4	0	0	0	0	0	0	0	0	0	0	0
22433871013b925eb750615ce75ebb67	0	0	0	0	0	0	0	4	0	0	0	0

97f7ee127534461da486fc6f3d835459	0	0	0	0	0	0	0	0	0	15	0	0
49e82a7fdfa4a38893f6d1f09767fdfb	0	0	0	0	0	0	0	0	0	3	0	0
b9d988c9142354843daa6c10d14a86c9	0	0	0	0	0	8	0	0	0	0	0	0
8187ae6c4fe962c85ef91eac32373a1d	0	0	0	0	0	0	0	0	0	0	5	0
6b8790eb0f3a9665115333e706447f00	2	0	0	0	0	0	0	0	0	0	0	0
382b806d317989997b55ff3e1ecc9af2	0	0	0	0	0	0	0	0	0	0	2	0
cb30ae40abbf3eab88e8c8bc9679d3a7	0	0	0	0	0	0	0	0	0	0	0	33
b32ce0868213d28035c667a7b1076ac7	0	0	10	0	0	0	0	0	0	0	0	0
c56f2b20b502b6b38bcfe7dc044fa57d	0	0	0	0	0	0	0	0	0	0	50	0
4f9497657ae86d7c071765ffe10129c3	0	0	0	0	0	0	0	0	0	0	3	0
210e992994a150898b8a501475aab0ae	0	0	0	0	0	0	0	0	0	0	2	0
fe4a6f03a2b4e059545a6a8174912b6a	0	0	0	0	0	0	0	0	5	0	0	0
020ad3fd37273403470d447dcc625afa	0	0	0	2	0	0	0	0	0	0	0	0
d478f74024d3d52767f3ef1149cdc886	0	0	0	0	0	0	0	0	0	0	3	0

0b41d491e5d8cdc707b2b9b3a1fcfd8e	8	0	0	0	0	0	0	0	0	0	0	0
7c400868cf9b1900f281a087d8a41d89	0	0	0	0	0	44	0	0	0	0	0	0
61a82318c282acb7bb1d540d5e43a2a3	2	0	0	0	0	0	0	0	0	0	0	0
c82dad12a7578ad715fedde0a3ddae04	0	0	8	0	0	0	0	0	0	0	0	0
f17ce05f914a36eea53cb94107c06dab	0	0	0	125	0	0	0	0	0	0	0	0
744631fd4165c775b8c754812f3b2e10	0	0	0	0	0	0	0	0	0	0	312	0
4c514590036b46ea6f882d2878299449	0	0	0	0	0	0	0	0	3	0	8	0
30d3b75808f4d3b1cc3e97371d5c82a4	0	0	0	0	0	0	0	0	0	0	0	11
e1fb99131f741a917c76c045a72c94cf	0	0	0	0	0	0	0	0	0	0	8	0
41ec748c7cedf7c11555d864a4f13f8d	0	0	0	0	0	0	0	0	0	2	0	0
97fc8cd8bf26396c3676f3f5a490daec	0	0	0	0	0	0	0	0	0	8	0	0
cbcf4fb06b4b3abfaa9d4b6f9f33f0c8	0	0	11	0	0	0	0	0	0	0	0	0
1d7fb3f7566573cea6bea883389064a5	0	0	4	0	0	0	0	0	0	0	0	0
431b2684126938ba1479e7a535dab3dc	0	0	0	0	4	0	0	0	0	0	0	0

5757fa8c90ab024ccc7b0da6055d6c71	0	0	0	0	0	0	22	0	0	0	0	0
cbebad29f6a98372c90eb063cadd8e26	0	0	6	0	0	0	0	0	0	0	0	0
778e183c39f9922ff07b7927fc1c7f57	0	0	0	0	0	0	0	8	0	0	0	0
c88a24c65b1c112b4ba3a07de66a5b4e	0	4	0	0	0	0	0	0	0	0	0	0
c1c111792e61a460f255db5b74717bf8	0	0	0	0	0	0	0	0	0	35	0	0
876c9c3d3549dd01fe97683eb6d9ddd1	0	0	0	0	0	0	0	5	0	0	0	0
a31acf256781a081d780fb93839849f2	0	0	0	0	0	0	0	0	0	0	0	8
2eb3814f3bdf387d143c408b4ef87978	0	0	16	0	0	0	0	0	0	0	0	0
73520916a9629a41d46ba18753b472e2	0	0	0	0	0	0	0	0	3	0	0	0
fde26e724380a3e0358640fdd5f2da85	0	0	0	0	0	0	0	0	0	0	118	0
90f61cdb1cbffb52bff94b2fd0dfb15d	0	0	0	0	0	0	0	4	0	0	0	0
b950b80e4d09ee0252dd80719c973845	0	0	0	0	0	0	0	0	0	8	0	0
a5560ff36b4fe0f69d387a34c91a636b	0	0	3	0	0	0	0	0	0	0	0	0
3482fcda1a8fe46fba994ca1b62ae585	0	0	0	0	0	0	0	0	0	0	0	48

417686165d21c15ebc88d3b59734ec88	0	0	0	14	0	0	0	0	0	0	0	0
214fedfeb429ecc519e157264d260f37	0	0	0	0	0	0	0	0	0	0	6	0
8f8dea53490ce533475c201a01188ece	0	0	0	0	0	6	0	0	0	0	0	0
62a3aa50e9815e4844674024f92d94b5	0	0	0	0	0	0	0	7	0	0	0	0
3e02ac0e1fb16de7e3444d5c5a1c97d4	0	0	0	0	13	0	0	0	0	0	0	0
56e59294a611732dbb9667e12664275a	0	0	0	0	0	0	0	3	0	0	0	0
0f31f29be099f6eb8a16ac2236b0e47f	0	0	0	0	0	0	0	0	0	0	0	42
76390291f13db99254c13ba226d69d65	7	0	0	0	0	0	0	0	0	0	0	0
9f713cfca6fe49587b5edfee9ba13b79	0	0	10	0	0	0	0	0	0	0	0	0
0e9ae8016302bb7a8f6e0830bda77b6f	0	0	0	0	0	0	0	0	0	2	0	0
038f44adeb85f6cb07e2f7b4f175ae46	0	0	0	0	0	0	0	2	0	0	0	0
6b8b9aaff484974fd11ae285a8c6ccba	0	0	0	0	0	0	0	0	0	0	4	0
5bf58423e737e6a17d52043606c402c6	0	0	0	0	0	0	37	0	0	0	0	0
eabae59e311ff53303ba0c37e40b535a	0	0	0	0	0	0	0	12	0	0	0	0

025e5c9d4aeed626b2d0654b5cb20b57	0	0	0	0	0	25	0	0	0	0	0	0
dc4d50fa20adec556a452bb788568b28	0	0	2	0	0	0	0	0	0	0	0	0
acdcdd66156579bf1043d3518282a6f3	0	0	0	0	0	0	6	0	0	0	0	0
3c784dc5f0375350d91b0fb5d734b469	0	0	0	0	0	0	0	9	0	0	0	0
00ead1572e5cd8e14f87d3add4b14bd8	0	0	0	0	0	0	0	0	0	0	0	12
f0d3aea8945b5449115480e554dcecb2	0	11	0	0	0	0	0	0	0	0	0	0
44c440be1bcc42049722bc8a71b9f5cb	0	0	5	0	0	0	0	0	0	0	0	0
32cea08cf52b9364d11b098fd5a56a1e	0	0	0	0	0	16	0	0	0	0	0	0
5412a8cdc89e2d5a5a505e6e4466e280	0	0	0	0	0	24	0	0	0	0	0	0
2ea46cd533eff4fa003080ca2ee868d4	0	0	0	7	0	0	0	0	0	0	0	0
b40ed77ec6cfd485aad265a5585df500	0	0	54	0	0	0	0	0	0	0	0	0
c850f6ea2a662db0a1fad5b137a14afa	0	0	0	0	0	4	0	0	0	0	0	0
c802f8c29b8017b1bbab0387d036bdcc	6	0	0	0	0	0	0	0	0	0	0	0
76b96f347d6cef073001858399250791	0	0	0	0	0	8	0	0	0	0	0	0

9589e4f33649ff206e7f9543b7896e13	0	0	0	0	0	0	0	0	0	0	12	0
eb4d5ffc1b8d09a011245a5a18f9680b	3	0	0	0	0	0	0	0	0	0	0	0
7ed585a5186390348e098d9163eebf9b	0	0	0	0	3	0	0	0	0	0	0	0
6e3d51ea803b951a652ed6b6f28e4837	0	0	2	0	0	0	0	0	0	0	0	0
c3d967cf67daacade24a33e053bc845e	0	0	0	0	0	5	0	0	0	0	0	0
a5c08d66453bf9abfe8f966727f504a6	0	0	0	0	0	3	0	0	0	0	0	0
2851c577e3793cc4284c3ea90f2b66fb	0	0	0	0	0	0	0	38	0	0	0	0
84b8178c1d20afb8b381661f6ed73dc6	0	0	0	0	0	3	0	0	0	0	0	0
f4c61900867d5a329d3e3f894ed8343a	0	0	0	0	0	0	0	0	0	0	0	11
5a7dcdc51d0bc15e3ae4056cd9a529cf	0	0	0	0	0	0	0	0	16	0	0	0
21a6a52b76fdbeba39b5e5d59d29c178	6	0	0	0	0	0	0	0	0	0	0	0
d94f8038a0a024435c8b988cd5b8b4e4	0	0	0	0	0	0	0	0	0	0	0	2
d8e71ceb1f924349760491726388ec44	0	0	0	0	0	0	0	0	0	16	0	0
9623ddc3b563b09e5b64399da22528c7	0	0	0	0	0	0	0	0	0	0	12	0

f0a57f3cf5310e97495bc37d9848adcf	0	6	0	0	0	0	0	0	0	0	0	0
14a60dacdac1fd225e06f3ff31b0cb4f	0	0	0	0	0	0	0	11	0	0	0	0
0f6feadc98f40fd1554b6bf5792a1a8c	0	0	0	7	0	0	0	0	0	0	0	0
d94c25a6c6c38ba154dcc91867812575	0	0	0	0	0	0	0	0	0	0	2	0
b9974610a0d8f8569075747db94b21ba	0	0	0	0	0	0	0	0	0	0	18	0
add1a2df5b2e2635e6dceb209cdac0e2	0	0	0	0	0	13	0	0	0	0	0	0
09e0269afa434c47dc74512a74ded33d	0	0	0	0	0	0	4	0	0	0	0	0
e20ddcce4e7ca6b8f8ca20ff32ab7845	0	0	2	0	0	0	0	0	0	0	0	0
ea3a1caf098b60365b9f463601805114	0	0	0	0	0	2	0	0	0	0	0	0
8105d8c3c3261bded58ab10959a89dff	0	2	0	0	0	0	0	0	0	0	0	0
bc0ef01456000badfcb0bcd7b7958597	0	0	0	0	0	0	0	0	0	0	0	11
ca2bccf0d6d3c4140fb80a270a61cc0e	0	0	0	0	0	0	0	0	0	56	0	0
0a65f02625591a197a21bc388a25b6f4	0	0	0	0	0	0	12	0	0	0	0	0
be8439d4a0b58db5d979d68a10a0fb31	0	0	5	0	0	0	0	0	0	0	0	0

d1840a6b055708642069199539d6dbe2	0	0	0	0	0	0	0	0	0	0	13	0
a9a4bc143513178617f19ee827bfc416	0	0	0	0	0	0	0	0	0	6	0	0
4a6f9eaf8dd0ff0970067eae9a8bdd18	0	0	0	3	0	0	0	0	0	0	0	0
02e435a5f7c89c883b7f0337b9fe8944	0	0	0	0	0	3	0	0	0	0	0	0
b6620928e6482998345a71b48717d4f9	0	0	0	0	0	0	0	3	0	0	0	0
339a8c3334f94589b00b063e38ae51ea	0	0	0	0	0	2	0	0	0	0	0	0
49b7b1e6c035abf6e9f2eb957512f435	0	0	0	4	0	0	0	0	0	8	0	0
a900df58b4a8a0fb397c7dd729c5e67d	0	0	0	8	0	0	0	0	0	0	0	0
5aa403729e94493f6c27aa78616d766f	0	0	2	0	0	0	0	0	0	0	0	0
d298cabbe4e7c22bee0f884912078a6a	0	0	0	0	0	0	0	10	0	0	0	0
236d3712364c03c5139898097fa3e17b	0	0	0	0	0	0	0	0	3	0	0	0
ddf4933d6dc653cd4cf325cb01aadf74	0	0	0	0	0	0	17	0	0	0	0	0
d178678e77a7ab370c5a564670bed64f	0	0	0	14	0	0	0	0	0	0	0	0
bbbbc9a19f29692ae38c85bbf74c7ff2	0	0	0	4	0	0	0	0	0	0	0	0

4c45703d40064e435cf008e9931faeab	0	0	0	0	0	0	0	0	0	9	0	0
4f85cc2b82cc26510c14a1b485d3ef8d	0	0	52	0	0	119	0	0	84	0	0	0
d9713e6ccbede77f2381ee391a769df2	0	0	0	0	17	0	0	0	0	0	0	0
a35ca43274f8f0e8ced92f07b9fa7ce5	0	0	3	0	0	0	0	0	0	0	0	0
35bc35bb6bf9aa5726e64dba9e14871e	0	0	0	0	0	0	0	0	0	0	0	2
67e49051b03f62a777d134c951daa3b2	0	0	0	0	0	0	0	17	0	0	0	0
3a05d71502843361e1140824ced5eda5	0	0	33	0	0	0	0	0	0	0	0	0
aed29a49efe462a3dfd89c2419630fa1	0	0	0	0	0	0	36	0	0	0	0	0
2b738240c0b3d96e93aae758eb930411	0	0	0	0	0	0	0	0	9	0	0	0
9cca1ca586787b311b45445780918ada	0	2	0	0	0	0	0	0	0	0	0	0
bd60ae3049deea646e85869a14368dde	0	0	0	0	0	0	0	0	0	0	0	3
7c25fc10cc2fdee8361f63ba7f7cde4a	0	0	0	0	0	0	0	0	3	0	0	0
83d056d57f32bbe0720502e494f160c0	0	0	0	0	3	0	0	0	0	0	0	0
9a4804414c00cc252ac0f1a5fc7d2b9b	0	0	0	0	0	0	0	2	0	0	0	0

047535dbce3f16bf39861209c0656f78	0	2	0	0	0	0	0	0	0	0	0	0
2a02ee3502922f77b3b21cf295e773d3	0	0	0	0	0	0	0	0	0	0	3	0
75dc5569e1942eece84d4416df5d7f18	0	0	0	0	0	0	0	0	7	0	0	0
a53daff5d64730456ce1ed3329e41b94	0	0	0	0	0	0	2	0	0	0	0	0
75535d8f0ce147508448ec50686b8abb	0	0	0	0	0	0	0	0	0	0	2	0
3b75337a75fd16f2adf9848a06badebb	0	0	0	0	0	0	0	0	0	0	3	0
d0972c2cd2d89588d1370c11f1dfee81	0	0	0	0	0	0	0	0	0	0	34	0
44393bef2b48b133e6f118034aa090bd	0	0	0	2	0	0	0	0	0	0	0	0
1817dcc25218bdda8c29acd8d482e51f	0	4	0	0	0	0	0	0	0	0	0	0
d2da6a928fea07f1108c8ba518c63fd7	0	0	0	0	4	0	0	0	0	0	0	0
bb95661acbf3d3cda7c59ea26a6e9ae7	0	0	0	0	0	0	0	0	29	0	0	0
a8ba9c3fd69f68ab84d78e80bf2e9f3a	0	0	0	0	0	0	0	0	0	161	330	0
78aa239ee44613bda2a3a2e8904f9a76	0	0	0	0	0	0	0	0	0	0	8	0
cd31abffa866fc84306a49a580e3af2f	0	0	0	64	0	0	0	0	0	0	0	0

b01376e9f2c2a539689b770509a431fd	0	0	2	0	0	0	0	0	0	0	0	0
911d6bea8f845f04d8da8dbe6f5ac477	0	0	0	0	0	0	0	0	0	0	0	17
4385d95aeafc694039c0bcada948abde	0	0	0	0	0	0	0	0	0	0	0	30
21ed64d5e2aa7a743d668cc30b21a62e	0	0	0	0	12	0	0	0	0	0	0	0
09cef4ba3db6920b529f428506b5ac21	0	0	0	0	0	0	0	0	2	0	0	0
e8f689824f214ed7b536e842ba404b85	3	0	0	0	0	0	0	0	0	0	0	0
8616c21c1bff0a8cf84908f9ce3212e4	0	0	0	0	0	0	0	0	0	5	0	0
532042becf91766fda81ba1282c505cb	0	0	3	0	0	0	0	0	0	0	0	0
be21e5fafaeb7ada018e1ce9c62d38d6	0	0	0	0	0	0	0	0	12	0	0	0
79c78711087d74147375918a66f44f86	0	0	0	0	0	29	0	0	0	0	0	0
5c73d04efbb5ceeccd6cf7e87314112e	0	0	0	0	0	0	0	0	0	0	3	0
c22f81a4cbf8ed80668585f93bca32d9	0	0	0	6	0	0	0	0	0	0	0	0
83855010c5c5b38a0965047837cd0eb1	26	0	0	0	0	0	0	0	0	0	0	0
93cd7804470986b258ed02ab46fcfecd	0	0	0	0	7	0	0	0	0	0	0	0

ff22f9a6ee1a5f4e012a59d89f94bbd5	0	41	0	0	0	0	0	0	0	0	0	0
9d8775761dc6807ff7aa6cbb8f72d595	0	0	0	0	0	0	0	0	0	0	70	0
70434c2e2a37bc023baacc032fc795ee	0	0	0	0	0	0	0	0	0	7	0	0
abfa24d5ddeb3adbf0e159d02862cbef	0	0	0	0	0	0	0	0	0	0	6	0
24c14a6b95913b2be3e3e372401b871a	0	0	0	0	0	0	0	0	0	0	12	0
59153ebcebad1bbbb619fccbe0f111a5	0	0	0	0	0	0	0	2	0	0	0	0
2f7499b4ce96ed6269b459cb9ea0a1f1	0	0	86	0	0	0	0	0	0	0	0	0
eff8a286dcf5f9e68f0201026c1de78b	0	0	0	0	0	2	0	0	0	0	0	0
ca2ef64a4d9666643bc40787c195cec1	0	0	0	0	0	0	2	0	0	0	0	0
89822a056165ccf6c4d8b86070987963	0	0	0	0	0	0	0	0	7	0	0	0
6eb3050d2e8f4f346e84d6239f8efae0	0	0	0	0	0	0	0	0	0	0	8	0
18dfa01be0289d29d66b141d85c11b21	0	0	0	0	0	0	0	6	0	0	0	0
4a67e8ec7b353157b252109380e47575	0	0	0	0	0	0	0	0	2	0	0	0
afe97173dae7e6397635b1583d4bef1a	0	0	0	0	0	4	0	0	0	0	0	0

41c70acd6dbfc021ea8e4f5f4f3704c6	0	0	0	0	0	0	0	3	0	0	0	0
5211a8f00a89b963349583e07614f607	0	0	0	0	0	94	42	0	0	0	0	0
0b893a23acc9680f7096515be818c2d1	0	0	0	0	10	0	0	0	0	0	0	0
daf4e497331c72c13057de7306293275	0	0	0	0	0	0	7	0	0	0	0	0
629a4f5c9a2f2174b03fdd237ac9dd0f	0	0	0	0	2	0	0	0	0	0	0	0
f9a6e0a5ef07dc5977bafd1b88253de4	0	0	0	10	0	0	0	0	0	0	0	0
ae14abb0deb932febd407dbe1e5a1138	0	0	3	0	0	0	0	0	0	0	0	0
d9107c281f66767bfab8aeb33a9831ec	0	0	0	7	0	0	0	0	0	0	0	0
3d5f576e9e7ff9b93d96d1725febd432	0	9	0	0	0	0	0	0	0	0	0	0
431aaf4a14326883501809daec01500f	0	0	0	0	3	0	0	0	0	0	0	0
86ab83640c5cdacf6b24f4c7d042fae0	0	0	0	0	0	10	0	0	0	0	0	0
9ff9d5d3ca6406f1d0e9e20e418dcd60	0	0	0	0	0	0	11	0	0	0	0	0
b5158e30ad625992efb365575ba1ae63	0	0	0	0	0	0	0	2	0	0	0	0
b30f4a3cb3ec827400644b92522a31da	0	0	0	0	0	0	0	0	0	3	0	0

87c1a0563e958d4547cd1035f29b0a29	0	0	0	0	0	0	0	4	0	0	0	0
c95ea99195adf57bed9c748a9b4604e9	0	0	0	0	0	0	0	0	0	0	12	0
5aa17814fab6b34bbb511b2c95505e4c	0	0	0	0	0	0	12	0	0	0	0	0
579d4727f540a260c3d0f3c2d117a893	0	0	0	0	51	0	0	0	0	0	0	0
76bbae9e8c1b6d7240fdc1007f4b5c59	0	0	0	0	0	0	0	0	0	2	0	0
acfbbafa833fe3d031747be2fb2ba772	0	0	0	0	0	33	0	0	0	0	0	0
cb8117ca849234bc622ebe5a74353652	0	0	0	0	0	0	0	2	0	0	0	0
749db99c2140f294c6e25d9f63e76017	0	0	2	0	0	0	0	0	0	0	0	0
fb4ac95d2dd478b88adf94ca28769bb9	0	0	0	0	0	0	0	0	0	3	0	0
41e3c728e98f7ec6181d6380dcae7162	0	0	0	0	0	10	0	0	0	0	0	0
44b443da4934ea3114c0ace6acf1c0c6	0	0	0	0	0	0	5	0	0	0	0	0
8fbef70d9b847f7c0bb7356b4c9a8edd	15	0	0	0	0	0	0	0	0	0	0	0
72a574127931cc9d9a392f524c1be394	0	0	0	0	0	0	0	0	3	0	0	0
1f010c0ed192cc98b73b00b8d37c9337	0	0	0	9	0	0	0	0	0	0	0	0

c156a586a89bcb27781cc6da03be26fd	0	0	0	0	0	0	0	0	0	3	0	0
8d233ab74cbee9e1d89c61e84d931ef1	0	0	0	0	0	0	0	0	0	2	0	0
b641016caa837a8d79f7987c9a5e379d	0	0	0	0	0	0	0	0	0	9	0	0
d2ce5d185883d5fec900498ff5acc5dd	0	0	0	0	0	0	0	19	0	0	0	0
6a6bd3340f35a1311770fb8f2f3f98a9	0	0	0	0	0	0	0	0	0	0	82	0
fcd8e22e9c1ba452d79a5912a3ef5948	0	0	0	0	0	0	0	0	0	0	0	6
147d2edcbad89d69cd09d510790af795	0	0	0	0	0	0	0	0	2	0	0	0
4b96d07bb27425ea44039245e972058a	0	0	0	0	0	4	0	0	0	0	0	0
2bd2256c2b0b1e2d90195d5b7f1c211e	5	0	0	0	0	0	0	0	0	0	0	0
98733c8ff9f6a4541fcfec6bf69f84a7	0	0	0	0	0	0	2	0	0	0	0	0
59e3defbedaf5330d534fa1885424841	0	25	0	0	0	0	0	0	0	0	0	0
da5273835263074dc46e410fd89967ac	0	0	0	0	0	0	0	0	0	2	0	0
e0f20436cbfac017f1f6d67d962009a6	0	0	0	0	0	0	0	0	0	0	0	10
88fce103fd2f8521918f5db375c46818	0	0	0	0	0	0	0	0	0	0	0	9

d5d4f870ca6d36a5b8a1a802834305a0	0	0	0	0	0	0	0	0	0	0	4	0
e4750beb53ef534fb6bf074d1557d80a	0	0	0	0	0	0	0	0	0	0	2	0
dd345bb6281cfc43a34d503e29463c29	0	0	0	0	0	0	0	0	0	19	0	5
3af7d38b8f405d82ee67848a73873d71	0	0	0	0	0	0	0	0	0	0	14	0
1f8dae82f9b16cddffafaae4234cae66	0	0	0	0	0	0	0	0	0	0	0	5
464d80c5504bb52eebfb3a2c7fa8bc07	0	0	0	0	0	0	29	0	0	0	0	0
a880fe6f3b8122c8b222b54bfefa9b1f	0	8	0	0	0	0	0	0	0	0	0	0
a047fb7332e0d290af161362433d01e8	0	2	0	0	0	0	0	0	0	0	0	0
c3b7f0b0599d4c3e836955203b6d3119	0	0	0	0	0	0	0	0	0	0	6	0
2d1ccaf2260cc0dcb46b8add38a56c20	0	0	0	0	0	0	0	0	0	0	3	0
2db5d1da469d374443bb9d91089be2b5	0	0	0	0	0	9	0	0	0	0	0	0
8ad631e816615033ca6f509ad1f93e9c	0	0	0	0	0	0	0	0	0	0	0	75
c62521cfadf0fc0f1f0a4decc324177c	0	0	0	0	0	0	0	0	0	0	5	0
be0131add1d9bcf1fd479b85358a4128	0	0	0	0	0	2	0	0	0	0	0	0

b03a94f8eabce1656fa29a9601e7a9f2	0	0	0	0	0	0	0	0	0	0	0	3
bd90984adb93ff3ae3cd87e400eed825	0	115	0	0	0	0	0	0	0	0	0	0
5fb12ccb677de6a6bc502105994b9957	0	0	0	0	0	0	0	0	0	0	0	7
e6a9e1980d028d7d1a51d135c24011fb	0	0	0	0	54	0	0	0	0	0	69	0
69b3a4b41a3139efb43dd3515b636139	0	0	0	0	0	0	0	0	6	0	0	0
b58c741f240ed5debfa3d69b19e76d32	0	0	0	0	0	0	0	0	0	0	5	0
84c07aace8b631f3ee14d9ac2b85acc3	0	0	0	0	0	0	0	0	0	0	0	3
9c37314328ed3fa3aa5474ed808e4c40	0	0	0	0	0	0	0	13	0	0	0	0
6c8757b5709213889074dbf28a8159f2	0	0	0	0	0	5	0	0	0	0	0	0
9bf0537011066e57ba9eed64bb1319cc	0	0	0	0	0	0	0	0	0	0	11	0
c5c6af93f205fef2adc6e9e4ced80dcb	0	0	0	67	0	0	0	0	0	0	0	0
f9575e40b00e7dc75939bf8001ed3dbb	0	0	0	0	0	0	0	0	2	0	0	0
c3b5c0382b4bb128748d18f052e2dbf2	0	0	0	0	0	0	0	0	0	0	0	9
01fe6dfa05ed8db33cc07cc7c97ead0c	0	0	0	0	0	0	0	2	0	0	0	0

f9e6ebe3bf4d89e9dd97681c77e7fe60	0	0	0	0	0	7	0	0	0	0	0	0
be4adab2d87be98d40f40efbae2551b9	3	0	0	0	0	0	0	0	0	0	0	0
f9e595b417feb9499b9aee0eaaa2cc77	0	0	0	0	0	0	0	0	0	0	0	20
2b2701c66fbc9a06dd015fcc069d88ea	0	0	0	0	6	0	0	0	0	0	0	0
1d5937e026da9952a3335c7223933eaf	0	0	0	0	0	0	0	0	0	0	0	6
ac49c0cf7ee50696405b989dd9f93970	0	0	0	0	0	6	0	0	0	0	0	0
9686fb9f288bd8e2a54948bbce9422e2	0	0	0	0	0	0	0	0	0	2	0	0
dd1fdaf25a8ee5d66d807aa4feb4bf77	0	0	0	0	0	0	3	0	0	0	0	0
d194d0b21686ade7af284f10bec43439	0	0	26	0	0	0	0	0	0	0	0	0
ca93d49bbe7bbe4f07ce74bf63b7c38f	0	0	0	0	0	0	0	0	4	0	0	0
3809c5d831892c2642b564a0e3b13700	0	0	0	0	0	0	0	0	0	0	17	0
fa21929f54ce9e842c5fae47e30c15af	0	48	0	0	0	0	0	0	0	0	0	0
91b0bdd0c6eb19bc205182ac2b1a6786	0	0	0	0	0	0	0	0	2	0	0	0
dc04394894f14b21dc5ebf3493284bc3	0	0	0	0	0	7	0	0	0	0	0	0

25872bf33de9a4ace556aeba95ab7682	0	0	0	0	0	0	0	4	0	0	0	0
97fcc201226a950897af67d0da9f2ab9	0	0	0	0	0	0	0	0	0	0	2	0
43509ca24ae11aebbec8f55746d51f55	13	0	0	0	0	0	0	0	0	0	0	0
7a80825c24ff097a6350a45193abb504	0	0	0	0	0	0	0	0	0	0	5	0
3fb007b6473de3f60a092682c6363c40	0	3	0	0	0	0	0	0	0	0	0	0
45d2db4f3ff3f411422fe754b6ebcc75	0	0	5	0	0	0	0	0	0	0	0	0
1e462b749d373191368c621c967098e6	0	0	0	0	2	0	0	0	0	0	0	0
18b5fd37312a11195ad631c6ef292b01	0	0	0	0	0	0	0	8	0	0	0	0
1a1de6bea22e91f0124b95091f8117e9	0	0	0	0	0	0	0	0	0	0	17	0
8648db23812347aff2a0d66821f531b3	0	0	0	0	0	0	0	0	0	0	6	0
dc49d1d78d39ec30c6dbd3cb5592a5fc	0	0	0	0	10	0	0	0	0	0	0	0
ab2474d0299ee41efb8af5f1d37e6fa1	0	0	0	3	0	0	0	0	0	0	0	0
c67119d8b9c08798a350025e73eb9849	0	0	2	0	0	0	0	0	0	0	0	0
c5162af4d2e8b5eac3f27c99f686c656	3	0	0	0	0	0	0	0	0	0	0	0

9a57bba7464e310f94ab63a74a6844ce	0	0	0	2	0	0	0	0	0	0	0	0
437ec3b6de95463269e2500f17f48fa5	0	0	0	0	0	0	0	0	0	2	0	0
333f04af589c473ed1d12d3c4a564363	0	0	0	0	0	6	0	0	0	0	0	0
1a3dd4559b4fe9603d725efb4adec224	0	0	0	0	0	0	0	0	0	0	4	0
0cd0f6e294da3ad6b0a30cdee36fac08	0	0	0	0	0	0	0	0	0	0	0	6
ac15c0207d7f2c1d9f545240271ff519	0	0	0	0	9	0	0	0	0	0	0	0
cfa1e22e8ecf113fbc96c2affb42fb4e	0	0	0	0	0	5	0	0	0	0	0	0
9bb0d5059235393ea3b2740280ddbc08	0	113	0	0	0	0	0	0	0	0	0	0
b3be7e2514b990453298a7375db70dc4	0	0	0	0	0	4	0	0	0	0	0	0
050ec71da7ba6c457975d3a795a76cb8	0	0	0	0	0	0	0	0	0	0	3	0
ebfb3d43c1014a47eb11e04207cae1fa	0	0	0	0	0	0	0	0	0	3	0	0
9ecf247be26dca6fd386e8b837d87fb1	0	0	4	0	0	0	0	0	0	0	0	0
a2a4acf440bf17279494d0e51ab106c6	0	0	0	0	70	0	0	0	0	0	0	0
bdec1b5389b43ed764f53783a01aeaa3	0	0	0	0	0	0	0	0	0	3	0	0

9e04f36211d2b5dc472d2074efa13155	0	0	0	0	23	0	0	0	0	0	0	0
3f69a922742161b190218809c96d71e1	0	0	0	0	0	0	0	2	0	0	0	0
57282115616a764216207f357c9fc77c	0	0	0	0	0	2	0	0	0	0	0	0
dda5f22f57edaf79a373b297231e4094	0	0	0	3	0	0	0	0	0	0	0	0
aba3a51114861d1ac25dc2d67637cb13	0	0	3	0	0	0	0	0	0	0	0	0
4fd12cddcd85924d686a9bf92a81f1ff	0	0	0	0	0	0	0	0	0	0	0	3
b449f24f0006644f4349bc5eac106ebb	0	0	0	0	2	0	0	0	0	0	0	0
ff1f6df46a92f6fd8338ccd8aed325b1	0	0	0	0	0	0	0	0	0	0	0	43
94e1a1df6f3e34477208d2fb98ebc691	0	0	0	0	0	0	0	0	0	0	23	0
8fb268b7a34ca50175817dc121d31632	0	0	2	0	0	0	0	0	0	0	0	0
b529c8405a48ecc153e6702472036b30	0	0	0	12	0	0	0	0	0	0	0	0
f830ff0ac2b4f0662883e54c48e21780	3	0	0	0	0	0	0	0	0	0	0	0
7f0620c62eeb77aa5afcda81c87da6d6	0	0	0	0	0	0	0	2	0	0	0	0
3a1ab86f9e0bba4b887bc96774460b12	0	45	0	0	0	0	0	0	0	0	0	0

382a29a052618af24bfff336e8b39690	0	0	0	3	0	0	0	0	0	0	0	0
0d3a4553d218e4685676651eb6523653	0	0	0	0	0	5	0	0	0	0	0	0
8bb8512395a9f3b6442e7318db8661ef	0	0	0	0	0	2	0	0	0	0	0	0
5aee7ac2be97cdf0a3634ebade95b42a	0	0	0	0	0	0	0	0	0	0	10	0
01d1b180541dde9c185fe169356cf262	0	0	2	0	0	0	0	0	0	0	0	0
66e16fd0360b88d80a155cabc227c258	0	0	0	0	0	0	0	0	0	0	8	0
ce549a17fc4e0bd1783df378d96183a3	0	0	0	0	0	0	0	0	0	0	3	0
8ffff90873eb99ed2525f4788fd5f6be	0	0	0	0	0	0	0	0	0	0	0	109
beb528e1d878197dec94f490e2c14e2f	0	0	5	0	0	0	0	0	0	0	0	0
080c43223d3ab8282370d5c673b0ee61	0	0	0	0	0	0	0	0	0	0	0	2
d20c032f7d67078bbf51d2e15c0b3962	0	0	0	0	0	0	0	0	0	2	0	0
730d2907896eb0c1decc4e0014729521	0	0	0	0	0	0	11	0	0	0	0	0
14d4d01541282c6681f7c1b376bdc565	0	0	0	0	0	0	69	0	0	0	0	0
6b4073fc88a8410f428b32175661fb41	0	0	0	0	0	6	0	0	0	0	0	0

d0f258d1a2644d4f4b4c5c70 4967860c	10	0	0	0	0	0	0	0	0	0	0
8f33e251fdf41e4ec04e79fe7 f11d47b	0	0	0	0	0	0	0	0	0	9	0
90bc80221cff5385b4f5cbc03 73f873a	0	0	0	0	80	0	0	0	0	0	0
3ea5bb52b23b45d6c9b48be 99a70e961	0	0	125	0	251	0	0	0	0	0	309	0
bacaee5b63dda72ee1788ad 31158f42a	0	0	0	0	0	0	0	0	0	0	3	0
de4ade0f87b2c17216b66e08 7ddfc7a1	0	0	0	0	0	3	0	0	0	0	0	0
97bfcedc307533cbdab29b20 f4920795	0	0	0	0	0	0	0	0	0	0	6	0
5ce9c3d681404c3ca35c6160 2a9a00e8	0	0	0	0	0	0	0	0	0	0	3	0
3a27a1261653d7c14e2de8e 5c0c807d3	0	0	0	0	0	0	0	0	0	0	0	22
27b3f419ee2cbea74209134b 5b8e1dea	0	0	0	0	0	3	0	0	0	0	0	0
3db6b3f518146e4c0e23edb9 7cc5b551	0	0	0	0	0	0	0	0	0	0	10	0
9f6cdc545c2bb7887373d292 822e8c7c	0	0	0	0	0	0	0	2	0	0	0	0
6a26fdb5b74a7a49b623e8b9 34b121c0	0	0	0	0	0	21	0	0	0	0	0	0
ed9972a18518213d9ca70fbd 62494fe6	0	0	0	3	0	0	0	0	0	0	0	0

9411cb49fa505cbe26418426d877fda8	0	0	0	0	0	0	0	0	24	0	0	0
ff87a4836b40f9bdb408d2722f5036cb	0	0	85	0	0	0	0	0	0	0	0	0
efd0f594f7a2b1eea1a3fde56cd42ce5	0	0	0	19	0	0	0	0	0	0	0	0
f039b3d0dc65c52cd5a945256f4689d9	0	0	0	0	0	0	0	0	0	0	2	0
2edd2d3279d350ab0c8e5cda096fc1eb	0	0	0	0	0	0	38	0	0	0	0	0
f65b8293d2ec57a4f2dbb7dfb2e4aa15	0	3	0	0	0	0	0	0	0	0	0	0
e45037c15b97fc2fe13d7605931e1b44	0	0	0	0	10	0	0	0	0	0	0	0
b3db4e815cb787c4b29bf69511894f09	0	16	0	0	0	0	0	0	0	0	0	0
2be1c581be1e2e6d452e2148776e4ccd	0	0	0	0	0	0	0	6	0	0	0	0
dffea6f5d609390294f41195c6dc4853	0	0	115	0	0	0	0	0	0	0	0	0
e8e34cc9b93728ea609a534ac6794d58	0	0	0	0	0	0	0	0	2	0	0	0
a829435f62ef5475e80ba57459d90a29	0	0	0	3	0	0	0	0	0	0	0	0
69e61cb29d5dc2d31b496906a980c5f1	0	7	0	0	0	0	0	0	0	0	0	0
306b0007507c96592cdd3c7f84de99f9	0	0	0	0	0	0	0	0	0	0	0	45

ad24a9cc29b2bf41b5119075 48a8e23b	0	0	52	0	0	0	0	0	0	0	0	0
b8b6d150b580cc6c6d17db5 d07142dd5	0	0	0	0	0	0	0	0	2	0	0	0
2545c187126d3b89cbb6dd8 4d8abaa52	0	0	0	0	0	5	0	0	0	0	0	0
ee2c8b8e223856edde76cf93 87e5cd1c	0	0	0	0	0	0	0	0	0	0	4	0
b4daee11b2b186ad647dcbb 0ed49834c	0	0	4	0	0	0	0	0	0	0	0	0
5ba3bd8233e966a8a0c676d 193a16b6d	0	0	3	0	0	0	0	0	0	0	0	0
728ce4bb133f563a462756bf 266b6acf	0	0	0	0	0	0	0	0	0	0	0	30
b702c085bd24ff3a239572fe5 fcf0945	0	0	0	0	0	0	0	0	0	3	0	0
88e24ab8862a1a244b83440 bb27f7ff1	2	0	0	0	0	0	0	0	0	0	0	0
aaaf2cac5161eaba5dc61db2f b36d3ed	0	0	0	0	0	0	0	0	0	0	21	0
c768871d2740e394907ae76 3dcfa2b68	0	0	0	0	0	0	0	0	0	0	0	9
4cbd871ed76fd6e4978935b4 553db0cd	0	0	0	0	0	0	0	0	0	0	0	15
15aeedd491215d00b19c4bc 6181b1b55	0	0	0	0	0	2	0	0	0	0	0	0
02fadfc2465d50b8b55337c4 8c9fff5b	0	0	0	0	0	32	0	0	0	0	0	0

952fef61dfbf3fe62abefd702cedc146	0	0	0	0	0	0	0	0	3	0	0	0
77be7b2a86cc35518f03160e34cc02b1	0	0	0	0	0	0	0	0	4	0	0	0
0387de7b6e1cb027100ec409545bf979	0	0	0	0	0	0	0	0	5	0	0	0
f13d4eeca5d205ce0cda49c20724fdc8	0	0	0	0	0	0	0	0	0	35	0	0
630f7bfad51ee5fb748d11af4fe2676b	0	0	8	0	0	0	0	0	0	0	0	0
068c65a4ce1371877a895fcf328d3d82	0	0	0	0	0	0	12	0	0	0	0	0
6fd42a275d704c7500e144a2ef4415e6	0	0	0	33	0	0	0	0	0	0	0	0
b00328ea669e1cf21e8740f2abaea14b	0	0	0	0	0	0	0	0	0	0	7	0
1c1cededc4da783668fdd5d8ea981520	0	0	0	0	0	0	0	0	0	0	0	21
ca71395abf091ec12f751e489d62f817	10	0	0	0	0	0	0	0	0	0	0	0
93caa27d6177bc596102beebaa84c721	0	0	0	0	0	0	0	0	0	5	0	0
cecef708e3c24830fdffe5f015e8dcb3	0	4	0	0	0	0	0	0	0	0	0	0
5e98033ff25fbbfab41c8da0acbf2a3b	0	0	0	0	0	0	0	0	0	2	0	0
b222dbe8bc8a8f8c3784a6f99069a8b9	0	0	0	0	0	0	0	0	0	0	39	0

534ee3c25485e9b59a07904 8ff2c9803	0	0	0	0	0	0	4	0	0	0	0	0
3257bf656dbd75e3138c3e1e 6ee3356c	0	0	0	5	0	0	0	0	0	0	0	0
55b66308e46745a0095b760 68b011a5c	0	11	0	0	0	0	0	0	0	0	0	0
72ae6e945c7f177bf9b8b360 c30b95fe	0	0	0	0	0	31	0	0	0	0	0	0
a32ae876bb02de62856c5e9 143bb52fb	0	0	0	0	3	0	0	0	0	0	0	0
a290239ba8b3c984d4dcc977 8c238d02	0	0	50	0	0	0	0	0	0	0	0	0
660a54b8dc70497ea1712b4 4ea3c65b6	0	0	13	0	0	0	0	0	0	0	0	0
3cbc7ce72a7e6d7b9788013b 429eaaba	0	0	0	0	0	0	0	0	0	0	2	0
251fb9db8b24e09a9c1e2617 3315e895	0	0	0	0	45	0	0	50	0	0	0	0
a4d960c12aacd863aa659ebe 0c0aeb00	0	0	0	0	0	0	82	0	0	0	0	0
5bf5bd48c7091fbd4dfbbc96 41829000	0	0	18	0	0	0	0	0	0	0	0	0
94b0d40c6eed2d7eb7571a7 aa0849fea	0	0	0	0	0	0	0	0	0	0	0	14
514df40440ffac812c03c6465 a5e2783	0	0	0	0	0	0	0	8	0	0	0	0
20290335fc6e9d67e394dbc8 bd054c90	0	0	0	0	0	0	0	8	0	0	0	0

33834f8242e1f136a711c92f1ec41161	0	0	0	2	0	0	0	0	0	0	0	0
38ec32c75f62a9a1f6e542b618fafe4c	0	0	0	0	0	0	0	0	0	0	0	9
d4f2b9568fb31f8c644541900d4fc28e	0	0	0	0	0	22	0	0	0	0	0	0
6c3eef30a0259a2a8cf78f56a5ac178c	0	0	7	0	0	0	0	0	0	0	0	0
bbc4a8685da6f77fc93e2db866fd30d7	2	0	0	0	0	0	0	0	0	0	0	0
1e7b150f761eafaa4633e0242fb8846a	0	0	0	0	26	0	0	0	0	0	0	0
3dcacc8614a45b4a3ce9be099fe53cd2	0	0	0	0	0	3	0	0	0	0	0	0
3367726b93e5d75832097cae3ba5c3a2	0	0	0	0	0	3	0	0	0	0	0	0
aa7953b905fbd30c3a5fc2753fdfe245	0	0	8	0	0	0	0	0	0	0	0	0
f4b4090bdc5afb1fbda86f545de6a849	0	0	0	0	0	0	0	0	0	5	0	0
d081a7349e3ff348590f6560bff23d33	0	0	0	9	0	0	0	0	0	0	0	0
5738ab735f11b572c98d37ceb575ab68	0	0	0	0	0	0	9	0	0	0	0	0
f250d70deabe10e50f416cf7b733e8a6	0	0	0	0	0	0	0	0	0	0	0	19
82b2573d1eba4a7f27bb562c9ab0ae37	0	0	0	0	0	45	0	0	0	0	0	0

1319588d12d0261355b77ffd6b2f8bfc	0	0	0	66	0	0	0	0	0	0	0	0
4a1b41debcb72df268d5e1681df9739f	0	0	0	0	3	0	0	0	0	0	0	0
a51d4c250c12e3ed6729cdf1a937a414	0	0	0	0	0	2	0	0	0	0	0	0
2b75614ba8a816ab5f93fe70ccd3518b	0	0	0	0	0	0	3	0	0	0	0	0
f13f9e27d09217c128a04b0c7fe98366	0	0	0	0	0	0	0	0	0	8	0	0
2c2eee1722b6e5db7ce09ab468825983	0	0	0	0	0	0	0	0	0	0	3	0
2484bce97d70082a1a473620e0fc42c4	0	0	0	0	0	0	0	0	0	6	0	0
bfb65477befd75ac99b530adef2a5497	0	0	0	0	0	0	0	0	0	0	12	0
e905e70098bc9a66d359ad344ef0f4ec	0	0	3	0	0	0	0	0	0	0	0	0
0769bcfb867762a278a92cf1d05a6b59	0	0	0	4	0	0	0	0	0	0	0	0
5683792362879d58a7f08b15de37c581	0	0	0	0	0	0	0	2	0	0	0	0
5730f20ef8eaf25f8d14450d38009246	0	0	0	0	0	0	0	0	0	0	5	0
6eb7a16f4166699eb67ca425e8f16daf	0	0	0	0	21	0	0	0	0	0	0	0
855f6f992623d2f76af5e78ac7543a3e	0	0	0	0	0	0	0	20	0	0	0	0

bbea782a3fdb9ffda0503830ead05e0e	0	0	0	0	0	0	0	0	11	0	55	0
ca7c1f1e94e37de3abd671054f2a33a4	0	0	0	0	0	0	0	2	0	0	0	0
3be4df10ab5aa7b5e275e4bd6121a3a4	0	0	0	0	0	0	0	0	0	3	0	0
26cb36636bbef3ee0a2cdb1e5fb7b02d	0	0	0	0	0	0	0	0	0	0	0	3
7748d9a0ad08bf39ffdd88998b994c82	0	0	0	0	0	0	0	0	0	0	4	0
6f692fca5dbd9e7cf3e358eaad4506a4	0	0	0	0	0	0	0	0	0	0	0	3
fac07c08154dbcd7e75ed86bb8c24d81	0	0	0	0	2	0	0	0	0	0	0	0
5019a2c2add0e57edd290eee565d6558	3	0	0	0	0	0	0	0	0	0	0	0
009c74e440945da685a453a3afe4d883	0	0	0	0	0	0	0	0	6	0	0	0
f54e2c2a1abc9716326f400cf3deffeb	0	0	0	0	0	0	0	0	0	0	33	0
e0291c3f0fcc9cd887f4d4453ea0da8f	0	0	0	0	0	0	0	0	0	0	9	0
b2bf76d0f3eecf4a904226cec5e2d797	0	0	0	0	0	0	0	0	0	0	0	2
7776ac4689c33422b753d3717dee2c5f	0	0	0	0	0	0	2	0	0	0	0	0
7882edcf3a6635a90c6bc12228d0c641	0	17	16	0	0	0	0	0	0	0	0	0

ea273f75164f460f73f95d802ef94d6e	2	0	0	0	0	0	0	0	0	0	0	0
ed17ce3cd4f704d7a9363e648f5f10b3	0	0	0	0	0	0	0	0	4	0	0	0
03fc755df30e0b2141254a512dffae47	0	0	0	0	0	0	0	0	0	177	0	0
998eb008d0da9683e5355913d70fef47	0	0	0	0	0	0	0	0	8	0	0	0
5e3b84f1191eee08aea7b668f36018cd	0	0	2	0	0	0	0	0	0	0	0	0
039f87578bbded41282a6c8fd4988766	0	0	2	0	0	0	0	0	0	0	0	0
8382c970f22d08ef49399d2472e9504f	0	0	0	0	0	0	0	0	0	0	14	0
bf3deab935c6479700f9dc71023dcf16	0	0	0	0	0	0	0	0	0	2	0	0
98e5bf3b1637956a5befcf638f83a34c	0	0	0	0	0	0	0	0	16	0	0	0
7348c7171acb0638fa9d73d8a86da678	0	0	0	0	0	0	0	2	0	0	0	0
56dbb8a6b1d30c90d6791d0a564b0476	0	0	0	0	0	0	0	13	0	0	0	0
8fa139f57ce302c6e4db5b10202901c5	2	0	0	0	0	0	0	0	0	0	0	0
7e7a14c1125760cb0eb1e539c208540e	0	0	0	0	0	0	0	0	0	0	7	0
f18a0479645eb223ab34aba38cee5498	0	0	0	0	0	0	0	0	0	0	0	4

ab9d2db884f8300abf02fbd11ceca399	13	0	0	0	0	0	0	0	0	0	0	0
76781c499294b0fbde83dcb029d67480	0	0	0	0	0	0	0	0	0	39	0	0
77e61dec66ae1e2e3ac22972dcdb172a	0	0	0	0	0	0	0	0	0	4	0	0
557096953ae057905635b67711803f28	0	0	0	0	0	0	0	0	0	5	0	0
f73abc1b6f5f60f6b70017743e48b2a6	0	0	0	0	0	0	0	0	0	0	11	0
cdc8ed8b227e2a29c6e92bc96b35e03f	0	0	0	0	0	0	0	0	0	4	0	0
673dbea9ee691bb9e3e7fdf4e12dba9d	0	0	0	0	0	0	0	0	0	15	0	0
bb6982443472b377f9fd44bf681680d7	0	0	0	0	0	0	4	0	0	0	0	0
dd708f79edf09dff9d3aa1ed43aa40e8	0	0	0	0	0	0	0	0	0	0	10	0
082ff7b7773d6205d649cd6dc401a623	0	0	0	0	0	0	0	13	0	0	0	0
4e619c2635e76f490b901b72ef94e1f7	2	0	0	0	0	0	0	0	0	0	0	0
4a5f7d6326f5a9597d1d1d6cc95a011e	0	0	0	0	0	0	0	0	0	0	2	0
4363801297c712ce9f53d13e70ada74a	0	0	0	0	0	0	0	0	0	0	11	0
b990821732dff01eb3aa6263ae9c8ca6	0	0	0	0	0	0	2	0	0	0	0	0

3a482b25d319615bb1e04daf b8a6399a	0	0	0	0	0	0	0	0	0	0	0	2
ccc168c426ddee57bb7fa68f4 f0e9937	0	0	0	0	0	0	0	0	0	0	10	0
b001104732eeed7477a828fc 23aac414	0	0	0	0	0	46	0	0	0	0	0	0
339c657a67eb027e372edbcc 8dd5ec80	0	0	0	0	0	0	27	0	0	0	0	0
89978b9f93d042691cd5dce1 ca17fe61	0	0	0	0	0	0	0	0	0	0	0	2
9666781336dd32a2d3f449d 7afe69c46	0	0	0	0	3	0	0	0	0	0	0	0
ff87599c5e35f1a454de1026d d2a9202	0	0	0	0	0	3	0	0	0	0	0	0
0bd88b55ae8e1cf4872ad372 d1e22fcc	0	174	0	118	0	350	124	0	325	333	0	369
22c572c4549495cc13531001 6c511d42	3	0	0	0	0	0	0	0	0	0	0	0
e0c85404fa32be3a917ed6c2 7d51330b	0	0	0	48	0	0	0	0	0	0	0	0
4a01eb63e030ac5553158f0e 603fe2de	0	0	0	0	3	0	0	0	0	0	0	0
5d4d20b1ebab0b02451550fc b669c097	0	0	0	0	0	0	0	0	33	0	0	0
068a32bf5a33c324492c8694 dca6fce5	0	0	0	5	0	0	0	0	0	0	0	0
d16b4761148e9af79fc7440a d8155d27	0	0	0	0	2	0	0	0	0	0	0	0

aab13f07bc4ff35d06b8b57d61ad4cf5	0	0	0	0	0	0	0	12	0	0	0	0
66b535aba276acaf64b733700fec42a2	0	0	0	0	0	0	0	0	0	0	20	0
4d9a9cf0348828234a703a433ebdab14	0	0	25	0	0	0	0	0	0	0	0	0
5e48c46bda0a378077830a20d1c0555b	2	0	0	0	0	0	0	0	0	0	0	0
3c3457a7f62ec752c4533cea58a7409f	0	0	0	0	0	0	0	0	0	0	2	0
28d51de9aee70b1cedd5b9b5362e6516	0	0	0	3	0	0	0	0	0	0	0	0
5a11805f96edd0ae03c289f107e50af5	0	0	0	0	0	0	0	0	0	0	0	40
e3c2564a7fc934027e87c39c0c0ce1e0	0	2	0	0	0	0	0	0	0	0	0	0
8115230ad27e0a5c5a93cc37b1f044e8	0	0	0	0	0	0	2	0	0	0	0	0
785513f67b1009c9ab88fbe5e435a844	0	0	0	0	0	0	0	0	0	0	28	0
4d04a7d89a4f614daf10fc60a7459273	0	22	0	0	0	0	0	0	0	0	0	0
5073ca8c98e709b2500e91680c0a205d	0	0	0	0	0	0	0	61	0	0	0	0
9eb574a4cc14f86dd0a5d67c201201de	0	0	0	0	6	0	0	0	0	0	0	0
3f2e10f72bccdf1bc0e39fb0cc4422d5	0	0	0	0	0	0	0	0	0	16	0	0

c737ca7a6234986d3e6977f5374ce35b	0	0	0	0	0	0	5	0	0	0	0	0
15e5f190c5d037bc6b18efec5f4e4c3d	0	0	0	0	0	0	2	0	0	0	0	0
87b7ee988c3245a3b609ec93c62cbf47	0	0	12	0	0	0	0	0	0	0	0	0
fd1265861ae026fe0965f2a6dcd28d71	0	0	0	0	0	0	0	3	0	0	0	0
14df2af2d0898f0a7d0ffb63705457cb	0	0	0	0	0	0	0	0	0	20	0	0
74c0a5900262c9820eb711a2077e7d1e	0	14	0	0	0	0	0	0	0	0	0	0
8cc582bcb80c0d90144d7a2d0f95a56f	0	0	0	0	0	0	0	0	0	2	0	0
69a728a4e53e07ff37c6e5fad1cdfa3f	0	0	0	0	0	3	0	0	0	0	0	0
ede737bab33aa564fff33172a039df59	0	30	0	0	0	0	0	0	0	0	0	0
797fdccce86ac105e1655031610b9d56	0	0	0	13	0	0	0	0	0	0	0	0
32d491f49c4972ed293d04ba34b6737f	0	0	0	0	0	0	0	0	0	0	0	65
774a44dc0d281eed0f62eff8cc8cfe6a	0	0	0	7	0	0	0	0	0	0	0	0
7034467ae263899f6ff2522411b1e22d	0	0	0	0	0	0	0	0	0	0	2	0
5aff896fa84197b6a969d4757bcb92d4	11	0	0	0	0	0	0	0	0	0	0	0

4ba9fcfbea478f5f378a3ef2ebd141f7	0	5	0	0	0	0	0	0	0	0	0	0
40e652bddd272c96bed47b6ad3bd0793	0	0	0	0	0	0	0	0	0	10	0	0
de8f148ed95b3751b40eb88fcdd3411c	0	0	0	0	0	0	0	0	0	0	4	0
d92fa3374b2b015df243e260e51bb2d3	0	0	0	0	0	8	0	0	0	0	0	0
306b71a76d774a6607530e7335ddb951	0	0	0	0	0	0	0	0	0	0	0	3
bfce0edbb461224531c7379531836aec	0	0	0	0	0	0	0	0	0	0	13	0
c69a8292f40745533688035df6a29360	0	0	2	0	0	0	0	0	0	0	0	0
89e20e19aa92dc110fee88a8272ff0dc	0	0	0	0	0	0	0	0	0	0	286	0
f09a1838f8c309def550814abdc95430	0	0	0	0	0	0	0	0	0	0	11	0
66d740a173d0020554536c790fc2f3de	2	0	0	0	0	0	0	0	0	0	0	0
bd6b65cc895276db064b92b7a5ed7c8c	0	0	0	0	0	0	671	0	0	0	0	0
e797e2bd8bb7cd89f9fe082f3f06f802	0	0	0	0	0	22	0	0	0	0	0	0
9e28478d988a33afba965a7667185dc6	0	0	10	0	0	0	0	0	0	0	0	0
4b87567825ea3118f088441995a41536	0	0	0	0	0	2	0	0	0	0	0	0

b74c6e0bdb1f5f83b71a4bfa7066b50a	0	0	0	0	0	0	0	0	0	0	2	0
70ccda63acf289d4b695a19b960e0061	0	0	0	0	33	0	0	0	0	0	0	0
2d3448158f621822370f4339086771ed	0	0	0	0	0	0	67	0	0	0	0	0
471053c4f48f10f2b41c6e4ba676580e	0	0	0	0	174	0	0	0	0	0	0	0
b919ac9f544efa816a7ba5548dca6df6	0	2	0	0	0	0	0	0	0	0	0	0
ba6b0546de5b3017400c138c495d2644	0	0	0	0	0	28	0	0	0	0	0	0
f2f95078425a18d610b8681c15203921	0	0	0	0	0	0	9	0	0	0	0	0
a446118260f96a74e403958b4c7946cb	0	0	0	0	0	0	0	8	0	0	0	0
01bde486f79bad15c4384855af32b08b	0	0	0	0	16	0	0	0	0	0	0	0
dd58d44aa6814b66f30490273ba5e928	0	0	0	0	0	0	0	0	0	0	0	4
0320bb17e3e7daece4ccee60c228b0a5	20	0	0	0	0	0	0	0	0	0	0	0
bb5e98fcb4131d0f73e63c0cfaf322db	0	0	0	2	0	0	0	0	0	0	0	0
4c6a07974f7d92af5d8a3a66bc171aaa	0	0	0	0	673	1072	0	140	0	842	0	388
bbea8e4f0f55ff92d821350abfcb203d	0	0	0	0	17	0	0	0	0	0	0	0

6030fec72887a712f271cd6c1588efda	0	0	0	0	0	2	0	0	0	0	0	0
8f566b78d0586adee57ed98677154d1f	0	0	0	0	0	0	14	0	0	0	0	0
08dc9d0b0de1670db1518d4dc581459e	0	0	0	0	0	64	0	25	0	0	0	0
a5c770548def4b82ac11b3715100d21c	0	0	0	0	0	0	0	0	7	0	0	0
83898757ce86e69917e479fe945cdfd1	0	0	0	3	0	0	0	0	0	0	0	0
e1d66533d6b43ce464451f121105d09d	0	0	0	0	0	0	0	5	0	0	0	0
c29b057bb2e61b37cca8529173bbd295	0	0	0	0	0	0	0	0	0	0	0	3
fdc61504bfc13366a0204b0805b11afa	0	0	0	0	0	0	0	0	0	15	0	0
411135da13bb28d99b3519cd14d14fc3	0	0	0	0	5	0	0	0	0	0	0	0
58ff925b34ea345070b1ebc36c1787f1	0	0	0	0	0	0	0	3	0	0	0	0
7390519b6eb06bb27fb137c0a21e4649	0	0	32	0	0	0	0	0	0	0	0	0
4f1800bb0563a64253c8abf05841a017	0	0	2	0	0	0	0	0	0	0	0	0
26194ea7d3ad69849ad919d57819f9fb	0	0	0	50	0	0	0	0	0	0	0	0
9e3392528659392b1a01e21da4f723ca	0	0	0	0	0	0	0	2	0	0	0	0

55071f73c8fea238425ea150cc634042	0	0	0	0	0	4	0	0	0	0	0	0
0f061c6810cf39c2c0ababc39a35f740	0	0	0	0	0	0	0	0	0	0	2	0
ad9cfa26d2479d01a74c4d927283e25f	0	0	0	48	0	0	0	0	0	0	0	0
91c21942374730aa1ce32335fa292a4d	0	0	0	0	0	0	0	0	0	0	26	0
fdf1b34f42b4506de1f2fbe8b38e4a8e	0	0	0	0	6	0	0	0	0	0	0	0
eb70232c79ca6612687ccce7598c126a	0	0	0	0	0	0	0	0	0	0	3	0
3206b9c460ac00c29b4d31490da8c736	0	8	0	0	0	0	0	0	0	0	0	0
6493118e133b1a65b7f167d372e98caa	0	0	22	0	0	0	0	0	0	0	0	0
fd7f4c63867717edf70bdfb61c864471	0	0	0	0	0	15	0	0	15	0	38	0
7c92ee4b243f9c06624e770551b5fc54	0	0	0	0	0	0	0	2	0	0	0	0
9b33b5440fb0173fdcd1bc5578a68ca2	7	0	0	0	0	0	0	0	0	0	0	0
77870674632769d209015ddeea658996	0	0	0	0	0	0	0	0	0	0	10	0
e08b908fc7807c3d32602ea6656c1d19	0	0	0	0	0	0	0	0	0	0	2	0
8802c839e4f8c373498d4455705f46e1	0	0	0	0	0	0	0	0	0	0	0	189

3778865c5f0028a2a8f685c965e6b341	0	0	0	4	0	0	0	0	0	0	0	0
d0fdd93b820e09f7144a5eb42e86177e	0	0	0	0	0	0	0	0	0	0	0	23
63b769b9cd8b047d05c23e6cb1415f94	0	0	0	0	0	22	0	0	0	0	0	0
62301c2ebfa53672b768bad30942b518	0	0	0	0	0	0	0	0	7	0	0	0
e0f2c24e6a7576384a5fdcbdf4d1712e	0	0	0	0	0	0	0	0	0	0	0	7
6e36bde7ddc97503f8ca8770fb2b2804	0	0	0	0	0	0	0	0	0	0	3	0
bd3704c102d40d4f69203b0dfa173dfa	0	0	0	0	0	0	0	0	0	0	3	0
4f331d8b7f012d860770b9f4f67c87c4	4	0	0	0	0	0	0	0	0	0	0	0
f1af79807c66d7def4b1aae757e0d2c0	0	4	0	0	0	0	0	0	0	0	0	0
fc754d17b058a9a8bca123e5ab00250d	0	0	0	0	3	0	0	0	0	0	0	0
884d8f1454510818b66de03878b934f2	0	0	0	0	0	36	0	0	0	0	0	0
7676b2f18fe5b6cd4e5d345605edefe9	0	0	0	0	0	0	0	4	0	0	0	0
4d7c24d602d4aa74defc3e665417711c	0	0	0	0	0	0	0	0	0	0	3	0
701608bb1ef78ba0b55c49886a379f8f	0	0	0	0	0	0	0	41	0	0	0	0

890042dbc1caf8751cf80814cee4d855	0	0	0	0	0	0	0	13	0	0	0	0
a24a6bece5d2855a8dde783b35605db7	0	0	0	0	0	0	31	0	0	0	0	0
f57314d3586c8fb6c64b5451a3fe765d	0	0	0	0	0	0	0	0	0	0	2	0
50e3732dc98d692b3f817d6e14e124d5	0	4	0	0	0	0	0	0	0	0	0	0
14ac495b960f47beea65d5bc4962617b	0	0	0	0	0	0	0	0	0	55	0	0
e1de46a167ea6767db4c319ced4ba35c	0	0	2	0	0	0	0	0	0	0	0	0
6e070d98e39152e1a3a680515ea5d73f	0	0	0	0	21	0	0	0	0	0	0	0
e6641b2b9a55ee6b31ab9e0b5ce7dc5c	3	0	0	0	0	0	0	0	0	0	0	0
167acbe5fa3208d857282b05aba8d7b9	0	0	0	0	0	0	0	0	0	0	0	4
7d03455d81c5da277fb01a0fb0c4bb26	0	0	0	0	0	0	0	0	0	4	0	0
8500f5f81b30822782a0ad28b97ef7c5	0	7	0	0	0	0	0	0	0	0	0	0
a11e8467ad2837ee1f1af887631d718f	0	0	38	0	0	0	0	0	0	0	0	0
dfb80c095ee9d42c5d22db49fb0f42af	0	0	0	5	0	0	0	0	0	0	0	0
214ff3c3000e09aeeedc0ae14c367d3f	0	0	0	0	0	0	0	0	0	0	3	0

319714e5b88fcde531714cbdbff468ab	0	0	0	0	0	0	0	0	0	0	11	0
f58e3ec1baee3934d071e04b8f85f492	0	0	0	0	0	0	0	0	0	0	5	0
46104573c15323539702e0f865060def	3	0	0	0	0	0	0	0	0	0	0	0
9475b7c9ca086b2ca03e7b4a0661a45e	2	0	0	0	0	0	0	0	0	0	0	0
8e54b487ba652489772a80e3d9880e3e	0	0	0	0	0	0	0	3	0	0	0	0
e3c550fe2108247d6e33c9e07bf04f11	0	0	2	0	0	0	0	0	0	0	0	0
87d51856b072f7fd28b64a20bccf1c40	0	0	0	0	0	0	0	0	0	0	33	0
de2f3a12471977fe44c38fdaf98cd1df	0	0	4	0	0	0	0	0	0	0	0	0
107481d8f500379a320871f984f068dd	0	25	0	0	0	0	0	0	0	0	0	0
0c64108e179f2d945a92b5a4d2f65c18	33	0	0	0	0	0	0	0	0	0	0	0
4478a8963a4a4f24c4ed82e307563510	0	0	2	0	0	0	0	0	0	0	0	0
12225cc191b8c88695475ca4a14ef0e1	0	0	0	3	0	0	0	0	0	0	0	0
676c2c1bdf4c9b74cac7f5fefd19c7d2	0	0	0	0	0	0	26	0	0	0	0	0
159d4e0e5ad7e9cd2a682dd35e1ec892	0	0	0	0	0	0	0	0	0	0	0	47

722fff8c200466f135ba618fcf c0f9d4	5	0	0	0	0	0	0	0	0	0	0	0
5383b3da210586cb39bca00 469c52028	0	99	0	0	0	0	0	0	0	0	0	0
c6c95cb122753cdae99b5b51 b5f9fd85	0	0	0	0	0	0	0	0	0	0	0	2
6bcbb108767466abfc679715 5e6feb26	0	0	0	0	101	0	0	0	0	0	0	0
ac314705fcda56dfbb8b6699 d3718a23	0	0	0	0	0	0	0	2	0	0	0	0
0da809a2106d230767e0e8e b6e5055f6	0	0	79	0	0	0	0	0	0	0	0	0
e672d4f7190e8c442a58e856 7e01d758	0	0	0	0	0	0	0	0	2	0	0	0
3dceb872c8d41d9fff693a646 b898a9c	6	0	0	0	0	0	0	0	0	0	0	0
98bf965fb44a5d50e5dd76be e9179c9f	0	0	0	0	2	0	0	0	0	0	0	0
20ea7cd4203af98f8b5401bd 8d2162ab	0	0	0	0	0	0	0	0	23	0	0	0
694d5f1c16f3fe91e858b4e59 e0e06a7	0	0	0	0	0	0	0	0	0	0	9	0
8cda52d160d4e76ea161347f 0d929ad5	0	0	0	0	0	0	4	0	0	0	0	0
a42550e3eed54457ac372db 2fa42bd17	0	0	0	0	0	0	0	0	0	2	0	0
37b94bef73c62bd5000ca177 9cd7c123	0	0	0	0	0	2	0	0	0	0	0	0

c1b655e1748a00b46d6eb4b07f3d0a0c	0	0	0	0	0	0	0	14	0	0	0	0
8a88a13e6fa40f4a9340197cad32e325	0	0	0	0	0	0	0	0	0	0	11	0
6461b4ad42346d93a01ca3880caab485	0	0	0	0	0	0	0	0	0	0	9	0
ef06548fcef38286d17a143048e15da4	0	0	0	0	0	0	0	0	15	0	0	0
68d93e7818c8fadd9dec9e2349381220	0	0	0	0	0	0	0	0	0	3	0	0
05e00fa6506c3a1e4604fb709784b480	0	0	0	0	0	0	0	0	3	0	0	0
d4fb76b9e8cd8830e443f0815ae4e942	0	0	0	0	0	0	0	0	2	0	0	0
f62347026d286a7ec488beba41bbb52a	74	0	0	0	0	0	0	0	0	0	0	0
f84c9070abdffa35e9de638aefca702e	0	0	0	0	0	0	0	0	0	0	0	6
466c326c31148cc7f66d53d11e1a4090	0	4	0	0	0	0	0	0	0	0	0	0
436cd7db7ec6c30d5cdca3b9da1563b2	0	0	0	6	0	0	0	0	0	0	0	0
94cc66627986300e5348aa18b5005818	0	0	0	0	0	0	0	0	0	0	0	5
7dab418446ffca8b12a7915702511e12	0	0	0	0	0	4	0	0	0	0	0	0
e823010ff93de7bd53183fefe8394ebb	0	0	0	0	0	0	0	0	0	0	0	3

f16be42781ddb06affdbe3ed68a2dfaa	0	0	0	0	3	0	0	0	0	0	0	0
99e9c487c1e82376261224ed2f88640c	0	0	0	3	0	0	0	0	0	0	0	0
3138feeab76ea44e6281fabb49b49c22	0	0	0	0	0	39	0	0	0	0	0	0
c897cf18d82604d6de88afa6de9538be	0	0	0	0	0	0	0	0	0	7	0	0
8c9efd0f3aa91d13da6ce4235e9d7606	0	0	0	0	0	0	0	34	0	0	0	0
0fc83a00976b423608aff65f4a839f47	0	0	3	0	0	0	0	0	0	0	0	0
26a4c19dda0bea8b2ac3dfe449f688bf	0	0	0	0	0	0	0	4	0	0	0	0
0064e612933d61006b7261dcec86b56e	0	0	0	0	0	0	0	0	0	0	4	0
3087ce564afb2a0daec7e6a41bc9a7f3	0	0	0	0	0	0	0	0	0	0	3	0
2cc439f32618e73df12de13d26a3f8ea	0	0	0	0	0	0	0	0	0	0	3	0
0eae442bbfddc0b5c098a1d9ecdb1474	0	0	0	0	0	0	0	0	0	0	4	0
97ffff188053aa60cb74fca7d808a135	0	0	0	0	0	0	2	0	0	0	0	0
e3dbdb4cac3f8581402fc3e31d87e753	0	0	0	0	0	0	0	0	0	0	4	0
e156c88e6c579af7f1f4d86f0f9bffc7	0	0	0	4	0	0	0	0	0	0	0	0

2cd98bfe9a4079c255942be42871f5be	0	0	0	0	0	0	0	0	0	2	0	0
21efdf7efc6ee84da76b835b283ffb49	0	0	0	0	0	0	0	0	0	0	0	12
65834b173febf23668b32573f3c006b3	0	0	26	0	0	0	0	0	0	0	0	0
3ce29cc9a1ef40d974f9d80604721078	0	0	0	0	0	0	0	0	5	0	0	0
0c7becff083bebf872f1273d1ad4d4d4	0	0	0	0	0	0	0	0	0	24	0	0
dbb5016e97206ab7a29050b764f743eb	0	0	0	0	0	0	0	0	3	0	0	0
62731b4fe708695d9efc6de50bb157f0	0	0	0	4	0	0	0	0	0	0	0	0
97b3014bb81b1170a68bdaabe5fb9b30	0	0	0	2	0	0	0	0	0	0	0	0
1583582bbda2eb521f4fb5991ef15568	11	0	0	0	0	0	0	0	0	0	0	0
5e51849d5ebc865b3fdc72ed6f283e9e	0	0	0	0	0	0	0	2	0	0	0	0
071e759b40dfea613a8b290724610408	0	0	0	0	0	2	0	0	0	0	0	0
5e60208de0e700f9ca3759b3ce86eb8f	0	0	0	0	0	0	0	0	0	0	5	0
a57e2211aae9e97c82c97f4f092c6d8a	0	0	2	0	0	0	0	0	0	0	0	0
b03c61ee7780fd17802959d375106d66	0	0	0	0	0	0	0	0	0	0	3	0

7ceb5756f46d1b5f42adfeb1a6cd1350	0	0	0	0	0	0	0	0	0	0	0	3
beaf7a4a8e8734e398d538c67d4df7e8	0	0	0	0	0	0	0	0	0	0	8	0
615c746e8c2c5954ad6b845abea3460a	0	0	0	0	0	0	0	5	0	0	0	0
813310f1063336a851034881ead56cec	0	0	0	4	0	0	0	0	0	0	0	0
8b45d360b4a257db7d2028cde8d54cbf	0	0	0	5	0	0	0	0	0	0	0	0
d131d45184301104b6b0e52e1a58ed5c	0	0	0	0	0	0	24	0	0	0	0	0
6c8358ebfe5a6b36bf34910f99dfd473	0	0	0	0	0	0	0	0	0	0	0	2
765462cd4a8d7b63476851e4d73a9cdc	0	0	0	4	0	0	0	0	0	0	0	0
507572a2b26b9cfa28b676648b3eb694	2	0	0	0	0	0	0	0	0	0	0	0
a90adc7bcc25005fa04299d77a94bceb	0	0	0	0	33	0	0	0	0	0	0	0
c751c18b00e65dbaa30cf12721a1e795	0	0	0	0	0	0	0	0	0	0	0	5
55c336b4e2d2d614ec5a895c670836a6	0	0	0	0	0	0	0	0	0	0	19	0
e86c2cfe6d822fae9e860616b0be87e2	0	0	0	0	0	0	0	0	0	0	3	0
c83aa5dd1a03a90281ebc4b9bfd399b5	0	0	0	5	0	0	0	0	0	0	0	0

0376bf66174631c50002e25ee82f62ca	0	0	0	0	0	0	0	0	0	0	0	47
38ae3435834860a6d950749bbc1e6cda	0	0	0	0	21	0	0	0	0	0	0	0
8bb71408c5222630e01af27f928b4ec8	0	0	0	0	15	0	0	0	0	0	0	0
a450f26a8980de78d0ffa12f982f1521	0	0	0	0	0	0	0	0	0	2	0	0
2199a0f7694adba7f081e7d4aa0f1634	0	7	0	0	0	0	0	0	0	0	0	0
54961197373f2b9325032898779cb698	0	0	0	0	0	0	0	0	0	0	0	148
597ef5fa2a70dee0e4f935cdc0fa5f6c	0	0	0	0	0	4	0	0	0	0	0	0
11e0d3584342226dc8f7b539b3ceccd0	0	0	0	0	0	0	0	3	0	0	0	0
0fedbbee8e9ad18427279d496e207556	0	0	0	0	0	0	0	3	0	0	0	0
f854db4383b33e1e4bfef4236921e5c8	2	0	0	0	0	0	0	0	0	0	0	0
04c2af5da78b0bddbf7e3f4e2075ba45	0	0	8	0	0	0	0	0	0	0	0	0
cafa1ca9e58d3f242a51e70b6c5f96f1	0	0	3	0	0	0	0	0	0	0	0	0
82a611d25105c93732ea843cffab9a18	0	0	0	0	0	0	0	0	0	0	0	13
6263fcac5615eb324bf8b4894d14fb0c	0	0	0	0	0	0	0	0	3	0	0	0

4213a68a1470e31f9b9d57fa347d36b2	0	0	0	0	0	0	0	0	0	0	9	0
7e54f7c423b650f29871561235e2347d	4	0	0	0	0	0	0	0	0	0	0	0
f987f3dedc90ed7ec29eb77829eb3251	0	0	0	0	0	0	0	5	0	0	0	0
c4699a7e3502b6742c056e7db2667223	0	0	0	0	0	0	0	0	0	0	0	12
648161c158b898a29a45f93a2f2ef194	0	0	0	0	4	0	0	0	0	0	0	0
83ab80c7d9d1a94dab00774316f92065	0	0	0	0	0	0	0	10	0	0	0	0
948377fa48863e9093e1da32b599440b	0	10	0	0	0	0	0	0	0	0	0	0
d4b59211f6a0f151686d6a2e91c3df90	0	0	0	0	0	0	0	0	0	0	66	0
cf03144bebc065741168adb40ee2863a	2	0	0	0	0	0	0	0	0	0	0	0
1900aa08a59bd1313e1263e80013eb84	0	0	0	0	0	43	0	0	0	0	0	0
d2fdc3f161172c8c77ee9f0069a81e58	0	0	0	0	0	0	0	0	0	8	0	0
ca57e979a01ff528fd6590b124009825	3	0	0	0	0	0	0	0	0	0	0	0
6d131041aa338b70d17143239b931dc9	0	0	0	8	0	0	0	0	0	0	0	0
837d225a27b7c1ee0b8a37af80e416c9	0	0	0	0	0	34	0	0	0	0	0	0

3d99da3b95a0332cad491b6492cb9529	0	0	0	0	2	0	0	0	0	0	0	0
cda14c55b341335b302e60c21f2a2834	0	0	0	0	0	0	4	0	0	0	0	0
45115641276fbebbb3ab90bf2996a1f4	0	0	6	0	0	0	0	0	0	0	0	0
2a2717c4c1b0ac993d983c0bb2c38b2d	0	0	0	3	0	0	0	0	0	0	0	0
73b0bb5d2a34fc289a1b6589a0badf09	0	20	0	0	0	0	0	0	0	0	0	0
bf96575520200a62d59a1e01cbfa470f	0	0	0	0	0	0	0	2	0	0	0	0
758f32f6c4bd00d6ef916f41a1ab966c	0	0	0	0	0	0	5	0	0	0	0	0
4b5176eda2cb531770e4ee47c090bfda	0	0	0	0	0	0	0	4	0	0	0	0
96b3536ae0a3b9b9523c0688d3df6a4f	0	0	0	0	0	0	0	0	0	4	0	0
35f837eac5807c04c21ca2ad731844ae	0	0	0	0	0	0	0	0	0	0	10	0
18529236224d5edc1db9b6fcde09ae5d	0	0	0	0	0	0	0	0	0	0	5	0
77fa9537310bd806dd0868d413a809e3	0	0	0	0	0	0	0	0	6	0	0	0
6e5511a9c581434bc9d42fcff56f78a2	0	0	0	0	0	0	0	0	0	0	0	15
82e47b8e3b8667e3bc65be838a6f32a9	0	0	9	0	0	0	0	0	0	0	0	0

92f584a4c2862ca8f93dc87faed9d196	0	0	0	0	0	0	0	2	0	0	0	0
967e45b7884588acc2d71780f0f66b32	0	0	0	2	0	0	0	0	0	0	0	0
eb0241e8715ab7c5b385d1f50d1555c5	0	0	0	4	0	0	0	0	0	0	0	0
27b7fe54c1ba062d1e3d910d0997972a	0	0	0	0	0	0	0	0	0	0	2	0
8c2ebba5c16d5c0d7e7b827bd44a2e54	0	0	0	0	3	0	0	0	0	0	0	0
de1e20e294e3cdd5362f9a4d9003cdf8	0	0	0	0	0	0	0	0	6	0	0	0
ddabfbe41d230b301e887f5b38a4b62d	0	0	0	0	0	0	0	0	0	0	22	0
19104d3e2098a63ff200114722ce48eb	0	0	0	0	0	0	0	0	3	0	0	0
323357c20e70ed1f6267c7725285437c	0	0	0	0	0	0	0	13	0	0	0	0
8db01ad5cc67e1e6c5fa6d4fbbf964a8	0	0	0	0	0	0	0	0	0	4	0	0
63588a2c47823c085b6aee3f04ed83f5	0	0	0	0	0	0	0	0	0	2	0	0
cef8675212f227ead4f5fb50641e9353	0	0	0	6	0	0	0	0	0	0	0	0
35ef7f129664fb510eb00a215024ba58	0	0	0	0	0	0	0	0	0	0	22	0
2600ededb18341accb2755d576e55c32	0	0	0	0	0	0	0	0	0	0	7	0

b9c0fdccf037759c20c983ee3624ca6c	0	0	0	0	9	0	0	0	0	0	0	0
e16ce0534e52f77d110cb72d31afdf10	3	0	0	0	0	0	0	0	0	0	0	0
05750917a2613debdaafbf9fb4bd1d12	0	0	0	0	0	0	0	0	0	0	4	0
1576202a1d17df97f6ead050c6957a04	0	0	0	0	0	0	0	0	0	6	0	0
e02e6070109ba668d4f3190a08bbcf2a	0	0	2	0	0	0	0	0	0	0	0	0
bb8d5349c7c01098e78383cec59e249e	6	0	0	0	0	0	0	0	0	0	0	0
d410ea3fd6d51e4c0d2b481c46db74f7	4	0	0	0	0	0	0	0	0	0	0	0
2a4f2721dc441e3368e0197d3f769f17	0	0	0	0	0	0	0	0	0	0	0	4
0550623b8e79d3f8df6c7281a9c5d997	0	0	0	0	6	0	0	0	0	0	0	0
9e64e09adae3d6f15add7bce2bb9aa11	0	0	0	0	0	6	0	0	0	0	0	0
418cb0347425291893246a7b34826dbe	0	0	0	0	0	0	0	0	6	0	0	0
d362732ff9a737b4868374a0309a118e	0	0	3	0	0	0	0	0	0	0	0	7
9de1c8eee400a98446485a35ca05c1fb	0	0	11	0	0	0	0	0	0	0	0	0
6e8196181b24b702977cb745f8edea14	0	0	34	0	0	0	0	0	0	0	0	0

300ccd6ad3f4bac9adccef0a0e390499	0	0	0	0	0	0	0	0	0	0	11	0
8270696a029af8e83c7d7c2a1bd03056	0	0	0	0	0	6	0	0	0	0	0	0
72a1108b224d9e3d451f1fda6e7fb48c	0	0	0	0	0	0	0	0	0	3	0	0
7c16daccfa77aa24e6416ec3425fcbc4	0	0	2	0	0	0	0	0	0	0	0	0
e3efced744071e37a816471df25dedaf	6	0	0	0	0	0	0	0	0	0	0	0
9af3bc97fff94fc458ee1fb9d4b30e81	0	0	0	0	0	0	0	2	0	0	0	0
800b522c60c99d4981dd0ee31ab26ee3	0	0	2	0	0	0	0	0	0	0	0	0
19f45656e8903460fe88a53ec3895222	0	0	0	0	0	0	0	0	0	0	0	6
e09b4f2a57f507d7a7e7f89b1edb47d3	0	0	0	0	0	0	0	0	0	0	0	4
708b74618eb05cad2d9eaf183c0efe11	0	0	0	0	0	0	0	0	0	0	40	0
90986c666ae768dda54041eb1d91c1f9	0	0	0	0	0	0	0	0	0	8	0	0
79a7a33b48bb83b6ff677b8397446349	0	0	7	0	0	0	0	0	0	0	0	0
ab33e14036409f69285a6b49b8da8756	0	0	0	0	0	0	0	0	0	0	3	0
a1fd60aeb5d5935ec4f3d5ca27c4df58	0	0	0	0	0	0	0	0	3	0	0	0

6924658a70931fefc0070e18 05cdfd64	0	0	0	0	0	0	0	0	3	0	0	0
2f0a48057505317c40050ecf 904cb8c4	3	0	0	0	0	0	0	0	0	0	0	0
b062d7db384c88b0a7d4245 d35348a90	0	0	0	0	0	0	0	0	0	0	2	0
6418d31db9ef879bbb10a4b bfc2d8daa	0	0	0	0	3	0	0	0	0	0	0	0
5fb630165baf9edc221792cc dfb5ef89	0	0	0	0	0	0	0	0	0	173	0	0
1095c6d1025909ca0c3a8edd 255ac909	0	35	0	0	0	0	0	0	0	0	0	0
a43877ed2eb9256e68a6971 69a8b132e	0	0	0	0	0	0	0	0	0	0	0	27
7d6265a8aa091ea1a3ec0fa9 e1dde1a5	0	0	0	0	0	0	0	0	0	0	2	0
cd2debe4fa517caa09ecfa48b 8b5c45f	0	0	0	4	0	0	0	0	0	0	0	0
63e967c0c846f9d38a938057 751f95ae	0	0	0	0	0	0	0	0	0	159	0	0
63e1a6a7762370e9a457494 113d49b59	0	0	0	0	0	0	0	0	0	0	0	23
1d2f146a2aab8197ec83800e 9168cff2	0	0	0	2	0	0	0	0	0	0	0	0
f74358753edd7d453bf54687 c1e4d811	0	0	2	0	0	0	0	0	0	0	0	0
8be0693475d6072679c0c89 7352c396e	0	0	0	0	0	0	9	0	0	0	0	0

8234e3678ab76ad6984a4f22 9a19b154	0	0	0	0	0	0	0	0	0	9	0	0
2d3d5379db797cb3a203553 db8a241de	0	0	0	0	0	0	0	0	2	0	0	0
2408b18ad8c9d8f29a0400f2 cadeb232	0	70	0	0	0	0	0	0	0	0	0	0
e6066cf5a40fd23348f7360e7 3aa1b63	0	0	0	0	0	3	0	0	0	0	0	0
7df4f721a258d7d1ca5e4635 769af11d	0	0	0	5	0	0	0	0	0	0	0	0
e6499fe39b7733e7d0583bab ab8cba75	0	0	0	0	0	0	0	0	0	0	2	0
85a2b4f123139872f09488fe b45e4250	0	0	0	0	0	0	0	7	0	0	0	0
83fc447b9524ab07aae0837b c9658e15	0	0	0	0	0	0	0	0	0	0	0	6
273a0f3be20d471b812f3cbb ce841ad6	0	0	0	3	0	0	0	0	0	0	0	0
355baf16a54a5257db209143 e786af43	0	0	0	0	0	6	0	0	0	0	0	0
3a98e75c143056fa0c16dc7e 9d5aa160	0	8	0	0	0	0	0	0	0	0	0	0
10932bc04d2e4035a5d3963 49bb9e6b4	0	0	0	0	0	0	2	0	0	0	0	0
5b883c2c77bc58cacce27dee 1dc60028	0	0	2	0	0	0	0	0	0	0	0	0
c344e1c5ef6fc083e3c9c6c7d 571c63e	0	0	0	0	3	0	0	0	0	0	0	0

e98133e880ddbe7bcc18ada170fea7fb	0	0	7	0	0	0	0	0	0	0	0	0
92bfaaf6d15efdc5f46cf52ec757925c	0	0	0	0	0	0	32	0	0	0	0	0
9bde4ec2a871a544abfe29cd2c2f1a44	0	0	0	0	0	0	0	0	0	0	0	3
19ffdf3501a4c3a1e8faed49b863704e	0	0	0	3	0	0	0	0	0	0	0	0
385f6460e0cc9dad0713d7b26512f8ad	0	0	0	0	0	8	0	0	0	0	0	0
5eb66522fb08eefe6a5bd134b80ed5dc	0	0	0	0	6	0	0	0	0	0	0	0
40094b042ddc9f15cb9f380c6d95ff83	0	0	0	0	0	0	0	0	0	0	57	0
49eff7b6b8e553b8458c43eee006c952	0	0	0	0	0	0	0	0	0	0	0	3
fc0df5ed059cca8e781b65d4b4e2290c	0	0	0	0	0	0	0	0	0	7	0	0
8363ae34dd20b20a16af532241876284	0	0	0	0	0	0	0	0	0	3	0	0
bc14fd1336d8272cf2a47eecf1ee0280	0	0	0	0	0	0	0	0	0	0	0	26
dec5dba9e81f895e5c899f4527765f55	0	0	0	0	0	0	0	0	0	0	0	2
576cdc5a43641d7af644da737b054d2b	0	0	0	0	0	0	0	0	0	0	0	6
6e7105398a43eb1c70b68b3be6b7340b	0	0	0	0	0	4	0	0	0	0	0	0

655137aacbbe1721343574f8bef8c1f5	0	0	0	0	0	0	0	3	0	0	0	0
838f5072e4ce661a81ed5a5a6ad2d121	5	0	0	0	0	0	0	0	0	0	0	0
a118859cdd5e63c0a18b9e83fb911b41	0	0	0	0	0	0	0	52	0	0	0	0
f8136ad1c9085150cc71a919972736c9	0	0	0	7	0	0	0	0	0	0	0	0
368321abaa47848b06264de27c626a73	0	0	0	0	0	0	0	0	0	0	2	0
0179a142686cded882dce292c6064637	0	0	3	0	0	0	0	0	0	0	0	0
b13a06853a3cbf3c41741cb3c86c5e51	0	0	0	0	0	0	0	0	0	0	5	0
b89c460099f695b1652ed37659b46136	0	0	0	0	0	0	0	0	0	0	8	0
0e13a1ff894cfa8cd05180becc04999e	0	0	0	7	0	0	0	0	0	0	0	0
c0d0a5d9ba12c11b4f737b5e12743403	0	31	0	0	0	0	0	0	0	0	0	0
50666e65b44b5d17a2dd593a65120cae	0	0	0	0	0	0	0	0	0	12	0	0
734cd50ec15bcffb19f3a2e34bf48dd5	5	0	0	0	0	0	0	0	0	0	0	0
dd89cee5486c4a67a84ba1b536242f99	0	19	0	0	0	0	0	0	0	0	0	0
7ab3aeef872987dc606057edec3c0946	0	0	0	0	0	0	0	3	0	0	0	0

3672efebdb8690467bf4102b93a46683	0	0	0	0	0	0	0	0	2	0	0	0
32f94868a8495348f744cc226e5d1d20	0	0	0	0	138	0	0	0	0	0	0	0
f0ddf182dbf6a87652b4121132547e71	0	0	0	0	0	0	10	0	0	0	0	0
8499ef21e005099c018910840bd96cf6	0	0	0	0	0	0	3	0	0	0	0	0
e24c5ee46c4e622c6201f38f4a4d2189	0	0	0	0	0	0	0	0	0	0	0	6
c2c1b06955df16bbf594428d4fa9993f	0	0	0	0	0	0	0	0	0	16	0	0
a91f438ec703022739c963a71712fbf1	0	35	0	0	0	0	0	0	0	0	0	0
ac6a256070df72b94784e8f8f24d014a	0	0	0	9	0	0	0	0	0	0	0	0
c5e4dfad8351f9adb7bc9fc70a89a7eb	0	0	0	0	0	0	0	0	0	0	2	0
e3fbdcfc1e7f2567fb3fb04a04709924	0	0	0	0	0	0	0	0	0	5	0	0
d55874f434eb4b32f313f7bdcab900b1	0	0	0	0	0	0	0	0	4	0	0	0
c3b849212c5af10fcade7576116ecc50	0	0	0	0	0	38	0	0	0	0	0	0
7cfb450bc04af80e7540e0d62257030f	0	0	0	0	0	0	0	0	0	0	0	2
954bb902be784e9c1973b666b33339c9	0	0	0	0	22	0	0	0	0	0	0	0

38693ef99db60030c7ab5524ac22c000	0	0	215	0	0	0	0	0	0	0	243	0
42f566f3fd1b8ad1677a435494bd0c54	0	0	0	0	0	2	0	0	0	0	0	0
e065d05be9c7445a0ef3bb3d0830907b	0	0	0	0	0	0	10	0	0	0	0	0
508679f4261b09d7b83ad7080f223d75	0	0	0	0	0	0	46	0	0	0	0	0
5c61773d2ecc753081f8cddad7cebbdd	0	0	3	0	0	0	0	0	0	0	0	0
7987b35a9390c55ae90a969684db30a6	0	0	0	0	0	0	0	0	3	0	0	0
cdabadc298a6506b8d677438bf3b2c97	0	0	0	0	0	0	10	0	0	0	0	0
ed047ffc293f776a6b8697ed8861ae5c	0	0	12	0	0	0	0	0	0	0	0	0
d1b25e330171f8c02b39123d5846cf0f	0	0	15	0	0	0	0	0	0	0	0	0
325af7db0c6a6f3a7185b50c0610cd6e	0	0	0	0	0	6	0	0	0	0	0	0
8cc1683c2f4c65a23ab978577256c431	0	0	0	0	0	0	0	0	0	0	16	0
98c809824ff07c96a3449fd42f704e20	0	0	0	0	0	0	0	0	0	3	0	0
7d4ba85d92987f4e1b0bdd0ecbfb1cb0	0	0	0	2	0	0	0	0	0	0	0	0
51bc65761ef45698a20effc2e44d8152	0	0	0	0	0	0	0	0	0	0	8	0

bd50c01830f37e751cbbc6c9 4e48a1f9	17	0	0	0	0	0	0	0	0	0	0	0
326eb4d94f2f4c7152149494 9d9f9051	0	0	0	0	0	0	0	0	0	0	0	7
4b5af465c670f3b48ff5b253a 70e8706	0	0	4	0	0	0	0	0	0	0	0	0
8b23d7b07080d4fc6f2e1fa37 ba8ac84	0	0	0	0	0	0	0	5	0	0	0	0
1d4d8033bd33651b538612b 36338b2b8	0	0	0	0	0	0	0	4	0	0	0	0
a3e6ab3a5ecb5df3148e3ebf 382d48c2	0	0	0	0	13	0	0	0	0	0	0	0
e06cecc142f6ab5815a558aa 6e52909c	0	0	0	0	0	0	0	0	0	2	0	0
f8bb7501cef03581f72206a1f ff20964	0	0	0	0	0	0	0	0	4	0	0	0
ac058e46054835ecfa061ea5 25794313	0	0	0	0	0	0	0	0	10	0	0	0
6503aa459ecff80204e867ab b59dba69	0	0	0	2	0	0	0	0	0	0	0	0
fe484d835d8daf33a974ae67 35f43f9b	0	0	0	0	0	0	0	2	0	0	0	0
3eddfebe6aaff361bd50c35f3 4ce10f2	0	2	0	0	0	0	0	0	0	0	0	0
a0e7cd10106b573eeab0a0ea ada564ed	0	0	0	0	0	0	0	0	0	7	0	0
cd889a10be7cd8d16e5ed78 d0821bc23	0	0	0	0	0	0	0	3	0	0	0	0

594d328f99a3351e0dcd8947274a9022	0	0	0	23	0	0	0	0	0	0	0	0
5b2de6805577f049759587cfb17ee33a	0	0	0	0	0	0	0	0	0	0	4	0
77e7bc820a75f9a08ca4d857e4b0691b	0	0	0	0	0	0	0	4	0	0	0	0
8f96e0ec9bbb38a1b9e2f9f8bcf34dd0	0	0	24	0	0	0	0	0	0	0	0	0
c90c68c0e2773d8ec8a4d31d4e11b525	0	0	6	0	0	0	0	0	0	0	0	0
d73da18a5dbed6db7a8cee2151ce6bf4	0	0	0	0	0	2	0	0	0	0	0	0
a0ea359d14a81542d87528f29dc2efae	0	6	0	0	0	0	0	0	0	0	0	0
975ac760b916ebaa199e7cdd0983d9dd	0	0	0	0	0	0	0	0	0	0	111	0
dd56ec594fdef04a9a8068058dc44a6e	0	0	0	0	0	0	0	0	0	3	0	0
b1a97591a826dadc4584b1c5121e15dd	0	0	0	0	0	0	0	0	0	0	54	0
da5af916f149f1101d7bf52e2bf79e1d	0	0	0	0	0	0	0	0	0	0	0	15
12b538300e38f9c63c79064e255027bc	0	0	0	0	0	0	0	0	0	15	0	0
58c4d4a81400a12d054a8ab2ed855bf0	0	0	2	0	0	0	0	0	0	0	0	0
05a8e1e60a5250817f69b764155c7d4e	0	0	0	0	0	93	0	0	0	0	0	0

3af87e83191cdd86cd8b9a9dc8ca2c52	0	0	0	0	0	0	0	146	0	0	0	0
fd34afd9c0db7717bb0aa8242dace14b	0	0	0	0	14	0	0	0	0	0	0	0
9e59ecdb4023769c572de49dfa88869c	0	0	0	0	0	4	0	0	0	0	0	0
66200cd5e526e269b70a0bfa84799f14	0	0	0	0	0	0	0	0	0	0	0	2
23b56a8006a750fb9fb97734af98e7b9	0	0	0	0	0	0	0	0	0	0	2	0
7b8a8f8c614817bfe66659af6c9ca1de	0	0	0	0	0	0	0	0	0	0	3	0
7c70835553d7bb7aee01f79e1a7c3c4e	0	2	0	0	0	0	0	0	0	0	0	0
560e77b1a7f760e29ce3791fb7e6fa92	0	2	0	0	0	0	0	0	0	0	0	0
c8079b82e750458c8f0d860ceafbb640	0	0	0	0	0	0	0	0	0	3	0	0
2f7f086740878f0fc959ea9cbe13d35e	0	0	2	0	0	0	0	0	0	0	0	0
93677581c1d07eeb07a82623facbe852	0	0	0	0	0	0	0	0	0	0	4	0
6a23ef21de6f8b90ddc5e36c55eeb1aa	0	0	0	0	0	0	0	0	0	0	4	0
c01645b6c949a4537358482e6b632b1f	0	0	0	0	0	0	0	0	0	26	0	0
1fcbed0670952cee9deab77e7ba31e9e	0	6	0	0	0	0	0	0	0	0	0	0

e8071684e2dffe526568a84ade4c90ca	0	4	0	0	0	0	0	0	0	0	0	0
9fd6bcbb8dbd4690b30ed5a2a7327d1b	0	0	0	0	0	3	0	0	0	0	0	0
782886f7926c635f4a30e65781360c10	0	0	0	0	0	0	0	0	0	0	60	0
10aa00c21ca2f0924159d8e4e554691d	0	0	0	0	0	0	0	0	0	0	57	0
886e97e58391e2841e447ff8f79f0a36	0	0	0	0	0	0	0	0	0	0	0	185
6aa3d819b6bf1730c27079fef9491f08	0	4	0	0	0	0	0	0	0	0	0	0
bcc15769e4225caf4fd5118a03e70429	0	0	0	0	0	2	0	0	0	0	0	0
d3d5ebe64a710984fb6ae13f23e8fcb7	0	0	0	0	0	0	0	0	0	0	17	0
598285d0930beab40d500c1d45560e39	0	0	3	0	0	0	0	0	0	0	0	0
85d124f62035207e218edee429f4c22b	0	0	0	0	0	0	0	0	0	0	37	0
e4ec14f3971b62843db48cda84e38875	0	0	0	0	0	0	0	0	0	0	3	0
7be28c8d36ace659bd4ad596dcc26ad5	9	0	0	0	0	0	0	0	0	0	0	0
efda7309b3e12db0f508754514e3896d	0	0	0	0	0	0	0	0	0	0	0	3
c1a792584e5f827c95258738949c96f6	0	0	0	0	0	0	0	3	0	0	0	0

da19c26be21ae488b1da9c322b69af60	0	0	0	0	0	0	0	2	0	0	0	0
867ad0531dffec16130db88419325cb9	0	0	0	0	0	75	0	37	0	0	0	0
7857f35ba7dd309e368786ade81442c9	0	0	44	0	0	0	0	0	0	0	0	0
c4d6b4dca7c75431aba7a6d66ba34205	0	0	62	0	0	0	0	0	0	0	0	0
562e3a60e6c94767e354093881d39e73	0	0	0	0	0	0	0	0	38	0	0	0
c28a523d065ddb885b8f347cd4fbb4ce	0	0	0	0	0	0	0	0	49	0	0	0
0448d35baa4bcf6bd40357df7e3eefc7	0	0	0	0	6	0	0	0	0	0	0	0
4ff5873216bc6e4cfe9c42bcc8dec69f	0	0	20	0	0	0	0	0	0	0	0	0
f4247d56b142bcebb54f8c3effb44297	0	0	0	0	0	0	0	0	0	11	0	0
aa9b48986f98450a67f88a07e475b7e0	0	0	0	0	0	0	0	0	0	7	0	0
49e4c762702df27fa5dee90044ad1c4b	0	0	0	0	0	2	0	0	0	0	0	0
d955bd5335be4d5d493b3442cf86c51c	0	0	0	0	0	0	0	0	0	0	0	4
a806b75f04ac38031974a9a83aa94ef0	0	0	0	0	0	0	0	0	0	0	2	0
77aeeb5bf551b0fe6a581a51bdcdb19b	0	0	0	0	0	0	8	0	0	0	0	0

a6eae171e6f61ce8a9e22be72cd59248	0	0	0	0	0	0	0	0	0	0	2	0
07f397e9419edda577adec95fe4dc2b5	0	0	0	0	0	0	0	5	0	0	0	0
28f5ba2ed4c5016ce59f7244dde0a12a	0	0	0	0	0	0	0	0	0	0	2	0
fecab6a043992bdb1f245fc6e42acd63	0	0	0	0	0	0	0	0	0	57	0	171
ca4505dbf12349bd2744b2b5afa2f37d	0	0	0	0	3	0	0	0	0	0	0	0
f6ec06668b30ffb44533e4fe085356aa	0	0	2	0	0	0	0	0	0	0	0	0
250c7f8e245bb0cde9b6c6525ef421a6	0	0	0	0	0	0	0	2	0	0	0	0
7def0c6d3dcb5ecf9cb7e7ef5abea563	0	0	3	0	0	0	0	0	0	0	0	0
3905c0e359fce0d8ad439336eb185089	0	0	0	0	0	0	0	0	0	0	15	0
a01b53996cf143c9ae03843afd97b16b	0	0	0	0	0	0	2	0	0	0	0	0
384269009b981e29b6c745e7b807a013	0	0	0	0	0	0	0	0	0	2	0	0
09bda399e7da385b89c6499d92487efd	0	0	0	0	0	0	0	0	28	0	0	0
966917376ad9112eaa1296aea4d12008	0	0	0	0	0	0	0	0	0	0	0	3
fd1c285288f4ad39aa0c0b5850b82021	0	0	0	0	0	0	18	0	0	0	0	0

36429ea225b7a83e5690bda6b49e9898	0	0	0	0	0	0	0	0	0	0	8	0
8622023f19ec7f0f368975379a16ddfa	0	0	0	0	0	0	0	0	0	0	6	0
7c33453fd81590b77479065f3a75df74	0	0	0	0	0	0	0	2	0	0	0	0
5042e1e8e00862c3fb3532295670ede1	0	0	0	0	0	0	0	0	4	0	0	0
9ef07004071e002c455cc53765328523	0	0	0	0	0	0	0	0	2	0	0	0
cd1a411309d4e653379cb2e38c31089f	0	4	0	0	0	0	0	0	0	0	0	0
d0f2a4a2b058842c8938ea3ca1371208	0	0	0	0	0	0	0	0	0	2	0	0
cff807979184cd929704ef24bf1b8b75	0	0	0	0	0	0	0	0	0	36	0	0
70659818492a6eee64fedf5b89c4716a	0	0	0	0	0	0	0	0	0	0	8	0
a431afb7bfc1ac51a95da15e179f0b97	0	0	0	0	0	0	0	0	0	0	0	2
44659edf94979978f12ac03abb6d6fb9	0	0	0	0	0	0	0	0	0	0	2	0
78e50f632bf5a79ccee5f22e2c5dfc65	0	0	0	0	0	0	0	0	0	14	0	0
738a51bf2541ae50a0c071d157029f07	0	0	0	0	0	0	0	0	2	0	0	0
46dde17e57e924bf53ef6a4942d94842	0	0	0	0	0	0	0	0	0	0	3	0

810ce43274e1f6b28a7dcaa73f1eec5e	0	0	0	0	0	0	0	0	0	0	0	5
b58eba8d7cd00bedcfd2467bac87b30d	0	0	0	0	0	0	0	0	0	0	3	0
e4520c9b1add6d9e8771a4e632704cb5	0	0	0	0	0	0	0	66	105	0	0	0
d0dde1a8a69d6801ee72be584fc9b70d	0	0	0	0	0	0	0	0	0	0	0	5
d75e15046c0666acbd90b8c252f1e8fa	0	0	0	0	0	0	0	0	0	0	0	2
fb0ff609aaef6466ca8a60aedd638694	0	0	0	0	0	0	0	0	0	0	16	0
0aada940be48937fd8419e84c376cc00	0	0	0	0	0	0	17	0	0	0	0	0
f2dc804281f0b4660dfc23a2acbcda7d	0	0	0	0	0	0	0	0	0	0	0	12
85fda693fce76b06d732ded69336c9dc	0	0	0	0	12	0	0	0	0	0	0	0
d5d7fa203d1d0449831d7656bbd185bb	0	9	0	0	0	0	0	0	0	0	0	0
bbf19de2224e68a410bec60a2cf2d587	0	0	0	0	0	0	6	0	0	0	0	0
49bc0802457499690bed5b28adaf5bd0	0	0	0	0	0	0	4	0	0	0	0	0
dcf1bd0f3107a4a84efb348e13d30ae2	0	0	0	0	0	0	0	0	0	0	0	4
a9808c83f097e2c464e8b7b449d6f6f1	0	0	0	0	0	0	22	0	0	0	0	0

d62a72917f35873f82b75f2aaf30a7cc	0	0	34	0	0	0	0	0	0	0	0	0
12f636ec1671ec576b3d7ddfac31f8ef	0	0	2	0	0	0	0	0	0	0	0	0
5419612d1a98dc48cb8c3bd5e0e70557	9	0	0	0	0	0	0	0	0	0	0	0
0ea7bcce4a35f955edb9ec60c6ea7cdb	0	0	0	0	0	12	0	0	0	0	0	0
92449b657cab08629fd8f2b7cdcf91d8	18	0	0	0	0	0	0	0	0	0	0	0
2d3c3b4f40a4dfb375f04fe0bf1d0423	0	0	0	58	0	0	0	0	0	0	0	0
177f4b207d319dca167bfde9201706ab	0	0	5	0	0	0	0	0	0	0	0	0
fdd0cc6422bff60fc52c4747dde8ac7c	0	0	0	0	0	0	0	0	0	0	3	0
e4bf51896fe058886e5b7edc9e43e2b7	0	0	0	0	0	0	0	0	2	0	0	0
ab37d560a107bb6d7e945c04f7c9b706	0	0	0	0	0	0	0	0	0	0	30	0
e12285c64becd05d98c23edc2d4304c9	0	0	0	0	0	0	0	0	0	0	3	0
e9d768848278f822ac1d62d4c7994f3e	0	0	0	0	7	0	0	0	0	0	0	0
112bc83c7c434f38a0085393dbb5f76b	0	0	0	0	0	0	0	0	0	0	0	24
59a39299339f9de21d95d048570f161e	0	0	0	0	0	0	0	0	0	0	4	0

c16cfff4b6188eac0cc808401f5b342e	0	0	0	0	0	0	0	0	0	10	0	0
98e2d0fc63b6a46ba21356fc4428919b	0	0	0	0	0	0	0	0	20	0	0	0
00d188e9742bd33748a35c89c4a1dbd8	0	0	0	0	0	0	0	0	6	0	0	0
0e073e1d2452bccaed418ff6f8415dc1	0	0	0	0	0	0	0	0	0	16	0	0
cec5e8f61c59918db55b1c117077f8a6	0	0	0	0	0	0	0	0	0	2	0	0
a14e3b59b3bee14b592fb357bc118c2a	0	0	0	0	0	0	0	0	0	0	2	0
742e9a2a9414a9a2b3b53f1a3ad18e37	0	0	0	0	0	0	0	78	0	0	0	0
5b45103848c9d03a2c9eb8a771eb808b	0	0	0	0	0	0	26	0	0	0	0	0
66dd452c75fdc776beac5f782bc90c9c	0	0	0	0	0	0	0	0	2	0	0	0
cd8c6282e1a749729f2bc203a78b3799	0	0	0	0	0	0	0	0	0	0	5	0
7d09ad0b4345951addfd545f7837237e	0	0	0	0	0	0	0	0	11	0	0	0
151e547c376859805ecdd4730e2f4405	0	0	0	0	0	0	0	0	0	0	14	0
ff4a4d128be98e1f49ee49259736625e	0	0	0	0	0	0	3	0	0	0	0	0
54d13f78e1df1ce21713840589805a39	0	0	0	0	0	18	0	0	0	0	0	0

32e656cbd0ed5f71425482c8c3ca6c22	3	0	0	0	0	0	0	0	0	0	0	0
798381ec49c4c1bd940065c18076ea28	0	0	0	0	0	52	0	0	0	40	0	0
b08b05c92f144a34ce8cf810f7643a2f	0	0	0	0	12	0	0	0	0	0	0	0
24a6f3d0e7f98750936f59e67708e34a	0	0	0	0	2	0	0	0	0	0	0	0
18850b69f9a04b0feffa48de4bffa321	0	0	0	0	0	0	0	0	0	0	15	0
f4fbf5462a247cab0764829600a22e54	0	0	0	3	0	0	0	0	0	0	0	0
db264e6613a347e5a93b00d85c318bb5	0	0	2	0	0	0	0	0	0	0	0	0
05405a16ac6b1844b28be2accbeb002f	0	0	0	0	0	0	0	94	0	0	0	0
7ab36f4ddc835a84ab082f2e2cc54272	0	0	113	0	0	0	0	0	0	0	0	0
fb75295c7866fd99f190ea2833ccaa31	0	0	0	0	0	5	0	0	0	0	0	0
3c97243647c7b0e9c7d2ca5bb2e92a38	0	0	0	0	0	0	0	0	0	0	23	0
ec7c607a9b9769eed3d3c1796dad5fda	0	0	0	0	0	0	0	0	0	0	4	0
7e2924008a084d35f29a11a2a610cdad	0	0	0	0	0	0	0	0	0	0	2	0
177993c1094e30281cf6865bc3264a12	0	0	0	0	0	0	0	0	0	0	30	0

4438caec09d223c302ec93a98325f040	9	0	0	0	0	0	0	0	0	0	0	0
3f8d53605054df7e56ae6b8aa5313466	0	0	0	0	0	0	0	0	0	0	3	0
8a7a41b2359444cb6dfad310c4a58f86	0	0	0	0	0	0	0	0	0	0	3	0
d0c9f67cc951689e7c8f9e9fce311d5a	0	0	0	0	0	0	0	0	0	0	2	0
10492a457ef55d35685f596b0194e5af	0	0	0	0	0	0	0	39	0	0	0	0
da56dcb27e5ac3032540351c543a61b0	0	0	0	0	0	0	0	0	0	14	0	0
21a9b8f416a7890b592f98c406879bd3	15	0	0	0	0	0	0	0	0	0	0	0
23d229ee93098b69160ce3a430141647	0	0	0	0	0	0	0	0	0	0	0	4
a2b9c796b6bf31c71c148d5d8f1896b1	0	0	0	0	12	0	0	0	0	0	0	0
249b3a0f20b6bf9dbe1f2e8e9da29a52	0	0	0	0	0	0	0	0	0	0	0	6
54d2de09653fad90773ea67db987f5b5	0	0	0	0	0	0	0	4	0	0	0	0
2f35c2a6856553db456509ef8f511b82	0	0	0	0	0	0	0	0	0	16	0	0
69e6801043eaf8f607a6d36fe9c5ec54	3	0	0	0	0	0	0	0	0	0	0	0
0da7716c61148f44eca0943c9d437edc	0	0	0	0	0	0	0	0	3	0	0	0

2e37d9b53dd6100f94c09e73d3b4b67a	0	0	2	0	0	0	0	0	0	0	0	0
a6d8e4edb9f4bc3e61c1bb553a395543	0	0	0	0	8	0	0	0	0	0	0	0
45add4cdf14850a6c626fb5d0f15d809	0	0	0	0	0	0	0	0	0	0	0	2
b275762091328ae742437858d356f798	0	0	0	0	0	0	3	0	0	0	0	0
a123ae333925ffa25867bef287dfa655	0	0	0	0	0	0	13	0	0	0	0	0
602a83e94a0475de3e58757a8e82fbb0	0	0	0	0	0	0	0	0	0	67	0	0
41421157e03dd5a0520569bd616b9900	0	0	0	0	0	0	0	0	0	0	2	0
4700684f071320bec65cfce8e5ee902d	0	0	0	0	42	0	17	0	0	0	0	0
fa0a7c1dcacb0cf339169985cf376f7d	0	0	0	0	0	0	0	0	0	0	5	0
bd2918d9e1abd83a6b343878fffceeb1	0	0	0	0	0	0	0	24	0	0	0	0
e5a995dc14af1e8fb4e7222042f669db	0	0	0	0	2	0	0	0	0	0	0	0
b3520e5718da5c1838faa6e776c59608	0	0	0	0	3	0	0	0	0	0	0	0
44b9c43ba553f0dcdff8c88e469400b7	0	0	0	0	0	0	0	0	0	0	4	0
e46e0a52ad86092998dbae22455425b7	0	0	0	0	0	0	3	0	0	0	0	0

f7d022243e947c141b2494d2c89db0f1	0	0	0	0	0	4	0	0	0	0	0	0
37bf49e71724c2dddd96175a704c30ec	0	0	0	0	0	0	0	0	0	0	20	0
fdb24fdcbc967169ff8ce2c9011ebbb9	0	0	0	0	0	0	0	0	0	4	0	0
8597391787018dcc303f7396f64ca449	0	0	0	0	0	0	0	0	0	3	0	0
dcf13928dc0f6a3b4d6c521daa02ca5d	0	0	0	0	0	0	0	3	0	0	0	0
fe5b712d443e37502f8e158b8d19af24	0	4	0	0	0	0	0	0	0	0	0	0
0c0d043a2a2a31e82f62a5f0b0779551	0	0	0	0	0	0	0	0	0	11	0	0
dd9601576e6cfdf900db5b64a35139f5	0	0	0	0	0	0	0	0	3	0	0	0
a459e9a958154971227017428243ee40	0	0	0	0	0	0	5	0	0	0	0	0
809d8fbf94dc18d08facd6733c6f727a	0	0	0	0	0	2	0	0	0	0	0	0
02deefd5e61d79837b5d53caab5d5630	0	0	0	0	0	0	0	0	0	0	0	4
1562a5c431e0de0563aef939267d93be	0	0	0	2	0	0	0	0	0	0	0	0
da0bbc466f42bed76071f54ba92de8ab	0	0	0	0	0	0	0	0	0	0	4	0
bb6bfcd734cb0b268d3b947e67c91593	0	0	0	0	7	0	0	0	0	0	0	0

2d196e09c5876979e1b04a97a4497dab	0	0	0	4	0	0	0	0	0	0	0	0
b1af55c0a658426d15ac03f5ebb64a07	0	0	0	0	0	3	0	0	0	0	0	0
4483dd4cef0e55bf78d3aa23298eda8d	0	0	0	0	0	0	0	0	0	0	3	0
463063137bd49a6b49470281e2ca8464	0	0	0	0	0	2	0	0	0	0	0	0
b43e79f01540bd3930fec0a4927cec81	0	0	0	0	6	0	0	0	0	0	0	0
1393831fcfa94169809dd766544d4da6	3	0	0	0	0	0	0	0	0	0	0	0
b2ae7ebdf0515edd7b868152c8df75f3	0	0	59	0	0	0	0	0	0	0	0	0
7c7ce31856628f77a504e287cd144dea	0	0	0	0	13	0	0	0	0	0	0	0
2f0da0168d58f0b5b6e3fa81506367f1	0	0	0	0	0	0	0	0	0	0	0	5
15d06151a3ec0d110831987868686ce2	0	2	0	0	0	0	0	0	0	0	0	0
ed218b050945a4d2c810067be621cf7e	0	0	39	0	0	0	0	0	0	0	0	0
e97d08625124e66a475c88780a1baac8	0	0	0	0	0	0	0	0	0	3	0	0
36a79009af99e18db56f15447b384b95	0	0	0	3	0	0	0	0	0	0	0	0
98f4a5052ea285a65164fd94644a10e1	0	0	0	0	0	0	0	27	0	0	0	0

3999a40b6e8197f4929bcb99a97590e5	254	0	0	0	0	291	0	0	0	0	0	0
c614100262d221b169ce48308b4a85ec	0	0	0	0	0	6	0	0	0	0	0	0
c3ea897e9c1b5565707427e6feec8e9a	0	0	0	0	0	0	0	0	7	0	0	0
01ea6b89da7d75ca69f8f41c7d4d2d41	0	0	0	0	0	0	0	0	0	13	0	0
fecac97ea9f4d95400148d281f013e4b	0	0	0	0	3	0	0	0	0	0	0	0
28d506656e60b0adacd3150b7de768fc	0	0	0	10	0	0	0	0	0	0	0	0
770ee89ab1e0982241c2eea80d4ca019	0	0	0	0	0	0	0	0	0	0	35	0
8775b176397ef02881c768e1343bfebc	0	0	0	0	0	0	0	0	3	0	0	0
b48e3003054bd77db46e93addfb3706a	0	0	0	0	0	0	0	0	0	0	13	0
3ebfb0a7909d4f0ce69edc7768f7aaed	0	0	0	0	0	0	0	0	6	0	0	0
0e0ed7b7b41a9da6f25f3973d131b6c2	0	0	0	0	0	0	0	0	0	0	2	0
f34b92c13da8e9a7f184b79a8bcc1ef1	0	0	0	0	0	0	0	0	14	0	0	0
f4c997c5d182f158b0517d1ee30883a8	11	0	0	0	0	0	0	0	0	0	0	0
4473cfb517136b49403f6cbdc634c3d7	0	0	2	0	0	0	0	0	0	0	0	0

fde1f73eabd0e7022e45c227f069a955	0	0	0	0	0	0	0	0	0	0	0	2
78cc6272b8f75a333e90de4eaa47a0b5	0	0	4	0	0	0	0	0	0	0	0	0
16729e08f05b7e28dd7cce38a808d5ac	9	0	0	0	0	0	0	0	0	0	0	0
cd225adbbc8a085e431e85236880e335	0	0	0	0	0	0	0	0	0	0	13	0
46ff4943d3a9dbfecde6c4a824456d31	0	0	0	0	0	0	0	0	0	0	0	4
efac0ad67ae143b5bcf21acd626778a5	0	0	0	0	3	0	0	0	0	0	0	0
b8cffff7597c81165536a84121e83ab7	0	0	0	0	0	0	5	0	0	0	0	0
7902659e68d219cdbb6ac9227797ff61	0	0	0	0	0	0	0	0	0	0	0	7
16bbde6b51022c38b0f416f8ec3086f0	0	0	0	0	0	0	0	0	3	0	0	0
590fa12470f30621d5f609c9e0840931	0	0	0	0	0	0	0	0	0	0	0	24
82b28f6a2394b5a686d8f7b46c815be4	0	0	0	0	0	0	0	0	0	2	0	0
be682ec93e0b15364fa0d46ed6653a34	0	0	0	0	0	0	0	0	0	0	0	2
04f5ed14a1b7794fb86d2ec079ba67fc	0	0	0	0	0	0	0	0	2	0	0	0
39785a42b946f99ffdb3f7f129b87662	0	0	0	0	0	0	42	0	0	0	0	0

3a7cdc190ffcd191bb809a909bb2d0a5	0	14	0	0	0	0	0	0	0	0	0	0
18ef4ad11f694265b32c7ebb66eefd0c	0	42	0	0	0	0	0	0	0	0	0	0
9d71c14e97ec261a7b251dbcfcf8dd1a	0	0	0	0	0	0	0	0	0	60	217	0
936ec4ddbc5f6d7058f8a25860c3f3c4	0	0	0	0	9	0	0	0	0	0	0	0
68067d204ab97b65e385e78a65998d96	0	0	0	0	0	0	0	2	0	0	0	0
60934b6f6739f29d6638d695a63f0877	0	0	0	0	0	0	0	2	0	0	0	0
96e079a2019ddebfd645c403cd9b9493	0	0	0	0	0	0	0	0	2	0	0	0
58af5d1ca3f06822596a61edabf23b0f	2	0	0	0	0	0	0	0	0	0	0	0
65035d45b049a61f292d3e25ca9c3428	0	46	0	0	0	0	0	0	0	0	0	0
82fead975513b914d8a9db20df55a8a2	0	0	0	0	0	0	0	0	3	0	0	0
d53a433f1e2b9954e7e0ec56206932e0	0	0	12	0	0	0	0	0	0	0	0	0
95eddcea980b7a1d21f4cffc9378d05e	0	0	0	0	0	0	4	0	0	0	0	0
202fd9c6dcba9a6003fd2ffee8d6d75e	0	3	0	0	0	0	0	0	0	0	0	0
b11f110da363a3e10840804a2c969073	0	0	0	0	0	0	25	0	0	0	0	0

ead534cb482cc736911ae8e381d3b48c	0	0	0	0	0	12	0	0	0	0	0	0
ba9ecab0fd3267239d2394eefb09afd7	0	0	0	0	0	0	0	0	0	0	0	3
9ce67688f52c27c815ab08caeaa93341	0	0	0	0	0	0	0	0	4	0	0	0
2be878233d6fef96a5e8b8db960129a5	0	0	0	0	0	0	0	27	32	0	0	0
4b4edd05813eb88d31b52ad9cf3305bc	0	0	0	0	0	0	0	4	0	0	0	0
48d50e1c7f280ebb37e0d0581b4f4ac5	0	0	0	0	0	0	0	5	0	0	0	0
400fd7d20d0f8a5f0637fded40cb4dd8	0	0	0	0	0	0	0	2	0	0	0	0
798e4254c33c80dcdf7ea86442265329	0	0	0	0	0	2	0	0	0	0	0	0
d5c84d008499c287d3d21cdc50d31449	0	0	0	0	0	0	0	0	0	0	2	0
01b9745b9bb6ca3f13817b60e5708ff5	0	0	0	0	0	0	0	0	0	3	0	0
6f97900eda246267474c73fa25789d06	0	0	0	0	0	0	0	0	0	0	4	0
8376f8b5606b275b5958a4d597eee641	0	0	0	0	0	17	0	0	0	0	0	0
b6c73accaf7446f7e58b665efcd8e231	0	0	0	0	0	0	0	0	0	0	2	0
0756f707c23e59996e1865a46952483c	0	0	0	0	0	0	0	0	0	0	0	5

ebcd143f09f7b124228e4548 1031787a	0	0	0	0	0	0	0	0	0	0	0	17
ab0fdbaadfc979914709dbf98 9c0a800	0	0	0	0	0	0	0	2	0	0	0	0
5c6d6018ba4f29cc546d3759 f8c1eeba	0	0	0	0	0	0	0	0	0	0	0	8
cb0282e8c1318df346e460c1 c26c3540	0	0	0	0	0	0	0	0	0	0	0	3
9f2ea279bcdcd8ac4cb554ad bfdd6d5b	0	0	0	0	0	0	0	2	0	0	0	0
be1241ffa0c3de80bb2ed3fb6 ca41302	0	0	0	0	0	0	0	0	10	0	0	0
ddb85f437bda7357d76f4f77 24120311	0	0	0	2	0	0	0	0	0	0	0	0
ec73af9a7ffd2662a8675cee7 e904a99	0	0	0	0	4	0	0	0	0	0	0	0
c47bc780f435416b160d3e12 f6e0cf5d	0	0	0	0	0	0	5	0	0	0	0	0
00efc983afea7210dd9e6184 eaf604f3	0	0	0	0	0	0	0	0	4	0	0	0
02b2553ca269818360fa62dd 82bfb707	0	0	0	9	0	0	0	0	0	0	0	0
7b672c08302f8a7c7aae0596 bc399d9c	0	0	0	16	0	0	0	0	0	0	0	0
1f4a86cdd15d3e22cf896694 bd68ab46	0	3	0	0	0	0	0	0	0	0	0	0
ee7e5cc3fe00f8b1ab31a597 42ff5c73	0	0	0	0	0	0	0	0	0	0	3	0

9dd68e0bcd68e3dc368d975cad149f9d	0	0	0	0	0	0	2	0	0	0	0	0
19634dae29f52424377a6575b059dd61	3	0	0	0	0	0	0	0	0	0	0	0
6c0023592688bf3f49bed65bffadfb6d	0	0	0	0	0	0	0	0	0	0	20	0
b5e5a2509e7e597626d5e98e857e0031	0	0	0	0	0	0	2	0	0	0	0	0
e5d662dc1733bc70e795e8ea3570e55d	0	0	0	0	0	0	0	49	0	0	0	0
0a4a48cb5a2ec930799d6977b14f4eba	3	0	0	0	0	0	0	0	0	0	0	0
d6b1414ac00130e0ceffbe6371fb6370	0	0	0	0	5	0	0	0	0	0	0	0
cbea17b90bc023da42bc1028ef4e53cb	0	0	0	0	0	0	0	0	2	0	0	0
a01a6479a2e1f896951c1516f0cb47a0	0	0	0	0	0	0	0	0	0	3	0	0
993b265dff2ba5adee92174932d8b90b	0	0	0	0	0	0	0	0	0	0	0	20
f3715dbaca2902a6c9d55188cca69a18	0	0	0	0	0	0	0	106	0	0	0	0
9275154d79168543767c081bb4cb30db	0	0	4	0	0	0	0	0	0	0	0	0
8fbfb5eacf3de1c6db3c5953fd50e521	0	0	2	0	0	0	0	0	0	0	0	0
b305a70aeabe4b089ace41c77dfded9c	0	0	0	0	0	0	0	0	0	0	10	0

51e66dc9c3f32b6846323287943346d8	0	0	0	0	0	0	0	0	0	72	0	0
3bc3189c0fdc0f87d3563b98532d6e36	0	0	0	0	0	2	0	0	0	0	0	0
0a50572811cfa151211381ec2b950a5e	0	0	0	0	0	0	0	0	0	0	0	3
ed97aed30b42e57fdc789e41ebddb511	0	0	0	0	0	28	0	0	0	0	0	0
aaf5be5992d65d93c8a467db0015abab	0	0	0	3	0	0	0	0	0	0	0	0
a02a9a9a898d7ab34db7eae404235d42	0	0	0	0	61	0	0	0	0	70	0	0
1131e1194ffbe54a203020c5c650e5de	0	0	0	0	0	0	0	5	0	0	0	0
e8c34fa190688ee8d3eac8cf3950cf70	0	2	0	0	0	0	0	0	0	0	0	0
afa2ff59db663b21a8161d502f53b036	0	0	0	0	0	0	0	0	2	0	0	0
781571b45f4bf54f5a97e603eed3f9a7	0	0	0	0	0	0	15	0	0	0	0	0
3a42fe044463270450f2e5351361105a	0	0	0	2	0	0	0	0	0	0	0	0
c41703d6fc698f8f6bbd2b34d3d8d3fd	0	0	0	0	0	0	0	0	4	0	0	0
4296c1d6076bb46e1bf04f91e7a1b34e	0	0	50	0	0	0	0	0	0	0	0	0
fb45223c151639b999175fa795877e01	0	0	0	0	0	0	0	0	0	0	0	95

1a0e54c04635c9b21d71f987ac4ddf2f	0	0	0	0	0	0	0	0	0	0	0	2
7ed6eee8c8b9a768416a58b2136269f9	0	0	0	0	3	0	0	0	0	0	0	0
6ee68db42a50e64ac13d1aa03c647565	0	0	0	29	0	0	0	0	0	0	0	0
488e35add5cb3bf332130591d8a16b9f	0	0	0	0	0	0	0	0	0	0	0	4
e41f79b2d31a528912d98994f7450c56	0	0	0	3	0	0	0	0	0	0	0	0
71c43152a0965e4a40ec7f880018b6e8	0	0	0	0	0	0	0	0	0	0	50	0
1ceb4341b1d5f34593f8d41f79fbec00	0	0	0	0	0	0	0	0	0	0	99	67
456c2c3c097cbf7acbc22ddf5c45d9b5	0	0	0	0	0	3	0	0	0	0	0	0
cc8fc3456df841f5392abc87cc52d428	0	0	0	0	0	0	0	3	0	0	0	0
2357dd4bf3ddefc01a80571fb7b3b757	0	12	0	0	0	0	0	0	0	0	0	0
31b90558771379783bc57b233b3c61a7	2	0	0	0	0	0	0	0	0	0	0	0
363e910aff299c85ac96aa51ee362f0b	0	0	0	0	31	25	0	0	0	0	129	0
380371592814484d7c83d624b2acb261	0	0	0	0	0	0	2	0	0	0	0	0
3ee5cb542f6085ff42073f3bb87a7f4c	0	0	3	0	0	0	0	0	0	0	0	0

4b1a1f6919c68ccb2158ede67338b4a5	0	0	0	0	0	0	6	0	0	0	0	0
b57a2d647f66513c4ec56db3afe47a64	0	0	0	0	9	0	0	0	0	0	0	0
efaec9f52d906d2664e4491c83783353	0	0	0	0	0	0	0	0	0	0	6	0
bc078b6a87f603c102f512580b9dd716	0	0	0	0	0	0	0	7	0	0	0	0
8e387d6c67644daaa7dfe222927664f4	4	0	0	0	0	0	0	0	0	0	0	0
7206f194dc61def12e45d5e29b2587cb	0	0	0	0	0	0	8	0	0	0	0	0
8bc75ff9afeef72d2d23f7cfa0df7159	0	0	0	9	0	0	0	0	0	0	0	0
25d6acacb0330f48d6c7d48e3e02b573	0	0	0	0	0	0	0	7	0	0	0	0
cd4ee6974d0608e2bd747d3b32586bd1	0	0	0	0	0	2	0	0	0	0	0	0
563d3472ed4ed97af7865a02e6ce3dbf	0	0	0	0	0	8	0	0	0	0	0	0
8833fcf7658f8f797f3362bc5c3a0bbf	0	0	0	0	0	0	0	0	0	0	4	0
3244e769122d255d19f8bf0b5af4a0bb	9	0	0	0	0	0	0	0	0	0	0	0
0cee3fab48dee00c603a53565917e85a	0	0	0	0	0	0	0	0	0	0	0	2
f2b11264b302809a250052b59017e109	0	0	0	0	0	0	0	0	59	0	0	0

6f25415a65ea2b67eb86bffbf94fcfe0	0	0	0	0	0	0	0	0	0	52	0	0
42668599d9287fa96f0b18adf71671c4	0	0	0	0	0	12	0	0	0	0	0	0
a9de37a175ae38dd1a7d570fe01789cc	0	0	0	0	0	0	0	0	2	0	0	0
bb282af887da0301ea8e518ce00fa7d8	0	0	0	0	0	0	0	0	0	14	0	0
ccd858aeffea002df4b9d65ff37f7af5	0	0	0	0	0	0	0	0	0	0	17	0
c70db2dcbb5fcb6b52478bd31ae2ee68	0	0	0	4	0	0	0	0	0	0	0	0
ba1b9c8ae5f1008e5f7d1646f7381c9e	0	0	0	0	0	0	0	0	0	0	0	7
52c50d82659a7068015d2249915f5b9c	0	0	0	0	0	0	4	0	0	0	0	0
c3e1d0fc189056a6019c2976831305f6	0	0	21	0	0	0	0	0	0	0	0	0
30995c9edce8bfce549f2971ab78b29e	0	75	0	0	0	0	0	0	0	0	0	0
56565fc21a4e3558049c96d3ec490c5b	0	0	0	0	0	0	0	0	0	0	6	0
4bd84fbe626c138b6919b6635c059a58	0	0	0	0	0	0	0	0	0	0	15	0
b5fbb75b165cce88ce27e80fe30c136a	0	0	0	0	0	0	0	0	0	0	4	0
e6002b86b42ef257b10d2010f86b6a25	50	0	0	0	44	0	0	0	0	0	71	0

2617c4e9daf2a25040c79e44462a1d96	0	0	2	0	0	0	0	0	0	0	0	0
e9f3c842916e34b4cdf84e7592432dae	0	0	0	22	0	0	0	0	0	0	0	0
520f256dcd47d5d67220c7c2ee9c7608	0	0	0	15	0	0	0	0	0	0	0	0
7212b39e0624523ff5e32e700fede581	0	0	0	0	0	0	0	0	0	0	35	0
903adb1e83426c787670fcea742adfa3	0	0	0	7	0	0	0	0	0	0	0	0
8c11783541fe88dcf99760abdb17abd2	0	0	0	0	0	0	0	0	0	3	0	0
ff1363f283554f03ed72547e8e78dbe0	0	0	0	25	0	0	0	0	0	0	0	0
904678b376908896a6383b6fb36819db	0	0	0	0	0	0	15	0	0	0	0	0
f4264ed99d458864f82ff73e3dfc6099	0	0	0	0	0	22	0	0	0	0	0	0
fc32868d97ad0a69208dd0748f76e08b	0	0	4	0	0	0	0	0	0	0	0	0
2610b0eed47799b627de52e7a7c42e2f	0	0	0	0	0	0	0	0	0	0	32	0
45bb19bc11f431ca2203d1e617441409	0	0	0	0	0	0	0	0	0	0	11	0
6f8c3d94b8623636abc6942bf681edd5	0	0	0	0	0	0	0	0	0	4	0	0
a2517e7a1398d7692c2911fff9509059	0	2	0	0	0	0	0	0	0	0	0	0

76f4b6ed845500af9adf3bac1f7aad55	0	0	0	0	2	0	0	0	0	0	0	0
13c13bc5a8c3aac235880e090dd44246	0	0	0	0	0	0	10	0	0	0	0	0
25c2e5301a90b8ebe2b985b032254faf	0	0	0	0	0	0	0	0	5	0	0	0
b2dbf7823017eae87980e255c04d752d	0	0	0	0	0	0	0	0	0	0	5	0
143e82aa2624854178bcefb22f898b17	0	0	18	0	0	0	0	0	0	0	0	0
a9bbfdf2f6e5dbec7c7dbfe5189aff92	0	0	0	9	0	0	0	0	0	0	0	0
379885a3cea39dc8ccd42ceef9666a30	0	0	0	2	0	0	0	0	0	0	0	0
f5a631b0909b8a53066aa99a2dd78e02	5	0	0	0	0	0	0	0	0	0	0	0
33b754766178440236ddace5894c4196	0	0	0	0	0	0	0	0	0	0	10	0
b83a8e931e7165855eeb6ca3d28e0632	0	0	0	8	0	0	0	0	0	0	0	0
aa63cad3a067f8e14d9b0a1374401f2a	0	0	0	0	0	0	0	0	0	3	0	0
d3fce912831029efbfbfcd4e51f6d782	0	0	8	0	0	0	0	0	0	0	0	0
5a744b654971e2a88249c85574207e66	0	0	0	0	6	0	0	0	0	0	0	0
0a7f187c3d746e351f285835a0a978d7	0	0	0	0	0	0	0	0	0	0	0	4

a8840d0df8780dc159d9ec4baf7b1882	0	0	0	0	0	0	0	0	0	0	4	0
30948ac00b314d5a144bb22af719e3c0	0	0	0	0	0	0	0	0	0	0	0	4
e2c0ecc4da76dda5df12e0f56e0e9439	0	0	0	0	0	0	0	0	0	0	16	0
ee199f75c2e73735e76eb75ff4461ab9	0	0	0	0	3	0	0	0	0	0	0	0
955c731992e434e9c5438ebd286ab8ae	0	0	0	0	0	0	0	0	0	0	11	0
6d10125eb564da1ddc1bd6194a044fae	0	0	0	3	0	0	0	0	0	0	0	0
64ef1b5eae961bd33478ec9bf7d5b846	0	0	0	0	0	0	0	0	0	0	0	6
88891e5a09bcd6cc7e1022476568e168	0	0	0	0	0	0	0	0	0	23	0	0
31cec08558b9a6ea2bfdf5741b7b0fe7	0	0	0	0	0	29	0	0	0	0	0	0
1ef68da961940d7db456f7cf59989aa3	0	0	0	0	0	0	0	0	0	11	0	0
1ed0c24d8952198e9b6c766fb0e42078	0	0	0	0	0	0	0	0	0	0	3	0
a122731f4dec046e4fdbaf6499928023	0	0	0	0	0	0	0	0	0	0	2	0
5211e2f8cfb580ae654a2adb3161b738	0	0	0	0	0	0	0	0	0	0	2	0
5f009c1a17ee4c9c5dc0d01ae6f38d52	0	0	117	0	0	0	0	0	0	0	151	200

263a96642124a41805fa40ff77ae6b1a	0	0	0	0	0	0	0	0	0	2	0	0
52ad0dd652b0174895e3f100e03af4e0	0	0	0	0	0	0	0	0	0	5	0	0
cd2dce50859180d9082a9b2a9231fe21	0	0	0	0	0	0	0	0	0	0	5	0
9e64a893978ed94202df66a2bc4a7c01	0	0	0	0	97	0	0	0	0	0	0	0
1896d1451403a45f216a40d07b422453	0	81	0	0	0	0	0	0	0	0	0	0
401c8e3cb52fe0984a4b5c7e4c141e34	2	0	0	0	0	0	0	0	0	0	0	0
6eda52c0c0b48c420523fd48827f722e	0	0	0	0	0	0	0	0	3	0	0	0
4ad205f1b5e5bc3fa5fef53ede7392d3	0	0	3	0	0	0	0	0	0	0	0	0
7e1c6affb73332477005e3b6be25734f	0	0	0	35	0	0	0	0	0	0	0	0
dd8683799a89f9f06e9e1edb2d0df517	0	0	0	0	0	0	0	0	0	0	0	2
8c0ae429fae2726f25689718267c8016	0	0	0	0	0	0	0	0	0	0	5	0
76071ffb5db81f019f728210424ce8bb	0	0	0	0	0	3	0	0	0	0	0	0
d24bfb88a0a6af9ec4b1842405bf3249	0	0	0	0	0	0	3	0	0	0	0	0
4c3770d85b09d299fdb7e95954dce9a5	0	7	0	0	0	0	0	0	0	0	0	0

d8d08918874791619 2725acc849a3b80	0	0	0	0	0	0	0	0	0	0	68	0
bc27b37cf7aa7e98c92fdaaa41b0288b	0	0	0	0	0	0	0	0	33	0	0	0
dcacaa6dd988cbbbeb7ce88d60e913e5	0	0	0	0	0	0	0	0	0	0	3	0
9a51959fd22c1e67a7fc43193b5b8789	0	0	0	0	0	12	0	0	0	0	0	0
8eec3680ea55562b2814cf8d319a8928	0	0	0	0	0	0	0	0	0	5	0	0
b2224cfdc102ac76de20fb7ea1ce22f2	3	0	0	0	0	0	0	0	0	0	0	0
cab47b06678fedbe7c050a418ff8ce78	0	0	0	0	0	0	0	0	3	0	0	0
d592cadf2ffec268f85d467cf1514393	0	0	0	0	0	0	0	0	0	0	4	0
71973d12279949c17b635927fb583273	0	0	0	0	0	0	0	0	0	24	0	0
fd5e070c9782f404d98893c8bd2454a9	0	0	3	0	0	0	0	0	0	0	0	0
9b3e91992f5a75e3f82eeea4e68226102	0	0	0	0	0	0	0	0	3	0	0	0
6dad142572a9cc06207c6f5434c2c2a1	0	0	0	0	0	0	3	0	0	0	0	0
46e7b8857dfcfe21147dadae9f7258e4	0	0	0	2	0	0	0	0	0	0	0	0
78e62bb0fb57ca6889dbd59b32072918	0	0	0	3	0	0	0	0	0	0	0	0

e4b2ea4a0429f43b4c1a037a 9f942c59	0	0	0	0	0	0	0	0	0	6	0	0
f25c36a3256ecb0b166ca59b 06cde8ce	0	0	0	0	0	0	0	11	0	0	0	0
491a41f150b0138bfeac24c3 79b7ca66	0	0	0	2	0	0	0	0	0	0	0	0
8e4defbbe81e39382b866e9c 31173047	0	0	0	0	0	0	0	0	0	4	0	0
7b27aa1c4163f9fe701e8e2fb a131fc3	0	0	0	0	0	15	0	0	0	0	0	0
0f253ca3f766774b7119529c 4d3d3d3e	0	0	0	0	0	0	0	0	0	0	0	181
fff30120205c8fc0fc5da6e331 49ddc2	0	0	0	3	0	0	0	0	0	0	0	0
32fb433e339b1ec6f854ea76 166830c3	0	0	0	0	0	0	0	2	0	0	0	0
c0b6995c8dec0ab1f2ed1f3a ee0c8536	0	0	0	0	3	0	0	0	0	0	0	0
2ed1d37afd36126ea62ac8ae c4161f2c	0	0	0	0	0	0	0	0	2	0	0	0
a2b4deda741675c116a688bf 6d85da10	0	0	0	0	4	0	0	0	0	0	0	0
a07af84b9b1696412757bbb 7a9de3280	0	0	0	0	0	0	0	0	0	0	0	5
a1c330500b79ad2aaee07129 23fcab17	0	0	0	0	0	0	0	0	3	0	0	0
b703234a1b49059a35755e5 0ebc9e603	0	0	0	0	0	11	0	0	0	0	0	0

d414f66372be9a17051092cf 0fedf206	0	0	0	0	0	7	0	0	0	0	0	0
24088cc439c17293148e05fe 1a912766	0	0	0	0	0	0	0	0	0	0	4	0
7444f71f4c5a408b396b5bdd 3d999f3f	0	0	0	0	3	0	0	0	0	0	0	0
e5e5303ed822be6c0d04f3e6 7c5acfea	0	0	0	0	0	0	0	0	0	0	0	5
c391245a52f6fc3ba112e596f c285325	0	0	0	0	0	0	0	0	0	0	0	26
042c2ab8e89eb07a536e4a7 82c40d207	0	0	0	0	0	0	0	0	0	0	62	0
a7af3d583a9cf3b41f4da881c 46b3f2c	0	0	0	0	0	0	0	0	2	0	0	0
7006f4f367297078a9db00db 034ea655	0	0	9	0	0	0	0	0	0	0	0	0
de3409b4a3c9478eb1a58a6f ddc24d8c	0	0	0	0	0	0	0	0	0	9	0	0
6ecc372e7b5d593afdad4f75 a918f414	0	0	0	2	0	0	0	0	0	0	0	0
a5aca6439553b3809c7d7ae0 29c6666c	0	0	0	4	0	0	0	0	0	0	0	0
15a09c73a5dc96a57a216b82 b2c2940e	0	0	3	0	0	0	0	0	0	0	0	0
46df0814d23da5f6add45373 9439e831	2	0	0	0	0	0	0	0	0	0	0	0
ef082f2b520a4cc008ad7cbfc a962af0	0	0	0	0	0	0	2	0	0	0	0	0

fe407b6b7cc97f1594922d25aeed108e	0	0	0	0	0	108	0	0	0	0	0	0
e99a5312eab6226986574f767d6a4e3b	0	0	0	0	0	0	0	0	115	0	0	0
5323fdc5d54d91afaf2643d3d3646271	0	0	0	0	0	0	0	3	0	0	0	0
0aeb1a9ccd7daf6f28848cf9cd3636cc	0	0	0	0	0	0	0	0	0	3	0	0
7a923dc4ebbafadd876d10ad58774c25	0	0	0	0	0	0	0	0	0	3	0	0
8b48ccdd25f184a28de7c9ebdd735e21	0	0	0	0	0	5	0	0	0	0	0	0
7eae46c5b960763b80d97c46369e87cd	0	0	0	0	0	0	0	0	4	0	0	0
4047cada51422f15e8950d77747e433b	0	0	0	0	0	0	0	0	0	3	0	0
214dcd6c851d867d4fa9ff7ff3b9ee6d	0	0	0	0	0	0	0	0	0	0	0	2
344e5c2b4e34fda1c63e4c33db248b4c	0	0	0	0	0	0	0	22	0	0	0	0
37c04ed17812a9c6e78ce991c27ac91e	0	0	0	0	0	2	0	0	0	0	0	0
b1c2e42c0f1032f7e248272b48bbe34d	0	0	0	0	0	6	0	0	0	0	0	0
81a8323ac3ac1ea057cc5c55d601d2da	0	0	0	0	0	0	0	0	0	0	0	16
cc2b67f4022eae1e02859588ec54c42b	0	0	0	0	0	0	0	0	2	0	0	0

6da1b968d17c4bb1169f535b0ff94e9a	0	0	5	0	0	0	0	0	0	0	0	0
f914cca0ed472df6fd2b778f509000e3	0	0	0	0	0	0	0	0	0	3	0	0
cb2a6f83b4783f763d793926b0142f0a	0	0	0	0	0	0	0	0	0	0	6	0
1d7c1a866560dd0d54ea1ca34d92c907	0	0	0	0	0	0	0	22	0	0	0	0
0dc7f8f956e80d08e41447967e0d6db8	0	0	0	0	0	0	16	0	0	0	0	0
5f2de98213312d36dae26fba7c44a828	0	0	0	0	0	0	0	0	0	0	0	3
ca9d8d98ee9c0be70c6ca26725ba9259	0	0	0	8	0	0	0	0	0	0	0	0
94e5c5986e511d00a768c0a0974a4c37	0	0	5	0	0	0	0	0	0	0	0	0
87149a1e783f92244c9bd1f3ee8464d9	0	0	0	0	0	0	0	0	0	0	2	0
1e50a38379932142a98c769f56f238fa	0	0	0	0	0	0	7	0	0	0	0	0
f681ae2bbe00b952b79f3c8857f94cb7	0	0	0	0	0	0	0	0	0	0	0	6
08de698ab68dd39e71ad9ee09bd530c4	0	0	0	0	0	0	0	0	0	3	0	0
adbc0594113b6da01d7616c22c468f56	0	0	0	0	0	0	0	0	0	3	0	0
f51ad3fdfe71fd42379104d08222066c	0	0	0	0	2	0	0	0	0	0	0	0

ea45e9c95cb8f7af16f09987f da27cf9	0	0	0	0	0	0	5	0	0	0	0	0
de29aadb62e06677441ba2c 6b31db6a0	0	0	0	0	0	0	0	0	0	0	0	2
2229352f84465928c4d3cb09 6fdb97fc	0	0	0	0	15	0	0	0	0	0	0	0
62b68de83f8690dce7b26dbf 5d8c9dea	0	0	0	0	3	0	0	0	0	0	0	0
dccdc82eebacc077524fa938 e81e4ccb	0	0	0	0	0	0	0	0	0	4	0	0
0e626a2b677a827cbb5f97ae 83144706	0	0	0	0	0	0	2	0	0	0	0	0
fa1bae1279e4172e45a77aa5 49570f2f	5	0	0	0	0	0	0	0	0	0	0	0
813a72d9ba06723949f54fea 70fa9faa	0	0	0	0	0	2	0	0	0	0	0	0
80d66464e58112e07746c7b 22c16f3dd	0	0	0	0	4	0	0	0	0	0	0	0
3ed3232a94b362dd0a096f97 6a1fd1a1	0	0	0	0	3	0	0	0	0	0	0	0
1f40eb702708298da067554b ec471151	0	0	0	0	3	0	0	0	0	0	0	0
a6327860977f98b48bfe4797 d9cca5f3	0	3	0	0	0	0	0	0	0	0	0	0
9d21446845fe07cbfd4c0f5fef 3b87e9	0	0	0	0	0	0	0	0	0	0	0	6
92dbda4c722e23c20bd5b61 b019f1396	0	0	0	4	0	0	0	0	0	0	0	0

109598487314d585970d615fe7bc5fd7	0	199	0	0	138	157	0	0	0	232	0	0
a05b0b024b7480d7f757967fb5ffcf2b	0	0	0	2	0	0	0	0	0	0	0	0
151d7957522a51d95c65dd6502f53688	0	0	15	0	0	0	0	0	0	0	0	0
24509140d787643b5b19ddf44d59128a	0	0	0	0	0	0	0	5	0	0	0	0
43169be2b2d5d4024ec9d5f155e63f53	0	0	0	0	0	0	8	0	0	0	0	0
2bc315ddac498506208924908a72874c	0	0	0	0	0	2	0	0	0	0	0	0
051f440523ab115ceb878efef9917516	0	0	0	0	0	5	0	0	0	0	0	0
1b515af2995b0c5feb1bbc3b7618a3c0	0	0	0	0	0	0	0	2	0	0	0	0
267217a352f8b9dc7f7e352411c9ef96	0	0	0	4	0	0	0	0	0	0	0	0
8d08614160e4c018ef9c6015611e6e85	0	0	0	0	0	0	18	0	0	0	0	0
ec22e8e8778d90d3417c053a54f8713e	0	0	0	0	0	0	0	5	0	0	0	0
e3302f79096d1d23321e8245edefc64e	0	0	0	0	0	0	0	0	0	2	0	0
b64e24af4ded2ce26cffc01d14a51fd9	0	0	0	5	0	0	0	0	0	0	0	0
dab58e25c445c39eadbb82133571d7cc	0	0	0	0	0	0	0	0	0	0	0	4

27d4c4b442938cce3b234d9794d107f1	0	0	0	0	0	0	0	0	3	0	0	0
5c07d9054cc40cf9d79d3a461a7f2dc6	0	0	0	0	0	0	14	0	0	0	0	0
9094164a3b29a134a397e17d3252640b	0	0	0	3	0	0	0	0	0	0	0	0
4fe1e36c651c3c99d3e8a7442fb833ae	0	0	0	4	0	0	0	0	0	0	0	0
d1bf4ca48c4eadadfdfeae3b5b240b36	0	0	26	0	0	0	0	0	0	0	0	0
a56d3dc3d15d2b2aa278af4c0bbe68ae	0	0	0	0	0	0	0	0	7	0	0	0
71dfeb8b8cf4fc87f4ee6434b79cc690	0	0	0	13	0	0	0	0	0	0	0	0
c165381ed769047e2669ac5c1d4aa731	0	0	0	0	0	0	0	4	0	0	0	0
813a1fb2bb13fda1569994e59e5c5244	0	0	0	0	14	0	0	0	0	0	0	0
37f4023d0cd5c086eb776a6a5970e6d2	0	0	0	0	0	0	0	0	0	0	0	42
caceac37481a17667f644fb1658cac41	0	0	0	0	0	3	0	0	0	0	0	0
e1d8fa881c75ecd5986a895eeb13fa2a	0	0	0	0	0	3	0	0	0	0	0	0
675c1be2a00ed86ee41f1469f3a9bd9a	0	0	8	0	0	0	0	0	0	0	0	0
7da4f2bbb638a18613d6125e177c4bd9	0	0	0	0	0	0	0	0	0	0	0	2

7dab6e7808d511cddef86e560da9c139	0	0	0	0	0	7	0	0	0	0	0	0
8d96867f4a8bc9d1f01a0e51d6086446	0	0	0	0	0	0	64	0	0	0	0	0
b8cdd8e2071d411e6a50a6d72e08334d	0	0	0	0	0	0	0	0	0	2	0	0
7ca6e06c8792134c0ee37827ee5547fa	0	0	0	0	0	0	0	0	0	0	0	2
ef0f19a86bbe779380f282e955aa3c26	0	0	0	0	0	0	0	11	0	0	0	0
01d93463ad705d059e990b2ef083674e	0	0	2	0	0	0	0	0	0	0	0	0
6108b49952b8860915561114df2b23d7	0	0	0	0	0	2	0	0	0	0	0	0
7c9adcefd489a2e21c24e4125cb59316	0	0	0	0	0	0	0	0	0	0	85	0
30ae4a7b45f8cdae1e5fc09f1f1cee3e	0	0	0	0	0	0	0	0	0	0	24	0
e78e5cf10e070ec8f9656f96cd597e49	0	0	0	0	0	0	0	0	15	0	0	0
e8b8407ab482086c056dca691ebf6db8	0	0	0	0	0	0	0	0	0	159	0	0
46ec8a32262a77b050c81bda796b497e	0	0	25	0	0	0	0	0	0	0	0	0
b5b5e2f8f57189b9d473e8ad1b9427ec	0	0	0	0	0	0	0	0	5	0	0	0
8cb755eacc330f6c3b7b1d0aefff7bb2	0	0	0	0	0	0	0	0	0	6	0	0

14a9fdf4ff74543185c10619f b2bd4cc	6	0	0	0	0	0	0	0	0	0	0	0
4e221117c94f9a56817c840e a2e4e913	0	0	0	0	0	0	0	0	0	0	8	0
cd0701b62463b9b1457f159a 6bf9d3cc	0	0	0	5	0	0	0	0	0	0	0	0
1909d7f5a11329ff515d8f790 061483d	0	0	0	2	0	0	0	0	0	0	0	0
94fcff445caaa11de3d1dc1d5 c615b70	0	0	0	0	0	3	0	0	0	0	0	0
d785dcff587372c1ee74f1ca3 5573908	0	0	0	0	0	29	0	0	0	0	0	0
6426d7d715b4b4671aec37e e121ba6b7	0	49	0	0	0	0	0	0	0	0	0	0
6a3cd82028d73dccd179f220 fbbedfa5	2	0	0	0	0	0	0	0	0	0	0	0
d8c831dc76f311264647cd74 927dcf3a	0	0	0	0	0	0	0	0	0	2	0	0
6e4cf89eb29b1fc0b6ede62d 3297e154	0	0	0	0	0	0	0	0	0	0	0	2
27ffd5d0d6f1d4c00463b01a 4a74deb5	0	0	2	0	0	0	0	0	0	0	0	0
b5187a346dd1956bf970ce1f 6dd042aa	0	0	0	0	0	0	0	0	0	0	0	26
86d66b1785dafc6a896851e2 048905f4	0	0	0	0	0	4	0	0	0	0	0	0
ef1386b36b8a614117334ea4 b29eaf13	0	0	0	0	0	4	0	0	0	0	0	0

a5f5c130aeefa24d75c4e948904410eb	0	0	0	0	0	0	0	0	0	6	0	0
10527b03623a4b2d7819cf350a8e2262	0	0	0	0	3	0	0	0	0	0	0	0
6fa2098eecf550316ef45748e1ffc8a7	0	0	0	0	0	0	15	0	0	0	0	0
27fc9527df5956ad162cc1de9248fc83	0	0	0	0	0	0	0	0	0	0	4	0
aff9a3663250f59a0adfb4dc28999c88	185	0	0	0	0	246	0	0	0	0	0	288
2a0e0faa78cd75a63338ba8a7f2eb59c	0	0	0	0	0	0	0	5	0	0	0	0
44426d10987fdb362a1c17c67c73f133	0	0	0	36	0	0	0	0	0	0	0	0
def87c031a12d86f1b05f364d882be8f	0	0	0	0	0	239	0	0	0	0	0	0
9cc603b83f7e0538c392db471ecbe94e	0	0	0	0	0	0	0	0	0	0	3	0
8a0ef821b622f258495ff86d9b097967	0	0	0	0	0	3	0	0	0	0	0	0
0b04d12d31885afee8f9947fdfdfd50c	0	0	0	0	0	0	0	15	0	0	0	0
eb78bef6b55fb0c27b7dcab4376e98ea	0	0	0	0	0	0	0	0	0	0	12	0
e38f0e66bdf32c776611fd6a2c1ddef4	0	0	0	0	0	5	0	0	0	0	0	0
afeda8db28cf6501d1fb152b4551be46	0	0	0	10	0	0	0	0	0	0	0	0

f4c607b8e5b6e47ca0ea158f5b8f7247	0	0	0	0	0	0	0	0	0	0	0	82
ba1aea2901b85656423700e62216cb79	0	0	0	0	6	0	0	0	0	0	0	0
176d59a6f8c3b7be4d668c63a6b4d316	0	0	0	0	4	0	0	0	0	0	0	0
95fe9058387ac164d85a7a143b8a1bd6	0	0	0	0	0	22	0	0	0	0	0	0
6049dbf23101f630f8ec163c1b86ed52	0	0	0	0	0	0	0	279	0	0	0	0
541323db3a87e596faa6eb3ee7d90fe8	0	0	0	0	0	0	5	0	0	0	0	0
2d91cb7adec57620f44007396a4a84b8	0	0	0	0	0	0	0	0	0	0	0	6
b635c9d71fc1503cea13d22363db971b	0	0	0	0	0	0	0	0	0	0	0	2
9dfbdacef863e98f5ec560afc8ed5ece	0	0	0	0	0	0	0	0	0	0	0	3
3ddbd5f6cf0ff1dcdb0a371a2a7d3ff0	0	0	0	0	0	7	0	0	0	0	0	0
e793a9f2c7fdf4d5ae49e4450e0163c0	2	0	0	0	0	0	0	0	0	0	0	0
925376c1dc3192fdca967a085d82104c	0	0	0	0	0	0	0	5	0	0	0	0
d20a5408049c2618a165ebc4a699ac36	0	0	4	0	0	0	0	0	0	0	0	0
6812478a46ffbe72e98c9d389b88378b	0	0	0	0	0	0	71	0	0	0	0	0

c56c1381a6f581bd7614b466708f3569	21	0	0	0	0	0	0	0	0	0	0	0
8d0969aa7d374e4dc00098295b1927ab	0	0	0	0	0	0	3	0	0	0	0	0
abeab8d22a55c2d3ce88640cb3ffea5d	0	0	0	8	0	0	0	0	0	0	0	0
e3c9f4eea0ddef65c8e1fb82ecc3948d	0	0	0	8	0	0	0	0	0	0	0	0
42c0bf128565c435b4deebeedbdc8240	0	0	3	0	0	0	0	0	0	0	0	0
e054acf2746448a1bb11577e0be2e15c	0	0	0	0	0	0	0	0	2	0	0	0
7f3b4912bed8f32372e02d7de95db699	0	0	0	0	0	0	0	0	0	4	0	0
24342d869e99ba426503998d31d7ed7e	0	0	0	0	0	0	0	0	0	0	0	7
eb489dbb8344c770e9b014c191de3a2c	0	0	0	0	0	0	0	0	68	0	0	0
44fcdcf2a02731b455121e2246dd2f07	0	0	0	0	0	0	0	0	0	0	2	0
e0c8bc1b6eeac7cc880039f328a2db3d	0	0	0	0	2	0	0	0	0	0	0	0
43783df455d54698fd940ae2b7f74dd0	0	0	0	0	0	0	4	0	0	0	0	0
38d665c0c2982a7b99a170df6c55dcab	0	0	0	0	0	14	0	0	0	0	0	0
4a2c86c020d7e677f89efb6aa3247309	0	0	0	0	0	0	0	3	0	0	0	0

4f5835a0bed0fe950e0175bbebe9f6b4	0	0	0	0	0	0	0	0	0	0	60	0
06c8c8ba393525219f039b8bc7ce95d3	0	0	0	0	0	0	8	0	0	0	0	0
4e56db33d4a390bb198a97658d6af0d9	0	0	0	0	0	0	0	0	5	0	0	0
d7678ddfd6b4d121c8da984628154ca1	0	0	0	0	0	0	0	0	0	0	5	0
91cf221600d5c135b54593e2fa8deeb4	0	0	0	0	0	4	0	0	0	0	0	0
caa5976a1faf3ea6bf7664aea883cdaf	0	0	0	0	0	0	0	0	0	0	21	0
cf6d24e02515d301bd53a4b783d3a2a7	0	0	0	2	0	0	0	0	0	0	0	0
6b6a6e9d1d2b4cb2d6c9a2cc229f0e22	0	0	0	0	0	0	0	23	0	0	0	0
414899bd5ffbfb8e84e676713f7ab2ed	0	0	0	0	0	0	0	0	0	0	0	20
c704a1e021590f202137c3071f0ee978	0	0	0	0	0	0	4	0	0	0	0	0
ec641443d70f660e2265e7cc86b82eda	0	0	0	0	0	0	0	0	0	0	0	7
976336673f88af0d5511314b50f9e4c6	0	0	5	0	0	0	0	0	0	0	0	0
95d9477bab0a26d900f8ff518ceb9e81	0	0	13	0	0	0	0	0	0	0	0	0
f391f1de65888c9cd7e096ae031b85dd	0	0	0	0	0	0	0	0	2	0	0	0

1c79cb5ec73b1363d00800b3d0d332a3	0	0	78	0	0	0	0	0	0	0	0	0
fa7d75a87dce9f5e169a051c18b2e8b0	0	0	0	0	3	0	0	0	0	0	0	0
72d6286bcfec35d84086044dcdad3652	0	0	0	0	0	0	0	0	0	5	0	0
5694981ac32af0ba4d6265e1a47029b1	0	0	0	0	0	0	0	0	0	0	23	0
3c28460d5b6af36a0c4847c48de53c46	0	0	0	29	0	0	0	0	0	0	0	0
af3fc9f0c0e0841694d0c15b601038d7	0	0	2	0	0	0	0	0	0	0	0	0
fc1795b8867d9467706de16bc477babf	0	0	0	0	0	0	0	0	0	4	0	0
fb3ddc3a0c62fdade4f740830e9d2356	0	0	0	0	0	0	0	0	0	0	5	0
790aece52d69b517ed60ab2b79c1f137	0	0	0	0	0	6	0	0	0	0	0	0
f828991ae7653ceaab84318215386415	0	0	0	0	2	0	0	0	0	0	0	0
0aa5c3e820429711eb56b5f592f80657	0	0	0	0	0	0	0	0	0	0	21	0
bccf9e222be2616454e7b3ea943c5203	0	0	0	0	0	0	0	0	0	0	2	0
45ce8be251eb6cdec12711d4799a43ec	0	2	0	0	0	0	0	0	0	0	0	0
e7809d32b7526f449d9f741a54be4cd5	0	0	0	0	0	0	0	0	0	0	3	0

1cf034aba05d63095ac21c61d11efb78	0	2	0	0	0	0	0	0	0	0	0	0
d7542e6f46d0d7078a3ded8736caca1e	0	0	0	7	0	0	0	0	0	0	0	0
5273b4b7ede8fb1ce324338f27fe811d	0	0	0	0	0	0	0	0	6	0	0	0
582489891ff8bb0ea9d390f261b72711	0	0	0	0	0	2	0	0	0	0	0	0
74b24e1e287d17a47a1eb33795dd57ff	0	0	0	0	11	0	0	0	0	0	0	0
a4a6d68c0d6887d3167f64d2b6b631ff	0	0	5	0	0	0	0	0	0	0	0	0
eef6a973ccf932c69e45491f4060f93b	0	5	0	0	0	0	0	0	0	0	0	0
068fc5935c0db20ddf89681a83e2bd08	0	0	0	0	0	6	0	0	0	0	0	0
f4c97b761fab2fbc707008c907994f1d	0	0	0	8	0	0	0	0	0	0	0	0
f5c3ad21372b17eb7d9975a927fab164	0	0	0	0	0	0	0	0	0	0	0	28
6e8b4dae413177b0750f98cd6d586a8e	0	0	0	0	0	0	0	0	0	0	2	0
61276620433de8c1e2a2e9d35296e046	0	0	0	0	0	0	0	0	0	0	4	0
37ee4362dfdda7d2c8a6f995c539b246	0	0	0	0	0	0	0	0	0	29	0	0
95f0499488c17b6407fe8c7e28953efc	0	0	0	0	0	0	2	0	0	0	0	0

b1ce26908a79fb40cb5fbcc6b56c1738	0	376	171	0	0	0	0	0	0	0	0	0
4bba29fd44f1321ebe93353c12d86aff	0	0	0	0	0	0	0	0	0	0	18	0
f085e14d1f6b892010e667f12374c896	0	0	0	0	7	0	0	0	0	0	0	0
306e1f4e277cb37e7584aac236570c06	0	0	0	0	6	0	0	0	0	0	0	0
65427882535b18f3c30afc2aa7acb3e3	0	0	0	0	22	0	0	0	0	0	0	0
86389ca8b32c52032a22b4bee219d253	0	0	0	0	0	0	0	42	0	0	0	0
da52769a320286a0eed0e2da87046fa9	75	0	0	0	84	0	0	0	0	165	0	0
afdaee090b4562292d1f8bc401a76c18	0	0	0	0	0	0	0	0	0	0	0	25
4232dc17f116b59900f99d9de04c89c2	0	0	0	0	0	0	0	0	0	0	2	0
fea93be039367fe10b520dbdf6e12a96	0	0	0	0	0	4	0	0	0	0	0	0
08a76d4be088fc840d05fd9aa8756e70	0	0	0	0	0	0	0	0	0	0	0	12
0a83b9f13e6b5568364ccf8c342743d1	0	0	6	0	0	0	0	0	0	0	0	0
c6e3abd823dfd4d028d35dca539bb947	0	0	0	0	0	0	0	2	0	0	0	0
42d0313b81456e5be73237dd210e19ca	0	0	0	0	0	0	0	0	0	0	37	0

1933c6055a786858893e06f09e0cb4c8	3	0	0	0	0	0	0	0	0	0	0	0
6ff9bb0335d4e613d590673a42ecef2d	0	0	0	0	12	0	0	0	0	0	0	0
869a6a291f2f830893c25f52525f32ab	0	0	0	4	0	0	0	0	0	0	0	0
ccd136215339ca4cc1cb48e21ab3ce4e	0	0	0	0	0	0	0	0	0	0	0	2
83501089ca3fdca52f9573a5e0e0798e	0	0	0	0	7	0	0	0	0	0	0	0
94b721852aa501b5d13d1519feca0851	0	0	0	5	0	0	0	0	0	0	0	0
d040e16898506a7ecf1b72bffea5b44a	0	0	0	0	0	0	0	0	0	3	0	0
729a4a43ba4aaa1caa42990823c79fbe	0	0	0	0	0	5	0	0	0	0	0	0
aa57dd50e900fd2dd6f7e8827d7dd2bc	61	0	0	0	0	0	0	0	0	0	0	0
4f0dc691b5a99882a06770a45f6e6bbe	0	0	0	0	0	0	0	0	0	0	0	11
26bf0eed437cbcf22b9f3ec9d810dd9a	0	0	0	0	0	0	0	0	2	0	0	0
ebb44a61c8a37469d5861c118ca463a4	0	0	0	0	0	0	0	0	8	0	0	0
7b869e485a045cfbca1753451a5fc481	0	0	0	0	0	0	0	16	0	0	0	0
85a66aac4e45cb698cf5c396b70b0650	0	0	3	0	0	0	0	0	0	0	0	0

9e88ace0df8fc523eb80ed450443d010	0	0	0	0	0	0	0	2	0	0	0	0
cd639e855450880ecf599cc334e62c81	0	0	0	0	0	0	0	0	0	64	0	0
e295795f16b7ca0996c7990319da3013	0	0	0	3	0	0	0	0	0	0	0	0
adaf6c3a1f130d82443ca21ee003fb90	0	0	0	0	0	0	105	0	0	0	0	0
75cf3f19481595761a3daea0ad5ef8a9	0	7	0	0	0	0	0	0	0	0	0	0
9ec495bf708185f5ea8a12bd6237b3f4	0	0	0	0	0	0	0	0	0	0	0	4
dfef1da13dc657dc24c7d6449824ac53	0	0	0	0	0	0	0	0	0	0	53	0
91abd642b6766d639bd4b7ab08dbb07f	0	0	0	43	0	0	0	0	0	0	0	0
e33bb71ae95ae5408d2ac6d4fe78f793	0	0	0	0	0	0	0	0	0	7	0	0
fbba8e2b85358d85bc58291f9aea5acc	0	0	0	0	0	0	0	0	0	0	0	2
47bdb8d7c3ed1324c481394b4e399451	0	0	0	0	0	0	0	0	0	3	0	0
cf313cf5952fb9d71d434cf7a5220158	0	4	0	0	0	0	0	0	0	0	0	0
f6c9bf3064d804fe661c251e1a0f6db7	0	0	0	0	0	0	0	0	0	0	24	0
a4a10cfc08433937453403578e0a7081	0	0	0	0	0	15	0	0	0	0	0	0

0827457f28612d7b8c0ca149a2b2899d	6	0	0	0	0	0	0	0	0	0	0	0
76bfab9331a578df758cfb7ae5765028	0	0	0	0	0	0	0	36	0	0	0	0
6079a6fdaa0398b17b70569f54d53db3	0	0	0	0	0	8	0	0	0	0	0	0
885a50d99210fef0dd02d9dc21acbaf4	0	9	0	0	0	0	0	0	0	0	0	0
9e788b7850e7089cb6f67b9f009af32c	0	0	0	0	0	0	0	0	0	0	2	0
4652b68223d408a8f350007ec4317648	0	0	0	0	23	0	0	0	0	0	0	0
d99f6d5d03d7bec40730d42bfa926425	0	0	0	0	0	0	0	0	0	0	3	0
4c5b8a4e08411c2bfa1b55a57c52cdf4	4	0	0	0	0	0	0	0	0	0	0	0
2108dc844cce9a1300e724a18d5d4d19	0	0	0	0	0	0	0	0	3	0	0	0
83f7befc5b8705ad64d040ff07e378eb	0	0	0	0	0	0	5	0	0	0	0	0
db35b5f84d057162feb70557177b252b	0	0	0	0	0	0	0	0	0	0	22	0
7b685ed61ebb4d25ec25dc2db844f852	0	0	2	0	0	0	0	0	0	0	0	0
bef48e9be9ba85f0e236a097b91a7b16	0	0	0	0	0	0	0	0	0	4	0	0
c3a40c113e7892f5fed17314c7411308	0	0	0	0	0	0	0	0	0	0	16	0

2d5c8c0e4b9859d8e9f82653 18b09058	0	0	0	0	41	0	0	0	0	0	0	0
06a6a881a72a1b972d9b432 2f7453de7	0	10	15	0	0	0	0	0	0	0	0	0
635317957bb08d09158e621 cbf0887b0	0	0	0	0	0	7	0	0	0	0	0	0
2b732b4f28632c4c9a681c5a d5df37ef	0	0	0	0	0	0	0	0	0	0	461	0
7a7a5ce5e7e46a062adae27c 0ef49ba3	0	0	0	0	0	0	0	0	0	0	0	23
d2aa64791bd1691c01f52dfd c4bebcc8	3	0	0	0	0	0	0	0	0	0	0	0
9c34dda4cf4f94274f057b9f3 d19ce40	0	0	0	0	0	0	0	3	0	0	0	0
c7cf2f427f15131b17433afed 7c65c5f	0	0	0	35	0	0	0	0	0	0	0	0
25e5bae008cbda964c700463 52b51a7c	0	0	14	0	0	0	0	0	0	0	0	0
7cb0ef4e8a6172943505c0aa 8bb49770	0	0	0	0	3	0	0	0	0	0	0	0
379282d095958257c86105f4 5eb9e332	0	0	13	0	0	0	0	0	0	0	0	0
25a9be3803f984ff17825629 1dfbe05f	0	0	0	6	0	0	0	0	0	0	0	0
a044f1dcb45434a077ff60be6 f0eba8f	0	0	0	4	0	0	0	0	0	0	0	0
226a31652e978e04260f7dae 7992b227	0	0	0	0	0	0	0	0	0	0	0	2

fa4c1f54581c76b2f0d20257bd9c8cea	0	0	0	3	0	0	0	0	0	0	0	0
cb150d1c5145f7047045cec8af0c6a35	0	0	15	0	0	0	0	0	0	0	0	0
5f1150971c006293970da00916e29909	0	0	0	0	5	0	0	0	0	0	0	0
15cc151b575b61261d70c31edac34cc2	0	0	0	0	0	0	0	13	0	0	0	0
c3ba6f2eb60d5821e691408821ef2ca8	0	0	0	0	0	0	0	2	0	0	0	0
5545460709b3da0ab9ff66a30cd6da4b	0	0	0	0	0	0	0	0	0	0	0	3
b29b396412785c57703363513f7c31d5	0	0	0	0	0	0	0	0	0	6	0	0
7f4333e3713dda7242646c5003c46da0	0	0	0	0	2	0	0	0	0	0	0	0
14b8193a687b4c551856946c88045054	0	0	0	0	0	0	0	0	2	0	0	0
a9b0357d87b80ef501402e1b5a943e2d	0	0	0	0	3	0	0	0	0	0	0	0
1c98ff4d0261dba2c1652c68784d3bff	0	0	0	0	0	0	0	0	0	86	0	0
29751be570496a79e693189146d11d68	0	0	0	0	0	93	0	0	0	0	0	0
9c94587f90688c0f8439c9b8e83d063e	0	0	0	0	0	5	0	0	0	0	0	0
2617df132d0a39fc574334708a198bfa	0	0	0	0	3	0	0	0	0	0	0	0

857ef8bc5efe964120ffed5feb cc678c	0	0	0	0	0	0	2	0	0	0	0	0
778487608f874f807e7826b5 495e42e6	0	0	0	0	0	0	9	0	0	0	0	0
748bf9c4c74149198fbd0f92b e0d0842	0	0	0	0	0	0	0	0	0	0	3	0
63b6b234234b8200bc2e0d8 48763a726	0	0	0	0	0	0	0	0	0	0	9	0
4de0b1c963c3a9205b5c9ccc c25d2cf0	37	0	0	0	0	0	0	0	0	0	0	0
fa25f1d685df89c4d6465bbb 1787a726	0	0	0	0	0	0	0	2	0	0	0	0
d675f0b8cd3380f9c7ec1812 3b8d42b8	13	0	0	0	0	0	0	0	0	0	0	0
ce53e78858061cbc68f86f66 36cd35ba	0	0	0	0	0	116	0	0	0	0	0	0
d44bcdef198b36fa59090376 bae1638c	0	0	3	0	0	0	0	0	0	0	0	0
0c354c579caccae1b44ca5a9 875e0781	0	4	0	0	0	0	0	0	0	0	0	0
5cd469afcc270263c806756b e1cf7db6	0	0	0	0	0	0	0	0	25	0	0	0
f76b3915156f0ddad89b31e9 e22c77b5	0	4	0	0	0	0	0	0	0	0	0	0
aad73821a5425616675545c 0ebfebb78	0	0	0	0	0	0	0	0	0	0	9	0
c24e891e8fa90663a945d9bd 6858b30f	0	0	0	0	0	0	0	0	5	0	0	0

a037466651721a97399edf8c81322e66	0	0	0	0	0	0	0	0	0	0	43	0
d39367a5d271ce4040c14cf241449e23	0	0	0	0	0	0	0	0	0	8	0	0
2b2df5edf83dfde40fb364458ce35ed1	0	0	0	0	0	58	0	0	0	0	0	0
38eb717bc5b1c76fb4b591abdc629131	0	0	27	0	0	0	0	0	0	0	0	0
6ef96279b85d2820b2ccc3495ea2bea2	0	0	0	0	0	0	0	0	0	18	0	0
24c844da20a06d654b305de838b34bce	0	0	0	0	2	0	0	0	0	0	0	0
94f069e71994e7bf97e9bb12c10440a9	0	0	0	0	0	0	0	0	0	127	0	0
bc435e83f86dd7f81ac0249f32a1da12	2	0	0	0	0	0	0	0	0	0	0	0
51bada273f3211fa84260195fefdf103	0	0	0	0	4	0	0	0	0	0	0	0
31baaf56a8f62c7b8fcb5d103d3ba7c0	0	0	0	0	0	0	0	0	0	0	0	9
06a2603bd9a56dcb7eb2c4027c19edc4	3	0	0	0	0	0	0	0	0	0	0	0
fdd7ae8e9583a3fc94fb172c2346b301	0	0	0	0	0	0	0	0	0	0	0	14
d82da67b51e6deb79950e3aa3ec9c487	0	91	0	0	0	0	0	0	0	0	0	0
a56c14dbbde2b91b9bfe4a95d7621e3b	0	0	0	3	0	0	0	0	0	0	0	0

dfd335df4f6513a413560f1aea8c1d95	0	0	0	0	0	0	0	0	0	0	12	0
451dea5a2e98a04ac524c38b5487160a	0	0	0	0	0	0	0	0	4	0	0	0
77131d8ea81f3c266a6f6a15a71a9b7f	0	0	0	0	191	0	0	183	0	0	289	0
69b8e2ccfb35ff4769c52e98b6cb0ee1	0	0	0	0	0	11	0	0	0	0	0	0
72f252341b2a7b4bf6f9bcf4e8caa715	0	0	0	12	0	0	0	0	0	0	0	0
5ec6cd7368ef221d74c64bc9b0d328fd	0	0	0	0	0	0	2	0	0	0	0	0
8d178a2bf3f63ea74d95aa88f0af7f4f	0	0	0	0	0	0	0	0	0	0	21	0
64fb486f69dfa46c51c519a9f21743a3	0	0	0	3	0	0	0	0	0	0	0	0
798cbdc2d2200380b2866a0634e6e0ce	0	0	0	0	0	0	0	0	0	0	22	0
440db98902ad56157ec30ace1bf0d246	0	0	0	0	0	0	0	2	0	0	0	0
bc800e843b1e1d680faf2c59a593ae73	0	0	0	0	0	0	0	0	0	3	0	0
93dcc5a8278b49bb197e60bce9989c7b	0	0	0	0	0	0	0	0	11	0	0	0
2f79047267d3e05837427f177d1818f3	0	0	0	0	0	0	0	0	0	0	3	0
fbbd41372ec58162343099d4d8fb5aed	0	0	0	0	0	0	0	0	0	9	0	0

fd95e6a0b4b07b4de07d565002bbd5f8	0	0	0	0	0	0	0	22	0	0	0	0
8072671094c69caf3b64fa97d8782f0f	0	0	0	0	0	0	0	0	0	0	2	0
7ea883106c0f41e17ad61c97680465bb	0	0	0	0	0	0	0	2	0	0	0	0
37b92b0e09ee358709b48928842461d7	0	3	0	0	0	0	0	0	0	0	0	0
c995cb95e965230a146c692003f28c2c	0	10	0	0	0	0	0	0	0	0	0	0
8bd273c3da147f98a802ed4703a9dabc	0	0	7	0	0	0	0	0	0	0	0	0
0ee9ee6ec03ed911715546e5021c76a0	0	0	0	0	0	0	0	64	0	0	0	0
c62d85fc229489358c5e8c6fc5bb6da9	0	0	4	0	0	0	0	0	0	0	0	0
7119b8b21a2672184e57ef7624dc0ed5	0	0	0	2	0	0	0	0	0	0	0	0
af3731c7d90c3db8814e735ffb296268	0	0	0	0	0	0	2	0	0	0	0	0
3dc94bc5e30ded4793be53a262b7be3b	0	0	0	0	2	0	0	0	0	0	0	0
a2641209c525fec5ceecdae8c80a01cd	0	0	427	0	0	0	0	0	0	0	0	0
38d11292f127de0c94afa9a638d333c4	0	0	0	0	0	850	0	0	0	0	0	0
b265e1ef379129c9fd6281d9cf4019e9	0	0	2	0	0	0	0	0	0	0	0	0

8b49b471019b6f7ce74df11a206b624d	0	0	0	0	7	0	0	0	0	0	0	0
dd8d9c95c26b5249d3920821f4ef8533	0	0	0	0	0	0	0	0	0	0	0	10
da7fb594c69e4fca4beae96fa61309d2	0	0	0	0	0	0	0	11	0	0	0	0
6b09443167acbe3322376dc16c4e987d	0	0	0	0	0	0	0	0	0	0	0	25
9c620faea494bf3d3ae02d5dc0d0710d	7	0	0	0	0	0	0	0	0	0	0	0
591c64e7de8dcb64e62fd47bd90b4262	0	0	0	0	0	0	0	0	0	0	0	4
de7d2360f5813bb2a754dd5a35e78a41	0	0	0	0	0	0	0	0	0	0	11	0
46fb1948e9a8a571fdb0173ab97d4486	13	0	0	0	0	0	0	0	0	0	0	0
050649c5d903e6b5cff968dfd31c48cf	0	0	0	0	0	0	0	0	0	0	8	0
432b3f262c0fdafd56bd36d4e822821a	8	0	0	0	0	0	0	0	0	0	0	0
6489ba0f0b31a49a8fbf30e2e92e7037	0	0	0	0	22	0	0	0	0	0	0	0
46338a3e3c17c38a0fd5c9c8476597a0	0	0	0	17	0	0	0	0	0	0	0	0
2fc8a6d542e1675fb2abbdcaf38a8efb	57	0	0	0	0	0	0	0	0	0	0	0
656c879d6a22e1d044f851a9953fc71e	0	0	0	0	0	0	3	0	0	0	0	0

1970872c0430cac3c9eeb2ab57b543ce	0	55	0	0	0	0	0	0	0	0	0	0
6f9c34525048536728e0074f249f48de	0	0	2	0	0	0	0	0	0	0	0	0
6a4ff607e649f0f5c94a595b96ac4a94	0	0	0	0	0	14	0	0	0	0	0	0
1727d1ec5554b07889410bd9ae6cd661	0	0	0	0	0	0	8	0	0	0	0	0
1b966a4ca944e9891c556f751873d8e9	155	0	0	0	0	0	0	0	0	0	0	0
0a493e1833ea3c7f7b4fdaec5ee5a028	0	0	0	0	0	0	0	4	0	0	0	0
c0145adb3f941d972ae13e0aa540b3ce	0	0	2	0	0	0	0	0	0	0	0	0
2b7a015e0daff895f2e3e854a24d7b16	0	0	0	0	0	11	0	0	0	0	0	0
e8be6b09fe405c0115321b594fcac91a	0	3	0	0	0	0	0	0	0	0	0	0
c1751a324ab161dc23c60bbad892b9b3	0	0	0	0	0	0	0	0	0	0	5	0
c16a14f469bd7fe123f92e448b0986ef	0	0	0	0	0	0	5	0	0	0	0	0
0a58ea44e7fe1a70e9c1ef6f08f543b2	0	0	0	0	0	0	0	0	0	0	0	2
9ea6239f2053a7861c296d68f771d94a	0	0	0	0	0	26	0	0	0	0	0	0
c302be9a5e6de0c1363667561c839bc8	28	0	0	0	0	0	0	0	0	0	0	0

bc0b6bee2cc1eb8678c21499f48a5e02	0	99	0	0	0	0	0	0	0	0	0	0
7f8a7cf3aa9ef13746313e8b679edee4	0	0	0	0	0	0	0	0	0	0	0	2
d627d6182fd0946ce9c7d52d748dc47f	0	0	17	0	0	0	0	0	0	0	0	0
1dc24feddba489104a3bcd1c3158a582	0	0	0	6	0	0	0	0	0	0	0	0
1747af80b7a5653dd6726dae347b19c3	2	0	0	0	0	0	0	0	0	0	0	0
0e2b83a1936971e60e8d5d9243f7b629	0	0	0	0	0	0	0	0	2	0	0	0
11763a8e17d40ec7449380544cc40263	0	0	32	0	0	0	0	0	0	0	0	0
c91f92b13f453af55bd0b7ad2dfdebe9	5	0	0	0	0	0	0	0	0	0	0	0
76e455dd54d2e82797a012005baa3109	0	0	0	0	0	3	0	0	0	0	0	0
1284358ce38e18ed36e5e8b621d24fdb	0	0	0	0	0	0	0	0	0	0	93	0
3d642b7342e2231a937f6ea073e7551b	0	6	0	0	0	0	0	0	0	0	0	0
24948f1fb9ea1bdd39c1650727cdc8cf	0	0	0	0	3	0	0	0	0	0	0	0
6fe5f47f66a1bbe722bf4795d868e7f5	0	0	0	0	0	18	0	0	0	0	0	0
0120d24b1144dec956ee5ef7f4b7e9b2	0	0	0	0	0	0	0	0	0	0	2	0

08157b0f4f9b0f17779f6a91585adc1c	0	0	0	0	0	0	0	0	0	0	17	0
cee327b1c78f5f17c97483e69db80b23	0	0	0	0	0	0	0	0	0	0	0	6
640e46c39919e3ef35586217ffe6168a	0	0	0	0	0	0	0	0	0	0	84	0
1ad3ac1af74eaecca712e00c125f5db5	0	0	0	0	0	3	0	0	0	0	0	0
ea36de4cf57c69138993c87438ac0d50	0	0	0	0	0	0	0	0	0	0	0	5
bceacac233284f1630e547528cd57d76	0	0	0	0	0	4	0	0	0	0	0	0
b9c1053b6ad58ed51a33d0e9ef4c3e5a	6	0	0	0	0	0	0	0	0	0	0	0
c4c9a93ab464f999ebebfb6229a8fbf4	0	0	0	0	0	0	0	0	0	0	0	3
212fc5a4d132a5fb3df63339bd7c72a4	0	0	0	0	0	0	0	0	0	0	3	0
31d4268a277e40453e6b5b94ee98eab0	0	0	3	0	0	0	0	0	0	0	0	0
c9b55bff7479927d14368edce1c04a79	0	0	0	0	0	0	0	0	0	3	0	0
4b5630c8eae47ad99e8650484dd2ad56	0	0	0	0	0	0	0	0	0	0	3	0
a515e6ea3df08c31f15698c804728fe0	0	0	0	0	0	0	0	0	0	9	0	0
9954f5812d49c7f1a253f1bb0bc5a5d0	0	0	0	0	0	0	0	81	0	0	0	0

e1fede58c3ce034d84cfcc52a43441b1	0	0	0	0	0	0	0	0	0	0	2	0
afa8a4326f22775fe47bcc03cccec5a7	0	0	0	9	0	0	0	0	0	0	0	0
5684c79a7010659d538b55686a5dbf1a	0	0	0	0	0	0	171	0	0	0	0	132
7ad6fa16eba9996002de0862b819617f	0	0	0	0	0	0	0	0	0	0	0	27
43d95b4d818b45cb1b75671c5fabaf97	0	0	0	8	0	0	0	0	0	0	0	0
38e300213a4b597adf40a9a1b7cb58cc	0	0	0	0	16	0	0	0	0	0	0	0
b6b916c5fc9421392dec8bf112d201a3	0	0	0	0	0	0	0	0	0	0	0	17
a959b482853ab9339edca67ddef0b22d	0	0	0	0	0	0	0	0	2	0	0	0
647d6e1cf880d8639cf8f02d54aa9a7e	0	0	0	0	0	0	0	0	0	21	0	0
410e5a3ae17ad619edaa6931019d1f08	0	0	0	0	0	7	0	0	0	0	0	0
241fc23b535e294ff3ad835b9af7ba9d	0	0	0	0	0	0	0	0	0	0	3	0
cce067e0377840c822f85b48d7a0ae4a	0	0	0	2	0	0	0	0	0	0	0	0
d77cc6f93acb1388ae89cf1bf80acb09	0	0	0	0	0	0	0	0	0	0	128	0
27bd00c0f20967ebe285e522b458a33e	0	0	0	0	0	0	0	0	0	0	0	2

982a6bd158290722b711a4df4f347e2a	0	0	0	0	0	0	12	0	0	0	0	0
5921c57de4a9d14311f9e522bd77ea2a	0	0	0	0	3	0	0	0	0	0	0	0
1ad3737bfac00a62e0a781c037e9f2f3	0	0	0	0	0	7	0	0	0	0	0	0
434465808366c90fca48f682c6dbfb65	0	0	0	0	0	0	0	0	0	0	0	39
bf2ed18f9cb47161ac84e38fdf386374	0	0	0	0	9	0	0	0	0	0	0	0
2e02dc2d4dd5b64d7a28b724e8fda0c3	0	0	0	0	0	0	0	0	3	0	0	0
19626a3b57a84029e772970b88041d03	0	0	4	0	0	0	0	0	0	0	0	0
da731ec623e9a4edebe6ae8bae884a93	35	0	0	75	0	0	0	0	58	0	0	0
39c203cd25bbe40e78de3f5d3bb33d68	0	0	0	0	0	0	0	0	13	0	0	0
6fe195dc8628f496f512072f7cde5a51	0	0	0	0	0	0	0	0	0	0	7	0
39cf03ae46c44b5b81c89dffa342ff41	0	0	0	13	0	0	0	0	0	0	0	0
9206446183eb0d8f2e171a9103fafcfc	0	0	0	0	0	0	0	0	0	0	2	0
19f7ded1d0e934beb8c83794d9d6fdf7	0	0	0	0	0	0	0	0	0	0	3	0
9034e5e0f778cd1a48abebc4526f1e04	0	0	53	0	0	0	0	0	0	0	0	0

bf8e041114f4d8d2b7f93b3c9c94bcb1	0	0	0	31	0	0	0	0	0	0	0	0
380ed49a8bedf31775765c88661e48fe	0	0	0	0	4	0	0	0	0	0	0	0
b720b4220a30ec1e342fd8d3e35befc6	0	0	0	0	0	12	0	0	0	0	0	0
cda85a6e1c9123a2858d92e8dc84e395	0	0	0	0	0	0	0	0	0	0	0	26
c14c7257f25518dd21c3561bf7680d6d	0	0	0	0	0	0	0	0	0	0	0	38
02dcef7b9384131c213d568ed3df473a	4	0	0	0	0	0	0	0	0	0	0	0
6762a89aef3c2fd62085e425345ca0ff	0	0	0	0	0	0	0	0	0	3	0	0
fc72e17580510f78a3554a6d26b92b65	0	0	0	0	0	0	0	0	0	0	0	2
7011bf8848550904730a38be868d1b63	32	0	0	0	0	0	0	0	0	0	0	0
fd614156704ad39bb2a88bb99eb130fb	3	0	0	0	0	0	0	0	0	0	0	0
4a8ac6399e6db4505a3af2ca43ecd60b	0	0	0	0	5	0	0	0	0	0	0	0
9e811b0551fcfcf353abc6adc0da0f6a	0	0	0	0	0	0	0	0	0	0	0	3
a0324015f928710cdd29b8f6047d20d5	0	0	0	0	6	0	0	0	0	0	0	0
f5e7251bb57be215eac2834e407125ad	0	0	0	0	0	0	0	0	0	0	0	7

a806a5503c3d7b701b189d2c9fa088da	0	0	0	6	0	0	0	0	0	0	0	0
8aa2d1a5e3e3d3e1dafe55dfffe5f6b4	0	0	0	2	0	0	0	0	0	0	0	0
7a4e25a592c5a7da4da04445ed6c1801	0	0	0	0	0	0	6	0	0	0	0	0
5785bc5d9ff278feaefe0919ff5efcb5	0	0	0	19	0	0	0	0	0	0	0	0
753672b539fb34e2101c2ef5b6a4ad8a	0	0	0	7	0	0	0	0	0	0	0	0
4f547fbc7c273e963305acbc14689812	0	0	0	0	0	0	0	0	0	0	9	0
337c8edd7a7636fd84221b5382836938	0	3	0	0	0	0	0	0	0	0	0	0
b6576120c525ff8bccafdfb1d76d151a	0	0	0	0	0	0	0	0	0	5	0	0
9a24b27fc8e5a9086911f6822acaf473	0	0	0	0	0	0	0	0	0	0	0	8
e38baf29778ef7463e4cd252a4f444d0	0	0	0	0	15	0	0	0	0	0	0	0
e04369a8a6cd4469dcdd7a2059be212b	40	0	0	0	0	0	0	0	0	0	0	0
57f083925717412d064152d43d623958	0	0	0	0	0	0	3	0	0	0	0	0
2ceea909ffbe6a64c5b864c752482c02	0	0	0	0	0	0	0	4	0	0	0	0
12649fe71e1cbde642b72fbfc292288c	0	0	0	0	0	0	0	0	2	0	0	0

e007e5caa8e8dd78fb2a7eb1702b3dc1	0	0	13	0	0	0	0	0	0	0	0	0
1cc7dcaee67f4a4a94002d0a01e5e980	0	0	0	0	0	0	2	0	0	0	0	0
6afd7c1879756e735ebac4c42ec958a0	0	0	0	0	0	0	0	0	5	0	0	0
8f477bd95bcc3c2cdcd8d707f480cdfe	0	0	0	0	0	0	0	0	0	0	0	4
a0c444c586fe20c55e8ecb5149c3f37f	0	0	0	0	0	0	0	0	0	0	0	39
c745e6f684142b73675d555419331a05	0	3	0	0	0	0	0	0	0	0	0	0
038347c311b11fa99dab78aeb5f80dec	0	0	0	0	0	0	0	0	0	34	0	0
ed0e55187cab4c3b2a443351b500ad1f	0	0	0	0	0	16	0	0	0	0	0	0
f821bf0a9bbfeeafd97731d6afef0041	0	0	0	0	0	0	0	4	0	0	0	0
400a5e83e77176474b72fb9c0f2becc0	0	0	117	0	111	0	0	0	0	0	0	0
09d2c47896668cfb5b15f4d3d3abe957	0	0	0	0	0	0	0	0	0	0	2	0
2acd865cf565e55bb9953ad03f28962b	0	0	0	0	12	0	0	0	0	0	0	0
7ab43cfed46cad319d9e8d3744c0028b	0	0	0	0	0	0	0	30	0	0	0	0
de578e72e4e1d32ffdc9905a2d8d7991	0	0	0	0	0	0	0	0	0	3	0	0

eeb76da4261fc5a1142d5eff08d10b07	0	0	69	0	0	0	0	0	0	0	0	0
f4ea07abe3aaa2fb9e94bf00cda68161	0	0	0	0	12	0	0	0	0	0	0	0
0095325fa62ad8f27420f19c4a7e83ea	0	0	0	0	0	5	0	0	0	0	0	0
0cd65333adf3e65d20cb0677d3a58c71	0	0	0	0	0	0	0	0	0	6	0	0
025b0c7a9721e97acf757e6435e2370c	0	0	0	0	0	0	6	0	0	0	0	0
09cd7a5dd24b5be50ac901eff903e39d	0	0	0	0	4	0	0	0	0	0	0	0
550e2ae4d836de32681f33a6e4cfdab9	0	0	0	0	0	0	0	0	0	0	0	3
0b3d0b7d529636611da439b62ee63156	0	0	0	0	6	0	0	0	0	0	0	0
38b87d42e3ca150e3dff396e8fc756ea	4	0	0	0	0	0	0	0	0	0	0	0
922782b2672863cbdec42a935dcebf9f	0	0	0	0	0	0	0	4	0	0	0	0
6130731ace42889c73164398286a6618	0	0	0	0	0	0	29	0	0	0	0	0
1ee328e9b2c5d67def70c8f37523e14f	0	0	0	0	0	0	0	0	0	0	8	0
793f4ad99dcdd6dfc206b31e001407e4	0	0	0	0	73	0	0	0	0	0	0	0
8122ae7fc53f37385c30db3b6af42cc5	0	0	0	0	0	0	0	0	0	189	142	0

48e3705b8fec9ff29f4b80b08d339909	0	3	0	0	0	0	0	0	0	0	0	0
1242a12eb85514c9db6a7f6e513f8384	0	0	0	0	0	0	0	0	0	0	0	2
e9c06c65ede07e79dd14936c368aca2c	0	0	0	2	0	0	0	0	0	0	0	0
79936873f9d10b30229db870ce5cbfe7	12	0	0	0	0	0	0	0	0	0	0	0
c02549c6b52f79c12d92f9acc88eb42f	0	0	0	0	0	0	0	4	0	0	0	0
d3b18f0f62bd3b54a9a73ade0ff5fa9c	0	0	0	0	0	0	0	0	0	0	0	8
7a84e65882c64bc3972ea88e5860db10	0	0	0	0	0	0	12	0	0	0	0	0
30e651ae4b5b5ef24439c95e3d7f0de6	0	0	0	0	0	0	0	0	0	3	0	0
53bf7babb3b502753f92ae0a5ad12b40	0	0	0	0	0	0	0	0	0	22	0	0
3aa65b5268fd901b77b44c59c3a2de9a	0	0	0	0	0	0	0	0	0	11	0	0
a939bbf921f6780ea7a72b95366f1c89	0	0	0	0	0	0	41	0	0	0	0	0
2e787ad4342b9868ca6f3d9615443ff1	0	0	0	0	2	0	0	0	0	0	0	0
b15f604d13e1988100e2d9f7307f2645	0	0	0	0	0	0	0	0	0	0	14	0
2e2da802a34521f93b0b43b959534f2b	0	2	0	0	0	0	0	0	0	0	0	0

2cd8788980e2107f412ccf878c178662	0	0	0	0	25	0	0	0	0	0	0	0
794ca841a5faef9aa125dcc38b94545e	0	0	0	0	0	0	0	0	0	4	0	0
ad67a97effc57f55f873f88792ae59fc	0	0	0	0	0	3	0	0	0	0	0	0
cb6cd00917028e1e9898e14d67ca13e8	0	0	0	0	30	0	0	0	0	0	0	0
10f3826d986d81360ae0c7e14342df38	0	12	0	0	0	0	0	0	0	0	0	0
9735db0499adccddb4d6c183385231b9	0	0	0	0	0	0	0	0	0	0	0	3
22fcfed24ca920b05cbe133bcae74bbc	0	0	0	0	0	0	0	0	4	0	0	0
734ad4c8d6bb2c9b7809a3268042eae9	0	0	0	0	0	0	0	0	2	0	0	0
25dd8fa0aabfdfdc6c395c85e6545b90	0	0	0	0	0	0	0	26	0	0	0	0
1d9426f2bf0d986c07aa64da8b93056f	0	0	0	0	0	0	0	0	13	0	0	0
aa2b74a1ac71b65f7709460b6d021360	0	0	0	0	0	0	0	0	2	0	0	0
7dd762a417457274e85a0378d2b393b4	0	0	0	0	0	0	0	0	0	0	4	0
fadd825ed4cd0967a181332becd252c4	0	0	0	0	0	0	0	2	0	0	0	0
f66183076058508e7e24e1daeac17d7b	0	0	0	0	0	60	0	0	23	0	0	0

4cae2ad9bd335cc6f3d3e7c1e51446ec	0	0	0	0	0	0	0	4	0	0	0	0
6591450d676241692cf635c3d02e9bc4	0	0	0	0	0	0	0	0	0	5	0	0
e763083c68fc7c0d21896ac626b9bd8a	0	0	0	0	2	0	0	0	0	0	0	0
610dc8c0285376add835f19a5b31f032	0	0	0	0	82	0	0	0	0	0	0	0
1e168f8ef9500d65b1eb4ce6e5905712	0	0	0	0	0	0	0	0	0	0	5	0
0a16523d7c85ae9adc7b6154ee2d03a7	0	0	323	0	0	0	0	343	0	0	0	142
619ab56e231985ac5ca337facc9fe6e7	27	0	0	0	0	0	0	0	0	0	0	0
465e3dff2eb80abfb843eb6382347498	147	0	0	0	0	0	0	0	0	0	0	0
d527d2c7e42eeea41957568e9b310a94	0	0	0	0	0	0	0	7	0	0	0	0
f800426dde39740358272a73a4859fcf	0	0	0	0	0	0	0	0	0	0	2	0
2327ee189bd373c9729e8ca68a868a4b	0	0	0	0	0	9	0	0	0	0	0	0
b827d871c02f1bec96544fb7b856de48	0	0	0	0	0	0	0	0	0	0	172	0
9885332c55570c69bcd2bac923caf45c	0	0	0	0	0	0	0	0	0	102	0	0
f5cb04ce713bd996d1ee99e9886819d4	0	0	0	0	0	0	0	0	0	0	5	0

9a180764e90b93d8d5bf3c8dc3c4dee1	0	0	0	0	0	0	0	0	0	2	0	0
f45fe19887fa2a13a9762f030bf621c9	0	0	0	0	0	0	0	0	0	0	7	0
2747eb08e0fa29138c41b65f702e0a64	0	0	0	0	0	0	0	0	0	0	0	4
7e52d39af94be9016b46f5be49d10e28	0	0	0	0	0	0	0	0	0	4	0	0
d0b69b07b8e28308050c45911466d8b7	0	0	0	0	0	0	3	0	0	0	0	0
e9136de7529af052a20602493558eec0	0	5	0	0	0	0	0	0	0	0	0	0
d67bb144d4d3f4f72616671225921dde	0	0	0	0	0	0	0	0	0	0	2	0
a014064f367cf65d72da44c5643c3871	0	0	0	0	0	0	0	0	0	0	184	0
5a3b31c341c1b7d31327168bec26119a	0	0	0	0	0	0	0	0	0	0	24	0
95314712e1cdc7754cdc99dee0b9b5bb	0	0	0	0	0	0	0	0	0	42	0	0
bf15e4549ddbabd149c3cb0c3917d505	0	0	0	0	0	0	0	0	0	0	4	0
9b9d0e2a06699f21abe041c4b327ca28	0	0	0	0	0	0	0	0	0	0	53	0
69d8a908572a8617ab0f773b7bdb8149	0	0	0	0	0	0	0	15	0	0	0	0
83cd1350e9b7bace27334704facf00cc	0	0	0	0	8	0	0	0	0	0	0	0

a63ac2df3572de9ffe28187b821bcfd3	0	0	0	0	0	0	0	0	0	2	0	0
5108d31641089a9dd517076c98490ff4	0	0	0	0	0	0	0	0	0	4	0	0
9ad707be584e0e345729cfc19cbb7ab6	0	0	5	0	0	0	0	0	0	0	0	0
07d2a8539298bd1b6a67947c9a9a20d2	0	0	2	0	0	0	0	0	0	0	0	0
0815bb585e89b948f66fe18a7f8f3da7	0	0	0	0	0	0	0	0	0	0	0	27
4c3c636cf0d6bedb606dec378e859816	0	0	0	0	0	0	0	0	0	0	3	0
c9db06b980da85c7c177dd787ae3a695	0	0	0	0	0	0	0	0	0	0	0	34
caf7e88c69c835e8573a90ffd09119a9	0	0	0	0	0	0	0	0	33	0	0	0
0205ea22c9dca13042536ed57215a4f8	0	0	0	0	0	0	0	0	4	0	0	0
4a53b8c6ab23e495786dc0b9bbab6ccb	0	0	0	0	0	0	0	0	0	0	4	0
db8e20f65735c953a8dc6d0b35582554	0	0	0	0	0	0	0	0	0	0	10	0
d563f75232bc92471121b0c766d9347c	0	0	0	0	0	0	0	0	0	0	0	3
d1f438692fffdc15732eeb469a37ee8d	0	0	0	0	0	6	0	0	0	0	0	0
0035e813f9026aa849eae1230e45f696	0	0	0	0	0	9	0	0	0	0	0	0

3a2530772233e6de4ac80a67133ca8ac	0	0	0	0	0	0	0	0	0	20	0	0
98023d01af089baebf7548d6a7da383b	0	0	0	0	0	0	0	0	9	0	0	0
5b18ee5804d230a13746aafef4de44ab	0	0	0	0	0	18	0	0	0	0	0	0
404a1fefd5ca5c242db5a4a6b24233c8	0	0	0	0	0	2	0	0	0	0	0	0
e864d39c3fcde42c39630c6fb01da4b6	0	0	0	0	0	0	0	0	0	0	59	0
f0fad5cc4e7190224836e8c978160ecc	0	0	0	0	0	28	0	0	0	0	0	0
ac6bc2c818218df375abd9d4592553e4	0	0	0	0	0	0	0	0	0	3	0	0
ceeea9e68aacf378d7cc188db2826dde	5	0	0	0	0	0	0	0	0	0	0	0
1ffb25638fccc18b09ebbdd52c6ac744	0	3	0	0	0	0	0	0	0	0	0	0
82d116a5a01f951aa6632cdee8213bd1	0	0	0	0	0	0	0	0	0	0	22	0
dba76f2f97fdc66be168f3c6ca8aa29b	0	0	0	12	0	0	0	0	0	0	0	0
f8f281d8634ecb7a174baa1e5c70f204	5	0	0	0	0	0	0	0	0	0	0	0
f614d5284c8f65e4d004d1c041deb6df	0	0	0	0	3	0	0	0	0	0	0	0
c81d6db8dc18b2fb1d2c40837a2870fa	0	0	0	0	3	0	0	0	0	0	0	0

0b1069cad823794665697d7897fc2bef	0	0	3	0	0	0	0	0	0	0	0	0
eb7e349714a7676b213698e704cc3c42	0	0	0	0	0	0	0	2	0	0	0	0
52adf7a92b70b56456ce63d44881d7e2	0	0	0	2	0	0	0	0	0	0	0	0
0c75cef1961f2aa82a4d1ca290c7e204	0	0	0	0	0	0	0	0	8	0	0	0
54b1edf43ae8e1aed2950689574a45c4	0	0	0	0	0	4	0	0	0	0	0	0
fb66c688635f2c5219756fced53a53fe	0	0	0	0	0	0	0	0	3	0	0	0
c271016cea6807ccf9716af2b9df7f40	0	0	0	0	0	0	0	0	4	0	0	0
2749f6a10cec2bb1f99526704b15ef0b	0	0	0	0	0	0	0	0	0	0	0	7
d4483481a24340f9f3db841ba56e48aa	0	0	0	0	0	7	0	0	0	0	0	0
9807e318363ca8ef5725c2aa96f2486c	0	0	0	0	24	0	0	0	0	0	0	0
9184abd9e4987b8accaefec250767449	0	0	0	0	0	0	0	0	0	0	8	0
3686b7d6b7a066dad4943853668372c1	0	0	0	0	0	0	0	0	7	0	0	0
71eb248b92cc651610a8fcce111ba43c	0	0	0	0	0	0	0	0	0	0	5	0
d364584cfc3dc6a4e95d53a50e7fec57	0	0	0	0	0	13	0	0	0	0	0	0

4cb0e662f4c558f8f476aca0f68c8a26	0	0	0	0	0	0	0	0	0	3	0	0
9e7d4f59b6f42a171771c9610c9165d1	0	6	0	0	0	0	0	0	0	0	0	0
592785b0707fb65c36ac313a3c154995	0	2	0	0	0	0	0	0	0	0	0	0
b81adfdf8ba2689556c1b671967864f9	0	0	0	0	10	0	0	0	0	0	0	0
64a3a53070c1be462fdc7e86d2f27941	0	0	0	0	0	0	0	0	8	0	0	0
67168f7b854107f906bbfc11e188a40b	0	0	0	3	0	0	0	0	0	0	0	0
0c1ea515e52181bbeccb283bf3c8e63a	0	0	0	0	0	2	0	0	0	0	0	0
7c84f7e43eaa679eae42fcd5d9754ca5	0	0	0	10	0	0	0	0	0	0	0	0
10cdc4a3a2fbcbca3625c2844c38639d	0	0	0	0	0	0	18	0	0	0	0	0
fedcb1c21b5d5e102da2651c83da170f	0	0	0	0	0	0	0	0	78	0	0	0
dc57867ac1f2b753898f39c9dfccf9e7	0	0	0	0	0	0	0	2	0	0	0	0
49c9b418931699e778f1dddf280427cb	3	0	0	0	0	0	0	0	0	0	0	0
1ecf03c6e90d6fb97039c187c79525b3	3	0	0	0	0	0	0	0	0	0	0	0
87f3c95da02c6f53907459321eab9130	0	0	0	0	0	2	0	0	0	0	0	0

8e2281fe5e03f82f0df5e0b9d4a42be8	0	0	0	0	0	0	4	0	0	0	0	0
3a2cc76f3724835fef55176fa6045644	0	0	0	0	0	0	0	0	0	3	0	0
f8459ac9faf9467f009ff7779789289d	0	0	0	0	0	0	0	0	0	0	10	0
a84338c036b6961db5b05ea1b95f5203	6	0	0	0	0	0	0	0	0	0	0	0
0e9efa498a0d1fdb092384e40f7adf93	0	0	0	0	0	0	0	37	0	0	0	0
f68ed8a5102d63eabcec887c91471454	0	0	0	0	0	0	0	3	0	0	0	0
877fac9e58e853ea7a8fd3ae31a89a4e	0	0	0	0	0	0	0	0	0	0	0	4
dac8ae98c563c3ea2182ea2e483b1532	0	6	0	0	0	0	0	0	0	0	0	0
dcaa2f28083b295580e0bca52a092113	0	0	0	0	0	0	0	0	0	0	0	5
2ced73ee943a6ba083c760c1b8adc06a	0	0	0	0	0	0	0	6	0	0	0	0
cbae72689341a0c9308030c8b59ae26b	0	0	0	0	0	0	0	0	0	0	0	4
0bc704fd572e6e2f5ce8127d8d76d90d	0	0	0	0	0	0	0	0	0	0	5	0
a24dac543c43dfec276137fc7a649dae	4	0	0	0	0	0	0	0	0	0	0	0
e83ff644a8754b3ee99abeff9f189a0a	0	0	0	0	0	0	0	0	0	0	0	3

971ff9ba01e6538ae0c0656a47ce01e0	0	0	0	0	0	0	0	7	0	0	0	0
c57698da10411c446ffb92e4d552f126	0	0	0	0	0	0	0	0	0	0	3	0
d35b77ec2847de06d711da6823eb87c5	0	0	0	0	0	0	0	0	2	0	0	0
51ded41ff5127aad4033623341c9d3a2	0	0	0	0	0	0	0	0	0	0	41	0
bcd84ca2f45ff797f4d33701e39d43af	0	0	0	0	0	0	0	16	0	0	0	0
91bca0e547d4c241896d075bf2e69b12	0	0	0	0	0	0	0	7	0	0	0	0
6ef805a3eeb9a6bd0e4dac41f7236314	0	0	49	0	0	0	0	0	0	0	0	0
f98fc758303b70d74f6e87dfa87afc7d	0	0	0	0	0	0	0	0	0	0	0	14
830a7d4345fc4286f9c371f0567fdf28	0	0	0	2	0	0	0	0	0	0	0	0
4bc3a62748a54b574d6baaf7b13056fb	0	0	0	0	0	0	2	0	0	0	0	0
2417938634b808a5e0aff00ea6b67efa	0	0	0	0	0	0	0	2	0	0	0	0
17ee85e02dc2b9aa9d7f89b56795bb57	0	0	0	0	0	0	0	0	0	10	0	0
b157f13045e07b37c9534d9a5a2b787e	0	23	0	0	0	0	0	0	0	0	0	0
4a6e635fd351d734e21446d80a6f8fb2	0	0	0	0	0	0	0	0	0	0	0	3

c98a64fdda92fc8f5ec7b0b00dcb7ce9	0	0	0	0	0	0	0	0	0	0	9	0
184fcedd83350312c4196e8f6f0a539f	0	0	0	0	0	3	0	0	0	0	0	0
4bfd3e9b66e06776ca62fb7f57898634	0	0	0	0	0	0	0	0	0	0	0	10
42384a32104e405d5895292c345aebd6	0	0	0	0	0	4	0	0	0	0	0	0
7a2b83f3a0b0c9329c6108d155dcefec	0	0	0	0	0	0	3	0	0	0	0	0
a9b6ddfc87fcd7e0a975439f48d9f7bb	0	0	0	0	0	0	0	0	0	0	0	2
3ee156fd188c1d1a0772866efb81a772	0	0	0	0	0	21	0	0	0	0	0	0
e5136bb3364554217d28d38f2bd68425	0	0	0	0	0	5	0	0	0	0	0	0
80028baa433ee5114f43f5cefaf1a2f6	0	0	0	0	0	0	0	0	42	0	0	0
5a4ca4fdf3a633d7cb1d2078f85de273	0	0	0	8	0	0	0	0	0	0	0	0
fa6c1f6929998e41e45e58bc8f951932	0	0	0	0	0	0	0	0	0	0	3	0
0867709d3c0addc76cf3fa73af4f70ac	0	0	0	0	0	0	0	0	0	0	3	0
cd64b09d420d7622f8c4d49b481daf0f	0	0	0	4	0	0	0	0	0	0	0	0
4f413d87ebffadfb804a72c98944c5f6	0	0	4	0	0	0	0	0	0	0	0	0

ceee698f07bf5d6ec6f5f1788cdb66c8	0	10	0	0	0	0	0	0	0	0	0	0
3aeac62f2bcb57fb34df0ed0230152ca	0	0	0	0	8	0	0	0	0	0	0	0
937715fab4c87e9af2ca19c2f020bbfc	0	0	0	5	0	0	0	0	0	0	0	0
a74bbce0925289e85eae963c50b7532f	0	0	0	0	0	0	0	0	0	0	0	2
8693669af620cc7965b8c05f7f168c24	0	0	0	0	0	0	0	0	0	0	0	2
d23fc3d84941395ee572e4c6166856d9	0	0	0	0	3	0	0	0	0	0	0	0
5e95215086b705f3af67abe588f5b2e8	0	0	0	0	0	7	0	0	0	0	0	0
d661c826e87b6cc7a87e9103d7786ded	0	0	0	0	0	0	71	0	0	0	65	0
048e6dc8bf600d3ce2fce3dc63c8f988	0	0	0	0	0	0	0	32	0	0	0	0
d2d0bb2bc0043a8c860ab90a9f52a922	30	0	0	0	0	0	0	0	0	0	0	0
2a93f6c88d6a7510b363c7292b03c9f7	0	0	0	12	0	0	0	0	0	0	0	0
3eca6d0d742a71688ff2186282e3f6a7	0	0	0	0	0	0	0	0	0	0	13	0
a6888b29d45468d885f9888b69a40b0d	0	38	0	0	0	0	0	0	0	0	0	0
97a6991ba5626ec05ac3bf0518cc3424	0	0	0	5	0	0	0	0	0	0	0	0

b57f75c70c6f2dbba3ffa8803282863c	0	0	0	0	0	0	0	0	0	0	0	4
01e49989a3a581ca60ab60f1a9f4c4f3	0	0	0	0	0	0	0	0	15	0	0	0
686b5ebf2eaac9e2c3f43e91a5b018c0	0	0	0	0	0	0	0	0	0	21	0	0
fae376550a2847415ac29084f89fb922	0	0	0	0	0	0	0	0	0	0	152	0
3f7cbde9c416408483f065257070dca8	0	0	0	0	0	0	0	0	0	0	4	0
aa3e0bedc63ce7b461b2a90d0a754f25	0	0	4	0	0	0	0	0	0	0	0	0
a5f387975b504c6dd58f800e20b69115	0	0	0	0	0	0	0	46	0	0	0	0
72305c655852333f0d59522404a8442e	0	0	0	0	0	0	0	0	0	0	5	0
1f673af034ab59e0cd6d1d943739d5df	0	60	0	0	0	92	0	0	0	98	0	0
57d4c545c8cea2a3f390a51f1ad474df	0	0	0	0	0	0	6	0	0	0	0	0
cd58c853bcedbe30dd55e0f0cd2bd5c4	0	0	0	0	0	0	0	0	0	0	0	5
6a6270e12058d48428ded433570410a3	0	0	0	0	0	0	0	0	0	0	8	0
4f7b202a8bd44b7c7ecf86e5e5dbcc6f	0	0	0	0	0	0	0	0	0	0	13	0
174a4d6d8014fa7a16c5cbdf2351a7fc	0	0	0	0	0	10	0	0	0	0	0	0

b6ea4804da2ab60b37c18e46000e4535	0	0	0	0	0	0	0	0	0	0	3	0
c018d9f3531274444c09ef1b7988b296	0	0	0	0	0	83	0	0	0	0	0	0
0437507ea53595f480099a693bf4bd6b	0	0	0	0	0	0	0	57	0	0	0	0
a1fe750674b0e4215deb9c564ba89789	0	0	0	0	0	0	0	0	0	0	16	0
62741444fedb7cdc3b03fd5796ace83a	0	0	0	0	0	0	0	0	0	2	0	0
a7f76bf828519d8bc6a75eec9dfd52cf	0	0	0	0	0	0	0	0	0	4	0	0
6bc70b99dbe1ea5b7d49cedec47b403b	15	0	0	0	0	0	0	0	0	0	0	0
97b959cec61895112563880af4afa4ee	0	0	0	0	0	0	0	0	9	0	0	0
779289c1b2218d8bd7dab18d779d7902	0	0	0	0	0	0	0	0	0	0	0	2
a99990cca343ae1bae6239f55c36bb75	0	0	0	0	0	0	0	0	3	0	0	0
332dff017f7b11e8a0c588a2ffc5f6d9	0	0	0	0	0	0	0	0	0	0	0	2
04f91a882ccfa82f37fac5cdf85dbb47	0	0	0	12	0	0	0	0	0	0	0	0
81c8615a3f412776a08d670e7e98dcb3	0	0	0	0	13	0	0	0	0	0	0	0
7966e443ac516a4bfabbbaf2e99a22f7	0	0	0	0	0	0	0	0	0	14	0	0

eca33ef5887cb45a2d784742 6523aa36	3	0	0	0	0	0	0	0	0	0	0	0
52ce97d0f3208b945ae98be1 5c4bee91	0	0	0	44	0	0	0	0	0	0	0	0
5d5c28dc3b3a1a32f32f8a56 63ea453c	3	0	0	0	0	0	0	0	0	0	0	0
14f544ab11cddd9508823ea8 9a1730e6	0	144	217	0	0	0	421	342	0	676	381	638
26c0f2b7036e05da9c9b1725 698cc2c8	0	0	0	0	0	25	0	0	0	0	0	0
253600792357107cdacd4f85 13b76b88	0	0	0	0	0	0	0	76	0	0	0	0
48ad2bc8bd5a758ebca358b 0a651e79f	0	0	0	0	0	0	0	69	0	0	0	0
17f29769a0e56d9175655cbd e15b78a2	0	0	0	0	0	0	0	0	0	0	4	0
f9c4b2ada5de82f31f61190d9 374ca58	0	0	0	0	0	0	0	4	0	0	0	0
bf514ddbc26412812c906490 b8fcb079	0	0	6	0	0	0	0	0	0	0	0	0
505f35db754d01af01c6763c 8de208a9	0	0	0	0	0	12	0	0	0	0	0	0
3138fd48e529382f5a5eec9e b6ced668	0	0	0	0	0	0	0	0	0	10	0	0
b8890dc2ac563ba48d3164c4 1595527b	0	0	0	0	0	0	0	5	0	0	0	0
f8ca86a1856e7f9d2027b217 1cb1c7bc	0	0	0	0	0	0	0	0	0	0	0	78

655ab901112fe78841e2c07a a8976bbf	7	0	0	0	0	0	0	0	0	0	0	0
f4dff0f3876eb7c9f1511cf82a 4aaa1f	0	0	0	0	7	0	0	0	0	0	0	0
5fab2c5eb58d734a8b520699 03d130f6	0	0	0	0	0	35	0	0	0	0	0	0
e1ca2b8e21c950f8469dc3c2 80bacd2c	0	0	0	0	10	0	0	0	0	0	0	0
73c75a187ea87c301108d991 b8a8d4ad	0	0	0	0	0	0	0	0	24	44	0	0
29e5c113499c1bf14caeb965 598d9084	5	0	0	0	0	0	0	0	0	0	0	0
486f09bd4371f39924507c43 43c95282	0	0	0	0	0	0	0	0	0	0	10	0
1302cc548cdd786980a74420 b87cb741	0	0	0	0	0	12	0	0	0	0	0	0
b314852a323f0e2211e69b5f aab8eae4	5	0	0	0	0	0	0	0	0	0	0	0
d4cff64ff366078da243f4c19d 8531dc	0	0	0	0	0	0	0	0	0	0	0	3
262d4c31f2625473d4803aa1 45ad0cfe	0	0	4	0	0	0	0	0	0	0	0	0
952b9d2edd2b984bb48f490 b61e48c06	0	0	0	0	0	0	0	0	0	14	0	0
07ff405b9d534a0bbc41c4dfd 924692f	0	0	0	0	0	8	0	0	0	0	0	0
f5b1b40254ccbe715f81c893 211e4907	0	0	0	0	0	0	0	0	0	0	0	3

dca347e41caa00ca97d48db7c6bb799c	0	0	0	0	0	0	0	0	0	0	3	0
b5444a7244888d9cc7da02656115aa3d	0	2	0	0	0	0	0	0	0	0	0	0
9dde96c3673650532cffdade08ebd6e0	0	0	0	0	4	0	0	0	0	0	0	0
5ee533495b9751631e975b987f44d5e1	0	0	9	0	0	0	0	0	0	0	0	0
c9c4f17d2b09f89599f292d92385f33d	0	0	4	0	0	0	0	0	0	0	0	0
1e31f4a335cc9ccf95d952a77e8e4be6	20	0	0	0	0	0	0	0	0	0	0	0
2118945f8d37d3558ecd3abee54b5e15	0	0	0	0	0	0	0	0	0	0	15	0
a3a55b1bf434143915b137450e87728f	0	0	0	0	0	0	0	0	0	0	2	0
7f23537e510b16625d2ee59798f5fff0	0	0	0	6	0	0	0	0	0	0	0	0
da9fe955976feba3a48c8995eeb7cbc7	0	0	0	0	0	18	0	0	0	0	0	0
abddaf07b7810ccde9fc78f8b48234b3	0	0	0	0	0	0	0	0	0	0	19	0
e54d8908a8833c600a1aed9cde0b42a5	0	0	0	4	0	0	0	0	0	0	0	0
15c7ea62b2c7d179dfd66c551b83d92f	0	0	0	0	0	0	35	0	0	0	0	0
0ab868c9766cc413ffa2afd4e8aa26bb	0	0	0	0	0	6	0	0	0	0	0	0

d0e205f6e25ed0d30cab0ad0b3c41b80	0	0	0	0	0	0	0	0	0	4	0	0
958e27aae8b29a5e9ad54ae86ad70d28	0	0	0	0	0	13	0	0	0	0	0	0
6891ec77babdac2d60f0fd5026fe137a	0	0	0	0	0	0	0	0	0	0	7	0
249889d26d3e5b8b65e8352196fd9130	0	0	0	0	0	0	0	5	0	0	0	0
4130d6fed8288acb6451e92d7c6d5f50	0	0	0	0	0	0	3	0	0	0	0	0
6fa2c56a767b9494204b7ecfc4ef9df5	0	0	0	0	0	0	0	0	6	0	0	0
cb0a69b048c151c84a3fc03ffc921678	0	0	0	0	0	20	0	0	0	0	0	0
6c1938f50339b1c8c7769e66ed48afc0	0	0	0	0	0	0	0	0	9	0	0	0
083d7eb1320e883e5bc2ce8b57feeb84	0	0	0	0	0	0	0	0	3	0	0	0
a6f5b0feb1f7de535219404ceb21c235	23	0	0	0	0	0	0	0	0	0	0	0
2597dd54729918ee7e55815ae470a871	0	0	3	0	0	0	0	0	0	0	0	0
15e8a2827e924609fb1bb7b0b9e0d976	0	0	0	0	0	0	10	0	0	0	0	0
8dda3c306aed12e8b968a6b587e21009	0	0	0	0	0	3	0	0	0	0	0	0
fbf7d1796a26d378594d26ba3902e89a	0	0	0	0	0	0	0	0	0	0	5	0

e5d420cd9d71e0380b1d0e40bcfd2c8f	0	0	21	0	0	0	0	0	0	0	0	0
d3dddb89720c17f9093ed3c90d2c4979	0	6	0	0	0	0	0	0	0	0	0	0
2b31448277926b307d02307869700216	0	0	0	0	0	0	0	0	0	0	0	3
8aa05c7491bc770ba39d4522e65f02f7	0	0	0	0	0	0	0	0	2	0	0	0
10949b50f71a4f2f50b587b7546d9aac	0	0	0	0	0	62	0	0	0	0	0	0
cfe0dc3584bb28201417f7b8706a9de4	0	0	0	0	0	0	0	0	0	0	0	3
67b9468cf145e541566f106cb8be66fe	0	0	0	0	0	0	10	0	0	0	0	0
fd9302a00b341740b4e826e19e9fac0f	0	0	0	0	2	0	0	0	0	0	0	0
7f5bccc7b16e9008b55e1da4a70f0912	0	0	0	0	2	0	0	0	0	0	0	0
8240fcc10e09784fcad434638d29e8c7	0	0	0	0	0	0	0	0	0	2	0	0
220c2b10a888e818ccd6da6b6e1df50a	0	0	0	0	0	0	0	3	0	0	0	0
c503631ac49053b494e59dbbc01d55ab	0	0	0	0	4	0	0	0	0	0	0	0
e7aa8c6182d2bdb59d0b2d9ca720101a	0	3	0	0	0	0	0	0	0	0	0	0
8ae73e4453d3efe451ef6b5f119c4bc1	0	0	0	0	0	0	0	0	0	42	0	0

78e4ebc0b76f1a3a8fafe6b8e09fb5fe	0	0	0	0	0	0	0	0	0	289	0	0
d4154b8e9fadcf55bdc913919c1bbe67	0	0	0	0	0	0	3	0	0	0	0	0
a018f0fc44a85b4b217a4140746d2c60	0	0	0	0	0	0	0	0	0	11	0	0
32c0c83f560850e51d08aef9a0b29554	0	0	0	0	0	0	0	19	0	0	0	0
8123711b1183881ae38bbf5f29a27802	0	0	0	0	0	0	0	0	3	0	0	0
2e30ae8eb7c404db39f2453cea7977fa	8	0	0	0	0	0	0	0	0	0	0	0
e58c11d03b9c5278f8321caf3dd16aba	0	0	0	0	0	0	10	0	0	0	0	0
07338d59e66b6f271c6784cee4200a88	0	0	0	0	0	0	0	0	0	0	3	0
8b28916683ad818ca077fdc452a10f13	0	0	0	0	0	0	0	2	0	0	0	0
22b336751a557e440eb6faa899e55f90	0	0	0	0	0	0	0	0	0	6	0	0
ec0384fe56d0f19768894bd71892c664	0	0	0	3	0	0	0	0	0	0	0	0
10668fd6426b7c9990879a7bcbbf7b0f	0	0	0	0	0	0	0	15	0	0	0	0
fcfb1d356b7cb12382bf3cf9f4c3f1a9	0	0	0	17	0	0	0	0	0	0	0	0
988f7cedb4e46a355472cb35070abd29	0	0	0	0	0	2	0	0	0	0	0	0

dc4339b476c3577ba34b9d6f9d871773	0	0	0	0	0	10	0	0	0	0	0	0
30640ec7a250ad88d43630d68091b03e	0	0	0	0	0	0	0	0	0	4	0	0
032083b2ac1059c0085140d0876eece6	0	0	0	0	0	0	0	0	0	0	32	0
a087a8b825e0bd3d8f73d74f8a0b4e6d	3	0	0	0	0	0	0	0	0	0	0	0
63c614adf23a2a5213e56911c410a4cf	0	0	0	0	0	0	0	3	0	0	0	0
8aa73e35a09060a381b587f7f838aad9	0	0	0	0	0	0	0	0	0	0	10	0
a57a81dbecc53ff66667850341271cd6	0	0	0	0	0	0	51	0	0	0	0	0
57102a9e62d4d31f46ca40b92727a415	0	8	0	0	0	0	0	0	0	0	0	0
27d0f3dd0c38e991a17e811baa1ace31	0	0	0	0	0	0	0	0	3	0	0	0
19958f8298535d833addeabb8e44e5a3	0	0	0	0	0	0	0	7	0	0	0	0
e4a6016b965e693d383990260f980014	0	0	0	0	0	0	0	2	0	0	0	0
7be6ac8a2de2a7c2dcac7a404a5e8049	0	0	0	0	0	0	3	0	0	0	0	0
59720dbe3b6ea2144d31564dd141a1c0	0	0	0	0	0	0	0	0	0	0	43	0
34324bc3849afd75ed537905efa6c8c0	0	0	0	0	0	0	0	0	0	0	0	2

b302c08e1475fc6f559501dc353667a8	0	0	0	0	0	0	38	0	0	0	0	0
3af975f046626f99e2fcb560bc614826	0	0	0	0	0	0	0	0	0	0	0	2
4d57791f4a5e274563ba21267cc4e704	0	0	2	0	0	0	0	0	0	0	0	0
779882026b97b23ca6a05aa66e029159	0	0	0	0	0	12	0	0	0	0	0	0
c8843b8f8ead14905af712bdde8f8105	0	0	0	54	0	0	0	0	0	0	0	0
2208e910e394a4a88d4b95eff571cf0b	0	0	0	0	0	0	0	0	0	0	9	0
82232cedae30060708d0aae8cb02bb66	0	0	0	0	0	0	0	0	0	0	0	10
02975d3dbf627123dafff16ab9e63788	0	0	0	0	0	39	0	0	0	0	0	0
15161b6913dc2b89ae00021cefdcfc5d	0	0	0	0	0	0	0	0	0	2	0	0
2116e44f057263edf82d5c98edb3d24c	0	0	0	0	0	9	0	0	0	0	0	0
8fcaf85449cafe5a2dabca104d535cfc	0	0	0	0	0	0	0	0	0	0	0	2
502eacedce13af43fe37281ac4496ef8	0	0	0	0	0	0	5	0	0	0	0	0
8b70706da34a9e1ca2f830f1d1cb1361	0	0	0	0	0	0	0	0	2	0	0	0
fdb207bfeccc159ea16afd007f467f77	0	0	0	0	0	0	0	0	0	0	0	2

6049457268f822261f08970301956c75	0	0	4	0	0	0	0	0	0	0	0	0
7e7b7a3e256037e8bd9bf2ec659b39af	0	0	0	2	0	0	0	0	0	0	0	0
dcc2951d66aa708a2d3b865f5322958f	0	0	0	0	0	6	0	0	0	0	0	0
3735a57ef70086ac30cdfc7486757580	0	0	0	0	0	0	0	0	0	6	0	0
67bf9bbe89a7e472f53ac049de595286	0	0	4	0	0	0	0	0	0	0	0	0
0b02fa8be0ef3e0f2ed22c10445be4e9	4	0	0	0	0	0	0	0	0	0	0	0
fb4438d72f61aa549b4fca92b17e916e	0	0	0	0	0	0	0	0	0	3	0	0
47096d3806ea9b8cee2614aeec7733ef	0	0	0	0	0	0	0	349	0	0	0	237
807c5649b5b1cd4a84a8a2605d96884b	0	0	0	0	0	52	0	0	0	0	0	0
147e885b695885b8fe9060e237698efa	0	10	0	0	0	0	0	0	0	0	0	0
23118dba309ee3f965adee0835d9677d	0	0	0	0	0	0	9	0	0	0	0	0
e5b863e3973a4db51045f8e7ede6dad1	0	0	0	0	0	0	0	0	0	0	0	3
7f6ffdc7680968dc9ee7cb5a19f25f0d	0	0	0	2	0	0	0	0	0	0	0	0
59fe466c507626488a6a274b1bde5156	0	0	0	0	0	0	0	0	0	7	0	0

315bbfa3ef8cb26e92c42e0182eb2324	0	0	0	0	0	0	0	0	0	0	2	0
752e90796ce840c059a9073cbc7eba64	0	0	0	0	0	0	0	0	0	15	0	0
04647d7941e0f44e52ef8b46e81c5c36	0	0	0	0	4	0	0	0	0	0	0	0
de4dd2bf2e3a74d24caecd360b0c543d	0	4	0	0	0	0	0	0	0	0	0	0
5babf49587916f92555a4b1c56208cad	0	0	0	0	0	0	0	0	0	0	0	2
2be76106156319629d4e639f72b534c6	0	0	0	0	0	0	0	0	0	2	0	0
97dcc958c0f931e4c9260c057682f64e	0	0	0	0	0	0	0	0	0	0	4	0
0c07c2435ec642fe345858ce14be266e	0	0	0	0	14	0	0	0	0	0	0	0
973abacc0453db674a4d81e5e24008eb	0	0	0	0	0	0	0	0	0	0	3	0
30604d423831b9ec654001de5766ea9f	0	0	0	0	0	0	0	0	0	0	7	0
6acc636e547e5d7cf0c65d5fbf26530a	0	0	48	0	0	0	0	0	0	0	0	0
b650a72384280c1cde5ccfaabf745852	0	0	6	0	0	0	0	0	0	0	0	0
ac8f0e215c1874f401041ffe4fa1f486	0	0	0	0	16	0	0	0	0	0	0	0
0e026e9e5d9f3f801b0fc768a92b67cb	0	0	0	0	0	5	0	0	0	0	0	0

34a3602a457568820d49368536bf8cf3	0	0	0	0	0	0	0	0	0	0	44	0
86a2c300fc68868247cb4f78c851affc	0	10	0	0	0	0	0	0	0	0	0	0
a4762c0149f34dbf5b91cb80d26c66b1	0	0	0	0	30	0	0	0	0	0	0	0
66b32f1d46031c724e17a8e611bea516	0	0	0	0	0	0	21	0	0	0	0	0
35672acbf9d068612e12f945d103240a	0	0	0	0	21	0	0	0	0	0	0	0
af944786c84f22be11aa715d25279ee4	0	0	0	0	0	0	3	0	0	0	0	0
3a7aae25586136fba92c3fdf4179b604	0	0	0	0	0	0	3	0	0	0	0	0
c6dc8317dacc4847ae8279defc3942aa	0	0	0	94	0	0	0	0	0	0	0	0
46cb53cf351369f5198ee63960d85f00	0	0	11	0	0	0	0	0	0	0	0	0
b43e06aa616c3caf568ca01fd8bd616d	0	0	0	0	0	0	0	0	0	0	4	0
7ef463deebbde774e601c11985140907	0	0	0	0	0	0	0	0	0	0	2	0
326e45797c5a5b549fbfa4ac172007e7	0	0	0	0	0	0	0	0	0	9	0	0
7ed3e1d8109bffa74243f53b2f0c1cce	0	0	0	0	0	0	0	0	0	0	3	0
c123f0c8d3babc7cf5bc59d1a8df3e41	0	0	0	0	0	0	0	0	27	0	0	0

3d0b60d1ed813e2d7a67e8d2e1a817d9	0	0	0	0	0	0	0	0	5	0	0	0
5cdeb61a215cbaa221943f9c2d0795cb	0	0	0	0	0	3	0	0	0	0	0	0
798570f608e0f8143810aede8ce499a4	0	0	0	0	0	0	0	0	0	0	0	6
618905bd9f9207fd7c0aea470acd1b87	0	0	0	0	3	0	0	0	0	0	0	0
750aaa02dd4c6088b07b5a876a2474af	0	0	0	0	0	0	5	0	0	0	0	0
b19fa520fd7b77fa4d0296ac8c0c34f9	0	0	0	0	0	0	0	0	0	0	3	0
e5c8ba0eb6ab374f94aee1e00f07b474	7	0	0	0	0	0	0	0	0	0	0	0
cd246d0e1199824365441b165528d24f	0	0	0	0	0	0	3	0	0	0	0	0
6f3b5dd8533617e8414ec6f47abf7f1e	0	0	0	0	0	0	0	0	0	3	0	0
3876b34386a9ba3d221890b09c8f9d61	0	0	6	0	0	0	0	0	0	0	0	0
1f5d092813426ac1c63f3ed10bf69733	0	0	0	0	0	0	0	0	0	0	0	7
ab50817a9b0d192af2a14e043a7168ff	0	0	0	0	0	0	0	0	0	0	2	0
467390be362699b9fad8ae5a5d94f762	0	0	0	0	0	0	0	0	0	0	44	0
498a81ac8e927b645e762310d43fafcf	0	0	6	0	0	0	0	0	0	0	0	0

b9e77f7bd57f7e6525f74dd520edd79a	0	0	0	0	0	0	0	0	0	0	0	16
ce81a50773dab54e005b6c36cd9bf7f4	0	49	59	0	0	0	0	0	0	0	0	0
5ff2d166145b8d5d74f3b2f510b2c454	0	0	0	0	0	0	7	0	0	0	0	0
96b4cd8b8c5d4c0e5110bed4d469192f	0	0	7	0	0	0	0	0	0	0	0	0
e412fa55b7ca5e55c04a877af1521dd5	0	0	0	0	0	0	0	0	0	0	0	7
e8380ae590c395ae6299a36c989adae6	0	0	0	0	0	0	0	0	0	0	2	0
ca2fe16981037d18e281d1adcfe4323c	0	0	0	0	0	0	0	0	0	0	0	5
720388488fa7006b779bd7b788da79bf	0	0	0	0	0	0	0	0	2	0	0	0
cc307b322a804ed42b7f36c71fdea848	0	0	0	0	4	0	0	0	0	0	0	0
9d8150f9062e72d128b09d36a56185c5	0	0	0	0	0	6	0	0	0	0	0	0
f4f7a55320c1e0659ee62eb4ee60349c	0	0	0	64	0	0	0	0	0	0	0	0
b9cb2fbc9b9becb7caf4a2e21b833f2a	0	0	90	0	0	0	0	0	0	0	0	0
f588672046bb69dd6334c894ae19ef30	0	0	0	0	0	0	0	0	0	0	3	0
2e1e0072aded495eb8e1f90706810740	0	0	51	0	0	0	0	0	0	0	0	0

f54b133d6b26c4ae1ee399da6fd8b213	0	0	0	0	0	0	3	0	0	0	0	0
ed51d81c0eb8f36bcc9b2b437905b66f	0	0	0	0	0	0	0	0	0	0	0	2
b137205b4f6df8a0105d24b425c194e1	0	3	0	0	0	0	0	0	0	0	0	0
07ece57569fb7e19ea3550bfdcad52fb	0	0	0	0	0	0	0	0	14	0	0	0
7c7242493955e5d08cdbefe1f480f48e	0	0	0	0	0	0	0	0	0	0	0	23
f5b73d0973fce861012b0fd6c2cb1774	0	0	0	0	2	0	0	0	0	0	0	0
2ed27aac385715e95a77957a4b187147	0	0	0	0	0	14	0	0	0	0	0	0
f7a4fca7ba047b9c259026af4ab41ae7	0	0	0	0	0	0	0	0	0	0	222	0
6f1ec35343b1130580078ebc26faedf3	0	0	0	0	0	0	0	0	0	0	4	0
12d42bb7acdc82cfba44dc4e6f766b4e	0	0	0	0	0	0	29	0	0	0	0	0
dbe4f88baaaeadb19fbbbdc2886c07ed	0	0	0	0	0	0	0	0	0	0	0	6
248fa8b06cd53c1109c655d1729f3415	0	0	5	0	0	0	0	0	0	0	0	0
e8e33c4789e33f6c33612b78016bd4e8	0	0	0	0	0	0	0	0	0	0	3	0
95108f9ead9ccec055d6fa005d0bf8b6	0	0	0	7	0	0	0	0	0	0	0	0

59207a3a3661ca64b065f0a10a0b4cc0	71	0	0	0	0	0	0	0	0	0	0
e5ef7c579a2b70884e4e67e9054c0caf	0	0	0	0	0	0	0	0	0	2	0	0
d72da3f0ea1e968b76919606e6f5735f	0	0	0	0	0	0	3	0	0	0	0	0
1ba60c0b56d533fa22569d5259f2564f	0	0	22	0	0	0	0	0	0	0	0	0
5428d82cf1184b95ff2b2cb113a71c10	0	2	0	0	0	0	0	0	0	0	0	0
b5e7c79caf78e4de79cd69b7f484176b	0	0	0	0	0	0	0	0	0	0	0	81
a6e025b12e253372c6ad342cae272b84	0	0	0	0	0	0	2	0	0	0	0	0
339beb405740bedc78f7e907e6993a29	0	0	0	0	0	0	0	0	0	0	4	0
282e67a02369a565980d4d572b80f560	0	0	0	0	0	4	0	0	0	0	0	0
414695ab0d0de0360cdd4794fa8bf437	0	0	0	0	0	126	0	0	0	0	0	0
8328a5134643e9ae3c34719cadbeee16	0	0	8	0	0	0	0	0	0	0	0	0
977b54408d2da83b4400c11b107eb19c	0	0	0	0	0	0	0	0	3	0	0	0
0ec756864198390c265c372a154c345e	0	0	0	0	4	0	0	0	0	0	0	0
6578be9194aa3472be5e7a251de623bc	0	0	0	0	0	0	0	3	0	0	0	0

aa76eae30a3321c0447db29c324584d5	0	0	0	0	0	0	0	0	0	3	0	0
e1d73b595676cffd2be110b7c4057713	0	0	0	0	0	0	0	0	62	0	0	0
81601ba70273117f0d3ec6e86d6005b7	0	6	0	0	0	0	0	0	0	0	0	0
d47e3a52d2efdafca2c3cb60616fe755	0	0	2	0	0	0	0	0	0	0	0	0
e7e134ea1c8d96c3395f7eedbe2de76b	0	0	0	0	0	0	52	0	0	0	0	0
c0ff3d9deb38a63161ff75fc2fd4fe1b	0	0	0	0	0	0	0	0	18	0	0	0
a068a055b66eab3d6b31a66a24420c0a	0	0	0	0	0	3	0	0	0	0	0	0
89b7181cbd2ab9f09127d708c99f44d5	0	0	0	0	0	0	0	7	0	0	0	0
130f539c404345deba500367d96739d1	0	0	0	0	0	2	0	0	0	0	0	0
ed147c1dc51d09d0f05d1b15d0316332	0	0	0	0	0	0	0	0	0	0	0	5
8a321d0d12376d32e354f620d2a45530	0	0	0	2	0	0	0	0	0	0	0	0
d24797f5b1d22fc975a6bf9aef21161e	0	0	0	0	0	4	0	0	0	0	0	0
1d1ddaa206eaa9102b97eb82083d46b3	0	0	0	0	0	0	0	0	0	0	39	0
025df5b2e73a579a166bec4a8b3ca9fc	0	0	0	0	0	0	0	0	0	0	0	21

622d38b9615fc2ca81121bab22f2a2c0	0	0	0	0	8	0	0	0	0	0	0	0
87800c38d14039620afe8d4d431e4419	0	0	0	0	0	0	0	0	0	0	0	7
d08b53e2cf8cf34535949536c4167e7f	0	0	0	0	12	0	0	0	0	0	0	0
a9ce9415cbac0c063aab37b61820946b	0	66	0	0	0	0	0	0	0	0	0	0
c40f22f60fe95167d5ee3231cd231317	0	0	0	0	0	0	0	0	0	105	0	0
2a542901ebfe5a2d2c621357a498721c	0	4	0	0	0	0	0	0	0	0	0	0
d664f6244aaaef29c1677e74339bd5c6	0	0	0	0	0	0	0	0	0	0	0	17
fc0b07f97783a478ce45af2e541bfe84	0	0	0	0	0	0	0	0	0	0	0	4
6eb8ea6ee453835cd42c6699670cac8d	0	0	0	0	38	0	0	0	0	0	0	47
6a3ff39a1cda42ee4fd1038e5d1819cd	0	0	0	0	0	0	0	0	4	0	0	0
30870fa0f397068107ecb51a7c84fa76	6	0	0	0	0	0	0	0	0	0	0	0
506417cd38869b6fc4950a296d23685e	0	0	0	0	0	53	0	33	0	0	0	0
ca8c2cf6455b1dd8ac58d492106d8cc1	0	0	0	0	0	55	0	0	0	0	0	0
d70f9e3254ea502223938a19f5805c36	0	0	0	0	0	0	0	0	0	0	48	0

172c4996a70be69c232b409 3be2f5bc2	0	0	0	15	0	0	0	0	0	0	0	0
f33dcbe5bebf120c96dc6356 ab1ab199	0	0	0	34	0	0	0	0	0	0	0	0
19b144cd5f7602e7698eb194 6925a19b	0	0	0	0	0	0	14	0	0	0	0	0
dca1262966f498fe8cb4cd11 e168b73a	0	0	15	0	0	0	0	0	0	0	0	0
651efccba12e5af477db21f19 8a8ebfe	0	0	0	0	0	0	0	0	0	0	6	0
bb1b1c69b18b2902a4d0d7e 477569a55	0	0	0	0	0	0	0	0	0	0	0	4
fd457416734df927d921b0f2 d9a328a2	0	0	0	0	0	0	0	0	2	0	0	0
da210c37f2958ff754ae137e6 cf23dc7	0	0	0	0	0	0	0	0	0	0	2	0
5fe24d7eaf7d0ee0a1f2ec96d 789e56d	0	0	0	5	0	0	0	0	0	0	0	0
0a02f1fcff7b4c7074ae42913 58c373d	0	0	0	3	0	0	0	0	0	0	0	0
a32e3f0c42aff1ca2b012840e d11ac19	0	0	0	0	0	0	0	0	0	0	7	0
c22f04f1cff5207f0ba4ba354b 41e064	0	0	0	0	5	0	0	0	0	0	0	0
9b0da499813937f52b2d99fe 14a91574	0	0	0	0	0	0	0	3	0	0	0	0
0d5c22d0885c2d68bb71178 af9be7c0b	0	0	0	0	0	0	0	0	0	0	0	6

63f62bb91a30decda9faf788d436b975	0	0	0	6	0	0	0	0	0	0	0	0
55858b3961db0593f61a598d68597b31	0	0	0	0	0	7	0	0	0	0	0	0
18584e631ab6fe48938461ceae698b7c	0	0	0	0	0	67	0	0	0	0	0	0
b7f9e7f4837a653f2a8de6d1389d85fa	0	0	0	0	0	0	0	0	9	0	0	0
363c607310d65a25bc4255f04e67ec12	0	0	0	0	0	0	0	0	0	0	8	0
b94424befc48822700c246691306a17a	0	0	0	0	31	0	0	0	0	0	0	0
1f6fe8b84b545a302524f50e8cde1438	0	0	0	0	0	0	0	0	0	0	0	5
a22002918d014472304ff8bb16f2d2b6	0	0	0	0	0	0	0	0	0	0	3	0
54298bd8e238d9d526ca1eb6c1a63dec	0	0	0	0	0	0	0	0	0	0	16	0
8374b9bd58e17c74950d2f882d89eabf	0	0	0	0	0	0	0	0	0	0	0	3
ab29fb78cd5da566e1d051623c050aa2	0	0	0	0	5	0	0	0	0	0	0	0
1cda692551b587b2e3150439b2d43113	0	0	0	0	0	0	0	0	0	2	0	0
d92aa7b8c95a7120c0fd1e3a85c88264	0	3	0	0	0	0	0	0	0	0	0	0
5f39eeb0441a27896acb9b1557667a17	0	0	0	0	0	0	0	0	0	0	0	2

8944e6003f536281c6df6717 2211e273	0	0	0	0	0	0	0	0	0	2	0	0
021e30619019ed12313d14d 686a0c3c7	0	0	0	0	0	0	0	0	4	0	0	0
363fde2305fd4fd4c6836e53b 238b808	0	0	0	0	0	0	0	0	0	9	0	0
be3f6428e9dbff01be465613 4e04b213	0	0	0	0	0	0	0	0	0	4	0	0
a7a90bd903f4338d5e349203 5b817e1c	0	0	0	0	0	0	0	0	0	0	5	0
76d98d15a3344820b2745f9c 881eb4e5	16	0	0	0	0	0	0	0	0	0	0	0
33119103a88f412d2405974d d62d423a	0	0	0	0	0	0	0	0	0	0	0	3
3d6c3fec4619ab0ed1761c27 64689e8b	0	0	0	0	0	5	0	0	0	0	0	0
281710b9c839ed8b8242c7ec 0a2bf94f	0	0	0	0	2	0	0	0	0	0	0	0
282d1d8359dabef030f5dc74 08557d7f	0	0	0	0	0	45	0	0	0	0	0	0
a347639b17e819fc28025286 3a1c9d35	0	0	0	0	0	0	0	0	0	0	0	4
aa6cbf19841b81d98740cab9 1905c1cc	0	0	0	0	0	3	0	0	0	0	0	0
63c495e248dd316c9d5e0e2 d1d5db72d	0	0	0	0	0	0	0	0	7	0	15	0
c50bdc40c1bfa812d9b6685e 1f0a30a9	0	0	0	0	3	0	0	0	0	0	0	0

c0cdac97c7b4f842072bfc8922f37ef6	0	0	0	0	0	0	0	0	0	0	4	0
0069064a35a5f45ff082c6a5d285b368	0	0	0	12	0	0	0	0	0	0	0	0
cb5545f2b1ecaaf9b25ea2f131df05ec	0	0	0	0	0	0	0	0	0	23	0	0
8f65b43086c8ae99831809457e857a31	0	0	0	0	0	0	0	0	6	0	0	0
21150f12d52dd572149c26522971a6e2	0	0	0	0	0	0	0	0	0	0	0	4
dc8f8ca856318fbff97f52e7f7174159	0	0	0	0	0	0	0	0	0	2	0	0
9b0fe4b1c90e83d6d63164900c8f210d	0	0	0	9	0	0	0	0	0	0	0	0
6988c66ae22c91fe7f6cc7e9bc630d0e	0	0	0	0	0	0	0	0	0	4	0	0
fff1a9da857df4f07e1f0696d9f00789	0	0	0	0	0	0	0	0	0	0	2	0
9e29ba6ecc0284b69c263560011a3181	0	0	2	0	0	0	0	0	0	0	0	0
79f2819d9595163b202a4ae2f061a826	0	0	0	0	0	0	0	0	0	0	344	0
3e4a541fad4f00d29d65518f157af207	0	3	0	0	0	0	0	0	0	0	0	0
5cdf3c7a46d29f9d52cfd90a4ef8bb00	0	13	0	0	0	0	0	0	0	0	0	0
beefa54277c0aa81dc7956ba86a9da1e	0	0	0	0	0	0	0	0	0	0	21	0

58e0bfddaffbfef8ccd358fb684463d9	0	0	0	0	0	0	0	73	0	0	0	0
839fc5486e60f29639897c886ebbaacd	0	0	32	0	0	0	0	0	0	0	0	0
b0735ecc6d4d0af062e680351789cf99	0	0	82	0	0	0	0	0	0	0	0	0
af02e87105a68b0f78992d8e824e616d	0	0	0	0	0	2	0	0	0	0	0	0
3b893e54dab5cb8471d1c53146bb8dae	0	0	0	0	0	0	0	0	0	0	3	0
f2e1e070205571cd38b0bd0f02b21c02	0	23	0	0	0	0	0	0	0	0	0	0
5bbf39ff9fc5529e99ae289ddb644637	0	0	0	0	0	3	0	0	0	0	0	0
26e7d9e17316e659bb272cbaa9d2667d	0	23	0	0	0	0	0	0	0	0	0	0
e18efa3e8d683883207f4ecbfc764af2	0	0	0	0	0	0	0	2	0	0	0	0
dc6ae9b6168ff97fac2d00a0c715a057	0	0	0	0	0	0	0	0	0	0	2	0
bd7fb202ae0ccbcfc17b0ca8e6a05fb7	0	0	0	5	0	0	0	0	0	0	0	0
be27b742f7f6569c189979d852d6b4ff	0	0	0	0	0	0	0	0	0	0	0	2
cfabcbb0d36a854afaac1a21754eca62	0	0	0	0	0	0	0	0	0	0	10	0
61e48df719790fe6af8e888c6eaf0287	0	0	0	0	0	17	0	0	0	0	0	0

ceca07c72c0a15aee63123fe7 a984fe7	0	0	0	0	0	6	0	0	0	0	0	0
4953c367c370d7e578a3faea c17827d9	0	0	0	0	0	0	0	36	0	0	0	0
40a6cd53737c38b0161651d 482dfbbbf	0	0	5	0	0	0	0	0	0	0	0	0
65af4bda3e3b1dbfe71bb595 dffa66bf	0	0	0	0	0	0	0	0	5	0	0	0
324edca38fac2c972b9cad5a 82abbfa9	0	0	0	0	0	0	0	2	0	0	0	0
e46511bc5ca04220c8ee2eef df01de29	0	0	0	0	0	0	0	0	0	0	0	3
9ccefb5cf0adad972e0ef6e3c 9d57c6b	0	0	0	0	0	0	0	0	0	0	0	4
fd8e7bd904ca096185bb4544 ae3428b4	0	0	0	0	4	0	0	0	0	0	0	0
00bd0120c06a88485625d8e 79a7b3955	0	0	0	0	0	18	0	0	0	0	0	0
5e645cced34ad0191782dc66 119585e9	0	0	0	0	3	0	0	0	0	0	0	0
b86f6fd7dd6d4fa2019e9fd20 ec479d4	0	0	6	0	0	0	0	0	0	0	0	0
cf5c901f1581223d9889f6354 9e470d3	0	0	0	0	0	0	10	0	0	0	0	0
2ccfe905a1558e0b0f90b7aa dbb2793a	0	0	3	0	0	0	0	0	0	0	0	0
c32a17219da98b4592f73774 fa739df4	0	0	0	0	0	0	14	0	0	0	0	0

279b83c47aa0ead940322feaca29c6fb	0	0	0	0	0	0	0	0	0	0	0	5
8ec6b04a49c5c5bc5232d29a1de4dbbc	0	9	0	0	0	0	0	0	0	0	0	0
dcb5ad68b1c6e7c53a05c7ed0edd7eb9	0	0	0	0	0	0	0	0	0	0	12	0
bb9548ad11b0a1c0bfc07cab13bf9e30	0	0	0	0	0	0	0	3	0	0	0	0
a87e724a8f34fa9751f21045725ad8a1	0	0	0	0	0	0	0	0	0	0	311	0
86b38eda150de3c3026b7ff6b2be7f86	0	0	0	0	0	0	0	0	0	9	0	0
843e5b6c503e64f0070354d3ca0f106a	0	0	0	0	0	0	0	14	0	0	0	0
a791edd10ef59424977b6beed1252f0a	0	0	0	0	0	0	0	172	0	236	0	0
8548fc5eca1de2559c0b8a8a9a46109c	0	0	0	0	0	0	0	0	0	0	14	0
44296baed4c1eedaf1a5e7c1814cba62	0	4	0	0	0	0	0	0	0	0	0	0
b53490cc48b04c2861393f908c416d44	0	0	9	0	0	0	0	0	0	0	0	0
8daaa1b688c4dbed97b657003c5cd215	0	0	0	0	0	7	0	0	0	0	0	0
664e9073b2a02795697716fd13a37835	0	0	0	0	0	0	0	0	0	0	7	0
a1f9971ce56375d13442ec0995e7e14a	0	0	0	0	0	0	4	0	0	0	0	0

e1ec91d5c9dca0ecdd267a469ce5b567	0	0	0	0	0	4	0	0	0	0	0	0
082986b765f45b150ef611fa331f17be	0	0	0	0	0	40	0	0	0	0	0	0
2a978f3b908e977f5615572e82e45dcc	0	0	0	0	0	3	0	0	0	0	0	0
44d492e0a9417fe4bf4ac99cac2c5b65	0	0	0	0	0	0	26	0	0	0	0	0
2981b6e222a4fc92db623e32bde2ba93	0	0	0	0	0	0	0	0	0	2	0	0
542f869389024d2164a75518ebaf2135	0	0	0	0	0	5	0	0	0	0	0	0
89467a9b903366595cbf7b8cdcf06a6a	0	0	0	0	0	0	0	0	0	8	0	0
97fc412a9a92a31d36fa38f213915e6a	0	8	0	0	0	0	0	0	0	0	0	0
706642b17bd1f09dabfd414d07856f65	0	0	0	0	11	0	0	0	0	0	0	0
3a64b3e0a9d9d48913db290e3cb632f1	0	0	0	0	0	0	0	0	0	47	0	104
251d56a935b816ca80cf3ffab5a24f86	0	0	0	32	0	0	0	0	0	0	0	0
69e696ea69ed9fb01d23deaf5e41fe00	0	0	0	0	0	0	0	0	0	13	0	0
14fa8ef25cd6991a0416de2aea6d6b82	0	0	0	0	47	0	0	0	0	0	0	0
47244d45e67dd6def706111b260bb8f6	0	0	0	0	0	6	0	0	0	0	0	0

9e4ce4d0904e92fa97374576 98d72e8f	0	0	0	0	0	69	0	0	0	0	0	0
f97cc2d6ec414be08972310fc 1132aba	66	0	0	0	0	0	0	0	0	0	0	0
5b1155f26b9c5c6fa57a75cd 5d19d233	0	0	0	0	0	4	0	0	0	0	0	0
43eb2791833416eb2b56bc9 62175751a	0	0	0	0	0	0	0	16	0	0	0	0
d97ea89a939855f25c08bf6d 2d21470f	0	0	0	0	0	2	0	0	0	0	0	0
42922750663e9e5bc42b4c6 df947e733	0	0	0	0	0	3	0	0	0	0	0	0
cbd03c7bf4ff5f621a1bf533b ac4436c	0	0	0	0	0	0	0	0	0	4	0	0
6413c1af2dec42948231342b 54ac4b04	0	0	0	0	0	4	0	0	0	0	0	0
4d02d843e4a98dc7b0a8702 2e7898c86	25	0	0	0	0	0	0	0	0	0	0	0
4dfde09b59872679a169916c bd4cf355	0	0	0	3	0	0	0	0	0	0	0	0
2a43c918953a25e623794d1 e02c10c9d	0	0	0	0	0	0	0	0	0	0	0	30
2cf2624b53322a5689e35a38 b3008458	0	0	0	0	0	14	0	0	0	0	0	0
a01a99ca465d5110d875a19 2274e6677	0	0	0	0	0	0	0	0	0	0	0	4
df7ddc543e7be1f5e59487d3 baa62475	4	0	0	0	0	0	0	0	0	0	0	0

e7665039729af089b5efc2a71972485e	0	0	0	10	0	0	0	0	0	0	0	0
2505a999bd6ea8d66902c35169e8bd16	0	0	0	0	0	0	23	0	0	0	0	0
e25c6937decea68a40e29cff265faf90	0	0	0	0	0	0	0	0	0	0	9	0
50881bbf0985ed072b4cb63f79aff467	0	0	0	0	0	0	0	2	0	0	0	0
330fac3d882f32c6c69e960e12eb3bc5	0	0	0	0	2	0	0	0	0	0	0	0
aea2c4723d15ff24e5df59a76ef0d085	0	0	0	0	0	0	0	0	0	0	2	0
cec1f1d321141df1f56b8298b79ae6d6	0	0	0	0	0	0	0	0	0	0	3	0
01969ac1096c29f63b017a78a009c9dc	0	0	5	0	0	0	0	0	0	0	0	0
bdb5e2c19ff86cf6b61fc18904a7cf33	0	0	0	0	70	0	0	0	0	0	0	0
795fc72cc6742ee29203139249469cf5	0	0	0	0	0	0	0	0	0	0	18	0
634cea3ae681799cf35f54aea2ad33ed	0	0	0	0	4	0	0	0	0	0	0	0
f4d86315555d94bf6333239e0d14675c	0	0	0	0	0	0	0	0	8	0	0	0
5ec8a5b9226febea9b2ddd60445df442	0	0	3	0	0	0	0	0	0	0	0	0
af167359aac1d37572c8da830899e0ef	0	0	0	0	0	0	0	0	17	0	0	0

d450099ca9e8e8c8e6fae09c 1490d57d	0	0	0	0	0	0	0	0	0	0	2	0
4dbcb721023eaecd87b6982 09046c73c	0	0	0	0	0	0	0	0	0	0	3	0
bafe7fa5b626426ee5495997 1d2ab536	0	0	0	0	0	8	0	0	0	0	0	0
3a68e255d754d8810e930cec 42b6e70c	0	0	0	0	0	0	2	0	0	0	0	0
67aa18db0a0e032ea201b94 a00f0b168	0	0	0	0	0	0	21	0	0	0	0	0
5588c452f36f305bf35fbf514 d89822b	0	0	0	0	0	2	0	0	0	0	0	0
facb286edd4b2bdf4ed0a8b4 a6f65715	0	0	0	0	0	0	0	0	0	0	0	7
1f455d33f3074b5a9223ac33 4a484f93	0	4	0	0	0	0	0	0	0	0	0	0
024ca0690dfefec7e620e73c2 f5d6895	0	0	3	0	0	0	0	0	0	0	0	0
35e95f5465bb142422f40cb3 1c5d360f	88	0	0	0	0	0	0	0	0	0	0	0
8ea1777e30803bae3fd6cb9e 72f21c01	0	0	0	0	0	0	2	0	0	0	0	0
0c0ecce6532dbffebf87a553d 1026722	0	0	0	0	0	3	0	0	0	0	0	0
408768e8c1ab99ecc1abc468 bf711104	0	0	0	0	0	0	0	0	0	17	0	0
710d997ce9fd59972a02acf3 a92c2121	0	0	0	0	0	0	0	0	0	0	0	2

5a43307aaed0ec74c3a2301904fb13c6	0	0	0	0	0	0	0	6	0	0	0	0
11472d7aca3f9e606ca848d487e2ec35	0	0	0	15	0	0	0	0	0	0	0	0
4b4d272c4c26bcc43b0234f37de58e19	0	0	0	4	0	0	0	0	0	0	0	0
631ec35d42d6e3cb0ae13541cc286a54	0	0	0	0	0	132	0	0	0	0	0	0
5f5be2e55792535b599cac4110795f55	4	0	0	0	0	0	0	0	0	0	0	0
50c1c55fe4957a806f9b9061d6f075e5	0	0	0	0	0	0	5	0	0	0	0	0
5f0f4bb9c63800142e7528f5521a7a96	0	0	0	0	0	0	0	0	0	0	3	0
fdf0043d3ea51a4944a7d5622dfa3b6f	0	0	0	0	0	0	0	2	0	0	0	0
a742c2c1cbe786ba056d7675cf42b8f2	0	0	9	0	0	0	0	0	0	0	0	0
0eae3216dc8932e92282a8b2455ff9a9	0	0	3	0	0	0	0	0	0	0	0	0
4a8aeccaccd1818bf899a98891737726	0	0	0	5	0	0	0	0	0	0	0	0
bfe7401ee0829e05310d2e8a4d2aec7e	0	0	0	0	0	0	0	0	0	0	0	7
8786c5f3d84c25a2cb588d414da8112b	0	0	0	0	0	0	0	0	0	245	341	53
6074d54d4b251851c15580e65293fb2b	0	0	0	0	7	0	0	0	0	0	0	0

1e669252d42f2bc579c27489a8b5050a	4	0	0	0	0	0	0	0	0	0	0	0
1236ee07171d269ea9134c0372cbfadc	0	0	0	0	0	0	0	0	9	0	0	0
fb327ddb13e788bc48b0a919db61f536	0	0	0	0	0	0	0	0	0	3	0	0
33684108f857adadd20a96ec4dcf0c55	0	0	0	0	0	0	13	0	0	0	0	0
9dccc5f3ded21c7e5b968ef91aff1fff	0	0	0	0	0	0	0	0	0	0	9	0
b2e2f8957018e2bea7d1e49ccd6e71ef	0	0	0	0	0	0	0	3	0	0	0	0
fe07d1937ada0248c9f9bc37f8eac199	0	0	0	0	0	0	0	0	0	0	8	0
6d26e7101dce488dc2ff708af843b562	0	0	0	0	0	0	0	0	0	8	0	0
27a7a4579c0581fa945a9b91e85b1587	0	0	0	0	0	0	0	0	10	0	0	0
7a8ab4ef4b08dcff1e1e2e505f81d92f	0	0	0	0	0	3	0	0	0	0	0	0
a009eb03b9e49778411d6fe085d4bc40	0	0	0	0	0	0	2	0	0	0	0	0
329d51b1d2f1b8a4cdec2a99f5f02fea	0	0	0	0	0	0	0	0	0	3	0	0
2792bf98bc93d7313983a56ff7968a58	0	0	8	0	0	0	0	0	0	0	0	0
ea2fbff8122765e796989e1ba9cf488d	0	0	0	0	0	26	0	0	0	0	0	0

d6511087c75660211edb1b42cfcf0504	0	0	0	0	0	0	0	0	0	0	11	0
3b3cbda0b20e973c9eb6ea167d1044e8	0	0	0	0	0	0	0	0	0	0	0	5
0ced7a8c3b763c520700a87754d9b08c	0	0	5	0	0	0	0	0	0	0	0	0
82b9fb6d70c08dea20a8d7dc9b8504ae	0	0	0	0	0	0	20	0	0	0	0	0
39165e62666940527034fcc5dddbc995	0	0	0	0	0	0	7	0	0	0	0	0
dc934bf7b07d99e0aa7714b320f5cddf	2	0	0	0	0	0	0	0	0	0	0	0
fe71a6bba7720709d2aff9ac3585651c	0	0	0	0	0	0	0	0	0	6	0	0
f81c965a0143deaa8610b61be35ccbf2	0	0	0	4	0	0	0	0	0	0	0	0
6a0439e51e57b1dfdd0551c60dccafd7	0	0	122	118	0	144	0	0	0	210	0	0
497f43e213407a567e707630089d392d	0	0	0	0	0	0	0	0	0	0	31	0
200537b2e706e664f4a67bf435d51be1	6	0	0	0	0	0	0	0	0	0	0	0
e7a9d029f33094a6d26522fa3c0f778f	0	0	0	0	0	0	0	36	0	0	0	0
c4b06ebaeef24ad931463835a00a5e91	0	0	0	0	0	0	11	0	0	0	0	0
4ee7a89fa2f0268e2a7f3add0f009d62	0	0	0	0	0	0	0	3	0	0	0	0

b2d557de0ae031a8a1c33c4de29fc387	0	0	0	0	0	3	0	0	0	0	0	0
c91bd91d61a704354e333a3bf2429321	0	0	0	0	0	0	0	0	0	0	0	7
5dc7e819d12ce72567fee4b02611fac9	0	0	0	4	0	0	0	0	0	0	0	0
cc8832db64d06c2b639c8a717feed254	0	0	0	0	0	2	0	0	0	0	0	0
b85ebccc7881d1db7112f30956071f1d	0	0	0	0	0	0	0	0	3	0	0	0
008e4d4327cbb151f0e85a7c686c898e	0	0	0	0	0	8	0	0	0	0	0	0
54b4b8a30677f45b86294137c27cb91c	0	0	0	0	0	0	3	0	0	0	0	0
7aba6c4899d45996cc792f22cec347be	0	3	0	0	0	0	0	0	0	0	0	0
e8cade065e54b96878f071be4072e64f	0	0	0	0	0	0	33	0	0	0	0	0
b6b92f34e1d3d3ff8282bf402abf4b77	0	0	0	0	0	0	0	0	0	0	0	3
e807edf8cf87011481a6d8ef64ef2a53	0	0	0	0	0	0	0	0	0	0	2	0
688227a0a7fd763325d700ad9e551fab	0	0	0	0	0	0	0	0	0	5	0	0
3752d5ac6da0e2a0711097ce524762fe	0	0	0	0	0	0	0	0	0	0	0	5
ea28c93bca4e8a8ae5d981112f3577f2	0	0	0	0	0	0	0	0	3	0	0	0

c321a493836970f7c1261d39ce6ebe91	63	0	0	0	0	0	0	0	0	0	0	0
fec1dd9a5653883e7fe081b3c345d1bc	0	0	0	0	0	0	0	0	0	0	2	0
552704465c5dc61a0165bf5a7f73a238	0	0	0	0	0	3	0	0	0	0	0	0
d2d4581b2d3cfcf6ca9d0c6ed13dfb72	0	161	0	0	114	0	0	0	0	0	0	0
5d93d1f02a4632ca518632a2fc0cdab5	0	0	0	0	0	0	0	3	0	0	0	0
4d5c0f6f93024687955704a473255234	0	0	0	4	0	0	0	0	0	0	0	0
e6f93240351d9b1b2b3d4494d75cc7a8	0	0	0	0	0	0	0	0	281	0	0	0
1b59691c08b54ba9a0cb3b11103e21e3	0	0	0	0	0	0	0	0	0	0	36	0
37d4ecdf81144175891c12a87c6421c5	0	0	0	0	0	0	0	0	0	0	0	5
9c0756e090c709efc8c17cb5ad329206	0	0	0	0	0	0	0	0	0	4	0	0
195e5f58140f2a42daf44eda1bba9a34	0	0	0	0	0	6	0	0	0	0	0	0
c2957b12e1daafd1d9d185d54eff83fd	0	0	0	0	0	0	0	0	0	0	0	11
d5a43ccc742c2ee5fc3d043c104c3909	0	0	0	0	0	0	0	0	0	0	18	0
0c3654d8b21d44d2d0ab55f38cee0939	0	0	0	0	0	0	0	0	0	0	45	0

9aa0f2ca5fef55939aa15dd934dc2456	0	0	0	0	0	0	0	0	0	0	4	0
292aa1e5650675b696a258950235f339	0	0	0	0	4	0	0	0	0	0	0	0
d518ffa311c95c0cefac8713e79bc1f2	2	0	0	0	0	0	0	0	0	0	0	0
adb649204aef9ac8371c38fe0c382f82	0	17	0	0	0	0	0	0	0	0	0	0
875c6ac3653c4ef76706632f93c952f2	3	0	0	0	0	0	0	0	0	0	0	0
54869d7b0e77fdeee63e17ecce361dac	0	0	0	0	22	0	0	0	0	0	0	0
ac412f73677dfd77c3f8dec03701ee64	0	0	0	0	0	0	0	0	0	0	111	0
db7560d9da433bca7ef1d61e1f130b10	0	0	0	0	0	4	0	0	0	0	0	0
fc6e5ad2250116f49f8703863a95f4e3	0	0	2	0	0	0	0	0	0	0	0	0
4081130d18234c76b0dced7a6c439285	0	0	7	0	0	0	0	0	0	0	0	0
da33df48816afe736ed42f2e3d62b4cd	0	0	0	0	0	0	0	0	3	0	0	0
0ef757cd68e70730e5f283eb287b8972	0	0	0	0	0	0	0	0	0	0	36	0
87c09810ba5be15927c364f4b697bdb8	0	0	0	0	0	0	0	0	4	0	0	0
a45a063c078d11f2fba50f4e488fbfb1	0	0	0	0	0	0	0	2	0	0	0	0

6778218ec4167d59b8af9208 95435f7d	0	0	0	0	0	0	0	0	12	0	0	0
0d35917a43c494538c1c4ea4 82f4d6ab	0	0	0	0	0	0	0	0	9	0	0	0
5d6aa1cb8a20bc61587e8508 0bad681f	2	0	0	0	0	0	0	0	0	0	0	0
99ad2ef2d9a525a60448589c 988ede5b	0	0	0	0	0	0	0	0	0	19	0	0
a23b71546ca376a48f79e0f4 9d4f55e1	0	0	0	0	10	0	0	0	0	0	0	0
302791bac379ab14ce53718d 6b738092	0	0	0	0	0	2	0	0	0	0	0	0
e89fa6c90d9d2ca923119470 ac401ac3	0	0	0	0	0	0	0	0	0	0	5	0
b86b2657df47304295c357c2 02c0908b	0	0	0	0	0	3	0	0	0	0	0	0
15938272e57924181829012 5cd648ab2	0	0	0	0	0	0	0	0	0	0	0	28
b138dd156727ae8894ef26dc 023df4b1	0	0	0	0	0	0	0	0	0	0	25	0
a6a135ade8a4f51ed740d948 fb1fc797	0	0	0	0	0	0	0	0	0	0	0	11
2ddc128b3f220386a5d27c46 29e0f46c	0	0	0	0	0	0	0	0	0	0	3	0
0f707b2d8c634089b4a052be 4b1b4b09	0	0	0	24	0	0	0	0	0	0	0	0
2b44f9d78d5a94834425f660 65f962d9	0	0	0	0	0	0	0	0	0	0	12	0

050f69f9fbfa2a7e5cb71b7996e27e80	0	0	0	0	0	0	43	0	0	0	0	0
30dbcc82866ab4525851d600bc800e1a	0	0	0	0	0	0	0	4	0	0	0	0
14151ca1c23599655721c383e29159ab	0	0	0	0	0	0	0	0	0	0	3	0
bb42bdea069b6c45c5ad4debdbe7ad77	0	0	0	0	0	0	0	0	0	0	0	12
4062f8fc47a025eaec4f35fc93e47c55	0	0	0	0	0	0	0	0	0	0	2	0
eba75a3d7a691cebeeb9868314150489	0	0	0	0	0	0	0	0	0	0	0	3
d90e21195b091145d65bb10205a32e14	0	0	0	0	0	0	0	0	0	0	5	0
10e36da8faf9bf15d1c42102a5e5884c	0	0	0	0	0	0	0	126	0	0	0	0
519cfe055181aee00f18bf1bc16cc1ad	0	0	18	0	0	0	0	0	0	0	0	0
8ef57acb5c3cc899ddad00e0975be2a6	0	0	0	0	0	0	0	0	0	0	2	0
cc73895a3cbd0a0622a4139f7a0fd932	0	0	0	0	0	0	0	0	0	0	2	0
f7642fd0e4f4ae6fd484c7b3c9268903	0	0	4	0	0	0	0	0	0	0	0	0
22cfe07f1fc505272880e79dbd2e5955	0	0	0	0	0	0	0	0	0	0	0	3
6ece9f8b1658046b49dcbfe7fb49d96b	0	0	0	0	0	0	0	0	0	36	0	0

c124600c58055aa972bca7fff045c49a	0	0	0	0	0	0	4	0	0	0	0	0
08873115bebb2e8c7d584f0d86dac152	0	0	0	0	0	2	0	0	0	0	0	0
9c41ad451e498f5a6cbdae35a44df941	0	0	10	0	0	0	0	0	0	0	0	0
5356d570683d951ad80994e9f7357054	20	0	0	0	0	0	0	0	0	0	0	0
48d20b4aa2e3d736a0ac568101c6d49e	0	0	0	0	0	3	0	0	0	0	0	0
0f45fb9664c56b6ff105eb84543836a6	0	0	0	0	0	0	0	0	0	0	10	0
caedfc880e7bff00ca3bc19143b1a08e	0	0	0	0	0	0	0	11	0	0	0	0
07f3999d8fe9eeaa4e8dea0ed3f028cb	0	0	0	0	0	0	0	0	4	0	0	0
3aec2942da58c6e9fe69b33230c984e0	0	0	0	0	5	0	0	0	0	0	0	0
6780f029632c3b9aef7b7dbaaf9e7ed0	0	0	0	0	0	0	0	6	0	0	0	0
a48a3c83b013089c228474cabf4c1771	0	0	10	0	0	0	0	0	0	0	0	0
f5b13645c6220dcdb448ad7108002b8b	0	0	0	0	0	2	0	0	0	0	0	0
240d766f800c1e121e289608ea4165be	0	2	0	0	0	0	0	0	0	0	0	0
5b4687c3470766f44ef3cbd2ac8c4ed3	0	0	0	0	0	0	0	0	0	0	0	7

c7d2b981a14cf89d75c35d1f39648542	0	0	0	13	0	0	0	0	0	0	0	0
8e32e14208f87709b5695f2b18b7a134	0	0	0	0	4	0	0	0	0	0	0	0
1a2498864d028b71ed90e3a96c4bf1b1	0	0	0	0	0	0	0	0	0	0	2	0
7c424508f9d76204fde77d76e7101af0	0	0	0	0	0	0	0	0	0	0	0	2
a99de175d24f75989e6ea4203b00a8a6	0	0	4	0	0	0	0	0	0	0	0	0
5e552df8b940a6e4651c6c0a6454f168	0	0	0	0	0	0	0	0	0	0	0	19
6b4a6c2ade18909b05116e1b6ae41bd6	0	0	0	0	0	0	0	0	58	0	0	0
715ff3dab773599166a4ac33b3b074bc	0	0	0	0	0	0	2	0	0	0	0	0
1c2b592f47327c467b914121daeb3ce2	0	0	3	0	0	0	0	0	0	0	0	0
4ac7dad45fccde1e5332ba8a9d312bca	0	0	0	7	0	0	0	0	0	0	0	0
c61a82dc6349427026205add4595d1df	0	3	0	0	0	0	0	0	0	0	0	0
8085d08405e78927992acbfb35d52165	0	0	0	0	0	0	0	9	0	0	0	0
69e477a4abc24106e32809044cb8ed3f	0	0	0	0	0	0	0	0	0	0	0	8
0e4d49d49604e3b837b8316815892986	0	0	0	0	0	0	0	0	0	0	0	7

388b92a806965ed66454583e66237d1e	0	0	0	0	0	0	34	0	0	0	0	0
8a4611c22ddf6b8c1aa37f4ec9b42c97	0	0	0	0	0	0	0	6	0	0	0	0
b9f0e77e5d1d246a2c5a1776efa1ae7e	0	48	0	0	0	0	0	0	0	0	0	0
795e4c67c364d291188eb29086320a68	0	0	0	0	11	0	0	0	0	0	0	0
c099f8c7ceb40c773ee3816417c74a1b	0	0	0	0	0	7	0	0	0	0	0	0
362241e322220019895f70b6262f979e	0	0	0	0	0	0	0	0	13	0	0	0
2ce8d3138758ce6d5ec4b304c4df2aed	0	0	0	0	0	0	0	0	0	3	0	0
c11cce47de5aaf31e38eb0920a6050c6	0	0	0	3	0	0	0	0	0	0	0	0
3e41182c20587fb6212d4fc7585f423a	0	0	0	0	0	2	0	0	0	0	0	0
62eaac07573af33aaf8f8d746ff4ba4d	0	0	0	0	0	0	9	0	0	0	0	0
ff3dbe26c41855ec71a556571b3ce222	0	0	0	0	0	3	0	0	0	0	0	0
aaea8a607759586b97d77b06ffc88a59	0	7	0	0	0	0	0	0	0	0	0	0
35a26c1714c680069a3cd3423787b52e	0	0	0	0	0	0	0	0	0	0	5	0
fc6bc085417a3894a0696207910214aa	0	0	0	0	0	0	0	0	3	0	0	0

589c9fa892f5841c12605d134f23934a	0	0	0	0	0	0	0	0	0	0	0	2
f34a7810bb1308308e4f335014241aec	0	0	0	0	0	0	0	3	0	0	0	0
159855b0e9e809c5b485040d99e2343b	0	0	0	0	0	0	0	0	0	0	4	0
de33695f24a3d667379c49a3a880f7fc	0	0	0	0	0	7	0	0	0	0	0	0
9b3e4d7bfd8cd8ba2bca9b9014032c0e	0	0	0	0	0	0	0	0	3	0	0	0
7e3b6a395b67703c0fc810367fa75349	0	0	0	0	0	0	0	0	9	0	0	0
9847179dabaf2c6e90522c815fd7577b	0	0	0	0	0	0	0	0	0	0	0	3
0796caa8cc2ab102875c6af40fc862dc	33	0	0	0	0	0	0	0	0	0	0	0
7ec43ea67075db4330041bc27f157a9e	0	0	0	0	0	0	9	0	0	0	0	0
0381466c636a3547c4a2a9932f7b7aa0	36	0	0	0	0	0	0	0	0	0	0	0
68d9f729272b7154192f23879b15c637	0	0	0	0	0	0	0	0	0	3	0	0
56674748fe741b16fddc340324611099	0	0	0	0	0	0	0	0	2	0	0	0
bc8193242d827086f5825ed29b4c7e5d	2	0	0	0	0	0	0	0	0	0	0	0
5d779ddca7e9eb0edf87d93545e87843	0	0	0	0	0	0	2	0	0	0	0	0

36b453f6e80087dec8203b2d983862df	0	0	0	0	0	0	0	0	14	0	0	0
3cebf954f1a4b91b418e94f1d2d6e936	0	0	0	0	0	0	0	3	0	0	0	0
7b6d38408e38f5ac04a10c2241a38e8e	0	0	0	0	0	0	0	8	0	0	0	0
9d6c1dc564700ec69feeadaff82e09a3	0	0	0	0	0	0	0	0	35	0	0	0
0a92e979abf78894940ae6b0f9a9102a	0	0	3	0	0	0	0	0	0	0	0	0
75b671e03d1367afa5a7529e7306ef02	4	0	0	0	0	0	0	0	0	0	0	0
01160473a403dea702402955e26fbeb3	0	0	2	0	0	0	0	0	0	0	0	0
dea76156521c0ba836873644f321b266	0	0	0	3	0	0	0	0	0	0	0	0
e505d546bc56c849b136c1e72724d963	0	0	0	0	0	0	0	6	0	0	0	0
faf2f238cd6b8d7caaa08cce28df8da0	0	0	0	0	0	3	0	0	0	0	0	0
884cabcbebb70fff66d7c804e99d9d57	0	0	0	0	0	0	4	0	0	0	0	0
33c0caad86fe64dd6359c6601cd217ba	0	0	0	14	0	0	0	0	0	0	0	0
5ddd692b4155f322e30697160a03a25f	0	0	0	0	0	0	0	0	0	0	0	5
470840499c9ef571a2b12a8345d0d3f0	0	4	0	0	0	0	0	0	0	0	0	0

3bac8afae22c8f42c409ed51ab7ca71a	0	0	0	0	0	0	0	4	0	0	0	0
a88d7d9b7704a7494b945ae51279e2e7	0	0	0	0	0	0	0	0	0	0	0	28
59e13aed4bd4da73e75f27be71324d5e	0	0	0	0	19	0	0	0	0	0	0	0
3d1b97dcf7871b160ae5fab54ccad881	0	8	0	0	0	0	0	0	0	0	0	0
5398e8ec966e5e0cc592ecd2da5a4faa	0	0	0	20	0	0	0	0	0	0	0	0
a5dc4ab6f5cd7dab1e2aea8c0bd72550	0	0	0	0	0	0	0	0	0	7	0	0
d15fdab8eaa66de63b9f31672a037f4f	0	6	0	0	0	0	0	0	0	0	0	0
61871691a9db4c37332e23493c7fcb48	0	0	2	0	0	0	0	0	0	0	0	0
3bbd719112004093dd961ef1094b425a	0	0	0	0	0	0	0	0	2	0	0	0
9c96b3caffee8c2854b8596cc72a9f7c	0	0	0	0	0	0	0	5	0	0	0	0
eb067f5c4627e101f85ec28645225f91	0	0	0	0	0	0	0	0	0	3	0	0
9062d69f220d96d4216a5b5fd8e4018b	0	0	0	0	0	0	0	0	0	5	0	0
f2b5c48a20a9205eb167340e103acf81	0	0	0	0	0	343	54	0	0	0	0	0
111ac600b1ec127472abbb3119450683	0	0	0	0	0	0	0	0	0	0	0	4

1b9351bee54cd91d803d4d939044c072	0	0	0	0	0	0	0	0	0	12	0	0
03d565b3d4cb5390a978b9adb2b71bbe	0	0	3	0	0	0	0	0	0	0	0	0
888e0372694708cb124294e1e1824399	0	8	0	0	0	0	0	0	0	0	0	0
429a4284fdfbfd3d50722d90464dccd5	0	0	0	0	0	0	0	0	0	0	2	0
cd8d1e0af8a80b1d1b97943d4608b0d6	0	0	0	0	0	0	167	0	0	0	688	209
a613e2eaa05e082c2e28a741351001ae	0	0	0	0	0	0	4	0	0	0	0	0
759beede7e1cef4dda0ec882c54eb924	0	0	0	0	0	0	0	0	0	0	2	0
90cf16ad00b077a59b96d33742a8c363	0	0	0	0	0	6	0	0	0	0	0	0
a6a189750a07a41e0a93c9756afbbddd	0	0	0	0	0	44	0	0	0	0	0	0
6b32be6ba257173073054cfedd3c28a9	0	0	0	7	0	0	0	0	0	0	0	0
07b7a6ac1354361b52eb8a9337d29d88	0	18	0	0	0	0	0	0	0	0	0	0
1558fb33b5fa75499d249ecbb43b5955	0	0	0	0	0	0	0	0	0	0	0	17
b028d41230074daaf16ad74cae681e12	0	0	0	0	0	6	0	0	0	0	0	0
7c83040f8426941b105034b9e46a1069	0	0	0	0	0	0	0	0	0	0	7	0

54e8801da91ebb9cad69df2ef5786878	0	0	0	0	0	0	0	3	0	0	0	0
8fad6f3d90f349c4c9f33bc7d4413222	0	0	0	67	0	0	0	50	13	0	0	0
3cca23c6e2cd5b75fec82fb85ae5a271	0	0	13	0	0	0	0	0	0	0	0	0
4e6ae57f8b58127829cc24f31fc2d7f8	52	0	0	0	0	0	0	0	0	0	0	0
3d7b97648ab1ed7c5d95efe9eaa56bc8	0	0	0	0	4	0	0	0	0	0	0	0
bc5ad7d3748bcdd3fff021ce0b0f9447	0	112	0	0	0	0	124	0	0	0	0	0
24d035e6597323096bf06c6eb50eac98	0	0	0	0	0	0	0	0	0	0	8	0
b4667225086c1459e6ebfc51165b7cc1	0	0	2	0	0	0	0	0	0	0	0	0
5a65867825108de7b245beef1a2d94f9	0	0	0	0	0	3	0	0	0	0	0	0
4a17febd77ea73d3bebe03dc3aba1fd1	0	0	2	0	0	0	0	0	0	0	0	0
b5029aaa4dc102ee587dc9549c8ad59c	0	0	0	0	0	0	0	0	0	0	0	6
7ff2117bbab4ba5c944f9ce72d48faeb	0	0	0	0	0	0	0	0	10	0	0	0
d71d6c48bab6f450e4b8fba869c32c58	7	11	0	0	0	0	0	0	0	0	0	0
cb686130d8f2c7b39754f3a8963e949d	0	0	0	0	0	0	0	0	0	0	0	37

b97f0f9709492f7ce7d9df164dd19df1	0	0	0	0	0	0	0	0	0	19	0	0
c74069d6e4c6abca2aca26e07dd3f7c5	0	0	0	0	0	0	0	0	3	0	0	0
7236c328dbe4bb5c546819af18624fe9	0	0	0	0	0	0	0	0	18	0	0	0
dd4ab7bbb668a3b9b4e2e67751637dc3	0	0	0	0	0	9	0	0	0	0	0	0
98115b835d74453b281e9858224d1159	0	0	0	0	0	0	0	0	0	0	2	0
28c17b9638e780e436f24766bd37c7ea	0	0	0	0	0	0	0	0	0	0	5	0
ad5b7b2474d131d501cb13b4e066798c	0	0	0	0	0	0	0	0	0	2	0	0
9641a5b12a5366f4b39212ee4165914a	0	0	0	0	0	0	0	0	0	0	17	0
036d8055cc0ccfd4443bc57d1debd182	0	0	0	0	0	0	0	0	0	3	0	0
bcc67e231bda71e177957abf5894fb57	0	0	0	0	6	0	0	0	0	0	0	0
6374d61a306ebd5e4bb88a21c5d6bcf8	0	0	0	0	2	0	0	0	0	0	0	0
c6d9610156ee07c4f084f1876a040386	0	0	0	0	0	0	2	0	0	0	0	0
d33e9d9a400df464b9895916ebce18fb	0	0	0	0	0	0	0	0	0	0	0	3
b8faaa66fccdc313a08135d26ce4cdd3	27	0	0	0	0	0	0	0	0	0	0	0

279289e34e30137e68ffddb866a46a74	0	0	0	0	0	0	0	0	2	0	0	0
f46e9ae686cd681de16a8f90d675cb4f	0	4	0	0	0	0	0	0	0	0	0	0
1e14d302cd09b5aa1d0322f7a6b2ec05	0	0	0	0	0	0	5	0	0	0	0	0
c8259a10f843adeccf8273aa20d29a31	0	0	66	0	0	0	0	0	0	0	0	0
a727a6d9446775f52c3577d39d6e727c	0	0	0	0	0	21	0	0	0	0	0	0
c975964cff563f7e82464f3c1a26fc7f	0	0	0	0	0	0	0	0	0	0	0	7
411db75b1e799e0f8aed75bf89c881cf	2	0	0	0	0	0	0	0	0	0	0	0
2c690bbaaaa78a8308a43315d6c2cfd5	0	11	0	0	0	0	0	0	0	0	0	0
2321a390f434ebb45f8b8821848e677e	0	0	0	0	0	0	0	3	0	0	0	0
f7510f6cb42390911ddb89fdb95fe8a5	0	0	0	0	0	0	0	19	0	0	0	0
4aa34975e8fc01f1aafa0d5ae3fb05da	0	0	0	4	0	0	0	0	0	0	0	0
fe41954d4a791f2b2cb00d3e92e7841d	0	0	0	0	0	243	126	0	150	0	0	0
54a716ac300e1f941edf4268b7d057fc	0	0	0	0	0	0	0	0	0	0	3	0
69e7194fd457e6fce058023ec4b5f138	0	0	0	0	0	0	0	0	0	0	3	0

6a79dc8bd1ffda0f15bb41995ab18da9	0	0	0	20	0	0	0	0	0	0	0	0
704a30552b9dd7566984cd55b447c826	3	0	0	0	0	0	0	0	0	0	0	0
ebef36c9f4588d5f06a173eed1f2fa71	0	0	0	0	0	0	0	0	5	0	0	0
a95ad9ea8395bb56205987034bee83a9	0	0	0	0	0	0	0	19	0	0	0	0
d2bd1835e503a583fc0535002b6e3904	0	0	6	0	0	0	0	0	0	0	0	0
7fda70b81fa93a276a1f89b277afa81a	0	0	0	0	0	0	0	0	7	0	0	0
b2740c2fec785e4873254267164d640e	0	0	0	0	29	0	0	0	0	0	0	0
68dbdf8b9a986d86ae496c9b994893c6	0	0	0	0	0	0	2	0	0	0	0	0
41538d49bba4b0e5964f9a3e96ad3de0	0	0	0	0	0	0	0	0	0	0	0	2
a55893164a9f61e143ad92b6427c3f5f	0	61	0	0	0	0	0	0	0	0	0	0
5389cb87769ba51299877f2485510af3	0	0	0	0	0	3	0	0	0	0	0	0
4383dd7f3d52fc6bec2eee14aa7a37cd	0	0	0	0	0	0	0	0	0	0	4	0
2ad1162b435c91b58a0c74049e1bcc97	0	0	0	0	0	0	2	0	0	0	0	0
5c984ead6ca8a177a873572974c4a388	0	0	0	0	0	0	0	8	0	0	0	0

5f1a2b1bac13654821813475 4328a3df	0	0	0	0	0	0	0	0	0	7	0	0
281c43a3ba4e6ba80d0ed33 3bf0854b2	0	0	0	0	29	0	0	0	0	0	0	0
40151c13f9fbc7e4b2e4ae48 b0e6e603	0	5	0	0	0	0	0	0	0	0	0	0
73069051a50b703de919b55 bd0a05a2e	0	0	2	0	0	0	0	0	0	0	0	0
d4220ff35e7c1dc9a9faef739 2323d14	0	0	0	0	0	0	0	0	336	176	0	0
adfd62706c4ebd74a1b4889d f82b737a	13	0	0	0	0	0	0	0	0	0	0	0
baffda69c20410fa1e411b520 dd7a5e6	0	0	0	0	0	0	0	0	0	0	0	7
656e8bc3b313f639d945254c 59f77d33	0	0	0	0	27	0	0	0	0	0	0	0
a3deab410b1860ca4112172 0a4dac35c	0	0	0	4	0	0	0	0	0	0	0	0
f5344eaa7f15be5099864790 778e067b	0	0	0	0	0	0	0	0	0	3	0	0
fd71d2393af94c617e034775 2d994b58	0	0	0	0	0	0	0	0	0	0	0	2
60670d9fa05bcb2c0100f142 a302873e	0	0	0	0	0	0	0	0	0	0	4	0
923c4e68f72d820cfb3b12a7 ce7ff189	0	0	0	0	0	0	0	0	0	0	0	47
df23a85a8ffbb755a92fba826 5cce881	126	0	0	0	0	0	0	0	0	0	0	0

0903370903272999da7cd9cc 1ed5c3b4	0	0	0	0	0	0	0	9	0	0	0	0
7add00ed2168c5cee38a2052 1ffb764f	0	0	0	0	0	0	0	31	0	0	0	0
2f769e170d3e023c41d89bb6 3e14538d	0	0	0	0	0	0	0	12	0	0	0	0
20b85c87478a3fc20bba5e27 fc8a2cb9	0	0	0	3	0	0	0	0	0	0	0	0
30669de21e0d60785316e2c 1d04cfcc5	0	0	0	3	0	0	0	0	0	0	0	0
ab9d07ea7703912a8921162 3d5681ff5	0	0	0	0	0	0	0	0	0	0	2	0
920a66df60a4f077807f23b2 697dd30c	0	0	0	0	0	4	0	0	0	0	0	0
23ec81a183719c44194eb127 3fce8e89	0	0	0	0	0	7	0	0	0	0	0	0
53ce3a8cd7cefc556d9a1b74 d8b1599e	0	0	0	0	0	0	0	5	0	0	0	0
6dd3f56d0d31c8caeb2a0d3e b3be1f0e	0	0	0	0	0	0	0	0	0	2	0	0
b8633682d8b68edb05e94ab 1a7c6eeee	0	0	0	0	27	0	0	0	0	0	0	0
9671c95cf64428d5162c860c 4ab2d76f	0	2	0	0	0	0	0	0	0	0	0	0
edc251fad07e32175574a52a ead160be	0	0	0	0	0	0	0	3	0	0	0	0
5957e96496c254064696c49f 6f07c531	2	0	0	0	0	0	0	0	0	0	0	0

1b82fcc0872ce6bbe44a264a61115859	0	0	5	0	0	0	0	0	0	0	0	0
e31f6848ee4d7db0b7647265a6aefe92	0	0	0	0	0	0	0	0	0	0	6	0
10f19fac6232bc71444f1df2c6332fb7	0	0	0	0	0	244	0	0	0	222	240	198
3b44767a31346cd55e41759d65ba0236	3	0	0	0	0	0	0	0	0	0	0	0
77cf8412ef607520e6320fb7a948b4f1	0	0	3	0	0	0	0	0	0	0	0	0
fb599eb302fb73ca27af44ff0a92282e	0	0	0	4	0	0	0	0	0	0	0	0
b0fe7ebe286e5a0cbfeb78b6cfae252c	0	0	0	0	0	6	0	0	0	0	0	0
92fd668ce21f8444120dab66dfcaf1c9	0	0	0	4	0	0	0	0	0	0	0	0
ab4eab67ea84a1c166498c97686287e5	0	0	0	0	0	0	0	0	0	2	0	0
8e59f7556608201688bb98c370ca071d	2	0	0	0	0	0	0	0	0	0	0	0
f95c26a33f640d34ba3b706655b51c58	0	0	0	0	0	4	0	0	0	0	0	0
eac81d6b1528b2dcea65c38a44a5e14c	0	0	0	86	0	0	0	0	0	0	0	0
39896fcdaa812ee2915766cf2e9dd5d1	0	0	0	0	0	0	0	0	0	0	5	0
576011e29f7add42f18d8070355f6403	0	67	0	0	0	0	0	0	0	0	0	0

37e7d9d3da3d85450f75384 67d5a7dd4	0	19	0	0	0	0	0	0	0	0	0	0
22ff361d02f8d20a49ecf8259 bc0494f	0	0	0	0	0	0	0	0	0	0	0	3
550d33cdefc60d696594a4bf ee50f4b5	0	0	0	0	0	0	3	0	0	0	0	0
e8bdfd986ec7382337c38638 f11926a6	0	0	0	0	0	0	0	0	2	0	0	0
33c13b17885d9dc304745c79 b59b87b2	0	0	0	0	0	0	0	3	0	0	0	0
b8063bd3177789dc37aeb96 c3890b1a5	0	0	0	0	0	0	0	0	0	0	2	0
d81edda8c5b881e8a15fb1ac 39c013e3	0	3	0	0	0	0	0	0	0	0	0	0
b6b52e80632d1bf288bfdd01 c79faff1	0	0	0	0	0	42	0	0	0	0	0	0
0b133e482c8520d86d2ac87 3ce5b57c3	0	0	0	0	0	0	0	4	0	0	0	0
ae3c38db847697c5421cfba8 6bc7f088	0	0	0	0	0	0	0	0	4	0	0	0
169adfd781c261d9dfd67b3e b6902374	0	5	0	0	0	0	0	0	0	0	0	0
a7cd7c8163c6f5245541e544 97633306	0	0	0	0	0	0	0	0	0	0	0	2
7b6002dd93e25403a450141 e7756154c	0	0	26	0	0	0	0	0	0	0	0	0
99caf118c52dd006e99e1a79 cc23eba6	0	0	0	0	0	0	0	0	3	0	0	0

a0706f4ba92fea7939b5f3550 2e39409	0	0	0	0	0	0	0	0	0	0	0	3
5628e78fb703ba6bce762ef5 31213bf2	0	0	0	0	0	0	0	0	0	2	0	0
b76fbf5151d93331be4bad92 aae6238a	0	0	0	0	0	0	0	0	36	0	0	0
2105dba7c7292be8ccfe49e7 21162eb1	0	0	0	0	0	0	52	59	0	0	0	0
cfc67b5852c21bf734c1e1885 7a6eaec	0	0	0	0	0	0	0	0	0	0	4	0
5ce8c9232c5ec2d2276a1fc0 15be1176	0	0	0	0	0	0	0	0	0	13	0	0
64bbcc1015e41ff7eaa8ca042 1123a13	0	0	0	0	0	0	0	0	0	3	0	0
1f20dd9d19cc1bf295d71ae1 7514a02f	0	0	0	0	0	0	0	0	0	26	0	0
eb21feeae5bb8665f1bd6974 77d27a3b	0	0	0	0	0	0	0	0	0	0	2	0
d047072bb3c235ab106d118 bd3cf911a	0	0	0	0	0	0	0	8	0	0	0	0
4f9b311b2a898fafc1cf9cddfb 243bc4	0	0	0	3	0	0	0	0	0	0	0	0
a030391f9f2a505d2a569ded 5096f685	0	0	0	0	0	0	0	30	0	0	0	0
7175e16698f9b394b93e0c23 3ebca13d	0	0	0	0	0	0	4	0	0	0	0	0
112cb75499a23dfe3230c5bb 5c2ccae5	0	0	0	0	0	0	0	4	0	0	0	0

69d422d05a2a131b0123361af1971e77	0	0	0	0	0	14	0	0	0	0	0	0
175a01cb95343504cfa98a8fffe840df	2	0	0	0	0	0	0	0	0	0	0	0
e3db6af386c1ee80c0b2bffd46960cec	0	0	0	0	0	0	0	10	0	0	0	0
40909d9d9ea91927cde658551f249c12	0	0	0	0	0	0	0	3	0	0	0	0
c16e89aa351d06bc04b154fcdcd3602e	0	0	0	0	0	0	0	0	0	0	32	0
15a41b3c5054b3d542e9dda649782d83	0	0	0	0	0	0	0	0	0	3	0	0
0312cfdb010151e47cfb23f6aaf7a7bc	0	0	0	0	0	0	0	0	0	11	0	0
2fea3e9375667537849f71d67cf19bfa	0	0	0	0	0	0	0	0	18	0	0	0
ec558f29719298a8247d6cfc8d41af38	0	0	0	0	0	0	0	0	0	3	0	0
be331ba1f54d3705e102610b0a0b3118	0	0	0	4	0	0	0	0	0	0	0	0
b392db298b2a5c47109553e1ed82b3b3	0	0	0	0	0	3	0	0	0	0	0	0
9c42ba86fb8b058c1439b76409aec2e1	0	0	0	0	0	2	0	0	0	0	0	0
6618da81a3cca131e5b36a4a22521a02	0	0	0	0	0	16	0	0	0	0	0	0
1000d4fc07c0126214dd635e55a1dc19	0	0	0	0	11	0	0	0	0	0	0	0

14e7d01b46408b9682169f37b7db0e01	0	0	0	0	0	0	0	0	31	0	0	0
0cbfc39a2a13a4e72d2f81f7f22da327	0	7	0	0	0	0	0	0	0	0	0	0
9f491a768856fb35db49fabbbc5d18b7	0	0	0	0	0	0	0	0	0	0	8	0
86e6c89463625eab1095eae54f1472b7	0	0	0	0	18	0	0	0	0	0	0	0
242ce3052cd37db3675c6931649612ed	0	0	0	0	0	0	0	6	0	0	0	0
3032257d866995eb0c7c33d4b8d75f1c	0	0	0	0	0	0	0	0	0	0	0	6
a10cfe5e5869760ab68ad5efc7e309b9	0	0	0	0	0	0	0	0	0	0	0	2
c4d01518b2f23fa46c2146787d3ff559	0	0	0	0	0	0	0	0	0	0	2	0
a4a06856ebf4aaa1cce663136d8fe8c4	0	0	0	0	0	0	0	4	0	0	0	0
fe563ebe057485cc9e765276472dc08c	0	0	0	0	0	0	0	0	6	0	0	0
08c2d2c536f8c29b8b4ee02d4062e8a9	0	0	0	0	2	0	0	0	0	0	0	0
ac205441df9f4b258de7bc746bc31ac9	0	0	0	7	0	0	0	0	0	0	0	0
bd6a085cba789f2432927ac84e84ee4e	0	0	0	0	2	0	0	0	0	0	0	0
219b59eb5d148ff82dd746d1abd32e3b	0	0	0	0	0	0	0	0	0	0	3	0

057161024184ebe7d39d4b98cf0331d0	0	0	0	0	0	0	0	9	0	0	0	0
d1943a63c3c943aefc49582db8631afd	0	0	0	0	0	0	0	2	0	0	0	0
841e2dd18464dbb85f71bb015b98cf1b	0	0	0	0	0	0	0	0	0	4	0	0
149ccdcf78ce2d7b97d4ea31af1f7047	0	0	0	113	0	0	0	0	0	0	0	0
c734256616e8382dbb2de9ed2af952b8	0	0	0	21	0	0	0	0	0	52	0	0
9f46d98620a5dc8c9f467c00bff2a1e0	0	0	0	0	0	9	0	0	0	0	0	0
9a289e7572039913e2d68fbe4103d732	0	0	0	0	0	0	0	0	0	3	0	0
3b48250c654de90f7c9f943263daf755	0	0	8	0	0	0	0	0	0	0	0	0
2158995c21185a02dfd43d0112b017a6	0	0	0	10	0	0	0	0	0	0	0	0
ba61e120b5b843085a839939d10d24bc	0	0	0	163	0	0	0	0	0	0	0	0
160f8c5571020335e86f44cec6f6a852	0	0	0	0	0	0	0	2	0	0	0	0
6d1290b0c8b9b861c3efb9c9c2b66ea9	0	0	0	0	0	0	0	0	0	0	0	6
c64d033a1f07fc12374c12d241437d06	6	0	0	0	0	0	0	0	0	0	0	0
51a365ed54569ddb2d7a142095098f3f	0	0	0	0	0	0	0	0	0	3	0	0

9de313d6f1d461bca67756b45b5bc461	0	0	0	0	0	0	0	0	11	0	0	0
3a3134fd00c8e1830f212d26d9ba4af1	0	0	0	0	0	0	0	0	0	0	3	0
8bd1ed93da42a9987b2b4b3c8828e69e	0	0	0	0	0	0	0	0	0	0	8	0
42eb4a934d6a676b94dbcacc3d6ce589	0	0	0	0	0	0	0	2	0	0	0	0
2cf83c4bc79d3303cbe0e713ac01aaba	0	0	0	0	0	0	0	0	10	0	0	0
47e192b2d8890dcc1bd3f65bc65a7409	0	2	0	0	0	0	0	0	0	0	0	0
aa5fddd8e2c7a623cd223db55ba98bfc	0	0	0	0	0	0	0	0	0	0	0	3
4461fcdd3afbc15c82b80782c717336e	0	0	0	0	0	8	0	0	0	0	0	0
4610d97ba1c0ce595cf86cc623c1a390	0	0	0	0	0	0	0	5	0	0	0	0
74cc404bdcaae2e2061cdbf105657536	0	0	0	0	0	0	0	0	0	0	0	6
2657b56525972b96e92a1b10b8ecb726	0	0	2	0	0	0	0	0	0	0	0	0
b12eba468435a28740d06d4d5884d8d8	0	0	0	0	0	0	0	23	0	0	0	0
6988cafc7f0dff93108a1d40aff2925b	0	0	0	0	0	0	0	0	0	0	0	3
1c413ed3136e5e05bdf135b60436b7fe	0	0	0	2	0	0	0	0	0	0	0	0

211d1dd02ed82604e4d1093 3f2a394e7	0	0	0	0	0	0	0	9	0	0	0	0
c3f85bb9e385b891bfc4ba8b 4adaa485	0	0	0	0	0	5	0	0	0	0	0	0
85ed95b391682172509873a 75e2342fc	0	0	0	0	0	0	0	0	0	0	12	0
f479523d2cf25ce4ed765429 6101c65e	0	0	0	0	0	0	0	0	0	0	9	0
b995bb6b56c178e7ccfb32ad 0cbba1b1	0	0	0	0	0	0	0	0	2	0	0	0
6f85468334a53a9b7f380b79 e5011f93	0	0	18	0	0	0	0	0	0	0	0	0
4b38553b482a29581806f86f 432ccd5c	0	0	0	0	0	6	0	0	0	0	0	0
672f91824b6cf560e79366d8 e49f786a	0	0	0	0	0	24	0	0	0	0	0	0
0bf0b03ae0fa8fbdc872cf3f38 e5faf6	0	0	0	0	0	0	2	0	0	0	0	0
d3c6fda734a785e2549311b6 49a17854	0	0	0	0	0	0	0	0	0	14	0	0
0faf11bde3a780c5d6789d4c 4640e97e	0	0	0	0	0	0	0	11	0	0	0	0
14535024bae3479eaa01f669 86bc5eec	0	0	0	0	0	0	0	0	0	0	37	0
b0a4f5f4137a11ce54ce27b2f b800b31	4	0	0	0	0	0	0	0	0	0	0	0
1ad0a3439a2039b242eab00 942c2b748	0	0	0	307	0	0	0	0	0	0	0	0

3d9ecfa3c1f0c39ddfb52daa26f0c4b8	0	0	2	0	0	0	0	0	0	0	0	0
91d2d0f89cbbfc418449214c5786ed99	0	0	0	0	0	0	0	0	0	0	0	5
9849edeb09e8e05ccf34e0d854c7d81a	0	0	0	0	0	0	0	0	2	0	0	0
2f663815e1350859e217323e85b3ca15	0	0	0	0	0	2	0	0	0	0	0	0
20db9d20311ff167b771a3d963bdecb9	0	0	0	0	0	0	0	0	0	0	0	3
e6112ee7869137566937491b7d076943	0	0	0	0	0	0	0	0	0	0	5	0
e6f99f86e0d36ae2f94f5401124cf092	0	0	0	0	0	0	0	0	0	3	0	0
0540e67168bd1234fd991584648157e4	0	0	0	0	0	7	0	0	0	0	0	0
8221da640ef2fbeb10a720e5b2212505	0	0	0	2	0	0	0	0	0	0	0	0
e2626b5aac6b82ad2c568585c9a3c47a	0	0	0	0	0	0	0	0	0	0	6	0
a7fd41ad83a007158d4354220c4143a0	0	0	9	0	0	0	0	0	0	0	0	0
6fea4ba3b8848d3f508b0fccd0edf90a	7	0	0	0	0	0	0	0	0	0	0	0
e7fcae7138b6c4691d31c00ad59b3442	0	0	0	0	0	0	0	0	0	0	13	0
a7865bc5980f40440c5bb5d95a2e662e	0	0	0	0	0	0	0	3	0	0	0	0

87e08e0d8b24538b7fb533b7a82a5d1d	0	0	0	0	0	0	0	0	0	2	0	0
4b006ca95ba583124d573c24a88cfd88	0	0	0	0	0	0	0	0	0	0	0	3
4b367864cc27020562891e2cd735e372	0	0	0	18	0	0	0	0	0	0	0	0
2d5535d301ada10371270703c0d67e9e	0	0	0	0	0	10	0	0	0	0	0	0
3c14a1e0d3af125afc93a1ce702c2b6c	0	0	0	0	0	0	0	0	0	0	82	0
8fddb76b0c3075cb2eed69631bbbe162	0	0	0	0	0	0	0	0	0	0	4	0
23049654179a1b0761a65524867a2f90	0	0	0	0	0	0	0	0	0	0	2	0
33d2cdc9fbf39d02d9da4aded42d8c44	0	0	0	0	0	0	0	3	0	0	0	0
76311d59d62a04f4e5bf6273bd5f076a	0	0	0	0	0	0	0	0	0	3	0	0
e5546e632f8d7885c834743d42dd047b	0	0	0	0	0	0	9	0	0	0	0	0
b43e76e35256145e74ef1ea3cef17df4	0	0	0	26	0	0	0	0	0	0	0	0
7e7019fe435da60b2e86b0dad20470cd	0	0	0	0	0	8	0	0	0	0	0	0
2492cce2a06f00566fddebfce6d7118d	0	0	0	0	0	0	0	0	0	0	14	0
d361ee64b27a045b350efc3ef4638826	0	0	0	0	49	70	0	0	0	0	0	0

d64eddd75965545e9c4085bf208f3db1	0	2	0	0	0	0	0	0	0	0	0	0
2c813c7b985ec0d991a62f72d17b15af	0	0	0	0	0	0	0	0	0	0	0	7
b1f824006d81fd9acfda81e0fba98a80	0	0	0	0	2	0	0	0	0	0	0	0
4d9ffe530552bae7b44a584114134132	0	0	0	0	0	0	0	0	0	0	9	0
97c5c3c3a089329bb906964425ec103d	0	0	0	0	0	0	0	0	0	8	0	0
cb1eaa368b77c88e8996037f210e23f3	0	0	0	0	31	0	0	0	0	0	0	0
be4ec60342c30dad224f577cad01c9b0	0	0	0	0	0	0	0	15	0	0	0	0
7b7754c99d7a4b429111b178968661cd	0	0	0	0	0	3	0	0	0	0	0	0
9a8ede3979b430919e1b4243c7b6bf27	0	0	0	0	0	6	0	0	0	0	0	0
bab2a8d5c22037de6ac117cb876f992c	0	0	0	0	9	0	0	0	0	0	0	0
0991b0ecfdd94f01dad220f664b0caa9	0	0	0	0	0	0	0	0	0	0	0	52
5767820e7181a6de12cd75811cf99d82	0	0	2	0	0	0	0	0	0	0	0	0
d04c1c72f5d2ac17f06416ed29d9bfd2	0	0	0	0	22	0	0	0	0	0	0	0
ccaaf9a5d948e2f28ff898e1e934a321	0	0	0	0	0	0	2	0	0	0	0	0

21970f2d344e87636cce5a133082eaf4	0	0	0	0	0	0	2	0	0	0	0	0
545bc9cd89632118264e0b0d5ccb39c7	0	0	0	0	3	0	0	0	0	0	0	0
9d05f475427b81a01285a63675e5600f	0	0	0	0	0	0	0	0	0	13	0	0
a37755db9e2b6821ef30db7a9d530edb	0	3	0	0	0	0	0	0	0	0	0	0
1099b596a7c836d42c18d09bdeb63b0d	0	0	0	0	0	0	0	0	0	0	0	3
882714698c3bb86605546d10a9181c58	2	0	0	0	0	0	0	0	0	0	0	0
84c90d523a408dfb1e8aeced24c01d62	0	0	7	0	0	0	0	0	0	0	0	0
ca87da85236d9585524c8d1f336e44e4	0	0	0	0	0	0	0	0	0	0	0	38
f9e8d5c7b71ee3acbc8200ee1207af22	0	0	0	0	0	0	0	0	0	0	0	9
5b5b3fd20fbffa57538f9288827014bd	0	0	0	0	0	0	0	0	0	3	0	0
216aef2ac31aa9952fad13b43c85e7aa	0	0	0	0	0	0	0	0	0	0	0	3
6dbc70141edacf715e784390afe9c4f6	0	12	0	0	0	0	0	0	0	0	0	0
d16864f8cd416f96a7e2f477b1a75ba7	0	0	0	0	0	0	4	0	0	0	0	0
6b561ef23f7f5217ed5c7ca5f1391829	2	0	0	0	0	0	0	0	0	0	0	0

0fd9e2109ac8884167f3d47ea84e0997	0	0	0	0	0	0	0	0	4	0	0	0
571a30c39ad8dec091e3008918ada37a	0	0	0	0	0	10	0	0	0	0	0	0
6e9f9a25ed5ff7bd3dd37c66d8ecee86	0	5	0	0	0	0	0	0	0	0	0	0
a1f710a97700af4c3054235d3364cf00	0	0	0	0	0	0	0	12	0	0	0	0
9415109e34a716684e259b2cbd7f6612	0	0	0	108	0	0	0	0	0	0	0	214
fd19ea36a523a567af306a40613ab293	0	0	4	0	0	0	0	0	0	0	0	0
5583738c0961f44a5c37be39f67a2ac6	0	0	0	0	0	19	0	0	0	0	0	0
9ed934297a8fec6489afbfcd9f23d8d5	0	0	14	0	0	0	0	0	0	0	0	0
d87d937ab92c57b815b267d9a9c1ec10	0	0	0	0	0	0	5	0	0	0	0	0
e1f6938ff0e414cd50a7f68f22ef3ac8	0	0	2	0	0	0	0	0	0	0	0	0
89ad4b005fefe4f1aac151faac504c4d	0	0	0	0	4	0	0	0	0	0	0	0
6a226680cb9babd478ec5cfe294606a8	0	0	0	0	0	0	0	0	0	3	0	0
bfeb439c6c31b14d9cd75538ecc85a86	0	0	0	0	0	0	0	4	0	0	0	0
d8bd854a53af1cf65100550b904956b1	0	0	0	0	0	0	0	0	3	0	0	0

249fc8ee8a8ed74235d11d93e6a48e3b	0	0	0	0	0	0	0	3	0	0	0	0
e53fb67fb7fc021f0e07bef26f9300ec	0	0	0	0	0	2	0	0	0	0	0	0
4ec594c05fe37a4f62bc0e36a78de8ef	0	0	6	0	0	0	0	17	0	0	0	0
b7b20a400fe60c840eb41aab43eb8d41	0	0	0	0	0	0	0	0	0	0	0	4
11beed6a4c70506c329a6a12f2d00c86	0	0	0	2	0	0	0	0	0	0	0	0
4efedcf2c0ad39a746a595c07bcf7bca	0	0	0	0	0	0	0	0	50	0	0	0
3a8e9296451a718094c50139c791f204	0	0	0	0	0	0	0	0	0	0	2	0
465e8b4838556ddc0cbf222e6c3c736c	0	0	0	0	0	0	0	0	0	6	0	0
2024062f30fea0bf018f6016ff397227	0	0	3	0	0	0	0	0	0	0	0	0
38c75bbd546b1ebee48a3d6c9d35eefb	0	0	0	0	0	0	0	0	0	145	0	0
e96f7807d926dce50b4105c43b8e6c0b	0	0	0	72	89	0	59	0	0	0	0	0
4927b3a3122e80e83290eed069e5756f	0	0	0	0	0	2	0	0	0	0	0	0
84a3ad2c6f295b1551d67c4c4d0ea1f5	0	0	0	0	0	0	0	0	0	3	0	0
5ca8ca596dfe719bf94d92e9f650b8b5	16	0	0	0	0	0	0	0	0	0	0	0

52ef7ae9acff7a0e3ac351cf6c74329e	0	0	0	0	0	0	5	0	0	0	0	0
59735e05f09aefedc581c55772704899	2	0	0	0	0	0	0	0	0	0	0	0
1db4df8938699446ecde44231efdb503	0	0	0	0	66	0	0	0	0	0	0	0
cd416e0a3fec37bd75cfad2cc8429442	0	0	0	0	0	41	0	0	0	0	0	0
e249fad8a863b047a08c0a803d20739a	0	0	0	0	0	0	0	0	0	0	0	67
72255115447caa62e4ca32f85b38eca0	0	0	0	19	0	0	0	0	0	0	0	0
a3012344188c5d612e2c0502882be16e	0	0	0	0	0	0	0	0	0	0	0	3
500356cd5383a386dcfc6d0c7df9d31e	0	0	0	0	0	0	0	3	0	0	0	0
3bc1d5a577d2949aee7c6c0a0062a226	0	0	0	0	0	0	0	0	0	8	0	0
d91d9d252c30a18aeb5777fb1098f955	0	0	0	0	0	0	0	0	0	21	0	0
6fc169fd3757b0761dc845ac36908694	0	0	2	0	0	0	0	0	0	0	0	0
fbc8111f1847c19cb4426b46824d2bdf	0	0	4	0	0	0	0	0	0	0	0	0
6a7ea7afa5251a679d25ec7c7ec391b8	0	0	0	0	0	0	0	3	0	0	0	0
40eb4c0da56f029d30aba602e6f46689	0	0	0	0	0	0	0	0	0	0	3	0

5d2df8c5f8959ff2a19a62c08 9606c75	0	0	0	0	0	0	0	0	0	0	2	0
ad0edde3a1d362cfbdec9b7d a97046a4	0	0	0	0	0	0	0	0	0	0	21	0
92e029d9ada30f4306ae0308 15b09a8d	0	0	0	88	0	0	0	0	0	0	0	0
a0d9d38d46aa2573f86cf5c8f 52d51b9	0	0	0	0	0	2	0	0	0	0	0	0
a209aed1de785210f7f4baa9 83f6944d	0	0	0	0	0	0	0	0	0	0	0	5
c272b91fc7c4ca164acf1f917 b2d0554	0	0	0	0	0	0	0	0	0	5	0	0
f3b891d82335603a923a053c 89d69686	0	0	0	0	0	0	0	0	0	0	4	0
5a47f5441c874af719185874 c6677f71	0	0	0	0	2	0	0	0	0	0	0	0
66f3011a0a9621009b3c429d a2f42674	14	0	0	0	0	0	0	0	0	0	0	0
bee2d450b1a11c88a42e5a5 82c222bed	0	0	4	0	0	0	0	0	0	0	0	0
c59c90843bf115b044cfcf618 3fec7cd	0	0	0	0	0	0	0	0	0	0	0	2
9dbbf3c1fd72d212aa84c47b 0371684b	0	0	0	0	9	0	0	0	0	0	0	0
e01753946d71e8fdf2d649d5 68cb6f7b	0	0	0	0	0	0	0	0	0	0	4	0
6c6ebbaeef37f1398e346693 b6c62967	83	0	0	0	0	0	0	0	0	0	0	0

0c52269a7e745b21cec70525686542e4	0	0	0	0	0	0	4	0	0	0	0	0
05598bdc234c38ee78bd16b492f5a641	0	0	0	0	0	0	0	2	0	0	0	0
d7672487205fa1732175eff8cc72c3f5	0	0	0	45	0	0	0	0	0	0	0	0
565ff085582ba85c68cda75b83ea9939	0	0	13	0	0	0	0	0	0	0	0	0
13d2204414710c965ac3929b607ace4b	0	0	0	0	0	2	0	0	0	0	0	0
5b3272021b3f62ae0b5c6cedfa20b450	0	0	0	9	0	0	0	0	0	0	0	0
b27dafd55161fcc35f4b056e16557e08	0	0	0	3	0	0	0	0	0	0	0	0
908247278dde0b61e15866758e9131d5	0	0	9	0	0	0	0	0	0	0	0	0
9a166098d6d3f29876d757d720358159	0	0	0	0	0	2	0	0	0	0	0	0
bc6d1937fabe1aad827981342f105f90	0	0	0	0	0	0	0	4	0	0	0	0
2e508801bd1a6464214b318efd68b6f6	0	0	0	0	0	16	0	0	0	0	0	0
268d8a326d1fd30730d770bf5921326a	0	0	0	0	0	0	0	0	49	0	0	0
6b459c6378bea46c5ae0efca96a62623	0	0	0	0	0	0	0	0	0	85	0	0
a3ad9c79a7e078f3446c1b20c3081125	0	0	0	4	0	0	0	0	0	0	0	0

67d3b023a0f7aaf2fb0c0830545bf208	0	0	0	0	0	11	0	0	0	0	0	0
196887274d65a39eaa2de059b2df6380	0	0	0	2	0	0	0	0	0	0	0	0
be6f37aadd2d99c7e1b260177f2189fe	0	0	2	0	0	0	0	0	0	0	0	0
93e249103892b9b8bd20b66e6bd96d48	0	0	2	0	0	0	0	0	0	0	0	0
0d34545479497ce9923d28e7a96c5d4a	0	0	0	0	0	0	0	0	0	0	4	0
c29bc24109494b5e4506e391822b728e	0	0	0	0	0	0	0	0	0	18	0	0
74c977f448dfc876908211ba673d7ded	0	0	2	0	0	0	0	0	0	0	0	0
75cc90698269343b684b018658df00d0	0	0	0	0	0	0	0	0	0	0	0	54
3a6fc8ffd016ca1288330222a96476b8	0	0	0	0	0	0	0	0	0	0	6	0
4315699652ddd07c0b87aa519fed1b44	0	0	0	144	0	0	0	0	0	0	0	0
e9fbe276a3fc7c247286e17921c69414	0	0	0	0	0	0	0	0	0	0	0	3
6870a8c185b5b94b7e884549fa5ca45b	0	0	0	0	0	0	0	3	0	0	0	0
de63390846ce4f8064b01a69622c5fde	0	0	80	0	0	0	0	0	0	0	0	0
b377c230db04005d3eeb98e4d181b9ca	0	0	0	0	0	0	0	0	0	0	3	0

46cfdb4989142f5979db4fbf8be59cb2	0	0	0	0	0	0	0	2	0	0	0	0
1fe70f08866a8029432218e18cf3b5ff	0	4	0	0	0	0	0	0	0	0	0	0
17f4f3cdc1927226edb0197504eb1cc6	0	0	0	0	0	19	0	0	0	0	0	0
3cdcdbc6c89440d0033968f2e060921c	0	0	5	0	0	0	0	0	0	0	0	0
08bd5f29eb292fa412e30ea198db426b	0	0	0	0	0	0	0	0	0	0	2	0
c643ab02ace3cf9c975dde62e172a86c	0	0	0	0	0	0	0	0	2	0	0	0
f2b48bf28529c9afaba94851dbfc0616	0	0	0	0	0	0	0	0	40	0	0	0
671600d179a9c1fcf3a2b6c06bcef126	0	0	0	0	0	0	0	0	0	0	5	0
deacf04775d2109754e38010b3a45da0	0	0	0	36	0	0	0	0	0	0	0	0
2143b4b244fa8ee507387b87581796ec	0	0	0	0	0	5	0	0	0	0	0	0
a348bcfab10bcc8b763ebd05db3f7a6e	0	0	0	0	0	0	0	16	0	0	0	0
971143f244cd130cbdda917155333989	0	0	0	0	0	20	0	0	0	0	0	0
bafc091db800fad4c70832781ad82b96	0	0	0	0	0	0	0	0	0	0	4	0
338bc06b2a583c82ad3930b3626eab3d	0	0	0	0	6	0	0	0	0	0	0	0

7cf9000b4bbdd00261f0e61fd5593e7e	0	0	0	0	0	0	0	0	0	0	0	2
91e99871ef300910fb5dfb5b460854fc	0	0	0	0	0	0	0	0	2	0	0	0
34c4826bc6f1ba159bab8f6d5476fe7e	0	0	0	0	0	0	0	0	0	0	7	0
779d7c0e780d778de9ff679cd1318b72	0	0	0	0	0	0	0	0	3	0	0	0
fc66d47dadca8e181214e72e52edcda7	0	0	0	0	0	0	0	0	0	0	0	4
d10cc48d58d6ff48f7fff29fe8a4429f	0	0	0	0	0	0	22	0	0	0	0	0
08419880491914e72dd280a5f7cd5fec	0	0	0	0	0	0	0	0	0	0	27	0
a5d7239d523b833e222fb1c7b402b79a	0	0	0	0	0	0	0	0	0	4	0	0
84eadf9a28fd49d4e1625ab5fda88220	0	0	0	0	0	0	0	0	0	0	2	0
1ecd7a98b6f85dbe8b14d9183f7ed3a1	0	0	0	0	0	0	0	0	0	0	0	2
168fe4a07ed349c32dfa8b7697d4d761	2	0	0	0	0	0	0	0	0	0	0	0
ce3fba71df8a64128db982905796b4eb	0	0	0	0	0	21	0	0	0	0	0	0
5abcfd412c2e44f5d5051645f4d7b2fc	0	0	0	0	0	2	0	0	0	0	0	0
030ba0625274a7a5292cee72ad174404	0	0	0	0	0	0	0	0	7	0	0	0

b49c366b6003c6f65ef0870fc c558f28	0	0	0	0	0	0	0	4	0	0	0	0
b39f4092112e4d4771e7d5ce 70f51b0e	0	0	0	0	0	0	0	0	0	4	0	0
9ec48615b10c661d7c6582e7 49e8aeb5	0	36	0	0	0	0	0	0	0	0	0	0
411c6a5c6e6621eaa2a27593 ddf9ab34	0	0	0	0	0	0	0	0	0	0	0	2
fe5bd99f01a69fb0ea5818d8 38819991	0	0	0	0	0	0	0	0	0	0	2	0
2a5d59a70bb26a10ddeef843 13b93f5b	0	0	0	0	0	0	0	0	9	0	0	0
b55eeb4b5affd5b983ef6a18 5024b3f4	0	0	0	9	0	0	0	0	0	0	0	0
0342ea3babc542e723f3f19f5 17aa7c5	0	0	0	0	0	12	0	0	0	0	0	0
e6a7a7d1f3496f6b675ae0cb ed88d5f9	0	0	0	0	0	0	0	0	5	0	0	0
d46ee94c5f270def89fcc43d7 1219714	7	0	0	0	0	0	0	0	0	0	0	0
dd9e4948731406698e60710 8f4bc7649	0	0	0	0	3	0	0	0	0	0	0	0
e2fd8cb4ff36f8aa91ae16dc5 1895c16	0	0	0	0	0	0	0	0	0	0	5	0
3f6275614b4c518b710dfeb4 6b963c2f	2	0	0	0	0	0	0	0	0	0	0	0
8ba6f9e743a6ff2d42797e3d 312a26d0	0	0	4	0	0	0	0	0	0	0	0	0

105a5a1b4303df29b8a476d812f17a33	0	0	0	0	0	0	0	18	0	0	0	0
741f8d16af58f9f2948fb5526686ed6d	0	0	0	0	9	0	0	0	0	0	0	0
a39991e5bc54a14e0547ed97b0746f9a	0	0	0	0	0	3	0	0	0	0	0	0
9b9b89fbe47584c999db41bc65d10c7b	0	0	0	0	0	0	0	15	0	0	0	0
b13b15e6b959b8e3ce6111bd8f8145ec	0	0	0	0	0	59	0	0	0	0	0	0
a44369cc31c02f24e1a49ed9ca016b2d	0	5	0	0	0	0	0	0	0	0	0	0
499957ca407da42eae2a6a708eefdf93	0	0	0	0	0	0	0	0	0	0	2	0
d91626f0bb5787d7e556e6e0876250f6	0	0	0	0	0	0	0	0	7	0	0	0
16885a924ec54e0150abe183bf0eaf15	0	0	0	0	0	0	0	0	6	0	0	0
b6257df66c2dc9de3837ee3f6c2100f1	0	0	0	0	0	0	0	0	0	0	2	0
830caa1da55b8063aab5d37fb1624f7b	0	0	0	0	0	0	0	0	0	2	0	0
70f0186eb2d4a4b3e5e8f72b5a9d1da2	12	0	0	0	0	0	0	0	0	0	0	0
9ee2e36561838c96dd952e58a0d9c45f	0	0	0	0	0	5	0	0	0	0	0	0
8d4618a414c1559a4aea26f2bd437697	0	0	0	0	0	0	0	20	0	0	0	0

2dd3766db8ef4d2a9c385d88494e564f	0	4	0	0	0	0	0	0	0	0	0	0
0c73488ff619ee9e93925c06ba2725a6	0	0	10	0	0	0	0	0	0	0	0	0
d0797b4723c2410ee29a055e2009de97	0	4	0	0	0	0	0	0	0	0	0	0
cac0b5611cacf6e78ba77425f8600c9b	0	0	0	0	0	0	0	2	0	0	0	0
0419709fcd0303f42ab5df1f83b7f6fe	0	0	0	0	0	0	0	0	0	0	0	15
3a29a2cbdac49534e85167571f76c303	0	0	0	0	0	0	0	7	0	0	0	0
933fd639c3a133b100790ad1f47c9093	0	0	0	0	0	0	0	0	0	0	6	0
d3a77e267197625d1ce897cab0664fd2	4	0	0	0	0	0	0	0	0	0	0	0
7d2bdb33c2831add541e7b49eb45467a	0	0	0	0	0	0	0	0	9	0	0	0
a2554ec7b6c478739d234dffdd87f6e5	10	0	0	0	0	0	0	0	0	0	0	0
f98af3730e0a218387bf5c01699f6098	0	0	0	0	0	0	0	0	0	6	0	0
c38060e6aafcc2eed857574406d8806f	29	0	0	0	0	0	0	0	0	0	0	0
01a8aee142b6254c0972e8792a476885	3	0	0	0	0	0	0	0	0	0	0	0
c126e73ce8b1cb9243bdd9368b8f259d	0	0	0	0	0	0	3	0	0	0	0	0

dd6f316581e5368e867272c1d7499a59	0	0	0	0	3	0	0	0	0	0	0	0
72d871efa162144434384e45b774dcbc	0	0	0	0	0	0	0	0	0	0	13	0
4ac468e474e9fc41ed0d45dd52c61fe2	0	0	0	0	0	0	0	0	0	0	102	0
a4d8f1763e5b7e31f9e57bf802cb25c4	0	0	6	0	0	0	0	0	0	0	0	0
b4f1ee4d38036578b56418bfc5003db9	0	0	0	19	0	0	0	0	0	0	0	0
48a15e0c864f0340af0591b94842605d	0	0	0	0	0	0	0	0	0	43	0	0
7ba53c39c11da7a54f98d9ed136a701a	10	0	0	0	0	0	0	0	0	0	0	0
62cc8e5ee4761a2f19c6cd5b33aea0fd	0	0	0	0	0	0	0	0	0	0	0	78
a446270dfa878145681e41db7e75dfbb	0	0	0	0	0	5	0	0	0	0	0	0
69d6e3035dd673f13ea73a082030f584	0	0	0	0	18	0	0	0	0	0	0	0
3ee2d2d7535cb749189c9b712445ed0b	0	0	0	0	0	0	0	0	0	0	0	3
3d9f457f4445096d77d065d4b8d969b7	0	0	0	0	0	3	0	0	0	0	0	0
9e075cd5669a042a04dc071ee0ad2fc2	0	0	0	0	0	0	0	0	0	0	0	2
acb86d30ebd7142d3e075ca36f494b8b	0	0	0	0	0	0	0	0	0	0	2	0

b47710c2bab9005bb721b2e9ab425c12	0	0	0	0	3	0	0	0	0	0	0	0
9c4cafe44facaf5dbbe4b7c2ba6d2796	0	0	0	0	0	0	0	2	0	0	0	0
c61702c851ddbdec3371e159b1c40031	0	0	0	0	0	0	0	0	0	0	34	0
6991a791de85079e4475fb06bce3e856	0	0	0	0	0	0	0	0	3	0	0	0
1df996b4db5edaacf661560f3562396e	0	0	0	0	0	0	0	0	0	15	0	0
defab5bf70e5f192f2f93278a0bd0a8d	0	0	0	5	0	0	0	0	0	0	0	0
908e2616e1d189011286671d670d75be	0	0	0	0	0	0	0	0	0	5	0	0
c45ce20e6b66f3d6fcfa1f3896eadedf	108	0	0	0	0	0	0	0	0	0	0	0
28640aa7482c5ab9524ef7fe128dab04	0	0	0	0	0	0	0	0	12	0	0	0
2c75aea995717ecdae4e0ca3a905fc43	0	0	4	0	0	0	0	0	0	0	0	0
6218cf389a43a1e5d92d0fbb10e10cf1	0	0	9	0	0	0	0	0	0	0	0	0
6ed91a6ae8d29a1ffb79ac6338e897bd	0	0	0	0	0	0	0	0	0	0	3	0
3ee1bdeeee66b168c8acd24c8183418e	0	0	0	0	0	3	0	0	0	0	0	0
3eb333a1fb2bf5d80ce9b78e868d0534	0	0	0	0	0	0	0	0	4	0	0	0

4ff2584173b908cf8a0782b2e89a4c0f	0	0	0	0	8	0	0	0	0	0	0	0
238602c9498145e7418715d95cfcab32	0	0	0	0	0	0	0	0	0	0	2	0
993184981bbcfe4a40b5e50e1dbea706	0	0	0	0	0	0	0	0	3	0	0	0
b966b91e0a1c200571ab4bc7d661091c	0	0	0	0	0	0	5	0	0	0	0	0
7ee464cd29767027b7a5334d30666f1f	0	0	5	0	0	0	0	0	0	0	0	0
0daf9f39a2e8d1cc7fe4686e846a79b6	0	0	0	2	0	0	0	0	0	0	0	0
1d90f8f55a1cbc613a970b1b45f24354	0	0	0	0	0	0	0	0	0	0	20	0
6655fd02b6da9661947d3e7bdf2765f6	0	97	0	0	0	0	0	0	0	0	0	0
3a543fca9b3e4f94dc1dc73d33fb530e	0	0	0	0	0	0	0	0	0	0	0	3
b8a29396b8c3315023cf0a5f700c4af3	0	0	0	0	0	0	0	82	0	0	0	0
bb6ec33390c5bb2a33ec174a73d1c957	0	0	0	0	0	0	0	0	0	0	6	0
33f779e741869ad45611f380bca6527a	2	0	0	0	0	0	0	0	0	0	0	0
14db5093eefae72131c24b009f5f8870	0	0	6	0	0	0	0	0	0	0	0	0
1e1d809200f7cd600665882444bd4915	0	0	5	0	0	0	0	0	0	0	0	0

d0a852474608776deeabe3fde2222446	0	0	0	0	0	3	0	0	0	0	0	0
1bc7967023ecec4eea9fd68db8bff050	0	10	0	0	0	0	0	0	0	0	0	0
10a6702dbb59c73e8b1fb6a0be57b231	0	0	0	0	0	5	0	0	0	0	0	0
2c65e9813dffc81619bf8a94992192dd	0	0	0	0	0	0	0	0	0	7	0	0
5e6f1a3fd774ce61bb4de48852e43fd1	0	0	0	2	0	0	0	0	0	0	0	0
b98ec22c7df6ff578a4bd4f454003803	0	0	0	0	0	0	0	42	0	0	0	0
e59ea49afc0b221057953dc90c0f567b	0	0	0	0	0	31	0	0	0	0	0	0
da39b6ab98eb2cd2646133e472feaf9c	0	0	4	0	0	0	0	0	0	0	0	0
8a8fce8f7a9558d6a4e4907451a0ce89	0	0	2	0	0	0	0	0	0	0	0	0
57ba980621edd1ba5528ead1aa27b766	3	0	0	0	0	0	0	0	0	0	0	0
77a23109e20994c9c0ed7d03ad29b512	0	0	0	14	0	0	0	0	0	0	0	0
b65b61d75da95ae861d9931663c505d5	0	0	0	0	0	3	0	0	0	0	0	0
083d72f99be678c70829dcd3ada23b54	0	0	0	0	0	0	0	0	0	4	0	0
56f7c4c7a00f9f602f863ea7a81dc507	0	0	7	0	0	0	7	0	0	0	0	0

3c3fdd3c826f93f8b8eed0745d1b5884	0	0	0	0	0	0	0	7	0	0	0	0
6c6faf8b30675fd4f48f88ba3203308e	0	0	0	0	0	0	0	0	0	0	0	6
d06c48b95a1c81f697655843e0d5660e	0	0	0	13	0	0	0	0	0	0	0	0
cfc7818e1222305e5984c1568dc4fd0c	0	0	0	0	3	0	0	0	0	0	0	0
07114165f1373ab4f9981bccb08d48f1	0	0	0	0	0	17	0	0	0	0	0	0
3c70636a2772edf0d1306f2246537ccd	0	0	0	0	0	0	4	0	0	0	0	0
8d6a92907fea8934fdc190e11fac356e	0	0	0	0	16	0	0	0	0	0	0	0
d80c3b45d6afcf643e464f213332faa7	0	0	0	0	0	0	0	0	0	0	2	0
a3f8b166686df73ecab7cb130e5e6ebc	0	0	0	0	0	0	0	0	0	2	0	0
1a632b7d06ca8b247cab829108a09721	0	0	0	0	0	0	0	3	0	0	0	0
be6054201ce80f34725d5d2fe1e440c2	0	0	0	0	0	0	0	0	0	0	0	3
60ac4ee5d254f71bed0f3065150b7165	0	0	0	0	0	0	0	0	0	0	3	0
4841ca5b81a3c88781e4f794fe8a3a31	0	0	0	0	0	0	0	0	0	0	5	0
01bf1e0d8168cb6e792e4e002e728d9c	0	0	0	0	0	0	0	0	0	0	2	0

a88294608bd284590c7aa55 2d934db73	0	0	0	0	0	3	0	0	0	0	0	0
4c5dd16b6b687eca49bdac70 82b91ef6	0	0	5	0	0	0	0	0	0	0	0	0
90f2c9c5f2c044d973524faef 19b181a	0	0	0	0	0	4	0	0	0	0	0	0
17d5e59536af771796e72081 80cabb31	0	0	0	0	0	0	0	0	0	0	17	0
89ef7f264f0f5b81e0ed38136 d75c0be	0	0	0	0	0	0	0	0	0	0	2	0
94b461a48608daff0cc091c78 538e6d3	0	0	0	0	0	0	0	0	0	0	0	36
b07bec30f982bd9480dce0d8 5ec11293	0	0	0	0	0	0	0	0	0	0	10	0
9efce0ca1e48c473cb93e487f fce8c01	0	0	0	0	0	0	0	31	0	0	0	0
2705e671e28e6f6ef0595363 6d5f8c19	0	0	0	0	0	0	0	2	0	0	0	0
6ba51ed68f09f528b19064ee 621e08b6	0	0	0	0	0	0	0	0	0	0	4	0
254b02c1c5e9f7bd35574b6b 555e5e1e	0	0	0	8	0	0	0	0	0	0	0	0
0109b86c635ec1ba108ebd8 461c43d94	0	0	0	0	0	0	0	0	149	0	0	0
45f5bc5c25e4bf3bce7ad588f 7c8148a	0	0	3	0	0	0	0	0	0	0	0	0
ff4c6be1668abfe91077f6a54 754d15f	0	0	0	0	0	0	3	0	0	0	0	0

ebe1592b7f470a7665a637fa206a314d	0	0	0	0	0	0	0	0	0	0	8	0
9e822426cf4f39d5fce7913b8cef51d7	0	0	0	0	0	0	0	0	0	0	0	19
9f68330bccc44f8e3df5e6627873caa4	0	0	0	0	20	0	0	0	0	0	0	0
ffea0850b12d470c620805ec26ad86aa	0	0	4	0	0	0	0	0	0	0	0	0
6cd18b0146ccd53baceae65a0e5eae58	0	6	0	0	0	0	0	0	0	0	0	0
8c2d6d49d437a3bfae57fdf6f2243c50	0	0	0	0	9	0	0	0	0	0	0	0
a68655776683cba1d5de64e880cd14b5	0	0	0	0	0	5	0	0	0	0	0	0
d22babd4a0aff810220580f54a64e44c	0	0	0	0	3	0	0	0	0	0	0	0
3ec9ea5f2c624322676779b8a73dd138	0	0	0	0	0	0	0	0	0	0	16	0
d5711982b11abf6035557c48b08a1ca8	0	0	0	0	3	0	0	0	0	0	0	0
12a8f844225621e84314dab6f9d09206	0	0	0	0	0	0	0	0	0	0	5	0
5cf29dd36c4abd8d3454c61734660ced	0	0	0	0	0	0	0	0	0	2	0	0
08f9b0e117e8a833e021c2575e9e4ff2	0	0	0	0	0	0	0	8	0	0	0	0
2476d07bbea5a22c34d44bfa14c12474	0	0	0	0	0	0	0	0	0	0	0	20

9081d2d0145bcff9df5b9f08cf7b5978	0	0	0	0	0	0	0	0	0	0	4	0
c54682d6d0929616c74715062390c067	0	0	0	0	0	0	0	0	0	63	0	0
f14f05f4748616ed4415a4dc57d64921	0	0	0	0	0	0	26	0	0	0	0	0
4482054bbacf35f16c2b61d6cb909f08	0	0	0	0	0	0	0	0	0	0	3	0
8f855e0daf2dbaaf3bf074e97029c03d	0	0	0	0	0	0	0	0	0	0	20	0
b71c3075932c50de94c9ae0969440da8	3	0	0	0	0	0	0	0	0	0	0	0
c45a13a48fa50da4ea6233c2e24e2145	11	0	0	0	0	0	0	0	0	0	0	0
5a51b66bc32c1890cbc2d31dd391370e	0	0	0	0	93	0	0	0	0	0	0	0
6f63126d9957e8bf52d2392cf08b7d4a	0	0	0	4	0	0	0	0	0	0	0	0
d357f8621506f81ed5a1e0973d0e9b5d	0	0	0	0	0	0	0	0	16	0	0	0
c1e3f792c46fdb52897efc751ca1202e	0	0	0	0	0	0	11	0	0	0	0	0
ff07b5a70a927dcec938bf9adbdf17b2	9	0	0	0	0	0	0	0	0	0	0	0
31cfaf50204546992b04e2f1155289fa	0	0	0	0	0	0	0	0	0	0	11	0
525492f576921a244d280032bea61e85	0	0	0	0	0	0	54	0	0	0	0	0

47c14b7fe11964abadcfccb0ef2c4910	0	0	0	0	0	0	0	0	0	0	7	0
254f63d96230d14e9cab8fe2716e0233	33	0	0	0	0	0	0	0	0	0	0	0
13271ccbb596d05b97f9ba8124601c31	0	0	0	0	0	0	0	0	0	0	3	0
3a7620897f1833cec86ce621acb5e62c	0	0	0	0	0	0	3	0	0	0	0	0
59e6782eb85e848db9c76c43d06bb64b	0	0	0	0	0	0	0	0	0	0	0	5
f5818ba4fc5fcc2d0c140cd112931c3f	0	0	0	0	0	0	0	0	0	0	0	23
d677c2dfe718904e4e553cd74ac17c5c	0	0	0	0	0	0	0	0	0	0	0	4
6c0af2edf49a3a2afc94535885e96da7	0	0	0	0	0	0	0	0	0	0	4	0
2b95e588a482d961796f57e78dc4809d	0	0	0	0	0	0	0	12	0	0	0	0
ca1063cf8f167c35f9a9428cbb902c30	0	2	0	0	0	0	0	0	0	0	0	0
bcfed8499b48ae3b3d3c3fc37ae922dc	0	0	0	0	0	0	0	0	0	0	20	0
1712fa2cd782aff2996aa49ad7ad67f8	0	0	0	0	0	0	0	0	0	0	23	0
e83a22cc10fe779e086a06d87a0e8f71	0	0	0	0	0	0	0	0	0	0	107	0
f99f66925ff461bf3f776802e9fafafa	0	0	0	0	31	0	0	0	0	0	0	0

b96fd6f3e096b459dfdb57a508515071	0	0	0	0	0	0	0	0	0	0	0	18
26b5d649212276d2eda33e6458250178	0	0	0	0	0	0	0	0	4	0	0	0
bf0a6c9a2480f5dce49ca2f7e2aba8a8	0	0	0	0	0	0	0	0	0	5	0	0
2aaeb9cb5463818f0ac23620bf5dedf2	0	0	0	0	0	0	0	0	15	0	0	0
8779e5504fb83228be14620c2b9518a8	2	0	0	0	0	0	0	0	0	0	0	0
d26281d1ab57464915ce81dc4a786be0	0	0	0	0	0	0	0	0	0	4	0	0
235b86793cec976910f1895e4270fc8a	0	0	24	0	0	0	0	0	0	0	0	0
ce93a43655630fa62279e2b6d2c12697	0	0	0	0	0	0	0	0	0	0	210	0
f90a35b8d9757a102232ea4352d442a9	0	0	0	0	0	0	0	0	0	3	0	0
039e223557de512a3f8bc2096cbe29bd	0	0	0	8	0	0	0	0	0	0	0	0
c1a046af7f3d834f1ff48d1b8b73881b	0	0	0	0	0	0	0	0	0	0	0	8
0e6fbbe037346049a983a009e2b1d0be	0	0	0	0	0	0	0	0	0	0	16	0
5fa37ac805872abfa081d8fe721267c6	0	0	0	0	0	0	0	0	0	0	4	0
0ab44b1baa47054c6a15a88ecb17b8a1	0	0	0	6	0	0	0	0	0	0	0	0

1903c0e8bb1264a1a7c24a75e5bc4e6b	6	0	0	0	0	0	0	0	0	0	0	0
87047e3643141013dc51d4220623ce13	0	0	0	4	0	0	0	0	0	0	0	0
30301136b8ba0d21de904bd359e48e6a	0	0	0	0	0	0	0	26	0	0	0	0
80c513498c0ca7c919c064fc44fb75d5	0	0	0	0	0	0	0	0	0	0	0	2
854a124015d0722216c8e5dc908e8747	11	0	0	0	0	0	0	0	0	0	0	0
0f045eae44b2a7dc58ccbe5536b3de30	0	0	16	0	0	0	0	0	0	0	0	0
12de26b53e4887c7cfca87461e324d51	0	0	0	0	0	0	0	0	2	0	0	0
ab4486d6398f97d552e6d8b04bbca0bc	0	0	0	0	0	0	0	0	0	0	12	0
e0437d4f549af6ff1a10cae7624293ac	0	0	0	0	0	0	0	0	0	3	0	0
0724c83d7cdc052ca458dcc3f01924a7	0	0	0	0	0	0	0	0	0	0	0	6
cac599c9c6db09b1cca662ede92fb2cb	0	0	0	0	0	0	0	0	0	0	22	0
22ab3f57322a518a0fbc408c1fb2be87	0	0	0	0	0	0	2	0	0	0	0	0
cb16c6be02c30dca0ba6109918c589f1	0	10	0	0	0	0	0	0	0	0	0	0
14b5a9ee63e64524bf97858fe273524c	0	0	0	0	0	0	0	3	0	0	0	0

e0f688e8c06734aeac414e41266ac4aa	0	0	0	0	0	0	3	0	0	0	0	0
4b4b250c140e3fc577af207c62e8c176	0	0	0	0	0	0	0	0	0	0	0	9
06bef60a50df2e94fe2ecf60285bc19e	132	0	0	0	0	0	0	0	0	0	0	0
f392a2e7cc33e8471f13dab58dd39292	0	0	0	0	5	0	0	0	0	0	0	0
3d036fde6c3409a8821434dfaf1d7bd9	0	0	0	0	0	0	0	0	0	0	11	0
18e930d81a9ff8d1f75ac6b9d4a8c668	0	0	0	0	0	0	0	0	0	0	0	3
ae0b550f9385a5da907d8fcfd57c5243	0	0	0	0	0	0	0	7	0	0	0	0
bcc0ac8299204522c7374fdbf433d704	0	0	0	0	0	0	0	0	0	0	7	0
22dc29cd1494d305e0c32a41bd489de3	0	0	0	0	0	0	0	0	0	0	5	0
5c340d467bd380cfbe88e854f3a17481	14	0	0	0	0	0	0	0	0	0	0	0
c4b43f7f100d9d498e7c52734bb8bdb5	0	0	0	0	0	0	0	0	0	0	4	0
7862cbef7edaef75b31378a714bb865f	0	0	0	0	0	0	0	0	0	0	6	0
44ffeefa231bd7031200ea9c0ae7906c	0	0	0	0	0	0	0	2	0	0	0	0
87dd5b0a15066a8ed27779d839a91707	4	0	0	0	0	0	0	0	0	0	0	0

877557a93438c710cc63a49b14904169	0	0	0	0	0	0	0	0	0	0	6	0
15c7e23f22545b7366a2fe30a094a8e7	0	0	0	0	0	3	0	0	0	0	0	0
fc17f2f8f9fa966284dd6ddc4e9b72c8	3	0	0	0	0	0	0	0	0	0	0	0
3cc457a72c3a56722a1ab9be7d16827d	0	0	0	0	5	0	0	0	0	0	0	0
649e6f892f4bf63b01ad22ba07990ca4	0	0	0	0	0	0	0	0	0	0	30	0
2b94a0c492929586d8f574fae6e4f685	0	0	0	0	0	0	2	0	0	0	0	0
3e9ca92efdf9b4e55a1a08a3a962dfe9	0	0	0	0	0	0	0	0	0	0	16	0
d28a31676139854fa67a883a19882dd6	0	0	0	0	0	0	26	0	0	0	0	0
145105e650b61d37e4eda735c70f97a3	19	0	0	0	0	0	0	0	0	0	0	0
496e6c006474a725c8cc9b73ff97e394	0	0	0	0	0	0	0	0	0	0	2	0
9ba87ff419b9a7c2024fc95425107516	0	0	0	0	0	0	0	4	0	0	0	0
a2652bd3e2da1ad0f0aff38193344eee	0	0	8	0	0	0	0	0	0	0	0	0
21898caee757d7cb30cdc15462a92543	0	0	0	0	4	0	0	0	0	0	0	0
4abc0e5292012c875022d3ebfd28a1ae	0	0	0	0	0	0	0	0	0	0	27	0

174bab583d756b1476109ee347d806ea	0	0	0	0	0	0	0	0	0	0	3	0
340399c575bdd0cf03c1662bf52ceeb3	0	0	0	0	0	0	0	0	0	0	12	0
4cfd04a3a8f2af86b99fcebe601444e6	0	0	0	0	0	0	0	0	0	0	0	3
9854fd538477575e2d0f76ed0c95dc9b	0	2	0	0	0	0	0	0	0	0	0	0
f716f12cf0e105b85beb4e2631dfb12e	0	0	0	0	0	0	0	2	0	0	0	0
ed114661246ede8550ae3d9fa30ccaa0	0	0	0	0	0	7	0	0	0	0	0	0
7ab94ef386f0ace03e7981b0f6b32ff0	0	0	0	0	0	0	0	4	0	0	0	0
c7bf829b34bc75f3635cea5ea2e15672	0	0	0	0	0	4	0	0	0	0	0	0
10ddc1629842b7450062e9c6b4f2a1ea	0	0	7	0	0	0	0	0	0	0	0	0
ab73612f7c40f59c14f91c2011cc0c62	0	0	0	0	0	0	0	0	0	0	0	2
5efb213c98ef849b926c89bf9b318396	110	0	0	0	0	0	0	0	0	0	0	0
588b14dabf0368df6c928eee098c4a95	0	0	0	12	0	0	0	0	0	0	0	0
eea66a66815374d4f226266349da8289	0	0	0	95	0	0	0	0	0	0	0	0
75174d117c789e3eaeb5656f0db55427	0	0	0	0	0	0	0	0	0	2	0	0

a97e8a1e83d0656800af8ada845ecc2e	0	0	6	0	0	0	0	0	0	0	0	0
7f7ffb6283f2bc767eb9c2edc9c98d60	0	0	0	0	0	0	0	0	5	0	0	0
ad439f9a17eb6ad1760ac9ddb073e012	2	0	0	0	0	0	0	0	0	0	0	0
6762effb662495d48165cf6d8743ec11	0	7	0	0	0	0	0	0	0	0	0	0
542d3ba201732d693af09a6dcf1ab008	0	0	0	0	0	0	0	3	0	0	0	0
7a30ad6182512e21567200f827dfe80c	0	0	0	0	0	10	0	0	0	0	0	0
b4ce17bd738d36d5d6c70eeccd6f0aa8	0	0	0	0	0	0	2	0	0	0	0	0
aa5a5f0fe473291447a2b26abafd75e5	0	0	0	0	3	0	0	0	0	0	0	0
62965b7fd25219214111d53895100f3e	0	0	0	0	0	0	0	0	0	2	0	0
ef68c3117be877dceb104a4efaa6e5e4	0	0	0	0	0	0	0	116	0	0	0	0
d9dd6c04248e5845057f914487fa7672	0	0	3	0	0	0	0	0	0	0	0	0
655048b7ea1452f1dabeb276fffaf3b5	0	0	0	0	0	0	0	26	0	0	0	18
1252e4c28ad09e6538b046db0cee6124	0	0	5	0	0	0	0	0	0	0	0	0
ab4f63eddfe4e223d7824ef7020d3653	0	0	0	0	0	0	0	0	0	0	5	0

403617a9be0c9d837c0ff3dd8cc015d8	0	0	0	0	0	15	0	0	0	0	0	0
b1889e07a9d01bc448f349ed9867c507	0	0	0	0	0	0	0	0	0	0	6	0
c44cdc9e522d4d010b9a380444b0c8e6	0	0	0	0	0	0	0	0	0	0	0	61
748dcca4a35213d0b37082106a77867f	0	0	3	0	0	0	0	0	0	0	0	0
6c4e22d399a3e4f22455befea92d6ef9	0	0	0	0	0	0	0	0	0	0	5	0
680da9093c7c69382e360eff481d1193	0	0	0	0	0	0	0	0	0	2	0	0
6a2e2e6837e37b2fd3add9ae1b62249d	0	0	0	0	0	0	0	8	0	0	0	0
9e09b98068c70d7fe5227357380881f9	0	0	0	0	0	2	0	0	0	0	0	0
afac7bcf73851dab84851b0fe8384d89	0	0	0	0	0	0	0	0	3	0	0	0
ed22c99f6d157c5ccdca5249433d273f	0	0	0	0	21	0	0	0	0	0	0	0
13addbd8dd1f804a4f6feb990b1f5081	0	0	0	0	0	0	0	0	0	0	0	47
4479392f6693de5653a67a609311a018	0	0	0	0	17	0	0	0	0	0	0	0
52c079dc2c6d7a89aa25a6ef93c5ce58	0	0	0	19	0	0	0	0	0	0	0	0
3c97955bf49936f2c15a2b7bc01bb22b	0	0	0	0	2	0	0	0	0	0	0	0

ce7372d7021179baca344d7750ce4b81	0	0	0	0	0	0	0	56	0	0	0	0
e1c8dd7704486bda840662e590e9da1c	0	0	0	0	5	0	0	0	0	0	0	0
98efd5aa7b068c7c0188ec72c09dcd4e	0	0	0	0	0	0	0	0	0	0	0	21
f0b6f020e52524eb0cb9dc16251d3650	0	0	21	0	0	0	0	0	0	0	0	0
dbb6feb6c06634fb9cc8b40de5e0d45b	0	0	0	0	0	0	0	5	0	0	0	0
31634aead9cbbccd8184c49b6cd79cc1	0	0	0	0	0	0	0	0	0	0	0	12
94e7f9545cd8e83fb5145a1828ca6f2c	0	0	0	0	49	0	0	0	0	0	0	0
a6c15d8a12fd0c4adc8d63d20af9b4f8	0	0	0	0	0	0	0	0	4	0	0	0
6528132a4b131d633e3b82d1d610a1c9	0	0	0	0	0	0	0	0	0	0	25	0
a34c1a7e40194d1df202935cd37b2477	0	0	0	0	0	0	0	0	0	0	0	6
7be7fca833bc5df2542fd7bb0e88c018	0	0	0	0	0	24	0	0	0	0	0	0
9ade48700fc09de1ba4c485cb8b11eb4	0	0	0	0	0	0	0	38	0	0	0	0
d9cf7b4ed869675e220b3fd5af355c89	0	0	8	0	0	0	0	0	0	0	0	0
89c9974ec45fea901be42b070569d5f9	0	0	0	0	0	73	0	0	0	0	0	0

eb7fc6b0831a9b9ca55c7692af7ba814	0	0	0	0	0	0	0	0	0	0	5	0
611df297f1ecf0e3b874d9f621d67222	0	0	0	0	0	0	0	0	0	0	2	0
d6aeece81ff3058bfda0ba41286c8015	0	0	0	0	0	0	0	0	0	0	4	0
3fdfbe5928cb833a61e514bd0c88350f	0	0	8	0	0	0	0	0	0	0	0	0
8ef925c53f851dddfd28bdd8f326406a	0	0	0	0	0	0	0	0	0	8	0	0
f738c8a29cfac785f3a07cd1ca23e6fa	0	0	0	0	0	0	0	0	0	2	0	0
223465b4378d975c6c014cd2cfe970f1	0	0	0	0	0	0	0	0	0	0	0	4
4952087cc9afd798ffb555840d6ed43b	0	0	0	0	0	0	0	0	0	0	0	2
a45f0bd720445f445820cebb8fce43f2	0	0	0	0	0	0	0	0	0	10	0	0
503b6b1839a3442d558afa083d1b77c4	0	0	0	0	0	129	0	0	0	0	0	0
4cd1dab32a92c63c6c536802141dd9d3	0	0	0	3	0	0	0	0	0	0	0	0
5ee997cd8f0f8ee3b543f00a1b48e1a7	0	0	0	0	0	0	0	0	5	0	0	0
9df0d6d161b678e407f95df0097c238b	0	0	0	21	0	0	0	0	0	61	0	0
43c7606bacd5433d1ca2d39d7195e68e	0	0	0	0	0	161	0	0	0	0	0	0

02d0a9e24b3a1cc1b2f584cc df2539bc	0	0	0	0	2	0	0	0	0	0	0	0
cb22649ad5257bb40237efbb d53dd722	0	0	0	0	0	0	0	2	0	0	0	0
47901c85b2b9e4b9b223523 855d5b5e5	0	2	0	0	0	0	0	0	0	0	0	0
a314c035bc0b02dc237721e6 ac2802f2	0	0	0	0	0	0	0	0	0	8	0	0
d8e9b99c75d49da216f3ede0 fa9c298c	0	0	0	0	0	0	0	0	0	0	4	0
38d68efa477138cbbddca5a4 d89c9e86	0	0	0	0	0	0	0	0	0	0	0	34
7ac18d69ec78defbb8eaaadd 93d25b45	0	0	0	0	0	0	0	0	0	0	16	0
75788ef65ce1cd258eeaf653 58146a5e	0	0	0	0	72	0	0	0	0	0	0	0
28f252514da0f884098b41cc b2f47643	0	0	0	69	0	0	0	0	0	0	0	0
8e8c8f8fd24f390e1bf4008f2 8e66269	40	0	0	0	0	0	0	0	0	0	0	0
82776e3ad89f3c9fb8c1ff1aa c328c07	0	0	0	0	0	0	0	0	0	3	0	0
eef745b24cb937a752ec26b6 3c5425d9	0	24	0	0	0	0	0	0	0	0	0	0
6982194210b060cdc561e0a 327cd4bee	2	0	0	0	0	0	0	0	0	0	0	0
c59902aeed24e279f55dfc64c c2826cb	0	0	0	2	0	0	0	0	0	0	0	0

c58cbaaa28be3552bcb0533ba4ee49f0	0	0	0	0	0	0	0	17	0	0	0	0
6a1158311a61f8cea10917d569402209	0	0	0	0	0	0	0	0	0	0	2	0
68db6edbd2d5f27d5ac7440391d53d15	0	0	0	0	0	2	0	0	0	0	0	0
8310522ac11f22a23060ad8bccf3184d	0	0	0	0	0	0	0	0	0	4	0	0
b57069604fd67864e7ccae3680ab1779	0	0	0	0	0	6	0	0	0	0	0	0
7f85f0c41b366edd5b0b5137b9b12acf	0	0	0	0	0	0	0	0	0	0	35	0
b48d5fc503957d5eaef02ef843e76249	0	0	6	0	0	0	0	0	0	0	0	0
e4b44a38f0dbacb8d0fd32937a106ad2	0	0	0	0	0	0	0	0	0	0	5	0
cf40befa7350cc31cd94f85c4a144e54	0	0	0	0	0	0	0	3	0	0	0	0
e80cd88880670cbfc54ef5cc2d280441	0	0	10	0	0	0	0	0	0	0	0	0
6c0b39e19b52ee788884d57bc2b8dc00	0	0	0	8	0	0	0	0	0	0	0	0
bf232924704f2e5bd1489eac31056d94	0	0	0	4	0	0	0	0	0	0	0	0
431ae847b1bdbbbbca16d694c5689ad5	0	0	0	0	0	7	0	0	0	0	0	0
d702cc4904f927b770610547762c7d48	0	0	0	0	0	84	0	0	0	0	0	0

e13c8064a9cc1bbf83d5962e e3e98c37	0	0	0	0	0	0	0	0	0	11	0	0
021eff875661019b804a852a 91a61084	0	0	0	0	0	0	0	0	0	3	0	0
8d228d67e2ef5074a384c2f9 a626425f	0	0	0	62	0	0	0	0	0	0	0	0
e2c90f36a31b5aba86449e8a ebd92a1a	0	0	0	0	5	0	0	0	0	0	0	0
8b1f9ad217743057a30b9288 c71ff494	0	3	0	0	0	0	0	0	0	0	0	0
dfd0c8fb56a56156b917cbb5 e17688fb	0	0	0	0	0	0	0	5	0	0	0	0
23c0c52da04188ecb7d70284 1eacc813	0	0	0	0	0	0	0	0	3	0	0	0
bf99c02d20dcd13c0f9c1a58a 1339742	0	0	0	0	0	0	16	0	0	0	0	0
bb57cee4306b85ebf62cc504 5732d881	0	0	0	23	0	0	0	0	0	0	0	0
2793317ee7fa217f4575cd38 65b5727b	0	0	0	2	0	0	0	0	0	0	0	0
38dc2ea67c3bb0e8e899d26 8db5c6ed2	0	0	0	0	0	10	0	0	0	0	0	0
bef3438e440d7b3fa42b15b2 2eba3fc0	0	0	0	0	0	0	63	0	0	0	0	0
c4136fb2cbe75a0cf84bff5ef4 5f11dc	7	0	0	0	0	0	0	0	0	0	0	0
c5654ea37aff26469fd085794 7529e5b	0	52	0	0	0	0	0	49	0	0	0	0

5e297a8fd45f8c6d4fcc9a3b42550eeb	0	0	0	0	0	0	0	0	0	0	0	3
3d6f9bf363c3cf03ffde0f9b6f488e70	0	0	0	0	0	0	0	0	0	0	2	0
2fdb2783257c92ff429648004aa6a04c	0	0	0	0	0	0	0	0	0	81	0	0
1770ff8ccccd3bfbc4d292dbb46a2eff	0	0	0	390	271	0	0	0	0	0	0	0
10c23d9f39388d688e8a36a48df783c0	0	0	0	0	34	0	0	0	0	0	0	0
2029634a23e9f7721d5ddcdfe07620bd	0	0	0	0	0	0	0	0	0	0	3	0
ecd4aa2777229ae817e8e7681ef0a2b1	0	0	0	0	0	0	0	0	0	0	4	0
1b035395452bb3ccbf1c9384c34f0a3a	0	0	0	0	0	0	0	9	0	0	0	0
50a1b97ca447e391401f148e86248376	0	0	0	0	6	0	0	0	0	0	0	0
89c70bfd378776a675d285c62c9dbdcb	0	0	0	0	7	0	0	0	0	0	0	0
b3154a61376dcb26dffd0199516f1397	0	0	3	0	0	0	0	0	0	0	0	0
2ed78923b191f5f7b802ebdb105a9535	0	0	0	0	0	0	0	0	0	0	86	0
d2b25a15c85fc8228de37cb22de23790	0	0	0	0	2	0	0	0	0	0	0	0
87a1ff7ab58d0270b3dda03275280e5f	0	0	0	0	0	21	0	0	0	0	0	0

1bf2e6c8131b09dd2e43ba760994a5e9	0	0	0	0	0	0	0	0	0	0	2	0
459e913d5ac4b523026505f604005811	0	0	0	0	3	0	0	0	0	0	0	0
1ad467bfab5f0963c519130d1d48caf6	0	0	0	0	0	0	0	0	0	0	0	2
1394513b43d1153aac07573e247b1f14	4	0	0	0	0	0	0	0	0	0	0	0
4187a43297e0b14a007dd72a538b8865	0	150	0	0	0	0	0	0	0	0	0	0
bac96dcf3a12ccacb15154a57d18722c	0	0	0	0	0	0	0	0	0	0	3	0
1860754d00147ad71271423e5addb765	0	0	0	0	0	0	0	0	0	0	0	10
1a2a16494492b7187a890425b9b8e8a2	0	0	0	2	0	0	0	0	0	0	0	0
f640b41c1a69d087c527195a22cf9647	0	43	0	0	0	0	0	0	0	64	0	0
cde3353af90555ada452983f9eaf0642	0	0	0	0	0	0	0	22	0	0	0	0
0fc3a9758f61ad2911e68d0c178089c9	0	0	0	0	0	0	0	15	0	0	0	0
de61b676aa352f2709830c65f43973b5	0	0	0	5	0	0	0	0	0	0	0	0
f546a2f636f69fdfd6232654669911c8	0	0	0	0	0	0	0	2	0	0	0	0
4e642105ca4c1fd332cb3e77a732380d	0	0	0	0	0	0	0	0	2	0	0	0

86b91c123dbd39649794f44ba8d75aed	0	0	0	0	0	2	0	0	0	0	0	0
b19ac2ee2c92f5926ab503a7be0922d1	0	0	0	0	0	0	0	0	0	14	0	0
01fbd5db983c9e345e2882af47d4e532	0	0	16	0	0	0	0	0	0	0	0	0
fef02874936b3aa86afa1c440854b464	0	0	0	0	0	0	0	0	0	0	0	2
bf32fcb21dec7399f626ee62e3e72981	0	0	0	0	0	0	0	0	0	0	2	0
7f9160455bb6c31cefa50fad9dc4ac04	0	0	0	0	0	0	0	0	0	0	3	0
b118a7cf510b0275b4c54ac104aae6f3	0	0	0	8	0	0	0	0	0	0	0	0
89879d364769a045d2741c2288584152	0	0	0	0	0	0	0	5	0	0	0	0
fd4fbe12163ee1a374e9a5c5129d5101	0	0	0	0	0	0	0	0	0	4	0	0
6a2a69dd70ed6d10da3b07e89f852713	0	0	0	0	0	7	0	0	0	0	0	0
0b335a48b05a8c80898d3962aebe4e55	118	0	0	0	0	110	0	0	136	0	0	0
35859df11425bda38423113f591b8ff4	0	0	3	0	0	0	0	0	0	0	0	0
44158fd0694a13981823e721a8bc3185	0	0	0	0	0	0	0	0	0	7	0	0
1e2d831fa6e69c348fdebac2db6707d6	0	0	0	0	0	0	7	0	0	0	0	0

6fc3ba19f98fdb46968f7e0d9ec9d67e	0	0	0	0	0	0	0	0	0	0	7	0
43f060d0cc084805e8e00be3b32768fc	0	0	13	0	0	0	0	0	0	0	0	0
944b2556cf6cd0270f6b9055cac87bd5	0	0	0	0	0	0	0	0	2	0	0	0
6914499f3966eb137b44124934943836	0	0	0	0	0	0	0	0	0	0	70	0
8dfb4f94061d4b688c3d19c0d03bdb7d	0	0	0	0	0	0	0	0	0	0	55	0
c6fe20d35800207e65913e550671c4ee	0	0	0	6	0	0	0	0	0	0	0	0
26ebdd2c2c0a13f2fc22aa307cebfcbb	0	0	0	0	0	0	0	0	4	0	0	0
480f0d52c5168752f23c463b4dd48cad	0	0	0	0	0	0	0	0	0	0	12	0
ffaf74a65808ce5e70cb736f951c864a	0	0	0	0	0	0	0	0	22	0	0	0
5793a99df150001252181e0f55bd5fdc	0	0	0	4	0	0	0	0	0	0	0	0
6b71838476085164e0ef28b04e96cae0	0	0	0	0	0	0	0	0	0	0	0	12
9fbeee640329edee380ea45d9956d7fd	0	0	0	0	11	0	0	0	0	0	0	0
5c579833c70322ca0ff381e2f31df745	0	0	0	0	0	0	0	37	0	0	0	0
f5e03d31743ff763ba07fc803554429a	0	0	0	0	0	0	0	0	0	3	0	0

c6e4f49cb798b961467693b154b00ccf	0	0	0	10	0	0	0	0	0	0	0	0
1eee83ae2724dcbea96e4762e7b0061a	0	0	0	0	0	0	0	0	0	0	3	0
89a2dfb4a5e7730fda2aa45971497b83	0	0	0	0	0	0	0	0	2	0	0	0
9bed14da48ce7804a15535fd1f49c283	0	0	0	3	0	0	0	0	0	0	0	0
f716d71fd046837d1503b7dc34b4d9f0	0	0	0	0	0	0	0	0	0	3	0	0
a4314ab931238f9c4903ec039e998685	0	0	0	0	5	0	0	0	0	0	0	0
8bbb7a6dd9a1432f89456d4fbe3adf8e	0	0	0	0	0	0	0	0	0	0	0	13
e5e4aeba4a4e17f483334a150d523f2a	0	0	0	0	0	0	0	0	0	0	24	0
aa8cb76f1b91afde02f850243532ca27	0	8	0	0	0	0	0	0	0	0	0	0
81ea2a4856c5c81e024f7804e3b8d73f	0	0	0	0	0	0	0	4	0	0	0	0
81268a7006fa6b8e8216f9e029ce1f15	0	0	0	0	0	0	4	0	0	0	0	0
ee3d6c349cf4c871c7d7d4af4faf9b8c	0	0	0	0	0	0	0	0	0	0	0	16
78290ba17335ba95d7a535eb383d33be	0	0	0	0	0	0	0	0	0	0	5	0
5b9c5d6b385e4dedd9b3a98b6b7542aa	0	0	0	0	0	0	0	2	0	0	0	0

0a22c214d8781d1cf60a2b68 12ddc1ae	0	0	0	0	0	0	298	0	0	0	698	0
f39121b3ccea59e49e1e51ad a182a1b9	0	0	0	0	0	0	0	0	0	0	11	0
47bd5ddd253e1f928342d64 ad2086c7d	0	0	0	0	0	0	0	2	0	0	0	0
0a7cf0d1bd7236de56268907 e41c1c47	0	0	0	0	21	0	0	0	0	0	0	0
1d63f8d5cb6e4a9345ca80e7 8c071a05	0	0	0	0	0	0	0	0	0	0	2	0
66186f37e817dad261e982f5 e5632830	0	0	0	0	0	0	0	0	0	6	0	0
b9f46f843f6e4a60142dc605a 4a24bb4	0	0	0	0	0	0	0	0	0	0	2	0
5f8dd428f8485d3f4d29f51b5 2b3f471	0	0	0	0	0	2	0	0	0	0	0	0
e05daea21d06c3e2d2621fe9 3da96c0d	0	0	0	0	0	18	0	0	0	0	0	0
d9f8a81461e13ce1a9683c10 b69d7a20	0	0	0	0	0	0	0	0	2	0	0	0
8bbb748f5d3cb99513d329a6 a56728f5	0	0	0	0	0	0	0	0	7	0	0	0
fba633a84c9cd448c49f749e6 4bce53e	0	0	0	0	0	0	0	0	3	0	0	0
a42c69f9e7d18a7f2930e528 c02b0099	0	0	0	0	0	0	0	0	0	0	19	0
b89822909d163e6f5b587a75 4769a200	2	0	0	0	0	0	0	0	0	0	0	0

52f592093e440b88597a0446dc775fe1	0	0	0	0	0	0	0	47	0	0	0	0
08eb46be962683874e036b0e88006321	0	0	0	0	0	6	0	0	0	0	0	0
2732be663221429e1357364900335c2b	0	0	0	0	0	0	0	0	0	0	2	0
67cb54fdac0f2f0be2666b94ba41d349	0	0	0	0	0	0	0	0	0	0	27	0
0ea1e57dd7c758f36e06d42aebdb3fe7	0	0	0	2	0	0	0	0	0	0	0	0
ecc7fc49e2f9074b342a265efd90df5f	0	0	0	0	0	0	0	0	0	0	0	5
36dcf986a0289077ecbcb2bad995eb19	7	0	0	0	0	0	0	0	0	0	0	0
b48fc27128494f3cda1fea1dbbdf1aeb	0	0	0	0	0	0	9	0	0	0	0	0
8aeefc70b3d2fcad7dcf28f6d9ac26af	0	0	0	0	0	0	10	0	0	0	0	0
09ed114d4500544c416dccb8b4db57c2	0	0	0	0	0	0	0	0	0	0	5	0
d3cb60de8873cb015c943b14b7bb5204	0	0	4	0	0	0	0	0	0	0	0	0
5c8a7b004a3e8770de8592ddcafaf866	0	0	0	0	0	0	0	0	0	5	0	0
1a5199aedd07e9eb6dae64da9f7e5c77	0	0	0	0	0	0	0	12	0	0	0	0
5938015e0b1c0f8c10dbccf703736f58	5	0	0	0	0	0	0	0	0	0	0	0

45bb636eb99115ff98aa8a230e8255ed	0	0	0	0	0	0	0	0	7	0	0	0
ad62f758023461e30012a5f1b1919d17	0	0	0	6	0	0	0	0	0	0	0	0
c54c3c91e6ea25fe7beadeb7ba5e17d4	0	0	0	0	7	0	0	0	0	0	0	0
5c30e5ee61152fd657114aeb1c8253f0	0	0	0	0	0	0	0	3	0	0	0	0
2786ffc7334632021f690a300e2c5451	0	0	0	0	0	0	0	0	0	0	0	21
600ca744bf8eb1fda5df183c3c370b87	15	0	0	0	0	0	0	0	0	0	0	0
e0515b4dd1a528c7b3b90b14a9d63b90	0	0	0	8	0	0	0	0	0	0	0	0
d782998b9ae0a2744633708fab54b6a6	0	0	0	0	0	20	0	0	0	0	0	0
668df5a46e0e86884e488c86a886d637	7	0	0	0	0	0	0	0	0	0	0	0
6258af61e106848e347009d8c30b3dac	0	0	0	56	0	0	0	0	0	0	0	0
9f591d5ab775acaa7a22c105cb36fba4	0	0	0	0	0	0	0	0	0	0	17	0
31926319c3705dc04042ef60cf441bf6	0	0	0	0	0	0	0	0	0	2	0	0
a3b49c9dc8853e143b7e051c6f3a0830	0	0	0	0	0	0	11	0	0	0	0	0
87479922890d054e4b8492b695ef1133	0	0	0	0	0	0	0	0	0	0	3	0

a1b5392f4763edd0b8966e7a6b9846da	0	0	0	0	0	19	0	0	0	0	0	0
8d9da6bbdd5610fdbe0b1595a01070ae	0	0	0	0	0	0	0	0	0	0	0	3
721a711ee474246b41db4139942c93a7	0	0	0	0	0	3	0	0	0	0	0	0
2191708bf6e340a085df8d8dfede5f16	0	0	0	0	0	0	0	0	2	0	0	0
64988ae5f7d396c5cbb82aa5fe7c689d	0	0	0	0	0	3	0	0	0	0	0	0
6adf36135541b52a5ad771bc45c64204	0	0	0	0	0	0	53	0	0	0	0	0
f4a4503fa6892062cbb1441334b33470	0	0	0	0	0	0	0	0	0	6	0	0
71c23aead1f055e192ce2a6c7130fa1d	0	14	0	0	0	0	0	0	0	0	0	0
9a34bde305f2e8f760af414a639627d4	10	0	0	0	0	0	0	0	0	0	0	0
8b2c37fdb005a42919081e9d3bb84baf	0	0	0	0	0	0	0	3	0	0	0	0
63e0c7e3a6a3232df4f67b0681cb5316	0	0	0	0	0	0	0	0	0	0	2	0
275ebbb1bc2b798ad1b819a8911ccfa7	0	0	0	0	0	0	0	4	0	0	0	0
4f58e2ed2e049c5fffb34e08bbb585c4	4	0	0	0	0	0	0	0	0	0	0	0
798a4fdfec488b9df08ef0fe05431811	0	0	0	0	0	0	0	12	0	0	0	0

bb254f0670d7eb1a332eaee181ca26f5	0	0	0	0	0	0	9	0	0	0	0	0
eff2834efe9843fd1e8e2cebf7d0150f	0	0	0	0	0	0	0	0	0	0	8	0
6d65c6b12fd4d8b8018a3a12f24b3c57	0	0	0	10	0	0	0	0	0	0	0	0
b2ad71176110fded921cc672426307fe	8	0	0	0	0	0	0	0	0	0	0	0
a0b12d59050995d87c82ec5f493df2e8	0	0	0	0	4	0	0	0	0	0	0	0
b4076739ffed505504609253597394f6	0	5	0	0	0	0	0	0	0	0	0	0
994372ab088a857d99a7ec6a6962f773	0	0	0	0	0	5	0	0	0	0	0	0
0408333c829923e0dcbbcdb5cca4d675	0	0	0	0	0	5	0	0	0	0	0	0
2799e876b29587f4843ae9015d16e7b4	0	0	0	0	0	0	0	0	0	3	0	0
a853a22e7c0b4e9733cc558cf4a3b91a	0	0	0	0	0	0	0	0	0	0	6	0
07f3e78d774f3bde2d19c86c19143ed6	0	0	0	0	2	0	0	0	0	0	0	0
acaff3ce37d105b8e8816029ce79ae2d	0	0	0	0	0	0	0	0	0	0	0	26
bd90346a5e6a79ee0117aff08456d6ab	0	0	0	2	0	0	0	0	0	0	0	0
c1f95eefede43b9c326600bfe06bef30	0	0	0	0	0	0	0	0	0	0	2	0

5f16bda06ac1fd352771b3eed5140ef8	0	0	0	0	136	0	0	0	0	0	0	0
a878d5a690269ca618f104e51d246da3	0	0	0	0	0	0	0	4	0	0	0	0
7efcd550812ca11b18df39027f289925	0	0	0	0	0	8	0	0	0	0	0	0
0487c57a65cb4b80b95ad15541d2fc45	0	3	0	0	0	0	0	0	0	0	0	0
6fd3160fd3a6f594bd02f1224ccef658	0	0	0	3	0	0	0	0	0	0	0	0
6733152451be6e1e8f4d983bcd0f5ef9	0	0	0	0	0	0	0	0	0	0	3	0
8bf401a25519ad40d10f431cc14b3c6f	0	0	6	0	0	0	0	0	0	0	0	0
b225ec2086d5e4ffa6ef0649a09f0a45	0	0	0	0	0	0	0	0	0	5	0	0
cdc4423016b154af979ece8fd40fcc91	0	0	0	0	0	0	0	0	0	0	4	0
7e5b7a199391b8da70ceac2e8a9602a5	0	0	0	0	0	0	0	0	0	0	8	0
592f7be22e0f85545004f46f5c3d01a2	0	0	0	0	0	0	0	0	3	0	0	0
7ee5be618eae2000b7cd0c32738a4eb9	0	0	0	0	3	0	0	0	0	0	0	0
00768d539ec6c3b8c0169f8d4b3949a3	0	0	0	0	0	0	0	0	8	0	0	0
981c4709664f80f0e870dbd0f2862c98	0	0	0	0	0	0	2	0	0	0	0	0

ef7d78cd5d4a3081b2268834ac5b4564	0	0	0	0	0	0	0	0	0	0	0	3
bdbc106ca2f5c9d845f78544b7a138a8	0	0	0	0	0	0	0	0	0	0	5	0
ced9c09548f434b0c03907b4f3d0c854	0	0	2	0	0	0	0	0	0	0	0	0
10ce0ef6a93cc3e88a2d127fc649c87c	5	0	0	0	0	0	0	0	0	0	0	0
e3bc3b2261c4e7f18933e1636369384f	0	0	0	0	0	0	0	6	0	0	0	0
ba37b9dda1ad84ba318a50b3362360ee	0	0	0	0	0	0	0	0	0	0	0	2
70cccfdb3a6a718f18f4f931fe371c17	0	0	0	0	0	0	0	0	8	0	0	0
a3b4ce09cb9d20fa562eacc4aa550709	0	0	0	0	2	0	0	0	0	0	0	0
c4fcb2ce42652646f04077e9c523e31f	0	0	0	0	0	0	0	0	0	0	2	0
a04dba832cd60881e203040c19a232bb	0	12	0	0	0	0	0	0	0	0	0	0
0479861ea2b673802b09fe2ac7dfa923	0	0	0	0	0	0	0	0	0	0	5	0
37c119a95a3305c3175a83c0f0d7e448	3	0	0	0	0	0	0	0	0	0	0	0
086ab1fef28c282b298c80d61a09e8c7	0	0	0	5	0	0	0	0	0	0	0	0
e01a6b0d4bc900224227bb0d9472eade	5	0	0	0	0	0	0	0	0	0	0	0

af87938077b8857e71731a003aa515fc	0	11	0	0	0	0	0	0	0	0	0	0
c67c56e73f786c8e6bf7c6469907ddf6	0	0	0	2	0	0	0	0	0	0	0	0
38cdec2ceef6736c1698556840b5220c	0	0	0	0	0	0	3	0	0	0	0	0
12619d49c2ec5b974b29586ca3bb4697	0	0	0	13	0	0	0	0	0	0	0	0
dd9190c726c97dc95cd7d20e28df1bfd	0	0	0	0	4	0	0	0	0	0	0	0
da11c519f550749412aec2b1879453d1	0	0	0	0	0	0	0	0	0	0	0	12
b307361f02eb900cc9b114670b018b36	0	0	0	0	0	0	0	0	2	0	0	0
6b106d476b045bb7c24c37c8eb008ebc	0	0	0	0	0	0	2	0	0	0	0	0
ca02e00a8632c820f61b1f5e9c535cfd	0	0	0	0	0	0	0	0	6	0	0	0
cb87b009bda4c55a04df8106df12e21c	0	0	0	0	0	0	0	0	0	0	0	59
0e9d74a39d9517f36f71face3ee6f282	0	0	4	0	0	0	0	0	0	0	0	0
dcebbc8e6e26f273c0bf37092a72c514	0	0	0	0	0	0	0	0	0	0	3	0
ecd854aa1e3da44e5d1492dacc587ff8	0	0	0	0	0	0	0	0	0	0	3	0
8a6832f76e4c0db931be7daa95562621	0	0	0	0	0	0	0	0	0	0	0	10

24f3496805d356b1e09e8466d5752f89	0	0	0	0	6	0	0	0	0	0	0	0
ac1485649eb4b73df184cbe7faf66fca	0	0	0	0	0	0	0	18	0	0	0	0
8724ef5d0de679b00b01e4980a1e2051	0	0	0	0	0	0	0	0	0	9	0	0
38c0900c1ce0a82a216f2202a8d33775	0	0	0	0	5	0	0	0	0	0	0	0
052109803d656670966bc111fe7b4a09	0	0	0	0	0	0	0	0	0	0	0	4
4f3e186366845eca768e16afffd3f24d	0	0	0	0	0	0	0	0	0	0	55	0
77adc2c38fb11329c44c7f9bf45be225	0	0	0	0	0	31	0	0	0	0	0	0
7c77530565b9f4470c75d43a35fc8470	0	0	0	3	0	0	0	0	0	0	0	0
5a6292fe7977634904570dc0359988a3	0	5	0	0	0	0	0	0	0	0	0	0
35fa2d02b9e5cc9823eeb7dfab00ab44	0	0	0	0	3	0	0	0	0	0	0	0
5dd8b6dfcffe488165df8523bd51aeff	0	0	0	19	0	0	0	0	0	0	0	0
b9f1f85dca27be9a6d75045ff89f4c6b	0	0	0	0	0	0	0	0	0	0	0	4
000323ca6d37db87e607eb3f0556ed3c	0	0	7	0	0	0	0	0	0	0	0	0
d86799cf15604add0930124cba6fdbb7	0	19	0	0	0	0	0	0	0	0	0	0

aa860190e7943621397c857802a6d19b	0	0	0	0	0	0	0	0	3	0	0	0
9b79c7a57e9aaa56adf69b3cd52521d2	0	0	0	0	0	0	0	0	0	143	0	0
5ea7a203cbf13bcff79354187b1c89e8	0	0	0	0	0	0	0	0	0	0	5	0
4ea7c7f8a8a785e3e3761fa0b85775d0	0	0	0	0	0	0	0	0	0	0	10	0
fa2f95a014f7fb6e253e92a9afb81990	0	64	0	0	0	0	0	0	0	0	0	0
43214c59a45735f1e9b1eba20eedc1df	0	0	0	0	0	0	0	0	0	2	0	0
0ef7209bddce67651c60817df2a2f963	0	0	0	0	0	0	0	0	96	0	0	0
8e30530a782ed72e7f1dd3e23ff6bffe	0	0	0	0	0	0	0	0	0	0	14	0
fc5275da32d43058f644f18528752556	0	4	0	0	0	0	0	0	0	0	0	0
e1369764279a26eea7e81472fe0a250d	0	0	0	0	0	5	0	0	0	0	0	0
55499b17d3c0e32a1931cc100024b766	0	0	2	0	0	0	0	0	0	0	0	0
d1b887187bc53883031ca7c79ad15d40	0	0	0	0	0	0	0	0	0	0	5	0
ccbd4a459ab6117ecbe73333b8603d6d	0	0	0	0	36	0	0	0	0	0	0	0
847691e9de8a1483f2e6f5d687ff2137	0	0	0	0	0	0	0	0	0	2	0	0

b1e0492b7f7bf848e0540913 3e588da6	0	0	0	0	0	8	0	0	0	0	0	0
4544584b7e1f7abf76e15041 49ed1d83	0	0	3	0	0	0	0	0	0	0	0	0
6521eb007fc56a07e0051137 5a8bf3aa	0	0	0	0	0	0	0	2	0	0	0	0
e62991ea4cfd2f0b4fd026e1b f352f23	0	0	0	0	0	0	0	0	0	20	0	0
e1adf8f532c1b2feaa5b897c2 06a7451	0	0	0	0	0	0	0	0	0	0	5	0
f09ce4230e9f99cb7228da6a 3b0cecc6	0	0	0	0	106	0	0	0	0	0	0	0
d26537c708f3e319ee2ff368d a5a0796	0	0	0	4	0	0	0	0	0	0	0	0
b795547348085ab754404ac 2d20b3ddd	0	0	0	0	0	0	0	0	0	0	0	13
9e9d319c14af97115d3ff0032 b5bdcf7	0	0	0	0	3	0	0	0	0	0	0	0
5bc6f4a02cf8bbde3a5f5970b aba0cf6	11	0	0	0	0	0	0	0	0	0	0	0
f34b18bcb7183dbdda88eb14 d3c387ff	9	0	0	0	0	0	0	0	0	0	0	0
aa56517a822253b0269d118 470676de7	0	0	0	0	0	0	0	0	0	0	13	0
7f6c6276e3e6e723aa78e1ef 5bb66578	0	0	0	2	0	0	0	0	0	0	0	0
764a3f72262562ddd13ff894 6d40c0a9	0	0	15	0	0	0	0	0	0	0	0	0

4aae40d9690ba951769a1cb b78cd99f2	0	0	0	0	0	0	0	0	0	0	3	0
d194279dd764cbefc81e404a a29be2fb	0	0	0	0	0	0	0	7	0	0	0	0
d47b763f3e237154b8adabb b1fc956ce	0	0	0	0	0	0	0	0	0	0	0	3
2dad2a7de1d0be5059511c5 a9ce344d4	0	0	0	0	0	2	0	0	0	0	0	0
8c7f6f853f24b8ce0a95414ad f0993e6	0	0	0	0	0	0	0	0	0	2	0	0
c727c8a12e93c56efc58fb3f8 776af48	0	0	0	0	0	0	0	0	0	0	0	12
c3ecf46931fc917cd0ec1e5d0 968d9a8	0	0	0	0	0	3	0	0	0	0	0	0
863fb9e6fcb0c91541cb3339 29119ad6	3	0	0	0	0	0	0	0	0	0	0	0
13db835e6591a58c4d7515c 50574c5a8	0	0	0	0	0	0	0	0	0	9	0	0
ef2f41b0674484ac08325026 17b95d13	89	0	0	0	0	142	0	0	0	0	0	0
b7c22b0b0c9946dc02a51c06 6cda6514	0	0	0	0	0	0	0	0	0	14	0	0
08858152f1e758ffc3eba7153 4bb2748	0	0	0	0	0	0	2	0	0	0	0	0
b3ebdde9af7e6b428d5a2ed 79e0eabb7	0	0	0	3	0	0	0	0	0	0	0	0
b4af0cdb531e1840706abd6f 9a9afc07	0	0	0	0	0	0	0	0	0	0	0	6

1c42c398b994fdb80a26d873 13b1f927	0	0	37	0	0	0	0	0	0	0	0	0
0fe7393db51e074545809468 d27bc573	0	0	0	0	0	0	0	0	0	0	0	10
3c3c01eb46f8bfa1978e9a30c c211168	0	0	0	0	0	4	0	0	0	0	0	0
1742b2d9f265ecb5d50acb4c 4c0eaf3b	0	0	0	0	0	0	0	0	0	24	0	0
7a38b09835a03406273e8a3 366c0f9ef	0	0	0	0	0	0	0	0	0	24	0	0
7dc43fe0cf56fcc9f35c53984f ce3470	0	0	0	0	0	10	0	0	0	0	0	0
a89f78ce6af9200a740943d9 2eaceda9	0	0	0	0	0	0	0	13	0	0	0	0
e47a130215bfe0cd2ca713ab 0160d290	0	0	0	0	0	0	0	29	0	0	0	0
42bf495f009f51aed88a535fb 271d443	0	0	0	0	0	0	0	0	0	0	11	0
d6d0674eda20e3e8548be02f 5b6d455d	0	0	0	0	0	0	0	0	0	0	0	3
770138e91dbae4ce5e3a921 28a4e6338	0	0	0	3	0	0	0	0	0	0	0	0
acbcb5b1d78d7f88f7c72c2b 711bb9ad	0	2	0	0	0	0	0	0	0	0	0	0
ea02580e4d44e69f6bfb768b 4feb9024	0	0	0	2	0	0	0	0	0	0	0	0
8a03649f8c6cefdc7f49411fc9 109493	0	0	0	47	0	0	0	0	0	0	0	0

7b250f9060d513c037ddbf17 0289a950	0	0	0	0	0	0	0	0	10	0	0	0
639ceb654a3604cc77445f5b 043b9439	0	0	0	0	0	0	0	0	0	0	9	0
e9db56d86e7b6489f7b357f0 aa41541c	0	0	0	0	0	0	0	0	2	0	0	0
96b342ebcc135847a8724cdc 43e8fa79	0	0	0	4	0	0	0	0	0	0	0	0
143387084dd75bc41c84f814 59464bce	0	0	0	0	0	2	0	0	0	0	0	0
5b5c2ba19ab1c1ebad735c81 6701fd0f	0	273	351	0	0	637	0	0	232	402	0	0
5e3814211be7622216e41ff2 2cca0720	0	0	0	0	0	0	0	0	0	0	0	9
392fafbc2f0bd549c8246dc36 507cf56	0	0	0	0	0	6	0	0	0	0	0	0
f0a30a42833feefccd1b23797 db0cb1c	0	0	0	0	0	0	0	0	0	0	2	0
483d3a364ae843f812f9de36 788a97d0	0	0	5	0	0	0	0	0	0	0	0	0
62cab43affe959f5ce91df8d7 265dde7	0	0	0	0	0	0	4	0	0	0	0	0
e614342d38ec79af30a869f9 e670c842	0	0	0	0	3	0	0	0	0	0	0	0
94562964ffdc15bfa682b50e0 62ded98	0	0	0	0	0	2	0	0	0	0	0	0
0c01983a96fc805b9dc3b0bd a62a24c0	0	0	0	0	0	0	0	0	0	0	2	0

c22ed08cdd1f2ac2ab3a414acdf4ab48	0	0	0	0	0	0	0	0	120	0	0	0
1d7bd805a41149ce88a19d0508022068	0	0	8	0	0	0	0	0	0	0	0	0
eb36925d988405e3f03ee4619f3c983e	0	0	0	0	0	3	0	0	0	0	0	0
abb88c89bd7e81c22c40a3cf1a4833a0	0	0	0	0	0	0	0	0	0	0	19	0
ad941729b11c57f9d251a2af515cdb56	0	0	0	0	0	0	0	0	0	0	2	0
9c9287880d29d3e0592ccae625ec930c	0	0	0	0	0	0	0	0	0	0	0	4
787f86d4a8d1f2a81ab7e927ababc374	0	0	0	0	0	0	16	0	0	0	0	0
8d8c7c982d08dcb13970191df68e6e74	0	0	0	0	0	4	0	0	0	0	0	0
0c3d2f7b030b6f78bc062eada16c62ba	0	0	0	0	0	0	0	0	0	0	0	6
2a3335ef468743cdda035327bedbda8b	0	0	0	0	0	0	0	0	0	0	8	0
c69f200c4db384c1f0069d3e4a5a418f	0	0	0	0	0	0	0	0	0	0	4	0
65da7ca3fcde81b70081249ef7d7b85b	0	0	0	0	0	0	0	0	0	6	0	0
e4c3c195293d98454c3baa452f3ce9c3	0	0	0	0	0	0	0	0	0	0	6	0
b9f56011768221bed0efa38742c48056	0	0	0	0	0	0	0	0	0	2	0	0

b23ee255abcd7e12aac0de6de527a761	0	0	0	0	0	0	0	0	0	16	0	0
9e5f8efbf57910bcf5a852a0d6f6b318	0	0	0	0	0	0	0	0	10	0	0	0
7e97ba55dc8ad7397c6a8562430ebc38	0	0	0	0	0	4	0	0	0	0	0	0
3dc14f1710250b81ff3f96e28f44aaf1	0	0	0	0	0	4	0	0	0	0	0	0
9c0c3fea94e853af8d9262b4db37a369	0	0	0	0	15	0	0	0	0	0	0	0
e266d5d84ba58b2c3c8b250dc51e8d23	0	0	0	0	0	0	0	16	0	0	0	0
c90092876e50581a8e30fec351b508a4	0	0	0	0	0	0	0	0	0	0	5	0
36f1de49bda3b256b6ca9df7b513b555	0	0	0	0	0	0	0	0	10	0	0	0
58fd387043c4ef744b0881778c2c3932	0	0	0	0	0	0	0	0	0	8	0	0
8444e83f5dccd22b7fbdeb65217e6bdd	0	0	0	0	0	0	57	0	0	0	0	0
16b3ef67d19097c46e95ed903741c5e6	0	0	0	0	0	0	0	0	2	0	0	0
898d458952011bd96f22327329375bd2	0	0	0	0	9	0	0	0	0	0	0	0
58523d1a7e40a190f5de73b3a8555446	0	3	0	0	0	0	0	0	0	0	0	0
a08c9606635a15e7e43dea2d7d97a4d1	0	0	0	0	0	0	0	0	0	0	25	0

97ef6f4dc1db6d94c3d91fe934ada421	0	0	0	0	0	0	15	0	0	0	0	0
2ca05d5f891045777f11f550ad99f4c2	0	0	3	0	0	0	0	0	0	0	0	0
844df1692b3923b8765a1d65781b428f	0	0	0	0	0	0	0	0	0	3	0	0
94b3ce018e7f396e6fc68fffe4774c07	0	0	0	0	0	0	61	0	0	0	0	0
d69c3b83128c69c45913106e40d2a706	0	0	0	0	0	0	0	0	0	0	68	0
a45f7a2fcbc2d3ec922cc820fa0a10d8	0	0	0	0	0	2	0	0	0	0	0	0
33e679a46c0d05404dbaa7f61acafcdb	19	0	0	0	0	0	0	0	0	0	0	0
3627b4e53257341bff2e3faf2560b281	0	0	0	3	0	0	0	0	0	0	0	0
bf2646ef7dbb486b26a5b6fcc893286d	0	0	0	0	0	0	0	0	0	0	4	0
68ba291fd510a64ed77664e5ba0235c2	0	0	0	0	0	0	0	3	0	0	0	0
1b2e24d8e6f431431a4940aa41fcb291	0	0	0	0	0	0	0	0	0	48	0	0
9375b76942cb8f26fbdd43adddd980f2	0	0	0	0	0	5	0	0	0	0	0	0
30bca10425091cc91e1dde717db85798	0	0	0	0	0	0	0	0	3	0	0	0
4fa46cf660a1b6d4a2940a6d9a3bed58	0	0	0	0	0	0	0	0	0	3	0	0

919dcc22a95bcb1f92329420f98e2999	0	0	0	0	0	0	0	0	0	0	0	6
d43411e54e8b5793b6ba9d87b0f4632b	0	0	0	0	0	0	0	0	0	7	0	0
d4521326ecde8bfc77c0b5a377dddaa6	0	0	0	0	27	0	0	0	0	0	0	0
4547fe216a7213819de2ea6f0c2964c9	0	0	0	0	0	3	0	0	0	0	0	0
5689d696803b2c2d4014caf6e41da070	0	0	0	0	0	0	0	0	0	0	180	0
7d8f2ac365a8c57608f7ec5c0b9492de	0	0	0	0	0	11	0	0	0	0	0	0
bd63eb9008e87b8246f385d541e348ac	0	0	0	64	0	0	0	0	0	0	0	0
fa15b027055aba856856e0da419ce1c7	0	17	0	0	0	0	0	0	0	0	0	0
2c3325b8363e0dd32b1177960880dba0	0	0	0	0	0	6	0	0	0	0	0	0
32af99dcc93166410845996bf861017b	0	2	0	0	0	0	0	0	0	0	0	0
2633d97fb8c65f8feec92fabccf148d0	0	0	0	0	0	0	0	0	0	10	0	0
5ef92225b54ad2119a1ae8444eb66195	0	0	0	0	0	0	0	15	0	0	0	0
a570a34af1c5a906962c5955220cc740	0	6	0	0	0	0	0	0	0	0	0	0
cd4a6895dc881abbda5d5cd0111987bd	0	0	0	0	0	0	0	0	0	0	252	0

d2b5765701a342afad57d4424da02335	0	0	0	0	0	0	0	0	7	0	0	0
fa4a39d970aa58a2036dd89da948c4f6	0	0	0	0	0	0	0	0	0	0	90	0
27ac36b45eb7aa3d6a282c1b3c6cc812	0	0	0	0	7	0	0	0	0	0	0	0
86bfbf21f30dbe98f67089399c2bf6c3	0	0	0	0	0	0	0	0	0	0	34	0
a96782199805dd6544847d2665334310	0	0	0	0	0	0	0	0	0	3	0	0
a982c8d1239b1503cd869d9c9a0dc266	0	0	0	0	0	2	0	0	0	0	0	0
855225ae7be26a73ccf45b213156e31e	0	0	0	0	0	0	69	0	0	60	0	283
7dd68f0021b2325aaf791ebeeba0b69a	0	0	4	0	0	0	0	0	0	0	0	0
d87229c4456b120895cb107f8ccf16ca	0	0	8	0	0	0	0	0	0	0	0	0
efeb4c4e4e7bf7c2a13d2dac9e99e594	0	0	0	0	0	0	0	0	0	0	101	0
4bf2863e4023460942be029bb4ed12b4	0	0	0	0	0	0	0	0	8	0	0	0
1a2130de4e63c325cfe9b6e2768e43a4	0	0	0	0	0	0	0	0	0	0	4	0
f7f30a900138f596c750203a2f24aa39	0	0	0	0	0	0	5	0	0	0	0	0
e7dbeaead96797c9ca15e9dfe1c899a4	0	0	0	0	0	0	0	0	3	0	0	0

b260050424caa9ec9253da04eaf9b6f2	0	0	0	0	0	0	0	0	0	2	0	0
9378a1e2cf62dc3cb70a7903dd7061d2	0	0	0	0	0	0	0	0	0	0	3	0
2a414eb89ae73b609ff3c0eb2e5d45aa	0	0	0	0	0	0	0	0	0	0	4	0
44205c9107016fd8e4baf226c6bf60e0	0	0	0	0	0	0	9	0	0	0	0	0
be61b7e2babf18d69d75c50591890bd3	0	0	0	0	0	0	0	0	0	0	0	3
6372b8c5928a11ec9daf6742ddb7b363	0	0	0	0	2	0	0	0	0	0	0	0
a632b86f173a6fbdcc0ea0c91f62045f	0	0	0	0	0	0	0	0	28	0	0	0
387d13b0bff71e635b94013adb21350c	0	0	0	0	0	0	0	0	0	0	4	0
03bc7731a54ecda74e7962799ff0ea0e	0	0	2	0	0	0	0	0	0	0	0	0
9121f5b6d4647f24b98c112369255010	0	0	0	2	0	0	0	0	0	0	0	0
3f59909df242ac7cfd119580ab5a12a4	0	0	0	0	0	0	0	0	0	0	17	0
70fe8fc55b208bd459aa058735c7ae87	0	0	0	0	0	0	0	0	41	0	0	0
b83b3cf99cf88932db0b2c2aeb3f140a	0	0	0	0	0	0	0	0	0	0	0	2
4f4a1a34d5274093d9e897bdafefcbf2	0	0	0	0	0	0	0	4	0	0	0	0

2c704b7925c67a5b9601cc425cb0080c	0	0	0	0	0	0	0	0	0	0	2	0
a0d7766d6bedb885a5d7b85c451fe605	0	0	0	0	0	0	0	0	0	0	6	0
61902c52295f256bd30c17f310114626	0	0	0	0	0	0	0	0	0	0	0	11
76b8a8e25616799a4e206291fe4d3386	0	0	0	0	0	6	0	0	0	0	0	0
4ded7e18847c4c296a1f61b4d61dabc9	0	0	0	0	0	0	0	8	0	0	0	0
6c7d4b21e08aac300219a966040156c1	0	0	0	0	2	0	0	0	0	0	0	0
ab84976d6f5cc31d691d614f3fd3c755	8	0	0	0	0	0	0	0	0	0	0	0
b72cd0271777ecd99e739694a9ff8598	30	0	0	0	0	0	0	0	0	0	0	0
7825aac85208f0ea4196abbf37636a64	0	0	0	0	0	0	0	0	0	0	0	4
0b22dd190eddbeba61d609c804a50558	0	0	0	0	0	0	0	0	0	0	2	0
6159ba7cf9e276ed353198974bfb0786	0	0	0	0	0	0	0	0	0	0	4	0
71ceb41ddd386bb8750ddf322243ed97	2	0	0	0	0	0	0	0	0	0	0	0
486a1d5fd91b0380c815ddf737ba5762	37	0	0	0	0	0	0	0	0	0	0	0
6fbed91b6b6d16f64c117e43491288dc	0	0	0	27	0	0	0	0	0	0	0	0

01a19bb969d71d226d4bd1e65f693023	0	0	3	0	0	0	0	0	0	0	0	0
31683a1825349483c004657228143623	0	0	0	0	0	0	2	0	0	0	0	0
3997af35a8beeb2ce52a242fcfc765c2	0	46	0	0	0	0	0	0	0	0	0	0
e4fa670cea9fcbd7407bc06fcb5cfe02	0	0	0	0	0	67	0	0	0	0	0	0
d49e6361eb0c40b4af671ef819de6bc4	0	0	0	0	0	0	0	0	5	0	0	0
ee878f489ef8d9b0e23a827c1d33ed80	0	0	0	0	0	0	3	0	0	0	0	0
0ef3970eed04b66708057b8d7f73b52d	0	0	0	0	0	0	0	0	0	0	0	21
1032d5f4c1c72a65923fa83697523492	0	0	0	0	0	0	0	6	0	0	0	0
fd9aa36ff8fad5f6410a177db58730c2	0	0	0	0	0	7	0	0	0	0	0	0
0522146971bcd6ebdf30f1294bf63ef3	0	0	0	0	0	0	0	0	0	0	7	0
4f64b0c6727329cd8250f0cba0f373be	0	0	0	7	0	0	0	0	0	0	0	0
9309f2328705c015a29ac1961080c061	0	0	0	0	0	0	0	0	0	0	0	6
74c10beb436b99961ea026b6a65341e3	0	0	0	0	0	0	0	0	0	0	8	0
d5ae471501a19b4a789074124084f616	0	0	0	0	0	0	2	0	0	0	0	0

f8e5d13295e8cc0423dcf3a5efeb430d	0	0	0	0	0	0	0	2	0	0	0	0
f71d0459fdbd7caa974f93ac9e9c2acf	0	0	0	0	0	0	0	0	0	4	0	0
ca53c8a77766678fbcb79b1b8002a911	0	0	0	0	0	0	0	0	0	0	3	0
2ad48885f2529adc543c3daa0e770693	0	0	2	0	0	0	0	0	0	0	0	0
9e8bb5021aef46d25a4d23d0c8af8b19	0	59	0	0	0	0	0	131	0	0	0	0
a1f0a80c7bfaf4ab0acb02949916bbb7	0	0	0	0	0	3	0	0	0	0	0	0
90c9b8a36b4fc0957d24d2d48cc32cec	0	0	0	70	0	0	0	0	0	0	0	0
6a6e14fda4d97a871b96c68bfd34d603	0	0	0	20	0	0	0	0	0	0	0	0
6ddb664e22467b0ec34e6942c41f09ab	3	0	0	0	0	0	0	0	0	0	0	0
028efa56662a3ac448928f5bc58ec2d3	0	0	0	0	0	0	0	0	0	2	0	0
177f9895f580ab66c381f57e24e217be	0	0	0	0	0	0	0	0	0	76	124	0
1b69e5b3d3b0d982bee780dee817924a	0	0	599	1208	368	1530	770	0	322	0	849	1014
23875773af4fac062b53b555befee54e	0	0	0	2	0	0	0	0	0	0	0	0
8ebc21b86aeecca427673bad9a0ba0ef	0	0	0	0	0	0	0	0	0	0	2	0

0bee53ef72bf75bf94d5f1829191d308	0	0	0	5	0	0	0	0	0	0	0	0
edb5d97f73202ff2cc6e6f1fbed55cb1	0	0	0	0	0	2	0	0	0	0	0	0
68b5f43bb2151b119c0b58b881a815c3	0	0	0	105	0	0	0	0	0	0	0	0
45471ba245a71fe7bf7e1f791d1f6773	0	0	0	0	0	0	6	0	0	0	0	0
64c47c29c18e5f5d6b41deea2622f60c	0	0	0	0	0	0	0	0	0	0	4	0
389b4450e16a9805fae091d45065a17b	0	0	0	0	0	0	0	0	0	4	0	0
e74cf187f8aa5b07d3d3c0c41ad52fd4	0	0	0	0	0	0	0	0	0	0	2	0
78730acac9a6d52db40d8670a385df9b	76	0	0	0	0	0	0	0	0	0	0	0
27791d3692236d269fbe6e7617c2654a	0	0	0	0	0	0	0	0	0	26	0	0
e0b23d826a91c0fd3af52dba18b46c1a	0	0	0	0	0	0	0	0	29	0	0	0
741fa30c46f8b1c53c8dd5af5d0979aa	0	0	0	5	0	0	0	0	0	0	0	0
fc482c39308a17c91a4fdbb9ec786fc4	0	0	0	0	0	0	0	0	2	0	0	0
c96f0b6e28b13f8b35b07264c4ea5b06	0	0	0	0	0	0	0	3	0	0	0	0
e8f3f0bf7855735a1d8ca7707e50b4aa	0	0	0	0	0	0	0	2	0	0	0	0

ac7df0c40c2f13b06e2ce594f3defbd1	0	0	0	0	0	0	0	0	0	0	3	0
cbfb30a089f675582ca060f5d4e58cc5	0	0	0	0	0	0	0	0	0	6	0	0
64774965ee47e756ba55360212e52bdc	0	0	0	0	5	0	0	0	0	0	0	0
fd4c87e8e186db9b36a6f14deba8af66	0	7	0	0	0	0	0	0	0	0	0	0
e889e501ed0c06f4a62b7d2001ee4261	0	0	0	0	0	0	0	266	0	0	0	0
1782977ae0b89fcb4b21d6cbb08fa079	0	0	0	0	0	0	0	0	0	0	7	0
3aa320775304e03108633dcb6e961650	0	0	0	0	0	0	0	0	2	0	0	0
ed6f318c12f72513526a7de9c1c11c97	0	0	0	0	0	0	0	0	0	6	0	0
d3e126762a6c3dea8900ad7ce52dabd8	0	0	0	5	0	0	0	0	0	0	0	0
8cb5e2f36ebdeb24b2defec67eb59c4a	0	0	0	0	0	0	3	0	0	0	0	0
358bce65162de2ce4ae6a42396103f85	0	0	0	0	0	0	0	0	0	0	21	0
d8f813844fd6ee4e12addf42eff1dd1f	0	0	0	0	0	0	0	0	0	0	0	7
0953b9021a5a16bf8068ab5427cad13d	0	0	0	0	0	27	0	0	0	0	0	0
f6cbd0dcb17f59e4a1824d4b511ac64e	0	0	0	0	0	0	0	0	0	0	44	0

e382b1bb46a75f4c227d0520fa46166a	0	0	0	0	5	0	0	0	0	0	0	0
66246184f201c302892da681395788ad	0	0	0	4	0	0	0	0	0	0	0	0
abce654508f6c1af975f73c0f8fdc6d3	0	0	0	0	0	0	0	0	0	0	3	0
011c303c70ed312192bbb184a6a3cda4	77	0	0	0	0	0	0	0	0	0	0	0
56c570b157b3ea4436b0da2cdfed459a	0	0	0	0	0	9	0	0	0	0	0	0
28de63b6ce9a76ca746b61e1489019c9	0	0	13	0	0	0	0	0	0	0	0	0
699401164d27d2fdf7f0c246bad7c64c	0	0	0	0	0	0	0	0	0	0	0	2
7e567d4f58e7e38e5dd7e092b23dc218	0	0	0	0	0	0	0	0	0	0	5	0
10820ae54e1767e5e5374b88e5cd1619	0	0	0	0	0	77	0	0	0	0	0	0
f384b8fbbd2e44b4e6633333002c08cd	0	0	0	0	0	0	0	3	0	0	0	0
171abfe3ed273969e51019193d77162c	0	0	0	28	0	0	0	0	0	0	0	0
a9c87a98c0f0c75c4bcd35dc0be0ed7b	0	0	0	0	0	0	0	0	0	0	18	0
e1551e7511788ef2f6856fb10e1d5449	4	0	0	0	0	0	0	0	0	0	0	0
888e839a1441a96a01c14a9b97399f18	0	0	0	0	0	0	0	0	0	12	0	0

6364a263953b27cc89e9b6e d516afc50	0	0	0	0	0	0	0	0	0	0	12	0
20c79c0a84dc1e0dd81a2f46 7045143c	0	0	0	0	0	2	0	0	0	0	0	0
eec89f0c76f7462d40a29153 0e846b02	0	0	0	0	0	0	0	0	42	0	0	0
41f613a7cff0c9dc57e657054 2f7d741	0	24	0	0	0	0	0	0	0	0	0	0
9842fd5c2957dee19271385f 6ebc8821	0	18	0	0	0	0	0	0	0	0	0	0
ac1361aa83a10821bc911dad fe55b756	0	0	0	0	0	0	0	13	0	0	0	0
cb27a6928e386c5a14ca2f44 10bfec97	0	0	0	0	0	0	0	0	0	0	13	0
934d5e457cc8ffa4968a85ef1 8365aed	0	0	0	0	0	0	0	22	0	0	0	0
01719e507636d6ed86b0fbcd 61a27f7a	0	0	0	0	0	2	0	0	0	0	0	0
ce29f5c675d4f44dcb13c47cd bef6206	0	0	0	0	0	0	0	0	0	0	24	0
e27358710715b91e2326b69 d005407e4	0	0	0	0	0	0	2	0	0	0	0	0
c80263e6abfdf2c93eb5d507 54400956	0	2	0	0	0	0	0	0	0	0	0	0
46cfb8e6fd0f1eeae03a3c0b3 18fde8b	3	0	0	0	0	0	0	0	0	0	0	0
fedc7e2d7563a4b32d492348 f2207446	0	0	0	0	0	0	0	4	0	0	0	0

317b204f96aad0891be98724b8a60a6a	0	0	0	0	0	0	0	0	0	0	4	0
c8978b0eabc9bc47750ee431c27bd3b3	0	0	0	0	0	0	0	0	0	5	0	0
0b162e66d495e71474351ca43ea0207c	0	50	0	0	0	0	0	0	0	0	0	0
1b9a18a0636f6068c89452372fc1ceac	0	0	0	0	0	0	0	0	3	0	0	0
2adba738b29f36259aed5b224217f8c5	0	0	0	0	0	0	0	0	0	0	5	0
73b86b175e3c2c61999d3ed792aa80d2	0	0	0	0	0	0	0	0	0	0	17	0
b8cc1d441704191aa6dd6047e16f003b	0	0	0	0	0	0	0	0	0	0	0	2
bd2d9703edfea27a6efcfa7644f04374	0	0	2	0	0	0	0	0	0	0	0	0
168839e530ca1cadffacf8d7eaef6a46	0	0	3	0	0	0	0	0	0	0	0	0
013a3f654e6523259b3a7f31a5a35c43	0	12	0	0	0	0	0	0	0	0	0	0
0e21c8d98cdda12228e71c5898bb3e6a	0	0	0	0	0	0	0	0	0	8	0	0
cc2419a2c63d1117fbaa2f2c2c364bfd	0	0	0	0	0	13	0	0	0	0	0	0
0597344db7e4689d41fc8b0e2ad0a671	0	0	0	0	0	0	0	0	0	0	12	0
1ed7e95e303976d3b35e9a8fa5b5bb22	0	0	0	0	0	0	0	0	0	0	4	0

3589dd12576aa4485bb11ea5eaab7449	0	0	0	0	0	0	0	7	0	0	0	0
2942fdc059c7f060eb1b7ec8885ff210	0	0	0	0	111	0	0	0	0	0	0	0
0337137cb5f952c65150e76f124910e6	0	0	9	0	0	0	0	0	0	0	0	0
528de373e7afa6ccfaa70788423baa09	0	0	0	0	0	0	0	0	29	0	0	0
b92debc620a240e7eec8fd066275dfca	0	0	0	0	11	0	0	0	0	0	0	0
b3338bde859f9c16c17ff5e786fcda51	6	0	0	0	0	0	0	0	0	0	0	0
74a7caa7032823ad47a0669f63ece425	0	0	0	0	0	0	0	7	0	0	0	0
84c31b7f6e480ff134ab313058fe813b	0	0	0	5	0	0	0	0	0	0	0	0
780ca3757f1d8d9472e017a208c9142a	0	0	0	0	0	0	0	0	0	0	4	0
a747605a5ec7e2e72137f7d0022cbc75	0	0	0	0	15	0	0	0	0	0	0	0
483bdf72051891dbaeb5ae2a07ac4a7f	0	0	0	0	0	0	0	0	0	0	19	0
4856226b7915dca65753af9210ce1448	0	0	0	2	0	0	0	0	0	0	0	0
cb15ae0181a156e56c78a7f08ada7304	0	0	0	0	28	0	0	0	0	0	0	0
71f863596c238a22b860d198862e934c	0	0	0	0	0	0	0	0	0	28	0	0

53125516a938cbe2f15bc2ea5efe1a14	0	0	0	0	0	0	0	4	0	0	0	0
91edf722d5f9f93502d6da7fe16d37c9	0	0	88	0	70	0	11	0	0	0	0	0
c206f785b1faefabddf5d3e800c605fc	0	5	0	0	0	0	0	0	0	0	0	0
d486ec56e0a6bce4a26766ed7c94eea3	0	0	0	0	0	0	0	0	0	0	0	10
7d3a071249e6f30e37a5e2a41700cf35	0	0	0	0	2	0	0	0	0	0	0	0
760daa5dd053babae7360c62385774a7	0	0	0	0	3	0	0	0	0	0	0	0
dd0bfe5794b4b7a613e3b0feeabdf625	0	0	0	0	0	0	0	0	0	0	0	6
90c671e6ccf120a1e3dcbef09c1fe281	0	5	0	0	0	0	0	0	0	0	0	0
349fafda7479b232009977a0ce4dd317	0	0	2	0	0	0	0	0	0	0	0	0
9c779716f0d366199c7314a8e539879c	0	0	0	0	0	0	0	0	0	0	17	0
44e8f4506ed0591047ad4775340519fd	0	0	0	0	0	0	0	0	0	0	4	0
4fc314e198bd952b58a2605e033b1cfc	0	0	0	0	0	0	0	0	0	0	23	0
89d18970235c57bbf4ca0a75813df710	0	0	0	0	0	0	0	0	0	0	7	0
d3340e42c8518cafe6914027701ea37b	0	0	0	0	47	0	0	0	0	0	0	0

6dc9d75fa567c525b9ade353987e8934	0	4	0	0	0	0	0	0	0	0	0	0
32889086576ebab7252a816247aedd1e	0	0	0	0	0	0	0	0	0	0	0	11
78641e0ff191630614a255de739c9cbb	0	0	15	0	0	0	0	0	0	0	0	0
d11d64d75fbf716b649295d4332ae560	0	0	0	0	0	0	0	0	0	68	0	0
ff4a73dcd3a40dbf164a6cddc7b95002	0	0	80	0	0	0	0	0	0	0	0	0
6983dd958b68919ebbe64daae6f3bf6f	0	0	0	0	83	0	0	0	0	0	0	0
1bcbe253b3b13b3c5befb00b7672df10	0	0	0	0	0	2	0	0	0	0	0	0
3466d0b8085f5bece4287f0a3134b87f	0	0	0	0	0	0	3	0	0	0	0	0
5a6f088dfebe0f9e71ff6e545357e0f4	0	0	0	0	0	0	0	0	0	72	0	0
ad90e37f178f34dadcd5f00994655408	0	0	0	0	0	0	0	0	0	12	0	0
d9b13d781bc958644ea58f6ebc427f6a	0	0	0	0	0	0	0	0	16	0	0	0
10f218460d75176492d25f54cbfb805e	0	0	0	0	0	0	0	0	3	0	0	0
b2ec27460a87f84866d41666bea50dd5	0	0	0	0	4	0	0	0	0	0	0	0
a13ab4eda4e0434ee8f2da3d0aff6a42	0	0	0	0	0	5	0	0	0	0	0	0

0ee54b9c75b74e108aefa46f cf06ef1b	0	0	0	0	0	0	0	0	0	0	0	7
660b32b70c06c5b6035f7d0c 1b30fbba	3	0	0	0	0	0	0	0	0	0	0	0
b29503e5c275cbdd0669889c 656eba52	0	0	0	0	0	0	0	0	0	0	0	6
54603705266fbb56687a0879 765e3695	0	0	0	0	0	0	0	2	0	0	0	0
085321e9c1d31caddbc4facf8 bd9c562	23	0	0	0	0	0	0	0	0	0	0	0
f4b91196b27d54e6b680747 28a89bf78	0	0	0	0	0	0	0	0	0	0	7	0
f03765e0a8afebda42656da2 5a5cc628	0	0	0	0	0	0	10	0	0	0	0	0
4bd5bc221720a02b20bc148 d663b3ca0	0	0	0	0	0	0	0	0	0	0	0	10
d8ea75181800991a8e2c64b 37a22b22b	0	0	281	0	0	0	0	0	0	0	0	300
4cac77202b283c59ed9960c8 674acf98	0	0	0	0	0	0	0	0	0	2	0	0
593778e33824d1c4ba85760 45a6cea19	0	0	0	0	0	0	0	0	0	4	0	0
3db5337d12020c596ed95b7 085efdd4f	0	0	0	0	0	0	0	0	0	0	4	0
e73e1861ec118a96a3e99b8a 564611ee	0	0	0	0	0	0	0	0	14	0	0	0
12b0f11ad18d51c778d19698 7dc21c43	0	0	0	0	5	0	0	0	0	0	0	0

b9fecf357563774d065106b9d219193b	0	0	0	0	0	0	0	0	3	0	0	0
64bc6921b84d4648cb9bb6a4435ca023	0	0	0	3	0	0	0	0	0	0	0	0
bf989897b290a4f492c8b33a0934797e	0	0	0	0	0	7	0	0	0	0	0	0
2fad5b51e1adf4904427589aa09e59ed	0	0	0	0	0	0	0	0	0	2	0	0
fa88c1a839bdab31a990aa07a3c2d855	0	0	0	0	14	0	0	0	0	0	0	0
7e508f0c4d7a0bb9a124ab2e21002a84	0	0	0	0	0	2	0	0	0	0	0	0
2354c335f32f58ae478a27713a8bdead	0	0	0	0	0	0	0	6	0	0	0	0
1327f4d5b3143e967a7c39727293efc7	0	0	0	0	0	0	0	0	0	0	11	0
6625b20ff5cd1eb6469d86dcc84c8c7c	20	0	0	0	0	0	0	0	0	0	0	0
9907e2e43b07709e243463c41f9233e7	0	0	0	0	0	0	0	0	0	0	3	0
c150e702d9479f8aec8d316b73c71db6	4	0	0	0	0	0	0	0	0	0	0	0
0486edc56b93393f5219f6881c7de4b8	0	0	0	0	0	0	0	0	12	0	0	0
b88d50e3d64cc486d19ba3ca93b7edb5	0	0	0	0	0	0	0	0	9	0	0	0
d72f0db53a8f3e81ccea73bdae6f000c	0	0	0	0	0	0	0	0	0	0	3	0

26e8267156778df84f7bf23c3e2b6d0d	0	3	0	0	0	0	0	0	0	0	0	0
029b9b59bf82d7eeb6488df0f94bbe7d	0	0	0	0	0	0	3	0	0	0	0	0
d9212311f9a4a1b835c3ef7bc6cf3a48	0	0	0	0	0	0	0	0	0	0	2	0
afa998216f76d933af28f062033e52e0	0	0	0	0	0	0	0	0	2	0	0	0
bf16b998eeb69952532ab3a83f0ca43f	0	0	0	0	0	0	0	0	0	47	0	0
daa7298d6b516f07c34f162b0b54ec51	0	0	0	0	0	0	0	0	0	0	0	62
5a803b8a49e1c13f89d631585b3630d4	0	0	0	0	0	0	0	0	0	0	86	0
ce34bb60c4f318a37dd52c79370e3b5f	0	0	0	0	0	0	0	0	0	0	0	6
871623928c33ea410fd10cf7c3b08796	0	0	0	0	0	0	0	0	0	0	2	0
bae7aed5a8161897275dd803ff2ba6a3	0	0	0	0	0	0	0	14	0	0	0	0
baba871ae08c2f58cc37828885c07464	0	0	0	132	0	0	0	0	0	0	0	0
1fa600ac3657841c9e5b67669a7fe15e	0	0	4	0	0	0	0	0	0	0	0	0
7aecfeec3bbea75b3ba17bf1b20484bd	0	0	0	3	0	0	0	0	0	0	0	0
089d3745f7bb571ef2f6027aa2271e93	0	0	0	0	0	0	0	0	0	6	0	0

105b7a1e0932470c5243199313924486	0	0	0	0	0	2	0	0	0	0	0	0
90756691c49ee38855ed8db50e88ca86	0	0	0	0	0	10	0	0	0	0	0	0
6829785ee7c4e89721ffa7e166553068	0	0	16	0	0	0	0	0	0	0	0	0
c57b309703356e5efd9d983f66c5d3a6	0	0	2	0	0	0	0	0	0	0	0	0
80d7d96cd90aefb63f121a92a4f2acdc	0	0	0	0	0	0	0	0	0	0	0	18
ebca86da8ca34db30c43f025a3a60c19	0	0	0	0	0	0	0	0	0	0	3	0
b2f8418659be5cc40d6bd14a995a08cc	3	0	0	0	0	0	0	0	0	0	0	0
922018ea1d6a03bd4fd8b6895ecea4de	0	0	0	0	0	0	0	4	0	0	0	0
5ea3b345d3775335bdb70fdfa9ce4076	0	2	0	0	0	0	0	0	0	0	0	0
83c2f38f40fecd93bf3b9e3a1d1c377c	0	0	0	24	0	0	0	0	0	0	0	0
12c9cda6dab35758f922d63b566dab5e	0	0	2	0	0	0	0	0	0	0	0	0
950231184da9b6b4fd78d2aa6a18c71a	0	0	0	0	0	0	0	0	0	0	0	2
7cc9a88868e654946ffab3dc5d7e5cbf	0	0	0	0	0	0	0	0	0	0	0	8
ab420a166eea9f32dfe0275891b753b6	0	0	0	0	43	0	0	0	0	0	0	0

5a6e826be0342b31adc5cea8e30a3ed0	0	0	0	0	2	0	0	0	0	0	0	0
17d7d61eaab702e14030cafdfbd20628	0	0	0	0	0	0	0	0	2	0	0	0
93c3ec010b58561d6df9858f215dce60	0	0	0	0	0	0	0	0	0	0	0	2
6d203df3c3ef71d99517b69905d24d9e	0	0	0	0	0	0	0	0	0	0	0	41
3b0bf5bc027321691dbe3c69bb29b155	0	0	0	0	0	0	0	3	0	0	0	0
a3a848756d43d1a829c9f20ce9f46b4e	0	0	14	0	0	0	0	0	0	0	0	0
a7824cd0655506ac416381fed460f56a	0	0	0	0	0	0	2	0	0	0	0	0
3db4744120f3c6b94d0a5023d076ce52	0	0	2	0	0	0	0	0	0	0	0	0
212c81611e3d26a383fb125b67937cff	0	0	0	0	0	0	10	25	0	0	0	0
c0a4b13fd3f0853544e07700a83d0dea	0	0	0	0	2	0	0	0	0	0	0	0
b62ec32c6a37a092755f1df384b397fc	0	0	0	0	0	0	0	0	0	2	0	0
ea648a144aebb44e43799113bceb3775	0	0	0	0	0	0	0	0	0	0	2	0
ebd430540266a4fa8ce55c60d400d834	0	0	0	0	8	0	0	0	0	0	0	0
db51f1f3047d5673484593b3342cda0a	0	0	14	0	0	0	0	0	0	0	0	0

ec025760bd255ea1e804df8c67ddb1f5	0	0	0	0	0	0	0	7	0	0	0	0
499a645ca8ef6da41c2790742fbeed5f	0	0	7	0	0	0	0	0	0	0	0	0
868a8b8695e0473aa42e961da12a7c1f	54	0	0	0	0	0	0	0	0	0	0	0
d3c62e0e389b8975087194c0d3ad73bf	0	23	0	0	0	0	0	0	0	0	0	0
348983da3f0e797a097ad82403560a08	0	0	0	0	0	0	22	0	0	0	0	0
83b1e1c69727cfac760b73332fba0d7d	0	0	0	0	5	0	0	0	0	0	0	0
1d8ac69c6406b73ac5619b9c17821fe4	0	0	0	0	0	0	0	0	0	6	0	0
aafd48de1edeb0de93398492be41c317	0	0	0	0	0	93	0	0	0	0	0	0
a9a369f4ebc2bbbb943d6985d0de0fd9	0	0	0	0	0	26	0	0	0	0	0	0
eb7664b2f31003fdb8eb2bf8068a7878	0	0	0	0	0	0	0	0	0	0	0	2
a07f29def7af0722632a8152472913fc	0	0	0	0	0	17	0	0	0	0	0	0
69c181878d11d4dd75b7df680755229d	0	0	0	0	0	0	0	0	0	0	0	4
039d541afee4b1cb0f61342b72a6403c	0	2	0	0	0	0	0	0	0	0	0	0
78dec873c2a4f1d0bd3c304fb63d85bd	0	0	0	0	0	0	2	0	0	0	0	0

57d4ffebd246907fc8f59b3eca6178ed	48	0	0	0	0	0	0	0	0	0	0	0
c7cf8666eeba2e99b3d29b00782d3d84	0	0	0	0	0	0	0	0	0	86	0	0
aee0d1d415b06ebc39c4c3538a95b906	0	0	7	0	0	0	0	0	0	0	0	0
636638b11088724f88ec98262ce86ec3	0	0	5	0	0	0	0	0	0	0	0	0
4927dc8a6e7015660bcb3889aa6f418f	0	0	0	0	0	0	0	0	0	0	3	0
3d761e4686ce12cc399de68268d6c538	0	0	0	0	0	0	4	0	0	0	0	0
a47b9019f768d4ecb4a9599a160d4871	0	0	0	0	0	0	0	0	0	0	0	10
6da8f4fbdaae32294c84c3943dccfc67	0	0	0	0	0	0	3	0	0	0	0	0
90205c85b659237ec6f3cf2fb1c7f8c7	0	0	0	0	0	0	0	0	0	4	0	0
e378823b20e19d464aac955b5792062c	0	0	0	0	0	44	0	0	0	0	0	0
243f3253c0cfd5766c843515f3923915	0	0	0	7	0	0	0	0	0	0	0	0
a7cc1343b90d253fd6202b17daab7cc9	0	0	0	0	0	0	41	0	0	0	0	0
0ec6724a862e7da1a147cb86f8b0df0b	0	0	2	0	0	0	0	0	0	0	0	0
da10d4415628158ac7586785dfaf7ea5	0	0	0	0	0	0	0	3	0	0	0	0

a4f1e760b63419262e5affa63b08ff26	0	0	2	0	0	0	0	0	0	0	0	0
cc1bfcdfa44f0227f8b67b649bf1acf2	0	0	0	0	0	0	0	0	0	0	0	6
d7bda874a8d1c2e0a2cc07c4e079a985	0	0	0	0	0	0	0	0	0	0	5	0
7d855b36cb57d4dea2614eabafd41f26	0	0	0	0	0	0	0	0	0	0	0	34
88ab2b023e57194b87f070691cdd0b7c	0	0	0	0	0	3	0	0	0	0	0	0
78e21fcf71f11709beb2c84870710fde	0	0	0	0	0	0	2	0	0	0	0	0
8f5c16aa977a4dbebe689832c025abc2	0	0	0	0	0	0	0	0	0	2	0	0
3eff6355f0c4d71053ad02ee2273be3b	0	0	0	0	0	0	0	7	0	0	0	0
0bb0149ba5f2c946d673df71004ea53e	0	0	17	0	0	0	0	0	0	0	0	0
8036ebbd3452c6694e5e936cf146e225	0	29	0	0	0	0	0	0	0	0	0	0
8e751ecd5b3f7524a14b0734a20e0a6f	0	0	0	0	0	0	0	3	0	0	0	0
a9460c34056aebf5f20c16272d85b3de	0	0	0	0	0	0	0	0	318	0	0	0
204c2db8c4e4664372ec796040e6b319	0	0	0	0	0	0	0	0	0	0	4	0
f51641fba8fb2a5ecf371d154b561075	0	3	0	0	0	0	0	0	0	0	0	0

300c3ba35e9c63f8147c4cdd48e61137	0	0	0	0	0	0	16	0	0	0	0	0
7714fbd71fc4ed3e1950feb43ebd9cfd	0	0	0	0	0	0	93	0	0	0	0	0
4235c4f6a351be41e6f822bcbe2e08e5	0	0	0	0	0	0	0	0	9	0	0	0
6d8dac347bf17fbe886785579dc5a919	0	0	0	0	0	0	0	0	0	0	7	0
2e5ab8d9a71cea7b840ae3a4e23a5ce2	0	0	0	0	0	0	0	0	0	0	0	2
69dbecc10534d8f30879b0a6511d4099	0	0	0	0	0	0	0	70	0	0	0	0
8745fdf994629a374a5cb5f2949fd665	0	0	0	0	0	0	0	0	0	0	0	6
8f74c14ad219ede84bfbea898e2b40a9	0	2	0	0	0	0	0	0	0	0	0	0
2fc37854bdb12a5bc43086e3501d30b1	0	0	0	0	0	4	0	0	0	0	0	0
c52cb804f8e2a03c5bda01fee350e6fa	0	0	0	12	0	0	0	0	0	0	0	0
0b3d4b0909a3fd1f0793dc9cf01009d1	0	0	26	0	0	0	0	0	0	0	0	0
31d3d362dd3e3a75d6c0b5c18cb637ee	0	0	0	0	0	0	0	63	0	20	0	0
a58757c046752b8f80962f86c33a9bd9	0	0	0	0	0	0	0	0	0	0	0	2
98501f0f9fb8aeb8aaf87ed66e17335f	7	0	0	0	0	0	0	0	0	0	0	0

20066cf064b7dbf3ffe5398376493f51	0	0	0	0	4	0	0	0	0	0	0	0
2be893575d1dea15db3956677cb26000	0	0	3	0	0	0	0	0	0	0	0	0
4e4b64bfb5f78c1d724073f1d11ca242	0	0	0	0	0	0	0	0	0	0	28	0
69b6db98ec07f8229e3f116fe36a75c5	0	0	0	0	0	8	0	0	0	0	0	0
79670fcb36dda3c69656574616c342dd	0	0	0	0	0	0	15	0	0	0	0	0
0d19f04a0564dc1992fe85dae0c04b9d	0	0	0	0	0	0	0	0	0	0	3	0
51bd0603cdc41f45dba8e5630f1cfbf5	0	0	0	0	0	0	0	0	0	0	17	0
bcda0c7f995b1ffc5e9ab97f5d2c66b2	0	0	0	0	0	0	0	0	0	0	9	0
1437637ca2f943f4c8e0f21dbe660315	0	0	0	0	4	0	0	0	0	0	0	0
0232217d5698b8d005331e8bd4da0531	0	0	0	0	0	0	0	3	0	0	0	0
d3f25f2ab7f9cae697efb8df0769862f	0	0	0	0	0	92	0	0	0	0	0	0
3db43f39230c74858f9a09a949d36950	0	0	0	0	0	0	0	0	0	0	3	0
1ae8608b3d2acd7deb6f3494c4120d46	0	0	10	0	0	0	0	0	0	0	0	0
697cc82b06f990700f7d863ac87130e2	0	0	0	5	0	0	0	0	0	0	0	0

1c780c2faf42870889a21f5f0979199d	2	0	0	0	0	0	0	0	0	0	0	0
f2746e781c1991913dc00689e59dd388	0	0	0	0	0	3	0	0	0	0	0	0
8a5d6780afb9f42ffd1552438eaef5c4	0	0	28	0	0	0	0	0	0	0	0	0
7d767854746ba5054db82714647ba9e8	0	0	0	0	0	0	7	0	0	0	0	0
8812cd551ad6bf7f1696d4a715313d7a	0	0	0	0	15	0	0	0	0	0	0	0
54a2b3ec81587838662b717fce90bcad	0	0	0	0	0	6	0	0	0	0	0	0
59f3d415d20f0e5a99a0451ee8083026	0	0	0	0	0	0	0	0	0	0	0	94
a1e86015c485473ee40cd8646a0283f2	0	0	0	0	32	0	0	0	0	0	0	0
99c01d1ad2e1ccc95a698cdc1bfb4f21	0	0	0	0	0	7	0	0	0	0	0	0
d47f4a4c87a2e9f484ac72431a204d36	2	0	0	0	0	0	0	0	0	0	0	0
77fd47a10a867678e73462b8ae2814cf	0	0	6	0	0	0	0	0	0	0	0	0
c3517713718164fb611cc00d25c69697	0	0	0	0	2	0	0	0	0	0	0	0
f7f8b0db0b5b3393d0d9118d10fa6da6	0	0	0	0	0	4	0	0	0	0	0	0
0c701c18f8b65f99c885ddf59a71ca8c	0	0	0	0	3	0	0	0	0	0	0	0

2b0e097fea66b64879651d3d32fc1c06	0	0	0	0	0	0	0	0	5	0	0	0
8c870b47cc9976e3b40953aa5e57f91a	0	0	0	0	14	0	0	0	0	0	0	0
0761505e9387d07f177645a35754f214	2	0	0	0	0	0	0	0	0	0	0	0
fbefb973f17c6f20df5a13a282758405	0	0	0	0	0	0	0	0	0	0	0	17
52c017520c4c7e78817aa7c5ffaba73c	0	3	0	0	0	0	0	0	0	0	0	0
536b666843a23e3842bdbf1a9eed5ee1	0	0	0	0	101	0	0	0	0	0	0	0
42e8e8a1e70d6c5c6251279418b43231	0	116	0	0	0	0	0	0	0	0	0	0
23864945629826b06a841cd6abde6f6e	0	0	0	0	0	0	0	0	0	0	2	0
fe7da90f48ef871960380f9c335f3b96	0	0	0	0	0	10	0	0	0	0	0	0
119a830328863ed63cf277cd3b9c69f5	0	0	0	0	0	0	0	0	0	0	9	0
366d73ea857c8c4d41950b8c3380b322	4	0	0	0	0	0	0	0	0	0	0	0
ce2883ae36f1bbebbcdc984826491b55	3	0	0	0	0	0	0	0	0	0	0	0
90b5c185fc3d28ee0f114549f1f858f1	0	0	0	0	0	0	0	0	0	0	0	9
25862918b3a038f644def26030c1ddfe	0	0	0	0	0	0	0	0	0	0	3	0

4492c57ebe05128bb1cee08 52d526161	0	0	0	0	0	13	0	0	0	0	0	0
aa9cdfa1f2076c6d1e730138 44a86e52	0	0	0	0	0	125	0	0	0	0	0	0
a5ee826f2cab2838f3c5f8bbf b2b625c	0	0	0	0	0	2	0	0	0	0	0	0
aad97db0a321a7bf5b40cd97 9c1317aa	0	0	0	0	0	0	0	0	0	0	8	0
9d1a401b5d17b6eb2a60e8b 2a83816ab	0	0	0	0	0	0	0	0	0	0	5	0
66b47e9c5eb8c3d9d1cb24a4 03d19bbe	0	0	0	0	0	0	2	0	0	0	0	0
5f882d0320d000f2d2d57607 198815f0	0	0	0	0	0	50	0	0	0	0	0	0
964fa24a0a89ff4275d6ef62f 4f1cf41	0	0	0	0	0	0	0	0	0	0	0	19
fae077531abad8c3cce33fe46 080cb79	0	0	0	0	0	0	0	0	0	25	0	0
351b701576bccf62c5559111 657ca05d	0	0	0	0	0	0	0	0	0	0	0	20
89aab664e8fa4e806fe5461d d8dc63ff	0	0	0	0	0	0	0	0	0	0	3	0
d5e38c978fd8acd9dae91822 4ec6ba6c	0	0	0	0	0	0	0	0	0	0	14	0
6607175b8b6be8d3a2acbdc d1c17b846	0	0	0	0	0	0	0	4	0	0	0	0
9638cf3955d634057e8318cb aa0e2425	0	0	8	0	0	0	0	0	0	0	0	0

1f9849d5f0e03c3de0b1c097 31d6eaec	0	0	0	0	0	11	0	0	0	0	0	0
62c25b0452d75a5111ad124 419228e08	2	0	0	0	0	0	0	0	0	0	0	0
f824854465e2f81992384b02 74856191	0	0	0	0	0	0	0	0	0	0	3	0
b0c0646bf0be75712a72b97c 3ee97f24	0	0	0	0	0	0	0	0	0	0	3	0
6b335c095637cfdc340cfcb7c d4ffc86	0	0	0	0	0	4	0	0	0	0	0	0
5a8e1c41abec11a56a4bc5bc e134fd17	0	0	0	0	0	0	0	0	0	0	0	4
9855d524f7586024fa85db43 562a9ff8	0	0	0	0	0	0	0	0	0	77	0	0
be1b06fc8737c901969f59e9f f87eb9b	0	0	0	0	0	0	0	0	0	0	0	3
947a9fb12e3a3d72586f3a55 56df8b68	0	0	3	0	0	0	0	0	0	0	0	0
4ef1e96eaa25006deea2f9a1f e3fe146	0	0	0	0	0	0	0	0	0	0	15	0
beda2b19c7674f7b19bd8da0 1deb5b3f	0	0	0	0	15	0	0	0	0	0	0	0
36b71d5852712c64b49a191 4279e255a	0	12	0	0	0	0	0	0	0	0	0	0
969bbc9604d35ce2fd3bb872 573cbcd7	0	0	0	140	0	0	0	0	0	0	0	0
f75d9c6dc328086298f10398 3154f4dc	0	0	0	0	0	0	0	0	0	0	0	6

e0471d8c3a443388df8d311f6917383e	0	0	0	65	0	0	0	0	0	0	0	0
2baa5f467adc06c7891a3e47f77c7fbd	0	0	11	0	0	0	0	0	0	0	0	0
6d33e078264fadd52a328e5af111b4c6	0	0	0	0	0	0	0	90	0	0	0	0
94fd9739909155e64959747e94051ac2	5	0	0	0	0	0	0	0	0	0	0	0
616b07c7c8764309b6171f75d59ada61	0	0	0	0	0	0	4	0	0	0	0	0
1b1d2ebd48b6c460b479fb3b6906f776	0	0	0	0	0	0	0	54	0	0	0	0
2ab2fba701ee61a573adc801b3e3c8e4	0	0	0	0	0	0	0	0	0	0	0	2
e3ee3c4037ba91dd1ca5a661941fb246	0	0	0	2	0	0	0	0	0	0	0	0
a42362a1feb5f280fbc50702883424d3	0	0	0	0	0	0	0	0	0	0	6	0
5fb436f1d65f62bac13f678bfff16e60	9	0	0	0	0	0	0	0	0	0	0	0
ae92b20c6ed2ee8eeb32c4da8cfe5d3a	0	0	283	0	411	0	118	0	0	431	0	0
bab66457777260322a1f86c12af338ec	0	0	0	0	0	0	0	0	0	0	0	3
1e0c586a2aeb8669c8088389579fe841	0	0	0	0	0	0	0	0	0	0	6	0
fe1b6bcc71ee42282fcd395cacddad79	0	0	0	0	0	0	0	0	0	0	3	0

e4ca3f6c631e32852054a7fd14fc26fc	0	0	0	0	0	0	0	0	0	0	0	2
9333dababae8c9ab6d522605dc00940a	17	0	0	0	0	0	0	0	0	0	0	0
2f549899730c172d9d94f0254545c2bf	0	0	0	0	0	0	0	0	0	0	4	0
f4a1ac879b08169f6be372e9915704df	0	0	0	0	0	0	0	0	0	0	0	2
31f4578e3c2f91cab048d9b8930aa82f	20	0	0	0	0	0	0	0	0	0	0	0
b7fe7541b9ea64c14e69421072eb3606	0	0	0	0	0	0	0	0	0	0	0	5
a017173f43af4c68f51a6b98853b117e	0	0	0	0	0	0	0	3	0	0	0	0
90b4c5aa2b38b823484a56bc2598c981	14	0	0	0	0	0	0	0	0	0	0	0
ceca3c5dc49d3d1aec3bb4e80a2d2914	0	0	0	0	0	0	0	0	0	0	7	0
7978cc075441e6b84e102a2f3a1f2027	0	0	0	0	0	0	0	2	0	0	0	0
cf4788fc0a5bdee8f53a3b394d40643f	0	0	0	0	0	0	0	0	0	6	0	0
2af071eef3c7ccd3d67c45bceef0f629	0	0	0	4	0	0	0	0	0	0	0	0
2fdf049758ec3e706b8e47840f7ed032	0	84	0	0	0	0	0	0	0	0	0	0
d29b9a53d91e90aa4d0be0f2c4910cec	0	0	0	5	0	0	0	0	0	0	0	0

75f83497f746a5f93c99c6187e3152d1	0	0	0	0	0	2	0	0	0	0	0	0
5b4404214017e8815c87989ee5715a9d	0	0	0	0	0	0	0	0	0	0	0	289
add1c703eaf52e592c189329d2574a6e	0	4	0	0	0	0	0	0	0	0	0	0
b88bd01baa7b1d11392c687e84ebc0d4	0	0	0	0	0	6	0	0	0	0	0	0
3d034b8d1ec6c0006a271cb99cb22824	0	0	0	0	0	0	0	0	0	0	2	0
013ad56bded278a6d3c41653a78e0cae	0	0	2	0	0	0	0	0	0	0	0	0
59b6ea035e0a74763d0bbca6523406b5	0	0	0	0	0	0	0	0	64	0	0	0
b0e9b83fbcd24dec989981911084f496	0	0	0	0	0	2	0	0	0	0	0	0
4e24b4253d8e725cb8f2a14250d9621f	0	0	0	0	0	0	0	5	0	0	0	0
d6d0e44300a78a43abda117d414d8a07	0	0	0	0	0	0	0	0	50	0	0	0
5545b61d867b30d9d2cf9b1cfbe1cb9b	0	0	0	0	0	10	0	0	0	0	0	0
e8bc06d9397be42f61fde3725ee062cc	0	0	0	0	0	0	0	0	0	59	0	0
0799388c55b768d2a4ba3e7d09ffb0ec	0	0	0	0	4	0	0	0	0	0	0	0
ef5b74efd4246fdd131c9a9390c968ba	8	0	0	0	0	0	0	0	0	0	0	0

769184f3a197dec651fb1b12 0a0f2ba0	0	0	0	0	0	0	0	0	0	0	9	0
c735b4b5a3f6bdfeec133657 2953d643	0	0	4	0	0	0	0	0	0	0	0	0
74d9338e1f37e3f94bd4244e a2691ad7	6	0	0	0	0	0	0	4	0	0	0	0
d26134034c698e90cfec79e4 3347418d	0	0	0	0	13	0	0	0	0	0	0	0
9922f6780840839389f9f778 75f4d6de	0	0	0	0	0	0	6	0	0	0	0	0
992b51248d72b1357b2f983 10164ca90	0	0	0	0	0	0	0	4	0	0	0	0
819b143c4c9272c05d90584a 26dfd2de	7	0	0	0	0	0	0	0	0	0	0	0
ea1760f4c5f6f6874eeb6807f 546ccb6	2	0	0	0	0	0	0	0	0	0	0	0
b44f353bca41611e6f8af4a7d ed92dd6	0	142	0	157	157	0	0	0	0	317	344	343
00b64025f3580c5926a3bb1e 8ddf3a9f	0	0	0	9	0	0	0	0	0	0	0	0
486154fbe57393a759032973 332db831	0	0	0	0	0	0	0	3	0	0	0	0
fabc71d715c38f2524e089e5f 46ce383	0	58	0	0	0	0	0	0	0	0	0	0
d7cf9793435a6c32499f4b27 b9fa795a	0	0	0	4	0	0	0	0	0	0	0	0
a4c1b03ca81f7dba5d2a3d9f 4a195193	0	0	0	0	0	26	0	0	0	0	0	0

d96a4c5070f5f997db69571559f36330	0	0	0	0	0	0	0	0	0	0	0	78
a953fe0f99fc21f7b6548ce64bdea930	0	0	0	0	0	0	0	0	2	0	0	0
22c048d5ecc751d610692ff2b0a6f018	0	0	0	0	0	0	0	0	0	0	0	3
89ee449085e91612f6e3c2e2a54d5b72	0	0	0	0	0	0	0	0	0	0	0	4
4eba42f388f362bd7693a7f2448b4d30	0	0	0	0	0	0	0	0	0	0	25	0
066b23434cb0abf4b6a27be40e6e17c2	0	0	0	0	0	0	0	0	25	0	165	48
dbe8c0fcf5f663a6738d49643d7cb96d	0	0	0	0	0	4	0	0	0	0	0	0
e9049c8954fddb866a2bc391af7e45b4	3	0	0	0	0	0	0	0	0	0	0	0
8595d20dc0f00de677e021a2ad64d5e8	0	0	0	0	0	0	0	0	0	7	0	0
b881bf01ccce9a249851762c34f5f7b3	0	0	4	0	0	0	0	0	0	0	0	0
edf1a0221db7444631311bbd87e38c26	0	0	0	8	0	0	0	0	0	0	0	0
58e3339d99867d7e942a1c924d852cab	0	0	0	3	0	0	0	0	0	0	0	0
cd4fe95cf6241d4a358c37ca15953d94	0	7	0	0	0	0	0	0	0	0	0	0
f67bd74c234c960cac533dae110cb2e4	0	0	0	20	0	0	0	0	0	0	0	0

178dbfcb5afa63a7d52b07b2c8a25e20	0	0	0	0	0	0	0	3	0	0	0	0
d0aca8635b99356fd1b9f860095aa1f1	0	0	0	4	0	0	0	0	0	0	0	0
72db30c0d6224e4367c31347b563f846	0	0	0	0	0	0	0	0	3	0	0	0
efe5f3dd90dcd3ca9280d61748be501f	0	0	0	0	0	0	4	0	0	0	0	0
1a396b04e87aa943b1ea83ac75a8b1a8	0	0	0	0	0	0	0	6	0	0	0	0
caef2b3e4619e8322aa3d1e8f653799d	0	0	0	0	0	0	2	0	0	0	0	0
c65868ca2192a906e4ae83148a34045e	2	0	0	0	0	0	0	0	0	0	0	0
d02fa2e986abb6534c60edea858134a1	0	0	0	0	0	0	0	0	0	0	11	0
bfce6f2a6c619af94f6070c77eaef60e	0	0	0	3	0	0	0	0	0	0	0	0
300f317b65b309d0cc88332a71dbe3ec	0	0	0	0	0	0	0	0	0	0	5	0
79b93ff2934b6954b2d1e51f5462ec10	0	0	0	0	0	0	0	0	0	107	0	0
144c5ddec2f562e77b16715af018d5d9	0	0	0	0	0	5	0	0	0	0	0	0
e7c5affcc7c1a5242fbd1f9e6b6ff674	0	0	0	0	0	0	0	0	0	0	0	28
2188d1c0debf20adf20f6763f7289d49	0	0	0	0	0	0	0	5	0	0	0	0

a63094d9e29088287e627fd1cfca7afa	0	0	0	0	0	0	0	0	0	0	0	4
193a8ea0419e4657b2c9a8a668682c5d	0	0	5	0	0	0	0	0	0	0	0	0
e362573f1ff4174ad46e06ce073d2d3e	0	0	0	0	0	0	0	0	0	0	14	0
7959835adfef8e624f462d1949b6c9c5	0	0	3	0	0	0	0	0	0	0	0	0
7abf3145b54dd980bdd003ee9354ad22	0	0	0	0	0	0	0	0	0	178	0	0
8af23090b9532cf8e50abd80ca39e0f8	0	0	0	0	0	8	0	0	0	0	0	0
6ce2399d25f3b8a38d5fc4a4c62e0499	0	0	45	0	0	0	0	0	0	0	0	0
105c13268e13f6ad0d49607f7ca5042d	0	0	3	0	0	0	0	0	0	0	0	0
e55e83bdb7ec230e26e616372f426cbf	0	0	0	0	0	2	0	0	0	0	0	0
49a5356a29607ef62ff84356bc2c3797	0	0	0	0	0	0	0	0	0	0	0	5
a9c3db51a3fc102d870d7d55d0cc8e36	3	0	0	0	0	0	0	0	0	0	0	0
8ad796c90f2702103007e5b1770a31fa	0	0	0	0	0	0	0	0	0	0	20	0
d74650dd78a0e5848f3a4f089cbda5d4	0	0	0	0	0	3	0	0	0	0	0	0
e4bf10f8e10b7ab012e7227d4b83ac89	0	0	0	0	0	0	0	0	0	0	14	0

7afc2343e19379458aa26791 3b86fd86	0	0	0	0	0	0	2	0	0	0	0	0
ad4f742dbe47beac09920151 01a87d4d	0	0	0	0	0	6	0	0	0	0	0	0
6aa171bf40bce328fed090e2 8e1b8817	0	0	0	0	5	0	0	0	0	0	0	0
15462e3146f4ee35f87c1c53 0a155111	0	0	0	0	0	0	0	6	0	0	0	0
5803e69ac31fc515c8b85964 82c76301	0	0	0	0	0	0	0	0	0	0	4	0
7bee0d2140a8afca0c629a0d 2d86c6d0	0	0	0	0	0	0	0	0	0	0	3	0
e719ac320afa6e30211ac3da 13843eae	0	0	0	0	0	0	0	0	0	0	9	0
dbe0537a180bb248b29a059 67f619a3f	0	0	0	0	0	0	0	0	0	0	3	0
2d72e204f1a983236c4417c5 834b7d3c	0	0	0	0	111	28	0	0	0	0	0	0
c464db42934e3917c6770ea3 648b8e37	12	0	0	0	0	0	0	0	0	0	0	0
3b31165a0f6768c3f23febd63 e83e98f	2	0	0	0	0	0	0	0	0	0	0	0
a885228ad740a0a8f8763b92 3fa7fb65	0	0	39	0	0	0	0	0	0	0	0	0
fc017e033ab7c9d819a3e331 55e32bd2	0	0	4	0	0	0	0	0	0	0	0	0
f4f6d0ed080b86a995a36fd4 aa53903d	0	0	0	0	0	0	0	16	0	0	0	0

278e5ec033100c15edcad34b78c8263f	0	0	0	0	0	2	0	0	0	0	0	0
33de094b2573de9180844731b9e33c19	0	0	0	0	0	0	0	0	0	0	4	0
9e97a4ade4beecb950263e031770dc7e	0	0	0	0	0	0	0	0	0	2	0	0
5799d40f2727cae43c07873f6a276e28	0	0	0	0	0	0	0	0	0	0	0	2
89ddf9db35c1ac65d37185918878e9b4	0	0	4	0	0	0	0	0	0	0	0	0
7385edd22a32a39eab63c039d4369be4	0	0	4	0	0	0	0	0	0	0	0	0
07dff69ccbea6d0ece126ac1cfa0ec77	0	0	0	0	0	0	0	0	0	0	34	0
a7162c7de6a28c14795df3f014b909f0	0	0	0	0	0	2	0	0	0	0	0	0
160c8b2e35c4eca1550d05c62b1c06df	0	0	0	0	0	0	0	13	0	0	0	0
2c0aa743186fe41b91bd73786a7d8781	0	0	0	0	0	0	0	0	5	0	0	0
20776a7cadab81fb1b74346c880f58b5	0	0	0	0	0	49	0	0	0	0	0	0
1b99ce2a83c80f9aa183789b1d72cdc8	0	0	0	0	0	62	0	0	0	0	0	0
12dbcd3a95e9bc82e977a93949b4b60c	0	0	0	0	0	0	0	0	0	0	16	0
5eb05e7e61ffd9e4b21d160fb36dab35	0	0	0	0	11	0	0	0	0	0	0	0

b9179ccd6298c2768951bd9a51a9fc7f	0	0	0	0	0	0	2	0	0	0	0	0
ce1beaef867e43ad47dfb9aae5834124	0	0	0	0	0	0	0	0	7	0	0	0
d4d9413db5aefa07e8aa30e5f566bbad	0	0	0	0	0	3	0	0	0	0	0	0
ca4a9e9c278e11af756eeaf275777bc5	26	0	0	0	0	0	0	0	0	0	0	0
beb725f4e3e25c3f47e5a4851a633634	0	0	0	0	0	0	0	0	0	0	15	0
5d86cc05c89151d8cd7b0ca32759177b	0	0	4	0	0	0	0	0	0	0	0	0
35b607160e6632344ddc93b898eac2ae	0	0	0	0	29	0	0	0	0	0	35	0
7a66d1a2e2196ecd4d24099ec7f834fd	0	2	0	0	0	0	0	0	0	0	0	0
f8585e2151316051d9cd0ebcf5b8d06f	0	0	0	0	0	0	0	0	0	0	5	0
fd48ac0de9b3686915acc66aac14be92	0	0	0	0	0	0	0	0	0	0	0	4
570384f565aa9c8243811e846fcbcbb7	0	0	0	0	0	19	0	0	0	0	0	0
4b51f167e4fef2e3fdc2928ae4f8868a	0	0	0	0	0	0	0	39	0	0	0	0
629f7b7d9ec35debd97080bfdd3914f8	0	0	0	20	0	0	0	0	0	0	0	0
46d5130fe041b29ac2ee8a0b7e5222ca	0	0	0	0	0	5	0	0	0	0	0	0

904df4973e5c7e4dc658be0713af7d5b	0	0	0	0	0	0	0	0	0	0	95	0
275ac1234be879d017cdfb9c9b85d5dd	0	0	0	0	0	0	0	0	0	0	13	0
74c3c751656a2ee3c09ca796f1ea71c9	0	0	0	0	0	0	0	0	0	0	0	108
d25a4de9b77998800b516ab5beba08df	0	0	21	0	0	0	0	0	0	0	0	0
a87761e0a175d33995ca1259900e6d87	9	0	0	0	0	0	0	0	0	0	0	0
2aaf45c5599cdb37d7fcd9903b9fb7b4	0	0	0	0	0	0	0	0	31	0	0	0
f3213b50c1cfc7a75d522056d5cf1baf	0	0	0	0	0	3	0	0	0	0	0	0
00090e946f64e26251d6b6a29efe1b47	0	0	0	0	21	0	0	0	0	0	0	0
d2190ed15066ea8a1472efc43e6da913	0	0	0	0	0	0	0	4	0	0	0	0
0bc11e0519747cf35238c534d78244b3	0	0	0	0	0	0	0	0	0	0	3	0
c57847f770dafd813ae6a6bfa122c2b7	0	0	0	0	0	0	0	286	0	0	0	0
b9895c4e33028b28a1c13ab354a14d8c	0	0	0	0	0	0	0	0	40	0	0	0
bef3fd22403cf0d453cdc9c1f7989ef2	0	0	0	0	4	0	0	0	0	0	0	0
52c1d4a21d2f2a08e4a9d8b94c80e0c1	0	0	0	0	0	0	0	0	0	0	0	7

82000bdf8be4071e5c38de8b5b8f3aae	0	0	0	0	7	0	0	0	0	0	0	0
49999e805c94343057351819bb873bc2	0	0	0	0	0	0	0	0	0	0	5	0
f4de427c83a038594c90798b3fb89749	0	0	0	0	0	2	0	0	0	0	0	0
afa25fbae5de04a442ae00d7efdda5cb	0	0	0	0	0	0	0	0	0	0	3	0
1616795dd6b6c1bb9ca2514e239f3d9f	0	0	4	0	0	0	0	0	0	0	0	0
05d87897f47969be140af23dd158c8fa	0	0	0	0	0	9	0	0	0	0	0	0
1239f0e91834ec6771e6a93f37c59c50	0	0	0	0	0	0	0	83	0	0	0	0
993f5ce53c7fb8d25efc11a5e7a609cc	0	0	0	0	0	0	0	0	0	0	11	0
f2f095b4edde2db388ea281aa8a22e00	0	0	0	5	0	0	0	0	0	0	0	0
884ffe05fc56353a4ac296bd0e6df449	0	0	0	0	0	0	0	0	0	0	0	4
63622a56111590a3d5275df09adf0628	0	0	0	15	0	0	0	0	0	76	0	0
cac63409d2cac63d71749d75ad7a16ba	0	0	0	0	0	3	0	0	0	0	0	0
9272d1466e9210970a2d6e7408da5680	0	0	0	0	9	0	0	0	0	0	0	0
b740c369a200be2ce929e3175ae3a5ff	0	0	0	0	0	0	9	0	0	0	0	0

bf0bed94326adb0bf540532f63e9e154	0	0	0	0	0	0	0	0	0	0	0	4
7b0fea43c9d9deda4e3edd8c1ee49d3d	0	0	0	0	0	0	0	0	24	0	0	0
a02cbae51a084c5905adac99cac3b08e	0	0	0	0	0	0	0	0	0	0	17	0
b5d5c9e144e6c5f145056ae1678c0a3c	0	0	0	0	0	0	0	0	0	2	0	0
24961199889709f1a48daea8bf2f3b13	0	0	0	0	0	0	0	0	0	0	2	0
7e97430ce4aa92b1753bde4cae11d025	0	0	0	0	0	0	0	0	0	0	3	0
359999cf878479d3c9e9c3a6b6df5852	0	0	0	0	0	0	0	10	0	0	0	0
22f5ccf3be414d115299e31a106135ec	0	0	0	0	0	0	0	0	0	0	0	11
eb83ee82c9336968459c3085c360a36d	0	0	0	0	0	0	0	32	0	0	0	0
420a05613c5de9fc173ba2686a404642	0	0	0	0	3	0	0	0	0	0	0	0
c850dba05b03b949ee6bf5ed805dc2c3	0	0	0	0	0	15	0	0	0	0	0	0
5e46126eca35c2d218a482d9fd4cfb33	0	0	0	0	0	12	0	0	0	0	0	0
07cba22daceb0689692d1dc70b74686e	0	0	0	12	0	0	0	0	0	0	0	0
636fce499b0724f06ec8b3546962d76c	0	0	0	0	0	0	0	0	5	0	0	0

c15ecdd7d8aedf5b84fb4211ae7f689f	0	0	0	3	0	0	0	0	0	0	0	0
4ab073bcd82028ce39411ba32bc37f01	0	0	0	26	0	0	0	0	0	0	0	0
a61376ef020dba03b0618dfe0088e1e7	0	0	0	0	0	6	0	0	0	0	0	0
adc3a8efb15e02191d592b09313b5814	0	3	0	0	0	0	0	0	0	0	0	0
3620635cf4866e9dc23c35bc1b59c5e7	0	0	0	0	0	29	0	0	0	0	0	0
b23780b43b3f7d23215ad97381c87243	0	0	0	0	0	0	0	0	0	0	70	0
0e748766faa064c535befd770c95ea21	0	12	0	0	0	0	0	0	0	0	0	0
f2eaaf75aee091c6ab17397f6cfb373a	0	3	0	0	0	0	0	0	0	0	0	0
431317f0d5b385c76c4f2c98464d5baf	3	0	0	0	0	0	0	0	0	0	0	0
b595d86a5f3c5bb817eaac5aba616d50	0	0	0	0	0	0	0	0	0	3	0	0
42110f72997041a61d67d401955d4a2f	0	0	0	0	0	24	0	0	0	0	0	0
febc2a957fd5a5a147d71b298a7b6a71	0	0	0	0	0	0	0	0	2	0	0	0
579f52e11eb510f4154068dee63ee09b	0	0	0	0	0	0	0	0	0	0	8	0
3111c6b02a5ec8fbdd4438068d9c6f81	0	0	0	0	0	0	0	0	4	0	0	0

5ae1b3d9645c942c1ca4eb4d6c64267e	0	0	2	0	0	0	0	0	0	0	0	0
72fdfaad9bc0b694ee10423fbad2f5cd	0	0	0	0	0	6	0	0	0	0	0	0
e285a3bec2d798ca4e3c96aed745c648	0	0	0	0	0	0	0	0	0	0	0	52
b7dd266fe1bd8f9c8b1d449a5803d64a	0	0	2	0	0	0	0	0	0	0	0	0
cd8e052fa2e38c74c53c5215d85f366a	9	0	0	0	0	0	0	0	0	0	0	0
a09da7ba1ae4dced691d844e8ffb13a3	0	0	0	0	0	0	0	0	0	0	0	17
24a38587fc4fc92a6b54a482da291006	0	5	0	0	0	0	0	0	0	0	0	0
43d9599963803fe5fd85d466907d733b	0	0	0	0	0	0	0	0	0	0	4	0
7549424fc2cb488828539dbff9b6a4d5	0	0	0	0	0	3	0	0	0	0	0	0
3116807a39fbfdbf29cd31e3442e9bfc	0	0	0	0	0	0	0	0	0	0	0	17
46500c1e46689c59d254bcdd1e260a8c	0	9	0	0	0	0	0	0	0	0	0	0
19fc59b58c83c05d634cd8ac6de6bc82	0	0	0	7	0	0	0	0	0	0	0	0
b84d89badd2872dd6e6164a079b32979	0	0	0	0	0	0	0	0	0	2	0	0
47a034ed0ef542c8eff544df576631c0	0	0	0	34	0	0	0	0	0	0	0	0

782df573d1098546f539639f3a933b55	0	0	0	0	154	0	0	0	0	0	0	0
3bc56d6caf8f7ad93391a35d807165fb	0	0	0	0	0	0	0	11	0	0	0	0
7dd945b913577288d7e7e9046f17c069	0	0	0	0	0	0	0	0	0	0	3	0
4869b39a948e8f9f6e812801e9fbd002	0	0	0	17	0	0	0	0	0	0	0	0
1ceedb918523d80a3746bd2a97f8297a	0	0	0	0	0	0	0	0	0	0	0	3
29dd86a1685d60123b807fcdb90b9393	0	0	0	0	0	0	0	0	0	0	0	5
9b777d8472b777b6d161624e3676310d	0	0	0	0	0	0	0	0	9	0	0	0
0d796c13bd3a766c148d4386e19e5f2c	0	0	0	0	0	0	3	0	0	0	0	0
7b7190d893e087eaf1374cc5cbf3d0de	3	0	0	0	0	0	0	0	0	0	0	0
51200a7b19d980f29c12422e60785e7b	0	0	0	0	0	0	0	0	0	0	4	0
fa9d35c094bfdd91838061465912a71c	0	0	0	0	0	0	0	0	0	0	6	0
59663c9ea6e75873c9221f444051fd96	0	0	0	0	0	0	0	0	0	0	23	0
d79e50c72d6ac3a3140f366a1d71540c	0	0	0	0	0	0	2	0	0	0	0	0
266daff6a1309eb40089413491248dd8	0	0	0	0	0	0	0	0	0	0	3	0

8f856242a202bdf04fa04100 41ea5769	0	0	0	0	0	0	0	0	0	0	3	0
b68669a0b896d6a2d9be1a4f cc663343	0	0	0	0	4	0	0	0	0	0	0	0
390d3e64c7de918fa29a0941 fa020305	0	0	0	0	0	0	0	0	0	6	0	0
37bc4c478f143576a2b93f8b 3cb5bba3	0	0	2	0	0	0	0	0	0	0	0	0
42e13a5686f137c606dc1e3c 0430e668	2	0	0	0	0	0	0	0	0	0	0	0
fd48589a2db3ef11929566a2 541a8514	0	0	0	0	0	0	0	0	0	0	3	0
a8469c44203dd590977b08e e0bc07db4	0	5	0	0	0	0	0	0	0	0	0	0
e68826a30f08517171840dfb 326f8c4e	0	0	0	0	0	4	0	0	0	0	0	0
0b09734227f7093b6184314 227fa2851	0	0	0	0	0	0	0	0	0	0	5	0
15b08d9cf104903c557400c7 11b3f2a1	9	0	0	0	0	0	0	0	0	0	0	0
93cca875551f80d3734f8667 85d8c553	0	0	0	0	0	0	0	0	8	0	0	0
6b02f78499fb5456c6347844 533cae9f	0	32	0	0	0	0	0	0	0	0	0	0
bd202d9d75acaf4c7b897e31 94081d3b	0	0	0	0	0	0	8	0	0	0	0	0
125c898719d16b1067077f12 70fa8a38	0	0	0	44	0	0	0	0	0	0	0	0

b677f53f21ecfc01f64ef11af1148fe2	0	0	0	0	0	0	0	0	0	0	5	0
c09deca3f678b609c9df9cf4e6936d0a	0	0	0	0	0	4	0	0	0	0	0	0
c04d3f55db37970de7b82f764a36a965	0	0	0	38	0	0	0	0	0	0	0	127
751988ab08f0cc7c1c73b0de84231124	0	0	0	0	0	0	0	0	0	0	2	0
cb9a758ee3b5744c954f1aed23915494	0	0	0	0	0	0	0	0	0	0	6	0
8fd0f156b6776df14ea0d1c0caee3136	0	0	0	0	0	0	0	0	0	0	2	0
b643f1b4093383c546a9a27029db90ae	0	15	0	0	0	0	0	0	0	0	0	0
0669285d1938028207188c67b1fb6676	0	0	0	0	0	0	0	0	0	0	0	25
b4e01a88b3102e5b51431e97dd928f27	0	0	0	0	0	0	0	0	0	0	10	0
d85742b2c26296b5c9aeae531f9bb478	0	0	0	0	0	0	0	10	0	0	0	0
1740c753649fe82851ca624c9634edb6	0	0	0	0	0	6	0	0	0	0	0	0
66761570dd07c695ede47aff16ba1c75	0	0	0	0	0	0	0	0	0	5	0	0
1ae5d06cd1eb3d2d0a8d1cbe728de123	0	0	0	0	0	0	0	0	0	0	15	0
2188fcf26ea3d91628de24d6d802c4c3	3	0	0	0	0	0	0	0	0	0	0	0

49118531ad3e9f30811f0768ad193e66	0	0	0	0	0	3	0	0	0	0	0	0
1eb67fd8185b27d86305cfd4304213e3	0	0	0	0	0	0	0	0	8	0	0	0
944e0a6795a0038d8b47ee5206ee15da	0	0	0	0	0	0	0	0	0	0	4	0
68134d320af47a7ab938ac6ddc084eb2	0	0	0	0	0	0	0	4	0	0	0	0
5a6c453b8753ec2bb1ecc857442622f8	0	0	0	0	0	0	0	0	0	0	3	0
a16833f8e5d583161312702da2c98c73	0	0	0	0	0	0	0	0	0	0	89	0
605a5bdf2d73cb962dbeb3b72879c6b9	0	0	0	0	0	4	0	0	0	0	0	0
2fff1d65f26d6d316ac999a4bcbcd90e	0	0	0	3	0	0	0	0	0	0	0	0
8e236d26c563038fd4aeaed96d0a9b16	0	316	0	0	0	345	0	322	0	0	365	0
01ccb0ca7426be79d5e68c8da3cda123	0	0	2	0	0	0	0	0	0	0	0	0
0390155f89db166dbd45e2c0b52b5021	0	0	0	0	0	4	0	0	0	0	0	0
bec1b8a4d8662ef3c5ddbd27a1e1e0c7	0	0	0	0	0	0	0	0	0	0	12	0
9b8171bb6b9c24420f33c85853749569	0	0	0	2	0	0	0	0	0	0	0	0
8ce3add226c4b09e3896651494519182	0	0	2	0	0	0	0	0	0	0	0	0

5402880867ef7d0315351d4bab1f3fbf	0	0	0	0	0	5	0	0	0	0	0	0
d970c82cfde09bdc71ab56e8444eecdc	0	0	0	0	0	0	83	0	0	0	0	0
daa9305a8be05e99dc7fe11875763d11	0	0	0	0	0	0	0	0	0	9	0	0
678bcffb60149c3c865dde5d85f253da	0	0	0	0	0	0	0	0	0	0	0	12
3224d31278ddb4a1c8d082f283837362	0	0	0	0	0	0	0	0	2	0	0	0
a62eb8933cd21a736ca412de51844f0d	0	0	0	0	0	0	0	0	0	0	0	23
41f53883f2c30b4ef643960e7213ea25	0	0	0	0	0	0	0	0	0	2	0	0
510cefad2dd2400d4ace6c80dc936f4e	0	0	0	19	0	0	0	0	0	0	0	0
bf5b5543554cf90e64413700794d8bb3	0	0	0	0	0	0	4	0	0	0	0	0
fac8a37db5d9804037bde275f8d081a5	4	0	0	0	0	0	0	0	0	0	0	0
d80785a6ace99a0d99106d0be9a86aa4	0	0	0	0	0	0	0	46	0	0	0	0
633514ccaeae11a9e817e1bcc94a79e4	0	0	0	0	0	0	0	4	0	0	0	0
054399b8963c21f49b5bc5beb6163207	0	0	0	0	0	0	0	0	0	0	2	0
2ab56fef8e5cad447109d5a191b9a8b7	0	3	0	0	0	0	0	0	0	0	0	0

54b17a27622940f40007711956364248	0	0	0	0	0	0	0	58	0	0	0	0
dc7895d759f51419a2d582f1a4c67c57	0	0	0	0	0	0	0	4	0	0	0	0
f57f9017b9eff51619ee38f25ad274ee	0	5	0	0	0	0	0	0	0	0	0	0
fdd7752c4571f71f6fb51776d45fb309	0	0	0	0	0	0	0	0	0	12	0	0
3d59bcd6ece3681922dcfa6f8e5b07cc	0	0	0	0	0	6	0	0	0	0	0	0
646512460402c46b587d82f028d97a9e	0	0	0	0	0	0	3	0	0	0	0	0
19fadd9ba39f5d0b2c35e26acbf5d24d	0	0	0	0	0	20	0	0	0	0	0	36
828b98d5596c75fd642624f62c0e11e6	0	0	0	0	3	0	0	0	0	0	0	0
31e457b9e548ce39fc2d47dc70c208cb	0	0	0	0	0	0	0	0	68	0	0	0
46a48e277352ba4ee85af96e6ec6a607	0	0	0	0	0	0	15	0	0	0	0	0
1d71b072d3f011df203ee59c98937c49	0	0	0	0	0	0	2	0	0	0	0	0
6d64e9cdbfdba9a73ea680fb773b6562	0	0	0	0	0	0	0	3	0	0	0	0
316bbdc1ebdc0c1fedcf625f4d3e262f	0	0	0	0	41	0	0	0	0	0	73	0
430137a068884606d5238e04a2df1eba	0	0	2	0	0	0	0	0	0	0	0	0

3b04941542f0a791af3032bbd770659d	0	2	0	0	0	0	0	0	0	0	0	0
a8c728ab9bd70b8c9f1586bde653f914	0	0	0	0	21	0	0	0	0	0	0	0
ccae5ca842a3fff8de6b81d6f159b97a	0	0	0	0	0	0	0	0	0	3	0	0
a490a9111dd75074d72601f9439423e9	0	0	0	0	0	0	0	0	0	0	2	0
fd9d2d3d7283b09417ea98ad5990f923	0	0	0	10	0	0	0	0	0	0	0	0
219e02667202156e8ff8beab7951d582	0	0	0	0	0	0	0	0	0	0	0	7
9fd86869a0a2b65e939b74cdf830099c	0	0	0	0	0	0	0	0	0	3	0	0
0f0f46fbca3757e30c4e1e494cd445a0	0	0	0	0	0	0	0	0	0	0	0	5
a1deb23bc8671b86e25a050d30199b5c	0	0	0	0	0	0	0	15	0	0	0	0
ece759ecc582d244d0998be543d50efa	0	0	0	0	0	0	0	35	0	0	0	0
958630fecdc35c75f4f08db0a72e49f5	0	0	0	0	0	0	0	4	0	0	0	0
a56a7dc50b6edc57cf031979adb53305	7	0	0	0	0	0	0	0	0	0	0	0
bafabfe4d1def1baa1f22c2bd3c82a95	0	0	0	0	0	0	0	0	0	4	0	0
9dd6b2a30b7188b428bc7b1185ba0025	0	0	0	0	0	0	0	0	0	0	3	0

43bf5458d3a8ccd9a1da4bd294d3e2d8	0	0	0	0	0	0	20	0	0	0	0	0
740f851071f363270196558e476f225f	0	0	0	0	0	34	0	0	0	0	0	0
327ed97a94a27d2c946dc5b3277f0ddb	0	0	0	0	0	0	0	0	0	0	0	4
8d93e217e5d98c8047a354167f7f2256	0	0	0	0	0	0	0	0	0	0	0	30
87a46e0f6a3b2062278982545d9535c5	0	0	0	0	0	0	0	0	0	0	0	25
6c5bf57cb569662af5a0f9ab4338ea11	2	0	0	0	0	0	0	0	0	0	0	0
72ba3077f6316a0e33d89951e7de7d6d	0	4	0	0	0	0	0	0	0	0	0	0
58c4ff85b4154d5c844b8313dddb55b7	0	0	0	0	0	0	0	0	3	0	0	0
a415c8025c4d6cc83633d30a5c9b7e1d	0	4	0	0	0	0	0	0	0	0	0	0
1fe8cbeb4094f3e3a1931a0c99418413	0	0	0	0	0	0	0	0	0	0	2	0
3d413d4bcab67a88fb677e1dea0100cd	0	0	0	0	0	0	0	0	3	0	0	0
a20ea3df5f52ba682fd5107be423f655	0	0	0	0	0	2	0	0	0	0	0	0
fd44f8f64671bee276ebd88a44ffe62d	0	0	0	0	0	17	0	0	0	0	0	0
4060947575978e4f46e07dddf7a1f067	0	0	0	0	0	0	27	0	0	0	0	0

e6d217c9eaa955139d5c7c94b73ab4ec	0	19	0	0	0	0	0	0	0	0	0	0
174b439cecc06e6e2520853dd871d749	0	0	0	0	0	0	0	0	19	0	0	0
81f3ee52b88d0f47b5e3f25260139787	8	0	0	0	0	0	0	0	0	0	0	0
fbfdbdafbb9b960748d8fca9a3e45ff0	0	0	0	0	0	0	0	0	0	0	0	2
3bf5e18d9a2281dbea034690a8a814f0	0	2	0	0	0	0	0	0	0	0	0	0
af29aa77ee82149cb501d87fc2ec99a1	0	0	0	0	13	0	0	0	0	0	0	0
8a7fe89eba99fcce889ae9b837ce2d08	0	0	0	7	0	0	0	0	0	0	0	0
4c3e860b4ea50fc413f7772f200b50be	0	0	0	0	0	0	0	2	0	0	0	0
e0a93c551ea388d7398c93c746019518	0	0	4	0	0	0	0	0	0	0	0	0
44b9998ca40aec39d7c482be11893189	0	0	0	0	0	0	0	21	0	0	0	0
e3b02e4a1962a3a798eee5c21f7bb802	4	0	0	0	0	0	0	0	0	0	0	0
061bed48d8a6b6ba1e36f78a6a47b302	0	0	2	0	0	0	0	0	0	0	0	0
ae8578991ed137330a62db96e5a2df1f	0	5	0	0	0	0	0	0	0	0	0	0
668a258ae93aa928a3ce02e09e6f251c	0	0	0	0	0	0	0	0	0	31	0	0

6e89bdda837f4c87ee37feb42d2d7cf4	0	0	0	0	0	51	0	0	0	0	0	0
d9ec690c6b69729c06fee059e7cda3ff	0	0	0	0	0	0	6	0	0	0	0	0
25b1e989a1b75543b51acba9f4e24597	0	0	0	0	0	53	0	0	0	0	0	0
12f07cba8feeab785d50585279d5de7e	0	0	0	0	0	0	0	0	0	0	5	0
d997abf5bc8e5a7f434905713a896d88	0	0	0	0	0	0	0	0	3	0	0	0
9a83d447217fa1f52af75165dbc778ef	0	0	0	3	0	0	0	0	0	0	0	0
09b17dde832afbe3a62660ee9bfec84f	0	0	0	0	0	0	0	4	0	0	0	0
18228f6ee0165c3d66943669f22d28df	0	0	0	0	0	0	0	0	0	3	0	0
9313344fb80ef3ed891ae5a451264894	0	0	0	0	0	0	0	0	0	0	21	0
6fb7b95503c624923c96ed77c3e7d1e0	0	0	0	0	0	0	0	0	0	0	14	0
22d0c26d34a5968ad8b6a30badbe1afd	0	0	0	4	0	0	0	0	0	0	0	0
a0ccae812af369308515cec75c68a073	0	0	0	0	0	3	0	0	0	0	0	0
86325e371cb8b3aa1a70bae0587199cc	0	0	0	2	0	0	0	0	0	0	0	0
3c491a916dd05f852308e2ef7595bfd0	0	0	30	0	0	0	0	0	0	0	0	0

94e5fa6401b4abd2224d271e97062fd1	0	0	0	0	0	0	0	0	0	0	2	0
b9c6b9c46d7423773e6b4e04dedcd710	0	0	0	0	0	0	0	0	0	0	0	20
e6b43ab1c0243cec862302c1d75ff947	0	0	0	0	0	0	0	0	0	17	0	0
0effc3165bfc5227ef4e7f9771175770	0	0	0	0	0	0	0	0	0	0	2	0
3cbb0bd73378401f1d2fdffbeb2116a8	0	0	0	0	0	0	0	0	4	0	0	0
58f5673f016a4dcac6e1a25078594f54	0	0	0	0	0	0	0	21	0	0	0	0
0cffbaee79e4606235e53a2894cd2e13	0	0	0	0	0	0	0	0	0	0	12	0
31827ff0c358eb450b3e107fae1c2658	0	0	0	0	0	0	0	0	0	4	0	0
3a4068108068789738ff3081a3729999	0	0	0	0	0	0	0	0	0	0	4	0
c72ab0c2b8f56b7361ccae1dc167b387	0	0	0	0	0	49	0	0	0	0	0	0
c40516764ccf93a70cfdd91de2931960	0	0	0	0	0	3	0	0	0	0	0	0
d25d97cab923b8c9f698f23d4770e683	0	0	0	0	0	0	0	34	0	0	0	0
82fc54e0f3a4ef2048aa3d1c34b230c6	0	0	0	0	0	0	0	0	20	0	0	0
6ddad332ebd9beaa11069d3a0d932966	0	0	0	0	0	0	0	0	0	0	0	3

128d6abbaccb0d03b2af8565ce1d371a	0	0	0	9	0	0	0	0	0	0	0	0
9d03b2ad54bf1e03dfd0d3bdfc283232	0	0	0	0	0	27	0	0	0	0	0	0
eed4cb2df8837d7d3d19bc623f092bfa	0	0	0	0	0	0	0	0	0	19	0	0
1cbd4d1fd17ef0ab9c20f55714a69520	0	0	0	0	0	0	0	2	0	0	0	0
a61145ad9a817784e23ddd3452fa7d9e	0	0	0	0	0	0	2	0	0	0	0	0
f0363b13d260fd7655abf870a2205c0e	0	0	0	0	0	0	0	0	0	0	0	25
f930e6e1cd9654d94f0384b278fb5afd	0	0	0	0	0	4	0	0	0	0	0	0
4e70e0330bb29ebd5aec84745cd56eb5	0	3	0	0	0	0	0	0	0	0	0	0
9384fa41a5767e2fa729a0740f2d370b	0	0	0	14	0	0	0	0	0	0	0	0
d4fc3b0d6a28ccba3e90de6aea14b5bb	0	0	0	0	0	0	0	0	0	0	0	26
9ce59d1a76d3870127676e08cc578f6e	0	0	14	0	31	0	0	0	0	0	0	0
64fc561402ff99893ed955a97a12cd6f	0	0	0	0	0	2	0	0	0	0	0	0
b3bebe7d29a66ea5f8cfa3e2d56b1772	0	0	0	0	0	0	0	0	0	0	25	0
045215ea3689d3e12a33abf22a4faacc	26	0	0	0	0	0	0	0	0	0	0	0

b1725914b9a2529295ff6f1d45dec24b	0	0	0	10	0	0	0	0	0	0	0	0
b20b5577ce7a622aeec7e5ada22a3300	0	0	0	0	0	0	0	0	0	0	59	0
421ea232f670c11f975b3dd862bda410	0	0	0	0	0	0	0	0	0	11	0	0
f5782f5b235a85c9d5ea4f45f821f338	0	0	0	8	0	0	0	0	0	0	0	0
ea95281175f5929ffc3d33026defb2fd	0	0	0	0	0	0	0	0	0	7	0	0
d1c58ae730b2c729dc9831d76739ab65	0	22	0	0	0	0	0	0	0	0	0	0
95f7ddd093f450ea8caf72a277ef3921	0	0	0	0	0	0	0	5	0	0	0	0
f7d076ada7bb327e37fd0ec4dd93a62e	0	0	0	0	0	10	0	0	0	0	0	0
6d5c898cdb7100fd3452909da22d1aec	0	0	0	0	0	3	0	0	0	0	0	0
ee917473d4133f43a55c59b024cff1cc	0	0	0	0	0	0	0	0	0	0	134	0
80b93594c616d98f09479767208deb1a	93	0	0	122	0	0	0	89	0	187	188	232
70b10a98017a09c8e59aaec0224b4d18	0	0	0	0	2	0	0	0	0	0	0	0
16c820e5cde149263c0c36150699f6d0	0	0	0	0	0	0	0	0	0	0	2	0
cfe85dfc853ab5d4efedd1dc864daf01	0	0	0	0	0	0	2	0	0	0	0	0

c466aef195c785f0dc471b268c002827	0	79	0	0	0	0	0	0	0	0	0	0
35a29e6fef762464dc15a4056076fe74	0	0	0	0	0	0	0	0	8	0	0	0
003f7022021d6c6349494cfd257f4777	0	0	0	0	0	0	0	0	0	0	3	0
55090065d2ab46a0b2c0d5fdef3c9fe8	0	70	0	0	0	0	0	0	0	78	0	58
fcf57ab52035ec2d9eb28b4ba69f9074	0	13	0	0	0	0	0	0	0	0	0	0
ebcb713588f6c23c62cd1461d8292aea	0	0	0	0	0	0	0	0	0	0	0	10
ca80282d6eb67b1af8a7a3da6ad570d8	0	0	0	0	0	2	0	0	0	0	0	0
26dd95b9c789c0448a43632b8fdb8596	31	0	0	0	0	0	0	0	0	0	0	0
2cda7c7b0892c9e55af9eee5e9e79342	0	0	0	41	0	0	0	0	0	0	0	0
4ed278a5cc75e8570fbb386c024325f4	0	0	0	0	0	0	0	0	0	0	0	7
7c76d79af6a52761a7e34877be6931b2	0	0	0	0	0	0	13	0	0	0	0	0
2b1444f13ddf311b53886fc3e1d65150	0	0	0	0	0	0	0	0	2	0	0	0
6a10203f23fb39beb6872075736abc10	0	0	0	0	4	0	0	0	0	0	0	0
4e9a226de9822858d728c8a37e154367	0	3	0	0	0	0	0	0	0	0	0	0

d0f291e5233be07c70c27d88932621c8	0	0	0	0	0	0	0	0	0	2	0	0
4b5e701b42702cf784c376043b45946a	0	0	0	0	0	0	0	8	0	0	0	0
d90751bc6f283da91d5954017eec18a8	0	0	0	2	0	0	0	0	0	0	0	0
128ac5c44621414482205e2c1d1b2a64	0	0	0	0	0	0	0	0	0	0	0	2
03c301c5019533c8187087ff5b38f5ff	0	0	0	0	0	0	0	0	45	0	0	0
4b393a086a4bb370970166f896aab0c4	0	0	0	0	0	0	0	0	0	0	13	0
57bf74024eeb066d57ed758a4e8c16aa	0	10	0	0	0	0	0	0	0	0	0	0
2151fa250c6ff0580bfeed960b481dc2	0	0	0	0	0	275	0	0	0	0	81	701
b32d8a457d931ce2e02af638d455e6d0	0	0	0	4	0	0	0	0	0	0	0	0
cd15e58024743988a0b27619d42bd41a	0	0	0	0	0	0	0	0	0	0	0	2
5c1a87dfe40c135a6fcd9db755d667e7	0	0	0	0	0	0	0	0	0	0	0	67
d4a45c76a94458f216ee1bf45d458c87	0	0	0	0	0	2	0	0	0	0	0	0
5467bda714aa12ae6185800f654dbd59	0	0	0	0	0	0	0	0	0	0	15	0
9a26304808eb0c34b5ebe980ba82e8e4	0	0	0	0	0	0	0	0	0	0	3	0

2eb628929f883e02e81ad44e5a630718	0	0	0	0	0	0	0	0	2	0	0	0
74e1863cbd4a364e4ac00147490dcff7	0	0	0	0	0	0	0	0	0	3	0	0
0ed872307ae6c499bc6932907dc6a608	0	0	0	0	0	0	0	2	0	0	0	0
df3a694067bb7741455f5ca8641e069f	0	0	0	0	0	0	0	0	0	3	0	0
d4f21cef558e99416044b5aeffcb2d1b	0	0	0	0	0	0	0	0	0	0	0	7
4bccb73cf03b5555228f7ad200734164	0	0	0	0	0	0	0	0	0	0	0	13
573c14df42d31b50921e5ceee97aed37	0	0	0	0	107	0	0	0	0	0	0	0
7ca1bccf0a303ce978304652a51a2b28	0	17	0	0	0	0	0	0	0	0	0	0
cccc6bf16840a79ffb00a90aee19f96a	0	0	0	3	0	0	0	0	0	0	0	0
247419559e808ced7624e3ea743020a5	141	0	0	0	171	0	0	0	0	0	316	0
60fa519a132b6f13492fe52719ac97af	0	0	0	26	0	0	0	0	0	0	0	0
906b6d22590055e0720e49cc8c052c29	0	0	0	0	0	9	0	0	0	0	0	0
3648ca42ad70e701cb83fc73c4f18f18	0	0	0	0	0	0	0	2	0	0	0	0
d8f0fb236567509741ad755be8429dd7	9	0	0	0	0	0	0	0	0	0	0	0

697f974b6271303dc57f0888891ef9c9	0	0	0	0	0	0	0	0	0	0	0	13
8bf7eda1b640036f45e575dcfbcc0b1b	0	0	0	0	0	0	50	0	0	0	0	0
01f4dd410e89545fc1293dcbd461017f	0	0	0	0	0	0	0	0	2	0	0	0
4236bd62dd8d0d7ddc8766893a326d7c	0	0	0	0	0	0	66	0	0	0	0	0
8d73c632d7f7c91bc27d0a68d3baabac	0	0	0	0	0	0	0	0	0	11	0	0
4dd3af817a1d0c0ebd7ee37163518f75	0	0	0	0	0	0	0	0	0	11	0	0
c9cae78994febfb7d8291f5215a358b4	0	0	0	0	0	0	0	0	0	6	0	0
3e9973ab89f982b81008ea0d3bd3f45a	0	0	0	0	0	0	0	0	0	5	0	0
c170823d85e8d3cbe66577019a991305	0	0	0	0	0	0	0	0	0	0	5	0
56340f807dc9158b6c9402a41519a83f	0	0	0	0	0	0	0	0	0	8	0	0
a635b9ed793b37544a4f7ba7fe338612	0	0	0	3	0	0	0	0	0	0	0	0
de14dfbc3e845d4f5cb607689564315b	0	0	0	0	0	0	0	0	3	0	0	0
1caba7635fec9317ef99ddfd1f0c31b7	0	0	0	0	0	0	0	0	0	0	28	0
6e7b7a92fe23530b7b5e7f403362e3a0	0	0	0	6	0	0	0	0	0	0	0	0

5e69cd87614af4bb0d94b594c7c27825	0	0	0	0	0	0	0	0	0	0	5	0
ea34a940ffccb86e0bcd87377e552d7d	0	0	0	0	0	0	0	0	0	0	4	0
ebe8256e83ac6b286637f3082ba07157	0	0	0	0	0	0	0	0	0	0	3	0
ad4d0994b8048eda9c2025ec407d6515	0	0	0	0	0	0	0	0	68	0	0	0
93a4fc97e488fbb8f21311a1ce1c4956	0	0	0	0	0	0	0	0	0	0	3	0
30c71cb0e06af765f7f8a83594c57933	0	0	0	0	6	0	0	0	0	0	0	0
41030dbcba050d889a591ae4e349ca1d	0	0	0	0	0	0	0	0	0	0	20	0
4830a8974c2131545ec2191f33a6d4e2	0	0	0	0	82	0	0	0	0	0	0	0
cbe5bec5431035d79b8952b4e57392be	0	0	0	0	0	0	0	0	0	0	41	0
06f428ae4eb7fdf8478d9d3ea064ab0e	0	0	0	6	0	0	0	0	0	0	0	0
39eb875a461a385e44ae7f2aefa82efb	0	0	0	0	0	0	0	0	0	0	69	0
4cadf62de4fc617939b91c69e7ecec77	0	0	0	0	0	0	0	0	0	0	39	0
188032354fcf175d5ae13cf560324fcf	0	0	0	0	0	11	0	0	0	0	0	0
bb4ca521b58c36fb25ef85d5474602b6	0	0	0	0	0	0	0	0	0	0	0	3

465ded6445213c2ed95afee18013478a	0	0	0	0	0	0	0	0	0	0	2	0
e409b42eebc90e5f14a4698f881011d9	0	0	0	0	0	50	0	0	0	0	0	0
233429d44f4606fa5d4b92eb803c12d0	0	0	0	99	0	49	0	0	0	0	434	89
29c469fce144d9e6955bab84f162eebc	0	0	0	6	0	0	0	0	0	0	0	0
44bedf4c2264b25376aa0f348847c84e	0	0	0	16	0	0	0	0	0	0	0	0
b066cf0880a179a99cc6abd014472088	0	0	0	0	0	0	6	0	0	0	0	0
bab941d9cb4cc482c9aef7a6fed01d41	0	0	0	0	0	0	0	0	0	0	28	0
8452674db77d574cafbe9a4ff0352c80	0	0	0	0	0	0	0	0	0	0	66	0
7dd6124d276d748e97c6740cd9658331	0	0	0	0	0	0	0	0	12	0	0	0
e21dd47b205170f05adbb37f167d2a74	0	0	0	0	0	0	0	0	0	0	18	0
a53ab4f5701b868e42e71403a912931a	0	0	0	0	0	0	0	2	0	0	0	0
3fab4f4f52e67f262c6909d00af14301	0	0	0	0	0	0	0	0	6	0	0	0
a3063d69b9cdb81093006a4294715157	0	0	0	0	0	0	0	0	5	0	0	0
46056246f677e24801448adeee71ff2c	0	0	0	0	0	0	0	0	0	0	0	7

c496cd843fed88ffa9c67106d188802f	0	0	0	0	0	0	0	0	0	0	3	0
3d4d0856583a036d19ad0bcf4d4858b9	0	0	0	0	0	0	0	0	3	0	0	0
b288cdc53d04156e008e640b9309c70b	0	0	0	143	229	0	0	0	0	179	0	179
5a505a5ed7a7e07255529e27c5e8d922	0	0	0	0	0	0	0	0	0	2	0	0
cb399db9c9439cdcc55f744032007a51	6	0	0	0	0	0	0	0	0	0	0	0
2bfa309d665a3f5298ccb0300eccf9ac	0	0	0	0	0	0	0	0	0	0	0	3
eb14c987cc8c06ba46a08e4dcc066e56	0	0	10	0	0	0	0	0	0	0	0	0
75fe48f2a32e7923d941277f4966a71a	0	0	0	0	0	6	0	0	0	0	0	0
9d726fd9ba5bbf2071b277f02d87571a	0	0	0	0	0	0	0	0	0	0	2	0
74245b78536234b87dc497958dd895d2	0	0	0	0	0	13	0	0	0	0	0	0
0c3dbe2da018202c7e9de7cad756031a	0	0	0	0	0	0	0	0	0	0	0	7
d2ff4f0751bad2f57ab0839c0aa9d514	0	0	0	0	0	0	98	0	0	0	0	0
bc2c1474343f06ec8947c1b41d80e62c	0	0	0	0	10	0	0	0	0	0	0	0
588f4b7aeb5584e7f5d43a9cf9500860	0	0	0	0	2	0	0	0	0	0	0	0

a04c9a00390d1e497db5582cdb808933	0	0	0	0	0	0	0	0	0	0	29	0
4f8acdce652c1b16be048b74468c2d71	0	0	0	0	0	0	0	0	47	0	0	0
a381cf308913bdceedf46db3b400e784	0	77	0	0	0	0	0	0	0	0	0	0
9aae410c228ee62a8c7af34987a5b5dd	0	0	0	0	0	0	0	3	0	0	0	0
ea9335d85f76bbb2c2f463a6a2e16c2b	0	0	0	0	0	0	2	0	0	0	0	0
308527b3b1215dc5630797008447e8db	0	0	0	0	0	70	0	0	0	0	0	0
c9037de0e08dbffd4c09acfa76986f7a	0	0	0	0	0	0	0	6	0	0	0	0
cf0a8320dcda32cc607fdf1803f02687	0	0	0	0	25	0	0	0	0	0	0	0
54303d6531fffadf9989800a89de6183	0	0	0	0	0	0	0	0	0	31	0	0
b81fe8329626d5949978576c90c41711	5	0	0	0	0	0	0	0	0	0	0	0
6d81bfc53c6fda776ab1aa688a6724bd	0	0	0	0	0	0	0	0	0	0	0	49
622cb87d31541e957cd5481f1b46e59d	0	0	0	10	0	0	0	0	0	0	0	0
0e0c203766a0ef81dc334618114c5dbf	0	0	31	0	0	0	0	0	0	25	0	0
3ef13478e0628318a218a4919740a096	0	0	3	0	0	0	0	0	0	0	0	0

e42ebfa5d87a4a3605b25e98ae9d3ba8	0	0	0	0	0	0	0	0	0	0	3	0
59bc50ce21c522a5b8583c3f632f0b6e	0	0	0	0	0	0	0	0	0	2	0	0
584ff152b85fe69f3e11da90ed424728	0	0	0	0	0	0	0	0	0	0	0	4
fa4967a3c05db4a95940eff0c54aee1f	0	0	0	0	0	0	0	0	0	0	3	0
d8e597722311013a85b6efa0c45561b8	0	43	0	0	0	0	0	0	0	0	0	0
b9ed1e42f95a0841f6a59a8fe5094a70	0	0	0	0	0	2	0	0	0	0	0	0
56d8ea818fd62724badd1c0f39fd3aa1	0	0	0	0	0	0	0	0	2	0	0	0
6ed40afab70584ffc082a8ef5131185a	0	0	0	0	0	0	0	0	12	0	0	0
4ef740e1e61dfeb9a4184a32681a992d	0	0	0	0	0	0	0	0	0	0	0	30
ed1497889e0e041cef3a2401ac592779	0	0	0	0	0	0	8	0	0	0	0	0
2b032c6ac700c586bae0228b0bf40fad	0	0	0	0	0	0	0	0	0	0	122	0
f9816c3348d4cb9dbb39739f60f00f92	0	0	12	0	0	0	0	0	0	0	0	0
2e7649647ed3c16331870770de118e52	0	0	0	0	0	0	0	0	0	0	0	6
7c7046176a4651ccbea7f96b10cc40ab	0	0	0	0	0	0	0	0	5	0	0	0

e87dcc152e8ba5e895e68d0d6f799410	0	0	0	0	0	0	0	0	2	0	0	0
20b9a30825cf08f1e4f1f1b5fefe4141	0	0	0	8	0	0	0	0	0	0	0	0
7a32313d5b395481efe8bb1dfaabf203	0	0	0	27	0	0	0	41	0	0	0	0
fa754d93e355d1d7bffa1d5a3a1bb1e4	0	2	0	0	0	0	0	0	0	0	0	0
eac72f6f322168e557f3bb9b8ac5df66	0	0	0	0	0	0	38	0	0	0	0	0
d9fbc2ec92824405826d730d4b0414bc	0	0	0	0	0	2	0	0	0	0	0	0
56322891aec65665c64cbdf73096b076	0	0	0	0	0	0	0	0	0	0	0	17
5b8645b399f09c844e001164dc7c9d15	0	0	0	0	0	0	0	21	0	0	0	0
991e447fc105c7e9357c1e2addf1fe02	0	0	0	0	0	0	0	0	0	0	6	0
cc13368913a9bb7037f66c7b05330084	0	0	0	0	0	0	0	0	0	0	0	6
21aff233f2fb09e04e58506fcc6befbd	0	0	0	0	0	0	0	0	3	0	0	0
d925df95ae08651fd755cfa7afa05353	61	0	0	0	0	0	0	0	0	0	0	0
5fd46b99f017e7740f175345dc07166e	0	0	0	0	0	0	0	2	0	0	0	0
1682df92e11427ee8d4da5790871f486	0	0	0	0	0	0	0	0	0	0	12	0

4edbcaed738d3e7894e0f9216ec89212	0	0	0	0	0	0	0	0	0	2	0	0
4d5e4bcda1fd8a118b1ddcb3a5511fe5	0	0	0	0	0	0	0	0	0	0	0	2
f9f19eeedf1d08372923c99b60ea9f66	0	0	0	0	2	0	0	0	0	0	0	0
7d72ba50827acaae0e8dd70042860cfc	0	0	0	0	0	0	0	0	0	0	58	0
b265b2823127985a43097e5880c54fd4	0	0	0	0	0	0	0	0	0	0	3	0
21c15c2b50820c4ee585bcb2de31369a	0	0	0	0	0	0	0	0	0	76	0	0
f5fcb8701975957525901e7923a957c7	0	0	0	0	0	0	0	0	0	0	4	0
402d2803441f813d6593aaab9a1d6b5a	0	0	0	0	0	0	2	0	0	0	0	0
3d5db3cc20d8cc49f2c3222376bc8bc2	0	0	0	0	0	0	5	0	0	0	0	0
b1bb0a006002c0f2c546b5aa174915e2	0	0	0	0	0	166	0	0	0	0	0	0
2d8f5a4bff6da595f9995c7a345531c6	0	0	0	0	0	0	0	0	0	11	0	0
b9b7882bee17ae106dc72e0d6fdc5eff	0	0	0	0	0	0	0	0	18	0	0	0
eb054cea389892335f7302780f79a9f6	0	0	0	0	0	0	0	0	0	0	2	0
41b4392a8c5aa7f6e5d09bcc4d9bef60	0	0	5	0	0	0	0	0	0	0	0	0

60981d918dc8cd300af02e0f8cedd217	0	0	0	0	0	2	0	0	0	0	0	0
312e19a021b1d4cb4ae72780e471fd49	0	0	0	0	0	0	0	0	0	0	0	12
81a7bb8ce497d0b4d313e556f09629ca	0	0	0	0	0	0	0	0	0	0	12	0
42dcd45e0fda5ba1208adff682366652	0	0	0	0	0	8	0	0	0	0	0	0
70a28eec2649deb3a6f7e773c84f9f04	0	0	0	0	0	0	0	0	0	0	0	5
817e56ab4cf344ad9ab13d887b889447	0	0	0	0	0	0	0	0	9	0	0	0
3ff30e8e3a23837f07c0d6698595d9d0	0	0	0	0	0	0	0	0	0	84	0	0
a28c04efcb6d102382fe077748f9b0dd	0	0	0	0	0	0	0	0	0	0	0	33
30c816b2100d480cfe999f7ba646f232	0	38	0	0	0	0	0	0	0	0	0	0
26a7663e204d6381cdab8bd6a13e16dd	0	0	0	0	0	0	5	0	0	0	0	0
e96ecadfb6142eacfd70dc8e206a6199	0	0	0	0	3	0	0	0	0	0	0	0
d063a77713478af8781b901efb29157a	0	0	0	0	0	0	0	0	5	0	0	0
2bfa94ce3c2a2ac85650017ff4099ba1	0	0	0	0	0	0	0	0	3	0	0	0
327d18cb4e25050d2d25ad27def46f41	0	0	0	0	0	5	0	0	0	0	0	0

8677500427344dd242cc731 1337e65a9	0	0	0	0	0	117	0	0	0	0	0	0
dd8abba0a3fc47784443176f 4f00e11d	0	0	0	0	0	0	3	0	0	0	0	0
25745acebc365b031fd1b304 e3e9f28f	0	0	0	0	3	0	0	0	0	0	0	0
a8be4286a9dbfdb6240dcf6a 25ff7c4a	0	0	0	0	0	0	0	0	17	0	0	0
2cc3fd917a526b898c742ca3 9200de29	0	0	0	0	0	0	0	5	0	0	0	0
66f5665a534ac0e6de00c6d6 39a8a581	0	0	0	0	0	0	0	3	0	0	0	0
4977f37f39226adf4a7ec8d4e dcd27f1	0	0	2	0	0	0	0	0	0	0	0	0
e6ef06dae7841eb5bd47ee7b 1e11b12a	0	0	0	0	0	0	0	0	0	0	12	0
7d94ae79ee86f20d5b1186a0 9f543893	0	16	0	0	0	0	0	0	0	0	0	0
a7ca4ca4ec2ecfab010de00d 1c28dc18	0	0	0	3	0	0	0	0	0	0	0	0
0de0804604cdfa4460ad9a95 07de6fa8	0	0	0	35	0	0	0	0	0	0	0	0
6ed5aeff70b8fcb652bb00848 a02a2c1	0	0	0	0	0	0	0	0	0	0	0	6
85af887a1252096aee1d2a97 0e7b3539	0	0	0	0	0	0	0	0	0	0	2	0
8737af38d6df542c865f90d7a 319ad41	0	0	0	0	0	0	0	2	0	0	0	0

c388e3946f427b70f1792a4f45392a01	3	0	0	0	0	0	0	0	0	0	0	0
6e75885a73da348147da3555fd2814d6	4	0	0	0	0	0	0	0	0	0	0	0
542003e769de33f57a766cb08facc1d7	0	0	0	0	0	0	0	0	0	0	0	19
4224bffa74aad46bda1fde7b65ee15cc	0	0	0	0	0	0	0	0	0	0	14	0
780424488fb94fe77a33bee4cf4eb77a	0	0	0	4	0	0	0	0	0	0	0	0
10e8207abbe5b44c5ea9ae9614f224a8	0	0	0	0	0	0	2	0	0	0	0	0
94e9e7803d9a283c8522fb1b02e17b1b	0	0	0	0	0	0	0	0	0	0	0	2
8d5d309da12da2da696f480aeab7e4c1	4	0	0	0	0	0	0	0	0	0	0	0
3a9b1ab23c3556433fe9d682cd2cb0eb	0	0	0	0	0	3	0	0	0	0	0	0
e8b08d522a36ae9916c357971fac17dd	0	0	0	0	0	0	0	6	0	0	0	0
89489382de515223a4d399dd2b52cddc	0	0	0	0	4	0	0	0	0	0	0	0
b77b4908966ec1ae83e4eedb34056cfc	0	0	106	0	0	0	0	0	0	0	0	0
9404f758bb2266bfed717d5536d43826	0	0	0	0	0	0	0	0	0	0	15	0
64d9096b47a7970e8ac1132c13424d72	0	0	0	0	0	0	0	0	0	0	4	0

13d3940f71408e27737935fa3fd6e5a2	0	0	0	0	0	0	0	0	0	7	0	0
8727022fa77c8e56abd5b1f72ded6ba0	0	0	0	0	0	0	0	0	0	0	2	0
5e13b5d3a950bfe69eccb630e555e314	0	0	0	0	0	0	0	0	0	0	0	10
4f95606b95a8a80e1ba3c86068480e36	0	0	0	3	0	0	0	0	0	0	0	0
c6e42d08c188051c248b3057992e54c9	0	0	0	0	0	0	3	0	0	0	0	0
436eaddd267140eec8f0cee5a36ccea1	0	0	0	0	0	0	0	12	0	0	0	0
e1ec3e55cb97c5b59ca8133f56d7235c	0	0	0	0	0	0	0	0	0	0	5	0
b25d186e07791129f0ae2da92db4cf61	0	0	0	0	0	0	0	0	0	0	0	2
c48a5bb06baca17e35b1be14cfde30ce	3	0	0	0	0	0	0	0	0	0	0	0
52527ad8f23f20a31fde45e6dcf4778d	31	0	0	0	0	0	0	0	0	0	0	0
86a9b938693fbee52719b4f26bf4a2dd	0	7	0	0	0	0	0	0	0	0	0	0
2b3aa85ceb20cd21638e546709388cb0	0	0	0	0	0	0	0	0	0	0	0	3
8e24f09b75057a2bef4c87fc9881ae7d	0	0	0	0	0	0	9	0	0	0	0	0
30669408b85f083211a0925312623f3c	0	0	0	0	0	0	0	0	0	0	3	0

9df1cbe6cc988944e4d1efeb8e74e5a6	0	0	0	0	0	0	0	0	0	11	0	0
ea463f6fbaa90696d5328936372a467a	0	0	4	0	0	0	0	0	0	0	0	0
ca17d522d34f983763a014292d669687	0	0	0	0	0	0	0	0	0	0	5	0
cc4a789b8ce98342f91cee59226406b1	0	0	0	0	14	0	0	0	0	0	0	0
29074c701356b91c73ff5c219a0f4958	0	0	0	0	0	0	0	0	0	0	32	0
e4524bae595a9d0e4d748f4e10c4684f	0	0	0	0	0	0	11	0	0	0	0	0
bad36bd4b5238300ca9f16f27034b41f	0	0	0	0	0	0	0	0	0	0	17	0
d3c1ee20be2ec5e32439578a394e4b09	0	0	0	0	0	0	0	0	0	0	3	0
a28d252387110ad9fd846b9edcaa8111	0	0	0	8	0	0	0	0	0	0	0	0
5f333660db31d5efa564b04b0aa752eb	0	0	0	2	0	0	0	0	0	0	0	0
92b074e21c1ffaf672d324f79aeb0335	0	0	0	0	0	60	0	0	0	0	0	0
a9db78978f4683130ddd5cada6a6e26d	0	0	0	2	0	0	0	0	0	0	0	0
1d08bdc1d09835b68f5ad2eb09086aa3	15	0	0	0	0	0	0	0	0	0	0	0
161340ec5bf0a7cd7dcd8342c5a0795c	0	0	0	0	0	0	0	0	0	0	2	0

da16e9c05558525cc2481924f1971f3e	0	14	0	0	0	0	0	0	0	0	0	0
7b4d5435a7d8ec6db0e9260074fbff15	0	0	0	0	0	0	66	0	0	0	0	0
a1d47c3869922a6fe42c4cc38f505f20	0	0	0	0	0	0	0	0	0	2	0	0
f9fc0726acbe9a59bd2055ca1a83bf6e	10	0	0	0	0	0	0	0	0	0	0	0
d0e8d1c9c4e3fd8891f5e70c6523f128	0	0	0	0	0	0	0	0	3	0	0	0
a13b616132a383494e48e0e58e6f9084	0	0	0	0	0	0	0	4	0	0	0	0
40fb94d1af14628c5893dc2ab73d41bf	86	0	0	0	0	0	0	0	0	0	0	0
ebdeafb1fca4cb5f72ecdbe8333074cb	0	0	0	0	0	0	0	0	0	35	0	0
fe0b1e2ae1a1acc09d98896fd9d00ede	0	0	0	0	0	0	0	0	0	95	0	0
98989b567fc43fb23e2ef0f79158e71d	0	0	0	0	0	23	0	0	0	0	0	0
3bc62f39d74a71baa0acb5f49ddea173	0	0	0	0	0	0	0	0	0	0	5	0
74e5ce7117a74e3b34870fbea53bb338	0	0	0	0	0	0	0	0	0	0	14	0
4c8a1c2e29a3e03b28a7ea1e89e1c4d2	0	0	0	0	0	0	0	0	0	0	7	0
f997c25f39b34ac12784b6dd9976b937	0	0	0	0	0	0	0	0	0	9	0	0

cc9dbb75a5dbf5dbf52b1f639710a028	0	0	123	0	0	0	0	0	0	0	0	0
8fb396964614d1d7813e01dd311d5212	0	0	0	0	0	0	0	0	0	0	5	0
913d991c3b47494c9f01df154e4bb101	0	0	0	0	0	0	0	0	12	0	0	0
fc5271bad9134ab69a74eca1fbde8386	0	0	0	0	0	0	20	0	0	0	0	0
a7d70b38fd548b8e3e0005ad7af12976	0	0	0	0	0	0	0	0	0	0	8	0
5e937948c3b86011fa4b32e45e9e169e	0	0	0	0	0	0	21	10	0	0	0	0
bb5158e1c8fae4c92614dc2c24488d92	0	0	0	0	0	0	0	15	0	0	0	0
22e26d2841003b68ff2b73f912525822	16	0	0	0	0	0	0	0	0	0	0	0
0d754d3644e06cd9ad1f1f9e4c3ef81d	0	0	0	0	0	0	0	0	0	0	0	8
e2908d99dabf2b5a8c53c13c1af069c6	0	0	0	0	0	0	0	0	3	0	0	0
ced2734635fd2b13f23c8dd1a0c0cc9d	0	0	0	0	25	0	0	0	0	0	0	0
3aff71cecb896e29e4d3d055d8d896f4	46	0	95	0	0	72	0	0	0	0	0	0
a061c2554884e3216853e114fd29c0f0	3	0	0	0	0	0	0	0	0	0	0	0
e235f8b71a60042a8ec72b84611e076a	0	0	0	0	0	0	0	0	0	2	0	0

7fe9c226e3acfeaaca098c65e7b24c29	0	0	0	0	0	0	0	0	0	0	3	0
1c14b287d973b9239279c9b47ec8d6e4	0	0	0	0	0	0	0	0	0	0	9	0
3251b805dab08cb3d70e7a78799e5bb6	0	0	0	0	0	0	7	0	0	0	0	0
c6e6ba3485b48e9e11a571f70980169d	0	0	14	0	0	0	0	0	0	0	0	0
88fd7d0717f89de10e733f8a6fae17d6	0	0	0	0	0	0	0	17	0	0	0	0
fd50f4bf7ae10b26ae00a3f90778130b	0	0	0	0	0	0	0	0	0	0	0	5
5c4d1f2b998f2a57fe32c8ad5d18e9f1	0	0	0	0	0	0	0	0	0	0	0	2
b2a7c3e5ce31f3d2ae972fe711790a36	0	0	0	0	0	0	0	0	0	7	0	0
d4fa2d573a62762ac3e5238ba22152f1	0	0	0	4	0	0	0	0	0	0	0	0
bc7f0a529fc2e70940d557e499b5839e	0	0	0	0	0	0	0	13	0	0	0	0
acba7d1a7d1cbe70c578b3c81a0ae737	0	0	0	0	0	0	0	0	0	36	0	0
d6f9b3cd8e10d2d58aa068e60bace05b	8	0	0	0	0	0	0	0	0	0	0	0
fc7b62451f7774da426855165efc6886	0	0	0	15	0	0	0	0	0	0	0	0
29b914a38c3b61a6b2d0288488426948	0	0	0	0	0	0	0	0	0	0	6	0

818f76520d169de73b9b840becf26195	0	0	0	0	0	0	0	0	0	0	3	0
6e8dc69b6e6f45d9563187b03e7c9d87	0	0	5	0	0	0	0	0	0	0	0	0
a5e872db585656f7b3d15b4a1c4885d2	0	0	0	0	0	0	0	0	0	0	0	63
6f34f5a5610fa3ba117db1bcee9bbfa4	0	0	0	0	0	0	0	0	0	0	3	0
cdd064908598c538249fbcca1fb0fbe3	0	0	0	0	0	0	0	0	0	0	0	11
b61d4d6d532a576736783d3c6940272b	10	0	0	0	0	0	0	0	0	0	0	0
08f8222601fa74a5cade751773d2862f	0	0	0	2	0	0	0	0	0	0	0	0
42b94bdbe1e9edb955a1445f7e465259	0	0	0	0	0	0	0	3	0	0	0	0
14a7d30092579cb62e07f92fc6e71443	0	0	0	0	295	297	0	0	0	0	0	0
91f8f9a37823e2589e8bea5b6d96291e	0	0	0	5	0	0	0	0	0	0	0	0
6aaede4087d6e2c49359d6043aa8b039	0	0	0	0	0	0	0	0	0	0	13	0
585786fe5427097558eb496f2862083f	0	0	0	0	0	0	0	0	0	0	0	9
4e282a26c48764a991f60031c8a2ad53	0	5	0	0	0	0	0	0	0	0	0	0
ba023f5637f694b9f69dc76875262b4c	0	0	0	0	0	0	0	0	3	0	0	0

a53f0cf39ca2f8808469a762f cf23bee	0	0	0	0	0	0	0	0	0	0	0	2
11316e5ed6b749a3f93ff6e6 05e8f8e6	0	0	0	0	0	0	0	0	0	5	0	0
6c5bcdcb866daee35518c5dc 0fd96302	0	0	0	0	0	0	0	0	0	0	0	4
abf1629d921c8bbb90e62754 8f85cb6b	0	0	0	0	0	0	0	0	0	0	4	0
972a7b9d5f76d90ee1dbf137 8632b618	0	0	0	0	0	5	0	0	0	0	0	0
16c03139efe69cf359daaa08 b0a040b1	28	0	0	0	0	0	0	0	0	0	42	0
6d26ea09c4127151c346f4b0 b6a1e30c	0	0	0	0	12	0	0	0	0	0	0	0
dffd8410fd405e1797f6bba00 3de2f77	0	0	0	0	0	2	0	0	0	0	0	0
cf89d4cd01bfce68b80d4b8f7 5b720d6	0	0	0	0	0	0	0	0	0	0	0	2
460242adf8ef83a71ac26fe0a 595ebc1	0	0	0	0	0	0	0	0	0	0	0	3
c1804e6a062920f23044410c d653877b	0	0	0	11	0	0	0	0	0	0	0	0
4eec45c24b4ce4c50b5dbad0 3cae0b3e	0	0	0	0	293	0	0	0	0	287	271	0
7e4682352b2fe660e7170e0f b1e4f883	0	0	0	0	0	0	0	0	0	0	21	0
16ee3480f5b1da6f8e5a874c 1e6c6dc1	0	0	136	0	0	112	0	0	0	0	0	0

0c146231b5ba0a81c56f78cce945e4b1	0	2	0	0	0	0	0	0	0	0	0	0
54342955b697aa11de87f07100d39bf7	0	0	0	0	0	2	0	0	0	0	0	0
6b1838e2c8ed122492639286bd1e684f	34	0	0	0	0	0	0	0	0	0	0	0
97b8174faf1054adb40cb405e4cb6487	0	0	0	0	0	0	0	0	2	0	0	0
a2bf8aae2bfc822cec919f3ae1fd7b74	0	0	0	0	0	0	0	0	0	3	0	0
772daafbd4b665ff39dec316dc00f52b	0	0	0	0	0	0	0	0	2	0	0	0
5876d4c1f7da7705df8ff3ba4d578227	0	0	0	0	0	5	0	0	0	0	0	0
c406d6725d0139b1f7be6471fe0298d4	0	0	0	0	0	0	0	4	0	0	0	0
79d8c0fb2e315fc8b885d480760365e2	0	0	0	0	0	4	0	0	0	0	0	0
bbe157066cc90af6ead11ea88a4a259d	0	0	0	0	0	0	0	0	0	0	3	0
ca6f6c27ade488c145d63cf509bac5d7	0	0	0	0	0	0	0	0	0	0	0	2
6843ffb696d2e4aaa2bbc12884f643a4	0	0	0	0	0	0	0	283	0	0	0	0
4e0584fc6af644654cf7571b9dff260c	0	0	0	0	0	0	57	0	0	0	0	0
182d4ad49c895075f5bed8927bbde89b	0	0	0	0	0	0	0	0	0	0	8	0

5cef9f0e8a43303701545f690917264c	4	0	0	0	0	0	0	0	0	0	0	0
0199c5b019e603fda344828e236aa388	0	0	3	0	0	0	0	0	0	0	0	0
d01143e8fb0198e5344d24b093bebf4d	0	0	0	17	0	0	0	0	0	0	0	0
09234551405791d0bc9d83dd01154397	0	0	0	0	0	2	0	0	0	0	0	0
c57733ec40be6cc32244fdb054c37013	0	0	0	0	0	0	0	0	0	0	4	0
6a332faa12e6414f63a387ffd7df517d	0	0	0	0	0	0	0	0	0	89	0	0
5f43d2377fb89c10e95c366dcd694397	0	0	0	0	0	0	0	0	0	2	0	0
1a03c74dadd797c5ccd4d7c681f96feb	0	0	2	0	0	0	0	0	0	0	0	0
5f5513a6cbfc5da35af93b518f6fe33e	2	0	0	0	0	0	0	0	0	0	0	0
b31186680be0acef0bc5436e6c83052b	0	0	0	0	0	0	0	0	0	3	0	0
f21786cac8f8192c592c7acb019431ff	0	0	0	0	0	4	0	0	0	0	0	0
b6fa78e9a33bd6b7ed75bf1ddb7b2492	0	0	0	2	0	0	0	0	0	0	0	0
68fe300b27d55a2bf35d1ea290fed8a2	0	0	0	9	0	0	0	0	0	0	0	0
81c2e19335afb18334e7a2a925852c55	0	0	0	0	0	0	0	73	0	0	0	0

ec966cb72f2c2eae45c0f11b5aa107eb	0	0	0	0	0	0	0	0	0	0	0	27
9b9d3e2762160aef33050797ffb01afb	0	0	0	0	0	0	0	0	0	0	2	0
7b46e5005e01b79b98460d6e0e8e4ec6	2	0	0	0	0	0	0	0	0	0	0	0
d16279d280c33653d379e36e8d638fd2	0	0	0	0	3	0	0	0	0	0	0	0
4df60a5056a947fdd6609ded164355c7	0	0	0	0	0	7	0	0	0	0	0	0
9789be201caafb5c99e1ed4346172f87	0	2	0	0	0	0	0	0	0	0	0	0
e8ec3fc238d43b353e7655f6c3a4082f	0	0	0	0	0	0	0	4	0	0	0	0
c3346a8894d0783f3ff503feb830d737	0	0	3	0	0	0	0	0	0	0	0	0
f0b656f5c8416e1c5c5d8e96301574ac	0	0	0	0	0	0	0	5	0	0	0	0
46e6a2271f4399bf10e0bcd065a8db1a	0	0	0	0	0	21	0	0	0	0	0	0
f5a7f01a046d03db843decc6f906d632	0	0	4	0	0	0	0	0	0	0	0	0
8ce8ebba96dceefca030cbcf7b8d3637	0	0	0	0	0	0	0	0	0	0	2	0
9599c21f95eee1b81f6fcb6d959f23e4	0	0	0	0	0	0	0	0	0	0	3	0
cbe89d31e898276ce2633ab4d3487c16	0	0	152	0	0	0	0	0	0	0	0	0

1efac65ee1ad0aff6859cfeb7 4ce96b9	5	0	0	0	0	0	0	0	0	0	0	0
094ec938c15726f4928b9952 1852b919	2	0	0	0	0	0	0	0	0	0	0	0
11f30e305600c7abb9671396 f99696d7	0	0	0	0	0	0	0	0	3	0	0	0
0f05c26aed0f5236fb4e88242 1152dbd	0	0	0	2	0	0	0	0	0	0	0	0
24813e56e6e031d124ebb5e 160cfcf2d	0	0	0	0	0	0	0	0	0	0	2	0
9beb6bca25635274d2efd8bf 475e30ad	0	0	0	0	0	0	0	0	4	0	0	0
7d35e6fe443cb6d7966fdec3 0a0ccc55	0	9	0	0	0	0	0	0	0	0	0	0
947db17ac49b0fcb8db4d738 f38e3121	2	0	0	0	0	0	0	0	0	0	0	0
e37038dc96312b380f1adbb3 d823871b	0	0	0	0	6	0	0	0	0	0	0	0
61e189c5872b8214273a2b5 e5ffca755	0	0	10	0	0	0	0	0	0	0	0	0
9c7f6d888f9277e9731b83cd 0bd740e1	0	0	0	0	0	12	0	0	0	0	0	0
3c74841aef4f6a2ec9e6bb6b 0c24a983	0	0	0	0	4	0	0	0	0	0	0	0
2ce738a8490f0b2d3dc417a1 dbd32288	0	0	0	0	0	0	0	18	0	0	0	0
a3dcfaa74179cbad09e21499 c4427059	0	0	0	0	27	0	0	0	0	0	0	0

3579901babc05d2b7ad953e9cb098be9	0	0	0	0	0	0	0	0	0	0	0	14
9240416a9692dae24160b637636631cb	0	0	0	0	0	6	0	0	0	0	0	0
3b999b27d165a3dc6efb984a0c27f367	0	0	0	0	0	0	0	0	2	0	0	0
f8581416538fc3be112131f3ddeada47	0	0	0	0	0	0	0	0	0	0	0	12
a5ff3b5cf3a7cb9d997ebd714d97e823	0	0	0	0	0	0	3	0	0	0	0	0
670260a2c4a89a5c336d232d613296ea	0	0	0	0	0	0	0	41	0	0	0	0
9acf102806c5dd69f16406ae21c10dbc	0	0	0	0	0	0	0	4	0	0	0	0
9f26703984f71554364334518e15af11	0	0	0	0	0	44	0	0	0	0	0	0
7d0c310ed3ecd49e139143c3d0e753fa	0	0	0	0	0	0	0	0	0	0	4	0
7b150abaa80d1ef9c8f64f33cc2a7e8a	0	0	0	0	0	0	0	0	0	6	0	0
a0607ea3702fde937c1d38a2f8bba2b8	0	0	0	0	0	20	0	0	0	0	0	0
a929c057c75f7bac994a34a1d0fc8da4	0	0	4	0	0	0	0	0	0	0	0	0
587adca5805a9c66a2786d40c3d7adc3	0	28	0	0	0	0	0	0	0	0	0	0
c2caed971630a3c908712726b90d12f7	0	0	0	0	0	0	0	0	0	3	0	0

b42152a06d880ec17d934ff614b3d4ed	0	2	0	0	0	0	0	0	0	0	0	0
64eacc93d02d2162e5fe23b63e5ecf2f	6	0	0	0	0	0	0	0	0	0	0	0
146d474e679c63b6e1f8f37620c2c84b	0	0	0	0	0	0	0	0	0	0	6	0
38f3102c46cbaf05822cb3bb624e806d	0	0	0	0	0	0	0	0	0	0	2	0
d732c3b0b3dd14cbd36a720e6dfc3607	0	0	0	0	0	0	0	0	0	0	5	0
b03ac7889f74ab5e8b5f7f391ef2336e	0	0	0	0	0	0	0	0	0	0	22	0
36be435cc5a983d5f9e2c2fdb47cc457	0	0	0	0	0	0	0	0	0	0	3	0
5e7b3b3446ca64d96999bf3e665d1bfd	0	0	0	0	0	36	0	0	0	0	0	0
743f8a3513756d41970cf336c693eba1	0	0	0	0	0	0	0	0	0	0	6	0
730d3fda855a62ee6e3ebff616a05de7	0	0	0	0	0	0	0	0	0	0	6	0
cd4a8621cbce8a35a0214f09eacaaed1	0	0	0	0	0	0	0	0	0	0	3	0
4b17123c618cc495044204e60b062daa	0	0	3	0	0	0	0	0	0	0	0	0
51920dddcb876253b71324d0e5418b4e	6	0	0	0	0	0	0	0	0	0	0	0
1e88a73f88a7bd4b8c01444589a9c068	0	0	0	0	0	0	0	0	0	17	0	0

d87d7b1fb78e65d9c3c5b5ed24ab656a	0	10	0	0	0	0	0	0	0	0	0	0
8d8006d896cfceb515fcdec41181080b	0	0	4	0	0	0	0	0	0	0	0	0
9422d002705df939a1eb5758718bea34	0	0	0	0	10	0	0	0	0	0	0	0
959403b927cab0054f41ca9655668afb	0	0	0	0	0	0	0	0	0	18	0	0
8026d6c4675308c725352722af29559a	0	0	0	0	0	0	0	0	0	42	0	0
f62bf516aa443f7e38f759c4f5fdf422	0	0	0	0	0	0	0	0	0	9	0	0
78c37b71c6e23fb9e16938060173d321	0	2	0	0	0	0	0	0	0	0	0	0
76e596495dca59738162fbebe1655691	0	0	0	0	0	17	0	0	0	0	0	0
c176e7b26fd70ddaa0eca5e847ea0cfe	0	0	0	0	0	0	0	3	0	0	0	0
0747e2d995784e1b9baadd449a35001b	0	0	0	0	0	0	0	0	0	0	0	9
145a4407fe12a2372959f10611308786	0	62	0	0	0	0	0	0	0	0	0	0
787c4c24dbb83d7aef69d6708ca77f07	0	0	0	0	0	5	0	0	0	0	0	0
403d9d7cab45d72eb39dcd7b16f55279	10	0	0	0	0	0	0	0	0	0	0	0
504033990d1dea5ce3e77badc4086dd7	3	0	0	0	0	0	0	0	0	0	0	0

84107110fcd4f423d2632a12a79468c3	0	0	0	0	0	0	0	0	0	0	6	0
6e01635fc8d7f6461a0745d93b9cdfc3	0	0	0	0	0	0	0	0	0	0	0	16
8b40948b04d41bac0b32451cb6d0ee19	0	0	0	0	0	0	2	0	0	0	0	0
07a777383c0ff2a858133d88a82b0630	0	0	0	0	0	0	19	0	0	0	0	0
ab11d09d16b2740e5a70ec4a2c10b03b	0	0	0	0	0	0	0	0	0	0	36	0
8f4fac7d50fa785f981e463a60722335	0	0	0	0	0	0	0	76	0	0	0	0
db877a03db5aca5d3596c8e37436486b	0	0	0	0	0	0	17	0	0	0	0	0
14645b970c23906267735c2a40dd9e45	0	0	0	0	0	0	0	40	108	0	0	0
69f5f4ef3755177cb1c3375936cfc4b3	0	0	0	0	0	0	0	0	0	0	31	0
832f41fd63f0227d8b849ba405a313b9	0	0	0	0	0	0	0	0	0	0	0	69
bc0df0c7b710bfedabcb10056e2300b2	0	0	0	0	0	0	0	0	6	0	0	0
5a36a02b99250c994fd2eaaca729fc65	0	0	0	0	0	0	0	0	0	0	0	5
7769c0abc8ce07451c664c80bf47a669	0	0	0	0	0	0	0	0	0	4	0	0
40b46f901b3fed799f3309588be3ae80	0	0	0	0	0	7	0	0	0	0	0	0

1ee1bcf559eff603d9ac4009ab6f065c	2	0	0	0	0	0	0	0	0	0	0	0
f3102644e8268a8e9310799a096996b4	0	0	0	0	0	0	0	0	7	0	0	0
b37ee1667cbdeb5c048cc434a7fb9e56	0	0	0	0	0	0	0	0	0	5	0	0
5971bf329277b096464316e529170146	0	0	0	2	0	0	0	0	0	0	0	0
ef3b281e2889c69c75f36e16a0d028ba	0	0	0	0	0	3	0	0	0	0	0	0
1740b7b7818cc99a632b8555316fc746	0	0	0	0	0	0	0	4	0	0	0	0
54baac8c7b0c90f3d0cd9bc8a5ba8649	0	0	0	0	0	0	0	0	75	0	0	0
de00e3c53d2947dc4d6aad39a041e301	0	0	0	0	0	0	0	0	0	60	0	0
a98a39f630262097b362f0d24e5bfebe	0	0	0	0	0	20	0	0	0	0	0	0
cefa67f9fdb05251106259e3348724a3	0	0	0	0	0	0	19	0	0	0	0	0
a97696b01447407b9c3ef8f7b9de048e	0	0	0	0	10	0	0	0	0	0	0	0
5e0c75b9c074439ba6b5da645f173ce8	0	0	0	0	0	0	0	2	0	0	0	0
aa1ccea6745f39939db20d597997b007	0	0	3	0	0	0	0	0	0	0	0	0
5f3c9eb1f8e82c3dd1e0581ce769f579	0	0	0	0	0	0	0	9	0	0	0	0

01ba92e08cdd5c00d26bde0309e50796	0	0	0	0	0	24	0	0	0	0	0	0
99326a7d559f939ddcf6a4b06bba77b1	0	0	0	0	0	0	0	0	0	0	2	0
2ca8f5a923a4ae6f648c93bce7b6d2c4	0	0	4	0	0	0	0	0	0	0	0	0
1cc8d9602b51eac349f4fa06f396a252	4	0	0	0	0	0	0	0	0	0	0	0
8b009c3f5c3d21b263a5255faf7ab3c4	0	0	0	0	0	0	0	0	0	5	0	0
a7ce8cbc8b9e0d78e9bc970e6d66f02c	0	0	0	4	0	0	0	0	0	0	0	0
0670e52d6f72022513fc2016fc32a303	0	0	0	0	0	0	0	0	0	0	0	4
f2e7dfa680fbb40e92055be1eabbe423	0	0	0	0	0	0	0	0	0	0	0	5
57966a38664122e2a4083d0f8fa6ccfa	0	0	0	2	0	0	0	0	0	0	0	0
a22a171b2d33823eaae777c9c75ec706	0	0	0	0	0	0	0	0	0	0	3	0
3dd8fcdf2b8e2a0f607fa049c1a1dce0	0	0	10	0	0	0	0	0	0	0	0	0
0b825b3abd0078e0cc2d4d838dcaa2ee	0	0	0	0	0	0	0	0	3	0	0	0
d8e0a4d8979a24fc14c278607c731f73	0	0	0	0	0	0	0	0	2	0	0	0
74c0d8dd176c53a1ff40e93d0fe65d8f	0	0	0	0	0	0	0	0	0	0	9	0

e81b7db7abe119e72b307f3762104af1	0	0	0	0	0	0	0	61	0	0	0	0
a915fe3dbcc2f526944475dec862f011	0	0	0	0	0	0	0	15	0	0	0	0
b0625a5a555978aabaa8a87f0207ac8d	0	0	0	0	0	0	0	0	0	3	0	0
b924132e4d859d9afadca6788cf76620	0	0	0	0	0	0	0	0	0	0	5	0
d67e8fa0a4380c3d6d227a916b76c555	0	0	0	0	0	71	0	0	0	0	0	188
c8db82f6fd5ca4d6f7e4d3c2b1194fdd	0	0	0	8	0	0	0	0	0	0	0	0
9f36049aebfeefd66b2f4da312926c83	0	0	0	0	0	0	9	0	0	0	0	0
7ed46d53d93a1c90b0ce965d7bbcea62	0	0	0	2	0	0	0	0	0	0	0	0
1b1ad362eef98d2861073e941bd81cf3	0	0	0	0	0	7	0	0	0	0	0	0
9d6047fdb3b51b1ec8efe61e91d36080	0	0	0	0	0	0	0	0	0	0	6	0
8733cdf3dc59dcb5765c8e1a52bf2ac9	0	0	0	4	0	0	0	0	0	0	0	0
d9f9297dfd0f4f6ec19efb7726887fe3	18	0	0	0	0	0	0	0	0	0	0	0
f2b04142f2e7c9bcd294967c65a26eb1	0	0	0	0	0	31	0	0	0	0	0	0
c54b105dcc198eef2ef25e3db036dd8d	0	0	0	0	0	0	3	0	0	0	0	0

12e300871541446f46cf5bb1819883ae	0	0	0	0	0	0	0	0	0	0	0	6
c055d8710cacac09c70411bd4556c6fa	0	0	0	0	0	0	0	0	0	0	6	0
1c4ad18355d6e9228f63e17fc75a70eb	0	0	0	0	0	0	0	2	0	0	0	0
4d5208014b495812ed8b6b1ac6678940	0	0	0	0	25	0	0	0	0	0	0	0
bccfa87fd5d11303a1f9d98647646804	0	0	0	0	0	0	0	0	2	0	0	0
915e55ae8520a5775d66b0b320f0503f	0	0	0	0	0	0	0	0	0	0	13	0
925cd52f985a6d69678d91e063b6b862	0	0	0	0	0	0	0	0	2	0	0	0
7187ec86bfff8ae08e2dee6fc9aea850	0	0	0	0	0	0	0	0	0	95	0	0
1e71df151d06f60042044546421b2b61	0	0	0	0	0	0	0	0	0	6	0	0
57e3c698c82eb7f8f420e4f819fd2c61	25	0	0	0	0	0	0	0	0	0	0	0
663a01833c5fcc29f56a6bdb904029bb	0	0	0	0	0	0	6	0	0	0	0	0
fc16f654a2573efddb0d840929877b7a	0	0	0	0	66	0	0	0	0	0	0	0
b96db305650f1b54300721a7e3747332	0	0	0	0	0	0	9	0	0	0	0	0
fed4d5d2d8af60dd3c8346d7a400b91f	0	0	0	0	0	0	0	4	0	0	0	0

7a99d31e29375667a790c38 9135ee969	0	0	0	0	0	0	2	0	0	0	0	0
f295282f1b53efd4a4d6555f7 a6d5d59	4	0	0	0	0	0	0	0	0	0	0	0
3d0d1b01000c97805c696c6f 1f0dafd5	0	0	0	0	0	0	0	0	0	0	2	0
8fa9694e8b126f356f2ae93b d120c210	0	0	0	4	0	0	0	0	0	0	0	0
a1c97a5bee87ebcd0074370b 538cdc93	0	0	0	0	0	3	0	0	0	0	0	0
a754332b29c32de3e6ed2f57 def142c2	0	0	41	0	0	0	0	0	0	0	0	0
a08acdb937616967c092c80a 0a716ac9	0	0	2	0	0	0	0	0	0	0	0	0
68efc4c9d6dbcc9b61da39c7 43ce8c8f	0	0	0	0	0	0	0	0	0	0	0	3
6c674e9583b6dace913d10a0 9c94dbc5	0	0	0	0	0	0	0	14	0	0	0	0
9518df43ba71682f9a1fa1f75 fb98df3	0	0	0	0	0	0	0	0	0	0	4	0
1543932a054b61876be63ed 372331424	0	0	0	5	0	0	0	0	0	0	0	0
3751483cbdc693692048f6fb aff515b6	0	0	0	0	0	0	0	3	0	0	0	0
6de631f7408db8f4bc378fed bad0f3a0	0	0	0	0	0	0	0	7	0	0	0	0
33c849695417fa13f4a35163 2f84e780	0	0	0	0	0	0	0	0	0	5	0	0

f88e07531b07e62ca6254098 6bfb3e30	0	0	0	0	0	8	0	0	0	0	0	0
8348ac43d325b62b1c66b97 0d03a4852	0	0	0	0	0	0	0	26	0	0	0	0
f68404b9109eb38792f9cbdf dcd498b2	0	0	0	0	0	0	0	0	5	0	0	0
0765c6aa468af8f38c9873a97 fd3b756	0	0	0	0	0	0	2	0	0	0	0	0
ea788caf6f29ffbc8fb3b0d719 a70544	0	0	0	0	0	0	0	0	0	11	0	0
a683f760dad2a0ae8bdb6632 54f1148e	0	0	0	0	0	0	0	8	0	0	0	0
ec068c2ab837190734b7c738 6b77c015	0	0	14	0	0	0	0	0	0	0	0	0
7070ef76ff40ce9088759a278 8b8d6ff	0	0	0	0	69	0	0	0	0	0	0	0
29ae712c3ba52958933ad68 a256c9cf6	4	0	0	0	0	0	0	0	0	0	0	0
b40c177c05b435ac2074e6b1 b867841e	0	0	0	0	0	0	0	0	0	0	4	0
8d1597183b5a0f2099717a7a 1dd3e9b7	0	0	0	0	0	0	0	0	0	3	0	0
e24f0f07b7a696f87a047673 26a78514	0	0	0	0	0	0	6	0	0	0	0	0
064f033c14dfd7e8cb1bed01 525d804c	0	0	0	0	0	0	0	0	0	0	16	0
2eae8b23d1ff63b7f3c9525f7 c6562a9	0	0	0	0	0	6	0	0	0	0	0	0

e0a6e688fa93d986b3e97842e087331a	0	0	0	0	0	0	2	0	0	0	0	0
28a2f76365189d9f4054ac2debf4398a	0	0	0	0	3	0	0	0	0	0	0	0
56eeb97382ad699cae6e102c143e77c2	60	0	0	0	0	0	0	0	0	172	113	0
7ee692f9209ded3029098cf1cdd5ffea	0	0	0	0	0	0	0	0	0	0	2	0
76f8c274e01c9ced006458a9d1750ebd	0	0	0	0	0	0	0	3	0	0	0	0
3af3eee6066d1661fd0d9637417f3439	0	0	0	2	0	0	0	0	0	0	0	0
28701cfafd4143e9ef70dbb18e13b318	0	0	0	0	0	0	0	5	0	0	0	0
bf156b86bb244fa2b4512638b5fa8cc2	0	0	0	0	0	0	0	0	0	0	8	0
995f2d105a0f58af72963e00f2e9a2ce	0	0	0	0	12	0	0	0	0	0	0	0
d77c07ec21106ea7257c8e23db28ad81	0	0	0	0	0	0	0	2	0	0	0	0
2bc7c18ac4b8ef1d35ade38a2c233426	0	0	0	0	0	0	3	0	0	0	0	0
73cba32226db6d7878efb8d8820ec1d9	0	0	0	0	0	0	0	0	86	100	0	0
2489d7d80478eff991cb9223186e1a54	0	0	0	0	0	0	0	0	0	3	0	0
b30b8d2f9cadb7351c909628d7d838e9	0	0	0	0	0	0	0	0	0	0	0	3

e649fc077ffdb12e9afe35aad6ad1654	0	0	0	0	0	0	0	0	0	79	0	0
c2cb5154350d98ad2e272b7ce52e1119	0	0	0	0	0	0	0	0	0	3	0	0
e88b15ed7dfa30d3ad4088057dff8082	0	0	0	0	0	0	0	0	0	3	0	0
b3647a987774f7080195cb5d9b520285	0	0	0	0	0	0	0	0	0	23	0	0
2b776d3b1f5f86bd3d12eb3390d22562	0	70	0	0	98	0	0	212	0	0	0	0
0b387fa55aaac6282100bc3d8fd01e2c	0	0	0	0	0	0	0	0	0	0	5	0
37f8e0bdc033ad31ef1fef1e15b928c8	0	0	0	0	0	0	7	0	0	0	0	0
35fa5c6b4d21fd9a5c6e7c8f72e2d2ed	0	0	0	0	0	0	0	0	0	10	0	40
d9c0bf132492b51945963636352b261f	0	0	0	0	0	0	5	0	0	0	11	0
69acb20f053b5e26bfe155761b03bdf8	0	0	2	0	0	0	0	0	0	0	0	0
6da07ae41f20d37ba4febe53e55e981b	0	0	0	0	0	0	0	0	0	0	11	0
7a3fdbe776f97422097168a0d0c13392	0	0	0	0	0	0	16	0	0	0	0	0
8120d382e9e188eaa9fff3117e117160	0	0	0	0	0	2	0	0	0	0	0	0
b311440388237e458fddde6149ba26e0	0	0	0	0	0	0	0	0	0	0	5	0

ed214baa24549d2aaf55344f83f74fe7	0	0	0	0	0	0	0	0	3	0	0	0
506579c461022f3a6e8a3e7865d39264	0	0	0	0	0	0	5	0	0	0	0	0
fee488a7f5af542e4037fb5dc959c5fa	16	0	0	0	0	0	0	0	0	0	0	0
4db15b99f69ac4b3a0d7bc132a3286ec	0	0	0	2	0	0	0	0	0	0	0	0
132749d85b41398f8a5141f19d15947a	0	0	0	0	0	6	0	0	0	0	0	0
92a667f37365dc3f7b6e06e86e30f2ae	0	0	0	0	0	0	0	0	63	0	0	0
7f0cfeef4764bcb5fe5cd672b06405d9	0	0	0	0	0	0	12	0	0	0	0	0
3ff8fa1baf80c90d2055c8c19799af6a	0	0	0	0	0	0	0	0	0	0	0	3
bc1fa02ec00885f756f468d8136ccccb	0	0	0	0	0	0	0	0	0	0	0	5
91335ce183bff900e8d732c444da316e	0	0	0	46	0	0	0	0	0	0	0	0
6f084a7a90af48064520b43dd3b2c4fd	0	0	0	0	0	0	0	7	0	0	0	0
bfc4ed35b0c6d582e5941230fec6dc42	0	0	0	0	0	0	0	0	11	0	0	0
72f6871fa1f639bac0a71bacce5d7085	0	0	0	0	63	0	0	0	0	0	0	0
5fa64b25280fc27d7bd42b736185df79	0	0	0	0	0	0	0	0	0	0	0	2

303e50dc3e8bfde31b76a9225cd497cb	5	0	0	0	0	0	0	0	0	0	0	0
2a9bcf25dc9412ebabdbf10a8e8900b1	0	0	0	0	0	3	0	0	0	0	0	0
4b41db67cd5f06a09b6ba12a937928cb	0	0	0	0	0	0	0	0	0	0	17	0
c0b453ed80ae6d41a9f1488cb040a715	0	0	0	0	0	5	0	0	0	0	0	0
588bff7340134829e5d98d07de2f8e30	0	0	0	0	0	0	0	0	0	11	0	0
bde5b7cebe43cca0e110b7bd9c8c1079	0	0	0	0	0	0	0	9	0	0	0	0
4b6865e1b73032477dc0a8e57ead9699	0	0	45	0	0	0	0	0	0	0	0	0
a18a32c098b9c95ee479dd8f839f32f4	0	0	0	0	0	0	0	0	2	0	0	0
a5bb0813ef32f9acf37450f12914c6fe	8	0	0	0	0	0	0	0	0	0	0	0
df114200a8769554fb0895ec5331347a	0	0	0	0	0	0	0	0	0	0	0	5
f3014c9898c97a8cee78883ad322d641	0	0	0	5	0	0	0	0	0	0	0	0
40464d121133c8b3e7381f359b9f09cc	0	0	0	0	0	0	0	0	7	0	0	0
a5befaca1786309c76c141ebae0a8197	0	0	7	0	0	0	0	0	0	0	0	0
b9617125b68cf1a8fa34858832721dae	0	0	2	0	0	0	0	0	0	0	0	0

e9db441dbb4b1f6931f6cbb24f33c494	0	0	0	0	0	0	0	0	0	3	0	0
07ff9a87ac665a7897bac78e8eb973e4	0	0	0	5	0	0	0	0	0	0	0	0
d1db33ca672c1022b2ab642c7794dc4d	0	0	0	0	0	0	0	0	0	3	0	0
25208fda7a122b0c90b3c597671041be	0	0	0	0	10	0	0	0	0	0	0	0
96f866cb2bb3f4efb7b42024c0cd497f	0	0	0	0	0	0	0	0	0	11	0	0
ace291fdd58883c26a0f819996ad9bf2	0	0	0	0	0	0	0	0	0	0	24	0
92f93ad3592e3989a5983de20f817af7	0	0	10	0	0	0	0	0	0	0	0	0
287b5876ceca8e5db1ea46ae31117550	3	0	0	0	0	0	0	0	0	0	0	0
deda66ef0a6cb50ee76708830f230229	0	0	0	0	0	0	13	0	0	0	0	0
95957e28b32596c713e13a020dc294c1	0	0	0	0	0	0	0	2	0	0	0	0
0601b67499a4b331fdee4dd4a30265ef	0	0	0	0	0	0	0	6	0	0	0	0
f2d1f0ae05568de1a6dcd4457f542958	0	0	0	0	0	0	0	0	173	0	0	0
9b0e92a0db6483b4dec97fe8d6671da8	0	0	0	12	0	0	0	0	0	0	0	0
4048209d617478302010444fb03856f4	0	0	0	0	0	0	0	37	0	0	0	0

55b4930ce94003e2b081326 77210ff54	0	0	0	0	0	0	0	0	0	0	0	123
8b1406dfaa06c97faba5f4d1d 14938c9	0	0	0	0	0	0	0	0	0	0	9	0
013b48f85db40cdd88221bb 99cf5cba6	0	0	0	0	0	0	0	0	0	0	6	0
9b1690c91fa0c6c5846f7cce3 3ffd203	0	0	0	0	6	0	0	0	0	0	0	0
e4421116532a6f20c4745a94 c8f774ad	0	0	0	0	0	0	0	0	0	0	3	0
a7e0460bf8b3154a10fbd410 e48fe546	0	0	0	0	0	0	0	0	0	7	0	0
c38c7865d7287f668b8e41fa 47fd6458	0	0	0	0	0	0	0	4	0	0	0	0
5b5ac4bf765b8f15d27113dd 1e615996	0	0	0	0	0	0	0	0	0	0	4	0
bd0bac9701e7709ba7e252f2 3b742c68	0	0	0	0	0	0	0	0	0	0	4	0
d442d51f2ce7bfb3351fc3c62 4d82947	5	0	0	0	0	0	0	0	0	0	0	0
b358b369d6557a8a5e2d8bd 039793a31	0	7	0	0	0	0	0	0	0	0	0	0
40e157b0ca66ff5992d38034 7df436b0	0	0	2	0	0	0	0	0	0	0	0	0
61efa4405b8fcce01af64c25f 3c39293	0	0	0	0	0	0	0	0	4	0	0	0
8c22eca4999b4492d936d49 051fa80bd	0	0	0	4	0	0	0	0	0	0	0	0

336ece5c9c466eeb530a0f7cc8a184b9	0	0	0	0	0	0	0	0	0	0	0	2
a0b757702e2c590eeff94ae020bae4de	0	0	0	0	0	3	0	0	0	0	0	0
6c891239f4ead76f708fa4ac2abfe685	0	0	0	0	3	0	0	0	0	0	0	0
77596be6c34b71045df0e1463be53c2b	0	0	0	0	0	39	0	0	0	0	0	0
bc2a285cf78cadc827b322dec244f61f	0	0	0	0	33	0	0	0	0	0	0	0
af2b3a70a8547f1046916e2c16f6727d	2	0	0	0	0	0	0	0	0	0	0	0
f380e69818cbdfa33d3532685116411d	0	4	0	0	0	0	0	0	0	0	0	0
d456988f499e9976829894bfa0f011ba	0	0	0	0	0	0	6	0	0	0	0	0
c20760185a901d4e78570c68db7401d5	0	0	0	0	0	0	0	0	0	0	0	10
31f4881b40abf5a8ea39fd5bf220e0c4	2	0	0	0	0	0	0	0	0	0	0	0
fff4a98b51fd13d43f39b0e0669b0b63	0	0	0	0	0	0	0	0	0	0	120	0
1b2343c5dabc45c86cf2558f5874c8cf	0	0	0	2	0	0	0	0	0	0	0	0
5f1b04471785a16c5d0ca1a1cebb71f6	0	0	0	0	0	0	0	4	0	0	0	0
de7358f46c479a692c486c2ba2e263f5	0	0	0	0	0	0	0	0	0	2	0	0

3e22d280167a9b351861846a6be1896c	0	0	0	0	0	0	0	2	0	0	0	0
9bcc1e41c05f04870f70c28ebed4115d	0	0	0	2	0	0	0	0	0	0	0	0
8bcbf2a6353173b549ff5731ba695650	0	0	0	0	0	0	3	0	0	0	0	0
b17066016186ce3357cc0140ae37708b	0	0	0	0	14	0	0	0	0	0	0	0
f87055a913c767e093bc20addb18a0c4	0	0	2	0	0	0	0	0	0	0	0	0
323b90edaade99e46d5fc60a476ceb26	0	0	0	0	0	0	0	0	0	0	12	0
e05bbb6c70615ea820a4c853d1ef24b1	0	0	0	0	0	0	18	0	0	0	0	0
80ec45a597d147973303bc46f6d0739f	0	0	9	0	0	0	0	0	0	0	0	0
ad3c88f721eadd13bc77ec0ddae858c3	0	0	2	0	0	0	0	0	0	0	0	0
b713df649b8371deea0accb813699e32	0	0	0	0	0	0	10	0	0	0	0	0
2fb9a4267824a5278138445a03ba5920	0	0	0	0	0	0	0	4	0	0	0	0
e63b0bc46e138fc8c127b2fc06da2dc3	0	3	0	0	0	0	0	0	0	0	0	0
fe02f97f5a8da7319e97a20436c5b4c4	0	0	0	0	0	0	0	0	0	2	0	0
ebf905b9040a3581a961d2578c714518	0	0	0	0	0	0	0	4	0	0	0	0

ee1e93506439d8908205b3c5cc8cb093	0	0	0	0	0	10	0	0	0	0	0	0
637a801f44027e3845318fdcb64e27c6	0	0	0	0	0	0	0	0	0	0	19	0
7c8261c733105cd693444e3be232e976	0	0	0	0	0	0	0	0	0	0	3	0
fd2491e7232fe2d949f1c0d0ac5e44da	0	0	0	40	0	0	0	0	0	0	0	0
98f29b6e71b2be4eea0f86fb0c398e20	0	0	0	0	0	0	0	12	0	0	0	0
06601dcfd210669c26163fc09bb94e7c	0	0	0	0	0	0	0	0	0	0	2	0
8a03f7a8c1f69b2db2028c3e3772ecd8	0	25	0	0	0	0	0	0	0	0	0	0
358b8ec55da0ad644e9ff8608a098eaa	0	0	0	0	0	0	15	0	0	0	0	0
ab1c4513237ae17f2361f14a82c68c85	0	0	0	0	0	0	0	0	0	0	4	0
167e4989b473e37bf1a6c42ec2718aa9	0	0	0	21	0	0	0	0	0	0	0	0
39b2b3f4c212b5423bb43eb7bf5c2efe	51	0	0	0	0	0	0	0	0	0	0	0
b8ba44ed259c9e98c3e34e045063db0e	9	0	0	0	0	0	0	0	0	22	0	0
3377f3780177b14871db365c3c97d532	0	0	0	0	90	0	0	0	95	0	250	149
76c8b55162a2b2dd2493026b8fd110e1	0	0	2	0	0	0	0	0	0	0	0	0

386edba3545008012acef3b5899604d8	2	0	0	0	0	0	0	0	0	0	0	0
265a8633056f59206c11e01a788bd6bd	0	0	0	0	0	0	0	0	0	0	7	0
88c31e00d8b02f40a63e14a2a0e31ddf	0	58	0	0	0	0	0	0	0	0	0	0
f92c84093d5d6592c938e0fb268eb6fe	0	0	0	0	0	0	0	0	0	0	46	0
0204093026acc5f690593ad478a938b5	0	0	3	0	0	0	0	0	0	0	0	0
ce0e8aa445daa573e05a36074cf30979	0	0	0	0	2	0	0	0	0	0	0	0
d084f3e4d08f82768ca1a99e6db5b64f	0	4	0	0	0	0	0	0	0	0	0	0
b66120f46773efaf14098bb0ae1849d5	0	8	0	0	0	0	0	0	0	0	0	0
83f134a27172d4942b4dcfed271e84e5	0	0	0	0	0	0	0	0	0	0	0	17
25f845615c69f48e69fa27545e49832d	0	0	0	0	0	0	2	0	0	0	0	0
18780b4cb4e4b5dcade9bf67449b30ee	0	12	0	0	0	0	0	0	0	0	0	0
6140e070f00e7e71e8e4f58029634de5	0	0	0	12	0	0	0	0	0	0	0	0
03a32db732564390c838c6b64ab69d30	0	0	0	0	0	0	0	0	0	2	0	0
a87d6b2d21f696187a29e560ea740849	0	0	0	0	0	0	0	0	0	9	0	0

b0f7fbb7889d5fef80caacaef5bf16ef	0	0	0	0	0	0	0	0	0	0	0	6
ac67652a90e28f7bb366efbb664a0a4d	0	0	0	0	0	0	0	0	0	0	3	0
90003638313be7d653295d8ba4dd388a	0	0	0	0	10	0	0	0	0	0	0	0
58aa4eb549c98a6d6de5fe3a0600d847	0	0	0	37	0	0	0	0	0	0	0	0
0fd02ccc84427ec716c871ac1d7ea86a	0	0	0	0	0	0	0	0	0	0	5	0
f726d98898a6ca3a7efb1ce9931b729b	0	0	0	0	0	0	0	0	0	13	0	0
768fae1a21a19f13f122d0d2b647b61d	0	0	0	0	0	0	0	0	2	0	0	0
7edd118bfee1a994def69741b6f0c30c	0	0	0	0	0	0	0	0	0	0	27	0
bfdad354b6fde97df134c347b5c65710	0	0	0	0	0	0	0	0	0	0	0	5
de05570a1a0870a2c2bea7edabe29394	0	0	0	0	0	0	0	0	0	0	0	2
08e2220b3e21a3708f88a654883baca2	0	0	5	0	0	0	0	0	0	0	0	0
cc995354097002864c44e15efdf6f04f	0	0	0	0	0	0	0	0	61	0	0	0
10aa4b1d68be1fe49d328307080225a5	0	0	0	0	0	0	0	0	0	0	0	3
8980ff8d767d4384ca96f58c2bbc2a51	0	0	0	0	0	0	0	3	0	0	0	0

01ffa255fb032391cf0627d5927bf32f	3	0	0	0	0	0	0	0	0	0	0	0
034078c61806c5d54fbd2e1566ded973	0	0	0	0	3	0	0	0	0	0	0	0
6be4248799b4a324ce8f5251b181a9fe	0	0	0	0	0	0	15	0	0	0	0	0
cc8f61486e0771b1c3de97a435a07785	0	0	0	16	0	0	0	0	0	0	0	0
2ad685fa188a3c19ae1945b306773072	0	0	18	0	0	0	0	0	0	0	0	0
b3bc31362047d75014fbffa8c3855953	0	0	0	0	0	0	0	4	0	0	0	0
fb163926db8e0ea6871f0ff8fa2903d1	0	0	0	0	0	2	0	0	0	0	0	0
edd7177875cf917e82e5b7318fba201d	0	0	0	5	0	0	0	0	0	0	0	0
98de3628c66d59792591daa8565539c7	0	0	0	0	0	0	0	0	0	0	0	10
dfe5f2b9f330e14edcc21407bccdb238	0	0	0	0	0	0	0	0	0	0	0	17
5af5a04662630f92613dd21ad86e919b	0	0	0	0	0	0	0	0	0	0	5	0
c1efd6840e736f28b128655bc15acbe9	0	20	0	0	0	0	0	0	0	0	0	73
ec4ec79b2dd5cf919aa5db205a9c2ada	0	0	0	14	0	0	0	0	0	0	0	0
0fc268ab7296dbf9bbd4fd4df6d8364a	0	0	0	0	0	3	0	0	0	0	0	0

4cca2fabbf623246e370a708b555959f	0	0	0	0	0	0	0	65	0	0	0	58
bc1f6669502abaa3820c4b6327dce235	0	0	0	0	4	0	0	0	0	0	0	0
67d54c774c82eeb391bb5e0cf6d313d2	0	0	0	0	0	0	0	0	0	0	9	0
4aabafaafa3a2fc3cad87df8163a57a3	0	0	0	50	0	0	0	0	0	0	0	0
15e825d9b07b4cc6cb8a676f488f5f33	0	0	0	0	0	102	0	0	0	0	0	0
7b54d35e6e1923971205beb48a9b605e	0	0	0	0	0	0	0	0	0	0	3	0
5c8dd033fb09f5c17239cdcd383854a6	0	0	0	0	0	4	0	0	0	0	0	0
7908d0dc113c203f4a2d66352c4441b9	0	0	0	0	16	0	0	0	0	0	0	0
6789ab0a36821a24f55786a1275dbb78	0	0	0	0	0	0	0	6	0	0	0	0
91906c71fd5579a102e6af2f30ac6b59	0	0	0	0	0	0	348	0	229	0	0	0
aaba42f763247d513c09af837d5ad35a	0	0	0	3	0	0	0	0	0	0	0	0
b0d6cb270f085f839d59f7fefd8c8ee1	0	0	0	0	20	0	0	0	0	0	0	0
27867de45fd3002c1d5cfc6ff529a59a	0	11	0	0	0	0	0	0	0	0	0	0
8304df84395b94414af14ee624d5145e	0	0	4	0	0	0	0	0	0	0	0	0

26e3bc28168c279df6b11859 2ab2c101	0	0	0	0	0	0	0	0	0	0	3	0
b6b10e235e3090d2331ef279 a4a31379	0	0	5	0	0	0	0	0	0	0	0	0
020c0b8afe77330aed79b258 01d8dfa9	0	0	0	0	0	0	0	0	6	0	0	0
bd143f4ec34a6870b429dd89 2c8bcd60	0	0	0	0	0	0	0	0	0	0	0	7
c61bae8ae549e442c4e67da5 ad92c595	0	0	0	8	0	0	0	0	0	0	0	0
1db480de2bcd36ab59961c6 3e587b57d	0	0	0	4	0	0	0	0	0	0	0	0
29105ef79ed0d62c04451961 76a9ef51	0	0	0	0	0	0	0	0	2	0	0	0
341ebe34ecaa8fedf93e8639 c0319bcc	0	0	0	19	0	0	0	0	0	0	0	0
92b6bedf17d3346c2b827e4b 8d92db32	0	0	0	0	0	0	0	0	0	0	0	5
20925d05917e9ce0a2b4c3d 0b8971dd8	0	7	0	0	0	0	0	0	0	0	0	0
9220471e637f0ea789cf6ab7 84c09293	2	0	0	0	0	0	0	0	0	0	0	0
acda4c7e25e797a911b5f171 73bc7762	0	0	0	0	4	0	0	0	0	0	0	0
c6e8a5154cdbe580e6dacf6f2 079fa67	0	0	0	0	0	0	0	0	0	22	0	0
52afe648fc477d0e1b7c9cf95 f878ad2	0	0	0	0	0	60	0	0	0	0	0	0

16eb99c3b6ac57d54d33629 7a7ffe249	0	0	0	0	0	0	0	0	0	0	5	0
302b3b5d137b931ed7b9ace 459c8a2d7	0	0	0	0	0	0	0	0	0	11	0	0
5c5e628b15c037fd62877701 a340d490	0	0	0	0	0	0	0	66	0	0	177	0
e6c77bcc5c03c85db539fa2b 68e77418	0	0	0	0	0	0	0	0	8	0	0	0
c05c01358a6db06fe0ef3649 ab3d4c7b	0	0	0	0	0	0	0	0	3	0	0	0
903c7f5a0255d5be1b0a1299 2cfc87dc	0	0	0	0	0	0	0	3	0	0	0	0
51e2d90ede07f7ccbf46c8e1 23d327b2	0	0	0	0	0	0	0	0	0	0	31	0
e26a5180d36c5e35652a0e0 77d80ea85	0	4	0	0	0	0	0	0	0	0	0	0
349f84e4ca0f3f60a7ce02815 c2761e5	0	0	0	2	0	0	0	0	0	0	0	0
e8ee122acd7c0cb6c134f8a8 b05bb551	0	0	0	0	0	0	0	0	0	0	4	0
1f354f441d95a75c3236456e 9fc3514a	0	0	56	0	0	0	0	0	0	0	0	0
fe09aee80365bb6e4574870e 83247cd8	0	0	8	0	0	0	0	0	0	0	0	0
c23ab63afd64441e507ff22a1 e0c90db	0	0	13	0	0	0	0	0	0	0	0	0
a840d530df73ddc919020f93 e06ac496	0	0	0	0	0	0	0	0	0	0	8	0

e782355d30ab87281040644ca3b000c8	0	0	0	0	0	0	0	0	0	0	0	5
6a8356a6e3ff418f5d8dcdcbf31c1b1d	0	0	0	0	0	0	0	0	0	63	0	0
7a947eaa2f0a3c55f3124018028fcf17	0	0	0	0	0	0	0	0	0	0	0	7
73e27ecb757719b2cd839f280db490a9	0	0	0	0	0	0	0	0	3	0	0	0
9add8753801d6ebfbcda62a8c1414d31	0	0	0	3	0	0	0	0	0	0	0	0
fa8886ccb02bfb8784a1c92cae22df46	0	0	0	0	0	0	0	11	0	0	0	0
5700f5916fc85df83e334f2bb78789f0	0	0	0	0	3	0	0	0	0	0	0	0
b13b7e50da9e002aa2effc20aa18a6ff	0	0	0	0	3	0	0	0	0	0	0	0
a815034f0fad564c6cefbf458f487df6	0	0	0	0	0	0	0	0	0	0	0	3
c4d853f95f1be0da24392876b037b00e	0	0	0	0	0	0	17	0	0	0	0	0
da9ce4dec455f6b9372b22ddd7969621	0	0	0	0	0	0	0	0	0	0	0	3
28f1d7bd95fb9541debd0372a6ea554a	10	0	0	0	0	0	0	0	0	0	0	0
804a0e8a56aae3b8e442afd7fea76809	0	0	0	0	0	0	0	0	0	0	7	0
5156ae1bffcabde2cf579f9da7199252	0	0	0	0	0	0	0	0	22	0	0	0

83cdd9c9da08a125a8818ff0ca335f95	3	0	0	0	0	0	0	0	0	0	0
7207844b8d5b824758da18f5100d2a87	0	0	0	0	22	0	0	0	0	0	0
32bdecce9da19fce385d3e5f1df25efc	0	0	0	0	0	0	24	0	0	0	0
f3746e9f93d4f4b7adccef1a52123256	0	0	0	0	0	0	53	0	0	0	0
22b3540b14192812e3a88869bbf415dd	0	0	0	0	0	6	0	0	0	0	0
40639d2d2e61d51a3ed0e88513efa285	0	0	0	0	0	0	0	0	0	2	0
df15a26328bf10f1c8bc480bfe7e2c5e	0	0	0	4	0	0	0	0	0	0	0
d63d82548fa323e2be8bb1c4f84b18cf	0	0	0	0	0	0	0	40	0	0	0
e4e6c981d21d9feb426ae0e413a41b49	0	0	0	0	0	0	0	0	0	0	16
e0d39d60e9237328f430c592ec6f3c2a	0	0	0	0	0	33	0	0	0	0	0
c063b24694ef29d2477f280545dc44e7	0	0	0	0	0	0	0	0	0	40	0
160438751b3d0a2545466262ee848a74	2	0	0	0	0	0	0	0	0	0	0
f46e90520404cd2c61625b6f56ab9038	0	0	0	0	0	9	0	0	0	0	0
15877ec00f1c81163030b1905aec0ed9	0	0	0	0	0	0	0	0	0	11	0

9c18799eefb137946d072e84d67f33c5	0	0	0	0	3	0	0	0	0	0	0	0
6dd0646e1c5617aed97573eab02d448c	0	0	0	0	72	0	0	0	0	0	0	0
105d1b074bbbd1118b16ca2c127a2af8	0	13	0	0	0	0	0	0	0	0	0	0
68da4c3510e550194c95c117a6b1caed	0	0	0	0	0	0	0	0	0	0	2	0
cb9994d2721370684ac8ebabc09b9d84	0	0	0	0	0	0	0	0	0	0	3	0
dcfa1717018b426cd52f2602b193833a	0	0	0	0	0	0	0	6	0	0	0	0
d3939b9dfd65afc6759d04c45637985a	0	0	0	0	0	0	0	0	0	0	4	0
a779373a2fac0ae983c3aa3d962c6fba	0	0	0	0	0	0	0	0	0	0	4	0
8aabf69dfa162c77cbe41ff39c396206	0	0	0	0	0	0	16	0	0	0	0	0
bfea751830f8729c2e47b581a6284af9	0	0	0	0	0	0	0	0	0	0	16	0
f6ac5b0e936e816c7cb6dfa1a94dd0eb	0	0	0	15	0	0	0	0	0	0	0	0
dcda20dcc2882dd800e986211d57706b	0	0	0	0	0	0	0	0	5	0	0	0
7244d8bdb38796d545d68324e6766241	0	4	0	0	0	0	0	0	0	0	0	0
b8f7170bc131a97887ef17f41db51a19	0	0	0	0	0	0	0	0	3	0	0	0

8677720a9b94d3b9b9341c2482b41e60	0	2	0	0	0	0	0	0	0	0	0	0
e9c8ce4d9f061fa3e3329d8e16d95bee	0	0	0	0	0	0	76	0	0	0	0	0
da469c6f60403e4aadd2abd8db7a4e2d	0	0	0	0	0	57	0	0	0	0	0	0
de55d8913cb85f19d6823e0306a7ed17	0	0	0	0	0	0	0	3	0	0	0	0
4c0690dd61812412cc1fe7fea67f0d8f	0	0	0	0	0	0	0	0	0	0	0	2
25de3d94736bcdd8b954702017e066d9	0	0	0	0	0	41	0	0	0	0	0	0
772adcf19665d8984bc438b14f04db08	0	0	0	0	0	0	34	0	0	0	0	0
48e1e1b8d09d0703c7bed48e89649611	0	0	0	2	0	0	0	0	0	0	0	0
144b20271e0420a6cb2f788978c61a48	0	0	0	0	0	0	0	0	0	0	3	0
39e44e709b724e16ceb8aea2cfb53bc6	0	0	0	0	0	0	0	0	0	0	21	0
570250512c96d107002dfe8ab1e42630	0	0	0	0	0	0	0	6	0	0	0	0
46707230dcd4ed775366ea7e4853005a	0	0	2	0	0	0	0	0	0	0	0	0
e1d2c3484c592fbed848a4c04c896c2c	0	0	0	0	0	0	0	0	0	0	2	0
7cda2eabc75676918158106e54aca462	0	0	0	0	34	0	0	0	0	0	0	0

a8db13ede32b186e6234c692e9d76dfe	0	0	0	0	0	0	0	0	0	4	0	0
1d0111221ef0c803ca58808d8f71c3a0	0	0	0	0	8	0	0	0	0	0	0	0
6f4f5f5f059b12b7126af0094776fd87	0	0	0	12	0	0	0	0	0	0	0	0
cad0b40ab7d97b32d3ce08dd4e87f920	0	0	0	5	0	0	0	0	0	0	0	0
9cd14e9479a2172d9b83746b7164e39b	2	0	0	0	0	0	0	0	0	0	0	0
79eec5ae7b3ca12b9209857fd67f56e4	0	0	0	0	0	0	29	0	0	0	0	0
f8ece0561d1c6d4ab4129876ed40f8b0	4	0	0	0	0	0	0	0	0	0	0	0
63ef57bfefc4fcc34ac159843618bb83	0	0	0	0	0	0	0	0	0	0	3	0
e0dbbccdc4a86b05a4386e7a4f9268d1	0	0	0	0	0	0	0	2	0	0	0	0
243635494df32967b9f136be4535e3a6	0	0	0	0	0	0	0	0	0	0	3	0
91f79b3c877085a944ab744ba8876dcd	0	0	0	0	0	0	0	0	0	0	0	3
1c8ccd1a3a63a240ea51e9284b567a28	0	0	0	0	29	0	0	0	0	0	0	0
0c35b0c2c3c43220a1453b59d02d9595	0	0	0	0	0	58	0	0	0	0	0	0
05d40785a6c8fe29a25713c53a856a3d	0	0	0	0	0	0	6	0	0	0	0	0

e461be240a9e18e630a58e100180d1c9	0	12	0	0	0	0	0	0	0	0	0	0
7a5e6699d418d25050040acaaf166520	0	0	0	0	13	0	0	0	0	0	0	0
ef372de54bbb975851ede9fbd084628d	0	5	0	0	0	0	0	0	0	0	0	0
18eb14b076c1c2e1976158552727dfd5	0	0	0	0	0	0	0	0	0	0	4	0
e483bb993cc2e397effed36386ae5cb7	0	0	0	0	0	0	0	0	0	0	14	0
74129d3ee8e2c99816a10b9df6a776c4	0	0	31	0	0	0	0	0	0	0	0	0
d498825bf844d17db4cbdca818fef2da	0	0	0	0	0	0	0	0	0	0	6	0
03710761a5c7b67a1ab8dbecece8f22a	0	0	0	0	2	0	0	0	0	0	0	0
06551ae6a7885256b208180d36810d4e	2	0	0	0	0	0	0	0	0	0	0	0
d179c0162c973bebb1f519dec4708b09	0	0	0	0	0	0	0	0	7	0	0	0
b089c0ad69aec0e891f80bd6e05762ce	0	0	0	0	0	0	0	0	0	63	0	0
c2a885ca125c1e47e95afef0ccada791	0	0	0	0	22	0	0	0	0	0	0	0
31d27a59d4c9fdf7fe3136a2dd0e6327	0	0	0	0	0	0	0	0	0	0	0	3
9c809ea5640c18f39513702aebc021f2	0	0	0	0	0	27	0	0	0	0	0	0

3d738106e3a30ca0f0b45d2a5c44a753	0	0	0	0	0	0	18	0	0	0	0	0
2290fec1a98e2565ec6aca37938a4b87	0	0	0	7	0	0	0	0	0	0	0	0
f3b55c2d1e67bfd744037ef2c378fc17	0	0	0	3	0	0	0	0	0	0	0	0
d51a2b7d433e4a4d7053958b4aee6119	0	0	0	0	0	0	0	0	0	0	45	0
300128174187a8e691f7bacbed629bc8	0	0	0	0	0	0	0	0	0	0	0	3
7b9551abe0fef9784b8f9c40a151d462	0	0	0	0	0	0	0	0	0	3	0	0
12ad628e03e7ea4b7dce03490e12b727	0	0	0	0	0	0	0	29	0	0	0	0
029d32ffe69c06ff635bc4b9724cbdee	0	0	0	0	0	3	0	0	0	0	0	0
8214365b7246cc95d9e00096d9bb0c91	0	0	0	0	0	0	0	0	0	0	5	0
0c30b574b8f11d051acab1f7f50e09f8	0	0	0	0	0	0	0	0	0	0	0	3
4b76e38f31fe58d5e1fd9131b4735368	0	8	0	0	0	0	0	0	0	0	0	0
6d2743ec69d9fa7357895a61e90115b8	0	0	0	17	0	0	0	0	0	0	0	0
8f56963dd8eb033fcc45cb0bd5205eee	0	0	0	0	0	19	0	0	0	0	0	0
9d527dcf109f275fe13f24aa59967c1c	0	0	0	0	0	0	0	0	0	14	0	0

5f837abdc1a295eba5bee73e584d43fa	0	0	0	0	0	0	0	0	0	0	0	5
716954b89e74056a8af4e4f09f5dd349	0	0	0	2	0	0	0	0	0	0	0	0
d11fb52e888ad900447297e3232b350d	0	0	0	0	0	0	0	0	0	0	0	2
2eb96a410bc0276baf9699d823207541	0	0	0	0	0	0	0	30	0	0	0	0
554fa6d92826ecf2296d5645b4e67e7c	0	0	3	0	0	0	0	0	0	0	0	0
93cfdccbad6a2d70f58d6e4b41f58983	0	0	0	0	7	0	0	0	0	0	0	0
17ca192c11d97bdbaba3f164d305565e	0	0	0	0	0	0	0	0	0	0	10	0
f5e0b30163140c93b278c74207ab2633	0	0	0	0	3	0	0	0	0	0	0	0
6d20ba6036e263bfd4f40b7b6bfaf281	0	0	0	0	0	0	0	0	0	0	3	0
83876df86aff81cc01190244fd5ac5f3	0	0	0	0	0	0	0	0	0	0	0	9
8fd2b419275f1c47c36cfb5cd543f3aa	0	0	0	0	0	0	0	5	0	0	0	0
54a2a4eba96cf85212f9ede06bd30e95	0	0	0	0	0	5	0	0	0	0	0	0
0a9a8c6ce614172d49fd8c515f3e6174	0	0	0	0	0	0	0	0	0	0	3	0
62eff75eae45e151eb056e4fa09609ba	0	0	0	0	0	0	0	0	0	0	2	0

00269b93049b5b9a5bf7098997f731a7	0	0	0	0	0	0	0	0	3	0	0	0
0247579275a19f5a80dc46292f6643b5	0	0	0	0	0	0	0	0	0	0	24	0
8a63946148a6fa571df79c7c90d3882b	0	0	0	0	0	0	0	0	0	0	0	4
53f6cab339e55d5e08fc18109c5ef269	0	0	0	4	0	0	0	0	0	0	0	0
65e965708856e7abc25eb45ca463cee6	0	0	0	0	4	0	0	0	0	0	0	0
2eba04bf940083d21e054111fa286aa5	0	0	0	0	0	0	0	0	0	2	0	0
f78415552e053318f53324c4d1f7fade	0	0	0	0	0	0	0	0	0	0	0	6
e98e746f72e8adc01f5a340ff5ba7a66	0	0	0	0	0	0	0	0	0	0	0	125
f471a6e6b208ee279e2f5aa2d04958a9	0	0	0	0	0	2	0	0	0	0	0	0
28fb56ccbed22f083a94fec5a9a19e24	6	0	0	0	0	0	0	0	0	0	0	0
8581508304b26a7b8288d3a5bc4be92d	0	0	0	0	6	0	0	0	0	0	0	0
c41fb6d15dde45397ff68ab9821a7109	0	0	0	0	0	0	0	0	110	0	0	0
9624d7bf5fec09b478aa18c99fbf6cf4	0	0	0	0	0	0	0	0	3	0	0	0
ecbc138e15ab2e95f1df78e6e4a6eaa1	0	0	0	0	0	4	0	0	0	0	0	0

33cf17397d45be918a0fa833 63441f5e	0	0	0	0	0	0	0	0	0	0	2	0
9afab7162ec7c4469d9f49fff2 b66b03	0	0	0	0	0	0	0	0	3	0	0	0
5138778b1a1af2d0fa74837b 5eda75a0	0	0	0	0	0	0	0	0	0	4	0	0
3f41bc625d49d67ff2313f27b 0fece90	0	0	0	0	0	10	0	0	0	0	0	0
712801d451edcc5b191a922 b9fdd0ba5	0	0	0	0	0	0	0	0	0	4	0	0
1dc17fabffde19f44e3dd168f 03a1954	0	0	0	2	0	0	0	0	0	0	0	0
a894ca4584a9bd668ffa75bb 7658a390	0	0	0	0	0	0	0	0	8	0	0	0
b9e9d6e484210fb4bdbcfcdb 72b18729	0	0	0	0	0	0	0	0	0	0	0	6
253cdabec574a3227b52a590 f6f4d84a	9	0	0	0	0	0	0	0	0	0	0	0
933b6a08aa50815a7649411c 1ee53e39	0	0	0	3	0	0	0	0	0	0	0	0
2415c19e30902d178e7c9e17 b56e8519	0	0	0	0	0	0	0	0	9	0	0	0
88aeea9f9311f50f9f4990f75 808dc6a	0	0	0	0	0	45	0	0	0	0	0	0
c2c68030001ea55ace3e94a5 a8a559e5	0	0	0	14	0	0	0	0	0	0	0	0
e768c3f7233d09c10211b3cf a8fb0dfe	0	0	0	0	0	0	0	4	0	0	0	0

2890c400a676602733b90c04 68b1e48f	0	0	0	0	0	0	0	0	0	0	11	0
e7ffcd78896da3d4ad26bc86 c03c105c	0	0	0	0	0	60	0	0	0	0	0	0
4a0e508658acd499b50dfcac df6282b2	0	0	0	0	0	0	0	0	0	0	0	6
79e4ebfd24665656f02b58e6 a0618fdf	0	0	0	0	0	0	0	0	0	0	74	0
30c223e7fcb3c5718e447161 97180ac4	0	0	0	0	0	0	0	0	0	0	3	0
9bbdd74f885240d17e8fe8e3 96516ee6	0	0	0	17	0	0	0	0	0	0	0	0
168d1b015f058f31971a2d91 fcb424be	0	0	0	2	0	0	0	0	0	0	0	0
d403923f2c270ee0d53a391b 0a08d6bd	0	20	0	0	0	0	0	0	0	0	0	0
0ecb55c46c3ae382b4d98ecf 59194c42	0	0	0	0	0	0	0	0	0	14	0	0
6713f7ca19eadb2ffde75ccc0f 86a32c	0	0	0	0	0	0	0	0	0	0	26	0
d17584aa6069bb1ece55bca2 8c4d137f	0	2	0	0	0	0	0	0	0	0	0	0
34cac2d85c56f72cf2cfe6853f 72235d	0	0	0	0	0	0	2	0	0	0	0	0
a1cb552962194a6bc83a43f0 f1d6da06	0	0	0	0	0	0	0	0	0	3	0	0
da2dce7c4864e111c3dcf859 c705f5c0	0	0	0	0	0	0	2	0	0	0	0	0

feee33d85d6ac135d0503cfe89315b0f	0	0	0	0	21	0	0	0	0	0	0	0
0a2927b44edf792f7738b039a9c778c2	0	0	0	0	0	0	0	3	0	0	0	0
74354f43532fc0716a8b27fc647b51f5	0	0	0	0	0	16	0	0	0	0	0	0
33a9a5fb6e67347addbb1f5d28e3bb05	0	0	0	2	0	0	0	0	0	0	0	0
6ce5e89ab8178f59ce17be97589048a8	0	0	0	0	0	0	0	0	5	0	0	0
a79472b4b0d7e1bb24d529c5f75243ac	0	0	0	0	0	0	0	0	0	0	0	5
c6f4c199f1e1131328e5bf92edaf2cd8	0	0	0	0	0	0	0	0	0	0	33	0
2fb6a6eec4c9ec9728bc2fd6a23f2bec	0	0	0	0	0	0	0	7	0	0	0	0
409e9cfae7d72ec43ecf2f07323494fd	0	0	0	0	0	0	0	4	0	0	0	0
d2448c5ed0bc19848af9624c9b186907	0	0	0	0	0	0	0	0	0	0	0	13
f750db1d469e056f112bde0144255798	0	0	0	0	0	0	0	0	0	0	2	0
0c53ec5f22b3503b09abc8be7a542149	0	0	0	0	0	0	0	0	0	0	2	0
ee26c94b11062e4c7d017297ad46ab64	0	0	0	0	105	0	0	0	0	0	0	0
eae2fb16fa9c2c71e6fb88b6d524d82a	0	0	33	0	0	0	0	0	0	0	0	0

87c88cc9f6549deb827c880f0a360c64	0	0	0	0	0	0	0	42	0	0	0	0
4c708c2a967fbb76199f1a49eb4f62b8	0	0	0	0	0	0	0	0	0	21	0	0
499b43d6272a37765908883ae2c7155a	0	0	0	0	0	0	0	4	0	0	0	0
f55ccbfe047c87972a5898dff1c61ed0	0	0	0	0	0	0	0	0	0	10	0	0
932a440dfc847019e1bfa887f4f80614	0	0	0	0	0	2	0	0	0	0	0	0
d9c12ec68e95848809c5282386cc7325	0	0	0	0	0	2	0	0	0	0	0	0
8b12b05403334b001437c07ee2cb311f	0	0	0	5	0	0	0	0	0	0	0	0
945a8265bb114944abc0d115c70c9603	0	0	0	0	0	3	0	0	0	0	0	0
af5febe777bf308d5857bd017d2d5667	0	0	0	0	0	0	0	0	0	0	2	0
41762609bc6cdb228c584cdf261712b8	0	0	0	4	0	0	0	0	0	0	0	0
d002ece84cf45482bbb7e7849366ed95	0	0	0	0	0	0	0	0	0	29	0	0
06347b2d96e9b4bdd92f6f3727fec99d	0	0	0	0	0	0	0	0	4	0	0	0
e21c1e79d35f2815dd516272573c74a0	0	0	0	0	0	0	0	0	0	0	3	0
daecd7d175bfc346de01bb71fd79d074	2	0	0	0	0	0	0	0	0	0	0	0

f54c50aa6c66d346ec1f35702f7eb198	0	0	0	23	0	37	0	0	0	0	0	0
c589451dd198dc6115d2423a5477beb3	0	8	0	0	0	0	0	0	0	0	0	0
1140965c0b8c47a65bf13807b7cbbf19	0	0	0	0	0	0	0	0	0	0	2	0
24511e8ecf7dafa5234c3a90b4a9bfa8	0	0	6	0	0	0	0	0	0	0	0	0
1d918d2a150b37f162ad85a9ea407edf	0	0	0	0	3	0	0	0	0	0	0	0
4a630605329eaff32c7d73bccd910fdb	0	0	0	0	0	4	0	0	0	0	0	0
27727988a0da2b6043bfccbc33aa6edb	0	0	0	3	0	0	0	0	0	0	0	0
406dfc38ea3c17cac4324a064b3f85e7	28	0	0	19	0	0	0	0	0	0	0	0
29028b5a47c6bb5bfe9dfa6db687900f	0	0	0	0	0	0	0	0	0	3	0	0
e80dc88c7d1e3379551287c7fbbd67f3	0	0	0	0	0	3	0	0	0	0	0	0
50d158255d06ebf04e3c3773d3b1ee2d	0	0	0	0	0	218	0	0	0	0	0	0
9a6b4afde0213b8a81155b2623ec2f0c	0	0	7	0	0	0	0	0	0	0	0	0
23a9d18f8a555a00ab02ffec63561d3d	0	0	4	0	0	0	0	0	0	0	0	0
c153972c6b15108a3b5075b10cbe28df	0	0	0	0	0	0	0	0	0	2	0	0

d9e7b78fc1f1e31f971e1a4548788960	0	0	3	0	0	0	0	0	0	0	0	0
88e87ccd6cbe4afa2318043252686025	0	0	0	3	0	0	0	0	0	0	0	0
dbf139dc2a6aaf9b2368cecc08b6cbbb	0	0	5	0	0	0	0	0	0	0	0	0
4e78a74f5e41fc4868bec08c5ddb5c29	0	0	0	0	0	0	0	0	0	0	23	0
5347ae61f8c8e887ad725103cd0282fb	0	0	0	0	0	0	0	20	0	0	0	0
d234d4e1c23d36ca448e5e694a7f684f	0	0	0	0	0	0	0	21	0	0	0	0
3da3f6522f6a6523ac0fe2cc99c4e3da	0	0	0	0	0	0	0	12	0	0	0	0
0f0fb24bb325dd83517dea4db521c41b	0	0	0	0	0	0	0	0	0	0	18	0
b2d90efba3e8d5b517974e80535c3b7a	0	0	0	0	0	0	0	0	0	0	0	2
e44d1cf30f179910b85d778e8ae4d2f8	0	0	0	0	20	0	0	0	0	0	0	0
821b75902ee66f2ce9842983e8001242	0	0	0	0	0	0	0	0	0	0	5	0
aa80ec61c3834346f22da8c4a01ee7ba	0	0	0	0	0	0	0	0	0	0	0	3
184563eed34d2dce951f47cae1068703	0	0	0	0	0	0	0	0	3	0	0	0
f6e8dbe854a80dfe0c84da855c6114c1	0	0	0	0	0	0	0	0	0	30	0	0

1bc29edf1091b10843156aec2bd6b94d	0	0	0	0	0	0	0	0	0	0	5	0
91e703d4d7fb72be6517f831c3e88e53	0	0	0	2	0	0	0	0	0	0	0	0
1b4bfaae76b5690d7334fa7c66ddcecf	0	0	0	0	0	0	0	0	0	0	2	0
3cbc42ae9459eebe830797d407b50f7a	0	106	0	0	0	0	0	0	0	0	0	0
9c03badac27d1b907b131842312c1b82	0	0	0	0	0	0	0	0	0	0	0	8
5ebef632fe69a66cf70763678eef60e8	0	0	0	0	0	0	0	0	0	0	0	3
488a6ccb811fea3429afa311b41298ad	0	0	0	0	0	2	0	0	0	0	0	0
aa7e3d109fcbdc1f3c79968ef6d1f4c8	0	0	0	8	0	0	0	0	0	0	0	0
89f2d5b88b54e810053dc1e5322b0849	0	0	0	0	0	0	5	0	0	0	0	0
a3894480f86070e15d3a813f37bacb1f	0	0	0	0	0	0	0	0	51	0	0	0
29bd39683e5e9845143bae37488c0582	0	0	0	0	0	8	0	0	0	0	0	0
2a4f0473a4a0b32825b13c4cf1950df8	0	0	0	0	0	0	0	57	0	0	0	0
d2a9fb7d0c49ed162afd78a6829651fa	0	0	0	0	0	0	0	0	0	0	3	0
a94cdb2404f2ae2687d254d4ac15e1ae	0	0	0	0	2	0	0	0	0	0	0	0

80f49f7935a44b5f1fc933861 8c0ad56	0	0	0	2	0	0	0	0	0	0	0	0
8d439eb7009035ea75a8382 1d245f483	0	0	0	5	0	0	0	0	0	0	0	0
a093d5d4a256477fe568852f 4c08c2c3	0	0	0	3	0	0	0	0	0	0	0	0
46af4fe5c1f37bfea510d363a aa313b3	0	0	0	0	0	0	0	0	6	0	0	0
b8a8c2c997b85b20e45fba5d c52b5cec	0	0	0	0	0	0	0	0	0	3	0	0
c1deb975da8d6236c17aec76 2fa97928	0	0	0	0	0	0	0	0	0	11	0	0
6ebe284b8d94eb190f23937f acc72bf4	0	0	6	0	0	0	0	0	0	0	0	0
9fe346013dfa637cda2bef053 5a3cfd2	0	0	4	0	0	0	0	0	0	0	0	0
37a75962b7766e928713625 5f448b449	0	0	0	0	0	0	6	0	0	0	0	0
407483dd4c489d4cd0d97e0 b4b97dcbd	0	0	0	0	0	0	0	0	0	0	3	0
f6dddbe3713560876050170 4fba9d1c8	0	0	0	0	0	0	0	0	0	2	0	0
723952aca1409db3bec643f4 5177bc5b	0	0	0	0	0	0	0	0	0	0	0	2
c11b2204e20f113735f3c0fa8 1d6945c	0	0	0	0	0	0	0	0	8	0	0	0
14d372cd7e708a2b1b17bde c9f9ee45e	0	0	0	0	0	0	0	0	0	0	0	2

4e575ba49d515e50bb93dca07abbedc2	0	0	2	0	0	0	0	0	0	0	0	0
2482c84dcac669ec482a08ebcfe73866	0	0	0	0	0	2	0	0	0	0	0	0
95b73a8cdb4d7614dd632ae19e515e44	0	0	0	0	0	0	0	0	0	0	0	4
262630d845e42142d748990246b24fd4	0	0	0	0	6	0	0	0	0	0	0	0
f13f4daef4da40fd2875f9ceba862ada	12	0	0	0	0	0	0	0	0	0	0	0
44d95ea4e9e0191de855460631a3ad33	0	0	90	0	0	0	0	0	0	0	0	0
1c521343c650568906ff232bbb6ee998	0	0	3	0	0	0	0	0	0	0	0	0
2a5720678af2e4825d258dd3ff9aa188	0	0	0	0	3	0	0	0	0	0	0	0
6a996a29bc21a2ba11961e0ace9b003e	2	0	0	0	0	0	0	0	0	0	0	0
5ede1c69994b89c725990ea9b6d2b7c8	0	227	0	0	0	0	0	0	0	0	0	0
f724bb9cba56f321101fadc6858060a8	0	0	0	0	0	0	0	0	0	3	0	0
ad1749ec68c0538e0c238d3f6b356ba1	0	0	0	0	0	0	0	0	0	0	0	2
d85b37715e67a6313bbba16f30cb2b1f	0	0	0	0	0	0	0	72	0	0	0	0
7bc2afbda00a6b5c8afbd6ea95ece6dc	0	0	0	0	0	0	0	0	0	0	4	0

a739382d208b2051f7441c48bcb561a6	0	0	4	0	0	0	0	0	0	0	0	0
b4e82c1ae2499f69acaff383565f7b46	9	0	0	0	0	0	0	0	0	0	0	0
ccef997da212b0ce1342c2c462c183de	0	0	0	0	0	0	0	0	0	0	4	0
bddc999c29491d068a119b9c2f40cbe9	0	0	0	0	113	0	0	0	0	0	0	0
48039cd3f3bc2802bcadd3e150a591c8	0	7	0	0	0	0	0	0	0	0	0	0
c243906c14251256aae0b0cb6cd704e6	0	0	0	0	0	0	0	0	0	0	0	24
28658aa923f3b23cc97141f37687a97c	0	0	0	0	0	0	0	0	0	2	0	0
6eb079dd0ab72517720a3e3d119480d1	0	0	0	0	0	134	0	0	0	0	0	0
5b5131da4073a23d7374c0773574a0b4	0	0	0	0	0	0	0	0	8	0	0	0
4fc42966ca59fc73ae694eac442c9786	0	0	0	0	0	0	0	0	0	21	0	0
88384765e87595fcd3a0c439eb79b4a5	0	28	0	0	0	0	0	0	0	0	0	0
5bef2d3a715818c6b46c8751baa9a5bc	0	0	0	0	0	0	0	12	0	0	0	0
0701ca5f75e5965533f37efdc3d620bc	0	0	0	0	0	0	0	18	0	0	0	0
cf5ea3a947c8416614c82f06a4db5d3e	0	0	0	0	0	0	0	0	0	0	0	14

1163d26e2a022a1c515b8bcedef122ca	0	0	0	0	0	0	0	0	0	0	6	0
0437aa02485580fe1b7052485fbe5dc8	0	0	0	0	14	0	0	0	0	0	0	0
e209e9024d20f39cd6b6e0a682de729b	0	0	0	16	0	0	0	0	0	0	0	0
3d206a6c1b91729ad24febf0aef6a2ae	0	0	0	0	0	0	0	3	0	0	0	0
34a3063628eaf275fde6d19c85e942d9	0	0	0	0	5	0	0	0	0	0	0	0
e1326db871048be713e709f4ea14b668	0	0	0	0	0	0	0	0	0	0	3	0
177a7990aba9882d7449558713945d75	0	0	0	0	0	0	0	2	0	0	0	0
0bb2cccef31dd7f5a6a487701a035291	0	0	0	3	0	0	0	0	0	0	0	0
e3ac0ef40eb7628699ad103ea8606f12	0	0	0	0	0	0	0	0	0	0	0	2
94016e3745c5ebe866d65922411f437d	0	0	0	0	0	0	4	0	0	0	0	0
d1fdac2348aca99318d20f2d94f53eeb	0	0	0	0	0	5	0	0	0	0	0	0
c52739427122f94d108cb0da56831d31	0	0	0	0	0	0	0	0	0	0	0	10
8a403003ffa5cf05ca18c31e7dfc5ac6	0	0	0	0	0	0	0	0	0	0	0	14
e594d373b7948a1e252fab18fddcb5dd	0	0	0	0	0	0	0	0	0	2	0	0

e44f42408dacad0c8ccb349bcab26e3f	0	0	0	0	0	0	0	0	0	0	0	2
5d6663a07ce8c39082a3314d430ce075	0	0	0	0	0	0	0	0	0	0	14	0
2dfacd4b03f2feb9a86f644923e622b7	0	0	0	0	0	0	0	0	0	0	0	8
2f04d4c25c8ff1c5c2b467e3221bb3e7	0	0	0	0	0	0	0	0	0	0	0	5
8fabf12d5505fc141efdc04d82eb678d	0	0	2	0	0	0	0	0	0	0	0	0
2219c3203f6c0181c111cf796216cc89	0	0	0	0	0	0	0	0	0	0	76	0
a04f903c24b3d0ee9ff28bc584dfc5ca	0	0	0	0	0	0	0	0	0	0	0	2
ba5e3b044fd60436a47553ea8a056a87	0	0	0	2	0	0	0	0	0	0	0	0
d9789d43a07333da2afcd6dcad300d3b	0	0	4	0	0	0	0	0	0	0	0	0
594672aa3c04537b1dd98cbec7a9d20e	0	0	0	0	0	8	0	0	0	0	0	0
5dcb24fd3be33fd60208cfc59ca017cb	0	0	0	0	0	0	4	0	0	0	0	0
595c0bbb75b4ee003df0fb6af50d2cfc	0	0	0	0	7	0	0	0	0	0	0	0
e93d095cfb2e2b525fcf9cdeb747e5ed	0	2	0	0	0	0	0	0	0	0	0	0
e0fa4a6ad324f931ec479dc2e7007f67	0	0	0	0	0	0	0	0	0	0	6	0

f99bd6ee4c2ed05418016316e10c2e03	0	0	0	0	0	0	0	0	0	0	0	5
02c710990d1df9e4e66fb0cdff62e35a	0	0	0	0	0	0	2	0	0	0	0	0
284b794537c15b0cf1b30abdb3ec1b11	0	0	2	0	0	0	0	0	0	0	0	0
747502d2fc9893e63b0addd374109ec4	0	0	0	0	6	0	0	0	0	0	0	0
8532d0e3b730181cc56663c467f85d01	0	0	0	0	0	0	0	0	0	0	0	3
468c7086a918d69155d671c2ed887ac7	0	0	4	0	0	0	0	0	0	0	0	0
efb5ad21e40b1aa7134cd891276514da	0	0	0	0	0	0	0	0	0	0	8	0
005c25ba85471f57d5d18d440d587b23	3	0	0	0	0	0	0	0	0	0	0	0
79222e582bafe42044556fe3bede4936	0	0	0	0	0	0	3	0	0	0	0	0
cadb25efbab88326324e79c17c8c35be	0	0	0	14	0	0	0	0	0	0	0	0
50dd9d6a216f04bc213232a5cad25226	0	31	0	0	0	0	0	0	0	0	0	0
928208ec9918d8c02a2673ca432a70fd	0	0	0	0	0	0	0	0	0	0	4	0
89ef28597ae420dce825ddc31abdf7c5	0	0	0	0	0	0	0	0	0	0	0	3
85276bc632924329f4929505d07e8d8f	0	0	3	0	0	0	0	0	0	0	0	0

98df911329ae0721b8380568e4119c9c	0	0	0	0	0	0	0	0	0	31	0	0
1617563194dfe119a52af0c381cf2975	0	0	0	0	0	0	0	2	0	0	0	0
2869ecf4160c2aa7892dd9f80e6c99b4	0	0	7	0	0	0	0	0	0	0	0	0
0c1484885f107cd457c72bb112c44db3	0	0	0	0	0	0	0	0	0	0	11	0
2b43fb8d439e020ec34c9ff39ee291d2	0	0	0	0	0	0	21	0	0	0	0	0
6da812ea720278ba6129620844f1d2bf	0	0	0	0	0	0	0	0	0	3	0	0
fd8de924cfe50bb3cf0ac476b471f1e4	0	0	0	0	0	13	0	0	0	0	0	0
1c20fa3b52810bff4cf1cfe04e98ced7	0	0	0	0	43	0	0	0	0	0	0	0
2870b91ff4aad6f279eba43ddd680fa6	0	0	0	0	0	117	0	0	0	0	0	0
6d7af68691d6eb84d9089d6ee41dcfc8	0	0	0	0	0	0	0	0	0	0	0	9
28d8d0195087f55f62f05fbf948c7e64	0	0	0	0	0	4	0	0	0	0	0	0
70511c151256562136a9981e103ac070	0	0	0	0	2	0	0	0	0	0	0	0
e1f1db1ae007d4b859de902e41efc807	0	0	67	0	0	0	0	0	0	0	0	0
47104fb91f8ef52151ad22d04c7965af	0	0	0	0	0	3	0	0	0	0	0	0

da0c669eb3ae81f5f58907126ae2b0a0	0	0	0	0	0	0	0	0	0	3	0	0
880e77039810bcf0a7ac16af406207da	0	0	6	0	0	0	0	0	0	0	0	0
a7f162f523714783f0308ecb2d1ccf32	0	0	0	0	0	0	0	0	0	0	0	4
5f3e268c58afb155c95e23845605efe0	0	14	0	0	0	0	0	0	0	0	0	0
9bbba2322b34cf5e39584099317f8ba6	0	0	0	0	0	2	0	0	0	0	0	0
a2577dfe606b799def238974293588c9	0	0	0	0	16	0	0	0	0	0	0	0
0c9d732988253c5926ae8f4d9f592e18	0	0	0	0	0	0	0	0	0	0	21	0
6c51e074991d387423b4ebe89e21874a	0	0	0	32	0	0	0	0	0	0	0	0
a39e348896ae9d97f0c2aab80474a5bb	0	0	0	0	0	0	0	0	50	0	41	0
c8ea2b188278230e0cbcc46d8656cb4a	4	0	0	0	0	0	0	0	0	0	0	0
2839552b23faaffca5c24906577d0fb5	0	0	0	0	0	2	0	0	0	0	0	0
40359f229e7673fb6bfffba52d45edeb	0	0	0	0	0	0	0	0	0	3	0	0
000837a6fac8f13ca0ab61376ec92970	0	0	0	0	35	0	0	0	0	0	0	0
235fd3899eb84e26d293575219caae5c	0	0	0	0	0	0	0	0	0	0	152	0

31a8e05eeeb5fa8fbeed7ae9ad6eda18	0	0	0	0	0	0	0	0	0	3	0	0
e170e98cf8e5f175ec3894ac3ddeb240	0	0	0	0	0	0	0	6	0	0	0	0
5c0791dd4d9208f737d3e4e2c1fe016d	0	0	6	0	0	0	0	0	0	0	0	0
ae89367ea1e029538815ce31312ec24c	0	0	0	0	0	0	0	7	0	0	0	0
5e6bb63d575de792fd774da978ed5ab0	2	0	0	0	0	0	0	0	0	0	0	0
aeebf5ec8d7f88a44c05f99c99ae9472	0	0	0	0	0	0	0	0	3	0	0	0
b183c80dd7ea7b212412234bb9b142bd	0	0	3	0	0	0	0	0	0	0	0	0
133321f058279ed2accae9c132c78063	0	0	0	0	0	0	0	0	0	6	0	0
de16880f94a4dc91fceb49951747ff07	0	0	0	0	0	0	3	0	0	0	0	0
fcf3e3bf054718281e6826e42f570a9e	0	0	0	0	0	2	0	0	0	0	0	0
5e6630ab5df0a0ff18e354a1bf0f8ac1	0	0	0	0	0	0	0	0	0	0	0	4
37180b2459346e68e194cc02f52dca22	0	0	0	0	0	0	0	0	0	3	0	0
c044a059c492bab274aeb6ba1ca4969a	0	0	0	0	0	0	2	0	0	0	0	0
4e1e9759beb57a14fd7230de266bce12	0	0	0	0	0	0	0	0	3	0	0	0

92d356cddbbed0387e1d764308a1b50a	0	0	0	0	0	0	0	0	0	0	0	10
e59b91310a6baa0dafd48ad2d08b8377	0	0	0	0	0	0	0	0	5	0	0	0
8894026222b5a7181c02cb3a0ad44292	3	0	0	0	0	0	0	0	0	0	0	0
59460ef694ba76c3de7b8942a2ee919c	0	0	0	0	2	0	0	0	0	0	0	0
b18b282ccb922e388b70154061cd7ba5	0	0	5	0	0	0	0	0	0	0	0	0
ec999766a3181410594fe07f408fe7da	0	0	0	0	0	0	0	10	0	0	0	0
44d918a149dc526e5a6ff21a712163d6	2	0	0	0	0	0	0	0	0	0	0	0
e8e608438781e33946c4966cf3238f4c	0	0	0	0	0	0	0	0	0	0	0	17
ffa07a6a1ce85a4fd00f253d07ee58d7	0	0	0	0	0	0	0	0	0	0	2	0
ffd1f8838ba2ca5164c04893663e5c83	0	0	0	0	0	0	0	0	11	0	0	0
fd91d0f3d5152c773fa80b314546fe07	0	0	0	0	0	0	0	2	0	0	0	0
b40c8590058f48f0dbcdb7c24965f7b2	0	0	0	0	0	0	0	0	2	0	0	0
45fa94f10daea6811e6edd82ba1e42a3	0	0	0	0	0	0	0	0	0	0	3	0
43c0e3e538ad79cd6cabf4ba3ebe8c08	0	0	0	0	0	0	0	0	0	2	0	0

d1384141e25c799b7bf07baa02cb000c	0	0	0	0	8	0	0	0	0	0	0	0
db4a8fd2becb0ec1d3eeb86ab117692a	0	0	0	0	0	0	0	0	0	0	8	0
38a0fe2029fe375fba2d95195bec36cc	0	0	0	0	0	0	0	0	0	0	0	2
c16dbb5e1b196c0552cc7fa2c618878b	0	0	0	0	0	0	13	0	0	0	0	0
ae462d1adb481e8e4d5c8a2a2d141045	0	0	0	0	0	0	25	0	0	0	0	7
ad066580da48df8fbf18b123f7fe55ba	0	0	0	0	0	0	0	0	0	0	0	2
d51ebd6ca3b4e12ba18da16378fd25ab	0	0	6	0	0	0	0	0	0	0	0	0
61079060e8f3ec1fc6b1e691149ea9b7	0	0	0	0	0	0	0	2	0	0	0	0
26090717aafd038ee562a9f92a82dbc1	0	0	0	0	0	0	0	0	0	0	5	0
4d272852d1b4fb323a8c6bcc3f0e2308	0	15	0	0	0	0	0	0	0	0	0	0
d154502f31115c9f64742ea2a4a8a455	0	0	0	0	0	0	0	0	0	0	245	0
b6e6d16124a1b2dcecd738553c692334	0	0	0	0	0	0	0	0	0	3	0	0
68a52a19bb9d6ec8ae057e4cbea0d4c0	0	0	0	2	0	0	0	0	0	0	0	0
e34f9e9dcddf31a69a023fef78696290	0	0	0	0	0	0	0	0	0	0	5	0

8b3c53e11add8936f6567b4bf6b0507d	0	0	0	0	0	0	0	0	0	0	6	0
fbc4bb658dd9f3979f3a682887d62b92	0	0	0	0	0	0	0	0	0	0	7	0
5529fc67017db7cb7c81c466bba955c0	0	0	0	0	0	0	0	0	0	0	30	0
b1a4cebd893a350652ff1bc3b03200f0	0	0	0	0	0	0	0	65	0	0	0	0
ce7ca9164237862ed05cb9578f418d96	0	0	0	0	0	10	0	0	0	0	0	0
447e379f9634f9cf61c95e123d61ddc8	0	0	0	0	0	24	0	0	0	0	0	0
e360fb20a9fba4a7f20decc01b49f8d3	0	20	0	0	0	0	0	0	0	0	0	0
24b116cb181ab71fe41d750f0a2d13d0	0	0	2	0	0	0	0	0	0	0	0	0
1d7e5b5a3b9e458dbac690bc2dbb77d3	0	0	0	0	0	0	0	40	0	0	0	0
f8cda785bb4804735fadab62d10af707	0	0	0	0	0	0	0	0	0	0	5	0
c62d35afb1d0b96538cdafd849a31070	0	0	0	0	0	3	0	0	0	0	0	0
7186abe2bb679919a178beeb36241939	0	0	0	0	0	0	0	0	0	0	5	0
9fc0c1f815847db8bbcc0b8ef880b6e5	0	0	0	0	0	0	0	0	0	0	0	13
d0e7ad1f8b0449d6a38ce6ce2ffe44bb	0	0	0	0	2	0	0	0	0	0	0	0

10a9fc66901e97c146f32299b3ba7b0b	0	3	0	0	0	0	0	0	0	0	0	0
8631eb2f48447a0fb9dad328ac4a9921	0	0	0	0	2	0	0	0	0	0	0	0
d9860fd861aab2e9109c2f7e9b87b20f	0	0	0	0	2	0	0	0	0	0	0	0
57b029dc7db67fcd2f09e1c02bd31b04	4	0	0	0	0	0	0	0	0	0	0	0
908adf9d2e0fbb0428a0588fff716af5	0	0	0	0	0	3	0	0	0	0	0	0
eec65c28305a2cbf279f16df73bc0cf5	0	0	0	0	0	0	0	0	0	0	3	0
6addb1057de60b7911fdb64fcdd28838	0	0	0	0	0	0	0	0	0	0	17	0
eade561893ece1e734d1fbec07b52dee	0	0	0	0	0	0	0	0	0	0	11	0
1bc2c0a275d1d57040fbd957d596c3e5	0	0	0	0	0	185	0	0	0	0	0	0
2adff00fe826950606926da77c9a42a0	0	12	0	0	0	0	0	0	0	0	0	0
9a209bc80717f492ce6baa564aadad43	0	0	0	0	0	0	0	0	2	0	0	0
fec27d41041c54a15a80f0b9d348a66f	0	0	0	0	4	0	0	0	0	0	0	0
b3505d42495f4d2ca0e71bfaf45cec0f	0	0	0	0	0	0	0	17	0	0	0	0
5702b15ad016359761c5812d30801847	0	0	0	0	2	0	0	0	0	0	0	0

6a3ae78d968be0c234bbf159 6c0fda53	0	0	0	0	0	15	0	0	0	0	0	0
28803c88f37ba7f5ef7c25c49 88e46b5	0	0	0	0	0	0	0	0	4	0	0	0
d35aa2b3d7801622e30cfd37 e2f5778b	0	0	0	0	0	0	0	0	0	0	2	0
d0a5629d0af47de403ee3f34 23766f39	0	0	0	0	0	0	0	0	0	0	41	0
a3fa49e91ecc4c82c61564b7 652f5178	0	0	0	0	7	0	0	0	0	0	0	0
6417558cdf1cd65e96b20903 42194b0e	0	0	0	0	0	0	2	0	0	0	0	0
b3034ce9102bd9649fb3771d 07674356	0	0	0	0	0	0	0	0	0	0	0	104
5445b9a6081af866aad581e5 d0b8e24e	0	0	0	0	0	0	0	3	0	0	0	0
76e8bf133e70f21b22f39364 77a89543	0	0	0	10	0	0	0	0	0	0	0	0
38c49c86f2ef55124df70a92a 53dedbb	0	0	0	0	4	0	0	0	0	0	0	0
1dda3ce09005366f7f544edb 4f8a08fc	0	0	0	0	0	0	0	2	0	0	0	0
fa481f1e9863364329d31acaf 6ce69db	0	2	0	0	0	0	0	0	0	0	0	0
c6cf6c880466f91b45cacde8f be95dfe	0	0	0	0	4	0	0	0	0	0	0	0
ebf07ab1d8e69599ba4e2d45 681dbc9c	0	0	0	0	0	0	0	2	0	0	0	0

9adc66b0e632bc743ac8881ac27ce7fe	0	7	0	0	0	0	0	0	0	0	0	0
b345326f9be49980d390f4c5b273562b	0	0	0	0	0	0	0	0	0	0	0	14
fe3c9483b180d73ca820e03a660f05c9	0	0	0	0	0	0	0	0	0	99	0	0
bed3b3b34948aefcbf5bb6a08223b682	0	0	0	0	0	0	0	7	0	0	0	0
b36d62b43e7f1f19a365c7207e983504	0	0	0	0	0	0	0	0	0	0	0	2
c307103b5362046f640ecbe78c8860df	0	0	0	0	0	5	0	0	0	0	0	0
3a78c7e42aca92bc053d93cd4a6a37ed	0	0	0	0	0	0	0	0	0	0	0	13
5f6e9a86e26cda6b297459e36277cebc	0	0	0	0	0	0	6	0	0	0	0	0
64c42a32dd307b064485e80ded9e5a06	0	0	0	0	0	0	0	7	0	0	0	0
b47a2f1b50793f78328f6719c96a50c0	0	0	0	0	0	0	0	10	0	0	0	0
d693fab10b13dbc395ee0a8a3911a39b	0	0	0	0	0	0	0	0	15	0	0	0
3eb9cc3f349a28c2ed0c91e2bbcf651a	0	0	0	0	0	0	0	0	0	0	21	0
224165e4c03177be83de76cd188b0d3e	0	0	0	0	0	5	0	0	0	0	0	0
a490079e1fe3ae0f270895583aa4203b	0	0	0	0	0	0	0	0	0	0	160	0

be3a4a1f91eff79a4ba0d76fbc18e889	0	0	0	0	0	0	0	0	0	17	0	0
0e3223a565f51d5935b4717a7d1d340e	0	0	0	0	0	0	0	0	10	0	0	0
f209a6b524903883d4f70f978e218c39	0	0	0	0	0	0	0	162	0	0	33	0
309564ec8f640ba44dc4991649659915	5	0	0	0	0	0	0	0	0	0	0	0
ccf51609f31c007a8345cb54b6798cd8	0	0	2	0	0	0	0	0	0	0	0	0
fbd998ade1022dad3737e33c3edd1e27	0	0	0	0	0	0	0	0	0	0	0	4
86d909853ba133b3a2e936effc6e53dd	0	0	0	0	0	0	0	10	0	0	0	0
a18f85617ca0a8b4af81131486b01016	0	11	0	0	0	0	0	0	0	0	0	0
09d5d3ee15f028f5e398a4cc452b6d5d	0	0	2	0	0	0	0	0	0	0	0	0
ffdc307c21869623be987d22b6155949	6	0	0	0	0	0	0	0	0	0	0	0
6b1a9fafb2680ef21ec8849201a71b80	0	0	0	25	0	0	0	0	0	83	0	0
551c3f367806c8d1d560e2715687c199	0	0	0	0	0	0	0	0	0	0	0	2
ce11a8ce1ef8e201d0df15f8384a068c	0	0	0	0	0	9	0	0	0	0	0	0
e874e03024e490ac126a961e2ade9798	0	0	0	0	0	0	0	0	0	0	17	0

ee52f7ae50bf240dd9cc80db2c4858b1	0	0	4	0	0	0	0	0	0	0	0	0
347f35c0bc196866c0a971c86006502a	0	0	0	0	0	4	0	0	0	0	0	0
5cd90bc8cf1713ef1c68dc5efa228531	0	0	0	0	0	0	0	0	2	0	0	0
51702bfe87388c78f60ffadbb1dfa1f1	0	0	29	0	0	0	0	0	0	0	0	0
0f72ad14a574af5d891567cdcd1bb92b	0	0	0	0	0	7	0	0	0	0	0	0
5f86f0567e3fdf7c7136de7b886e9654	0	0	0	0	0	0	0	0	0	0	0	35
b38cb5828c3ef472fd9b6a3bf95b325b	0	0	0	6	0	0	0	0	0	0	0	0
fe8adbaac2c8f024d6a0668dce55edd9	0	0	7	0	0	0	0	0	0	0	0	0
cbcf02a634e9e4bfe9f7744be762ab2d	0	2	0	0	0	0	0	0	0	0	0	0
a8beedb806afebaa068e4a21b6911227	0	0	0	0	0	0	0	3	0	0	0	0
3be176d16ccd4d3749a5860906193784	0	0	0	0	0	0	2	0	0	0	0	0
408a621a091e1b5b3fda6dea0af6bffe	0	0	0	0	0	0	0	0	0	0	7	0
7d478b6189aa209950bb55d165ad4893	0	0	0	0	0	0	3	0	0	0	0	0
8b8fed17718581c63389cead96a5aa83	0	0	0	0	0	0	7	0	0	0	0	0

e6a9daf3aa13215964ede5636f3cf3be	0	0	0	0	0	0	0	0	0	0	4	0
7fe32648a7631e9a82bd4d27f4db12b0	0	20	0	0	0	0	0	0	0	0	0	0
6faccfa04fb4a4ec094f24edd4cea6a2	0	0	3	0	0	0	0	0	0	0	0	0
917bcc26a00fa11232992b6c6a67b786	0	0	0	0	0	0	0	0	0	11	0	0
8b728ec807911b89aa657801822a1d7d	0	0	0	0	0	0	4	0	0	0	0	0
2f801a27175d0f91f559ade845070f0d	0	0	0	0	0	0	0	0	0	0	51	0
b2a6797c3df532ebd70932ec8fe0a73b	0	0	3	0	0	0	0	0	0	0	0	0
70919789c9064f46168aed07eff54b29	0	0	0	0	0	0	0	0	0	3	0	0
84d383af0d3bb0ba5a249f29b6914fa3	0	0	0	0	0	0	0	0	0	0	4	0
071c2c3f86af35ed209d8d472aa4380a	0	0	2	0	0	0	0	0	0	0	0	0
367d76f6368f4f1785dbbfcc828cc444	0	0	0	0	0	0	0	0	37	0	0	0
6adb674846641f6617ea498b2d14fd96	0	2	0	0	0	0	0	0	0	0	0	0
ab31b99b43b179d158ca972534fc4c0c	0	0	0	0	3	0	0	0	0	0	0	0
5f0d4f1827defbf363ddfedf2ee39f7a	0	0	0	26	0	0	0	0	0	0	0	0

056f66212c66a5655346473c6a8923b0	0	0	0	0	0	0	0	0	0	0	8	0
addf44e0992f036d0a7332ffc11582a7	0	0	0	0	0	0	0	0	2	0	0	0
9eab093e059809bf736839cc5dcb35a0	0	0	0	0	0	0	0	0	0	0	0	4
287e921505433475df412faa54f439c7	0	0	0	0	0	0	0	0	0	52	0	0
48f3538ab18414953803df0cfb911aea	0	0	0	0	0	0	0	3	0	0	0	0
4f1d123758ac2a3f1d4c55220953485b	0	0	0	0	2	0	0	0	0	0	0	0
654a513e485d06f3258cf0b63387e18f	0	0	5	0	0	0	0	0	0	0	0	0
6397f0627e45d8d69cfbadc9e8f9b609	0	0	0	0	0	0	0	0	0	2	0	0
e31209341731f33e7dc7be0c73f36879	0	0	7	0	0	0	0	0	0	0	0	0
f79ef0019e5d1157cb69c3e7364e5ddf	0	0	0	17	0	0	0	0	0	0	0	0
433ec1ebb3736cded8eb5560f1848eba	0	0	0	0	0	0	0	0	0	0	0	132
0689de50a95701aaa365667babc661ce	0	0	0	18	0	0	0	0	0	0	0	0
3a4d832a7c2f6a0a19a4c7ff0b7348a6	0	0	0	0	0	8	0	0	0	0	0	0
79a1540c8dd68dfc6ddaa60f9b36d437	0	0	0	0	0	0	2	0	0	0	0	0

892bc2a42dd775cba5316bca1849d462	0	0	0	0	0	0	0	2	0	0	0	0
65069635459f8468094f0e4e2fdf1eeb	0	0	0	0	0	0	0	0	0	0	3	0
d7494ccd44ac07c17cb3c2fa2591c604	0	0	0	0	0	16	0	0	0	0	0	0
98a92f8026050d23a7209080a8fd0cfc	0	0	7	0	0	0	0	0	0	0	0	0
d8d10c46e60fd69255f88207f943e501	0	0	0	0	0	0	0	2	0	0	0	0
55737477b7ff43dd1f73f5843aba47b5	0	0	0	0	0	0	0	0	0	20	0	0
e63297e91cda67648f473f7935fb5a33	0	0	0	0	3	0	0	0	0	0	0	0
03f8dfa60ec3ae4dce34ea25cdf81f1d	0	0	0	0	3	0	0	0	0	0	0	0
819098e503e98a2abf0f6c7e07ba96db	0	0	0	0	0	0	0	0	0	0	0	2
0407591df8c3ca85feb1d68a02700238	6	0	0	0	0	0	0	0	0	0	0	0
ed202d71cf9fc86810c2a6edb16bcd04	0	0	0	0	0	0	0	0	0	0	0	5
87fb55930eb302431ae5a17cf7409b76	0	0	0	0	0	0	0	0	0	0	0	22
d814e6f4b4af55ec32b413a77fb17177	0	0	0	0	0	0	0	0	0	0	2	0
53ce2a8e02a0c1703df8c2cdff63b715	0	0	0	0	0	0	0	0	0	0	0	2

45f838e66cda61d008efe919fbb9ceeb	0	0	0	0	0	0	0	0	0	0	0	5
ea3b7106d645531d94a9d4a912d4a714	0	0	0	0	0	0	0	0	0	0	52	0
b04278e57b87e2add463318731a29afb	0	0	0	0	7	0	0	0	0	0	0	0
eb7957ad6cb46dcd09391a0266419e25	0	0	0	0	0	0	0	0	0	0	27	0
7710dc16cce929db694165c4fd06f4a3	0	0	0	0	0	3	0	0	0	0	0	0
07b9e2b14fb73f6d1a81fa98daf8eb75	24	0	0	0	0	0	0	108	0	0	0	0
04bdf73853d3aca6205ec64250917ac4	0	0	0	0	0	0	0	2	0	0	0	0
8778c4863ca0188a2bb8e8e6a007927a	0	0	0	0	0	0	0	0	0	0	2	0
63d94181f097f05fc29f74575ef357bc	0	0	0	0	0	0	3	0	0	0	0	0
e89e71f98f43b625c8a4f57b10115d92	0	0	0	0	0	0	3	0	0	0	0	0
9d0b72d1439f9c2de8cf609e2e249826	0	0	0	0	0	0	0	3	0	0	0	0
9a532f02a0b40e46a10347ccc02440a2	0	0	0	0	0	6	0	0	0	0	0	0
a777d13350e5f4b1c093f383b592fc1d	0	0	0	0	0	0	0	0	0	0	18	0
9b7bc51a2bff87ef78873378f7a886bb	0	0	5	0	0	0	0	0	0	0	0	0

d1305551cde4fb96c67993172aaa1df2	0	0	0	0	0	0	0	21	0	0	0	0
3ce53b10b9d7b7822cba2801932a93de	0	0	0	0	0	3	0	0	0	0	0	0
f7f324e30f99930dbe878d9f471a6cd7	0	0	0	0	0	0	2	0	0	0	0	0
16015bad2e30008d2de7c875a6d9ddbe	0	0	0	7	0	0	0	0	0	0	0	0
b45e266c6f27f79b2b7b1fa3beeb006c	3	0	0	0	0	0	0	0	0	0	0	0
800724d3522cb283deb3c930199c3bb9	0	3	0	0	0	0	0	0	0	0	0	0
01d733b8816fcd42090c126cf714ee59	0	0	0	0	0	6	0	0	0	0	0	0
4515eac8c22b3a49986bdce767327ec5	0	0	0	8	0	0	0	0	0	0	0	0
16015e6823d3fec114e5f78f81611a7f	0	0	0	0	0	21	0	0	0	0	0	11
dca978d15b958221d407345d22455a7f	0	0	0	0	0	0	0	0	0	2	0	0
01c1293badb723222fc2d55365bc1464	0	0	0	0	3	0	0	0	0	0	0	0
6aab990fb6586f2ea0dca663c7a493ee	0	3	0	0	0	0	0	0	0	0	0	0
cf31aadb859993d02d468d576f99474a	0	0	0	0	408	0	0	0	0	0	0	350
dfba156b611dec2c33c12b0f4407b011	0	79	0	148	0	0	37	179	0	201	0	0

7c5aa51b717364c650a989f2464abe17	0	0	0	0	0	0	0	13	0	0	0	0
20104ba56c21513e2fbd2ad8a33b805e	24	0	0	0	0	0	0	0	0	0	0	0
4dd3697ca9cc6a6e9b955a562215310a	0	0	0	0	0	0	0	3	0	0	0	0
d2b326d4c30505084d0622ea2474bba8	0	0	0	0	0	0	0	0	0	3	0	0
2c602c56063d2d6ce587cea640d27fd0	0	0	0	0	0	0	0	0	8	0	0	0
b336342b872ed24ad494976fbeee9b3b	0	0	16	0	0	0	0	0	0	0	0	0
eedec5137fa82fe97f5dde904c021c41	0	0	0	0	0	0	0	0	0	0	19	0
f6139f34b88d522f47a9b601a5394231	0	0	0	0	0	0	0	0	5	0	0	0
16175c979069f2113492b2aaafb6c782	0	0	0	0	0	24	0	0	0	0	0	0
740197c16ceaae875cfd65e8ba071f96	0	0	0	0	0	20	0	0	0	0	0	0
ce8ea2918a6b66e10b5386bf5bf88b5a	0	0	0	0	5	0	0	0	0	0	0	0
85c4041e18da96540ba70741b49800d6	0	0	0	0	5	0	0	0	0	0	0	0
72dff6ed7ba043d9d09c486982b27692	0	0	5	0	0	0	0	0	0	0	0	0
5f74367400247637f3fd01ca6a4b829f	0	0	0	0	0	0	9	0	0	0	0	0

072736d2ed896f70a2634609 4dc726e5	0	0	0	0	0	0	0	0	0	0	0	227
3955c98390dedc58dd5dc96e 7a689e8f	0	0	0	0	0	0	0	0	24	0	0	0
f75f96520f8c037b813d44a1f 7e52851	3	0	0	0	0	0	0	0	0	0	0	0
f1ae38a1d9c6b55e3f6d0888 6ee46069	0	0	0	5	0	0	0	0	0	0	0	0
45304273c4275171afd28b4c 3a2d0cf7	0	0	0	0	0	0	5	0	0	0	0	0
6a6192fdf9eda30fb0b513bb 6bc8980a	0	0	13	0	0	0	0	0	0	0	0	0
fad49d6ae0b5027a9df56f1a 6a631120	0	0	0	0	0	0	48	0	0	0	0	0
24f50d6002b61510794c5f66 95d41df1	117	0	0	0	0	0	0	0	0	0	0	0
91b22231b075d71540accd9 e33c30fe1	2	0	0	0	0	0	0	0	0	0	0	0
ed3c33201a24467c177793b df7785b10	0	0	0	0	0	0	0	0	0	0	22	0
71640841414039ed051653e 23a3e5ef9	0	0	5	0	0	0	0	0	0	0	0	0
21f3face77de812b6ca37a54f 5e3e12d	0	0	0	0	0	0	0	0	0	41	0	0
cd033e5992be08397f422c16 3c28ab3f	0	0	0	0	0	0	0	0	0	2	0	0
79292e64b772f2e4c9dc7ecd c475e744	0	0	0	0	0	0	0	0	0	0	4	0

a65567d70bb8585e1266514 7aa1cbc1f	3	0	0	0	0	0	0	0	0	0	0	0
f31cafcc4ea378cfacaacf8877 1b2e50	0	0	4	0	0	0	0	0	0	0	0	0
626d992703709a99ef2a4fbd b9f7e597	16	0	0	0	0	0	0	0	0	0	0	0
054579fb2868de4ad8670268 9a86f8e2	0	0	0	0	0	0	0	0	0	0	0	14
9a554c8b41953138214e67b 7ff98427e	0	0	0	0	0	0	0	0	0	0	0	7
7b6435683b6166949c0b273 95ee8623b	0	0	0	0	0	0	0	26	0	0	0	0
48ec58ef027c7b3978cfdbea bb243068	0	0	0	0	0	0	0	0	0	0	0	44
763a6207abb52f5cb6fb0102 3772272c	0	0	0	0	0	0	0	0	0	0	6	0
ffc25824c25e105ebed624d1 b8981526	11	0	0	0	0	0	0	0	0	0	0	0
a53e767ed16f0117ad384004 6838b68b	18	0	0	0	0	0	0	0	0	0	0	0
85ea71bbe1e222e0d64f9969 21b1267e	0	0	2	0	0	0	0	0	0	0	0	0
2abf39d6db55cd68ac252fbc 5b1fcab5	0	0	0	0	0	84	0	0	0	0	0	0
4811e5b46f496c88bb108e67 f2284502	0	0	0	3	0	0	0	0	0	0	0	0
cc312ffd87669facb913729ea 4109d52	0	0	0	0	0	0	3	0	0	0	0	0

16ffffd2d6b10023c8aeb8106492b2f4	0	0	0	0	0	0	0	0	0	0	5	0
7b82eccdd712a47538a5b1a574e80ae8	0	0	0	0	0	0	0	0	0	0	30	0
b22db8bd161c6d503dd8d588b35d6382	0	0	0	0	10	0	0	0	0	0	0	0
01408adec55c6965a63fe7185eb1ae45	0	0	0	0	28	0	0	0	0	0	0	0
944cec2c9e1a446d5a0f53d60fe43644	0	0	0	0	0	0	0	2	0	0	0	0
4ea404cbde05e8e632a9dc872000a6ac	0	0	0	0	0	0	0	3	0	0	0	0
385b4d0d6ac0c38f4d7ac4f6a73af7ec	0	0	0	0	0	0	0	0	0	0	6	0
62951497e4c90575a389d66f4efbf176	0	0	0	0	0	0	0	0	0	0	3	0
7151c62cacca62e20b77e47677dd977d	8	0	0	0	0	0	0	0	0	0	0	0
bc6915b7fbc40e8c6d8a381275e14566	0	0	0	0	0	0	0	0	0	0	0	4
1ffb841d2080ed3a4ce056e8e838e08a	0	0	0	0	0	0	0	0	0	0	0	5
6cc865527a2f0693c2ae451613ebe8da	0	0	0	5	0	0	0	0	0	0	0	0
a973f7f443b78c02e44f02afb94fcee8	0	0	0	0	0	0	0	2	0	0	0	0
7b57f9dfa7d0259931e93a317dddd715	0	0	0	0	0	0	0	0	0	0	0	11

283b0c521ac15d5145d05ea df3b47581	0	0	0	0	0	0	0	0	0	0	17	0
e8876acb8b58733e6130cd6f f2a560fe	0	0	0	0	0	0	0	0	0	0	0	14
f4af65cad37795a91ab09572 c4b58e0c	0	0	0	2	0	0	0	0	0	0	0	0
e33ec7bc5733c859865bdac7 93187622	0	0	0	0	0	0	0	0	0	0	3	0
4bbd0c50925826cfeb844f7b d82a02b2	0	0	3	0	0	0	0	0	0	0	0	0
b7cab4b16daed7a9ea1fcef9f 73bd865	0	0	0	0	43	0	0	0	0	0	0	0
b90a8f71a2b7069a2bfe84bb 6249f798	0	0	14	0	0	0	0	0	0	0	0	0
c2613c2fcf9621629f48c6069 f353f31	0	0	0	0	0	0	2	0	0	0	0	0
e10d02298f06b249ba9ae7c3 7c00ca1a	0	0	0	0	0	0	0	0	5	0	0	0
41789dd74b262f996479bf21 126f892e	0	0	0	0	0	0	0	0	0	0	0	52
464ff0977ab3e12eb0807c65 886b07ab	0	0	0	0	0	0	0	0	2	0	0	0
c8c41a2b10a8f84dbc46c23a 299eac4a	0	0	0	0	0	27	0	0	0	0	0	0
cd56b55ad5caa929c2d839de 5760e54c	0	0	0	0	0	0	0	0	0	0	0	141
601e5b0311db6cf9071941d0 c14e391a	0	0	0	0	0	0	0	0	0	0	3	0

ebbd6fed5a9459f28bc667cbf0d43141	0	0	0	0	0	0	0	2	0	0	0	0
ea579877590cb49ac5517c915536fe1c	0	0	0	0	0	0	0	6	0	0	0	0
d0029d9e07cd2cdf33c83ff9527a8cd3	2	0	0	0	0	0	0	0	0	0	0	0
05cb7ae8a310387639a3dd7b9ee67829	0	0	0	0	8	0	0	0	0	0	0	0
5a9107c917f3121106530bc82a40e09e	0	0	0	0	0	0	12	0	0	0	0	0
d7dc9d530a7a825143318afc46f0a6c1	0	0	0	0	7	0	0	0	0	0	0	0
621228909bb27ba8866941ce99acd0ae	0	0	0	0	0	0	0	4	0	0	0	0
e11b1baeef28b85a6d98315608640a19	0	0	0	0	0	0	0	0	0	4	0	0
2021ec37a6d4f18fa7427b5514b2df66	0	0	0	0	0	0	0	7	0	0	0	0
363df68d08b12331c89c62d20b25db14	0	0	0	0	0	0	0	0	0	28	0	0
cb64818ea1949a839352c431d6bf4c59	0	13	0	0	0	0	0	0	0	0	0	0
470add9ff35eb5f9e298789b06d50cb5	0	0	0	0	0	5	0	0	0	0	0	0
c11075305fdc0770e92e8ce8a8236e35	0	0	0	0	0	0	0	0	0	0	0	52
4460889a0a40fd553c9b64ac061aae6c	3	0	0	0	0	0	0	0	0	0	0	0

876b4da2fdec56e7c04196c5 7b6b9ab8	0	0	0	0	0	4	0	0	0	0	0	0
89c27e588960abe191bd511f 94d2c575	3	0	0	0	0	0	0	0	0	0	0	0
d847efd5d3d12ef4c1df4689 0183b773	0	0	0	0	0	0	0	0	0	0	0	2
9b257b88e0907f4f33cd81cc a3ef05f2	0	0	0	0	0	0	0	0	0	7	0	0
d9d6fcdebc6df7dc616876ad cf7bbf5a	8	0	0	0	0	0	0	0	0	0	0	0
daa50774b8a9bd21bbf5a93b c2c17914	15	0	0	0	0	0	0	0	0	0	0	0
494be8244be5ff979703f6ff1f 56342e	0	0	0	8	0	0	0	0	0	0	0	0
2864126976e3ad7c2ca6d615 1de15298	0	0	0	0	0	0	0	2	0	0	0	0
3af534a9148931341551f109 d6e127d5	0	0	0	0	0	0	0	0	0	0	0	17
04a55081d85ac3897bdcc9d0 2ffab455	0	0	0	0	0	0	0	5	0	0	0	0
295f5a90bf6957086fd1e62dc 35e6830	0	0	0	0	0	0	39	0	0	0	0	0
d028f6d6af293d867840d662 45e3a585	0	0	0	0	4	0	0	0	0	0	0	0
a1753425f53672adbf794407 12db5fee	0	0	0	0	28	0	0	0	0	0	0	0
02c3574e47b2b1115fd333f2 f322b316	0	0	0	0	0	29	0	0	0	0	0	0

036109f02e7cf8072b98dbfbc df8b546	0	0	0	0	3	0	0	0	0	0	0	0
e5e97892b4898d2d1f67c689 363ee9e5	0	0	0	0	0	0	0	0	0	0	0	5
a8067048a128b996696e3ee 7f901733e	0	0	0	0	0	0	0	4	0	0	0	0
4e6cb873d9f3e89e2ec6a220 1d58b7ba	0	0	0	0	0	0	4	0	0	0	0	0
5d9ce4d472fd00a139dffe6e6 304adc2	2	0	0	0	0	0	0	0	0	0	0	0
c8bfcb9ccffdd206e7d44b8dfc 495eeb	0	0	0	0	0	0	0	0	0	0	0	12
c3c10fe5408756d9dd6e660a 01c8d1c2	0	0	0	10	0	0	0	0	0	0	0	0
1719a41f9bf99a6e0b081384 35ddb4f3	0	0	9	0	0	0	0	0	0	0	0	0
699988fde8ebc1d4d5b23a06 a80bee66	0	0	0	4	0	0	0	0	0	0	0	0
4ce0a103a0d90733b4bd723 a2ce606c2	0	0	0	0	0	0	0	0	0	7	0	0
f48663242cff061d7a1ffa7810 9efc3c	3	0	0	0	0	0	0	0	0	0	0	0
73f9fa1fab213fa8be97c1f7ba 6e2c2a	0	0	4	0	0	0	0	0	0	0	0	0
eabf5b3ddaae64333b6acc85 96299abf	0	0	0	0	0	0	0	0	2	0	0	0
268d775e93764705d5f4b68 5cea12532	0	0	0	0	10	0	0	0	0	0	0	0

e7ae95aa289df43eb62fb9f2 0bb72ea3	0	0	0	22	0	0	0	0	0	0	0	0
fcf6ca4916dda8d24e29eb11 3aa1754b	0	0	0	0	0	0	5	0	0	0	0	0
5a9a8f4186c4311bbb34a7b1 943dfba1	0	0	0	0	0	0	0	0	8	0	0	0
09904faf9af5d22413bf5d751 11968f4	0	3	0	0	0	0	0	0	0	0	0	0
25496c12203f3068774e2d50 9c0eac97	0	0	0	0	0	42	0	0	0	0	0	0
06ebacd65fd06f5c31d96ad7 296fd409	0	0	0	0	0	0	0	2	0	0	0	0
7c5136390b9fedfd72d4a928 4c715a23	0	2	0	0	0	0	0	0	0	0	0	0
ff7f3f5827067814c42cee619 76cf506	0	0	0	0	0	0	0	0	0	0	3	0
c5538b20096976f9daa063d4 ee52ea5a	0	0	0	0	6	0	0	0	0	0	0	0
949292a46b9ec9ca0e9a2823 71e99d78	0	0	4	0	0	0	0	0	0	0	0	0
c2c9af1bf516c6e11b88890d 9e6fcd05	0	0	0	0	0	0	0	2	0	0	0	0
21e29c6b104b6511ebe9123 ecf9a2cbb	0	0	230	0	0	0	0	0	0	0	0	0
792c467531f8dcf5b1296f072 4dc6969	0	0	0	0	0	0	12	0	0	0	0	0
995ee57290ca9214f6191f71 9ab3402c	0	0	0	0	0	0	0	0	0	0	2	0

7e2056c8b6b1d03b57fe803870b4ceef	0	0	0	0	0	0	6	0	0	0	0	0
41f0afe802554e64520f0c53601df2fe	0	0	0	0	0	0	0	0	0	4	0	0
460a1d658478443460d8901ad0bfac4c	0	0	0	0	0	0	0	0	0	0	13	0
55df8ea0977fa3c76c2c7e05fbd8275a	0	0	0	0	6	0	0	0	0	0	0	0
400e7148a3db6adc9fa53eea9a1ab84a	0	0	0	0	0	0	0	0	2	0	0	0
0c826d75eccd34e8778a0ab598a5aceb	0	0	0	0	3	0	0	0	0	0	0	0
a11d5742aa3bf922314443ed0a9d1988	0	0	0	0	0	0	0	0	0	0	11	0
a8b3fded29dacd24a82aaf828075cdf5	0	0	3	0	0	0	0	0	0	0	0	0
f48dd078613ae07051dfc3b8a2a673db	0	0	0	0	11	0	0	0	0	0	0	0
d719245754f6569906b346b9711e9718	0	0	0	0	0	0	0	0	0	0	0	2
502efe2dd6be5b58ffb304d089d2c9dd	0	0	0	0	0	0	0	0	2	0	0	0
5b006812bf806cd2ec3d896adccefa97	4	0	0	0	0	0	0	0	0	0	0	0
9dd30e0f8da246926910f40e61c217b3	0	0	0	0	0	0	0	0	0	0	3	0
dbd12e1680d8158148026bfc39bb68d0	0	0	0	0	31	0	0	0	0	0	0	0

4a821da34d772c6b122b6be37163834c	0	0	0	0	0	6	0	0	0	0	0	0
820fec8b7c22aa5389b22e94815176b2	0	0	0	0	0	2	0	0	0	0	0	0
c0c6bd21784aeb15cd78f9f406ffb38d	0	0	0	0	0	0	0	0	0	0	42	0
839b32ebcd83d778dbbd8dcae71efc70	0	0	2	0	0	0	0	0	0	0	0	0
832761860bbdfe1905619d1d35f73ee4	0	0	0	0	0	0	4	0	0	0	0	0
4f4b03fc97710ca5d89027ed3318d858	0	0	0	0	9	0	0	0	0	0	0	0
788fa3a76dbfef09398f07944a86daab	0	0	0	0	0	0	0	0	0	0	0	14
88e797f956a934581c13f52a89f77946	2	0	0	0	0	0	0	0	0	0	0	0
5d8403a6586742aca7735d5a578880be	0	0	0	0	0	0	0	0	4	0	0	0
ed5435ef434127f311a8c043bb92ca84	0	0	0	0	0	65	0	0	0	0	0	0
c7ad85caff5053193a578728fb88522a	0	0	3	0	0	0	0	0	0	0	0	0
07a5b4ca13095e0198fef8ad01ad141d	0	0	0	0	0	0	0	4	0	0	0	0
6ec9a8aba9cc358c39bcb7a2f14a6ca7	0	0	0	0	0	0	0	8	0	0	0	0
8ad8a94796bfad3b2ae9135b9394ae8a	2	0	0	0	0	0	0	0	0	0	0	0

3812f429d836ed77d245b3df4de20023	0	0	0	0	0	3	0	0	0	0	0	0
9eb26e6b794d89e271c05b4e4689330c	0	0	0	0	0	0	0	0	0	2	0	0
6c9e6024c03550655b1a8469b649dd1d	0	0	0	0	0	0	0	0	5	0	0	0
20b95e0b421d8ef6d6bd46ff7c95eb30	0	0	0	0	0	0	0	0	2	0	0	0
2dc1bf4bb9f96107239834e13f151529	0	0	0	0	0	0	0	0	0	31	0	0
c127c52f8f0339b4d4defc67cf841f95	0	0	0	0	0	0	0	0	0	0	0	4
b7f61911f7e05c2a6093f6840729199c	0	0	0	0	0	129	0	0	0	0	0	0
f69997578a3ace3d9ab7a4dffb5fc7c5	0	0	0	0	20	0	0	0	0	0	0	0
7f4cfc1ff5862afee12c97b824493bdf	0	0	0	0	11	0	0	0	0	0	0	0
17e1d0a871a12e88149b9659cb9b7b25	0	0	0	0	0	0	0	3	0	0	0	0
d4fe7503a04b227839356a5c66f0334b	0	0	0	0	0	0	0	0	0	18	55	0
1ff6cde74c4f3a39c91fb142b742a1bb	0	0	238	0	0	0	0	0	0	0	0	0
97fb16cb5d1d8a4994d9ee23dd6d20cc	0	0	16	0	0	0	0	0	0	0	0	0
d73ec1651e70161c18d8f2d967724442	0	0	0	0	0	0	0	0	0	0	4	0

2f13642341e5984ac013dc1001bb19b3	0	0	0	0	0	0	84	0	0	0	0	0
8846568a36b017744db8f6aa2b4062ed	0	0	0	0	4	0	0	0	0	0	0	0
3b442b45993fc20ae989ea53b820dd9e	0	0	0	0	0	0	0	0	0	0	8	0
608b00c49b126c129da02f5c7a75fc18	0	0	0	0	0	19	0	0	0	0	0	0
e96cb5354ef039773ea5d689abff7b32	0	0	0	2	0	0	0	0	0	0	0	0
e63d8a9bf86f6418116b6e4eb7d3f62f	2	0	0	0	0	0	0	0	0	0	0	0
388bea4dbb4dca2db1ca06a39593c7ed	0	0	0	0	0	0	17	0	0	0	0	0
766d144ddf361268c1a43c1da4890ae6	0	0	0	0	0	0	0	0	0	0	13	0
d6ad73d8e8c6b779e63ddad7a2c2e074	0	0	0	0	0	0	0	43	0	0	0	0
ab6f6fbcbd9a6f8e404cb25bb58f479d	0	0	3	0	0	0	0	0	0	0	0	0
28df9e429ebbee7b644d5c977b3ea023	0	0	0	0	0	0	0	0	0	0	0	114
0b25c522f8e20de17ae2ce0f933a340c	0	0	0	0	0	0	0	0	0	0	3	0
5e160184eb3b33872546defa5fa6032a	0	0	0	0	0	0	0	0	0	0	2	0
04d19bbd5d5e59ef4bb1f14248ceb1b0	0	0	0	0	0	0	0	0	0	0	0	2

d5220f98f5ba024cf56d9ee4cadc340c	0	0	0	0	0	3	0	0	0	0	0	0
b0254e962885438eb937a0e385cb4b6a	0	0	0	0	0	0	0	0	0	0	0	8
f8b06d73a29d0c4c51df88e49c6ce4ce	0	0	0	0	0	0	0	0	0	3	0	0
429652c1d065774b51a65fe32e6fd7df	0	0	0	0	0	0	0	0	0	0	4	0
e681fa892f5692a06a12ef4db0eaa549	5	0	0	0	0	0	0	0	0	0	0	0
1b968523271350bec2d84ef26f316359	0	0	0	0	0	0	0	24	0	0	0	0
6bebe219a048e2fcdc8e9fcd5dbaa4e2	0	0	0	0	0	0	0	0	0	0	0	2
5b07b40912c42bd876bf2bef2784e4a3	0	2	0	0	0	0	0	0	0	0	0	0
c954c6911a29b41c7bde23b10491520b	0	0	0	5	0	0	0	0	0	0	0	0
0f1bce18ced72a4ac38e53a20237e324	2	0	0	0	0	0	0	0	0	0	0	0
a29cd3a20328b74cf4e5b3b89f21da4d	0	0	0	0	2	0	0	0	0	0	0	0
893a3676105708d087e0db7b69f117c4	0	0	0	0	0	0	0	0	4	0	0	0
5a53d6a7aeee01f28dca1ff941bf758c	0	0	0	0	2	0	0	0	0	0	0	0
67c6f05055791af4d6451be5dbbf2a3f	0	0	0	0	0	0	0	0	0	0	0	8

1c1fd9a69a7ee6609875723a99053122	0	0	2	0	0	0	0	0	0	0	0	0
8b762929e94c786e40417907ec185781	0	0	0	0	0	0	0	0	0	0	30	0
665a51a39520842fe3d96889029c578d	0	0	0	0	0	0	0	0	18	0	0	0
bb1dc1b04b41da7b1abb86d47e6aa1a6	0	0	0	0	0	3	0	0	0	0	0	0
b02b027036109180360ecbb7fea49ab8	0	0	0	0	0	0	0	0	0	2	0	0
621bcd74b514756048eec5d8ab51f214	0	0	0	15	0	0	0	0	0	0	0	0
5c38374c464cda9538ce6dcb86ea7823	3	0	0	0	0	0	0	0	0	0	0	0
6206ce02d4faeab1e58bd8cf80dcab05	0	0	33	0	0	0	0	0	0	0	0	0
bdf648593ae19316b2de10aee736da74	0	2	0	0	0	0	0	0	0	0	0	0
a4e00534d08c0350184cda780dd9070d	0	0	0	0	0	0	0	0	0	4	0	0
722dab9b25e4f380b2959e1d60e49b0c	0	0	0	0	0	0	0	7	0	0	0	0
f06cafa9db13a3fcf50e6305f3899a51	7	0	0	0	0	0	0	0	0	0	0	0
4c6100a0e361f7efe2280ffa77ad45c0	0	0	0	0	0	0	0	0	0	0	8	0
0191854f8b31983ff84183f7282bb4b9	0	0	0	0	0	0	0	0	0	0	0	2

9fe26a37c481eb879ab33853 18fee78d	0	0	0	0	0	0	5	0	0	0	0	0
a573d2f89e4c56117bb75e41 5a02c927	0	0	0	0	0	0	0	0	2	0	0	0
01158f2d4a6d28c65910272c 2c9797ab	0	0	0	0	0	0	0	0	0	4	0	0
b6867e4525bdb596af7de39 9d5e800ab	0	0	0	0	0	4	0	0	0	0	0	0
092bbab98e387138491984e 405c0c616	0	0	0	0	0	0	0	0	0	331	0	0
a9e70d64ccee968505f66581 14d4f553	0	0	0	0	0	0	0	0	0	0	0	8
9e443e604697e043eef06094 114a6fd7	0	0	0	0	0	0	0	0	0	9	0	0
22047c85fd7096e3377f6327 2aad0bbf	0	0	0	0	0	0	0	0	0	0	2	0
1e80447832dbcddfe2714842 8661cf0f	0	0	0	0	24	0	0	0	0	0	0	0
150f74468775824280f6fa83 2c3f43d7	0	0	154	0	0	0	0	0	0	0	0	0
5ce18c540bfbbfba3111de8e 90ce219a	0	0	0	0	0	0	0	0	0	0	0	3
8a547dd68a33d378c68824b 929ddce40	0	0	0	0	0	0	0	0	0	0	0	11
cacf269ea167d4e5f0f014001 2cba154	0	0	0	0	0	2	0	0	0	0	0	0
f5ee5d3991300c9eff9e4893b 7ef4b91	0	0	0	0	0	0	0	0	0	2	0	0

4444db608da4741909cfe7029a1f9185	0	0	0	0	0	0	0	0	0	0	21	0
69c91c19213354d8bc658b733a8d951d	0	0	0	0	0	0	0	0	0	0	0	11
95a3c39d2693cb3fe345c06be7bd7b02	0	0	0	0	0	0	0	0	10	0	0	0
0e446bf68d61ac49f668d450820a3c06	0	0	0	0	0	0	0	0	0	0	3	0
183d078012e22772b199871655f5a615	0	0	0	0	0	0	0	0	0	0	6	0
db36d21aa759a71f9afd98ab9fa6dc16	0	0	0	0	0	0	0	0	6	0	0	0
ff420172206a2b224ff538b5e1f65637	0	0	0	0	0	0	0	0	2	0	0	0
39848ef853c6a3f9ee94e1f1f5a82e35	0	0	0	0	0	0	0	0	0	3	0	0
b3d7d0db44fa2b2b0c7eae69657aacbd	0	2	0	0	0	0	0	0	0	0	0	0
f4285880a8d563b7393471c0052fc6a4	0	0	0	0	0	0	0	9	0	0	0	0
0af2d65ff2fc07cad8d30b294149909a	0	0	0	0	0	0	8	0	0	0	0	0
410a8dca390be0ca99f2e4e68a517628	0	0	0	6	0	0	0	0	0	0	0	0
43c54c1b7304b0212499895ba172f1e5	0	0	0	0	0	4	0	0	0	0	0	0
4180262b774341f34355dcb915d7eb53	0	0	0	0	0	0	0	0	0	0	0	4

0abcf1f144ec75dc1278fd792 09311af	0	0	2	0	0	0	0	0	0	0	0	0
4c348b2915dd21a415eb8ec 692842c6c	0	0	0	0	0	44	0	0	0	0	0	0
5be5f7411705e57b264d37e0 f1ed22c3	0	166	0	0	0	0	0	0	0	0	0	0
b9be126bf79f19cfcd6bb2d21 9a01461	0	0	0	0	8	0	0	0	0	0	0	0
b10cc29dfea973198d4fc35b 426f7eee	0	0	0	0	0	0	0	0	0	9	0	0
529681ca6ee0b62a0e60deb 6b1fcb306	0	0	0	0	0	0	0	0	0	0	4	0
7fcc8c45ff563e10f1a2b9575 9cd6b05	0	0	0	0	0	0	0	3	0	0	0	0
42af0b2f2cf76e20257cdf6b6 d1ac8dc	0	0	0	0	0	0	0	0	0	0	66	0
8bdec12daa22bbb9a18a3a5 4f71b333e	2	0	0	0	0	0	0	0	0	0	0	0
1f75d9054c9acc48681eb015 18e752d2	0	0	0	0	0	0	0	0	2	0	0	0
93ebf44d73eb67cd10fa3e94 e32a82cf	0	0	0	0	0	0	0	0	0	3	0	0
50ad32d06b6ba0381be6545 a858089a8	0	7	0	0	0	0	0	0	0	0	0	0
203f22f831a151faa9c7334e2 4941d3f	0	0	0	0	0	0	0	0	0	0	0	5
be0ef41dba10b1d9fd81ac19 17880371	0	0	0	0	0	0	0	0	0	221	0	0

b65a4e9f382470ec352c89f1f8731bcf	0	0	0	0	4	0	0	0	0	0	0	0
a8d41de56701e6fb718a04e6fb123b5a	0	0	0	3	0	0	0	0	0	0	0	0
93cefbeba40fab64017577487ceb9dd2	0	0	0	0	0	0	0	0	0	0	0	12
cc786b21586cf300c4729c6349115ef8	0	0	0	0	0	0	0	0	0	0	0	12
cdf41147e8e5050a96885d48e587927c	0	0	0	0	0	7	0	0	0	0	0	0
0adae9404b29ecebb55da0fa35f7ffee	0	2	0	0	0	0	0	0	0	0	0	0
00ffae099f5632638b0ab3d0392c602c	0	0	0	0	0	0	0	2	0	0	0	0
585a8dd3b74d88dd62435567f41d5fb7	0	0	0	0	0	0	0	0	0	0	5	0
37e99cb0e9c19cad91ad786a00e8e876	0	0	0	0	0	0	0	0	0	0	0	5
795b7e72cb1006bd041497205aa5392b	0	0	0	0	0	2	0	0	0	0	0	0
08086e1fa3b275516836f2b3fa11f419	0	0	0	0	0	0	0	0	0	0	14	0
460e6912add0ea2414f4074e7c997ebe	0	0	0	0	0	0	0	0	0	0	14	0
e2d20a43d9d755d9baa31c0acb1386f1	0	0	2	0	0	0	0	0	0	0	0	0
ea633f9ffb8e0578d62d492458853d77	0	0	0	13	0	0	0	0	0	0	0	0

1f4e0d5447ed9925d71a1180ca1269f0	0	0	0	0	0	7	0	0	0	0	0	0
729d8a741e8d9675f484d1a7d88a4fcd	0	0	0	0	0	20	0	0	0	0	0	0
2f7a82989252a825d0eedac687119b42	0	0	0	0	0	0	0	3	0	0	0	0
bbc8be34cc869eca4bff3b47135ddbaa	0	26	0	0	0	0	0	0	0	0	0	0
b75cffa0725e7eb978f8ab2df130f60c	0	208	0	136	0	0	0	0	0	0	979	228
ee81408fd0dff337061971c1373b5909	0	0	0	0	0	0	5	0	0	0	0	0
09b4a179cc6af1826e873bd0568197d5	0	0	0	0	155	0	0	0	0	0	0	0
eb0d76dc082ecf973f5341f176ce094a	0	0	0	0	0	0	3	0	0	0	0	0
1fcb5b1bdeef61d67e9ab6355893c5b9	0	0	0	0	24	0	0	0	0	0	0	0
5a69509980eb0d6407ad1ae966c44680	0	0	0	0	0	2	0	0	0	0	0	0
41b44d628d855a100333878aa5ebd224	0	0	0	0	0	0	0	0	0	0	0	3
39119da963e168bf05244b22d6cea6d5	0	0	3	0	0	0	0	0	0	0	0	0
c50e4908775c92fb0e75e0e9fddb2f0f	0	0	0	0	0	0	0	0	0	0	2	0
2bdbcddf50829814e43fd5e588830c01	0	0	5	0	0	0	0	0	0	0	0	0

8ba0e263cd2e345788050208d3476078	0	0	21	0	0	0	0	0	0	0	0	0
9941c09ed18030bc8d127e622824abba	0	0	0	0	0	0	0	0	0	32	0	0
add0338a293d7ed9b8505b28bdc54889	24	0	0	0	0	0	0	0	0	0	0	0
b91a23549c89c5e64bd6ffb1c50d73e7	0	0	0	0	13	0	0	0	0	0	0	0
13ebbf774078c1a8ae0c3e8284b65919	4	0	0	0	0	0	0	0	0	0	0	0
7a97d34d276c1dda4493e04ee5a77d55	0	0	0	0	0	0	0	0	0	0	0	4
095b41356812e20c40a1410f396a9ac9	15	0	0	0	0	0	0	0	0	0	0	0
ab217d08464ca91ab99bcf766c32b531	0	0	0	0	29	0	0	0	0	0	0	0
44baa6a87443509537e54b9468a36ef3	0	41	0	0	0	0	0	0	0	0	0	0
5ab1e5fc15525de5c9b7d71694f708af	0	0	0	0	4	0	0	0	0	0	0	0
ac119556778864068d5844a315954f48	0	0	0	0	0	0	0	0	4	0	0	0
357628856b012c2e9f0eb3cbf10ce0a0	0	0	0	0	0	0	0	0	0	0	53	0
5823a50aa5f3a2002f0c5a3311f0b0fd	0	0	0	0	0	0	0	0	0	0	3	0
51cfeef975511db77aca9c02a296d061	0	0	0	0	3	0	0	0	0	0	0	0

08c5c6c63bce698f9dc46882 4fffacb7	0	0	0	12	0	0	0	0	0	0	0	0
af5bcbbe8b1a0876538ea10a d1946dd7	0	0	119	0	0	0	0	0	0	0	0	0
944a11274eca281571ae47a0 2017ad0f	2	0	0	0	0	0	0	0	0	0	0	0
b44e1fe655c3c3bf96d95012 1d7f3b0e	0	0	0	0	0	16	0	0	0	0	0	0
e1997f5d7a51122d49a326b1 25dcd56f	0	0	0	0	0	0	0	44	0	0	0	0
54acfb53181d34f5d380489c 1964e9bc	0	0	0	0	0	0	0	0	4	0	0	0
fa37ea50f42a6baeccbead1f9 2b56bec	0	0	0	0	0	3	0	0	0	0	0	0
559184f9a4341ea9e8723a1d d6b064e6	0	0	0	0	0	0	43	0	0	0	0	0
bc73a993c6caea16db5086ec cf8ccbf8	0	0	0	0	0	0	0	0	0	0	4	0
bea2fb9c3b0e22b1c04edcf2 90662249	0	0	0	10	0	0	0	0	0	0	0	0
fede84e754e2d1b3ba3646fa bf631e54	0	0	0	0	10	0	0	0	0	0	0	0
47a56b542acb55378110b2c 71cc2a4a5	0	0	0	0	0	0	0	0	0	0	0	6
005c92e726258b05ce03ebce aaf18037	0	0	0	0	0	0	0	0	0	0	0	24
2095c68094e158128c75087c da152df8	0	0	0	0	0	0	0	0	0	0	14	0

7fe72e31597bf8efa470b4f9f bf813e0	0	0	0	0	0	0	0	0	0	4	0	0
3d8b9c6259aec085ade43d7 4c040a5e0	0	0	0	0	0	0	0	0	0	17	0	0
3929a6901cb3d3c7e9fb788e 9dd064c0	0	0	0	0	0	0	0	0	0	7	0	0
2b4bfab55c4161527965d862 911d6a93	0	0	0	0	0	0	0	0	0	0	24	0
b8b51dfc97ed3350d50e07aa 202e387c	0	0	0	0	0	0	0	0	0	0	3	0
67cde3f43f611a63c4c2e3b5 d64764e1	0	0	0	0	0	0	0	0	0	0	5	0
312b43cf2144b0b484d0742c 4b1a59f2	0	3	0	0	0	0	0	0	0	0	0	0
6b8483567b2f23f4359fb5b0 cb42829c	0	0	0	0	0	0	0	5	0	0	0	0
44ff6b8821fd070e6bb7cd6b a2a0f8b9	0	0	8	0	0	0	0	0	0	0	0	0
72de2cdcbf0fded1dc9d65e9 a495c77b	15	0	0	0	0	0	0	0	0	0	0	0
aacf28c2984840101bcf70fe2 c4ef4ad	0	0	0	0	0	0	0	0	6	0	0	0
d1dbc70db2c7c15ef2c16e85 f0ee71c7	0	0	4	0	0	0	0	0	0	0	0	0
4c01b33687e9d31b25b9066 dc4cbf9df	0	0	0	0	0	0	0	0	0	0	0	7
c3d1cb0dc43aad755851769c 4effffbc	0	0	0	0	0	0	0	0	0	0	0	4

6acea9856c307c23f73a9283455867aa	0	0	0	0	0	0	0	0	0	0	41	0
e02df5c7dceb5dfed465a1865386e27b	0	0	0	0	0	0	0	0	20	0	0	0
c648db97a60d8c39d673e5ac97b6d2cf	0	0	0	0	0	0	0	0	0	0	0	4
e322ed5d7e0a9e6a58696531ee5adda7	0	0	0	0	0	0	0	0	0	0	54	0
78cd662b82acd8112a6dad339c53a852	0	0	0	0	0	0	0	0	0	0	0	3
a94823682534af8f4b9686aea3775da2	0	0	0	0	48	0	0	0	0	25	0	0
b98c43055f0219dc5c61871916ad3cec	0	0	0	0	0	0	0	0	0	0	7	0
7dd2e58f5f9a7e9607ccedf407337cc4	2	0	0	0	0	0	0	0	0	0	0	0
8b09c873c880066885d6eb7a1fa9c8ad	0	0	0	0	4	0	0	0	0	0	0	0
77a74b17c1ca25515c58070fa25f7246	3	0	0	0	0	0	0	0	0	0	0	0
0ab54e80fbd8715bc18b593805301b2c	0	0	0	0	34	0	0	0	0	0	0	0
8db027aea4ac678247596d2462aa3344	5	0	0	0	0	0	0	0	0	0	0	0
71de48bd72f5bbea7a5a0338313a4ffd	0	0	0	0	0	0	0	0	0	0	12	0
b64ff513685b87d00fefbb585870f26e	0	0	0	5	0	0	0	0	0	0	0	0

f5d99c2976c8ab5673ad881a4fe9ffaf	0	0	0	0	0	0	0	0	0	3	0	0
8e45357169a35cb76ddb5ad0ddbf7440	0	0	0	3	0	0	0	0	0	0	0	0
a83f0ae1b11934eaeeb5fa0dbc2220bb	0	0	3	0	0	0	0	0	0	0	0	0
39a0f23a70cc829530a40f0357322464	0	0	0	23	0	0	0	0	0	0	0	0
1f6a3303e82619c9d40b7cec9a38c725	0	0	233	0	0	0	0	0	0	0	0	0
a33ec0a95e91478cb0fbb555787dde10	0	0	0	0	0	0	4	0	0	0	0	0
2ccd7685a4383945564c155505ae183f	0	0	0	12	0	0	0	0	0	0	0	0
9a7fd9151d9f885f1aeaa4bff9d60a14	0	0	0	0	0	2	0	0	0	0	0	0
5f865e91ae35d8590c73527c03348812	0	0	0	0	0	0	0	0	0	2	0	0
6758cb751246359d249365017b15a866	0	0	0	0	0	0	0	5	0	0	0	0
d25faaeef679f84ebaba2d59e59f2200	0	0	0	0	0	0	0	0	0	16	0	0
43361b1adfdea06eec78463afc15595a	0	0	0	0	0	0	0	7	0	0	0	0
4b536449a39f2106a2bf604fc365c08f	0	0	0	0	0	0	0	2	0	0	0	0
09e7d0bce965bcc5fe2204bcebdc35f7	0	0	0	0	0	0	0	0	4	0	0	0

f40ff63cb0790fcea3416c8efe913e62	0	0	860	0	0	0	0	0	0	0	0	0
4ecb484ec04cdc378ac744ac265d5f0a	0	0	0	5	0	0	0	0	0	0	0	0
968591c8dee40a759c3d2623ee22eec5	0	0	0	0	0	0	7	0	0	0	0	0
8bc4fecb45f5a25610830a6cf6df8c3b	0	0	0	0	0	0	0	0	0	0	7	0
d5fa954542d52cb9aca12d623cab2078	0	0	0	0	0	0	0	0	0	5	0	0
50bd227f0bff1afead1c01dbb7d518b0	0	0	2	0	0	0	0	0	0	0	0	0
29a69b07e21d43040769588034a2d301	0	0	0	0	0	0	0	0	0	0	19	0
9136a0aa25515ec03646b3134f2b7b02	0	0	0	2	0	0	0	0	0	0	0	0
77e4be9824904b5c61cb95d5f5c7ea0f	0	0	0	0	0	0	0	0	0	0	12	0
ae6f0eb8e695c5bda82ff54da47b2b35	0	0	0	0	0	0	35	0	0	0	0	0
74cf986c071f1563828ae817bc135bae	0	0	91	0	0	0	0	0	0	0	0	0
658b21885366644534b0664fe8c206a6	0	0	0	0	0	0	4	0	0	0	0	0
4bc7f99a9b4d25949bceab8a4b300895	0	0	0	0	0	0	0	0	7	0	0	0
7114b8841d5be080ddad5056b8539145	0	0	0	0	0	0	0	8	0	0	0	0

10a055166fda53ff8237c7f872850c1f	0	15	0	0	0	0	0	0	0	0	0	0
7c8f787a4a25dc21247b1bb0472c22e3	0	0	0	0	0	0	3	0	0	0	0	0
1e58784f43bb6003e4e7ff014b4d0842	0	0	0	0	0	0	0	0	0	6	0	0
5b1a2a2209e3206165d97284a124d1ef	0	0	0	0	0	0	0	0	0	2	0	0
ebb83a33b8a8b91f43a41f12f5b5bbb0	0	0	0	0	0	0	0	0	0	0	6	0
cd7e481b877abc571f2729fc592a5952	0	0	0	0	0	0	9	0	0	0	0	0
59232881cf0ae6dcac4c814b4a6b4452	0	0	0	0	0	3	0	0	0	0	0	0
b18147c7e80751359b5109e741811d0d	0	0	0	0	0	0	0	0	0	0	0	6
4f005346e8acb3d410cf63a7b8a33b50	0	4	0	0	0	0	0	0	0	0	0	0
ef80fd75c7f67185e1c70f9602f152d4	0	0	2	0	0	0	0	0	0	0	0	0
557ff96401370616b3114681997ed05e	0	0	0	0	0	0	0	0	0	0	2	0
1cdf57ed1b55e20704934645e2f09b9c	0	0	0	0	0	77	0	0	0	0	0	0
525a6e516edf0c3e807d13beeab2fd9c	0	0	0	0	4	0	0	0	0	0	0	0
a9960e20abfae2f11e6ac1de3289c6c0	0	0	0	0	0	0	0	0	0	3	0	0

1495be954f4195ddefdfb98f3e3d5a35	0	0	0	0	0	0	0	0	0	0	10	0
76ababd25c3fd908e481826c28415f02	0	0	0	0	0	0	0	0	0	0	0	3
6bc2ff424631e00a5f7fc54b102673a2	0	0	0	0	0	0	0	0	0	19	0	0
67599e6f555b50726d642d35ef6acf44	0	0	0	0	0	6	0	8	0	0	0	0
c863d7573d6ed8cc94bc97fbeffa5489	3	0	0	0	0	0	0	0	0	0	0	0
218991de0a67278d399e912e249f4d5f	0	0	0	7	0	0	0	0	0	0	0	0
9d23ef8e3ad72f527c54a7c462306715	0	0	0	0	0	0	0	0	0	0	7	0
7667c93463db153faf462d617176fdeb	0	0	0	0	0	0	7	0	0	0	0	0
c2aecdd74b749f4a5bcd8c1d652d5ea0	0	0	0	0	0	0	0	0	0	0	0	3
77d2730318876af97f03045b3b180e04	0	0	0	0	0	0	0	0	0	0	2	0
9416f5b07546b5a72b03671cd5bc59ca	0	0	0	0	0	0	0	0	0	0	2	0
122b8cc532a53dbb44e1eeaf44de6542	0	0	3	0	0	0	0	0	0	0	0	0
339a269e92fb22a924b91583c8fc631e	0	0	0	96	0	0	0	0	0	0	0	0
3fcda18c021c3b68e9b736102ecee963	0	0	0	0	0	0	0	0	16	0	0	0

64424c305f9e5b10bf27ea3ed86a9bb4	0	0	0	0	0	0	0	0	0	3	0	0
47467d9907f80a92b364f50c4be679b5	0	0	0	0	0	0	8	0	0	0	0	0
0d33b2e5b665b91a2c00e7bdf6de264c	0	12	0	0	0	0	0	0	0	0	0	0
2420be001f06e950007b77ac69ca5d8e	0	0	0	0	0	0	0	0	0	0	2	0
82ee76caab60ecfea22ff8536fe0dd86	0	0	0	0	0	0	0	0	0	0	47	0
c7f5a48e53788c8ff94b575fe64287e2	0	0	0	6	0	0	0	0	0	0	0	0
038854d098ab1ca3b0a597b71663c8fd	0	0	0	0	0	0	0	2	0	0	0	0
c2965d76e8183c0da2ec82fc66c119ee	0	0	0	0	0	0	0	0	0	0	0	6
c723c44eabd9493598cb456a7c5fdffd	2	0	0	0	0	0	0	0	0	0	0	0
34718b96120eaae60cd13eac0ca1e141	0	0	0	0	0	0	0	0	0	0	5	0
8fbffffc1c8db6e455b991d3ee6522f0	0	0	0	0	0	0	0	0	0	7	0	0
cad56acff9f096516af5e55b480e58bd	0	0	0	0	0	0	0	0	0	0	2	0
94324028f1e7736c73ff8e606871c54c	0	0	0	0	0	0	0	0	0	15	0	0
7bfd65c4de61b9eb8eb1f0f9f0ff1f64	0	0	0	0	0	0	0	0	0	0	0	5

764d8818978493b4d398c9df fd6a9fa2	0	0	0	0	0	0	0	0	0	0	7	0
1d96777cc49ae09391f90fdb 09a44050	0	0	0	2	0	0	0	0	0	0	0	0
8c40815eae2a3d72314f3fce 4da45ace	0	0	0	0	0	0	0	0	0	0	21	0
ab2c2d251e7b88d99fc8bdfe 6e009deb	0	0	0	0	0	0	0	0	0	0	0	11
bf5a7f7615853b810b6a8552 edd015e6	0	0	0	0	0	0	0	0	0	0	7	0
04ce55e18c70e220a55258cd d0eb014a	0	0	0	0	0	0	0	0	0	0	0	10
d94542e1628a297c71afab4e ac922691	21	0	0	0	0	0	0	0	0	0	0	0
d51c4e9ddc486a5f7ac93296 faec5ffe	0	0	0	0	0	0	0	0	0	3	0	0
19544274f5ff264f19ca388b1 448b8d7	0	0	0	0	3	0	0	0	0	0	0	0
c586f4ebcbe2d08507109b83 6a7c97a2	0	0	5	0	0	0	0	0	0	0	0	0
f46e2677330c867b4222a963 5505b6b5	0	0	0	0	0	34	0	0	0	0	0	0
bd44a647d25b23598dbcdf6 b5f5e9a43	0	0	2	0	0	0	0	0	0	0	0	0
694d1ccf54f5c06484572263 19a9ab02	0	0	0	0	0	3	0	0	0	0	0	0
2b092a2dd3cf44e314256345 7cf5a318	0	0	0	0	0	0	0	9	0	0	0	0

1d416ce46cde0399d6148d4ba342f5e1	0	0	0	0	0	17	0	0	0	0	0	23
cf5a2e3f55c8b130f80904d59a1c5bfb	0	0	0	0	0	0	0	5	0	0	0	0
6d51c64be942891256eeb0ba560f15b6	0	10	0	0	0	0	0	0	0	0	0	0
be55c7267b564153b08bb976c71659b8	0	3	0	0	0	0	0	0	0	0	0	0
4a76e4a6bf249b044eb75a98fb2de25e	0	0	0	0	0	0	0	0	0	0	5	0
e7fb4b680c05fdcf81add81723a9b789	0	0	0	0	0	0	0	0	5	0	0	0
86ef11505ee2c3eb001a28ffbdd51e65	0	0	0	0	0	0	0	0	0	0	4	0
c3e4770c0e270209406bf54c90286623	0	0	7	0	0	0	0	0	0	0	0	0
8b55fb88f9e29ac34cc67629f2e1e113	0	0	0	0	0	0	0	0	0	4	0	0
a32e0bff8fa4ab8afd13e65969e6b02e	0	0	0	0	0	17	0	0	0	0	0	0
b833ea831502b13975cfb36de7c7f1da	0	0	0	0	0	0	0	0	11	0	0	0
0f93f139b0740f79cb4b8ef25ba28d88	0	0	0	0	0	0	0	0	0	0	8	0
f87a000d843a73758097d195f0cd8e0e	0	0	0	0	0	3	0	0	0	0	0	0
5eefa77ffd093f9fbc1be10cf8da8320	0	0	0	0	0	0	0	0	0	0	0	33

09cf055d419f95e19883903ef4ea5f2d	0	0	0	0	0	0	6	0	0	0	0	0
d2f3c5aef76789598dfb2db80b9ba480	0	0	0	0	2	0	0	0	0	0	0	0
64f508237f8fecc2d29ecf142ffa70b4	89	0	172	0	0	0	46	0	284	0	242	0
9641157a4e22169418f507814e14d2e0	0	0	2	0	0	0	0	0	0	0	0	0
c0da3b336e44808c9c34775049347fa0	0	0	0	0	0	0	0	17	0	0	0	0
dba6fd8bb1ab879157995fca2c423ffa	0	0	0	7	0	0	0	0	0	0	0	0
7138fed4e35499ce736c43313ad5ab68	0	0	0	0	0	0	0	6	0	0	0	0
7e8e81fd4867285da72bf45e2338966a	0	0	0	0	0	0	0	0	0	0	59	0
3c8b6611d54dbd20a6ada4eae6ff2a75	0	0	0	0	0	0	0	0	0	0	14	0
c0cbcc7b2305180033615d45fdc3fa36	0	0	0	0	0	14	0	0	0	0	0	0
52f7ea02be37cc1af58ccc720da8dd39	0	3	0	0	0	0	0	0	0	0	0	0
118ca45a1e3173036f695dbf56390c8c	0	0	0	146	0	0	0	0	0	0	0	0
a0edd461bddee334049e42429f4aa4da	0	0	0	0	0	0	0	0	0	0	0	22
eb2c8c212bfb7da7927deb1a61a75029	0	0	0	0	0	0	139	198	162	0	0	0

6d46e2e05521f18a4499a59a7a7657a5	0	0	0	0	0	0	0	14	0	0	0	0
d876c47a36c5424d1a36e518a3fe30c0	0	0	0	0	0	0	0	0	7	0	0	0
aa7555a493f2b4813424f9fc4d0e3e5f	0	0	0	0	0	2	0	0	0	0	0	0
ec00c11bc76b5a5fbfe3c65ceaac205d	0	0	0	0	0	0	0	0	0	0	2	0
a1031d15fea49c71dda552f92ba6834e	0	3	0	0	0	0	0	0	0	0	0	0
5c9e87925a59b32cf17d2f9e3e6600cf	0	0	0	0	0	0	0	0	0	0	45	0
0e75f87ff9abcd1b9d35c18bb0a1d19d	0	0	0	0	0	0	0	0	0	4	0	0
28fe5f713a4059720e208d0f94703ecf	0	0	0	0	0	0	0	17	0	0	0	0
65127f8299d62f40d0cbdc797bd10392	0	0	0	0	0	0	0	2	0	0	0	0
c198d85c2ca44fd43a196886062919b8	0	0	0	0	0	0	0	0	0	0	3	0
abb042e5216b07cf8fe713f4b3a7a12a	2	0	0	0	0	0	0	0	0	0	0	0
d87bfcf1d9a1416a831ee7d52c6b508b	0	0	0	0	0	0	0	0	0	0	3	0
8a0e57eccd4bc85225d13073db78ba9b	0	0	0	0	0	10	0	0	0	0	0	0
2153fcaf1ba9f2b165b6fd4f3db42da0	0	0	0	0	0	0	0	0	0	0	0	8

e8a8d7cf3960cb15cb513a9de5684713	0	0	0	0	0	0	0	0	0	0	40	0
f3eb11fe40a79f036dc9c84cb92102c2	2	0	0	0	0	0	0	0	0	0	0	0
a6a5ba3d90c48e0ad6e8dcdcd535ea68	0	0	0	0	0	0	0	19	0	0	0	0
75b99435f0763d761a52396138dc9766	0	0	0	0	0	0	0	0	9	0	0	0
00ba31c8674638b3dc9c09cdfabb6db3	0	0	0	0	0	0	0	0	6	0	0	0
6b97f1e7d71436eff5ccf39b8906a32c	0	0	4	0	0	0	0	0	0	0	0	0
97ba9e11ff2db162741bcb1d624e15b1	0	0	0	19	0	0	0	0	0	0	0	0
d47bf51ed01705c80d6d43c233f5abe9	0	0	0	0	0	0	0	0	51	0	0	0
d06dbea4b2db83331a999b3581410bb1	0	0	0	0	0	0	0	7	0	0	0	0
c5baca3ba2fc9a9dcaffd192e892d9e2	0	0	0	0	0	0	0	0	0	0	2	0
2bd7b7fbdfa495d0cf28399d550851ee	3	0	0	0	0	0	0	0	0	0	0	0
9c42ce30e640bcb2d58edf80e41c74e2	0	0	0	0	2	0	0	0	0	0	0	0
4c64bc2583f4903b141d4b1c11e969df	2	0	0	0	0	0	0	0	0	0	0	0
3699bb21fc09779a0f7e1fee502eea5e	0	0	0	0	0	0	0	0	6	0	0	0

fd6383f4484364b1c4737e079179081f	0	0	0	0	0	0	0	0	0	0	4	0
3c01bf624db1dbd6b29c481cd438e430	9	0	0	0	0	0	0	0	0	0	0	0
33a94aeb5950a7ca243b905ee3cf8692	0	0	0	0	0	0	0	4	0	0	0	0
08c304e4a8cebfb55460bd8c86adf12a	0	68	0	0	0	0	0	0	0	0	0	0
4272decf71370984b6755ae89c06c3e0	0	0	0	0	0	5	0	0	0	0	0	0
5e2c27b12e68e8a452b39a0eecafc6cd	0	0	0	0	0	0	0	0	0	0	3	0
098fd68b4737379d51d79414c072fc32	0	0	0	0	0	3	0	0	0	0	0	0
3bc12f8f9f242fe10a4801d9740883cb	0	0	0	0	0	0	0	14	0	0	0	0
480da8a44728d85119ae7a9547e4642f	0	0	0	0	0	0	0	0	0	0	6	0
c5a8c57864251e2982343cf2903c6d25	0	0	0	0	0	0	0	0	0	0	8	0
42a6fcd4db8646fe5b2d50b22947c3b1	14	0	0	0	0	0	0	0	0	0	0	0
53eb6ef85f10b7e86e83a5c621b8454c	0	6	0	0	0	0	0	0	0	0	0	0
53a518b997fa6fa20054d0d50100b691	0	0	0	4	0	0	0	0	0	0	0	0
18daec46e4123a5e4f820a452a95617b	0	0	0	0	0	0	0	0	0	0	7	0

f8b8778455ffe963eebcb249a57304e9	0	0	0	0	0	2	0	0	0	0	0	0
2b9a6427e7c6a2f0fbf389210ceea568	0	0	4	0	0	0	0	0	0	0	0	0
2bf81ade526c567841471c6d20a39f7d	0	0	0	0	2	0	0	0	0	0	0	0
0d2a93e2d243a18516e4ae65f6a50993	0	0	0	0	0	0	0	0	3	0	0	0
cdd84598fe543d3375fce800497bc49a	0	0	0	0	0	0	0	0	0	0	134	0
0d7c73e10cba6ccbfb7c1df2a932452e	0	0	0	0	0	0	0	0	0	0	0	4
a7c85a1a9d8b5d0c644f20e7f45f5564	0	0	0	7	0	0	0	0	0	0	0	0
0f95f62e8385ff7dae6876b5b16eb7d0	9	0	0	0	0	0	0	0	0	0	0	0
0bbf862837c253e23f9b85c635e92d99	0	8	0	0	0	0	0	0	0	0	0	0
219d1abc2b6384551f29e2ef8721833e	0	0	0	0	0	0	0	0	0	0	0	7
f740dc4e3f816489d1f2277099921054	0	0	0	0	0	0	0	0	0	0	10	0
31eeb7cb0c7901a246cbf26c134bcb24	0	0	0	0	0	0	0	0	0	11	0	0
fe6585a9c4cacaec3b5d7bc500186ff9	0	0	0	0	0	11	0	0	0	0	0	0
22c933efcca7d05c019506acf17a84b1	0	0	0	0	0	3	0	0	0	0	0	0

93556679f9a142bdc691cbdf b51347ef	0	0	0	0	0	0	7	0	0	0	0	0
3cac7b1f99e42596a8128d40 0c0335c2	0	0	0	0	0	0	0	0	2	0	0	0
39cb433f74e5526ad3aa7242 8e7f4717	0	0	0	0	2	0	0	0	0	0	0	0
084dfab61b6bfa33bedfd0b3 c773354e	0	0	0	0	4	0	0	0	0	0	0	0
180da40299e2051c3292bb1 c1073da98	0	9	0	0	0	0	0	0	0	0	0	0
76b854102f26a6d98c47c6ad 88d49d44	0	0	0	5	0	0	0	0	0	0	0	0
aec0655eadabac2290192a0a cb82a6e6	0	0	0	0	0	0	0	9	0	0	0	0
1859d849f1c322e59a456e3e 1dda751d	0	0	0	0	4	0	0	0	0	0	0	0
ff616969cabe700c032697bf3 ba0633a	0	0	0	0	0	0	0	0	0	32	0	0
c548450110e7893860a6caa3 ef1e642a	0	0	10	0	0	0	0	0	0	0	0	0
902bae44480748e5aa79f534 b949cc7e	0	0	0	0	0	0	0	0	0	0	0	40
bab5a0203bd3782c512cbdce 8c5d514b	0	0	0	2	0	0	0	0	0	0	0	0
52ada786a1f0dc129e9d7722 0441a336	0	0	0	0	0	0	0	0	3	0	0	0
68c7c5192b0dbeb2643872d 483f070ff	0	0	0	0	0	0	0	0	0	0	2	0

5f80b047573d491654a731d17448f052	0	0	0	0	2	0	0	0	0	0	0	0
8c3228db296e0acfe6e8791cef08b351	0	0	0	0	0	0	5	0	0	0	0	0
d88b8939c6da8b5039514586825a1797	0	0	12	0	0	0	0	0	0	0	0	0
641359abca8da7f04229dcce7e5d3b6c	0	0	4	0	0	0	0	0	0	0	0	0
eef030f8bcdf60c0c5ed327c8da2075a	0	0	21	0	0	0	0	0	0	0	0	0
73a2a939f742b354247a013e50b50c9b	0	0	0	2	0	0	0	0	0	0	0	0
57e71c21c5b44f8faed8d75c9a49e418	0	0	0	0	0	0	0	0	10	0	0	0
8d2805124bee5dd2955ec882bdb299c2	0	0	0	0	0	9	0	0	0	0	0	0
a15364ca51b8b881bce3374b54bc97a8	0	0	0	0	0	0	26	0	0	0	0	0
19d0404e7c2a561cc03f65f7a534e00b	0	0	0	0	0	43	0	0	0	0	0	0
611bd2a53dffe61c9c6e599a57f3383b	10	0	0	0	0	0	0	0	0	0	0	0
35660c463721f068afbcacb8f73500ff	0	0	0	0	0	0	0	16	0	0	0	0
c4c8f67899ca9f2043f3e5288cf53cd4	0	0	0	0	0	0	37	0	0	0	0	0
99e826c2534742427861a7cb107edb5b	0	0	5	0	0	0	0	0	0	0	0	0

9653319a1d41ac6fff3fac0926b9fd8f	0	0	0	0	0	0	0	0	0	16	0	0
8d3764c8bb52016c9658f9cd2908806c	0	0	0	57	0	0	0	0	0	0	0	0
3a5272c0e2b26539aea68405a9abf861	0	0	0	0	0	0	340	0	0	0	0	0
1f158d52730ff424ed0fc4b1a4963f73	0	0	0	0	0	0	0	6	0	0	0	0
1c05568577baa50e3a0770990fdf0586	0	0	0	0	0	0	0	0	0	4	0	0
a9d5cda5538621a922f6f8c81488b7a4	0	0	0	0	0	4	0	0	0	0	0	0
2087e36f93208da23139215d16cd553b	0	0	0	0	0	0	3	0	0	0	0	0
f5ec0636f46a23ebdc0cf6e7aee460ff	0	0	0	0	0	0	0	0	0	0	35	0
4418297d345ac972df070b6ec26b293a	0	0	0	0	0	0	0	0	0	0	11	0
00a9774964703eac4bdf96f2bbc22a03	0	0	0	0	0	0	0	0	0	2	0	0
279bcc6d59118a2b0ea977896eb17f99	0	0	0	0	0	0	0	0	3	0	0	0
1549611966f29fdfd8fc59ac3523c6b9	0	0	0	6	0	0	0	0	0	0	0	0
0d83ad26ad839d1826169e8ea24e668f	0	0	0	0	0	0	0	0	0	2	0	0
21fb0d642c09c2546c7b545eb578a40a	0	0	0	19	0	0	0	0	0	0	0	0

11730fe20a8662bc9fdad168f6e47869	0	0	0	0	0	0	0	0	0	0	13	0
5383ea2a78b3fb6ddd035c1c7d4de32c	0	0	0	0	0	0	0	0	0	0	5	0
d7fa1c80ae5518377599012bba76adaf	0	0	0	0	0	0	0	0	0	0	3	0
4a0eda863536a90bb49cb555a9de03bd	0	0	0	3	0	0	0	0	0	0	0	0
138a5bc3aa7bc2ba25fb8fe9ea8bc2ba	0	0	0	0	0	0	0	0	0	0	5	0
1a342a5de52fff038c7ea7ab138c3104	0	0	0	0	0	0	0	0	0	6	0	0
ad6bd2cc892f3e24bd4b18568c69ef6c	0	0	0	0	0	0	13	0	0	0	0	0
8033f1b53c87c55d650bb31106d2d313	0	0	0	0	0	0	0	0	0	0	2	0
21c1cdfbe8fd00d0e9c59dcac4633d33	0	0	0	0	26	0	0	0	0	0	0	0
1755ee6c7e1cebd21569800bcdea5941	0	0	0	0	0	0	0	0	0	11	0	0
a0eb4765bf2f218cd104e2128697fcdc	0	0	0	0	0	0	0	0	0	2	0	0
677b57aa47c048f99f84eb0e376b01b0	0	0	0	0	0	0	0	0	4	0	0	0
2de07d1d589d319ed53868ebeafc2e19	0	0	0	0	0	0	0	0	0	0	0	3
d146fc3df080960d13bbdf9c10c89b48	0	0	0	0	0	0	0	0	0	0	69	0

2763d22890d384966eba0f5c97e5b89e	0	0	0	0	0	0	0	0	0	0	4	0
5c3c356ea1ba9a93a7b381f681fe67dd	0	0	0	0	0	0	0	0	0	0	0	6
4052e49f07596fdd59f467833c39345a	0	0	0	0	0	0	0	5	0	0	0	0
763847a5380220575f32b869eccdbd36	0	0	0	0	0	16	0	0	0	0	0	0
aa1943d094a9a458be3990580f3e90f0	0	0	0	0	0	0	0	0	0	0	19	0
a48538120061dff891ec0703f4d9f028	0	0	0	0	0	18	0	0	0	0	0	0
e6047f7d44c7baaafd0dec866a379c07	0	0	0	0	0	0	0	0	4	0	0	0
9edbd33137abc4e05ccf67f5dff312ba	0	0	0	0	0	0	0	0	0	4	0	0
30d67904aca7539ded717c317050e976	0	0	0	0	0	6	0	0	0	0	0	0
db528673a5ba179c4d9a07e9f1f7a1e2	0	0	0	0	0	0	0	0	0	0	11	0
bfc20e424ec72f7f517f1bf5f7601e35	0	0	0	0	0	0	0	0	0	2	0	0
032c014db8c5569de32e54ba9169130f	0	0	3	0	0	0	0	0	0	0	0	0
61b51373feed9629ea5e809140fc6b1e	0	0	0	0	0	0	0	0	0	0	0	28
7adc48d03f5b6fae2d23ef49e918bc87	0	8	0	0	0	0	0	0	0	0	0	0

277eb98cfbdd55153c3016d470ef541e	0	0	2	0	0	0	0	0	0	0	0	0
8da01b4eda5800c200b3a5dc32d0cbac	0	0	0	0	0	28	0	0	0	0	0	0
6dd46863389dd7e96af56df863b6ca22	0	0	0	0	0	0	0	0	0	0	5	0
690d8fa92b5f0f29e4f159f6d83577d4	0	41	0	0	0	0	0	0	0	0	0	0
d9eaa1542b4ab3bae0493dc83b441590	0	0	0	0	0	0	0	0	0	0	2	0
282c3cb4f6e5fc867dc50a8177ea256e	0	0	0	0	0	0	0	0	0	0	0	13
281ddcf6fd64532fefe4d2d043dafba2	3	0	0	0	0	0	0	0	0	0	0	0
dab03fb100cf3b0f229c6e25c1235cb1	0	0	0	0	0	0	0	0	0	0	0	2
b86ccc9d357a2819d2ce12d82b38c343	0	0	0	0	0	0	0	0	28	0	0	0
8de0c582d436c484c60ce127986a89f0	0	0	0	0	0	0	0	0	0	0	23	0
f26b67ecb7a88fa34ca7d27755f0506b	0	0	0	0	0	0	0	0	0	14	0	0
9f7500fca6a76d7b9fc9a648296488ba	0	0	0	0	0	0	0	0	11	0	0	0
e7f8205b82c13fb082e2dcfd159b9843	0	0	0	0	0	0	0	5	0	0	0	0
f7876aac916f06e7ea2ab1cbd4df9161	0	0	0	0	0	0	0	0	0	0	0	2

46e6650082f23974c4f50d2a8e9bd871	0	0	0	0	0	0	0	0	0	0	0	7
ce6463dc4d15b4c6a760ed4b573b1cea	0	0	0	5	0	0	0	0	0	0	0	0
f712b659f431b76f7d15c01f4b81e7c3	0	0	0	0	17	0	0	0	0	0	0	0
4f7f831991670034b4b0a0ab6093fb38	0	0	0	0	0	0	0	0	0	0	0	2
ddb46b7e878d8545007e1428256224e1	0	0	0	0	6	0	0	0	0	0	0	0
3f35e2776c90d2e0e6a65ee7a05d2ebe	0	0	2	0	0	0	0	0	0	0	0	0
467e376566929eb190d35b7d3fd13880	0	0	0	0	0	8	0	0	0	0	0	0
cbfc9fa5524f058d7420e91ed639f561	0	0	0	0	0	0	0	0	0	2	0	0
ce1577e649caf5e24fb3ba297219fc10	0	0	0	0	0	2	0	0	0	0	0	0
8f26d0cd818640c6a9772911905922bd	0	0	0	0	0	0	0	2	0	0	0	0
ea064845a0a4c46bf6e297809c7c8029	0	0	0	0	0	0	0	0	115	0	0	0
fcd38519682909dc47ed2c26febed408	7	0	0	0	0	0	0	0	0	0	0	0
6b51bd0ed9b3145253dc85320b05a354	0	0	0	0	0	0	0	0	0	0	0	2
01eb66a059d80148a9439e33fb4d2d40	0	0	0	0	0	0	0	0	0	20	0	0

2d04d36d35004f408805f8545e38fcd9	0	0	0	0	0	0	0	0	0	0	2	0
b8bce59657546adda91cb74327f44f62	0	0	0	0	0	0	0	0	0	0	0	13
945e43425f86b1940bb723a8c9841628	0	0	0	0	0	9	0	0	0	0	0	0
3d11f4381eecc3b8b46c76cbf047f4fc	0	0	10	0	0	0	0	0	0	0	0	0
6d8166b0c05b14273a1b4c3ff4bc1890	0	0	0	0	0	0	3	0	0	0	0	0
faa0b7681d13356c9eaef089f74e14e4	0	0	0	0	0	0	0	0	0	0	0	2
110845f38340614129159662752c6bb2	0	0	0	0	0	0	0	0	0	0	15	0
829cb5935bda66ee0b1b7945d407908b	0	0	0	0	0	0	3	0	0	0	0	0
d252f8d83c86485268bdfc3a3c9de1e2	0	0	0	0	0	0	0	0	0	2	0	0
743a2791763a575b97273a9aea51dec6	0	3	0	0	0	0	0	0	0	0	0	0
bc2e6eb4c2f8d165d8a48586be6a5835	0	0	0	0	0	4	0	0	0	0	0	0
d231fe5f4f1208f7d1e4e37f250a296d	3	0	0	0	0	0	0	0	0	0	0	0
6c964a1ca0436926f03180f6eccd2c4c	0	0	5	0	0	0	0	0	0	0	0	0
de68d5086db16b5abebb87a80f4f290a	0	0	0	0	0	26	0	0	0	0	0	0

33bd19b9931b6eb28270554 4f6c8b4a8	0	0	0	0	0	0	0	0	0	177	0	0
12ba38c2e66e47fa6a01c23f 02a5f16c	0	0	0	0	0	0	0	0	0	3	0	0
7394f20b978253291f789ef0 905ed62d	0	0	0	0	10	0	0	0	0	0	0	0
950fecaeb190a1984b03afd6 2db20bec	0	0	0	2	0	0	0	0	0	0	0	0
df6524ee99c88a9810a91cea f5072939	0	0	0	0	0	0	0	0	16	0	0	0
3fd59cc7da4e71c82cf63b06d 153578f	0	0	0	0	0	0	0	0	0	0	2	0
40fab337aa11fd6522b972ee 47a20fac	0	0	0	24	0	0	0	0	0	0	0	0
33f9d7c285ff5dbfc905ef3aff 7a3161	0	0	0	0	0	0	0	0	0	0	3	0
bb1044b07d376043cb99cbe 49cceffb3	0	0	0	0	0	0	0	0	0	0	2	0
6130ec14dcb2e0491e7e74f3 f3211152	0	0	0	0	0	0	0	0	0	0	4	0
b5d950043b322ac85c2a4dc9 c2834818	0	0	0	0	0	2	0	0	0	0	0	0
866d00ce23abf3e521944a05 c87d09d4	0	0	0	0	0	0	0	6	0	0	0	0
1fbc154d53d51f8ebc07cfd8a 592ef6b	0	0	9	0	0	0	0	0	0	0	0	13
b38c3c6e69974f2284f76340 8506935e	0	0	0	0	0	14	12	0	0	0	0	0

7475d3e0c74a4e1d149d9e4fdcd461a3	0	5	0	0	0	0	0	0	0	0	0	0
4dee869ac022eee4714f01d03fd04c20	0	0	0	0	0	0	0	0	0	117	0	0
57441c251f02f3b3083c6652624f7428	0	0	0	0	0	0	0	0	0	0	0	6
e9bf61664931523bd2b1bb710ff9661e	0	0	0	0	0	0	0	0	0	0	0	2
38470d538eaa9109b462534b0584426b	0	0	0	0	0	0	0	0	0	0	0	3
d1afede97b943ab2067c7128cf9ff84a	0	2	0	0	0	0	0	0	0	0	0	0
f7d70f73f8049861498d737839f177c6	0	0	0	0	0	3	0	0	0	0	0	0
d6504f8cc1c5f688a6a5bf57772e2331	0	0	0	0	0	0	0	0	0	0	34	0
fb4a550c43aa918d309476c3468e7e2a	0	0	0	4	0	0	0	0	0	0	0	0
21790829a38dc39fa97a7e9998893869	0	0	0	0	0	29	0	0	0	0	0	0
e63b4d67054c65e75bed79e0dc9b5d58	0	0	0	0	0	0	0	0	0	0	13	0
6c8b50e4abb3891dc630b5c555d17a89	0	0	0	0	0	0	0	0	0	0	0	6
89ea0086a38f5844944810845747a157	0	0	0	0	0	0	0	0	13	0	0	0
1af5da536650705f3667bc7ace5c8d35	0	0	0	2	0	0	0	0	0	0	0	0

44cb40bc0f2315c25e5b4b48e6eef486	0	0	0	0	0	0	4	0	0	0	0	0
b9fc6232ab7fe8051622e40baec2b873	0	0	0	0	0	17	0	0	0	0	0	0
43cf77cfde192afa6163b3e8fc0bfe67	0	0	0	0	0	0	0	0	0	0	0	12
fdbdbae0de15b50ce9a254d3dd81e298	0	0	0	0	0	0	0	47	0	0	0	0
4861f1a7b6c12a1ed91a59035887314d	0	0	0	0	0	10	0	0	0	0	0	0
210b37a04081454ff6a05fcdcb0ef4a7	3	0	0	0	0	0	0	0	0	0	0	0
0b0e6ae0c47575363dfa7d73833d1abd	0	0	0	0	0	0	0	0	0	0	2	0
918f60bc77e181b98ea4f09b37299178	0	0	5	0	0	0	0	0	0	0	0	0
32a921efd69a7a3dd61a087a0164e51f	0	0	24	0	0	0	0	0	0	0	0	0
65f1d4f552e0999af75b5292ce06fda2	0	3	0	0	0	0	0	0	0	0	0	0
5ac131973e07c2c6b641768cd016459a	0	0	0	0	0	0	0	0	0	0	0	3
3eb5e103e63ec95a571e806c15c8f8d8	0	0	0	0	0	0	0	0	0	0	0	20
6ddd57be6f6613a7b526eb5d8dd909ee	0	0	0	0	0	0	36	0	0	0	0	0
595e01ecc51b4c05081ebc11eba2a584	0	0	103	0	0	0	0	125	0	0	0	0

16ff19c852cd21003ac9b9a6a2af202c	0	0	0	0	0	0	0	0	0	6	0	0
618c7e50e7111267a7905d99ab34aae2	0	0	0	0	0	0	0	0	0	0	0	3
b8e6d15adb7924d181d8542b1aa33fa9	0	0	0	0	0	0	0	0	0	0	0	5
1ed428f3666a52090646e7c5f4b0b6a1	0	0	0	0	0	0	0	0	0	0	0	5
51b96b128aa71c17949668c2958a97d3	0	0	0	0	0	0	0	0	0	0	9	0
4a2c131ddec9fe1d8cc47c92f4f7c36f	0	0	0	0	0	11	0	0	0	0	0	0
e4338751c6dd6c2b1330b531cdb46389	0	0	0	0	0	0	0	8	0	0	0	0
75b882ce13356983e4b19be37c666df3	0	0	16	0	0	0	0	0	0	0	0	0
b33ece687d1b957a9b9ef13e66a60a4f	0	0	0	0	0	0	0	0	0	0	12	0
02a2440139c6fba2f8c115910ffb6c29	0	0	0	17	0	0	0	0	0	0	0	0
531df718c69158e206d113366c0bacc7	0	0	0	0	0	0	0	0	0	0	2	0
f84a141dcf560aeb9ad285cbad12683a	0	0	0	0	0	0	0	0	0	0	0	3
a71ebc895cccabf27f4172d9b23fdcf0	0	0	0	0	0	0	0	0	0	0	0	66
0272a467ecc63f0b68f5ffd7342ccdf0	0	0	0	0	0	0	0	0	3	0	0	0

9d9e8a7bf514e8d843215076 06def3c1	4	0	0	0	0	0	0	0	0	0	0	0
6c9d6a5f9b85102a130db7b6 dc29d9ac	0	0	0	0	0	0	0	0	0	0	10	0
31e8c901c090edb3f2e5543f 280a980d	0	0	0	0	0	0	0	0	0	0	2	0
858851ae9efedfac7f8a4e846 2cc80b8	0	0	0	0	0	5	0	0	0	0	0	0
640185648eaed9aeddf69d77 8c6c1e05	0	0	0	0	0	0	0	0	0	0	10	0
f4067a0710c1240a739ec56a 9d08e764	0	0	0	0	0	0	0	0	0	0	4	0
9305626942dc70e0964e185 8ef520cfc	12	0	0	0	0	0	0	0	0	0	0	0
e2c19646bde494cdc7e6f65c 1efcdf77	0	2	0	0	0	0	0	0	0	0	0	0
7c1eb760c10c5c3c42a4f9a3 b814a826	0	0	0	0	0	3	0	0	0	0	0	0
9e747000ffbe0ea05e47d712 30fcdb56	14	0	0	0	0	0	0	0	0	0	0	0
905294c2dcba6580b671371 ec5e9bd66	0	0	0	0	0	0	0	4	0	0	0	0
b3571c71773de9349a963a3f 5fc1fddf	0	0	0	0	0	0	0	29	0	0	0	0
d9577f5c0e5e9d5ad97e1ef1 508af6d6	0	0	0	0	0	0	0	12	0	0	0	0
d212df26576587545329339e bb75bdd9	0	0	0	0	0	2	0	0	0	0	0	0

162160172b90823ff6011179 3d10e4a7	0	0	56	0	0	0	0	0	0	0	0	0
9f26e0811f01c9732409043a 48bbb0a9	0	0	0	0	0	0	0	0	0	2	0	0
40501560ca3775a24216dd4 dfa4d7206	0	0	0	0	0	0	0	0	0	0	5	0
133f1a2d3ddfbfa6b522e6ab 9197a0d4	0	6	0	0	0	0	0	0	0	0	0	0
7106cc82785f35e82f47bd17 a937a747	0	0	0	0	0	2	0	0	0	0	0	0
44b9c0d81013fd753cb7fb16 9ef57112	0	0	0	0	6	0	0	0	0	0	0	0
82a89b7d50add449b257354 04e851f62	0	0	0	0	0	0	0	0	0	0	0	5
c200ea15f82337e47d2a4a9a e78f5064	0	0	0	0	17	0	0	0	0	0	0	0
4bfd1325a55dec5baa1ca9b7 011fb59b	0	0	0	0	0	0	2	0	0	0	0	0
8ae81626905e87bc963ad3d 2d48ddf74	0	0	0	0	0	0	0	0	23	0	0	0
341c2affaf63f5c458a090074c dd7df7	0	0	0	0	0	0	0	0	0	0	3	0
ed321105637350d1c3add33 32078c08b	0	0	0	0	0	0	0	10	0	0	0	0
ca1f7d96b10c6889c0dd60af 200a5dae	0	0	0	2	0	0	0	0	0	0	0	0
454187de079379d0a568eb8 7c5c768e9	0	0	2	0	0	0	0	0	0	0	0	0

b49eb38bb24f25d7114f63f6f153da1c	0	0	36	0	0	0	0	0	0	0	0	0
ad946cf48655669c2330632b0bd9ab39	0	0	0	0	5	0	0	0	0	0	0	0
9ff434c65290ca6debeb2139eb562765	0	0	0	0	0	0	0	0	6	0	0	0
8109eb9a7c6e6c96aff3a8b52e35543f	0	0	0	0	0	0	0	0	0	0	7	0
796dcf10f529bd4d106bb4050a87236f	0	0	0	0	0	0	0	0	12	0	0	0
ff9e09f75bec10f3fe27770f9ef95822	0	0	0	0	0	0	0	0	0	0	3	0
f18dedb5f7ca7420f9500ae46f77e568	0	0	0	0	0	4	0	0	0	0	0	0
c8ecb6504074c53d5276aaa0c4b2a5f2	0	0	0	0	0	0	2	0	0	0	0	0
87a7fac22b013a786ab5cec394369d80	0	0	0	0	0	0	0	0	0	0	24	0
72856c38714c4a19df86d144f6e35c13	0	0	0	34	0	0	0	0	0	0	0	0
d06dde41b8dd6e21f9dc836a007e6f9c	0	0	0	0	0	0	0	0	0	0	0	2
f28225389a79e9602cc5010b1eac9e77	3	0	0	0	0	0	0	0	0	0	0	0
6ab0d765910d433d24b289a4daba4167	0	0	9	0	0	0	0	0	0	0	0	0
c2dd38aeb9c33ad8529eea5cf5360892	0	9	0	0	0	0	0	0	0	0	0	0

057e3ff6b54d29f548438ec4769f7bfd	0	0	0	0	0	0	0	0	0	0	6	0
bec6416cbac43ffaaf795f91be502ed7	0	0	0	0	14	0	0	0	0	0	0	0
46498ba3694dc54caeb7f783f17f42d7	0	17	0	0	0	0	0	0	0	0	0	0
9442b28ea9fa278332e7cde4f87d4f84	0	2	0	0	0	0	0	0	0	0	0	0
9740100c5837a215d71d22d23ff0d37b	0	0	0	0	0	0	0	0	0	0	4	0
e6f6001ccd041e19201ec26a0bb5fecd	0	0	0	0	0	34	0	0	0	0	0	0
4bcdacd2fec44127c5b7eee433ace1e9	0	0	0	0	0	0	0	5	0	0	0	0
fcc1eb83bb2f681ed1f4334de2a3d400	0	0	0	0	0	0	0	0	0	4	0	0
84284fcc6b182a13f20ab1a68e7b18cf	0	0	0	0	0	0	0	0	0	0	4	0
291015cc44697f1e453b2fc3c2184e93	0	0	0	0	0	2	0	0	0	0	0	0
153cc655887e1ca957e307f6c6a42d9a	0	0	0	0	0	0	0	0	0	0	5	0
ea456d663f062d13fc3376ea26e7d8e4	0	0	0	0	0	0	6	0	0	0	0	0
f27b7523e56ae3ff1a183a218eb1f9f4	0	0	0	15	0	0	0	0	0	0	0	0
a6b5ad3439492c1727b48990da7978e0	0	0	0	0	0	0	0	6	0	0	0	0

a5d36945fd2f96607f7a3f5fa a575a4e	0	0	0	0	0	0	5	0	0	0	0	0
d92d7c82782172666b30a58 2915966f1	0	0	0	0	0	0	0	2	0	0	0	0
36f4ef23fa5c93c2025847b5a 1623065	0	0	0	0	0	0	0	0	0	0	0	39
cf6cf77d21ea66ea21b9fd64c a66886f	0	2	0	0	0	0	0	0	0	0	0	0
b2563eb28a3dd323e243232 45250fdb6	0	0	0	0	0	0	0	5	0	0	0	0
f83b4b97a22a4b1b9d4fae83 f3eb03d5	0	0	0	0	0	0	0	0	6	0	0	0
b01fbf9c5714a635559df312a c2e651e	0	0	0	0	0	0	0	0	0	0	0	3
19065f28483a393689a0e5e7 c382114f	0	0	0	0	0	0	19	0	0	0	0	0
e37d18960fa64217d3fdc161 820351d4	0	0	0	0	0	0	0	0	0	0	60	0
53bf1ae7d7e7138d1d80edba a175cb4b	0	0	0	0	0	0	0	86	0	0	0	0
6d4c8a99d0b17e13cf4f06b7 9e8d4b24	0	0	0	0	0	0	0	0	0	0	0	3
8aaf5c35c1d7cdd43c8fb3ea4 917ad5e	0	0	0	0	0	0	0	0	0	0	3	0
58abe57637a1e11f00fae450 c519a907	0	0	0	0	0	0	0	0	0	2	0	0
f582b44db69453fe8dbeac09 16342a64	0	0	0	0	2	0	0	0	0	0	0	0

45c4b9501ec5c9e580f44317fc8b3904	0	0	0	0	0	6	0	0	0	0	0	0
062d1b163be1a892ed09d9a163e5a24b	0	0	0	0	0	0	5	0	0	0	0	0
232c5b9466c4589b3579d44c36b99fdb	0	0	0	0	0	0	0	0	20	0	0	0
8765d3b80519da9c58582bcee0828260	0	0	0	0	0	0	0	0	0	0	67	0
378621435e9ea068847b54c1c25aeb0c	0	0	0	4	0	0	0	0	0	0	0	0
70e7f50f1e3646fc2d6c1df2dae12fda	0	0	0	0	0	0	0	0	0	0	2	0
5cb603eac102eff083cb3f31228f5b69	0	0	104	0	0	0	0	0	0	0	0	0
ac763463cacc0e87564fb04e5f106145	0	0	0	0	0	6	0	0	0	0	0	0
56e5329e446f96955d78c04d133f7d62	0	0	0	0	0	0	0	0	0	0	4	0
fa8541f518b148225deab9c5a01a754b	0	0	0	0	0	0	0	0	0	0	4	0
c708e5f54556fdc0eb8c07b0caa258fe	0	0	0	0	0	0	0	0	0	0	3	0
871f9e60211e17790fb7486d5ad2ca67	0	0	0	0	0	0	0	0	0	18	0	0
f73a99f9f5ed2bb11f7cdc0125328f77	0	0	0	0	0	0	0	0	0	0	4	0
27aa3758377e7d19b6c5fe98f94cf4a8	0	0	3	0	0	0	0	0	0	0	0	0

8c0e152aabaf2079f1884f1160d8946e	0	0	0	0	0	0	3	0	0	0	0	0
d3d51cfdb5243ba2c8513b797adb7df8	0	0	0	0	0	0	0	5	0	0	0	0
2ef07be29e707447d09c3d813b515197	0	0	0	0	0	0	0	0	0	3	0	0
d498c610c3807bf3b5c3dfb04e12ce17	0	0	0	0	0	0	0	0	0	0	0	8
6af0209a08ab37e511647c88edd3e8d8	0	0	0	0	0	4	0	0	0	0	0	0
7da4b50ced8f3fd139ca097f441c2da6	0	0	5	0	0	0	0	0	0	0	0	0
9d5e39a23499855eb456a17974c2b3f7	0	0	13	0	0	0	0	0	0	0	0	0
4d746d1db45715a1537b2ec139c20767	0	0	0	5	0	0	0	0	0	0	0	0
65cc6196c1d7c2b2377178de1221cba1	0	0	2	0	0	0	0	0	0	0	0	0
3fc0ac48c434a9063fa10d70592b4bc6	0	0	0	0	0	0	0	0	0	0	29	0
5e16fc85dddd126ae78de8d1049e494b	0	0	0	0	0	0	0	0	0	2	0	0
f348d10e1a4fb18cc4943065adbcf29b	0	6	0	0	0	0	0	0	0	0	0	0
4e9b2e5845230998eb31185e2c6d5a9c	0	0	0	0	0	2	0	0	0	0	0	0
a6062f7ced4654c18e93b3e94adb2050	0	52	0	0	0	0	0	0	0	0	0	0

2e21a173a1e49826ca03efb6ca45b95e	0	0	0	0	0	0	25	0	0	0	0	0
d5ddf4bde2b6309f4b14c07c746919a0	0	0	0	0	0	0	0	6	0	0	0	0
935c0abf8a885ee50eed9ee7f5fd5f86	0	0	0	0	0	0	14	0	0	0	0	0
8d8b9fed713b6e3df0fc262e33273a8d	208	113	0	0	0	214	0	245	0	351	359	0
bb3c493ecf8243d2586489e9858a7aaa	0	0	0	0	0	0	0	0	0	0	0	2
d8f0721de33a51545f36779a2dfab988	0	0	0	0	0	0	4	0	0	0	0	0
9563a1cd367bcea293797c1e83d2d350	7	0	0	0	0	0	0	0	0	0	0	0
e4723e4f96c775d54747bce6b117652e	0	0	0	0	0	0	0	0	0	31	0	0
02447296ed75392572b4b90afad511cd	0	0	0	0	0	0	2	0	0	0	0	0
e52523866f5df72d19458c0f7d999314	0	0	0	0	0	0	0	0	0	0	4	0
69f7f388c6631bc515d318795b96d72a	0	0	2	0	0	0	0	0	0	0	0	0
06474d7ef903f4eb6278f083747f5da4	0	13	0	0	0	0	0	0	0	0	0	0
f00b402471836d4d91db54b7b57e0474	0	0	0	0	0	0	0	9	0	0	0	0
b68705556ed518cfe96971a0d489d615	0	0	0	0	15	0	0	0	0	0	0	0

fbd9797e0daff32f5d77ab83967406c9	0	0	0	0	0	0	0	0	0	5	0	0
2dec062a5a4247486b264fedae7a530e	0	0	0	0	0	0	0	0	2	0	0	0
b01680f7b640d54e17805d978f8bc699	0	0	0	0	0	0	0	0	0	0	0	3
00bd19e5117fd3d1b223af88bfc8705f	0	0	0	0	0	0	0	0	0	0	0	3
2807545a0b34409e068300176b697840	0	8	0	0	0	0	0	0	0	0	0	0
de9ef9bf10977af4564b15d1103e61cb	0	0	0	0	0	0	0	0	0	0	9	0
e08c633a6fb45b0ab69e734eea40da37	0	0	0	0	0	0	0	0	12	0	0	0
65c50ee78ab6da34d4f4d1b83c714eb9	0	0	0	0	2	0	0	0	0	0	0	0
56a831fe4c385b1eb806a55343fed7c4	0	3	0	0	0	0	0	0	0	0	0	0
6a16d44221a08c169a16aea2eb04d860	0	0	0	0	0	0	0	0	0	0	0	2
06a59666cbc33b393ea7f51be91b6355	0	0	0	0	0	0	4	0	0	0	0	0
42d4e7e91cdf51917f95a2480a8f3b1a	0	0	0	0	0	0	0	0	0	0	0	6
5543a4cd0268a48c0c5a7893cd785fcd	0	0	0	0	0	0	0	0	0	0	25	0
41a551baef1e49a4970d00c00f8fd7d1	0	0	0	0	0	0	0	10	0	0	0	0

7c642e35d7c0f76b608551bf826f69b4	0	0	0	0	0	30	0	0	0	0	0	0
23d90d51ac95115f12661f70e2c2bc5c	0	0	0	0	0	0	0	0	0	0	37	0
b9f5315ba7fb392d92fb0a4dc5d647b7	5	0	0	0	0	0	0	0	0	0	0	0
73b9474ff36ac5d9df57393edac6fb75	0	0	0	0	0	0	0	71	0	0	0	0
434c8ea80e95c4e2d9fdd8f54deeb33e	6	0	0	0	0	0	0	0	0	0	0	0
378bfa015c714e2daeed26bc9f9f7fd6	0	0	0	0	0	0	0	0	0	0	2	0
ca2c0c18dc6378c688fa4ac0ddd20581	0	0	0	0	0	0	0	0	0	4	0	0
9d0dc1e4d1b2b9d19e86de33b597bcb2	0	0	0	0	0	0	0	3	0	0	0	0
05187580a27a5ef73eefef2536797b0a	0	0	0	0	0	0	0	0	0	2	0	0
80b7f5c9083435426f6c7668f8ffc8aa	0	0	0	0	16	0	0	0	0	0	0	0
fd64c0ec9189b952dec48f948daa4cda	0	0	0	0	0	0	15	0	0	0	0	0
b3f6c3f262c2c953edc7890f3d55a9e4	0	0	0	0	0	0	0	0	0	3	0	0
6232a9381c8a4c7ea627535b84f55b0e	0	0	0	0	0	0	0	4	0	0	0	0
bfb083cf6ddada627824a00526777fc2	0	0	0	0	0	0	0	0	0	0	125	0

a974e431017fbbbea0476ee58d5a45a2	0	0	0	0	0	0	0	0	0	0	2	0
ef7a43505b2b7fed67421b0a618b6abc	0	0	0	0	0	0	0	3	0	0	0	0
157098f48f182fc532acedbaac29d9a8	0	0	10	0	0	0	0	0	0	0	0	0
12bb058b8c3380ea82d70b0bd9f11f44	0	0	0	12	0	0	0	0	0	0	0	0
c0acc59193704fdd6f3f6e41349c7371	0	0	0	0	0	0	0	0	0	0	6	0
1f4784ade12a5a3d51ed61810351da18	0	0	5	0	0	0	0	0	0	0	0	0
bd6fb43d3a5c83cc7e40f05346fcee6c	0	0	0	0	0	0	0	0	0	0	0	11
94a3c217ea42ceb97d568640b57cb1fb	0	0	0	0	0	0	0	0	0	8	0	0
bc900959def2d9df43e66b7886d19295	10	0	0	0	0	0	0	0	0	0	0	0
1ffd62de5ba259cfb132867d5780107b	0	0	0	0	0	0	0	0	0	0	0	50
e5dc78e2c68c1df1e7ee1bc7dfc1c9d2	0	0	0	0	0	0	0	0	0	2	0	0
6eb67a131928b3ddbd952e78743f03f7	0	0	0	0	0	0	0	0	0	4	0	0
b5726dfb47f0265c85fbeb1bc0a06c66	0	9	0	0	0	0	0	0	0	0	0	0
94f07842e0246022cc6bb362634fd96e	0	0	0	0	0	0	0	0	12	0	0	0

3ba705d33d96ccaa8fe55a73d7223043	0	0	0	4	0	0	0	0	0	0	0	0
9b45c18d0d9a1c2fb31f45b4c2cc9622	0	0	0	0	0	0	0	0	0	0	5	0
c34bc8c4354f2548dc7d95b0143d3d3f	0	0	0	0	0	0	0	0	0	12	0	0
9f5324e17643a57ab2140f86823cd0f3	0	0	0	6	0	0	0	0	0	0	0	0
4f021c22ed16a79ce557ab99acd469c4	0	0	0	27	0	0	0	0	0	0	0	0
bd31f5ce3526f7694e4cc704b7176c74	0	0	0	0	0	0	2	0	0	0	0	0
39602dec6b9dc0fbb44dfad12c662f43	0	0	0	35	0	0	0	0	0	0	0	0
39032521279db48bfd825caa1e35d093	0	0	0	0	0	0	0	0	0	0	3	0
db54bb88d83372b347a8ce3715a331f9	0	0	0	0	0	0	0	0	0	166	0	0
653619cc724e3f119d0de314b6645b2c	0	0	0	0	0	0	0	3	0	0	0	0
b25c5f336c0cd12c6cf3791c8f88af88	4	0	0	0	0	0	0	0	0	0	0	0
64ccf4604a0fdb0bd089643514c53970	0	0	0	0	0	0	3	0	0	0	0	0
4db95eb225fdf27a4e8a113c154aaad2	0	0	0	0	0	0	0	0	0	0	16	0
c1d561a83b30a97d84898db69605a176	0	0	0	0	0	0	0	0	0	16	0	0

965339c88d0b38a83a22ff9a5a3299b2	7	0	0	0	0	0	0	0	0	0	0	0
d5c2d6830ec265af4598842edae29f11	0	0	0	0	0	0	0	11	0	0	0	0
526789bcef07585d4fe44857fd81fff6	0	0	0	0	0	0	0	0	0	0	28	0
aa9f7564f29e677e363cf3a03ac937d2	0	0	0	0	0	0	0	0	0	0	4	0
a7b9fc4e41f55620b7d7308a4204f818	0	0	0	0	0	3	0	0	0	0	0	0
a980853b1c8ac5bab7222f2d6d08a244	0	0	0	0	0	0	0	0	0	0	6	0
169d0c683f76b528792dd58e343bbe69	27	0	0	0	0	0	0	0	0	0	0	0
0ce4407aa1d5af666014d88a717afcfc	0	0	0	7	0	0	0	0	0	0	0	0
0c96b99300c98552001bf40b1688dff2	0	0	0	0	0	0	0	19	0	0	0	0
64029bc111bdd368ab44f6239dc1f7b3	0	0	4	0	0	0	0	0	0	0	0	0
1160fd5dd87a4b67b49dc620d6379dbf	0	0	0	18	0	0	0	0	0	0	0	0
a17bab6b8fd1f004ea34939a851c516e	0	0	0	0	0	0	0	0	0	0	0	34
c61d3c4c361d64e797e14e7fcaae38a5	0	0	0	5	0	0	0	0	0	0	0	0
bf5611d23e74966b2a95e34fc5d741e4	0	0	3	0	0	0	0	0	0	0	0	0

1198408bc6708bed0a96d90b0d19a0a0	0	0	0	0	0	0	0	7	0	0	0	0
65d006281a468e12722e245db31bd27f	0	0	0	0	0	0	0	0	0	0	0	50
47ef47cf24218b677dcbfb0099b11a01	0	0	0	0	0	0	0	0	0	17	0	0
b9bb7d5b9de97b84e8cc7a2eaf6fee1b	0	0	0	0	0	0	0	0	3	0	0	0
e847027069c6fa5b35e20d2c5fe3016a	0	0	3	0	0	0	0	0	0	0	0	0
1e97037c2b4c08921983efc136708d70	0	0	0	0	0	0	0	0	0	0	7	0
65c2eba239d26cedf9404a80cb096f9e	3	0	0	0	0	0	0	0	0	0	0	0
10f7ac048bf0bff0d03286f9e6c74696	0	0	0	166	260	0	0	0	0	0	0	0
878ec1ee5f6d6d5305d01481a6f9f555	14	0	0	0	0	0	0	0	0	0	0	0
4c1fc72ad263341577bd8358a4d23619	0	0	0	0	0	0	0	0	0	0	2	0
2dcf24776bc31bc6f063c0eafd0493b2	0	0	0	0	0	0	0	21	0	0	0	0
847a98e1df7296195f2533d3ec0504a6	0	0	0	0	4	0	0	0	0	0	0	0
d87891bc8e44cf7ffcd5db647a8ab64a	207	0	0	0	0	0	0	0	0	0	0	0
7092848e55d2868c1892fa0adf347007	0	44	0	0	0	0	0	0	0	0	0	0

01b1ddbf5c22675bf4fbb45a d3b89e26	0	0	0	0	0	3	0	0	0	0	0	0
520887e6930175d3e318a70 b5f4ec2e4	0	0	0	0	0	0	0	0	0	0	0	2
592c92c068d445fbb279751f d4c53a54	0	0	0	0	0	0	0	6	0	0	0	0
a04d3c9b86a52e25444dcc63 312fc6a0	0	0	0	0	0	5	0	0	0	0	0	0
463236b112a74e889ecba54f a25e9cf1	0	0	0	0	0	4	0	0	0	0	0	0
18b2321e4d93f4b66cf0da6b a3da0381	0	0	0	0	0	0	0	3	0	0	0	0
3a00c67e11f1234d0075eaf2 bff34153	0	0	0	0	0	0	0	0	0	0	0	9
d34943999115208d7127ed7 5c31f88cb	0	0	0	0	0	0	27	0	0	0	0	0
631dc363eb3974a51654084 76cf819c9	0	0	0	0	0	23	0	0	0	0	0	0
5936002e6427359fc430c0e0 a7a8b121	0	0	0	0	0	0	3	0	0	0	0	0
450ea7c116b17e5274b4fb7e ebd2c775	0	0	0	0	0	0	0	0	0	0	24	0
1c0382c17615fd7b8be61963 a21f2165	0	0	271	0	0	0	0	0	0	0	0	0
2c7031b77a12b8b19ed8839 223ba1bb9	0	0	0	0	0	3	0	0	0	0	0	0
fb7624862fa25ecca7e917f1b cf3619b	3	0	0	0	0	0	0	0	0	0	0	0

5bebb7015aac51ab5db9302a61d83b80	0	0	0	0	0	2	0	0	0	0	0	0
0b92414957a18d2d5a13ffa7bf8f24f5	4	0	0	0	0	0	0	0	0	0	0	0
d681f62c1bf65a15c687764e04210730	0	0	0	0	0	0	0	0	0	0	0	4
6eef20ef27f6f4adad00a0618441ec12	0	0	0	0	0	0	0	0	0	0	4	0
2c61dfdca0db384b5be2636c6ab9dd5a	0	0	0	0	0	0	0	55	0	0	0	0
cc431013fca4523a83f3104089928d4f	0	0	0	0	0	0	0	5	0	0	0	0
b0888b7d129ab19c651b65deb10eca3f	0	0	0	0	0	0	0	2	0	0	0	0
b4f7e9715192c3ac1f53ea3d842ecbf3	0	0	0	0	0	0	0	0	0	0	0	2
79617a30cb94ccd36c680c2ca948977d	0	0	0	0	0	0	29	0	0	0	0	0
da77012061a4cd7ee19a6d8cc8a58af7	0	0	0	0	0	0	0	0	0	0	0	3
4b449dfd1a9313053d47ccc2af77cf8e	4	0	0	0	0	0	0	0	0	0	0	0
a96669e1338856b77dd7c352f4c85b0a	0	0	0	0	0	0	0	0	0	0	7	0
c54817220570ad949d16e4f8da9d2057	0	0	0	0	2	0	0	0	0	0	0	0
70b2197042b837fbad615ada7edb8ed2	0	0	0	0	0	0	0	3	0	0	0	0

6490d260b9cba338fefbf849d4beefc3	0	0	0	0	0	0	7	0	0	0	0	0
9b3308cf673118fd16afe2cb4dddf52a	3	0	0	0	0	0	0	0	0	0	0	0
2d5423e5272476513d13a769152f63b5	0	0	0	0	0	0	249	0	0	0	0	0
1c21dc4057421ede576181690b54f1d2	0	0	0	0	0	0	0	5	0	0	0	0
898a9bc3b742ac09a40886041c0f163e	0	0	0	0	0	4	0	0	0	0	0	0
546de3664522112bfd9274d3879134a0	0	0	0	0	0	0	0	0	0	0	0	2
f2824e9e37fd116aa9b1356d52604bd1	0	14	0	0	0	0	0	0	0	0	0	0
6ed0793ef7ad0292941386f1ce94de69	0	0	0	0	0	0	0	0	0	0	0	3
2ea669f8a75bd4c4a6877fc151340aab	0	0	0	0	0	0	0	0	0	0	10	0
c7ff1715cc8c26d815d5522db99e9328	0	0	0	0	0	0	0	0	0	0	0	5
aee8767b67f73200ec0dcc07bbb70f43	3	0	0	0	0	0	0	0	0	0	0	0
9e242f4db5cc708bd0be58bb0c50ed40	0	0	0	0	0	0	0	0	0	0	2	0
0758abc16abcebf15ef00235806df5ad	0	0	0	0	2	0	0	0	0	0	0	0
7bfc6a074359f7f33699c0403a8b4140	0	0	0	0	0	0	0	0	8	0	0	0

0f61ab6be0c8b2bf27cef19dd1ebddc7	0	0	0	0	0	36	0	0	0	0	0	0
40ec95baccdae270043af187b003d9f6	0	0	0	0	0	0	0	0	2	0	0	0
0b3b5b64a11ecd988ef59cd62b645650	0	0	0	3	0	0	0	0	0	0	0	0
cadf16bd141c79cf194a825649f16f77	0	0	0	0	0	106	0	0	0	0	0	0
27721a9e85c5d651ede710894cd46ad2	0	0	0	0	0	0	0	0	0	0	0	2
58f1c8a820aa1e70bf03ebbdcc840de6	0	0	0	0	18	0	0	0	0	0	0	0
13ed59672ab1d495a781de4195a7599b	0	0	0	0	0	0	0	0	0	0	5	0
7b9b45a68662ab1fc4e07760ad830d5d	0	0	0	0	0	0	0	0	2	0	0	0
9f6855bff3c74d60d10f1ebc255bc8ef	0	0	0	0	0	0	0	2	0	0	0	0
081c3787079638409c7e1483c963be7e	2	0	0	0	0	0	0	0	0	0	0	0
a4477a3fc9b94cf87aafd0efdb87f87d	0	0	2	0	0	0	0	0	0	0	0	0
a30de556974bde8eb04d6151c3379036	0	0	0	0	0	0	0	10	0	0	0	0
54ba3a3bfc8aa90c9713b294567b998f	0	0	0	0	0	0	0	0	0	0	0	21
10e9659b537c3a36c2879510e1b940d7	0	0	0	0	0	7	0	0	0	0	0	0

b70e46db689935faeeaa649f57be0591	0	0	0	0	0	0	0	0	0	0	2	0
599a17a57206d9d6ee3889efdfeef473	0	0	0	0	0	0	0	0	0	0	0	4
f544da6efe42bde6c69be8c5f5b98a25	0	0	0	0	0	0	0	2	0	0	0	0
ec34f9562bd36419fc0d58cd528b00b8	0	0	0	0	0	0	0	0	0	0	2	0
16fa6df2f74ab12924a93965b645b553	0	0	0	0	0	2	0	0	0	0	0	0
c1dec75171c8026d0420a9eba62352e3	0	0	0	0	0	0	0	3	0	0	0	0
62922b4f8703e19a776a140e4d0dc7ad	0	0	0	0	0	0	0	0	9	0	0	0
50bb9c991ab63d728b8dee97017cab9b	0	0	0	0	0	0	0	0	0	0	7	0
d1ed70986425cfd08bfd4baf9a1d8c3c	0	0	0	0	0	0	0	0	0	2	0	0
e41c65c422616b70d1b3f94cec47aa1d	0	0	0	0	0	0	0	0	0	3	0	0
1966a2834e5c1c53cfb7dbe48b77ad2d	0	0	60	0	127	0	0	0	0	0	0	0
ebca52c449ba8f03d0861a95eeaf30bc	0	0	0	0	0	0	0	0	0	18	0	0
23f62679d3c04de66c0d2f4a3222bc99	0	0	0	0	0	42	0	0	0	0	0	0
fd5ba16f893e17d5e0095bb66f4be6da	0	0	0	0	0	0	0	0	0	0	7	0

1cfe2406c00ab8d20c4720ae efe928dc	0	0	0	5	0	0	0	0	0	0	0	0
25c3f1e8bee93e383e117dc1 c3435dcc	0	0	0	0	0	6	0	0	0	0	0	0
aca64473b0de5d87c666ed70 18e1f36d	0	0	0	0	0	0	0	0	0	0	0	8
ae6e62fa3698542c634a9180 6bd38fe0	0	0	0	2	0	0	0	0	0	0	0	0
53f092674d99792b0418438c 7d4b043c	0	0	0	0	0	0	0	21	0	0	0	0
f47e8ed46d5c79137485f392 2437b621	0	0	0	0	0	0	0	14	0	0	0	0
d0b33090af07f648cd18f3eab f8b1d19	0	0	0	0	0	0	0	0	17	0	0	0
ba8bb85b183bc2881e16bc5 6509d7b91	2	0	0	0	0	0	0	0	0	0	0	0
ec70f15cfb68f4c5f33d3abe1 55076b4	0	0	0	0	0	0	0	0	0	0	0	4
ede10b479bfc7853c1f658f4c 2795e36	0	0	0	0	0	0	3	0	0	0	0	0
9e050b179502180d97d9c1df 58690dc6	0	0	0	0	0	0	0	0	0	0	0	38
0e35f8d2df850807b88036ae a602d201	0	0	0	0	0	0	0	0	0	0	0	4
f907ca0159da8221b18d1cec f86e6d2c	0	0	90	0	0	0	0	0	0	0	0	0
7c64769ce0b5bd64417e543 1c37384cb	0	0	0	0	0	0	2	0	0	0	0	0

fdbbd27b2f21a96ee5e982e07a7bbb8f	0	0	0	0	0	0	0	0	0	0	3	0
6d2dfef0ddd00417a177575a5193d4f1	4	0	0	0	0	0	0	0	0	0	0	0
0b3d641a95494c41c3a87ad388caf2aa	0	0	0	0	38	0	0	0	0	0	0	0
bd3202e02d25d87f6ad9d4ed0adf692a	0	0	0	0	0	0	0	0	0	0	0	4
17e0cc5254712e625559408e90e339a6	17	45	0	0	0	0	0	0	0	81	0	0
110587a6397bc47b6a1203020aaf11e5	0	0	4	0	0	0	0	0	0	0	0	0
2d6c161897ad42517aad6c99441db9b5	0	0	0	0	0	0	0	0	0	0	2	0
7243fc79b817ac1ad80ea7910fe55cdd	0	0	0	0	0	0	2	0	0	0	0	0
189016b2a41dfc1b3031da1cbec9f5b1	0	0	0	0	0	2	0	0	0	0	0	0
31b544a25b4a2dab63b5fe100a60b92e	0	0	0	0	0	0	0	0	0	0	0	6
4b712a72386e92993f9cbdb62e0f4b96	0	0	0	0	0	3	0	0	0	0	0	0
9dc687e675d25c1c3daf43932e3c1f00	0	0	0	0	0	0	0	0	0	0	3	0
3a4af229036a7b9c945e4312b465cb59	0	0	0	0	0	0	80	0	0	0	0	0
79680f20065c944dc44bcdca31b73968	0	0	0	0	0	0	0	0	6	0	0	0

852118bc3fcde1e13e784c6155cdd651	0	0	0	0	0	0	0	2	0	0	0	0
a724a0f9b4bc885f873bb43ae2e03ce1	0	0	0	0	0	0	0	0	0	0	8	0
33fa5a2edaedeaf79477e6f2c9450fc9	0	0	0	0	0	0	0	0	0	4	0	0
6d7cf6d99e0f8faf96602cdb40c1cf36	0	5	0	0	0	0	0	0	0	0	0	0
f90a7472debb5f19ada5323a3c889d8b	0	0	0	0	0	0	0	0	5	0	0	0
1104852a359a3a93ebd15400a737a3b9	0	0	0	0	0	0	0	0	0	0	0	2
f88cc37c4577c2646b76341ec244b0fa	0	0	0	120	0	0	0	0	0	0	0	0
cfa685fab1ed8d54b3da72488f12248c	0	0	0	0	0	0	0	0	0	0	0	22
6f904258f46f7040b8e1377b55a93cac	0	0	17	0	0	0	0	0	0	0	0	0
032002d56a41b16bb8a9bf1c819586f0	0	0	0	0	0	0	0	0	0	0	4	0
f09070f0bad9e01389a206897145d9f7	0	0	0	0	0	0	0	0	0	0	21	0
cc9a88e2ef82c46c0bc623abc7bf5bc8	0	0	0	0	0	14	0	0	0	0	0	0
15c2d19397dc3d90fb37381a0d020114	0	0	0	0	0	0	0	0	0	24	0	0
37fd5e5ed22a7e088de51e79e6e869ff	0	0	0	0	20	0	0	0	0	0	0	0

4a040bb60eb85c2cd91c3dfa22d653c3	0	0	0	0	0	0	0	0	0	0	4	0
6ef7133ae9f4f2ad9d2d8e79d83d9d2e	0	0	0	0	0	0	0	0	6	0	0	0
bf3e480dd59e9a6152b62632d7e1b68a	0	0	0	0	0	0	0	0	0	0	0	5
1c129365c91a4b13174a0147cd0f0d14	0	0	0	17	0	0	0	0	0	0	0	0
5d17b1e2bfb8565a478a598874e55384	0	0	0	0	0	0	0	0	0	0	2	0
25b41d9dc6dcc44b3692aa7d5989845a	0	0	0	0	0	0	0	11	0	0	0	0
c50ad5d3f425aa01f041583b6bf5b48b	0	0	0	0	0	0	0	0	0	0	0	16
dfb2f8c5e7c42864f3e61a457f68884e	0	0	0	0	0	121	0	0	0	0	0	0
f0ef261d5d716cdee3becf831357075a	0	0	0	0	0	0	5	0	0	0	0	0
bf3e2437d083e7656071459f5e44ad60	0	0	0	0	2	0	0	0	0	0	0	0
5ad3d7b45fb2d07d6e4e592b5d37e69a	0	0	0	0	0	0	0	0	5	0	0	0
bddb5db1cb07a2f159cfaffed11c063b	0	0	0	0	88	0	0	0	0	0	0	0
e05f13bcf52fa7260ceb764d7cb99964	0	0	0	0	0	0	0	4	0	0	0	0
af1b9fe1bec758c561d7b0bd549322a3	0	0	0	0	0	2	0	0	0	0	0	0

a4674cd5d9b8291c7ba90d5d2f0cdcc1	0	0	0	0	0	0	0	4	0	0	0	0
1dbb96b769c53a84b169467e669210f9	0	0	0	0	3	0	0	0	0	0	0	0
775d44a2a475ca4de88d65ae5d52baab	0	0	0	0	0	0	3	0	0	0	0	0
c562581be53f9eeadaa4eac219f68f85	0	0	0	0	0	0	0	0	17	0	0	0
0806af1e92e547a80d1fa6686f54410f	0	0	3	0	0	0	0	0	0	0	0	0
e6f5c478bbf52f6a05dba23fdcaffded	0	0	0	0	0	0	0	0	4	0	0	0
518ca948208762aa8ed5f72748d97cc8	0	0	0	0	0	0	0	0	0	2	0	0
4654470e8590e2d8a14197861b7b74be	0	0	0	0	0	0	0	0	0	0	0	3
99298a1baefa33c0f5b42f513805e147	0	32	0	0	0	0	0	0	0	0	0	0
63121d764aad7aca04e8173c67d0dc59	0	0	0	0	0	0	0	0	0	0	0	14
315650de62e819eccd849358d25521a8	0	0	0	0	0	0	0	0	0	0	9	0
ac8e4f3bf17ee550394ff9736ce5fd1c	0	0	0	0	0	0	0	35	0	0	0	0
e4da62a1619feca021c3240a05685edb	0	0	0	0	0	0	0	0	4	0	0	0
9ee9ad7f40a967a3a47ad0f370c73246	9	0	0	0	0	0	0	0	0	0	0	0

03c7ea06beee8ca658f532a8a9436ee4	0	0	0	0	39	0	0	0	0	0	0	0
e5132a4da3888b5f1961c4f65664bb8b	0	0	0	0	0	0	0	8	0	0	0	0
d1ad2de978da60251ad60673345afde4	0	0	0	0	0	0	0	0	0	35	0	0
7ad51ea9a1d9c64032ff81ac07db06ea	0	0	0	0	32	0	0	0	0	0	0	0
8d0884be348939b496ae628eef1dc647	0	0	0	0	116	0	0	0	0	0	0	0
ccae3d0d1d0eb9bcb017538e1b473ff2	0	0	0	0	0	0	5	0	0	0	0	0
bc88338ae60d1801d88b1712661fc95d	0	0	0	5	55	0	0	0	0	35	0	0
684717e69b7d680d2f9cdac070ffe322	0	0	0	0	0	0	0	2	0	0	0	0
4b050725172d17632ab21c5e80685579	0	0	0	10	0	0	0	0	0	0	0	0
62741293cb3db99432d550e77a05ed97	2	0	0	0	0	0	0	0	0	0	0	0
8371f168e867f86fbd0d9be92edbaeb3	0	0	0	0	3	0	0	0	0	0	0	0
3de768010fcb2d5e8539d670d347c628	0	0	0	0	0	0	0	0	0	0	0	10
ba0cd6de063dd7cd7876152ef641e289	0	0	0	0	0	0	2	0	0	0	0	0
151b0fdc9118c8fb275bbddeee3ef2e5	0	0	0	0	0	0	0	0	0	0	2	0

2708b6e72f24be4ae8f90fb3b6a9c86d	0	0	0	0	0	5	0	0	0	0	0	0
89e71a3ea50503fe6e1a765c58ea4a27	0	0	0	0	0	0	0	30	0	0	0	0
4783dd55b11cc44053c05341e6086c0e	0	0	5	0	0	0	0	0	0	0	0	0
e068505f1e6e6663c59a4dcef37c5e30	0	0	0	0	0	0	0	0	0	0	11	0
181ca5fdad161f2d7d92c1a6c67fa871	0	0	0	0	0	0	0	0	0	0	35	0
1460c73e9183ff84b87cf551b4173619	0	0	0	0	0	0	0	0	0	0	0	107
dec84fbc0dd0f67a18a8359eae2296d7	0	0	0	0	0	0	0	15	0	0	0	0
e0fe7bf8e3c3a1cc2adaf91c90aab0ab	415	0	0	0	0	272	0	0	0	0	882	528
c4b7a3964b4911aeaaf483e99dc9bc80	0	0	15	0	0	0	0	0	0	0	0	0
9b72a29b8c288f8d776f9fcf6d0db372	0	0	0	0	0	0	0	0	0	0	6	0
570e599c48bb40b01a3604690d8ee79e	0	0	0	0	0	0	0	0	0	0	0	31
d58bc2fc9e3b96e57f728a5dfc896c9e	0	0	0	0	0	0	3	0	0	0	0	0
a4e5360868a6d5c60504e448186f45f8	0	0	0	0	0	0	0	0	0	0	0	3
fa9f0d55f64d2724433395f73cf6b2c7	0	0	0	0	0	3	0	0	0	0	0	0

4e575ee3f23cc075679eced8403215b9	4	0	0	0	0	0	0	0	0	0	0	0
f26989c55a9bf29c248260d658519d52	4	0	0	0	0	0	0	0	0	0	0	0
cd7e7a24997f83c135b505d43ef15fe7	0	0	0	0	0	0	0	0	0	0	0	10
d0cf667cc60aeef809fbbe5f072cff23	0	0	0	0	0	3	0	0	0	0	0	0
921d083fc77fc56d0d7574ac1b4f4e37	2	0	0	0	0	0	0	0	0	0	0	0
dec4d8ddea200c1f231b3c80982b4914	0	0	0	0	0	0	0	164	0	0	0	0
6e83c7fab07af0fb2d587eef7ec3c924	0	0	0	0	0	0	0	0	0	0	0	30
c3881d4a7f643efc3bfb1b6a44db7dbc	3	0	0	0	0	0	0	0	0	0	0	0
66e4f482d40320201bf57899aedf2ea7	0	0	0	0	0	0	0	0	0	0	2	0
f4bd82f4261469601c48b27d9caa1788	0	0	0	0	0	0	0	0	0	0	3	0
3d1a97b6398a56e4d9ecc577b685f37b	0	0	0	0	0	0	0	0	7	0	0	0
adbff95dcb90bc76f3d869b075c6cc9f	0	0	0	0	0	0	0	0	0	6	0	0
27f2fb6a5a7a1b6c805256ea8e6e3c41	0	0	0	0	0	14	0	0	0	0	0	0
3d47ff6fcd4f45413b329af885c5ea7a	0	4	0	0	0	0	0	0	0	0	0	0

76ea4c8a3d949f57312ff35dd965a446	0	0	0	0	0	0	0	0	0	0	0	2
97b849404d4d9d2ac412019695b7ecb0	0	0	0	0	2	0	0	0	0	0	0	0
63b1faed6c34588f3d0f94ade3610633	0	0	0	0	0	0	0	45	0	0	0	0
35ff85ec8ee15d85e7cf244074b37ded	0	0	0	41	0	0	0	0	0	0	0	0
93bb916eda5200913ea3e24e0ae9d603	11	0	0	0	0	0	0	0	0	0	0	0
0e4fec32b1e66aa35707c1d686c2c03e	0	0	0	0	0	0	0	0	0	32	0	0
8752a662215b8b901f3c0d8f508f588d	0	0	0	0	0	0	0	0	0	0	0	3
582a32dbe6eef26ed56d2dc5472adec9	5	0	0	0	0	0	0	0	0	0	0	0
1ea5b4307e7ddfea8c98a2073bc094a7	0	0	0	0	0	0	0	0	0	0	7	0
0b73d0999ce8d8af38a1cb5eab7b3ec5	0	0	0	0	0	0	0	0	0	0	20	0
5db8966accec54da64f01d19059ae16e	0	0	0	0	0	0	0	0	0	0	0	3
5d335b28a03aa0857c13862e6c3bd140	0	0	0	0	0	0	0	0	0	0	0	49
0df86f5eb417f2dec49e1bcd439fcdae	0	3	0	0	0	0	0	0	0	0	0	0
7639f7436588b77e509374d368b5b666	0	0	0	0	0	0	0	0	0	0	0	2

8942644e6a5bd1db2715454 57202ee10	0	0	0	2	0	0	0	0	0	0	0	0
b1a8c312ab5238dcd2db461 8ad34e593	0	0	0	0	0	0	0	0	34	0	0	0
4761986de79b867d25b9243 6513b4088	0	0	0	0	0	0	0	0	0	6	0	0
d1a86c06af18721f0ca1637b dde9a3c1	0	30	0	0	0	0	0	0	0	0	0	0
668b6ea48b82123f36cb3308 6bd50ee0	0	0	2	0	0	0	0	0	0	0	0	0
e0d53fa4ee8d9f8d8e46b105 c6ea0a10	0	0	0	0	0	0	0	0	0	0	122	0
0570de7f851ad0722505ef8b 9fa99ab6	0	0	0	0	0	0	0	0	0	0	60	0
a16c238866de672becffd6ad 0b800aa5	0	0	0	0	0	0	0	0	0	0	0	2
22a8c9f48ff0046e776e10723 57be514	0	0	0	16	0	0	0	0	0	0	0	0
7f929150e15772e398c778d4 3096f937	0	0	0	0	0	0	0	0	0	0	0	15
e81e78d7004d5a079cd102fc 96cea55d	0	170	0	0	0	0	0	0	0	0	0	0
a82f521bc470c87889b22437 b27a6ff9	0	0	0	0	0	0	0	0	0	0	46	0
8c8823a71ef8511df865e324 096199bc	0	0	0	0	0	0	0	0	4	0	0	0
d0984113625663a1c988b04 6a3c1f2e3	0	0	0	0	0	0	0	0	0	0	2	0

a5061fdbe8bad81c155fb401c4fbd6dd	0	0	0	0	0	0	0	16	0	0	0	0
52098773d38be43a8ffa2dfc4333d440	0	0	0	0	0	4	0	0	0	0	0	0
b309840a9b533ae055fcebf11e0a2888	0	0	6	0	0	0	0	0	0	0	0	0
bd2416c1b1e5ebe3254e6f18c97c318a	0	0	0	0	0	4	0	0	0	0	0	0
63b631b9f5ad6a98f082ad27475afde6	0	0	0	43	0	0	0	0	0	0	0	0
4b6f9b6f82c9feae5ac89ffa2575f9d6	0	0	0	0	0	0	0	0	82	0	0	0
712bac3c1d5a0a7064504b05b7d495cc	0	0	0	0	7	0	0	0	0	0	0	0
8f8ac9e39f1bf3f072e67f590a26cba8	0	0	0	0	0	0	0	0	0	0	0	15
aaf016691650ab027fc31722a3060d86	7	0	0	0	0	0	0	0	0	0	0	0
8e7dad507f717cdc1e6d40ca6b83f062	0	0	0	0	0	4	0	0	0	0	0	0
269c44ac2b7c446b449aa5bfe42efcbb	0	0	0	6	0	0	0	0	0	0	0	0
5ed6b808c9812468ab139b011c98815e	0	0	0	0	0	0	0	0	49	0	0	0
a3ba1dc8a11b9aa4b21309586d192a51	0	0	0	0	0	0	0	0	0	6	0	0
f58fd085f9b9ab354f5eebb251a05336	0	0	0	0	12	0	0	0	0	0	0	0

15ba7bf786a5c8dc50124e837311a86a	0	0	13	0	0	0	0	0	0	0	0	0
408426b329e40276d1acef63fe457b7e	0	0	0	0	0	0	0	0	0	0	3	0
539f2f0184760a8004335d76b8fd2d72	0	0	0	0	0	0	0	3	0	0	0	0
0804158c40c5b8d45168db86716c6b38	0	0	0	0	3	0	0	0	0	0	0	0
0d63333099489950d789498465a978e4	0	0	0	0	0	0	0	0	0	0	176	0
4d5cfdb70d7304a0eada941a17acc547	0	0	0	0	0	0	2	0	0	0	0	0
d313f783c2cfb980132040783686d03b	0	0	0	0	0	0	0	0	0	0	0	7
a4353c917e43159d6e5a667c9430c26b	0	0	3	0	0	0	0	0	0	0	0	0
cd419163ae6b90f7bd03243c65076082	0	0	0	0	0	0	0	0	0	0	136	0
3ac3d3a3b415853a80d42ce397e124b7	0	0	63	0	0	0	0	0	0	0	0	0
dce9cc340566f16527ead1cf963ce8d0	0	0	0	0	0	0	0	0	0	62	0	0
51bb39bc6d45bf615e69d9bef981d091	0	0	0	0	0	16	0	0	0	0	0	0
a66ba3bccc5a6d0dbbf23a1f23ad5ae2	0	0	2	0	0	0	0	0	0	0	0	0
2f0182a576a20de6876d46a7b5abc3ef	0	0	0	0	0	0	0	0	0	0	4	0

7aa12cb2a854d879a23c9110 5d84c92f	0	0	0	12	0	0	0	0	0	0	0	0
404fffcddca3b3a90e32f23c8c 1665b8	0	0	43	0	0	0	0	0	0	0	0	0
c6df71af818ae0c7843b2fbd3 22cb56c	0	0	0	0	0	169	0	0	0	161	181	0
8d05bc583876669128500db a3b84bd0b	0	0	0	0	0	0	0	0	0	0	24	0
ac2db0f01f370c0df326fd4d8 0684802	0	0	0	0	0	0	0	0	0	0	0	4
aac33e3b85c475ac757b1cf8 b597d723	0	0	0	0	0	8	0	0	0	0	0	0
89c649aeaeacba8b99796757 fd6b3ade	0	0	0	0	0	0	0	12	0	0	0	0
178b9022fe452008cf23137f0 8a79c03	0	0	0	0	0	230	0	0	0	0	0	0
594a741ac3228fa282ea44b6 5706269d	0	0	0	0	0	0	0	3	0	0	0	0
306f218f7f3c3e00aa3a068e1 ec7a0fa	25	0	0	0	0	0	0	0	0	0	0	0
ddde3470da5ace31cf07c1cf0 2f8da4f	0	0	0	0	0	0	0	0	0	0	3	0
66b9778de1f7407d1a1e4dfb 25bbb623	0	0	0	0	0	0	4	0	0	0	0	0
04b5dc9ab1e9205ddc9e30fb 12dbfd6a	0	0	13	0	0	0	0	0	0	0	0	0
c405cb737a3c399309b34b6c eae1dab7	0	0	0	0	0	7	0	0	0	0	0	0

802944285ceba59f8dad54f51f51e6ab	0	0	0	0	0	3	0	0	0	0	0	0
63296087fc55e382d3238642d4f7b53b	0	0	0	0	0	0	0	0	0	8	0	0
fb10078bc612a674c63c2b9fa6996ae1	0	0	0	0	0	0	0	16	0	0	0	0
657bcb6688bc9ed1a0fb5233c2a9569c	0	0	0	0	0	0	0	0	0	53	0	0
65885809c0f4b4a7c4f5c17fc3433db1	0	0	0	0	0	0	0	0	0	0	2	0
a313e73c6a0b6e26b7484711705517a2	0	0	0	0	0	0	0	0	6	0	0	0
9bfcd4137a2957ff1d62022c1a5d1a5f	0	0	0	0	0	0	0	0	0	4	0	0
1ee5ec02fa320fdbb7e889d8c3c44556	0	0	0	0	0	3	0	0	0	0	0	0
b0321ddb97770d4a006ce7f39c4a577a	0	0	0	9	0	0	0	0	0	0	0	0
31d71f53205490d4350c6ebc719a6aa7	0	3	0	0	0	0	0	0	0	0	0	0
1ec4b684919fdc6749e8c721740a545f	0	0	4	0	0	0	0	0	0	0	0	0
b50211391d30b23c4052371160794c04	0	0	0	0	0	0	2	0	0	0	0	0
14ee70816bc5c26ede24bf89f972a855	0	0	0	56	0	0	0	0	0	0	0	0
93d8426767f2ed9df0aa1cde7bc6b73b	0	0	0	0	0	0	0	0	0	0	0	34

576f8e7368b437bef7b7d29e545ca1d0	0	0	0	6	0	0	0	0	0	0	0	0
965e78c5dfd0bc50bdfa59e4b4d9af82	0	0	0	0	0	0	0	0	3	0	0	0
50b571e6eee52284e6a3cd248ce1bba3	0	0	137	0	0	0	0	0	0	0	0	0
f4732c78a7e3818d651f1c1dba0bc76e	0	0	0	0	0	0	0	0	31	0	0	0
737bc2c98dbfe72256175b328f4e33b5	0	0	0	0	0	0	0	0	0	0	0	14
8c5206ab402795fc4ede214d41a66b80	0	0	0	0	0	0	0	23	0	0	0	0
f2aec4ab8b934b2982c904301c9d50a4	0	0	0	0	0	0	3	0	0	0	0	0
739d80ef8b2967927e28686e6299acec	0	3	0	0	0	0	0	0	0	0	0	0
3f7c075678e61ac4807426a7e3610d26	2	0	0	0	0	0	0	0	0	0	0	0
5740f50c9b48a0783e3631c977fd96f2	0	0	0	47	0	0	0	0	0	0	0	0
51a3cb2b5561b3b9ba6d71e3034e0079	0	54	0	0	0	0	0	0	0	0	0	0
a321f924a37ada617172e02c1496d755	0	0	0	7	0	0	0	0	0	0	0	0
2e1a0752bbab8a34a16589632c52d363	0	0	0	0	0	0	0	0	0	0	0	12
5808debc17580037499c9317ffe41c16	0	0	0	0	0	0	0	0	0	0	0	6

2859190083349e12827898b8f399c064	0	16	0	0	0	0	0	0	0	0	0	0
5509d1a592da4afeb988d79dabd8ad40	0	0	0	0	0	0	0	0	0	3	0	0
875f46a606f5026d2bdea3a20a29f3ea	0	0	0	0	6	0	0	0	0	0	0	0
90342830442926cd2cce757cbe2990c4	0	0	0	0	0	2	0	0	0	0	0	0
7c32d8a19c0248ad6f6b3cd0f12a8792	0	0	0	0	0	0	0	0	0	0	23	0
177e793ca5d4a4d82c84abfdfe9adb61	0	0	0	0	0	0	0	0	0	0	0	34
9d5f353e926ad6a0a081547f9e2fff4b	0	0	0	11	0	0	0	0	0	0	0	0
ae4a89f0c9af0293e7c25eebdd78ff6e	0	0	0	0	0	0	0	16	0	0	0	0
3460dda93ca98f2d9ac3276015c9fbf5	0	0	0	0	0	0	0	0	0	0	9	0
380346a8dae7c2f448ff6a093a3bd4df	0	0	0	0	0	3	0	0	0	0	0	0
25e220ba9c0aa618535310a33555656f	3	0	0	0	0	0	0	0	0	0	0	0
4ebf385eb64bd9891e912530583c263b	0	0	0	0	0	0	0	3	0	0	0	0
e4cf35d4d654b0771860364295432d4d	0	0	0	0	0	7	0	0	0	0	0	0
eaf8d4febde4461e24f38d9f12094c22	0	0	0	0	0	0	0	0	0	2	0	0

a0bef42ad31ab804a6602d9f1f1334fd	0	0	22	0	0	0	0	0	0	0	0	0
36878b05dbec2d77f147a93b66cfc940	0	0	0	0	0	33	0	0	0	0	0	0
9d423b3d608ffe93a1336115706b1325	0	0	0	0	0	0	0	0	0	99	0	0
4c3fbe48e5a255af75b1a87eaa328228	0	0	0	0	0	0	0	6	0	0	0	0
e3cb0fffab38f080a0a66e43b065cc6b	0	0	10	0	0	0	0	0	0	0	0	0
18e25605cd988db00181e9e2998afc9e	0	15	0	0	0	0	0	0	0	0	0	0
9525afbc7f8647dca7b86f01ed5ef601	0	0	0	0	0	0	0	0	0	5	0	0
4b6b71a0ca0cee9f5719831860870dfb	0	0	0	0	2	0	0	0	0	0	0	0
bc6c87942709cf05bd01f0a33aef9bea	0	0	0	0	0	0	0	0	0	0	0	19
1b568208c8fb299bd22f6f98b0447dc1	0	0	0	0	0	0	0	0	0	0	0	5
f06cf6b85a9ccdb7240623107689c3bf	0	0	0	0	0	0	0	0	0	0	3	0
8a6b48e327395693bef41e7ccdcf515a	62	0	0	0	0	0	0	0	0	0	0	0
21dce46ecbdee4c98ce5ba7ea50df0f3	0	0	185	0	0	0	0	0	0	0	0	0
916394acc50a3c66065f1001ae9fe101	0	0	0	0	0	5	0	0	0	0	0	0

d7636bdf20226da06a208a5c8b7db402	0	0	0	0	0	0	0	0	0	0	0	24
3c5933361bfd930800f0c8bd221ed86e	0	0	0	0	0	5	0	0	0	0	0	0
df1c2b0cbfb635d36db86b766b64d675	0	0	0	0	0	0	0	0	0	0	0	3
34985af84706548015b477527e243517	0	0	0	0	0	0	0	0	0	0	3	0
98356dc4157049dce71b159af13e3a41	0	0	0	0	0	3	0	0	0	0	0	0
878852b0a183f954735d635cc9d0fa98	0	0	0	0	0	30	0	0	0	0	0	0
050737450bf9259d13a57d6714fbcba5	0	0	0	0	0	0	0	0	3	0	0	0
782d370a202266958cb4b3f11bffc9f0	0	0	0	0	0	0	0	4	0	0	0	0
f5a8137be6adf844f2a611be265f1d40	0	0	4	0	0	0	0	0	0	0	0	0
52e5fae02839466b4d448a1e87fff7fc	0	0	0	0	0	0	0	2	0	0	0	0
dae79c5bbf436edb3f3d9e813450dac9	0	0	0	0	0	0	0	0	0	0	2	0
080fab4e9764f3f377baf0e102a138dd	0	0	0	0	0	3	0	0	0	0	0	0
8e8601b95582df9225f5d2fb9ec05792	0	0	3	0	0	0	0	0	0	0	0	0
e43903635c83be6edfbd09c757830b47	0	0	0	0	0	0	3	0	0	0	0	0

786d8963a3554f0d6da39000e0ef2303	0	0	0	0	0	0	0	0	8	0	0	0
8a69bf2f630ed169ceb1a0d00ba860ac	0	0	27	0	0	0	0	0	0	0	0	0
9180b2bf338c2cd88d5ffa85e8d610bf	0	0	0	57	0	0	0	0	0	0	0	0
0e8d07aed28649ad37947026547e9ab2	3	0	0	0	0	0	0	0	0	0	0	0
934779b1be9a4c4a296292a417201796	0	0	0	0	0	0	0	0	2	0	0	0
e9988af6bdb08fdcea7b32f3ec0e12d6	0	0	0	0	0	0	3	0	0	0	0	0
3979de1a8fa81c0222d896e8d9562e06	0	0	0	0	3	0	0	0	0	0	0	0
2824de7b87b0940f351d76f137e4a6ca	0	3	0	0	0	0	0	0	0	0	0	0
33c0d71fe24d206f52cf80fdbb24c027	0	0	0	0	0	0	0	0	0	0	0	4
3d1d05e7f70208d731ab3f67279a4a9b	0	0	0	0	0	0	0	0	0	0	0	26
93c8a2a26ba3475af580070c27d5600b	0	0	0	0	0	0	0	0	7	0	0	0
7dd48a05e44b4e25d4887ca3251d8acd	0	0	0	0	0	0	0	0	0	0	32	0
8096bfa87f0d6c38ded0bf55e6420b97	0	0	2	0	0	0	0	0	0	0	0	0
2b2e2ec7e340d3356cc5a7651dae0bec	0	0	0	0	0	9	0	0	0	0	0	0

70d41ec685c85cc2e56338ed6c8fabe9	0	0	0	0	0	0	0	0	0	0	2	0
95bafb7c19b870b26b3e696b968947a8	0	93	0	0	0	0	0	0	0	0	0	152
2fa01bdbb761907c674864c2c8e28f5b	0	0	0	7	0	0	0	0	0	0	0	0
52cf4453f8dcf5ede537d5262fa01992	0	0	0	0	0	13	0	0	0	0	0	0
b012d37da58c98038c96ce4ddabe08a4	0	0	0	0	0	0	0	2	0	0	0	0
d3b0f51778c50bd30407864401b76a1b	3	0	0	0	0	0	0	0	0	0	0	0
1fb4ff83f3f0685b1b4b5020ca114387	0	0	312	0	0	0	0	0	0	0	0	0
6214a3641ec9b74568a8cfb25f8a6843	0	0	11	0	0	0	0	0	0	0	0	0
132f00bb4f7988ad7dda9d98ae627e80	0	0	74	0	0	0	0	0	0	0	0	0
ed57fa230ec417a9dd3294ff563eef77	0	0	0	0	0	0	0	40	0	0	0	0
589f199164e8c66cf32f89f06aa10f9d	0	0	0	0	21	0	0	0	0	0	0	0
99c09ea97a92d1e022a6a05318a0e2fa	0	0	0	0	2	0	0	0	0	0	0	0
ed71499af2d44b546b1f99d471d39bd6	0	0	0	0	0	0	0	0	0	0	29	0
8e05164f45d1cbe7edc9fb74f8d88fb4	0	0	0	0	9	0	0	0	0	0	0	0

fc3d53ce3a43381670fed25f6a37086c	0	0	0	4	0	0	0	0	0	0	0	0
b72c1f3cfa2edfc6c65fc098bcddec3a	0	0	0	0	0	0	0	0	0	0	0	12
4e450a676ff7e89222929c66454b2323	0	0	0	0	0	0	0	0	0	0	12	0
724942fa0de79f1640bda90fe61dc637	0	0	0	0	0	0	0	0	0	2	0	0
5f71b2acbcba8f596c2cf49c1e6045a8	0	0	2	0	0	0	0	0	0	0	0	0
0834a67ebc8bd9e55d6f3e8f64178b4e	0	0	0	0	0	0	0	9	0	0	0	0
5454b45eb05327553a6d2ee3950e8fa6	0	0	0	0	0	3	0	0	0	0	0	0
f277924c2873783e49ed395ba17580d5	0	0	0	0	0	3	0	0	0	0	0	0
45dc9eae2ea460152a0573c9202d9528	0	0	9	0	0	0	0	0	0	0	0	0
765d0f1dc8815252d1f6a356be373187	0	0	0	0	0	0	0	0	2	0	0	0
bdadfb69d68ca3051699c171436d6234	0	0	0	13	0	0	0	0	0	0	0	0
0bf7a7fc0ea0baddca54ae28cedf01f1	0	66	0	0	0	146	0	0	0	0	0	196
578a58cd72e702981b89e977f1b1da14	0	80	0	0	0	0	0	0	0	0	0	0
0a9174eea5076222a139f2893afe57e2	0	0	3	0	0	0	0	0	0	0	0	0

c04bf587db10416f017fe872356165e8	17	0	0	0	0	0	0	0	0	0	0	0
e6f6acaed6e00e0a4f48c718954f88ee	0	0	0	0	0	3	0	0	0	0	0	0
5c0750cae073a161dccf33dd676a1265	0	0	0	0	0	0	0	0	0	0	8	0
18683d5b1757728eed5f2d57d68d8c55	0	0	0	0	193	0	0	0	0	0	0	0
2011a51a00aa0e51c1d07da13c24fb63	0	0	0	0	0	0	0	0	0	0	12	0
78296757001ce70a5c85d77c14b97dbd	0	0	0	0	0	0	0	0	0	3	0	0
08533dfb3d4cc3027a691f4d0d82413d	0	0	0	0	0	0	0	0	0	0	12	0
5d0aa5b68bcebf3b24c1e2d3215f886d	0	0	0	0	0	0	0	0	0	0	22	0
f7b5ce050c0b49cfd4e6152b21a0cf69	0	0	0	0	0	0	4	0	0	0	0	0
e77630fedcdadf2dbf15edb3642cf787	0	0	0	0	0	0	0	0	13	0	0	0
a699ecc8fe5d0f074ed1b08793eebee1	0	19	0	0	0	0	0	0	0	0	0	0
2645fd9164a8471d5cb5042bfc1b427b	0	0	106	0	0	0	0	0	0	0	0	0
fba4672dc710efb3bb4504f8cedd6524	0	0	0	0	0	0	0	0	0	3	0	0
81e9aed1798ae18789d03dd98fed876f	0	0	0	0	0	0	0	2	0	0	0	0

1a245172452d9137c15bc16028f4f784	0	0	0	0	0	0	0	0	0	0	0	6
218fa13e95a30144e891bc1b91c6e05e	0	0	0	0	23	0	0	0	0	0	0	0
f3a8715e3624237015a8358a4dbe2cd8	0	0	0	0	0	0	0	0	32	0	0	0
6f94ddca6572de461c3328d5f2bc9c69	0	0	0	0	0	4	0	0	0	0	0	0
6c9cf6dca55144e5f17a9795eae5c87e	0	0	0	4	0	0	0	0	0	0	0	0
301d9edc42442d6ecb50450bfe38080e	0	10	0	0	0	0	0	0	0	0	0	0
fa571bc3446d3087617eb58a3b4d7d10	0	0	0	0	0	0	0	3	0	0	0	0
6ce59f8778b97a4c5d47003400712c9e	0	0	0	0	0	0	0	0	0	0	2	0
bad0d5bebc57da6311e86d32bd5958e8	0	0	0	0	0	0	0	3	0	0	0	0
b8b2c0d3ad7925dc046f894bb6a25308	0	0	0	0	0	0	0	0	0	0	49	0
b81b61038896e2abd6952fda0485d9ab	0	0	0	0	0	0	0	0	0	0	5	0
6d0c7ee9c60474a706557f8e329ac53f	0	0	0	0	0	0	0	0	0	0	5	0
9a2ca0993d5c348008e7a0fd1b7befc7	0	0	0	0	0	0	0	0	0	2	0	0
1312dd4c6fca6eb74954fe8451feb395	2	0	0	0	0	0	0	0	0	0	0	0

0ee8b8ee9a5da9aee38b8f16ca04506f	0	0	0	0	0	0	0	0	0	0	2	0
85907e1664e4d3e6ee931f264a95b973	4	0	0	0	0	0	0	0	0	0	0	0
13ae95f2c6157153db8d43070f232cbc	0	0	3	0	0	0	0	0	0	0	0	0
5f78bfa6f9a9107c3d743c21317833ed	0	0	0	0	0	0	2	0	0	0	0	0
fba34b2c5149daf424bcf6bc4b50112e	0	0	0	0	0	0	0	0	0	0	0	2
ed105fa8fa53b9c4137f78ca8ab66c8d	0	0	0	0	0	0	0	4	0	0	0	0
1c4e1a4d240bb159ebbbbc857e1f2772	0	0	0	6	0	0	0	0	0	0	0	0
261122198a36fc17747043babd4a9a3a	0	0	5	0	0	0	0	0	0	0	0	0
df865c7d5c9182766f375a2d437529a6	0	0	5	0	0	0	0	0	0	0	0	0
bbb469b4990c24b999d90ead9e66d117	0	0	0	0	0	24	0	0	0	0	0	0
9f130535abc7a3899178960653419886	0	0	0	14	0	0	0	0	0	0	0	0
869a9edb6855d86c83e303a2fbde5d8a	0	38	0	0	0	0	0	0	0	0	0	41
d82a28fca6c8bb22734af7f3ae4c7181	12	0	0	0	0	0	0	0	0	0	0	0
1c8a585b70047901ff5f90e99cd12138	0	0	0	0	0	32	0	0	0	0	0	0

12c517a1449fb362a0b1bdc0425e60ca	0	0	0	0	0	0	0	0	65	0	0	0
df71298563193cd6947fa75cae91ed73	0	0	0	0	0	0	0	17	0	0	0	0
5f436e41325adcfddf47238adac4da0e	0	0	0	0	0	0	0	0	0	0	50	0
a21299a75b3f7025d23e54ef440fd3a0	42	0	0	0	0	0	0	0	0	0	0	0
003872e7c71b8d98cca452e4d046ce41	0	0	0	0	0	0	0	0	0	0	0	5
e3d3144d301283d06300e275e5a7febd	0	0	0	0	3	0	0	0	0	0	0	0
d3caa5cbd0d3cee17a3d4f25495d6b23	0	0	0	3	0	0	0	0	0	0	0	0
21050bd65feda9ba6af93dd8b748749e	0	0	0	10	0	0	0	0	0	0	0	0
a8c419f4421e5988a537d2a5190cb199	0	0	0	0	0	0	0	0	0	2	0	0
24e0ff9830f2fafb2db491f9778fe047	0	5	0	0	0	0	0	0	0	0	0	0
e9881998fdc09d4b58399c493f93a11e	13	0	0	0	0	0	0	0	0	0	0	0
d982b0f389b1d8a9d65ca270524e4714	0	0	0	0	0	30	0	0	0	0	0	0
b9fd3f70b141df12ada2520bb60cfa5d	0	0	0	0	9	0	0	0	0	0	0	0
10f37b697805be1e4a71d990e87cb74c	0	0	0	0	0	0	4	0	0	0	0	0

42275b44af6c6641a2b9f3fe912fabb7	0	0	4	0	0	0	0	0	0	0	0	0
09c0367dc2b158b802d02ca7487a5a24	0	0	0	0	0	0	0	0	0	0	11	0
ed0e760ec01275d5a8b34edab8559f97	0	0	0	0	0	0	0	3	0	0	0	0
d5c2cdff5b160bca90b17a93c173617b	0	0	0	7	0	0	0	0	0	0	0	0
59f020442acf5dc6385922f767ae7f5a	0	0	0	0	0	0	0	0	0	0	0	8
42ef3bba6faa53512cc7a8455a24cc4d	0	0	0	0	0	0	0	0	3	0	0	0
3cc70ab959dad05e610f35c5a061452b	0	0	0	3	0	0	0	0	0	0	0	0
03772a8a40e590653383f86991243c5b	0	0	0	0	0	0	0	0	0	0	3	0
95a53abc8cfb2c4cb7a1ebe32751763d	0	0	0	0	0	0	0	0	0	0	13	0
10ab99c04236d12b84fe6d74c4123f90	0	0	0	0	0	0	17	0	0	0	0	0
37ce4bf252bbf5ef0e02889566706146	0	0	0	0	0	0	0	2	0	0	0	0
183628113ef91229e67600568355496c	0	0	2	0	0	0	0	0	0	0	0	0
f433063a0637b3499d4824f78f7b5f5f	0	0	0	0	2	0	0	0	0	0	0	0
bf4f650f32366c272afe02c7a737adca	0	0	0	4	0	0	0	0	0	0	0	0

ca2fb8908ec0ae9efe274dda2a302bea	2	0	0	0	0	0	0	0	0	0	0	0
b45723cddb0189a271638d02a0bd6ef8	0	0	0	0	0	0	0	0	0	0	0	10
5b1d78fff813fe49723a6ddad98277fe	0	0	0	0	0	0	0	0	0	5	0	0
5e37ef6028e29685ffda0a2a8e8137bd	0	0	0	0	0	0	0	0	4	0	0	0
6edbcfebe33f23d3b55485abae80793a	0	0	0	8	0	0	0	0	0	0	0	0
15bc2e7ba4ae73f6b6b3961364ff7fa4	0	0	0	0	0	0	0	0	0	0	2	0
96722f390f59e5bbaddc27db787aedec	0	0	0	0	0	0	0	0	0	0	4	0
4759b26f12e70afb296714f550a46eeb	0	0	0	0	0	0	0	2	0	0	0	0
deda269b7fa8ab60890527628a521a3f	0	0	0	0	0	0	0	0	0	15	0	0
e000ca01c5dbdcde573c23666fbd2f89	0	0	0	0	0	0	0	0	0	25	0	0
a82c0f34524840a0e6ef2894fa46776d	0	0	3	0	0	0	0	0	0	0	0	0
67ec6f345bbfeec86762a0d329000fec	0	0	0	2	0	0	0	0	0	0	0	0
e4b97d015d16bb1a82bff83832384c48	0	0	0	0	0	3	0	0	0	0	0	0
7113af1768d3e2637fcd0db5d7a6a77a	0	0	0	0	0	0	0	0	0	6	0	0

5eb61cb96492eb62fb5e6a472c9c2cef	0	0	0	0	0	37	0	0	0	0	0	0
6e5254a1480ac503b3664da2190ec46b	0	0	0	0	0	3	0	0	0	0	0	0
44502b73ffe5a4016d234903ec5f7997	0	3	0	0	0	0	0	0	0	0	0	0
8288cb9a716c8e637bf9037012b0ec71	0	0	0	4	0	0	0	0	0	0	0	0
2382d3c34bcd7d6f24e8f2dfa84b230c	0	0	0	0	0	0	0	0	0	0	0	191
03c9db22ecab3ff5da6e0bd47277a547	0	0	0	0	0	0	0	0	15	0	0	0
90cc9c403be96499ece420473aadcec9	0	0	0	0	0	2	0	0	0	0	0	0
a0db97849aa6fa5acb0592217d3331d7	0	0	0	0	0	0	0	0	0	4	0	0
ce8ff9cea54c792877446b8e0855fe75	0	0	0	0	0	0	0	0	0	76	0	0
efd9cbeb4a441232a9ce081a7fe0a242	0	0	0	0	0	5	0	0	0	0	0	0
f8524827bf479fbcebb925215555c615	0	0	0	0	0	0	0	0	0	0	2	0
8ea92a9645e9657ecc10bd1f5c3a6481	0	0	0	0	0	0	0	12	0	0	0	0
7f1b17145b731475da5744ef513afcc8	0	0	0	0	15	0	0	0	0	0	0	0
ea0c1f0b9142c84d9856e804a1d57dd7	0	0	0	0	0	0	0	0	3	0	0	0

8c7884ae19e97808e65ffa136e31dbe0	0	0	0	0	0	0	7	0	0	0	0	0
a1cbc16e27799ccc3e1bdccae0b65542	0	0	0	8	0	0	0	0	0	0	0	0
dc43aaf7c60c5b802fb14c42ac73e2a1	0	0	0	12	0	0	0	0	0	0	0	0
24157727d9527ef29fb2e2113e1b7b5a	0	0	0	0	5	0	0	0	0	0	0	0
f8bf3f08edc281bfb50e9894d91d4581	0	0	29	0	0	0	0	0	0	0	0	0
60def4814f061cf6381669e53b244463	0	0	0	0	0	0	0	0	5	0	0	0
5c69c458729b3ec74b71ac567c25eed4	0	0	0	0	0	0	0	0	0	0	0	2
f7e2813f8f0b1933fc1c0abb5a7e601c	0	0	0	0	0	0	0	0	0	0	0	28
6495b1bbe42192cb6b81b2e76b07e1ec	0	0	0	0	0	0	0	0	7	0	0	0
4996512723a61208822ba85084a156bf	0	0	0	3	0	0	0	0	0	0	0	0
8fa2ee20cf78b601bef1d036349dcd18	0	0	0	0	0	0	0	0	0	0	49	0
416aaeba13c00df5905eefc1af139992	0	0	0	0	2	0	0	0	0	0	0	0
70ca4692d8f1c69ebb3dc9db9f2dc232	0	0	0	0	0	0	0	14	0	0	0	0
697a8331549a2042765f74ffd442df41	0	0	0	0	7	0	0	0	0	0	0	0

77130b4dbcdaf29f291de5d62b375769	0	0	0	0	0	0	9	0	0	0	0	0
9997785d06aff0751e2d7bd3d54b931e	5	0	0	0	0	0	0	0	0	0	0	0
3aee5a7d62b6c8e61a9385023ffe3327	0	0	0	5	0	0	0	0	0	0	0	0
647e578d62ff7360bbb5e7e8b3429769	0	0	0	0	0	0	0	0	2	0	0	0
209f895d0c541e482e5f365a23ab6425	0	0	0	0	0	0	0	0	3	0	0	0
da4d7b336f7c38cbf8bda0b0638c0112	0	0	0	0	0	0	0	0	0	0	0	22
aa3801f85cd26834de6e5e26d85feabd	0	11	0	0	0	0	0	0	0	0	0	0
702c7ed6c46df80ed9fae23ae8d793ac	0	0	0	0	5	0	0	0	0	0	0	0
53ea9f9058206b91d80f0fd6fbc09f9a	0	0	0	0	0	0	0	0	0	37	0	0
45588a7d4570f2684c28b4c9b9a03dca	0	0	0	0	4	0	0	0	0	0	0	0
befb14f39a6024a88430b709f5951f06	0	0	0	0	0	6	0	0	0	0	0	0
166b3e851905cf7d4e22049854f4a0fa	0	0	20	0	0	0	0	0	0	0	0	0
b9dc8fa07465bdd37d9224d6073ccf93	0	0	0	0	4	0	0	0	0	0	0	0
77d3ee707592435ff2522d73047facf3	5	0	0	0	0	0	0	0	0	0	0	0

761c219c177fb40224743ce468d01332	0	0	0	10	0	0	0	0	0	0	0	0
09579f08feb3d5cbe77afbf4458981b6	0	0	0	0	0	0	0	27	0	0	0	0
d2a5467a362c836e8b09b23369261363	0	0	0	0	0	0	0	0	0	5	0	0
1d73aac9026eee507360f262eeece88b	0	0	0	0	0	0	0	99	0	0	0	0
787fd3cbd5404e8812c3ec6b6d8857bf	0	0	0	0	0	0	0	0	0	0	3	0
f74ac4446e99120e8e61dc574778d379	0	0	5	0	0	0	0	0	0	0	0	0
6eb644a6aa401a22d7cd9673265bbb4d	0	0	0	84	0	0	0	0	0	0	0	0
893d76fa83b81e6bd6485cc4f1bb4e8b	0	0	0	4	0	0	0	0	0	0	0	0
783d2f7d7268764d153ba61b8ac1dc4a	0	0	0	0	0	0	0	0	0	0	10	0
1b2d9b97722e926369a2a54df27941d7	0	0	0	0	0	0	6	0	0	0	0	0
d9cdfe314e2d93b940ded93833e760b8	0	0	0	0	0	0	0	0	0	0	0	12
857a3f4c2c1a4fb3191132c35ea7356a	19	0	0	0	0	0	0	0	0	0	0	0
9c4d10055ce80e2867b028f434c72f87	0	0	0	0	0	0	0	0	103	0	0	0
11f913952a41445bdcebf14e6bd99f42	0	0	0	0	0	0	0	0	0	0	4	0

3b078ee3c80148c8528ad0ca db840e8d	0	0	0	0	0	0	0	0	0	0	11	0
716fce714f3376e0c5eb7218 b13de6e4	0	0	0	0	3	0	0	0	0	0	0	0
28ff1167d0415c76dfb49b0fa 67cea45	0	0	0	4	0	0	0	0	0	0	0	0
17668d89d3579bf615aa239b d63a6ce2	0	0	0	0	6	0	0	0	0	0	0	0
cd35b9c6a36cae31dcddcbe0 1402121f	0	0	0	0	0	76	0	0	0	0	0	0
7926e6d7c778537f778ab154 65cf7853	0	0	0	0	0	0	0	2	0	0	0	0
c09007b7c3a467aa6a25284c 2d1fb4be	0	0	0	0	0	0	0	80	0	0	0	0
a557c15b59f7022fc2893ff0e 51c3fe3	0	0	0	0	0	0	0	0	0	0	20	0
d6d67fb9c273087b40bc2d4b f9e5d4ff	0	0	0	0	0	0	2	0	0	0	0	0
d77d2b432fd4e6549ed6674 66e1617cf	0	0	0	0	0	0	0	0	3	0	0	0
0535a91dc7bbf6319bc595a2 39f4fed3	0	0	0	0	0	0	0	0	0	0	2	0
656400a7af695f0f8767df593 d5e3334	0	0	0	0	0	0	0	0	0	0	2	0
6e12c22338b1cb19def658f3 ab3ef48f	0	0	0	0	8	0	0	0	0	0	0	0
b11ace8dad90f6101aaacfe12 33c4f91	14	0	0	0	0	0	0	0	0	0	0	0

8ab5a3271222e8c73ab99c4037e0b1e5	0	0	0	0	0	0	0	0	0	0	2	0
8c2babe4f75120967bdf3d849f6928b8	0	0	0	0	0	0	0	0	0	0	11	0
08e53ea0d80672fe435c515a430e47e6	0	0	0	0	0	0	0	0	0	0	0	10
7ce2e91d608526acc33db59d59ef7fff	0	0	0	0	0	0	0	0	0	0	0	2
13e0878c8d364bd7cd0d208b0ec05d15	0	0	0	0	0	0	0	0	0	0	0	3
59f9a4fab898e2e1c927ca55031f6f21	0	0	0	0	0	0	0	0	0	0	35	0
01087a5616497001da05a0f2153a2615	0	0	0	0	0	0	0	0	0	0	2	0
3e7def6cefe2763d28ca6756e039db1b	0	0	0	0	0	0	0	0	0	0	0	2
0856ad68249e7e7d30f234a919bf5603	0	0	0	0	0	0	0	0	0	0	0	3
6f36f303eee4bddbca3f9aaec6d5b86e	0	0	0	0	0	0	0	0	0	0	0	7
cf2c891a691117dca1f73394cdd4fec7	0	0	0	0	0	0	0	0	4	0	0	0
79f5f6b4f2011b4db24b44820be8cf30	0	2	0	0	0	0	0	0	0	0	0	0
6b8f1d1642aaed657d9095aea69111d0	0	0	0	0	0	3	0	0	0	0	0	0
ce787d3329a342b1376cb3e364824e24	0	0	0	0	0	0	0	0	0	0	4	0

7e4332a57c0674379d69db9ff7b413f0	0	0	0	0	0	0	0	0	0	9	0	0
00201fe3420fa122ad3b1c6c2da5a515	0	0	0	0	0	0	0	0	0	0	2	0
776a03ce9add3288544648a6cd4aa854	0	0	0	0	0	201	100	0	0	0	0	0
bc37d9ebc2872a9f06cf834008b26b0f	0	0	0	0	0	0	0	0	0	0	2	0
6f1516902d5f0d250ad051c18e0c09e9	0	0	0	0	0	0	0	0	0	14	0	0
01db5f64e4062d5e51c0b442ce472ea2	0	0	0	0	0	0	0	0	0	0	19	0
658ec6e0657f27527e886aed97cd9079	13	0	0	0	0	0	0	0	0	0	0	0
9a2a76c7a5a492ffc76c582be99563d8	0	0	4	0	0	0	0	0	0	0	0	0
d0bfa291d9a199392a6b20e5e3963ba1	0	0	0	0	0	0	0	0	0	7	0	0
164db8fa890482aa1c720c904c808a08	0	0	2	0	0	0	0	0	0	0	0	0
1b3b499848c51ff508e049cedaec3638	0	0	0	0	0	6	0	0	0	0	0	0
9e59c394c03ea746cea75337ccf3483f	0	0	0	0	0	0	0	0	0	0	4	0
ffd192b4bab604a1ec2ca673d953c6cb	0	0	0	0	0	0	0	0	0	0	140	113
af54b0b0796c720f26e3afe1fd676bc7	21	0	0	0	0	0	0	0	0	0	0	0

dd04004483701796cf35362abe6db23f	0	0	0	0	0	0	0	0	0	0	17	0
28a2c08fc2bf66b9d43ae7f644ca69df	0	0	0	0	0	0	0	0	0	0	23	0
03262dc58329c2d994ac81beefef6f20	0	0	0	0	0	0	0	0	0	0	2	0
8f39e6949177a8fff497e040da04d74d	0	0	0	0	0	0	0	11	0	0	0	0
4bf740504ad0b7efb23e5a6be34e10f5	0	0	0	0	0	0	0	0	0	0	0	2
6102cc431efd9a5056aeeaa4032e7e42	0	0	0	0	0	0	0	0	0	0	0	4
135906c329bcee9f29ea649fb7ffb2ec	0	0	0	5	0	0	0	0	0	0	0	0
54b3fc77667b40d88e4efa4f5a4daa96	0	0	0	0	0	6	0	0	0	0	0	0
d2c7ec763ff764dd4760b4d24ca9404e	0	0	0	0	0	0	0	8	0	0	0	0
92032e6d92c2fd705a8f4a0f794da8a0	0	0	0	0	0	0	0	0	0	0	4	0
720690ebb04d30c7d10d2f100064c960	0	0	0	11	0	0	0	0	0	0	0	0
70aadf34c0f9ead1739076bd2cec63a7	0	0	0	16	0	0	0	0	0	0	0	0
e2b2ebcbd7e1cf88bd00baccf4b1d188	0	0	0	0	16	0	0	0	0	0	0	0
293d9c6e80225e4897ab9be7c3ed1e65	0	0	0	0	0	0	0	0	4	0	0	0

9f3bf6ae118124f9f397b37dd19199d0	10	0	0	0	0	0	0	0	0	0	0	0
ab82e6145a3c4fab8bb45e97822cf6a8	2	0	4	0	0	0	0	0	0	0	0	0
3bc072f910d6e898966fc5408ed43c6a	0	0	0	0	0	0	0	0	0	0	0	19
493312bcc092b658cd45fe77f5ea81ff	0	0	0	0	0	0	0	0	0	0	4	0
6ae73f0d6f00da3c60ee999f2f87328c	0	0	0	0	0	8	0	0	0	0	0	0
85e660bc25f0121158d935bc73af42d1	0	0	0	0	0	0	0	0	0	0	2	0
fe42a515600953e1bc6acf71cf648616	0	0	0	0	0	0	0	0	0	0	11	0
0ca325b224fa8e0d71d178abd90acb6f	0	0	0	0	0	0	0	0	2	0	0	0
b122b5b1ef1e50644982653304ae2e95	0	0	7	0	0	0	0	0	0	0	0	0
b2fe95542d77b38e65f9538273c154af	0	18	0	0	0	0	0	0	0	0	0	0
93811e6d61b005bea00eec16ccd6bd83	0	0	0	0	0	0	2	0	0	0	0	0
5afa72e765fa100525d0128d125ad006	0	0	0	0	15	0	0	0	0	0	0	0
a8b977cb0b48331a13c42bc21f43084a	0	0	0	13	0	0	0	0	0	0	0	0
c6dab5e5d44c050d16830db4e920f92c	6	0	0	0	0	0	0	0	0	0	0	0

00b061c004d572cf68dfc41ca2fb2f35	0	0	0	0	0	0	0	0	2	0	0	0
bdc369bc8e5ca3bfcb36374540a711aa	0	0	0	0	0	0	0	11	0	0	0	0
243e807a8b0080c0181fb897528f04a5	0	0	0	0	0	0	0	0	0	4	0	0
95d6cde413f92939441f669ff28fa2f1	0	0	0	15	0	0	0	0	0	0	0	0
cdfedede6a12509ce1f189e7191af604	0	0	0	0	0	0	11	0	0	0	0	0
b61379b3268d3a6b13ba93a7d85cb3ba	0	0	0	0	45	0	0	0	0	0	0	0
9357013ce45da2c49b0b9560680877e7	0	0	0	0	0	43	0	0	0	0	0	0
5981768e9377683f0fd54140e757f78a	0	0	0	0	0	0	2	0	0	0	0	0
05be826e0c9ecc330f62a582cf89011a	0	0	0	0	0	0	0	0	0	0	0	13
62a3fb04874f2eaf97765f46c8076385	0	0	0	0	0	0	0	4	0	0	0	0
8d881384b85d23cf6aa70777a759da2d	0	0	2	0	0	0	0	0	0	0	0	0
c56516e3d0100e9cc8d6b0c2296570b6	0	0	0	0	5	0	0	0	0	0	0	0
a86716a4a797e84ebd1044fec456812d	0	0	0	0	0	0	0	0	0	0	0	5
ad23f3d0b23c06b6aa4f4abc04dc132a	0	0	30	0	0	0	0	0	0	0	0	0

1dd73faf610a66bb31a60f1e25dbec06	0	0	0	0	0	0	0	16	0	0	0	0
67229b58c538f995cb8949e03e7cc2fc	0	0	0	0	0	0	0	10	0	0	0	0
c548391f6ed297fc8c1e980eac020b43	0	0	0	0	0	0	0	0	93	0	0	0
ef441bd8913e8c3c11fbc1e9565935a0	0	0	0	0	0	0	0	63	0	0	0	0
0b7411a20f9f56f4f77d8a25261d910f	0	0	0	0	0	0	0	9	0	0	0	0
900360042976e492ea7976bcaf1a9951	0	0	3	0	0	0	0	0	0	0	0	0
27be135fcc71ee157d952af46af7d26c	0	0	0	0	0	0	0	0	0	0	10	0
946b18417e2d49ecde5911ea7be5f002	0	0	0	0	5	0	0	0	0	0	0	0
52759e7f733d8fec8b8debea39a1f1f3	0	0	0	0	0	6	0	0	0	0	0	0
c5b664af1ce402c8e7d0704ae32cadb2	0	0	0	0	0	0	0	0	0	0	0	5
c208fd42be550af2273681d6fd538c9c	0	0	0	0	0	0	29	0	0	0	0	0
fbc6b678ff6b1bfa6461c2a85a303852	0	0	0	0	0	0	0	0	0	0	0	4
fe7f4b9ab4e171d15dd0116ef2c12f2b	0	0	0	0	0	0	0	0	0	0	3	0
acb397564354d9a51afea545a7c7c488	0	0	0	0	0	0	6	0	0	0	0	0

bf81d6d5997fe62536985d7352b35186	0	51	0	0	0	0	0	0	0	0	0	0
f1b2d80638561ed9e94666d5d38db974	0	0	0	0	0	0	0	0	3	0	0	0
d07c88ba73ab7658efaf77dfa2c97af2	0	0	0	0	0	0	19	0	0	0	0	0
0bff5d88fbdff9b00566c6f5e4efc582	0	0	0	0	0	28	0	0	0	0	0	0
39b52a35ba9ae13c3a97db14a18737ef	0	0	41	0	0	0	0	0	0	0	0	0
664704d91e5b59f9f80c2a87c6c16940	0	0	70	0	0	0	0	0	0	0	0	0
dcc2a282a11aec168deddfdbb69113d2	0	0	0	0	0	0	0	0	0	0	2	0
0665d7991586ff1e56ffed86bac5eefb	0	0	0	0	0	0	0	0	0	14	0	0
3c1af1c19d8ccaeb9731dd44226570d4	0	0	0	0	0	0	0	0	0	2	0	0
5afc7e95686e12c3a8a968b68808cce8	0	0	0	0	0	0	0	0	0	4	0	0
143f5095e00a243e93e1074fc1a44d7d	0	0	0	0	0	0	0	0	0	0	7	0
431ee1224707ae6c19479dbabd0cdaee	0	0	0	0	0	0	0	0	0	0	6	0
3672e06b9dcc2fb2a26dcc6f31022064	0	3	0	0	0	0	0	0	0	0	0	0
e44415b3dd30a7d68c2907bf2a5f205c	0	0	0	0	0	0	0	0	4	0	0	0

cb7b3a5a98ba215acf5070f1b3ad48d6	0	0	0	0	0	0	0	0	0	12	0	0
909beba4915499e0b886b9ff9a712ccd	0	0	0	9	0	0	0	0	0	0	0	0
445862b2a692ad5429f809043eca4c05	0	0	0	0	0	0	0	27	0	0	0	0
88bcf8a689e2d99d485f0f1ea5bc223e	0	0	0	13	0	0	0	0	0	0	0	0
e4fd2e2ba93d25ca373ad716f9e6be26	0	0	0	3	0	0	0	0	0	0	0	0
06cbfa46033998b29a5cd6b6dd35f8ba	0	0	0	0	13	0	0	0	0	0	0	0
2ce33aa2cdba77d0b1b63218d8b5cd8b	0	0	0	0	0	0	0	0	0	0	9	0
32e304dca3f6d5f70232aec1da4c01d0	0	0	0	0	0	5	0	0	0	0	0	0
0a803900a5f44f11ff6b9fc63165d6ba	0	0	0	0	0	0	0	0	0	0	58	0
50dbf649ee945bca31486e0e3d9c1191	31	0	0	0	0	0	0	0	0	0	0	0
d30958d8fdbd6da8f119d8e754aafc6d	0	0	0	0	0	0	0	0	0	0	9	0
50fbd9e21383f285f68a1c8fde2fc2e4	0	0	0	0	0	0	0	0	0	0	0	55
50b7ef030ee48a3017ffa80f5de14d85	0	0	0	0	0	0	2	0	0	0	0	0
4c826fc27ca9653c8ae096a7489e3402	0	0	0	0	0	0	0	7	0	0	0	0

2b43b5cf6cf616426c09908a b745c49e	0	0	0	0	57	0	0	0	0	0	0	0
c7a5ec057148d59fd2d28d0b 1dfb6995	0	0	0	0	0	2	0	0	0	0	0	0
f4a306ef2f6cf4693d5779294 f3919c4	0	0	0	36	0	0	0	0	0	0	0	0
df523fad28563e61ce8d11fec cb800e7	9	0	0	0	0	0	0	0	0	0	0	0
e084a065dd2635cbddaad40 345c5ed96	0	0	0	0	0	0	197	0	166	0	0	0
2e220d71cb3f241398d4b371 5388405d	0	0	0	0	0	0	0	3	0	0	0	0
0d28551134d27b148ba1069 c5fc965d2	0	0	0	0	0	0	0	0	0	0	0	4
8464e93e5e36963e98ee61e a006852de	0	0	0	0	0	0	0	0	11	0	0	0
9d213283b1dea01f44f8179e 6b46461b	0	0	0	0	0	0	0	0	0	0	2	0
3ea9133f9993a41eee534693 954e657b	0	0	0	0	0	0	0	0	0	0	0	3
4b8dce1a397ba8b1a33c9423 e66b7eae	2	0	0	0	0	0	0	0	0	0	0	0
ea51ee44958f930b02cdfbea edc08d04	0	0	0	0	0	0	0	0	0	3	0	0
fe4897c93afeeeb432051506 9405852d	0	0	0	0	0	0	0	0	0	0	24	0
0143f2a48ffa4de8ea862bf64 94407cb	3	0	0	0	0	0	0	0	0	0	0	0

20d3ccb5e97d62ce2f3b48b986b7603a	4	0	0	0	0	0	0	0	0	0	0	0
2ac765eac19385a6730a58591120dc5e	0	0	0	0	0	0	0	3	0	0	0	0
2aebf730fb4fb89d5afc54a2ebf5038b	0	0	0	0	29	0	0	0	0	0	0	0
c5e5e09be1136a2925d2d8395b1e595c	0	0	46	0	0	0	0	0	0	0	0	0
6277fd222989523a9382dad6594e8cdb	0	0	0	0	0	0	0	0	0	0	0	12
5d3e31b24012e3c0cf1628b7d9d2a010	0	0	0	0	0	0	0	0	2	0	0	0
b2f30fbff02d6308192a114294a12b35	0	0	0	0	0	0	2	0	0	0	0	0
3c99fcfc06e2d31d84d08037104fca08	0	0	0	0	0	0	0	6	0	0	0	0
81f34437f070eaadd569bea179be0e49	0	0	0	0	0	0	0	0	0	0	3	0
5376d8c54b6437686c42853cff3f1f61	0	0	0	0	0	0	0	9	0	0	0	0
8f60eaec1df06fcf86c6ecdeae2aae97	0	0	0	0	0	0	0	0	0	0	3	0
88e43e313b2418b4121499a472578e4a	0	0	2	0	0	0	0	0	0	0	0	0
d9f8bad8b2e17a019320125641dca909	0	0	0	0	0	0	0	0	0	11	0	0
88fc8a6711bcc5dda84085c193a9abe9	0	0	0	0	0	0	2	0	0	0	0	0

35a7fbcb33d7c959d6d5937f2d446da5	0	0	0	0	0	8	0	0	0	0	0	0
cc7d43cda6dfa81c5d133cba43948487	0	0	0	0	0	43	0	0	0	0	0	0
3214e171aab8842dc527a82201931640	0	0	0	27	0	0	0	0	0	0	0	0
4e7bcf8ab038496f88f6c7016e6885a7	0	0	0	0	0	0	0	4	0	0	0	0
c10f48a82d8daf135953d524743d43dd	0	0	0	0	0	0	0	0	0	18	0	0
d18951cea07ebac5957a99495872737c	0	0	0	0	0	0	4	0	0	0	0	0
bd303fb62bfdb575f06a400cf8129679	0	0	0	0	0	0	14	0	0	0	0	0
be3aec07f3b02c1eb30525d12689a191	0	0	0	0	0	0	0	0	0	0	0	2
437625056d21c01be0d630db4601593d	0	0	0	0	0	0	0	0	0	0	0	4
20781d01a94849414dcc350d22f36a66	0	0	0	0	0	0	0	19	0	0	0	0
9dbd44aaa09ec4b2c9765704d84c4c70	0	0	0	0	0	0	0	0	0	0	0	3
f5d7c8b96bb6e03b3016b9a1237d2707	0	2	0	0	0	0	0	0	0	0	0	0
6e911f4df235e16325dce4c1588de195	0	0	0	0	0	5	0	0	0	0	0	0
f72c5397b929d4380af2e8997d6f8d8d	0	0	0	0	6	0	0	0	0	0	0	0

e4c555f3056bec75dbd5adb62499372c	0	3	0	0	0	0	0	0	0	0	0	0
210411529823d397d4c789bb0221cd1e	0	0	0	0	0	12	0	0	0	0	0	0
eab1fc2acef8230592ff6d6abdf40e4f	0	0	0	0	0	0	6	0	0	0	0	0
3e77164447ce02884b4d6ebb5b5d484c	0	0	8	0	0	0	0	0	0	0	0	0
f11ee4f8d6e0900b3fa01f3807408b14	0	0	0	0	0	3	0	0	0	0	0	0
9e78866f64b9598cc7f2605962aab67c	0	0	0	0	0	0	0	0	0	0	4	0
a56aa7e418a59f1c71ec9b26d43aa526	0	0	0	0	0	0	4	0	0	0	0	0
ba364c169423bb26744c3e0559c3de10	0	0	0	0	0	0	0	0	0	0	9	0
0e73a9793591289e71409ada78f159bf	0	0	0	0	0	0	0	0	0	0	2	0
b7ea9d9e75548317aa25ed1020c291fc	0	0	0	0	0	0	0	0	0	0	0	39
dea7aa216284a644896a412a40509589	0	0	0	0	0	12	0	0	0	0	0	0
4166d34aad101a3966020519f599cd21	0	0	0	0	0	0	0	0	3	0	0	0
6f7491f1827a527c47c9f0d981557772	0	0	0	0	0	0	0	0	2	0	0	0
8ab394e1cf48b8c737ab29049bb518fc	2	0	0	0	0	0	0	0	0	0	0	0

9eb157fb5dcb2aec0ed8bdc61034abf2	0	0	0	0	0	0	0	0	0	0	0	15
b4523ac6fc18c50268187178d5cb06a0	0	0	0	0	2	0	0	0	0	0	0	0
7e2976bead363e842a14bd95a591a452	0	0	0	0	0	0	0	2	0	0	0	0
7922dc65b56a91f98cf7a561023f0797	0	0	0	0	0	7	0	0	0	0	0	0
049ce8baabb009ad0cfb99a7a0920326	6	0	0	0	0	0	0	0	0	0	0	0
e27b06103718b91608ac4d02469cdfed	0	0	0	2	0	0	0	0	0	0	0	0
55284d6f415010a2d7f8295cc5ca993f	0	0	0	2	0	0	0	0	0	0	0	0
016ac9258465a7c5d215cd268329626b	0	0	0	0	0	0	0	0	0	0	0	7
feedb2639dd9a18c0eaf63bdd541bf79	0	0	0	0	0	0	0	0	0	0	14	0
923e334222ff82051414ec15920f04c6	0	0	0	4	0	0	0	0	0	0	0	0
4558ea351257342e84bce8be5557f5d0	0	0	0	0	0	0	0	0	0	0	0	2
6749fc47fd5ea9e41bcf60ec4b716c60	23	0	0	0	0	0	0	0	0	0	0	0
a30516ea190258ff1f6cb409785b46fc	0	0	0	0	0	0	0	0	0	0	0	3
83f7de10bc7cbb62eb29be3e9265c5c0	0	0	0	0	0	0	0	0	0	6	0	0

71cf883f2d113908f9309bccd da095f2	0	0	0	0	0	0	0	4	0	0	0	0
269e576af6162c3e9bfc5426 d3a67906	0	0	0	0	0	0	0	0	0	0	4	0
41b264f11ed88fef87f9e32b1 92c7df9	0	0	26	0	0	0	0	0	0	0	0	0
2bc445dde51844180fc4364a 7248383a	0	0	0	0	0	0	0	0	0	22	0	0
c7c3d3f5c15ac9c0f3d5aee22 aa26793	0	0	0	0	0	0	0	0	0	0	0	4
46ba237cee12d28b04ce17f6 cb44d668	0	5	0	0	0	0	0	0	0	0	0	0
0ba3218fc4355bac5f2bf361d 0854ccc	0	0	0	0	0	0	0	0	0	0	5	0
893fdc685879a0f21c66c7f62 997cf98	0	0	0	0	0	0	2	0	0	0	0	0
884263253d512e61a362606 d99308cda	0	0	3	0	0	0	0	0	0	0	0	0
fba29cce7d0fbffcdb8988828 70e4563	0	0	3	0	0	0	0	0	0	0	0	0
67b81f7ba867bc55d2243e95 351fa4c6	0	0	0	0	0	0	0	0	0	3	0	0
17972a11aef7123ececd7340 99973b58	0	0	0	0	0	0	0	0	0	0	0	16
113275b3987419ee63b271a 2c14f88e3	0	0	0	0	0	0	0	0	0	0	0	35
af259b8ab60f875fbe3f08ebc e040055	0	0	32	0	0	0	0	0	0	0	0	0

eb91c3fe517a8ba55acce8dd63595a47	0	0	0	0	0	15	0	0	0	0	0	0
8d872e76e24fe3101cbbab9807131b4c	0	0	0	0	2	0	0	0	0	0	0	0
1249f95f8671319e7763fab65795d63f	0	0	0	0	0	0	0	0	0	6	0	0
b0977d25b49094fc7a922395db88706e	0	0	0	0	0	0	0	0	0	0	0	28
5b987f7ce873897650eb92a1bfe56b04	0	0	0	0	0	0	0	12	0	0	0	0
93dc18f4a8b7f834c1812f3edca2932d	0	0	0	0	0	0	0	10	0	0	0	0
72ba18dcd4da041f043e186d4a9e8f5f	0	0	0	0	2	0	0	0	0	0	0	0
978a091309ea6ae895a24014d249516c	0	0	0	0	3	0	0	0	0	0	0	0
21a2befb639bdf393b3ab3424e8298a0	0	0	0	0	3	0	0	0	0	0	0	0
fa703199515ccb829ba0bc583cfecbfb	0	0	0	0	0	4	0	0	0	0	0	0
383b402e8e1ccdba0dac69d4de49ef79	0	0	0	0	0	0	0	0	0	0	5	0
220963d22fe52bd6531ed070d0b828b0	0	0	0	0	0	0	0	0	0	0	5	0
0d7247ce157bb8b9880f353008701e80	0	0	0	0	0	0	0	0	0	0	4	0
7eeaf41505849aac9192f6347466a333	0	0	0	0	0	11	0	0	0	0	0	0

50021dbeb2b0f09f47d8fedfc d11d08d	0	0	0	0	0	0	0	3	0	0	0	0
45e9d1c8953436f1d73ef8e3 b342f690	0	0	2	0	0	0	0	0	0	0	0	0
842369172af8af60ed80c274 cae330f1	0	0	0	9	0	0	0	0	0	0	0	0
aed15459a885528f02c98490 b8012b6a	0	0	0	0	0	0	0	0	5	0	0	0
bd50b31100f50129607cc813 fa96f8fc	0	0	0	0	0	0	3	0	0	0	0	0
47946a37b25ee032acb3bf71 ea214ba7	0	0	0	0	0	0	0	9	0	0	0	0
2c108602deced682479bebd 06b921aa9	0	0	0	0	0	0	0	0	0	0	8	0
2e048dfaed280cacb5735719 eecfdfab	0	91	0	0	0	0	0	0	0	0	0	0
03f94fb5d606b4f625f59a11e b1b39bd	0	0	0	0	0	0	0	0	2	0	0	0
02cd00b037c510cdf4a9ebf1 43ecf217	0	0	0	0	0	0	0	0	0	0	0	5
d3a77e8ad837db36cd5a927 978a8f781	0	2	0	0	0	0	0	0	0	0	0	0
2f0919adc76566de41de59ca 22be30af	0	0	0	4	0	0	0	0	0	0	0	0
0f0a4eeeffec691e979f92175 04399ec	0	0	0	0	0	0	0	0	4	0	0	0
8b5043f7493dca44ff8e5c62c 2e9c4e9	0	0	7	0	0	0	0	0	0	0	0	0

c076c22b23b67ca7eeef2f1ef289f6c1	0	0	24	0	0	0	0	0	0	0	0	0
a90fa2dad49eb5636ed75a96fad7fd41	0	0	0	0	0	0	0	0	0	0	2	0
9c1b9f12829da0af722c1724247a8569	2	0	0	0	0	0	0	0	0	0	0	0
bc6e3dbbb980d190783d2990940b4d91	0	0	0	0	0	0	0	3	0	0	0	0
9000d5ce04c67f78eb85ebe178216f90	0	0	0	0	0	0	0	0	0	20	0	0
1836ce876e149f774ae888daaad07492	0	0	0	0	0	0	0	0	0	0	0	2
41dff026d1f988bf27e869b97519c0f8	0	0	0	0	0	0	0	0	0	0	9	0
5e434aff5e6b1717e8a441422f4ad0bf	0	0	0	0	0	2	0	0	0	0	0	0
25a4fbb9e6363d3ffae67286337448eb	0	7	0	0	0	0	0	0	0	0	0	0
9ac7887b005de2e83cab0bb77945d970	0	0	0	0	0	0	0	0	0	2	0	0
1bb25176a3e370e5847c139ead938ba9	0	0	0	5	0	0	0	0	0	0	0	0
c96d6377c0c9cb7851101fcaa206fa04	0	0	0	2	0	0	0	0	0	0	0	0
19c28d5efd62efae8d435e1d29451b3b	46	0	0	0	0	0	0	0	0	0	0	0
810c1b146ef560658949e94cc6a287f9	0	0	0	0	0	0	0	0	0	0	0	3

12d7afe5ff1c4ec924b3fb631 6013398	0	0	0	12	0	0	0	0	0	0	0	0
d3c9317990f36d6bf7248b63 092b7074	0	0	0	0	0	0	6	0	0	0	0	0
b88b36ec06dd41224548b39 e045ce3de	0	0	0	0	0	0	0	0	0	0	0	7
97c5a61655b673f59cb0a081 c8a1e16d	0	0	0	0	10	0	0	0	0	0	0	0
95d830a1acfc1f87ffe493a8d 25f1ab8	0	0	0	0	0	0	0	0	0	0	0	5
4e6387af0cef0f94003f2501f 94d1da0	0	0	0	0	0	0	0	4	0	0	0	0
bd3b03fe97d2a3752bae8f43 8e53d06b	0	2	0	0	0	0	0	0	0	0	0	0
aafb438137bde2dabf032755 aa191832	0	0	127	0	92	0	0	0	0	0	0	0
67569ccad4945f2caf3d457b 6adc6030	0	0	0	0	0	4	0	0	0	0	0	0
40e6ec7a6caa907e645d4ae6 aa389d90	0	0	0	52	0	0	0	0	0	0	0	0
85352f60358b8bf757cb4177 c9a1add5	438	495	472	357	0	873	525	687	915	0	1174	898
87a1eba2bed42b621c8ec805 f8194aac	4	0	0	0	0	0	0	0	0	0	0	0
6b374b9ee17dc6517a794ae ac8f42bf0	0	0	0	0	0	0	0	0	0	0	20	0
2b0259b5d9141211579f78c6 14de2d4e	0	0	0	0	22	0	0	0	0	0	0	0

596a9307632893934f75d62ee1745223	0	0	0	4	0	0	0	0	0	0	0	0
0aa26ff92b75e5f3123c76086023da7b	0	0	3	0	0	0	0	0	0	0	0	0
779781f4017fc8289ec66e1d355f302b	0	0	0	0	0	4	0	0	0	0	0	0
50b26c98f184ce1e280890fbf11241eb	0	0	0	0	0	0	0	6	0	0	0	0
4f55e7add71a5b0a11898082f394837c	0	0	0	0	0	0	0	0	0	2	0	0
6b569b58ac302e94f8184bfe04659892	0	0	0	0	0	0	0	0	4	0	0	0
bcc56afd543700ff5710a04921b9092e	38	0	0	0	0	0	0	0	0	0	0	63
bc98c8bfcd1864b4ee4909320249b059	0	0	0	0	0	0	0	0	0	0	6	0
b7e6a2ea378507167478d625bfeb096a	0	0	0	6	0	0	0	0	0	0	0	0
fb85e81ea4c99227c59aa6dc54a314b0	0	0	0	0	0	0	0	0	0	0	0	8
b19f3110d66a4938474fae02787fa008	0	0	0	0	0	0	0	0	9	0	0	0
cef3ee0591eaaf316878dec5db911d35	0	0	0	0	50	55	0	0	0	0	0	0
042bd5e707db14fe8a211f2dd5965a85	0	0	0	0	0	2	0	0	0	0	0	0
4f427369a2b1ac66ac63ff4da19d3882	0	0	0	0	0	0	0	0	0	0	0	4

867c6b45b27b2f70500305ce5ec31e8a	0	0	0	0	0	0	3	0	0	0	0	0
078734721464bb460906dcea44170362	0	0	0	0	0	0	0	0	0	0	31	0
58c76ecc9224cc20755e1037b34b8bd3	0	0	0	0	0	0	0	0	21	0	0	0
9b7ef93cd1829e82c7fe4f323fc73877	0	0	18	0	0	0	0	0	0	0	0	0
903ce573e3cc65adb9a3c5f4e5382d83	0	0	0	0	0	0	0	0	0	0	0	6
ef31a97911400f4fc7ee9bcfe29ee34b	0	0	0	0	0	0	2	0	0	0	0	0
245ba23ab4b7238588dbdbbda6109c1f	0	0	0	0	0	0	0	0	0	0	0	22
1dc843a882cfef9cfc44cdd926c8b347	0	0	4	0	0	0	0	0	0	0	0	0
3dbabd2c914dfe495432bbd5f641038a	0	0	0	0	0	0	0	0	7	0	0	0
f8f2421f2e356a0b5aa4859894045589	0	0	0	0	0	0	0	0	0	0	0	40
a369763dd77879a9fda2500b7ccb72e1	0	0	0	0	0	0	0	0	0	0	2	0
0e51471095c70c7ebb18b74e0619efef	0	0	0	0	0	0	0	0	0	82	114	0
0a9817a21da8c46e0cd564d528e598a5	0	0	0	0	0	0	0	0	0	0	0	5
4aeb15ffd8901897036bdcf9220b7457	0	0	0	0	42	0	0	0	0	0	0	0

b3de24f6739f954086377ed7303a6dc0	0	0	0	0	0	0	0	0	0	12	0	0
10f8a98a3c31bf681cc8b94a826ba659	0	0	0	0	9	0	0	0	0	0	0	0
34d5019a240b488167538b97dceefeda	0	0	0	0	0	0	0	0	0	0	16	0
e532db894cb9e35c4b4aeecfdfddb6b0	0	0	0	0	0	0	0	0	0	0	0	38
b6340c250fe1466d32adfa7265ccf417	0	0	0	14	0	0	0	0	0	0	0	0
65e81dce9544263f99abf80e040919cb	0	73	0	0	0	0	0	0	0	0	0	0
25dc901aeb6b22590b4b7c96317bb755	0	0	0	0	0	0	2	0	0	0	0	0
4a153c02c7944a1dd5c855637e2098aa	0	0	0	0	0	0	0	0	0	0	0	7
54115218bd5605d0408ab12954e895db	0	0	0	0	0	0	0	0	66	0	0	0
ef0d9a9d1a7db162def9f3df0a7f40cc	0	0	0	0	0	0	0	0	0	0	0	3
2e716b73691ed4efc63d342bf73312de	0	6	0	0	0	0	0	0	0	0	0	0
972098fb6cf189e23afcfbfc5db1976b	0	0	0	0	0	3	0	0	0	0	0	0
e29517ba4e3d8caa22d25b23abe053b2	0	0	0	0	0	0	0	0	0	0	2	0
9bd9aa46d6d1a3208e3470d2a84ce744	0	0	0	0	0	0	0	0	0	0	10	0

2e1477cf6d4546f03902ac5a80f837df	0	0	0	0	0	0	0	13	0	0	0	0
64aac79cfd61e221d954332a170bd960	0	0	0	0	0	0	2	0	0	0	0	0
68c953213c358b7608805875678ebce7	0	0	0	0	0	0	0	0	0	3	0	0
3808c09d73fa6a7b07c8a22b1e613d5c	0	0	0	0	0	0	0	0	0	0	3	0
157f14676f0c501041a2c69e069a2a80	0	0	0	0	0	19	0	0	0	0	0	0
bca1b57705d818c41590f729e19b2d2b	0	0	0	0	0	0	0	0	0	0	29	0
cb1f323673a5bc6da3de52fb4da3cb08	0	0	0	0	0	0	0	0	0	15	0	0
1431c9d3925db5bfdd572edf4c4da3b6	0	0	0	0	0	0	0	6	0	0	0	0
a3011e719b66cdb59a084e4b723716cd	0	0	0	0	4	0	0	0	0	0	0	0
7cf3b60cf116b764a543129190a2b0c5	0	10	0	0	0	0	0	0	0	0	0	0
154fc5b943a413f3c9e0094029c71241	0	0	0	0	0	0	0	0	96	0	0	0
a919abd9789d073e7a8e80d3aa127df3	0	0	0	0	2	0	0	0	0	0	0	0
6ad943942d1b4e803023f82dbfc40f67	0	0	0	0	0	63	0	0	0	0	0	0
07b926a0ffac2048e4c76d8f19afb6ce	0	0	0	0	0	0	0	0	0	2	0	0

6d41eb6131f4cb4be2391df543ace6b6	0	0	0	3	0	0	0	0	0	0	0	0
cdb5cfd2cf22c3b2b670edcbed787376	0	0	0	0	0	2	0	0	0	0	0	0
85389a62774a02c0b9076dc247beb9ea	0	0	0	0	0	0	0	0	0	0	13	0
cb5e4c02e828ca32d582775e13196ee0	0	0	0	0	0	0	0	0	4	0	0	0
b1b8293f6cd9a2d9c28bbfd22ec100fc	0	0	0	0	0	0	0	0	0	0	0	4
1df7507461d481ccd214f40d442113c9	0	0	0	4	0	0	0	0	0	0	0	0
a0779a849e4ad107af51bcea9e6de81d	0	0	0	6	0	0	0	0	0	0	0	0
64e2016ee3ce4f8e46c9111383b39c82	0	0	0	0	0	0	0	0	2	0	0	0
2065e27a16aaa32d99eb2ec24fc53712	0	0	0	0	0	0	0	5	0	0	0	0
9b3ec814a29bfb520d52927a987c48d9	0	0	0	0	0	0	0	0	0	19	0	0
87df5a782819b6dab76e07122c41b844	0	0	0	0	0	0	3	0	0	0	0	0
26b56495a2bfe3d35bc1b5b8aa29e73b	0	0	0	0	0	0	3	0	0	0	0	0
f4442a6394d044074a4c83db17d59dee	0	0	0	0	0	0	0	5	0	0	0	0
adc3823a6fd23db9fc8eb3cd3562248e	0	0	0	0	0	0	0	0	11	0	0	0

ad1ce66d0398b80db4f607de6ec31241	15	0	0	0	0	0	0	0	0	0	0	0
c25849229436ef71bde4df335a96d264	0	0	0	0	0	0	0	0	0	0	0	3
5a0abdf85a7e67cfab4dd149efd3d69c	0	0	0	0	0	0	0	0	0	0	0	4
df73e395cbade3d071993ecfd0adac73	0	0	0	0	35	0	0	0	0	0	0	0
3a04aaaf557a1b9c2250d607e213d1c0	0	0	0	0	0	0	0	0	0	0	11	0
8296f3cdac9e6fe635874b9c974f0498	0	0	0	32	0	0	0	0	0	0	0	0
fcbf65dcf0c672cded95b77f33fd58d3	0	0	0	0	0	0	0	3	0	0	0	0
27683c7fe3f2b66524025739cb8b725d	0	0	0	0	0	0	0	9	0	0	0	0
6da9fa244b07960f53f9beb8326465d1	0	0	5	0	0	0	0	0	0	0	0	0
e99f903054b5a6f347a0f603a32ae4c4	0	0	0	0	3	0	0	0	0	0	0	0
8323afefd983f9f733a8893256e97c43	0	0	0	0	0	0	0	8	0	0	0	0
8f46884d28e777b40a025cbfdfe7ebaa	0	0	0	0	0	0	0	3	0	0	0	0
6362e4ebee1d78bffc6c99b405f6dec2	0	0	0	0	0	0	0	0	0	0	0	4
385c5adf8a58454c9e146324797949da	2	0	0	0	0	0	0	0	0	0	0	0

2a6a2cd7700ccb6dd3e98e414bdeaa4b	0	0	0	0	0	0	0	0	0	0	36	0
e46551612738520484f6e0af4c1b5762	0	0	0	15	0	0	0	0	0	0	0	0
505f0383fc275dc0379f78397f4820bb	0	0	0	0	0	0	0	0	3	0	0	0
9ee848b91bc4c25d2f3c6e6aeef637f2	0	0	0	0	0	0	0	0	6	0	0	0
88021e45aac8cd9a18cd674ac7863d25	0	0	0	0	10	0	0	0	0	0	0	0
be1165a8dc18ad398e85dbc2644ea87d	0	3	0	0	0	0	0	0	0	0	0	0
0e8dfbf69a9199e44bfd5c8d4d05fbd2	0	0	0	0	0	0	0	0	0	0	0	33
38b2239d9fd8bdbf68561b23565bcca1	0	0	5	0	0	0	0	0	0	0	0	0
fc1cbb10d7fb9de10431cc24e620ac03	0	0	0	0	0	8	0	0	0	0	0	0
06ee362af15929eac8dd24c0cd95d2f7	0	0	0	0	0	0	0	0	0	2	0	0
4eee3ff4af364959e846c6c30d24bb94	0	0	0	0	0	0	0	0	0	4	0	0
fb86eb51ce2f46e8790aac64b5ee65d8	0	0	0	0	0	0	2	0	0	0	0	0
52ff1427fd52aec447fe792859f16d01	0	0	0	0	0	3	0	0	0	0	0	0
e1b1834080c5ccf6b1d3bcec6dfd2d64	0	0	0	0	0	0	18	0	0	0	0	0

3c69ee0d9b4980b63d6cdff772fa8911	0	0	0	0	0	0	0	0	0	0	0	5
e098ca83c0d08a8bee3331579e8b6a93	3	0	0	0	0	0	0	0	0	0	0	0
37bf680d528fe2cb502406653b44d810	0	0	0	0	0	0	0	0	0	0	0	52
e7bffd9492bb16785f844bdef00ab8a6	0	0	0	0	0	0	0	3	0	0	0	0
e312cd89bf417f092864b7866acce940	0	0	0	0	3	0	0	0	0	0	0	0
5231e1f816b88d10ccefbd872ff9b488	0	2	0	0	0	0	0	0	0	0	0	0
f71456c90700203fbe516a0818c61578	0	0	0	0	0	0	6	0	0	0	0	0
681a4edf39cd0024d0ecb36c831565a0	0	0	0	0	0	0	6	0	0	0	0	0
3a4fe2d04ba62b6395e604f675466530	0	0	0	0	17	0	0	0	0	0	0	0
9bc1e25ac4f7eb6359d28f5658c84136	32	0	0	0	0	0	0	0	0	0	0	0
879973413b6f3dc7bdafd376a91358b2	0	0	0	0	4	0	0	0	0	0	0	0
a644cb5065c317af63a11b16e9d93d7c	0	0	0	0	0	0	0	28	0	0	0	0
e9bd2bfd79ff7317dc5a489cf8978b47	0	0	0	0	0	0	0	0	0	0	13	0
89b90dc4cd144a33ad57967f921f290a	0	0	0	53	0	0	0	0	0	0	0	0

521fb979c6754ad071f61a046ffb77d3	0	0	0	0	0	2	0	0	0	0	0	0
3763f7d07a8878a6c314e80f845c2b36	0	0	11	0	0	0	0	0	0	0	0	0
104eb8ddba6e99eb5aaf7ce8850c3f75	0	0	0	0	0	0	0	0	0	0	10	0
a3a0d8b843d8505f0d66f65e39cae674	0	0	0	0	0	8	0	0	0	0	0	0
ee34c440386bb01aaa47930187dc0818	0	0	0	0	0	0	2	0	0	0	0	0
f20b1ae4c2fa7cc426bca53ac5741157	0	0	0	0	0	0	0	0	0	16	0	0
cbd027063e0b729d9c6dade0697ec006	0	0	0	0	0	0	0	0	0	6	0	0
a49aa401cd229d65ed5feae109f7563a	0	0	0	0	0	0	0	0	0	0	0	2
71cde9e626e39642655f25b9e1216fe4	0	0	0	0	0	0	0	0	0	0	4	0
e06fe507f9cbedeba1f2c50bcd755843	0	0	0	0	0	0	5	0	0	0	0	0
c359d196596535df40b5323f4f4abe47	0	0	0	0	8	0	0	0	0	0	0	0
fede6c165e4e2b22641f3909c02678b6	0	0	0	0	0	30	0	0	0	0	0	0
94982b6135b41820bd76c47148ff9de5	0	0	0	0	0	0	0	0	0	0	0	2
bbd34f628cd8df4e4d7f9982d0e19ac5	0	0	0	0	0	0	0	0	2	0	0	0

1c66fb6d70584e5bbaef4056bfd72285	0	0	0	0	0	0	0	0	0	0	5	0
742d5fba7d3fdc0a201212be1dc4e788	0	0	0	0	0	0	2	0	0	0	0	0
40e6dc336e1d9ca21ce727c1cb4c8b03	0	0	0	27	0	0	0	0	0	0	0	0
bce6730161b76491e919fe1648505307	0	0	0	11	0	0	0	0	0	0	0	0
628ec8bc8949253b1b5a3a89aaa8320d	0	0	0	0	0	0	0	2	0	0	0	0
953838333d8def781de39418ed9f5b0c	14	0	0	0	0	0	0	0	0	0	0	0
49c2b89f027eda54095bbfe4eb7058f0	0	0	0	0	0	0	0	0	0	0	0	15
41f409f1c68f2e05203e42e908c10525	0	0	0	0	0	0	9	0	0	0	0	0
ad7902f87ab4fc264c1f93ae89538016	0	0	0	0	0	0	0	0	0	0	0	3
bc17719ae6b2359ba528f84f94676c5f	0	0	0	0	0	0	0	0	0	6	0	0
2470f5c160e76e6f90a32a8c492d6177	0	0	0	0	0	0	0	0	0	0	0	3
33bc9af379546b17b35ea6f7c659a80e	39	0	0	0	0	0	0	0	0	0	0	0
5af65521adbfdff4a76cd4252324cb0d	53	0	0	0	0	0	0	0	0	0	0	0
5d7d4f8a4d6bbf8e6238c6cb13c10fc7	0	0	0	0	0	0	0	7	0	0	0	0

1e4900ac8f6f5db41955b1ab97efa0f4	0	3	0	0	0	0	0	0	0	0	0	0
e238dfd1e6f73989fbc8c5ec8c5ba335	0	0	0	0	0	0	2	0	0	0	0	0
42e7ef9bf820f697adb2aee864d504ba	0	2	0	0	0	0	0	0	0	0	0	0
6d1b0ab4d1ff4202e387900f4222cedf	0	0	0	0	0	49	0	0	0	0	0	0
d30462c3e186690246c6677ab46eeead	0	0	0	3	0	0	0	0	0	0	0	0
70e5cdc53498f3f61e165281428873b5	0	0	0	0	0	0	0	0	10	0	0	0
17e6e975a5719d635bbb060fe6d32442	0	0	0	0	9	0	4	0	0	0	0	0
345d345f772c43610f21fe26a2fc2044	0	0	0	0	0	0	0	0	0	0	2	0
87b52e0fdeb43d78077cafb03901f421	0	0	0	8	0	0	0	0	0	0	0	0
6b30823dde515bba663cc26848b42725	0	5	0	0	0	0	0	0	0	0	0	0
491b2d30ff04fad579ea161a5a462715	2	0	0	0	0	0	0	0	0	0	0	0
141072971ed5034a8f1d930de366a20b	0	0	0	0	0	0	0	0	2	0	0	0
d2eb042d0d688097e67a26696cf29dd7	0	0	0	0	0	0	47	0	0	0	0	0
a48db34307c2929f85d6b6b0a8d530ec	2	0	0	0	0	0	0	0	0	0	0	0

2756fdf6a942cf9c11de6e1e1d861944	0	0	0	0	11	0	0	0	0	0	0	0
ad5950861377f3b6698afafacf219172	0	0	0	0	0	0	20	0	0	0	0	0
2c557ce98d78f1f76f66502ace712d09	0	0	0	0	0	0	0	2	0	0	0	0
787f8d093d13ea22464bf32f027d2cb9	0	0	0	0	0	0	0	0	0	2	0	0
f7fcaa273d5caf1dbfc6806ce3395807	2	0	0	0	0	0	0	0	0	0	0	0
b15ca0f08d683b213254c2890a30bc68	0	0	0	0	0	3	0	0	0	0	0	0
e2df3c3668387df83d40525d9c0a6ce2	2	0	0	0	0	0	0	0	0	0	0	0
31f59f80df8b923924969545191b0ba8	0	0	0	0	0	0	14	0	0	0	0	0
3d2297368db51a558b3ee3dc94bdeb55	0	0	0	0	0	0	0	0	0	0	61	0
7dadf5ebc57923e825d47c1ae7a36ace	0	0	0	0	0	0	0	0	0	0	41	0
a9da9863431dbf52431b07426c4fbff3	0	0	15	0	0	0	0	0	0	0	0	0
a0531374a3d04e4c5161ebe956f31e55	0	0	0	0	0	39	0	0	0	0	0	0
0565ae6623a32d9032ad3d17b7e08f29	0	0	0	0	0	0	0	0	0	0	340	0
e9d037f48261dcbececcbbaffae5592d	0	0	0	0	0	20	0	0	0	0	0	0

872265cfdc26020d68a90a9dca485896	0	7	0	0	0	0	0	0	0	0	0	0
bd1f804a861e456a2d4d560346bec8e1	0	0	0	0	0	0	0	0	0	0	3	0
594fd89fe6fbf7823f488714947d8576	0	0	0	0	0	0	0	0	0	0	0	6
955b6cb7874fb680ea056a9d6cf3e47b	0	0	0	0	0	46	0	0	0	0	0	0
f4cb51a0823baeee216197405d425af0	0	0	0	0	0	0	0	0	9	0	0	0
1b508e32755db9f28facf2faa86d3b55	0	0	0	0	0	0	0	0	0	0	0	3
ab51dc11f65ec58444e33f8201dfee42	0	0	0	0	0	0	4	0	0	0	0	0
bf30d2ad20877feeeda3c798c37a1361	0	0	0	0	0	0	0	0	7	0	0	0
1fd12753a4f40b57c68dc5535df05547	0	0	0	0	0	0	0	0	0	0	4	0
a9f78a176175c57fb308c1c655e82b7c	0	0	0	0	0	0	0	4	0	0	0	0
cd19df9e939e25980abd61f8f8e82c36	0	0	0	2	0	0	0	0	0	0	0	0
4316852c5d035ca853f6bc8754572332	0	0	0	0	0	0	214	0	0	0	0	0
f528e5399572146a823abe5199b7d335	0	0	0	0	0	0	0	0	0	0	0	9
18cc14733b1ffb0fa2c5b802dd28fbef	0	0	0	0	0	0	0	0	0	0	2	0

22650335d3f696fa2dcb41e365418142	0	0	0	0	7	0	0	0	0	0	0	0
bee80229b01b3a0f4c4ef31c16f53366	0	0	0	0	0	0	0	0	0	0	9	0
57dbbea58b52232afabf0fd7d6f50ecb	0	0	0	0	0	0	0	5	0	0	0	0
dfc5dbf1103ce1613a29ea76189fd60c	0	0	0	0	11	0	0	0	0	0	0	0
4f04c0c862609aaae5b4a516b8370529	0	0	0	0	0	0	0	0	0	0	3	0
602623ce60b21f5518fc026df224437d	0	0	0	0	0	0	0	0	0	0	22	0
d9acebd2003f0dce55de94b778432533	0	0	0	0	0	0	0	4	0	0	0	0
d7f1901aa73a4fef9833655a41c11ad1	0	0	0	0	0	0	0	15	0	0	0	0
c21e6ead4faf3f8557e39f113982b429	0	0	0	0	0	0	0	4	0	0	0	0
d9e438e13ac8e9a30af905ba015280f1	0	0	31	0	0	0	0	0	0	0	0	0
a8a86c35e95f7f17cf04d0bc992b316c	0	0	0	0	0	0	0	0	0	0	3	0
be375433c811552d7ee328d2188af86c	0	0	7	0	0	0	0	0	0	0	0	0
b1be843303bc35e30a64b608e7973e4e	0	0	0	0	0	0	0	115	0	0	0	0
a6adeec666a090f61c66eee02c8dbe5e	0	0	0	0	0	0	0	0	5	0	0	0

9191447ab208d153ccc0fbfd 90ed7c26	0	0	0	0	0	0	0	0	0	0	22	0
a38ad7951c7f43589ae35163 ad177847	0	0	0	0	0	0	3	0	0	0	0	0
4619254b4cfc9f376eff23a50 5d708ff	0	0	0	0	0	0	0	23	0	0	0	0
8fc607069f21f9f937ea731df 59f7221	0	0	0	0	0	0	44	0	0	0	0	0
8b4fe00ef2d915ff29e08526d fa0dea2	0	0	0	0	0	0	0	0	0	0	2	0
f9f97ecc6d6022cdeea87226 446064cb	0	0	0	0	0	0	0	0	0	0	0	6
0a3326c1e3d23da0e508fb39 46199959	0	0	0	8	0	0	0	0	0	0	0	0
fd254f04aa82a8839ff6db40e e75775e	0	0	0	0	0	0	0	0	0	0	2	0
448a0e7d860223c1882fa94e cb3dfce6	0	0	0	0	0	0	0	12	0	0	0	0
d44d765b8c323069749f9bb9 23e99cc8	0	0	0	0	0	0	0	0	0	3	0	0
8a421b4b3af5e5e2ca9aefab ed196632	0	0	0	0	0	0	0	0	0	2	0	0
3ec925ff8f589934ab16e1a37 7331434	2	0	0	0	0	0	0	0	0	0	0	0
9f98e95e8f98b39f45e5d371 bd29c6f0	0	0	0	0	0	0	40	0	0	0	0	0
4edb39261d812c130673f0b9 1ed73cd8	0	0	0	0	0	0	0	0	0	0	2	0

0a6269475ccc3f4ce8728abf5f5e50fa	0	0	0	0	0	0	0	0	12	0	0	0
51043899f597b1f9b03f0fdaf57c6cf0	0	0	0	425	0	0	0	0	0	0	0	0
650e6803bcf7dfd8afa6477faed151c4	23	0	18	0	0	23	0	0	0	0	71	186
19bc352c15c971f36ebce7f330569f2d	0	0	0	0	0	0	0	0	0	0	4	0
ea95a422a88dcf9686c77249f60249c6	2	0	0	0	0	0	0	0	0	0	0	0
7032bb5e5ca515ec0e1e33eb69161663	0	0	0	0	0	0	0	0	0	34	0	0
a4aade276c301710c959f89f3cfd8c25	0	0	12	0	0	0	0	0	0	0	0	0
4c82e03ad740914f8d1eb97d6f2d1b6e	0	0	0	0	0	0	0	0	0	0	0	12
4d77f8eefdf6a2c79511eae1bb75bee2	0	0	0	0	5	0	0	0	0	0	0	0
012096415b7736aafc8516d988f9eada	0	0	0	0	0	0	0	0	0	10	0	0
812bebd2e4ebeb27ed3af59d6ebe8076	0	0	0	0	0	0	0	4	0	0	0	0
9b60c742bde457ad795eadc76202e008	0	0	0	0	0	0	0	54	0	0	0	0
8be4ada6c64767d8a8e5a95ff9c61cec	5	0	0	0	0	0	0	0	0	0	0	0
5219680413d6966072e4860c0ea4c347	0	0	0	0	0	2	0	0	0	0	0	0

1992cdcd108f5da60aa2c0b61451b2b3	0	0	0	0	0	0	0	0	0	0	2	0
3dee6beb5fd2f680393e7ea44a26d5f8	0	0	0	0	0	0	0	0	0	0	0	9
8071edbd9eeaa030f1740eb1875a8ed8	0	0	0	0	0	10	0	0	0	0	0	0
7a7fbe3c673651cb5de6251512468e6d	0	0	0	0	59	0	0	0	0	0	0	78
45cb4d0398449a7424097b9f03fd35df	2	0	0	0	0	0	0	0	0	0	0	0
f5d54fa2a3954b58c5ef19e4b07896b9	0	0	0	0	0	0	0	0	0	0	2	0
4e6d75c20f9173a6053b0c193025f649	0	0	0	0	0	0	3	0	0	0	0	0
d90292b7d69b818aa8e2f8974d592cfa	0	0	0	0	0	0	0	0	0	0	34	0
616d5545f609a6015687cfef71c35164	0	0	0	0	0	0	0	0	0	0	0	2
564a257471b301829429c5eb123312df	0	0	0	0	0	0	0	0	0	0	0	8
e7934f99fa5cbd9541e410e2e7ad2980	0	0	0	0	0	0	0	0	0	0	0	51
0f292adac4a7ded5647d41a3e4c93dce	0	0	0	0	0	0	0	0	0	0	18	0
dffaba273523dabc313bd2841d82741a	0	0	0	0	0	0	0	0	0	6	0	0
c4006d3d8be9b7396032d1cc65bba82c	0	0	0	0	0	2	0	0	0	0	0	0

f495761d217d2d1d81c57cf4f52cf9a9	0	0	0	0	0	0	0	0	0	0	3	0
e8d24a3494da180f4c1ad9c7c03233d7	0	0	0	0	0	0	0	3	0	0	0	0
7d31179d8d28dec25c7649c6627f1a72	0	0	0	0	0	0	0	0	26	0	0	0
17cb705fcea8f5e3c86c75bb3ed75a8a	0	0	0	0	0	0	0	0	0	0	0	4
dc6d195ec1d05309273996ab00152d7d	0	0	0	0	0	0	0	0	0	0	0	15
676b2da5ad8a2e42dc607be40ec9a002	9	0	0	0	0	5	0	0	0	0	0	0
7ad78d378764cc405961ec17f1030cc5	0	0	0	0	0	0	0	0	0	7	0	0
d95a4a329a0c0dad128cc30fd75fae09	6	0	0	0	0	0	0	0	0	0	0	0
9b7e6ad4e5e92ed33f725e78857e06e1	0	0	0	0	0	3	0	0	0	0	0	0
aa87e0e34bf29cd1cbcda5dacfb675d6	0	0	0	0	0	0	0	0	0	0	5	0
6bebf884c94cbd331bd20c7c5c794d4a	0	0	0	0	0	0	0	0	0	0	0	5
56ec49e07ee840592db61f28b8772043	0	0	0	0	0	0	0	0	0	0	0	9
624c3336af744bb9c1bbe5c3402fac61	0	0	0	0	0	0	28	0	0	0	0	0
6d1f270d5062490850830a96f76637c0	0	0	8	0	0	0	0	0	0	0	0	0

eb92a8a28468ea30a2c2c50e89998882	0	25	0	0	0	0	0	0	0	0	0	0
bdf50c5454234e35350df82aeea967fd	0	0	0	0	0	0	0	0	2	0	0	0
e794f43a2ce8e0a9bd0832e38df22bc6	0	0	0	0	0	0	0	0	0	7	0	0
b736921491e7b472e11d0a2dd47c45ee	0	0	0	0	0	0	0	0	0	0	0	7
b3f6129e3f3b2fb1a41c5a11fd832568	0	0	3	0	0	0	0	0	0	0	0	0
8145bf4db6c4766e4fdcbfc63870f579	0	0	0	0	0	0	0	0	0	0	0	4
b1db2ea27d73631de2af5fcaa00f42d5	0	0	0	6	0	0	0	0	0	0	0	0
1106a87cf5695aba64ae07b05f1f502c	0	0	0	0	0	0	0	0	0	0	5	0
3ae30f7cac8e96199cffeeb0bb3797b8	0	0	6	0	0	0	0	0	0	0	0	0
65bc3ee804004e15edff3bc508638c3c	0	0	0	0	0	0	0	3	0	0	0	0
c9fe0a405b3046e756f30187a5cd4c70	0	0	0	0	0	0	0	0	0	4	0	0
b1199906e462948cae2c3921446cd5bd	0	0	0	0	0	0	0	0	0	0	2	0
fc51eeba21405bb549b7c6faff0e8f94	0	0	0	0	0	0	0	44	0	0	0	0
7e06f685ad4fedec277bb48beba37691	0	0	0	0	0	0	0	0	17	0	0	0

5470b2a76b8ee778d550f916 5443972b	0	0	0	0	0	0	0	0	0	0	2	0
ea494e4f84758cfb2b6823fa6 7056f5b	0	0	23	0	0	17	0	0	0	0	47	0
41c6629f6bfbfd2b99542463 6704a360	0	0	0	5	0	0	0	0	0	0	0	0
42afed97f3da8732fc953d75f 7329e8d	0	0	0	0	0	0	0	0	0	0	0	9
d029e64319fa8532bdd9e26a b20ede20	0	3	0	0	0	0	0	0	0	0	0	0
fcaf4df6646999abe786fc1da c4258f1	0	0	0	0	0	0	0	0	0	0	0	15
99f76538413aae6e812b61cf 140b3060	0	0	0	64	0	0	0	0	0	0	0	0
231deff1ec976d6706e37d93 2878feff	0	0	8	0	0	0	0	0	0	0	0	0
f72d2e68a3cacb60e9890066 76d8331d	0	0	0	0	0	2	0	0	0	0	0	0
1e24b910ff697439c9d8abb6 66fe01d8	0	0	0	0	0	0	0	0	0	0	0	2
77281b1c0147a64dc03bf14c f1354ba3	0	0	58	0	0	0	0	0	0	0	0	0
80136b684e9ffacc086866ba 2d1e25f0	0	3	0	0	0	0	0	0	0	0	0	0
dc19dfc7db060115a4861e8e f7ba57e9	0	0	0	0	0	0	0	18	0	0	0	0
4020bdf69e725b29fc1c17cb 16ef012a	0	0	0	0	0	0	0	0	0	0	0	3

d00b541fced30a4eefc924d39764c175	0	0	0	0	0	0	8	0	0	0	0	0
c001cd46c44d9919c51134a460f4bd8e	0	0	0	0	0	0	0	0	0	0	0	33
be7252d217f6491ad5fb1911a8a41032	0	0	0	0	0	0	0	0	0	0	0	36
7ac3db3d0ab94f47e30486105074fcf2	0	0	0	0	0	0	0	0	0	0	15	0
efa26853ba8d671a78f44bfc5607f00d	0	0	0	0	0	0	0	0	0	0	5	0
bf0a33cef4c76cd6253e4be8cbd474c6	0	0	0	0	0	2	0	0	0	0	0	0
ec95e74346a817c56ad30e46198565a4	0	0	0	0	0	0	0	0	0	0	4	0
e3b2d90d35e63089026c1433d10b1d50	0	0	0	0	0	0	8	0	0	0	0	0
f27f5999879f3d42827ab9ff58f1f8e0	0	0	2	0	0	0	0	0	0	0	0	0
4203a7256650708f020d4c8b0b840001	0	0	0	0	0	0	0	0	0	8	0	0
cbff6e316e2a57b3eeb1d4a1f2b7ecc3	0	0	0	6	0	0	0	0	0	0	0	0
09095c1aa2b8173344d4f8dddcc2a827	0	0	0	0	0	0	5	0	0	0	0	0
a5a73b3301bfe5257462d0ca06c3b3bb	0	0	0	0	0	0	0	0	0	0	115	0
32f19cb59d35f0cc121458be3ef0fd9b	0	0	0	0	0	0	0	0	0	0	5	0

877c6eb23b8edd2369d6982 31d9dc641	0	0	0	0	0	5	0	0	0	0	0	0
c4857d4e2be8078e95c025c3 919c1ed2	0	0	0	0	0	0	0	17	0	0	0	0
9d332391dabd2e6767a3732 3b8207aac	0	18	0	0	0	0	0	0	0	0	0	0
2aebf819ec46372f0e5de402 29cca6be	0	0	0	0	0	0	0	0	36	0	0	0
4bc0606839e5ba4fea191897 6c804f86	0	0	4	0	0	0	0	0	0	0	0	0
bd9c586f39600656229dc8a3 d528ba1e	0	0	0	0	0	0	26	0	0	0	0	0
6c979ea817de8c85b178937f e0375e3a	0	0	0	0	0	0	0	0	0	0	0	6
4ebbda1e89cb4b8dec94d93f f51ae5c9	0	0	0	3	0	0	0	0	0	0	0	0
906bca3fa8aab4197fb2e24d a88a3f27	0	0	0	0	0	0	0	0	3	0	0	0
6f24b80e9bb11a35869a61ce b411ccfc	0	3	0	0	0	0	0	0	0	0	0	0
f32a46715e3324b339c873d4 66028e44	0	0	0	0	0	0	0	29	0	0	0	0
78f1cb90f8a995da97e5611f1 070836a	0	0	0	4	0	0	0	0	0	0	0	0
4002a36a0dabf616aa61db05 135a3b36	0	0	0	0	0	0	0	2	0	0	0	0
b086fe76f1ffc9632e2a42f9c3 675bd1	2	0	0	0	0	0	0	0	0	0	0	0

e49e5d95cc5ec1a819755c0c dbcf2206	0	0	10	0	0	0	0	0	0	0	0	0
b5bc3684c052e611e1dde20 53d973ca2	0	0	0	0	0	0	0	0	0	15	0	0
c23bac8120fd9cb3168d8110 81ed90f3	0	0	0	0	5	0	0	0	0	0	0	0
946c81de14365e6473a71bd 5db7216fe	0	0	0	0	0	0	0	0	0	0	3	0
dd0898adbeb652e29700934 c29ed3601	0	0	0	3	0	0	0	0	0	0	0	0
a3eadf82ec38774b3e86f49f2 308be8c	0	0	0	0	0	0	0	0	0	0	2	0
c4ca60812f7318aff88f9e017 427f8cf	0	87	0	0	0	267	0	0	0	0	0	192
872fd3ce62aa6e8240323913 ac13e867	0	0	0	0	0	0	0	0	0	0	4	0
725aad34f40e30dfb10ae49d cf482271	0	4	0	0	0	0	0	0	0	0	0	0
4d2a406142037dbaf9ee6f53 7f000e65	0	0	0	0	0	0	0	0	0	0	47	0
f510f048bfe255f45d5008aeb f667898	0	0	0	0	0	0	0	0	0	2	0	0
b4ce7fb43f88e938412b3900 390c5225	0	0	0	0	0	0	0	0	0	4	0	0
f53a1ddf1a75db4a716eebf5 e88df6d7	0	0	0	0	0	0	0	3	0	0	0	0
f43054270b1b6693c6b808a0 211ac1e4	0	0	0	0	0	2	0	0	0	0	0	0

98833ab50ab24def1a316805 28ffdaa3	45	0	0	0	0	0	0	0	0	0	0	0
ec8135aba7e47a27cd1d3b81 ca71f883	0	0	0	0	0	0	0	0	0	0	2	0
5c1516ef49a064684cf3ba4f4 edd3376	0	0	0	0	0	0	0	28	0	0	0	0
bd7c5310de36da73cb5c96e6 5e1adc27	2	0	0	0	0	0	0	0	0	0	0	0
f68a868e6a6b8f37993efc6c8 ade02b0	0	0	0	0	0	0	0	0	0	0	8	0
64f1b70240893531abd37cac 0d04ab89	0	0	0	0	0	0	0	0	8	0	0	0
033c2962b069cea8746c3d48 a4d54600	0	0	18	0	0	0	0	0	0	0	0	0
1dd6bdfd624768b676e1357 b58847ba0	0	0	0	0	0	0	32	0	0	0	0	0
6da8fb5757977844ca443f5e 83d1de39	0	0	0	0	0	0	0	0	0	14	0	0
b8081ba7cff255b33cfb7a330 81b66e5	0	0	3	0	0	0	0	0	0	0	0	0
b46fed2ad29ef3e2cb3c08b3 5ea12935	0	3	0	0	0	0	0	0	0	0	0	0
31b1bd348d83748d034344e f84bb22ef	0	0	0	0	0	2	0	0	0	0	0	0
f6060401a0a4782ff334f6f4cc 5e5a0b	0	0	0	0	0	5	0	0	0	0	0	0
33e75af9e97827f031b1c82d 4c1f8673	0	0	0	0	0	0	3	0	0	0	0	0

d833e8c9be4f03ae6896274c1223ed6e	0	0	0	0	0	0	168	229	0	0	0	0
4acf2d4aa0574775bd4ad4ac72ce8f20	0	0	0	0	0	2	0	0	0	0	0	0
96dec25fe7162a7cb4a360a17342952a	0	0	0	0	0	0	0	0	0	0	79	0
251167f165e93e91246b3e36c12dc2f6	0	0	0	6	0	0	0	0	0	0	0	0
c72b59185de8a0c0ec54d898a871937a	0	0	0	0	0	0	0	0	0	0	2	0
752bddab6cc3487661897b21317a3604	0	0	0	0	0	0	0	0	0	0	5	0
0798556a81ef50d52fd05e2abebfa3ff	0	0	0	0	0	0	0	0	0	5	0	0
cae6f8d1a0647a8c1e5a4b5a3f34ead9	5	0	0	0	0	0	0	0	0	0	0	0
3e44ba69d267de0a388db7defc4b5d28	0	0	0	0	0	0	0	0	0	0	2	0
5f5ce63113fe7d0731e3fc080cb97e3f	0	0	0	0	0	0	0	0	0	0	19	0
a25080e41df09737804aa07a24bf1511	0	0	0	31	0	0	0	0	0	0	0	0
b51a75ab823476d03dca19623813a65c	0	0	0	0	0	0	0	21	0	0	0	0
433252e951b476b1dbe58db890210289	0	0	0	0	0	0	0	0	0	0	0	19
7cdbe7af9a88978a952de6e9b8cbe370	0	0	0	0	0	0	0	5	0	0	0	0

f15f035885a772d6e2ffb9942 64f41f8	0	0	9	0	0	0	0	0	0	0	0	0
3ff0d7d6902a4c470f5b6a96c dc318d0	0	0	0	27	0	0	0	0	0	0	0	0
fd325e42a4ffe6940b386fb81 134e412	0	0	0	0	0	0	0	3	0	0	0	0
e4859ffd58bad648f438f3878 8d578d3	0	0	0	0	0	0	0	0	0	5	0	0
79d3d02746ae2f95d405f43fc 11f40e9	0	0	0	3	0	0	0	0	0	0	0	0
d556b922fc1fc4ed8013094d e3cbb1e3	0	2	0	0	0	0	0	0	0	0	0	0
a47322be2baa5c7470b61df6 a0ed6961	0	0	0	0	0	0	0	0	0	0	0	47
d35cb33d3699cb88df2abd8e 9b3a66b2	0	0	0	0	0	0	0	5	0	0	0	0
a96fb10dd18bdba9e3ce6e4f 69bcf5d2	0	0	0	0	0	0	0	0	0	0	0	14
22e678c0165f05e23ea46678 737c5eb2	0	0	0	0	0	0	0	0	2	0	0	0
3bae20ccdbfec561b623e8d7 e0ac940f	0	0	0	0	0	0	0	0	0	0	0	6
eed98c23bf8cc89fb28e3d87 26ef5aa8	0	0	0	0	0	2	0	0	0	0	0	0
1daf9d79bff9b9909195f603c 068024d	0	0	6	0	0	0	0	0	0	0	0	0
88f139ac3ef2caab81cc977e4 75035c5	0	0	0	0	5	0	0	0	0	0	0	0

c1f6d5a598bada2d4f2d17ec bcdbde2b	0	0	0	0	0	0	0	0	9	0	0	0
0c8739c10e64615bc62e83d4 215a79ad	0	0	0	0	0	0	0	0	0	0	0	3
526a2c02ed60429d6b3fea13 5d33a219	0	0	0	0	0	0	0	0	2	0	0	0
bd472fd68008d19653061bc5 44c6dc2d	0	0	0	0	0	0	0	0	0	15	0	35
419c6a372787693c46bc7b9c 671ba750	0	0	0	0	0	0	0	0	0	0	5	0
43509ffc47866ea81b4b3944 6c0086c1	0	0	0	0	0	0	0	0	0	0	0	4
db56f955fb0b6a1a5d344754 48f04c44	0	0	0	0	0	0	0	0	0	0	4	0
48fcdfa5e45781d7e797fad57 d23da99	0	0	0	0	0	0	0	0	0	0	0	7
0c6f6765f989f873a5b3abcdb 619610a	0	4	0	0	0	0	0	0	0	0	0	0
76021ce089c388998d2e9f4e 2869881f	0	0	0	0	0	0	0	0	164	0	0	0
6573341f5be01b46ab9f2784 f1c2e148	0	0	0	0	0	0	0	0	0	0	2	0
f49e711b4ae1c7b577164668 1b395b3c	0	0	0	0	0	0	0	0	0	0	0	8
412c5de3f39f62a1397027d1 45b8b24f	0	0	0	0	0	11	0	0	0	0	0	0
ecdd4923feed2ff125eb9afbf bb25665	0	10	0	0	0	0	0	0	0	0	0	0

65d9d2398b05f994907ebb530b8cf5f3	0	30	0	0	0	0	0	0	0	0	0	0
92038b6a51c9567ce6feae2521090874	0	0	0	0	0	0	0	3	0	0	0	0
5df390d48d8a5d2758f59cc566a15e9e	0	0	0	0	4	0	0	0	0	0	0	0
665906e01983bb2152385cfb6eae1abd	0	0	0	0	0	0	0	0	0	0	0	7
36dd5bd04008b39b0e920a8d928578de	0	0	0	4	0	0	0	0	0	0	0	0
298e4a66640212c2f63e3ae41ffd7d6e	0	4	0	0	0	0	0	0	0	0	0	0
9995dc5059df6d98321e143208b1f6de	0	0	0	0	0	0	0	0	0	9	0	0
7a180d5907741e7449e95f04da73b853	0	0	7	0	0	0	0	0	0	0	0	0
88635b3fda2df423ceb93cec0bc0bad7	0	0	6	0	0	0	0	0	0	0	0	0
23575462c20444ffae91f214eead315f	0	0	0	4	0	0	0	0	0	0	0	0
da3fc1ccf8a0ebfbc353d065703bb092	0	0	0	0	0	0	0	0	0	4	0	0
80757581b574a34fe3464e7db51872ce	0	0	0	2	0	0	0	0	0	0	0	0
69e981bf0d7d06baa6cf6f0dd4de3ed2	0	0	0	0	0	0	0	0	10	0	0	0
4a93ef620f50c40dbe96f3628dd477ce	0	0	0	0	0	0	0	0	0	4	0	0

498b487360de2fd520469d6a8187c015	0	0	0	0	0	4	0	0	0	0	0	0
852c09881534959271012b41737dac39	0	0	0	0	0	17	0	0	0	0	0	0
6eebf12fbb31f2fe87e5b01f8d0df3e2	0	0	0	0	0	0	0	0	0	0	0	2
f1d859f644999a638e73c9d7e2601e2c	0	2	0	0	0	0	0	0	0	0	0	0
644eed16c7f625ce6b51ee6ebbcea7dc	0	0	0	0	0	0	9	0	0	0	0	0
f4e0b294a22aa6e8b26ac4dde59a9cf3	0	54	0	0	0	0	0	0	0	0	0	0
d9ea9376714676a4433a2f3a4144bd84	0	0	0	0	0	0	0	0	0	0	15	0
5c8122fade2c3d97b7fe5c74558b6a4c	0	0	0	0	0	0	0	0	0	0	0	24
ddde41836264c11a0650b9dee0972f92	0	0	0	0	0	0	0	0	0	5	0	0
da5e722d9a1dea5cc7ca430beb441cf9	0	0	0	0	0	0	0	0	0	0	50	0
bbe03de2d68c1c2dc38842049c03c33f	0	0	0	0	0	0	0	0	2	0	0	0
474c511e1272981b79b35cfab3bf85db	0	0	0	0	0	0	0	0	0	0	0	3
5ca5782a039217b5b4166f5df6adefd0	0	0	0	0	0	0	3	0	0	0	0	0
173b445bee49f3ae09fd6d61559e8271	0	0	0	0	0	0	0	0	0	7	0	0

11410128a3dc76b61f944eaee2512c49	0	0	2	0	0	0	0	0	0	0	0	0
7d134840218f318499edf0355326aff4	0	0	0	0	0	0	0	0	2	0	0	0
65a2f7be3ece4158053751b83eb08a66	0	0	0	40	0	0	0	0	0	0	0	0
3b1811454b8349e5dceb9f675ea5e40d	0	0	2	0	0	0	0	0	0	0	0	0
0200363abf860401a61800314f71684f	0	0	0	0	0	0	0	0	2	0	0	0
e9f4658671e58dfa52818c592ad377ed	0	37	0	0	0	0	0	0	0	0	0	0
778e392eccd1f7bb10c4368fd4c3dae2	0	0	0	0	0	0	0	0	4	0	0	0
0b7881509820689ff62bf73f8159d41e	0	0	0	0	0	0	0	0	0	0	0	3
4e769625c685688fcca931f7d39fd4fc	0	0	0	0	0	0	0	0	0	9	0	0
26e05860cada5dca9be4b6050a7709b4	0	0	0	3	0	0	0	0	0	0	0	0
784e588413a55f5f9c5808a62064a5ff	0	10	0	0	0	0	0	0	0	0	0	0
0a181ce09d1a5fb22d8c9aca5c20ec53	0	0	0	0	0	0	0	0	0	0	0	7
5ea1940b299fa90000db9de9936605d9	0	6	0	0	0	0	0	0	0	0	0	0
c80717e2e25976e4ed42aa4942a01114	0	0	0	0	2	0	0	0	0	0	0	0

876a7e5fa783f1d1b25d81c44a0f94a3	0	0	0	0	0	6	0	0	0	0	0	0
4e6ea22be59706cd7da5b0e1e9ca8491	0	0	0	0	0	0	0	163	0	0	0	0
54870633388e0085f8e80f8234274b8a	0	0	0	0	0	0	26	0	0	0	0	0
cd9ab323a3f9135339eaff92d712917f	0	0	4	0	0	0	0	0	0	0	0	0
d133f865b33ed9dceb5e559d8fc9b5f5	0	0	0	0	0	4	0	0	0	0	0	0
733dfdb919baaa001fa710adf6cb77aa	0	0	0	24	0	0	0	0	0	0	0	0
4a539613045445de68068c5df3c4e2a4	0	0	0	0	0	0	0	0	3	0	0	0
4a8c3b32d4f5ee5e8a851a951fc4e061	0	0	0	0	0	2	0	0	0	0	0	0
794a2c782e723d8899e29d5870e29c16	0	0	0	0	0	0	0	0	0	36	0	0
9b1fac94119f449f10c47a031a48f418	0	0	0	0	0	0	0	0	0	5	0	0
74868974c6d6e859b362b7aa1aad2305	2	0	0	0	0	0	0	0	0	0	0	0
af55b7681f72891fb91e3916782f0b71	0	0	0	0	0	0	0	0	0	13	0	0
ef246f24fae800fd6eec5437160e6c57	0	0	0	0	0	0	0	0	0	0	0	15
bed8337c43c6d9a90e09aa041dcc04c4	0	6	0	0	0	0	0	0	0	0	0	0

e9c357ae71a6ba63986714ed3b9f589b	0	0	0	0	0	4	0	0	0	0	0	0
e2afae26681bfe735cc7185e6d270045	0	0	0	0	0	0	0	0	0	4	0	0
8d87cc7e782900ac124510bde6029900	0	0	0	3	0	0	0	0	0	0	0	0
1771999989012d6020cb4007cdcf3156	0	0	0	0	0	0	0	0	0	0	0	5
a0e41834544219b64154c1529b10773d	0	0	0	4	0	0	0	0	0	0	0	0
b96463077e5cf1992b8b6d670eb78808	0	0	0	0	0	12	0	0	0	0	0	0
3062ced684b5a4653d28b3826d5d0922	0	0	0	0	16	0	0	0	0	0	0	0
479f2e7384da3c9e4d910821545b9e1c	0	0	0	0	0	0	0	0	9	0	0	0
4ed7f77eddbe3fae825bf4ae2b026aaa	0	0	0	0	4	0	0	0	0	0	0	0
82d40d402167e2c5ef9942a29244df01	0	0	4	0	0	0	0	0	0	0	0	0
c4682e1fab363b4902c7a23d6a7319c0	0	0	0	0	0	0	0	0	0	0	0	12
460887dfba4aaa4ca12965c37e3a32b2	0	0	0	0	0	0	0	3	0	0	0	0
683b2e48c1cfe73f33fead25696abe65	0	0	0	0	0	0	0	43	0	0	0	0
7dc48912292674958fc093606a6add29	0	0	0	0	13	0	0	0	0	0	0	0

c3e3d75b08b27f1c656e0299 18a4db19	8	0	0	0	0	0	0	0	0	0	0	0
d88e0270bee2bc8377c0f518 c2b84048	8	0	0	0	0	0	0	0	0	0	0	0
3ad051f010836fc999db8178 d2f09771	0	0	0	0	0	0	2	0	0	0	0	0
4545e3af710e09d45885452e c7df8b46	0	0	0	0	2	0	0	0	0	0	0	0
f2a537826c8ec01f7d3a9aaad 07f17cd	3	0	0	0	0	0	0	0	0	0	0	0
8b5a1743048cd9e22ab2072 69ce117eb	0	0	0	0	0	3	0	0	0	0	0	0
1128f86cb192a5264c9a497e 27484222	0	0	2	0	0	0	0	0	0	0	0	0
7f791559761fbd94d962cb38 671fa5bc	0	3	0	0	0	0	0	0	0	0	0	0
bf264ebe1b5a25fa3e97947a 378b9bff	0	0	0	17	0	0	0	0	0	0	0	0
d452c401f07e704c9aa623d5 175f907a	0	0	0	0	0	0	0	0	0	0	0	7
c08077163ff1413435d43101 a8f77645	0	0	0	0	0	0	0	0	0	0	2	0
54930850f651557a445e7f60 d959eaf1	0	0	0	0	0	0	0	0	0	0	3	0
4b14c622ae254969b4adba8c 4c9198d5	0	0	0	0	0	0	0	3	0	0	0	0
70ed5d02b43709463a14eb1 d5becabb8	0	0	0	0	0	0	0	0	0	12	0	0

0afcf3d957da427d6091868f310a8041	0	0	0	0	0	0	4	0	0	0	0	0
6647ede3877eb8a14508cba2c89568d1	0	0	0	0	0	0	0	0	0	25	0	0
e4724c3dec46935c72b311218139b9ff	0	0	4	0	0	0	0	0	0	0	0	0
ceafb997227237fd0525c78e6eae0357	0	0	0	0	16	0	0	0	0	0	0	0
0870be5a9d3fbc7fcdb2d1b6248ebd85	0	0	8	0	0	0	0	0	0	0	0	0
5c01279e6170dc7ed8cb2ea89683ddc4	0	3	0	0	0	0	0	0	0	0	0	0
2d84f30e708f6dd305680c8e1f51aa68	0	0	0	0	0	0	0	0	0	0	2	0
2db6342835134a86b2b28031ebb3e5e6	0	0	0	0	0	0	0	0	0	0	0	9
9a3a445df3e6e9be8e4f2837608611d8	0	0	0	27	0	0	0	0	0	0	0	0
f0fc16b3b86ffab2e1f1ffbade7e3344	0	0	0	0	0	0	10	0	0	0	0	0
85af1fcb0a8f6b5d62bc242178de3699	0	0	0	0	0	0	0	0	0	0	0	8
2f6f20b410356351e7912489612e1256	0	0	0	0	84	0	0	0	208	45	240	17
c026c36eeabbcdcde15b54943e3074c9	0	0	0	0	0	0	0	0	0	3	0	0
5bee5949c10c04600c97bc6b19fa30ae	0	0	0	0	0	0	0	0	9	0	0	0

04885556a50c99f5dac8a303235859cb	0	0	0	0	0	0	0	0	0	0	3	0
a959c37e92229053e05fec237fee5d0f	0	0	3	0	0	0	0	0	0	0	0	0
f7aba5d265fa64809874dd78d3e9925d	0	0	0	0	0	0	0	0	0	0	7	0
5cd240930447976a746a1cbcd082d54a	0	0	0	0	0	0	0	0	0	0	9	0
27ae9aa0ddd924c5e3724eec4191f1b7	0	0	0	0	0	0	0	0	0	0	0	4
521c9bcc14475cf010b5c5d4a095f06b	0	0	0	0	0	0	5	0	0	0	0	0
121400bba5811f2070907f6dae115b72	0	0	6	0	0	0	0	0	0	0	0	0
7b8500741278d13f617c187a976163f8	0	0	0	36	0	0	0	0	0	0	0	0
660f89b96ebd481f2faabce16e22dd8b	0	0	0	0	0	0	0	0	0	93	0	0
22cfe3d6db138bf299c01cc81ecd6d71	0	0	0	0	0	6	0	0	0	0	0	0
3abd674767c61aa3e4df13b52a2c02d4	0	0	0	11	0	0	0	0	0	0	0	0
1788837614766fc5e643c0a73913706d	0	0	0	0	0	0	0	0	0	0	0	4
5e4d3d98e6bd6fa6ba0f69fdd0364b35	0	0	0	121	0	0	0	0	0	0	0	0
b94e05434fd70c11148fe1c1bbe00579	0	0	0	0	2	0	0	0	0	0	0	0

c05ba78a92de9d74e6a4063d1b8dd20b	0	0	0	0	0	0	7	0	0	0	0	0
169ad5aef57f5f662c36dce3831dacea	0	0	0	0	0	0	0	0	4	0	0	0
7f74ef6ed1bcf9dc4fe7bdce33c50463	0	29	0	0	0	0	0	0	0	0	0	0
89fc9a31c9b33c1b06e0ad045ca30101	0	0	0	0	43	0	0	0	0	0	0	0
da7551d498899784752b41bb6e84cbd1	0	0	0	0	0	0	4	0	0	0	0	0
cc279680a8298cff7bc8964e6713d209	0	0	0	0	0	0	0	0	0	0	11	0
2b2e5f25cd5d8327397125ab1bd287d4	0	0	0	0	0	0	0	0	0	0	4	0
7ff04bf0bdf26b184f99cc4951aa01db	0	0	0	0	3	0	0	0	0	0	0	0
358d8c45bcd1e438b2085cc7d4e68719	0	0	0	0	0	0	0	0	0	0	0	2
91622298e4e1a76e3a1ff45b3295e45a	0	0	0	9	0	0	0	0	0	0	0	0
4e68616cf76b739118efe7203180d13d	0	0	0	0	0	93	0	0	0	0	0	0
c5f09aa0b3506addf4d4e38f42187c01	0	0	0	0	0	6	0	0	0	0	0	0
1ff5637002e765533b094226c8cee5db	0	0	5	0	0	0	0	0	0	0	0	0
a3df4f86e50cad1b35598efb25128579	0	0	0	0	13	0	0	0	0	0	0	0

6836b8bbd41fadd4f2a9ab0b2aa24528	0	0	0	0	0	0	0	0	0	0	4	0
8b822ca82455b382b72b04ba128b9c29	0	0	0	0	0	0	0	0	0	4	0	0
54621fc763c024c899a82a30fb7bddcb	0	0	0	0	0	0	0	0	0	0	0	6
0a2f23a9ef7f8cbbe5ee9fca6a0ae052	0	0	0	0	0	0	5	0	0	0	0	0
29d5b0dde1acd82321e393806b5b1d2b	0	0	0	0	3	0	0	0	0	0	0	0
9edaf532c7dc05aa1a60f68dfaa44ec7	0	0	0	0	0	0	11	0	0	0	0	0
8b62e08c4b65510cb2c8d824cf6a9999	0	0	0	0	0	0	0	0	0	3	0	0
5445e0c45080b02704964e5f5056c489	4	0	0	0	0	0	0	0	0	0	0	0
ad9cfe2a3f02e9c1fa541825290ffc97	0	0	0	0	2	0	0	0	0	0	0	0
7eb94ae11091b583e906b73c49d0aa81	0	0	0	0	0	4	0	0	0	0	0	0
bd57c3964697ea7c2ec1e508fcbdcaa9	0	0	0	6	0	0	0	0	0	0	0	0
7fa10a16ca645088b12f946d8520fd1a	0	0	0	0	0	0	0	0	0	0	0	7
1bc2e8873dedcf69b12f9ec0a64f3517	0	0	0	0	0	0	0	0	0	0	8	0
1722a83dc9b202aa1b52ddc1abe0bb6e	0	0	3	0	0	0	0	0	0	0	0	0

5a16245b72e225e7cfda343ab5b82540	0	0	0	0	0	0	0	0	0	0	0	5
e3f95472a296931821fb657f745d340c	0	0	0	0	0	0	0	0	0	0	3	0
a53dd146c59889c007cfe3c198c1a658	0	0	0	0	0	0	0	0	0	0	17	0
3bd4b75fd1e33113afc14720feafda62	0	0	0	0	0	0	0	0	0	0	0	4
0a3c6de2dda1cae53e26e625c7bf78a8	0	0	0	0	0	0	0	0	0	0	0	12
85e2972ce12acda0f524c59d821eb97e	30	0	0	0	0	0	0	0	0	0	0	0
a1deb5f613169e0f47badef7bb82ef21	0	0	0	0	0	0	2	0	0	0	0	0
fc17ee657a962aef2ec4005dc6023b8c	0	0	0	0	0	0	3	0	0	0	0	0
9a6b76794bf9fc353cfac64066bd1733	0	9	0	0	0	0	0	0	0	0	0	0
04814f38a635d5c6fb09fa81d6bfd5c6	0	0	0	2	0	0	0	0	0	0	0	0
59df38aa10018f67b8a0d71a63625d13	0	0	0	0	0	0	0	5	0	0	0	0
30deaf9cf282d74c8094f0a5f636e056	0	0	0	0	0	0	0	0	2	0	0	0
1b5a26595055e5fee6c450da0a307d51	0	0	0	2	0	0	0	0	0	0	0	0
4550249334c675c4907b23b662a01abb	0	0	0	0	0	0	0	0	4	0	0	0

4dc712e7b9eec240f7668d238ce19318	89	0	0	0	0	0	0	0	0	0	0	0
a8c97e71acc39428f27ba8b6b46901c4	0	0	0	0	0	0	0	0	0	0	3	0
d4dd1dba5fe4e1dce7648d5943ae8c2a	0	0	0	0	0	0	0	0	11	0	0	0
599b2f7538f610dc3826ee1cb61cbcc9	0	0	0	0	0	5	0	0	0	0	0	0
e13dc1dec5ea3d3bd065a38278c715f8	0	0	0	0	0	0	0	0	0	0	6	0
e04fcd63fad75db3224e7e9059bb191d	0	26	0	0	0	0	0	0	0	0	0	0
5d225226c097a4e6d499bb9d2aedee3f	0	0	0	0	0	3	0	0	0	0	0	0
c2afa6b13ec8202e4670152a0bcd37f7	0	0	0	0	0	0	0	0	0	0	4	0
78c0c38179449d31ad3159a545e20dba	0	0	0	0	0	0	4	0	0	0	0	0
351e41b514931d008a8ed3d278946a0d	0	0	0	0	0	3	0	0	0	0	0	0
8f7a228765766b4990b78808c5ccfa3a	0	0	0	0	0	0	0	0	0	0	0	5
51f0e41d66990f6abd908055ac752884	0	0	0	6	0	0	0	0	0	0	0	0
b44de5a227d101359ce5c5e68a5fcd46	0	0	0	0	26	0	0	0	0	0	0	0
0084d646844d004fcd9f5636c3c44136	0	0	0	0	0	0	0	2	0	0	0	0

3a7f7e132d70bb4e2be54eac74e601f9	0	0	0	0	0	0	0	0	0	0	0	5
f4145ae8454bd6562ffa641454c9de3f	0	0	0	0	0	0	0	0	0	0	0	2
702385fecc7f99d6f28edb4346df9686	0	0	0	0	0	0	0	0	0	0	3	0
eb0106d8b907d8f8d31b924b57c82afe	0	0	0	0	0	0	0	0	0	0	60	0
918b7b6c53eda243e005715f6eedd6ee	28	0	0	0	0	0	0	0	0	80	0	0
5355e3a14b23d27339167a09bc041b5c	0	0	0	0	0	0	0	0	0	0	5	0
5d564d8b5ef360747dc079a0475d38f7	0	0	0	0	0	0	0	0	0	2	0	0
40b3de98087fb12c00f48fa45f794e11	0	0	0	0	0	0	0	0	10	0	0	0
fe7430f1a1650ddf65ff6944fdbfbf78	0	0	0	0	2	0	0	0	0	0	0	0
739eb600d12249fd9c3ba6435aae518d	0	0	0	0	0	0	0	0	0	0	15	0
e01566ad2e091d43ce37b5a7e39b65f3	3	0	0	0	0	0	0	0	0	0	0	0
1e2cdf5444b2d16f67860ab3d7d0ef1f	0	0	0	0	0	0	0	8	0	0	0	0
515ed9d0cc36e6bf65cd7b6383eaa211	0	0	0	0	0	0	0	0	0	0	0	2
6435c33e5e49b6a4629564d7ea950154	0	0	0	0	0	0	0	0	11	0	0	0

ee7bbb863a7e550abc14e1fa6bb66edf	0	0	0	0	0	0	0	0	0	49	0	0
a2a33c26a21e926bb95f6b09aaed5a67	0	0	0	0	0	0	0	0	0	0	7	0
a3d9c7088ad7261a2d1b230c27c851c2	0	0	0	0	0	0	3	0	0	0	0	0
89f0b906b4df39fff2d1933ab2f3d703	0	0	0	0	0	0	0	0	0	0	0	3
80a025348861936bf3878d0e0249e422	0	0	0	17	0	0	0	0	0	0	0	0
d656678a2e7cc4fd2ebe21e1ee366bed	0	0	0	0	4	0	0	0	0	0	0	0
d92547968117da116f744a723615aa2d	0	0	0	0	0	0	0	0	0	0	3	0
0ae9da972031e947a70078292d943a49	0	0	3	0	0	0	0	0	0	0	0	0
33d8e5a9dda949ffac086f2333051ab1	0	16	0	0	0	0	0	0	0	0	0	0
0adcea9a657c4a97a00a40215994ddc4	0	6	0	0	0	0	0	0	0	0	0	0
edd8b2699ed65204a7244df8e0da6c9b	0	0	0	0	7	0	0	0	0	0	0	0
c3b0d83cb00be71c77bc11c4b1e9b0a4	0	0	0	0	0	0	3	0	0	0	0	0
68db1fc0b0ae430266541feafcbcef04	0	0	0	0	0	0	0	0	0	0	2	0
a2f2cf3fc6f371ee298085cd1480baf6	0	0	0	0	0	0	0	6	0	0	0	0

3efd73b8e0ec9102e22b0757 1865f950	0	3	0	0	0	0	0	0	0	0	0	0
20875e06388cf878ce5a00cb 4ba1b2e4	0	0	0	0	0	0	0	0	0	0	5	0
25e378bc84cd409c7a049e00 8fa4f2a4	0	0	80	0	0	0	0	0	0	0	0	0
baef94163dafe0a6ef36b09ea 7bd968b	0	0	0	0	0	0	0	3	0	0	0	0
f23f83267b50987446b8597f 13ae6e56	0	0	0	0	0	0	0	0	6	0	0	0
66b521d4273c80847bcd54f8 12020e36	0	0	0	0	0	0	0	104	0	0	0	0
a2a5785ed6ec8be07a9aa63b e0f8a23e	0	0	0	0	0	0	0	0	0	0	3	0
0acb4af57ef7f6b7e3dee539f 2f3fcfa	0	0	0	0	0	0	6	0	0	0	0	0
fbb7fc1b0772295e4c1f846ec d399e68	0	0	0	4	0	0	0	0	0	0	0	0
fb73515fa8a01c49308c42c82 294cd83	0	0	0	0	0	0	0	0	2	0	0	0
b8e184abdcefa0b8eafb8105 1945aede	0	4	0	0	0	0	0	0	0	0	0	0
1c12c936a80c80f0696ef500e 2158ec5	0	0	0	0	0	5	0	0	0	0	0	0
1e1c7a5d4fd7cfb90e1d4778 66fe0a31	0	0	0	0	0	0	0	0	0	32	0	0
79c532f5484589b682667228 023ee682	0	0	0	0	0	0	0	0	7	0	0	0

7bfba3cc2cb092c0a48508bf5f212e0d	0	0	0	0	0	0	0	0	3	0	0	0
75a45bb19dcab6a9a0cac9642cc78ae3	0	0	0	11	0	0	0	0	0	0	0	0
fb2fbd514d15726c9b0b62e17924b1a0	0	0	0	0	0	0	0	0	0	0	8	0
1273cfcd029933de787c6d8a05bd9379	0	0	27	0	0	0	0	0	0	0	0	0
12c1cceb3684703114cb7b3984d83489	0	0	0	0	6	0	0	0	0	0	0	0
67dcbd38647ec470f7a308a20ede3e51	0	0	0	0	0	0	0	0	0	0	0	2
991750ad82290cd597d6adafcfb74bbd	0	0	0	2	0	0	0	0	0	0	0	0
8ac8881962637be5df3489774edda88f	0	0	5	0	0	0	0	0	0	0	0	0
30322680ff0e0405c8ad6e8fea5264c9	0	0	0	0	0	0	0	0	0	0	2	0
6835660cee9de6e8de388be572386f88	0	0	0	0	0	4	0	0	0	0	0	0
8267aa2b619492d77bd9cf29d72c2287	0	0	0	0	0	0	8	0	0	0	0	0
51d412df51d753e818dd63e14d594e03	0	0	10	0	0	0	0	0	0	0	0	0
316bbc040527ea6b57fcf7c3acb7acbe	0	0	0	0	0	0	7	0	0	0	0	0
a380466aa3364c0762cf3084ddb315e8	0	0	0	0	0	0	0	0	0	0	4	0

6146fd25378d9b1392b3b08 35d3a6098	0	0	0	0	0	0	0	0	55	0	0	0
31916d777c40d872bfbd8eaa fb8b904f	0	0	0	0	0	0	0	0	0	0	0	56
f7e5bd48077f7c5eae2c79cc7 c588d44	0	0	0	0	0	0	0	0	0	0	2	0
60389151fb05520dea34d156 c1e6fa89	0	0	0	0	0	0	0	0	0	0	0	106
60c12079efd98a8ddaca5dca d5bcf07d	0	0	0	3	0	0	0	0	0	0	0	0
4b7d3573a0ae1d60080835b e5b816d9b	0	0	0	0	0	0	22	0	0	0	0	0
3ecf704528bbe75229515ad3 7280bd03	0	0	0	0	0	0	0	2	0	0	0	0
6a95875b7ced5e2bff34af8f5 e2ad235	0	0	0	7	0	0	0	0	0	0	0	0
b0b4f8ee53a6e2ca647e177d dbed7558	0	0	0	0	0	46	0	0	0	0	0	0
43b2111cdba26b436db93bf3 01e1d74e	0	0	0	0	0	0	0	0	0	2	0	0
c8a4a39bc8597b17fec56224 1972a32e	0	0	0	0	0	0	0	0	6	0	0	0
9fcabf0a337b9e37072b85f09 b0c22b3	0	0	0	0	5	0	0	0	0	0	0	0
36e43a80d9e33f451ebe8b0f 60dec365	0	0	0	0	0	0	0	0	13	0	0	0
2f483f833049cf55c8f3f6c7ab 205b4b	0	0	0	0	0	0	4	0	0	0	0	0

14fd38f4ce87d24c2f06db226f61419a	0	0	0	0	0	0	0	0	0	0	0	10
0dc899ad837d3b3a0bd295d274191b53	0	0	0	0	0	0	0	0	3	0	0	0
01019056948cc90fe4e5d0dcbb37cc51	0	0	0	0	0	0	0	0	6	0	0	0
bb8a2d4de91608532d4e440f4ac45c60	9	0	0	0	0	0	0	0	0	0	0	0
6baa282b9d3b936c924dcb4f73d34375	0	9	0	0	0	0	0	0	0	0	0	0
8be20dc1fe6751f470241f7903f5c3f3	0	0	0	0	0	0	7	0	0	0	0	0
690adf5e6f751b83ad4c9f9d5c4ccbd5	0	0	0	0	0	0	0	10	0	0	0	0
b35124183eee150c4d99e19866bc3c63	0	0	0	0	0	0	0	0	0	0	2	0
7163c4a682af9ab25ef86bf4215cbb82	0	0	0	0	0	0	0	0	0	3	0	0
a926f698a829fc4a9f2931c833ab599f	0	0	0	0	0	0	0	4	0	0	0	0
8efd036186000d9a94dc5371f2547daa	0	0	0	0	0	0	5	0	0	0	0	0
d7770f23c8522be0546a0068e03bbd69	0	0	0	0	0	0	0	19	0	0	0	0
da53b63e3aa73efd241f5f385e8580bb	0	0	0	0	0	2	0	0	0	0	0	0
1690f6e6dd0d53124832bcf485a80a9c	0	0	2	0	0	0	0	0	0	0	0	0

c151c29931a88ccd8b1f09e2caab40b2	0	0	0	0	0	0	0	0	3	0	0	0
f440771f72b06ec3a282e67bdfd649a6	0	0	0	4	0	0	0	0	0	0	0	0
0a795e1a64226adea0f3a8954e62cc1a	0	0	0	0	0	8	0	0	0	0	0	0
fb7e1cfc1c2ba3ce6371c43340d6e025	0	0	0	0	0	12	0	0	0	0	0	0
dce31106c3ce095c1a20b2ad44d0cda6	85	0	0	0	0	0	39	0	0	0	0	0
3ed8a1bc1d5e842e203b690176c5caec	0	0	0	0	0	0	0	0	0	0	0	3
c9b4f253d95145b1f2187d07192a3bac	0	0	0	4	0	0	0	0	0	0	0	0
341e9b69a1e351c0aaaf511625e8ff19	0	0	0	0	0	0	0	0	0	2	0	0
1e507168143bdbbc8639d00c6790c75d	0	5	0	0	0	0	0	0	0	0	0	0
8f80f1cd4b88406c3c041cadbb6a7d26	0	0	0	0	7	0	0	0	0	0	0	0
31fb3460deb9c2615502548ac765bd6f	8	0	0	0	0	0	0	0	0	0	0	0
ab7bbdac335aa68092a8aeeff675704e	0	0	0	0	0	0	0	0	0	0	10	0
57a3415b283d65b9cf3160590772a271	0	0	0	0	0	0	8	0	0	0	0	0
da51b650e4252c3da631ff6c43ce28e2	0	0	4	0	0	0	0	0	0	0	0	0

7935d8ce59ae81252b02c2c836b2bfec	0	0	0	0	0	3	0	0	0	0	0	0
20285bc99d40870638683f3dc6b63a15	0	0	0	0	0	0	0	0	0	0	0	5
d52d80dabf67f88d807331e706246624	0	0	0	0	0	14	0	0	0	0	0	0
de48b66d5460c0e07a334f3487d45f1b	6	0	0	0	0	0	0	0	0	0	0	0
2b4d6836bd9281532c4f94c4f53f0db0	0	0	0	0	0	0	0	0	70	0	0	0
043815ae5591f6d7e441753d26ed8cbe	0	0	0	0	0	0	2	0	0	0	0	0
8e33745b239537fdfa1082feab6f5c91	0	0	0	0	0	3	0	0	0	0	0	0
51675e67e8cbf9066d0b1fed49628009	0	0	0	0	0	29	0	0	0	0	0	0
42aa99be450959d8fe05ed84206c8c49	0	0	0	0	0	0	0	0	0	0	0	7
93a697ea78c66c8e96b091e2c12a0f1d	0	0	0	0	0	5	0	0	0	0	0	0
7083ec57c9350563ef3c2731094f83aa	0	0	0	0	0	0	0	0	0	0	5	0
43ed03667cfc96140f0c62457dcc12fc	0	0	75	0	0	0	0	0	0	0	0	0
c1ccacaabfa71f36e07b18106108fca0	0	0	24	0	0	0	0	0	0	0	0	0
46545605108fb053526dd584d19cafad	0	0	0	0	0	0	0	0	2	0	0	0

effe6ef7f8772b64e5c3ef3f7b573caf	0	0	0	0	5	0	0	0	0	0	0	0
b7015ddd584393991700d815b84b6d2f	0	0	0	0	0	0	21	0	0	0	0	0
912bcbb241db078b74b77a5579086eac	0	0	0	0	0	23	0	0	0	0	0	0
9cceae238aba3249f1de4c21872336d0	0	0	0	0	0	0	0	0	11	0	0	0
d3efd78dd94f1b38262699df80ddbe14	0	0	0	9	0	0	0	0	0	0	0	0
dd4efd10a521ea87d671d22fbf5f82aa	0	0	0	0	0	0	0	0	0	36	0	0
5bdec1ec2dc0dbd92a0ec4f8a58fc5a7	0	0	0	0	0	0	0	0	0	2	0	0
130c4be1b87826d1fe9c34ceb17b7fd2	0	0	0	0	2	0	0	0	0	0	0	0
4faf57221768e39587b197fd3f52a156	0	0	0	0	0	0	0	0	0	0	160	0
7443dcb66668088828906b27cc863fde	0	0	0	0	0	2	0	0	0	0	0	0
bb04d06f1f02e3a9263dbb310fe00aab	0	0	0	0	0	0	0	3	0	0	0	0
45cb77be806b82fe6dd1bf0b88f65bd1	0	0	0	0	0	0	0	0	0	0	13	0
c468cfcb3f9dbce059930bee4a95ae57	0	0	0	0	25	0	0	0	0	0	0	0
15f317271bf48a7c60a5d47db813677c	0	0	0	0	0	0	0	0	0	2	0	0

134a4b70bdb088a52afacdb383c547aa	0	0	0	0	0	6	0	0	0	0	0	0
bd4feaee5a92731034905e1d23209b6f	0	0	0	0	0	0	0	0	8	0	0	0
25f2c1a2d6a66d2869175870f05edf7a	0	0	0	3	0	0	0	0	0	0	0	0
22b3326c23663ce427454bee30136c15	0	0	0	0	0	0	0	0	0	6	0	0
a4f81c03dfcca98a5bd269e4f0466499	0	0	0	0	0	0	165	0	0	0	0	0
0dc721fde35045bd747769cc5ce7d554	0	0	0	0	0	9	0	0	0	0	0	0
6ee3b298174fea7c30e6af34f0e6f1c0	0	0	0	0	0	0	0	0	0	2	0	0
6de4f075903ca90dcdd33b93b7a33346	0	0	0	2	0	0	0	0	0	0	0	0
aabc28da2e040afe2cdbc518d8beae77	0	0	0	0	0	0	0	0	0	3	0	0
faf31eca6b45c8317376ec039f3412ad	0	0	0	0	0	0	0	0	0	0	0	32
72d3e060b74b0281828d04d1bda817b2	0	22	0	0	0	0	0	0	0	0	0	0
7fe978323670bef79e35283ce08e37ab	0	0	0	0	0	0	0	0	0	0	7	0
b71c69055fccee807e68152c9c256fd4	0	0	0	0	0	0	0	0	0	3	0	0
e67ef0359d02e07adeb7187cc796fb36	0	0	0	0	0	0	0	2	0	0	0	0

fe5118579dbb13a1f1564d7e227681a7	0	0	0	0	0	0	0	0	0	3	0	0
1d7aa7f2e9f356fb8a0636c6af87b586	0	0	0	0	0	0	0	0	0	0	52	0
dbeef74cd883dc393805cc346eedbebe	0	0	0	0	0	0	0	0	0	0	14	0
031b1179840e7d886a97074cc626767c	0	0	0	0	0	0	0	0	0	0	0	311
4cc85a03be14992cb9767f96a1e3bdba	0	0	0	0	0	0	0	0	0	0	33	0
bbd3413d06547c1aa973d2ba8386ee40	0	0	0	0	0	6	0	0	0	0	0	0
d33d9b78c5c1ce029dab800adb1d74b9	0	0	0	0	0	0	0	0	0	0	0	52
bb0bcb3e9889298d506e9116a95ad13f	0	0	0	0	0	0	0	0	0	32	0	0
c75a55a355f024dc88017f26c08faa2f	0	0	0	0	0	0	0	0	366	803	0	0
ca354b004387054543135b1ed19f24da	0	2	0	0	0	0	0	0	0	0	0	0
69b8fdd3b72c1ce9666a7c2a7dd2390f	0	0	0	0	0	0	0	2	0	0	0	0
e38d40ecde80ddb6d25100ebcabe023e	0	0	0	142	0	0	0	0	0	0	0	0
e8829d16ed604cf97cf976be93134161	0	158	0	0	0	0	0	0	0	0	0	0
2661fc7c3815913edbf1df72636c08ef	0	0	0	0	0	0	0	2	0	0	0	0

d2ac13cc2397c29c3957f9f3ca89a222	0	0	0	0	0	0	0	0	0	0	0	6
144db689b1ab8907839a596e4db01a2a	0	0	0	0	0	2	0	0	0	0	0	0
16f7a4901684a626162283f6f36d528e	0	0	0	6	0	0	0	0	0	0	0	0
9956565dddf20f73b8ba3c5d51971a17	0	0	0	0	0	0	0	0	4	0	0	0
6f978a3869742f5223bb7260d77e648f	0	0	0	0	0	23	0	0	0	0	0	0
62aba4d54cdef0073c878ad6552cf3d6	0	0	0	0	0	0	0	0	0	0	15	0
51925956e303345628ccfc00c265998e	0	0	0	0	0	14	0	0	0	0	0	0
d9e06933f807f789e54c21ee46c5bcb0	0	0	0	0	0	0	0	4	0	0	0	0
810efc149201a7cecf3d93d26a3144ec	0	0	0	0	0	0	0	0	14	0	0	0
8766c9e20c8939c7667936909cd6f576	0	0	0	0	0	100	0	0	0	0	0	0
46767c103863a0cff66d837b344dfb59	0	0	0	0	0	0	0	0	0	0	3	0
455edc6748148c7e51a3bec8d3b8d321	0	0	0	0	0	0	0	0	0	8	0	0
503fc5125f612202798d6d16fcb722c1	0	0	0	0	0	5	0	0	0	0	0	0
5e0617d9dcfca98237bbb31d54cb0988	0	0	0	0	3	0	0	0	0	0	0	0

7e5ab50281ae594a80f04b9f75640f54	0	0	0	4	0	0	0	0	0	0	0	0
32c6220464b0e2da247c0e708e9eb6c5	0	0	0	0	0	0	0	0	0	2	0	0
533a48986c72392e1a60e2df6e09e677	0	0	0	0	0	0	0	0	0	0	0	5
49084513ca201d1181b76d3eeda75acf	0	0	0	0	0	0	0	24	0	0	0	0
9adb145002870bf0256ea6fc4178ca8a	0	0	0	0	0	0	0	0	0	0	2	0
99494d98897ad9116198a59b0998bd20	0	0	0	0	0	0	0	0	14	0	0	0
a787a5e96861ef6ea8e80435217c2852	0	0	0	0	0	11	0	0	0	0	0	0
258e2cec5cef07263e26665b4b3ead21	0	0	0	0	0	0	0	0	0	0	159	0
8f71bb18ef3d1a1b9c73339dd8939ab7	0	0	0	0	0	0	0	0	0	3	0	0
f7e5dd3e1adf8bc571d203a6b8dc4138	0	0	0	0	0	4	0	0	0	0	0	0
e2a4edea320f33fa1a37a62ff2f5b416	0	0	0	0	0	0	0	0	0	0	108	0
f39ada0896da045a1139f62d08360b33	0	0	0	0	0	2	0	0	0	0	0	0
31c85c67d890308c80d2eb74fe9f8622	0	0	0	0	0	0	0	4	0	0	0	0
62715ed31252555b84241cb6c982128f	0	0	0	0	4	0	0	0	0	0	0	0

21ef57a4ca129fe402504219ced3ad66	0	0	0	36	0	0	0	0	0	0	0	0
1341dffc2348281de27da41741f966e8	0	0	0	0	0	0	0	0	0	87	0	0
1e4f7158a496ca6daa8e65e47473778c	0	0	0	0	0	2	0	0	0	0	0	0
fd1ec3b1dd668b75259be2c4a91e07fd	0	0	0	0	0	0	0	0	0	0	0	14
cbcdc6044bf59e504ffd14ecf7390c0f	0	0	0	0	2	0	0	0	0	0	0	0
5142c5358954174a269470b0f138163d	0	0	0	0	0	0	0	0	0	0	0	5
9976cf679fea635f81257e4649317a80	8	0	0	0	0	0	0	0	0	0	0	0
9849eb3dbcd3d969344c75341437ce14	0	0	0	0	0	0	0	10	0	0	0	0
16d7d933a62100d01424565dfe52b044	0	0	0	0	0	0	0	0	0	0	0	3
bc305ba32a2ee2ba2d784887b95ee9bf	0	0	0	0	0	4	0	0	0	0	0	0
0860516136879620899fbaea0bbf4f70	0	0	0	0	38	0	0	0	0	0	0	0
9a0be7245d9325019e6a545b764751ba	0	0	0	6	0	0	0	0	0	0	0	0
9bc5764ed66fbe2008f63f329d7b9e03	0	0	0	0	0	0	0	0	0	25	0	0
1f3b2f573e94114c67576562f19ad3a1	0	0	0	0	0	0	0	0	0	0	16	0

bee298e786daa8c4ceb16563802c96ff	0	0	0	0	0	40	0	0	0	0	0	0
8d50a2c2f81e4e7954cc630eb83fb6f4	0	0	6	0	0	0	0	0	0	0	0	0
9b4ea5babb7ce620ec769ff2c2b585de	0	0	0	0	0	6	0	0	0	0	0	0
2e15831a2b9095b22654a140e40ec603	0	0	0	0	0	0	0	17	0	0	0	0
10a64bf5d1cbf3d9e347ac82ab6fc7b6	0	0	0	0	0	0	0	0	0	0	8	0
185ea25b5627b4691bb2f97cdccc4ac5	0	0	0	0	0	0	0	0	0	0	0	19
d3b0958cddd13f8ab0b7cb54934e41b7	0	3	0	0	0	0	0	0	0	0	0	0
797e421dd48fb8b57e73590d3129a084	0	0	0	0	0	0	0	0	0	0	13	0
33f875d7dd0cdb939482fc9a173bdea0	0	0	0	0	0	0	0	0	0	0	2	0
e851cb41bdd48e77bab48ff9ce7ae9ea	0	0	0	0	0	4	0	0	0	0	0	0
7f373d97afd65748469b080d7429f758	0	0	0	0	0	0	0	0	0	2	0	0
35943200f3abb93611514abb8200480d	0	2	0	0	0	0	0	0	0	0	0	0
1e52cb29545deb851def494dc2f443c3	5	0	0	0	0	0	0	0	0	0	0	0
ea981f661e152f45dcbc9c96d49042e7	0	0	0	0	0	64	0	0	0	0	0	0

3fa1f743d5310684ed9ca73cb964b9e5	0	0	0	0	0	0	0	0	109	0	0	0
4957569ab10c02083fc64a8b5cfa19fd	0	0	0	0	0	2	0	0	0	0	0	0
2a868fe1dd5150ecc861f3248b077e69	0	0	0	0	0	3	0	0	0	0	0	0
00f044487fd77f3691278481c03843b7	0	0	0	0	0	0	0	0	0	0	2	0
123b344530d41eecc21716690524b7aa	0	0	0	0	0	4	0	0	0	0	0	0
847f89c8bf5fccb5d363ee7b268116f5	0	0	0	0	0	0	0	0	0	52	0	0
b917bd0e9ea642ae49d002845dbbbc9c	0	0	0	0	0	0	4	0	0	0	0	0
cd53e248ee02ecebae26d7efa69dfef9	0	0	0	13	0	0	0	0	0	0	0	0
8ad0a6cb5d88fd34977337c58ab38007	0	0	0	0	0	18	0	0	0	0	0	0
bde70ad47a59db90fa413e52ee3c85f9	0	0	0	0	0	0	0	0	0	108	0	0
e15631274e71f653bd5d75b2e0722e09	0	0	0	0	0	0	0	0	0	0	0	10
1daf313bfe529f2ccca7c5497b3ffc4e	0	0	14	0	0	0	0	0	0	0	0	0
3a5f7efd12c99c09f4f36a965465cab1	0	0	0	0	0	0	0	0	0	69	0	0
8a2ecfbb19c0956ee287d18bf5accb55	0	0	0	0	0	3	0	0	0	0	0	0

2f95f763c1e412f6b12de8a7d11de40b	0	0	0	0	0	0	0	0	6	0	0	0
adc1cc0e301922c0467745a98d3a2e87	0	0	0	0	0	2	0	0	0	0	0	0
8838234d6dc1409a48351ed72c1b65bc	0	3	0	0	0	0	0	0	0	0	0	0
26b6c64ca8bcd1b273fbbb0a083d903e	0	0	0	0	0	0	0	0	0	38	0	0
0bb507222e907ef466585f4092711823	0	0	0	0	0	0	0	5	0	0	0	0
e03feaa9ca506cd33768fabee3e5a3c7	0	0	0	2	0	0	0	0	0	0	0	0
712d94d9656a0c649e0ad5898299fd09	0	0	0	0	0	0	0	0	0	0	2	0
90ea34a3065cd634ef250b774562ecd4	0	0	0	0	0	5	0	0	0	0	0	0
527811f09f3e6cd4fc2744c63fe2df56	0	0	0	0	0	0	0	0	0	3	0	0
c8419fee53a4138e779c1db4c96c3677	0	0	2	0	0	0	0	0	0	0	0	0
b21194e6ba4b2ad731e25f5b6de9a67c	0	0	0	0	0	0	0	0	0	0	5	0
5b9fa9183a28260bdea31f10db647d4b	0	0	0	0	5	0	0	0	0	0	0	0
39915a927b668c2aa60a5e190694d6c2	0	0	0	0	0	0	0	41	0	0	0	0
6b0da2de1385f4223f28d53511eb7512	0	0	77	0	0	0	0	0	0	0	0	0

7c53e3cb52b05c3ad406a5dc2755d0c8	0	0	0	0	0	0	0	0	0	0	2	0
9a2f9e076551f481114d9452e148557a	0	0	0	0	29	0	0	0	0	0	0	0
5edb3224c8a633da465bff67c7d23abc	0	3	0	0	0	0	0	0	0	0	0	0
60464dc1d9e10634ff6863a1608fd209	0	0	0	0	0	0	0	0	0	0	0	5
2f0ef6194a28937e7fb0772a915c0638	0	0	0	0	0	7	0	0	0	0	0	0
773afba2e2fcadf0043b20059a95fb81	2	0	0	0	0	0	0	0	0	0	0	0
8b387a2167858740048bb75cf8991967	0	0	7	0	0	0	0	0	0	0	0	0
fbfb2e5c51f22bf8ec00e5a640099728	0	0	0	0	0	0	0	0	0	0	94	0
084e083330156aa0627d0f1c0f01f675	0	0	0	0	0	0	0	0	0	0	0	2
e1f79ad93fd03d0f74ca492d1c5984a6	0	0	0	0	2	0	0	0	0	0	0	0
3b09f39a1a43eb879b4f778cbbbb4b5b	0	5	0	0	0	0	0	0	0	0	0	0
3af520354eed8b884ceb744ad757767a	0	0	0	0	0	0	0	0	0	4	0	0
a437cb6d1c7561345b7f9abaf021349e	0	0	0	0	0	0	2	0	0	0	0	0
d9528de4906d0fa9171f75cbe0f55c8b	0	0	0	0	0	0	0	0	0	0	3	0

b30945c6bb46bf435699b21d810da0ee	0	0	0	0	0	0	3	0	0	0	0	0
f5e3d3be4f6a58ad9e3720b27bfcc1bf	0	0	0	0	0	0	0	2	0	0	0	0
50c96858fcec3baf9c6faadeb1e6394f	0	0	0	0	0	0	0	0	0	0	0	2
a9903931e55e7cd17b8785312d9487a8	0	0	0	0	0	0	0	0	0	11	0	0
de588e80253d88c7ecfecc23981f51d2	0	15	0	0	0	0	0	0	0	0	0	0
effcbd3dba38ccc78e3db10daad0e98c	0	0	0	0	0	0	0	0	0	0	10	0
b01099b5587932a0f882ed4d88ebef4b	0	0	0	0	0	9	0	0	0	0	0	0
2e2f84026718147ca3774b71ad7bb70e	3	0	0	0	0	0	0	0	0	0	0	0
c9fb5319903b7c3ad6cf6edf1a31489d	0	0	0	0	0	0	0	0	5	0	0	0
7b58adf6d3c3292aba18849fb4565a65	0	0	0	0	0	0	0	0	0	0	8	0
f96d9ed24143fb8dc66dcd2ddc9b06b9	0	0	0	0	0	0	0	0	0	0	3	0
65d1332f84bc324d535dc9b83ab95c1a	0	0	0	0	0	13	0	0	0	0	0	0
6d6e6067c89cd008c9416354e6a1857a	0	0	0	0	127	0	0	0	0	0	0	0
735599ad1f1baa3b230e42cc2bddfe06	0	0	0	0	18	0	0	0	0	0	0	0

6570f47d3397ef51a96a9e04 1467ef1b	0	0	0	0	3	0	0	0	0	0	0	0
438da122fdee653e7241b33b c84075a9	0	0	0	0	0	0	0	0	0	0	2	0
5f2627407fd1b959e539af47f 30283ce	3	0	0	0	0	0	0	0	0	0	0	0
84711d1976cca51234222c67 a9d496cc	0	0	0	0	0	0	0	0	0	0	2	0
b42a0cf8d97a02d3e0181f66 1d14c304	113	133	377	507	179	552	0	498	0	341	0	487
0fd60f122aad2003ab992093 3c044aff	0	5	0	0	0	0	0	0	0	0	0	0
a4fb84ea7009b02ad941a1c2 e0331a11	0	0	0	0	9	0	0	0	0	0	0	0
8c7267ccaa35f58b1fbb30ffcd 9b2b19	0	0	0	0	0	17	0	0	0	0	0	0
997fbd9098a5fa18bec60587 49c4ee06	0	0	0	0	2	0	0	0	0	0	0	0
9c23e4ca8731daf7996b6ced 562ed7df	0	0	0	0	0	0	0	0	0	0	0	8
35e91c7205ac89d7b90243b 007308ebd	0	0	0	0	0	0	0	0	0	15	0	0
1cbef114d50888e62658785d 7b49f80d	0	0	0	0	0	0	0	0	0	0	37	0
4e17044c5f1c4ca8fae7e8dec a6ca9ca	0	0	2	0	0	0	0	0	0	0	0	0
6224936aca5e333a9e5be85 da45fdf59	2	0	0	0	0	0	0	0	0	0	0	0

8aa8f06f96305eb84d4cf096ba388672	0	0	0	0	0	0	2	0	0	0	0	0
583db9866679eb7e5d3b35208658c592	0	0	0	0	0	0	0	0	2	0	0	0
74bda45587dd0c3da85e9a8f4afc2da9	0	0	0	0	0	0	0	0	6	0	0	0
c61e908ffe4b9ee2d7c9e0ad52416141	0	0	0	0	0	3	0	0	0	0	0	0
24fa91e6db31ba2bb13f4a0e43f00433	0	0	0	0	0	7	0	0	0	0	0	0
3173871a0e663be6a4746499a61be81c	0	0	0	0	0	0	0	0	0	0	0	23
2b654f730f3b7d6f7d44593501a74766	0	0	0	0	0	2	0	0	0	0	0	0
f279792a18ccf9b15b32259be80fb3c2	0	0	0	5	0	0	0	0	0	0	0	0
695a61eea604e00f480d77edf2423644	0	0	0	0	0	0	0	0	0	0	0	38
0ccc3588027d2fa2072b5ccb298fed51	0	0	0	0	2	0	0	0	0	0	0	0
8b91d7ec01f777304e36c763cb304f2f	0	0	0	0	0	4	0	0	0	0	0	0
4602b37878f52bc4481ad8723fa445c5	0	0	0	0	0	0	0	0	0	90	0	0
20da0587ca61dc33194c04110e3d5aae	0	0	0	0	0	0	2	0	0	0	0	0
af44bb1f09187392c5be6d61cd0debe0	0	0	0	0	0	0	0	0	0	0	12	0

44e260cc6212465cd5558e6431c5f585	0	0	0	15	0	0	0	0	0	0	0	0
e1561b19ed2dac06d5250b0545ec2078	0	0	0	0	0	0	0	0	0	0	235	0
747d01433a3c5640ed89c3c87dbd56c6	0	0	0	0	0	34	0	0	0	0	0	0
572c5e1bb9bf37ffcc70d65a176586af	0	5	0	0	0	0	0	0	0	0	0	0
4b29a3c47a6962ef8bf008a1cb5997a7	0	0	0	0	47	0	0	0	0	0	0	0
504db23dbaeb4daed0f82f0667c2ff74	0	0	0	0	0	0	0	0	0	4	0	0
8a5d2ee1c2d534d6a3b7eb5822912cd7	2	0	0	0	0	0	0	0	0	0	0	0
33a49bf5561c514515beda47d8d40ad3	0	0	0	0	0	0	0	5	0	0	0	0
3af3719c3bf52c20579ee3d879c46859	0	0	0	3	0	0	0	0	0	0	0	0
f8ca7bebc476b4cc0b6de2484b9c1cb9	0	0	0	0	0	0	0	0	0	6	0	0
c64d2a93f232471c8a68bbc4544bd37c	11	0	0	0	0	0	0	0	0	0	0	0
dd59e330e21e30e17da8c089b87890c8	0	0	0	0	0	0	3	0	0	0	0	0
a48f3cb299e33c638ad039039f9dab4b	0	53	0	0	0	0	0	0	0	0	0	0
c351e0650294097a1b169dbdfd83ec13	0	0	0	0	0	0	0	0	0	0	3	0

97bb4221f94ae441ae7049c0e8977586	0	0	0	0	0	0	0	0	5	0	0	0
6fadf98de560aebb3d4521d2fdad2990	0	0	0	0	0	0	0	0	0	0	16	0
426a19f0338102bda87958b9ba65f7b2	0	0	4	0	0	0	0	0	0	0	0	0
8562b009915370c47dbc2492dc89fe5f	0	0	0	0	0	0	0	6	0	0	0	0
ec1f5b92c2da375bf2d3d3636b2f0eec	0	0	0	0	0	0	0	0	0	0	0	5
6d45970820e6412814be6df1cc11b0f0	0	0	0	0	0	0	71	249	0	0	0	0
94ebd01f057e1f49d1fcb29fc400c9e9	0	0	0	0	0	0	0	0	34	0	0	0
47e9ff05677437fd3473305b503c339f	0	0	0	0	0	0	0	0	0	4	0	0
9d81cf29f3735fe59905bbf8cdbc0466	0	0	0	0	0	6	0	0	0	0	0	0
3e61800a5843bc21fc95140255653d3a	0	3	0	0	0	0	0	0	0	0	0	0
363048030051450eee891848c021de77	0	0	74	0	0	0	0	0	0	0	0	0
568299e362e44ff0ea4df80b01180773	0	0	0	0	0	0	0	0	0	0	0	20
e1a3427a5e4eb21e1dc7c84aba4b033c	0	0	0	0	14	0	0	0	0	0	0	0
297a420926403be4ca47fd9080d98b71	0	0	0	13	0	0	0	0	0	0	0	0

be62b2defb46ea6f0415070167c3ac08	0	0	0	0	0	0	0	9	0	0	0	0
4806c2b0aeaf20b7d249c7fa771c05bb	0	0	0	0	0	0	0	4	0	0	0	0
2cd1269487eef831356da5292ac3abdc	0	0	0	5	0	0	0	0	0	0	0	0
25313470b1b8297aeadbcac451123324	0	0	6	0	0	0	0	0	0	0	0	0
783e4bcbcd8768d138bfe5510df7ea52	0	0	0	0	0	0	0	0	0	0	15	0
8fe494d44487872fe130aa3eec06888d	0	0	0	0	0	0	0	0	0	0	0	3
e6003fd1f6a6ec8d81924d0e0cc6812b	0	0	0	0	0	0	2	0	0	0	0	0
f7c87c5fbb46abdd6bf0a9b43a1a1c62	0	0	0	0	0	0	0	0	0	0	0	2
f020d9e1431aa71f3b48c70debc3b9a2	0	0	0	0	0	0	0	0	0	0	0	3
772067a9c7cb0831bd091ac883743fab	0	0	0	0	0	3	0	0	0	0	0	0
22beaf48a18ca4aaf2d0254c5d9127ba	0	0	0	0	0	0	0	0	0	0	0	5
f0fc681228c13af622ac18634e1fc5bd	0	0	0	0	3	0	0	0	0	0	0	0
b23c9ec7a208ef30fddb070e59182b3a	0	0	0	0	0	0	0	0	0	6	0	0
5a10710adf1c167e5e5287f1a7bd0c8f	0	0	0	0	0	0	0	0	0	0	48	0

171a4631936ac6852647ef6b1a559e40	0	0	0	0	0	0	0	0	0	2	0	0
bb66b66aa2701db4d3dffb90a664d3cb	0	0	0	0	0	0	0	0	0	0	0	3
be8b857cb30be2fbfc67cd3e1fd1fcea	0	0	0	0	0	0	0	0	6	0	0	0
3bbec988f776918abc0fe07b45dd3dcf	23	0	0	0	0	0	0	0	0	0	0	0
e68fad7783abc7c04189d3d949212bad	2	0	0	0	0	0	0	0	0	0	0	0
a8d4115b81e206dc79aab645912f08a6	2	0	0	0	0	0	0	0	0	0	0	0
415a21610c31c2e9f0a369f9b96c9f35	0	0	0	0	0	6	0	0	0	0	0	0
fe7e1ba14a5929a834509ed1fa44af5b	0	0	0	0	0	0	3	0	0	0	0	0
d504d4b98566e5e4d952675609a92aa9	0	0	0	0	0	0	0	0	0	0	2	0
a5faed475fd246ae4d74fee1698bc660	0	0	0	0	0	0	0	0	0	0	2	0
2a9a87b474b1205dfb83a61cfb030d3c	0	0	0	0	0	0	0	0	4	0	0	0
420c6dfa5924856afafd5f45a9912e04	0	0	0	0	0	0	0	0	0	10	0	0
56471d4675821805fa0c2d7e10a2a878	0	0	24	0	0	0	0	0	0	0	0	0
e178d3488b604be044dc2216a3c35558	0	0	0	0	0	0	0	0	0	3	0	0

d6dc1a3921c3be10d865031 2378744ba	0	0	0	0	0	17	0	0	0	0	0	0
fbae5ef2e5a37f0231c400b8b 36a9a22	0	0	7	0	0	0	0	0	0	0	0	0
9838f7e93a70d417cd997a4d eac24326	0	0	0	0	9	0	0	0	0	0	0	0
b25538ceae73d1d4657006b dd8ed577d	0	0	22	0	0	0	0	0	0	0	0	0
af2427bb302ee71a403c6869 7e921311	0	0	0	0	0	0	0	0	3	0	0	0
f3a71ccb262bdfe3125effca8 37b78f5	0	3	0	0	0	0	0	0	0	0	0	0
471f885e495dfbfdb1b0c4ee 80ca3721	0	0	0	0	0	0	0	0	0	6	0	0
03d1bb32ee09bffef8516d7f7 4d45989	0	0	0	0	0	0	0	0	0	0	0	3
749b3673e1610177a84ba17 edb406f17	0	0	0	0	0	0	0	5	0	0	0	0
41a2d88d9a32417b028ae1f7 61013c0d	0	0	57	0	0	0	0	0	0	0	0	0
9f87e84146983a39c1a69402 7619a30e	0	0	0	0	0	0	0	0	0	0	2	0
0ed854ac500a0adda701687 33d521551	0	0	0	0	0	0	0	17	0	0	0	0
bf7c94af20aeebe0c348bb83 daf19c50	98	0	0	0	0	0	0	0	0	0	0	0
8ec9dbed87700fc43a8d3461 32cab8c7	0	0	0	0	0	0	0	0	39	0	0	0

9ec10b9de68db395c789405 5cd299121	0	0	0	0	0	3	0	0	0	0	0	0
2e1e787b8a0fbabdd9457115 f489e8af	0	0	0	0	0	0	0	0	0	0	6	0
eee2d9de8ba353de1da1987 03f0c3413	0	0	0	0	0	0	0	0	0	0	0	47
e66147e6b750db69dda2826 28c5a8411	0	0	0	0	0	0	0	0	0	0	8	0
4b94aba4304edb765fdc4347 6df30ec0	0	0	0	0	7	0	0	0	0	0	0	0
c1b49ee11c345a50280d5dee 58785926	0	0	0	0	0	47	0	0	0	0	0	0
2e7bf90cbd1c4ec9b0f3271f6 44e212e	0	0	0	0	0	0	0	0	5	0	0	0
cde610864026148631c0bf6d 1b186d9e	0	0	0	0	0	0	0	0	0	16	0	0
099cd3957f4adeb98a5177d8 021d5352	0	0	0	0	0	0	0	0	0	25	0	0
85f62d323cf2823381556b01 92104ee4	0	0	0	0	0	0	0	0	0	0	2	0
5ce62cb0cc3a333a1d4c9e65 b66cbeeb	0	0	0	0	0	0	0	0	0	0	0	16
2fe6c466c839b65d1d5b86ba 0f6c9eaa	0	0	0	0	0	0	0	16	0	0	0	0
8fa48f49669ee836d9c1bdde 59f5e85f	0	0	0	0	0	0	0	0	0	0	4	0
c6048c6d355922885a435d2 317bec5fc	0	0	0	0	3	0	0	0	0	0	0	0

ad4b666c1bb6285435e4c8d8db70c660	0	0	0	0	0	0	0	12	0	0	0	0
29f0e45d35a76febca4121d2894f334d	0	0	0	0	0	0	0	0	0	3	0	0
b11434341dc117b25ec24ecf639b615f	3	0	0	0	0	0	0	0	0	0	0	0
603e57bab39db4fff19627a838fc57b4	0	0	0	0	0	0	0	0	0	0	10	0
c38dc9d3c3ccb0e7e1eee2c630daf945	0	0	0	5	0	0	0	0	0	0	0	0
ff7a91d7bbcd1a017d504b8911f850c9	0	0	0	0	0	0	97	0	0	0	0	0
cbaa03f32cf0a67aeda25a29697205de	0	0	0	0	0	0	0	0	0	0	9	0
e146b173744d3508fc5f2e3979b2e7c0	0	0	0	0	0	0	0	4	0	0	0	0
ba638f08154080ef8c36dbf380401bb7	0	0	0	5	0	0	0	0	0	0	0	0
9daa7180749c9e80b06b667705d04b30	0	0	0	0	0	0	0	0	2	0	0	0
a45348e075e57e9ea61a7cdd2f9b9a31	0	0	0	0	0	0	0	0	0	0	0	26
98b379242b054ec7f4c4f633173f16a0	0	0	0	0	0	3	0	0	0	0	0	0
8be2e89b58fc4bda3713ef1779ae7a37	0	0	0	37	0	0	0	0	0	0	0	0
47a180d1494ec8a11c17d8bf1be40bb0	0	0	0	0	0	0	3	0	0	0	0	0

10b4c96fc48648b2f3ebf767f26d2e5f	0	0	0	0	0	0	0	6	0	0	0	0
2a9ea1cd0626aab263f9debfba67fc9f	0	2	0	0	0	0	0	0	0	0	0	0
65735ad2e6db63b14363445f571691fa	0	0	0	0	0	0	0	0	0	11	0	0
fc71ab538ed41eb4be0ecbf5b2e366e8	0	0	0	0	0	0	0	0	0	0	0	2
c72ea14801f76fa7778c100960e299fd	0	0	0	0	0	0	0	0	0	0	11	0
14dac4422963864c0227a87bbc994ca2	0	0	0	0	0	0	3	0	0	0	0	0
b8d27f89addb33ab5c7877991542aa43	0	0	0	0	0	0	0	0	0	0	2	0
dd5791d953003594841d282dee62a00c	0	3	0	0	0	0	0	0	0	0	0	0
3af3641680ffcffe5c0b3aa693039b49	0	0	0	0	0	0	39	0	0	0	0	0
fbdad8cad1698e484a0f6452f0f4bd6e	0	0	0	0	0	0	39	0	0	0	0	0
97538352e4611ee665a7b03c4f978d5c	0	0	0	0	0	0	0	3	0	0	0	0
388e0027aee46d15395164cb4fd66f6d	0	0	0	4	0	0	0	0	0	0	0	0
11685739b92bb70984a67e0fbb1a9af9	0	0	0	0	0	0	0	0	0	6	0	0
b4d359a55a82a40cc76b42e31b071e32	0	0	0	0	0	0	0	0	0	0	2	0

674569989c430055c103004 8d098bbce	0	0	0	0	2	0	0	0	0	0	0	0
7321a6f451337489aa62a363 3e814766	0	0	0	0	0	3	0	0	0	0	0	0
7098dcad79828b589b5357af 6696e3ed	0	0	0	0	6	0	0	0	0	0	0	0
5d63a57440eeded5a0388edf d4e6f5da	0	0	14	0	0	0	0	0	0	0	0	0
a22573c2675c8d9a9afd29b4 69bfda58	0	0	0	2	0	0	0	0	0	0	0	0
bb4ca288fcdf282b7bf7745a2 358eae5	0	0	49	0	0	0	0	0	0	0	0	0
a73a051a330b10c328f3fea5 32aea80f	0	0	0	19	0	0	0	0	0	0	0	0
9b6eae7310a73c509b8a03d 426446067	0	0	0	0	0	0	0	0	0	12	0	0
ee7c235a4d44570ee89ce7a2 7e2a94f5	18	0	0	0	0	0	0	0	0	0	0	0
b53f3016f0888af2cad48218b 74bdd36	0	0	0	0	0	0	0	0	0	0	0	14
d0b7cf77a805486f34ee9ff7e 47fc0cf	97	0	0	0	0	0	0	0	0	0	0	0
dad852d18d404e2e12f9df74 a44e6e04	0	0	0	2	0	0	0	0	0	0	0	0
683e4b1c2f564bd50633e701 459bd27f	0	0	0	0	2	0	0	0	0	0	0	0
5fcfa6a2c8020bb2f97425af8 51efeda	0	0	0	7	0	0	0	0	0	0	0	0

8b7c91dfa19f9e0b5fd17ae7e11b6e49	0	0	0	0	0	0	0	0	0	0	30	0
e1bd62d5a2c7fc512fb9e48a5f326d68	0	0	0	0	2	0	0	0	0	0	0	0
722ff5120345229d90b5ae1e46725d03	0	0	0	10	0	0	0	0	0	0	0	0
1e82ca5ab54d1d43464c772be87e78b1	0	0	0	0	0	0	0	0	3	0	0	0
c0a4a5fdfbb71bd00e33bc600ff71204	0	0	48	0	0	0	0	0	0	0	0	0
50f8c2760ffd92ba40a28dfcb0731abf	0	0	5	0	0	0	0	0	0	0	0	0
98378f126d77991dbd660051838bb06c	0	0	0	0	0	0	0	0	0	0	4	0
cd5657e5342a3f79021079bcf1c0ec6b	0	0	0	0	0	0	0	0	0	0	3	0
9881a014e7ca13a6fb100e88113ffd55	0	0	0	0	0	0	0	21	0	0	0	0
7eeff9c0f4e1dfc821bf84495b68358b	0	0	2	0	0	0	0	0	0	0	0	0
8a41e76e7119ff46e8e34932430c98ef	0	0	0	0	0	0	58	0	0	0	0	0
d2215064b876979785027d3836224ff4	0	0	0	0	0	0	0	0	0	0	5	0
0c8e81efd37b8b24595f4ce1deb6cf1e	0	0	0	0	0	0	0	57	0	0	0	0
7fcbab698ef85fc08073bfe751818df3	0	8	0	0	0	0	0	0	0	0	0	0

a3c141f6ebeb1916f67750d40c206883	0	0	0	0	0	0	0	0	0	0	2	0
382ec804c8333178f14cad5019ad802c	0	0	0	0	0	3	0	0	0	0	0	0
49cfc0caeb84255cbb84678a2a8ad3d6	0	0	0	0	0	0	0	0	0	0	2	0
810cf772bd2695cf444c07323398c71f	0	0	0	0	0	0	0	0	6	0	0	0
c32f54aa4b9cbd56fcea5843f37a8551	0	0	0	0	24	0	0	0	0	0	0	0
2835fffb46fab0025b08774895b0bb09	0	0	0	0	0	0	0	0	0	0	14	0
bcf7ecbefe56c4a92715423422a59b92	0	0	0	0	0	0	0	0	0	3	0	0
4868188edcf17c2070bfbe43d6f648a6	0	0	0	0	0	4	0	0	0	0	0	0
a6c23937bd8beb7c6c267531759090f3	0	0	0	0	0	0	0	3	0	0	0	0
4b71b18dee8b9ae6dd2466cbc8672d42	363	809	286	204	726	0	0	640	0	0	1027	445
8af0ddf9f9695afc98cfd2ca295bb259	0	0	0	0	0	0	0	0	0	0	31	0
ea497fca8d386aedf9e892ecb8ce1e3b	0	0	0	0	0	122	0	0	0	0	0	0
0a98d571838b4daefd957b21a51b5147	0	0	4	0	0	0	0	0	0	0	0	0
89958ac8d4ca2a681be1030ad7ebb922	0	0	5	0	0	0	0	0	0	0	0	0

fa67087253c936848a954b51a35dd203	0	0	0	0	8	0	0	0	0	0	0	0
5f3485461e9c1123a7d924605fb18d0a	0	0	20	0	0	0	0	0	0	0	0	0
f8c75d7e45b647b1c770647d7bf371c1	0	0	0	0	0	274	0	0	0	0	0	0
5bf988ce7293cb7b999026d56c6912bb	0	0	0	0	0	0	0	0	7	0	0	0
e49d14aed6b533a35aa8e33293e91716	0	7	0	0	0	0	0	0	0	0	0	0
bd75c6e2c537d89d848b16f108eff420	0	0	0	0	0	0	0	3	0	0	0	0
768be2e502ef57353ed6c00556949c76	0	0	0	0	0	0	0	0	0	0	4	0
eb94711c6b05a4dcc115c58d897bf402	0	0	0	0	0	3	0	4	0	0	0	0
d4d3655eb3f58d1ec59bbfa3c9526212	0	0	4	0	0	0	0	0	0	0	0	0
c847ecc0a369bb8421a0610d388ad422	16	0	0	0	0	0	0	0	0	0	0	0
06251530ea084b95d569e0f06f885d0b	8	0	0	0	0	0	0	0	0	0	0	0
8adbcb952a811a453fcc496f190e3761	0	0	0	0	0	0	0	0	0	19	0	0
b603e5257c5f570405db6acaf63f47ec	0	0	0	88	0	0	0	65	0	0	0	0
689cb9971c8f4d271b87358455100c04	0	0	26	0	0	0	0	0	0	0	0	0

5212b5b5107bf729e3d962ec43d7e7ba	0	0	6	0	0	0	0	0	0	0	0	0
5f159bb20253f12221d56ed9bdc1056b	0	0	0	0	0	0	0	0	0	0	3	0
ebe5a904b42f81a51c394f579d69ec3e	0	0	0	0	3	0	0	0	0	0	0	0
2e1d8a21874e346b1668a4d0a01d599c	0	0	0	0	0	0	0	0	33	0	0	0
9c5dbb6545d8c9691a953ebfee727056	0	0	0	0	0	0	0	0	5	0	0	0
1ec3441500dce1ffd3edf2d48c9fc4ac	0	0	0	0	0	0	0	0	0	3	0	0
da8676eb40a8f37f3f724aa640941cf3	0	0	0	0	0	0	0	0	0	5	0	0
2388107e5f2ee720ebd913562541635f	0	0	0	0	0	0	0	0	0	0	17	0
df2850769fd33fb02cf17c2a06770c78	0	0	0	0	14	0	0	0	0	0	0	0
c0f7c432822305bcc25346b3f6e73477	0	0	0	0	0	0	0	8	0	0	0	0
5d99a084e0a73d7bb693c74f5503a6b1	0	0	0	0	0	0	0	0	0	0	2	0
0c20b5106b6a01eacfdaf13695552c51	0	0	0	0	0	0	0	0	0	0	0	3
739420329ffc246a47b182eea78ae798	0	0	0	0	0	0	0	0	0	5	0	0
5cf5f63465fa24ca94e3d5ec504dd5c7	0	0	6	0	0	0	0	0	0	0	0	0

b11a0f22de758e2a09c1498e c09b91ca	0	0	0	0	0	0	0	0	0	0	0	3
595a0d0df4a9c86cc45b96ca 80e356c6	0	0	0	0	0	0	0	0	0	0	2	0
68fa63ae92d8891a77d592ca 1b5041ae	0	0	0	0	0	0	0	0	0	0	0	2
b5673e783c6e064e0d22d4af 1effd722	0	0	0	0	0	0	0	0	0	243	0	0
834960df1f83841ace5825e7 a900ad78	0	0	0	0	0	0	0	0	0	0	54	0
cf0ce0fd3c491cd88cde1a83b ef62355	0	0	0	0	0	0	0	0	0	0	4	0
3bc6fe62337980f0e6257f4a3 d143212	2	0	0	0	0	0	0	0	0	0	0	0
237be8cdbd4619fca30ca133 0e6e2517	0	0	0	0	0	38	0	0	0	0	0	0
425d79fac7ffe1f4986200a8e bc4332a	4	0	0	0	0	0	0	0	0	0	0	0
c72cdf33d5b911b635edd3a9 91612540	0	0	41	0	0	0	0	0	0	0	0	0
f9e3fbe1001c1211afe219287 704ba4f	0	0	0	0	5	0	0	0	0	0	0	0
8ff22a42ecc044b520833bc59 cc01030	2	0	0	0	0	0	0	0	0	0	0	0
f84ca633086adb7aec8ac7f0c 0dbdc1a	0	14	0	0	0	0	0	0	0	0	0	0
b38663c37acb692e69cf3a7c 7b4e2053	0	0	0	0	0	0	0	0	0	0	0	3

023003f449d15d72cc02727f5b322ec7	0	2	0	0	0	0	0	0	0	0	0	0
1c45e9a466db08585794d9db3f43140a	0	0	0	2	0	0	0	0	0	0	0	0
cd7ef67dc869b52e59a178f377f19163	0	0	0	0	0	14	0	0	0	0	0	0
fcd23831e65f363d1dd734ebf21b7229	0	0	0	0	0	0	0	0	19	0	0	0
13f91a52ab2b9dad0c0e2e15391fc84f	0	0	0	0	0	0	0	0	4	0	0	0
7a1ff8203f724484d62af760942abbdb	0	0	0	0	0	0	0	0	0	0	7	0
cad9cc406b2a4fab8844e143264a77e3	0	0	0	0	0	0	0	0	85	108	0	71
733b329a5a368e1c9963e687bffa6446	0	0	0	0	0	0	0	0	0	0	3	0
ab6d9504916b154eed4c0ead7844fb9a	0	0	0	0	3	0	0	0	0	0	0	0
06cda7c3e6f7086367a5fd8ca73eb026	0	0	0	5	0	0	0	0	10	0	0	0
68c917dbcccc640783e09244ffec0796	0	0	0	3	0	0	0	0	0	0	0	0
1e4e5377a47067bbdac7b60b37d1b30d	0	0	0	0	0	0	0	0	0	0	0	2
ff67404e9210c89cac0bb645a3fca130	0	0	0	0	0	0	0	0	0	0	0	3
00b7a7a437103e295e40c11bedc679de	0	0	3	0	0	0	0	0	0	0	0	0

3605924c2174907787be841332a7e331	0	0	0	0	3	0	0	0	0	0	0	0
c447ba9a02f777035f5e9c8bbfd71d6f	0	0	0	0	0	0	0	3	0	0	0	0
360cf549b61e344b868faeefc7f9abcc	0	0	0	0	0	0	0	0	0	0	2	0
38bf32807c17334538b52707f1f52124	0	0	5	0	0	0	0	0	0	0	0	0
3df69f47cf58cb6dd9ab9319e09f4401	0	0	0	0	0	0	0	0	0	2	0	0
fab05d6967c809e41307fdbdd2a68436	0	0	0	0	0	0	29	0	0	0	0	0
bdc17bb9ce1e6c7c54656cb9115deb12	0	7	0	0	0	0	0	0	0	0	0	0
64607678c23d77bb05f02c38835bfbfb	0	0	0	20	0	0	0	0	0	0	0	0
d9aabd5ebbfa5ce1796fadb474f5120a	2	0	0	0	0	0	0	0	0	0	0	0
60814dc95267808a6c896d8603b23fed	0	0	0	0	0	0	3	0	0	0	0	0
48ea7d6655b112c82a0e201d00c106ca	0	0	0	0	0	0	0	0	8	0	0	0
a266f3af6ad9b08c892b91596c9262fe	0	0	0	5	0	0	0	0	0	0	0	0
cddd78ffd790a312ed118692e463dd6a	0	0	0	0	0	0	0	0	0	0	12	0
6d42aec6c2d467695f5b8f06be83976e	4	0	0	0	0	0	0	0	0	0	0	0

3e061623575d31b35d2bff8f3d8b274b	0	0	0	0	0	0	0	3	0	0	0	0
bda1de5c5605dbfff5757b4605e3c290	0	0	0	0	0	15	0	0	0	0	0	0
4ff497c9b7c665880b697b996789cfe7	0	0	0	5	0	0	0	0	0	0	0	0
e7759e68f8d9a0a067afd8e7620aca5b	0	0	0	0	0	6	0	0	0	0	0	0
ea56f5d3eea16ad540abe0a86689f4e4	0	3	0	0	0	0	0	0	0	0	0	0
9bb64f95f19c7a09563c6fb914434194	0	0	0	2	0	0	0	0	0	0	0	0
9bc1c1694a8f7236e20f9156e90b9fe1	0	0	0	14	0	0	0	0	0	0	0	0
f64c1a9067e376045e3ab428b8f8f476	0	0	0	0	0	0	0	3	0	0	0	0
60199c2c5eee40ca1cfca24aa9107ce5	0	0	0	0	0	0	0	0	0	0	2	0
1a2dc67e2f35239e487e0633a7d8fff4	0	0	0	0	0	0	0	0	0	3	0	0
ce4ca99b302d8b4dfa9e8fb73efee7a3	0	0	40	0	0	0	0	0	0	0	0	0
8e7cbbcdc2086d1305a9cc056cb972ee	39	0	0	0	0	0	0	0	0	0	0	0
4c58fec54b520d4a0d452e8bd756a334	0	0	0	0	2	0	0	0	0	0	0	0
695edf47961b4780aa38539c4bcc9bbd	0	0	0	0	0	0	0	0	0	0	6	0

6ca52f9a5e6c447dd8f9d81f6d80e9f4	0	5	0	0	0	0	0	0	0	0	0	0
7551b3823b06c903e53475ab3eddfc25	0	0	0	0	0	4	0	0	0	0	0	0
b74651b84b99a4ede51e377042dde33d	0	0	0	0	0	0	0	0	0	18	0	0
76e51a5a980f6097c3175177f1c22530	0	0	0	0	0	28	0	0	0	0	0	0
941d0ad3d384bb75b88cb46c8e193ac0	0	0	0	0	3	0	0	0	0	0	0	0
0f273e7c9c71dcb6eebfcb9086c49b41	0	0	0	0	0	0	0	14	0	0	0	0
bf002e07282c01defe0f5644d8cdc7c2	0	0	0	0	0	0	0	0	0	0	14	0
f6ecc44b8a84da6d96f38e45cda82f3f	0	0	0	0	0	5	0	0	0	0	0	0
c03b0ec945e7cd5447bfbb734366636f	0	0	0	0	0	0	0	0	0	164	0	0
192f26b0b478bf6ac1419f22ce8106bc	0	0	0	0	0	0	0	0	0	18	0	0
d691543fb79a004fb7572d95505b304a	0	0	0	0	0	0	4	0	0	0	0	0
6beea98f5d3f0cceb7fffd2d14dab7f7	0	0	0	0	0	4	0	0	0	0	0	0
cadd71a2b0a88ac724a91e1a2091eb53	0	0	0	0	0	4	0	0	0	0	0	0
413191d1653fa57864819e996dd94efc	0	0	0	0	0	0	0	0	0	0	40	0

0217e326050f21cdffb00d35 31abd257	0	0	10	0	0	0	0	0	0	0	0	0
34318e1a0cbac96ac0caeaec 3ddebe0b	0	0	0	0	0	0	0	0	0	0	0	8
1f393e64f98e814ed5ef2f6e9 6dff5f9	0	0	0	0	0	3	0	0	0	0	0	0
266e732b94224040dd6dab0 58cb7e76e	0	0	0	0	0	0	0	0	0	5	0	0
ed2a92ac16c8f800a51022a9 d1de8e26	0	4	0	0	0	0	0	0	0	0	0	0
1a45be60476531feb671288c 88b50b62	3	0	0	0	0	0	0	0	0	0	0	0
4cf3f7b9deb627505dfd3778c e1a841e	0	0	0	0	4	0	0	0	0	0	0	0
37dfa98bf9bb48494f1017a4 75e06efc	0	0	0	0	0	0	0	0	0	205	0	0
4758c721ec9cd0ed929b02fd 037b0caf	0	0	0	0	0	4	0	0	0	0	0	0
43c8ee16ddd3180010111d8 b1def1dcd	0	0	0	0	0	0	0	0	37	0	0	0
e4b6d28b958722482d705df bbc71fdc8	0	5	0	0	0	0	0	0	0	0	0	0
87fe9588f7cec405d1c23b0b 5106aefd	0	0	0	0	0	0	0	0	0	4	0	0
4e47cf5468ebd77ab36221b2 6ec2e048	0	0	0	0	25	0	0	0	0	0	0	0
2ff4e6d505f248927975fbbb7 9075ba1	0	0	0	0	0	0	0	0	0	8	0	0

f53e54aed04c8fc6790f02757 1d0e0e0	0	0	0	0	0	7	0	0	0	0	0	0
aa4d10ce3d04b53b1b57d09 b4174db15	0	0	0	4	0	0	0	0	0	0	0	0
eeeda9f538ea8c788a11b0ffe ea501e5	0	0	0	0	0	0	0	3	0	0	0	0
530340c6b77a24240cb5806 6dce21b77	0	0	0	0	0	0	0	0	0	0	7	0
b88cc7bba60009baddb80d8 531204d7b	0	0	0	0	0	0	0	21	0	0	0	0
cbe7cbee33ab671ca4b9f44cf f051f68	0	0	0	0	0	0	0	128	0	190	0	0
ae6d1af07cfa05b9cf4fae54d c40737c	0	0	17	0	0	0	0	0	0	0	0	0
dda90ebf33cac451fc0018d7c 89fab0c	0	0	0	0	0	0	0	0	0	0	0	7
ee34564caff303d2cb2dcfb95 01fb592	0	0	0	0	0	0	0	0	0	5	0	0
a71ff1d46f94371196f788db4 e46e5b6	0	3	0	0	0	0	0	0	0	0	0	0
13d6479e45489a339b53100 d2759b3ee	0	0	0	6	0	0	0	0	0	0	0	0
f3b4e8dad0097e12da62e2ed d15c70cc	0	0	0	0	0	0	0	0	0	3	0	0
c07c960b72bdde52118a79b 0cf4040f1	0	0	0	0	0	0	0	0	2	0	0	0
2d13f00139659cfe61ba12db a8b21818	0	0	0	0	0	0	0	0	132	0	0	0

52df939e99b27b325573e22 02f339efc	0	0	0	0	0	0	0	0	0	0	5	0
624307645e362c4dcb053c24 f832e269	0	0	0	0	0	0	0	0	0	6	0	0
bb1da4d32e3e22efd661863e 5c465954	0	0	0	0	0	0	0	0	0	0	0	29
e5d8c00ad9c1f0bce1dc5b1d af493ef2	0	0	0	0	0	0	0	0	0	0	0	10
850f1e06f4dee308c81e4c00 6d6cbaf2	0	0	0	0	0	0	0	0	0	3	0	0
b614bd3029131e82a02842b 9e8484da7	0	0	0	0	0	0	0	0	0	0	8	0
2eb6729161d26c72cf7b11ef a0c112fd	0	0	0	0	0	0	0	0	9	0	0	0
498b90d14131b6a4df996c2e 36685cac	0	0	0	0	0	0	0	0	2	0	0	0
7404e28ddf4c98fa3bb915f7a f44eb73	0	17	0	0	0	0	0	0	0	0	0	0
7dbac4de06dc98668ef55b27 f63f1c9e	0	0	39	0	0	0	0	0	0	0	0	0
df04f3eb1a4856a10d3f40f4d c4a974a	0	0	0	0	8	0	0	0	0	0	0	0
e0b0ffc0e6f17aa3c6393085c 116f9d7	0	0	0	5	0	0	0	0	0	0	0	0
c363ff45d7f42d5418ecd40c8 11029ff	0	0	3	0	0	0	0	0	0	0	0	0
d7251c8817bed20bc12797c7 73a69c60	0	0	0	0	0	0	8	0	0	0	0	0

de161a450b907ce38ac80f11cbc26b58	0	0	2	0	0	0	0	0	0	0	0	0
f56451de4cad56d2f72c9e69546a6e30	0	0	0	0	0	0	0	0	0	4	0	0
6223bbf3a0f91600960bf2b31da489ab	0	0	0	0	0	0	0	0	0	2	0	0
4611ad955f292d116d4e2a40d222bfda	0	0	4	0	0	0	0	0	0	0	0	0
254f718cf8f0709e77a2d32c298b50b2	0	0	0	0	0	0	0	0	0	0	4	0
05858a1d38e9c97b83bdba4a048ea8df	0	0	0	0	0	0	0	0	0	0	0	16
e254e4211954d71eabfb3695eaea508a	0	0	0	0	0	0	0	10	0	0	0	0
1219f0e26a91b0fced4df9e44cc70443	0	0	0	0	0	0	3	0	0	0	0	0
0b38016dad13f7c374fa42f4d77e823e	0	0	0	0	0	0	0	0	0	2	0	0
240fbaddee2ec7a99043736493dfbf19	0	0	4	0	0	0	0	0	0	0	0	0
69eae48256f9c7d66abcfbe42f87a07f	0	0	0	0	0	0	0	0	0	0	16	0
04ba5dd20dbdc905eb76acfd67b7dc14	0	0	0	0	0	0	0	0	0	0	0	3
7a40441656fb97a841f22be5d80f3912	0	0	3	0	0	0	0	0	0	0	0	0
46401f80d9e8b7d2d440f0779554f49c	0	0	0	0	0	0	0	0	0	0	3	0

1c677a348f0b0f77bd2676b0bd5907b9	0	0	0	0	0	0	0	0	0	0	0	35
24976ae4af6f339faef58031a892fa20	0	0	2	0	0	0	0	0	0	0	0	0
bad6fd627a762fc3c713cdfee1fe9025	0	0	7	0	0	0	0	0	0	0	0	0
07caee4924d75688c90ae1cf422f5792	0	0	0	0	0	9	0	0	0	0	0	0
097a38b8089f4d534d4989a846175bd5	0	0	0	0	0	0	0	7	0	0	0	0
3cdf1fdefe484ae027a051fdf460778f	0	0	0	0	0	0	2	0	0	0	0	0
227b1fa1b544c1a141b4989c5ab3c5a7	0	0	0	0	0	62	0	0	0	0	0	0
a37bff9933212f5bc532c1a55e21fb32	0	0	0	0	0	0	0	13	0	0	0	0
a96a2e08499f1087b52e75cf0ac7d06d	0	0	0	6	0	0	0	0	0	0	0	0
a3da5ccf33b6d517106928a032e7dec0	0	0	0	0	0	11	0	0	0	0	0	0
ab491675afe8f9b18088eb1ba43152fe	0	0	0	0	0	0	0	0	3	0	0	0
fd4e09893619ce96a32eba954270dd0d	0	0	0	3	0	0	0	0	0	0	0	0
cf5c8df0c37b4d5512fa2149a9cfa8a0	0	0	0	0	3	0	0	0	0	0	0	0
03547db6b0ebabe0f1ffcea32894a434	0	0	0	0	0	8	0	0	0	0	0	0

7f10d5ae599412e709dd6610ef54f685	0	0	0	0	0	0	0	0	0	5	0	0
9ce011a31d06d47645326245d2c6700f	0	17	0	0	0	0	0	0	0	0	0	0
af79dc45f3615ac826afeae246cb6e5f	0	0	0	0	0	0	0	0	0	0	0	83
0b663df74700e5ad79709e3c7216337d	0	0	0	0	0	0	0	0	0	0	7	0
636c1899b344be5f9f4add9ccf4a4c50	0	0	2	0	0	0	0	0	0	0	0	0
b8a8a43cde8517ef6be9a79bdf52839b	0	0	0	0	0	0	0	0	0	94	0	0
3772ea2b5e4c5d599fab8bde7fda7843	0	0	0	0	0	0	0	0	0	3	0	0
76bec37383288e4cdee699d8e7bf76a6	0	0	0	0	0	0	0	202	0	0	0	0
479277907034d69aecd900a9d27fdc66	0	0	0	0	0	35	0	0	0	0	0	0
5a1dc1b07b8780fe2e89f58a75ba8292	0	0	0	0	4	0	0	0	0	0	0	0
fe8181b24785a49e048689c71e166dd4	0	0	0	0	0	0	0	0	0	0	9	0
7093f8beab69b574e9471925b8b9a1ee	0	2	0	0	0	0	0	0	0	0	0	0
582307d53d23d8b2ae8c30b01334b5e2	0	7	0	0	0	0	0	0	0	0	0	0
af16ceea7169e6b584f3cd8617495a8b	0	0	0	0	0	0	40	0	0	0	0	0

0f64c5e80f26fee830a20df117f4066b	0	0	0	0	0	0	0	0	0	0	0	3
deefd858af4568631b54c723b1e9d87a	0	0	0	0	0	0	0	0	0	0	0	2
3b112c51ca81fbf5c50ea3f3ec64e33d	0	0	0	0	0	0	0	0	0	0	10	0
39df7d30033791025f95fbade04712a7	0	0	0	0	0	0	0	0	0	0	2	0
905e96c0b5d387e8d372063833bde5c8	0	0	0	0	0	0	0	0	0	0	88	0
e78c26c5d2f5e1287cb4c40c3fabd33d	0	0	0	0	0	0	0	0	0	3	0	0
db751c3506e3fc5a2d9b0e5ac2a20231	0	25	0	0	0	0	0	0	0	0	0	0
ef2c2bf61ee2b01bc6223140c6df09b2	0	0	36	0	0	0	0	0	0	0	0	0
5c52e14ac0a505b070a322a005135514	0	2	0	0	0	0	0	0	0	0	0	0
385d24b1670824222953527aef7b82b7	0	0	0	0	0	0	0	0	0	2	0	0
b6f158d77dd01751ffefac1e8f003795	0	0	0	0	0	0	0	0	0	7	0	0
e36bd3df468b1b5376795a9f91d97337	0	0	0	0	0	0	0	0	0	19	0	0
3d1664968636c2589c713b72512469f6	0	0	0	0	0	0	33	0	0	0	0	0
0b88ab8b91542250c7cf1e652106d65a	0	0	0	0	2	0	0	0	0	0	0	0

0ae3f70cd20052cb769119e894b5919c	0	0	0	0	0	0	0	0	37	0	0	0
b3a73883631e3eead9a2db9d6a0a9441	0	0	0	0	0	0	0	0	0	0	0	4
1e61810359a6ae6d41ae491a3edf9ea5	0	0	0	0	3	0	0	0	0	0	0	0
d71a1004f7d06c9ce896c20b6b41758c	0	0	0	0	0	0	0	0	0	0	0	19
b029f277dbcef469e0749dd05c1851e3	0	0	0	9	0	0	0	0	0	0	0	0
3209aeef7f0feaf50f98a5749b0587b1	0	0	0	0	0	0	0	2	0	0	0	0
c150533dc200f1f9bd94a3013b7dfac2	0	0	0	0	0	0	0	0	0	0	0	10
21981e2d828e2676a722550cee6e2fd5	0	0	0	0	0	0	0	0	0	0	2	0
a22cd8b65eddfa4c27814c43179e12c3	0	0	0	0	0	0	4	0	0	0	0	0
e57048e6103715261886200730b1f7c0	0	0	0	0	0	4	0	0	0	0	0	0
fec90864e3670abcd295cff831fed641	0	0	0	0	2	0	0	0	0	0	0	0
aa0d829cc06f90dd1f366bb476b19da1	0	0	0	0	0	0	0	0	0	0	3	0
ed3779722c25bdd69b40200582e22335	0	0	0	0	0	0	0	0	0	0	0	2
93e73dddcac5efff8de16954395bad6f	0	0	0	0	111	0	0	0	0	0	0	0

1724bda3e3b5cb90903e561e4d2f6382	0	0	0	0	0	0	0	0	0	0	0	2
7e1364164950958e4b904a864f3786e5	0	0	26	0	0	0	0	0	0	0	0	48
c3d710e864a68fd2ff91ce1f2894a1c4	0	0	0	0	0	0	0	0	0	0	6	0
359cc9b958e95c8dcf7d002d2f47f890	0	0	0	0	3	0	0	0	0	0	0	0
9f875f192fa0303d6989221b4860e1da	0	0	6	0	0	0	0	0	0	0	0	0
a37c2d0e05a1dd18785e8ca09b17a0d1	0	0	3	0	0	0	0	0	0	0	0	0
fc7080de0817b8f8df465a7ca1545e32	0	0	0	0	0	0	0	0	5	0	0	0
28177aa65e1645727e4c6d90046dfa4a	0	0	2	0	0	0	0	0	0	0	0	0
29226a3ec729469f7928a111ebbfdd00	3	0	0	0	0	0	0	0	0	0	0	0
4240cd7fd58f6962681b260b877f22d8	0	0	0	0	0	0	0	0	0	27	0	0
3ad06f160417f769d437bb07ce3be526	0	0	0	0	0	0	0	0	0	0	6	0
a14c2d04da6136cdc77c67bb7783f00a	0	0	0	0	0	0	0	0	0	32	0	0
3e5e532cdd2b3bcf90fd85d825d98fa9	0	0	0	2	0	0	0	0	0	0	0	0
649ed5452fabea266d55d90f755456b5	0	0	0	0	0	0	2	0	0	0	0	0

65162a2bbc48cbf926011d528d3f85f9	0	0	2	0	0	0	0	0	0	0	0	0
d359b2e9db789bd41e189fb752cd4b3b	0	0	0	0	5	0	0	0	0	0	0	0
81d3ea531e58831f4c544a91fca69c9e	0	0	0	0	0	0	0	0	0	0	24	0
72b2f9849cb811a9c00fdda0dce62075	0	0	0	0	0	0	0	0	0	7	0	0
3e8a5042e2df3f80d19091bb41435995	3	0	0	0	0	0	0	0	0	0	0	0
723e82bc16d88ce34ab1b3c9d03749ee	0	0	0	0	0	0	0	0	6	0	0	0
e6c95a7ad5ffe4e9f12f744269ca1938	0	0	0	51	0	0	0	0	0	0	0	0
9ebbc558d534ec7ee7e9a803a7a0f1f8	0	0	0	0	0	0	22	0	0	0	0	0
66c3d75a4f22f5ca92239e5711c7dc50	0	0	0	0	0	0	0	0	0	5	0	0
dce549d0e1f39860ddcd15f5248ae9c7	0	0	0	0	0	0	0	0	0	0	2	0
ae9c9825417ffa76efffa3576e2b0be5	0	0	0	0	0	0	0	0	0	6	0	0
7d91bd9c463827fe6bf5b16c87e03be3	3	0	0	0	0	0	0	0	0	0	0	0
59576ced81b0f54b380761da5c4fe13f	0	0	0	0	0	0	0	0	0	18	0	0
63a40fda30249be970e408248abcf4f4	0	0	0	0	0	395	0	0	437	0	0	0

edae04d2541e97f8654eabd6132e350b	0	0	0	0	0	0	0	0	0	0	0	4
0642eb5248d59c356c6bcf41bdba9854	0	0	0	0	0	0	0	0	0	0	15	0
db66d36eb214b71d2f0610cd2a2151b0	0	0	0	0	0	0	0	0	0	0	2	0
8f8c769eb704912fc2db10968655e563	0	0	0	0	0	0	0	0	0	0	0	2
a72f1798a833fa880e7402c13dd63626	0	0	0	0	0	0	15	0	0	0	0	0
75a25d23a93011e267f97a1e2db95ac9	0	0	0	0	0	0	0	0	0	0	0	2
3ccb9b127270555d6a57a781f00b7bea	0	0	0	0	0	0	0	10	0	0	0	0
24e0ec76cbafb3515d2c335808ad6f57	0	0	0	2	0	0	0	0	0	0	0	0
a252596800426b341a9bdac54aec37c9	0	0	0	0	134	0	0	0	75	0	138	0
7808e92be0f7fc1a9268c30128c49970	0	0	0	0	0	0	0	0	0	0	6	0
c7b151928cc07eccecef86d30b673a14	0	0	0	0	0	0	3	0	0	0	0	0
a5e6ebcf4aab645e7a3455c16c2ae0e5	0	0	0	0	0	0	4	0	0	0	0	0
b72ebdc1cd1100e6c738059c037d4c12	0	0	0	0	0	0	0	5	0	0	0	0
1935aa0f652e3882c0d0f9d563331887	0	0	0	0	0	0	0	0	0	3	0	0

ea594d8a7683e1b424371b814662f45f	0	0	0	0	0	0	0	0	0	3	0	0
999d94441912118f484b7a6fb12ad4be	0	0	0	0	0	0	0	5	0	0	0	0
70ddc1fedfbc8bcb965405e4a65b6cfa	0	0	0	0	0	2	0	0	0	0	0	0
9537dc5c5a79d98434e566ccd5be5080	0	0	0	0	0	0	0	14	0	0	0	0
edb9a20e366df431ecd36478a1dd0582	0	0	0	4	0	0	0	0	0	0	0	0
88911ef9aa5f2eadef5f5301ccc1ad19	0	0	0	0	0	0	0	2	0	0	0	0
8e9f6418a785098a183133bddeaa896f	0	0	0	0	0	0	0	0	0	0	0	16
5d666304923fe553c45aa918816d72d5	0	0	0	0	0	0	0	0	0	0	4	0
dc5b599e60208eccbcd6ee31e77e7866	0	0	0	0	3	0	0	0	0	0	0	0
43436c8681c6bd9bcd7adc5f03821972	0	0	0	0	0	14	0	0	0	0	0	0
35cd3a6cee99bc899d840903e95b2354	0	0	0	0	0	0	0	0	3	0	0	0
9dd45c07076d8f34044eeca4a173951f	0	0	0	0	0	0	0	0	0	0	5	0
1d4c9c074b8a72164c4f27bf715d59cb	0	0	0	0	0	0	0	0	0	0	7	0
56e925a33640b0d4d9d54064e4b96121	0	0	0	0	0	0	0	0	0	4	0	0

453b426856d5825e66df6a5ba98e8bfd	0	0	0	0	10	0	0	0	0	0	0	0
9fab772f8cd4b2634372c09b9a34d3f3	0	0	0	0	0	0	0	0	0	0	0	2
8397dd9a7411c3c02f1929f432397b11	8	0	0	0	0	0	0	0	0	0	0	0
68e9f2d2f4bd7dddd25eedddfc92c084	5	0	0	0	0	0	0	0	0	0	0	0
04a1c77006c50231efaee9ad1de335fc	0	0	0	2	0	0	0	0	0	0	0	0
905a7d8039efe96741645b0e11680b0e	0	0	0	0	0	0	6	0	0	0	0	0
95b5b1688cb0388b340d5f769014c310	0	0	0	0	0	0	0	0	0	0	0	18
0131340a7b2718146f68efb43808112a	0	0	0	0	17	0	0	0	0	0	0	0
9b310f1a56d748622edc6044bbe86951	0	0	39	0	0	0	0	0	0	0	0	0
a088b80661e97abef5ab7fad28486a8e	0	0	0	0	3	0	0	0	0	0	0	0
9b89b778ff2c8a59360493f447c0f975	0	0	0	0	0	0	0	5	0	0	0	0
054e0291697dcde23949540f4f0afe54	0	0	0	2	0	0	0	0	0	0	0	0
764fe53fb7d2df816efe011e35110e3a	0	0	0	0	0	0	0	0	0	0	5	0
edc08141aa3ca08defdb95a0cd6d1ffa	0	0	0	0	10	0	0	0	0	0	0	0

94b63546f8334291712de626c0bc3e4a	0	0	0	2	0	0	0	0	0	0	0	0
d49954b9d78c046e68f46b81fbb9fae6	0	0	0	0	0	0	0	4	0	0	0	0
e544b485219cc3a16785da5fc140f7fb	0	0	0	0	0	3	0	0	0	0	0	0
9a9e6e5edd823522945c050d9d4c3ed3	0	4	0	0	0	0	0	0	0	0	0	0
d3edc4ae9898bd7fd18130d91b85ba73	0	6	0	0	0	0	0	0	0	0	0	0
83438a219504489a74d422efc341338e	0	0	0	0	0	0	0	0	0	0	0	12
7691601bb327fd2a413ffbb181692d58	0	0	0	0	0	6	0	0	0	0	0	0
cdc90647c7f2aec820b29777b94fc8f9	0	0	0	0	0	0	0	0	7	0	0	0
996d7645e6ec1673f60cea78f76c28f2	0	0	0	0	0	2	0	0	0	0	0	0
17269ab7eeba75d29895ff9384e9547f	0	0	0	0	0	0	0	0	0	0	0	8
f7592d3b33b32d611638124280127654	2	0	0	0	0	0	0	0	0	0	0	0
0aae8bb2f54e9965ca80b6c92eae9e79	0	0	0	0	0	0	7	0	0	0	0	0
bfd4cbd52e95076a67f909beafef0c91	0	0	0	0	0	4	0	0	0	0	0	0
efd97b24705435b8529ef258525be1a5	0	0	0	0	6	0	0	0	0	0	0	0

9aaf64cfa4ff8439c4706cb38 3b0924d	0	0	0	0	0	0	0	0	0	0	5	0
ce391fb8b99f7f636dcf80fff9 d26613	0	0	0	0	0	0	0	0	0	0	0	20
975301bd3f2fdc3bc02d2b41 183ef45b	0	0	8	0	0	0	0	0	0	0	0	0
c719359d00ab4b5af947a717 d2c397aa	0	0	0	0	0	0	0	0	0	0	0	3
c61d01847a4d97ed12146f02 0f71810a	0	0	0	0	0	0	0	0	0	2	0	0
89c7189748dc20f5770666ba 6b193027	0	0	0	0	15	0	0	0	0	0	0	0
d9de8c7b9d2d4a91cb06aa2 b8c932d3f	0	0	0	0	0	0	0	0	0	2	0	0
2d3dbffa6a5bb9bab9e92f77 062298e4	0	0	0	0	0	0	0	6	0	0	0	0
bdca16d5000077e46051507 48908d4fb	0	0	0	0	0	2	0	0	0	0	0	0
0d4a2aa5b11be0d2654496c 6aeb06f04	0	0	0	0	0	4	0	0	0	0	0	0
8beff3eb4c59c0934dbae634 dd350943	0	0	5	0	0	0	0	0	0	0	0	0
e61885cd30a09e1bed6e3d6 6e878c1f4	0	0	0	0	0	0	0	0	0	7	0	0
f9389292a8f0e8a924b68090 f7e956f6	0	0	0	0	0	0	0	17	0	0	0	0
afdfe7ebe047ff58cee6ad10d 1cff294	0	0	0	0	0	0	0	0	0	0	7	0

a00981d20a513a26ba42f11d8c867f73	0	0	0	0	0	0	0	0	0	0	13	0
9c1e9d2d5b4fa10d9d2d8b0e2c7c18a3	0	0	0	0	0	0	0	7	0	0	0	0
899f897a7f381b86e9fdd2fd862659f6	0	0	0	0	0	0	0	0	0	0	2	0
cb9903f28f630bc827b2c79e9ca5d2c1	0	0	0	0	3	0	0	0	0	0	0	0
67755ea738f01896722ae0872fbb904f	0	0	0	0	0	0	0	0	0	20	0	0
5ddf7f0395576069c3a0f5e808ebf704	0	0	0	0	0	0	0	0	0	8	0	0
b33a42902fabb2acbea41d2c39893165	0	0	0	0	0	0	0	4	0	0	0	0
cf77aac3b0e41353ae86a1b6db499e32	0	0	0	0	0	0	0	0	6	0	0	0
39b92011af321a3c4b51fe2ce2db1cfa	0	0	0	0	12	0	0	0	0	0	0	0
1add197e3969985f65208515744708a2	0	0	0	0	0	0	0	0	0	6	0	0
218843b92deffd2d1c533fcb17083dbd	0	0	0	0	0	0	0	0	0	0	9	0
21178219e62f72903371ad9b08741e59	0	0	0	0	0	0	0	3	0	0	0	0
b19527b570250434ff4d2fc3877ecca6	0	0	0	0	0	0	0	0	0	0	3	0
9edabadee38f53f09779e10bb6c64d7c	0	0	0	0	0	0	0	0	33	0	0	0

9f7c7a00707abfe8aa668638 94d65996	37	0	0	0	0	0	0	0	0	0	0	0
7f231aaef432cc3050c91010d e943f80	0	0	0	2	0	0	0	0	0	0	0	0
3a55ba57a5a55c272fb4f964 aeedff3d	0	0	0	0	0	0	0	19	0	0	0	0
31f952e0c8e33b6ef0a1f9e2c e93baf0	0	0	0	0	0	0	0	0	8	0	0	0
3dfbbab0b745da489daefa64 ea0d29f6	0	0	0	0	0	0	0	0	0	0	0	9
1c2db089420f435b8392d876 e15e4c2d	0	0	0	0	0	0	0	0	0	2	0	0
f299529562db336487a07099 25dda48c	0	0	0	0	0	0	0	0	0	4	0	0
596c538b26df5e2302c1964b db709681	0	0	0	0	0	0	0	165	0	0	0	0
8468d1f74e449835c6a8d9e8 fbfb8b41	0	0	0	18	0	0	0	0	0	0	0	0
4683be0eea42a279d4ec8f91 7b771362	0	0	0	0	0	0	0	5	0	0	0	0
9939c4c1daece612d7f92529 9b365c9f	997	0	0	0	0	0	0	0	0	0	0	0
f6f6c5dc917bb942b3d5f4906 b1875c0	0	0	0	0	0	0	0	0	0	4	0	0
170593e76c1e4f4d305db72c b9032f45	0	0	0	0	0	0	67	0	0	0	0	0
15f4280251bb25d1d9499f83 be828c8b	0	0	0	0	0	0	0	0	0	0	0	4

a9c460cb273c179bc8d17dc101d3404c	0	0	0	0	0	0	0	0	0	2	0	0
6c2d91105da20995024d1c9fbb592b30	0	0	0	0	0	0	14	0	0	0	0	0
a2e6c275083f8d06c6fd99e1b4bdf754	0	0	0	0	0	0	16	0	0	0	0	0
6ab439831bce3bbdd5bccd1bc81fb2db	0	0	0	0	15	0	0	0	0	0	0	0
434e70b135cbc7ebf2327634c6539db6	0	0	0	0	0	0	0	36	0	0	0	0
7444aa456073de508f768ebe6679dae9	0	0	0	0	0	19	0	0	0	0	0	0
b65779f8e634fa95ea1a57d77b554881	0	0	0	0	0	0	0	0	0	0	0	6
85a3e8fbda7164f2b4f87523c0723c8b	0	3	0	0	0	0	0	0	0	0	0	0
d666869af7cb533d9d7079cb0cb8b986	0	28	0	0	0	0	0	0	0	0	0	0
dc81952f92faa6a414bccb7b65b9711a	0	0	0	0	0	0	0	0	0	0	2	0
766e9c9a23a2bdbaa223483ffdebc597	0	0	0	23	0	0	0	0	0	0	0	0
c36d8385f43ece6d357b6b309364032e	0	0	0	0	0	0	0	0	0	0	0	8
2f5b73e2321e60b5788bc069b12f4895	0	0	0	0	0	2	0	0	0	0	0	0
9cc3cdda649121f9269331ea253fe6bf	0	0	0	0	0	4	0	0	0	0	0	0

5471fe46fa45225b91aeec18 49142c99	0	0	0	0	0	0	0	0	7	0	0	0
fa0b69cbfa7cd776ce01d9c1d 67d0055	0	0	0	0	0	0	0	0	0	0	2	0
a78772fa088827bfe6f8b0b8 b8d318af	0	4	0	0	0	0	0	0	0	0	0	0
68ea9d6dca0b5172e04fdb58 bdbf2c97	0	0	15	0	0	0	0	0	0	0	0	0
10c4e4669833a20d22df212a b0de5ac2	0	0	0	0	0	0	0	0	0	0	66	0
5b32780b6edb27a329c631d 6b2ad515a	0	0	0	3	0	0	0	0	0	0	0	0
792e6ba7f3afa51853a0bc6c6 9b5ec5d	0	0	0	0	0	0	0	0	0	0	2	0
529c211beb833e4c3367ca55 c583411d	0	0	0	0	0	0	0	0	0	0	3	0
2985347402cebe9ad41a97fa 82538c05	0	0	0	0	90	0	0	0	0	0	131	0
dc29f65786e20b5d20adfc3b 2c527ead	0	0	0	3	0	0	0	0	0	0	0	0
4296d8ea83e88c295a6c7c6d 28130527	0	0	0	0	0	0	0	0	0	0	14	0
5845efede7eabca87316c8f2 e675c62d	0	0	0	0	0	0	0	5	0	0	0	0
2fed632cbe1a982d926df24f2 fb2f4fd	0	0	0	0	0	0	0	0	0	0	14	0
9bd6f05b7e4814fea7907712 cfe6881c	0	0	0	0	0	0	0	0	0	0	2	0

d8f33a092f18b4104e279d52 70263cd9	0	0	0	0	0	0	0	17	0	0	0	0
876cf0ba33cbc275dcc6d195 7cca8d72	0	0	0	0	0	0	0	0	0	0	2	0
869a41e8311b7afbf2504f59 67854065	0	0	0	0	0	0	0	2	0	0	0	0
17e5744f2f38e82cfb81c2e1d af29a95	0	0	0	0	3	0	0	0	0	0	0	0
1aac3f9df0e8d93f6d9119fd0 59877a3	0	0	0	0	0	0	0	0	0	3	0	0
24d4c5dbe4365ba8d107d66 a4b1c82ed	0	0	0	0	0	0	0	0	4	0	0	0
8690e2ad76ce31d7721d138 72a1359ec	0	0	0	3	0	0	0	0	0	0	0	0
34a36433b1c041e0ccc1ae64 22ad9f78	0	0	0	0	32	0	0	0	0	0	0	0
d2715eae83b23283586965d 5f3f8d27d	0	0	0	0	0	0	0	0	0	0	0	186
c0028111b5d6aa996c523e33 ac2e1e7b	0	0	0	0	0	0	0	3	0	0	0	0
6066f0d3824695371ce56cde 8c72f412	0	0	2	0	0	0	0	0	0	0	0	0
94e90721119c67c00b69a467 1eb680ce	0	0	0	0	4	0	0	0	0	0	0	0
7c3e094c4e48435ef628fc7c8 afa079a	0	0	0	0	0	0	0	0	0	20	0	0
f33ff4c2c9784ce8fdba32de9 c71b171	0	0	0	0	0	3	0	0	0	0	0	0

96da08e6a219225195a988d7949a997e	23	0	0	0	0	0	0	0	0	0	0	0
6634db5452c379a09b2ed756f6fa7c4c	0	0	0	0	0	0	0	0	0	0	42	0
2e139b34a369eeeed4903f64bd802935	0	0	0	0	0	12	0	0	0	0	0	0
68aba289be1d7300db858d848d483af7	0	0	0	0	0	51	0	0	0	0	0	0
0703b27c7367b05b05aa4d2e414d4d85	0	0	0	0	0	0	0	20	0	0	0	0
7f2eb12b8468e0737bf733014db925bf	0	0	0	0	0	0	0	0	4	0	0	0
410a4ce00734fb09c18b4460b585a8ec	0	0	0	0	0	0	0	0	0	0	0	34
d0d113d7cdddcf73d0bd5678c6e95a8c	0	0	0	0	0	0	0	0	0	0	0	6
98ab37ebffbb410cd93fa145b8ae1046	0	0	0	0	0	0	0	0	0	0	2	0
373f545a60093815bcf2e9b1570590da	0	0	18	0	0	0	0	0	0	0	0	0
a85173e923ad07c16d3e1811ab277651	0	0	0	2	0	0	0	0	0	0	0	0
a5182c876f36600236a7924dc023a0ef	0	0	0	0	0	0	0	4	0	0	0	0
8e5c730b1140b74242b53499030130b3	0	0	0	0	0	0	0	0	61	0	0	0
048f5025e6cb7decc6319d201b0bfcc9	0	0	0	0	0	2	0	0	0	0	0	0

6efcc55ac2958720862ee3d42852fe3d	0	0	0	0	0	0	0	0	0	0	0	7
c7125ba4195c07bcc3a9f72be71a0f45	0	0	20	0	0	0	0	0	0	0	0	0
a14b422bae354c674bcf707ef369bc2c	0	0	0	35	0	0	0	0	0	0	0	0
93583a60ab2c258ca9ff4c3e4eea65ae	0	0	0	0	0	0	3	0	0	0	0	0
953aaa097e14716ac175567035173fd7	0	0	0	29	0	0	0	0	0	0	0	0
4340718fb91e29aa1e3747baa6157bd6	0	0	0	0	0	0	0	8	0	0	0	0
9fafae4978a18f4f7cb99a371ef3f9b2	0	2	0	0	0	0	0	0	0	0	0	0
b339f5dc6e0dc0861e18cb606895ed1a	0	0	0	0	0	0	0	0	5	0	0	0
136c723cd5c9d94dfc5d1a730f8994f7	0	0	0	0	0	0	0	0	0	0	39	0
8f93d59df5c19af5c76fc220ef9513d6	0	0	0	0	18	0	0	0	0	0	0	0
86f4c9a4bd97a0ba9f67d546e2d5aee2	0	0	0	0	0	0	0	0	0	0	0	4
e81d8f7a571ca997a4eede9d230c90dd	0	0	0	0	5	0	0	0	0	0	0	0
aed40e241efcc808799c0c0a1b3cccaf	2	0	0	0	0	0	0	0	0	0	0	0
65c0fc5859817c96583334defac6175b	0	0	0	0	0	0	0	0	0	0	69	0

190f8031447a666cb9e4c14aff996866	0	0	0	0	0	0	0	0	0	2	0	0
6c6720a7e478735c554ffcafa9322acf	0	0	0	0	0	0	0	0	0	2	0	0
f0dd5947ea639812891ea0d8c80f2f97	0	0	0	0	0	0	0	0	0	0	0	2
3cb31218af902d5acb33b36fe17c3962	0	0	0	0	0	0	0	0	4	0	0	0
8c60453abe481f6859a34553dd8effa1	0	0	0	0	0	0	0	0	0	0	2	0
19e7325e6fe822da07e5775ac54d30d9	0	0	0	0	0	0	0	0	2	0	0	0
834cada3be00b67750fecb631de61903	0	0	0	0	0	0	0	0	0	0	4	0
7f099395371ff7ea8a0f980609da29d0	0	0	0	0	0	0	0	4	0	0	0	0
5697cf94494cd33e6dc6ae4c7b7ab39c	0	14	0	0	0	0	0	0	0	0	0	0
c4731ab7e056bb419eba4844863b155b	0	0	9	0	0	0	0	0	0	0	0	0
0b86ab3fc678487f67e10728ef751686	0	0	0	0	0	0	0	0	0	61	0	0
b2e041fa31b6026387eae5c60d70ac53	0	0	0	0	50	0	0	0	0	0	0	0
eaa58f80fd0663b9dc8e15f47485b304	0	0	0	4	0	0	0	0	0	0	0	0
0bd48ed1ae3770d060156f2d2bbbe51f	0	48	0	0	0	0	0	0	0	0	0	0

130faac4c09325864f53129ec52b90e4	4	0	0	0	0	0	0	0	0	0	0	0
00705d68d3c988652b4b95012027cdc8	0	0	0	0	0	0	0	0	0	0	6	0
554f87054654bc87965affd414ccab12	0	0	0	0	0	0	0	0	37	0	0	0
7d5a1d751e8818d23b3e2dfb73f8415e	0	0	0	0	0	0	0	0	0	0	0	4
de25f1ca24998a14a3d037962622d4fe	11	0	0	0	0	0	0	0	0	0	0	0
4df52200a98909eeda8e9d0f0b9f56c7	0	0	0	0	0	0	0	0	0	0	0	17
a5785ce08eb5a7f0692f1aaf9ced5cc7	0	0	0	0	3	0	0	0	0	0	0	0
e14c2d8a893324ad164cbd08f2d457c7	0	0	5	0	0	0	0	0	0	0	0	0
857e0ce39e169c3d2c49c2e7371d8ca4	2	0	0	0	0	0	0	0	0	0	0	0
77b38e9d7b1543a331504e93cd2f11b6	0	0	0	0	7	0	0	0	0	0	0	0
9b19c7907491aeccdf98560813be4198	0	0	0	0	0	0	0	0	0	0	3	0
6d71d3ff042a9b7697bf8342a1520c20	0	0	17	0	0	0	0	0	0	0	0	0
14da5328c2f955104214059df1e27e98	0	0	5	0	0	0	0	0	0	0	0	0
63100046b1c6a35f6e75d5f5639a9954	0	0	0	0	0	0	0	0	0	0	5	0

5e7f0469d40c8c60c4b63bbaa0f15ba6	0	0	0	0	0	0	0	0	0	0	0	2
951fa50a8044ea2cbb92cba8427f46ee	0	0	24	0	0	0	0	0	0	0	0	0
d0844a6fe729d4e18df323e3695341bf	0	0	0	0	0	0	0	0	0	0	0	4
b27f24708559c4ab060b40750bde4c85	0	0	5	0	0	0	0	0	0	0	0	0
f1bd69b0e2d283e64c339a1d1a775131	0	0	0	0	0	0	0	0	26	0	0	0
4812d0758f6e96ae68cb5638211092ae	0	0	0	0	0	0	0	0	0	0	0	4
ff64493e271a6ba7acbd25e4ea7b40a9	0	0	0	0	0	0	10	0	0	0	0	0
55f9f3724354faf7112e3a8d2818c4c7	0	0	0	42	0	0	0	0	0	0	0	0
a89a4c635b0eee332c1075f0d86c8936	0	0	0	0	0	0	7	0	0	0	0	0
84f50a934797e0abd14d6c1216c720bc	0	0	0	0	0	0	0	6	0	0	0	0
1bb2e33a185ec396befaa716263800cb	0	0	0	0	0	0	0	7	0	0	0	0
101778a04e2fc0837265a0475e7c9c99	0	0	0	0	0	0	0	6	0	0	0	0
8031023fc23c788af163fdccb2ea7625	0	0	0	0	6	0	0	0	0	0	0	0
b3221eb5cd7b709434e397d43cc11637	0	0	0	0	0	0	0	0	0	13	0	0

bbe56399ffbac603d0f21d80471fa16f	0	0	0	0	0	0	0	0	0	0	0	5
051e4d48f217d707e0765af466e6a6a8	0	0	0	0	3	0	0	0	0	0	0	0
af538a914834e083caed800b18aaa66a	0	0	0	0	2	0	0	0	0	0	0	0
d0674f025ef580b8c5fa2bf63abff811	0	0	0	0	0	0	0	0	0	0	15	0
440e70c9c98c91e68055c19e78922cdc	0	0	0	0	0	0	0	0	0	0	18	0
9ebb6ac7b7c638cdff7f60526318898b	0	0	0	0	0	0	0	0	0	0	3	0
6309172be7a0eee9e82157f9a98b9173	0	0	0	0	0	77	0	0	0	0	0	0
ecffdc2240c56265741d73c9c5f8d175	0	0	0	0	0	68	0	0	0	0	167	0
c2c5f570544080e37d2db19fc866219a	0	0	0	0	0	0	0	3	0	0	0	0
fb307e786fc41d248e61be27a716ed6c	0	0	0	0	0	0	0	0	0	0	5	0
df02d3255ced451c7b47335d6c1c9694	0	0	0	0	2	0	0	0	0	0	0	0
1e1b0732259633686b7b427c6c96fa01	0	0	0	0	0	0	0	0	0	0	122	27
459cd5a92b546348f92736afab977646	0	0	0	0	3	0	0	0	0	0	0	0
aee194783ad38f3d21e7939a68a57473	0	0	0	0	0	0	0	0	25	0	0	0

fca9d5005d38676b995ffc7f362affac	0	0	7	0	0	0	0	0	0	0	0	0
1ce0de1141d3a5cdf98130eca897b913	0	0	171	0	0	0	0	0	0	0	0	0
433f9fee1fd23a80715404923834d2be	0	0	0	0	0	0	0	0	0	4	0	0
7e834851ed1a4dbe3401d72476fcf4d6	0	0	0	0	0	0	0	0	0	0	0	3
3c146a3708af4d222bd1069e919c8a81	0	0	0	59	0	0	0	0	0	0	0	0
d0bfe8fe9a749f4b844d5fd7b389aa14	0	0	0	0	0	0	0	0	0	5	0	0
81e29103610f52bfa3301f676607a7f6	0	0	0	0	0	0	0	4	0	0	0	0
7730546dcd487abbd7b83535b1f0b321	0	0	0	0	0	0	0	0	0	0	7	0
2713a21827cff87d68f56e4c527f673a	0	0	0	0	0	0	0	0	0	28	0	0
43f0b777a559cba88cab1c1d069187d3	0	0	0	0	0	0	0	0	0	0	0	3
5470611f61e92386670b81d9e8caf966	0	0	0	0	0	0	0	0	0	0	3	0
f609c2e869cd4f0f00d69eda324a3b30	0	0	1790	0	0	0	0	0	0	0	0	0
4be428ccaaa3f149c7b4def5ef112498	0	0	0	0	0	0	0	2	0	0	0	0
2e00713223df72f81db10b9cf055ce38	0	0	0	0	3	0	0	0	0	0	0	0

15481e59034e1b924c3db3199b262647	0	0	0	0	0	0	0	0	0	8	0	0
bc89928a5c3a5d0fd1fbfda99079aab2	0	0	0	0	0	0	0	0	0	12	0	0
6fce26e206307ca3dcaed3a4fba389e2	0	0	0	0	0	0	0	0	0	0	0	3
490f8d001bc942f95978b385be372835	0	0	0	0	0	0	0	0	0	3	0	0
ebcf4dd3e3c216477891af2c927cd012	0	0	0	0	0	0	0	0	0	0	0	5
75788761846bdf7bb25c64f169975a2e	0	0	0	0	0	0	0	0	0	0	4	0
ee1d52488e4a74d4c586bdd34c5ba222	0	0	0	0	0	0	0	0	0	0	3	0
0a7dc91e7fba8cff373d0217794db8a1	0	0	3	0	0	0	0	0	0	0	0	0
b948c4ffdb49282834bac2288f202455	6	0	0	0	0	0	0	0	0	0	0	0
8a6c9033398a06a720126c6c487fedf5	0	0	0	0	0	3	0	0	0	0	0	0
73809a2d94417593810e04eab679e9c8	0	0	0	0	0	0	0	0	0	0	0	14
7cd49dfd6a0f2ed90cb03a9cb7f90132	0	0	0	0	0	12	0	0	0	0	0	0
9a28a3abb96bdffff6bc4fb6c8a0df29	0	0	0	0	0	2	0	0	0	0	0	0
4267237c62fb5d898d14e9e7a5c757c8	0	0	0	0	0	0	0	0	0	0	2	0

f6025fcb387b319e7d457af2ba4c1cba	0	0	2	0	0	0	0	0	0	0	0	0
4e9784ec929544e79211630f48b5d2f6	0	0	17	0	0	0	0	0	0	0	0	0
c211be4c81ef6af1b354ba4d0d16be95	0	0	0	0	0	0	0	0	0	0	0	42
6cb576045365b29c8e5acac2f8da64d4	0	0	0	0	0	0	0	0	0	0	0	6
75123a0e7079a46431dd0dad53940a8a	0	0	0	0	0	0	0	0	16	0	0	0
d7440b5e921ce3ddbec0b92d385c668c	0	2	0	0	0	0	0	0	0	0	0	0
d15d54a200725c797b9952e49cafa0ec	0	0	0	0	0	0	0	0	0	0	0	9
0cd50a0caf1fa8983999b78ba3bb0ea7	0	0	0	0	2	0	0	0	0	0	0	0
5f281cc4f9f87ec8351ab3feb027e58e	0	0	0	0	0	0	0	0	0	5	0	0
4c5b7ee3cff7cf52af1701b4ce2dc384	0	0	0	0	0	0	0	6	0	0	0	0
15956d30b30748831baa73aa52cacb99	0	0	0	0	0	0	0	85	0	0	0	0
be6986b845295221151c6a5438064755	0	0	82	0	0	0	0	0	0	0	0	0
be1d98355917ee04da582dec5230e211	0	0	0	0	0	0	0	0	15	0	0	0
18db0fd0186217a93d1eeb71bdf636d3	0	0	0	2	0	0	0	0	0	0	0	0

4439c7914a481997487df081458c5027	0	0	0	3	0	0	0	0	0	0	0	0
664613a5fdf8098c47d776ca50ffe16c	0	0	0	0	0	0	0	0	0	0	0	6
a56c139274795096f1272727eed408a1	0	0	0	0	2	0	0	0	0	0	0	0
a024061b7ee564576a2ae0f0fcfc651f	0	0	0	33	0	0	0	0	0	0	0	0
80515039960a21e24c852b859bb24de3	0	0	0	0	0	0	0	0	14	0	0	0
dd9bfdcb6394a324414867bd84835a0f	0	0	0	0	0	0	0	0	0	0	0	13
06e8c0ec40ff41ec60a112a2382b89fe	0	0	0	0	3	0	0	0	0	0	0	0
8ec05c2f344e16716e79ee91dcd3ca06	0	0	0	0	0	4	0	0	0	0	0	0
cf2f75cb3ae58bce25c7298ece01de36	0	0	0	0	0	0	0	0	0	0	3	0
cd559f683bc7d2a5f31ad1a75bfd15ac	0	0	0	0	0	0	0	15	0	0	0	0
556bdba01c9a7d4551c59c816755d0be	0	0	0	0	0	0	0	0	0	4	0	0
cfbfbc511e82fc0e1116dc7354b8a7f7	0	0	0	0	0	0	0	0	0	0	6	0
741223f6075677a2703363215ed41346	0	0	0	0	11	0	0	0	0	0	0	0
cc72b86813ce468f98d39b2029c2e67c	0	0	0	0	0	0	0	3	0	0	0	0

eb6b3ac864292bf26b96432f4ee2d17f	0	23	0	0	0	0	0	0	0	0	0	0
3ed550f0759b7d402e7b151df690970e	0	0	0	0	0	0	0	0	0	0	0	2
80d3928b7ac751d3b98c6a0bcf889133	0	0	0	0	0	23	0	0	0	0	0	0
0ec625710c294278a5220678a7cd3c2f	0	0	0	0	0	0	0	0	0	0	0	11
4fcbecf5e73afc401aa8662b5a3f6a72	0	0	0	0	0	0	3	0	0	0	0	0
9385039308c0e4baf1806b3db5db7955	0	0	0	0	0	0	0	0	0	0	0	113
2fb4a15acebfade340a5cbbe74cb3de5	0	0	0	2	0	0	0	0	0	0	0	0
431fcb4e8595baff3794a56d8f0fa3ce	2	0	0	0	0	0	0	0	0	0	0	0
1d3f0f800f329a879f112051bdeb1833	0	0	0	0	2	0	0	0	0	0	0	0
5300b61bf3a23965b7629cb242fa6f44	0	0	0	0	0	0	0	0	3	0	0	0
2554bd2933259368993f77bc3eedfccd	0	0	0	0	0	0	0	5	0	0	0	0
353fae31de33233aa39ceec2936ded24	0	0	0	0	0	8	0	0	0	0	0	0
d932c02592f91275d833857d18a85be3	0	0	0	0	0	0	0	0	13	0	0	0
557cf7f373beff6a034b851daef704ff	0	0	10	0	0	0	0	0	0	0	0	0

83a7eb0e2471a19eaf1967f79845f651	0	0	0	0	0	0	0	0	0	0	13	0
4080bc0a7db1c3960a7c33bb9db19594	0	0	0	5	0	0	0	0	0	0	0	0
876c68359c7edfa26621b8f370481894	0	0	0	0	0	0	0	0	0	0	0	10
378e78f5d1b7d770b37c3409551e34e9	0	0	0	0	0	0	0	0	5	0	0	0
1276c56f2e8f82a0b38e7634a39ae24c	0	0	0	0	0	0	0	0	0	0	0	5
b06c54ed631fbf15d3e7b288d8d57e97	0	0	0	0	0	0	0	0	0	0	0	8
bec1c6ce5de567b15739f56aedea68b1	0	0	0	0	0	0	0	0	0	0	2	0
433fefd330ee856ea378610ff0a327b5	0	0	0	0	0	0	0	0	0	0	41	0
4f26aa580a6c3459ee486a59d3e160da	0	0	0	0	0	0	0	0	0	0	10	0
42113b7eb00dfa59df66995b10b7dc82	0	0	0	0	0	0	0	3	0	0	0	0
634ac843f0f2cb7ff145c10b6bac5d2f	0	0	0	0	2	0	0	0	0	0	0	0
aacb7d61b7bb9685f6beec85e1740bde	0	0	0	0	0	0	0	8	0	0	0	0
a8783964e16ebfd558c7afbb3bd20bf0	0	0	0	20	0	0	0	0	0	0	0	0
ba10fe0fd0804d6010f48dda237bf299	0	0	0	0	0	0	0	9	0	0	0	0

3cb31d74445df56105facff331a37b59	0	0	0	0	0	0	0	0	0	5	0	0
616c48b2cc9b5a63ef8de463d41c7319	0	0	0	0	0	0	0	0	0	0	0	10
a3d24b723999935a381936c3607fc8ee	0	0	0	0	0	2	0	0	0	0	0	0
9390e6a4a181834d225c0ffb38c23d12	0	0	0	0	0	0	0	0	0	0	14	0
0fe9418852c2443a167533a0cf3acc83	0	0	0	0	0	0	0	0	0	0	3	0
2462885e77fc072e97001677e9a740f2	0	0	0	0	0	0	0	0	0	0	0	15
2c5364fac71f6e56455edf4dc7bef35f	0	0	0	5	0	0	0	0	0	0	0	0
2d7f11b8c618bd1a1d755e47324858a6	0	2	0	0	0	0	0	0	0	0	0	0
1b67e5f22d13e96484ee93e963791ae0	0	0	0	2	0	0	0	0	0	0	0	0
8827c3ba9edd91ce52f33a5a47045ac3	0	0	0	0	0	0	0	0	0	0	0	9
d5e1e793be94c2f095232d09fa7ab7a1	0	0	0	0	0	0	0	3	0	0	0	0
fe65696342029435ce17a3fed964676b	7	0	0	0	0	0	0	0	0	0	0	0
14e425cc1909ae392bb5713a00e8fb6b	0	0	0	0	0	0	0	0	0	0	11	0
41e34229171dd21b171189b04b89d070	0	0	0	0	0	0	2	0	0	0	0	0

c099211e734080d6d0930b65ab1d9041	0	0	0	0	0	0	9	0	0	0	0	0
1da4a1efbac53b3677030e6c2acb43b9	0	0	0	0	0	0	0	0	0	4	0	0
05f027f446c86a33a5c2c1a504dc9955	0	41	0	0	0	0	0	0	0	0	0	0
812b4ec3de61ca2d848f47d2ffcf37ba	0	0	0	0	0	0	0	0	0	0	7	0
ffad7f880a4fc279b6ea81c05cda3537	0	4	0	0	0	0	0	0	0	0	0	0
68c5902011a63eab39fb1fc1bfccbd3d	0	0	0	0	0	0	0	0	3	0	0	0
d2345d8adff4530a269592ba1f3be76f	0	0	4	0	0	0	0	0	0	0	0	0
2c5772cd5a6ae2453bc35bcc3a8996a0	0	0	0	0	0	0	0	0	8	0	0	0
78a12f0e10c53dc3ac72edee913f9fdf	0	0	0	0	0	0	0	0	0	7	0	0
c93bd9fbb6701db84e231b07e2c200f6	0	0	0	0	0	0	0	2	0	0	0	0
d96d35a81727e41ce30fb15047c4e1cb	0	0	0	0	0	0	0	0	0	3	0	0
41eb0d60bb4a5b2f2a92e55b539c957f	0	8	0	0	0	0	0	0	0	0	0	0
39a95a999dd0af3e1885395a10b0ae49	0	0	0	0	0	0	0	0	0	16	0	0
51a3b5b82b88a64647e6c90c02cbc00c	0	0	0	0	0	0	0	0	0	0	8	0

fa93ecf47e51d940aebe5396a258a9ae	0	0	0	0	0	0	0	0	0	4	0	0
20d57bde16783b4136461acaae5603a6	0	10	0	0	0	0	0	0	0	0	0	0
a2a79965e5f63ba372dfe5a181bce094	0	0	0	0	0	0	0	0	0	3	0	0
19b747076f65e4343f3ea10530d7440c	3	0	0	0	0	0	0	0	0	0	0	0
dde432f823c5f6f57a5d1bfb333dcd89	0	0	0	0	84	0	0	0	0	0	0	0
93289c46246dee23e0d9919bf5119957	0	0	0	0	0	0	0	35	0	0	0	0
a54aa92c67992429f385c57473ac764d	0	0	0	0	0	0	0	0	0	0	4	0
a9e642c1c4872fbfc1f2eb6f184102d3	0	0	0	2	0	0	0	0	0	0	0	0
bd6bf1ee8054b6bf8b2b855b0d2f9909	0	0	0	0	0	5	0	0	0	0	0	0
ca120f2f783ac95785b90c9233051f84	0	0	0	0	0	0	0	0	0	0	5	0
f1a0dc346c77626aa13850a8e3c645b0	0	0	0	0	70	0	0	0	0	0	0	0
c9e9d14b574d31824e7b3d605d3b964e	0	0	0	0	0	0	0	0	0	0	5	0
61d7f07ca01db3d4f44555ba9f05f617	0	0	0	0	0	0	0	0	0	6	0	0
245777b6bbf03cce357deea6bf4a3182	0	0	0	0	0	0	0	0	0	0	0	4

4e806747acfaa3d26265220eac3b6bc1	7	0	18	0	0	0	0	0	0	0	27	0
30175696d40089274ac6561e4987c2d1	0	0	0	0	0	0	0	0	0	6	0	0
02a1df8bea89941b552dbc8b6fc990b8	0	0	0	0	345	0	0	0	0	0	220	0
26961340a993e7cfa11c2f140bf7200f	0	0	0	0	0	0	0	10	0	0	0	0
f2fa9d8ee7c481cc2a4b7b3a41e5d2c1	4	0	0	0	0	0	0	0	0	0	0	0
0b8630fd8faf9d27f480c00f45eaef7a	0	0	0	0	0	0	0	7	0	0	0	0
497f3f2df4badf6815ee496d9d0517e3	0	0	0	0	0	0	0	0	0	0	289	0
a3b00300d25da690a5f270dcc651fa36	0	0	0	0	0	0	0	0	0	0	3	0
9f5fdd5b9d4455f9cd757f5e483d9e64	0	0	4	0	0	0	0	0	0	0	0	0
f8fd22397ec2993d0e83e4ffb557b985	0	0	0	7	0	0	0	0	0	0	0	0
6944c34d1c9dade4de0186b7745e08d2	0	0	0	0	0	0	0	0	0	6	0	0
2ceb6ecb905353b99fd4d2acc7ff6f29	0	0	0	0	2	0	0	0	0	0	0	0
3ff99f470b0df7ca65cac1f54eb6509e	0	0	0	2	0	0	0	0	0	0	0	0
916303a9a363505dc5f8db2cfb843d50	0	0	6	0	0	0	0	0	0	0	0	0

dd78a4b51ca09a506750b4378a4474a7	0	0	0	5	0	0	0	0	0	0	0	0
da611f83d998230def05f0d570a297fa	9	0	0	0	0	0	0	0	0	0	0	0
8566dd130c7d8d762e26b19b74d0d981	0	0	0	0	0	0	3	0	0	0	0	0
fba0ae474b82b343d829eed5e7d5c2e7	0	0	0	0	0	0	0	0	0	3	0	0
8e5836457190c920f832449d66664c4a	0	0	0	0	3	0	0	0	0	0	0	0
41f098d89cd76293967cf23d78b033f5	0	0	0	3	0	0	0	0	0	0	0	0
4efd2a61ee0d6944865775295214b90d	0	0	0	0	9	0	0	0	0	0	0	0
7d1274539ab0c4aff3ee7f4de23be008	0	0	0	0	0	0	0	0	0	2	0	0
098b605a8923ccb8018898bd3dd7fbd2	0	0	0	0	0	11	0	0	0	0	0	0
542cc53c8751212357d8374c0a5c4ead	0	0	0	0	0	0	0	0	0	0	7	0
2569e9908c521660a3dbb8f46d175cd2	0	10	0	0	0	0	0	0	0	0	0	0
e56f78fdadc05f041aa7cc32e3e68187	0	0	0	0	0	0	2	0	0	0	0	0
d073ff0c2975f570f1634816fe04d679	0	0	0	0	0	0	0	0	0	0	10	0
2d897f0045939e23b42b969f9e7f5cfb	0	0	38	0	0	0	0	0	0	0	0	0

49850da17ffae3c96d7c5b67867b773d	5	0	0	0	0	0	0	0	0	0	0	0
bd6f60886e5e7b5dde1fb71029a74706	0	0	0	0	0	0	0	0	0	0	12	0
cfaab901824c26f3dc03f14f0d41591c	0	0	0	0	0	0	2	0	0	0	0	0
556d73c7eea3705ede6389d01475f23d	0	0	0	0	0	0	0	111	0	0	0	0
3ca8a897788e438f3c7b4e86fb321995	0	0	0	0	0	0	0	0	0	0	5	0
00b5f2fdf71ca88ce2ec0b60d8be1aa1	0	0	0	0	0	0	0	0	0	0	43	0
96b53d90b34e97941a8d5936b37b5819	2	0	0	0	0	0	0	0	0	0	0	0
b05d530d1eac2715425c5a5d604e4a75	0	0	0	0	0	0	0	0	2	0	0	0
2557240157ed8ec7f812536040ea72c0	0	0	0	0	0	0	5	0	0	0	0	0
d1380c486bd5d83e9b3d12bc9c088d3f	0	5	0	0	0	0	0	0	0	0	0	0
50d14361a44877a216b80530078ce293	0	0	0	0	0	6	0	0	0	0	0	0
20be9b23219e8c7577a747eecc01e6e9	0	0	0	0	0	0	12	0	0	0	0	0
f4780308c5282a7463732f0ccb45d127	0	0	10	0	0	0	0	0	0	0	0	0
d010400f1bc2d6f097fb6510e9dd4224	0	0	0	0	0	0	0	0	0	0	3	0

dfd40053c15497b3f2b8d5f8 4dde2990	13	0	0	0	0	0	0	0	0	0	0	0
8a9b6d16d40c16955a33a2f7 7c298096	0	6	0	0	0	0	0	0	0	0	0	0
020f9d59c028f4e44f6325731 b68de0c	0	0	0	0	0	0	0	5	0	0	0	0
157a5346235676eb2f82f9d3 583c5f07	0	0	0	0	0	0	0	23	0	0	0	0
41df8ccd0614266ac00cade8 e05466a5	0	0	0	0	0	51	0	0	0	0	0	0
54e82e62623e53551712c84 0e8468a53	0	0	0	0	0	0	0	0	39	0	0	0
0f61a70727bff1de65380963 3d55a36b	0	0	4	0	0	0	0	0	0	0	0	0
23700c60809e4a84c8ab2c26 c19ad6f3	0	3	0	0	0	0	0	0	0	0	0	0
cef564859344ed524a12471f 6c7b8b10	0	0	0	27	0	0	0	0	0	0	0	0
6629b5ae0c652d5d040426ac fc0ffce4	0	3	0	0	0	0	0	0	0	0	0	0
53932587cf2e7f4d0444180d 11b0a838	0	0	0	0	0	0	0	0	0	0	4	0
8d40a38c0fa1e8ff5fbe174f9f bca60d	0	0	0	0	12	0	0	0	0	0	0	0
e386d1304759fdcfc7f7ffd8fe eea3a9	0	0	0	0	0	0	0	0	0	0	4	0
9f5742a2ac2ed34e93dd088c 59e51ff6	0	0	0	0	0	0	0	5	0	0	0	0

3fa3499291a9e812c2d5443220399e77	0	0	0	0	0	0	0	0	0	0	0	3
cc18bccb87fc03d8a1e9c34e6c9172c7	0	0	0	0	0	0	0	0	0	0	0	13
d36dff7cc581c3ee78daaf442998001b	0	0	0	0	0	0	0	0	0	0	169	65
1aed6f44863139b3c4987ae959dd9ea5	0	0	0	0	0	2	0	0	0	0	0	0
f9600614f4fa1ba019fbd0c6b0ddc613	0	0	0	0	0	0	0	0	0	0	0	5
496ecb9f4ce2281f523225be378be3e8	0	0	0	0	0	0	0	0	0	0	8	0
1ee4a283241816d6f73021931dfd1419	0	0	0	0	0	0	0	0	0	0	77	0
1da86ba63763e55fce0cd5f2cf55b9bc	0	0	0	0	0	0	0	0	0	0	8	0
570a2028741de97b70b28c42c37f58e7	0	0	0	0	0	0	0	0	0	0	10	0
094730106b9d108b7068511832cb0653	0	0	0	0	0	0	0	0	0	0	2	0
0ebc6b9c001926ee00d7cde665c9440b	54	0	0	0	0	0	0	0	0	0	0	0
6c36db5147769cb2817379c85e81584b	0	0	0	0	0	0	0	0	0	0	4	0
6bf1145e25243400006e431f043fd884	0	0	0	3	0	0	0	0	0	0	0	0
6a47197be87bfa0080e303d4f8dbb756	0	0	0	0	0	0	42	0	0	0	0	0

20eb167437a49a6d57bafe42796395c1	0	0	0	0	0	0	0	0	0	0	0	154
066697415b479aa585867d1ad5f73343	0	0	0	2	0	0	0	0	0	0	0	0
da4b1dc1085548e64fb2495640f5097b	0	0	0	0	0	0	0	0	0	0	2	0
5434f79b1f3cf5ab21a5646722d42f45	0	0	0	0	0	0	5	0	0	0	0	0
cbf0a3b67772d0d1a5f19424da57fe25	0	0	0	0	0	0	0	0	0	0	0	8
0fed61ffbca7cad00e5018022507df67	0	3	0	0	0	0	0	0	0	0	0	0
c7353cfbf26bfdad1c2c409a9210c36e	0	0	0	0	6	0	0	0	0	0	0	0
c9c98c45776dbea79f3fb976ceb685c9	0	0	0	0	0	0	0	0	0	0	3	0
e3cc80d1267ff7a75cdd4e3f2d48795b	0	0	0	0	0	0	0	0	0	0	0	4
93251618ecebe349b2ee2873f99cca99	0	0	0	0	0	0	0	0	0	0	5	0
d4ce5980be4cc227b886e5db78beadc2	0	2	0	0	0	0	0	0	0	0	0	0
5a941f9582ba9bd3f0282454bf4fb95e	0	0	2	0	0	0	0	0	0	0	0	0
8f0ab2817ff930f0be2f6d2f7d81bf57	0	0	0	0	0	0	0	0	0	0	5	0
b9e62ed7c334e7aac1dc21dcc732c118	0	0	0	0	0	0	0	0	0	3	0	0

fc67465e2f67e575f5f1846dd b5fd3dc	0	0	3	0	0	0	0	0	0	0	0	0
6e348c1769c7f5a9d8add3ab 51a3f7af	0	0	0	59	0	0	0	0	0	0	0	0
384438927845420b670f2ee0 98707952	0	0	0	0	0	0	0	0	0	0	3	0
2020220bb8b185b78e6f0df5 8139b8f5	0	0	0	0	0	0	4	0	0	0	0	0
2879bebce5fb99597ac2700f 3605f0bc	0	0	0	0	0	0	0	0	0	53	0	0
b1c95aef79e54aaebb6bca4a 72c044a5	0	0	0	0	0	0	0	0	13	0	0	0
06e6c3208d0259ecbc291f0f 01fb72f4	107	0	0	0	0	0	0	0	0	0	0	0
adfe6592e95597199922ab55 bc01e937	0	27	0	0	0	0	0	0	0	0	0	0
dcfec85cc5fe6c22fadb6bf2ee 6d6e86	0	0	0	0	0	0	0	0	0	2	0	0
31440a983f98fdb599079396 4d1f6316	0	0	0	0	3	0	0	0	0	0	0	0
a826e8b7d533d22a907d9b4 bf1a0c3fd	0	0	0	0	18	0	0	0	0	0	0	0
8cae6d9d298483c5413be9af 7f922fc0	0	259	0	0	0	0	0	0	0	0	0	0
9cd1af1645c4c5511523f881a d5fccea	0	0	0	0	0	0	0	2	0	0	0	0
d8fadb70eb1b6dc08ac3629d 47863a23	0	0	0	0	2	0	0	0	0	0	0	0

e482e19541d17197d4699c6e0d498bac	0	0	0	0	0	0	6	0	0	0	0	0
dce2473a94452955bb418f6d5dbef534	0	0	0	0	0	0	0	0	9	0	0	0
62fcfbbd3885a920bc967c139b482b28	0	0	0	0	0	0	0	0	0	0	2	0
584989455d6a9fbe922c27863ea3e7f9	0	0	0	0	0	0	0	0	0	0	48	0
570cc8df0dfe1c0b406c82972b6fba6f	0	0	8	0	0	0	0	0	0	0	0	0
0214830e44dce064c1089962a0c4250b	0	0	0	0	0	5	0	0	0	0	0	0
67eb49680f5324062a2f264965ab1f3f	0	0	0	0	0	0	0	0	0	56	0	0
2c9b96570144d49a6467a74d592b2101	0	0	9	0	0	0	0	0	0	0	0	0
c51ad042606c8d5ce36f18a63e26ddb7	0	0	0	0	0	0	0	0	0	3	0	0
bbd10ce780526831eb5100ae050ccbf4	0	0	0	0	0	33	0	0	0	0	0	0
792e160dc520756f38122fc46c6f8bdb	0	0	0	0	0	0	2	0	0	0	0	0
db0d7a8d78abbff48e366168c86bfd78	0	0	0	0	0	0	8	0	0	0	0	0
43501b07363806215ad84ffc6b56250d	0	0	0	13	0	0	0	0	0	0	0	0
2d94219c44cd3b5a3bed988bb2b958a1	0	0	0	0	0	0	0	4	0	0	0	0

770a23d9c1ef3d6586726609c4e68b2a	0	0	0	0	0	0	0	0	0	0	11	0
3e8bcf6cacb21a3daec097c7d4f10e55	0	0	0	0	5	0	0	0	0	0	0	0
a032c7fd309da67ecbf6b80160789d9f	0	0	0	0	0	0	0	0	0	2	0	0
f1a1bad09dc35b170a21d586d78c17c4	0	0	0	0	0	0	0	0	0	0	0	5
b30880283a5cab4211a1ded5a290b2b1	0	0	4	0	0	0	0	0	0	0	0	0
830c5423e22d2ff1cffa26e339642497	0	0	0	4	0	0	0	0	0	0	0	0
bd55e70f68795da05fb4ebbc9aa12364	0	0	0	0	0	0	0	0	0	0	2	0
b38cf0c56046ab92554d150cf57dfef1	0	0	0	0	0	0	0	0	0	3	0	0
b8b91138a6f4474fa88c8883f9f363c3	0	0	3	0	0	0	0	0	0	0	0	0
d93d83f5f35c72baad201b8af24d5e03	0	0	0	0	0	0	0	0	0	0	3	0
f177396327253e614f8c50f3c4481358	0	0	0	0	0	0	0	0	0	0	0	3
74b5ba8c20409aff0777e3d3c2e3c606	0	0	0	0	0	0	0	32	0	0	0	0
72e12fc325527d8ac0fbdd438316a6dd	0	0	0	0	0	0	0	0	0	0	112	0
67469081b6687c0d3e965da262ddb83d	0	0	0	0	0	0	0	0	0	2	0	0

83414adebc20dc8244550ad e802b5d74	0	0	0	0	0	0	0	0	0	0	0	10
b05f9a9a4d334ef9703d512e 7fdf4fac	0	0	126	0	0	0	0	0	0	0	0	0
2b4cf5a48b50a1962ea812cd 906b2583	0	0	0	2	0	0	0	0	0	0	0	0
579a6233f359232a373f34db ddadc570	0	0	99	0	0	0	0	0	0	0	62	0
6ae8382878e92f6ca14acba7 b7065e08	0	0	0	0	0	0	0	0	0	0	5	0
04e40c4d748f4d8b6652dfd3 089425e1	0	0	0	0	0	17	0	0	0	0	0	0
cea8945c5c57e5abac79ed51 547caf5f	0	0	0	0	0	0	0	0	0	0	0	2
0fa324b4f01c7f4116becc9b1 cf3f17c	0	0	0	0	0	11	0	0	0	0	0	0
1643e583791d130b3ce23f83 f60a006a	0	0	0	2	0	0	0	0	0	0	0	0
6d456d5d7964dca7080512d 416a3f9fe	0	0	0	0	0	0	0	0	2	0	0	0
34655422b4bbcba5f3e2ff7e b5585127	0	0	0	0	0	0	0	0	0	0	3	0
37b1ae510ef74b2c51e811b3 b2a16c7d	0	0	0	0	0	0	0	0	0	0	0	61
706e7cfcbe16843ad23e4ed7 6e846a62	0	0	0	0	0	0	0	15	0	0	0	0
74c9680923e76e471690af99 9dc70eda	0	0	0	0	0	10	0	0	0	0	0	0

a2773e1a218f91d050f78bf5755e48ff	0	0	425	0	325	0	0	236	0	0	710	0
9df329594d881b678cbc51b2acb7c280	0	0	0	0	0	0	0	0	0	0	0	8
b8523fcb42ab858de0568a49bb8f190b	0	0	0	0	0	0	2	0	0	0	0	0
a677fb28bdf89ca2df58c0dc0fc36d7a	0	0	0	0	0	0	0	0	0	41	0	0
4b1c836c55793d93e510955c90a07456	0	0	41	0	0	0	0	0	0	0	0	0
fd56d5c6fe9102d44da5a7996e854de4	0	0	3	0	0	0	0	0	0	0	0	0
9ba743e187fc70f30bc968b14835f60d	0	0	0	0	0	5	0	0	0	0	0	0
4a762a6317dea08bc46dd69472f89f76	0	0	9	0	0	0	0	0	0	0	0	0
51a09469e0d0e7184ad77a46723681ee	0	0	0	0	90	0	0	0	0	0	0	0
dbd0df0b0eeb713403b88c02d28fa727	0	0	0	0	0	0	0	0	0	0	0	38
c0c2ef7fee33cf24afb05faba75073be	0	0	0	0	0	0	0	0	0	14	0	0
8cbac1b29b6dd1d50e7e0335d978b7fc	0	0	0	2	0	0	0	0	0	0	0	0
02f9435246341f6d9b0bdd19a42dfdb0	0	0	0	0	0	0	0	0	0	3	0	0
c1a2b270f30ef2f5cbb399c25c83139e	0	0	0	0	0	0	0	0	0	0	9	0

ac324616522c1f46f9e19f8680591d17	30	0	0	0	0	0	0	0	0	0	0	0
46538e472213e073817139523b42a25a	0	0	0	0	0	0	0	0	12	0	0	0
c18baaad99cde2d81349076bd7723185	0	0	0	20	0	0	0	0	0	0	0	0
dd7167b2489785317b843ffa92f482e9	0	0	4	0	0	0	0	0	0	0	0	0
7d124339b7cedae44c98f1a6324eb43b	0	0	3	0	0	0	0	0	0	0	0	0
748b46e1fd7009fc5e181572e0f665eb	0	0	0	0	0	0	0	0	0	0	4	0
abf496ae87d27c6b3164812e54bf6ae5	0	0	0	0	3	0	0	0	0	0	0	0
6554583963717cdfd30d004f8c8a2d93	0	0	0	0	0	30	0	0	0	0	0	0
d7a368d78c3f3c2177c763e8d2d84d40	0	0	0	0	0	0	0	0	0	0	4	0
12fa6dd8ebe6a34d420d055e0160251b	0	0	0	0	2	0	0	0	0	0	0	0
7dca2b7c2e013fd4d2ac3217c9186709	2	0	0	0	0	0	0	0	0	0	0	0
fcf2cc1d0e5ddd47611e8eea69cc57be	0	0	0	0	0	0	0	0	0	0	8	0
5d1d7b4c9663c21f7f006ca02cbf4e8b	0	0	0	0	5	0	0	0	0	0	0	0
041d5a42c2898b92f67fe82bf1d7b1ef	0	0	0	0	0	0	0	0	0	2	0	0

9ff43febca724e46dbdd4a893ab8ed05	11	0	0	0	0	0	0	0	0	0	0	0
9f600d51bc81254cd29338830ada0aa0	0	0	0	0	0	0	0	0	5	0	0	0
91b89f034a9f6373fbaa10c1b6b3ab78	0	0	0	0	0	0	0	0	0	0	3	0
4cca79c023df725d2661e49a47a02ef7	0	0	0	0	0	0	0	0	3	0	0	0
6722a04ec196b988b5ada29e8f70041c	0	0	0	4	0	0	0	0	0	0	0	0
b94247275b849defe512de0ff715a6b1	0	0	0	0	0	0	0	0	2	0	0	0
642bbc362ad54c6650cf7b8b1c1aa65d	0	0	0	0	0	0	0	0	0	5	0	0
67e396d8eaa9b467dad291e2d790663e	0	0	0	0	0	0	0	0	0	33	0	0
3491ee9653410382e705cdc71ead0870	0	0	0	0	0	0	5	0	0	0	0	0
a58c60c61bcc973b1710922e1a1c4ba0	0	0	0	0	0	0	0	0	0	0	157	0
63d9814a8ccf5bfbd5ae8bee98ecb0a3	8	0	0	0	0	0	0	0	0	0	0	0
680defacc2f1434beaa5c650b12d54be	4	0	0	0	0	0	0	0	0	0	0	0
40c9709c0ebece53434c71556bbf4a87	0	0	0	0	0	9	0	0	0	0	0	0
d92065ef41b1f099626e102473174e6b	0	0	0	0	0	0	0	0	0	89	0	0

e3900e58ff70581bb5bd2be0 87fe0052	0	0	0	0	0	0	0	0	0	0	0	2
7375777758757eda9611e72 adc33a708	18	0	0	0	0	0	0	0	0	0	0	0
f8a8e7309f70a1a1e999b388 e4e8d163	0	0	0	33	0	0	0	0	0	0	0	0
783036f407e0ef6c3767686f3 4f10a29	0	0	0	0	0	0	0	2	0	0	0	0
d79f6a4f550ee43ae6929884 158f341e	0	0	0	0	0	122	0	0	0	0	0	0
d16f8b9d5ef9c8cb4f482fae6 db50d11	0	0	7	0	0	0	0	0	0	0	0	0
7dc08154ab3662a0d750dc8f 8315d9e3	0	0	0	0	0	0	0	3	0	0	0	0
ab0b1154af1e90f7fbabb21ff e599686	6	0	0	0	0	0	0	0	0	0	0	0
a5b4134806fc138b099e05bd dec90007	0	0	4	0	0	0	0	0	0	0	0	0
52ead70cf83c0aa11408fe287 106b4d2	0	0	0	0	0	0	0	6	0	0	0	0
1962e035b7e2a240956a0bff 9f735cc4	0	0	0	0	2	0	0	0	0	0	0	0
e0827863d0265494d7314b7 2d02d9bfe	0	0	0	0	42	0	0	0	0	0	0	0
26dd67f3cf24bb36650c4c1e 1000944b	0	0	0	0	0	0	0	29	0	0	0	0
51a3e810811c738fc3bf0ee2 ecedd468	0	0	0	0	0	0	2	0	0	0	0	0

0c98db083499df1d17d7e661c8d44f57	0	0	0	0	2	0	0	0	0	0	0	0
844ee38ab9cb7a2f0412d9bbfba73214	0	0	0	0	0	0	0	0	0	0	106	0
d437c663382844931ee8bb0c33e54897	0	0	12	0	0	0	0	0	0	0	0	0
c5cc36016fb8b2774b514426cc715aae	0	0	0	0	4	0	0	0	0	0	0	0
137ccf6c6c3f84551a4f2b26d5e7ebb6	0	3	0	0	0	0	0	0	0	0	0	0
ee52a9f147ba5fb9e5053032cef600a6	0	0	0	0	0	0	0	0	0	4	0	0
b90f5fc715627a19513f80480cbee727	0	0	0	0	5	0	0	0	0	0	0	0
9ee215c870d60ae1e8d37646e6fd16ea	0	0	0	0	80	149	0	0	0	0	0	0
a0ccda5a2bd4060ceaa189057a4d25a0	0	0	2	0	0	0	0	0	0	0	0	0
a004bc4636f8ab315e779d4a410bd178	0	0	0	0	0	0	0	0	0	9	0	0
3f2e4bb2ac321af098ef96c0af24506e	0	0	0	0	0	0	0	0	0	0	0	28
813e160fd4215ecc3add94027d96dee7	2	0	0	0	0	0	0	0	0	0	0	0
e1e95b04d9897940555af400d5445551	0	0	0	0	0	5	0	0	0	0	0	0
75224e37118f5d08e3492ad9b1f6591b	0	0	0	0	0	0	0	0	0	6	0	0

2af306652410d0dfeeddc6f0b654d515	0	0	0	0	0	5	0	0	0	0	0	0
d7cdd4eb26928df78c9da15f64f43d46	0	2	0	0	0	0	0	0	0	0	0	0
27ed47ac4e8ac10fa7773dd021258d9d	0	0	0	0	0	0	0	0	0	7	0	0
667b4f879070a8d3c31e9063cacc8dad	0	2	0	0	0	0	0	0	0	0	0	0
1a447749aa082ddcd5407b6ef291cd8b	0	0	0	0	0	0	0	0	0	0	45	0
e527e116f2b61d972e8e24fb3a64fc36	0	0	0	0	0	0	0	36	0	0	0	0
7076ce5442af36a1604632fffb84477f	0	0	0	0	0	0	2	0	0	0	0	0
1557ffb5b8a5208157e76e38d56b6c61	0	0	0	0	0	0	0	0	0	0	0	5
99e8065216af02533642a5033c2b902b	0	0	0	0	0	0	0	0	3	0	0	0
5b6f7185d1f43d9795bd11f86d3f28a5	0	0	0	0	0	0	2	0	0	0	0	0
4cfd9cb4b0f70bf791fcdb75eae36c0f	0	0	0	0	0	0	0	0	0	2	0	0
9ff0efd8b60d131a7cf5d7697af1b2f2	0	0	4	0	0	0	0	0	0	0	0	0
5d870d8dbe14c5f94c4210bd9d9ad7d8	0	0	4	0	0	0	0	0	0	0	0	0
d41d2aef3cc8a7f4a10e607acbdfe274	0	0	0	0	0	0	0	0	32	0	0	0

1c742cc3a4bad52c1dc97900d199bb4f	0	0	0	0	0	0	0	0	0	0	0	2
e30e0e260d1f30edf4cf7e1bc74ffbf1	0	0	3	0	0	0	0	0	0	0	0	0
bcd6eef49483e9e302f6a94d457b1a4a	0	0	0	0	0	0	0	0	0	0	5	0
8a8e415a04bed3f7088959c137f02a63	0	0	0	0	6	0	0	0	0	0	0	0
88ff1eb081a808a6571164d50075f6c6	0	0	0	0	0	33	0	0	0	0	0	0
79f470af0f79a568f31aea0f88542cb9	0	0	0	0	36	0	0	0	0	0	0	0
2d98cd29c433d0e53229e05349a3f073	0	0	0	0	0	0	0	0	111	0	0	0
97817a86de0f92db081ba95e7650226c	0	0	0	0	0	0	0	0	0	0	0	4
8e2e595baff765d331c04490b317f15a	0	0	0	0	0	0	0	3	0	0	0	0
8d32d67fd392511c06f517c961fdd38d	0	0	0	0	0	0	0	9	0	0	0	0
862b517d41060a8987687878bfa45612	0	0	0	0	0	0	0	3	0	0	0	0
16467d6fb54360335006221fc2b58362	0	0	0	0	0	0	4	0	0	0	0	0
8171257d24860b4042565926cf8d2a00	0	0	0	0	0	0	0	0	185	0	0	0
70711c955d81c9793c964f0a38f5663a	0	0	0	0	0	0	0	0	0	0	0	77

659616ee1705c4314db8860 2a865c0b7	0	0	0	0	0	3	0	0	0	0	0	0
6d33ea3187249185f4f10e5d f2fed12f	0	0	0	0	64	0	0	0	0	56	0	0
40cb40d4acc8de37509eb94c d09bc270	0	0	0	66	0	0	0	0	0	0	0	0
206f1eee6c18dbed94863b14 5ad73949	0	0	0	0	18	0	0	0	0	0	0	0
e6febfc6bd62598306579a27 d71930c2	0	0	0	0	0	0	0	0	0	0	8	0
4e9475ca33df1347ee5aeb45 b257e6dd	0	0	0	0	0	0	13	0	0	0	0	0
535e1cc3d0a3023a45aeeab1 092d831d	0	0	0	0	0	36	0	0	0	0	0	0
17176a344a0cb7af255185bf badf03cf	0	0	0	0	0	0	14	0	0	0	0	0
43033ead6d266d59b5ec1e0 24b41e34e	0	0	0	0	0	13	0	0	0	0	0	0
474e49d8ba49c83ce4a200cf 76dccd57	0	0	0	0	0	0	0	0	0	0	0	2
e15f847d3200a48054a86f2e c78a3b97	0	0	0	0	0	0	0	0	0	0	0	25
58251ca4340b4f126ba4f639 5edb2a39	0	0	12	0	0	0	0	0	0	0	0	0
5e76dfd277e9c7e0085f5c4a 741fbb08	0	0	0	0	0	0	0	5	0	0	0	0
6f58a0f3f022c2af68a2abe77 02d540b	0	0	0	0	0	0	0	0	0	30	0	0

d849e015681b2b345ac8076bf9625750	0	0	0	177	0	281	210	0	0	0	0	0
ac7c0f23b5bddd4542f8aa7c03600551	0	11	0	0	0	0	0	0	0	0	0	0
0290700b5000448b8661ac40da7f5ab7	0	0	0	0	0	39	0	0	0	0	0	0
af9614c15f6dc6027e88202b311e96b7	0	0	0	0	0	0	0	0	0	0	18	0
05e71fc83016b964185ccf835e66ed78	0	0	0	0	0	0	0	0	0	4	0	0
60f3b9a3317d6dfaf9af7b054b5ea818	0	0	0	83	0	0	0	0	0	0	0	0
e5d0a72d189c851e4dd81a6ea8a7e143	0	0	0	0	0	0	0	0	0	0	19	0
62480bd66e93ea903648800f7d3d4bfb	0	0	0	0	0	0	0	0	0	0	14	0
b66280351bf413e9b116bba6f307d4d4	0	0	0	2	0	0	0	0	0	0	0	0
76d345ba663bdbac055f314e847e304c	0	0	0	0	0	3	0	0	0	0	0	0
3c21276736bb419fb9ba555b177e8e39	0	0	0	0	0	0	5	0	0	0	0	0
1926595304a5fda00a08bd6df442dcae	0	0	0	0	347	0	0	0	0	0	0	0
e6d0cbd80206d90fda7e7be608c49c21	0	0	0	0	0	0	0	0	0	0	7	0
c82a762ef31a2bf980c01fba5d9d6951	0	0	0	0	0	18	0	0	0	0	0	0

ad2172a4664893bc9d69484 77d95fd5c	0	0	28	0	0	0	0	0	0	0	0	0
35e071a04a03199af0a9f07e 403535bf	0	0	0	0	0	0	3	0	0	0	0	0
5c985fac91ca4cd15fb6b2343 4b36c41	0	0	0	0	0	0	0	7	0	0	0	0
cafffb6aa514f177d3a30db81 7ac0ce7	0	0	0	0	0	0	0	0	2	0	0	0
bb45eef285a3a394fd8ca595 302c71c7	0	0	0	0	0	0	0	0	0	45	0	0
c433264d1b33a62d63ed8b2 dff8b7e21	0	0	0	0	0	0	9	0	0	0	0	0
7a3fdc74ee5fd280f50384d32 163402a	0	0	0	0	0	0	0	0	0	0	50	0
15ab15ed8a1ab394cf977d29 4e7daa95	0	0	0	0	0	0	0	5	0	0	0	0
1dc4c9d9cf8d91a938fab54f3 4f13e4a	0	0	8	0	0	0	0	0	0	0	0	0
e4bad30738f6bef4383ca28f0 2012f89	0	0	0	0	0	0	0	0	0	0	8	0
e58385b0e436d0d89fdf1e0d ec907622	0	0	0	0	0	0	0	0	0	0	2	0
6432e76fe70581f52d6c83fce 29da103	0	9	0	0	0	0	0	0	0	0	0	0
82385973bdfdf920252a86f5 2484a067	0	0	0	0	0	0	0	0	16	0	0	0
9bb731362f464942ec039bdb 63eebd16	0	0	0	0	0	0	0	0	0	0	0	3

0b5f31013599fb1d01ea3a4f58d3837e	0	3	0	0	0	0	0	0	0	0	0	0
85997c2648f9a37a7b7f6cda9d1cf2a1	0	0	0	0	0	0	0	0	0	0	2	0
9ea2fad6129a2bfe8dab50fa2af6288d	0	0	0	0	10	0	0	0	0	0	0	0
69e5a7ace1f7252fd0081c63dda458e6	0	0	0	0	0	0	0	0	0	0	22	0
7d817de624b0f4f88fa8c7c7b43ae0dc	0	0	0	0	0	0	0	12	0	0	0	0
92e5148422e8bfef9b2b2c0b92fa8147	0	0	0	0	0	0	0	0	3	0	0	0
dcc0f6610aea228e228a7a674b962f8e	0	0	0	0	0	0	0	0	0	0	3	0
b5f8692babadfb1959878ae9dc15c95b	0	0	0	0	0	0	2	0	0	0	0	0
72cbdd4cb9c288f79910574b0629c8f5	0	46	0	0	0	0	0	0	0	0	0	0
c56a258befcddda4597c59e867668e6a	0	0	0	0	27	0	0	0	0	0	0	0
9a9f498e55156251e3908330706055f8	0	0	0	0	0	0	0	0	0	0	0	4
07d6d713792bfa31b34767784c565509	0	0	0	0	0	0	0	0	0	0	10	0
aa8e5945b1b2556ff040f34b6ebdf94f	0	0	0	0	0	0	0	0	0	0	0	76
d976d09830972c4dd3abfcc1134eb0b8	0	0	0	0	0	0	0	0	0	0	4	0

b204d40d4070a8fce88e41e14174125d	4	0	0	0	0	0	0	0	0	0	0	0
06ac5279994e1c20f73a373b5c7ce342	0	0	6	0	0	0	0	0	0	0	0	0
1030faab530b829df8585b68943d8894	0	0	0	0	0	0	0	0	10	0	0	0
82eb2e761cc822027e8151b4e190c9db	0	0	0	0	0	0	0	0	0	8	0	0
b83be0239fa2e6c18f3ad0d14eafaabc	0	0	0	0	0	5	0	0	0	0	0	0
550bed05ae8d8c18c3d4584918548406	0	0	0	0	0	0	3	0	0	0	0	0
af66bbc282f8747cdfca4d15c7d35388	0	0	0	0	0	0	0	0	0	3	0	0
cfaac59db06a6f0be4f8c0b3091fbb68	0	22	0	0	0	0	0	0	0	0	0	0
133d19c5c90c3d12991943b033e4eddd	0	0	0	0	0	0	0	0	0	0	0	9
e91292abb4961c26e1c9fd102137ffbf	0	0	0	0	0	0	0	0	0	0	6	0
b1667cfb08defc0538e9ebb2d2158906	0	0	0	0	0	0	0	0	0	0	2	0
1d0f516518eea963ae639ec2bee091ea	0	0	0	0	105	0	0	0	0	0	0	0
151edb73665792514b5a193439c4f92f	5	0	0	0	0	0	0	0	0	0	0	0
f7a42e6f22e4dfca4da9adef3b622803	0	0	0	0	0	2	0	0	0	0	0	0

136c0e74603a9d0f6a7534a41bff39d8	0	0	0	0	0	5	0	0	0	0	0	0
d4ae04f163187e17e2cf667526efa224	0	0	0	0	0	2	0	0	0	0	0	0
7306bf167f4e85aec03825a213733757	0	4	0	0	0	0	0	0	0	0	0	0
67ffc7236a0b8ba2d60b8aa9a5b8dbd4	0	0	0	0	0	0	0	4	0	0	0	0
db9e033bfc666914ccdb74bce8cf76ab	0	0	0	0	0	0	0	0	0	0	2	0
61fe30d19677c8564fb03d1651bdc079	0	0	2	0	0	0	0	0	0	0	0	0
118c19effb31b0c770b58d17be9aea7e	0	0	0	0	0	5	0	0	0	0	0	0
a4261dc0872fe1f2eafc27e7e3176271	0	0	0	0	0	0	0	2	0	0	0	0
0797e80d823c6620beeb7b12b31396cd	0	0	0	2	0	0	0	0	0	0	0	0
d111311f2aee7b8e64f21bff4aa04af2	0	0	0	0	0	111	0	0	0	0	0	0
89d522c7950941770f7ef81666b04c90	0	0	0	0	0	0	0	0	0	0	52	0
7f40b4e508ddf4185c6eef17138afbf1	5	0	0	0	0	0	0	0	0	0	0	0
fa03b07a1c2671062d2c182bd7e245ee	0	0	0	0	0	0	0	0	0	6	0	0
9a8134f320dbcb0dd335dd5002978552	0	0	0	0	0	0	0	0	0	0	0	5

809f9e3c876bc90def9d99f0996c107d	0	0	0	0	0	0	0	0	0	0	4	0
acc3ec5f241d04aecab40c67dc2422b9	0	0	0	0	11	0	0	0	0	0	0	0
bcfaf7ecb451dd1025954a41376b878b	0	0	0	0	0	0	0	0	3	0	0	0
263c7b3deef7be9615273ae2c122d9a4	0	0	0	0	0	0	0	0	0	0	0	104
83ccec4c7690b44f821f1dd834bc4d03	0	0	0	0	0	0	0	0	12	0	0	0
77d38ffd53203b985308921cf8b166d0	0	0	0	0	0	0	0	0	0	3	0	0
2e38450a8e62b7d9646e28cb0c1ccbe1	0	0	0	0	0	2	0	0	0	0	0	0
a23394b04a46f3e6d5c5e2ebd35bee0b	0	0	0	0	0	0	0	0	0	0	44	0
8554d1843ec5b5c0404923a4b9cd137a	0	0	0	0	0	0	0	3	0	0	0	0
5ad58fee9d7bb0d119c3bfab03f39273	0	0	0	0	0	0	0	0	0	0	6	0
d16ba0f73454ec62b72dd06b24c305e3	0	0	0	0	0	0	3	0	0	0	0	0
889b94b8ccd4c02d4f4c528b1786de41	0	0	0	0	0	0	3	0	0	0	0	0
cc5a62b7272dbc5aeaae37c1a1692b2e	0	0	0	0	0	0	0	13	0	0	0	0
add4a7e0531d5d7d86baf399b6000f46	0	0	0	0	0	0	0	0	23	0	0	0

a24cd761b73ba78268398bbf43fdecf3	0	0	87	0	0	0	0	0	0	0	0	0
78a6b30605dff74a3ab0da6f06d0f3de	0	0	0	0	0	0	0	0	0	0	0	2
f29fabedf862be46caa226a1eb6e4253	0	0	0	0	0	0	0	0	2	0	0	0
2158d1f84ef0884dccd1ba071f50c642	28	0	0	0	0	0	0	0	0	0	0	0
79641d5b9aae95d8a09dbfc0516a0910	0	0	0	0	0	0	2	0	0	0	0	0
3507c4f95f5de2cb24c7fd5d43a6719c	0	0	0	0	0	0	0	0	0	0	34	0
3d9db90d8ba1c5cebc0f6bd8b48b2310	0	25	0	0	0	0	0	0	0	0	0	0
7d3bf566f0d0385c3bb9d5bf3dd5d4de	12	0	0	0	0	0	0	0	0	0	0	0
e5d9dfa41050a0cb96320842d64730cb	0	0	0	0	0	0	0	0	0	0	0	18
b2b05b510dc0e28f8836c7fa369840de	0	0	0	0	0	0	0	0	0	0	0	9
027ea81c7df551bd3861559eee791402	3	0	0	0	0	0	0	0	0	0	0	0
4c0a820bba8900114d0d906e99ee947b	0	0	0	0	0	0	0	0	0	0	5	0
b0b42cfd7754731cc9b7aa4861e0fa87	0	0	2	0	0	0	0	0	0	0	0	0
809fb9bf8e994d42bd498eb40e70bd7c	3	0	0	0	0	0	0	0	0	0	0	0

6a96d63df8fd48f207c3f5eb9d922b71	0	0	0	15	0	0	0	0	0	0	0	0
50e5f0bd2c1886c0e931806558a41641	0	0	2	0	0	0	0	0	0	0	0	0
eb93d0fac94e0c1bea2ed2adba237bf2	8	0	0	0	0	0	0	0	0	0	0	0
50820bc4b79061a04309a620a2dbef72	0	0	0	0	2	0	0	0	0	0	0	0
7935e269adcf30501f272f6f2cb36131	0	0	0	0	0	6	0	0	0	0	0	0
2a38831b0813e3ea698beb7235108b4f	0	0	0	0	0	0	0	0	0	0	3	0
93f21ec2ab1c47c02cf3c2ae2c0c5c8c	0	2	0	0	0	0	0	0	0	0	0	0
bafd5c5a36feff8a25b7b4c723e1c6b3	0	0	0	0	0	8	0	0	0	0	0	0
bec6323bc5a8f51c2a0c6661f0cd1a38	0	0	3	0	0	0	0	0	0	0	0	0
f1fc2294b9e396e90fe7e1e794a77cf0	0	0	2	0	0	0	0	0	0	0	0	0
d1bb1a9cc04a66f82ae8e92575edefb8	0	0	0	6	0	0	0	0	0	0	0	0
bfb0ef0282e27cc49cf64ce421d3f864	0	0	0	0	2	0	0	0	0	0	0	0
33b9394ee2f6d7b4587941ee922c2816	3	0	0	0	0	0	0	0	0	0	0	0
30d9e758ba5b6fdcff021e8a706c47e1	0	0	0	0	0	0	0	0	0	0	3	0

1b4600b09badcb65f8095cb1aa6bf040	0	0	0	0	0	0	0	5	0	0	0	0
6711a7b4bd96d565c423cd816d0169d0	0	0	0	0	0	0	0	8	0	0	0	0
49640ed3d21853f86609042061fbd75f	0	0	0	25	0	0	0	0	0	0	0	0
658619cc1f98f40fcec40fda9c1f91c1	0	0	0	0	23	0	0	0	0	0	0	0
d0b0a31728c254edeb2dba057f8c67d4	0	0	0	20	0	0	0	0	0	0	0	0
f6994a58dd8f60564ba960b73e4353ff	0	0	0	0	5	0	0	0	0	0	0	0
ce170d4f5d80bff1deb8cb9457b8a376	0	0	3	0	0	0	0	0	0	0	0	0
3b35e9d09194fd5816977864d220bfc2	0	0	0	0	0	0	0	0	0	0	8	0
8630b6790ebfbccb50e5190cac54aa62	0	0	0	0	0	0	0	0	0	23	0	0
d618d136459d62dfccb1d50bc4811326	0	0	0	0	0	0	0	0	7	0	0	0
7e7432fe6fb9003a0243cecf21ac019f	0	0	3	0	0	0	0	0	0	0	0	0
17eada4575730816ca6435d05659033e	7	0	0	0	0	0	0	0	0	0	0	0
e5e78b75ac488a0d5c1316845c157355	0	0	0	0	0	0	0	0	0	0	0	11
5320ce59266235eecb8042e155f9cee8	0	0	0	0	0	0	0	0	0	0	5	0

9926448e824e855173e7dac3a59a1cdb	0	0	0	0	0	0	0	20	0	0	0	0
8a9eaecab41893901924fcb4c7731e5d	0	0	0	0	0	0	0	0	33	0	0	0
274c543569171aef3f1283aa9475116d	0	0	0	0	32	31	0	0	0	0	0	0
06cc4f2b5f7723efd022daa4c01d3ab8	0	0	0	0	0	0	0	8	0	0	0	0
8dc31fb886be7ed6e2c9d81b1b1f7df6	0	0	0	0	0	0	5	0	0	0	0	0
3b105c26f97f3ebb03e24912700b1a48	0	0	0	0	0	0	0	0	6	0	0	0
1692aa498a822a4f49aa1e2a9cd146df	0	0	0	0	0	0	0	0	9	0	0	0
563d6498556205d671d7ad4859f67958	0	0	0	0	179	0	0	0	0	0	0	0
16003ea9d78c87b6afe69a6bc8888156	0	0	0	0	0	0	0	0	3	0	0	0
e9dac528d00769b5666309129ee3fbab	0	3	0	0	0	0	0	0	0	0	0	0
40b5adcd2f53634d5a14742c063a6853	0	0	0	0	0	0	0	93	0	0	0	0
2a26c2650c213f6c3b7a6313e61667c8	0	0	0	0	0	0	0	0	0	0	0	9
01b10fe9a4a59bb4727077ed17a2e0cf	0	0	0	0	0	8	0	0	0	0	0	0
11af2cf1c77b76d4a3b5c1ef3c56e92f	0	0	0	0	0	0	0	0	18	0	0	0

8fcc2856ab73fd821b9b166df cc2613c	0	0	0	0	0	0	0	129	0	0	0	0
63d238ebedd68161e280efc1 90819d04	0	0	0	0	0	0	0	0	0	0	0	11
14418767dfb8c5fffeabd924f 02f79fd	0	0	0	0	0	0	0	0	0	0	0	19
c3081b5b01b8e82eb0c7820 3d91d1e45	0	0	0	0	0	0	0	0	3	0	0	0
22a1cea0ed45f69d04ea4772 8de8adfb	0	0	0	0	0	0	0	0	2	0	0	0
b27827b20e9fa23063c10006 e19d67a9	0	0	0	0	11	0	0	0	0	0	0	0
746c1697908a37d1abf63140 8cba6f94	0	0	0	0	0	0	0	0	0	0	0	3
f7fde539eacbbf83a41c29057 9487da5	0	0	0	0	0	0	0	51	0	0	0	0
0b4bf383e7f8a0a00a39d0a7 5d286dc1	0	0	4	0	0	0	0	0	0	0	0	0
14942c6310b27f36c038bc36 198b22a6	0	0	0	0	0	0	0	0	0	0	3	0
af3d4fd30222bb1ea24eb410 84eb0fc9	0	0	0	0	0	0	0	0	0	39	0	0
1e6f723fe7e92484688da055 d52544a3	0	0	0	0	0	0	4	0	0	0	0	0
7a64329b3925215069bf0d59 25c241c4	0	0	0	0	0	0	20	0	0	0	0	0
0027b116458535ad605ee79 1e15bc885	0	0	0	0	0	0	0	3	0	0	0	0

6f57db49580ff234b4c38e62 73609308	0	0	0	0	0	0	0	0	0	0	0	3
c1cb04ce17882ab3805d50bd d1037252	0	0	0	0	0	4	0	0	0	0	0	0
d5017c5f8bac59d11c23c031 15d726d6	0	0	0	0	0	0	0	0	0	0	4	0
7b120644c1ac106d4e73ad1 b4895a709	0	0	0	0	0	0	0	54	0	87	0	0
ac742112b98f35dde7009705 ad00622e	0	0	0	0	0	0	0	0	0	0	23	0
fcb6df4da820b293c864e8f6b 289f41a	0	0	0	0	0	0	6	0	0	0	0	0
154869a02a21ef1226db18ab 7fcad5fc	0	0	0	0	0	12	0	0	0	0	0	0
db827e6e886e5a0215c93ab b6013ed38	0	0	0	0	0	0	0	0	0	0	0	12
bf3f56d51b0e4ed84ae2134c b14f6d9b	0	0	0	0	0	0	0	0	0	0	0	3
49e09517ed2eb94599c4342 01aa09069	0	0	0	0	0	3	0	0	0	0	0	0
ecf84aa4d9377242bed9892c 79157a02	0	2	0	0	0	0	0	0	0	0	0	0
90c7154ebf2feebabf3b63129 e134bd8	0	2	0	0	0	0	0	0	0	0	0	0
c9a72a8bbab65f943aca1d15 f331fa53	0	0	0	0	0	0	0	0	0	5	0	0
1de3e51498d1e19e8da996b 467db3efb	0	0	0	0	3	0	0	0	0	0	0	0

4811e0bd34a8e3a651f3b228a1e1012b	0	0	0	0	0	0	0	4	0	0	0	0
72b43224fcd9fd8420e60985861a2da3	0	0	0	0	0	0	0	0	0	0	0	57
a20aec5709f7453c9ff4ee231fca2e04	0	0	0	0	0	0	0	0	0	0	0	6
515b3b478576bb5bfa4e6dc56df7c47f	0	0	0	0	0	0	0	0	0	39	0	0
9164f1cc4d4065b7d76a025c9f018e72	0	0	0	0	0	0	0	0	0	0	28	0
f8126689bda234f92cde793151263f8d	0	0	0	9	0	0	0	0	0	0	0	0
4f32646a3b1111c7af5360072cf1f7cd	0	0	0	0	0	0	0	0	0	4	0	0
1456381fd6b8998ddbf7f6aad25029d4	0	0	0	0	0	0	0	16	0	0	0	0
d987faff7991c1b166a5dd8a3a8f049d	0	0	0	0	0	8	0	0	0	0	0	0
d312bf45719a320d0cec28a5279ecf28	0	0	0	0	0	4	0	0	0	0	0	0
076f4e48b34ca456d15e5537baa81dc7	0	0	0	0	0	0	0	3	0	0	0	0
a8248bbfcd8177e78f63c3b7eb004774	0	0	0	0	0	0	0	0	0	0	310	0
fab773b95af60962b78b728a72cbdb88	0	0	3	0	0	0	0	0	0	0	0	0
46f27fd13d038f9e666fd920c8988c9f	0	0	0	0	0	0	0	0	0	0	2	0

85897e7dbd4f8daedb1ce5028a0c1372	0	0	0	0	0	0	47	0	0	0	0	0
b29289f8bfd4201abead9ee857b443ad	0	0	0	0	0	0	0	0	0	7	0	0
01dde9d8131c433d21d34b723fbd636d	0	0	0	0	0	0	70	0	0	0	0	0
a642fe944f975d44c5168acd69539be6	0	0	0	0	0	0	0	0	0	0	5	0
f9d8e98dcd84a08b01421c319a8c3cb2	0	0	0	0	0	0	0	0	0	0	3	0
67cdbdaa4d808e019f21fca44f778d02	0	0	0	0	0	0	0	0	0	0	128	0
8dfd965d10bff462c6be972c91066b34	0	0	0	0	0	0	0	0	3	0	0	0
597921e18847b247302711d29ceb24a2	0	0	0	0	2	0	0	0	0	0	0	0
64a1a23f06736edfe26530a041121b50	0	0	0	0	0	0	0	0	0	0	0	19
5918edf1a265634ff8fb319a7490f873	0	0	0	0	0	0	0	0	0	7	0	0
51474528a2c80aeb731c6d328bf66780	0	0	0	0	0	0	0	0	0	28	0	0
a512261e3a2588450d680836ceefb39f	0	0	0	0	0	20	0	0	0	0	0	0
57e0b2c2d3ba51006de8e657bb0010fd	0	0	0	0	0	0	0	0	0	0	2	0
e970d065bcdcaf83891ade638f8ecd10	0	0	0	25	0	0	0	0	0	0	0	0

8b74503a4752c267cfbfe65f67652ed6	0	0	0	0	0	0	0	0	0	26	0	0
959e0c100a4178875b00cd2a6474cb4d	0	0	0	0	0	0	0	0	0	0	32	0
fbd7e8cee0c7fa267bbd76126e05981f	0	0	0	0	0	0	0	0	0	10	0	0
09d5e390f495f29ee0002ea9afff7dd3	0	0	0	0	91	0	0	0	0	0	0	0
f830781bedb0b4754274e33edea00689	0	0	0	2	0	0	0	0	0	0	0	0
a5b00dfa4f7bd59c358f7f694eb75cc4	0	0	0	0	0	0	0	0	0	0	0	48
4febf8095820b238927d816855dd4e9e	0	0	0	0	0	0	0	0	0	0	0	13
895bf834335a0b8e94f2458c94dc1ab4	0	0	0	0	0	0	0	0	0	0	0	30
4252df540c4e9237feb7666a738a2132	0	0	0	0	0	0	0	0	0	31	0	0
eeb64a38b4c8bfa98e36b30dd4b44acb	0	301	0	0	0	0	0	0	0	0	0	0
03a2ca0570fb40c78e948588f66a4d12	0	0	0	8	0	0	0	0	0	0	0	0
67e90efb290f7e731036f118fee367c9	0	0	0	0	2	0	0	0	0	0	0	0
99aa8164a68b1e158746170a14b59393	0	0	0	0	0	0	0	0	0	0	8	0
be3163b113ce5dbafa07dd9582ee472e	0	0	0	0	0	0	0	0	0	0	4	0

5b59ecec318f0ecd23d00a3c07cbc8ae	0	0	0	2	0	0	0	0	0	0	0	0
09ed18422bc31ae32f67d2fcbff839e8	0	0	0	0	0	0	0	0	0	0	0	2
b02668c5ab3328a10b4f6bc7b4f6caa2	0	0	0	0	0	0	0	4	0	0	0	0
43ffaf78ca24527f81d03fc481ed068a	0	0	0	0	0	0	0	0	0	139	0	0
edfad9b54ffe5bc44ae1962ca2d2fa45	0	0	0	0	0	0	0	0	3	0	0	0
5347a4e8dc82907644af5eea1d93f62f	0	0	0	0	0	0	0	0	0	0	0	4
f7b5010457be7cdedba1cd21760d29c1	0	0	15	0	0	0	0	0	0	0	0	0
44874cd6205e897cbefa7dab6139417c	0	0	0	0	0	0	0	0	0	6	0	0
83ecd52dfff7a7836103e6d6c8f8a58a	0	0	0	0	0	0	0	0	0	0	16	0
f3673e4d735cffeabb9ea3ab9ba046f4	0	0	0	0	0	0	0	0	0	0	4	0
7b2491897bb5a2bdcd1d323eaff289e3	0	0	0	0	3	0	0	0	0	0	0	0
4e03abe2757fcb6d5472a6e1295fa7dd	0	0	0	0	0	0	0	0	0	112	0	0
81b50db14c17f1f13ce6ce901320cde9	0	0	0	0	0	3	0	0	0	0	0	0
6a22ef8df32256654b781bc42b83226c	0	0	0	6	0	0	0	0	0	0	0	0

957bfa0a3da3851c8ca37093a3a42288	0	0	0	0	0	0	0	0	4	0	0	0
68783f72da746202c8895bbbcc636fac	0	0	0	22	0	0	0	0	0	0	0	0
d7b03d64eca548be63716b05ab7d328c	0	0	0	0	0	0	5	0	0	0	0	0
94f908776a1d3c31799ccc7f88f55d21	0	0	0	0	2	0	0	0	0	0	0	0
10466c3acec77834ffcbaca5de459100	0	93	0	0	0	0	0	61	0	0	0	0
49ecb346e7b232917fd193e63a91d863	0	0	0	0	0	0	0	20	0	0	0	0
3a0823b07d4388248676e654c8d0e01b	0	0	0	0	0	16	0	0	0	0	0	0
8f42b49f200d1991c46b92771cbc3b0b	4	0	0	0	0	0	0	0	0	0	0	0
b2283d60c8667a7579013cbff35d01b1	0	0	0	0	0	3	0	0	0	0	0	0
00568ebe8457528f08f910a60037c217	0	0	5	0	0	0	0	0	0	0	0	0
7e8c8d58b4bc7b2850e40d9b7cb13394	0	0	0	0	0	0	0	0	0	0	0	4
69dfd2d925a06843bd3be6a847597988	0	0	0	0	0	0	0	0	0	0	0	7
c43ac23cc3c7363e80312d6a19dffcc8	0	0	0	20	0	0	0	0	0	0	0	0
d333d91cf2bf4ae7eaf4f93e92fc69d4	0	0	0	0	0	0	2	0	0	0	0	0

3a7e7ecb7a1b111bea88a6f6de1569a5	0	0	0	0	0	0	0	0	0	14	0	0
22eb0414fc95e61475f4e7e52398f136	0	0	0	11	0	0	0	0	0	0	0	0
6970805e1d9050ceaea8e739ffa585fe	0	0	0	46	0	0	0	0	0	0	0	0
85c4fab9198958211e7c22da6c95a2ae	0	0	0	0	36	0	39	0	0	0	0	25
f1a072d2e377e04d8b36e951cd316a07	0	0	0	15	0	0	0	0	0	0	0	0
f4b35868b4cf952fe2d0aa130750ddfa	0	0	0	0	0	0	0	0	0	0	120	0
7e5df2e7af5f6451e42ef70c33c1141d	0	0	0	6	0	0	0	0	0	0	0	0
25f2bbe663c9b34d0d6d1d9d5463156f	0	0	0	0	0	0	0	2	0	0	0	0
644f81d1d3daa0c876cb477d723d812d	0	0	0	0	0	0	0	0	0	16	0	0
118c55fb8eb09823cc3a79ba6b73793c	0	0	3	0	0	0	0	0	0	0	0	0
8c7c2e948007eb0307cc630e40e36ae4	0	0	0	0	0	0	0	0	0	24	0	0
66954886f068c0e215ed75915e07998a	0	3	0	0	0	0	0	0	0	0	0	0
77c755ceaaef91b2903d8c52106dc78d	0	0	0	0	0	3	0	0	0	0	0	0
efc8916b7d6b6afe29f251bec0a405c0	0	0	10	0	0	0	0	0	0	0	0	0

ff1401b5563fd520e9259376f5030bb0	0	0	0	0	0	0	0	0	0	0	0	2
758a9ff230e7847e7f3444775556da33	0	0	0	0	0	0	0	0	0	0	6	0
059de4fa35639cf0d261b29a31b6145e	0	0	0	0	0	0	0	3	0	0	0	0
62573e5b2c9808764997e0e951412814	0	0	0	0	0	0	0	7	0	0	0	0
eb1a8838e287dc63d7f6eda2468bcc71	0	0	0	0	0	0	0	0	0	4	0	0
afa3e16d83848e592e7a2d9990d5a20c	0	0	0	0	0	0	0	0	0	0	0	5
4c4498838c793fcb03e8a8777449a22d	0	0	0	0	0	0	0	0	0	0	3	0
0c238ddad9adf90831452e15c52a77ae	0	0	0	2	0	0	0	0	0	0	0	0
22155f2d22a84f341d39c2c29059be74	0	0	4	0	0	0	0	0	0	0	0	0
6b8893f94cfd5fc6cedd618011f4b64b	0	12	0	0	0	0	0	0	0	0	0	0
5609e10a12286b6ac87b0498bd7480f3	0	0	0	0	0	0	0	0	52	0	0	0
913fffc0a530364fd6e8b31ea5584de3	2	0	0	0	0	0	0	0	0	0	0	0
92191b12a2069267eed441068ec5b889	0	0	0	0	0	0	0	0	0	0	0	30
498bc2935989100f1e0eaa8c467605aa	0	0	0	0	3	0	0	0	0	0	0	0

133ba9e7e765c38d240ec68e74883d57	0	0	0	0	0	0	0	0	0	0	0	67
1ef9e5f6beac56aea1c43d9255218c88	0	0	0	0	0	0	0	0	0	5	0	0
6f0f188f4a27c85b4450584cc0a2e65e	0	0	0	0	0	0	0	0	0	7	0	0
30fe673a8ccfaab3251ef37d385a1a83	0	0	0	0	0	24	0	0	0	0	0	0
01affa6795afd046817cc0bf801d93ae	0	5	0	0	0	0	0	0	0	0	0	0
59edff40f2a8e1ea2f7c4aa9912a4e77	0	0	0	0	0	0	12	0	0	0	0	0
e438078b9f51e3ac2fda1f5742132ae4	0	0	0	0	0	0	2	0	0	0	0	0
e71ba6617ea34ca77ad66071c2ca0e20	0	0	0	0	2	0	0	0	0	0	0	0
fa6d803142b135efd432faac5933b9f8	0	0	0	0	0	0	0	0	6	0	0	0
d16869a76529c52860efa15ddcbd93f9	0	0	0	0	0	0	0	0	0	66	0	0
0f1e892485a7ef218debd9cf23e52f90	0	0	0	3	0	0	0	0	0	0	0	0
0f8d0aee45d51936d410dbf0afba5aeb	0	0	0	0	13	0	0	0	0	0	0	0
9e2cb45583a7906c0f9e0a654bea367e	0	0	0	0	0	24	0	0	0	0	0	0
e7bd1db25b803cf3db6dd3af6689af2b	0	0	0	0	38	0	0	0	0	0	0	0

8bc6bd24d44d5bb117214d2e84b5b5a0	0	0	0	2	0	0	0	0	0	0	0	0
8c8e318097d27788592a6017dc4dd01c	0	12	0	0	0	0	0	0	0	0	0	0
1df124fb02bd3e98d5b82eda80b9ffbe	0	0	0	0	0	5	0	0	0	0	0	0
2737bd5d7496a142de1d25773bc9d060	0	0	0	0	0	0	0	0	0	0	6	0
aa93c3301c9586f11334848f656f787c	0	0	0	0	0	0	0	0	0	2	0	0
6c1ef62329b0de15c0987b1f9c217221	0	0	3	0	0	0	0	0	0	0	0	0
b2508ed40c3dc648fe83d42447535fdf	0	0	0	0	0	2	0	0	0	0	0	0
666e9d8f9cfe97b92c064d315ba8b14e	0	0	0	0	76	130	0	0	0	0	0	0
ddfe175ec4ef917f04639b15827c7d5a	0	0	0	0	0	0	0	0	0	0	8	0
08a82c1ae67d34e408229fce030f8c1d	28	0	0	0	0	0	0	0	0	0	0	0
a9e8ee5753e1f4f407a0d9f5c780e19a	0	0	0	0	0	0	0	3	0	0	0	0
65ed9e9a712a96dc1e5d10ace419ca85	0	0	0	0	0	0	0	0	0	0	19	0
c8b6ec44ff9868fe07d3f0586a65ee95	0	0	0	0	0	0	0	0	0	0	0	11
6f43bf3855474aa80228e820ebfce61a	0	0	2	0	0	0	0	0	0	0	0	0

515fb64c5ac9230f02e564ba832228e6	4	0	0	0	0	0	0	0	0	0	0	0
42426e35b51f8fc9e0411e0c6b3699c2	0	48	0	0	0	0	0	0	0	0	0	0
703447cbb3106cb80c56d54704c5aaa1	0	0	0	0	0	0	0	0	0	0	5	0
55df621d4804039ab6cfb4e7a3a16fb6	0	0	0	0	0	0	78	0	0	0	0	0
b987183393c77688a4ebac38d7ebccb9	0	7	0	0	0	0	0	0	0	0	0	0
23505e733e1a489c00ecb8f1da7f8e5f	0	0	0	0	0	0	0	0	0	0	3	0
8cf3cb0c82afdf9109eae48baf90d064	0	0	0	0	0	2	0	0	0	0	0	0
53c2bc737d43c6c2285749efa36847ac	0	0	0	0	0	0	0	0	0	0	0	5
6f00fa0d2447bf15d7b3e5ba253e9097	0	0	0	0	75	0	0	0	0	0	0	0
84998fef259892594ce5d8ce1607d4e2	0	0	0	0	0	0	0	0	0	3	0	0
1e62fa14d9ce6fe69e0f17b9c129d6fe	0	27	0	0	0	0	0	0	0	0	0	0
6625a150aba0d72dbf3c2d0f85f47c8f	0	0	2	0	0	0	0	0	0	0	0	0
614996ca6fc8a4360f994f4d398ba4e7	0	0	0	0	0	4	0	0	0	0	0	0
27859e57533115db9c0ea27e6a8d2a84	0	0	0	0	0	0	0	0	0	0	4	0

a15134c16ab4717da2971f39d396f9f9	0	0	0	0	0	0	0	0	0	0	4	0
4dac81430aa1c59f8d53fc4339fb4bcf	0	0	0	0	0	0	0	0	0	0	3	0
47a11dcb99d5aa9cb6c9880953171d48	0	0	0	0	0	0	0	0	0	0	3	0
2f89ccd3293c2db347bb130496514e72	0	0	0	0	0	0	0	0	0	0	8	0
2cfafad9a57e2426b90038a2c198fe7b	0	0	2	0	0	0	0	0	0	0	0	0
77a4ae8a579eb0331c3a88c856f43def	0	0	0	0	3	0	0	0	0	0	0	0
2bdecc07bb0cdda84cd5d95b1f642f1f	0	0	0	0	0	0	0	0	0	4	0	0
73b538c99402f8d66f955156775128fa	0	0	0	0	0	0	0	0	3	0	0	0
7c607708088729707629e983fd4a109c	0	0	0	0	0	0	0	0	0	0	5	0
3c7c0ced5cb8eca427c2c60e6aa77181	0	0	0	0	0	0	0	0	0	0	11	0
eeb157cf9c32b9b07993cd641e3d1cc7	0	0	0	0	0	7	0	0	0	0	0	0
70adcc215caeb8666cc7e6b2d44226ae	0	0	0	0	0	0	0	0	0	0	2	0
f67561b2a4be5f86913005da679b0591	0	0	0	0	0	0	0	0	0	0	0	16
0dd8974fd296681245617c4f5e56da25	0	0	0	0	0	0	0	0	0	0	3	0

9883fb82348210a93588be355cef0d7c	0	0	0	0	0	4	0	0	0	0	0	0
b8830347fc529c51851234c02a020232	0	0	0	0	0	0	0	0	0	0	24	0
74603b5c66a5ef04a5852f4d026b671a	0	0	0	0	0	0	0	30	0	0	0	0
9c748b879c2f35d58bd294ed7c65750f	0	0	0	0	0	0	10	0	0	0	0	0
3649cd1ddc94ab79f191fcf5b4502723	0	0	0	0	0	0	0	0	0	53	0	0
15c91729a753438f4255c3e81d6a4b47	0	0	0	0	0	0	0	0	0	0	8	0
23a5177b487438be75fd343729830b5b	0	0	0	0	0	5	0	0	0	0	0	0
35b6002cf2d6612ff84296e969f09c5e	0	2	0	0	0	0	0	0	0	0	0	0
f51c74fe4d8ec22ea868d3076607857d	0	8	0	0	0	0	0	0	0	0	0	0
4499ced35f375ff4e2f70a049574e083	0	0	0	0	0	0	0	0	0	0	0	11
0b7dfb12e57846dec9619a974b34d41a	0	0	0	20	0	0	0	0	0	0	0	0
d855705ff582bb50623496b39d25e92d	0	0	0	0	0	0	0	0	2	0	0	0
8da813b65ec1254cc9c7419579906b12	0	0	0	0	0	0	0	0	0	0	13	0
378f1eaebe46debca0831f89ef963213	0	0	0	0	0	0	0	12	0	0	0	0

ffb4c6804331fc7f860398d1e08047fb	0	0	0	0	0	0	0	0	0	0	0	3
8f607b813dcf0ef89ed50092ae11fe23	0	0	0	0	0	0	0	0	3	0	0	0
421b357edd2de0eb9b03d4e47abde03f	0	0	0	0	0	0	6	0	0	0	0	0
5f2357581225d6b33d3bf76cf31def11	0	0	0	0	0	0	0	0	0	0	5	0
4e3fac6abfaff2149f7798f9dc9b89c2	0	2	0	0	0	0	0	0	0	0	0	0
d108342778646fe578c1cf790091e1af	0	0	0	0	0	0	0	0	0	31	0	0
5b324b3e3bd9661f4c5df158632e38db	0	0	0	0	3	0	0	0	0	0	0	0
a677730f7239d41654b2e54276622251	0	0	0	0	0	0	0	11	0	0	0	0
9a7de61bb6a61a5751129a5d9be2ddfc	0	0	0	0	0	0	0	0	0	0	4	0
939d240e4c23e987761506d40086fd78	0	0	0	0	0	0	0	0	9	0	0	0
2148fd1f5a179a1f870f6e6123601ed6	0	0	0	0	0	0	0	0	0	0	6	0
fee50a4be3621480e247495ba8aa4b67	0	0	0	0	0	0	0	0	0	0	2	0
a2c472e5f72883858a8b27f0203309a6	0	0	0	0	0	0	0	9	0	0	0	0
2ffe58803c28886a07d604a31a205fc7	0	0	0	0	0	0	0	0	0	0	2	0

c4ac102cec3f2a3c214cde39bd51b492	0	0	0	0	0	0	0	0	0	0	26	0
77e93e8e51d2c1225fa040edc6b8d701	0	0	2	0	0	0	0	0	0	0	0	0
a9daea1d62c81bbb232f3ce2f53ac50f	0	0	0	0	0	0	0	0	0	0	2	0
5c65e433cc8863fc2fd49820a104b345	0	0	0	0	0	2	0	0	0	0	0	0
6d45c16e9640c1afeafabeaf435e51cb	0	0	0	0	0	0	8	0	0	0	0	0
c810ff2f4be19bf1ae96928dccf426c3	213	0	0	0	0	0	118	304	0	0	214	0
12ec0b8583a273444af0404c8ab383bc	0	0	0	0	0	0	0	0	0	5	0	0
7f0a9dc86f8c5b00060f8a2af4a0a895	0	0	0	6	0	0	0	0	0	0	0	0
459dac4401ab88208e616a7bbdf42584	0	0	0	0	0	0	0	0	0	0	0	35
35bc240dbf76132bd9810147cec92a12	0	0	0	0	0	6	0	0	0	0	0	0
f79fc33c897d0066fff6234c15716c20	0	0	0	0	0	3	0	0	0	0	0	0
b5f3c1af469f3d0d779e6862c9b44c6e	0	0	0	0	0	0	0	0	0	0	0	106
8166fdf022ebe462356485932c4bf869	0	0	0	12	0	0	0	0	0	0	0	0
b0b7de072cc7fd8a0820c0ded1dfb184	0	2	0	0	0	0	0	0	0	0	0	0

2e74db82321286284326dd9626b65e88	0	0	0	0	0	0	0	6	0	0	0	0
a0d552ed01a2cd50a67d6f44aee89787	0	0	0	0	0	0	0	0	0	0	8	0
92b40c2f5493657de6ea44849679e111	0	0	0	0	3	0	0	0	0	0	0	0
47b90201f4b51e25b8026b6f728bba4b	0	0	0	0	0	0	0	0	0	24	0	0
efeb31b0ed278d1124c0c0b98c65379a	0	0	0	0	0	0	0	0	0	3	0	0
b4611295523a6c0f0564c5d5d9ec64f3	0	0	0	0	0	0	0	0	0	0	2	0
c51bb791b0af931503a85f4918d27b14	0	0	0	0	0	0	0	0	0	0	0	3
bb8518e616635c57d54173a0875b0561	0	0	0	0	2	0	0	0	0	0	0	0
e77e8d7c3cd112e772c6a5e56d03e41b	0	0	0	0	0	0	0	0	0	0	26	0
a74902bd5b310aa09bc4ecaf948a3270	0	0	0	0	0	7	0	0	0	0	0	0
551b868ad5fe222a16a2be0c3b25f98f	0	0	0	3	0	0	0	0	0	0	0	0
d0ca7b5f4e10cdd7de9bccc1cf010444	0	0	0	0	0	11	0	0	0	0	0	0
8cc5d88cf6fefc1556d12948cbcb20dc	0	0	0	0	0	0	0	0	0	0	7	0
b6e9180f049f7b265071c0c86f7e03db	0	0	0	0	0	0	0	10	0	0	0	0

ebcb15b3f29043f80cc4a122 7074ce0f	0	0	0	0	0	0	0	0	0	2	0	0
87f6a51012a318f2dc827ba4 cb7d18e1	0	0	0	11	0	0	0	0	0	0	0	0
5f9b18627b6d5ca8a9af5a16 4daa3b37	0	5	0	0	0	0	0	0	0	0	0	0
a47d3b6d4cc55e0aee44281d b4aabb34	0	15	0	0	0	0	0	0	0	0	0	0
2a1c44d8a85b3b9b74780c9 4708be75d	0	0	0	0	0	0	0	2	0	0	0	0
c9b4d3c92b12a06df60d152d abe652d7	0	0	0	0	0	5	0	0	0	0	0	0
4490693b398227ddcc39488 271d91f66	0	0	0	0	0	0	0	0	32	0	0	0
06ecae3715f8aab1f0879d15f 4d4172e	0	0	0	0	0	0	0	4	0	0	0	0
bf60f8d28d8fbfa3115bf8734 7243b62	0	0	0	0	0	0	0	0	0	0	6	0
098b7d427aeeac697c9f2e51 9559a009	0	0	0	0	0	13	0	0	0	0	0	0
57618cafed917aeca51198ce e5e017db	0	0	0	0	0	0	0	0	0	0	0	31
c5f382f49f2f498073f72cddd 7a1ff7f	0	17	0	0	0	0	0	0	0	0	0	0
fb5401b60593970cb76ad935 a24cfb30	0	0	0	0	0	0	28	0	0	0	0	0
709d356a30e2af598404d1be 330c3c77	0	11	0	0	0	0	0	0	0	0	0	0

a33b6be5e8a8de13dd55807c5e907d78	4	0	0	0	0	0	0	0	0	0	0	0
d8abf9a035aec91f31919c566477be9d	0	30	0	0	0	0	0	0	0	0	0	0
46ebeb9e10753270bc50235b58cb1617	0	0	133	0	0	120	144	0	0	0	165	0
d8c4265e22f77ebf1cfdb70b772455e5	0	46	0	0	0	0	0	0	0	0	0	0
d28d9322c66f377391bdbe92f4185ef0	0	0	0	0	0	0	0	0	3	0	0	0
f1245e5818fa8d65a068704e13c8240a	0	0	0	0	4	0	0	0	0	0	0	0
17d469923d6eb4575c64c6509752c797	0	0	0	0	0	7	0	0	0	0	0	0
f02fee0ad5289294bcd6417d8acd6341	0	0	0	0	0	0	0	0	2	0	0	0
da27b0555d0f10e44f249b970f891756	0	0	19	0	0	0	0	0	0	0	0	0
221f61795635d422b9495dbd37228a53	0	0	0	0	0	0	0	0	0	0	26	0
21a1e78a4843da54afb7e1eb2cd8b4cd	0	0	0	0	0	0	0	0	0	0	0	4
f7807646f6dac60b3382e95f6c8ee89a	0	0	0	2	0	0	0	0	0	0	0	0
ee71f8c0beec0e3de9b1b897290c8ece	0	3	0	0	0	0	0	0	0	0	0	0
e38be1b0962ea35966a9eadd8e786bdf	0	0	0	0	0	3	0	0	0	0	0	0

7db46ef5f3d294d6c6bdc9c0ae8b202a	0	0	0	2	0	0	0	0	0	0	0	0
032b514d61f45cfc52f57f1c3f75560c	0	0	0	0	0	2	0	0	0	0	0	0
11efd5cede7ee56c8c3b973deaa58b36	0	0	0	0	0	0	0	3	0	0	0	0
5c234dfffd686a141f78bd7b77a9d7f0	0	0	0	0	0	0	0	0	0	0	0	7
0f48a15326c986443e9e1b86b4661061	2	0	0	0	0	0	0	0	0	0	0	0
945d18e75b0558a0d4fd6cd4e02b829f	0	0	0	0	0	0	0	0	0	0	7	0
db088801a6875332c2d6d7e8a68d829b	0	4	0	0	0	0	0	0	0	0	0	0
7e0040c9f0b357eb685cbcfd1f86cf7c	0	0	0	0	0	0	0	0	0	2	0	0
478fb692b050aa47c44c6daa5d8a5ac6	0	0	0	0	5	0	0	0	0	0	0	0
e149e77ff9f6f459b833530c9c8f65f2	0	0	0	0	0	0	6	0	0	0	0	0
aeb6b4ac3d719eff707453a21486f03f	0	0	0	0	0	0	0	0	0	0	3	0
f6461c53a72609c9b78754500a0cae5b	0	0	0	0	0	0	0	0	0	0	0	78
27d09074bda4b1765daaf83b78dfb327	0	0	0	0	0	0	0	0	0	60	0	0
2af6c2bcdc87656dad72bd7fc4ac1287	0	0	0	13	0	0	0	0	0	0	0	0

a662d06d3e6e282a0d2fc63a3634e7ee	0	0	0	0	2	0	0	0	0	0	0	0
d8c8343fa78eca34043c12e7155d908f	0	0	0	0	0	18	0	0	0	0	0	0
b92655e58f3eaade40502c7615d3769c	0	0	0	0	0	40	0	0	0	0	0	0
3166e482b304864a0c1918b6b86de7e8	0	0	0	0	0	0	0	6	0	0	0	0
5f9720ac6ff29ecf9afbd25a508cf673	2	0	0	0	0	0	0	0	0	0	0	0
461ba9941a23cad031db575f09627a83	0	0	0	0	0	0	0	5	0	0	0	0
4bd8060e276fe4b274dd293fc4fb60d7	0	0	2	0	0	0	0	0	0	0	0	0
b1981f859bc779ce2d37fc1ee672b519	0	0	0	0	0	0	0	5	0	0	0	0
62dbff75bf6513b0abaf91ac6cc82988	0	0	0	0	0	0	0	0	0	0	0	7
20621b098d9251a406e911d78f8b046a	0	14	0	0	0	0	0	0	0	0	0	0
fb3824dd20e28b987631334615f159a9	0	0	0	0	0	0	0	0	0	0	3	0
cd02755830b8131acf871c124139364c	0	3	0	0	0	0	0	0	0	0	0	0
5a75da6817b9d68c9352939e503df4f2	0	0	24	0	0	0	0	0	0	0	0	0
da0afe2b21f594ae56df49ff30c867e9	0	0	0	0	2	0	0	0	0	0	0	0

7f87920071ec8377aeb53244 4051698b	11	0	0	0	0	0	0	0	0	0	0	0
7e9d0e8ab1e7c5d0c8579e7e ed75c728	0	0	0	0	0	0	4	0	0	0	0	0
c11640cbd033ad8286a6cc0b 767a0efb	0	0	0	0	0	20	0	0	0	0	0	0
288351de320d866cf62eff9a5 1e7a946	0	0	0	2	0	0	0	0	0	0	0	0
ab7e67c8f04f971ff1f89a972f dea411	0	0	0	0	0	0	0	0	0	0	2	0
e5f7613f81c70850547d7e64 51c50d13	0	0	0	0	0	0	0	0	0	8	0	0
eeee2fc97762fcefb6912210d 6b8562f	0	0	0	0	0	0	0	0	0	0	2	0
f458c75c459e9f3b7e477111 8da0ab7e	0	0	0	0	0	20	0	0	0	0	0	0
76883f9c688c153355d574b2 0fd4636e	0	0	21	0	0	0	0	0	0	0	0	0
1f749cedbe9ae38f06f771e33 54cd1cc	0	0	0	0	0	0	0	10	0	0	0	0
977eefaa6359b75b37a1a29c 87764277	0	0	0	0	0	0	0	0	0	0	0	2
77a6383b8e0d26ca99a014b 74a0c0d65	0	0	0	0	0	0	0	0	0	3	0	0
4c84c7a3c9f1ee9dc821780a cccb5c57	0	0	0	0	0	320	0	0	0	0	447	0
8b40cf2db9d3fc247cf4b32e4 2a726c3	0	7	0	0	0	0	0	0	0	0	0	0

934cde06a85c937499c9a630e74401a0	0	0	0	0	0	0	0	0	2	0	0	0
f1f54daae5eea3c9baf5dcb30f95aca4	0	0	0	0	2	0	0	0	0	0	0	0
fe9e21b5c5abbdc94d07a3ef9ab26a2b	0	0	0	0	0	0	0	0	0	0	16	0
9c838b00a966e85d34faabe6371dd8df	15	0	0	0	0	0	0	0	0	0	0	0
0daa2696728f0990a0d56a949feb1c38	0	0	0	0	0	0	0	0	0	0	88	0
2a6415583180e6a0f79d52fe12df13cc	0	6	0	0	0	0	0	0	0	0	0	0
5c010abe75c8cdbae3be362526e85495	0	0	0	0	2	0	0	0	0	0	0	0
aa9c37fca42caddbba12c9d6da71141c	0	0	0	0	6	0	0	0	0	0	0	0
2699a66f3e07ddea7064d760326af3c1	0	0	0	0	0	0	0	0	0	0	0	6
b6ada00e67a1f168c3395a791b710f22	0	0	0	0	11	0	0	0	0	0	0	0
315d908571d405de505a38f1022592ef	0	0	0	0	0	0	0	0	0	3	0	0
4ed9daa88a1b00dffe7215638228cadb	0	0	0	4	0	0	0	0	0	0	0	0
48e97b54ef7f5c55f9c29ff4c0e4c35a	0	0	0	0	0	0	0	3	0	0	0	0
5d525c834a8449a3ec42e5f5aa32c6ab	0	0	0	17	0	0	0	0	0	0	0	0

3c923c393d1794965bb0eea5d570df44	0	0	0	0	0	0	2	0	0	0	0	0
66848334c8f1ca59fa307b39d3963da0	0	0	0	0	0	0	0	0	0	23	0	0
766c244ba9e6aa61873cf2b244b60df7	0	0	0	0	0	0	0	0	0	0	4	0
fa1321446ac3db3de15b5595a4af9a69	0	0	0	0	0	46	0	0	0	0	0	0
dddfd9c88fc586a5e84001fd9e789262	0	0	13	0	0	0	0	0	0	0	0	0
197ec91d8e2cba6d7a3acc44fc768e7a	0	0	0	0	0	0	0	0	5	0	0	0
6d5bcabe7011e5c3dadc5894a078c4ac	0	0	0	0	0	0	0	0	0	9	0	0
5d64563eccbddb268b6c45a32f27674f	0	0	0	0	0	0	0	0	0	0	19	0
562eeb78bc1cc1a2f6bab480dae7604e	0	0	0	0	0	0	0	0	0	0	0	11
5d682940be15dfc7633f5c6a1ea0b09a	0	0	0	3	0	0	0	0	0	0	0	0
d1591c0c82a9016be967ce03b281ffd9	0	0	0	0	0	0	0	9	0	0	0	0
63e3c46139061c3ab741b2ff1ce5db6f	0	0	0	0	0	6	0	0	0	0	0	0
c7666cbd7704c1f2f701f93349e4884e	0	0	0	0	0	0	0	0	0	0	4	0
8351b977cfdcc83b851cbbbc531411dc	0	0	0	0	0	0	0	0	0	0	0	128

57b6b54ef5a678467bbbac568b02bebf	0	0	0	0	0	0	0	0	0	0	4	0
110c85e70c27a6df120063d545bb07aa	0	0	0	0	0	0	0	22	0	0	0	0
c89a50d0469e82c53d14001899d62098	0	0	0	0	0	0	9	0	0	0	0	0
757681708eb90e4a987f13a25825fff6	11	0	0	0	0	0	0	0	0	0	0	0
af7ecd9b2bf8a32c837459615fe3079f	0	0	0	0	0	0	3	0	0	0	0	0
953b501f0ef6d693fd4a43f90a08a352	0	0	0	0	0	0	0	19	0	0	0	0
9a2a8a22effca4435d3096038985d1fe	0	0	0	0	0	0	0	0	4	0	0	0
32f1092da254dc6b12bc8a8f5142d106	0	0	0	0	0	0	0	0	0	0	38	0
465d1bd44ed2c8c823243d426dfbd1ce	0	0	0	0	0	5	0	0	0	0	0	0
a681970c8a0b48d3008bfd4c0d4c9a39	0	0	0	0	0	0	0	4	0	0	0	0
8847d56bca09e120308492273f31d59f	3	0	0	0	0	0	0	0	0	0	0	0
155d1b0012a720fd59f551b8ddb00ce6	0	0	0	0	0	0	0	0	0	0	8	0
a3a973830de072d2ec6921972208310a	0	0	0	0	0	0	3	0	0	0	0	0
1d43f3f8e8720989f4142460c706bff5	0	0	0	0	6	0	0	0	0	0	0	0

34d9c814b24bca6fe662b4e69f61992d	0	0	0	0	0	0	0	0	0	0	12	0
7319931012172c89951610027ff4bff3	0	6	0	0	0	0	0	0	0	0	0	0
18c068e6d6584f1f3da0e5ffa36ea594	0	0	0	0	0	0	0	0	0	0	2	0
debc6898cb97c24a0ab808e77d5f71e4	0	0	52	0	0	0	0	0	0	0	0	0
44d0da9b189de65232d2b957cf66b62c	0	0	0	0	0	12	0	0	0	0	0	0
1fa2c2f41d5dc8cb93c3fc5d594621be	0	0	0	0	0	0	2	0	0	0	0	0
1dc375a97ba607264566d393d15ad378	0	0	0	3	0	0	0	0	0	0	0	0
b037bec1c8d304c3226cd05e83fddde3	0	0	0	0	0	0	0	0	0	0	3	0
74c9b22ba329db7f74d0bfcd841f4c3c	0	0	0	0	0	0	0	0	10	0	0	0
27d846e73bf147dbceb459bcf13d671a	0	0	0	11	0	0	0	0	0	0	0	0
ec046ff659045d34ff388ae1904fe375	0	0	0	0	0	0	0	34	0	0	0	0
a78b5dc8fb1202220f2bbc3a127f9dfd	0	0	0	0	33	0	0	0	0	0	0	0
69e1048baad6ca1243fc681b2fd54330	0	0	0	0	0	0	0	0	0	6	0	0
dd94a506f9b8afd88e99c28a77e2d6fc	0	6	0	0	0	0	0	0	0	0	0	0

a6d9f973b9e0af005a1cbd36c28a3d3e	0	19	0	0	0	0	0	0	0	0	0	0
900ff4a1c6cd0449938f008170dc2d29	0	0	0	0	4	0	0	0	0	0	0	0
cd816cbc746955fd1546576992407b87	0	0	0	0	0	0	0	107	0	0	0	0
cd7f1597457506e8161eae8cfe3d5d07	0	0	3	0	0	0	0	0	0	0	0	0
2ee1f60b64cb9a936545932f24c26eba	0	0	0	0	0	0	0	3	0	0	0	0
60c2a4ef348f85d7e21ab1dc23070e5a	0	0	0	0	0	0	0	3	0	0	0	0
7fdf93e9d4a782ab616122c770ead723	0	0	2	0	0	0	0	0	0	0	0	0
9523fa877c3f990830fd882c06a0008c	0	0	0	0	0	0	0	0	0	5	0	0
13f3265b586fb36e7fcc303fa1269783	0	0	6	0	0	0	0	0	0	0	0	0
2070fe111b56e924aa034ae55c2914d8	0	0	0	0	0	0	0	0	0	0	35	0
fa6cea36c3c91ed52dc12cdd90f80427	0	0	41	0	0	0	0	0	0	0	0	0
7795d107644a42f5b650e27186bfb965	0	0	0	0	0	0	0	2	0	0	0	0
b79791fafe35eacec53944548bee5512	0	0	0	0	0	0	0	0	0	5	0	0
910bdbb959e445bac5ffd0677e6bf3d4	0	0	0	0	0	12	0	0	0	0	0	0

06de9b86815f08a652f3d83792e10dad	0	0	0	0	0	0	0	0	0	5	0	0
0fcd5d1aef94d02ed25418bdf8881a5d	0	0	0	0	0	0	0	0	29	0	63	0
2c594b9ab51374789a17e5c18397ae74	0	0	0	0	11	0	0	0	0	0	0	0
89bad159eb90bf39ab6286ca2aa23bf8	2	0	0	0	0	0	0	0	0	0	0	0
03ee2e026b8eb73ef3e8783f094371a1	0	0	0	0	0	0	0	0	0	0	3	0
fce7e2d8ecdee7da2f62975cf324c2f4	0	0	0	0	0	0	0	0	0	0	0	6
3dce0f88bac7e2d2a54cf90300497854	0	2	0	0	0	0	0	0	0	0	0	0
e9bcf96565716620ee86bd8c5755dbf7	0	0	0	14	0	0	0	0	0	0	0	0
2b292bf3bebb181aa0cf5027bfd6f36e	0	0	0	0	0	0	0	11	0	0	0	0
5599eda0966a0fb0a3de06d2e38ceece	0	0	0	0	0	0	0	0	0	0	36	0
c99b4d7dbc4f81b64f0151bc37752065	0	0	0	0	0	24	0	0	0	0	0	0
bdb55eb2e7ca585e1a81f786131d9c32	0	0	0	0	0	9	0	0	0	0	0	0
8136e9b942be93c72ef43fc2493ac32c	0	0	0	0	3	0	0	0	0	0	0	0
c1a2e64a40a232a64e1176261446f4b8	0	0	0	0	0	0	0	0	0	0	0	22

7a400591f95cd8a490474e3c2109ff16	0	0	0	0	0	0	0	0	0	0	0	26
5a35d39dc33ef2701a05a0cb5cd5fca4	0	0	0	0	0	0	0	0	0	0	0	13
b456a67fd762130fd3192f2903f66911	0	0	2	0	0	0	0	0	0	0	0	0
c024c5548024087e8415b0a35486722b	0	0	0	0	0	2	0	0	0	0	0	0
1c9f5939243cfe690c2e795ebee606cc	0	41	0	0	0	0	0	0	0	0	0	0
28c432fc4ce15d47fd6f24a2fc3c4554	0	0	0	0	0	0	0	0	0	0	0	10
8c5e5735abc5ea453a5b5aa67a6fb03e	2	0	0	0	0	0	0	0	0	0	0	0
de59c230290307e9d1c7f544d06b1aa3	0	0	0	0	0	0	0	0	0	4	0	0
e1749f2f6f46109c7681d6df5f91cf7a	0	0	0	0	0	0	0	0	0	0	7	0
896d8472ef2ed392023f11dfdf7799f1	0	0	0	0	3	0	0	0	0	0	0	0
bdcf29b0eb74d96d3bdff34e047e5ccf	8	0	0	0	0	0	0	0	0	0	0	0
7bc70c830338d1a47813bdc0eb85b28c	0	0	0	0	0	0	0	0	0	0	25	0
b4d344e5f01284442fb93ab3c5a3262e	0	0	31	0	0	0	0	0	0	0	0	0
a79a53eba18836e05c7a49dc21a91a6f	0	0	35	0	0	0	0	0	0	0	0	0

504074205297388ccf34cfe054ecaadf	0	0	2	0	0	0	0	0	0	0	0	0
47fc1f122659366f82a47a58f1607f7d	2	0	0	0	0	0	0	0	0	0	0	0
f10335ed9dfde991e1548d14b2a899bf	0	0	0	0	0	0	0	0	0	0	4	0
847ffaab1f2c101a488c7f5adc10b3b2	0	0	0	0	0	2	0	0	0	0	0	0
00b1252a0c447d4411202042c2df10ec	0	8	0	0	0	0	0	0	0	0	0	0
27160897409123c0ad29254620135c52	52	0	0	0	0	48	0	0	0	0	0	0
9035a9b162e79bd219b378158c56e747	0	53	0	0	0	0	0	0	0	0	0	0
9b361a47f6881e71a0e922266d6f05b7	0	0	0	0	0	2	0	0	0	0	0	0
84a6ea231465ac1ee361eddbeae9709b	0	0	0	0	0	0	0	0	0	0	8	0
6b0bd9ad96e83e3133f55f106a966572	0	0	0	0	0	0	0	2	0	0	0	0
c54f395b423c67b5608bd16adb9b1fce	0	0	0	0	0	3	0	0	0	0	0	0
efeeb025a98eff8a0099923cafc49b3b	0	0	0	0	0	9	0	0	0	0	0	0
3a8db99b0f577cf0760a1112a4be910a	0	0	0	0	0	12	0	0	0	0	0	0
1cab05e89dc0b9c76c36f343f1b83ac1	0	0	0	0	0	0	0	0	2	0	0	0

1757f5f1a48927c078afbf10f bf1baba	0	0	0	0	0	0	0	0	0	2	0	0
84b29ec052ed41536d221d8 0f0cf2242	0	0	0	0	0	3	0	0	0	0	0	0
96808526679cf35bccb9fdf0a 489c609	0	0	61	0	0	0	0	0	0	0	0	0
b99af7933ad8fcd9cd7771c7c 0584a0a	0	0	0	0	6	0	0	0	0	0	0	0
91258c4ea3fa998cbdda5e1a 792d4974	0	38	0	0	0	0	0	0	0	0	0	0
b2199f6e4ae302e2216264b4 f84651ae	0	0	0	0	0	0	0	0	0	0	2	0
0afd4fba537d23658f16d80ec 132ecfb	0	0	0	0	0	0	0	0	0	0	45	0
737530859bdf059b9aa56e6f 8663d938	0	0	0	0	0	0	0	0	0	2	0	0
363e01b5de716c138b28245 79928f626	0	0	0	0	0	0	0	0	0	0	4	0
d6bdf2bc13267c574f63d092 52dc8daa	0	0	0	0	0	0	0	0	0	7	0	0
fe48b5faed041dc1665baf2c9 ecbd356	0	0	2	0	0	0	0	0	0	0	0	0
ba1f3b017efa56a259f69b5c6 398e5cb	0	0	0	0	0	4	0	0	0	0	0	0
f4fbf334fc422b421157053f4 33de3c0	0	0	0	0	0	0	5	0	0	0	0	0
7665028d963cc9054b58a4ea 2a46f272	0	0	0	0	0	33	0	0	0	0	127	0

8f5922cd372ea3eb6d780414ebaae865	0	0	0	6	0	0	0	0	0	0	0	0
8a5eeed5075846ad90c624561f8fdfc2	7	0	0	0	0	0	0	0	0	0	0	0
fedc1abbec83bf7293e713177ac301c0	0	0	0	0	253	0	0	0	0	0	0	0
a0ca7a6f774e8cd03c640f35aa0a5ec3	0	0	0	0	0	0	0	0	0	2	0	0
b0dd5484b9212aecb66a175b6b817a3d	0	0	0	0	0	0	0	0	30	0	0	0
2b81b3775ae48f9702921d97e3a9b03c	6	0	0	0	0	0	0	0	0	0	0	0
2c4df6d390fe3e3ca5de5410fdc00d35	4	0	0	0	0	0	0	0	0	0	0	0
c8a49b2d92ab7523bf77e694ac595a0e	0	0	0	0	0	0	0	0	6	0	0	0
5c0467fa8581f5ebd2fc7fb9a4cac114	0	0	0	0	0	0	0	0	0	0	0	6
8ab5fa9282a6cb0e3d1b573fcbf8c17b	0	0	0	0	29	0	0	0	0	0	0	0
61538f3efc2bcd70a16804d91c0c226d	0	0	0	0	0	0	0	0	0	29	0	0
b3877662ea2181fdd3725e9939aef178	3	0	0	0	0	0	0	0	0	0	0	0
7b4a6ae7af28fed025971fda2e73f5b7	0	0	0	0	0	0	0	0	0	0	2	0
76ab0eb1d577dd841360a5a418be841d	0	0	2	0	0	0	0	0	0	0	0	0

468996c72fa77f2ea4304599f2950924	0	0	0	0	0	0	0	0	0	0	37	0
0d8566a3ba036d47e1134dd834f9125a	0	0	0	0	0	0	0	0	9	0	0	0
6b09df3af8755f3476d8a9143725e3af	0	0	0	0	2	0	0	0	0	0	0	0
3b75679840c41ab770bc1ad71ce7604a	0	0	0	0	0	0	0	0	0	2	0	0
d8ce78c41f04bb36830659419b8510b2	0	0	0	0	0	0	3	0	0	0	0	0
f1e6a01200da2316280a35b07512355d	0	0	0	0	0	0	0	3	0	0	0	0
1106eddbaef5ca91febba73d793e1be5	0	0	0	0	0	0	0	0	0	0	2	0
1b418308de371ae36a73bf614f58c57c	0	0	0	6	0	0	0	0	0	0	0	0
f00e857d0b30883dab2c2e9c54459bf5	0	0	0	0	0	0	0	0	0	0	7	0
58639211dc4694b2c87afb76017f58d2	0	0	0	0	0	0	2	0	0	0	0	0
b0793c7b15e3d22a9acca52d52f93fe1	0	0	0	0	0	0	0	0	95	0	0	0
0cd707a72a67a6710c8e405b2433f6e2	0	0	0	0	0	0	0	3	0	0	0	0
584d047dcbb0be8d8815cd79d98162ca	0	38	0	0	0	0	0	0	0	0	0	0
2b2710aedaa8369fc9300df24502f814	0	0	0	0	0	0	0	19	0	0	0	0

98866e1b6a6d243aaa835c1 9c73d016a	0	0	0	0	0	0	0	0	0	0	14	0
8cf948cd7adcaef2b09e037b 0490d2eb	0	0	0	0	0	0	0	0	0	0	115	0
c40fc767b58cacd8eb53c4c8d 7dc2d1b	0	0	0	0	0	0	0	3	0	0	0	0
8af9b4bb75f3b21c7b3ed72f 03e08058	2	0	0	0	0	0	0	0	0	0	0	0
53e57413d6c360078d9d95c a56eebeb3	0	0	0	0	0	0	0	20	0	0	0	0
a1ab01e0e7475c485a3b4cb7 7e97e4d3	0	0	0	0	0	0	0	0	3	0	0	0
5716f0e15f34d31c58d35986 282c85d9	0	0	0	0	0	0	2	0	0	0	0	0
b897ca3d463dc9af0df6d435 1a658026	0	0	0	0	15	0	0	0	0	0	0	0
a68f109124b6a90fc975b6de b7899187	0	0	0	13	0	0	0	0	0	0	0	0
681f69ad58ecdb9a60d572b9 a54381a9	0	0	0	0	0	0	0	5	0	0	0	0
b140306c1703fb2d62a3eac8 9bb07867	0	0	0	0	23	0	0	0	0	0	0	0
2da30f6418328318155306bf 7237f05d	0	0	0	0	0	0	0	0	0	0	0	2
80242e79ddf4d6b41005a77 23a3463d3	0	0	0	0	0	0	0	0	0	0	0	2
44b7224461b616ff1d841ca4 84ddcdbf	0	0	0	0	0	2	0	0	0	0	0	0

9df6e8be490ef07da5c3b661ac6d468a	0	0	0	0	0	0	0	5	0	0	0	0
fead46b068df6d28642993b3d2c2de9b	0	0	0	0	0	0	0	0	0	0	0	5
192c0061d4a65aa325b9eca64fa4c8f1	0	0	0	0	0	0	0	0	0	0	0	3
079b7a472b7df79b8e666ede93a34b22	0	0	0	0	0	0	0	0	0	8	0	0
2c2d6fb2f808887a9aefa1e077c81ccc	0	0	0	0	0	0	0	6	4	0	0	0
91f299fa503c42f5f69aa41c6fc9cb06	0	43	0	0	0	0	0	0	0	0	0	0
35c274f19db8c00039cefd1567923cad	0	0	0	0	3	0	0	0	0	0	0	0
62ffdfa86027ea11390bf148bc454493	55	0	0	0	0	0	0	0	0	0	0	0
b33fb9058ecdd8ccc6669f8f7ccb5872	0	0	0	0	0	0	4	0	0	0	0	0
6c946f40f7d658e86a863e13efb2f19a	0	0	0	0	0	0	0	0	0	0	0	5
ba967d15dc2b3b8e9df1c6bd50841e2b	0	0	0	0	0	0	0	0	0	3	0	0
3c718f579bbf71536314f85eb153b0f9	0	0	3	0	0	0	0	0	0	0	0	0
dbc603214623a64e5a6bf8dca5cb0fd8	0	0	0	12	0	0	0	0	0	0	0	0
92672a53dad476b23934881527406bf3	0	14	0	0	0	0	0	0	0	0	0	0

1eb3d4cc42cc41cb22cb6676 7d685a72	0	0	0	0	0	0	0	0	0	5	0	0
ac285bfe0f626e09334f7c345 da22399	0	0	0	0	0	67	0	0	0	0	0	0
1a56709c9e6ef0f0f45cb347b a8fd2b8	0	0	0	0	0	0	0	0	0	0	0	4
e2977630eac53eb19ee995fe e246d2e6	0	0	0	0	0	2	0	0	0	0	0	0
b9b37e5a04d6e5662ecd4e1 0d74d2cdb	0	0	0	13	0	0	0	0	0	0	0	0
2c8a3a6f0f50ba5d11f123ef4 8b73901	5	0	0	0	0	0	0	0	0	0	0	0
87d4e32e07d63dfd0d9c6c11 3193600e	0	0	0	0	0	0	0	21	0	0	0	0
2fcfaf797db8e6ebf225a1622 62f8aeb	0	0	0	0	0	0	0	0	0	0	0	2
c99f940665a4cc01d8f4560fe 13dd2c9	0	0	0	0	3	0	0	0	0	0	0	0
a94e5e81f33b370a69f16ebd 2dad2148	0	0	0	0	0	0	0	10	0	0	0	0
db49b8db229d32335e1afa0 d11ed4443	0	0	0	0	0	0	0	0	0	0	18	22
50e36cf84665620a33d0ff20d 3ff0d84	0	0	0	0	0	0	0	0	34	0	0	0
7c8b9fa277f5103fd07ef0432 b12b23e	0	0	0	0	0	0	0	2	0	0	0	0
302687fd8025b1d46506ee2 be5d45f74	0	0	0	0	4	0	0	0	0	0	0	0

182c638f2ab1061a6b78ac4b8785b841	0	0	0	23	0	0	0	0	0	0	0	0
de7938bc58f61798c01077d9f1dbfb7e	0	0	2	0	0	0	0	0	0	0	0	0
09fb387d4cfebe9df2320168e11e2c26	0	0	0	0	0	0	0	0	0	12	0	0
c112f81d716acbc9de39e92e5583592c	0	0	0	0	128	0	0	0	0	0	0	0
21d343684054722eb65388872a239f3d	0	0	0	0	0	0	0	0	3	0	0	0
492581ef2828b20bd4f473feca485fb9	0	0	0	0	0	0	0	0	0	0	6	0
4bb63965f3c621d7bd09b0fe05edb6ea	0	0	0	0	0	0	0	0	0	0	0	7
595f4ef0df8cd959c9bfa982d3d3966f	0	0	0	0	0	0	0	39	0	0	0	0
57e508c0c571013012fe5a54c9d8a791	0	87	34	0	0	0	0	0	0	0	0	0
38f16c0e03398b8e1f315830d898d8e7	0	0	0	0	0	0	0	0	0	0	0	25
7ce98c7987f975029c39b1ab815260f9	0	0	3	0	0	0	0	0	0	0	0	0
c95a99151d4d460c011feeca64530015	0	0	0	0	0	0	0	0	0	0	0	6
8649c5d0c5379e9d60efb656db5674b2	0	46	0	0	0	0	0	0	0	0	0	0
206cb66706bf7b05c91422cb5a875e98	0	0	8	0	0	0	0	0	0	0	0	0

293a247cf5867c33b54551c2c8cf27a6	0	0	0	18	0	0	0	0	0	0	0	0
101628a8ce7999088c4b2c5430bf9e61	0	0	0	0	0	0	0	0	13	0	0	0
9221fa7712b25d07470e0f0d52956804	0	0	0	0	0	0	0	0	0	0	8	0
86459bdddce612f900c878cb3b3a440f	0	0	0	0	8	0	0	0	0	0	0	0
a22d143780e845a8532b24a950dccfc4	0	0	0	0	0	0	0	0	0	0	0	2
13256adcbe88ba5f16d53603cf697aa4	0	0	0	0	0	0	0	0	0	0	6	0
f3c53e61b8b0c73fd41dc19190f5958e	0	0	0	0	3	0	0	0	0	0	0	0
f79755ae915a66afeba55786d6fbc235	0	0	0	0	0	0	0	0	0	0	3	0
92f9c7eec965b1eb531a0c2f81e7b576	0	0	0	0	0	38	0	0	0	0	0	0
d904d5dc887d40960ee0f505a00d7a11	0	0	0	0	0	6	0	0	0	0	0	0
dd352b603828d507ac37e3b735cdbbfa	0	0	0	0	0	0	0	0	0	11	0	0
19dc446b59f351efdea86ac086c2b7ae	0	0	0	0	0	0	0	0	0	17	0	0
4b825166a9f001a7e15b4eea7d8b09b5	0	0	0	0	0	0	0	0	0	0	12	0
f114fc1e6dc2d20bd708ecb4c14a6b65	0	0	0	0	0	0	0	0	0	0	0	4

597b478604c5207f47ca1c6ebd2a4ead	0	0	0	0	4	0	0	0	0	0	0	0
2d0fc5f43ad838fd22c2e1a3762f3384	0	0	0	0	0	0	0	0	7	0	0	0
1a4e859bc903027bddf03e25203bbc2b	23	0	0	0	0	0	0	0	0	0	0	0
bc58c3d20ff18303c1fa0da1ba54537f	0	0	4	0	0	0	0	0	0	0	0	0
e610aff058de25806d503f04a98de804	0	0	3	0	0	0	0	0	0	0	0	0
0c960d48a21be08df9c9ec2980476263	0	0	0	2	0	0	0	0	0	0	0	0
c2566938352edbe39b481deb9d5bd972	21	0	0	0	0	0	0	0	0	0	0	0
9d24e9d87289d6c7821279656b4ba641	0	0	2	0	0	0	0	0	0	0	0	0
fde5bab6738b971afb687d69fd45bdfb	0	0	0	0	0	0	0	0	10	0	0	0
26a2200d3c0398905b27dd0f59f4cf13	0	0	0	0	2	0	0	0	0	0	0	0
3efbadb6264b21897efe96f259de18be	0	0	0	0	0	0	0	6	0	0	0	0
6c172f423489b2ea2742412888d918a2	0	0	0	0	0	0	0	0	0	0	8	0
394a6f9c6986145304b41b2075b03f64	0	0	0	0	0	0	0	2	0	0	0	0
f0029ed74794e206a9b8838f84da6b83	0	0	0	0	0	0	0	0	0	3	0	0

73817d99d06bd631539a2113b05537f3	0	0	0	0	0	0	0	0	10	0	0	0
a8ad9eac985511a7d0f4746c23dd477e	0	0	0	0	3	0	0	0	0	0	0	0
ea30faac404d2136da4ccc38d0fae9c6	6	0	0	0	0	0	0	0	0	0	0	0
ed610a45c1a390f45db73ed6cbf3374d	0	0	0	0	0	0	18	0	0	0	0	0
61cb63ebd86ff0fd1e0a490a1d86602e	187	0	0	0	0	0	0	0	0	0	0	169
649b66c65c348a751c4fb9cb948d083b	0	0	0	0	0	0	0	2	0	0	0	0
646f6bfed72778d5337a6376ded28e62	0	0	0	0	0	0	0	0	0	0	0	13
6c686c751e1cd041fd71e5262f0ef65f	0	0	0	0	0	0	0	0	0	0	0	7
377b44393839dfb83057dadbf652d1cc	0	0	0	0	0	4	0	0	0	0	0	0
e2f13191fa2febc27205c56a2753d1c1	19	0	0	0	0	0	0	0	0	0	0	0
9d5c85223060374ac26ca640e01d9b41	0	0	0	3	0	0	0	0	0	0	0	0
2ade5aec70c939367912d4a545d35fbc	0	0	0	0	3	0	0	0	0	0	0	0
e5aa19cc8565301586bd243348979cbc	0	0	0	0	0	0	0	0	3	0	0	0
2454600c7e7ddf80528e15278fb3e6f0	0	0	0	0	0	0	0	18	0	0	0	0

ab3f418b8d879574213d8597f3310b2d	0	0	0	0	0	0	0	0	58	0	0	0
7712dc7031f053c3540f5acd8ad6ff21	5	0	0	0	0	0	0	0	0	0	0	0
d807a46fc071c964bd024e11e287feec	0	0	0	0	0	0	0	0	0	0	0	215
e3372c47e5d6c8709daada9163bb6771	0	0	0	0	0	0	8	0	0	0	0	0
6d7964a60d0cddfd23233d432cb4eb24	0	0	0	0	18	0	0	0	0	0	0	0
43c0ee23af288221e3ec583cad2fa8f8	0	0	0	0	0	0	0	0	0	15	0	0
48be0731e3327abb1c0b0f654232f127	0	0	0	0	0	0	0	3	0	0	0	0
408e88f9300b841585165300b081fc72	0	0	0	0	3	0	0	0	0	0	0	0
4f3f71449275aeda7dd954f95100b7d6	0	0	0	0	0	0	0	0	0	0	3	0
6007e072d0eb80f06580214db6af39a3	0	0	0	0	0	0	7	0	0	0	0	0
039220e5c5d272f1d4f7347550ec21b0	0	0	0	0	0	0	0	0	0	0	0	42
22c298a8f1f44acc9c4d279f39d8065c	0	0	0	0	0	0	7	0	0	0	0	0
5b721194617f83d5c131d2e1177e16cd	0	0	0	0	0	5	0	0	0	0	0	0
573bca86e349570e2b82709ed36beb02	4	0	0	0	0	0	0	0	0	0	0	0

7c746ecc074633016aafe265e098b2d1	0	0	0	0	0	0	0	0	0	0	3	0
9f22c20156c44e03201ec3d6d316603e	0	0	0	0	0	0	0	0	0	0	3	0
d9780da715a0a593dc0ff59b7cd6531a	0	0	0	0	0	0	0	0	0	0	2	0
3482e0c11237662a90a1368196601c99	0	0	0	0	0	0	0	0	2	0	0	0
f977cc8bdaab7effe571f54a9b4ef78c	0	0	0	10	0	0	0	0	0	0	0	0
0ae9837ff9daed98b20aba37c9ba1a03	0	0	0	0	0	2	0	0	0	0	0	0
65b1322e016089dbaaafdd546c3e6703	0	0	0	0	0	0	0	0	0	0	2	0
3a7951b463fdce6627787c38a5ee256a	0	0	0	0	2	0	0	0	0	0	0	0
9b5c83992dac9342ba299b33a983f19b	2	0	0	0	0	0	0	0	0	0	0	0
4636f0e2d79ff28fd492b583e90f9981	0	0	0	0	3	0	0	0	0	0	0	0
5724b2d8c241a487525c66bb5c76f9f9	0	0	0	0	0	0	0	0	0	0	0	4
d0f61b821ce69669a7f7e3eacdcd5019	0	0	0	0	0	0	2	0	0	0	0	0
7e84393c680ae70dafd028ed247bc6d7	0	0	2	0	0	0	0	0	0	0	0	0
81210c0e7f42241f9f1bf4c16ca2d013	0	0	0	0	0	0	0	0	0	0	0	9

514262e8d5e2a193b040dd4e76b984c6	0	0	0	0	0	0	0	2	0	0	0	0
0854c3997e35a34f41fa259a0ebf63b6	0	0	0	3	0	0	0	0	0	0	0	0
3481c5ddc7ed6faf59e7287b6246f5f0	0	0	0	0	0	0	0	0	0	16	0	0
0390bc9fb26387dea42c178484dc4c1f	0	0	0	4	0	0	0	0	0	0	0	0
adf3168a72a8bf7aafa481be40161260	2	0	0	0	0	0	0	0	0	0	0	0
8f62f884eb39a5e580e73d31c0b9ef31	0	0	311	0	0	0	0	0	0	0	0	0
e87d50271c0f2c2f8f79876022bbc743	0	0	0	3	0	0	0	0	0	0	0	0
bdb2799ca0c50e3e051a9d1f808f2648	0	2	0	0	0	0	0	0	0	0	0	0
f33742c4ce7f4f2608f1b1b6d3363820	0	0	0	0	0	0	0	0	0	0	0	2
737f0adcc80c16f65f6fb233786993f5	5	0	0	0	0	0	0	0	0	0	0	0
8bdf6e3d9f4efde0723ef86c04dea688	0	0	0	0	0	0	0	0	0	0	0	3
d8266d11e149745c27776ae779211391	0	0	0	0	0	0	4	0	0	0	0	0
b557bbce7e58a1d4a83fb3061bc5fb00	0	0	12	0	0	0	0	0	0	0	0	0
90cf9e9e4de4ae7211563fb358489449	0	0	0	0	0	0	0	0	0	8	0	0

f100ed2bad5aa2f2d9f40b08 1b953847	0	0	0	0	0	0	0	0	0	0	2	0
eff3c900bf7c1714b05745dc4 833a0fb	0	0	8	0	0	0	0	0	0	0	0	0
fb1c5f78e25f4d8cc398ceacc bdf2ec7	0	0	0	0	0	0	0	100	0	0	0	199
9272373eb12ef8ab3a6bdad8 8e1bc5cc	0	0	0	4	0	0	0	0	0	0	0	0
59ab2216092f811f5d3c6b6d 53b04351	0	0	0	0	0	0	0	3	0	0	0	0
99b605f453368e7dbf23137c fb32c346	4	0	0	0	0	0	0	0	0	0	0	0
9dba2634121916830ce9cc83 3e014bc3	0	0	0	0	0	2	0	0	0	0	0	0
e2b8d33470a89bce1516011 7fbec058d	3	0	0	0	0	0	0	0	0	0	0	0
f5fb5ce04caa9149c553df362 274be61	0	0	0	0	0	0	0	0	0	50	0	0
30a3463aca4de1b0ee20d85 e1f26c7b0	0	0	0	0	0	0	0	0	0	0	65	0
21afbd1df09d16caf084e68c6 dcbcfd6	0	0	0	0	0	0	0	10	0	0	0	0
fb5dcdcf8eb8083645211ee6 85c6f964	0	0	0	0	82	0	0	0	0	0	0	0
2cd40441593b6991ecc15de7 bfe48a03	0	0	0	0	0	0	0	0	0	0	15	0
56d51b7a40463455d5e9302 020e16edb	0	0	0	0	0	0	0	0	11	0	0	0

2bf70919c769ea741642beb4ed59791e	0	0	0	0	0	0	0	0	0	0	0	5
2856e25248df40dc8c2d0d1b7d084e6c	0	233	344	558	191	0	496	997	127	582	399	545
780e9a2aa32252d36bfef42813b8b91a	0	0	0	0	0	0	0	0	0	0	0	3
926da7cf2e995ba412971566d7d31f10	117	0	0	0	0	0	0	0	0	0	0	0
41a6b3a1141616ef3427465c80781ff1	0	0	0	0	0	10	0	0	0	0	0	0
b5e6eefd33e0dbeade4f821a704a16c3	4	0	0	0	0	0	0	0	0	0	0	0
5d0813018ef8696cbff2e35dacf99866	7	0	0	0	0	0	0	0	0	0	0	0
d5a14956fad5b0e613ed127d99c5068c	0	0	0	0	0	0	0	0	0	0	0	3
3f518bf3d404b8b254ffd972cb4e0160	0	0	0	5	0	0	0	0	0	0	0	0
78dc9a310448096286e75fe3a9fb9b72	0	0	0	0	0	8	0	0	0	0	0	0
2c8699ea504559d6d8b4b22927dd029a	0	0	0	0	0	0	0	0	0	0	71	75
1ff6ae52e7f17551973bdc96efc6203a	0	0	0	0	0	0	0	3	0	0	0	0
b92b0439ef4f432594abe89f6e99d5f2	0	0	0	0	0	0	0	0	6	0	0	0
793cf980daeaf752384ec5bc03158cc8	0	0	0	0	0	0	0	0	0	6	0	0

64f396830e8ea7ef3a6abead d5aa7a69	0	5	0	0	0	0	0	0	0	0	0	0
2d231457343c7ac5fdfabb43 4e9c5a18	0	0	0	0	0	0	0	0	2	0	0	0
d6a35a807eaaf9b5a02de853 14949ea6	0	22	0	0	0	0	0	0	0	0	0	0
66edbfeb383121a1362951cd 74c3f52f	0	0	0	0	0	0	0	0	3	0	0	0
993d8286364602ebee0a804 42882bcaa	0	0	0	0	0	0	0	0	0	0	2	0
83372d4da56286e264fda0e1 a94f4836	0	0	0	0	0	0	0	0	0	0	4	0
b3b05cb54e937c2ef791a7dc 5a728f0c	0	0	0	0	6	0	0	0	0	0	0	0
e24239b2ed00c6b922bd952 eacf14a78	0	0	0	0	0	0	0	2	0	0	0	0
c9f19316d8c15b4f69ad5e04 651ba081	0	0	0	0	0	0	0	0	0	0	0	62
8fdd2793c4e413de464cc152 2f6b6e82	0	0	0	0	0	0	0	5	0	0	0	0
1c4c68f1df821e4bef32fc1cce e0d2ce	0	0	0	0	0	0	0	0	0	0	0	3
02c2f3a875bda52d4b7953d0 765be1f3	0	0	3	0	0	0	0	0	0	0	0	0
fc53b27d88825e2aaaad132ff 23af343	0	18	0	0	0	0	0	0	0	0	0	0
4c7581cd24bf02614810813a 7dfe16ac	0	0	0	0	0	0	0	17	0	0	0	0

50a111bed8730f7bd88cbf28 9c08ec97	0	21	0	0	0	0	0	0	0	0	0	0
e8201908cdb9b22188cdc40f 8b691cf7	0	0	0	0	0	0	0	0	2	0	0	0
3846574e24a45d7c49dd4cb 7b4809166	0	0	0	0	0	15	0	0	0	0	0	0
b72ccaaeb096652b1f64bbd4 3ff11572	0	0	0	0	0	0	0	0	0	0	20	0
513c45d5fb37577a288613bd 02287e4c	0	0	0	0	0	0	0	0	0	0	0	4
4e2345b2e9f6abe9ca512152 d5c99524	0	0	0	0	0	0	0	0	2	0	0	0
099fc5d52f8e4c974e9c546d bb24acf4	0	0	0	0	0	0	0	0	0	2	0	0
98768f837e4d7833995ea540 47a45eef	0	0	0	3	0	0	0	0	0	0	0	0
a1d2385d4239e846ec4f5da8 fa1a672b	0	0	0	0	0	0	0	0	0	0	3	0
7e56f13c0259d770131e85c8 156f07c7	4	0	0	0	0	0	0	0	0	0	0	0
bb80eed76d98e71d76ea466 c35557f0b	0	0	0	5	0	0	0	0	0	0	0	0
d64e00d2a290d78d4eea790 ae5d6cb38	0	0	0	0	0	0	0	2	0	0	0	0
2e8f1917771196e9d1d3e9f8 9fb2b1aa	0	0	0	0	0	0	0	0	0	2	0	0
97713ec57a583b8b3a5318b 9a98539aa	0	0	0	0	0	0	7	0	0	0	0	0

57219e9883b7a4dec3f1a93d578315c1	0	0	0	0	0	113	0	0	0	0	0	0
82bb2ba8b81334f522b2b74eb891b41a	0	0	10	0	0	0	0	0	0	0	0	0
cc4bffb1bdc63001b4f75808fea808ba	0	0	0	0	2	0	0	0	0	0	0	0
c0dc00152c6a28a02c1dc324b58a3dec	0	0	0	0	0	0	0	0	0	2	0	0
4c7b632e6a869aa9623e0f76a368ce62	0	0	0	0	0	0	0	60	0	0	0	0
8ab153c24336a9b5cec4889e1031d7b5	0	0	19	0	0	0	0	0	0	0	0	0
85a5e19ed8203ed5bd089777fc4d54c4	0	5	0	0	0	0	0	0	0	0	0	0
a5417ea1fa6d55ccc29d07fff91b4756	0	0	0	0	0	0	49	0	0	0	0	0
adc4d00e491622e829a67596269bba4d	0	0	0	0	0	0	0	4	0	0	0	0
697fe20133d6671395977496f8d7f015	0	0	0	0	0	0	0	0	0	0	8	0
4214cd01978b7c90d31a78d8185e54fa	0	0	0	0	0	0	0	0	0	0	18	0
1d92303af9c0b4dd9c3c3f7606936d4d	0	0	0	0	0	0	0	0	0	0	0	4
3c4cd1e9b2b6c8c6b4b36576d3d4b5ea	0	0	0	0	0	0	0	0	0	115	0	0
221b9c001e86396bb8e7f253e724862e	0	0	0	0	0	0	0	0	0	0	0	2

f500a56a9600232d5f876f6d2d75fd6a	0	0	0	0	0	0	8	0	0	0	0	0
fae3d8fcbc4510c97a73b94e69131b6f	0	0	0	0	0	0	0	0	0	0	3	0
3ed8e3830ed732eed00aa53a64b41924	0	0	0	0	0	0	0	0	0	8	0	0
fd52127650fe77f55d13e23f8a670383	0	0	0	0	0	3	0	0	0	0	0	0
267ed6a8241c7017d9162b7edfeb6b1c	0	0	0	0	0	11	0	0	0	0	0	0
058bcac53f26d77dca02690314bab357	0	0	0	0	0	0	0	0	0	0	2	0
ba29b29045db729fa99a72f57b6c64c3	0	0	0	0	0	0	0	0	2	0	0	0
deef8f7336cfb10e440a6137dc7dbe90	0	0	0	0	0	0	0	0	0	0	3	0
c84157af00c3f634fac77e4e28004bbe	0	0	0	0	0	0	0	0	0	0	2	0
5de3fa78620d3acb0e6f364116caa020	0	0	0	0	0	18	0	0	0	0	0	0
80e01883ab1a7551021ec1c717148234	0	0	0	0	0	0	0	0	0	0	0	3
382e63491917adef369dfd19cc9d0cf8	0	0	2	0	0	0	0	0	0	0	0	0
737c34d2de519c9a024e229114aa1a43	0	0	0	0	0	0	0	0	0	0	0	49
7fd14b2c17f83baa8566acc192e2a0d4	0	0	0	0	7	0	0	0	0	0	0	0

11f670ec737b41b55693d8ae a345912e	0	0	0	0	0	0	0	0	0	0	0	12
794ef388fe95c61a4e6a06df7 48a78b1	0	0	0	0	0	0	0	0	13	0	0	0
1f11a7990fc58a7c4b5a7357c 5da568a	0	0	0	0	0	2	0	0	0	0	0	0
a02dab2058588e323e8a3eb 20ad3ff46	0	39	0	0	0	0	0	0	0	0	0	0
2190f399dd9cc1f93f055cab7 1b450e5	0	0	0	0	0	0	0	0	8	0	0	0
952dcb42e231199a435f5490 92bfe064	0	0	0	9	0	0	0	0	0	0	0	0
9809e7d90893af60229f0b53 479f779c	0	0	0	0	0	0	0	0	0	0	0	3
005fc15299a3113b5bff97bf7 31cb522	0	0	0	0	0	0	0	0	2	0	0	0
d3aa23dfb6f510e9fae27165 5cda806d	0	0	0	0	0	0	0	0	18	0	0	0
7b8b0fce6d6d1d3b86712542 d737090e	43	0	0	0	0	0	0	0	0	0	0	0
25912b83eb1b74ee3aa5ba6 b177d6bcd	0	0	3	0	0	0	0	0	0	0	0	0
3fd54f388b94fe27a1deb0e0 7e2ce452	0	0	0	0	0	0	0	2	0	0	0	0
b475521fda194af738d687a8 a8435001	0	0	0	0	0	0	0	0	0	0	50	0
b74ce71096ad2b6d1cf6a8ef 28ad0c11	0	0	0	0	0	0	0	2	0	0	0	0

42a86db9851cc92573ff890b b11f11a6	0	0	0	0	0	0	0	0	0	0	14	0
8d778a7fbc214ed04c93f6be 71c7a89d	0	0	0	0	0	0	0	0	0	0	8	0
ebedb13d4029aeca51873ad 56542dd0b	0	0	0	0	0	0	0	0	0	7	0	0
7396bf91482c44b16b62d90e f483341e	0	0	0	0	0	0	0	0	0	0	3	0
f47c98acf981bdd22a1de007 6cd187b9	0	0	0	0	0	0	0	0	0	0	19	0
5cb0e86aad238d592924cecf 448c1361	2	0	0	0	0	0	0	0	0	0	0	0
1ff914ec063ef247f423a169ff 3cc5ee	0	0	0	0	0	0	0	0	0	0	0	2
8db30d6bae647d38d61dc7e 75bf0010b	0	0	0	0	0	0	0	0	0	21	0	0
9012a2007765ae6f973bf3dd 1ab50f93	0	0	0	0	0	0	0	0	62	0	0	0
5fc0999945d87429a40a994d c3c1e704	0	0	0	0	0	52	0	0	0	0	0	0
843f85962559109932659612 6226d890	0	0	0	0	0	13	0	0	0	0	0	0
30e6eaeefbdd7efdac2c2e4af 2e5553a	0	0	0	0	0	0	0	0	0	0	11	0
df4b5b4017c506200930f05e 4c762404	0	0	0	0	0	0	0	0	0	0	0	31
e4d90570349190de4104662 eea47e532	0	0	0	0	0	4	0	0	0	0	0	0

b7fad3e4ba55d3d7100d7b330dddfc62	0	0	0	0	0	0	0	0	0	0	3	0
53e5248dbb3da8c611d35058aa2a6287	0	0	0	0	0	0	0	0	0	0	0	29
effd6eaa3d5ff0771ea4dc963f470762	0	0	0	0	0	0	14	0	0	0	0	0
dd727839701ebc9a3308f03cecf945f6	0	0	0	4	0	0	0	0	0	0	0	0
c067d1e9ee59ed03d5b869e96144a637	0	0	0	0	0	0	0	0	0	0	0	4
15ed00b08a73beb9d51f0730faa2d318	0	0	0	0	0	0	0	0	0	0	10	0
31c088e6d782f087ebb268949ff0e554	0	0	0	0	0	0	0	0	0	7	0	0
99b32b66a3c5965dd59dd57c5abc7cd9	0	0	0	0	17	0	0	0	0	0	0	0
21e3123dbbf0717791dfa9aa2dc65beb	0	0	0	0	0	0	0	0	0	45	0	0
9641451cbcbc542f095ae030cc02fcf6	0	0	0	0	0	0	0	0	97	0	0	0
519b1dac0bd7add6908dfc37bd69094b	34	0	0	0	0	0	0	0	0	0	0	0
252023551bde74092b496112959bed4b	0	0	0	4	0	0	0	0	0	0	0	0
36a06c3ceeaf1a489a4bac0f822aba17	0	0	0	0	0	0	0	0	0	16	0	0
0354649c7a3896d0745257faabcadf23	0	0	0	0	2	0	0	0	0	0	0	0

b56006bed9aed5f997294ab9eaca8dab	0	0	0	0	0	0	51	0	0	0	0	0
1a905886f64a037872fd22df36c5d712	0	0	0	0	0	0	0	0	2	0	0	0
5b7f29e3669f30debd3a5aeef17ec198	0	96	0	0	0	0	0	0	0	0	0	0
7740d1546b84d2ece5f134fbdde1b0f9	0	0	0	0	0	0	0	3	0	0	0	0
a26b1b08595be0d7d69761628ecbda75	0	0	0	0	0	0	0	88	0	0	0	0
6e7085f6313424c7c14ae0f78200637d	0	0	0	0	0	0	0	3	0	0	0	0
9a8c5599306b924616e11cacaec6b4e5	0	0	0	0	0	14	0	0	0	0	0	0
77d76878a82849c6725d5fafcd1c4cc7	0	0	0	0	0	0	0	0	0	3	0	0
d8aec1cfc710dcff06475a025fd5ecd8	0	0	0	6	0	0	0	0	0	0	0	0
c4ca569343000c6dcca2502cd046fe07	3	0	0	0	0	0	0	0	0	0	0	0
2b0e649b834bef25e7912ea547f3da06	17	0	0	0	0	0	0	0	0	0	0	0
8ab94c9b6c2f1d805feb1724c2707e6d	0	0	0	0	8	0	0	0	0	0	0	0
fe76ecbf3f52b70ebd92786b4dfd1f30	0	0	0	2	0	0	0	0	0	0	0	0
4e7452c3fbbdebc06b62adbddfeb6fcd	0	0	0	2	0	0	0	0	0	0	0	0

0395d558bb37c2fb75961b55058b8766	0	0	3	0	0	0	0	0	0	0	0	0
c1584c369b934e1eedda1ea18e932c97	0	0	0	0	0	3	0	0	0	0	0	0
bb0bf95dd904a520b51709fd440f9309	0	0	0	0	0	0	0	0	0	0	19	0
af551800eb22e815e88dcf5b03af9cd1	0	6	0	0	0	0	0	0	0	0	0	0
38bf3eaf36941a80cdf4b5cf9a021e88	0	0	0	0	0	0	0	0	0	0	0	9
254530c2a7182d42be1309ff8cd22739	0	0	0	0	0	0	0	0	0	2	0	0
01dcd388cfe7aac8432ad6e10c653513	0	0	0	0	0	0	4	0	0	0	0	0
3680ec7a5e4df9be68c2db5e0e886f0e	0	0	4	0	0	0	0	0	0	0	0	0
dee16e38dfb2e6bd4bb43047800164fc	0	0	0	14	0	0	0	0	0	0	0	0
1abebaa889dfa1eb6bbd095634715815	0	0	2	0	0	0	0	0	0	0	0	0
2cd60d50e94641ddb321e4dbd2b6aa8e	0	0	2	0	0	0	0	0	0	0	0	0
0db46034fabfcd5d30d4a4b434991359	0	0	0	0	0	0	0	0	0	0	6	0
bf06b4ef25e9a8fccfb302fd8f7ee950	0	0	0	0	0	0	0	0	11	0	0	0
de86261337b9380fa070f782df14c748	0	0	0	0	0	0	0	0	4	0	0	0

12a2bc3c80559cf08f9e81e81 8ae4d8e	3	0	0	0	0	0	0	0	0	0	0	0
b333ca4a2f8b3eb4b5f39e9e 6d403748	0	0	0	0	7	0	0	0	0	0	0	0
32007b45db6cb90894e9e1b b04cb9b84	0	0	0	0	0	2	0	0	0	0	0	0
b8abc1066e530817c43c817e c07dc892	0	0	0	0	0	0	5	0	0	0	0	0
c587908a47422c90ba1bd44c bf4192c1	0	0	0	0	0	0	0	0	0	0	5	0
a0f6424e8a62ac8d332fd28d bedc7a48	0	0	0	0	0	0	2	0	0	0	0	0
0eebb0d5efff91658ba3025c0 7410ecf	0	0	0	0	0	0	0	0	0	5	0	0
008a3fcc5f612fe672120e34a 8a15ae3	0	0	0	0	0	8	0	0	0	0	0	0
8b9bd27823adbb2ad0fb2c3b 3ee163e9	0	0	0	0	0	0	0	0	0	0	0	3
2926398713cfbe8fbed84eb9 83b55ca8	0	0	0	0	0	0	0	5	0	0	0	0
77e019438f8ab9e5992fbd5d f70c28dd	0	0	0	0	0	0	0	2	0	0	0	0
b977a850abc5aa65bca173e5 07b02911	21	0	0	0	0	0	0	0	0	0	0	0
6b3e508b7ffc73ed66ef33a56 c66b355	0	0	0	0	0	0	0	0	0	0	3	0
43ff87bf65309cefa52e655eb c338cf3	0	0	0	0	0	0	0	0	0	9	0	0

9fdcc161ea2938fae1286887e344da6c	13	0	0	0	0	0	0	0	0	0	0	0
8a91c6805d511e1a14e6bedd09a45902	0	0	0	0	0	5	0	0	0	0	0	0
b4a751c9f2f685a77aec7b02e5ece2a2	0	0	0	0	0	0	0	0	0	0	0	3
0e2e89968368e20c15fdf5c962637b66	0	0	0	0	0	0	0	0	0	0	0	3
bf8bea9c137dde45768fb3be16889c2c	0	0	6	0	0	0	0	0	0	0	0	0
90f3c64003871e41e91c9786dd551393	0	0	0	0	0	0	0	2	0	0	0	0
a92931d48e82abdcfa19c382ab16743c	0	0	0	0	0	7	0	0	0	0	0	0
ed31caa63f7dcf8dde1fe92b655151bc	4	0	0	0	0	0	0	0	0	0	0	0
599a0c64147ab9c091a0dcd633921fe1	0	0	0	0	0	0	0	0	0	6	0	0
d012f873d5feae5850834013dc047d50	0	0	0	0	0	0	0	0	0	0	0	19
03b9ca951094de9c88bc3f5e6402ce0a	0	0	0	0	0	0	0	2	0	0	0	0
d77a9f423bfb2bd8cd8391f24c6c2d4b	0	0	0	0	0	11	0	0	0	0	0	0
50d6396722e644f72c6e6b291f4b3dce	0	0	0	12	0	0	0	0	0	0	0	0
1979d15c861cf458c734fc8e09137c42	0	0	0	0	0	0	0	0	3	0	0	0

47f31c9d0fd2333092fade3ee57d6179	0	0	0	0	0	0	0	0	25	0	0	0
bf5671b5192e895a72ceae5db91089f4	0	0	0	0	0	0	2	0	0	0	0	0
eee59a530e29a497bf10139e23fc42c6	0	0	0	0	0	0	0	0	0	0	0	24
c997fe6d58321e806b6a40eef83cc8d5	0	0	0	0	0	0	0	0	0	0	10	0
dbf1439c1db8e17006b993bf8a0ec1bc	0	0	0	0	0	0	0	0	2	0	0	0
6a8fa0931f9c3f2fe040a797bc858131	29	0	0	0	0	0	0	0	0	0	0	0
6c049a0e45b30e221bf8e014861471ed	0	9	0	0	0	0	0	0	0	0	0	0
798d27e042ee3df630bd1b94323b4232	0	0	0	0	0	0	0	0	0	0	14	0
3c5cf087374d83c8d316f59c2147f539	0	0	0	0	0	0	0	0	0	0	4	0
5f4cb56594989458ad436353bd423110	0	0	4	0	0	0	0	0	0	0	0	0
4a3c72bd6217ad9ad0923e07e3569b42	0	0	0	0	0	0	0	0	5	0	0	0
835a7bb919f50e8f0394d3ede4c38b5e	0	0	0	0	0	0	0	0	0	0	0	6
3f7cc86492af4a6f44fce528a778402d	0	0	0	0	0	0	0	0	0	0	10	0
4e126a30d62e5bb38b43d2537e99c6fd	0	0	0	0	2	0	0	0	0	0	0	0

a155c270e630ccf9bc959e45f34e7025	0	0	0	0	0	0	0	0	0	0	7	0
7ba0563e6e13a29d37896c44e0b171cd	0	0	0	0	0	0	0	0	5	0	0	0
1ee482bf94de64fcc8ef1e7915a5cc35	0	36	0	0	0	0	0	0	0	0	0	0
5c4fd6683b26ae9c6ddb8e83cb2ee225	0	0	0	0	5	0	0	0	0	0	0	0
f8da1deba7271180e0cb207250489303	0	0	0	0	0	0	0	3	0	0	0	0
4a53b53c776151e1d1716065d25fea1a	0	0	0	0	0	0	0	0	0	0	7	0
5edd62c87feb2f2ce42c3828948d9e62	0	0	0	0	4	0	0	0	0	0	0	0
4b39abe89dc2e477e2da7fd60d298bd5	6	0	0	0	0	0	0	0	0	0	0	0
c76884c2120e7d86b9e5d8d1ff3ea796	0	0	0	0	0	0	0	0	0	0	2	0
4a287e43190044157674e185d00fe4d8	0	0	0	0	0	0	0	0	0	5	0	0
9c677406bc0cc693c62cb43dd16dacce	0	0	0	0	0	11	0	0	0	0	0	0
182bf491c7b32c2acb46e89827cd6091	0	0	4	0	0	0	0	0	0	0	0	0
47b919bae0e7ded8204873d23e87ac8a	0	0	0	0	0	30	0	0	0	0	0	0
e49c26f405f663ff04cd12731d805046	0	0	0	0	0	0	2	0	0	0	0	0

d7406bcdd7971834e073f6338b17b7de	0	0	0	0	0	0	0	0	0	3	0	0
e53682c23a6ffb1d3dfc2e989c5cfa4a	0	7	0	0	0	0	0	0	0	0	0	0
51c8d301b103c1ee9d84933606bace36	4	0	0	0	0	0	0	0	0	0	0	0
7747083cdeb40413dab1bcb2b839bca5	0	0	0	0	0	0	0	0	0	2	0	0
4d4b8e18e6a8472d26eaa63c1b88470f	0	0	0	0	0	0	0	0	0	0	0	2
ebb397ba70d66d19c4911509d7d3f286	0	0	0	0	0	0	0	0	0	0	3	0
6bf6e9edc2e9f2af7b1d725f6e0c3f71	0	27	11	0	0	0	0	38	0	0	0	0
2c339023ab5a1a05d60423dfe974c448	0	4	0	0	0	0	0	0	0	0	0	0
3b34647d08a2393db4c29a34125a0d92	0	0	0	0	0	0	0	26	0	0	0	0
679fdfb2cc20fd9f4db2df22a6408a28	0	44	0	0	0	0	0	0	0	102	0	49
665052e1abc67eb6972c99d76f37dba5	0	0	0	5	0	0	0	0	0	0	0	0
b2253f01faad2dc657dbc88cfb8f7870	0	0	0	7	0	0	0	0	0	0	0	0
7e0bcc354c378826a6e1ef7073d845ce	0	0	0	0	0	0	0	0	0	0	0	73
92c679cb928d3d96214955c2ea46ce1b	0	9	0	0	0	0	0	0	0	0	0	0

e0be61e11b7c8d56caee8eed2592ac29	0	0	8	0	0	0	0	0	0	0	0	0
171d2445876a730ba1dcdff65a77c8ca	0	0	0	4	0	0	0	0	0	0	0	0
2edb59f40cefd3871b089cda11ea9c5c	0	0	0	0	0	0	0	13	0	0	0	0
6493664c7b229a022a28c6a951c4d8db	0	0	27	0	0	0	0	0	0	0	0	0
8728c239c500ad91af41045702d74f94	0	0	0	9	0	0	0	0	0	0	0	0
4be05424f5c8315f88cd2bf88c75787d	0	0	0	0	0	0	0	0	0	4	0	0
30ddc72589fd0fbc34e5a342e7ce9a34	0	0	0	0	0	0	0	7	0	0	0	0
2dde9756f92b1565d7342348e653a3f5	0	0	4	0	0	0	0	0	0	0	0	0
acd275189e1b9d0ea814e29a6ac8bbcb	12	0	0	0	0	0	0	0	0	0	0	0
df3e73579109270d917dfcd14238ac13	18	0	0	0	0	0	0	0	0	0	0	0
fb72522ac59408dfa15de0b7c0371850	0	0	0	0	0	0	0	3	0	0	0	0
2c3e80f8b024c6233eaa43711a63cb9f	0	0	0	0	0	0	0	0	0	0	3	0
8f7d23d441a6597eedc72693919665de	0	0	0	0	0	0	0	0	0	0	2	0
5ef147fe81afc7ba84e69323cb8a9ac6	0	0	0	0	0	0	0	2	0	0	0	0

32c057bd4f31549145f439c49940f65c	0	0	0	0	0	0	0	0	0	0	39	0
5568f0680d69f915adaa11e8fe1eff23	0	0	0	0	0	0	0	0	0	0	4	0
c872a009e4e7dd7102395bf1d4de270c	0	0	0	3	0	0	0	0	0	0	0	0
754bddff47ff1693a56c0f84b3858573	0	0	0	0	0	0	0	0	0	0	3	0
175d1bf49bf896ee5d971c928d6fa062	0	0	0	95	0	0	0	0	0	0	337	0
dfbf1d7765421618de185aa4bdd473fc	5	0	0	0	0	0	0	0	0	0	0	0
d3a1042bb9ddab97f5fb1c22b26e9ca4	0	0	0	0	0	0	0	0	0	0	3	0
c259d863f643cb726bc63ce3120ee0b0	0	0	0	0	0	0	0	0	2	0	0	0
eb768bad6c501817328e4c00a3e81943	0	0	0	0	0	0	0	0	0	16	0	0
595e1e330f68df0c0ca9f0d3b6d772bc	0	0	0	0	0	0	0	0	0	0	5	0
4f1bb8ab42727ae4d4304f459824b222	0	0	0	4	0	0	0	0	0	0	0	0
8dd5f2a4b6f9327da04757afe63459d1	0	0	0	0	2	0	0	0	0	0	0	0
9ca007ee17c73b63bb613c1092faed4e	0	0	0	0	3	0	0	0	0	0	0	0
9015f2d8f09aeea27ef727e1fa174ba2	0	15	0	0	0	0	0	0	0	0	33	0

2c9862b8da71e0caa5ca47c2859bd516	0	0	0	0	0	0	0	0	4	0	0	0
b4bac2b257c5bcb82589f03065d8d2dd	0	0	0	0	0	2	0	0	0	0	0	0
403db225f56ac6d76f369418c4438154	0	0	0	0	0	0	0	3	0	0	0	0
3a591c6b5aa9be1023aad859b34e6652	0	0	0	0	0	0	6	0	0	0	0	0
14ce6edfa2e55332d931f9e092b23834	0	0	0	0	0	0	0	0	0	0	4	0
57227ac3fdf9ef8471f289e341b1cad4	0	0	0	0	0	0	0	0	0	0	6	0
f81b084b2e6c29a16f9d97f135656a72	7	0	0	0	0	0	0	0	0	0	0	0
00ff619e42e6bd5514d2db9cbb3aa063	0	0	0	0	0	0	6	0	0	0	0	0
86316bf17ba7c60f8b24b5dfcde1ab72	0	0	0	0	0	0	0	0	0	0	16	0
2cb3ab17cd0e4d7ae62a372eb7b4cfcb	0	0	0	0	0	0	17	0	0	0	0	0
b834d326b762e15c7612e91e69a6127d	0	15	0	0	0	0	0	0	0	0	0	0
77113a510d739ea2bf2c75976c154769	0	0	0	4	0	0	0	0	0	0	0	0
6926d94ed0f0f684911d0ada9aa4cecc	0	3	0	0	0	0	0	0	0	0	0	0
54fca7e774a103a3d8b00ad3522d1fa2	0	0	0	0	112	0	0	0	0	0	0	0

7ae6a4165f803fe97dec56c23789a430	0	0	0	0	0	0	0	0	0	0	0	3
69ba43c2fed471c05a06ac91c6adfc2e	0	0	0	0	0	0	0	0	0	0	21	0
c2ca29e0bbf104d2d314614c85065ba2	0	0	0	0	0	0	0	0	0	0	14	0
39033d004163acd6c33a1a80bab3b7ed	0	0	0	0	0	0	0	0	0	0	0	10
0e73f09ae4dd5aa229988ece97bf1986	0	0	2	0	0	0	0	0	0	0	0	0
2a8e035a3f0cbd1d5b09f86370c4916f	0	0	0	0	0	153	0	0	0	0	0	0
ec0b0cf1062d72b408918ba9cbed518f	0	0	0	0	0	0	0	0	0	8	0	0
70e98cb52406c12a764012c9fc7a1043	0	0	0	0	0	0	2	0	0	0	0	0
d482d43aeae102768e9ca7302c4d06de	0	0	0	0	0	0	0	0	0	0	141	0
4889e3b4e59affb7f69f70d9c66189f6	0	0	0	0	0	199	0	0	0	0	0	0
15a61a8d07a00a7ec23d032ec88751d3	0	0	0	0	0	5	0	0	0	0	0	0
08a30cb26a94fbde9aef9bdb6871b4da	0	0	0	0	0	2	0	0	0	0	0	0
6a89e0b61ca40057db6538e5992ef82a	5	0	0	0	0	0	0	0	0	0	0	0
1a51f4f4f669f69d8fdc67d52d6559d9	0	13	0	0	0	0	0	0	0	0	0	0

7dc9fb3897333e05e05ad999f956870e	0	0	0	0	0	0	0	30	0	0	0	0
815227fb4e6ed3f9824e11c680af1258	2	0	0	0	0	0	0	0	0	0	0	0
cf9ba3c295f78f1e7a6e5064788c4d4a	0	0	0	0	0	3	0	0	0	0	0	0
46da3dc0b1831125e17167759d2addcb	0	0	0	0	0	0	0	0	12	0	0	0
48c7b4417089bd9358b205bd480f066c	0	0	0	0	0	0	0	30	0	0	0	0
df34d1679fd5ed7d4d31572694a9959f	3	0	0	0	0	0	0	0	0	0	0	0
dcabb26c23feafd1c4bea2ba54f6dd1d	0	0	0	6	0	0	0	0	0	0	0	0
0bc04e246688bb0e215cb635afdbd216	0	6	0	0	0	0	0	0	0	0	0	0
f23a10a8282bf350e6e08a15158b23b8	0	0	0	0	0	0	2	0	0	0	0	0
d550612b2f65ee395b9cd29e98a01fbe	0	0	0	0	0	0	2	0	0	0	0	0
0c3aae0e4a361942fb90b3ecf135a091	0	0	0	0	60	0	0	0	0	0	0	0
41cf34ec592983703f521461f4097131	0	0	0	0	0	0	0	2	0	0	0	0
967ad21f73dc4ba0ab254a938d111874	0	0	0	0	0	5	0	0	0	0	0	0
ece59e98a611e070a900ecf37ce15dda	0	291	0	0	0	0	0	0	0	0	0	0

d6b83211da82678e5866b43a5b9ac984	0	2	0	0	0	0	0	0	0	0	0	0
6e9578a20d77c13c99aa61a9c7cc8168	0	0	0	27	0	0	0	0	0	0	0	0
f9693411ac9c1d7aec0b61f9157335f4	0	0	0	0	0	0	0	0	0	4	0	0
b6abec01ff5751809cdf80d9fe698e60	0	0	0	0	0	0	5	0	0	0	0	0
5b9a3715e7737796f248baf7fa4a4766	0	0	0	0	0	0	0	0	0	2	0	0
397f243d7e0bd51fb308abaaa63a6d92	0	0	0	0	0	0	0	0	0	0	3	0
629c9f3b7ebc4dcf81c60ea620941a49	0	0	0	4	0	0	0	0	0	0	0	0
637ed0bea5662052de6215c3443bfebf	0	0	0	0	10	0	0	0	0	0	0	0
82d777e80dfaf3c8f7e5c27011662975	64	0	0	0	0	0	0	0	0	0	0	0
182497dd34b3652fb50670cfdf5da477	0	0	0	0	0	0	0	3	0	0	0	0
28c3bc9d4d30fdbe2507cf55235273fd	0	0	2	0	0	0	0	0	0	0	0	0
a55c300fdbb650036952387f2fc96a74	0	0	0	0	0	0	0	0	0	0	36	0
1c055f983628952ecc03d8a86903b672	0	0	4	0	0	0	0	0	0	0	0	0
690cde0f245da8a775d4d384974ceef0	0	0	0	0	0	0	0	0	0	8	0	0

12f9d2630f41f648d6c29b9ad8c9112e	0	0	0	0	0	0	0	3	0	0	0	0
af7683f224a439f379d3ae274b7f7161	0	0	0	2	0	0	0	0	0	0	0	0
4d93743ba63b13075ee577089030c1e2	0	0	4	0	0	0	0	0	0	0	0	0
07ceff96905b84ce7b416cf09baaf94e	0	0	0	0	0	0	0	2	0	0	0	0
7ff19df1194a8d32ff53921b386c1487	4	0	0	0	0	0	0	0	0	0	0	0
9fbb0a6f368d3620cf8ae3adcb0c736d	0	0	0	0	0	0	0	0	0	0	9	0
5a5e845c0f3c1f7763a1026b6e5ca999	29	0	0	0	0	0	0	0	0	0	0	0
ed563ce162bf7860ff859ad26aac4213	0	2003	1497	2892	1231	4018	2371	2526	557	2258	914	3953
21dc3c1d5b8571a27b6293f3fc98fa12	0	0	0	0	0	3	0	0	0	0	0	0
a1560096e1cbd6a293ce2012cf8041a3	0	0	0	0	0	0	0	0	0	0	0	10
09ee695fd5669b2b9b3f451a3ef1c64c	0	0	0	0	0	0	0	0	0	0	0	3
6c2a3d20cb17ee6af0ba3c13b5320794	0	0	0	0	0	2	0	0	0	0	0	0
a0409ab792522fd648dbe997349202af	0	0	0	0	0	0	0	0	0	2	0	0
5965d3883239139f779a3fe0e3349137	0	0	0	7	0	0	0	0	0	0	0	0

fc21ac12e5c054a9eb567e51fca3c8b6	0	45	0	0	0	0	0	0	0	0	0	0
7d6681ad449575430139d72993855bc3	0	0	0	0	0	0	0	0	0	0	13	0
8fda20c57560e7df2db6db15f9412487	0	0	0	0	0	0	15	0	0	0	0	0
815f2bdb4454f20bf4c13c97f5f3f080	0	0	0	0	0	0	0	12	0	0	0	0
cf1230354f964063666651af273125ea	248	0	0	0	0	0	0	0	0	0	0	0
3166e0888e81dcbf601bb48bb2ec928a	0	0	0	0	0	0	0	0	0	4	0	0
d311afd66cd3de5d15f0d97c094e8541	0	0	0	0	0	10	0	0	0	0	0	0
d9baf605bbe624a5f35c32d238e2d506	28	0	0	0	0	0	0	0	0	0	0	0
f6af22a6d915365c11909b4a457fdcf8	0	0	0	0	0	0	0	0	0	0	13	0
6a308ce1d0066f0f24833160a2ad9cde	0	0	0	0	0	0	0	0	0	0	0	9
cf236a5412c3fd58f8b952e814c43c14	0	0	0	0	0	0	0	0	0	0	0	2
c7bc73e1ecb871787d4340683cd3570c	0	0	0	0	0	0	0	0	0	212	276	0
a4f106f321db6f174fa7cc2b0c1da492	0	0	0	0	0	0	0	0	0	14	0	0
0da805d2d18fb14d0db0f7354da21373	0	0	0	0	0	0	0	0	3	0	0	0

9f49ad622361a7c1e4a147db b6bd86ea	0	0	0	0	0	0	0	0	0	0	0	2
ef565d0052f8681ba659d1a5 f39520ea	0	8	0	0	0	0	0	0	0	0	0	0
4099cda244e60a973d11ceb8 a9a126c3	0	0	0	0	8	0	0	0	0	0	0	0
c26bd4cf1e86cc2fe6b311f2a f12a0cb	31	0	0	0	0	0	0	0	0	0	0	0
cd2f6bb9cc3f279cccfb70758 3a4e882	0	0	0	0	0	0	5	0	0	0	0	0
c6267b2605287d5f3aca5852 7fedd7a5	0	0	0	0	0	0	0	0	0	4	0	0
36892de622fb007b43e4e74e ea22d7bf	0	118	188	0	225	57	0	0	0	0	0	0
eb60526a6338b16796db38a 055a7e448	0	0	0	0	0	0	0	0	22	0	0	0
a77ca46e75d8eb9a38076f1e 083eb091	36	0	0	0	0	0	0	0	0	0	0	0
8a891d928099a3c79ca8b300 65f167b1	0	0	0	0	0	0	0	0	0	0	0	4
e9316e439dd821f447d23c37 271f79ec	0	0	0	0	0	0	0	0	0	0	22	0
67b7bda3c7e4273595af9f5c 42e47df5	0	0	0	0	0	0	0	0	36	0	0	0
5a8d130833ba25c1d48bd76 24519a59b	0	0	0	0	0	0	0	27	0	0	0	0
4b1bcec2220d5fdd4c460748 6ed56673	0	0	0	0	0	0	0	170	0	0	0	0

0de888eaac77f90855743cb666b06777	0	3	0	0	0	0	0	0	0	0	0	0
1219e6310610ee939dbb15b4e095fb58	0	0	0	0	0	0	0	0	0	8	0	0
7bb73e8ad0ead9df98dc11838110f3f8	0	0	0	0	0	0	0	0	0	5	0	0
b142a3fd375592d906e08eae71285685	0	0	0	0	0	0	0	0	0	0	7	0
f37f4c5fa5ba245edbd024fd6fa39600	0	0	0	0	0	0	0	0	0	10	0	0
00968081a7d22cc8b925e10a2b3239cb	0	0	0	0	0	0	0	0	0	59	0	0
92a59941772e3126cd0a8e8dc3910650	0	0	0	0	0	0	0	0	0	0	0	15
c637a8562c93c2acf30d96ddf2b383b7	0	0	0	0	0	146	0	0	0	0	0	0
f00200ebc436555a79bb4b09044ddf21	46	0	0	0	0	0	0	0	0	0	0	0
06c58bd9205d7fca0ad48ee400f78d3b	0	0	0	0	0	0	0	11	0	0	0	0
52c909f4f94e0eccf909017d6067b99d	0	0	0	0	0	0	0	0	0	0	12	0
332ae17871bf6f433604f88918a62cec	0	0	0	6	0	0	0	0	0	0	0	0
39f4cd35727126d9d2352db2764597ab	0	0	0	0	0	0	0	0	0	0	3	0
b25ea834a37943f52a993f43712fb61d	0	0	0	0	0	2	0	0	0	0	0	0

0ca08ac87720379916f3368e78ea07dd	0	11	0	0	0	0	0	0	0	0	0	0
04c7021c217447d5647f749a4d0a3ebb	0	0	0	0	0	0	0	0	0	2	0	0
e6eb416862a6db91fdea519b2ca782c2	0	0	0	0	10	0	0	0	0	0	0	0
80a465e8385756c1883218465da520da	0	0	0	0	0	0	0	0	0	0	2	0
e2f5146138d0c6877ad3eae75e50069b	0	0	0	0	0	0	0	0	0	0	2	0
fa25a85eb45a495159e8cf9a0e1c387a	0	0	0	0	0	0	0	0	0	0	0	9
ab850a7d8425a487d2e0c149834ded99	0	0	0	0	0	0	0	0	0	0	0	2
cb13c8f91b8c66ad044e0817dfdd3330	0	0	0	0	9	0	0	0	0	0	0	0
7cc9e776ff48d72caa106b027f374231	0	0	0	0	0	0	0	0	7	0	0	0
4b7390ad5934d13746373041608c0658	0	0	9	0	0	0	0	0	0	0	0	0
5301757a8d6089ce65fdcd8e78b26209	0	0	0	0	4	0	0	0	0	0	0	0
368aa0760d7579c418fb2d65c6834f0c	0	0	0	0	58	0	0	0	0	0	0	0
8af7c377ef4be0944381faef089247c7	0	0	0	0	0	0	0	0	0	0	0	3
52163e15e933890009af212a0b0d2243	0	0	6	0	0	0	0	0	0	0	0	0

814a558a060dad229abd3e10124fbedf	0	11	0	0	0	0	0	0	0	0	0	0
3c3e802b4972e94720d78df28b9587c4	2	0	0	0	0	0	0	0	0	0	0	0
98e27f58ce182853bf02e706832a1fc5	0	0	0	0	0	0	0	0	0	0	3	0
86ca1b2aefbcb77c813f89e5eb2b55a9	0	0	0	2	0	0	0	0	0	0	0	0
b11e8b0e8842796e351ed0e67bd9372d	0	0	23	0	0	0	0	0	0	0	0	0
f30efdc38e35fec4f2058f2785dcbe87	0	0	0	0	0	0	0	4	0	0	0	0
71d79f1ef1970bdc631c2fb27bb95568	4	0	0	0	0	0	0	0	0	0	0	0
c59e1cabb564e048c8f5e830e515e2b1	0	0	0	0	0	3	0	0	0	0	0	0
093f4a09fd93595bf12deb1ea1e38d17	0	0	0	0	0	0	0	0	6	0	0	0
487c8b750573eb48f12dfef14281432c	0	0	0	4	0	0	0	0	0	0	0	0
eef7c1db770feac030ed7b5d9654d7f8	0	0	0	0	0	0	0	0	0	0	0	2
130f2998fb93ebf39421fa8afa2e3d85	0	0	6	0	0	0	0	0	0	0	0	0
84ce7e81112488e3d2516ee2b354c2bc	0	0	0	11	0	0	0	0	0	0	0	0
dd31aaa47066c997b6fd6d3b18198c93	0	0	0	0	0	0	0	0	0	0	30	0

87e542a5a3646477de0a125 0c50d100a	0	0	0	0	0	6	0	0	0	0	0	0
e36b4dd06359d51969dd67a 70ae3da34	0	0	0	0	0	0	0	3	0	0	0	0
046bf015f22b02c05cef94f1b 8fbb02c	0	0	0	0	0	0	0	7	0	0	0	0
3a1f830652dce0ca85e8fbcd8 1e87a44	0	0	0	0	0	0	0	0	3	0	0	0
b5f25c8193fb74c4d4046e76 1cc911fe	0	0	0	0	5	0	0	0	0	0	0	0
d7e99d1bb5987c4e61eb5aa 10a8b0c38	2	0	0	0	0	0	0	0	0	0	0	0
7eda1be2f0c3a48b5260fc1fc 2351400	0	0	0	5	0	0	0	0	0	0	0	0
39e32a164c917e201d7a22aa 5e0d5133	0	0	0	0	0	0	0	0	0	0	9	0
42945953049b2347bcb14c0 7dc742eff	0	0	0	0	0	0	0	69	0	0	0	0
8aed9324e7f1b3e50a42339d 6c7fbebf	0	0	0	0	0	0	0	0	3	0	0	0
e03fe7f0cde1aee1432a0688 decf892b	0	0	0	0	0	0	8	0	0	0	0	0
97201874c774e7ba3ef9af28 820d6581	0	0	0	0	0	0	0	0	4	0	0	0
3238dba5290a0709b82844c 1b5fa560e	0	0	0	0	0	0	0	3	0	0	0	0
2888184fad42467eee69d7a8 ca392e51	0	0	0	6	0	0	0	0	0	0	0	0

7e950aaa3767df9083f1d1f7a36410e3	0	0	0	0	0	0	0	0	0	0	11	0
7141a501323eae9af2ee75edfcbe3541	0	0	71	0	0	0	0	0	0	0	0	0
251ff238a59e0e765376911a5da0ec6f	0	0	0	0	0	0	0	0	0	0	4	0
da2f2dc007dfcaa8f539c490f2da817e	0	0	0	0	0	0	0	0	2	0	0	0
002183fe06f410092edfd8dd1e790c4a	0	0	0	0	10	0	0	0	0	0	0	0
a00aef2d1008e5921b3e955763d04a04	0	0	0	0	0	0	0	0	0	0	13	0
27a3e20f96e1a352463cc11414fb2f69	0	0	0	0	0	0	3	0	0	0	0	0
d6005f2ad4577615a04611619bc8a825	0	0	0	0	0	0	0	0	0	0	0	4
806bb3532e551c25845140970f7c5561	0	0	0	0	38	108	0	0	0	0	0	0
2effe3b3a540bf65fa15f307a6d25eef	0	0	0	0	0	0	0	0	0	6	0	0
7e907c18bf57b21f3c42b85796538b99	0	0	0	0	2	0	0	0	0	0	0	0
f3f5b2db509b457c44b0b4be80b789f8	0	0	0	0	0	0	0	0	0	22	0	0
a746244387fee191c9b2bb986388a8a9	0	0	0	0	0	92	0	0	0	0	0	0
5bf6310e4ccc4ec1e58d258b8e860b5c	0	0	0	0	0	27	0	0	0	0	0	0

69b7ebfa0cabdee0bd091f3b14ad9bf7	0	0	0	0	0	42	0	0	0	0	0	0
d1f5b7e7596336f2e4c248101cb91f76	0	349	0	643	0	707	305	666	0	393	296	366
019ed7327461d12597012ee5aa8d4f90	0	0	2	0	0	0	0	0	0	0	0	0
2e5cfc9b973c49df5d2e183cc2dfe41d	0	0	0	0	0	0	0	0	0	0	0	111
8e99d23e4d06ce5b007943d63eb71208	0	0	0	0	0	0	0	0	0	0	0	8
f749add3deee1e14a4dcd476f4f771e6	34	0	0	0	0	0	0	0	0	0	0	0
9a8117b239da77dc18e2958a967f5e13	0	0	0	0	0	0	0	0	0	0	0	11
27989ed84a52106acd9af2758264c653	0	0	0	130	0	0	0	0	0	0	0	0
54c2cfd1fe66ff0046082964186e91e7	0	0	0	0	0	0	0	0	0	3	0	0
d666f532d4167dbbd9e7620b3e822b4c	0	0	0	0	0	0	6	0	0	0	0	0
c5d967b5627e31ef56d724fac7b6d6b9	0	0	0	0	0	19	0	0	0	0	0	0
5cd4382d74a00172b5b1bc3114ddfe4b	0	0	0	0	0	0	0	0	0	0	18	0
d1a8cca9f90ae79507655b5a6f753c30	0	0	0	0	8	0	0	0	0	0	0	0
3f9d378929a6cdb88a5cbd8b79a23128	0	0	0	0	0	0	0	0	0	0	3	0

b5e9bc2ff39b6c242b9e8b1ff3b1e52c	0	0	0	0	0	0	3	0	0	0	0	0
3e91b12e6fe2a2ef8a1c3939d2e608ab	0	0	0	0	0	0	0	0	2	0	0	0
69027eee3958360c8279f48c525103ac	0	0	0	2	0	0	0	0	0	0	0	0
6ac38588221951039435dbf027f5e776	0	0	0	0	0	10	0	0	0	0	0	0
82fc3c5ceb0fe8580ed9d2574e44a877	0	0	0	0	0	0	0	0	5	0	0	0
8c2645976e8d8c496b8fc7476ba8acc7	0	0	0	0	0	0	0	0	0	12	0	0
15a82e174670b3ed95b3167bf32b9758	0	0	0	0	0	0	8	0	0	0	0	0
4391735365c00bf11770b8e4607840da	0	4	0	0	0	0	0	0	0	0	0	0
5a8e817047a25e47b6b46f4400fe057a	0	0	0	0	0	5	0	0	0	0	0	0
4f7c5e0cc414484676414b4f37476aef	0	0	0	0	0	0	0	0	0	14	0	0
8ddf771ea3ad11dcc4025438c42313e4	0	0	0	0	0	0	0	0	0	4	0	0
76932261bf698f80d0510a688d2dc531	0	0	0	0	0	0	0	0	0	2	0	0
a4f36e5606ca1c5d79c3f024d084cdd9	0	0	0	0	0	0	0	0	0	0	13	0
a27baa1546ef60a26c0d2ec6555122b1	0	0	0	0	3	0	0	0	0	0	0	0

9713626434ad1ba52a151ac7c981e18b	0	0	0	0	0	0	0	0	0	0	2	0
a1fb4d1f437b72c4e3790b146ce6bf36	0	0	16	0	0	0	0	0	0	0	0	0
01b4907405d26e84e7bb216800e6f0c5	0	0	0	0	72	0	0	0	0	0	0	0
83b08d29453cc63e359ffc8b6b91d069	0	0	0	0	0	0	3	0	0	0	0	0
a490483930c2638efdde5dc96d79d7b3	0	0	0	0	0	0	0	0	0	12	0	0
5c754a470077018fd5511f7d75926b38	0	0	0	20	0	0	0	0	0	0	0	0
7dfea04b0f0c1b258f83fc5e7f90fed1	0	0	0	0	0	0	0	6	0	0	0	0
62667d2135338077710ccb599e46ef3a	0	0	0	0	0	8	0	0	0	0	0	0
36229dce90f975b2aeef1f4f09e43091	0	0	0	3	0	0	0	0	0	0	0	0
1d291c1becc870858bffa86ae000f43f	0	0	0	0	0	6	0	0	0	0	0	0
6807226553369b407353dc27bcee6cc9	0	0	0	0	0	0	0	0	0	0	2	0
b8411368346dc24ff3e3c2ee86363e8e	0	0	0	0	0	0	0	0	0	0	2	0
43844798f53cf96de87cf801ee72eec7	0	0	0	0	0	0	0	0	3	0	0	0
5f927ec7bcc881ca5412d136f8b3071e	0	0	0	0	0	0	0	0	0	0	4	0

6ead8d21323eba64c25cc9fbc54ca7bd	0	0	3	0	0	0	0	0	0	0	0	0
e4c5d7542801cc9db1929e956e8f641d	0	0	0	0	0	0	0	0	0	0	8	0
7eb1a8d63ba3fb73eac72e64157302b5	0	0	0	0	0	0	0	0	0	0	10	0
dbb3406c942a0f7dd0ed2916e62f8e2e	0	0	0	0	0	0	0	0	0	0	0	5
235160fbd1e891c881e2264841bbab73	0	0	0	0	0	0	0	0	0	0	5	0
9b56a886c37989696491107d7cef92f8	0	0	0	0	0	0	0	0	0	5	0	0
2eb691635b74a9afe8300d47e64b6bf0	0	0	37	0	0	0	0	0	0	0	0	0
1f02b98e3c2171f3ffbfb150fbb39678	0	0	0	0	0	0	0	0	0	0	8	0
a0c9fb8f82bc8fbe8b2736d90ab2e638	0	0	0	0	0	0	0	0	0	2	0	0
9a5e39fed9870be2943afe63df9c0720	5	0	0	0	0	0	0	0	0	0	0	0
57cc2f4820d4f274a2b9d1fd8f5dbb59	0	0	0	0	0	0	0	14	0	0	0	0
cd2562852d1286187a7cb8de44b88b62	0	0	0	0	0	0	0	0	7	0	0	0
3f9cfe6e853f11b2fc528a0008edce28	0	3	0	0	0	0	0	0	0	0	0	0
3d3ba008ca56fa302afefeba13489200	0	0	0	3	0	0	0	0	0	0	0	0

cdf2e8d6e873233c5a521c6fa4564cf0	0	0	0	0	0	0	0	0	0	0	5	0
5381dd2771564289bcdbfcc3d2d3f225	0	0	0	0	0	0	0	0	0	0	8	0
64af12e0942c9edb285afcb0cbd0d888	0	0	0	0	0	0	0	0	0	0	9	0
bb8c8dbdda40366188cb79ebb23be603	0	0	0	0	2	0	0	0	0	0	0	0
dd51ff526b00563fcd5812ff7bf785d7	0	0	0	0	0	2	0	0	0	0	0	0
ad7ab8591e3965cbf27a4db095f8f85c	0	0	0	0	0	0	0	0	0	7	0	0
2d41ca0c69a1642d14388d46ce14b8c1	0	0	0	0	0	0	0	0	0	0	30	0
dd26e9642469c8321f871f0be7067eee	0	0	251	0	0	0	0	0	0	0	0	0
49ba9fa54d31dcdfd0f372806138f4e7	0	0	0	3	0	0	0	0	0	0	0	0
9bc363a078804fbfc61fe17c6ceca79b	0	0	0	0	2	0	0	0	0	0	0	0
d5de1d48632690db17a6268aefa144f0	0	0	0	0	0	0	0	0	0	3	0	0
c24a426250dff2e9ca9ea346a6932a75	0	0	0	0	0	0	2	0	0	0	0	0
9ac74c82c8868ae8441fe406c7a68b4a	0	0	2	0	0	0	0	0	0	0	0	0
4e1709245849b9c11cb7d6d5d3669f23	0	0	0	10	0	0	0	0	0	0	0	0

05342b0a6272585c106be5a94c07b784	2	0	0	0	0	0	0	0	0	0	0	0
496ae750153d1406980332eb50772e83	0	0	0	0	0	0	0	0	0	0	56	0
aba6e7f0e3bfeef3b5f6d4351841e17e	0	0	0	0	0	0	0	0	0	0	4	0
5c0ca9ae1f4ad06aa2e6f768c513cbf0	0	0	0	0	0	0	3	0	0	0	0	0
706d11d8d85d5e94fe87abbcad142eeb	0	0	0	0	0	0	0	0	0	0	0	3
5e3968a0b9077fa7058f67f6f82b94b0	0	0	0	0	0	0	0	0	0	0	0	2
8c127ba98a86039d8917a1b2da6c5d47	0	0	0	0	0	0	0	0	0	0	2	0
e945261aa0922f5c26b944e1b093d3cd	0	0	0	0	0	0	0	0	0	0	2	0
33b5fe93db34903fabc9e919c46fb253	0	0	0	0	0	0	0	0	0	2	0	0
9d7a285b1665fe8b624b9f6f63c70aa8	0	0	0	0	0	11	0	0	0	0	0	0
f0619ee82e1d523a23ebba0fe5decd35	0	0	0	0	0	0	35	0	0	0	0	0
88020f8c70eb0244bfb4b75e505a7518	0	0	0	0	0	0	0	0	0	0	0	3
371bf4f07b175cc619aeab12eaeafc51	0	0	0	0	6	0	0	0	0	0	0	0
d7c01073d51a9f78e59d9ad04393ed20	0	0	0	6	0	0	0	0	0	0	0	0

b171e729ff70e5103b0e3767 74c87bca	0	0	0	0	0	0	0	2	0	0	0	0
75c6048d2fd13b36c2d0a9fa 117686dc	0	0	3	0	0	0	0	0	0	0	0	0
98154a19905a938ff2a014a6 26b69e8b	0	0	0	0	0	0	0	0	0	0	51	0
3dcff42f82ac65c85f335b5a5 e7953f8	0	0	0	0	15	0	0	0	0	0	0	0
24d6ae5420d6e65a2b6794a 06c0a035d	0	0	0	0	0	0	0	0	0	0	2	0
3dcf96deb905987bec182e60 7898af6e	0	0	0	0	0	0	0	0	0	26	0	0
9d73c45fa8f961f033de1d46f dce3f78	0	0	0	0	0	15	0	0	0	0	0	0
5df073f56c6adee98f71b0119 bc084cc	0	0	0	0	0	0	0	0	3	0	0	0
26fdfbe74fa22a5f8ae25f16a b34c8bd	0	0	0	0	0	0	0	0	0	3	0	0
66f2e44e6f7d1d82efd3c49cd eced49d	0	0	0	0	0	0	0	0	0	0	0	5
ba19716a1e0c6f11a9fabde8 5e4f0635	0	0	0	0	0	0	0	0	0	0	37	0
8bd27f75ceae1ac838bf135c4 50bdd7b	0	0	0	0	0	0	0	2	0	0	0	0
9ed3cee5b373babda299b6c 994d4c917	0	0	0	0	0	0	0	0	3	0	0	0
4739688433fffdd7067da9e0 a03ee4bd	0	0	0	0	0	0	0	0	0	4	0	0

82ee740fd4fefb34daa0e0a2df638a02	0	0	0	0	0	35	0	0	0	0	0	0
ae19c72dd393e899cbccf57a4a647378	0	0	0	2	0	0	0	0	0	0	0	0
f4918bbf03015a16626fa93cac993bfe	0	0	0	0	0	13	0	0	0	0	0	0
a5efe7ea47762d23622fd9bdb0f34d4b	0	0	0	0	0	0	0	9	0	0	0	0
614d48fb42c4a15d106ffb2b6cdfcc66	0	0	0	0	0	0	2	0	0	0	0	0
4041c7235fb9d484df824d660a92517e	0	0	0	0	0	0	0	0	0	0	14	0
2f9cf190a49c957a8c0407c3585cb6f4	0	0	0	4	0	0	0	0	0	0	0	0
2a10c12665f1183038331f79d5ae0cd9	0	0	0	0	4	0	0	0	0	0	0	0
e29b354198566e40249c9fda53b18e97	0	0	0	0	0	0	0	0	0	0	196	0
3cd3bee4554fb8a372734fb64d38d015	0	0	0	0	0	0	0	0	56	0	0	0
a231e528692f03d3d6b6181a6b471da6	0	0	0	0	0	0	0	0	0	0	9	0
f1a1320328599b21618d24c3d56ada33	0	0	0	0	0	0	3	0	0	0	0	0
7d03426ff3c15bbe939f0ea9c1806844	4	0	0	0	0	0	0	0	0	0	0	0
80231651024097e9c6b6c7d084b1958a	0	0	0	0	0	0	0	3	0	0	0	0

390279ab1f2010e6288a67db b358d01d	33	0	0	0	0	0	0	0	0	0	0	0
e9de18361d77e7368fbc16e2 71762d81	0	0	0	0	0	0	0	0	0	0	5	0
f4ee41c14b5847121874664c 15da1bd7	0	0	0	4	0	0	0	0	0	0	0	0
e27cda1b7b5955ff8a21dd1c 73ab9454	0	0	0	0	0	0	4	0	0	0	0	0
b359305186fe5a431bcfd5af3 b3df053	0	0	0	4	0	0	0	0	0	0	0	0
cb48a3d8804c7b69fb64befb 1653ac51	0	6	0	0	0	0	0	0	0	0	0	0
1a422d92b6ad351a13ac960 ec0b7dc6e	0	0	0	0	0	0	0	0	0	12	0	0
529ec5f61586c9001cae62fcb b107d09	0	0	0	0	0	0	0	0	0	7	0	0
89b9fcc2f39dfa50f42d06eba 1087353	0	0	0	0	0	0	21	0	0	0	0	0
46368f4ed4d9853ec4265593 c6109187	0	0	0	0	0	0	0	3	0	0	0	0
5976932ab7b5d7b2bdf05e3 1df92e5da	0	0	0	0	0	4	0	0	0	0	0	0
b0c1f3150dbf0f7e04728357 bc867fc9	0	0	0	0	0	64	0	0	0	0	0	0
09a3b83fce9cc5df4bc2b62f8 040cfcb	0	0	0	0	96	0	0	0	0	0	0	0
fa00b410bb14a604cc9aeb19 3da67737	0	0	0	3	0	0	0	0	0	0	0	0

b74d867f0a29ff351a866f194179ed1f	0	0	0	0	0	0	0	0	0	0	0	6
be93ab92bb898bcf86f1ca5f406a114b	0	0	0	0	0	0	0	0	0	227	0	0
4f24aaa53ea7d81102e3e281eb648347	0	0	0	0	0	0	0	0	0	0	0	3
14f484185d6f77be29f0a84d72d0a0aa	0	0	0	0	0	0	0	3	0	0	0	0
a79a902e57f32dca4e008648af02ca55	0	0	0	0	0	0	0	0	0	0	0	2
ca61aa8686270cf7b0dd442d7d8108cb	0	0	0	0	0	0	0	0	0	2	0	0
f4bf3c602d6522b4fef47b7ab365881e	0	0	0	0	0	0	0	0	0	0	0	8
1e77d716b170ddfd7e3447e3ae976250	0	0	0	0	4	0	0	0	0	0	0	0
0e59101210535113f9f06e679890bd51	0	0	0	0	0	0	2	0	0	0	0	0
51edf4a5f61957892a4daf19ac8c6186	0	0	0	0	0	0	4	0	0	0	0	0
62ce4b66e92d6404aab465791b940bcf	0	0	0	0	0	24	0	0	0	0	0	0
1c2e44ed0ee5af5be641f802b817f348	0	0	0	0	0	4	0	0	0	0	0	0
651e23f0ff30b5ccf575cd8cd61f53db	0	3	0	0	0	0	0	0	0	0	0	0
6fe17ebc4aefcbfe726c20361a07d821	0	0	0	5	0	0	0	0	0	0	0	0

5f58746f98c01ac93e33a71ab8f61441	0	0	0	0	2	0	0	0	0	0	0	0
5d3e5a112b014848aeb0daaae6f0f3e0	0	0	0	0	0	0	2	0	0	0	0	0
a6e509dbcc4446eeb1b01c07d1cf0808	0	0	0	0	9	0	0	0	0	0	0	0
53840d9129d82626080ffcc1660826d2	0	0	0	0	0	0	0	0	3	0	0	0
3547bcc300c1f1508019bc31cc84250f	0	0	0	0	0	0	0	0	0	0	9	0
8d78fd0ac4e8f06aca5d8b6c559a90ed	0	0	0	0	0	3	0	0	0	0	0	0
5c2bbbe0b482ce71fd30a481d117fe42	0	0	0	0	5	0	0	0	0	0	0	0
2dd8aab8fc722d12962841e555e9bb37	0	0	0	3	0	0	0	0	0	0	0	0
eec3e34759a7d53debe607f041e4abf6	0	0	0	0	0	0	0	0	0	0	18	0
647b21ae874d323781c472586cc5fc86	0	0	0	0	0	0	0	0	3	0	0	0
442db5e76a504a8141606fd215e9fab5	0	0	0	0	0	0	0	103	0	0	0	0
2652ffa355d8bea72fa96cd96ea016c5	0	0	0	0	0	2	0	0	0	0	0	0
566380bc8b4f1071750accd934878292	0	0	0	6	0	0	0	0	0	0	0	0
58c82f1bb6f99b2e2bb619c87d39a6e8	0	0	0	0	0	0	0	0	2	0	0	0

822c7ca50198c8a505c2ba69f8baf167	0	0	0	0	0	0	0	0	0	2	0	0
9cf1b52352de52a8b20d1b589afa2545	0	0	2	0	0	0	0	0	0	0	0	0
13ab8aee42b9bc060f3f63cf083caff5	0	0	0	221	0	0	0	0	0	0	0	0
b667717dddc1cb849745aefabbaa9a8c	0	0	0	0	0	0	0	0	24	0	0	0
1d463b13633e16a276bc0c2683f39d84	0	3	0	0	0	0	0	0	0	0	0	0
09b80ad84cced7a2cc7d89b407b3e114	0	0	0	0	0	0	0	0	0	0	0	4
73bdfcea522ad9ad91a845bc27c36f89	0	0	0	0	0	0	0	0	0	4	0	0
98112ac662f876ab2762dee6b399c2a2	0	5	0	0	0	0	0	0	0	0	0	0
55efc7ee5ed65e412f78265137a95feb	0	0	0	0	0	0	0	0	0	11	0	0
b91ee1b7c8037ef9fc95e5bd00f5820c	0	0	6	0	0	0	0	0	0	0	0	0
6da5f437c5a28148936fe69d9979ffc8	0	0	0	0	0	40	0	0	0	0	0	0
918c65971370203485be73e0fc07cd8c	0	0	0	0	0	0	0	2	0	0	0	0
fecf49a743c5e28c84b069335ad10c6e	0	0	0	0	0	0	0	0	0	0	0	7
d45d3f18359ceab72eee79e0842ab917	0	0	0	0	0	0	0	0	0	0	44	0

07efe063627039dd24baf7a00a5aad2b	4	0	0	0	0	0	0	0	0	0	0	0
aa1e654ab266447b540db19f4f4542f2	0	0	0	0	0	4	0	0	0	0	0	0
ecbf931524ac8bc55b749a0a1b603da6	0	0	0	0	0	0	0	0	6	0	0	0
e0751ccfe132ea2bdd2ef72698dfeb0f	0	0	0	0	3	0	0	0	0	0	0	0
9ec13320269838b55247ad7dbc1b2053	0	0	0	0	0	0	0	0	0	0	6	0
898a3e1ad72efd67c77700bcaf8d5514	0	0	0	0	0	0	0	0	0	0	0	2
b4cbc402d946299880863d977738faad	0	0	0	0	0	3	0	0	0	0	0	0
60f6a118cc87335f498f7c917b6c04f8	0	0	0	0	0	0	0	0	0	12	0	0
8f021a40e4b881cb48012c7a7a52506a	0	0	0	0	0	0	3	0	0	0	0	0
8c8ae5fb86df42f2e95693458b1061fb	0	0	0	4	0	0	0	0	0	0	0	0
d5b5341246d6497469996795420bbadb	0	3	0	0	0	0	0	0	0	0	0	0
539fb4bb90795983c74723dad3d4204c	0	0	0	0	0	0	51	0	0	0	0	0
a23c9616c1ba52deb7cb073f51c58152	0	0	0	0	0	0	0	0	0	0	12	0
71ebf68743d90e791677eefb61112a13	0	0	0	0	0	87	0	0	0	0	0	0

91c6dfdbd1a9b3978e9c6039b29177ec	0	0	0	0	0	0	8	0	0	0	0	0
9b9c23138f059b2bc6c348642b23ecbc	0	0	0	0	0	0	0	0	0	0	24	0
f8a1745e076448ab08e2713f9fb35307	0	0	0	0	0	0	0	5	0	0	0	0
d02bcaf750a720a8b0251e3c6aef93bf	0	0	10	0	0	0	0	0	0	0	0	0
cb35c360473494274b500856ab57ed1c	32	0	0	0	0	0	0	0	0	0	0	0
93662c733517d82791fb4c65b4a8ede0	0	0	0	0	2	0	0	0	0	0	0	0
1fa92eff5d4b1f493149067ceb08753e	0	0	0	0	7	0	0	0	0	0	0	0
641553f6d466de08d171eac225bfe87a	24	0	0	0	0	0	0	0	0	0	0	0
c4df1ab9176b4635b5dcaa2ca45109b4	0	0	0	0	0	0	0	0	0	0	0	3
d73b4ffccb43b3c02bb709059b9fc3d5	0	0	0	0	0	0	0	3	0	0	0	0
0882f2ef10fe947a800b645915c3eea4	0	0	6	0	0	0	0	0	0	0	0	0
a61203f9ced49e6a8725c1d9476eb0df	0	0	0	0	3	0	0	0	0	0	0	0
a6e1a4006af95a490ec5214d006ce9f2	0	0	0	0	0	0	0	0	0	0	0	4
e7e40f7f6cabe63d4342d2df41acc267	0	0	0	4	0	0	0	0	0	0	0	0

5acfe7d919c378548e700051b5d2298f	0	0	0	33	0	0	0	0	0	0	0	0
339a65e66ac6d28da207f75db51618b4	0	0	0	0	0	0	0	0	0	0	0	12
0cea329aee2c01dbb22e64f888a2fdc5	0	0	0	0	0	0	0	0	0	35	0	0
1f0eb1834b1525ef36517b3d3fc314dd	0	0	0	0	0	0	0	0	0	0	5	0
6ec2af92fec788a5670d30c46c6c7488	0	0	0	0	0	0	0	17	0	0	0	0
861e46d3a48504c42507bc580a22314f	0	0	0	15	0	0	0	0	0	0	0	0
3e6a40f4a3544452e8cb5c6c98bd28be	0	0	0	0	0	0	0	0	2	0	0	0
af7c5499203c8ca7732eaf44e2968901	0	0	0	0	0	0	0	0	0	0	0	152
d630d59d6f9be2d222ef92976075aef1	0	0	0	0	0	0	0	0	0	0	17	0
6fd7ad398a79a61a175a20155bf661c6	0	0	0	0	0	3	0	0	0	0	0	0
44876645aea8402dfc023f54f37368e2	5	0	0	0	0	0	0	0	0	0	0	0
61a647f9b253df9d6461d99ab7891166	0	0	0	0	0	0	0	0	0	2	0	0
1ff03f359c29c0a7253ec9de0b7a55b3	0	0	0	0	0	0	6	0	0	0	0	0
9863612ead37d1db92433347fd8b6534	0	0	37	0	0	0	0	48	0	0	0	0

4fd5785773b5333bd04aa67726768ba6	0	0	0	0	0	81	0	0	0	0	0	0
1b104eed069837c874e39b09931e37f0	0	0	0	0	0	5	0	0	0	0	0	0
b77295588dd88d7da432f7edfc7e8fbe	4	0	0	0	0	0	0	0	0	0	0	0
4aa66443a05f91353713cbe7178c762a	0	0	0	0	0	0	0	0	0	2	0	0
40faafb5796e53e6c08e76197e6cd870	0	0	0	0	0	2	0	0	0	0	0	0
9994bce9ab5e455242856bf3fbbf7ad9	0	0	0	0	0	0	0	0	0	36	0	0
5ce92ee01118945811c97c3b020edb17	3	0	0	0	0	0	0	0	0	0	0	0
dbdb82108ab3a9c1ad8af3f6c2660787	0	0	0	0	0	0	0	0	0	6	0	0
a4173bdf1318690befb31fc353fcf3c5	0	0	16	0	0	0	0	0	0	0	0	0
6cafffdea93fa7a646db078562711e3d	0	0	0	0	0	0	0	0	0	7	0	0
eefcd301687e36d1455a02cac0d36a57	0	0	0	0	0	0	0	0	0	3	0	0
bb7f04d768adbbc92085ead5f565b2ff	0	0	0	0	0	0	0	0	0	0	116	0
626fb3e727339e57db604cce2eec8212	0	0	0	0	0	3	0	0	0	0	0	0
08933abb891422b01a2e378d7dbd75fd	0	0	0	0	0	0	0	0	0	0	5	0

be1226fcc285aface84c3a442 6683b64	0	0	0	0	0	0	0	0	0	3	0	0
4ba54a566399ce7f74b65a35 9bde89f9	0	0	0	0	0	0	0	0	0	0	0	6
9dc31dcc971c2febd7ae6d62 11385c8b	0	0	0	0	0	0	2	0	0	0	0	0
30a91bacf4045f522ed11ece eb2a372e	0	0	0	0	0	0	0	0	0	0	0	2
bc26b65b61fd4cbef6210ca0 9cf703a7	24	0	0	0	0	0	0	0	0	0	0	0
e4e37cab7f5ddb7cc75601d3 c557720c	0	0	0	0	0	0	0	0	142	0	0	0
0e85b499375f81391232dd5 9f10de590	0	0	0	0	0	0	0	0	0	0	2	0
a5f15cf5116826edf586d57f9 ac317cb	0	0	0	0	0	0	0	25	0	0	0	0
704a02da96c28f6777c48afd 9d18e90d	0	0	0	0	4	0	0	0	0	0	0	0
96c2ec739e715f341fe404f27 9588a20	0	0	0	0	0	0	0	17	0	0	0	0
39669d0d2538de586e3ac6e 7242b5aeb	0	0	0	81	0	0	0	0	0	0	0	0
c9b88266f7f1b1c53f820c8ed 94f38fb	0	0	0	0	0	0	0	0	0	2	0	0
e2e122af2f498772b9dc4e33 5383a209	0	0	0	0	0	0	0	0	0	0	4	0
3b2f1053e9e9b465832cb332 d6c8c410	11	0	0	0	0	0	0	0	0	0	0	0

de8f07b8873ea7d4a921c8bbad86dc5f	0	0	7	0	0	0	0	0	0	0	0	0
2ea704e4604487062e20b780d3f47bd5	0	0	0	0	0	5	0	0	0	0	0	0
cf77c059d2cee3cf1ff325938e8d38cd	0	11	0	0	0	0	0	0	0	0	0	0
6d4204fb2b460538053abe99626d9a4d	0	0	11	0	0	0	0	0	0	0	0	0
6edd3785615b9866587c8a21eb7fa4e9	84	0	0	0	0	0	0	0	0	0	0	0
9909ff534d75540a06f3475b0e942520	0	0	0	0	0	0	0	0	0	0	11	0
17977d9a294349b91b070256dc11ceb9	2	0	0	0	0	0	0	0	0	0	0	0
fd6122dd63b3447a5eb0930ebd3537f3	0	0	0	0	0	0	0	3	0	0	0	0
c6715a7361e98bcf926586f0a6e3393b	0	0	0	0	0	0	0	0	0	0	25	0
d2710e52a246944cd6218ebdc5af8bae	0	0	0	0	0	0	0	0	0	0	0	32
e1a8617397031438662f5b573f7a84fa	0	0	0	0	0	0	0	0	2	0	0	0
c95d1bbb4173b875d50e5a49272ceebf	0	0	0	0	0	0	2	0	0	0	0	0
d2dfd3016b3db3fa132df052dca0a896	0	0	0	0	0	0	0	0	12	0	0	0
da1c77dfded01c43a9468782c1817fee	0	0	0	0	0	0	0	0	0	7	0	0

5aed6c35cc75f0e576493b7e75860196	0	0	0	0	0	0	0	5	0	0	0	0
d2c173758686f3d4674ae4db91afac04	0	0	0	0	0	0	0	0	0	0	0	3
c5a969a9bdcadaaf975bbc8e21a8cdb0	0	0	0	4	0	0	0	0	0	0	0	0
e75880caca4587b4054efc91a49a1fc3	0	0	0	0	0	0	0	0	26	0	0	0
50b2c4855dbd357099955bbf3c181b7f	0	0	0	0	0	0	0	0	0	0	18	0
ec0b23735ba7ca9874c4f4db5c572138	0	25	0	0	0	0	0	0	0	0	0	0
d4b8517f27146a0c3afed74921ecdbe2	0	0	0	0	0	0	0	0	0	0	24	0
76c062b4e0ffabc330fb754547842b87	0	0	0	0	0	2	0	0	0	0	0	0
ca3801c8caad06a19bbc7509de9340c9	0	0	0	0	0	0	0	0	0	0	16	0
e2c3dbd2bfbd6d57314be31ae63c59ff	0	0	0	0	0	0	5	0	0	0	0	0
cefb22d28e2cc3d4115907425f0ba719	0	0	0	0	0	0	0	0	0	0	0	28
85a5d1f2c614af04adc61dff42f3a843	14	0	0	0	0	0	0	0	0	0	0	0
ebcd192de8ec855ac9e866f753cf443c	0	0	0	0	0	0	0	0	3	0	0	0
9baf49bfcf353ac4d774dfbffe6da44a	0	0	0	0	754	0	0	0	0	0	0	0

3ed97be3ae3f342771c0fe896af2df1e	0	0	0	4	0	0	0	0	0	0	0	0
3400d4a2e47fa634b2519657e7ca76bc	0	0	0	0	0	0	0	0	0	0	2	0
8e50b28b62e80df860a72e7c8d47cf4e	3	0	0	0	0	0	0	0	0	0	0	0
7b1b3a36d1340d063beecaf53969cf2f	0	0	0	0	0	0	0	0	0	0	4	0
de55a8de42752e485e00932c940a4fe7	0	0	0	28	0	0	0	0	0	0	0	0
d7ab93df92d2e500a5658fa2967bf7f0	0	0	0	3	0	0	0	0	0	0	0	0
cfc2a7934b59c610b9835b113ce37f02	0	0	2	0	0	0	0	0	0	0	0	0
422ea4e594460111c280e444435499df	0	0	0	0	0	0	0	0	2	0	0	0
7e4b0ef62f8e34bb8f7810f879499c14	0	0	0	0	0	66	0	0	0	0	0	0
bab806dcbd8c1adb848bf7a9c611a93e	0	0	0	0	0	0	0	0	0	0	0	9
8391df75b7305be82b2f3fce59daee55	0	29	0	0	0	0	0	0	0	0	0	0
84b2e91e3bbbac56d6ef0edf4f0e19de	0	9	0	0	0	0	0	0	0	0	0	0
d1b4f6390abf4faf6568e86581be3312	0	0	0	0	0	0	0	6	0	0	0	0
9f92513a75a768d961d227aa1674e193	0	0	65	0	0	0	0	0	0	0	0	0

7cad816a7a47f3b19985a661 99f4c715	0	0	0	0	0	0	0	0	0	0	13	0
02e4e3b0f6e86131381f6412 f5e6ea28	0	0	0	14	0	0	0	0	0	0	0	0
23fcd2226dfde0a079b6129c cfd06176	0	0	0	0	0	0	0	0	2	0	0	0
00589a254fc8e190505d98b1 f27321e8	0	0	0	0	0	0	5	0	0	0	0	0
b5084360e79315cf8733d415 147a2fe8	0	0	0	0	0	0	0	0	0	3	0	0
4734bf72518be42c9e71fb0b 6d8beb93	0	92	0	0	0	0	0	0	0	0	0	0
d999b16a93e0fbb4d0c83770 824e9152	0	0	0	5	0	0	0	0	0	0	0	0
4e31d56ef636adf0b9e5de06 6025533f	0	0	0	0	0	0	0	0	0	11	0	0
6d291115ac59781d2d0af829 1b444b01	0	0	0	0	0	0	0	96	0	0	53	0
88232a7bafa1d68e8697cf0d e3b2c3d2	0	0	0	0	0	0	0	63	67	85	0	0
c42dd5f5431d150d7bfa02cc 6b320fe3	0	0	0	0	0	2	0	0	0	0	0	0
7c31bcb2e2f64a650b725026 d69b6c03	0	0	0	0	0	0	5	0	0	0	0	0
d66b36a5ded520605c64c47f 3c82ef12	0	0	0	0	0	0	0	0	0	0	0	3
09dc4abf739c46043ba9b5ad afd8c1bb	0	0	0	0	2	0	0	0	0	0	0	0

c4cddf67e6f5bd8ee1d4fdcf7703ec6c	0	0	0	0	0	0	0	0	0	0	0	5
48ade0236be77421e8555b4a26687f94	0	0	0	0	0	0	0	2	0	0	0	0
1580e01c0d4819f50c280230263ca18b	3	0	0	0	0	0	0	0	0	0	0	0
c208f742ab0cc62c850520c58a6a4ac1	0	0	0	0	0	0	0	5	0	0	0	0
9cdf39a7d41a3ff65543af99267d9f8d	0	0	12	0	0	0	0	0	0	0	0	0
99e4248404cefcde962c4ae3831ff86a	0	0	0	0	0	0	0	0	4	0	0	0
1aa2bb7800f3dfbce0462f843e220fb6	0	0	0	0	0	0	0	0	12	0	0	0
c0e80b4772f32a376b1f6365048ddd36	0	0	0	0	0	0	2	0	0	0	0	0
986abcb70c05c0f8819ef0fcdec537fe	0	0	0	0	0	6	0	0	0	0	0	0
2168e5a10188cd560b5a5cc09b274601	0	0	0	0	0	0	0	0	0	0	0	3
21e99bce868831d1481206afe0690b39	0	3	0	0	0	0	0	0	0	0	0	0
871f56c4391f84dd3bda735729a64430	0	0	0	0	0	0	0	0	0	0	0	2
be2eb51191d214f7b94ff5e217533e71	5	0	0	0	0	0	0	0	0	0	0	0
d344681bf2aad5f902a0e58439e1b15b	0	0	8	0	0	0	0	0	0	0	0	0

679401d2a429693c46322f49a9104f49	0	0	0	0	0	0	0	0	0	0	4	0
35f77052c32fd38aa4a69285f5a51f64	0	0	0	0	0	0	0	0	0	7	0	0
26953cf3d8799d7ccb0ccd37b8cd6619	0	0	16	0	0	0	0	0	0	0	0	0
734c2fab90b915f757819ea43481eff8	0	0	0	11	0	0	0	0	0	0	0	0
8b81fb1649e952b99a262c20c22331e0	0	0	0	0	0	0	3	0	0	0	0	0
a82869798cf8e9a9c040097475a2d924	0	0	0	0	0	0	0	0	0	4	0	0
62a38b843d6550f61b67c377ff56b14e	0	0	0	0	0	41	0	0	0	0	0	0
c0a4f56c6326f85dc2a6accdaf9128ab	0	0	0	0	0	0	0	0	0	0	2	0
9db9400192e9b9988753965a85fbfa1f	0	0	0	0	2	0	0	0	0	0	0	0
99b0efa8aecb6518b16c4212ae0ec038	0	0	0	0	0	2	0	0	0	0	0	0
3dcedcfc9be68030603970ec3fc0b0f4	0	0	0	0	238	0	0	0	0	0	0	0
e50353939b5eeffbf0499fe9037c3bb1	0	0	0	0	0	0	0	0	0	17	0	0
eb5b435bec95380e42527097c018b93c	0	0	0	0	0	2	0	0	0	0	0	0
ed57018acb290231f2d006fd3e4b53fb	0	11	0	0	0	0	0	0	0	0	0	0

7a7614c2494f2a8c9aac8a4a9faff62f	0	0	0	0	0	0	7	0	0	0	0	0
b270e477dca11416e549e6ec3d0be756	0	0	4	0	0	0	0	0	0	0	0	0
be33b72ad0eab4857061f542d6267ff7	0	0	0	0	0	0	0	43	0	0	0	0
88fd0b64cacdf6f616a13a9333192571	0	0	0	0	0	0	0	0	0	12	0	0
7bf9e83ed5cd6c1942117c4478866944	0	0	0	0	0	0	0	0	0	40	0	0
10eacbfd1dafbc252038c2428d856718	0	0	2	0	0	0	0	0	0	0	0	0
939e8a9cda6f6a4f748717474b20b9a1	2	0	0	0	0	0	0	0	0	0	0	0
2e686faf47ebf5a645c65795a0ddaf1a	0	0	2	0	0	0	0	0	0	0	0	0
f9026b85350780d35051a1fb696b1afe	0	0	0	0	0	0	0	3	0	0	0	0
93c268695410130301a6e59670d9fac0	0	0	0	0	5	0	0	0	0	0	0	0
c877bbefe2abc1c784f1884fda6fa5c7	0	0	2	0	0	0	0	0	0	0	0	0
f4f89e5d0c7f094d944cb69b12250161	0	0	0	0	0	0	7	0	0	0	0	0
95c919db70bfa266e2978d4ae83a58aa	0	0	0	0	0	0	0	0	0	0	0	3
0caa065a87149442b745a3af19ed3e3d	0	0	6	0	0	0	0	0	0	0	0	0

b622206c420a302a9ba240f6ab1ab62b	0	0	0	0	0	0	0	27	0	0	0	0
081ee10d391b91e9afeb1c125707b806	0	0	51	0	0	0	0	0	0	0	0	0
a0fc15fffc840d5e8d756fdff144a86c	0	0	53	0	0	0	0	0	0	0	0	0
4e40daf186dee8f0f58ec9e361cbe9c9	0	0	0	0	0	26	0	0	0	0	0	0
8ad7230cdebf41e10f2118eda0a21785	0	11	0	0	0	0	0	0	0	0	0	0
720b3a957c735a8335bcd5505e30218a	0	0	0	0	0	0	0	3	0	0	0	0
c2c9766dfd1234735e1fdc28fe5a3bac	0	10	0	0	0	0	0	0	0	0	0	0
804ef0ff545532bb6a414cc15c42584b	0	0	0	0	0	0	0	2	0	0	0	0
ea0535d303a1998527c8afee18908c77	0	0	0	0	0	0	171	0	0	0	0	0
ad9305c58713f5aa3eaad704327fd438	0	0	9	0	0	0	0	0	0	0	0	0
81eda05470617ecc7ba78a1df4bdb665	0	0	0	0	8	0	0	0	0	0	0	0
20bddee507a82e91bfaba4bf71cdb4bd	0	0	0	0	0	0	0	0	0	0	9	0
7a0b6f52533b3e69c9d28540bece4b53	0	0	0	0	0	0	0	0	7	0	0	0
9bdb6eecc3d874fc6ccc2a86bda8d2f4	0	0	0	4	0	0	0	0	0	0	0	0

fa9bc80f077dae701469d49ed446f848	0	0	2	0	0	0	0	0	0	0	0	0
b009858d543aab825dc6944706dcb20f	0	0	0	7	0	0	0	0	0	0	0	0
22673f87c223418d391accfc8ba46e73	0	0	0	0	0	0	69	0	0	0	0	0
43e6b5f10e9939b017f4b91df070691c	0	0	0	0	0	0	9	0	0	0	0	0
85fc032f15399d3875bd23d593875bf3	0	0	3	0	0	0	0	0	0	0	0	0
840e8facd89391e2afb57e4ad8a152f0	0	0	8	0	0	0	0	0	0	0	0	0
9e6c0f223b101bc43d80af876a95407c	0	0	0	0	0	0	0	0	0	0	5	0
00f23f98a4651cbbc7ac85539f482ecd	0	0	0	0	0	0	0	0	0	4	0	0
101f797ce59260ee420f1b99b5c43453	0	0	0	0	0	0	0	0	7	0	0	0
a857a37212785cd43d2f12c7796c7a85	0	0	0	0	0	0	0	23	0	0	0	0
98ed986c524f32731730d204cbf5f0aa	0	0	2	0	0	0	0	0	0	0	0	0
9c58a5a943bea8c0fbb79c24101851c8	0	0	0	10	0	0	0	0	0	0	0	0
2fe220ce4d22f1ac63918eb35b1a2db6	0	0	0	0	0	0	0	0	0	2	0	0
312cbd54289f6bf2224dba0ce36ff8b8	0	0	0	0	0	0	0	0	0	0	0	3

ac0482a94dd10216d6d65ec33a30fb3e	0	0	0	83	0	0	92	85	0	0	169	0
cbab79497fa5d106da195b4133245984	0	0	0	0	0	0	0	0	0	0	5	0
0e634ce6c86ca9f8ac557f2f531f04fa	0	0	0	0	0	0	0	0	0	0	2	0
818471b6a9ced0300326a77330e6c2bf	0	0	0	0	0	0	0	0	0	2	0	0
981ff9a840cd2c47d8913fdb994d5f5b	0	0	0	0	0	0	3	0	0	0	0	0
4e561bb5bc59e2f0423b6ae2b24e9e0d	0	0	0	0	0	0	0	0	0	0	4	0
0e8a15c1b6516bc865f872e2751e22ce	0	0	0	0	4	0	0	0	0	0	0	0
eff2fb6ca88da62644e35a477eaa4b6e	0	0	0	0	0	0	0	131	0	0	0	0
e6a4dc3ab44edb6919f2cd24dabac341	0	0	0	0	63	0	0	0	0	0	0	0
cceb0fe1168de00e727dd1f585ea5958	0	0	0	0	0	0	0	0	0	0	0	5
2b586c29d7560132d920ad9023a3dff1	0	0	0	0	0	0	20	0	0	0	0	0
098f3f9deb6e2292ac0a9ed2c389a000	0	0	0	0	0	0	0	0	0	0	0	5
889d059c9b159f5a321741c4aa4dc58d	0	0	3	0	0	0	0	0	0	0	0	0
ae64cb38a5c1c6e1bc6b99c5e8e183fc	0	0	0	0	0	0	0	0	0	0	9	0

6648e04365c0b2cc05c007857a9691c3	0	0	20	0	0	0	0	0	0	0	0	0
e40ee73a21c4b609e6b78a3b2ab982c3	18	0	0	0	0	0	0	0	0	0	0	0
4a321fbfa509fd8df916b0bf0aa5df8d	0	0	12	0	0	0	0	0	0	0	0	0
56d472135c621baaaa4ed3c10a4b9732	0	0	0	2	0	0	0	0	0	0	0	0
ade2fab1f6fd276d5c90d06a0ad4edf3	13	0	0	0	0	0	0	0	0	0	0	0
81d8d631a63e167274bf6d3ebe6aa1b7	0	0	0	0	0	24	0	0	0	0	0	0
1f6956263fe6e2809294d0464e9eda89	0	0	0	0	0	0	23	0	0	0	0	0
fa0da66ca6783a090e70ec5cace8f0a9	0	0	5	0	0	0	0	0	0	0	0	0
0f9cd09770a2f52f5b2779059de8935c	13	0	0	0	0	0	0	0	0	0	0	0
02882a059d06351276fdf737d39e5336	0	4	0	0	0	0	0	0	0	0	0	0
15375ba45ca7c139a15d9b44f9a48a62	0	0	0	0	0	0	0	0	0	0	0	4
32d78195c9883829027a6a7edf154a19	0	0	0	0	0	40	0	0	0	0	0	0
26450b21ebf2146a490c1fcd7b8df704	0	0	0	0	0	0	0	0	0	0	15	0
6f52db24c95375f6c787e07ab641fe38	0	0	0	0	0	0	0	0	0	0	4	0

27b2110946fb96e42c160869 5855cf09	0	0	0	0	0	0	0	0	0	0	6	0
05875280b87dc41c1f64dcf6c 86e332c	0	0	0	0	0	0	0	92	0	0	0	0
030f4818871cb899cb954f73 7e56c72c	0	0	0	0	0	0	0	0	2	0	0	0
1964062d212d1494a1e2323 593fcc353	0	0	0	0	16	0	0	0	0	0	0	0
799045a6bb8c130140a3ddb 0e3373299	60	0	0	0	0	0	144	0	0	0	0	0
0e76b05d77b514e57bb970d 24388e1b0	4	0	0	0	0	0	0	0	0	0	0	0
edecd971d16521f81bfd7e5a 372dc013	0	0	0	0	21	0	0	0	0	0	0	0
2ce69f8683522d30609be96d 6ec20636	0	0	0	0	2	0	0	0	0	0	0	0
b742c27c16db8d2c118e5572 881bce5f	0	0	0	0	0	0	0	0	0	10	0	0
1bec492eda4b31ad50e0eb9 46653645e	0	14	0	0	0	0	0	0	0	0	0	0
96df12533cea2d4315c9623b 0ec89d9b	0	0	0	0	0	0	0	0	0	0	0	45
1d25362722d0fd80d9e1364 0ae9fd0da	0	0	0	0	0	0	0	0	0	13	0	0
5c4212d9b4a57a1c4cb73273 7c2e077c	0	0	0	0	0	0	16	0	0	0	0	0
50e687d28bfe8b30f6541b49 e43ad8c8	0	0	0	0	0	0	0	0	0	0	2	0

45aaaa259a448579e33e359d5843f99c	0	0	0	0	0	0	0	0	0	2	0	0
260f5e96cffb5eaef485a8fbfd3f496b	0	0	0	0	0	0	0	0	14	0	0	0
54fc643143f16e3bef8d06cc1bf79a05	0	0	0	9	0	0	0	0	0	0	0	0
9d6ce4fa2682f5c5146f1b46126a836c	0	0	0	0	0	0	0	0	0	0	0	2
9c9dadddf42ef207db9d3643927d945a	0	0	0	0	0	0	0	0	0	0	592	0
c240bbb92fb698366c7a3ce8813c1380	0	0	0	0	0	2	0	0	0	0	0	0
aacbd728cb1230f54a8be8c2fbf84df2	0	0	0	0	0	0	0	0	0	0	23	0
2a26f61908cf1f8d55ce15fa8439ae3e	0	0	0	0	7	0	0	0	0	0	0	0
672e8ef4b73e04000a8b19512076f714	0	2	0	0	0	0	0	0	0	0	0	0
7bd67c451f2b37c10e78715334e6bc60	0	0	0	0	0	0	21	0	0	0	0	0
1924a14c340a902c9a4e84b95d00b957	11	0	0	0	0	0	0	0	0	0	0	0
607143d86466ec7c51b7528c39627ca6	0	10	0	0	0	0	0	0	0	0	0	0
d85c499d88caf50693a7763ee167a5c9	0	0	0	0	0	0	0	0	0	5	0	0
522e4f7543baff853fe4629b2d7ea801	0	0	0	0	0	0	0	0	0	0	3	0

3676e70cc7766e6b72c9d71be0c82969	0	0	0	0	0	0	0	0	0	33	0	0
a4995b3bbdd367c2ee2f4aabdbc057f9	0	0	0	0	0	0	0	0	0	0	0	3
64857b76f237c97f39be532df76f0d00	0	0	0	0	0	0	0	6	0	0	0	0
d996403f3c975f94c7e4c8f451ad515a	0	0	0	0	0	0	0	0	0	0	4	0
bda5f56c82b0d626869c9e0064b74451	0	0	0	0	0	0	0	0	7	0	0	0
469529105ad075bd479610e7b2afbd96	0	0	0	2	0	0	0	0	0	0	0	0
95a397812297eaf205a8b45158a2969e	0	0	0	0	0	0	0	0	0	0	2	0
5fdc0df80c20cb20ceef3fe43e9e32d8	0	0	0	0	0	2	0	0	0	0	0	0
255f70467a1dc77d6a0be795e251702d	0	0	0	0	0	0	0	0	2	0	0	0
eec5444c9ba8e09627fe1a738d735847	0	0	0	0	0	0	0	0	0	0	7	0
38a892d47893e21d274e9142d6b34d6d	0	0	0	0	0	0	0	0	0	0	31	0
f1d3b05d0e2cc8588649ffb0b233dede	0	0	0	0	0	0	0	0	0	0	3	0
8cbc84fe106a6c9ddba109aebe4bebf3	0	0	0	0	0	4	0	0	0	0	0	0
afad6768ba05cc44b45e75eb9dd10cae	0	0	0	0	0	27	0	0	0	0	0	0

d1919d3c1cd61fda5aaf13fb5faf848a	0	0	0	0	0	0	0	0	94	0	0	0
c876bcf784b902101e2888c32ecdcb7c	0	0	0	6	0	0	0	0	0	0	0	0
4464f4b126231e4aeca6e0f119e5457d	0	0	0	0	8	0	0	0	0	0	0	0
899b2d5cecda6d69f41fb29197be4e55	2	0	0	0	0	0	0	0	0	0	0	0
c4f9b1130b85a214811de46be7948f11	0	50	0	0	0	0	0	0	0	0	0	0
993dd8eee5b10795de3b56812f2cd9ce	0	0	0	0	0	0	0	0	0	0	0	3
624aca216aac7e4e4439a5ffff9df84d	0	0	0	7	0	0	0	0	0	0	0	0
0076af2d4839247e4bf7973eb6c23107	0	0	0	0	0	26	0	0	0	0	0	0
a03225a29ed2bf191f28009db27bc311	0	0	0	0	11	0	0	0	0	0	0	0
5d9846d56af908425ec0b0f82640705a	0	4	0	0	0	0	0	0	0	0	0	0
001f9cb010a69bac0132bd3d8d5f999e	0	0	0	0	0	0	0	11	0	0	0	0
8f1e11bd0511025c688442ec305cbad6	0	0	0	0	0	0	0	0	8	0	0	0
9b48ea0014bebd55970603e19f3c73a8	0	0	0	0	0	2	0	0	0	0	0	0
f7cfee4e7e85580e2306b70b7d962078	0	0	0	0	0	0	0	0	0	0	0	2

6370bce1cf4e9dccba3dd16393ff1e7c	0	0	0	0	0	112	0	0	0	0	0	0
2d58ac71a9c2cf55e9e0e882fccf4355	0	0	0	0	0	0	0	0	0	0	6	0
7ed9b1322be34e7dc19437bf6f76aa12	0	0	0	0	0	12	0	0	0	0	0	0
8854df95916cdfc0621fe2c50574f4d6	10	0	0	0	0	0	0	0	0	0	0	0
aa476f8b9c15c19c215f47a623e78a32	0	0	0	0	87	0	0	0	0	0	0	0
af8c8fa25ff7a42315fd52659df0f705	0	0	0	0	0	0	0	2	0	0	0	0
6ca14e9bff41af8ed1e76af5cd02f0c2	0	0	0	0	0	0	0	0	3	0	0	0
cf4268dcaf587655ad79ab707e2c37ad	0	0	0	0	0	0	0	0	4	0	0	0
d7cca27a4771fb7bf6a9b4328e20acfb	2	0	0	0	0	0	0	0	0	0	0	0
97851b742df56e937c5fd067c49a96ca	0	0	0	0	29	0	0	0	0	0	0	0
cb1c1da7f4c62275655aae479f928741	0	0	0	0	0	0	0	0	0	0	4	0
803e65d5a1822a1ef6f8266650ab22e6	0	0	0	0	0	0	0	0	2	0	0	0
730144d2b9be7477c5f0dfc507986631	0	0	0	0	0	0	6	0	0	0	0	0
591d234d1542139710c9e3310f925d87	0	0	3	0	0	0	0	0	0	0	0	0

6b51748820cda64ca47c8199be6d2263	0	0	0	0	0	0	2	0	0	0	0	0
5b108ddf56893553647c37ba50d51c2d	0	0	0	0	0	2	0	0	0	0	0	0
8a49c6b98e36f4e0fb795f8dfac3aca8	0	0	0	0	0	0	0	0	0	0	8	0
33844b2241e55d363db1bb0e205b381b	0	0	0	0	6	0	0	0	0	0	0	0
f0ee36e770d89bfef24cf10c88b4ca11	0	0	0	0	0	25	0	0	0	0	0	0
6b40a24d2c21a2b92228661b84479673	0	0	0	0	0	0	0	0	0	0	0	11
cef17ef10dfcecdfa4b34212ba16a290	0	0	0	0	0	2	0	0	0	0	0	0
124f0df4f36c78c867532fcf056a9622	0	0	0	0	0	0	0	0	0	0	6	0
2c70d5a88dd89fc4ca4250086f8537b9	0	50	0	0	0	0	0	0	0	0	0	0
8c2f47b4c3c5bd8265226f218f15eeb0	0	0	0	0	0	0	0	5	0	0	5	0
17b8a12de9a9711a5aaaebc409920392	0	0	0	0	0	0	0	0	0	0	5	0
8ebbbe2dc146a3b0f0ea78538edbab3b	0	0	0	19	0	0	0	0	0	0	0	0
49d7daaf879a874320a3b790c52499cd	4	0	0	0	0	0	0	0	0	0	0	0
57f300fd91692c2974a17ea4994b2645	2	0	0	0	0	0	0	0	0	0	0	0

783284bdda0b90eadc77377b630c6174	35	0	0	0	0	0	0	0	0	0	0	0
e5a608ca697108968ef84b822b61bd55	0	0	0	0	0	0	0	0	0	0	4	0
e2a35b8f26cad2fbc45865d27dd2dd69	0	34	0	0	0	0	0	0	0	0	0	0
f3d41f7b5b78afda41dbf63bd2b25a9a	0	0	0	0	0	0	3	0	0	0	0	0
27796b101cc30d784b4c5745a6b8eec7	0	0	3	0	0	0	0	0	0	0	0	0
fd4ff149c40c693e6e0b93d4d25bf961	0	0	0	0	0	5	0	0	0	0	0	0
eedf9cebcd9bacc6d01a0e58e45df036	0	0	0	0	0	0	0	0	0	0	0	3
48eb1127aebbc487f4ac3452afbdb0b9	0	0	0	0	0	0	0	0	19	0	0	0
d18964325dd46a51e40437c75c098eb3	0	0	0	0	0	0	13	0	0	0	0	0
be48a2fadb923d39402f4d097c40f89f	0	0	0	0	2	0	0	0	0	0	0	0
463cab827d1de4ad3d27f2e6b9a89783	0	0	0	0	4	0	0	0	0	0	0	0
607119d0104ce0738e14fc788483493b	0	0	0	0	0	13	0	0	0	0	0	0
d036d82aeedb67d000b56001ee171e7d	0	0	0	0	0	0	0	0	0	0	0	2
5334fcf4ce86addb251a692cb804defd	0	0	0	0	0	0	0	0	0	0	0	10

03ccfda8ea2c33c696bf1f377 6accd4b	0	0	0	0	0	0	0	0	0	0	29	0
b5bc0e260262f0dc6fbfbbb7 461a0b9a	82	0	0	0	0	0	0	0	0	0	0	0
a3a99fcf56633a193fd4868b8 2ad97df	0	0	0	0	0	0	0	15	0	0	0	0
acc04020c3c18da323a32299 ae4e895e	0	0	0	0	0	0	0	0	0	0	2	0
c0667739ff577d0e5f9d42a8b bb342f3	0	0	0	0	0	0	0	0	2	0	0	0
be1ac4dcdab4cbb2c15f8d25 d17c0eb3	0	0	0	12	0	0	0	0	0	0	0	0
8517b7f64b2c372e5e40d57e 05cc471a	0	0	0	0	0	0	0	0	0	8	0	0
2e779a4f1d8f9db67359e500 1a1e54f2	0	0	0	0	0	0	0	3	0	0	0	0
38d008325bc34f7f24ed5b54 66f8ee42	0	0	0	0	0	0	0	0	0	0	2	0
2fc51f2f4785184adc23503ce c6eab58	0	0	0	0	0	0	0	0	0	0	4	0
333b367d284e060120472b7 3a842ed02	0	0	0	0	0	0	0	0	49	0	0	0
2625bfb428fc5309be3ae557 6917bc4a	0	0	0	0	2	0	0	0	0	0	0	0
37477228e629f9fc35cf01149 190f094	0	0	0	0	0	0	0	0	8	0	0	0
5b4f825732a4e7138250a3c9 f6535211	0	0	0	0	0	3	0	0	0	0	0	0

aec6047126f8002f519ce002 82c4a13a	0	0	0	0	0	0	0	137	0	0	0	0
13020e5b2480d34514fd1ac1 8505e7cd	0	0	0	0	0	0	0	0	0	0	4	0
1933bc8da6cc35bd7d68405 d5d7f113e	0	0	0	0	0	31	0	0	0	0	0	0
e18359eb8ecf3ad2d5dad791 225f8cde	0	0	0	0	0	0	2	0	0	0	0	0
cba0933ec57eb40b201c8164 ece1bec6	0	0	67	0	0	0	0	0	0	0	0	0
35bda81686911e49f399076e c6c86eae	0	0	0	6	0	0	0	0	0	0	0	0
674b585951a0f63f084024b5 305b9fde	0	5	0	0	0	0	0	0	0	0	0	0
91f9b8b4ad055cd54c2a2adb 45c00535	0	0	0	0	0	0	0	0	0	58	0	0
9b401e00abe69bbe3dfe0be7 b3bbbba3	0	0	0	0	0	0	0	0	0	0	0	43
a27a3e70e19cda327c2bee26 b6ec8498	0	0	0	0	0	0	0	0	0	0	0	5
37ecdd4b85111565517276cf f1814e29	0	0	0	0	0	0	0	0	0	20	0	0
ccd6375db6c8ae047c2e2c1b e6fb44b4	2	0	0	0	0	0	0	0	0	0	0	0
fb5ec3b5032e7b1398368d1d a8525220	0	0	0	0	0	21	0	0	0	0	0	0
825f417eacee29f18816f84c5 57d9447	0	0	2	0	0	0	0	0	0	0	0	0

937aba0b48eea642acd316d0f6fd2877	0	0	0	0	0	0	0	0	3	0	0	0
c6a0205b66d23bc3a5376688581d07aa	0	0	0	2	0	0	0	0	0	0	0	0
c2d4b176099791d46d5170c09baa83a2	0	0	0	30	0	0	0	0	0	0	0	0
b22e96ad4821cb2d2faf6c2da7b76010	0	0	0	0	0	0	0	0	0	0	2	0
666084f04c673ab5ee72cca8fcdfeddf	0	0	0	0	0	0	0	47	0	0	0	0
ee8001a7da397f58a5c7f6f709d94c29	0	0	0	0	8	0	0	0	0	0	0	0
268b3b9a123eec2fa5bf2aeef256cdd3	0	0	0	0	0	4	0	0	0	0	0	0
0d482a8b2b7c77a61afc327b6495971d	0	0	0	0	0	0	0	0	0	0	6	0
f09032d3240ca8a60489f10300d340ca	0	0	0	0	0	0	0	0	714	0	0	0
8a8bde504aa640f65c477f56500ac926	7	0	0	0	0	0	0	0	0	0	0	0
a3127ed8351625164b8be368c33e95cd	0	0	0	0	0	0	0	3	0	0	0	0
b4b6576538260c7eb3aa9de463c57922	0	0	0	0	0	0	0	0	0	0	0	10
0946db1d6b666300fef13ce5dc7afabb	0	0	0	0	0	0	0	0	0	0	0	2
bb605c01c4e7db023f24d9c7173ad8dd	0	0	0	0	0	0	0	0	0	0	0	7

fb97c473d17d5a7f6514f729 68d52c62	0	0	3	0	0	0	0	0	0	0	0	0
fb5a849c974cbccaead16d0b 7920adda	0	0	0	0	0	87	0	0	0	0	0	0
893c82f9005ca083244a53a8 95ebf40d	0	0	0	0	7	0	0	0	0	0	0	0
7bfe652b5cbb4355a4531b0a d78c4164	0	0	0	5	0	0	0	0	0	0	0	0
7bd567f1074776f63f66e518f 7c76fb6	0	0	0	0	0	0	0	0	0	0	5	0
09fbe980396b9b670a27b96 7b7484e17	0	0	0	0	0	15	0	0	0	0	0	0
246815cd85000d0770f3553b b52a994e	0	0	0	0	0	0	0	0	53	0	0	0
10906ced06c3b79624f31c44 f9ea72de	32	0	0	0	0	0	0	0	0	0	0	0
ddccdfc4afa1d91075ccea58a f6af872	0	0	0	92	0	0	0	0	0	0	0	0
1a8926158c7629ce9bb462f4 d8252d2f	0	0	0	0	0	0	5	0	0	0	0	0
8e55fc0de90ca97e94978687 1181cc5f	0	0	0	0	0	0	0	0	0	3	0	0
39eb743dd59d6ea564a9522 38ec8166c	0	0	0	2	0	0	0	0	0	0	0	0
1b7bf10039a6de9095e8e12d afba102d	0	0	0	0	0	0	4	0	0	0	0	0
5fcbeab071a5f01db7f523a62 d71b8b8	0	0	0	0	0	0	0	0	0	0	2	0

776515de1ec71adc94044cf970e4623c	0	0	0	0	0	0	0	0	0	0	0	2
145740c958fd6078b42d47fb64244322	0	0	0	0	0	0	0	0	3	0	0	0
3dc9317d5994fbc5640c299dc4b122e7	0	0	0	0	0	0	0	5	0	0	0	0
25153d2532f5fe370229fe995736d5e2	0	0	0	0	0	0	0	0	3	0	0	0
01ecd6dc5012cb4791fa5428837f6f78	0	0	0	0	0	0	0	0	0	0	0	10
aca5277460063d0b2a80cbcad8c03c6a	0	0	0	0	0	0	0	0	0	0	0	9
512d9faf7e96af531ddb2e5d240da0df	0	0	0	0	0	0	0	0	2	0	0	0
bfd57fd1b1f3005320fa6281ba0983e5	0	0	0	2	0	0	0	0	0	0	0	0
0c540441815d17468fae7ce1b6d0527c	0	0	0	0	0	0	6	0	0	0	0	0
f35e5081bebf6782d460ed84dcb69a54	0	0	0	0	0	0	0	0	0	0	0	199
43b2184349750fbb70d879595e62e274	0	0	0	0	0	0	0	0	0	2	0	0
5d87fbeb6ee32a6579fc4f2d951ce8f6	0	0	0	0	0	0	3	0	0	0	0	0
2a4585607455e6f520ea2518f48d20c4	0	0	0	0	0	0	0	4	0	0	0	0
9cc60e69fe6c04fc3d49b1abde1e0ee2	0	0	0	0	0	0	0	0	0	0	0	2

2d3edfcc0ac5c96ac71f0f3bad9c9182	0	0	0	0	0	0	0	0	0	21	0	0
60d492fc760b677843a05d7da70b8acd	0	0	0	0	5	0	0	0	0	0	0	0
a76ebb75a3b300d87db01eb3a09943bf	0	0	0	0	0	0	0	0	0	0	57	0
b344c83681b8cf8959dc3dd365585439	0	0	0	0	0	0	2	0	0	0	0	0
605219374cfbee5914e6603e7951dc24	0	3	0	0	0	0	0	0	0	0	0	0
72d6b1056ca558610804a3da9a57dadc	0	0	0	2	0	0	0	0	0	0	0	0
a6b4ed7dbaa10c164af183360b428a70	0	0	8	0	0	0	0	0	0	0	0	0
9e4398ecfc903484d3338e8053c04636	0	0	0	0	0	0	0	0	0	11	0	0
6d48b192a4c14967dc64c3b4acdf6343	0	0	0	0	0	0	0	0	0	0	5	0
fa56570b9df8c0f7ecc52bed6bd96e95	0	0	0	0	0	0	0	0	340	0	0	0
0ec51ac7f865fde015453d5c60890d04	0	0	0	0	0	0	0	0	0	0	0	9
5345fdadf250e034c8434581177a92f6	0	0	0	0	0	0	0	0	0	0	0	2
703b5c5a599698d0efa9936e29d30e1b	0	0	0	0	0	0	0	0	37	0	0	0
68b4681b46cb8454cbab72094c272556	0	0	0	0	0	0	0	9	0	0	0	0

64aab4903a40f80b0622e348f6b6ee38	0	0	0	0	0	0	0	0	0	0	0	3
457c5b8b808df8775b651168fe3a24de	0	0	0	0	0	0	0	0	3	0	0	0
b085329e75620302b53c51b171dfc60b	0	17	0	0	0	0	0	0	0	0	0	0
c2d6e3fe77ce2fb8be9ef51fef1a41b8	0	0	0	0	0	0	0	0	0	0	0	5
8586d2da1014e75afc587f47537a3b95	2	0	0	0	0	0	0	0	0	0	0	0
b20ecd4fedc7e93b58b6bd95f28bd192	0	0	0	10	0	0	0	0	0	0	0	0
9306375fdd639d7bf54464885a27cc53	0	0	0	0	0	28	0	0	0	0	0	0
d579ffeb1ad845b2bfe3e47e4b07ba27	0	0	16	0	0	0	0	0	0	0	0	0
e55f2bc14c53e2b4f29bd07c3a85459c	0	0	0	3	0	0	0	0	0	0	0	0
787bdcb7f48c16678f80ae258be6fa50	0	0	4	0	0	0	0	0	0	0	0	0
a28f29d58eca5610041b108d627972fc	0	0	0	0	0	0	0	0	0	0	0	4
3a3a7ded8c085cfd62910fc79c5d6138	0	0	0	0	0	0	2	0	0	0	0	0
5bed579eef56247ad093e10ad9bb2674	0	0	0	10	0	0	0	0	0	0	0	0
729ab8df3fd8b38f3374add1ba3d5fae	0	0	0	0	0	0	2	0	0	0	0	0

d6a74b846a1b5402269c7d70c172b7a1	0	0	0	0	0	0	0	46	0	0	0	0
78121e99b615522b549012bfd92fd2c2	3	0	0	0	0	0	0	0	0	0	0	0
1c3fb8c9725e3fbbc6f2eb919847c2af	0	0	0	0	0	0	29	0	0	0	0	0
2b3d0957a58c42017e737adb56f7dc95	0	0	0	0	0	0	0	0	24	0	0	0
cb77be40e549796de48f0c9ff1577acb	0	0	0	0	0	0	0	0	0	0	28	0
8dc1a4717b2bc5ac76d490e864ebb3f3	3	0	0	0	0	0	0	0	0	0	0	0
d4163d3a4f739d0a91d40f22dadc9f34	0	0	0	0	0	0	0	0	0	0	0	2
399a80f8425fbbfe7f3e8e25416c0bdc	0	0	0	0	0	3	0	0	0	0	0	0
c49297fc15289334a61da622ae3bc4c2	0	0	0	0	0	0	0	0	0	0	5	0
9623a3ceff7d1859c3647f8dac245d76	0	0	0	0	0	0	0	0	0	0	0	2
546ccd7c2e1f23e009932d0b317ef3be	0	0	0	0	0	0	3	0	0	0	0	0
b3707cf1dccf2a5be365acc61440499a	0	0	0	0	0	0	0	0	0	0	11	0
ceeb0db9a98fbd99065ba4d6e0aff4e4	0	0	0	0	0	0	0	0	0	0	4	0
cacfbb2be05f7b71b6da18a1d1b73136	0	0	0	0	0	3	0	0	0	0	0	0

23af0fbb7a757ebd8020dd47a64bb431	0	0	0	0	0	0	0	0	0	0	41	0
36009d7ef3ca88c6a3e3fcc887446460	0	0	0	0	0	0	0	0	0	23	0	0
c2cc455499f8a5fc57b0b99b80b9286a	0	0	0	0	0	0	4	0	0	0	0	0
93ff1b8b3f5a498ff0b5c90683bbabf2	0	0	2	0	0	0	0	0	0	0	0	0
44781393b851c8dc7de515d974ce41ae	3	0	0	0	0	0	4	0	0	0	0	0
63587600ba68a1518663ca15b3a6a43b	3	0	0	0	0	0	0	0	0	0	0	0
7018db1d72b2568d09fcfe1902f3719e	0	0	0	0	0	0	0	0	0	0	13	0
77e2b2d91da0cc9633a4788f370d3b8c	0	0	0	0	0	0	2	0	0	0	0	0
f67cb76b2866f3cf7292a3418790ff0a	0	0	0	0	0	0	0	0	0	8	0	0
8bb2a32deb7d1b6531ede5957380e61c	0	0	0	0	0	0	0	0	0	0	4	0
52a804f51df64b1471aa998828d9d43b	2	0	0	0	0	0	0	0	0	0	0	0
b55c0eccb7a841d6d2750852872907e7	0	0	0	0	0	0	0	29	0	0	0	0
bfc3623117a093c628996cffddb11381	0	0	0	0	0	0	0	4	0	0	0	0
698762e85283c34ff64ceb1b50a36d15	0	0	0	0	0	0	0	0	0	0	3	0

14041c5a789458376c6d816f9d49e7a7	0	0	0	0	2	0	0	0	0	0	0	0
ac5801e818fcd5181ebb27f97e20c9b5	0	0	0	0	0	0	0	0	0	12	0	0
5f5d9055db36339c75854d0f29b04d50	0	5	0	0	0	0	0	0	0	0	0	0
f3a3cc8c57aa5b4eaf3720613a89c9b3	0	0	0	0	0	0	0	0	0	0	0	12
60d43d6aaad7038f4d4cf13e5312e839	0	0	0	0	0	0	0	13	0	0	0	0
0d2ab14f8dfce76d7f205eb0227401f5	0	0	0	0	0	0	0	0	0	0	0	4
b45b03c36a62564a5c598b0567dd5eff	0	0	0	0	0	0	0	0	0	0	0	6
b1a9aefb31a5d6f7d73719fdd52016bd	0	0	0	0	0	0	0	0	0	0	0	44
0f7b3f31d6434d989d5de506bdc39020	0	0	0	0	25	0	0	0	0	0	0	0
b164069d3ffeb4bc5622d973328a22fe	0	0	0	0	0	0	0	0	0	0	6	0
4e534f2a6958036978b6da57fc7732b8	0	0	0	0	0	19	0	0	0	0	0	0
8afcf55d3fb41e6434a04c0487cb5bc6	0	0	0	0	0	7	0	0	0	0	0	0
fa2a21b64d7015cd6c22413ef279fe18	0	0	0	0	0	0	0	0	0	0	0	70
ad3c599a0336fbf8c6a36841e8f441d7	0	0	0	0	0	0	0	0	2	0	0	0

0c5811436e639bfba2c30d98<br>8716f967	0	0	0	0	0	0	3	0	0	0	0	0
4030583c5efcf8fbd1e6bf97c<br>4aadfd6	0	0	0	0	0	3	0	0	0	0	0	0
41e79bf6d3f841aff02cbde4d<br>59085a0	0	0	0	0	0	3	0	0	0	0	0	0
7669e436946ed4d69a12a15<br>59a66c8f0	0	0	0	0	0	0	2	0	0	0	0	0
0a298930b5d1a0b2b2a16ee<br>ae3f8fa35	0	0	0	0	0	0	0	0	0	0	0	3
1b54293d2085670f758333ce<br>57199847	0	0	0	0	0	0	0	0	0	12	0	0
adb474eb5c6cbea5a201e01e<br>3d419a20	0	0	0	0	0	0	0	0	0	4	0	0
c1698a52616eac4acaa49da2<br>2ac1131d	3	0	0	0	0	0	0	0	0	0	0	0
685bc65e0e7e3bca3abc693c<br>69ebeff6	0	0	0	26	0	0	0	0	0	0	0	0
5d8eae97f7b8de6522bfc8f64<br>9666c8f	0	0	0	0	0	0	0	0	6	0	0	0
5b4a09c2c1cd9e17d4c10e3b<br>6b6e8dfe	0	0	0	42	0	0	0	0	0	0	0	0
2ad1e81eba3f3ea9bb1a5ac3<br>35ff1722	0	0	0	0	0	0	0	0	4	0	0	0
3492e627815b2d4ff5187e61<br>de41ec40	0	0	0	0	0	0	0	0	0	4	0	0
9423f72f7a7079d920ec09f4b<br>e32da58	0	0	0	0	0	9	0	0	0	0	0	0

c503cbe1578ffc720bfd41872d3ebc2a	0	0	0	0	0	0	0	0	0	0	15	0
2db4f0119362a2f741f93d2f675ad1c3	0	0	0	0	0	0	0	0	0	0	0	25
95ae2bd3371e83c6d5f33da17fd933e2	0	0	0	0	0	0	0	0	0	0	0	15
2ad1073a56b6633719499a0bb564df51	0	0	0	0	0	11	0	0	0	0	0	0
c2539d72007595dcff1a579604aefb0e	0	0	0	0	0	0	0	0	0	4	0	0
348ffae6601aeb44337ab171ee41737f	0	0	0	0	0	0	10	0	0	0	0	0
65638e39d524d5082f86b6c5ef085411	0	0	0	0	3	0	0	0	0	0	0	0
6c29aa3340b8f7f6d747b7b7816a48b5	0	0	3	0	0	0	0	0	0	0	0	0
36dc18bd3286e41965531a37a869e6de	0	0	0	0	0	0	0	0	0	30	0	0
c64803a783dc79d2330efc6ef1458806	0	0	0	0	0	0	0	0	0	0	0	13
828b7557e6b57ebd72106aee63227697	0	0	18	0	0	0	0	0	0	0	0	0
186972f6337f338551708651a4a63e71	0	0	9	0	0	0	0	0	0	0	0	0
95d3196c9566ee0b1ad4cd2ed3fd6573	0	0	0	0	0	0	0	4	0	0	0	0
e53fc7b2798f906ac3d140b7a834cf61	0	0	0	0	0	0	4	0	0	0	0	0

f54b05154adbf8677ea82d8eeabd55f8	0	0	0	5	0	0	0	0	0	0	0	0
6cbc5239d61c9ac2dbb6546305e696b7	0	0	0	0	3	0	0	0	0	0	0	0
ca5cbc2ac42495d134e53f0a25c8eb3f	0	0	46	0	0	0	0	0	0	0	0	0
860099543f0f04e8bd00572181893325	0	0	0	0	0	0	0	0	0	0	7	0
5862729150c486fce90172e004752e28	0	0	0	0	0	0	0	0	0	0	12	0
beb4c4ac071fd9d8d9bb6275a4f394a5	0	0	0	0	0	0	0	0	0	0	0	7
a1f4a0543415cea3c16fd599385d79a3	0	0	0	0	0	0	0	3	0	0	0	0
4de625bec515064767b85c064b752de1	0	0	0	0	0	0	0	0	0	9	0	0
c0b80faf14c5487d0bf2c6bb98d03208	0	0	0	0	0	0	0	0	0	15	0	0
380adeddeaab4ca3a62995c1e360cd24	0	0	0	0	0	0	0	0	0	3	0	0
41527641818071c3e3b57e876838499c	0	0	0	5	0	0	0	0	0	0	0	0
99c6e54c031e510f4ffae4b36340d051	0	0	0	0	0	0	12	0	0	0	0	0
41ca45c06f393b23bd35343d72d5a6d1	0	0	0	0	0	2	0	0	0	0	0	0
afe883426dc8fb530dccc68f99aaf17e	0	0	0	0	0	0	5	0	0	0	0	0

867b90a8e3ed561becae31c5b5239288	0	0	0	0	0	0	46	0	0	0	0	0
eca705dc293dbc7e2815a00aceed3285	0	0	0	0	0	0	61	0	0	0	0	0
fb150a22f8a956f044b226751344a2db	0	0	0	0	12	0	0	0	0	0	0	0
99ad1c965f7c545d8c44d74d7470ba58	0	0	0	0	12	0	0	0	0	0	0	0
7e7a0cff746bbc53e61c5bb65dd64ff1	0	0	0	0	0	8	0	0	0	0	0	0
1190fed44e2d497edb5082f14f507455	0	0	2	0	0	0	0	0	0	0	0	0
ece6fb5e571cba786e8f11ba2d72bd62	0	0	2	0	0	0	0	0	0	0	0	0
1af445ba4b4c8513e168404b924e410d	2	0	0	0	0	0	0	0	0	0	0	0
02985c94be8720eb44ad09d7f467c346	0	0	0	0	0	0	0	4	0	0	0	0
9ea3453fe5ee3429d73d718b1c5c256b	0	0	0	100	0	0	0	0	0	0	0	0
8027f6d12f48a01d0ba8116226450e03	0	0	0	0	0	0	0	0	0	4	0	0
453b08a6472f0a90997f30a6e5195fc4	0	0	0	0	0	0	0	0	0	8	0	0
9975879554d5fda0ef8e1b866e321b6a	0	0	0	15	0	0	0	0	0	0	0	0
032f95bf473125e1266a334b29da338c	0	6	0	0	0	0	0	0	0	0	0	0

e0e7b2d35066250d633859f4284cc4de	0	0	0	0	0	0	0	0	3	0	0	0
6a8bd23701dfcc23a9d1de0596dd3109	0	0	0	0	39	0	0	0	0	0	0	0
7e6dc4d90f2ba2798bb6df16c2de2ebf	2	0	0	0	0	0	0	0	0	0	0	0
cacd0e97764722bee5316ad081d733d5	0	0	0	0	0	10	0	0	0	0	0	0
5c8f2dad81ffc5fc28825d454acc0feb	0	0	0	0	0	0	0	0	0	0	3	0
c011ff2cf75c97c5c4413b7a9885fdb5	0	0	0	0	3	0	0	0	0	0	0	0
4b11682d472b90b2d76d0821cd681f3d	0	0	0	0	0	0	31	0	0	0	0	0
b59b72542bd8a07c09a328cb0c77707e	0	0	0	0	0	0	0	12	0	0	0	0
0d91eb4e8fce5ff5ce9473aba08c4f90	0	0	0	0	0	0	0	0	0	0	3	0
2b1b99f7551a9c4abc30e9af13500fba	0	0	0	0	0	0	19	0	0	0	0	0
dc4ef48aa8de3a57f5532cef93c35b1c	0	0	0	53	0	0	0	0	0	0	0	0
3103b62c76a6e6d200e792d687bd747e	0	0	0	0	0	17	0	0	0	0	0	0
1f6263f5159ad62e99b6b77fcdb1b03b	0	0	0	31	0	0	0	0	0	0	0	0
c518d1ad343c9e76ac0b418d84d4c029	0	0	0	0	0	0	0	0	0	0	0	6

4abf086c9e78f6361cafcb72aa34a75a	0	0	0	0	0	0	0	0	9	0	0	0
37fa39f159d9fc729864d3cc3fc980a4	0	0	0	0	0	0	0	0	0	0	3	0
7a7c8928071722a0e64b36519acf5d95	0	0	0	0	0	0	0	46	0	0	0	0
996759ac2ebf2f04b6942ca9902f4860	0	0	0	0	3	0	0	0	0	0	0	0
62748ff4c15116f8e5ecdc602762cbd8	0	0	0	0	0	2	0	0	0	0	0	0
77e05250c7b4ff2094665aa087d6a207	0	0	0	0	28	0	0	0	0	0	0	0
ecd2911c421d742614330ad7b5077b10	0	0	0	0	0	0	4	0	0	0	0	0
812cc5b8366f570e5313eb7f6eb45422	0	0	0	0	0	0	0	0	0	0	3	0
fabacfc30fe6152fefec687fee9732c9	0	0	0	0	3	0	0	0	0	0	0	0
927d02d3f388a09d7fb912ec6b74c8f0	0	0	0	0	51	0	0	0	0	0	0	0
c6a5b8b647e1ca7f1564858085dd17e3	0	0	0	0	0	0	0	0	0	0	0	6
a056b2b2494388239b77ac7550ba871a	0	5	0	0	0	0	0	0	0	0	0	0
4e8ce57482d386c9835313bb8bd2f7b4	0	0	0	0	0	0	0	0	0	0	10	0
49c4be653a2b881833d67cbdf9b96970	0	0	0	0	0	0	0	0	0	0	2	0

e77f9dcf0455b2b24916187d40b9fb72	0	0	0	0	0	0	0	0	0	0	10	0
d2be03f6bf3718cdb69cff8b0a79ab88	15	0	0	0	0	0	0	0	0	0	0	0
43fd4c884aa838414960833191dd3e37	0	0	0	0	0	0	0	0	0	0	0	61
b5a51ef17faa9ab3027f259a8f7f92c8	0	0	0	33	0	0	0	0	0	0	0	0
182e0a6e8fb7cea902315ccefff168e68	0	0	0	0	0	2	0	0	0	0	0	0
36dc0a2679260ac3f3e6b766cbfe3e96	0	0	0	0	0	0	0	0	0	0	0	7
0adbfdebf5e4d05f315f6761849d3134	0	0	0	0	0	2	0	0	0	0	0	0
ce31f9d17241a3825ea2361417e909d2	0	0	0	0	0	0	0	0	0	3	0	0
8eb997b9f169cf0bec3779122126b8ab	0	0	3	0	0	0	0	0	0	0	0	0
f7ac1e2116e4dd26eda95cc540ec719c	4	0	0	0	0	0	0	0	0	0	0	0
6c802987d4fd369e2be9c3ba675f84a8	0	7	0	0	0	0	0	0	0	0	0	0
7eec90e75ad8d4d9ac0211d813088450	0	0	0	0	0	0	15	0	0	0	0	0
73cd571e3f1692d4286339e3e71f374b	0	0	0	0	0	0	0	31	0	0	0	0
03e4b10075f57cae2be5d39d30993796	0	0	0	0	0	0	2	0	0	0	0	0

00d804aba90c8f555c9c9db9bafeabbf	0	0	0	0	0	9	0	0	0	0	0	0
ca22fa0dcdb748bd4d3432e1701d1e4a	0	0	0	0	0	0	0	0	0	0	0	4
a3748afb3bbbe2f904037efa9f847c3a	0	0	0	0	0	2	0	0	0	0	0	0
a74f04530aa75b6024c578a02f7dabcc	0	0	0	0	0	0	0	0	0	0	8	0
1d418677465a76af451c1d168203b31d	0	0	0	0	0	0	3	0	0	0	0	0
d22984546bef3380c43f0a41c645055d	186	115	0	90	202	0	0	255	0	0	0	202
abc4a11c6c9c986ac11420286b7e0a40	0	0	0	0	0	0	0	0	0	0	19	0
196d1b3e0f13647aebe6b9ec7b5ecada	0	0	0	13	0	0	0	0	0	0	0	0
b22f95dd810698bea4b9fa278bc28377	0	0	0	0	0	0	0	0	0	0	0	3
013522c3e75bb67af9a328827b16b111	0	0	0	0	0	0	0	78	0	0	0	0
292e5990a9af71c9283bce0ca3608e1c	0	0	0	51	0	0	0	0	0	0	0	0
9bb52c1ca9fbdfa486d9a58e0148a902	0	0	0	0	0	0	0	0	0	0	4	0
2ae914fa0ba0cce578214287910e02b4	0	0	0	0	0	4	0	0	0	0	0	0
eee5448ed5a3f2db705c85d815ca11d7	0	0	0	0	0	6	0	0	0	0	0	0

a7bf7508aaf3bd5aab90e841 20b9843a	0	0	0	0	3	0	0	0	0	0	0	0
3b43c3ee5e0b92424cde53e d0cc76406	0	0	0	0	0	0	0	0	0	0	0	5
1529dc2dac5c9c5a647db496 042eed44	0	0	0	0	0	0	0	0	6	0	0	0
68e687b095cfebe9b495bbb5 836a5420	0	0	0	0	6	0	0	0	0	0	0	0
b84a4601c72d5f493efcd68f7 d04be44	0	0	0	0	0	15	0	0	0	0	0	0
7887b59120976108c44e860 c34ab3ac0	0	0	0	0	0	0	0	0	0	0	171	0
0f955af28c4531a833b4d6aa 2f12ce1e	0	0	0	3	0	0	0	0	0	0	0	0
b1d7ae319b1bc5982d7a226 6fa71f987	0	0	0	0	0	0	0	0	0	13	0	0
62a977404620b1e0f47dc7f4 92c22b62	0	0	0	0	4	0	0	0	0	0	0	0
d920c62f6a124ac7f9ccbc9a7 d698f55	0	0	0	0	9	0	0	0	0	0	0	0
93dac2c0e17dab07da43e27d d9b94282	0	0	0	0	11	0	0	0	23	0	0	0
86a46dc1c5c719195b663265 4ba6eead	0	0	0	0	0	0	0	0	0	62	0	0
318759a0fefbfe79bb6f55634 204f445	0	0	0	0	0	0	0	0	0	0	0	5
aa7aff5448ce7a9c5c7069bcd 827d2b0	0	0	0	0	0	0	15	0	0	0	0	0

1364eacdc051e7f408ee7c27ce3f893d	0	0	8	0	0	0	0	0	0	0	0	0
60c459e043c87d4ac43b41384efc041e	0	0	0	0	0	0	0	0	0	0	175	52
dde84d0c22dbbc401df35ca919804d69	0	0	0	0	0	0	0	0	0	3	0	0
e998f9841f4ae021212d9f4680454a9f	0	3	0	0	0	0	0	0	0	0	0	0
633d81cce3f53014cbef251bc305f64e	0	0	0	0	0	4	0	0	0	0	0	0
745f9af40690d5ac8c5a650bdaec27a7	0	0	0	0	0	0	0	0	0	0	55	0
c1edfbfaf604bc3fa0d523e83991a3ac	0	0	0	0	0	0	0	0	0	11	0	0
48968de7e1352384e135dccc38106289	0	0	0	0	0	8	0	0	0	0	0	0
48745f48039fb2d7f2f05fbd06902179	0	0	0	0	0	3	0	0	0	0	0	0
4d046ab8aad9236d3e4e30b372ae8b48	0	0	0	0	0	0	0	16	0	0	0	0
2d612756b5d8b58df86dedd8af19a850	0	0	0	0	0	0	0	0	10	0	0	0
ea2c2cac75167826a147a4b13da828c7	0	0	0	0	0	0	0	13	0	0	0	0
276617b846d2a8f0fd4e8fadec1f86bf	0	0	0	0	0	0	0	0	0	0	93	0
d21c56d8dff54f671c39d8054bfad74f	0	0	0	34	0	0	0	0	0	0	0	0

5eaeea732e7443a879c47b39d5456baf	0	0	0	0	0	0	0	0	0	0	4	0
06789092a78cb04176f15f7e270ad645	0	0	0	0	0	0	63	0	0	0	0	0
d2ba6c6e0f00ac6c69a12aceaf18867f	0	0	0	0	0	0	0	0	0	2	0	0
e7fda2b2c6934a20f93b95cad5ad6a32	0	0	0	5	0	0	0	0	0	0	0	0
2282417bb0993a919467a43b93ca2f30	0	0	0	0	0	4	0	0	0	0	0	0
8473066dc8c76182069c3885db10f9b6	0	0	0	0	0	0	0	0	4	0	0	0
fb4db8d24e51655320a42b964077c141	0	0	0	0	4	0	0	0	0	0	0	0
868e5bf564f9967fb18f968ec7b7e0df	0	0	0	0	0	0	0	0	0	0	79	0
72d642a81e26e93c702ed55d72cf6818	0	0	0	0	0	14	0	0	0	0	0	0
a8685e5a03332dbb14312e94df2d14ab	0	0	0	0	0	0	0	0	0	0	3	0
e45251da86255a946794c303d669a953	0	0	0	0	0	0	0	0	0	0	98	0
156ddea6c5f008f9acfc77d1807a290a	0	0	0	0	0	0	0	0	0	0	2	0
79409dbd4ec49827249ee22bd714eccd	0	0	0	0	0	0	0	0	0	0	0	87
4cb1f4ee8a9dc6ac938fb9ea7f2c3776	0	0	0	0	0	0	0	0	0	4	0	0

03ee78ca0b3bfa43efe3392ff d7567ad	0	0	0	0	0	15	0	0	0	0	0	0
a9c8aa3557d554ea4cc5b11b 2554af08	0	0	0	0	0	0	0	0	0	11	0	0
936a759d6c80829d232aab6 5f5d6fcab	0	0	0	0	0	7	0	0	0	0	0	0
2e81d64948f829eab1d6eff2 677790bf	0	14	0	0	0	0	0	0	0	0	0	0
3001c8dda9c47bc7abc13025 c26db2bd	0	0	0	0	0	0	0	0	0	0	2	0
cf505e99908c8253526ccc00 170b22a9	0	0	0	0	0	0	0	19	0	0	0	0
4d8cf081498a8cfc640a247d9 18760b2	0	0	0	0	0	0	0	0	0	0	0	9
effec0f8003a761e7af25b12f5 07bcb0	0	0	0	0	0	0	0	0	0	0	197	0
00bae9d62157bd69464bd25 ac0652f45	0	0	0	0	0	0	0	0	0	0	4	0
eaa7e628496d1c4de3580bd d5d4e7407	0	0	0	0	0	0	0	0	4	0	0	0
ed2900d25969fe84f03456f0 5eba321d	0	0	0	0	0	0	0	5	0	0	0	0
8416e1610588781491fab8f7 4889e7ba	0	0	2	0	0	0	0	0	0	0	0	0
30140c45c128c81991fdf665 b15d4655	0	0	0	0	0	0	0	3	0	0	0	0
4ef2792658c6c7729289c6d4 bf28c234	0	0	0	0	4	0	0	0	0	0	0	0

1c007b3e94a5cbcd09d5997b603c8bb3	0	0	0	6	0	0	0	0	0	0	0	0
b30d19e139d496340335e857a64df58a	0	0	0	0	0	0	0	2	0	0	0	0
93655e21e3c8f1228318dc846bc5c62d	52	185	0	0	0	0	0	0	94	0	0	0
e04670adda54d3fef54b201f1730f456	0	0	0	0	0	0	0	5	0	0	0	0
0d9add6afb0cef6b5364fd2868d80ee5	0	0	0	0	0	0	0	0	0	0	2	0
1136acbaccf36d44c20497dfcfae9078	0	0	0	0	0	0	0	0	0	11	0	0
6ba769ff30994b65c384687bd9882b57	0	0	0	0	0	0	0	0	0	0	0	57
c0a41f3d76e9f84fba0424987a4526d3	0	0	0	0	0	0	0	0	0	0	2	0
b164b6f3713e421275162e5e036111d5	0	20	0	0	0	0	0	0	0	0	0	0
3884019e0e83c8da36ca08734aa019d2	0	0	0	0	0	0	0	0	29	0	0	0
366cec85a3f2d2a0668889f3bb98de9b	0	0	0	0	0	0	2	0	0	0	0	0
69a01b648b9e2e3aeefa394a2976c235	0	0	0	0	0	0	0	0	0	4	0	0
d986b37e186f189f9466db4c181c16e6	0	0	0	0	0	0	0	0	0	0	5	0
7f4ab1d0a8822e6f9893cd0ebc8abc9c	0	0	0	0	0	5	0	0	0	0	0	0

c5da6db85eca1fcbef7695451cb50e68	0	0	0	3	0	0	0	0	0	0	0	0
fcca8177e7dad0760bd90de18e4b9339	0	0	0	0	0	0	0	0	0	0	4	0
98e0ca4448eef37a0fe86e7501f044da	0	0	0	0	0	0	0	0	0	0	0	8
f8662afbe7b781649a00052db4b6b501	0	0	5	0	0	0	0	0	0	0	0	0
3c11f593bfd2dc292811beeed29fd593	0	0	0	0	0	0	0	4	0	0	0	0
984558d69c33f17e88cda2e8d6be83e9	0	0	0	0	0	0	11	0	0	0	0	0
3db2bb2394a9dccba7cd4490001c2227	0	0	0	0	5	0	0	0	0	0	0	0
f19502f6d517de51bcdd1348a4994e42	0	8	0	0	0	0	0	0	0	0	0	0
c179e424eb7ffd20d5f5ea11614b8b85	0	0	0	0	0	3	0	0	0	0	0	0
7a8730b16dcd9c416dd78d65da4b8848	0	0	4	0	0	0	0	0	0	0	0	0
4fc626dcdb708fb61d44875489cac0f5	0	0	0	0	0	0	0	0	0	14	0	0
dce2087ef31ad99a3f6fdd583581936a	0	0	0	0	0	0	0	5	0	0	0	0
f9703224afdc0b401237fbebec520d92	0	0	0	0	0	0	0	0	0	0	0	2
238132f683555529a7651ef277cece9d	0	0	0	0	0	0	0	0	0	3	0	0

7601a5dd25e4efd2635da9473f52d61f	0	0	0	0	0	0	0	0	0	0	0	9
a105f7e1c93071a1f6a42cc43c800faf	0	0	0	0	0	0	0	0	0	0	0	3
ae3962ae7b5f99f964aa7c87797fdd29	0	0	0	0	0	0	0	0	0	7	0	0
7a8d65ba334409b98855dd06254ce2a2	0	0	0	3	0	0	0	0	0	0	0	0
b5a07b4318e16c0e90ab532b276f4c95	0	0	0	0	0	0	0	0	0	9	0	0
64f281eb35c93c486da378e3643c3356	0	0	0	0	0	0	0	0	0	2	0	0
d0bf2c315e775efdb25992b63237f802	0	0	0	122	0	0	0	0	0	0	0	0
1b5ef9aeb320e37697d73e98961c85f5	0	0	0	2	0	0	0	0	0	0	0	0
8903dddc869a09379234c58efc64632f	12	0	0	0	0	0	0	0	0	0	0	0
20bd20ab0e2a78f5dd57a76e806c3f71	2	0	0	0	0	0	0	0	0	0	0	0
06e3c1f8fec0c5ecf0a527c5942a723e	0	0	0	0	0	0	0	0	0	0	9	0
598e24a3f15616bfdce44864af8424d0	0	0	0	0	0	2	0	0	0	0	0	0
0c9307dea41361025a3ab5e1d2e92290	0	0	0	3	0	0	0	0	0	0	0	0
f30e0036ce823b47c5fad627885ed930	0	0	0	2	0	0	0	0	0	0	0	0

08db39d9f1c666015a82e34158c9ebeb	0	0	0	0	0	0	0	0	0	0	2	0
6bb511280c02938f48722e099466829a	0	0	30	0	0	0	0	0	0	0	0	0
0f85ed50aa0227d09ef108cf17a34fd2	0	0	0	0	0	0	0	4	0	0	0	0
c46553f2e0b18432a3f04e545366a852	0	0	7	0	0	0	0	0	0	0	0	0
df809b3a3f723f3bec94cc3caa128227	0	0	0	0	0	0	0	0	0	0	18	0
076a1b134d779169fda085fa18178456	0	0	0	0	0	0	0	0	0	2	0	0
a299615fac92c519424eded4253497e4	27	0	0	0	0	0	0	0	0	0	0	0
238dc589b3b762037bd04e00b999c6a7	0	0	0	0	0	0	0	0	0	0	2	0
43aa5c79df916c03e62bab30dc023235	0	0	0	22	0	0	0	0	0	0	0	0
21875a49440e6fc3649cb369c78df45e	4	0	0	0	0	0	0	0	0	0	0	0
bb09e152b58b11dbf71470712d4b27c5	0	0	0	0	0	0	0	0	0	0	51	0
980edc7cc65f9b1fea332cb1f651b074	0	0	0	0	0	0	0	0	0	3	0	0
c0a035b982809d54b935f3b01817a52f	0	0	0	0	4	0	0	0	0	0	0	0
21c69567c6a738b994318762762fd98d	0	0	0	0	0	0	0	0	0	5	0	0

d45ebd7dcf295dcfd8cf073acbe61581	0	0	0	0	0	0	0	0	0	41	0	0
43dfde82222c44ebec57f43c1fb0e47a	0	0	0	10	0	0	0	0	0	0	0	0
582e877f92badbfecbcb18d715691d78	0	0	0	0	0	0	0	18	0	0	0	0
9560c460627f272d020a99fe9d44b72e	0	0	2	0	0	0	0	0	0	0	0	0
4c3c747a4744ca9461e7212e857ac56d	0	0	0	0	0	0	0	0	0	0	2	0
6ff9cc95851d35cdaec7cb542b0e50fb	0	0	0	0	0	0	0	12	0	0	0	0
64a52702c37dc2303fe261b95d00c100	0	0	21	0	0	0	0	0	0	0	0	0
d13e1f6db32c5a82e96cce3a562c74b9	0	0	0	0	0	0	0	18	0	0	0	0
b4915cca360e4f4aab3d91e0bc742585	0	0	0	0	0	0	0	0	0	0	8	0
f2d774a8865b41caaf89d463a133c3c1	0	0	0	0	11	0	0	0	0	0	0	0
361f6abd461c1704525eb1716886b9ff	0	0	0	2	0	0	0	0	0	0	0	0
eb7833ea75645fb7ad2806c7d86dd0a0	0	0	0	0	0	0	0	0	0	58	0	0
737c188b390291a638aaeabd27a4b0c4	29	0	0	0	0	0	0	0	0	0	0	0
685f2103cf721f1260761d258997516d	0	0	0	0	3	0	0	0	0	0	0	0

d5cefb87bc74b06aa573557d30c86a14	0	0	0	0	0	0	0	4	0	0	0	0
a1e75be9f34538a403b5978319c9ebec	0	0	0	0	0	0	0	0	0	2	0	0
491dc2c163800650902b381e249bd603	5	0	0	0	0	0	0	0	0	0	0	0
a2c787512a2f9d914fe9d57d41d949ad	0	0	0	0	0	0	0	0	0	0	12	0
c01523bdff6396298379d8161754dfba	0	0	0	0	0	0	0	0	0	0	9	0
895ec9a57070a9cc9a3a1c39f4479c23	0	0	0	0	2	0	0	0	0	0	0	0
d5b31879c4d928e1a39ff02bc7d310ac	0	0	0	0	0	0	0	0	0	0	15	0
23af878fba6c6c9b88c9e9334c0f429c	0	0	0	2	0	0	0	0	0	0	0	0
1f0303fe40a7fe70dcb5dd18d701c752	0	0	0	0	0	4	0	0	0	0	0	0
d5a6cd286e5ef613c403036af032578b	0	0	0	0	0	0	0	5	0	0	0	0
c3b6da91bc38fa03e3b05c5b09c8cbfb	0	0	0	0	0	0	0	9	0	0	0	0
da16df9a675f5bc3c642ba7c5dfb7a78	0	0	3	0	0	0	0	0	0	0	0	0
18866f4f578c0e6ed4c46104489a99c4	0	0	0	0	0	0	20	0	0	0	0	0
53e1d51ba96a962a5adc577b6f59c8b7	0	143	0	229	0	0	124	0	52	90	0	276

ae0459ba6d5f5e5b5d78beca6d726a22	0	0	0	0	0	0	0	0	0	0	4	0
ff803f4903269f97339369fc1c908332	0	0	15	0	0	0	0	0	0	0	0	0
af1512ce5aeefcc7cf5595ed5adb39cc	0	0	0	0	4	0	0	0	0	0	0	0
d0456dee7420221df586dffc5b260e34	0	0	0	0	0	0	0	0	0	3	0	0
343baa2623e3a357dc2dfc56fc247944	0	0	0	0	0	0	0	0	0	0	0	4
cab67af95a38bb01fcfc274931387e96	0	0	0	0	0	6	0	0	0	0	0	0
5f0bc52cfbfa01f69a6fd40c54f67f73	0	0	0	0	0	0	0	0	0	0	2	0
bf17f4c927a5c525e64d745bb2ae9b49	0	0	0	0	0	4	0	0	0	0	0	0
bed60e5028332e75ab31e4f3adf0e8e6	0	131	0	47	0	0	0	41	0	139	0	0
fbcccef7fc835e8e96634a94abde5247	0	0	0	8	0	0	0	0	0	0	0	0
ad18e02936ebc576c4074808e5055f69	0	0	0	0	0	0	0	0	0	0	0	9
1ccb96526a659449a25bf4c42fd2050c	0	0	0	0	0	0	0	0	0	0	0	180
628c212807a3aaa692f27a17a15d2042	0	0	0	0	0	0	0	0	0	3	0	0
d7719d7ed0b0f7ae3ab9bdfbc4f4b520	0	0	0	0	0	0	0	0	0	0	67	0

4227da94387e1d6fc63851c3bc703169	0	0	0	0	0	0	2	0	0	0	0	0
e811c40eff107a98bd4533f12e843b0e	0	0	0	0	0	0	0	0	0	2	0	0
caaed05a4c227c0da3fd27f8c3a6ed3d	0	0	0	0	0	0	10	0	0	0	0	0
ca99a0dd2d0cd2be317a19d9dd9db09b	0	0	0	0	20	0	0	0	0	0	0	0
eb0a610bc93cf3ee950f7ec4e8e1cdbf	0	0	0	0	0	0	0	0	0	0	0	65
f6db97b2a18f30cac59ebdb12f6fbbd3	9	0	0	0	0	0	0	0	0	0	0	0
6ad5135452a27d541e55025722891e26	0	4	0	0	0	0	0	0	0	0	0	0
10070c318f0e1d502ba16214e4d76b1c	0	0	0	0	0	0	0	0	47	0	0	0
4e29ba9339d6a941a32da9c2b8870ae8	0	0	2	0	0	0	0	0	0	0	0	0
422f2377cd3c21447292cbdf5283b74d	0	0	0	0	0	0	0	3	0	0	0	0
341fe684db9dc6f747a67c2c1b858e2b	0	0	0	0	0	0	0	0	0	0	5	0
40f83e2fffcf148f75a15706bcdd4dd3	0	0	0	0	0	0	0	0	0	0	35	0
270e68293442c394d3d485377129c610	0	0	0	0	0	0	6	0	0	0	0	0
5a3430b9e33cd69658a82ac4ce9f7d93	0	0	0	0	0	0	0	0	34	0	0	0

b0e6e37291544faab0b74d58e085effc	0	0	0	0	0	0	0	0	0	14	0	0
14a15e3bad6120305cf353735c11d7a1	0	0	0	0	0	0	0	0	0	3	0	0
183d33b53688583cf055972299acae5e	0	0	0	0	0	0	31	0	0	0	0	0
237e882ff6b58eeb4a3f9194fad18d48	0	0	0	0	0	0	0	0	0	0	25	0
dfa642f921845ca0ec261fa1ca0d0572	0	0	0	0	0	0	0	0	13	0	0	0
7e172e5182c8256780c5456a69834ed3	0	0	0	0	12	0	0	0	0	0	0	0
b79a8efeac0af6de887d5f7791376f3c	0	0	0	0	0	0	0	0	70	0	0	0
bf88a78708ab43d738644ba73a708348	0	0	0	0	0	0	0	0	0	0	0	9
4211a9a4443c85cedded98a6624ddbad	0	0	0	0	0	0	0	0	0	4	0	0
8a3806861c7692a0d92b3eb2e1cd9614	0	0	0	0	36	0	0	0	0	0	0	0
1bc35c2a7aa88f98b9dded0572f16196	0	0	0	0	0	7	0	0	0	0	0	0
a4f62cca6aa6571122c49a2d9f98a175	0	0	0	0	0	0	0	0	0	0	23	0
3ebe318ae706e32f4f711365b4c5c471	0	0	0	0	0	0	105	0	0	0	0	0
61e468744bd76e0a02e62e9fde195d8c	0	0	0	0	0	2	0	0	0	0	0	0

865cfeb2945c669e74c12e27 9d99e55e	0	0	0	0	0	0	0	123	0	0	0	0
6ae9e961635d499ad16251b 13bf8f0f4	0	0	0	2	0	0	0	0	0	0	0	0
b5cf3d015ac7e6cc50bacc12e 32177a9	0	0	0	0	0	0	0	0	0	0	3	0
9d50b842d788dc2d5d3b12f 131cf74ce	0	0	0	0	0	0	0	3	0	0	0	0
8402ce45d25adcc1d46c5df7 5168dcb4	0	0	0	0	0	0	0	0	5	0	0	0
ea320ec4c4cc483785fa7084 a9deae11	0	0	0	0	0	0	0	0	0	0	3	0
35b4b50288ea723b458178f1 61bd9ac6	0	0	104	0	0	0	0	0	0	0	0	0
2dcdd8d9b4dd5d4c5ce9dea d92ff8786	0	0	0	0	0	0	0	0	0	3	0	0
4a594641034ef22298f6331a a601a439	0	0	0	0	0	2	0	0	0	0	0	0
369c7042b769cf0613790b5a 799101fa	0	0	0	0	0	0	0	0	0	0	3	0
dd29bb26a384d022f676607 3ff45c029	0	0	0	0	24	0	0	0	0	0	0	0
b72ec11340dd9e8cb926eb0 9b16cfae6	76	129	0	0	0	0	0	0	252	0	221	0
8e8d764d0c2920e718b28cc7 e8b9188d	0	0	0	0	0	0	0	0	0	46	0	0
1f143d815c3d511490254671 8cd5748f	0	0	109	0	0	0	0	0	0	0	0	0

5786035dd0da65c06a40b28b88101248	0	0	0	0	0	0	0	0	2	0	0	0
b0341c63e54ed6037fb0c4e5be4184d3	4	0	0	0	0	0	0	0	0	0	0	0
d445516e76a43e16c3e74fddb81b26e7	0	3	0	0	0	0	0	0	0	0	0	0
6584e7ea710c1efe00b5aba7d066c1a8	0	0	0	0	0	0	0	0	0	0	101	0
b96f9da97fd79432a171fc9f490f3b35	0	0	0	0	0	0	0	0	0	199	525	0
0b6c5c87532a81d559654fa2b2e5c759	0	0	0	0	0	0	0	0	0	0	0	6
a2bac9c202dbfd95a858d7e3f0a3c34b	0	3	0	0	0	0	0	0	0	0	0	0
2d0e002a8ce8d28aa1c6b3167819b158	0	0	0	0	0	0	0	67	0	0	0	0
a481bbfea4598134ef91848349b31ef7	0	0	0	0	0	0	0	3	0	0	0	0
999503c4c2d986494317c26fd51d1e19	0	0	0	0	0	0	0	0	0	6	0	0
be5bb785ef68834ec23de068ba74bbf5	0	0	0	0	0	0	0	0	0	0	0	51
1326674c6e5db4b67116e8ef76a5ac2f	0	0	0	0	0	0	0	0	0	0	5	0
3f2a6a28224393cb0fb401149e0d5959	0	0	0	2	0	0	0	0	0	0	0	0
484fc037e3e5096772d71530d2c4d2a5	0	0	0	0	0	20	0	0	0	0	0	0

66b797d4ce895962f8ca9d68dfa3b5d3	0	0	0	0	0	0	0	0	0	9	0	0
f3d82abd6c3bd9549a6af6e3a43cb034	0	0	0	0	87	0	0	0	0	0	0	0
95bde2b29d0b2225a9d5b72f895411ab	0	0	0	0	0	0	0	0	0	0	0	3
6d747691a2aca67913cebe2f9f5a0524	0	0	0	15	0	0	0	0	0	0	0	0
a13b2bb6ceca841d16c9f3dd3d805518	0	0	0	0	0	11	0	0	0	0	0	0
ced42748110bec845331bc04ecdcdcc4	0	0	0	0	0	0	0	0	0	6	0	0
1462cfc2a77ae3447e20c61948ec1b35	9	0	0	0	0	0	0	0	0	0	0	0
3836083bc4ae15072df48762ed768e75	0	0	0	0	0	0	4	0	0	0	0	0
d0cf3a0dc5ec54d181b88082aa7a18f3	0	0	0	0	7	0	0	0	0	0	0	0
084b9cc56c7d49ab2c5a76eaba55853d	0	0	0	0	0	0	0	0	0	0	0	8
1f845ada035a08587e758bad8d022034	0	0	0	0	0	2	0	0	0	0	0	0
f05326573a2508f15d9fa13537caec8a	0	0	0	0	0	0	0	0	0	0	4	0
955892cfea0d24012d3511cde1a2d3e5	0	0	0	0	0	0	0	0	0	0	2	0
da797ffd8a66bf0f0c2171b02fb080dd	0	0	0	0	0	0	0	0	0	0	3	0

ed82a16b61bd0282bc7b08f1185c0d8d	0	0	0	0	0	3	0	0	0	0	0	0
a3438af6dd25b329305fe288dbe7b0bb	0	0	0	0	0	0	0	0	0	0	9	0
807239e3238dcd27361861594272908a	0	0	0	0	0	0	0	0	0	2	0	0
e7ed79b69558dbc40006423cc4894f68	0	0	0	0	0	2	0	0	0	0	0	0
4da1b3311284b99143f17f1eaeb4f1f0	0	0	0	0	2	0	0	0	0	0	0	0
7bc4859794d3a880336b12c52ae6bea1	0	0	0	0	0	0	0	0	0	0	32	0
e350d1cfe95c76207cef6de9c6d0f09c	0	0	0	0	0	0	0	0	0	1301	0	0
c3d3c3af3bdfd57de211e654144d6b1a	0	0	0	0	0	0	19	0	0	0	0	0
ee5c790b611805e73341d65e197d9bbb	0	0	0	0	0	0	0	3	0	0	0	0
4b127fcfd0414afe41552044ee89207b	0	3	0	0	0	0	0	0	0	0	0	0
dca2e0521d5de37535fd3959d844c971	0	0	0	0	0	0	0	0	0	59	0	0
503be2c6ca09620330053a2d59085f01	0	0	0	0	7	0	0	0	0	0	0	0
a02f134b76e5f1ea935aba2664d9abd5	0	0	0	0	0	0	0	0	71	0	0	0
4e9c4e68bc6a1a16ec8327df9134f54b	0	0	0	0	0	0	0	11	0	0	0	0

89c4523e350b378e2e3f7f0a0a8dba91	0	0	0	0	0	6	0	0	0	0	0	0
c4443ba1b52da8af4448a05454dc94ce	0	0	0	0	0	4	0	0	0	0	0	0
96427162bef562bbc9d8db8cbbca31bd	0	67	0	22	0	0	0	0	0	0	0	0
ef5b8d9e0394016a38899648652ccdf8	35	0	0	0	0	0	0	0	0	0	96	0
e016160974779b181cae66f12e297f39	0	0	0	0	0	5	0	0	0	0	0	0
db3352e58144171a2fa2a56078ef0096	0	0	0	0	0	0	0	0	0	0	29	0
ac98e759092d366d5b23fe4f9b7b0ea5	0	0	0	0	0	0	0	0	0	3	0	0
8c835e2a3033d61d9a4dc92edf1b7644	0	0	0	0	0	4	0	0	0	0	0	0
8a26f4572cb76ea675fad38e33a6894c	0	0	0	0	0	0	0	0	0	0	10	0
095c1c10986c9767f30b5c8f5546383b	0	0	0	0	0	0	0	0	0	0	0	10
4f444e19e1912f6ef79ec1aaa677afc3	0	0	6	0	0	0	0	0	0	0	0	0
d06ba144c324efea374d3528265c92b2	0	0	0	0	0	0	0	0	0	0	4	0
f5dba51b8ae4fcf4bbb52019fc82b80d	0	3	0	0	0	0	0	0	0	0	0	0
34ebb02c480979febd6e66c4e67f03c0	5	0	0	0	0	0	0	0	0	0	0	0

67c3b00fc3447473129c250b462a5845	0	0	0	0	0	0	0	0	0	0	0	37
fa39cd8ec5983f3d05d9b3494a209e7f	0	0	0	0	0	0	0	0	2	0	0	0
deda73a775ed0952df273d46097cd2a0	0	0	0	0	0	0	0	6	0	0	0	0
6624e32760aa5f3edc072b7915fc6aa1	3	0	0	0	0	0	0	0	0	0	0	0
cb74909128cfb1ee88cdada26bfbea42	0	0	0	0	0	0	12	0	0	0	0	0
01ca6126a9502a2951150b3b43ce40db	0	4	0	0	0	0	0	0	0	0	0	0
cb33b770d29fe3d52368740f413187a3	0	0	0	0	0	3	0	0	0	0	0	0
f50b036d00b99af79e8206d286248e4b	0	0	0	6	0	0	0	0	0	0	0	0
756aa25cb922275943126e7a8c77de71	0	2	0	0	0	0	0	0	0	0	0	0
2373786dea12e4304c21c488979e5d7f	0	0	0	0	2	0	0	0	0	0	0	0
b1751dc07c4ff79d689444b7d6e1de86	0	0	0	0	0	0	2	0	0	0	0	0
76e26850ccb25e3cfab650c5f5527e0f	4	0	0	0	0	0	0	0	0	0	0	0
d5969a42325c6713cd8dfd598c4f62f4	0	0	0	0	0	0	0	0	0	0	0	7
0180b23afd93b4b43ca8c459fd862090	0	0	0	0	0	0	0	0	0	0	7	0

d0e6836bc14e0970fb30573682d5f4ce	0	0	0	0	0	12	0	0	0	0	0	0
87786bf0cde65003f0afaba8fca0de3e	0	0	0	0	0	4	0	0	0	0	0	0
8b97dd519cf468e4185a6d63c4883714	7	0	0	0	0	0	0	0	0	0	0	0
a144239879979d587023c8db1985b30a	0	53	0	0	0	0	0	0	0	0	0	0
33b064bc7dd572b63aed94c4c30a0612	0	0	0	0	0	0	0	5	0	0	0	0
05b7b1576fbf5d09991fb3305850d4a8	0	0	0	0	0	0	0	0	0	3	0	0
d2eeb3e9067927871767cf82fdcbf4fc	0	0	0	0	0	0	4	0	0	0	0	0
e440e58b776a81d7d720fa9235549077	0	0	0	4	0	0	0	0	0	0	0	0
67f9445f30fbded382837bb836f53993	0	0	0	0	0	0	0	0	0	3	0	0
ef7936104b838d0bccf774fa6778ab28	0	0	0	0	0	0	0	0	0	0	0	18
9a381aa91093020d8ae8716c397f2db1	0	0	0	0	0	0	5	0	0	0	0	0
2454897f10d47545781f9a479d47d762	0	0	0	0	0	0	0	0	0	0	0	41
8f5a4a6dd511be09c83595f670fc9531	0	2	0	0	0	0	0	0	0	0	0	0
40e532d3b453c9e4f141834c8b9abead	0	0	0	0	0	0	0	0	6	0	0	0

901ede11eaf7ae461c6bd22e69be18a9	0	0	0	0	0	0	0	21	0	0	0	0
1c8082df58b8e74e051e77f98e1aaf61	0	0	0	0	0	0	0	0	0	0	0	2
9c2f861f1a30e0ad96c771c05aa090ce	0	0	0	0	0	0	0	0	0	0	0	14
5aabcbe44f25fe9d263c97efd3c8a183	3	0	0	0	0	0	0	0	0	0	0	0
a0c9fdfb75484f5fb7fdd5a81a49070b	0	0	0	0	0	0	0	5	0	0	0	0
eaef6dd918bc5a88d8b01e9129087519	0	0	0	0	0	0	0	0	0	0	4	0
3a4cbe0cddefefed5c821df3c9f6e54a	0	0	0	0	0	0	0	0	0	4	0	0
1433c64e06fa12f85da218c5522ee25a	0	0	0	0	0	0	0	132	0	0	0	0
46097ebcadf6ce5559bd011fc7e24ed6	0	0	0	0	0	0	0	0	0	13	0	0
f1aff5f1f77c2292fcc0928b55c9f5cf	0	0	0	0	0	0	2	0	0	0	0	0
40d61c45dd81eebf6aa0effae35131f6	0	0	0	0	0	6	0	0	0	0	0	0
a4f150266b95c4490165ba264d81642c	0	0	0	0	0	0	0	0	0	0	0	8
c6cd9aee5be21a6cacd94a54f96f7b11	10	0	0	0	0	0	0	0	0	0	0	0
d21ea3c74b72b235c952597c67efe2e2	0	0	0	0	3	0	0	0	0	0	0	0

7666a218d14cc6e584be94f15d788541	0	0	0	0	0	0	0	0	8	0	0	0
33f6cb811129e8af2ec712e1a8849e93	0	0	7	0	0	0	0	0	0	0	0	0
4d6e8a0177daf6e21506aa53b77f0679	0	0	0	0	0	0	0	0	0	0	0	3
96f8961de63455e85cc55db44d91e3f7	3	0	0	0	0	0	0	0	0	0	0	0
f48b4ce27e2a421fa83f74b14b760ee5	0	0	0	0	0	0	0	0	3	0	0	0
473d7c5c53b047261816656c53d4bc73	0	0	2	0	0	0	0	0	0	0	0	0
927c6e1a8ae1643a858bfb5dcbc539fe	0	0	0	0	0	0	0	0	3	0	0	0
64cade79c6105f4f2d513211d61dc20f	6	0	0	0	0	0	0	0	0	0	0	0
7c4cc49b05f9e3e46c8ded632167cdc8	0	0	0	0	0	0	0	0	0	755	0	0
728279116048ab12476c6e17b1490ad8	0	0	0	0	0	10	0	0	0	0	0	0
9469cafce4cab8d6d0e8c380230ecfe0	0	0	0	0	0	0	0	0	26	0	0	0
fdc66a9989f740541fac40068ad8292e	0	0	0	0	3	0	0	0	0	0	0	0
928c9d7c6945be3973ab8599c3e02463	0	0	0	0	0	0	0	3	0	0	0	0
ee2940451632d3ed38fe85cc00c628d0	0	5	0	0	0	0	0	0	0	0	0	0

265558a858573a197af1f341 82d39f72	0	0	10	0	0	0	0	0	0	0	0	0
ebbdd55ae730f30308239bdc 51d3d43f	0	0	0	0	0	0	0	0	0	0	8	0
557ecc5d9c4ede9249459108 419b795a	0	0	0	0	2	0	0	0	0	0	0	0
a47668e5de73ea126e7d05f5 9bbaf77c	0	0	0	0	0	0	0	0	0	0	0	2
5b956c1a5321bb8fb6a6388e eb6d336c	0	0	0	46	0	0	0	0	0	0	0	0
efe6c696e92885736de9ab18 7c45a34b	0	2	0	0	0	0	0	0	0	0	0	0
76e7e3ef1f56149493cac4f9a d635802	0	0	0	0	0	0	0	0	0	0	2	0
3f34e03723f47cda94fd9f8bb 0c29162	0	0	0	0	0	0	0	35	0	27	0	0
66178605176dcf54bcb71b76 9565ed2a	0	0	0	0	0	0	0	0	0	4	0	0
18568b052d3f59aeee1b627d f0da0c74	0	0	0	0	0	5	0	0	0	0	0	0
ec217205375805d597c4000 77cf40c81	0	0	0	0	0	0	0	14	0	0	0	0
f0c76f0687be7520e70d0994 61ea7bee	0	0	0	73	0	0	0	0	0	0	0	0
d3283171db55a3f7e4d733e6 d22eafd3	0	0	0	0	0	8	0	0	0	0	0	0
767d1c0b080181718e2be2d bed9c38ea	0	0	0	0	0	54	0	0	0	0	0	0

0b2f54d235ad0c1d598db0986af61087	0	12	0	0	0	0	0	0	0	0	0	0
7b10c2c4cb7dd7b795b475eae5cf3181	0	0	0	0	0	19	0	0	0	0	0	0
51001831cb44a53d8b7fedf679a72e92	0	0	0	0	0	0	0	0	3	0	0	0
c162b86b45617c445a3585585e1e640f	0	0	0	0	0	0	0	0	0	0	25	0
f2eccb023fa1bd7e86a42af9dbd32c88	0	0	0	0	0	0	0	0	0	0	5	0
0e3efc3a54c7aaa972f194a72476ab0c	0	0	0	0	7	0	0	0	0	0	0	0
d17e4f400aec33bbff5a6170b8481f2b	0	0	0	0	0	0	0	0	0	0	9	0
e89f5a99682d086fc6c9a75f6ace885a	0	0	0	0	0	0	0	0	0	6	0	0
bd8f9f3d3698c8d98d08d4becc92d5a6	0	0	0	0	0	0	0	2	0	0	0	0
cb1ea32b6b36d7653e16b399d763867f	0	0	0	0	0	0	4	0	0	0	0	0
33c268b6b768540663a49b770611f3ea	0	0	0	14	0	0	0	0	0	0	0	0
580c7e707efa7f5b0808258f9d059c75	0	0	0	0	0	0	2	0	0	0	0	0
85da822dd6ec3544826e91ded85c5479	0	0	0	0	7	0	0	0	0	0	0	0
c7d4ff0a1205e0b94888d5a38d4dc7ab	0	0	0	0	0	0	0	0	0	0	31	0

b989333a01b7116e936652d ad1937140	0	0	0	0	0	0	0	0	0	0	3	0
404b6f3f2589bd8ce9c39aa1 babf1713	0	0	0	0	0	0	0	3	0	0	0	0
a0984671cc63b66c19a2f5f75 6b3aad1	0	0	0	0	0	0	0	2	0	0	0	0
f0f83198309e2cd19bb3a00fa c531498	0	0	0	151	0	0	0	0	0	0	0	0
f9bc343b355a9f5e6add3b02 6bf74db8	0	0	0	22	0	0	0	0	0	0	0	0
324056de7196999b838b008 b50fd3c89	0	0	0	0	0	0	0	0	0	0	0	2
a7c53cf1c64cf63a10cfaed21 285adc1	0	0	0	0	0	0	0	0	0	0	0	4
5491645ee8db2926b9de087 d17a6a884	0	0	0	0	0	0	0	0	4	0	0	0
32096976348f8eb823649844 b1fb2b91	0	0	0	0	0	0	0	0	3	0	0	0
95aed8e1df84cf1dbad1e74e 60787704	3	0	0	0	0	0	0	0	0	0	0	0
39abe31d7e86c77e6093075 97198c7e3	0	0	0	0	0	0	0	0	0	3	0	0
17b3d8c815f10a8ce94ce03f 86714462	0	0	0	0	0	0	0	0	0	0	0	14
b8de7389b7c14a5127cc0195 9b40d59e	0	0	0	0	0	0	0	0	0	62	0	0
893b2c26a4a8d9f0ad15352a 38f22231	0	0	0	0	12	0	0	0	0	0	0	0

77d56e431b6d96064b39fc177a3ac0d3	0	0	25	0	0	0	0	0	0	0	0	0
545541a9701a9f98103daff9e54c551c	0	0	0	0	0	0	0	0	2	0	0	0
4a412490a59975063a802d1812042c51	0	0	0	0	7	0	0	0	0	0	0	0
c3a5518d46dc0477e9c21db37897e1c3	4	0	0	0	0	0	0	0	0	0	0	0
d1da4d9b10b6f8c53f4cafa519c7e404	0	0	0	0	0	0	0	0	0	0	0	4
808b7b295e582d8d60c987c03473d7ee	0	2	0	0	0	0	0	0	0	0	0	0
a8889759360f97695b796e46d66f9807	0	0	0	3	0	0	0	0	0	0	0	0
54c0c2c19c3d7552b4fdff3330b48dfa	0	0	0	4	0	0	0	0	0	0	0	0
12c325ba3ffe5cbc8f940d606ff51c1c	0	0	0	0	0	0	0	0	0	0	4	0
d618652ed6cf696662023c2dd25bbb83	0	0	0	0	0	0	0	0	0	0	6	0
1d20bb846004fecbf009cc6adaa77fdd	0	0	0	0	0	3	0	0	0	0	0	0
e1f925d88dfe6c15e74666592fe95ed8	0	0	0	0	0	2	0	0	0	0	0	0
210e9314160354d74393ff6d7b6ae51b	0	0	0	0	0	0	0	0	0	49	0	0
1ad0ddbe62aa8e9938a3f7d1f5c88afe	0	0	0	0	0	0	0	0	0	0	0	2

105841e98b4cef16a4b55d8c3e65fc90	0	0	0	0	0	0	18	0	0	0	0	0
21cf9a5b5a084ccecf063c7edceda674	0	0	0	0	0	0	0	0	0	6	0	0
04091901217b83a84de786ca87d1343f	0	0	0	0	0	4	0	0	0	0	0	0
920ed8ff473310cf6552bdd97d86f1a4	0	0	0	0	0	0	0	0	11	0	0	0
63047f63ef5d707aa88da282e768a50a	0	0	0	0	0	0	0	0	0	3	0	0
05be82bea0f8fed81ad873d18dc7551d	0	0	0	14	0	0	0	0	0	0	0	0
ad605b08403665168b8d834be0e18064	2	0	0	0	0	0	0	0	0	0	0	0
e01be4e1396350c8920e07c38cd68c83	0	0	0	0	0	0	0	0	0	0	2	0
fac9a5dee7d6aa927fa3497dde97fc74	2	0	0	0	0	0	0	0	0	0	0	0
604ea5e59c3926786692aed5ce9f11c6	0	0	0	0	0	0	0	0	0	3	0	0
fcd4f1e5fddd06faa7b89a5a5dcf6d2a	0	0	0	0	0	3	0	0	0	0	0	0
0a37547aa11a3a02f364a17294593b97	0	0	0	4	0	0	0	0	0	0	0	0
0e5e70709b42b8872b5919d45a299e5b	11	0	0	0	0	0	0	0	0	0	0	0
ff1e75ef9e2f367d5e123c2c4e4cc167	0	0	0	0	5	0	0	0	0	0	0	0

5cfd4d8a164f88c457e6b3ca6419e8b1	0	0	0	0	0	0	0	0	2	0	0	0
2e09775a81ef867f9f18f01a8e82c8cb	0	0	0	0	0	0	0	0	0	6	0	0
b5da38962c96ccf2849e9db269cda7c4	3	0	0	0	0	0	0	0	0	0	0	0
fe79ec400debca18712004b68d17664d	0	0	0	0	75	0	0	0	0	0	0	0
305ffbf6b4025e42f568b420e173d5d4	0	0	0	0	0	0	0	0	0	2	0	0
975cf615a9b08b2cdaa93f2166d4fccf	0	0	0	0	0	0	0	0	0	0	0	6
23c1efa601ad67306f7e733df3862f45	0	0	0	0	0	0	0	51	0	0	0	0
e720f359036df9ea7c111515fc731959	0	0	0	0	5	0	0	0	0	0	0	0
1b05ce94d8328cf14d0b6a38b27d3066	0	0	0	4	0	0	0	0	0	0	0	0
844c06039cf6de13b7730b67fcd14138	0	0	22	0	0	0	0	0	0	0	0	0
fa0e580da900506294ace926a3183119	0	0	0	0	4	0	0	0	0	0	0	0
6060e82b40d3cf206528ca1f006a85e0	0	0	0	0	0	0	0	0	0	0	3	0
5304085eaed6d7f3344587ec55d0a157	0	4	0	0	0	0	0	0	0	0	0	0
ef5aeeab6348307c2e2febaa09dff83e	0	0	0	0	0	0	0	0	0	7	0	0

91c692396f6c6ae09a2900e6f543c344	0	0	0	0	0	0	0	0	0	0	0	14
d6a8bad675874e69228efa818a2d18a6	0	3	0	0	0	0	0	0	0	0	0	0
6ef87627404f3831f9640579647ce3c7	0	0	0	0	0	0	0	2	0	0	0	0
1e457b9dec9f63dfe0029363f45bf322	0	0	0	0	0	0	0	2	0	0	0	0
2afbbaa9267bb6c7b77504196dba51c9	0	0	0	0	4	0	0	0	0	0	0	0
510a58f4b61f55375ae4d2240de2c4b9	2	0	0	0	0	0	0	0	0	0	0	0
313712c78adfd78f7a0f6485a18da153	0	0	0	0	0	0	0	0	0	0	3	0
7eaa714ffa7550e9369de4dc95cd885e	0	0	0	0	0	0	0	0	0	0	0	4
af219c307f4f1e07907e5b960fa1e89d	0	0	0	0	0	26	0	0	0	0	0	0
19450c36ee8de3c79f088325351c5d87	0	0	0	0	0	4	0	0	0	0	0	0
5873076f696f7ae08b2088abbeffe763	0	0	0	0	0	0	0	0	0	0	0	19
3fbdb4504aaa49c9a486c5525023800d	0	0	0	0	0	0	0	0	0	0	0	3
1432cd85d32d200bb91b7044ff810ec8	0	0	0	0	0	0	0	0	0	0	6	0
f91eaa207a2c26e1aff425d9d9de6dda	0	0	3	0	0	0	0	0	0	0	0	0

97855d34357e113877045b0b726437b9	0	0	0	0	0	0	0	3	0	0	0	0
8b7c0ad1b8bad937387b04135e3c6ba9	0	0	0	0	0	0	0	0	0	0	6	0
7b490aa7f641b66f76611308a89c6458	0	0	0	0	0	0	0	0	0	3	0	0
bc1f9d78949be96570448df62e00c9b4	0	0	0	0	0	0	0	2	0	0	0	0
33b7a3d1aa4b73a4c374f90601b4005f	0	0	0	0	0	53	0	0	0	0	0	0
352eb10d470c5811bcbbc6b00bab2a41	0	0	0	0	0	0	0	0	3	0	0	0
0df5e851a285b02543cb3bede1fe3c52	0	0	0	0	0	2	0	0	0	0	0	0
bca767128c68eda1586fca7bab0c75c2	0	0	0	0	0	7	0	0	0	0	0	0
4108b922fdc779091447902c3611203e	0	0	0	5	0	0	0	0	0	0	0	0
787c58d1aa951329a4afe0f3137e2fbe	0	0	0	0	0	63	0	0	0	0	0	0
000bc9c8d2eb7c52a82b0fa5812b85a1	0	0	0	0	0	0	0	0	0	3	0	0
8508201d97e12d72fb2d386a300dd2bb	0	0	0	0	0	0	0	0	0	0	8	0
5963561c7085bbd70bd560116b517f59	0	0	0	0	0	0	104	0	0	0	0	0
e57015886aad50fc964e636c6582fabb	0	0	0	0	0	0	5	0	0	0	0	0

7cd2736c058850fbcf20cc7c92d0f86f	0	0	0	0	42	0	0	0	0	0	0	0
460de1e4a65d83e322b88edbfef993a9	0	0	0	0	0	0	0	0	0	0	2	0
1ce65f240951c041b3a5e1e9250be3ca	0	0	0	0	0	0	0	0	0	2	0	0
9d6532a92b9574675bf9805d390bd8aa	0	0	15	0	0	0	0	0	0	0	0	0
28a9bf62de48d1ff900453af25f1a78b	0	0	0	0	0	0	0	0	0	0	0	2
c0adf491211d5639e099a340de7f71f3	0	0	0	0	0	0	0	0	0	0	4	0
c8eda11e20ac964d904f117e769ee8b7	0	0	0	0	0	0	0	0	0	0	8	0
77f59eb5477d5e6eb119873bb01b08c0	0	0	0	0	0	0	0	0	0	37	0	0
8af083a4072b202284fe856218789e96	0	0	0	0	0	0	0	0	0	116	0	0
14bc63c8d576c836fe761f301dcf684f	0	0	0	0	0	12	0	0	0	0	0	0
6bc707a80fa10968795ebff3ebe0afa2	0	0	0	0	0	0	0	0	0	0	0	2
66eacfb4aa06d095bf5a53665fd48fd7	0	0	0	0	0	3	0	0	0	0	0	0
d60b19b22b897b5ffb631a078ce5d54c	0	0	0	0	0	0	0	0	2	0	0	0
a22e622babfff5fa41ad09e7d08169ea	0	0	0	86	0	0	92	0	0	0	0	0

3b0f7640ff13b6256e9c170889aa4e0a	0	9	0	0	0	0	0	0	0	0	0	0
154bfb71119ba9118af2af2c9e554319	0	0	0	0	0	0	0	0	0	0	5	0
b4ad17c21aa688a3ab611a93c662c149	0	0	0	0	0	0	0	6	0	0	0	0
fc88a482b426ae10f10f52e0d26b9942	0	0	0	0	0	0	0	0	0	0	3	0
58f4a4f3b2623b0234a2ed90be65c3a0	0	0	0	0	0	2	0	0	0	0	0	0
2aeab5922be3625fa15b15dd3ffe2625	4	0	0	0	0	0	0	0	0	0	0	0
51896f04af319c7316fb641408176c88	0	0	30	0	0	0	0	0	0	0	0	0
78d6f977fb53bea25004d684c9eaac39	0	0	8	0	0	0	0	0	0	0	0	0
f0b50c026e4330af19feec5ba09f852a	0	0	0	0	0	0	0	0	6	0	0	0
685ee15a5537029fa21d36aee4830ba3	0	0	0	0	0	0	0	0	0	0	0	5
eb45e8032ff6edd317da4e82264c84f0	2	0	0	0	0	0	0	0	0	0	0	0
d68eaad4e2f696086869be25422a0764	0	0	0	3	0	0	0	0	0	0	0	0
4bd7d14451061ad6fded732b1a488c31	0	0	0	0	0	0	0	0	0	0	21	0
92de3b9082083a9050f0a87196cea315	0	0	0	0	17	0	0	0	0	0	0	0

51c2b42384c9c86ff6074f204b2f294b	0	0	0	3	0	0	0	0	0	0	0	0
9b6bd89efd51b48a905187a845b0e6aa	0	0	0	3	0	0	0	0	0	0	0	0
1deb40c3f6de5cba2d07bd1d3cbeb9b1	0	0	0	0	0	0	0	2	0	0	0	0
3278b96a7fa48cdd361f81c26192cc7f	0	0	0	0	0	0	0	0	0	3	0	0
254eb096b0fda011d698b54bf9b3bb57	0	0	0	0	3	0	0	0	0	0	0	0
e7550c232785a351f9a7c5ecd0be2be2	0	0	0	0	0	0	0	0	0	0	10	0
e503d88a12a9d051fec0a9ef9ccddb14	0	0	0	0	3	0	0	0	0	0	0	0
49ed55dfe23138ba495265c75adc44fd	0	0	0	0	0	21	0	0	0	0	0	0
ac46e074f976686eef88cb98d227e328	0	0	0	0	3	0	0	0	0	0	0	0
f7248359a0e969b6135ba3aac65ab919	0	0	0	0	0	0	0	0	0	37	0	0
41a4c2e05d5846f62a327ded03c45298	0	0	0	0	0	0	0	0	0	0	2	0
876ff2557f7f67afef9b6a9f8b499c4a	0	0	0	0	0	0	0	2	0	0	0	0
d2f1f54e3fe858cb9793e81a00df733a	0	0	2	0	0	0	0	0	0	0	0	0
3991a1b75068e1104f0366c80ed339e4	8	0	0	0	0	0	0	0	0	0	0	0

f3e24bda2152fd8e191c6f11c067929c	0	0	0	0	0	0	0	0	0	0	3	0
05826de944c67115de308e8d26efdc17	0	0	0	0	0	0	0	0	0	0	0	10
e266a36ad4ab8f241057f9a56b655d6a	0	0	0	0	0	0	3	0	0	0	0	0
8b9ac58aa3a6fd49fd577f4cfe6a8594	0	0	0	0	0	0	0	0	0	0	17	0
0beed589b4ca3646eeb6c88edf1753ca	0	0	0	0	0	3	0	0	0	0	0	0
2c1a36289cf50e5fee20c4db87bda97f	0	0	0	0	0	0	0	14	0	0	0	0
7cf698fc1ccee72feee665df7110353f	0	0	0	0	0	0	0	0	0	0	9	0
a66f11adf2ee1ba30ee63d694c0b44db	0	0	0	0	0	0	0	2	0	0	0	0
737b81651da3f62730b54c976f812535	0	0	0	0	0	0	0	0	0	5	0	0
bcc99c661888b6335655eccc1b0f33a6	0	0	0	0	0	0	0	0	38	0	0	0
dfa0f5df1d0a8bbc7fa1bf45e2cd4478	0	0	0	0	9	0	0	0	0	0	0	0
ba9dc4c7cd96af85f44e5dfdc08bd58f	0	0	0	0	0	0	0	0	0	0	0	8
27bb4ce7c3258290527060cda44d41f7	0	0	0	0	0	0	0	0	0	0	5	0
bb5942c4161ff75568cc324216c153c5	0	0	0	0	0	0	0	0	0	5	0	0

e3ebc6fbcea6db160f938792679685d8	0	10	0	0	0	0	0	0	0	0	0	0
e3c5e8e003f766247cc6f3eac6da4cb9	4	0	0	0	0	0	0	0	0	0	0	0
60966efb557a16afa1795f7ee7793b21	0	2	0	0	0	0	0	0	0	0	0	0
8078dbdc9b370646daa8a97150fe2f33	0	0	0	0	0	7	0	0	0	0	0	0
b5744d7ddcd931223a603f10e2b3d21f	0	0	0	0	0	0	42	0	0	0	0	0
1db4322fa6f7a97bb78d3ccf70f5be6d	0	0	0	0	6	0	0	0	0	0	0	0
56ccd0f6217e5ce6c7dbc3d6984c21ab	0	0	0	0	0	0	0	0	0	0	2	0
7c52978dcc7492fa0687d97f0e4f03ac	0	0	0	0	0	0	0	6	0	0	0	0
c94b4a3361f8a999cd8f885ff8583c7d	0	0	16	0	0	0	0	0	0	0	0	0
d328a969c5f18bfc195a2a727f896976	0	0	0	0	0	12	0	0	0	0	0	0
8b3014e6e76e6cf2a8b649f7c14f1b3f	0	0	0	0	0	0	0	0	0	0	3	0
f8e7e23f8aa41f0f20ed3a5b7993065b	0	0	0	0	0	0	0	0	0	0	4	0
547d7d7433d9d54aa13764260227ab4b	0	0	0	0	0	0	0	0	0	0	0	4
c99e3528c7343fa59818aa1d9380c1ed	0	0	0	0	0	0	0	0	0	9	0	0

072911abcbd7b78a2a427e520e36ca28	0	0	17	0	0	0	0	0	0	0	0	0
3838342a0636ff034d0fbb4a3fc544c8	0	0	0	18	0	0	0	0	0	0	0	0
776e18ebb0b6cc5cfd707fe5ac60f34f	0	3	0	0	0	0	0	0	0	0	0	0
197b503fca6188d9f2f5c2a6a53327a2	0	0	0	0	0	0	0	0	0	0	0	23
73a91cc930419dccd3535e80c59f96ed	0	0	0	4	0	0	0	0	0	0	0	0
0b69c36c8ba0895ed5de5eadee09d4dd	0	0	0	0	0	0	3	0	0	0	0	0
6658580c596a245cda821f4ca8e68360	0	3	0	0	0	0	0	0	0	0	0	0
8a0fffd9963c2c1c69a98250dcb8ef56	21	0	0	0	0	0	0	0	0	0	0	0
ed6afdc071dad2e14c52174598dcf3cd	0	0	0	0	0	2	0	0	0	0	0	0
0148625f3761d5ca5f3432a9b83818ba	0	0	0	0	0	0	0	0	2	0	0	0
177b1872e5fd7567fac5d26c9b6cd13c	0	0	0	0	0	0	0	0	0	0	3	0
08532c1b206d6dc7688e363e630284bf	0	0	0	0	0	0	0	0	0	3	0	0
7621f77f0a6bbaee416bbf6bcdb7fec9	0	0	0	0	0	0	0	9	0	0	0	0
58df9ee73a541df8b9b145b3dbb94b12	0	0	5	0	0	0	0	0	0	0	0	0

6a8728f33879311dcbf2f39f60226002	0	0	0	0	0	0	0	5	0	0	0	0
17cea323454d9024725d3a07e20f525f	0	0	0	0	0	0	0	0	0	8	0	0
387e3aba7bd2c640d0bac56f80ea5670	0	0	0	0	0	0	0	0	0	0	2	0
5ff1ef4f828caa3b5d92d48c427fd7af	0	0	0	0	0	0	0	0	0	0	0	2
010508743e45f474f94023397c0da7c6	0	0	0	0	0	0	0	0	0	0	9	0
a8d60ce5cdc4d257586388d20e423661	0	0	0	0	0	0	0	0	6	0	0	0
767b807645435cd518febc420f8a5947	0	0	0	0	0	0	0	0	0	0	4	0
d8d9722996e89d83b199bbf40820b87b	0	0	0	0	0	0	0	0	2	0	0	0
dc71b69cd02b6d25a193b1ce83603972	2	0	0	0	0	0	0	0	0	0	0	0
1c0e470a1244da6311d7dba2771e36c7	0	0	0	0	0	0	0	0	0	48	0	0
97398c4d28798bd9246c73045c1b5f36	5	0	0	0	0	0	0	0	0	0	0	0
f980a2514c86fc0721f3ba3760748a69	0	0	0	0	0	0	0	0	4	0	0	0
b396e2ecd111301e9436e1275d910893	0	0	0	0	0	4	0	0	0	0	0	0
f7778d93788e03106d15956291e80347	0	0	0	0	0	0	0	0	3	0	0	0

ca5e4c2a467dfd19cfa5483e902c4e0c	0	0	0	0	0	0	0	0	2	0	0	0
7ff85bb519440a94569397eb19d69351	15	0	0	0	0	0	0	0	0	0	0	0
3032fdb5fd36eda0d66d7c65290def1b	0	0	0	0	0	0	0	3	0	0	0	0
b02ace255123cb5b470a20e319e766b6	0	0	0	0	0	0	0	0	0	0	2	0
089a148bae9eb70ffbe8b9288cb4137e	0	0	0	0	0	0	0	19	0	0	0	0
0b60674f998e5affc03219e83894be7c	0	0	0	0	0	0	0	0	0	0	2	0
31739f34dd0fb4b8099e746828993062	0	0	0	0	0	151	165	0	0	0	0	0
41f5cac709eab9cda0229a7df63db2ba	0	0	0	0	0	0	2	0	0	0	0	0
6542971f2e531727149b5a1f1b482fc0	0	0	0	14	0	0	0	0	0	0	0	0
a2778927922a7b586e02c582337f5c1c	0	0	0	0	0	0	0	0	0	0	26	0
3253cec863eba5db7d1f7de92c9db0c0	0	0	0	0	0	0	0	0	0	0	9	0
03c8c0d93585efe9c02f556d3ec42f0d	0	0	0	0	0	0	0	0	0	0	19	0
600ce973f4712d87af8c250bdf4fcbba	0	0	0	0	3	0	0	0	0	0	0	0
3307a5f05ab0ed16f0bcc003911fc700	0	0	0	0	3	0	0	0	0	0	0	0

d53903bf07750b849b9cafbaef43fd6a	0	0	0	0	0	0	0	0	5	0	0	0
b986ec8a831264636044b844fadc0dc3	0	2	0	0	0	0	0	0	0	0	0	0
a84711d432aba97a28aecb3ebd3aafa9	0	0	0	0	0	0	0	0	0	0	15	0
f7c7aa594a9370bb682aac8dc447789a	0	0	0	0	0	3	0	0	0	0	0	0
62feb47d846e03803cbb9c12bb8b2713	0	0	0	0	0	0	0	0	0	0	32	0
55ecd8808ac6d7df2a72f3493f8cf485	0	0	0	0	0	0	0	12	0	0	0	0
ac3d18427d277ef4d243a41c35ddebe1	0	0	0	0	0	0	0	0	0	43	0	0
677508c0873024dc3f1a2c71b6edf03f	0	0	0	0	0	0	0	2	0	0	0	0
7fcd6472ae977f6088eda37fd1e918f1	0	0	0	0	0	0	0	0	0	0	3	0
0dc3c9f4d6d9983a93fd1429d95667a8	0	0	0	0	0	0	2	0	0	0	0	0
ef13d0ed80d14c1d21a3f3760c203830	0	0	0	0	0	0	0	3	0	0	0	0
bf2550b30f6a59e458d961e093dbc28c	0	0	0	0	0	0	0	100	0	0	0	0
99d981d0666eff13bf8fefda7312ec62	0	0	3	0	0	0	0	0	0	0	0	0
9d9d38e19bb3980b082880680aa5797b	0	0	3	0	0	0	0	0	0	0	0	0

6f3e075af7c235baae0262c5acae08df	0	0	0	8	0	0	0	0	0	0	0	0
0a54dba1e24d6123ec91e6343109f260	0	0	0	0	0	0	0	5	0	0	0	0
f142db9c4f4ab46d0896319b29cb7fc4	3	0	0	0	0	0	0	0	0	0	0	0
d30d60d48b5fa65a672b233847ccacc7	0	3	0	0	0	0	0	0	0	0	0	0
547124bce2a7a6a32ba94c6bd080150a	0	0	0	0	0	0	0	0	0	0	0	2
76491ef48fed2161fbbc78591fde4d8e	0	0	0	0	0	17	0	0	0	0	0	0
67885d33e8c4c578898c1feb9b522d28	0	0	0	0	2	0	0	0	0	0	0	0
a5b7369e128dd340e9df2d2f5aa66312	0	49	0	0	0	0	0	0	0	0	0	0
b04f83c11ad588a8ec4e34129d3149bc	0	0	0	0	0	0	0	2	0	0	0	0
2e5fd5d13b07af830f50e7d8d96c5419	0	0	0	0	0	0	0	2	0	0	0	0
a1d2d8d840df33de20e197734c367bf8	0	0	5	0	0	0	0	0	0	0	0	0
53879dc76590b0c08b2afa2ef97f1e20	0	0	0	0	0	0	0	0	0	0	0	9
71e1a00fa03ce1319fa88795a0d3babd	0	0	5	0	0	0	0	0	0	0	0	0
51be032081d80cdbf42105bb68cd2f61	5	0	0	0	0	0	0	0	0	0	0	0

07edb300013438d584f75dd35431f9cc	0	6	0	0	0	0	0	0	0	0	0	0
4307773d9c5f6dabaf8602b4e75f6567	0	0	0	0	0	6	0	0	0	0	0	0
9b51967c6043e527f947e368f36be628	0	0	0	0	66	0	0	0	0	0	0	0
73420935f0e87794d9394759094ea515	0	0	0	0	0	0	0	0	0	0	0	2
b70961c4c495c72a73000cd95e6d9305	0	0	0	0	0	7	0	0	0	0	0	0
9e3fc1b839c7bd3a563a828d7254aa64	0	0	0	0	0	0	0	2	0	0	0	0
3975fdca9bca1ef4cef86f305b0249b9	0	0	0	0	0	0	0	0	0	0	0	8
41dd8a5f1c3ba30b5547863c347d39cf	0	0	0	0	0	0	0	0	0	0	0	21
58a54ae66cc73127611a2ea60ab110c9	0	0	0	0	0	0	22	0	0	0	0	0
9d73e1818f7523ce1f948b799291bed9	0	0	0	0	0	24	0	0	0	0	0	0
6c69d4f81e03326e96b8c52bb65db670	0	0	0	0	0	0	2	0	0	0	0	0
2a47f3913813964cbdb6ee9f5db9a88a	0	0	0	0	0	0	0	0	29	0	0	0
18429f7b69e0c6ddc3c3ef08438ba941	0	3	0	0	0	0	0	0	0	0	0	0
373eb99b9ee99ba1acccbd4ef71d4d6a	0	0	0	0	0	0	0	29	0	0	0	0

7468bf88d8645973d1f8cfab09cc0997	0	0	6	0	0	0	0	0	0	0	0	0
24c8beda5250414beb3b0741bd63ab82	0	0	0	0	0	0	0	2	0	0	0	0
daa031186c15f8aa7c95238bd7f4cca6	0	4	0	0	0	0	0	0	0	0	0	0
c36df2ef953486c4f33f88c8b7aad5e5	0	0	0	0	0	0	0	17	0	0	0	0
5819d1694f5f3ec1a8d38c6b5d99b73f	0	0	0	0	0	0	0	0	16	0	0	0
b89847de4cc7df99068c68198f89c425	0	0	0	0	0	0	0	0	0	0	18	0
7025c3848982646194e370b296c2972b	0	0	0	0	0	0	0	0	0	0	3	0
6153363ef8fc09affe3eebb0af9da407	0	0	0	0	0	0	0	0	0	0	3	0
ac180ecf7f57e3f861f7b8676a0864d1	0	0	0	0	0	0	0	0	211	0	0	0
ee1396f786bc29927dba66ccfe4db2e5	0	0	0	0	0	0	0	0	0	0	90	0
aeea4721fb92fa9056d098ddd81b3848	0	11	0	0	0	0	0	0	0	0	0	0
507f081b9339fa92f5853cbeaca5565f	0	0	0	0	0	15	0	0	0	0	0	0
7d2aa71161f5b73e372b6faa0bd4d644	0	0	0	0	0	0	3	0	0	0	0	0
eebd9696c164b45547729d843d98c86a	0	0	0	0	0	0	0	0	2	0	0	0

98ae3e20efec2a9f5035211e670041f1	0	0	0	0	0	0	0	0	0	0	3	0
c252933ef7b64c85c211781c5f8b54e9	0	0	0	0	0	0	0	0	0	0	0	4
f0026a0e0f0ba3949af089b05078a5e0	0	0	0	0	0	0	0	0	0	0	8	0
37f1f6b510dcb1f08014a07a08fd317b	0	0	0	0	0	0	0	0	3	0	0	0
7d81bd83cb51238498e4d2f4a243db7d	0	0	0	0	0	0	0	0	0	0	0	28
09617bed0884edfa4a60e74c200c481b	0	0	0	0	0	92	0	0	0	0	0	0
8bf84d87130e9d5d2866e935829f4907	0	0	0	0	0	0	0	0	0	7	0	0
f5782a65306fb4c99b14a6ab775a588d	0	0	0	0	0	0	0	0	0	31	0	0
9720719769cbb64ea48e6974bf9a20cf	0	0	0	0	17	0	0	0	0	0	0	0
c2f8b62cc610b3f1ee5ccf802caedc9b	0	0	0	0	0	0	0	66	0	0	103	0
c0f75925609f19663d902e3355b052a7	0	0	0	0	0	0	0	0	0	0	0	8
295be3d13e6f4494f17778451035131a	0	0	0	0	0	0	0	0	0	7	0	0
702a74894b294143b3778311a742ef1b	0	0	0	0	0	13	0	0	0	0	0	0
2965280828143ade7898bad1608e029d	0	0	0	0	0	0	0	0	0	0	0	2

2defad8078860dd9a796518c3bb855dd	0	0	0	0	0	0	0	0	0	8	0	0
45c3a23e87e3b277f549de81a7d48e49	0	0	0	0	0	0	0	4	0	0	0	0
2919caf04514bcad619418e15d2246d8	0	3	0	0	0	0	0	0	0	0	0	0
3ce5c8bfdd9acebdade48f4fd165ce8a	0	0	59	0	0	0	0	0	0	0	0	0
06ed0e8792195a10ef20dc2a3bd243c3	0	0	0	0	0	0	6	0	0	0	0	0
d42d6f1b282224e378f3adbc26516028	0	0	0	0	0	0	4	0	0	0	0	0
a2de4a585889c580572f91fea7387da8	0	0	0	0	0	0	0	0	0	0	0	25
e950499e71effb99509a09daf57e9ee0	0	0	0	6	0	0	0	0	0	0	0	0
68de09e6a7f541b5223a67f78d70d981	0	0	0	0	0	0	0	5	0	0	0	0
f9d94dd1c588e5eb542a5a01af585ce4	0	0	0	0	0	0	0	0	0	0	0	7
0c9b22ff82b39f5ea889f37cdd00d984	0	0	0	0	0	0	0	0	0	0	6	0
b65025c4579d733e03a39c1af9753619	0	0	0	0	0	0	0	6	0	0	0	0
a1fbe55513d29525074bd0c82b6ddea4	0	0	0	0	0	0	0	0	0	18	0	0
53d85945a7879404febc385251a3dfde	0	0	0	0	0	2	0	0	0	0	0	0

665876f947e34c5dce299d49 02e42b29	0	0	0	0	0	0	0	0	0	28	0	0
7482549c44135a6bcde1e71f a7507e3c	0	0	0	0	0	0	0	0	0	0	0	15
c54ea4c00bbf4e9fe990d973 5c5a54bd	0	0	0	12	0	0	0	0	0	0	0	0
091729c76ba46df9d960fc86f 5d78cd6	0	13	0	0	0	0	0	0	0	0	0	0
1c1837036aecb56a4d790449 e58e1c2a	0	0	0	0	0	2	0	0	0	0	0	0
75b5e19dab47b775126e27a 167473494	0	0	2	0	0	0	0	0	0	0	0	0
d96cabe5dfa86da8525bd42e ef56f3e4	0	0	0	3	0	0	0	0	0	0	0	0
47941e5f8b65537aa781bc9b 9bfb05bd	0	0	0	0	0	0	0	0	0	0	6	0
053ed86703aecf0cba8f94d4 9e2a3fae	0	0	0	0	0	0	0	0	0	389	0	0
ce357ebbae46d28436ef7f58 48d55b9d	0	0	0	0	0	5	0	0	0	0	0	0
fe2f3a6723172e5bc45e681d 06d11c20	0	0	0	0	0	0	0	0	0	0	8	0
c831d7e10d0995759153053 947934599	0	0	0	0	0	0	0	15	0	0	0	0
81c3536557e063f8632d2b85 fdd7b099	0	0	0	0	100	0	0	0	0	0	0	0
8afca05f295f3e725ce1e86f6 bb7cb93	0	0	0	0	0	0	0	0	6	0	0	0

f7be752a52a1ac793eedc5ae a5ee052e	0	0	0	0	0	37	0	0	0	0	0	0
97c27bf9530f90e0f726ebc2b c961896	0	0	0	0	0	0	0	16	0	0	0	0
f4a124c7f47b4a98d8fea4461 516fa14	0	0	0	0	0	0	0	0	0	178	0	0
62ffc69604ea5e7d7751c6f24 b22add7	0	0	0	0	0	0	0	0	2	0	0	0
93d74e63bfb137cec756e092 a0e0e573	0	0	0	0	0	0	5	0	0	0	0	0
fb6a4c60ac60dc61e7846cc4 d9ef8398	0	0	0	0	0	0	0	0	0	4	0	0
bcc7f66559685e363af8686b 5a5ec65c	0	0	0	0	0	0	59	0	0	0	0	0
c551fd7f354404da7f32c1cf4 c8b3fa2	0	0	0	0	0	0	0	3	0	0	0	0
6bc853896c34b8e765ec2f28 c3dbb98e	0	0	0	0	0	0	0	0	2	0	0	0
f797f76e7a0d5ecfe59063435 085a833	0	0	0	2	0	0	0	0	0	0	0	0
6b0a62f00651d90a153e10e9 4d8c84d9	0	0	0	0	0	0	0	0	2	0	0	0
3c4d0e0d66d32f6d54960fa6 a3581416	0	0	0	0	0	22	0	0	0	0	0	0
fb8ba2e991658746f7d551d1 4fb2f344	0	0	3	0	0	0	0	0	0	0	0	0
d45829e9ec286c0b5f299dd6 8c6bd205	0	0	0	0	0	0	0	0	0	0	0	2

ca0ccc08dbfdc551dcb222cd375e50a1	0	0	0	0	0	0	0	0	0	0	57	0
4672867bbc688c95ed4eb5dfb035fac3	0	0	0	0	0	0	0	0	0	0	0	14
eadf321e59e027c232828a2e29246e79	0	0	0	0	0	0	0	3	0	0	0	0
f3c5b7a10f468b414c1f3706a79a47c6	0	0	0	0	0	0	0	0	0	6	0	0
afa7b0407e62f12693a55123bf1328de	0	0	0	0	11	0	0	0	0	0	0	0
b440b3f1283ca14f2f57a38c791b734f	0	0	0	0	0	3	0	0	0	0	0	0
1fd254cf6ee4cbe239737e12f2c5b725	0	0	2	0	0	0	0	0	0	0	0	0
0f4b7e26fb6f3299d7d2c600e74ff92f	0	0	0	0	0	0	0	0	0	30	0	0
9675347cfd73b943d82c7d60a2a7d165	0	0	0	0	0	0	13	0	0	0	0	0
4523e78bef471fd9ca5afef6e22b40fa	0	0	0	0	0	0	0	0	0	0	3	0
b02849ed67863088381c3320630f4e51	0	0	0	0	0	0	0	0	0	0	20	0
58992bcfd83b0006f1e5824cc4c1a1e4	0	0	0	0	0	10	0	0	0	0	0	0
f2ba7f42e672085a8a98cf0646d978a9	0	0	0	0	0	0	0	0	0	0	0	9
cfffabc6e301821f439a29ac048c2d6c	0	0	0	0	0	0	29	0	0	0	0	0

07ff7b4e13be3a2ca2165243119abf19	0	0	0	0	0	0	0	0	0	0	0	2
3d22159d4a9dd6f03cef44e56f625e1d	0	0	0	0	0	0	0	0	0	2	0	0
05d7ebc780986f668270ab00175063b2	0	0	0	0	0	0	0	0	0	4	0	0
6328ff451b8cebed56a89349fc12fe20	0	0	0	0	0	0	0	0	6	0	0	0
de12513199f822f712e8f3af24f91992	0	0	0	0	0	0	0	7	0	0	0	0
148126cba1a103b6f9f39a2fe9a20768	5	0	0	0	0	0	0	0	0	0	0	0
b6655b8e79a0773a8cdf154b8c900f14	0	0	0	0	0	0	0	67	0	0	0	0
65cf9299b397d0ed18b232271558cb13	0	0	2	0	0	0	0	0	0	0	0	0
ef162b2826275ee1f7d862fbca358767	0	0	0	2	0	0	0	0	0	0	0	0
f9a801ab4a0ef6edaab0c41b7521d07b	0	0	0	0	0	0	0	0	24	0	0	0
9ba2a67a7f2347a6da0721e916868470	0	0	0	0	0	0	0	11	0	0	0	0
ac7a7dddfefc293cefc2cfa899f53b4a	0	0	0	0	0	0	0	0	0	0	2	0
3a3048aa6e00cd73881258ab521db2df	0	0	0	0	0	0	0	0	0	0	4	0
5178b809f4a497c94c038b0d1f047739	0	0	0	0	0	0	0	0	0	0	0	68

bea29892093360f519a71ef7e89b6175	0	0	0	0	0	0	0	0	0	0	6	0
4714808164ddcf1228063ed441696c90	0	17	0	0	0	0	0	0	0	0	0	0
81bfcbdcf8e912b1cef1f88d074d7a40	0	0	0	0	0	0	0	6	0	0	0	0
c9504291de66dcf3446fadd7a2cca21b	0	0	0	0	0	26	0	0	0	0	0	0
93bd005e4caeb770f0f26dec2b886a1c	0	0	0	0	0	0	0	0	0	0	0	6
bf4abf466f5ab6c140fb4bd0f90ff7c9	0	0	2	0	0	0	0	0	0	0	0	0
362eaf555351a9ccaa96a15b5996e507	0	0	0	2	0	0	0	0	0	0	0	0
6a6c4aefbd20dd85e7cb4e642b9603a5	0	0	0	0	0	0	0	2	0	0	0	0
4039174fc006b48586e698935dcd7fb4	0	0	0	0	0	0	0	0	0	8	0	0
ae5e7774b2717dbbec68c69eeed9711d	0	0	0	0	0	0	0	0	4	0	0	0
fbe769df9fa3e118c2cc1b43dea7ce28	0	0	0	0	0	0	0	0	0	0	0	12
44f7ad7741e57f9625331e0549a06418	0	0	0	0	0	2	0	0	0	0	0	0
9f64c1204c7a2a7064222d4ad3596911	0	0	0	0	0	3	0	0	0	0	0	0
3bd90fec7133a87cf27505fdeaf2a0fb	0	0	0	5	0	0	0	0	0	0	0	0

eac57771f7c5bd10ed95706a405855c8	0	0	0	0	3	0	0	0	0	0	0	0
da69003e4f4edb7a2eecbf694e45cdf1	0	0	0	0	0	0	0	0	19	0	0	0
8567498156bf871092b941676fa73172	0	0	0	0	0	2	0	0	0	0	0	0
fa7c2d6b8250200c885de37790fbb3fa	0	0	0	0	0	0	2	0	0	0	0	0
e7bc2bc1fee652da4111483d5853c617	0	0	0	0	0	0	0	0	0	0	5	0
8c27645b87cad211e3d1641ff2618229	0	0	0	0	0	0	0	43	0	0	0	0
8061967d075f89ec5f108212ba963fbb	0	0	0	0	0	0	0	0	0	0	0	2
807dc87162eec2af3ca4dac6347909dd	0	0	6	0	0	0	0	0	0	0	0	0
4ace44db61478950ad5a4e5049936e35	0	0	0	0	0	2	0	0	0	0	0	0
4ddf4fe99e6fa838ed5a2ede0ad77bca	0	0	0	0	0	0	6	0	0	0	0	0
baf62609b9dfd1912fff0740fe50672a	0	0	0	0	0	0	0	4	0	0	0	0
3462831b5ce5b984625f036f02ee4c7b	0	0	2	0	0	0	0	0	0	0	0	0
43e7a728ca1dfa76db2d3593f25c8936	0	0	0	0	0	0	0	0	0	3	0	0
46fb13c6624fa4492dd0b00e22e5f268	0	0	0	0	0	0	0	0	0	0	4	0

41c5902d33d15c7110e94db b6c3f3424	0	0	0	0	0	0	3	0	0	0	0	0
ae2c38fb55d17cb0b948639c 1eb3ec3e	0	0	0	4	0	0	0	0	0	0	0	0
27160f18ef4029472a12a820 b971ef51	0	0	0	0	153	0	0	0	0	0	0	0
6e210378708ad0f16289d7da 20704c59	0	0	0	0	0	3	0	0	0	0	0	0
52a9a1ce4fa220361b8521f3 a4dc33e1	0	0	100	0	0	0	0	0	0	0	0	0
002c704dfebfba3632e19aa0 694d87b2	0	0	0	0	0	0	0	0	0	0	3	0
db40bd36ad313ce13cf80ac1 1a46f677	0	0	0	0	0	0	0	0	0	0	20	0
4822aef76fe98c453a386fb29 36f9524	0	0	0	0	0	0	0	0	0	22	0	0
a81f61df830560bda181c578 356f07d2	0	0	0	0	3	0	0	0	0	0	0	0
cb4b9498beea43ba5c6dfe75 b9c8de10	0	15	0	0	0	0	0	0	0	0	0	0
d3408e1e2fb7834111b008b 7c51834b0	0	0	0	0	0	0	0	0	0	0	0	142
553ef0db415099257f38ad92 3fe11edb	0	0	0	0	0	0	0	3	0	0	0	0
2397cd504538355758d5a1a af4db2ea5	0	0	0	13	0	0	0	0	0	0	0	0
c84dae0803a1829a989f09f9 de074c16	0	0	0	0	0	20	0	0	0	0	0	0

080c6f5ab8cf7fbb72edde87d1b90e24	0	0	0	0	0	0	0	0	3	0	0	0
4b8cadb78cecac1d7cd391265023d2ff	0	0	0	0	0	30	0	0	0	0	0	0
afb4ea1f9dca699a80e74fa8efd4a2cd	0	0	0	0	0	0	0	0	0	33	0	0
8d1e5e85fde1255e9b383910b32f78ac	0	0	0	0	0	0	0	0	0	37	0	0
fc51c461943ea8d06db91e0db7353d58	2	0	0	0	0	0	0	0	0	0	0	0
701c61895b3e3fe4e59574a2f16f3332	8	0	0	0	0	0	0	0	0	0	0	0
c7fbcba580d1c9ad368178305a93294a	0	0	0	0	151	0	0	0	0	0	0	0
f3bf5bc94b7bd875eb7a5d998a6ef454	0	0	0	0	0	0	0	0	0	0	0	3
e17951a0ba2617c28eb5bce643025d29	0	0	0	0	0	0	0	0	0	0	11	0
59506d85eab4fabe13340f182dcdc9d9	0	0	0	0	0	0	0	0	0	0	0	9
e08c9da3019b309626fc5b58c0ab9d1a	0	0	0	0	0	0	0	0	0	0	24	0
938900266673651fedd33b19c1f8e9f2	0	0	0	0	0	0	0	0	0	18	0	0
79b638505b1ab47c8f0f2d5c2b31cda6	0	0	52	0	0	0	0	0	0	0	0	0
7d9719f81aa33a45d9342b8a00eb8f01	0	0	0	0	0	0	0	0	0	2	0	0

993fa9186b31d0262e024a14fcf4db4d	0	0	0	0	0	0	0	0	0	0	76	0
4beae5a4d3fda3e9f5cdd584ae9450d3	0	0	0	0	0	11	0	0	0	0	0	0
5a16058829963f294f4e421cfc30835d	0	0	3	0	0	0	0	0	0	0	0	0
ade19c2d61aaa679ebf3c5b9086c549e	7	0	0	0	0	0	0	0	0	0	0	0
6b5e1014f752df380ec6237c7948ac3c	0	0	0	0	0	0	0	2	0	0	0	0
8d16d2cb22b4677c5787019dff2a3e5e	0	0	0	0	0	0	0	0	5	0	0	0
fa05796f3b1fcc8cce4e599e956285f9	0	0	0	0	0	0	0	0	0	0	10	0
11950df4bd53efd6148e6625f43a9141	0	0	0	0	0	0	10	0	0	0	0	0
dedea243ba1825b9baf3276e04babebd	0	0	0	0	0	0	0	0	0	0	5	0
0ed8089f3f547128211aee566fc6f9f3	0	0	5	0	0	0	0	0	0	0	0	0
83b85ee9e5627b28c58934c0f1434bbe	0	0	0	0	0	0	0	0	0	0	0	2
a97dd18cd93285f3ebe6db84ae102376	0	0	0	0	0	0	0	3	0	0	0	0
da778598fb7c4daeb24588ccad05cfb8	0	0	0	0	0	0	3	0	0	0	0	0
ce9fc0be556124b7e996d831b1c77d8b	0	0	0	0	0	0	0	0	0	0	2	0

1474deaa34e65540387035cbcd156af3	0	0	0	0	0	0	10	0	0	0	0	0
8bbbefca267cc3c561d921ba8d5e5c71	0	0	0	0	0	0	0	0	0	0	6	0
ab4b1c7832354da394298b717073ccb0	0	0	0	0	0	0	0	0	2	0	0	0
4a20fe05f10a038da73dd3665f701e9b	0	0	0	0	0	0	0	0	0	0	3	0
613dd67b0caf6fbcd5354f52a92a9dfd	0	0	0	9	0	0	0	0	0	0	0	0
d5e37a3eb47fcc62c81bbe5bd6a4dd5c	0	0	0	0	0	0	10	0	0	0	0	0
d275b708a9b3a33e47d69b5c1d1dc446	0	0	0	0	5	0	0	0	0	0	0	0
3eba42602cb384992e2e8e48b309f66d	0	0	0	0	0	0	0	0	0	0	0	3
89eda9fd7caec04145ff5bb78391de26	0	0	0	0	0	0	4	0	0	0	0	0
139d03173231983226351a2af3ed66c7	0	0	0	0	0	2	0	0	0	0	0	0
03bc01551a82f0dca8b4d6a010a3caa6	0	0	0	0	0	0	0	0	0	0	8	0
5c2d92b7de484add077ddaf18d7468ad	0	0	0	0	0	0	0	47	0	0	0	0
9ccd39f82242e51a395b7e360b7e12fe	0	0	0	47	0	0	0	0	0	0	0	0
df6798eabffd92bd8578f97aced74921	0	0	0	0	0	0	0	0	0	0	11	0

618b7f8e06842b887da242ec69016c69	0	0	0	0	0	0	0	46	0	0	0	0
91033c03ea959951f0f672b1a8be6b1c	0	0	0	0	0	0	0	0	0	0	0	2
3c047623f7e3aa9f37343a3a0f72b7a3	0	0	0	0	0	0	0	0	0	0	0	7
590d970f80d6e0bfba16d650a4a68454	0	0	4	0	0	0	0	0	0	0	0	0
964702704d9876d6fe0cca15664126f3	0	0	3	0	0	0	0	0	0	0	0	0
80e7ceab1625aea80dc7e1959f284b5a	0	0	0	0	0	44	0	0	0	0	0	0
4a3e28424375ca89e9d43dbe47c111a0	0	0	0	2	0	0	0	0	0	0	0	0
301b79e2c0af11e673569f910f9ff4e9	0	0	0	0	0	0	0	0	28	0	0	0
729acd646dded39f7e57a99088d32b08	0	0	0	0	0	0	4	0	0	0	0	0
915b045955420f5148220dc4398d5667	0	0	0	0	0	0	4	0	0	0	0	0
e855de9ac2dcff92a2286d23b425b3a2	0	0	0	0	0	32	0	0	0	0	0	0
a9f2955cfb73be2742e8d6fb11275ddc	0	0	0	0	0	0	0	0	0	0	3	0
3a4d0214c6538797b998c9e7ccd32a78	0	0	0	0	0	0	0	0	0	7	0	0
ecc5dd2265b5709c9534c196084bcf70	0	0	0	0	0	0	0	6	0	0	0	0

b6c7e4b690ee6f9cc61350572c8edfcf	0	0	0	0	0	0	0	0	3	0	0	0
58d862a4e1a154cf17fe5423156d5d6d	0	0	0	0	0	0	0	0	0	33	0	0
48a199a2fe773d31b788e12b0e0a88de	0	0	0	0	0	152	0	0	0	0	0	0
3317a377a21e138fee13273d3b928694	0	0	4	0	0	0	0	0	0	0	0	0
4a8a52e513f5cc4d371ffd9fcf57be1c	0	0	0	0	0	0	0	0	0	5	0	0
ca05eb9bbd88054094e34ce1753fe7bc	0	0	0	0	0	0	0	0	0	0	4	0
155c1e66a75c2b51030346b314fee74e	0	0	0	0	3	0	0	0	0	0	0	0
394d822ce466bbb2bc2959ef1a863fed	0	0	0	0	21	0	0	0	0	0	0	0
3a3e487d29164a2b7494a2eb6f6620b4	0	0	0	0	0	0	0	0	0	2	0	0
500d849941c04049fac46327381c75d3	0	0	0	0	0	0	0	0	0	7	0	0
0a99debc5e225d592003c6c3429aec73	0	0	0	0	0	0	0	0	3	0	0	0
1e21281418af917a1a3f05411f7b9794	0	0	3	0	0	0	0	0	0	0	0	0
b148cf3bc022e042c99145f3f4cb94dd	0	0	0	0	0	47	0	0	0	0	0	0
111251f67856fd19d40588f8f971a278	0	0	0	0	0	4	0	0	0	0	0	0

8148d9e1373cf70313c29260 21e9afd0	0	0	0	0	2	0	0	0	0	0	0	0
769cae5c7aca3050b4c1f60f5 66e2aae	2	0	0	0	0	0	0	0	0	0	0	0
7f4e096446018892e9e09f3c 21ae8a3d	0	0	0	0	0	0	0	8	0	0	0	0
e2ee1e7b6fbbe8fc9d2c868c eab69273	0	0	2	0	0	0	0	0	0	0	0	0
33dd3d74a9ff0b33dd46530c a06e38aa	0	13	0	0	0	0	0	0	0	0	0	0
b5200e69e8b474ec50c2f96b 88401b68	0	0	93	0	0	0	0	0	0	0	0	0
434d626ee18b9b99766f24d 3eaa56911	0	8	0	0	0	0	0	0	0	0	0	0
6bc69e33432a99ac600dd4b e1736241d	4	0	0	0	0	0	0	0	0	0	0	0
19b50ccb82cad459f1191104 5a6eb7d0	0	0	7	0	0	0	0	0	0	0	0	0
655001624c29da44c2c6bd33 7845888c	0	0	0	0	0	0	0	0	21	0	0	0
cc183030c1f5668724aec521f 2b22065	0	0	0	4	0	0	0	0	0	0	0	0
583ae44220cfc449365ca363 35e9cb92	0	0	0	0	3	0	0	0	0	0	0	0
e1bf1b046713b7f7aabbb57d 76bed7d7	0	0	0	0	0	0	0	0	0	0	0	10
7a47d23624613099621e4c0 9772a7338	0	0	0	0	0	6	0	0	0	0	0	0

6511df6aee8d5c16014932d8dbf6f5fa	0	0	0	0	0	0	0	0	0	18	0	0
8dc0f9853d4be019776509d0c9d2fab1	0	0	0	0	0	0	0	2	0	0	0	0
995b5e98fe226dd2619917fcb79b64b1	0	0	0	0	0	0	0	0	4	0	0	0
4cd1f51c71243ff22c7bda2c5e7bfcfd	0	0	0	0	0	0	0	0	0	0	7	0
00f075ea67a2675b6e5a9c978dde384c	0	0	0	0	0	0	0	0	0	0	13	0
ee246593457fd3cdf50ad0eba1ba04a8	0	0	0	0	0	0	0	0	0	0	11	0
fae952243a5909fe6525a54347da192e	0	0	0	0	0	0	0	0	0	0	0	8
df839753ba535d3901019a6dc5be3b36	0	0	0	0	0	0	0	0	0	2	0	0
e656542128c8a8b7c3d288a16f25f276	0	0	0	0	0	0	2	0	0	0	0	0
06987fa4c1a8c31ade04b30a455b7fcb	0	0	0	0	0	0	2	0	0	0	0	0
bf0c5cddeeffe0b8955789e03436cf5b	0	0	0	0	0	0	0	0	0	0	0	5
fb08926c8d71ea83a9cd12c301b789f0	0	0	0	0	0	0	0	0	0	5	0	0
2be437335e946212a8a8ca4d11dd9958	0	0	0	0	0	0	0	0	0	0	2	0
80277ed36f4984891f0962f6ff2488e6	0	0	0	0	3	0	0	0	0	0	0	0

82a806bcd919adda12698d5f60024fc2	0	0	3	0	0	0	0	0	0	0	0	0
8466aecd1779dcdb722ea2cd7865fae8	0	0	0	0	0	0	0	0	9	0	0	0
95847168ca2274f2d803eb8dfc647419	0	0	0	0	4	0	0	0	0	0	0	0
5b2437f5785e5da18a434bbeec00b9f8	0	0	0	0	0	0	0	0	0	0	0	5
5c60e48df19c9adb36ce57d98a70d948	0	0	0	0	0	0	0	0	0	0	9	0
52506866fbd05e895f5ecd1f585f2077	0	0	0	0	0	0	0	0	0	0	0	4
1ba205149bbd9b9d6967f9543bb8614d	0	0	0	0	0	0	0	10	0	0	0	0
9a59746e985e729c2e018132ac0ae634	133	0	0	0	209	0	0	0	0	0	0	0
661e8887a6dab59d733423286123363d	0	0	0	8	0	0	0	0	0	0	0	0
d346d96b4f37d20a113e062ebb1c0725	0	0	0	0	0	0	0	0	0	0	0	7
8629fba8118f7f4f84522549af8cb9ea	0	0	0	0	0	0	0	3	0	0	0	0
d3b08df1fc02b7ba0dafbff62ff3c34e	0	0	0	0	0	0	0	0	0	0	3	0
1ef4fb7abdc4a2fa9b2c7b0c8782a9ae	0	10	0	0	0	0	0	0	0	0	0	0
c5820425ab414230481f8cd53436fbcf	0	0	0	0	0	2	0	0	0	0	0	0

aa93e2bef1c792ced610d0d840e60c80	0	0	0	0	0	0	0	0	0	0	0	4
c505dcf159d754a35df25c092841597d	0	0	0	0	0	0	22	0	0	0	0	0
a81096b68cad0347b55ff4f11d44e54e	0	0	0	0	0	0	0	0	0	0	31	0
374af3b7a4acc77bc5dea4f566a7085b	0	0	0	0	0	0	0	0	0	6	0	0
582b50d77772d6d0c65b7a1d687474fe	0	0	0	0	0	0	0	0	0	0	0	60
56fc1b740f5b74cc237c2b60f8db4a18	17	0	0	0	0	0	0	0	0	0	0	0
034bb15c1734261217372c168f62daa9	0	7	0	0	0	0	0	0	0	0	0	0
e99236fc29123edb8f1142b4b2d0f8b9	0	0	0	0	0	0	0	0	0	0	2	0
080fb372d031766d69ead7f8abda4b67	0	0	0	0	0	0	0	0	0	12	0	0
d3ce2caa8413adb4afdc7d696291c5d1	0	0	0	0	0	0	0	15	0	0	0	0
843ad02b69dc892a1988c4b77a15b762	0	0	0	0	0	0	0	0	0	0	0	10
e90373f394b58e262e8bd144c58ec0fa	0	0	2	0	0	0	0	0	0	0	0	0
7657d2a76b8b1c6a5c42f32de0053ea4	0	0	0	0	0	0	0	0	0	0	51	0
aaf4b188d3d8ac55dc293589bf54f9de	0	0	0	0	0	0	0	0	0	4	0	0

050091ad9e0a3bb022a1a34dc6412945	0	0	0	8	0	0	0	0	0	0	0	0
8f55f9a41afd87d6e2ed0d37f04f94f2	0	0	0	0	0	0	0	0	2	0	0	0
2cec3f343d791869b2174acf4292d045	0	0	0	0	0	0	0	0	4	0	0	0
62d2a702fdbbe55b7da05e28b77a8ab4	0	0	0	0	0	0	0	0	5	0	0	0
00be3080e587b8ac6dfe5e46cb63c9cb	0	0	0	0	0	0	0	0	0	0	2	0
4a3d6b4bcdbfe80875361f0192a323ee	0	0	0	0	0	0	0	0	0	0	6	0
7be9412a7644522e8fe07cb2dfe3b189	0	0	0	0	0	0	0	0	0	0	0	5
379bbf7938575d7893c8c8a6450d1837	0	0	0	0	49	0	0	0	0	0	0	0
84f646860f88e998a4b4a853d239999d	0	0	0	0	0	0	0	0	0	2	0	0
e4574b155337206b665ee79b289f3128	0	27	0	0	0	0	0	0	0	0	0	0
b7232faefd5e5b637377dcef4a052424	0	27	0	0	0	0	0	0	0	0	0	0
450063a9e0f10bf86cdf5f29011c8d1d	0	0	0	0	0	0	0	0	0	8	0	0
d50460c60f25136ba34887c3ed2fc6c8	0	0	0	0	0	0	0	0	0	0	3	0
a46df8ce1630c93eb3d85a87165966c3	0	0	0	0	0	0	0	0	0	0	7	0

83bfd7350c4a5d4f7275919e8ff2b876	0	0	0	0	0	0	0	0	0	12	0	0
3898291abe454a1878ab423050431ef8	0	0	0	10	0	0	0	0	0	0	0	0
9848f17a3b917f513612d8b8a539c40b	0	0	0	0	0	0	10	0	0	0	0	0
e81df451580adaf726585ef9487b4792	8	0	0	0	0	0	0	0	0	0	0	0
b212b5bf5886cb98ef30fab2ed9eb1f4	0	0	0	6	0	0	0	0	0	0	0	0
c1c21a2d09e04242b6fbc82a11a1fe10	0	0	316	0	0	0	0	0	0	0	0	0
4cd5391b605a844ea3688275549825e6	0	0	0	0	0	0	5	0	0	0	0	0
950dcf1ee3de4807ecf9b5e8c565acdb	0	0	0	0	0	0	0	25	0	0	0	0
76c81b721296c39f6d3a2ab595b5d479	0	0	2	0	0	0	0	0	0	0	0	0
17d8a21abe690061d763bdbcd15d4ed3	15	0	0	0	0	0	0	0	0	0	0	0
dc55a4d4c93a4de86356a97e39c651fe	0	0	0	0	0	0	0	0	0	2	0	0
c83f4a9d141ef8851b17b298062b17eb	0	0	0	0	0	0	0	2	0	0	0	0
01375e89cf3da03ae23513d4eef7a9df	0	0	0	0	5	0	0	0	0	0	0	0
eb75a6351cf925ed9dd03d43272c9dd4	0	0	0	0	0	0	0	0	0	0	0	7

aacff49d27ea209f3eb3257af15eca01	0	0	0	0	10	0	0	0	0	0	0	0
655630f843dfb0a1a0f1e7316cf9a80c	0	0	0	0	0	0	3	0	0	0	0	0
24ae820579766b9728198de843c1c778	0	0	0	0	0	0	0	0	0	0	2	0
4d8a9a7e70638a2d12fbf18de8f71a84	0	0	0	0	0	0	0	0	3	0	0	0
b1cf39282bd8f090501154e5fa7dd20d	0	0	0	0	0	0	0	0	3	0	0	0
9317b1443c769143e67f8343fe0c8baa	0	0	55	0	0	0	0	0	0	0	0	0
d15188525f34ee0ca49ca1f77a49ca9d	0	0	0	0	0	0	0	0	0	0	0	22
c61e3901d2e0c661be36c6f640b96f61	0	0	0	0	0	0	0	0	0	0	4	0
9c70438c3068a207ab37bccdb67ecc4b	0	0	0	0	0	0	0	0	0	0	5	0
7a61da829d261dc07529b2aed6af43ab	0	0	0	0	0	0	0	0	0	0	2	0
3c5f9a1c136afb51a2ded77d19db60b8	0	0	0	0	0	0	0	21	0	0	0	0
c58696fcd21f815f9347e9c016e28af2	0	0	0	0	0	8	0	0	0	0	0	0
3d3bb0c456102fdbd08f6769935a4935	0	18	0	0	0	0	0	0	0	0	0	0
5822d4f5d6adb5b75a767e313e0b1213	0	0	0	0	0	0	9	0	0	0	0	0

a6686c30454c20ae45c3bc604432a0f6	0	0	0	0	0	34	0	0	0	0	0	0
ea6afa40fb0caeeb70c09f5b84839a92	0	0	0	3	0	0	0	0	0	0	0	0
fcc84de880f90ba34ec1297b927d834c	0	0	0	0	0	0	0	0	0	0	22	0
be17a6e64362d7e0db7ab9dcd2125f68	0	0	0	0	0	0	0	0	27	0	0	0
eaa7d1a6207abad90a8bb8b8fe23f7d1	0	7	0	0	0	0	0	0	0	0	0	0
4e98e7e58ef31aaca8905ba3230c3cc6	0	0	0	0	0	0	0	3	0	0	0	0
fdc77eb0ef8a24a9602a6111e26f320c	0	0	0	5	0	0	0	0	0	0	0	0
35496f99ee17c15de2732e97d9384bd8	0	0	0	0	5	0	0	0	0	0	0	0
6654235f4c21a944520a864efb895308	0	0	0	0	0	18	0	0	0	0	0	0
903ea3b2269a3d8edd6f62a0d447fcbe	0	0	0	0	0	0	0	0	0	0	0	45
447f52cfe49536fdce6d78b316168461	0	0	0	0	0	0	6	0	0	0	0	0
3a78ba7e633d0fa50941cc07af6345fb	0	0	0	0	0	0	0	15	0	0	0	0
6e4e7f5fbb5c6ec95daf61f5f3479113	0	0	3	0	0	0	0	0	0	0	0	0
842fbc9f68c1624d7bafc76f7d2667d5	0	0	0	0	0	0	0	0	5	0	0	0

9caf365db9b457a095302be95700bfd5	0	0	0	7	0	0	0	0	0	0	0	0
3039376a767573fd5a0b37caa4fe57c8	0	0	0	0	0	0	0	0	0	0	26	0
85070aa41e7e856bcc1f9a2abffedd77	0	0	0	4	0	0	0	0	0	0	0	0
705ea4593b61091a13ccbcd07a63e5ce	0	0	0	0	0	0	0	0	0	0	0	7
43247459cf09eadb79378ea1f38fdabe	0	0	0	16	0	0	0	0	0	0	0	0
17e1e98735ba733f5f9c1f2c9d41f71d	0	0	8	0	0	0	0	0	0	0	0	0
7ec7b7270f67b0db501967f4299d2035	0	0	53	0	0	0	0	0	0	0	0	0
c123bab7f4898b3329a55c5704b7989d	4	0	0	0	0	0	0	0	0	0	0	0
c3ca6dbbb1dd7451fd6b177e2394da47	0	0	0	0	0	0	0	0	9	0	0	0
b110ecf24ade193a8b9940b5101dfaa6	0	0	0	0	7	0	0	0	0	0	0	0
7f176a0625500fd6746453f2ffc90e0f	0	0	0	0	0	0	9	0	0	0	0	0
63a612d30dab531791e34f106ceb340a	0	0	0	0	0	0	0	0	0	0	5	0
36f34a6d7f56f5026aee324f21d4cbfe	0	0	0	0	0	0	0	4	0	0	0	0
39ebd88eddd668df37da92e69707570a	0	0	0	0	0	6	0	0	0	0	0	0

69a343bdc19b0202b6e5cbf8e69c2542	0	0	0	2	0	0	0	0	0	0	0	0
262773a777b63cb883b33d69335403c2	0	0	0	2	0	0	0	0	0	0	0	0
77d759a4b502f0a9a1f3198f00dbd7dc	0	0	0	6	0	0	0	0	0	0	0	0
73204d539a1da950cc2bc46d7646f4ba	0	0	0	0	0	0	0	3	0	0	0	0
0b7023d36b56b6d021f99c41cbd3e4c1	0	0	0	0	0	0	0	0	0	0	3	0
0a02d52f83b72758bcd509aa30245e49	0	0	0	0	0	23	0	0	0	0	0	0
abdbe6c6e8bb5bfa471c3b8205e8cc8f	0	0	0	0	0	0	0	0	0	0	2	0
cf829b7d8304286020a447f925020387	0	0	0	0	2	0	0	0	0	0	0	0
8c71385354a238de609d897d6aaa625d	0	0	0	0	0	3	0	0	0	0	0	0
1484d2b8ee24308224a1f1dd42a26f04	0	0	0	0	0	0	0	0	0	3	0	0
6294511112b5897cf7cea95b6603022b	0	0	0	0	0	0	0	55	0	0	0	0
8d5bea01e6dd566be10560474de490cc	0	0	0	0	0	0	0	0	0	5	0	0
a1aef0088a425e9937be8f7cf2355859	0	0	0	0	0	0	0	58	0	0	0	0
58c310feb3d0307e306f48954eeebd5d	0	0	0	0	0	101	0	0	0	0	0	0

119eaadd1d3a954157180c58c434942e	0	0	0	0	0	0	2	0	0	0	0	0
9e8c19ee0e78a479404f1809e65d7f48	0	5	0	0	0	0	0	0	0	0	0	0
e282ff9def5b3f55db3883bf74aa4e8d	0	0	0	0	0	0	0	0	0	0	52	0
8403017cf039ff9961053bd6c4068d80	0	0	0	0	0	0	0	0	123	0	0	0
333eb386d4bdaaebabc8e69b2863e7fb	0	0	0	0	0	4	0	0	0	0	0	0
4b7efb62fb5d340d4c3610ba6ceea1d5	0	0	0	0	0	0	3	0	0	0	0	0
bd8d0f4693b780f3bdc7be900233ca1a	0	0	0	0	0	0	0	0	4	0	0	0
15270fdce4669894ef8a93ca757c7a1e	0	0	0	0	0	0	0	0	0	0	0	5
604b263f334be00bcf8af53647b5f86f	0	0	0	0	0	0	0	0	0	3	0	0
9b033557ceea82897ee25d0cad68fc24	0	0	0	0	0	2	0	0	0	0	0	0
655e258809579bb7f3f52dd814ce0ba2	2	0	0	0	0	0	0	0	0	0	0	0
206b02abe8fc32df6253bb7cfe429249	0	0	0	0	0	0	0	0	0	0	13	0
de870cedd7ea0dc4d0e581dcd62d6f91	0	0	0	0	0	0	0	0	0	6	0	0
fe8425b93293923c9b7a318bb0930589	0	0	99	0	0	0	0	59	0	0	0	0

4b37de281eaa61790b94ea6da34b2e2d	0	0	0	0	0	0	0	0	0	0	0	93
87dea27c4363f229d9fa7092217de783	0	0	0	0	3	0	0	0	0	0	0	0
3accb0d3870a2e56caf7efbb622843c2	0	5	0	0	0	0	0	0	0	0	0	0
4524a4a8a2f12108abaf1033fd995ed8	0	0	0	0	0	0	2	0	0	0	0	0
e166ffa7152edeb6268a0176e5848dbe	0	0	0	0	0	0	3	0	0	0	0	0
ecf46364b7812824544ad734dadbe0df	0	0	8	0	0	0	0	0	0	0	0	0
d0aadfd3e5a6e119acfca73477a554f2	0	0	0	0	0	0	0	5	0	0	0	0
eef8a8af9a9248917ed231c3a46f9d23	0	0	0	0	0	0	0	0	0	3	0	0
b57f8561c87b1b1ea4206caf070c5664	0	0	0	0	3	0	0	0	0	0	0	0
b8facd5a25ce7a7a79c4f2ea6fcae84b	0	0	0	0	0	0	0	0	0	5	0	0
0adc3803b22e1c131d183dacd1fb7f7e	0	0	0	0	0	0	0	0	0	0	6	0
e3dad5b7d1d30cf39b765cce7602bccd	0	0	0	0	0	0	0	10	0	0	0	0
17670ae13a6eeb9cd11a5569d3612a5b	0	0	0	0	0	0	0	0	0	0	5	0
cbd5231a588fd32ef396359e1ffd251b	0	0	0	0	0	17	0	0	0	0	0	0

a3f876c1721f3d2e3ef3746de592daff	0	0	0	0	0	0	0	0	12	0	0	0
442b4602e492ee11e7673e3ac265f8ea	0	0	0	0	0	0	0	0	0	0	9	0
bf1ca2ce63cb2d281ca4792460b19b93	0	0	0	0	0	0	0	0	2	0	0	0
7996571393c54754a7ab4f72c12aa423	0	0	0	0	0	0	0	0	0	0	3	0
9b721acdbda69cf8e2eee530b7b35377	0	0	0	0	0	0	22	0	0	0	0	0
14ee0c72e3e6a9cb918468847188d58b	0	0	0	0	0	0	0	0	0	2	0	0
6d20f4c127d87214f6f3e54926069ce5	0	0	0	0	0	0	0	0	0	13	0	0
d159bd08de1d90cc4a3408c87de29ef0	0	0	0	457	0	0	0	0	0	0	0	0
823a34cb496cc2b74372c357b691edb2	0	0	0	0	0	0	0	0	0	0	20	0
b0d296f4dd6131dca256f054f971faf1	0	0	0	0	8	0	0	0	0	0	0	0
96ae048257ece5600151705e35eddc6d	0	0	0	0	0	0	0	0	0	0	4	0
30f746402fec4b74a4304958502855f9	0	0	4	0	0	0	0	0	0	0	0	0
30ee49034a4b6358e5e4b179d72e42b3	0	0	0	0	0	0	0	0	0	4	0	0
94569d8de5617d51a72771a9a08fad34	0	0	0	0	0	0	0	0	0	0	0	25

aba72f52e035117f0681c618fb98d720	0	0	0	0	0	0	0	0	0	0	0	3
992a972f2f8d6fc20aa6765296468eb8	0	0	0	0	0	0	0	114	0	0	0	0
bfe0021694bc75f2d87cad3c5321c778	0	0	0	0	0	16	0	0	0	0	0	0
e6ead438e87e7b0d6c6bd650fc98a005	0	0	0	0	0	0	0	0	0	0	3	0
a08d1e3c6f36cd452c8b11a7989cc665	0	0	0	0	0	0	0	0	0	0	0	2
87f426449269f3c0fec0feed83a9c23a	0	0	76	0	0	0	0	0	0	0	0	0
e63a51bd485cb3f663148735f9b5e0c4	0	0	0	0	0	0	0	0	0	0	0	2
ff8f85b66cb6d2cb1cbfe962df297383	0	0	0	0	0	0	0	0	0	2	0	0
56dae0fc5385cff987debb0662affacd	5	0	0	0	0	0	0	0	0	0	0	0
7c5e89d4016fc6eb4b05d6ca7feb5919	0	0	0	0	0	5	0	0	0	0	0	0
ff596f423462ebcbe1c73a60e84951b3	0	0	0	0	4	0	0	0	0	0	0	0
90dff1499513a9fd8253cadb7d29c2ef	0	0	0	0	0	0	0	0	2	0	0	0
724b0df5cb34c524539acc69aafb9710	0	0	0	0	0	0	0	0	0	0	2	0
c0ce5d8e1ec537e51e36164b4525af78	3	0	0	0	0	0	0	0	0	0	0	0

2c3ea60ca462788ed6dcff071577bb35	0	0	16	0	0	0	0	0	0	0	0	0
f6c3d8f8835c97213b5239e4726a2214	0	0	0	0	0	0	0	0	0	16	0	0
219f3b3bb9f585b9d00bb3a9e4e6b62f	0	0	0	0	0	0	0	0	0	0	6	0
8a923ebc64699f92dadde0dcc55bc459	0	0	0	2	0	0	0	0	0	0	0	0
9c26b9bbbb27cb9b35a76e83bc843578	0	0	0	4	0	0	0	0	0	0	0	0
b1f654a3137efb315a9fcfbbf54311ff	0	0	0	0	0	0	0	0	0	0	6	0
a52e801ff5779eee75fb06701d9970ae	0	0	0	0	25	0	0	0	0	0	0	0
9b8380d494e2de9139cd3f88b9a47109	0	0	0	0	0	0	3	0	0	0	0	0
1a7ba7de04fe04ad306821eea73e4f3c	0	0	0	0	0	0	0	2	0	0	0	0
27f5fe50842255de79ac141e1156f1c6	0	0	0	0	0	0	2	0	0	0	0	0
7261f0e0c025dbd0f68ade1812ac2daf	0	0	0	7	0	0	0	0	0	0	0	0
8c9e54c47a1a0d9994fb23c983510d16	0	0	0	0	0	21	0	0	0	0	0	0
dfce3c3d82fa7cd9c32fef789dee9dcd	0	0	0	0	0	0	0	0	0	0	6	0
415cd3ffb095a47c1897f83b69df1f4b	0	0	0	0	0	0	0	0	0	0	2	0

b5ab29899feed8fc065f8014d37a513b	0	0	0	0	0	0	0	3	0	0	0	0
9ac2f7ae2f0f461a7a850dedc47d0ca1	0	0	0	0	0	0	0	7	0	0	0	0
498c05cb1834d2a893a46867c5ac6e40	0	0	5	0	0	0	0	0	0	0	0	0
5dd5e1fc3160485db3c6c162206ca041	0	0	0	0	0	0	0	0	0	0	0	3
cf140dfdfec62f924ceb547118d6f020	0	0	0	0	0	0	0	0	0	0	0	5
ae311ff26289a84d42057139cb55907f	0	0	0	0	0	0	0	0	0	16	0	0
45c12cb2e298f725a557c9b27ca44be4	0	0	0	0	0	11	0	0	0	0	0	0
98d475aba2c8a280bb9d811bec69ae0f	0	0	0	3	0	0	0	0	0	0	0	0
9c35a521a2fc9304c9db296e9d35a20d	0	0	0	0	0	0	0	0	0	0	3	0
ad6237f1dd2218930b70770854d96c24	0	0	0	0	0	0	0	0	0	3	0	0
d868cc11e9056ffbbf188f9b83e35bcf	0	0	23	0	0	0	0	0	0	0	0	0
3971844bc98bc780ac8834cf00db0840	0	0	0	0	0	3	0	0	0	0	0	0
72febdab2e09f8afa3ebe372a9361138	0	0	0	44	0	0	0	0	0	0	0	0
b397adbca837a9e94b00cec2b8c4161c	0	0	21	0	0	0	0	0	0	0	0	0

31f615ff9c4c070afe87023c0ba31723	0	0	0	0	0	38	0	0	0	0	0	0
da3c953c1af80ec8e01e739555103f4f	0	0	0	0	0	0	0	0	0	0	0	48
26bad370414efab0ed422a3b15c5744c	0	0	0	0	0	0	0	0	0	0	2	0
61a01143277692fde157f31477900e49	0	0	0	0	0	0	0	0	0	0	0	4
8d5213923e952634ba3a0d145b9a5976	0	0	0	0	0	0	0	0	0	3	0	0
98d5f83608945af3505d60a1c9e421e7	0	0	0	0	0	0	0	0	0	3	0	0
04d502caed75dd44ccbd004b6c1f93cb	0	0	0	0	0	0	0	0	0	0	0	6
e31826c23249e743f57ba639908880a1	0	0	0	0	0	0	0	0	29	0	0	0
4aa536607c713f48c1872c891042c6ff	0	0	0	0	0	40	0	0	0	0	0	0
7487a1aba5d9407ec51cbadff2332a7d	0	0	0	0	0	0	0	7	0	0	0	0
ec44a8910d17b102ed0b062ddf9ce7c0	0	0	0	0	0	0	0	0	2	0	0	0
8cf8f283d30fe1ddd8ee03947f63842b	0	84	0	0	0	0	0	97	0	0	0	0
90cb6b2d49d0bcd46ad52dc796a991c5	0	0	0	0	0	0	0	0	2	0	0	0
863d1e14797b3927ffb042e6ea128fc3	0	0	0	0	0	0	13	0	0	0	0	0

665d276adfc2e83f7304d423f0d12fc0	0	0	0	0	0	0	0	0	0	0	2	0
3bf764773de1c2d526699f2ad5508d7f	0	0	0	6	0	0	0	0	0	0	0	0
903f97d858503a5b681ae920c59df789	0	0	0	0	2	0	0	0	0	0	0	0
416ca4abea7d2e1681ace932df1cb422	0	0	0	0	35	0	0	0	0	0	0	0
ca9b5465c91b7ac66a87b4d7ede8244c	0	0	0	0	0	6	0	0	0	0	0	0
5d3fbf9b75e5d434959b8ba8f537cffd	24	0	0	0	0	0	0	0	0	0	0	0
d11de46985f12d42a611e4813e30cf9d	0	0	0	0	0	0	0	0	0	0	0	4
78e40d7a6b07c5c272f66bc1da07dae5	3	0	0	0	0	0	0	0	0	0	0	0
ae05af2430aa854df4a832e478b7ba9c	0	0	5	0	0	0	0	0	0	0	0	0
ed04440d60fe201d6463857bba477253	0	0	0	0	0	0	0	0	0	0	6	0
426c905f682ac37e674ea22d7ff50bd7	0	0	0	0	0	0	0	0	0	0	6	0
7255adf5a1b07a26b277e3e307cdf8eb	0	0	0	0	0	0	0	0	0	0	0	5
d833a290c293614b1cc0be4ea0fcc486	0	0	0	0	55	0	0	0	0	0	0	0
bde7ac3e4d05c2a9e7a741a80bf00a50	11	0	0	0	0	0	0	0	0	0	0	0

04fb7f74eac8e3e102979aae aeaf36d3	0	0	4	0	0	0	0	0	0	0	0	0
56631f508af247b44545a2c0f 2618819	0	0	0	4	0	0	0	0	0	0	0	0
e63b23c1a9e0acbb50713853 d841c438	0	0	0	0	0	0	0	0	0	0	4	0
1e924656580732391b3962b ce91838fe	0	0	45	0	0	0	0	0	0	0	0	0
c0d809be7bf6dfadeb5be80c a8256bde	0	0	0	0	7	0	0	0	0	0	0	0
de6594eca05a457d4d84364 e48f7689b	0	0	7	0	0	0	0	0	0	0	0	0
c87a58e1e36fe6da86e46fe4 2518c61b	0	0	0	0	10	0	0	0	0	0	0	0
93aef407c0082d2210cb61e2 176a2791	0	0	0	0	0	0	0	0	9	0	0	0
5620bcbb0ca531278926ff2c d797413c	0	0	3	0	0	0	0	0	0	0	0	0
5f0559c4ed72a9bfb1c98caf0 31fb3d9	0	0	0	0	0	0	0	0	0	2	0	0
14a9d6ab9941d724fe8b825d 1acaa41a	0	0	0	0	0	0	0	0	0	0	13	0
bbfaa9d2324755bf9fd6a65b e71519da	0	0	0	0	0	0	0	0	0	11	0	0
60e2a017123e7bc862dee27 e6f3df637	0	0	0	0	0	0	0	0	0	0	0	23
f852b110a1b2bf420cd55406 8feec60b	0	0	0	0	0	0	0	6	0	0	0	0

107e140bfe9ef446509145c8 3d60b2b4	0	4	0	0	0	0	0	0	0	0	0	0
a3ae17351dcb7485dda8fa8d 33e367ea	0	0	0	0	0	0	0	0	5	0	0	0
1068edf035804e9a782fcb83 a3f37b19	0	0	0	4	0	0	0	0	0	0	0	0
1c924f5b1e17aa8efa00d6c2 d3e9c6bd	0	0	0	4	0	0	0	0	0	0	0	0
2445bc4ddcc86168804effcc1 e2cf7d1	0	0	0	0	0	0	0	0	0	4	0	0
0f7b13f3e2f5ae3e37385553 052f270c	6	0	0	0	0	0	0	0	0	0	0	0
332372fe1d4a63bfdd01fac9f 3ca55fc	0	0	0	0	0	0	0	0	0	3	0	0
3f31fd3f19efa83822dba1a48 bf0c0b2	0	0	47	0	0	0	0	0	0	0	0	0
9b0232d461b1b3a8ff85ca11 8b2cc3cb	0	0	4	0	0	0	0	0	0	0	0	0
f2daa82176704045908068f9 93988cde	0	0	81	45	0	0	0	0	0	0	0	104
178d56b038955e270a466c9 1d48d30b5	0	0	0	0	0	0	0	0	0	3	0	0
35d458745bd5275ed411f7fa 64c42ef8	0	0	0	0	0	0	0	0	0	0	14	0
3a405cea554ec4665b8f9e4cf 42afdf6	0	0	0	0	0	0	0	0	0	0	24	0
0200435edb3f117ff130c6ad6 ef6b61e	0	0	0	0	0	0	0	0	0	0	4	0

de30ae9f26898d2cfbc0c801633f174f	0	0	0	0	0	12	0	0	0	0	0	0
8d3c8868ea083eef48a9d0b51e885835	7	0	0	0	0	0	0	0	0	0	0	0
e5e85b334d6d07548ed76218999d0789	0	0	0	0	0	3	0	0	0	0	0	0
fa5ef4f6e5a8501b035318cb74833185	0	0	0	0	0	0	0	2	0	0	0	0
537908eb9748d957fabc9c56848a6927	0	0	0	0	0	0	0	0	0	0	2	0
06bc014ea6640a44bb04d718a1a0e044	0	0	0	0	0	0	0	0	0	0	0	2
803a9ebd8a7d2b798e61c356d3c05c82	0	0	0	0	0	0	0	0	0	12	0	0
d2a46d20d69a820fdac9233167710dc1	0	0	0	6	0	0	0	0	0	0	0	0
24f61f70a506a02d200b934daa956efb	0	0	0	0	0	0	0	0	0	0	2	0
9b2d6829bcedd8baadc11b8208fbf147	0	0	0	0	0	0	0	0	0	0	5	0
cd8211be4a953f872e39831ce7312656	0	0	0	0	0	0	0	0	0	0	4	0
b73e51d62976b6c0e50b13a1a94df834	0	13	0	0	7	0	0	0	0	0	39	0
480b331ded05c5146558dde00e609d01	0	0	0	5	0	0	0	0	0	0	0	0
e769ec0bb5375a414fee2d7a4c73fb3c	0	0	0	0	0	0	0	155	0	0	0	0

a99035cd1d42542ba03b596ecf7a7ed9	0	0	0	0	0	0	0	0	0	2	0	0
9da3ea05e9fc6fc768f85ac352f11fc1	0	0	0	0	24	0	0	0	0	0	0	0
9b829ab74f2b0353909576fd0d3556f4	0	0	0	0	0	0	0	0	0	11	0	0
3b4c77897c196f5f612fa3526c3b37a5	0	0	0	0	0	0	0	0	9	0	0	0
a21dcf227622a99a99c9f15d7d1d98e2	0	0	0	0	2	0	0	0	0	0	0	0
ac15407de46c66aeef28172d2f4c8b30	0	0	0	0	0	0	0	0	0	0	2	0
10f2f6f722d49f4d7556b213c906b261	0	0	0	0	0	0	0	0	0	0	0	2
3154247c32cd6b27737dc2e7039f5037	0	0	0	0	0	0	0	0	0	2	0	0
5c93c0e6751fa4dfb809bc2473f51908	0	0	0	0	0	0	0	0	0	21	0	0
d1e60c9887e68b9c4648747b6a1fdbe0	0	0	6	0	0	0	0	0	0	0	0	0
6101b99614e1a7676b08692e292f6b26	0	0	0	0	0	0	0	0	0	0	0	8
0791e5bc0c12a3e8258d73352fb9562a	0	0	0	3	0	0	0	0	0	0	0	0
a7561ea6dff662f51c7fb31aa2d2573c	0	0	0	0	0	0	0	2	0	0	0	0
67ab362d9b22d8da1221b5e9ffbcf87c	0	0	0	0	0	0	0	0	0	0	3	0

339fa2714ba2c629412f58c28dfb0b09	0	0	0	0	0	0	0	109	0	0	0	0
e85f96a4ea3951ee18edbdb31eae054c	0	0	0	0	0	0	0	0	0	35	0	0
c1cdff3ee7712f365bd2e70eae5c8506	0	0	0	0	0	0	0	0	0	0	3	0
c6e73d6b5deb9fc60227ca7d1d723ddd	0	0	0	0	0	0	40	0	0	0	0	0
82ae75ffb1e2052d0be14a8b1bfc085e	0	0	0	0	0	0	0	0	0	0	5	0
8f55f68808f9a3c6ad2b4dc9fb01bd3e	0	2	0	0	0	0	0	0	0	0	0	0
86de85f1961008cdc53dcd299ec24455	0	0	0	0	0	0	0	0	0	0	16	0
899054151b98532c6d3b264bf2bde9e6	0	0	0	0	0	0	0	55	0	0	0	0
6ccefb280b6dfbea2eb6faccb6bf6455	0	0	0	0	0	0	0	0	0	3	0	0
c55619465d51ee4a2c4a467b5b7cc498	0	0	0	0	0	0	0	0	3	0	0	0
6bae586d08103fdaab8fc7a6b7b2925e	0	0	0	0	0	0	0	0	0	0	0	4
1160f3106e49d7a2a64514e06114e196	0	0	0	0	0	0	0	123	0	0	0	0
23cb2379f0fa3bed8edd3768ad599aff	0	0	0	0	0	0	29	0	0	0	0	0
7eef50c1a3f8ffb792349f178ab7884c	0	0	0	0	0	0	0	0	0	0	0	3

35830816056db54f4680c114ce18ac27	0	0	0	0	0	0	0	21	0	0	0	0
5abc7d0b78e22626ce08bb1be7baa26d	0	0	0	0	0	0	0	0	0	0	0	3
05f00220218acd0099fc8ced282ed469	0	0	54	0	0	0	0	0	0	0	0	0
4c3e763207b22855b036f1453de3ccdc	38	0	0	0	0	0	0	0	0	0	0	0
3598fb32d7a29acca8c75cad799e8cd8	0	0	0	0	0	2	0	0	0	0	0	0
3ca7f55fa89faf71fa76324059cbbd60	0	0	0	0	0	0	0	6	0	0	0	0
46ede6daf5b3a4d9f968e7ec9dafccb4	0	0	0	0	0	4	0	0	0	0	0	0
91987f46703a8b8ee8150ae2d35949bf	0	0	0	0	0	0	0	2	0	0	0	0
f10464ed055d16f2af7bccef8c28f922	0	0	0	0	0	0	4	0	0	0	0	0
483b3340e089772cf10ced1c8497d02b	5	0	0	0	0	0	0	0	0	0	0	0
83b11a84b2ba1017e350d247d26fbaa8	0	0	0	0	0	0	0	0	8	0	0	0
a3f22692250627fbdb0b6a64eed68384	0	0	0	0	12	0	0	0	0	0	0	0
8f997d3e77a7813e1d142b8034b07c46	0	0	0	0	0	4	0	0	0	0	0	0
2120501385d220cd902c957c13a005b5	0	0	0	0	0	0	0	0	0	0	8	0

07218cf287c395c7d050a13d5eeb0c95	0	0	0	0	0	0	0	0	2	0	0	0
42a3fa80d542db9350bf6cdafa550694	0	0	16	0	0	0	0	0	0	0	0	0
23edd5018b1bd50f25f130b54302c968	0	0	117	0	0	0	0	0	0	0	0	0
32faf7a093e3372e3d28c6a8336f3392	0	0	0	0	0	0	0	2	0	0	0	0
8c6860d1d41854f105c43f01134ce6ab	0	0	5	0	0	0	0	0	0	0	0	0
5e53211458fb7c3e42facd84b182e0a2	0	0	0	0	0	0	13	0	0	0	0	0
b5f8940d190ce09075ed626b7993449a	0	0	0	2	0	0	0	0	0	0	0	0
9c2fdd983a879a90e62002463288a09f	0	0	0	0	0	0	0	0	0	5	0	0
4961ad906aeaaf0f21d6eb3b1d98b398	0	0	5	0	0	0	0	0	0	0	0	0
8ee49dcac2321fb9083b7d69cbc94b6c	0	0	0	0	0	0	0	0	0	0	132	0
a4c753601a78d1abbce48415a6a698e5	0	0	15	0	0	0	0	0	0	0	0	0
bd5c1c6b0b37da91068ddeb7b63d6331	0	0	0	0	0	0	0	0	0	0	4	0
25ea6ab93062f0a3a8bcee3d5366566c	0	0	0	0	0	0	0	190	0	147	0	0
c28a0c54ffa7044a4b9edb3611c7d083	0	0	0	0	0	0	0	0	0	0	0	4

e7a6a09fa7ad39be6a8d9c49ac6e195d	0	0	0	0	0	0	0	0	0	0	0	8
61e147d6234f897065281aaea8579d94	0	0	0	0	9	0	0	0	0	0	0	0
ef6d401954dccf806e4c4f55ae6b3ec5	0	0	0	0	0	0	0	0	0	0	0	5
3f74f8bcf51cdd2c5319462e11780a0d	0	0	0	0	0	0	0	20	0	0	0	0
e94bc477b08ee3d86bb765f2143a82b2	0	0	0	0	0	0	0	0	0	0	0	5
f3eed344f48cee91b5ec18e28752ef12	0	0	6	0	0	0	0	0	0	0	0	0
0f2673504950bd774de194dd8dc41f8a	0	0	0	0	0	0	0	17	0	0	0	0
e10ca2239f6c03f3cc79e2f8bdf47b12	0	0	0	0	0	0	0	0	0	0	381	0
670b9ee450b2e8728e5f80e1848ef14f	0	0	0	0	0	0	6	0	0	0	0	0
655f690cd02956b3c283b8d5e82ef795	0	0	0	0	0	0	0	0	0	0	0	3
53592671947c7abd58278a6f5d679027	0	0	0	2	0	0	0	0	0	0	0	0
c09fc37f5e79243285722fd75b9b2406	0	0	0	0	0	2	0	0	0	0	0	0
1550cef50aaa167dfac542c477d7c3ac	0	0	0	0	0	5	0	0	0	0	0	0
0370e9dd2c4c15bf850a925343c36638	0	0	0	0	0	0	0	0	0	0	0	4

eb1cc11a12a98e7041df2dac a9d3f9d2	0	0	0	0	0	0	4	0	0	0	0	0
6fbd289a83e928c094c63df0 71109b6d	2	0	0	0	0	0	0	0	0	0	0	0
e1715f587970614b6c87c193 e49e6beb	0	0	0	0	0	0	0	0	0	0	0	30
f3bbaa4e8f0b43d1c327eed8 143ac8e6	0	0	0	0	0	0	0	0	0	4	0	0
9524f1d1ab845586b6acac32 8b74cf4b	0	0	0	0	0	3	0	0	0	0	0	0
22cb8fab6eb4422f5100696d 58ed14cf	34	0	0	0	0	0	0	0	0	0	0	0
e209c4ca6870b0d48952645f 04876f74	0	0	0	0	0	0	0	0	0	7	0	0
71b15e216b039d16b364033 5c9ce02a7	0	0	0	0	0	0	0	0	0	4	0	0
4f156ea2e273a59f403dc941 57f8d829	0	0	0	0	0	2	0	0	0	0	0	0
a31865cad9aaa498cb27b73a 946fee19	0	0	0	0	0	0	0	0	4	0	0	0
d9b76a0f1ab80448313b2032 2d445e77	0	0	0	0	0	0	0	0	0	0	0	4
3a0585fad3e5ec9d5e305331 37893130	0	0	0	0	0	0	0	0	6	0	0	0
50eaffc35583953d14744614 37b8bd70	0	0	0	0	0	0	2	0	0	0	0	0
b9a7637af7413e6418375039 0d45cabf	0	0	0	0	5	0	0	0	0	0	0	0

b28b1a22df4b2af97d0eefc457c1c32c	0	0	0	0	66	0	0	0	0	0	0	0
9441e1da07ccaa30b230bf41a749f140	0	0	0	3	0	0	0	0	0	0	0	0
c2e902abced1e9699dd263a3bfb147bd	2	0	0	0	0	0	0	0	0	0	0	0
c6096f2552f8420f0fc91c00247f7b31	0	0	0	0	0	0	0	0	0	0	0	14
0fffef827359bb6bdb1b6a71ff42d939	0	23	0	0	0	0	0	0	0	0	0	0
989f72fe8e62661b868ec03ce886a036	0	0	0	0	0	0	5	0	0	0	0	0
76d795076c71f8f4c1a8bb75f8c92e3f	0	0	0	0	0	0	2	0	0	0	0	0
edbbb7cdbfc3012a7941c850fe05fe49	0	0	0	0	0	0	0	0	2	0	0	0
5d3e79ac974736ba94c82cdcc6ddcb4c	0	0	0	0	0	0	0	0	0	0	16	0
2fa1c8068ba1868787a34e5ff15b9d49	0	0	0	2	0	0	0	0	0	0	0	0
e7fb43c63cf9850d74169fe9a6802cf1	0	0	0	0	0	29	0	0	0	0	0	0
083fa45a60e596526461334eb057eeab	0	0	0	0	0	0	0	0	0	0	2	0
e8e489b3a9b4309c5ea54192e34970fe	0	0	0	0	0	2	0	0	0	0	0	0
1c1f7a59da98ec4bf6f1fcfd1333271c	0	0	0	0	0	0	0	0	27	0	0	0

1ef4bb97171de1085a2ac3f1070a6067	0	0	0	0	0	0	0	3	0	0	0	0
5feb59b79fed8dec74bf9da09fbc203f	0	0	0	0	13	0	0	0	0	0	0	0
57ca934fc7ec74854320beb9ddde6e85	0	0	0	0	0	0	0	0	0	7	0	0
aa386b293d091deff8d0d878a9aff820	0	0	0	0	0	0	0	0	0	0	0	2
c740438e9c0a93f3db26a9f021a41f8d	0	0	0	0	0	0	0	0	3	0	0	0
ecb5cbbcc70247ed4fdaecf65684c16d	0	0	0	0	0	0	0	2	0	0	0	0
4dc08ede469ce7df3d175c584646ab58	0	0	0	0	0	0	0	10	0	0	0	0
39fd4f75120a0f794ee7131c59fe007d	0	0	0	0	0	0	7	0	0	0	0	0
0e34023072c158237de1e6d30214a2b4	0	0	0	0	9	0	0	0	0	0	0	0
9ce982938efd0c6dd7e1655949959846	0	26	0	0	0	0	0	0	0	0	0	0
ca38eb1d4944cf58b63df0ca5ceca45c	0	0	0	0	0	0	0	0	0	0	0	2
3a5e0f054be1bdcf332919a769b22801	4	0	0	0	0	0	0	0	0	0	0	0
979107c2cfac9f3d97b2380012831e5f	0	0	0	0	0	0	0	0	0	3	0	0
bc6e0ee2ca0e29148ca6cdac305b193d	0	0	0	0	0	0	0	54	0	0	0	0

8110650eda307c49847e0d3 0f9e7526d	0	0	0	0	0	0	0	0	0	5	0	0
5f80c654a1e827bd9b3cac03 8dd497aa	0	0	0	0	0	6	0	0	0	0	0	0
689a40e9caaf438fa9a9c7cc8 684b66f	0	0	0	0	0	107	0	144	0	0	0	142
da7303a9498e01eb3968e3e 444a4ea09	0	0	0	0	0	0	46	0	0	0	0	0
5c2e8b1c74b9a629164f2395 b37fe665	0	0	0	0	0	0	0	0	0	0	0	3
9ed580e80b345863714f2820 69c74c7e	0	0	0	0	0	0	0	0	0	0	0	3
a53dca24127070b504ba8d5 33ea58678	0	0	0	0	0	0	0	0	0	0	0	2
573423ed1548e77845a53e2 828475379	0	0	0	0	0	0	0	0	0	0	0	9
e1f109217df764b3740865d9 3e56f800	0	0	0	0	0	0	0	0	0	2	0	0
b1b1e78b0cf7112f9d3e5495 6a989cff	0	0	0	0	0	0	0	26	0	0	0	0
e3fd23ea9ebe0ccd4a0c5abb 610e164d	0	0	0	0	0	0	2	0	0	0	0	0
c4f665401b26324a6eb6c619 6531a680	0	0	0	0	0	0	0	0	0	0	10	0
465f0ba614eb32bb06d6dd0 e3a90cd69	0	0	0	0	0	123	0	0	0	0	0	105
5452633be37231a8908ecfb5 6213574b	0	0	0	0	0	0	0	0	0	2	0	0

89e8c32e568f619d63f3eb781cdeda5f	0	0	0	0	0	0	2	0	0	0	0	0
650b76d8dd547444eb71d750b8179847	0	0	0	3	0	0	0	0	0	0	0	0
90b39b47ead45b8ca8d6018cd8f5ab95	77	0	0	0	0	0	0	0	0	0	0	0
b54cd4c64d964d39a433ca49774a3bb3	0	0	0	0	0	0	0	0	0	3	0	0
77727412745cfe0bb2e405580aa56916	0	0	0	0	0	0	0	0	0	0	13	0
4b97ed8156772aa799a623da852e2b0e	0	0	0	2	0	0	0	0	0	0	0	0
3992ee9e623ce9f2e6e965f12aaba18b	0	0	0	0	0	0	0	0	0	0	0	17
0af2d46277d5664ef0a38689f3ab5035	0	0	0	0	0	7	0	0	0	0	0	0
708268c7487d6dd81fc9ee60f9321a6b	0	0	0	2	0	0	0	0	0	0	0	0
af6d40e5fe75b5674b042629658251ef	0	0	0	0	0	0	2	0	0	0	0	0
cf6dc099e0178bee4aa757e0b97011ca	0	25	0	0	0	0	0	0	0	0	0	0
ef0cbc8bfa3c1a99a29cc314fd8c9c64	0	0	0	0	0	0	0	0	0	3	0	0
9970eed4ad2691c10c7476b4db96f027	0	0	0	0	0	278	0	0	0	55	0	0
0d4249b56f5f707846fba9d0f5501173	0	0	0	0	0	0	3	0	0	0	0	0

7ea3bee55ebd20d39d26ed39b5664bd1	0	0	0	0	0	0	0	0	0	11	0	0
b029150081ca97febce2776db062eae3	0	0	0	5	0	0	0	0	0	0	0	0
c1fa48685280cf6db7bb7517a2b15c8e	0	0	0	0	0	0	11	0	0	0	0	0
3f22ef26b5bc9ed1ba5716f1d3bad479	0	0	0	0	0	0	0	0	9	0	0	0
e70e9c3f62afce7632664c6d0b479795	0	0	12	0	0	0	0	0	0	0	0	0
2143675d92fe1a33c74cbedb9caf6dd2	0	0	0	0	0	0	0	0	0	0	23	0
08fcfe962c5365247fa5b93612c24150	0	0	2	0	0	0	0	0	0	0	0	0
387cd3ff9084bc1bcce62f13bafc24fc	0	0	0	0	0	0	0	0	0	0	0	51
441e1062a40fb4844881e7a3ff29f4eb	0	0	0	0	0	0	0	0	0	0	2	0
53564061ee0d2d0803f24adfca0d2be6	0	0	0	0	0	0	0	0	0	0	11	0
94981499c4170d7ff5bb5d888e156036	0	0	0	0	0	0	0	2	0	0	0	0
14761eddbe364d04edab91c79056bbee	0	0	6	0	0	0	0	0	0	0	0	0
402d209353d64dc4dd172f7ee8196425	0	0	0	0	0	0	39	0	0	0	0	0
8a3c5ae2388780a418ef2030e7ea5244	0	0	0	0	0	12	0	0	0	0	0	0

0acc0e2c796f3184015fbdd882bf69d4	0	0	0	0	0	0	0	0	0	0	0	21
0cfefe84cb7c6a2405f5abf87e0093d9	0	0	0	0	0	0	0	4	0	0	0	0
955fe7599293f738656c246e4c8e4e6e	0	0	0	3	0	0	0	0	0	0	0	0
533f08700203b3da92d951c6e5ab49fc	0	0	0	0	0	0	0	0	0	0	0	4
5e0483127d364cd8325f1089905b5518	0	0	0	0	0	0	0	0	0	0	0	151
97b0f89f24b46d33ad8335c4a0a751fe	0	0	0	0	0	0	0	0	0	0	0	4
99792a3df6585b608efd9486b6335924	0	0	3	0	0	0	0	0	0	0	0	0
5608ef1613703cb673ecd79d442c0d6b	0	0	0	2	0	0	0	0	0	0	0	0
61fab7581a05e04bf1afcf4c5426c615	0	0	0	0	0	0	0	0	0	9	0	0
7a16a2c57da4addd612d4c9ae3273d5b	0	0	0	0	0	0	0	25	0	0	0	0
a3a41e59444b0deebfb825c934bb1cad	9	0	0	0	0	0	0	0	0	0	0	0
8363fb61c86aa50ad7aaea3ee4ec442b	0	0	0	0	0	2	0	0	0	0	0	0
e6b8014d976cccc5dc57bd18df592462	0	0	0	0	0	0	0	0	32	0	0	0
c6d468738886ca53f3ab5ac55cbf47eb	0	0	0	0	0	0	0	0	0	2	0	0

bb222d439cef5f7cb6644c1548b2fbc3	0	0	0	0	0	0	0	0	0	0	0	2
3c242da6c0b1aa5034cc2cc920062668	0	0	0	0	0	0	0	0	0	0	5	0
30a3f2556e079925e6c5c48dd924e52f	0	0	3	0	0	0	0	0	0	0	0	0
a845760f8ded7e9ba4c8f849092c9aa7	0	0	0	0	0	0	0	0	0	0	8	0
530ceae63cfac323faae1e4c37159df8	0	27	0	0	0	0	20	0	0	0	0	0
850814bf66e431625b28afead9daea0f	0	0	0	0	0	0	0	0	0	0	7	0
8597ee3a297df8ceaebfc1d69abe533e	0	0	0	0	0	0	0	0	0	0	20	0
630c354364a58ba0ac3cd0b5aeb32245	0	0	9	0	0	0	0	0	0	0	0	0
afdf369f5a73c58bf72a2a6f02e8af54	0	0	0	6	0	0	0	0	0	0	0	0
5fcf3b6a32ddaac36706f686a2b59c64	0	0	0	0	0	0	0	0	6	0	0	0
645f365ccc02a64a4db209b89418fca0	0	0	0	0	0	0	0	0	0	30	0	0
da09f47002589e3c9af03af7e2ab1537	0	0	0	0	0	0	0	0	0	6	0	0
7a144affd8c29900819b649c4d3dac31	0	0	0	0	0	2	0	0	0	0	0	0
7e0f434bc0cfeb72c5b0bfee09ff89ea	0	0	0	0	4	0	0	0	0	0	0	0

bb3aeff23fef00ff5aaf6d8418 8a0142	0	0	0	0	0	0	0	0	10	0	0	0
129eb823d9b4b98d608162c 024171656	0	0	0	0	0	0	2	0	0	0	0	0
d4f098b15033e4cca577b2ad 9b334326	0	0	0	0	0	0	0	0	0	6	0	0
c83f70fd8f7db9d7edc00cd02 25803ad	0	0	0	0	0	0	0	0	0	0	0	2
118d7cc45a1d68fa85e38e49 6828f5c1	0	0	0	0	0	0	0	0	0	0	0	94
2eeb994446c089dfc8b518a1 87427b6b	0	0	0	0	0	3	0	0	0	0	0	0
4a369c26a12d04a14bef4d6c 3c5c6fd1	0	0	0	0	0	0	0	0	2	0	0	0
19ae39ddd291d8d9bf1d771f f54fd8eb	0	0	0	0	0	0	4	0	0	0	0	0
8055e9b7a886e581fa5fc93c 6bfa2ce3	0	4	0	0	0	0	0	0	0	0	0	0
f49e8b269026b4f39ff197f3f6 aa6b5b	0	0	0	12	0	0	0	0	0	0	0	0
d7c8ed9196303f18d5f09e64 302839df	0	0	0	0	0	0	0	17	0	0	0	0
fd691faa6868dcdd567154f3f 92f9154	0	0	0	0	0	0	0	4	0	0	0	0
6d50ae9b1cea215fab093edd 8fc74247	0	0	0	0	2	0	0	0	0	0	0	0
8d7f2d221320c1cf2ccbb806c 5f91960	0	0	0	0	0	0	2	0	0	0	0	0

08ca608f21b60eef1b042dda22bd65aa	0	0	0	0	0	0	0	0	0	0	5	0
e19499e0d02c4809ea3f5a3dd20e3a01	0	0	0	0	0	0	0	0	0	0	0	6
b5f24d54654c733bf260c4a3b0c7be52	0	6	0	0	0	0	0	0	0	0	0	0
8371ce2364ecc6a6c9e75e3d392debd6	0	0	4	0	0	0	0	0	0	0	0	0
4f22629e43f2619774c8364892823bb1	0	0	0	0	0	2	0	0	0	0	0	0
080b7ee8697741c1b4efe7bf2aa5430f	0	0	0	0	0	0	0	0	0	0	4	0
12df044d131c02745757cb895be4031d	15	0	0	0	0	0	0	0	0	0	0	0
088476a47a8320fc3a6a68b3f098992b	0	0	0	0	0	0	0	0	0	0	3	0
84fe253b33c6b773854b560bb86fa73c	0	0	0	0	0	0	0	4	0	0	0	0
84185946ed8a07e347f7f3100169751a	0	0	0	5	0	0	0	0	0	0	0	0
ba3574b2f55835023654af1250508702	0	0	0	0	0	0	0	9	0	0	0	0
9b5c7a7a2559f92dcfb73ee2937eb06a	0	0	0	0	0	0	0	0	0	0	0	4
391e307dd7ac126bbb8045f89c203e24	0	0	3	0	0	0	0	0	0	0	0	0
2d3f26152c38b931eb3483fd0809c878	0	0	0	0	0	0	0	0	0	0	80	0

001de1e028fdfa2308987e06a541294e	0	0	0	0	0	0	0	0	11	0	0	0
97390e0a98a718ecd2b68c2630639d49	0	0	0	0	0	12	0	0	0	0	0	0
ae58eedcc1a95a5824ad68d9fc38fae7	0	0	0	0	0	0	0	6	0	0	0	0
920720b7a1e94a0f0f92818623a1205d	0	0	0	0	0	0	2	0	0	0	0	0
dc1b57166719218fd67dc08796890146	0	0	0	0	0	0	0	0	2	0	0	0
eeea87a06d1400792caeb9cd51d06aa8	0	0	0	2	0	0	0	0	0	0	0	0
4e4a0617030e39d1b1404c75e692c21c	0	0	0	0	0	0	0	0	0	2	0	0
5a3614573415b618251328fa1149eeac	0	0	0	2	0	0	0	0	0	0	0	0
45d616f21b79dcb16e5caab47436dc32	0	0	0	0	0	0	0	5	0	0	0	0
d32d362a0102b4eda3e959013ba6a1aa	0	0	0	3	0	0	0	0	0	0	0	0
d37b52ca0aebe4286e59a3b59938debc	0	0	0	0	0	0	0	120	0	0	0	0
6b113dda33e7db3d490f81c1f9fe825e	0	0	0	0	0	0	0	0	0	0	6	0
0d9b72dffee2909f3e5cfca44bd5e989	0	0	0	0	0	0	0	5	0	0	0	0
bb36e546d851586b980f3c5f6469ac8e	0	0	0	0	0	0	0	0	2	0	0	0

44ca8358b955f9afc8bd120b531916ac	5	0	0	0	0	0	0	0	0	0	0	0
8c5c455b72d69d8d8fd6baea3b1f1681	0	0	0	0	0	0	0	3	0	0	0	0
c1eb48feeb7051fa1b6731a7f2556bdb	0	0	0	0	0	0	0	3	0	0	0	0
9131f3202c860ac2dbdff571652b549a	0	0	0	0	0	0	0	0	0	17	0	0
9d6988665d8a36c820e926b402c9e878	0	0	3	0	0	0	0	0	0	0	0	0
65a6c71e7ecd9b3144dc07a7267ace29	0	0	0	0	0	0	0	0	0	0	0	2
444027106fd03219a97fb2185586574a	0	0	16	0	0	0	0	0	0	0	0	0
4037377d9513598c80889530bf73852c	0	0	0	0	0	0	0	0	0	3	0	0
df99cdaa0b71fb5c3f5604dccc631ba3	0	0	0	0	0	0	0	0	5	0	0	0
927f6c4009c594b10679ff549355733c	0	0	0	0	0	0	0	0	0	53	0	0
63f4ce7272c6a6bea6e409b26dd9b124	0	0	0	0	0	50	0	0	0	0	0	0
4ef3155431cdfaef063d8f951ffdeac6	0	0	0	0	0	0	0	0	5	0	0	0
bf9b334d880b30a895a972537c21c4d8	0	0	0	0	0	0	0	16	0	0	0	0
6195a9689e1e4f507719f946e420d003	0	0	0	0	0	0	0	0	0	0	13	0

05b71cbdcb6ea89350e191aa784e1a1e	0	0	0	0	0	23	0	0	0	0	0	0
55e5c12a35be31f3ad64582c50774bca	0	2	0	0	0	0	0	0	0	0	0	0
795c071b77116bfeba28cb9ccce42bf5	0	0	0	0	0	0	0	0	0	2	0	0
7f463a8dffeda30bb83df8a713618551	0	0	0	0	0	33	0	0	0	0	0	0
b7cc8960dda9a876709f9db856fa91c1	0	0	0	0	0	0	0	0	0	19	0	0
f4bc4179f4aad183ced8a28d3b432710	42	0	0	0	0	0	0	0	0	0	0	0
a9a3bec2a5ad919e5b2717d3c49dd63e	0	0	0	0	0	8	0	0	0	0	0	0
47a6e4237ea159b320e4ebecf7523d30	0	0	14	0	0	0	0	0	0	0	0	0
f4d913d0bccf69fb76cf6d4e653f245e	0	0	0	0	2	0	0	0	0	0	0	0
93e8bf22ed184cf0bdbdbff10db4dadc	0	0	0	0	0	3	0	0	0	0	0	0
b4dfd7f8f456996889989df48a3a5e96	0	2	0	0	0	0	0	0	0	0	0	0
9684f2a5a0d47154c91f25a6ef9dbace	0	0	0	0	0	0	0	0	0	0	35	0
9b23595ad760ffd1931261d052e0c896	0	0	0	0	0	0	0	0	0	0	2	0
aa8304617c5fb702de205509f580c3ce	0	0	0	0	0	0	0	0	3	0	0	0

7616094fb657ca5ca3a4dbff7111f4ae	0	0	0	102	0	0	0	0	0	0	0	0
c008cb5947838ac3ed364fea7e8f1c5f	4	0	0	0	0	0	0	0	0	0	0	0
174de0d48884cf7f11cc298296cf4f35	0	0	0	0	0	0	0	0	0	0	0	9
94bd09c122aa17bf7fdf0f1aca8975c0	0	0	0	0	0	0	3	0	0	0	0	0
7387f146ee68e47ee15aff3aed068742	4	0	0	0	0	0	0	0	0	0	0	0
0221891bcc8d229e2e020c0399dd525b	0	0	0	0	0	0	0	0	0	70	0	0
fd3d830ad151bee18ad8abb311fc8bee	0	4	0	0	0	0	0	0	0	0	0	0
bbd2a2b5679c465cf6c9d99b2edbcf32	0	0	0	0	0	0	0	0	0	142	0	0
b946ad9fbab43118c0e1b73b2fc6ba54	0	0	0	0	0	5	0	0	0	0	0	0
10f61aef30cdece38cb4af9e350a5140	0	0	0	0	0	5	0	0	0	0	0	0
74329b1c56c5869980448f0827ab5e21	0	0	5	0	0	0	0	0	0	0	0	0
0ca9d3a85045846a55cc66d4b79fa229	0	0	0	0	0	2	0	0	0	0	0	0
b92af2caba9dd1b787d850707c949703	0	0	0	0	0	0	0	0	0	0	66	0
b86d7d9dd22d990b9e6f3ed5326268ea	0	0	0	0	0	0	0	9	0	0	0	0

f0572490695574d66993f7747efa4a03	0	0	0	22	0	0	0	0	0	0	0	0
381dd6f660b8f39e31c011557bd514c9	0	0	12	0	0	0	0	0	0	0	0	0
6d3ae27c8cde53ba93a1da1a2b673985	4	0	0	0	0	0	0	0	0	0	0	0
2981d9b5af721e4c389dc87380ac7c0d	0	0	0	0	5	0	0	0	0	0	0	0
80276b47c1bc5ea1d05c53991523373e	0	0	0	0	0	0	0	0	0	0	2	0
177bd30aa49363f0c4c388c69518f75a	0	0	0	0	4	0	0	0	0	0	0	0
20582b3f5a6b3c197463351f462cf383	0	4	0	0	0	0	0	0	0	0	0	0
696e0ee634b3192440bb5f94a6667475	0	0	0	0	0	0	0	13	0	0	0	0
d1582ab0a644255c94cf1db68ab4f513	5	0	0	0	0	0	0	0	0	0	0	0
fbeeaaa8c758fa7026556fb4a0151d72	0	0	0	0	0	2	0	0	0	0	0	0
25a4834af8a5fc4fd14dac5b4236b146	0	0	0	0	0	0	0	114	0	0	0	0
c30ed0083028c6386ab197f8c1778a7e	0	0	0	0	0	2	0	0	0	0	0	0
0b9ebe3b6a3e040ab20196370c0be66c	0	0	0	0	0	0	0	2	0	0	0	0
bd174998bbca057e62e9ec2a1bdfaf69	0	0	0	0	0	0	0	0	0	0	4	0

16167ac884b074f5372a9d5b a474b322	0	0	0	29	0	0	0	0	0	0	0	0
8af590a999f5148479d2d0d9 d7e65e1b	0	0	0	0	0	0	0	0	3	0	0	0
794cb795ab74c26a3d0baf52 8d6bbd79	0	0	0	0	0	2	0	0	0	0	0	0
7971e2c9569f5a637b6870a1 e74ca337	0	3	0	0	0	0	0	0	0	0	0	0
c3ffd0fe823c1b9dd0aae48c6 3857228	0	0	0	0	0	0	0	0	2	0	0	0
61b6403fe71885ff3b3d6fe56 d27569e	888	1571	0	2345	1514	2462	1356	2924	1147	1862	1751	1994
3050abc73013e5acc13d70c7 c585c9a3	0	0	0	30	0	0	0	0	0	0	0	0
a24c182a3862ed3b5393337 1bcd72d3b	0	0	0	0	0	0	0	0	0	2	0	0
01f81be2054013aa06129d37 8e93a6e0	0	0	0	0	3	0	0	0	0	0	0	0
b0b26e7f55f6cfea2346c34a0 ef0f668	0	0	0	0	0	0	0	4	0	0	0	0
722ef40271059d5c09360ad5 91770dc6	0	0	0	0	0	0	0	0	3	0	0	0
f9d362b20f85053a246326ae 943994a7	2	0	0	0	0	0	0	0	0	0	0	0
7cda0fcb9d155ff66ddeceb8d abd0fc1	0	0	0	0	0	0	0	0	0	10	0	0
7244c1bf74717810ec7e233b 93a0aafd	0	0	0	0	4	0	0	0	0	0	0	0

ec7b3ff91e2fe01f78eef87b04715c4b	0	0	0	6	0	0	0	0	0	0	0	0
b54d5244b287c74002bb24ae76a7361d	0	0	0	0	0	0	0	0	0	0	13	0
b194dc21c885ab1efd56e12a413db952	0	0	0	0	0	0	0	0	0	0	0	2
8d10a94efa332d511eba7c565a5e7e39	0	0	0	0	0	0	0	0	0	0	9	0
a91e6f2dce0d2d101084824ae5e37726	0	0	0	0	0	0	0	0	0	4	0	0
7bf45b0dfe77ba8881911666b5b1e3e9	0	0	0	0	0	7	0	0	0	0	0	0
dda30fdf640d99faed0e45163aa1e578	3	0	0	0	0	0	0	0	0	0	0	0
fc08c7abff303ce3bb8041ca66be8a71	0	0	0	0	0	0	0	0	2	0	0	0
60676013231629255ae8911276bf677d	0	0	0	0	0	0	0	0	0	0	49	0
4f017ec3ceca8b020f49aef985a6dea4	0	0	0	0	0	0	0	0	2	0	0	0
290770d3bc497c8e59b9494d33a4e47a	0	0	0	105	0	203	0	0	0	0	266	0
dbdfb2c45ea67870cda253c7b26340da	0	9	0	0	0	0	0	0	0	0	0	0
cdc7e81fd8061948611aed4848efce76	0	0	0	0	0	0	0	0	0	0	75	0
32a942a9408df55333e085da45f7ee25	0	0	0	3	0	0	0	0	0	0	0	0

d0e22c37cdb09a8777048cf0d0b6a925	0	0	0	0	0	7	0	0	0	0	0	0
5d65c37b33d94d5bbfadde52cc683027	0	0	0	0	0	0	0	3	0	0	0	0
3569e63072c21319118c1810175935bb	0	0	0	0	3	0	0	0	0	0	0	0
d89018b0d047999f083703a571a9ce77	0	0	0	0	5	0	0	0	0	0	0	0
63f5d6cf45b8bc06fb75cd0adfe8d460	0	0	0	0	0	0	0	0	0	0	0	3
6c416cdfd16400f842ebd7632e5f6270	0	0	0	0	0	0	0	0	10	0	0	0
cf515770f3488a8ddfd10e515fe275bf	0	0	3	0	0	0	0	0	0	0	0	0
3b576cbc6047c205fadb9f100ae4c237	0	0	0	52	0	0	0	0	0	0	0	0
127c86999c3b018ec78faebad3293702	0	0	0	0	0	7	0	0	0	0	0	0
8f00de9492de698b58cf77483e14a355	0	0	0	0	0	0	0	0	3	0	0	0
e39bb1b9e2e955a611bf1eddbc45b38c	0	0	2	0	0	0	0	0	0	0	0	0
d053b0b50c079e9164c2ecd04fed040f	0	0	0	0	0	3	0	0	0	0	0	0
4c7ca3e5d2a0127072bf4ad82ca863ea	0	0	0	0	0	0	0	0	2	0	0	0
59f5fa7523cc80f5865dafce7ff16d1c	0	0	3	0	0	0	0	0	0	0	0	0

e96b569b798b95f74ab5eb18f1773e91	0	0	0	0	0	0	0	0	0	14	0	0
e6b68795c14da4688c388a927728640e	0	0	0	0	0	9	0	0	0	0	0	0
daa69c0b54f48044161a093d0c597b8f	0	0	0	0	0	8	0	0	0	0	0	0
734a14aae8fc375b3522e8d46d7837eb	0	0	0	0	0	0	0	0	0	3	0	0
b0d8b73b99a149a1bf9b645f45b5c1d2	0	0	0	0	0	0	9	0	0	0	0	0
da13058c6c154862b958a7fad3d848f9	0	0	0	0	0	0	0	0	0	0	0	4
541d892aa08f3e3ef7836c43fce1fc0c	0	0	0	0	0	0	132	0	0	0	0	0
4619c091f28171e85b084e2e028542d9	0	0	0	0	0	0	0	0	0	0	4	0
c60aaabd780f69d268aea4fc5a75ebb5	0	0	0	0	0	0	0	0	0	0	12	0
9b2c57a4cc8c86e4af0e9f278644e6c3	0	0	0	0	0	0	0	0	0	0	0	12
f4750c06018fc1f89c45c346c6482f8a	0	0	0	0	0	0	0	6	0	0	0	0
4a3157a7aea51e0cc6aee57d10677e13	0	0	0	0	0	0	10	0	0	0	0	0
4e7a8630fd9adba3e5504d41c0307fe8	0	0	0	0	0	0	0	0	3	0	0	0
986a41efa730a423bcf87c0dff1fb42a	0	0	0	0	0	0	0	0	0	0	0	3

1c0b630fa836730ed24f2bc9175d3675	0	0	0	0	0	0	0	0	0	0	4	0
88ac448703deab0f7e86dc80e5b1aa52	0	0	0	0	0	0	0	0	0	0	3	0
26a71d7682627bcecd3c208cfe7c9d26	0	0	0	0	0	0	0	0	0	0	3	0
bdf17cc3a95cd8d4b74b683590983a43	0	0	0	0	89	0	0	0	0	0	0	0
584b5b67da5dd74bbd7d424f317f6dca	0	0	0	0	0	2	0	0	0	0	0	0
1bd8ca97dc31158fd1d77ef356dabc00	0	0	0	0	0	4	0	0	0	0	0	0
69b17875710809cdc906e317ff838a8a	0	0	0	0	0	0	0	0	0	0	6	0
7665f821075bb4fafc1bf151103b55da	0	0	0	0	0	0	3	0	0	0	0	0
32698a026b14443bfdd7021676ab63ad	0	0	0	0	0	0	0	6	0	0	0	0
80ab717f9daa7de8fc12cc6adfc48a07	2	0	0	0	0	0	0	0	0	0	0	0
49f8582d478dba6a00c4a941fc928490	0	0	0	0	0	0	0	0	0	16	0	0
253ae3b07b7431e6ca8e29453c0c09a9	0	0	0	4	0	0	0	0	0	0	0	0
32637b1626b129fb78f6a76c1dba1d03	0	0	0	0	2	0	0	0	0	0	0	0
d6b5dba2eaa2d53cc78a49b144123ca3	0	0	0	0	0	0	0	0	55	0	0	0

24aacfe30c5d7580f521aa0c99bbc4ea	0	0	266	0	187	0	0	388	0	0	0	108
de60966c9babee752a2f45a16e2787b6	0	0	0	0	0	0	0	0	0	4	0	0
48ddfe3363574321b3985aa894d23d85	0	0	0	0	0	723	0	0	0	0	0	0
6402ec8595e49c50abb52e40f0926736	0	0	0	0	0	0	0	5	0	0	0	0
ce31fcac74e5eb83ea3dc7eb67a57c5b	0	0	0	0	0	0	0	0	34	0	0	0
5496f55b8aa3e747b1dfed8673d08505	0	0	0	0	0	0	0	0	8	0	0	0
b22b023353e263a50eece7e578ca3d91	0	0	0	0	0	0	0	0	0	0	0	2
60eba9ff7403ffd547deb5c364bdd9c3	0	0	0	0	3	0	0	0	0	0	0	0
5325bc1cc06edcc22c5a035c49f6243c	0	0	0	0	0	0	0	0	5	0	0	0
b1a2c1e1e678200c4d87714a666d3491	0	0	0	13	0	0	0	0	0	0	0	0
2707b573f40a5a63e1cc27497f7f4fd1	0	0	0	0	0	0	0	2	0	0	0	0
ab3c468d5f434921dd5d26c45fa2f295	0	0	0	0	0	0	0	0	0	0	5	0
7339206b25a9c4a9b52295c49b508a61	0	0	0	0	0	0	30	0	0	0	0	0
442546dea441dd5eeeaed3f38808aaea	0	0	0	0	0	0	0	0	0	32	0	0

ab2230104b461edc985ebc6df4d1024b	0	0	0	4	0	0	0	0	0	0	0	0
65742cd7309131c066d50254c5ba36a5	0	0	0	0	0	0	0	23	0	0	0	0
473bac2cf2ae1d373fbebf149093ac8a	0	0	0	0	0	0	0	0	0	0	0	5
fdbb51abb321b45898cb58e51b004266	0	0	0	0	0	0	0	0	0	0	20	0
4f44fb1941f1d158004aa8b839af889f	0	0	0	0	0	0	2	0	0	0	0	0
c8eeb6f114369e38a58483dfb86c86be	0	0	0	4	0	0	0	0	0	0	0	0
916f917cca65e6d94979916ac9d3ac5d	0	0	0	0	0	0	0	0	0	0	2	0
ca04a0a1120cd758074fe412e99e8e26	0	0	0	0	0	0	0	0	0	2	0	0
6c4d87d341b2c84e8cdb2b4d325566a9	0	0	0	0	4	0	0	0	0	0	0	0
64551363752fc7f51e11c28e7c9545db	0	0	0	0	0	0	0	0	0	0	4	0
607c230e5f8a880f0defc855200b0395	0	0	0	0	0	0	0	2	0	0	0	0
96b72affff8d64a9135f0d2fd1e48470	3	0	0	0	0	0	0	0	0	0	0	0
5343b120b57e88a2e64ff9d8476fb0f7	0	0	0	0	0	0	0	3	0	0	0	0
57f64a345f7940620281ace4882d0115	0	0	0	0	0	0	0	0	0	0	0	17

3494e6be063ef5a3975c90a48cef3ae4	0	0	0	0	20	0	0	0	0	0	0	0
e4fe9b4f997386e6e8c7181b54430313	0	0	0	0	0	0	0	19	0	0	0	0
3efbffe7eee922273b564821b638561d	0	0	0	2	0	0	0	0	0	0	0	0
35d1b290c1e6bc0495d2e323474c05dd	0	0	0	0	0	0	3	0	0	0	0	0
bd1f6ecb4a5d55dd508a9ee63018e7a9	0	0	0	0	0	7	0	0	0	0	0	0
a98091297b8c6cef3cb1d84ffc607688	0	0	0	0	0	0	0	0	3	0	0	0
c0bd8b66c65784773fad9fdbb8a6faa1	0	0	0	0	0	94	0	0	0	0	0	0
49f0e2c93d59856203d0ead98d336164	0	0	0	0	29	0	0	0	0	0	0	0
e7769845eedc9d00e7af086fa888c6da	0	0	0	37	0	0	0	0	0	0	14	0
47afe2a2197dead52c51844b074d6f7e	0	0	0	0	0	0	0	0	0	0	0	30
c3b5402be5f894db1513f5f5aa8f938a	3	0	0	0	0	0	0	0	0	0	0	0
0d941e1619d16ee7fb247e8994ad0d82	0	0	0	0	0	9	0	0	0	0	0	0
62c7263a48a7ddd584f9ef59d99fe19c	0	0	0	0	0	0	0	2	0	0	0	0
1078e505361a3bb3b898cb76d15a851f	0	0	0	0	0	0	0	0	0	0	13	0

42df97e17efab483d5cc8f84d d4abb49	0	0	5	0	0	0	0	0	0	0	0	0
f4ef77114ed0f4a30a014a04a 8e74682	64	0	0	0	0	0	0	0	0	0	0	0
4e1a7996daf757c71088528a cead9149	0	5	0	0	0	0	0	0	0	0	0	0
a3e879ec082fa11842a48ea5 5ab30163	0	0	0	0	0	0	0	0	0	5	0	0
6e5a166655afff8478a390a70 3f5d7ff	0	0	0	0	0	163	0	0	0	0	0	0
0fb86dbe351dcd2a8b1047bb 5c69ebba	0	0	0	0	0	0	0	5	0	0	0	0
c9bbf4e187af99e7ab38996c 6d7462c1	0	0	0	27	0	0	0	0	0	0	0	0
9547173deebf7733dd3472cd 419d6832	0	0	0	7	0	0	0	0	0	0	0	0
c0517c22f85b12b81f74b51b ea59480d	0	0	0	0	0	0	0	0	0	0	0	2
aca88a7022ab826452a5ba37 d03353fe	0	0	0	0	0	0	0	0	0	0	0	5
92f311946b8605afb82116bd 23797474	0	0	0	0	6	0	0	0	0	0	0	0
adfb279ca11de481e04873e7 4131f165	0	0	0	0	0	0	0	0	0	0	0	12
c43cfc293d8a0c2e5fa9422ac fbaaeeb	0	0	0	2	0	0	0	0	0	0	0	0
f57618811dd33f522ab2e8ad 3a0010a7	0	0	0	0	0	10	0	0	0	0	0	0

20a33ea25ab67011f9ddc0b7bb681f4d	0	0	0	0	0	17	0	0	0	0	0	0
491944f02225c4f9dbb3ea96feee5ded	0	0	0	0	0	0	0	0	28	0	0	0
bd8e3a05b7cb2bfefbee58417b26f593	0	0	0	0	0	0	3	0	0	0	0	0
e5f2426bd4a4e6517447964f926ae7f5	0	0	0	0	0	0	0	0	0	0	0	2
f3a74ea8af2956fb90471d5d65d0cab7	0	0	2	0	0	0	0	0	0	0	0	0
6916365fcf91d8fbcb96e9ff14900c16	0	0	0	0	0	0	0	0	0	0	4	0
3fb146730c69c7c68f6e442b2142ee12	0	0	0	0	0	0	22	0	0	0	0	0
06f747d302cbfd525e4343e055f96933	0	0	0	0	0	0	0	0	0	0	3	0
244d722c2e977226c1f82ac59e8ad263	0	0	0	0	0	0	0	12	0	0	0	0
f3c3b26a4772ea0472e6a55637b15a7d	0	0	0	0	0	0	0	0	0	27	0	0
b04b84226440977f99a453d9a8461f05	0	0	0	0	0	0	40	0	0	0	0	0
4a81f9609cc63e5874ab4db105ba4208	0	0	0	0	0	0	5	0	0	0	8	0
3160085334be15afe95eebb4f69814fe	0	0	0	0	0	0	10	0	0	0	0	0
abe31d19c590465f55e7478bb8c7844f	0	0	0	0	0	0	0	0	0	0	3	0

1b78e3ee5c573cb4501b7dd14a827c71	0	3	0	0	0	0	0	0	0	0	0	0
db3cad8a42f02a898a03f51cd83ed71e	4	0	0	0	0	0	0	0	0	0	0	0
72e44e36d6b72470acff4df333eac4ad	0	0	37	0	0	0	0	0	0	0	0	0
802fe3390b60b4afd69f01721d5e5182	0	0	0	0	0	0	0	0	0	0	0	10
ac156657faea2ead869a4527c54bffba	0	0	0	0	0	0	0	0	0	0	0	15
a136f1447b85043e95d89f4c927d1615	0	0	0	0	0	0	0	3	0	0	0	0
22d7bd35efdf77b5d3ec9bc67430d676	0	0	0	0	15	0	0	0	0	0	0	0
baaa16f78877fe63e179a59d5bec9770	0	0	0	0	0	0	0	0	0	0	25	0
e1d2a8e7b7a206118b1cf9f3a37b192e	0	0	0	0	0	0	0	0	0	0	5	0
76a5632c4463106426978dc50a461e2d	0	0	0	0	0	0	0	0	3	0	0	0
13b7b65571139369e25c97870b86c4f3	0	4	0	0	0	0	0	0	0	0	0	0
27626f6d9c6db1833457612d6004a1b5	0	2	0	0	0	0	0	0	0	0	0	0
8b5d943d7372f46ff35ed98ac9577bae	0	0	0	0	0	0	0	3	0	0	0	0
27ca09d1ef70cbd6113be89e12013353	0	0	0	0	0	0	0	0	2	0	0	0

90b0f790d46918b9fd66a56cfa8ce4ea	0	0	0	0	0	0	0	0	0	0	5	0
b7dba3ca1f89b84adc82e30d863e9700	0	0	0	0	5	0	0	0	0	0	0	0
7e2b2dd27a5929714b4823977876aa33	0	0	0	0	42	0	0	0	0	0	0	0
89b49b1daf80017f161cc31c5fe30848	0	0	0	0	0	0	0	0	0	3	0	0
e997799ef0cbb3382127280f97931cf9	0	0	0	0	0	0	0	0	0	3	0	0
a11b5726ad682959b841d52ee4ee3405	0	0	0	0	0	0	0	0	0	0	0	3
b1b097b33b10de3ddc32c9b56ed8cda0	0	0	0	0	0	0	0	0	0	0	11	0
b365f3ed04fd020a8d7daf5c4c897ec9	0	0	0	0	0	0	0	0	0	0	0	10
f83a59876ca95db02bbd99ddd77c5840	0	0	0	0	0	0	0	0	0	0	0	2
9d06f4bbe285a90a1c8c52edef1f414b	0	0	0	0	0	8	0	0	0	0	0	0
58e272dd15ce993c15771d5429b9871f	0	0	0	0	0	0	0	0	0	2	0	0
ef06394752bbcb5a51a43bdebbb0513b	0	0	0	0	0	10	0	0	0	0	0	0
e354ca157b502269377d81e67a936a80	0	0	0	3	0	0	0	0	0	0	0	0
a9bbc094a50a8877427f417130b7f2ac	0	0	0	0	0	0	0	3	0	0	0	0

72211860fc891e58f6cd3c56 32b6dd3d	0	0	0	0	0	3	0	0	0	0	0	0
5f09b23a3756e496ced38b2e 0dbf1895	0	0	0	0	0	0	0	0	0	0	2	0
a8c5dec04a0af496c5084c32 bc97a778	0	0	2	0	0	0	0	0	0	0	0	0
cd6dc0afb4b4c401db233f8df 194d913	0	0	0	0	3	0	0	0	0	0	0	0
4a9f871bf97f16ed7c7ad7eb3 5ffad01	0	0	0	0	0	0	0	0	0	0	19	0
710fd51be8353da1323aa931 f3936635	0	0	0	0	5	0	0	0	0	0	0	0
a656a0bc7df2e9882494952e 3efe4bf1	0	0	0	0	0	0	0	0	0	0	0	27
328b43a9c0c3d13f394c649e e2700e8a	0	0	0	0	0	0	0	33	0	0	0	0
b9ccdbbe257bdc7e3782d6b 368038f47	0	0	0	17	0	0	0	0	0	0	0	0
25e5c1e4dbed1ec3d711150 39ca55a20	0	0	0	0	0	0	3	0	0	0	0	0
abb0df2da3d19ec095027e42 8417fb2a	0	0	0	0	42	0	0	0	0	0	0	0
6d209077c3698b939fe738de 33b339f2	0	0	0	0	0	0	0	0	0	0	11	0
273f73f481786533b9b39006 9be09e78	0	0	0	0	0	0	0	0	0	0	3	0
cb7950967ac2a73b00e1abc0 4189c1e6	0	0	0	0	0	0	0	0	0	0	0	2

fdcbb5e54ffd70c8c9eac882e5df0e57	0	0	0	0	0	0	0	2	0	0	0	0
d1927cf39be8dd9564a122ff6d030bca	0	0	8	0	0	0	0	0	0	0	0	0
8f2d13f4b834aea8f010f80a377d7bc9	0	0	0	0	0	0	0	0	0	58	0	0
343c8ff846f5a52ce48f88cd9c98bd34	0	0	0	0	0	0	0	4	0	0	0	0
cfbb1d7817230d72021f8a96548b5103	0	0	0	0	0	0	0	0	0	0	10	0
8e65dbe20556a5eb8cdce04c870086dc	0	0	0	0	0	0	0	0	0	7	0	0
953c1fd63b45fd174001cb4a64f727d1	0	0	0	30	0	0	0	0	0	0	0	0
99ab973e926914668f3e5aa1c90087d9	0	0	0	12	0	0	0	0	0	0	0	0
72b8109ac79bc1e679b05e7a5695be15	0	0	0	0	0	0	0	0	0	0	41	0
b60f225b0634ef47cf41569d177bd3ba	0	0	0	0	3	0	0	0	0	0	0	0
69869f82f08d067c8a7a1f045e4f455b	0	0	0	0	0	0	0	0	0	3	0	0
60bfa02ee8f0b58572b7844938d0eaee	0	0	0	0	0	5	0	0	0	0	0	0
79ed09d58513db7871426f34939284ed	0	0	0	0	5	0	0	0	0	0	0	0
1fc4d76d49a031a31275a47d080a0942	0	0	0	0	0	0	0	6	0	0	0	0

a3ed2a8d5f0732047ed014a76b5164ee	0	0	0	0	0	5	0	0	0	0	0	0
cb9aaf0edb903de56bf863408dc79849	0	0	0	0	0	0	0	0	0	0	15	0
8dc00f80452287d1925d0f4015e04e73	0	0	0	0	0	0	6	0	0	0	0	0
718aef0184f676ad4808c3b35167cff2	0	0	0	0	0	0	0	0	0	0	5	0
96ff4704ca8a8de9b768a347379476a8	0	0	0	0	0	0	0	0	0	0	8	0
605478a7cefb8a6d5f3188320cc16a30	0	0	0	0	0	0	6	0	0	0	0	0
f8de18feecce7e9a72311dde27c7c431	0	0	0	37	0	0	0	0	0	0	0	0
acbb779be10a0eae7d4b42c02c216389	0	0	0	0	0	0	5	0	0	0	0	0
e682fea45559b8eaf65ad209a65e5ac4	0	0	0	0	0	110	0	0	0	0	0	0
5a7d99da8c33c82b59a825026e207ef5	0	0	0	0	10	0	0	0	0	0	0	0
ba064430072cbb8f2ec9a5a6b745fb82	0	0	0	0	0	0	0	5	0	0	0	0
5bd4e0e4aedc99c9e21a8bfb0989f0c4	0	0	0	0	2	0	0	0	0	0	0	0
dd1ef9842a8fdb9f6cda4f0f1c406b17	0	0	0	0	0	0	4	0	0	0	0	0
0d74da2ccd7f1fa5b9f0119322a2cca0	0	0	0	0	0	0	0	0	0	0	0	50

0c07eebd7c10bf095718f2c43f662c8d	14	0	0	0	0	0	0	0	0	0	0	0
636d14673b6e2718265a38887aabaca3	0	0	0	0	0	0	0	0	0	0	3	0
3f8abe7bc39421b0d12e5f67b86e4802	0	0	0	0	0	0	0	0	0	6	0	0
df763d07b5dd91f30d83be59caf9dbc3	0	0	0	0	0	0	35	0	0	0	0	0
a0e2024b5b40ac1748603a222cbe01e5	0	0	0	0	0	0	0	0	0	0	4	0
0a0cc20a8e4c425f57a6e3fc7c139ea5	0	0	0	4	0	0	0	0	0	0	0	0
0fe5d508c932934a5aae1c858af96727	0	0	0	0	0	0	0	0	18	0	0	0
085c4068e414a17d5f21144b33757e46	0	33	0	0	0	0	0	0	0	0	0	0
d5c0d5ecbdd7ee364dceade65931bfa9	0	0	0	0	0	0	0	0	0	3	0	0
818fc5d2be59c558e1994d52bc64a12c	0	0	3	0	0	0	0	0	0	0	0	0
7cf95d283072742faf4a607d224f660d	0	0	0	0	0	0	0	0	0	0	0	9
b2898967bb1ced27df61b6f5ee7bbd86	0	0	0	0	0	0	4	0	0	0	0	0
4a52babcc843775003bc5ca75711343a	0	0	0	0	0	0	0	0	0	0	4	0
74f68066a1f427d898959dcb9938792a	0	0	0	0	0	0	0	7	0	0	0	0

4359b5185c8a929a042b0d642c1cdb6d	0	0	0	0	0	0	0	0	0	0	0	6
701383968fe1f7d6534994cce431be92	0	0	0	0	0	0	0	0	5	0	0	0
d2d1b96bf4657c61480862de7e9e8491	0	0	0	0	0	0	0	0	9	0	0	0
4eeaf0927ab2d0f15961877b2f1a542e	0	0	0	0	0	0	0	0	0	0	0	67
d597851782d2675cf38ec3e8a5a58f47	0	0	0	0	0	40	0	0	0	0	0	0
f5a0617f3f7a8d4de5fc1a1394a91e94	0	0	0	3	0	0	0	0	0	0	0	0
95149d41fc93c9c0887a6c7f0e1e7565	0	0	0	0	15	0	0	0	0	0	0	0
1842cb235531c2e83c51718262edb910	6	0	0	0	0	0	0	0	0	0	0	0
0b9b14b099682c1d1dba68fbac63086a	0	0	0	39	0	0	0	0	0	0	0	0
7f7322b0b93e2127946e4a43138b4cc9	0	0	0	0	0	0	0	0	0	0	0	12
0a490386225cc0db9fb33f6990e0bf07	0	0	0	0	0	0	0	0	0	3	0	0
eed91976305e023f96ff648bbb88ffa2	0	0	0	0	0	0	0	0	0	3	0	0
44f07ba62b6858215fad28e94e1beeda	0	0	0	0	0	6	0	0	0	0	0	0
874ccbc5083977eab2f80752e33a2541	0	68	0	0	0	0	0	0	0	0	0	0

c90617ac9d7c90a3f65037e26b2dcc62	0	0	0	0	0	0	29	0	0	0	0	0
0d87681b76adc41e24d2f3e1e4a8de3b	0	0	0	4	0	0	0	0	0	0	0	0
4b559b03330bb562167efc58700ce168	0	0	0	0	8	0	0	0	0	0	0	0
e18e7735ddc596fe8fb232766b84147c	0	0	0	0	0	2	0	0	0	0	0	0
87208cd1c0da4df55335392a2f4e31a5	0	0	0	2	0	0	0	0	0	0	0	0
b89159f2ad2b2367b3ad6a65ab89ff7b	0	0	0	0	0	0	0	0	0	4	0	0
6318c10fd4d137250edb3954169c7fc7	0	0	0	0	0	3	0	0	0	0	0	0
51f943e97c2849893f98eb67c3414d02	0	0	0	0	0	0	0	0	0	0	5	0
8c8189b993a9d379f9dc7022909f5c4c	0	0	0	0	0	0	0	0	0	0	22	0
f9f0a1d78258c112ac8dc5f0c435f69a	0	0	0	0	0	0	0	0	15	0	0	0
79ef28a829d25bf46a0b84ab1e87ded8	0	0	0	0	0	0	0	0	0	0	3	0
dc45c0e682cffbe4dec44e1b564772d6	0	0	0	28	0	0	0	0	0	0	0	0
42c3636e2002fde16deec8cb7207909a	0	0	0	0	4	0	0	0	0	0	0	0
b9a73385c0e5058c94b806080c21e84f	0	0	0	0	0	0	17	0	0	0	0	0

e8467b2ba2116a1f8f9e0835 93a98355	0	9	0	0	0	0	0	0	0	0	0	0
964d5f2db6a8ecb3c7e3c841 9b40771d	0	0	0	0	0	0	0	0	0	0	9	0
81d274d97a85f1a3746b9180 e5c65a84	0	0	0	0	0	0	0	2	0	0	0	0
79441d11bf46f87b0894ef4fd 1ba6b3f	0	0	0	0	0	0	0	0	0	0	2	0
91325a6b1cca7a2516f909a9 6e90dfe4	3	0	0	0	0	0	0	0	0	0	0	0
08e4600b487b85c21667e23 802301ccc	0	0	0	0	0	0	0	9	0	0	0	0
dd3ecd00036dc12580b5e82 d9d1451ec	0	0	0	0	0	0	0	0	0	0	3	0
e20d9e7889c414a9021642f7 388398d5	7	0	0	0	0	0	0	0	0	0	0	0
a9042f691d91444743e678fc 0fcc287d	0	2	0	0	0	0	0	0	0	0	0	0
88707f12e2f9f21ebd97bbb5 3626ad7a	0	0	0	0	0	0	0	0	0	3	0	0
b8304ed6bf72aecd9accd7de 487a7766	0	0	0	0	8	0	0	0	0	0	0	0
18d408492e3a8e7648f6a61e 73e0d0f5	0	86	0	0	0	203	0	0	0	0	0	0
e999da7ce51c58ffd095ebbf2 a644788	0	0	0	0	0	0	0	0	0	0	7	0
ad23f6304e0c1177cfb80934 5313d79d	0	0	0	0	19	0	0	0	0	0	0	0

8ae4be9a21b408bb0a2e254376f8d05e	0	0	0	3	0	0	0	0	0	0	0	0
4ce66f5d6b7d877366402a447120b382	0	0	0	0	0	0	0	0	0	43	0	0
40738ca03bc355573de2997d94f0af72	0	0	0	0	0	6	0	0	0	0	0	0
938f30b8980616c09ef2ad129cfcfc25	0	0	0	0	0	0	0	0	3	0	0	0
1c43ffecb07e2e75780bd1236bf9ee6a	0	0	0	0	0	0	0	0	0	0	11	0
71539a5a6d9c1edeed17f188800915dd	0	0	0	0	0	4	0	0	0	0	0	0
b6fae710f1ea50a4dd9996eb8530760c	0	0	0	0	0	0	0	0	0	0	0	2
c933e37596a35d336370bedaad2f0bb9	0	0	0	0	0	4	0	0	0	0	0	0
da1ab29ffe5e11b7e2efd63be9e784ba	0	0	0	0	0	0	39	0	0	0	0	0
1396ab57039bc133c120df4f3aa4faf3	0	0	0	8	0	0	0	0	0	0	0	0
9bfed13c34a34e31e5ea1bfc7b48031b	0	28	0	0	0	0	0	0	0	0	0	0
7ba691354276c47fb09cd9ae23ffe9f3	0	0	0	0	0	18	0	0	0	0	0	0
86c057280c5664ba5ad37ee9bc4d2ecd	0	0	0	0	28	0	0	0	0	0	0	0
1eddfe1625979ca371a8aa71d76722b4	0	0	0	0	0	0	0	0	0	0	6	0

34e1215a11f2e81e597507c372a6232f	6	0	0	0	0	0	0	0	0	0	0	0
766dd7d017a36df5ca0b1dca0fc43c6c	0	0	0	0	0	0	0	0	0	17	0	0
5c82ba4c51f264393550a1cd676e1553	0	0	0	0	0	0	0	0	0	0	0	10
5160224a825fe53c5bd1d1321bca46e8	0	0	0	0	0	0	0	0	2	0	0	0
4f2c326eec0aefa5d71d58834ba8ceca	0	0	0	0	0	0	0	0	0	0	3	0
9332f5c881b5a4dd905b8752e6c9e063	0	0	0	0	0	0	0	0	0	2	0	0
59c1d77811b960c0b05b553d6daed042	15	0	0	0	0	0	0	0	0	0	0	0
c626225636fc2afb202bdd1e26586789	0	0	0	0	0	0	0	0	0	0	0	2
d2ce2841d7d5263c4f300d4a2d0c72a1	0	0	3	0	0	0	0	0	0	0	0	0
e1b7d89362d744870ee2c4322a9ad3bc	0	0	0	0	0	0	0	34	0	0	0	0
8c4bf7ed2dae54d2bef6d9c9e64e4d5c	0	0	0	0	0	0	0	0	0	17	0	0
cfeabcb615d338aa914dac6d6d7b659e	0	0	0	0	0	0	0	0	0	0	0	44
da282253ebda41221fe8eda09b331000	0	0	0	0	0	0	0	0	0	0	3	0
8f09c6dffa8e7a3c36806ad5aacb0f40	3	0	0	0	0	0	0	0	0	0	0	0

e586fef16119029d0417f81c60487777	0	0	0	0	0	0	0	0	0	4	0	0
fef6e6e0e1e6f42f8c1f332224072789	0	0	0	0	0	0	0	2	0	0	0	0
f8ccd0b2d69fedf8b224fe0d967d01b6	0	0	0	0	0	0	0	0	4	0	0	0
6dd0755f04f742a2835a85f59b4acaf3	0	0	0	3	0	0	0	0	0	0	0	0
9b07603fe9fd5a07159c4e5a08f03c3d	0	0	0	86	0	0	0	0	0	0	0	0
8e91102526f3a2524ac29fb12de23a7e	0	0	0	0	0	0	0	0	0	0	27	0
bdad69736cdc0e58f37ad27e1b018b12	0	0	0	20	0	0	0	0	0	0	0	0
babff2ba08d93ec385f565b72af0d161	0	0	0	0	0	0	0	0	0	3	0	0
181c7fd34fd2e0c28fe6800609ba25bf	0	0	0	0	0	0	3	0	0	0	0	0
a1535ea8e6ab3c51574c676ccd7e00c9	0	0	0	0	0	6	0	0	0	0	0	0
62c10eae3727d7ba188bc8c9deb5b97b	0	0	0	3	0	0	0	0	0	2	0	0
37b13e3190e51ac18159999220f32cb2	0	0	0	0	0	0	3	0	0	0	0	0
691b24e57b8f5d9ea3172966bd873f03	0	0	5	0	0	0	0	0	0	0	0	0
edf0985c654463e973f1960ced3dbe9c	0	0	0	0	0	0	0	0	0	0	20	0

a21596fc70be4237e0b80758339871b7	0	0	0	0	0	0	0	0	0	0	0	16
741c041590ba23fdd03c67921426a9c1	0	0	0	2	0	0	0	0	0	0	0	0
6585e60d9c55f69cac309957d8cd2b5e	3	0	0	0	0	0	0	0	0	0	0	0
01466f90f61a503f78986911816d183e	0	0	0	0	0	0	0	0	0	0	26	0
cbbab092c70b935136d1f7d60c0135f2	0	0	0	5	0	0	0	0	0	0	0	0
d9ef4a7a6b4f51b0c61e3ec46034003b	0	0	4	0	0	0	0	0	0	0	0	0
6abc84e5b9ca103612c34a0a1800f541	0	0	10	0	0	0	0	0	0	0	0	0
d0217d00fea1b292e33ff0c94bdc0082	3	0	0	0	0	0	0	0	0	0	0	0
f81bdb5985d3ef4d92f73f94ce2db578	0	0	0	0	0	0	0	0	44	0	0	0
30db77625b057879a29e0d79f262661c	61	0	0	0	0	0	0	0	0	0	0	0
4e12728709c6c63ea7b5aa8ef4c018b4	0	18	0	0	0	0	0	0	0	0	0	0
b2bc97aad51285cfb144c2dcc4478a1b	0	0	0	0	0	0	0	0	2	0	0	0
2a031e64acca2545eb1bdf836b9f3d0e	0	0	0	0	0	20	0	0	0	0	0	0
226487007aecaf5b84723fd68e4793eb	0	0	0	0	0	0	0	0	0	0	0	3

9b316e4c67e70f1a2bb316d0684113af	0	47	0	0	0	0	0	0	0	0	0	0
a5a6a8a93132b4c534b8a795bc09e278	0	0	0	0	0	0	63	0	0	0	0	0
3e8c2b65217462327875abd2d3bfa3db	0	0	0	0	0	0	0	10	0	0	0	0
15de745dcf1a75344382c88f258cb2ae	0	0	0	0	0	0	0	0	0	14	0	0
79be868b479ed3fbf6e19f1df41f4ac6	0	0	0	0	0	0	0	0	0	3	0	0
7675989b1952dc1287933a06624e6855	0	0	0	0	0	0	0	0	0	2	0	0
ac536a5f5d6e25c414012a081198cb94	0	0	0	0	0	0	0	0	0	0	6	0
137ee54bd853ea4d0258e8495fe0edbf	0	0	0	0	5	0	0	0	0	0	0	0
41608940cbfd2a613a2c4e50951dfc54	0	0	21	0	0	0	0	0	0	0	0	0
3cc344cb8bc71034716953e1c3bb60a1	0	0	0	0	0	0	0	0	0	0	2	0
d21c07d357f47de7105293d7c93167ce	0	0	0	0	0	0	0	0	0	0	50	0
d1008c0906266724197aa8bf6cb92e22	0	0	0	0	0	0	10	0	0	0	0	0
8091a36e52a23adcee33cda9dee06a22	0	0	0	0	0	0	0	0	0	0	0	11
853a0b6cb9a24c5cdfbbd4e3722de799	0	0	0	0	0	0	0	0	0	0	3	0

a4fbe4e8dc05652a9b58d66e1d341b97	0	0	0	0	0	0	0	0	0	0	0	25
5cc259256cb925803b5a43308eb4d0e8	0	0	0	0	0	0	0	0	3	0	0	0
9e6f2622a6c20fd861a32204dca42db2	0	0	4	0	0	0	0	0	0	0	0	0
f37e4aab8d2d63b52f72852788127ad9	0	0	0	0	0	0	0	0	0	0	22	0
fd6e5a1c3363d972bf709ffa3a65c354	0	0	0	0	0	0	0	2	0	0	0	0
363d9596ad2043e527e7d6961174f38d	0	0	0	0	0	10	0	0	0	0	0	0
63d59d4bc4e500114ee05042e3850632	0	0	0	0	0	2	0	0	0	0	0	0
00e6a1bfc2c1e8a373250b1c4344bce9	0	0	0	0	0	0	0	0	0	9	0	0
4917ed48a6dd17540b7674ab0a0fc2d5	0	0	0	0	0	0	0	0	0	0	0	11
bffbf5c95aee7ca13b317f283a278722	4	0	0	0	0	0	0	0	0	0	0	0
a8cf93bd61283dc052a640e9ff7955f7	0	0	0	0	0	0	0	20	0	0	0	0
f0e8f10141f91dcf8f8adb4200a947a6	0	0	0	0	0	0	0	0	0	5	0	0
e89cc348748edcc0a1bf6b20cc7e617a	0	0	2	0	0	0	0	0	0	0	0	0
296b16e740ed91caab2a16d42a819bcd	5	0	0	0	0	0	0	0	0	0	0	0

f311a52b0a4f2ed4024597abd9d7e1c0	0	0	0	0	0	0	5	0	0	0	0	0
fa1810e94bfb9875b5af0ede90037795	0	0	0	0	0	0	0	0	0	4	0	0
1c4f737ea64300e893c5766da72050f4	0	0	0	0	2	0	0	0	0	0	0	0
8a925f0d6d8b953a96106bd613cd7c7c	0	0	0	0	0	0	0	0	0	0	3	0
1428fa206688a0a26024513f1e22f1fe	0	0	0	0	0	0	0	0	0	3	0	0
4f9ba89189e819f20756ee733e5b6cdb	0	0	0	0	0	2	0	0	0	0	0	0
1c0dbd4a9d22913f12869f66aab192c4	0	0	0	0	0	0	0	0	4	0	0	0
780cf4a690feef09e18ef5545ee89c2a	0	0	0	0	0	6	0	0	0	0	0	0
1b41223c840ab3c819f8b4313b71e273	10	0	0	0	0	0	0	0	0	0	0	0
dde29aaed849c47c817ed62e37f0ce74	0	0	0	0	0	0	0	0	0	12	0	0
a6278c054c4cab12ed42de35da4a4eba	0	0	0	2	0	0	0	0	0	0	0	0
a9325c51bf23bc293a37779a828d848a	0	0	0	0	0	0	3	0	0	0	0	0
01ee7e3046c11445631d838fe72616f7	0	0	0	0	0	0	0	0	0	2	0	0
ba0b59a5114002c305cf24aa3627c21a	0	0	0	0	0	0	0	0	5	0	0	0

c6456cb391422022bbba75b40add8fb8	0	0	0	0	0	6	0	0	0	0	0	0
b2c89254abf82d8dbfb27b6f4a847c43	0	0	0	0	0	5	0	0	0	0	0	0
a54906edb12f8f94b3e779f7bb59a42a	0	0	0	2	0	0	0	0	0	0	0	0
1ebc64cf622e645365ce700c516b2921	2	0	0	0	0	0	0	0	0	0	0	0
2cf71abf2b150fba60028ad79c0aa267	0	0	0	0	0	0	0	0	0	0	3	0
e7bad19820cd06fd5fd6c0c74c4caac5	0	0	0	0	0	0	11	0	0	0	0	0
3962d3590c9b353c8e75d0a57acd3105	0	0	0	0	0	0	0	0	0	0	15	0
cdb4719de55349ff9da9c346d17e9869	0	0	0	0	0	0	0	0	0	0	0	6
a4943524005f47822127d435874bfd99	0	0	0	0	0	0	0	0	0	0	0	3
eb0b9010ad4b82badbc6c778412bc7d3	0	0	0	0	2	0	0	0	0	0	0	0
fe0e57d6eab2f9d91458303685c44dbf	0	0	8	0	0	0	0	0	0	0	0	0
764b46462f6586934aa5c6112f747245	0	0	0	0	0	12	0	0	0	0	0	0
f7d9780350d6e61ca426791fa309ceb6	0	0	0	6	0	0	0	0	0	0	0	0
79ed4b62d9eef0cc41b2fab6363c9a5f	0	0	0	0	0	0	0	0	0	0	0	5

604232e6fc17c1e748381f728445fad9	0	0	0	0	3	0	0	0	0	0	0	0
44665b8df2a79f1bd4a6ad3952403611	0	0	0	0	0	0	0	0	0	0	3	0
23b0dbbd375078c796aed5b8c76a6bba	0	0	0	0	0	4	0	0	0	0	0	0
a1b60a5c2d5e90c065338780b17c21e3	0	0	0	0	0	3	0	0	0	0	0	0
4ad6e83fd455200de94b2860222519e6	0	0	0	0	0	45	0	0	0	0	0	0
fda101c6dd92dd68e51be05035712421	12	0	0	0	0	0	0	0	0	0	0	0
ad4a3627f495bea86028d024449f63f1	0	0	0	0	0	0	0	0	0	3	0	0
849ec3b5b5f206dd57d3a6ae7c7651a5	0	0	0	0	0	0	0	0	2	0	0	0
f88bae792e99f68b6523e8ff96e92c71	0	0	0	0	0	0	0	0	0	3	0	0
97c197e6cf9b5e1d3e94a67e637a9dd6	2	0	0	0	0	0	0	0	0	0	0	0
864eac6f244dd78024451fb19d851e50	0	0	0	2	0	0	0	0	0	0	0	0
b2430d3e26f4d255dc293e47cdd73a5f	0	0	0	0	0	3	0	0	0	0	0	0
062d6d72579c6225d636d5a93942b1b5	0	0	0	0	0	0	0	0	0	0	5	0
0155dd9bc286ef60d8f7a5acc150f6a1	0	0	0	0	0	34	0	0	0	0	0	0

49d66f80208f3f923cfbf2bd5 84b527a	0	0	0	0	0	2	0	0	0	0	0	0
a9ce3b4e9130293ca1bc2d3a cc7f6b08	0	0	0	0	0	0	0	0	0	0	88	0
83cd80feab064169d185c714 ffae3844	0	0	0	0	0	0	0	0	0	0	14	0
4afd3274b017107101275950 8ca5dbc5	0	0	0	0	3	0	0	0	0	0	0	0
f529e609beb5dcd35b85f73d f599e801	0	0	0	0	0	4	0	0	0	0	0	0
f73ec9c3ab051e3c03a2d37ff 8206fdd	0	0	0	0	0	0	0	8	0	0	0	0
f60dd6593475e336d547e7d 05d3c8bfc	0	0	0	0	0	0	0	0	0	0	6	0
44b35bc0dcd43b1b98949a3 b60c7c716	0	0	0	11	0	0	0	0	0	0	0	0
2b758ff7d38195e93c5dc048 b1cd82b5	0	0	0	0	3	0	0	0	0	0	0	0
cc7226f327d7565f18966871 2c5f6634	12	0	0	0	0	0	0	0	0	0	0	0
59df1c0f29c0cfaead23d423a f84f4bd	0	0	0	0	0	0	0	0	0	2	0	0
57c43ae2db8a4c99a294d62e 28a3a168	0	0	10	0	0	0	0	0	0	0	0	0
df44f90b10a47006631e8f23 26d7f36f	0	0	0	0	0	0	0	0	0	0	3	0
f1725d796dbecac89a88de63 033fce29	0	0	0	0	0	0	0	0	0	8	0	0

4c00ba0e3ddd1b20a60d568 1475d1fb1	0	0	0	0	0	0	0	0	0	0	0	4
8e25b0857ea4b27b8ec5875 2c0339354	0	0	0	0	0	0	0	4	0	0	0	0
c4d7f4d866c496be01f71174 1ccf9077	0	0	0	0	0	8	0	0	0	0	0	0
16e0f258334bc6c6c4deafd5 0ce430d1	0	0	0	18	0	0	0	0	0	0	0	0
b4af942369bcdca7c7d8b72c 9205410c	0	0	0	0	13	0	0	0	0	0	0	0
d1e1c2573a30a4fdca902a2a 74066ce7	0	0	0	0	0	0	5	0	0	0	0	0
9bef2885ac751941bdf0999b f71aaa11	0	2	0	0	0	0	0	0	0	0	0	0
28ca1f38ae2bbb99486c0e06 8d8ce74f	0	0	0	0	0	0	0	0	0	0	2	0
1d4adc064992650c2873af98 d11096e5	0	0	0	0	0	14	0	0	0	0	0	0
2b7864afbff7466b7fa1c7b95 7276262	0	0	0	0	0	0	0	0	0	4	0	0
77051c653b844458f0a84a24 5bc34604	0	0	0	0	0	0	0	0	4	0	0	0
a0b25eb56734373a586d4b0 4aee08099	0	0	0	0	0	0	4	0	0	0	0	0
cd7ab60f1c7bc99f19c6adf46 c9f3a24	0	0	0	0	0	0	0	0	0	0	68	0
a5a24ac9cb87ea44a2989c5f 9b233820	0	0	0	0	0	0	0	0	0	0	0	8

159d32c49b28b1a01ecac6b314c032ea	0	0	0	0	0	0	0	0	0	0	0	4
d23566e399591b565aabd9a6e3cda8f8	0	0	0	0	0	433	0	0	0	0	0	0
7b68899e07e64ea09b1ac942cb487a8a	0	0	0	11	0	0	0	0	0	0	0	0
f7d66664ff5db1f53cde708d7be55543	0	0	0	0	0	0	55	0	0	0	0	0
e56593955973ea9673c8ad40c2f26216	0	0	0	0	0	0	0	0	3	0	0	0
44f878486d8899a40b17e772ae428c65	0	0	0	0	128	0	0	0	0	0	0	0
57571642251410ac718fd572c79dccf0	0	0	0	0	0	0	0	0	0	0	51	0
ea07dd35fa11572d5ec45dd949d1c811	6	0	0	0	0	0	0	0	0	0	0	0
a36710efdbab1ae27411f83a81271a43	0	0	2	0	0	0	0	0	0	0	0	0
afa7d9124b0a4053229c6c35156255d6	0	2	0	0	0	0	0	0	0	0	0	0
a948b5292aa5b26df93bc4b773e7b76a	0	0	0	0	0	0	0	0	0	4	0	0
b2c25dc7e029a5647f8345bb67dc19f7	0	0	0	0	0	0	0	0	0	0	24	0
7a0e3514636708513863e23eca9ef23d	0	0	3	0	0	0	0	0	0	0	0	0
3a194da98f638b4120e591d528db29c1	0	0	0	0	7	0	0	0	0	0	0	0

63c3f6ffb1301be2a71c4ee1830f8ce2	0	0	0	0	0	0	0	0	0	24	0	0
936db2ce51b7f7c348983ae80bd4d0c5	0	0	0	3	0	0	0	0	0	0	0	0
6ad42c924811a2017fa4d4408e3a4835	0	0	0	0	0	6	0	0	0	0	0	0
67347cd06d8fccc656ad1ff73054c9b6	0	0	0	0	0	3	0	0	0	0	0	0
a822bd5dbeec96d4b1ab9faff17d7e3d	0	0	0	0	0	0	0	0	0	0	2	0
da280eed43f2012f0d68e5f21a491e41	0	0	0	0	0	0	0	0	0	0	0	7
ca466c241b5840acca136a0994b2e469	0	0	0	0	0	0	0	0	0	2	0	0
7d07cf9a73c428a0918d304ae6024d43	0	0	9	0	0	0	0	0	0	0	0	0
af132e062f680752474c709dc5947474	0	0	0	0	0	8	0	0	0	0	0	0
8eafbd35b8a0cb3bc4f20a04a53ffc20	0	0	0	0	0	0	3	0	0	0	0	0
9b0e25b11a8c8c1da853d22056c3383c	0	0	0	0	0	0	0	0	0	9	0	0
7e31458f86eabcdf34bd2c1b931435d1	0	0	0	0	0	0	3	0	0	0	0	0
23587fe47e607a8150e03f597bb3af11	0	0	36	0	0	0	0	0	0	0	0	0
36348153d805e4cffebc5b5fdc52564f	0	0	0	0	0	0	0	0	0	0	17	0

ab98144a869f01f38226f632 2b35d70b	0	0	0	0	0	2	0	0	0	0	0	0
f74e05c53af927d63602a955 68e22405	0	0	0	38	0	0	0	0	0	0	0	0
d5c773eaf15077cb02818516 2318baa9	0	0	0	0	0	0	0	70	0	0	0	90
22ae080c32b28d344d6471c bfd0b0771	0	0	0	0	0	0	0	0	0	0	4	0
7934810b6e3d17788466418 82f5b39f7	0	0	0	0	0	0	33	0	0	0	0	0
73212e94bc8e3d990627783 9768402ce	0	0	0	5	0	0	0	0	0	0	0	0
f225710231079b474a10a8a5 2f176a8c	0	0	0	0	0	0	0	0	0	4	0	0
61caca7d969d829e3fde8b80 0de35bbf	0	5	0	0	0	0	0	0	0	0	0	0
f60d4315a545fc6a45c43423 b8bf5e1f	0	0	0	0	0	0	0	0	0	0	26	0
225affbf25fa2d4052a8448b8 53c5c29	0	0	0	0	0	0	0	4	0	0	0	0
7b58c27f75b6773c18702676 4b258436	0	0	0	0	0	0	5	0	0	0	0	0
6e64fdc44e05e86371d16cae cefd6b9b	0	0	0	0	0	0	0	7	0	0	0	0
4f04318628bcc0b0c0abcea7 30c9a710	0	0	0	0	0	0	0	0	2	0	0	0
a2dfa12e5e82f9663749aaef7 2aaec7f	0	0	0	0	0	0	0	0	6	0	0	0

c67b3164a921e9a44766b35 21c71ff02	0	6	0	0	6	0	0	0	0	0	0	0
2fc2d58b380f0bc40e35bd78 a14a7798	0	0	0	0	0	0	0	0	0	0	2	0
629b3194f00979fc47d074a3 94d4ba4e	0	0	0	0	0	0	0	56	0	0	0	0
e6350d2b060d9e0677b9012 33907096c	0	0	0	0	178	0	0	0	0	0	0	0
4e1e1dbb36d1991ffa1a9d60 8a326cc5	0	0	0	0	0	0	0	0	0	5	0	0
9a503db119d9cd46a79a30bf 728e1bbf	9	0	0	0	0	0	0	0	0	0	0	0
7cd39792916c827f6b9d5dd4 736791e6	7	0	0	0	0	0	0	0	0	0	0	0
826f88d7dde547acde5b71f7 28b80c84	0	0	0	0	0	13	0	0	0	0	0	0
923d1d505cc065f6ef173b17 d02ba3e2	0	0	0	0	0	0	3	0	0	0	0	0
ab6250d4abc2992ef7665573 5cfe41b8	0	0	0	0	0	0	0	0	0	0	12	0
be38e41c3ae2e0a1358697ef c4c5a286	0	0	0	0	0	0	0	0	0	0	0	47
b94b1f0d86fb5a905a9f1766 a4785de8	331	0	0	0	0	0	0	0	0	0	0	0
3e4762baa3c699dc3a574fce 98401e9b	0	6	0	0	0	0	0	0	0	0	0	0
15eda211cb0a4a10f41b36e3 752e694e	0	0	0	0	0	0	0	0	0	0	2	0

5ba49a1c8cd98357abf88a4535261f76	0	0	0	0	0	0	2	0	0	0	0	0
885bd096e0d4cd3da2650988b3a5f37e	0	0	17	0	0	0	0	0	0	0	0	0
68cd680e07c3c5119bfb59771f5b2045	0	0	0	0	0	0	0	0	0	40	0	0
5edd035156d6bfc024ff2b30a858d9f6	0	0	0	0	0	0	0	0	0	0	11	0
455a83cfe98400b57799aea700861b36	0	0	0	0	0	2	0	0	0	0	0	0
e9540229ae0037236c0336327a89d11c	0	0	0	0	0	0	0	4	0	0	0	0
98cb573528d0bfe1756b103ffd472744	0	0	0	0	0	0	0	0	0	0	3	0
7c11cb04ced8b014ac9b8d321cf50f07	0	0	0	4	0	0	0	0	0	0	0	0
c04a58ad5eef4eff18c31a1c0aee298d	0	0	0	0	0	0	0	3	0	0	0	0
c365a6259b2ef0de1fb95daca3b889d7	0	0	0	2	0	0	0	0	0	0	0	0
5e77850fb9f2976a18d417fa28931b0b	0	0	0	0	0	0	0	0	0	0	0	4
b894490137e21545802a34005b66893f	0	0	0	0	0	3	0	0	0	0	0	0
a3a233b2826f750860846cba2049d67d	0	0	0	0	0	3	0	0	0	0	0	0
c2b0f01c1c2fa380269a3f6c7e4265ed	0	0	0	15	0	0	0	0	0	0	0	0

664371b2bbea7bb021a219d32050e2a2	0	0	0	0	0	0	0	2	0	0	0	0
52e59e2060e530ffb717565d4fbc44cf	0	0	0	0	0	0	0	0	0	5	0	0
a91cdafbd5095ebb24f04da9382cbbb4	0	3	0	0	0	0	0	0	0	0	0	0
7f0da87a3d89fe65f2ca6e7e1ccb3603	0	0	0	0	0	0	3	0	0	0	0	0
35d59a582893fbeb22e875fcb405721e	0	0	0	0	0	4	0	0	0	0	0	0
a9640d2ef2b6120a5261759dbb83045f	0	0	0	0	0	0	2	0	0	0	0	0
599765c7441b7ae0456d4db4f1742c6f	0	0	0	0	0	0	0	0	0	3	0	0
43ecbe03c07838c059d4d8346137d9d7	0	0	0	0	0	0	0	0	0	14	0	0
d23ff6557043bde516f6a44a5f00e170	0	0	0	0	0	12	0	0	0	0	0	0
50fefbd5f392f1b0388669c15cd58211	0	0	3	0	0	0	0	0	0	0	0	0
1063c01d5d172a7ab46c32ff5a0b0ef8	0	0	0	0	0	0	0	0	88	0	0	0
5ed17ed0a406d53a330cc81293d84a81	0	0	3	0	0	0	0	0	0	0	0	0
59c021972b5ce35bb08bd9b3f41eefca	0	13	0	0	0	0	0	0	0	0	0	0
544fdc2f72dcfc1e5f046e9bf23c3c66	0	0	0	0	0	0	0	0	0	0	3	0

9ab919e46d8628c36625824c92bac437	0	0	0	0	0	0	0	0	0	5	0	0
eecef0f68acc6fd685471cb9ad6a8c6b	0	0	0	2	0	0	0	0	0	0	0	0
21d48020f625420c82949541d5e4c5db	0	0	0	0	0	0	0	0	0	0	0	2
efc68027607d495d730728df12402e42	0	0	0	0	0	0	0	0	0	0	3	0
fa7b87e1b3e6aebed6ebaeceee844239	0	0	0	0	0	0	0	0	0	3	0	0
cf439774007f05d1378473db091bf54d	0	0	0	7	0	0	0	0	0	0	0	0
5c84e575a849eae19f319b66e655ed7b	0	0	0	0	0	32	0	0	0	0	0	0
7c3ff67892a1286aee376162b21b636f	0	0	0	0	0	0	0	0	0	0	0	3
c2077fb90966d40e5ce0a2c7619d1bb3	0	0	0	5	0	0	0	0	0	0	0	0
8345a41ee13f3df012a877b06b0ba873	0	7	0	0	0	0	0	0	0	0	0	0
1cd3390aa6f4ff89f91ea2ef7be8d40f	0	0	0	17	0	0	0	0	0	0	0	0
968166ce6bf8c4ca80e7e857de323f90	0	0	0	2	0	0	0	0	0	0	0	0
7d0b9aa7575b7bc7fa93a2643ba3cd73	0	0	0	0	0	0	0	0	0	0	0	4
54cb9728d9f505bc30aee003c30494f9	0	0	0	0	0	0	6	0	0	0	0	0

ea52eba21ede53c5b98b0e5 44b82d60c	0	0	0	0	0	0	0	0	0	27	0	0
0338625d0b1378f7452a9072 a368b18a	0	0	0	0	0	0	0	9	0	0	0	0
b00a4382d4f55795153801da 477b8f07	0	0	0	0	0	0	0	0	0	0	0	212
f0b6a0f02e07480559dabeeb fdfc66e5	0	0	0	0	0	0	0	0	13	0	0	0
faac961198d20ed036d39686 6a3a1ac8	0	0	0	3	0	0	0	0	0	0	0	0
