# Supplementary material for: The impact of OsASR5 on rice microbiome: Implications for nutrient cycling and stress response
Source: Genet Mol Biol. 2026 Oct 26;49(Suppl 3):e20250064. doi: 10.1590/1678-4685-GMB-2025-0064 (PMC13720868; doi:10.1590/1678-4685-GMB-2025-0064)
Supplement: Supplementary file 4 [file 1415-4757-GMB-49-s3-e20250064-s4.pdf]

Genetics and Molecular Biology

https://doi.org/10.1590/1678-4685-GMB-2025-0064

Supplementary Material to "The impact of OsASR5 on rice microbiome: Implications for nutrient cycling and stress response"

Table S4 - Bacterial phylum abundance per sample from leaf microbiome.

Bacteria taxonomy	L1_ASR5i	L2_ASR5i	L3_ASR5i	L4_ASR5i	L5_ASR5i	L6_ASR5i	L1_WT	L2_WT	L3_WT	L4_WT	L5_WT	L6_WT
d__Bacteria;p__Firmicutes	564	243	270	97	38	48	43	23	22	205	16	19
d__Bacteria;p__Proteobacteria	655	1580	1247	1252	205	175	455	227	543	715	453	173
d__Bacteria;__	4	21	28	0	4	0	29	12	9	21	4	5
**d__Bacteria;p__Acidobacteriota***	**52**	**29**	**49**	**34**	**6**	**24**	**0**	**0**	**0**	**7**	**0**	**0**
d__Bacteria;p__Cyanobacteria	0	26	9	0	0	0	0	0	0	12	9	0
d__Bacteria;p__Bacteroidota	31	17	31	35	4	8	11	3	36	13	22	0
d__Bacteria;p__Bdellovibrionota	0	0	0	17	0	0	2	0	0	0	0	0
d__Bacteria;p__Deinococcota	0	0	11	8	0	0	2	0	10	10	0	0
d__Bacteria;p__Actinobacteriota	40	48	58	52	13	7	36	13	8	24	23	2
**d__Bacteria;p__Desulfobacterota***	**33**	**9**	**29**	**15**	**5**	**6**	**0**	**0**	**0**	**0**	**0**	**0**
d__Bacteria;p__Verrucomicrobiota	4	6	9	0	0	3	0	0	0	0	0	0
d__Archaea;p__Halobacterota	2	0	0	0	0	0	0	0	0	0	0	0
d__Bacteria;p__Myxococcota	0	8	7	11	7	0	61	80	9	12	7	14
d__Bacteria;p__Chloroflexi	8	10	0	19	0	0	2	0	7	0	0	0
Unassigned;__	3	6	5	0	0	0	10	6	11	0	0	0

Bacteria taxonomy	L1_ASR5i	L2_ASR5i	L3_ASR5i	L4_ASR5i	L5_ASR5i	L6_ASR5i	L1_WT	L2_WT	L3_WT	L4_WT	L5_WT	L6_WT
d__Bacteria;p__FCPU426	8	0	0	0	0	0	0	0	0	0	0	0
d__Eukaryota;__	0	0	4	7	0	0	0	0	0	0	0	0
d__Eukaryota;p__Protalveolata	0	0	0	0	2	0	0	0	0	0	0	0
d__Bacteria;p__Gemmatimonadota	14	0	6	0	0	2	0	0	0	0	0	0
d__Bacteria;p__Patescibacteria	7	0	0	7	0	0	0	0	0	0	0	0
d__Bacteria;p__Fusobacteriota	0	0	0	0	0	0	0	0	3	0	0	0
	1425	2003	1763	1554	284	273	651	364	658	1019	534	213
