# Supplementary material for: The impact of OsASR5 on rice microbiome: Implications for nutrient cycling and stress response
Source: Genet Mol Biol. 2026 Oct 26;49(Suppl 3):e20250064. doi: 10.1590/1678-4685-GMB-2025-0064 (PMC13720868; doi:10.1590/1678-4685-GMB-2025-0064)
Supplement: Supplementary file 5 [file 1415-4757-GMB-49-s3-e20250064-s5.pdf]

Genetics and Molecular Biology

https://doi.org/10.1590/1678-4685-GMB-2025-0064

Supplementary Material to "The impact of OsASR5 on rice microbiome: Implications for nutrient cycling and stress response"

Table S5 - Bacterial family abundance per sample from leaf microbiome.

Bacteria taxonomy	L1_ASR5i	L2_ASR5i	L3_ASR5i	L4_ASR5i	L5_ASR5i	L6_ASR5i	L1_WT	L2_WT	L3_WT	L4_WT	L5_WT	L6_WT
d__Bacteria;p__Firmicutes;c__Bacilli;o__Staphylococcales;f__Staphylococcaceae	16	21	11	0	3	6	18	0	3	43	6	4
d__Bacteria;p__Proteobacteria;c__Gammaproteobacteria;o__Burkholderiales;f__Burkholderiaceae	6	0	21	6	5	4	5	14	12	30	0	0
d__Bacteria;p__Firmicutes;c__Bacilli;o__Alicyclobacillales;f__Alicyclobacillaceae	0	0	0	15	0	0	0	4	0	81	0	0
d__Bacteria;p__Proteobacteria;c__Gammaproteobacteria;o__Salinisphaerales;f__Solimonadaceae	45	0	0	12	0	6	0	0	0	20	0	0
d__Bacteria;__;__;__;__	4	21	28	0	4	0	29	12	9	21	4	5
d__Bacteria;p__Proteobacteria;c__Alphaproteobacteria;o__Sphingomonadales;f__Sphingomonadaceae	13	29	41	47	12	4	32	12	16	43	45	9
d__Bacteria;p__Acidobacteriota;c__Acidobacteriae;o__Acidobacteriales;f__uncultured	2	24	0	0	0	5	0	0	0	0	0	0
**d__Bacteria;p__Proteobacteria;c__Gammaproteobacteria;o__Xanthomonadales;f__Rhodanobacteraceae***	**25**	**5**	**17**	**14**	**0**	**0**	**0**	**0**	**0**	**0**	**0**	**0**
d__Bacteria;p__Proteobacteria;c__Gammaproteobacteria;o__Enterobacterales;f__Enterobacteriaceae	30	80	378	363	94	55	16	46	69	11	61	0
d__Bacteria;p__Firmicutes;c__Bacilli;o__Bacillales;f__Bacillaceae	290	87	107	19	9	29	11	6	13	22	0	0
d__Bacteria;p__Proteobacteria;c__Gammaproteobacteria;o__Burkholderiales;f__Oxalobacteraceae	9	29	73	36	0	0	0	0	130	5	4	0
d__Bacteria;p__Firmicutes;c__Clostridia;o__Clostridia;f__Gracilibacteraceae	4	19	10	0	0	0	0	0	0	0	0	0

Bacteria taxonomy	L1_A SR5i	L2_A SR5i	L3_A SR5i	L4_A SR5i	L5_A SR5i	L6_A SR5i	L1_ WT	L2_ WT	L3_ WT	L4_ WT	L5_ WT	L6_ WT
d__Bacteria;p__Proteobacteria;c__Alphaproteobacteria;o__Rhizobiales;f__Beijerinckiaceae	9	10	42	10	6	0	9	8	10	50	5	0
d__Bacteria;p__Cyanobacteria;c__Cyanobacteriia;o__Cyanobacteriales;f__Nostocaceae	0	0	5	0	0	0	0	0	0	12	9	0
**d__Bacteria;p__Firmicutes;c__Clostridia;o__Clostridiales;f__Oxobacteraceae***	**3**	**0**	**9**	**4**	**4**	**0**	**0**	**0**	**0**	**0**	**0**	**0**
d__Bacteria;p__Proteobacteria;c__Alphaproteobacteria;o__Rhodobacterales;f__Rhodobacteraceae	0	24	22	0	0	0	10	3	0	4	0	0
d__Bacteria;p__Proteobacteria;c__Gammaproteobacteria;o__Enterobacterales;__	0	888	0	0	0	0	8	0	58	0	0	10
d__Bacteria;p__Proteobacteria;c__Gammaproteobacteria;o__Burkholderiales;f__Methylophilaceae	6	0	0	0	0	0	0	0	0	0	0	0
**d__Bacteria;p__Proteobacteria;c__Gammaproteobacteria;o__Burkholderiales;__***	**53**	**0**	**30**	**44**	**15**	**24**	**0**	**0**	**0**	**0**	**0**	**0**
**d__Bacteria;p__Firmicutes;c__Clostridia;o__Clostridia;f__Hungateiclostridiaceae***	**8**	**6**	**0**	**0**	**3**	**2**	**0**	**0**	**0**	**0**	**0**	**0**
d__Bacteria;p__Bacteroidota;c__Bacteroidia;o__Chitinophagales;f__Chitinophagaceae	8	3	19	12	4	2	3	0	24	8	15	0
d__Bacteria;p__Firmicutes;c__Bacilli;o__Paenibacillales;f__Paenibacillaceae	19	11	2	0	0	0	5	0	0	5	0	0
d__Bacteria;p__Bdellovibrionota;c__Bdellovibrionia;o__Bdellovibrionales;f__Bdellovibrionaceae	0	0	0	17	0	0	2	0	0	0	0	0
d__Bacteria;p__Deinococcota;c__Deinococci;o__Deinococcales;f__Trueperaceae	0	0	11	8	0	0	0	0	0	0	0	0
d__Bacteria;p__Actinobacteriota;c__Acidimicrobiia;o__uncultured;f__uncultured	0	0	3	0	0	0	0	0	0	0	0	0
d__Bacteria;p__Desulfobacterota;c__Syntrophia;o__Syntrophales;f__Syntrophaceae	0	0	4	0	0	0	0	0	0	0	0	0
d__Bacteria;p__Proteobacteria;c__Alphaproteobacteria;o__Acetobacterales;f__Acetobacteraceae	9	4	7	7	0	0	6	8	4	13	0	0
d__Bacteria;p__Firmicutes;c__Clostridia;o__Clostridiales;f__Clostridiaceae	48	28	51	22	9	2	3	9	2	28	8	4
d__Bacteria;p__Bacteroidota;c__Bacteroidia;o__Cytophagales;f__Amoebophilaceae	2	0	0	13	0	0	0	0	0	0	0	0
d__Bacteria;p__Proteobacteria;c__Gammaproteobacteria;o__Pseudomonadales;f__Moraxellaceae	15	36	53	30	0	0	0	0	4	29	8	0
d__Bacteria;p__Actinobacteriota;c__Actinobacteria;o__Micrococcales;f__Micrococcaceae	0	3	0	0	0	0	0	0	0	9	0	0
d__Bacteria;p__Actinobacteriota;c__Actinobacteria;o__Corynebacteriales;f__Corynebacteriaceae	12	5	0	0	6	2	4	11	3	0	0	0

Bacteria taxonomy	L1_A SR5i	L2_A SR5i	L3_A SR5i	L4_A SR5i	L5_A SR5i	L6_A SR5i	L1_ WT	L2_ WT	L3_ WT	L4_ WT	L5_ WT	L6_ WT
d__Bacteria;p__Proteobacteria;c__Alphaproteobacteria;o__Rhizobiales;f__Xanthobacteraceae	26	43	44	0	2	4	8	0	0	17	9	4
d__Bacteria;p__Deinococcota;c__Deinococci;o__Deinococcales;f__Deinococcaceae	0	0	0	0	0	0	2	0	10	10	0	0
d__Bacteria;p__Verrucomicrobiota;c__Verrucomicrobiae;o__Pedosphaerales;f__Pedosphaeraceae	0	6	9	0	0	3	0	0	0	0	0	0
d__Bacteria;p__Proteobacteria;c__Alphaproteobacteria;__;__	10	3	0	0	0	0	4	4	0	0	0	0
**d__Bacteria;p__Acidobacteriota;c__Acidobacteriae;o__Acidobacteriales;f__Acidobacteriaceae_(Subgroup_1)***	**13**	**2**	**17**	**15**	**0**	**8**	**0**	**0**	**0**	**7**	**0**	**0**
d__Bacteria;p__Actinobacteriota;c__Actinobacteria;o__Pseudonocardiales;f__Pseudonocardiaceae	3	0	2	0	0	0	8	0	5	0	11	0
d__Archaea;p__Halobacterota;c__Methanocellia;o__Methanocellales;f__Methanocellaceae	2	0	0	0	0	0	0	0	0	0	0	0
d__Bacteria;p__Bacteroidota;c__Bacteroidia;o__Bacteroidales;__	16	0	8	0	0	4	0	0	0	0	0	0
d__Bacteria;p__Proteobacteria;c__Alphaproteobacteria;o__Elsterales;f__uncultured	0	15	0	15	0	0	0	0	0	0	0	6
d__Bacteria;p__Bacteroidota;c__Bacteroidia;o__Cytophagales;f__Hymenobacteraceae	0	0	4	3	0	0	8	3	0	0	7	0
d__Bacteria;p__Myxococcota;c__Polyangia;o__Polyangiales;__	0	0	0	0	7	0	0	0	0	0	7	11
**d__Bacteria;p__Proteobacteria;c__Gammaproteobacteria;o__Burkholderiales;f__Rhodocyclaceae***	**38**	**7**	**11**	**35**	**0**	**21**	**0**	**0**	**0**	**0**	**0**	**0**
d__Bacteria;p__Actinobacteriota;c__Actinobacteria;o__Frankiales;f__Acidothermaceae	0	3	4	10	3	0	0	0	0	0	0	0
**d__Bacteria;p__Desulfobacterota;c__Desulfuromonadia;o__Geobacterales;f__Geobacteraceae***	**26**	**9**	**10**	**11**	**5**	**6**	**0**	**0**	**0**	**0**	**0**	**0**
d__Bacteria;p__Proteobacteria;c__Gammaproteobacteria;o__Gammaproteobacteria_Incertae_Sedis;f__Unknown_Family	0	4	9	0	2	0	0	0	0	0	0	0
d__Bacteria;p__Acidobacteriota;c__Acidobacteriae;__;__	2	0	0	0	0	0	0	0	0	0	0	0
d__Bacteria;p__Proteobacteria;c__Alphaproteobacteria;o__Rhizobiales;f__Rhizobiaceae	315	359	404	572	62	48	64	117	188	457	299	144
d__Bacteria;p__Actinobacteriota;c__Actinobacteria;o__Streptomycetales;f__Streptomycetaceae	16	6	11	0	0	0	0	0	0	0	0	0
d__Bacteria;p__Chloroflexi;c__Chloroflexia;o__Thermomicrobiales;f__JG30-KF-CM45	0	0	0	0	0	0	2	0	0	0	0	0
d__Bacteria;p__Bacteroidota;c__Bacteroidia;o__Sphingobacteriales;f__Sphingobacteriaceae	0	9	0	0	0	0	0	0	12	0	0	0

Bacteria taxonomy	L1_A SR5i	L2_A SR5i	L3_A SR5i	L4_A SR5i	L5_A SR5i	L6_A SR5i	L1_ WT	L2_ WT	L3_ WT	L4_ WT	L5_ WT	L6_ WT
d__Bacteria;p__Firmicutes;c__Clostridia;o__Peptostreptococcales-Tissierellales;f__Peptostreptococcaceae	8	4	11	0	0	0	3	0	0	8	0	0
Unassigned;__;__;__;__	3	6	5	0	0	0	10	6	11	0	0	0
d__Bacteria;p__Actinobacteriota;c__Actinobacteria;o__Propionibacteriales;f__Nocardioidaceae	0	0	0	0	0	0	0	0	0	0	2	0
d__Bacteria;p__Firmicutes;c__Clostridia;__;__	0	6	0	0	0	0	0	0	0	0	0	0
d__Bacteria;p__Firmicutes;c__Clostridia;o__Peptostreptococcales-Tissierellales;f__Peptostreptococcales-Tissierellales	11	6	15	0	0	0	3	0	0	0	0	0
d__Bacteria;p__Acidobacteriota;c__Holophagae;o__Holophagales;f__Holophagaceae	14	0	16	0	0	4	0	0	0	0	0	0
d__Bacteria;p__Actinobacteriota;c__Actinobacteria;o__Micrococcales;f__Dermacoccaceae	0	0	0	0	0	0	0	0	0	10	0	0
d__Bacteria;p__Chloroflexi;c__TK10;o__TK10;f__TK10	0	0	0	5	0	0	0	0	7	0	0	0
d__Bacteria;p__Actinobacteriota;c__Actinobacteria;o__Frankiales;f__Frankiaceae	0	0	0	11	0	0	0	0	0	0	0	0
d__Bacteria;p__Actinobacteriota;c__Actinobacteria;o__Frankiales;__	0	0	10	0	0	0	0	0	0	0	0	0
d__Bacteria;p__Proteobacteria;c__Alphaproteobacteria;o__Elsterales;__	6	0	0	7	0	0	0	0	0	0	0	0
d__Bacteria;p__Proteobacteria;c__Alphaproteobacteria;o__Defluviicoccales;f__Defluviicoccaceae	0	0	0	0	0	0	2	0	0	0	0	0
d__Bacteria;p__Firmicutes;c__Desulfitobacteriia;o__Desulfitobacteriales;f__Desulfitobacteriaceae	12	0	5	0	2	2	0	0	0	0	0	0
d__Bacteria;p__Acidobacteriota;c__Acidobacteriae;o__Solibacterales;f__Solibacteraceae	0	3	0	19	0	0	0	0	0	0	0	0
d__Bacteria;p__Proteobacteria;c__Alphaproteobacteria;o__Micropepsales;f__Micropepsaceae	0	8	0	0	0	0	0	0	0	0	0	0
d__Bacteria;p__Proteobacteria;c__Alphaproteobacteria;o__Rhizobiales;__	0	0	0	0	0	2	0	0	0	0	0	0
d__Bacteria;p__Desulfobacterota;c__Desulfuromonadia;o__Geobacterales;__	0	0	11	4	0	0	0	0	0	0	0	0
d__Bacteria;p__Acidobacteriota;c__Acidobacteriae;o__Bryobacterales;f__Bryobacteraceae	9	0	0	0	6	0	0	0	0	0	0	0
d__Bacteria;p__Proteobacteria;c__Gammaproteobacteria;o__Burkholderiales;f__Comamonadaceae	11	11	27	18	0	0	7	0	21	0	11	0
d__Bacteria;p__Proteobacteria;c__Gammaproteobacteria;o__Xanthomonadales;f__Xanthomonadaceae	0	4	0	0	0	0	19	0	0	0	8	0
d__Bacteria;p__Acidobacteriota;c__Acidobacteriae;o__Acidobacteriales;__	4	0	5	0	0	0	0	0	0	0	0	0

Bacteria taxonomy	L1_A SR5i	L2_A SR5i	L3_A SR5i	L4_A SR5i	L5_A SR5i	L6_A SR5i	L1_ WT	L2_ WT	L3_ WT	L4_ WT	L5_ WT	L6_ WT
d__Bacteria;p__Chloroflexi;c__Anaerolineae;o__Anaerolineales;f__Anaerolineaceae	0	2	0	0	0	0	0	0	0	0	0	0
d__Bacteria;p__Proteobacteria;c__Gammaproteobacteria;o__Burkholderiales;f__SC-I-84	11	0	10	0	0	5	0	0	0	0	0	0
d__Bacteria;p__Firmicutes;c__Bacilli;o__Aneurinibacillales;f__Aneurinibacillaceae	82	22	17	32	3	4	0	0	0	5	0	0
d__Bacteria;p__Proteobacteria;c__Gammaproteobacteria;o__Pseudomonadales;f__Pseudomonadaceae	2	12	13	2	0	0	239	0	31	14	0	0
d__Bacteria;p__Firmicutes;c__Bacilli;o__Bacillales;f__Planococcaceae	8	11	0	0	0	0	0	0	0	13	0	0
d__Bacteria;p__Proteobacteria;c__Gammaproteobacteria;o__Diplorickettsiales;f__Diplorickettsiaceae	0	0	0	4	0	2	0	0	0	8	0	0
d__Bacteria;p__FCPU426;c__FCPU426;o__FCPU426;f__FCPU426	8	0	0	0	0	0	0	0	0	0	0	0
d__Bacteria;p__Cyanobacteria;c__Cyanobacteriia;o__Cyanobacteriales;f__Phormidiaceae	0	19	0	0	0	0	0	0	0	0	0	0
d__Bacteria;p__Actinobacteriota;c__Actinobacteria;o__Corynebacteriales;f__Dietziaceae	0	2	0	0	0	0	5	0	0	0	0	0
d__Bacteria;p__Myxococcota;c__Polyangia;o__Haliangiales;f__Haliangiaceae	0	0	0	0	0	0	0	3	0	0	0	0
d__Bacteria;p__Proteobacteria;c__Alphaproteobacteria;o__Caulobacterales;f__Caulobacteraceae	0	0	35	6	0	0	13	0	0	0	3	0
d__Bacteria;p__Myxococcota;c__bacteriap25;o__bacteriap25;f__bacteriap25	0	0	0	0	0	0	0	0	0	0	0	3
d__Bacteria;p__Firmicutes;c__Clostridia;o__Lachnospirales;f__Lachnospiraceae	7	7	0	0	0	0	0	0	4	0	0	0
d__Bacteria;p__Myxococcota;c__Polyangia;o__Polyangiales;f__Phaselicystidaceae	0	0	7	0	0	0	0	3	0	0	0	0
d__Bacteria;p__Proteobacteria;c__Gammaproteobacteria;o__Methylococcales;f__Methylomonadaceae	4	0	6	0	7	0	0	0	0	0	0	0
d__Bacteria;p__Actinobacteriota;c__Acidimicrobiia;o__Microtrichales;f__Ilumatobacteraceae	0	0	0	0	0	2	0	0	0	0	2	0
d__Bacteria;p__Firmicutes;c__Clostridia;o__Peptostreptococcales-Tissierellales;f__Anaerovoracaceae	2	0	0	0	0	0	0	0	0	0	0	0
d__Bacteria;p__Chloroflexi;c__Ktedonobacteria;o__Ktedonobacterales;f__JG30-KF-AS9	0	8	0	0	0	0	0	0	0	0	0	0
d__Bacteria;p__Proteobacteria;c__Alphaproteobacteria;o__Paracaedibacterales;f__Paracaedibacteraceae	0	0	0	0	0	0	2	8	0	0	0	0
d__Bacteria;p__Proteobacteria;c__Gammaproteobacteria;o__KF-JG30-C25;f__KF-JG30-C25	0	4	0	0	0	0	0	0	0	0	0	0

Bacteria taxonomy	L1_A SR5i	L2_A SR5i	L3_A SR5i	L4_A SR5i	L5_A SR5i	L6_A SR5i	L1_ WT	L2_ WT	L3_ WT	L4_ WT	L5_ WT	L6_ WT
d__Bacteria;p__Firmicutes;c__Bacilli;o__Entomoplasmatales;f__Entomoplasmatales	0	0	0	0	0	0	0	0	0	0	2	6
d__Bacteria;p__Myxococcota;c__Polyangia;o__Polyangiales;f__Polyangiaceae	0	0	0	11	0	0	0	74	9	0	0	0
d__Bacteria;p__Acidobacteriota;c__Thermoanaerobaculia;o__Thermoanaerobaculales;f__Thermoanaerobaculaceae	0	0	8	0	0	0	0	0	0	0	0	0
d__Bacteria;p__Firmicutes;c__Bacilli;o__Lactobacillales;f__Carnobacteriaceae	2	0	0	0	0	0	0	0	0	0	0	0
d__Bacteria;p__Chloroflexi;c__AD3;o__AD3;f__AD3	0	0	0	9	0	0	0	0	0	0	0	0
d__Bacteria;p__Firmicutes;c__Thermoanaerobacteria;o__Thermoanaerobacterales;f__SRB2	0	0	0	0	0	3	0	0	0	0	0	0
d__Bacteria;p__Proteobacteria;c__Alphaproteobacteria;o__uncultured;f__uncultured	0	0	0	4	0	0	0	0	0	0	0	0
d__Bacteria;p__Bacteroidota;c__Bacteroidia;o__Cytophagales;f__Microscillaceae	0	5	0	0	0	0	0	0	0	0	0	0
d__Bacteria;p__Proteobacteria;c__Alphaproteobacteria;o__Rhizobiales;f__Rhizobiales_Incertae_Sedis	0	0	4	0	0	0	0	0	0	0	0	0
d__Eukaryota;__;__;__;__	0	0	4	7	0	0	0	0	0	0	0	0
d__Bacteria;p__Verrucomicrobiota;c__Verrucomicrobiae;o__Chthoniobacterales;f__Chthoniobacteraceae	4	0	0	0	0	0	0	0	0	0	0	0
d__Bacteria;p__Chloroflexi;c__Ktedonobacteria;o__Ktedonobacterales;f__Ktedonobacteraceae	2	0	0	5	0	0	0	0	0	0	0	0
d__Bacteria;p__Firmicutes;c__Bacilli;o__Thermoactinomycetales;f__Thermoactinomycetaceae	0	7	0	0	0	0	0	0	0	0	0	0
d__Bacteria;p__Bacteroidota;c__Bacteroidia;o__Bacteroidales;f__Porphyromonadaceae	0	0	0	0	0	0	0	0	0	5	0	0
d__Bacteria;p__Proteobacteria;c__Gammaproteobacteria;__;__	0	5	0	20	0	0	0	0	0	0	0	0
d__Bacteria;p__Desulfobacterota;c__Desulfovibrionia;o__Desulfovibrionales;f__Desulfovibrionaceae	3	0	4	0	0	0	0	0	0	0	0	0
d__Bacteria;p__Actinobacteriota;c__Actinobacteria;o__Micrococcales;f__Microbacteriaceae	0	0	13	0	0	0	4	0	0	5	0	0
d__Bacteria;p__Proteobacteria;c__Gammaproteobacteria;o__Burkholderiales;f__Alcaligenaceae	0	0	0	0	0	0	0	0	0	14	0	0
d__Bacteria;p__Proteobacteria;c__Alphaproteobacteria;o__Rickettsiales;f__Rickettsiaceae	0	0	0	0	0	0	8	7	0	0	0	0
d__Bacteria;p__Cyanobacteria;c__Sericytochromatia;o__Sericytochromatia;f__Sericytochromatia	0	5	0	0	0	0	0	0	0	0	0	0

Bacteria taxonomy	L1_A SR5i	L2_A SR5i	L3_A SR5i	L4_A SR5i	L5_A SR5i	L6_A SR5i	L1_ WT	L2_ WT	L3_ WT	L4_ WT	L5_ WT	L6_ WT
d__Bacteria;p__Acidobacteriota;c__Acidobacteriae;o__Acidobacteriales;f__Koribacteraceae	8	0	0	0	0	7	0	0	0	0	0	0
d__Eukaryota;p__Protalveolata;c__Syndiniales;o__Syndiniales;f__Syndiniales	0	0	0	0	2	0	0	0	0	0	0	0
d__Bacteria;p__Actinobacteriota;c__Actinobacteria;o__Catenulisporales;f__Catenulisporaceae	2	0	0	0	0	0	0	0	0	0	0	0
d__Bacteria;p__Firmicutes;c__Clostridia;o__Clostridiales;__	3	0	14	0	0	0	0	0	0	0	0	0
d__Bacteria;p__Proteobacteria;c__Gammaproteobacteria;o__Burkholderiales;f__Neisseriaceae	12	0	0	0	0	0	0	0	0	0	0	0
d__Bacteria;p__Firmicutes;c__Bacilli;o__Lactobacillales;f__Aerococcaceae	0	0	2	0	0	0	0	0	0	0	0	0
d__Bacteria;p__Bacteroidota;c__Bacteroidia;o__Bacteroidales;f__SB-5	0	0	0	0	0	2	0	0	0	0	0	0
d__Bacteria;p__Actinobacteriota;c__Actinobacteria;o__Propionibacteriales;f__Propionibacteriaceae	0	0	0	2	0	0	0	2	0	0	0	0
d__Bacteria;p__Actinobacteriota;c__Actinobacteria;o__Corynebacteriales;f__Mycobacteriaceae	0	0	2	0	0	0	0	0	0	0	0	0
d__Bacteria;p__Firmicutes;c__Clostridia;o__Oscillospirales;f__Ruminococcaceae	0	0	0	5	0	0	0	0	0	0	0	0
d__Bacteria;p__Gemmatimonadota;c__Gemmatimonadetes;o__Gemmatimonadales;f__Gemmatimonadaceae	14	0	6	0	0	2	0	0	0	0	0	0
d__Bacteria;p__Actinobacteriota;c__Actinobacteria;o__Micrococcales;f__Cellulomonadaceae	0	0	0	0	0	0	8	0	0	0	8	0
d__Bacteria;p__Actinobacteriota;c__Thermoleophilia;o__Solirubrobacterales;f__Solirubrobacteraceae	0	0	0	0	0	0	0	0	0	0	0	2
d__Bacteria;p__Patescibacteria;c__Parcubacteria;__;__	4	0	0	0	0	0	0	0	0	0	0	0
d__Bacteria;p__Actinobacteriota;c__Actinobacteria;o__Micrococcales;f__Dermatophilaceae	0	0	0	0	0	0	7	0	0	0	0	0
d__Bacteria;p__Actinobacteriota;c__Acidimicrobiia;o__IMCC26256;f__IMCC26256	0	11	0	5	0	0	0	0	0	0	0	0
d__Bacteria;p__Actinobacteriota;c__Thermoleophilia;o__Gaiellales;f__uncultured	3	0	13	0	0	0	0	0	0	0	0	0
d__Bacteria;p__Cyanobacteria;c__Cyanobacteriia;o__Cyanobacteriales;f__Chroococcidiopsaceae	0	2	4	0	0	0	0	0	0	0	0	0
d__Bacteria;p__Firmicutes;c__Clostridia;o__Oscillospirales;f__Oscillospiraceae	0	0	9	0	0	0	0	0	0	0	0	0
d__Bacteria;p__Chloroflexi;c__Anaerolineae;o__SJA-15;f__SJA-15	6	0	0	0	0	0	0	0	0	0	0	0

Bacteria taxonomy	L1_A SR5i	L2_A SR5i	L3_A SR5i	L4_A SR5i	L5_A SR5i	L6_A SR5i	L1_ WT	L2_ WT	L3_ WT	L4_ WT	L5_ WT	L6_ WT
d__Bacteria;p__Patescibacteria;c__Parcubacteria;o__Candidatus_Moranbacteria;f__Candidatus_Moranbacteria	3	0	0	0	0	0	0	0	0	0	0	0
d__Bacteria;p__Firmicutes;c__Bacilli;__;__	4	0	0	0	0	0	0	4	0	0	0	0
d__Bacteria;p__Patescibacteria;c__Parcubacteria;o__Candidatus_Zambryskibacteria;f__Candidatus_Zambryskibacteria	0	0	0	7	0	0	0	0	0	0	0	0
d__Bacteria;p__Myxococcota;c__Myxococcia;o__Myxococcales;f__Myxococcaceae	0	8	0	0	0	0	61	0	0	0	0	0
d__Bacteria;p__Bacteroidota;c__Kryptonia;o__Kryptoniales;f__Kryptoniaceae	0	0	0	7	0	0	0	0	0	0	0	0
d__Bacteria;p__Firmicutes;c__Bacilli;o__Bacillales;__	10	0	0	0	0	0	0	0	0	0	0	0
d__Bacteria;p__Firmicutes;c__Bacilli;o__Lactobacillales;f__Streptococcaceae	0	8	7	0	5	0	0	0	0	0	0	0
d__Bacteria;p__Firmicutes;c__Desulfotomaculia;o__Desulfotomaculales;f__Desulfotomaculales	6	0	0	0	0	0	0	0	0	0	0	0
d__Bacteria;p__Desulfobacterota;c__Syntrophobacteria;o__Syntrophobacterales;f__Syntrophobacteraceae	4	0	0	0	0	0	0	0	0	0	0	0
d__Bacteria;p__Actinobacteriota;c__Actinobacteria;o__Actinomycetales;f__Actinomycetaceae	4	0	0	0	0	0	0	0	0	0	0	0
d__Bacteria;p__Actinobacteriota;c__Coriobacteriia;o__OPB41;f__OPB41	0	0	0	0	0	3	0	0	0	0	0	0
d__Bacteria;p__Acidobacteriota;c__Acidobacteriae;o__AKIW659;f__AKIW659	0	0	3	0	0	0	0	0	0	0	0	0
d__Bacteria;p__Firmicutes;c__Clostridia;o__Clostridiales;f__Caloramatoraceae	7	0	0	0	0	0	0	0	0	0	0	0
d__Bacteria;p__Firmicutes;c__Bacilli;o__Lactobacillales;f__Lactobacillaceae	0	0	0	0	0	0	0	0	0	0	0	5
d__Bacteria;p__Firmicutes;__;__;__	12	0	0	0	0	0	0	0	0	0	0	0
d__Bacteria;p__Actinobacteriota;c__Actinobacteria;o__Frankiales;f__Geodermatophilaceae	0	18	0	0	0	0	0	0	0	0	0	0
d__Bacteria;p__Actinobacteriota;c__Actinobacteria;o__Corynebacteriales;f__Nocardiaceae	0	0	0	14	0	0	0	0	0	0	0	0
d__Bacteria;p__Firmicutes;c__Clostridia;o__Christensenellales;f__Christensenellaceae	2	0	0	0	0	0	0	0	0	0	0	0
d__Bacteria;p__Actinobacteriota;c__Actinobacteria;o__Catenulisporales;f__Actinospicaceae	0	0	0	10	0	0	0	0	0	0	0	0
d__Bacteria;p__Proteobacteria;c__Alphaproteobacteria;o__Azospirillales;f__Azospirillaceae	0	0	0	0	0	0	3	0	0	0	0	0

Bacteria taxonomy	L1_ASR5i	L2_ASR5i	L3_ASR5i	L4_ASR5i	L5_ASR5i	L6_ASR5i	L1_WT	L2_WT	L3_WT	L4_WT	L5_WT	L6_WT
d__Bacteria;p__Fusobacteriota;c__Fusobacteriia;o__Fusobacteriales;f__Leptotrichiaceae	0	0	0	0	0	0	0	0	3	0	0	0
d__Bacteria;p__Actinobacteriota;c__Actinobacteria;o__Micromonosporales;f__Micromonosporaceae	0	0	0	0	4	0	0	0	0	0	0	0
d__Bacteria;p__Bacteroidota;c__Bacteroidia;o__Bacteroidales;f__Bacteroidetes_vadinHA17	5	0	0	0	0	0	0	0	0	0	0	0
d__Bacteria;p__Myxococcota;c__Myxococcia;o__Myxococcales;f__Anaeromyxobacteraceae	0	0	0	0	0	0	0	0	0	12	0	0
	1425	2003	1763	1554	284	273	651	364	658	1019	534	213
