# Supplementary material for: The impact of OsASR5 on rice microbiome: Implications for nutrient cycling and stress response
Source: Genet Mol Biol. 2026 Oct 26;49(Suppl 3):e20250064. doi: 10.1590/1678-4685-GMB-2025-0064 (PMC13720868; doi:10.1590/1678-4685-GMB-2025-0064)
Supplement: Supplementary file 6 [file 1415-4757-GMB-49-s3-e20250064-s6.pdf]

Genetics and Molecular Biology

https://doi.org/10.1590/1678-4685-GMB-2025-0064

Supplementary Material to "The impact of OsASR5 on rice microbiome: Implications for nutrient cycling and stress response"

Table S6 - Bacterial phylum abundance per sample from root microbiome.

Bacteria taxonomy	R1_ASR5i	R2_ASR5i	R3_ASR5i	R4_ASR5i	R5_ASR5i	R6_ASR5i	R1_WT	R2_WT	R3_WT	R4_WT	R5_WT	R6_WT
d__Bacteria;p__Cyanobacteria	831	6544	1640	6038	4234	6022	3652	13668	11046	7634	4152	16874
d__Bacteria;p__Proteobacteria	10487	22827	20446	19122	13919	36690	17616	30031	37958	30288	27960	35938
d__Bacteria;p__Acidobacteriota	3469	3830	4697	3476	5312	11891	2775	3139	5088	5188	5455	6615
d__Bacteria;p__Bacteroidota	967	1444	1085	1011	1266	2989	887	2006	2295	2073	2545	2331
d__Bacteria;p__Myxococcota	807	1512	1557	1127	2230	3471	988	893	1470	958	1836	1719
d__Bacteria;p__Actinobacteriota	727	1073	1410	840	1328	3531	1534	846	1329	1508	1898	1386
d__Bacteria;p__Firmicutes	2270	4008	4075	3383	3466	8421	3466	3371	8433	4661	7642	7495
d__Bacteria;p__Desulfobacterota	1082	2086	2417	1143	1890	4113	1571	2362	1383	2490	2169	2533
d__Bacteria;p__Chloroflexi	736	457	950	437	775	2892	862	527	1439	1407	1839	957
d__Bacteria;p__Gemmatimonadota	210	79	285	77	168	793	146	61	319	416	576	317
d__Bacteria;p__Fibrobacterota	110	377	171	329	112	307	115	407	325	333	249	546
d__Bacteria;p__Patescibacteria	318	383	575	140	368	1483	263	406	287	569	491	341
d__Bacteria;p__Spirochaetota	172	275	242	145	354	507	140	330	247	350	269	362
d__Bacteria;p__Verrucomicrobiota	217	358	442	265	383	1410	242	470	379	620	563	773
d__Bacteria;p__Nitrospirota	42	36	157	23	32	334	35	189	44	137	22	139

Bacteria taxonomy	R1_ASR 5i	R2_ASR 5i	R3_ASR 5i	R4_ASR 5i	R5_ASR 5i	R6_ASR 5i	R1_W T	R2_W T	R3_W T	R4_W T	R5_W T	R6_W T
d__Bacteria;__	492	1004	709	855	875	2442	299	1068	1604	1090	1009	1567
d__Bacteria;p__Halanaerobiaeota	17	7	22	0	9	0	4	50	42	10	70	13
d__Bacteria;p__RCP2-54	39	19	79	10	9	93	49	19	31	22	82	32
d__Archaea;p__Halobacterota	51	32	46	27	106	73	35	17	101	34	158	71
d__Bacteria;p__Bdellovibrionota	17	50	101	23	31	168	41	18	63	157	83	52
d__Bacteria;p__WPS-2	20	8	34	10	3	51	46	8	13	26	53	24
d__Bacteria;p__Campilobacterota	0	0	0	24	0	0	15	4	8	16	31	0
d__Bacteria;p__Armatimonadota	0	6	19	11	5	95	18	14	11	22	24	31
d__Archaea;p__Crenarchaeota	8	17	9	0	12	43	6	4	21	11	7	30
d__Bacteria;p__Elusimicrobiota	18	27	29	19	34	114	23	41	26	45	50	45
d__Bacteria;p__Margulisbacteria	0	15	11	16	0	6	0	0	0	0	7	7
d__Bacteria;p__FCPU426	0	6	3	0	2	30	14	27	4	43	6	22
d__Bacteria;p__Planctomycetota	14	8	28	7	24	101	48	21	24	24	43	40
d__Archaea;p__Euryarchaeota	0	0	7	0	6	26	11	3	30	7	19	3
d__Bacteria;p__Sva0485	2	3	2	2	0	14	2	4	2	18	9	0
d__Archaea;p__Nanoarchaeota	7	0	0	2	3	11	3	18	6	17	4	6
d__Bacteria;p__SAR324_clade(Marine_group_B)	0	0	4	0	0	3	9	0	0	0	2	2
d__Bacteria;p__WS4	0	7	7	0	0	7	0	4	0	0	8	8
d__Archaea;p__Thermoplasmatota	0	7	4	0	2	2	0	8	5	0	0	0
d__Bacteria;p__Sumerlaeota	8	3	9	0	6	0	0	0	0	3	3	9
Unassigned;__	3	3	0	0	0	0	6	4	0	0	0	7
d__Bacteria;p__Dependentiae	6	13	4	2	9	4	5	8	6	8	12	7
d__Bacteria;p__Hydrogenedentes	2	3	8	0	0	9	0	4	0	0	3	3
d__Bacteria;p__Methylomirabilota	0	0	2	0	0	0	0	2	0	3	0	6
d__Bacteria;p__Deferrisomatota	0	0	5	0	0	0	0	0	0	0	0	0
d__Bacteria;p__Latescibacterota	0	0	0	0	0	4	0	6	0	2	0	5

Bacteria taxonomy	R1_ASR 5i	R2_ASR 5i	R3_ASR 5i	R4_ASR 5i	R5_ASR 5i	R6_ASR 5i	R1_W T	R2_W T	R3_W T	R4_W T	R5_W T	R6_W T
d__Bacteria;p__GAL15	5	0	0	0	3	0	0	4	0	0	0	0
d__Bacteria;p__MBNT15	0	0	0	0	0	3	2	3	0	0	0	0
d__Bacteria;p__Entotheonellaeota	0	0	0	0	0	0	0	2	0	2	0	0
