# Supplementary material for: The impact of OsASR5 on rice microbiome: Implications for nutrient cycling and stress response
Source: Genet Mol Biol. 2026 Oct 26;49(Suppl 3):e20250064. doi: 10.1590/1678-4685-GMB-2025-0064 (PMC13720868; doi:10.1590/1678-4685-GMB-2025-0064)
Supplement: Supplementary file 7 [file 1415-4757-GMB-49-s3-e20250064-s7.pdf]

Genetics and Molecular Biology

https://doi.org/10.1590/1678-4685-GMB-2025-0064

Supplementary Material to "The impact of OsASR5 on rice microbiome: Implications for nutrient cycling and stress response"

Table S7 - Bacterial family abundance per sample from root microbiome.

**Bacteria taxonomy**	**R1_ASR5i**	**R2_ASR5i**	**R3_ASR5i**	**R4_ASR5i**	**R5_ASR5i**	**R6_ASR5i**	**R1_WT**	**R2_WT**	**R3_WT**	**R4_WT**	**R5_WT**	**R6_WT**
d__Bacteria;p__Cyanobacteria;c__Cyanobacteriia;o__Chloroplast;f__Chloroplast	826	6502	1623	6017	4213	5965	3631	13645	11017	7623	4120	16839
d__Bacteria;p__Proteobacteria;c__Alphaproteobacteria;o__Rickettsiales;f__Mitochondria	524	1559	896	2068	1478	2630	2058	8368	6119	4220	2814	7647
d__Bacteria;p__Proteobacteria;c__Gammaproteobacteria;o__Burkholderiales;f__Rhodocyclaceae	2905	7822	4278	8183	2493	6127	4398	8013	12194	8022	7648	11292
d__Bacteria;p__Proteobacteria;c__Gammaproteobacteria;o__Burkholderiales;__	2121	5016	3889	3619	2885	6557	3755	7764	7645	6860	5063	6060
d__Bacteria;p__Acidobacteriota;c__Acidobacteriae;o__Acidobacteriales;f__Acidobacteriaceae_(Subgroup_1)	1162	1388	1537	1198	1678	3970	852	891	1939	1594	1949	2390
d__Bacteria;p__Proteobacteria;c__Alphaproteobacteria;o__Rhizobiales;f__Xanthobacteraceae	558	455	1113	608	730	2254	1155	416	838	962	1286	896
d__Bacteria;p__Bacteroidota;c__Bacteroidia;o__Bacteroidales;__	288	632	307	483	379	737	246	882	845	714	771	892
d__Bacteria;p__Proteobacteria;c__Gammaproteobacteria;o__Xanthomonadales;f__Rhodanobacteraceae	458	644	834	440	349	1664	730	455	825	660	1009	1029
d__Bacteria;p__Myxococcota;c__Myxococcia;o__Myxococcales;f__Myxococcaceae	215	417	385	230	567	749	246	146	400	243	412	403
d__Bacteria;p__Actinobacteriota;c__Actinobacteria;o__Kineosporiales;f__Kineosporiaceae	123	486	325	251	393	838	167	313	122	236	159	283
d__Bacteria;p__Myxococcota;c__Myxococcia;o__Myxococcales;f__Anaeromyxobacteraceae	280	405	606	255	1019	1367	282	168	498	205	566	447
d__Bacteria;p__Acidobacteriota;c__Acidobacteriae;o__Solibacterales;f__Solibacteraceae	402	396	533	491	619	1610	303	461	770	716	747	947

Bacteria taxonomy	R1_ASR5i	R2_ASR5i	R3_ASR5i	R4_ASR5i	R5_ASR5i	R6_ASR5i	R1_WT	R2_WT	R3_WT	R4_WT	R5_WT	R6_WT
d__Bacteria;p__Acidobacteriota;c__Acidobacteriae;o__Acidobacteriales;f__Koribacteraceae	406	283	553	363	689	1206	269	293	707	725	948	794
d__Bacteria;p__Acidobacteriota;c__Holophagae;o__Holophagales;f__Holophagaceae	743	1278	1108	968	1592	2452	935	814	766	1219	883	895
d__Bacteria;p__Proteobacteria;c__Alphaproteobacteria;o__Micropepsales;f__Micropepsaceae	512	648	827	470	503	1907	527	650	1793	1127	1655	1422
d__Bacteria;p__Firmicutes;c__Bacilli;o__Bacillales;f__Bacillaceae	285	383	572	339	189	1382	366	186	423	313	403	633
d__Bacteria;p__Firmicutes;c__Clostridia;o__Clostridia;f__Hungateiclostridiaceae	381	864	563	660	643	1207	469	801	1591	1057	1519	1990
d__Bacteria;p__Proteobacteria;c__Gammaproteobacteria;o__Burkholderiales;f__Comamonadaceae	281	869	1033	561	673	2249	403	441	347	1063	572	373
d__Bacteria;p__Proteobacteria;c__Alphaproteobacteria;o__Rhizobiales;f__Rhizobiaceae	72	419	572	137	308	585	229	85	257	926	295	85
d__Bacteria;p__Desulfobacterota;c__Desulfovibrionia;o__Desulfovibrionales;f__Desulfovibrionaceae	89	236	261	172	200	501	274	357	134	289	222	575
d__Bacteria;p__Chloroflexi;c__Ktedonobacteria;o__Ktedonobacterales;f__JG30-KF-AS9	347	88	486	134	341	1109	451	142	807	721	816	368
d__Bacteria;p__Firmicutes;c__Clostridia;o__Clostridiales;f__Clostridiaceae	590	906	1466	824	1057	2128	1084	450	2629	1181	1490	1203
d__Bacteria;p__Proteobacteria;c__Alphaproteobacteria;o__Elsterales;f__uncultured	261	135	266	171	136	655	178	139	453	340	460	391
d__Bacteria;p__Desulfobacterota;c__Desulfuromonadia;o__Geobacterales;f__Geobacteraceae	834	1456	1678	673	1497	2882	1048	1527	1069	1588	1575	1388
d__Bacteria;p__Proteobacteria;c__Gammaproteobacteria;o__Methylococcales;f__Methylomonadaceae	137	757	1202	149	852	923	420	63	864	142	368	49
d__Bacteria;p__Firmicutes;c__Negativicutes;o__Veillonellales-Selenomonadales;f__Sporomusaceae	93	217	218	187	190	917	261	208	435	112	597	425
d__Bacteria;p__Proteobacteria;c__Gammaproteobacteria;o__Gammaproteobacteria_Incertae_Sedis;f__Unknown_Family	324	287	342	250	192	788	307	130	513	342	604	675
d__Bacteria;p__Proteobacteria;c__Alphaproteobacteria;o__Rhodospirillales;f__Magnetospirillaceae	137	454	371	290	209	636	170	748	631	524	517	955
d__Bacteria;p__Proteobacteria;c__Gammaproteobacteria;__;__	86	174	185	114	126	479	173	377	525	372	418	547
d__Bacteria;p__Chloroflexi;c__Anaerolineae;o__SJA-15;f__SJA-15	109	27	108	41	112	575	104	31	190	145	322	70
d__Bacteria;p__Gemmatimonadota;c__Gemmatimonadetes;o__Gemmatimonadales;f__Gemmatimonadaceae	210	79	270	77	165	766	144	61	313	403	571	313
d__Bacteria;p__Firmicutes;c__Clostridia;o__Clostridiales;f__Caloramatoraceae	142	312	217	302	158	454	330	345	646	395	724	443
d__Bacteria;p__Proteobacteria;c__Gammaproteobacteria;o__Burkholderiales;f__SC-I-84	190	67	313	82	120	598	245	0	297	173	383	195

Bacteria taxonomy	R1_A SR5i	R2_A SR5i	R3_A SR5i	R4_A SR5i	R5_A SR5i	R6_A SR5i	R1_ WT	R2_ WT	R3_ WT	R4_ WT	R5_ WT	R6_ WT
d__Bacteria;p__Proteobacteria;c__Alphaproteobacteria;o__Rhizobiales;__	88	124	181	90	159	540	80	140	119	170	152	198
d__Bacteria;p__Proteobacteria;c__Alphaproteobacteria;o__Caulobacterales;f__Caulobacteraceae	189	309	410	211	339	831	239	154	347	485	508	382
d__Bacteria;p__Proteobacteria;c__Alphaproteobacteria;__;__	357	439	450	294	392	1268	318	354	772	572	486	734
d__Bacteria;p__Proteobacteria;c__Gammaproteobacteria;o__Burkholderiales;f__Burkholderiaceae	112	289	241	188	190	298	261	167	188	124	391	252
d__Bacteria;p__Proteobacteria;c__Alphaproteobacteria;o__Rhizobiales;f__Beijerinckiaceae	201	374	692	125	339	1212	345	53	370	349	492	229
d__Bacteria;p__Bacteroidota;c__Bacteroidia;o__Bacteroidales;f__Paludibacteraceae	86	153	82	49	194	173	157	511	338	167	371	294
d__Bacteria;p__Fibrobacterota;c__Fibrobacteria;o__Fibrobacterales;f__Fibrobacterales	100	366	153	308	112	220	104	357	313	323	234	520
d__Bacteria;p__Actinobacteriota;c__Coriobacteriia;o__OPB41;f__OPB41	50	70	91	61	68	257	148	55	143	52	173	53
d__Bacteria;p__Myxococcota;c__Polyangia;o__Haliangiales;f__Haliangiaceae	193	511	393	498	435	698	332	430	450	369	626	615
d__Bacteria;p__Proteobacteria;c__Alphaproteobacteria;o__Acetobacterales;f__Acetobacteraceae	282	275	478	192	307	926	456	130	435	283	714	387
d__Bacteria;p__Firmicutes;c__Clostridia;o__Clostridia;f__Gracilibacteraceae	63	69	24	67	158	220	38	201	379	396	476	677
d__Bacteria;p__Patescibacteria;c__Saccharimonadia;o__Saccharimonadales;f__LWQ8	31	92	70	15	0	315	8	0	4	10	47	0
d__Bacteria;p__Patescibacteria;c__Saccharimonadia;o__Saccharimonadales;__	92	40	160	7	164	374	54	43	64	59	121	39
d__Bacteria;p__Proteobacteria;c__Alphaproteobacteria;o__Rhizobiales;f__Rhizobiales_Incertae_Sedis	17	100	133	33	19	352	45	16	65	13	99	28
d__Bacteria;p__Firmicutes;c__Clostridia;o__Oscillospirales;f__Oscillospiraceae	76	117	93	83	72	159	73	71	216	73	319	174
d__Bacteria;p__Actinobacteriota;c__Actinobacteria;__;__	39	76	118	77	172	355	126	84	172	198	196	89
d__Bacteria;p__Acidobacteriota;c__Acidobacteriae;o__Acidobacteriales;f__uncultured	293	167	486	229	329	1166	182	367	402	462	332	845
d__Bacteria;p__Proteobacteria;c__Alphaproteobacteria;o__Rhizobiales;f__Pleomorphomonadaceae	9	271	137	13	13	142	0	12	33	295	0	50
d__Bacteria;p__Proteobacteria;c__Gammaproteobacteria;o__WD260;f__WD260	79	71	136	63	85	267	155	49	213	118	207	167
d__Bacteria;p__Proteobacteria;c__Gammaproteobacteria;o__KF-JG30-C25;f__KF-JG30-C25	124	34	182	77	49	255	58	122	296	311	287	344
d__Bacteria;p__Chloroflexi;c__KD4-96;o__KD4-96;f__KD4-96	66	100	70	96	104	472	66	56	143	111	171	146
d__Bacteria;p__Proteobacteria;c__Gammaproteobacteria;o__Burkholderiales;f__Methylophilaceae	0	33	168	5	143	196	26	4	48	57	32	14
d__Bacteria;p__Spirochaetota;c__Spirochaetia;o__Spirochaetales;f__Spirochaetaceae	172	275	240	145	352	500	140	327	245	350	266	362
d__Bacteria;p__Firmicutes;c__Clostridia;o__Lachnospirales;f__Lachnospiraceae	34	107	95	104	91	207	77	137	395	178	234	389

Bacteria taxonomy	R1_A SR5i	R2_A SR5i	R3_A SR5i	R4_A SR5i	R5_A SR5i	R6_A SR5i	R1_ WT	R2_ WT	R3_ WT	R4_ WT	R5_ WT	R6_ WT
d__Bacteria;p__Proteobacteria;c__Alphaproteobacteria;o__Sphingomonadales;f__Sphingomonadaceae	109	179	242	104	132	420	181	61	285	346	242	138
d__Bacteria;p__Actinobacteriota;c__Actinobacteria;o__Micrococcales;f__Cellulomonadaceae	0	99	42	56	45	106	70	0	62	42	78	58
d__Bacteria;p__Bacteroidota;c__Bacteroidia;o__Chitinophagales;f__Chitinophagaceae	206	217	288	108	125	971	232	120	291	360	473	264
d__Bacteria;p__Proteobacteria;c__Gammaproteobacteria;o__Burkholderiales;f__Oxalobacteraceae	27	128	132	34	141	257	87	107	96	243	34	92
d__Bacteria;p__Myxococcota;c__Polyangia;o__Polyangiales;f__Polyangiaceae	89	95	79	67	116	411	101	69	80	58	160	141
d__Bacteria;p__Verrucomicrobiota;c__Verrucomicrobiae;o__Pedosphaerales;f__Pedosphaeraceae	154	256	285	171	264	989	169	305	278	402	427	546
d__Bacteria;p__Firmicutes;c__Clostridia;o__Peptostreptococcales-Tissierellales;f__Anaerovoracaceae	27	64	32	39	72	45	46	52	137	39	130	82
d__Bacteria;p__Desulfobacterota;c__Syntrophobacteria;o__Syntrophobacterales;f__Syntrophobacteraceae	51	153	182	89	39	226	114	206	44	226	118	328
d__Bacteria;p__Actinobacteriota;c__Acidimicrobiia;o__uncultured;f__uncultured	39	46	77	19	45	148	77	69	53	34	89	85
d__Bacteria;p__Bacteroidota;c__Kryptonia;o__Kryptoniales;f__BSV26	70	24	69	33	144	216	7	36	61	92	145	113
d__Bacteria;p__Firmicutes;c__Clostridia;__;__	124	170	151	249	231	355	143	226	372	276	478	395
d__Bacteria;p__Desulfobacterota;c__Desulfuromonadia;o__Geobacterales;__	16	78	112	76	11	22	52	145	13	197	98	4
d__Bacteria;p__Actinobacteriota;c__Actinobacteria;o__Frankiales;f__Acidothermaceae	225	133	417	161	250	726	449	137	389	573	622	364
d__Bacteria;p__Firmicutes;c__Clostridia;o__Peptostreptococcales-Tissierellales;f__Peptostreptococcaceae	38	4	44	12	37	102	97	5	58	70	81	33
d__Bacteria;p__Proteobacteria;c__Gammaproteobacteria;o__Burkholderiales;f__Gallionellaceae	0	154	74	20	84	82	65	148	62	131	99	71
d__Bacteria;p__Proteobacteria;c__Gammaproteobacteria;o__Burkholderiales;f__Nitrosomonadaceae	47	24	74	0	0	205	30	43	42	68	80	73
d__Bacteria;p__Proteobacteria;__;__;__	46	175	62	181	104	227	41	383	548	265	159	526
d__Bacteria;p__Nitrospirota;c__Thermodesulfovibrionia;o__uncultured;f__uncultured	27	27	110	17	30	256	26	125	30	81	12	84
d__Bacteria;p__Firmicutes;c__Bacilli;o__Paenibacillales;f__Paenibacillaceae	20	145	38	13	17	72	41	13	83	21	82	50
d__Bacteria;p__Acidobacteriota;c__Acidobacteriae;o__Acidobacteriales;__	136	50	96	40	141	362	51	61	149	81	133	108
d__Bacteria;p__Chloroflexi;c__Anaerolineae;o__Anaerolineales;f__Anaerolineaceae	54	149	95	92	111	367	58	145	121	111	214	181
d__Bacteria;p__Actinobacteriota;c__Actinobacteria;o__Streptomycetales;f__Streptomycetaceae	22	30	33	19	18	183	50	24	34	6	41	36
d__Bacteria;__;__;__;__	492	1004	709	855	875	2442	299	1068	1604	1090	1009	1567

Bacteria taxonomy	R1_A SR5i	R2_A SR5i	R3_A SR5i	R4_A SR5i	R5_A SR5i	R6_A SR5i	R1_ WT	R2_ WT	R3_ WT	R4_ WT	R5_ WT	R6_ WT
d__Bacteria;p__Proteobacteria;c__Alphaproteobacteria;o__Elsterales;__	2	33	36	40	21	105	27	35	0	55	95	80
d__Bacteria;p__Proteobacteria;c__Alphaproteobacteria;o__Rhizobiales;f__Devosiaceae	15	44	33	40	38	108	48	56	49	36	74	64
d__Bacteria;p__Bacteroidota;c__Bacteroidia;o__Sphingobacteriales;f__Sphingobacteriaceae	37	57	50	49	51	111	72	64	75	131	108	93
d__Bacteria;p__Chloroflexi;c__Ktedonobacteria;o__Ktedonobacterales;f__Ktedonobacteraceae	72	50	123	50	49	148	115	65	105	197	198	95
d__Bacteria;p__Acidobacteriota;c__Acidobacteriae;o__Subgroup_2;f__Subgroup_2	106	87	134	24	39	270	60	102	136	150	124	154
d__Bacteria;p__Firmicutes;c__Clostridia;o__Clostridia;__	6	0	5	0	11	48	0	0	34	19	116	15
d__Bacteria;p__Firmicutes;c__Desulfitobacteriia;o__Desulfitobacteriales;f__Desulfitobacteriaceae	47	158	138	87	71	163	100	121	132	108	94	150
d__Bacteria;p__Acidobacteriota;c__Acidobacteriae;__;__	34	43	55	64	34	247	3	49	36	60	79	108
d__Bacteria;p__Proteobacteria;c__Gammaproteobacteria;o__Enterobacterales;f__Enterobacteriaceae	9	29	60	20	54	26	27	0	92	7	5	10
d__Bacteria;p__Bacteroidota;c__Kryptonia;o__Kryptoniales;f__Kryptoniaceae	16	14	21	18	0	47	0	20	122	77	42	78
d__Bacteria;p__Bacteroidota;c__Ignavibacteria;o__Ignavibacteriales;__	50	3	74	84	63	150	33	58	113	130	106	110
d__Bacteria;p__Bacteroidota;c__Ignavibacteria;o__Ignavibacteriales;f__LD-RB-34	28	78	33	42	55	36	14	103	73	105	83	42
d__Bacteria;p__Bacteroidota;c__Bacteroidia;o__Bacteroidales;f__Prolixibacteraceae	41	36	14	29	48	42	23	57	34	80	50	83
d__Bacteria;p__Firmicutes;c__Limnochordia;o__Limnochordia;f__Limnochordia	63	152	68	83	56	128	55	90	204	94	195	260
d__Bacteria;p__Proteobacteria;c__Alphaproteobacteria;o__Dongiales;f__Dongiaceae	0	19	23	22	20	59	31	26	53	40	73	8
d__Bacteria;p__Verrucomicrobiota;c__Verrucomicrobiae;o__Opitutales;f__Opitutaceae	26	53	72	63	48	170	41	112	45	180	88	183
d__Bacteria;p__Patescibacteria;__;__;__	19	11	21	15	10	120	23	32	16	56	26	28
d__Bacteria;p__Halanaerobiaeota;c__Halanaerobiia;o__Halanaerobiales;f__Halobacteroidaceae	17	7	22	0	9	0	4	50	42	10	70	13
d__Bacteria;p__Patescibacteria;c__Gracilibacteria;o__Gracilibacteria;f__Gracilibacteria	30	24	36	16	21	129	14	51	7	97	15	31
d__Bacteria;p__Actinobacteriota;c__Actinobacteria;o__Micrococcales;f__Microbacteriaceae	0	0	6	2	5	37	28	3	60	36	38	31
d__Bacteria;p__Desulfobacterota;c__Desulfobulbia;o__Desulfobulbales;f__Desulfobulbaceae	32	67	41	26	32	66	12	21	21	0	37	11
d__Bacteria;p__Actinobacteriota;c__Thermoleophilia;o__Gaiellales;f__uncultured	17	6	69	26	32	147	51	28	35	35	62	59
d__Bacteria;p__Desulfobacterota;c__Desulfobulbia;o__Desulfobulbales;f__Desulfocapsaceae	0	10	18	22	8	72	18	17	0	0	0	30

Bacteria taxonomy	R1_A SR5i	R2_A SR5i	R3_A SR5i	R4_A SR5i	R5_A SR5i	R6_A SR5i	R1_ WT	R2_ WT	R3_ WT	R4_ WT	R5_ WT	R6_ WT
d__Bacteria;p__Acidobacteriota;c__Acidobacteriae;o__Bryobacterales;f__Bryobacteraceae	140	95	114	66	111	364	70	55	121	116	177	259
d__Bacteria;p__Actinobacteriota;c__Actinobacteria;o__Micromonosporales;f__Micromonosporaceae	15	10	28	10	19	69	39	12	32	102	70	40
d__Bacteria;p__Firmicutes;c__Bacilli;o__Alicyclobacillales;f__Alicyclobacillaceae	77	6	27	17	11	60	68	16	26	28	77	34
d__Bacteria;p__Firmicutes;c__Clostridia;o__Christensenellales;f__Christensenellaceae	70	162	120	173	144	218	72	173	205	114	258	184
d__Bacteria;p__Proteobacteria;c__Alphaproteobacteria;o__Rhodospirillales;f__uncultured	27	39	65	18	23	107	11	49	51	84	70	37
d__Bacteria;p__Bacteroidota;c__Ignavibacteria;o__Ignavibacteriales;f__BSV40	30	30	17	13	33	43	15	22	64	22	62	62
d__Bacteria;p__Verrucomicrobiota;c__Verrucomicrobiae;o__Chthoniobacterales;f__Terrimicrobiaceae	3	21	22	0	6	61	0	6	5	0	0	9
d__Bacteria;p__Actinobacteriota;c__Acidimicrobiia;o__IMCC26256;f__IMCC26256	22	0	16	0	39	109	42	0	28	25	85	40
d__Bacteria;p__Actinobacteriota;c__Actinobacteria;o__Frankiales;f__Sporichthyaceae	15	10	31	7	32	60	0	11	31	11	0	20
d__Bacteria;p__RCP2-54;c__RCP2-54;o__RCP2-54;f__RCP2-54	39	19	79	10	9	93	49	19	31	22	82	32
d__Bacteria;p__Proteobacteria;c__Gammaproteobacteria;o__Steroidobacterales;f__Steroidobacteraceae	9	27	21	12	29	78	13	26	5	35	22	57
d__Bacteria;p__Actinobacteriota;__;__;__	13	5	11	7	33	79	19	23	36	25	20	39
d__Bacteria;p__Proteobacteria;c__Alphaproteobacteria;o__Rhizobiales;f__Ancalomicrobiaceae	0	0	0	0	62	0	0	0	0	0	0	0
d__Bacteria;p__Desulfobacterota;c__Desulfobaccia;o__Desulfobaccales;f__Desulfobaccaceae	16	0	41	17	12	104	17	19	20	37	44	45
d__Archaea;p__Halobacterota;c__Methanocellia;o__Methanocellales;f__Methanocellaceae	32	27	27	27	73	51	14	14	68	18	117	55
d__Bacteria;p__Bacteroidota;c__Bacteroidia;o__Bacteroidales;f__Bacteroidetes_vadinHA17	27	73	39	27	66	100	6	30	112	27	78	121
d__Bacteria;p__Patescibacteria;c__Parcubacteria;__;__	22	8	51	16	21	83	22	37	30	81	64	25
d__Bacteria;p__Firmicutes;c__Clostridia;o__Oscillospirales;f__Ruminococcaceae	10	31	9	4	17	30	13	26	74	7	7	19
d__Bacteria;p__Patescibacteria;c__Parcubacteria;o__Candidatus_Jorgensenbacteria;f__Candidatus_Jorgensenbacteria	16	92	46	26	23	120	39	98	21	72	38	67
d__Bacteria;p__Actinobacteriota;c__Actinobacteria;o__Frankiales;f__uncultured	22	0	0	5	0	0	53	11	0	0	0	0
d__Bacteria;p__Proteobacteria;c__Alphaproteobacteria;o__Rhizobiales;f__Rhodomicrobiaceae	9	0	0	15	28	34	20	0	34	12	53	5
d__Bacteria;p__Bacteroidota;c__Bacteroidia;o__Flavobacteriales;f__Weeksellaceae	0	0	0	0	0	59	0	0	0	0	0	0
d__Bacteria;p__Fibrobacterota;c__Fibrobacteria;o__Fibrobacterales;f__Fibrobacteraceae	5	6	12	11	0	68	6	37	0	8	0	3

Bacteria taxonomy	R1_A SR5i	R2_A SR5i	R3_A SR5i	R4_A SR5i	R5_A SR5i	R6_A SR5i	R1_ WT	R2_ WT	R3_ WT	R4_ WT	R5_ WT	R6_ WT
d__Bacteria;p__Proteobacteria;c__Gammaproteobacteria;o__Burkholderiales;f__Alcaligenaceae	9	47	32	21	16	16	12	3	81	10	16	20
d__Bacteria;p__Nitrospirota;c__Nitrospiria;o__Nitrospirales;f__Nitrospiraceae	9	6	9	2	0	45	5	20	10	18	10	40
d__Bacteria;p__Myxococcota;c__Polyangia;o__mle1-27;f__mle1-27	7	24	48	34	13	94	0	34	0	30	14	15
d__Bacteria;p__Actinobacteriota;c__Thermoleophilia;o__Solirubrobacterales;f__Solirubrobacteraceae	33	39	39	30	77	157	90	32	56	17	72	32
d__Bacteria;p__Bacteroidota;__;__;__	0	10	0	10	7	41	15	6	0	14	17	37
d__Bacteria;p__Bacteroidota;c__Bacteroidia;o__Flavobacteriales;f__Flavobacteriaceae	0	0	0	0	0	0	0	0	40	0	57	0
d__Bacteria;p__Bacteroidota;c__Ignavibacteria;o__Ignavibacteriales;f__SR-FBR-L83	13	22	33	3	8	30	0	23	11	14	47	27
d__Bacteria;p__Proteobacteria;c__Gammaproteobacteria;o__Burkholderiales;f__Chromobacteriaceae	3	77	19	13	7	64	0	0	0	17	21	7
d__Bacteria;p__Patescibacteria;c__Saccharimonadia;o__Saccharimonadales;f__Saccharimonadales	16	25	37	6	24	49	11	15	50	7	14	9
d__Bacteria;p__Proteobacteria;c__Alphaproteobacteria;o__uncultured;f__uncultured	28	7	27	18	8	84	24	35	20	25	67	57
d__Bacteria;p__Bdellovibrionota;c__Bdellovibrionia;o__Bdellovibrionales;f__Bdellovibrionaceae	8	25	44	8	7	80	4	7	34	108	17	22
d__Bacteria;p__Proteobacteria;c__Gammaproteobacteria;o__Burkholderiales;f__Chitinimonadaceae	0	16	43	10	0	18	15	33	0	67	0	29
d__Archaea;p__Halobacterota;c__Methanosarcinia;o__Methanosarciniales;f__Methanosarcinaceae	19	5	17	0	33	22	13	0	27	7	41	13
d__Bacteria;p__Patescibacteria;c__Parcubacteria;o__Parcubacteria;f__Parcubacteria	39	15	40	9	37	122	18	58	26	83	62	46
d__Bacteria;p__Firmicutes;c__Clostridia;o__Clostridiales;f__uncultured	13	15	19	15	18	21	18	2	40	0	17	0
d__Bacteria;p__Proteobacteria;c__Gammaproteobacteria;o__Xanthomonadales;__	22	4	0	0	4	2	0	4	54	4	0	0
d__Bacteria;p__Actinobacteriota;c__Actinobacteria;o__Corynebacteriales;f__Mycobacteriaceae	11	10	22	18	32	45	21	10	21	13	79	36
d__Bacteria;p__Actinobacteriota;c__Acidimicrobiia;__;__	0	0	0	18	8	40	0	3	0	17	0	0
d__Bacteria;p__Proteobacteria;c__Alphaproteobacteria;o__Rhizobiales;f__Methyloligellaceae	17	8	18	10	0	40	10	5	18	0	7	16
d__Bacteria;p__Verrucomicrobiota;c__Verrucomicrobiae;o__Chthoniobacterales;f__Chthoniobacteraceae	29	21	52	26	19	112	20	16	37	23	14	31
d__Bacteria;p__Proteobacteria;c__Alphaproteobacteria;o__Rhodobacterales;f__Rhodobacteraceae	0	0	8	0	0	8	29	0	16	27	17	0
d__Bacteria;p__Proteobacteria;c__Alphaproteobacteria;o__Azospirillales;f__Azospirillaceae	14	71	21	25	8	22	0	18	14	0	39	11

Bacteria taxonomy	R1_A SR5i	R2_A SR5i	R3_A SR5i	R4_A SR5i	R5_A SR5i	R6_A SR5i	R1_ WT	R2_ WT	R3_ WT	R4_ WT	R5_ WT	R6_ WT
d__Bacteria;p__Proteobacteria;c__Gammaproteobacteria;o__Pseudomonadales;f__Pseudomonadaceae	0	0	0	0	3	0	0	0	38	0	3	6
d__Bacteria;p__Acidobacteriota;c__Vicinamibacteria;o__Vicinamibacterales;f__uncultured	13	11	15	14	38	47	17	0	28	32	42	24
d__Bacteria;p__Actinobacteriota;c__Actinobacteria;o__Frankiales;f__Frankiaceae	9	0	0	0	0	49	0	0	0	27	0	0
d__Bacteria;p__Desulfobacterota;c__Desulfobulbia;o__Desulfobulbales;f__uncultured	0	0	9	4	0	37	8	33	5	9	0	10
d__Bacteria;p__WPS-2;c__WPS-2;o__WPS-2;f__WPS-2	20	8	34	10	3	51	46	8	13	26	53	24
d__Bacteria;p__Proteobacteria;c__Alphaproteobacteria;o__Rickettsiales;__	0	0	4	0	0	0	0	14	0	49	0	20
d__Bacteria;p__Proteobacteria;c__Alphaproteobacteria;o__Azospirillales;f__Inquilinaceae	8	12	16	30	5	35	14	14	16	21	32	19
d__Bacteria;p__Proteobacteria;c__Alphaproteobacteria;o__Rhizobiales;f__Hyphomicrobiaceae	0	0	0	5	0	35	27	28	8	0	16	0
d__Bacteria;p__Patescibacteria;c__Parcubacteria;o__Candidatus_Kaiserbacteria;f__Candidatus_Kaiserbacteria	10	32	21	10	8	27	25	13	0	33	9	31
d__Bacteria;p__Firmicutes;c__Clostridia;o__Clostridiales;f__Oxobacteraceae	19	2	13	16	40	19	0	39	16	19	44	38
d__Bacteria;p__Firmicutes;c__Clostridia;o__Clostridiales;__	4	16	4	8	22	36	29	13	78	7	44	23
d__Bacteria;p__Campilobacterota;c__Campylobacteria;o__Campylobacterales;f__Sulfurimonadaceae	0	0	0	21	0	0	15	4	8	16	31	0
d__Bacteria;p__Acidobacteriota;__;__;__	13	4	12	6	22	43	6	14	11	0	9	26
d__Bacteria;p__Armatimonadota;c__Fimbriimonadia;o__Fimbriimonadales;f__Fimbriimonadaceae	0	6	14	6	5	81	14	10	9	22	7	27
d__Bacteria;p__Proteobacteria;c__Gammaproteobacteria;o__Diplorickettsiales;f__Diplorickettsiaceae	17	30	36	13	3	79	77	4	51	52	94	54
d__Bacteria;p__Proteobacteria;c__Gammaproteobacteria;o__Burkholderiales;f__Hydrogenophilaceae	7	19	0	0	0	8	0	29	0	20	0	11
d__Archaea;p__Crenarchaeota;c__Bathyarchaeia;o__Bathyarchaeia;f__Bathyarchaeia	8	13	9	0	12	34	6	0	21	4	0	19
d__Bacteria;p__Actinobacteriota;c__Actinobacteria;o__Micrococcales;f__Intrasporangiaceae	28	4	0	0	0	0	11	0	0	3	21	4
d__Bacteria;p__Elusimicrobiota;c__Elusimicrobia;o__Lineage_IV;f__Lineage_IV	8	11	19	11	12	42	11	16	17	22	28	15
d__Bacteria;p__Firmicutes;__;__;__	22	34	34	21	30	183	0	109	123	30	59	77
d__Bacteria;p__Myxococcota;c__Polyangia;o__Polyangiales;f__BIrii41	6	12	7	4	10	34	0	15	5	4	20	21
d__Bacteria;p__Patescibacteria;c__Saccharimonadia;o__Saccharimonadales;f__Saccharimonadaceae	9	0	27	5	4	10	0	0	4	7	17	0

Bacteria taxonomy	R1_ASR5i	R2_ASR5i	R3_ASR5i	R4_ASR5i	R5_ASR5i	R6_ASR5i	R1_WT	R2_WT	R3_WT	R4_WT	R5_WT	R6_WT
d__Bacteria;p__Margulisbacteria;c__Margulisbacteria;o__Margulisbacteria;f__Margulisbacteria	0	15	11	16	0	6	0	0	0	0	7	7
d__Bacteria;p__Nitrospirota;c__4-29-1;o__4-29-1;f__4-29-1	6	0	38	4	2	30	4	44	4	38	0	15
d__Bacteria;p__Actinobacteriota;c__Thermoleophilia;o__Solirubrobacterales;f__67-14	4	7	9	5	7	12	19	0	0	0	27	0
d__Bacteria;p__Proteobacteria;c__Gammaproteobacteria;o__Burkholderiales;f__TRA3-20	0	3	5	0	0	26	5	0	0	19	17	10
d__Bacteria;p__Proteobacteria;c__Alphaproteobacteria;o__Rhodospirillales;f__Rhodospirillaceae	10	8	5	0	3	26	10	4	16	15	28	10
d__Bacteria;p__Bdellovibrionota;c__Oligoflexia;o__0319-6G20;f__0319-6G20	9	17	54	12	18	81	37	11	29	46	54	26
d__Bacteria;p__Desulfobacterota;__;__;__	13	28	28	37	43	88	0	23	37	54	26	73
d__Bacteria;p__Bacteroidota;c__Bacteroidia;o__Bacteroidales;f__SB-5	3	0	0	0	3	39	9	0	0	0	0	0
d__Bacteria;p__Desulfobacterota;c__Desulfuromonadia;__;__	10	15	5	7	24	51	0	0	13	0	30	17
d__Bacteria;p__Actinobacteriota;c__Actinobacteria;o__Frankiales;f__Geodermatophilaceae	0	0	0	0	0	0	0	0	0	0	0	25
d__Bacteria;p__Bacteroidota;c__Bacteroidia;o__Sphingobacteriales;__	17	30	23	0	8	26	9	20	49	15	18	11
d__Bacteria;p__Proteobacteria;c__Gammaproteobacteria;o__Xanthomonadales;f__Xanthomonadaceae	3	2	0	0	0	0	0	2	31	0	11	2
d__Bacteria;p__Chloroflexi;c__Anaerolineae;o__RBG-13-54-9;f__RBG-13-54-9	11	0	8	0	3	26	4	11	11	3	11	0
d__Bacteria;p__Acidobacteriota;c__Acidobacteriae;o__Subgroup_13;f__Subgroup_13	10	14	28	13	0	54	10	18	20	13	8	36
d__Bacteria;p__Firmicutes;c__Bacilli;o__Aneurinibacillales;f__Aneurinibacillaceae	0	12	6	11	14	27	0	0	0	0	0	0
d__Bacteria;p__Actinobacteriota;c__Actinobacteria;o__Catenulisporales;f__Actinospicaceae	0	10	10	19	6	11	20	9	14	11	22	26
d__Bacteria;p__FCPU426;c__FCPU426;o__FCPU426;f__FCPU426	0	6	3	0	2	30	14	27	4	43	6	22
d__Bacteria;p__Acidobacteriota;c__Acidobacteriae;o__Acidobacteriae;f__Acidobacteriae	0	0	0	0	0	34	12	0	0	0	0	0
d__Bacteria;p__Chloroflexi;c__Anaerolineae;__;__	22	13	3	4	27	56	3	24	22	33	36	37
d__Bacteria;p__Actinobacteriota;c__Actinobacteria;o__Pseudonocardiales;f__Pseudonocardiaceae	10	2	31	8	5	26	0	0	6	0	6	6
d__Bacteria;p__Elusimicrobiota;c__Endomicrobia;o__Endomicrobiales;f__Endomicrobiaceae	10	16	10	8	18	46	12	19	9	13	19	18
d__Bacteria;p__Proteobacteria;c__Alphaproteobacteria;o__Reyranellales;f__Reyranellaceae	4	6	0	4	12	34	5	9	20	8	25	22
d__Bacteria;p__Planctomycetota;c__Planctomycetes;o__Isosphaerales;f__Isosphaeraceae	8	4	16	0	5	40	37	0	8	7	14	10

Bacteria taxonomy	R1_A SR5i	R2_A SR5i	R3_A SR5i	R4_A SR5i	R5_A SR5i	R6_A SR5i	R1_ WT	R2_ WT	R3_ WT	R4_ WT	R5_ WT	R6_ WT
d__Bacteria;p__Desulfobacterota;c__Desulfobulbia;o__Desulfobulbales;__	0	21	10	0	2	0	0	0	0	5	0	0
d__Bacteria;p__Firmicutes;c__Desulfotomaculia;o__Desulfotomaculales;f__Desulfotomaculales	0	0	29	8	0	23	0	25	5	12	2	32
d__Bacteria;p__Chloroflexi;c__Anaerolineae;o__SBR1031;f__A4b	0	8	0	0	0	15	0	2	0	21	0	3
d__Bacteria;p__Bacteroidota;c__Ignavibacteria;o__Ignavibacteriales;f__Ignavibacteriaceae	0	0	0	0	0	0	0	0	11	0	21	0
d__Bacteria;p__Patescibacteria;c__CPR2;o__CPR2;f__CPR2	7	4	15	0	5	0	15	3	4	7	27	0
d__Bacteria;p__Elusimicrobiota;c__Lineage_IIa;o__Lineage_IIa;f__Lineage_IIa	0	0	0	0	0	21	0	6	0	10	0	12
d__Bacteria;p__Proteobacteria;c__Alphaproteobacteria;o__Rhodospirillales;__	0	5	0	0	0	0	0	20	0	12	0	5
d__Bacteria;p__Myxococcota;c__Polyangia;o__Polyangiales;__	3	20	15	4	15	36	5	4	10	23	7	32
d__Bacteria;p__Proteobacteria;c__Gammaproteobacteria;o__Salinisphaerales;f__Solimonadaceae	0	6	5	0	0	10	2	0	0	20	0	0
d__Bacteria;p__Bacteroidota;c__Bacteroidia;o__Cytophagales;f__Microscillaceae	0	6	0	5	9	22	7	0	5	41	0	12
d__Bacteria;p__Acidobacteriota;c__Subgroup_18;o__Subgroup_18;f__Subgroup_18	0	8	7	0	18	18	0	0	3	6	20	18
d__Bacteria;p__Patescibacteria;c__Parcubacteria;o__Candidatus_Wolfebacteria;f__Candidatus_Wolfebacteria	9	2	10	3	8	20	9	13	12	17	12	12
d__Bacteria;p__Verrucomicrobiota;c__Verrucomicrobiae;o__Chthoniobacterales;f__Xiphinematobacteraceae	0	0	4	0	14	34	10	5	0	0	8	0
d__Bacteria;p__Proteobacteria;c__Alphaproteobacteria;o__Rhizobiales;f__Kaistiaceae	0	0	0	0	0	0	25	0	0	4	0	0
d__Bacteria;p__Proteobacteria;c__Alphaproteobacteria;o__Rhizobiales;f__KF-JG30-B3	0	0	0	4	2	0	0	0	16	19	2	8
d__Bacteria;p__Bacteroidota;c__Bacteroidia;o__Sphingobacteriales;f__Lentimicrobiaceae	0	0	0	0	19	10	0	0	0	0	12	8
d__Bacteria;p__Myxococcota;c__Myxococcia;o__Myxococcales;__	3	5	4	7	39	12	0	0	2	4	0	7
d__Bacteria;p__Proteobacteria;c__Gammaproteobacteria;o__R7C24;f__R7C24	0	0	0	0	0	19	0	0	0	0	4	0
d__Bacteria;p__Actinobacteriota;c__Actinobacteria;o__Micrococcales;__	3	6	7	3	2	23	0	3	0	9	3	0
d__Bacteria;p__Firmicutes;c__Clostridia;o__Clostridia_UCG-014;f__Clostridia_UCG-014	0	3	8	0	0	4	6	9	5	28	0	0
d__Bacteria;p__Chloroflexi;__;__;__	14	0	30	5	3	24	18	6	16	26	35	12
d__Bacteria;p__Firmicutes;c__Bacilli;o__RF39;f__RF39	5	11	21	10	13	14	11	17	17	3	54	17
d__Bacteria;p__Firmicutes;c__Negativicutes;o__uncultured;f__uncultured	5	7	7	0	5	17	7	6	9	8	22	10
d__Archaea;p__Euryarchaeota;c__Methanobacteria;o__Methanobacteriales;f__Methanobacteriaceae	0	0	7	0	6	26	11	3	30	7	19	3

Bacteria taxonomy	R1_A SR5i	R2_A SR5i	R3_A SR5i	R4_A SR5i	R5_A SR5i	R6_A SR5i	R1_ WT	R2_ WT	R3_ WT	R4_ WT	R5_ WT	R6_ WT
d__Bacteria;p__Firmicutes;c__Bacilli;o__Erysipelotrichales;f__Erysipelotrichaceae	16	3	0	0	0	3	0	0	8	0	0	0
d__Bacteria;p__Desulfobacterota;c__Syntrophia;o__Syntrophales;f__Syntrophaceae	0	3	2	0	0	0	0	3	10	16	5	0
d__Bacteria;p__Firmicutes;c__Syntrophomonadia;o__Syntrophomonadales;f__Syntroph omonadaceae	6	0	8	0	23	18	2	0	16	4	8	15
d__Bacteria;p__Actinobacteriota;c__Actinobacteria;o__Corynebacteriales;f__Nocardiace ae	5	4	9	10	19	14	11	0	3	8	7	11
d__Bacteria;p__Bacteroidota;c__Bacteroidia;o__Sphingobacteriales;f__CWT_CU03-E12	6	0	7	10	2	0	5	7	8	18	16	25
d__Bacteria;p__Bacteroidota;c__Bacteroidia;o__Chitinophagales;f__uncultured	0	0	0	5	4	12	16	0	4	9	16	8
d__Bacteria;p__Proteobacteria;c__Gammaproteobacteria;o__Pseudomonadales;f__Morax ellaceae	0	4	2	10	0	14	0	0	12	9	16	21
d__Bacteria;p__Firmicutes;c__Negativicutes;o__Veillonellales-Selenomonadales;__	2	0	0	6	5	22	0	0	6	0	19	2
d__Bacteria;p__Acidobacteriota;c__Holophagae;o__Subgroup_7;f__Subgroup_7	9	4	14	0	2	30	0	4	0	12	0	9
d__Bacteria;p__Firmicutes;c__Bacilli;o__Bacillales;f__Planococcaceae	2	14	14	11	0	23	7	0	0	2	10	12
d__Bacteria;p__Patescibacteria;c__Parcubacteria;o__Candidatus_Moranbacteria;f__Cand idatus_Moranbacteria	0	15	5	3	10	50	13	15	11	13	8	16
d__Bacteria;p__Actinobacteriota;c__Actinobacteria;o__Frankiales;__	0	0	5	15	0	0	0	0	0	0	0	0
d__Bacteria;p__Firmicutes;c__Negativicutes;o__Veillonellales- Selenomonadales;f__uncultured	0	4	0	0	6	15	4	0	5	3	7	0
d__Bacteria;p__Desulfobacterota;c__Desulfomonilia;o__Desulfomonilales;f__Desulfom onilaceae	0	3	11	8	0	18	5	0	0	6	2	4
d__Bacteria;p__Patescibacteria;c__Parcubacteria;o__Candidatus_Yanofskybacteria;f__C andidatus_Yanofskybacteria	7	19	19	3	4	19	4	10	3	6	8	5
d__Bacteria;p__Bacteroidota;c__Bacteroidia;__;__	11	37	4	25	18	42	2	25	28	18	26	27
d__Bacteria;p__Sva0485;c__Sva0485;o__Sva0485;f__Sva0485	2	3	2	2	0	14	2	4	2	18	9	0
d__Bacteria;p__Gemmatimonadota;c__S0134_terrestrial_group;o__S0134_terrestrial_gr oup;f__S0134_terrestrial_group	0	0	6	0	0	15	0	0	6	8	5	2
d__Bacteria;p__Proteobacteria;c__Gammaproteobacteria;o__Burkholderiales;f__A21b	8	0	2	0	4	13	0	3	0	0	0	0
d__Bacteria;p__Myxococcota;c__bacteriap25;o__bacteriap25;f__bacteriap25	0	0	0	2	0	13	4	6	7	2	0	0
d__Bacteria;p__Firmicutes;c__Bacilli;__;__	7	2	3	5	3	28	3	6	2	2	0	14
d__Bacteria;p__Actinobacteriota;c__Actinobacteria;o__Catenulisporales;f__Catenulispor aceae	0	7	0	7	0	12	12	7	0	0	9	23
d__Bacteria;p__Chloroflexi;c__Anaerolineae;o__SBR1031;f__SBR1031	0	11	3	5	0	15	3	23	4	8	6	19

Bacteria taxonomy	R1_A SR5i	R2_A SR5i	R3_A SR5i	R4_A SR5i	R5_A SR5i	R6_A SR5i	R1_ WT	R2_ WT	R3_ WT	R4_ WT	R5_ WT	R6_ WT
d__Bacteria;p__Fibrobacterota;c__Fibrobacteria;o__Fibrobacterales;f__B122	2	2	6	6	0	15	3	6	12	0	13	13
d__Archaea;p__Nanoarchaeota;c__Nanoarchaeia;o__Woesearchaeales;f__GW2011_GWC1_47_15	0	0	0	0	3	5	0	10	4	12	4	3
d__Bacteria;p__Firmicutes;c__Clostridia;o__Thermincolales;f__Thermincolaceae	0	3	3	0	6	17	5	7	6	12	9	0
d__Bacteria;p__Proteobacteria;c__Alphaproteobacteria;o__Defluviicoccales;f__uncultured	0	0	0	0	0	0	0	0	0	0	12	5
d__Bacteria;p__Armatimonadota;c__Armatimonadia;o__Armatimonadales;f__Armatimonadales	0	0	3	0	0	3	4	2	0	0	15	4
d__Bacteria;p__Chloroflexi;c__TK10;o__TK10;f__TK10	13	0	5	8	0	20	0	7	0	0	4	2
d__Bacteria;p__Desulfobacterota;c__Syntrophorhabdia;o__Syntrophorhabdales;f__Syntrophorhabdaceae	5	9	15	4	13	25	12	4	17	28	6	19
d__Bacteria;p__Actinobacteriota;c__Acidimicrobiia;o__Microtrichales;__	11	4	5	0	0	0	0	0	7	0	0	5
d__Bacteria;p__Actinobacteriota;c__Actinobacteria;o__Propionibacteriales;f__Propionibacteriaceae	0	0	3	3	6	0	5	4	16	0	0	0
d__Bacteria;p__Desulfobacterota;c__Desulfobulbia;o__Desulfobulbales;f__Desulfurivibrionaceae	0	0	0	3	0	3	0	0	0	11	0	3
d__Bacteria;p__Proteobacteria;c__Alphaproteobacteria;o__Paracaedibacterales;f__Paracaedibacteraceae	0	0	7	2	3	10	5	4	0	0	6	12
d__Bacteria;p__Proteobacteria;c__Gammaproteobacteria;o__Legionellales;f__Legionellaceae	0	6	9	2	0	5	7	0	0	8	11	0
d__Bacteria;p__Firmicutes;c__Clostridia;o__Oscillospirales;f__Ethanoligenenaceae	0	0	10	0	3	5	4	5	18	0	11	8
d__Bacteria;p__Chloroflexi;c__Ktedonobacteria;o__C0119;f__C0119	0	0	3	0	7	13	6	0	2	7	3	3
d__Bacteria;p__Bacteroidota;c__Kapabacteria;o__Kapabacteriales;f__Kapabacteriales	22	8	13	16	21	44	10	14	3	13	19	6
d__Bacteria;p__Cyanobacteria;c__Sericytochromatia;o__Sericytochromatia;f__Sericytochromatia	2	0	3	0	7	16	4	13	2	5	2	3
d__Bacteria;p__Planctomycetota;c__Phycisphaerae;o__Tepidisphaerales;f__CPla-3_termite_group	0	0	0	0	5	14	0	0	2	0	0	5
d__Bacteria;p__Proteobacteria;c__Alphaproteobacteria;o__Elsterales;f__URHD0088	0	0	0	0	0	11	0	0	0	0	0	0
d__Bacteria;p__Proteobacteria;c__Alphaproteobacteria;o__Ferrovibrionales;f__Ferrovibrionales	3	0	0	2	4	15	0	0	0	0	0	0
d__Bacteria;p__Actinobacteriota;c__Actinobacteria;o__Streptosporangiales;f__Thermomonosporaceae	0	3	0	0	9	11	3	3	0	3	4	0
d__Archaea;p__Crenarchaeota;c__Nitrososphaeria;o__Nitrosotaleales;f__Nitrosotaleaceae	0	0	0	0	0	9	0	4	0	7	7	11

Bacteria taxonomy	R1_A SR5i	R2_A SR5i	R3_A SR5i	R4_A SR5i	R5_A SR5i	R6_A SR5i	R1_ WT	R2_ WT	R3_ WT	R4_ WT	R5_ WT	R6_ WT
d__Bacteria;p__Actinobacteriota;c__Actinobacteria;o__Propionibacteriales;f__Nocardioidaceae	0	4	0	0	0	3	6	0	0	10	2	11
d__Bacteria;p__Myxococcota;__;__;__	7	15	4	9	10	13	3	6	9	0	4	13
d__Bacteria;p__Firmicutes;c__Clostridia;o__uncultured;f__uncultured	0	5	0	4	0	0	10	0	0	0	0	2
d__Bacteria;p__Chloroflexi;c__AD3;o__AD3;f__AD3	0	0	0	2	3	15	6	10	0	2	10	2
d__Bacteria;p__Acidobacteriota;c__Acidobacteriae;o__AKIW659;f__AKIW659	0	0	0	0	0	0	0	10	0	0	0	0
d__Bacteria;p__Proteobacteria;c__Gammaproteobacteria;o__Coxiellales;f__Coxiellaceae	3	9	10	7	4	8	11	2	0	0	11	12
d__Bacteria;p__Cyanobacteria;c__Vampirivibrionia;o__Gastranaerophilales;f__Gastranaerophilales	0	17	8	15	14	25	17	10	19	0	22	20
d__Bacteria;p__Proteobacteria;c__Alphaproteobacteria;o__Caulobacterales;f__Hyphomonadaceae	0	0	0	0	0	0	0	0	20	19	0	3
d__Bacteria;p__Patescibacteria;c__Gracilibacteria;o__Absconditabacteriales_(SR1);f__Absconditabacteriales_(SR1)	0	2	6	0	18	0	0	0	0	0	0	0
d__Bacteria;p__Firmicutes;c__Bacilli;o__Bacillales;__	5	2	0	6	0	11	5	4	0	16	10	15
d__Bacteria;p__Proteobacteria;c__Alphaproteobacteria;o__Holosporales;f__Holosporaceae	0	0	0	0	4	10	0	0	0	0	10	0
d__Bacteria;p__Desulfobacterota;c__Syntrophia;o__Syntrophales;f__uncultured	4	0	0	2	0	10	4	0	0	11	0	8
d__Bacteria;p__Acidobacteriota;c__Subgroup_5;o__Subgroup_5;f__Subgroup_5	0	0	5	0	0	10	0	0	0	0	0	0
d__Bacteria;p__Firmicutes;c__Clostridia;o__Oscillospirales;__	0	0	0	3	0	0	0	4	6	0	3	10
d__Bacteria;p__Planctomycetota;c__Phycisphaerae;o__Tepidisphaerales;f__WD2101_soil_group	2	0	3	7	2	18	3	12	2	7	16	18
d__Bacteria;p__Fibrobacterota;c__Fibrobacteria;o__Fibrobacterales;__	0	3	0	4	0	4	2	5	0	2	2	10
d__Bacteria;p__Chloroflexi;c__Ktedonobacteria;__;__	9	0	11	0	0	0	0	0	6	0	0	0
d__Bacteria;p__Actinobacteriota;c__Thermoleophilia;__;__	9	0	0	0	0	3	2	0	0	5	0	0
d__Bacteria;p__Chloroflexi;c__OLB14;o__OLB14;f__OLB14	4	3	0	0	3	6	9	2	9	4	0	3
d__Bacteria;p__SAR324_clade(Marine_group_B);c__SAR324_clade(Marine_group_B);o__SAR324_clade(Marine_group_B);f__SAR324_clade(Marine_group_B)	0	0	4	0	0	3	9	0	0	0	2	2
d__Bacteria;p__Bacteroidota;c__Bacteroidia;o__Sphingobacteriales;f__S15A-MN91	0	9	0	0	0	0	0	0	0	0	0	0
d__Bacteria;p__Cyanobacteria;c__Cyanobacteriia;o__Oxyphotobacteria_Incertae_Sedis;f__Unknown_Family	0	12	0	0	0	0	0	0	0	0	0	0
d__Bacteria;p__Firmicutes;c__Clostridia;o__Lachnospirales;f__Defluviitaleaceae	0	0	0	9	5	2	4	0	2	10	8	8
d__Bacteria;p__Bacteroidota;c__Bacteroidia;o__Cytophagales;f__Cytophagaceae	0	0	0	0	0	0	4	0	3	15	0	0

Bacteria taxonomy	R1_A SR5i	R2_A SR5i	R3_A SR5i	R4_A SR5i	R5_A SR5i	R6_A SR5i	R1_ WT	R2_ WT	R3_ WT	R4_ WT	R5_ WT	R6_ WT
d__Bacteria;p__Verrucomicrobiota;c__Chlamydiae;o__Chlamydiales;f__cvE6	0	0	3	0	7	11	0	7	2	0	9	0
d__Bacteria;p__Myxococcota;c__Polyangia;o__Polyangiales;f__Sandaracinaceae	4	0	7	5	0	0	15	6	3	9	19	11
d__Bacteria;p__Firmicutes;c__Bacilli;o__Bacillales;f__Sporolactobacillaceae	0	0	4	0	0	9	6	0	0	0	7	0
d__Bacteria;p__Proteobacteria;c__Alphaproteobacteria;o__Rickettsiales;f__SM2D12	5	4	12	3	2	18	15	11	7	8	9	9
d__Bacteria;p__Firmicutes;c__Clostridia;o__Monoglobales;f__Monoglobaceae	0	0	0	0	10	0	2	0	4	0	0	11
d__Bacteria;p__Proteobacteria;c__Alphaproteobacteria;o__Rhizobiales;f__Amb-16S-1323	0	0	0	0	0	0	8	0	0	0	0	0
d__Bacteria;p__Actinobacteriota;c__Actinobacteria;o__Micrococcales;f__Micrococcaceae	0	0	4	0	0	0	8	0	4	0	4	5
d__Bacteria;p__Patescibacteria;c__Berkelbacteria;o__Berkelbacteria;f__Berkelbacteria	8	0	6	6	11	21	2	5	23	11	5	20
d__Bacteria;p__Proteobacteria;c__Gammaproteobacteria;o__Acidiferrobacterales;f__Acidiferrobacteraceae	0	0	3	0	0	0	0	0	0	12	0	0
d__Bacteria;p__WS4;c__WS4;o__WS4;f__WS4	0	7	7	0	0	7	0	4	0	0	8	8
d__Bacteria;p__Verrucomicrobiota;c__Chlamydiae;o__Chlamydiales;f__Simkaniaceae	3	2	0	0	2	13	0	3	2	5	7	0
d__Bacteria;p__Myxococcota;c__Polyangia;__;__	0	8	7	10	4	44	0	9	4	11	8	14
d__Bacteria;p__Patescibacteria;c__ABY1;o__Candidatus_Falkowbacteria;f__Candidatus_Falkowbacteria	0	0	0	0	0	5	4	0	4	0	4	10
d__Bacteria;p__Firmicutes;c__Desulfotomaculia;o__Desulfotomaculales;f__Desulfallas-Sporotomaculum	0	0	0	0	0	0	0	0	0	0	0	8
d__Bacteria;p__Bacteroidota;c__Bacteroidia;o__Cytophagales;f__Amoebophilaceae	7	3	5	0	0	8	0	4	3	0	0	6
d__Bacteria;p__Actinobacteriota;c__Thermoleophilia;o__Solirubrobacterales;__	0	0	0	0	0	3	7	5	0	0	0	0
d__Archaea;p__Thermoplasmatota;c__Thermoplasmata;o__Methanomassiliicoccales;f__Methanomassiliicoccaceae	0	7	0	0	0	2	0	3	5	0	0	0
d__Bacteria;p__Cyanobacteria;c__Vampirivibrionia;o__Obscuribacterales;f__Obscuribacteraceae	3	7	6	0	0	8	0	0	8	3	8	10
d__Bacteria;p__Firmicutes;c__Clostridia;o__Oscillospirales;f__[Eubacterium]_coprostanoligenes_group	4	0	4	0	6	0	0	0	10	0	0	0
d__Bacteria;p__Proteobacteria;c__Alphaproteobacteria;o__Rhizobiales;f__uncultured	0	0	0	0	0	0	0	0	7	0	0	0
d__Bacteria;p__Verrucomicrobiota;c__Chlamydiae;o__Chlamydiales;f__Parachlamydiaceae	0	0	4	0	12	7	2	2	2	3	8	0
d__Bacteria;p__Patescibacteria;c__ABY1;o__Candidatus_Kerfeldbacteria;f__Candidatus_Kerfeldbacteria	3	0	0	0	0	7	2	0	0	0	0	0
d__Bacteria;p__Gemmatimonadota;__;__;__	0	0	6	0	3	12	2	0	0	5	0	2

Bacteria taxonomy	R1_ASR5i	R2_ASR5i	R3_ASR5i	R4_ASR5i	R5_ASR5i	R6_ASR5i	R1_WT	R2_WT	R3_WT	R4_WT	R5_WT	R6_WT
d__Bacteria;p__Planctomycetota;c__Planctomycetes;o__Gemmatales;f__Gemmataceae	4	0	3	0	8	12	4	0	8	6	6	4
d__Bacteria;p__Sumerlaeota;c__Sumerlaeia;o__Sumerlaeales;f__Sumerlaeaceae	8	3	9	0	6	0	0	0	0	3	3	9
Unassigned;__;__;__;__	3	3	0	0	0	0	6	4	0	0	0	7
d__Bacteria;p__Firmicutes;c__Desulfitobacteriia;o__Desulfitobacteriales;f__Heliobacteriaceae	2	0	3	0	0	0	0	0	0	0	0	7
d__Bacteria;p__Firmicutes;c__Clostridia;o__Oscillospirales;f__UCG-010	0	4	0	0	0	0	0	0	0	0	15	9
d__Bacteria;p__Dependentiae;c__Babeliae;o__Babeliales;f__Vermiphilaceae	6	7	4	0	6	0	0	4	4	4	10	5
d__Bacteria;p__Chloroflexi;c__Ktedonobacteria;o__B10-SB3A;f__B10-SB3A	4	0	0	0	0	4	6	0	0	0	0	0
d__Archaea;p__Nanoarchaeota;c__Nanoarchaeia;o__Woesearchaeales;f__Woesearchaeales	7	0	0	2	0	6	3	8	2	5	0	3
d__Bacteria;p__Planctomycetota;c__Phycisphaerae;o__Phycisphaerales;f__AKAU3564_sediment_group	0	4	4	0	0	9	0	9	4	0	0	0
d__Bacteria;p__Hydrogenedentes;c__Hydrogenedentia;o__Hydrogenedentiales;f__Hydrogenedensaceae	2	3	8	0	0	9	0	4	0	0	3	3
d__Archaea;p__Halobacterota;c__Methanomicrobia;o__Methanomicrobiales;f__Methanoregulaceae	0	0	0	0	0	0	0	0	6	3	0	0
d__Bacteria;p__Chloroflexi;c__Anaerolineae;o__ADurb.Bin180;f__ADurb.Bin180	0	0	0	0	0	0	0	0	0	6	3	0
d__Bacteria;p__Bacteroidota;c__Bacteroidia;o__Chitinophagales;f__37-13	4	0	0	0	6	9	0	0	0	0	2	2
d__Bacteria;p__Verrucomicrobiota;c__Verrucomicrobiae;o__B29;f__B29	0	0	0	0	6	0	0	0	0	0	0	0
d__Bacteria;p__Firmicutes;c__Clostridia;o__Oscillospirales;f__UCG-011	0	0	0	0	6	0	0	0	0	0	0	0
d__Bacteria;p__Bdellovibrionota;c__Oligoflexia;o__Oligoflexales;f__uncultured	0	4	0	3	6	5	0	0	0	0	12	2
d__Bacteria;p__Proteobacteria;c__Gammaproteobacteria;o__Enterobacterales;f__Yersiniaceae	0	0	0	0	0	0	0	0	0	0	6	0
d__Bacteria;p__Firmicutes;c__Bacilli;o__Lactobacillales;f__Lactobacillaceae	0	0	0	0	0	0	0	0	0	0	6	0
d__Bacteria;p__Actinobacteriota;c__Actinobacteria;o__Streptosporangiales;f__Streptosporangiaceae	2	0	0	0	0	0	0	0	0	0	6	0
d__Bacteria;p__Firmicutes;c__Clostridia;o__Peptostreptococcales-Tissierellales;f__Sedimentibacteraceae	0	0	0	0	0	6	0	0	0	3	0	0
d__Bacteria;p__Acidobacteriota;c__Blastocatellia;o__Blastocatellales;f__Blastocatellaceae	2	0	0	0	0	6	0	0	0	0	0	0
d__Bacteria;p__Bacteroidota;c__Bacteroidia;o__Sphingobacteriales;f__NS11-12_marine_group	0	0	0	2	0	6	0	0	0	0	0	0
d__Bacteria;p__Armatimonadota;__;__;__	0	0	0	0	0	6	0	0	0	0	0	0

Bacteria taxonomy	R1_A SR5i	R2_A SR5i	R3_A SR5i	R4_A SR5i	R5_A SR5i	R6_A SR5i	R1_ WT	R2_ WT	R3_ WT	R4_ WT	R5_ WT	R6_ WT
d__Bacteria;p__Desulfobacterota;c__uncultured;o__uncultured;f__uncultured	0	0	4	0	0	5	4	3	0	0	0	14
d__Bacteria;p__Methylomirabilota;c__Methylomirabilia;o__Methylomirabilales;f__Methylomirabilaceae	0	0	0	0	0	0	0	2	0	3	0	6
d__Bacteria;p__Firmicutes;c__Desulfitobacteriia;o__Desulfitobacteriales;__	0	0	0	0	3	3	0	0	0	0	2	6
d__Bacteria;p__Firmicutes;c__Clostridia;o__Peptococcales;f__Peptococcaceae	7	0	0	0	0	2	0	0	2	0	0	0
d__Bacteria;p__Desulfobacterota;c__Desulfobacteria;o__Desulfatiglandales;f__Desulfatiglandaceae	5	7	0	3	5	0	0	4	0	11	6	4
d__Bacteria;p__Bacteroidota;c__Bacteroidia;o__Chitinophagales;__	3	0	0	0	0	8	5	0	2	3	0	5
d__Bacteria;p__Chloroflexi;c__SHA-26;o__SHA-26;f__SHA-26	5	0	0	0	7	7	5	0	0	4	4	0
d__Archaea;p__Halobacterota;c__Methanomicrobia;o__Methanomicrobiales;f__Rice_Cluster_II	0	0	0	0	0	0	5	0	0	0	0	0
d__Bacteria;p__Patescibacteria;c__Parcubacteria;o__Candidatus_Nomurabacteria;f__Candidatus_Nomurabacteria	0	0	0	0	0	3	0	10	0	0	5	0
d__Bacteria;p__Verrucomicrobiota;c__Kiritimatiellae;o__WCHB1-41;f__WCHB1-41	2	0	0	0	0	0	0	8	0	0	2	0
d__Archaea;p__Thermoplasmatota;c__Thermoplasmata;o__Methanomassiliicoccales;f__uncultured	0	0	0	0	0	0	0	5	0	0	0	0
d__Bacteria;p__Deferrisomatota;c__Defferrisomatia;o__Defferrisomatales;f__Defferrisomataceae	0	0	5	0	0	0	0	0	0	0	0	0
d__Bacteria;p__Chloroflexi;c__JG30-KF-CM66;o__JG30-KF-CM66;f__JG30-KF-CM66	0	0	5	0	0	11	4	0	0	0	2	0
d__Bacteria;p__Proteobacteria;c__Gammaproteobacteria;o__Methylococcales;f__Methylococcaceae	0	0	5	0	0	0	0	0	0	5	0	3
d__Bacteria;p__Proteobacteria;c__Gammaproteobacteria;o__Enterobacterales;__	0	0	0	0	0	0	0	0	7	0	0	0
d__Bacteria;p__Armatimonadota;c__uncultured;o__uncultured;f__uncultured	0	0	2	5	0	5	0	2	0	0	2	0
d__Bacteria;p__Firmicutes;c__Bacilli;o__Staphylococcales;f__Staphylococcaceae	0	0	0	3	5	0	0	0	0	5	0	0
d__Bacteria;p__Actinobacteriota;c__Acidimicrobiia;o__Microtrichales;f__Microtrichaceae	0	0	0	0	0	0	0	0	0	5	0	0
d__Bacteria;p__Patescibacteria;c__Parcubacteria;o__Candidatus_Liptonbacteria;f__Candidatus_Liptonbacteria	0	0	0	0	0	0	0	0	0	5	0	0
d__Bacteria;p__Planctomycetota;c__Planctomycetes;o__Pirellulales;f__Pirellulaceae	0	0	0	0	2	0	0	0	0	4	5	3
d__Bacteria;p__Bacteroidota;c__Bacteroidia;o__Bacteroidales;f__Williamwhitmaniaceae	0	0	0	0	0	0	0	0	0	0	5	0
d__Bacteria;p__Patescibacteria;c__Microgenomatia;o__Candidatus_Shapirobacteria;f__Candidatus_Shapirobacteria	0	0	5	0	0	4	0	0	0	3	9	0
d__Bacteria;p__Proteobacteria;c__Alphaproteobacteria;o__Azospirillales;__	0	0	0	0	0	0	0	0	0	0	5	0

Bacteria taxonomy	R1_A SR5i	R2_A SR5i	R3_A SR5i	R4_A SR5i	R5_A SR5i	R6_A SR5i	R1_ WT	R2_ WT	R3_ WT	R4_ WT	R5_ WT	R6_ WT
d__Bacteria;p__Proteobacteria;c__Gammaproteobacteria;o__BD72BR169;f__BD72BR169	0	0	0	0	0	5	0	0	0	0	0	0
d__Bacteria;p__Elusimicrobiota;c__Lineage_IIb;o__Lineage_IIb;f__Lineage_IIb	0	0	0	0	4	5	0	0	0	0	3	0
d__Bacteria;p__Verrucomicrobiota;c__Verrucomicrobiae;o__Verrucomicrobiales;f__Verrucomicrobiaceae	0	0	0	0	0	5	0	0	0	0	0	0
d__Bacteria;p__Spirochaetota;__;__;__	0	0	2	0	0	5	0	3	0	0	3	0
d__Bacteria;p__Firmicutes;c__Bacilli;o__Brevibacillales;f__Brevibacillaceae	0	4	0	0	0	5	4	2	3	3	3	3
d__Bacteria;p__Actinobacteriota;c__Thermoleophilia;o__Gaiellales;__	0	0	0	0	0	8	0	0	3	0	0	0
d__Bacteria;p__Firmicutes;c__Firmicutes;o__Firmicutes;f__Firmicutes	0	0	0	0	4	5	0	0	0	0	0	0
d__Bacteria;p__Latescibacterota;c__Latescibacteria;o__Latescibacterales;f__Latescibacteraceae	0	0	0	0	0	4	0	6	0	2	0	5
d__Bacteria;p__Actinobacteriota;c__MB-A2-108;o__MB-A2-108;f__MB-A2-108	0	0	0	0	0	0	0	0	0	3	0	5
d__Bacteria;p__Desulfobacterota;c__Syntrophobacteria;o__Syntrophobacterales;f__uncultured	4	0	0	0	0	3	3	0	0	2	0	0
d__Bacteria;p__Planctomycetota;c__Phycisphaerae;o__Phycisphaerales;f__Phycisphaeraceae	0	0	0	0	0	2	4	0	0	0	0	0
d__Bacteria;p__Bdellovibrionota;c__Oligoflexia;o__Silvanigrellales;f__Silvanigrellaceae	0	4	0	0	0	0	0	0	0	3	0	0
d__Archaea;p__Crenarchaeota;c__Nitrososphaeria;o__Nitrososphaerales;f__Nitrososphaeraceae	0	4	0	0	0	0	0	0	0	0	0	0
d__Bacteria;p__Cyanobacteria;c__Vampirivibrionia;o__Vampirovibrionales;f__Vampirovibrionaceae	0	4	0	6	0	4	0	0	0	0	0	0
d__Bacteria;p__GAL15;c__GAL15;o__GAL15;f__GAL15	5	0	0	0	3	0	0	4	0	0	0	0
d__Archaea;p__Thermoplasmatota;c__Thermoplasmata;__;__	0	0	4	0	0	0	0	0	0	0	0	0
d__Bacteria;p__Proteobacteria;c__Alphaproteobacteria;o__Caulobacterales;__	0	0	4	0	0	0	0	0	0	0	0	0
d__Bacteria;p__Firmicutes;c__Clostridia;o__Eubacteriales;f__Anaerofustaceae	0	0	0	0	0	0	0	0	4	0	2	0
d__Bacteria;p__Verrucomicrobiota;c__Lentisphaeria;o__Victivallales;f__Victivallales	0	2	0	0	0	0	0	3	4	0	0	0
d__Bacteria;p__Firmicutes;c__Symbiobacteriia;o__Symbiobacteriales;f__Symbiobacteraceae	0	0	0	4	0	0	0	0	0	2	0	3
d__Bacteria;p__Proteobacteria;c__Alphaproteobacteria;o__Rickettsiales;f__AB1	0	3	0	4	0	0	0	0	0	0	0	0
d__Archaea;p__Halobacterota;c__Methanosarcinia;o__Methanosarciniales;f__Methanoperedenaceae	0	0	0	0	0	0	3	0	0	4	0	0
d__Bacteria;p__Firmicutes;c__Moorellia;o__Desulfitibacterales;f__Desulfitibacteraceae	0	0	0	0	0	0	0	0	0	7	0	0

Bacteria taxonomy	R1_A SR5i	R2_A SR5i	R3_A SR5i	R4_A SR5i	R5_A SR5i	R6_A SR5i	R1_ WT	R2_ WT	R3_ WT	R4_ WT	R5_ WT	R6_ WT
d__Bacteria;p__Verrucomicrobiota;c__Verrucomicrobiae;o__S-BQ2-57_soil_group;f__S-BQ2-57_soil_group	0	0	0	0	0	0	0	0	0	4	0	0
d__Bacteria;p__Proteobacteria;c__Gammaproteobacteria;o__CCD24;f__CCD24	0	0	0	0	0	0	0	0	0	4	4	0
d__Bacteria;p__Firmicutes;c__Clostridia;o__Lachnospirales;__	0	0	0	0	4	3	0	0	2	0	0	0
d__Bacteria;p__Firmicutes;c__Thermoanaerobacteria;o__Thermoanaerobacterales;f__SRB2	0	0	0	0	4	0	0	0	0	0	0	0
d__Bacteria;p__Actinobacteriota;c__Actinobacteria;o__Corynebacteriales;__	0	0	0	0	4	0	0	0	0	0	0	0
d__Bacteria;p__Desulfobacterota;c__Syntrophia;o__Syntrophales;__	0	0	0	0	4	0	0	0	0	0	0	0
d__Bacteria;p__Acidobacteriota;c__Thermoanaerobaculia;o__Thermoanaerobaculales;f__Thermoanaerobaculaceae	0	0	0	0	0	0	2	0	0	0	4	0
d__Bacteria;p__Cyanobacteria;__;__;__	0	0	0	0	0	4	0	0	0	0	0	2
d__Bacteria;p__Verrucomicrobiota;c__Verrucomicrobiae;__;__	0	0	0	0	0	4	0	3	0	0	0	0
d__Bacteria;p__Verrucomicrobiota;c__Verrucomicrobiae;o__Methylacidiphilales;f__Methylacidiphilaceae	0	0	0	0	3	0	0	0	0	0	0	4
d__Bacteria;p__Proteobacteria;c__Alphaproteobacteria;o__Rickettsiales;f__Rickettsiaceae	0	2	0	0	0	2	3	0	0	0	0	4
d__Bacteria;p__Chloroflexi;c__Dehalococcoidia;o__Dehalococcoidales;f__uncultured	0	3	0	0	3	0	0	0	0	5	4	4
d__Bacteria;p__Fibrobacterota;c__Chitinivibrionia;o__uncultured;f__uncultured	3	0	0	0	0	0	0	2	0	0	0	0
d__Bacteria;p__Chloroflexi;c__Dehalococcoidia;o__Dehalococcoidales;f__Dehalococcoidaceae	3	0	0	0	0	0	0	0	0	0	0	0
d__Bacteria;p__Firmicutes;c__BRH-c20a;o__BRH-c20a;f__BRH-c20a	3	0	0	0	0	0	0	0	0	0	0	2
d__Bacteria;p__Chloroflexi;c__Dehalococcoidia;o__S085;f__S085	3	0	0	0	0	2	2	3	0	0	0	5
d__Bacteria;p__Desulfobacterota;c__Desulfobacteria;__;__	3	0	0	0	0	0	0	0	0	0	0	0
d__Bacteria;p__Firmicutes;c__Bacilli;o__Entomoplasmatales;f__type_III	0	0	0	0	0	0	3	0	0	0	0	0
d__Bacteria;p__Firmicutes;c__Bacilli;o__Lactobacillales;f__Streptococcaceae	0	0	0	0	0	0	3	0	0	0	0	0
d__Bacteria;p__Acidobacteriota;c__Blastocatellia;o__Pyrinomonadales;f__Pyrinomonadaceae	0	0	0	0	0	0	3	0	0	0	0	0
d__Bacteria;p__Dependentiae;c__Babeliae;o__Babeliales;__	0	4	0	2	0	4	5	4	2	2	0	2
d__Bacteria;p__Nitrospirota;__;__;__	0	3	0	0	0	0	0	0	0	0	0	0
d__Bacteria;p__Chloroflexi;c__Ktedonobacteria;o__B12-WMSP1;f__B12-WMSP1	0	3	0	0	0	0	0	0	0	0	0	0
d__Bacteria;p__Verrucomicrobiota;c__Chlamydiae;o__Chlamydiales;__	0	3	0	5	0	2	0	0	4	3	0	0

Bacteria taxonomy	R1_ASR5i	R2_ASR5i	R3_ASR5i	R4_ASR5i	R5_ASR5i	R6_ASR5i	R1_WT	R2_WT	R3_WT	R4_WT	R5_WT	R6_WT
d__Archaea;p__Halobacterota;c__Methanosarcinia;o__Methanosarciniales;f__Methanosaetaceae	0	0	2	0	0	0	0	3	0	2	0	0
d__Bacteria;p__Patescibacteria;c__Parcubacteria;o__Candidatus_Staskawiczbacteria;f__Candidatus_Staskawiczbacteria	0	0	0	0	0	0	0	3	0	0	0	0
d__Bacteria;p__Proteobacteria;c__Alphaproteobacteria;o__Parvibaculales;f__Parvibaculaceae	0	0	2	0	0	0	0	3	0	0	3	0
d__Bacteria;p__MBNT15;c__MBNT15;o__MBNT15;f__MBNT15	0	0	0	0	0	3	2	3	0	0	0	0
d__Bacteria;p__Bacteroidota;c__Bacteroidia;o__Cytophagales;f__Spirosomaceae	0	0	3	0	0	0	0	0	0	0	0	0
d__Bacteria;p__Gemmatimonadota;c__Longimicrobia;o__Longimicrobiales;f__Longimicrobiaceae	0	0	3	0	0	0	0	0	0	0	0	0
d__Bacteria;p__Bdellovibrionota;__;__;__	0	0	3	0	0	2	0	0	0	0	0	2
d__Bacteria;p__Bacteroidota;c__Bacteroidia;o__Bacteroidales;f__Bacteroidetes_BD2-2	2	0	3	0	3	0	0	0	0	0	0	3
d__Bacteria;p__Firmicutes;c__Clostridia;o__Peptostreptococcales-Tissierellales;f__Peptostreptococcales-Tissierellales	0	0	5	0	0	0	0	0	0	0	0	0
d__Bacteria;p__Firmicutes;c__Symbiobacteriia;o__Symbiobacteriales;__	0	0	0	0	0	0	0	0	3	0	0	0
d__Bacteria;p__Patescibacteria;c__Dojkabacteria;o__Dojkabacteria;f__Dojkabacteria	0	0	0	0	0	0	0	0	3	0	0	0
d__Bacteria;p__Patescibacteria;c__WWE3;o__WWE3;f__WWE3	0	2	0	0	0	0	0	0	3	0	0	0
d__Bacteria;p__Chloroflexi;c__Chloroflexia;o__Thermomicrobiales;f__Thermomicrobiaceae	0	0	0	0	0	0	0	0	3	0	0	0
d__Bacteria;p__Campilobacterota;c__Campylobacteria;o__Campylobacterales;__	0	0	0	3	0	0	0	0	0	0	0	0
d__Bacteria;p__Actinobacteriota;c__Coriobacteriia;__;__	0	0	0	3	0	0	0	0	0	0	0	0
d__Bacteria;p__Chloroflexi;c__Anaerolineae;o__SBR1031;__	0	0	0	0	0	0	0	0	0	3	0	3
d__Bacteria;p__Bacteroidota;c__Bacteroidia;o__Sphingobacteriales;f__env.OPS_17	0	0	0	0	0	0	0	4	0	6	0	0
d__Bacteria;p__Cyanobacteria;c__Vampirivibrionia;o__Vampirovibrionales;f__Vampirovibrionales	0	0	0	0	0	0	0	0	0	3	0	0
d__Bacteria;p__Dependentiae;c__Babeliae;o__Babeliales;f__Babeliales	0	2	0	0	3	0	0	0	0	2	2	0
d__Bacteria;p__Actinobacteriota;c__Acidimicrobiia;o__Microtrichales;f__Ilumatobacteraceae	0	0	0	0	2	0	0	0	2	0	3	0
d__Bacteria;p__Patescibacteria;c__ABY1;__;__	0	0	0	0	0	3	0	0	0	0	0	0
d__Bacteria;p__Planctomycetota;c__Phycisphaerae;o__Tepidisphaerales;f__Tepidisphaeraceae	0	0	0	0	0	3	0	0	0	0	2	0
d__Bacteria;p__Firmicutes;c__D8A-2;o__D8A-2;f__D8A-2	0	0	0	0	0	3	0	2	0	2	0	0

**Bacteria taxonomy**	**R1_A SR5i**	**R2_A SR5i**	**R3_A SR5i**	**R4_A SR5i**	**R5_A SR5i**	**R6_A SR5i**	**R1_ WT**	**R2_ WT**	**R3_ WT**	**R4_ WT**	**R5_ WT**	**R6_ WT**
d__Bacteria;p__Chloroflexi;c__Anaerolineae;o__Anaerolineae;f__Anaerolineae	0	0	0	0	0	3	0	0	0	0	0	0
d__Bacteria;p__Bacteroidota;c__Bacteroidia;o__Sphingobacteriales;f__AKYH767	0	0	0	0	0	3	0	0	0	2	0	0
d__Bacteria;p__Proteobacteria;c__Alphaproteobacteria;o__Rhizobiales;f__A0839	0	0	0	0	0	3	0	0	0	0	0	0
d__Bacteria;p__Planctomycetota;__;__;__	0	0	0	0	0	3	0	0	0	0	0	0
d__Bacteria;p__Nitrospirota;c__Thermodesulfovibrionia;__;__	0	0	0	0	0	3	0	0	0	0	0	0
d__Bacteria;p__Proteobacteria;c__Alphaproteobacteria;o__Defluviicoccales;f__Defluviicoccaceae	0	0	0	0	0	3	0	0	0	0	0	0
d__Archaea;p__Halobacterota;c__Halobacteria;o__Halobacterales;f__Haloadaptaceae	0	0	0	0	0	0	0	0	0	0	0	3
d__Bacteria;p__Firmicutes;c__Clostridia;o__Clostridia_vadinBB60_group;f__Clostridia_vadinBB60_group	0	0	0	0	2	0	0	0	0	0	0	3
d__Bacteria;p__Proteobacteria;c__Gammaproteobacteria;o__Burkholderiales;f__Neisseriaceae	2	0	0	0	0	0	0	0	2	2	0	0
d__Bacteria;p__Firmicutes;c__Desulfitobacteriia;o__Desulfitobacteriales;f__Syntrophobotulaceae	2	0	0	0	0	0	0	0	0	0	0	0
d__Bacteria;p__Chloroflexi;c__Dehalococcoidia;o__GIF3;f__GIF3	0	2	0	0	0	0	2	0	0	0	0	0
d__Bacteria;p__Bacteroidota;c__Bacteroidia;o__Bacteroidales;f__Rikenellaceae	0	2	0	0	0	0	0	0	0	0	0	0
d__Bacteria;p__Cyanobacteria;c__Cyanobacteriia;o__Cyanobacteriales;f__Nostocaceae	0	2	0	0	0	0	0	0	0	0	0	0
d__Bacteria;p__Acidobacteriota;c__Acidobacteriae;o__Subgroup_12;f__Subgroup_12	0	2	0	0	0	0	0	0	0	2	0	0
d__Bacteria;p__Proteobacteria;c__Gammaproteobacteria;o__Cellvibrionales;f__Cellvibrionaceae	0	2	0	0	0	0	0	0	0	0	0	0
d__Bacteria;p__Actinobacteriota;c__Actinobacteria;o__Micrococcales;f__AKAU3644	0	2	0	0	0	0	0	0	0	0	0	0
d__Bacteria;p__Entotheonellaeota;c__Entotheonellia;o__Entotheonellales;f__Entotheonellaceae	0	0	0	0	0	0	0	2	0	2	0	0
d__Bacteria;p__Proteobacteria;c__Gammaproteobacteria;o__PLTA13;f__PLTA13	0	0	0	0	0	0	0	2	0	0	0	0
d__Bacteria;p__Methylomirabilota;c__Methylomirabilia;o__Rokubacteriales;f__Rokubacteriales	0	0	2	0	0	0	0	0	0	0	0	0
d__Bacteria;p__Planctomycetota;c__Planctomycetes;o__Planctomycetales;f__uncultured	0	0	2	0	0	0	0	0	0	0	0	0
d__Bacteria;p__Myxococcota;c__Myxococcia;o__Myxococcales;f__Vulgatibacteraceae	0	0	2	0	0	0	0	0	0	0	0	0
d__Bacteria;p__Actinobacteriota;c__Acidimicrobiia;o__Microtrichales;f__uncultured	0	0	2	0	0	0	0	0	0	0	0	0
d__Bacteria;p__Spirochaetota;c__Brevinematia;o__Brevinematales;f__Brevinemataceae	0	0	0	0	2	2	0	0	2	0	0	0
d__Bacteria;p__Patescibacteria;c__Gracilibacteria;o__Candidatus_Peregrinibacteria;f__Candidatus_Peregrinibacteria	0	0	0	0	0	0	0	0	2	0	0	0

Bacteria taxonomy	R1_A SR5i	R2_A SR5i	R3_A SR5i	R4_A SR5i	R5_A SR5i	R6_A SR5i	R1_ WT	R2_ WT	R3_ WT	R4_ WT	R5_ WT	R6_ WT
d__Bacteria;p__Armatimonadota;c__DG-56;o__DG-56;f__DG-56	0	0	0	0	0	0	0	0	2	0	0	0
d__Bacteria;p__Firmicutes;c__Negativicutes;__;__	0	0	0	0	0	0	0	0	2	0	0	0
d__Bacteria;p__Myxococcota;c__Polyangia;o__Blfdi19;f__Blfdi19	0	0	0	0	0	0	0	0	2	0	0	0
d__Bacteria;p__Firmicutes;c__uncultured;o__uncultured;f__uncultured	0	0	0	0	2	0	0	0	2	0	0	0
d__Bacteria;p__Myxococcota;c__Polyangia;o__Polyangiales;f__Phaselicystidaceae	0	0	0	2	0	0	0	0	0	0	0	0
d__Bacteria;p__Patescibacteria;c__Parcubacteria;o__Candidatus_Azambacteria;f__Candidatus_Azambacteria	0	0	0	0	0	0	0	0	0	2	0	0
d__Bacteria;p__Firmicutes;c__Bacilli;o__Thermoactinomycetales;f__Thermoactinomycetaceae	0	0	0	0	0	0	0	0	0	2	0	0
d__Bacteria;p__Actinobacteriota;c__Actinobacteria;o__Corynebacteriales;f__Corynebacteriaceae	0	0	0	0	0	0	0	0	0	2	0	0
d__Archaea;p__Thermoplasmatota;c__Thermoplasmata;o__Marine_Benthic_Group_D_and_DHVEG-1;f__Marine_Benthic_Group_D_and_DHVEG-1	0	0	0	0	2	0	0	0	0	0	0	0
d__Bacteria;p__Chloroflexi;c__Chloroflexia;o__Elev-1554;f__Elev-1554	0	0	0	0	2	0	0	0	0	0	0	0
d__Bacteria;p__Planctomycetota;c__Phycisphaerae;o__Tepidisphaerales;__	0	0	0	0	2	0	0	0	0	0	0	0
d__Bacteria;p__Firmicutes;c__Clostridia;o__Oscillospirales;f__Oscillospirales	0	0	0	0	2	0	0	0	0	0	0	0
d__Bacteria;p__Myxococcota;c__Polyangia;o__MSB-4B10;f__MSB-4B10	0	0	0	0	2	0	0	0	0	0	0	0
d__Bacteria;p__Proteobacteria;c__Alphaproteobacteria;o__Elsterales;f__Elsteraceae	0	0	0	0	2	0	0	0	0	0	0	0
d__Bacteria;p__Verrucomicrobiota;c__Omnitrophia;o__Omnitrophales;f__Omnitrophaceae	0	0	0	0	2	0	0	0	0	0	0	0
d__Bacteria;p__Proteobacteria;c__Gammaproteobacteria;o__CHAB-XI-27;f__CHAB-XI-27	0	0	0	0	0	0	0	0	0	0	2	0
d__Bacteria;p__Bacteroidota;c__SJA-28;o__SJA-28;f__SJA-28	0	0	0	0	0	2	0	0	0	0	0	0
d__Bacteria;p__Chloroflexi;c__Chloroflexia;o__Chloroflexales;f__Roseiflexaceae	0	0	0	0	0	2	0	0	0	0	0	0
d__Bacteria;p__Chloroflexi;c__Dehalococcoidia;__;__	0	0	0	0	0	2	0	0	0	0	0	0
d__Bacteria;p__Patescibacteria;c__ABY1;o__Candidatus_Magasanikbacteria;f__Candidatus_Magasanikbacteria	0	0	0	0	0	2	0	0	0	0	0	2
d__Bacteria;p__Acidobacteriota;c__Vicinamibacteria;o__Vicinamibacterales;f__Vicinamibacteraceae	0	0	0	0	0	2	0	0	0	0	0	0
d__Bacteria;p__Firmicutes;c__Symbiobacteriia;o__Symbiobacteriales;f__uncultured	0	0	0	0	0	2	0	0	0	0	0	0
d__Bacteria;p__Verrucomicrobiota;c__Verrucomicrobiae;o__Chthoniobacterales;__	0	0	0	0	0	2	0	0	0	0	0	0
d__Bacteria;p__Bacteroidota;c__Bacteroidia;o__Flavobacteriales;f__Crocinitomicaceae	0	0	0	0	0	2	0	0	0	0	0	0

**Bacteria taxonomy**	**R1_A SR5i**	**R2_A SR5i**	**R3_A SR5i**	**R4_A SR5i**	**R5_A SR5i**	**R6_A SR5i**	**R1_ WT**	**R2_ WT**	**R3_ WT**	**R4_ WT**	**R5_ WT**	**R6_ WT**
d__Bacteria;p__Bacteroidota;c__Bacteroidia;o__SM1A07;f__SM1A07	0	0	0	0	0	0	0	0	0	0	0	2
d__Bacteria;p__Acidobacteriota;c__Blastocatellia;o__11-24;f__11-24	0	0	0	0	0	0	0	0	0	0	0	2
d__Bacteria;p__Chloroflexi;c__Dehalococcoidia;o__FS117-23B-02;f__FS117-23B-02	0	0	0	0	0	0	0	0	0	0	0	2
d__Bacteria;p__Firmicutes;c__Clostridia;o__Peptostreptococcales-Tissierellales;__	0	0	0	0	0	0	0	0	0	0	0	4
d__Bacteria;p__Chloroflexi;c__Dehalococcoidia;o__vadinBA26;f__vadinBA26	0	0	0	0	0	0	0	0	0	0	0	2
