# Supplementary material for: The impact of OsASR5 on rice microbiome: Implications for nutrient cycling and stress response
Source: Genet Mol Biol. 2026 Oct 26;49(Suppl 3):e20250064. doi: 10.1590/1678-4685-GMB-2025-0064 (PMC13720868; doi:10.1590/1678-4685-GMB-2025-0064)
Supplement: Supplementary file 8 [file 1415-4757-GMB-49-s3-e20250064-s8.pdf]

Genetics and Molecular Biology

https://doi.org/10.1590/1678-4685-GMB-2025-0064

Supplementary Material to "The impact of OsASR5 on rice microbiome: Implications for nutrient cycling and stress response"

Table S8 - Bacterial genera abundance per sample from leaf microbiome.

**Bacteria**	**L1_ASR5ii**	**L2__ASR5**	**L3__ASR5i**	**L4__ASR5i**	**L5__ASR5i**	**L6__ASR5i**	**L1_WT**	**L2_WT**	**L3_WT**	**L4_WT**	**L5_WT**	**L6_WT**
d__Bacteria;p__Proteobacteria;c__Alphaproteobacteria;o__Rhizobiales;f__Rhizobiaceaeg__Phyllobacterium	315	359	404	570	62	48	0	117	0	457	299	144
d__Bacteriap__Proteobacteriac__Gammaproteobacteriao__Enterobacterales____	0	888	0	0	0	0	8	0	35	0	0	10
d__Bacteriap__Proteobacteriac__Gammaproteobacteriao__Enterobacteralesf__Enterobacteriaceaeg__Escherichia-Shigella	30	80	357	363	94	55	0	46	42	11	54	0
d__Bacteriap__Proteobacteriac__Gammaproteobacteriao__Pseudomonadalesf__Pseudomonadaceaeg__Pseudomonas	2	12	13	2	0	0	239	0	31	14	0	0
d__Bacteriap__Firmicutesc__Bacillio__Bacillalesf__Bacillaceaeg__Bacillus	290	87	107	19	9	29	11	6	13	22	0	0
d__Bacteriap__Proteobacteriac__Alphaproteobacteriao__Rhizobialesf__Rhizobiaceae__	0	3	0	2	0	0	64	4	188	0	0	0
d__Bacteriap__Firmicutesc__Bacillio__Aneurinibacillalesf__Aneurinibacillaceaeg__Aneurinibacillus	82	22	17	32	3	4	0	0	0	5	0	0
d__Bacteriap__Proteobacteriac__Gammaproteobacteriao__Burkholderialesf__Oxalobacteraceaeg__Massilia	9	20	64	36	0	0	0	0	130	3	4	0

**Bacteria**	**L1_ASR5ii**	**L2__ASR5**	**L3__ASR5i**	**L4__ASR5i**	**L5__ASR5i**	**L6__ASR5i**	**L1_WT**	**L2_WT**	**L3_WT**	**L4_WT**	**L5_WT**	**L6_WT**
d__Bacteriap__Myxococcotac__Polyangiao__Polyangialesf__Polyangiaceaeg__uncultured	0	0	0	0	0	0	0	74	0	0	0	0
d__Bacteriap__Myxococcotac__Myxococciao__Myxococcalesf__Myxococcaceaeg__Cystobacter	0	0	0	0	0	0	61	0	0	0	0	0
d__Bacteriap__Proteobacteriac__Gammaproteobacteriao__Salinisphaeralesf__Solimonadaceaeg__Nevskia	45	0	0	12	0	6	0	0	0	20	0	0
d__Bacteriap__Proteobacteriac__Alphaproteobacteriao__Rhizobialesf__Xanthobacteraceaeg__Bradyrhizobium	0	0	44	0	2	4	0	0	0	0	0	0
d__Bacteriap__Firmicutesc__Bacillio__Staphylococcalesf__Staphylococcaceaeg__Staphylococcus	16	21	11	0	3	6	18	0	3	43	6	4
d__Bacteriap__Proteobacteriac__Gammaproteobacteriao__Burkholderiales____	53	0	30	44	15	24	0	0	0	0	0	0
d__Bacteriap__Proteobacteriac__Alphaproteobacteriao__Sphingomonadalesf__Sphingomonadaceaeg__Sphingomonas	9	18	41	39	9	4	30	8	16	0	31	9
d__Bacteriap__Proteobacteriac__Alphaproteobacteriao__Rhizobialesf__Xanthobacteraceae__	26	37	0	0	0	0	8	0	0	17	9	4
d__Bacteriap__Firmicutesc__Bacillio__Alicyclobacillalesf__Alicyclobacillaceaeg__Tumebacillus	0	0	0	0	0	0	0	4	0	81	0	0
d__Bacteriap__Proteobacteriac__Gammaproteobacteriao__Pseudomonadalesf__Moraxellaceaeg__Alkanindiges	0	25	49	13	0	0	0	0	0	0	0	0
d__Bacteriap__Proteobacteriac__Gammaproteobacteriao__Pseudomonadalesf__Moraxellaceaeg__Acinetobacter	15	11	4	17	0	0	0	0	0	29	5	0
d__Bacteriap__Proteobacteriac__Alphaproteobacteriao__Rhizobialesf__Beijerinckiaceaeg__Methylobacterium-Methylorubrum	0	10	21	0	0	0	7	5	10	28	5	0
d__Bacteriap__Proteobacteriac__Gammaproteobacteriao__Burkholderialesf__Rhodocyclaceae__	38	2	0	35	0	21	0	0	0	0	0	0

Bacteria	L1_ASR5ii	L2__ASR5	L3__ASR5i	L4__ASR5i	L5__ASR5i	L6__ASR5i	L1_WT	L2_WT	L3_WT	L4_WT	L5_WT	L6_WT
d__Bacteriap__Proteobacteriac__Gammaproteobacteriao__Enterobacteralesf__Enterobacteriaceae__	0	0	21	0	0	0	16	0	27	0	7	0
d__Bacteriap__Proteobacteriac__Gammaproteobacteriao__Burkholderialesf__Burkholderiaceaeg__Burkholderia-Caballeronia-Paraburkholderia	3	0	15	6	5	0	5	11	12	26	0	0
d__Bacteriap__Firmicutesc__Clostridiao__Clostridialesf__Clostridiaceae__	2	13	24	0	0	2	0	0	2	0	0	0
d__Bacteriap__Proteobacteriac__Alphaproteobacteriao__Sphingomonadalesf__Sphingomonadaceae__	4	0	0	8	0	0	0	4	0	24	14	0
d__Bacteriap__Proteobacteriac__Gammaproteobacteriao__Enterobacteralesf__Erwiniaceae__	0	0	0	0	0	0	0	0	23	0	0	0
d__Bacteriap__Proteobacteriac__Gammaproteobacteriao__Xanthomonadalesf__Rhodanobacteraceaeg__Rhodanobacter	22	0	8	0	0	0	0	0	0	0	0	0
d__Bacteriap__Desulfobacterotac__Desulfuromonadiao__Geobacteralesf__Geobacteraceaeg__Geobacter	22	9	10	11	0	0	0	0	0	0	0	0
d__Bacteriap__Proteobacteriac__Alphaproteobacteriao__Rhodobacteralesf__Rhodobacteraceaeg__Paracoccus	0	10	22	0	0	0	10	3	0	0	0	0
d__Bacteriap__Proteobacteriac__Gammaproteobacteriao__Burkholderialesf__Comamonadaceaeg__Aquabacterium	7	0	0	0	0	0	0	0	21	0	0	0
d__Bacteriap__Proteobacteriac__Gammaproteobacteria______	0	5	0	20	0	0	0	0	0	0	0	0
d__Bacteriap__Proteobacteriac__Gammaproteobacteriao__Xanthomonadalesf__Xanthomonadaceaeg__Stenotrophomonas	0	4	0	0	0	0	19	0	0	0	0	0
d__Bacteria__________	4	21	28	0	4	0	29	12	9	21	4	5
d__Bacteriap__Firmicutesc__Clostridiao__Clostridiaf__Gracilibacteraceae__	0	19	10	0	0	0	0	0	0	0	0	0
d__Bacteriap__Cyanobacteriac__Cyanobacteriiao__Cyanobacterialesf__Phormidiaceaeg__Tychonema_CCAP_1459-11B	0	19	0	0	0	0	0	0	0	0	0	0

**Bacteria**	**L1_ASR5ii**	**L2__ASR5**	**L3__ASR5i**	**L4__ASR5i**	**L5__ASR5i**	**L6__ASR5i**	**L1_WT**	**L2_WT**	**L3_WT**	**L4_WT**	**L5_WT**	**L6_WT**
d__Bacteriap__Proteobacteriac__Alphaproteobacteriao__Caulobacteralesf__Caulobacteraceaeg__Brevundimonas	0	0	19	0	0	0	13	0	0	0	0	0
d__Bacteriap__Firmicutesc__Clostridiao__Clostridialesf__Clostridiaceaeg__Clostridium_sensu_stricto_1	0	15	19	6	5	0	3	9	0	28	4	4
d__Bacteriap__Bacteroidotac__Bacteroidiao__Chitinophagalesf__Chitinophagaceaeg__Cnuella	0	3	19	0	0	0	0	0	0	0	0	0
d__Bacteriap__Acidobacteriotac__Acidobacteriaeo__Solibacteralesf__Solibacteraceaeg__Candidatus_Solibacter	0	3	0	19	0	0	0	0	0	0	0	0
d__Bacteriap__Proteobacteriac__Alphaproteobacteriao__Sphingomonadalesf__Sphingomonadaceaeg__Novosphingobium	0	11	0	0	3	0	0	0	0	19	0	0
d__Bacteriap__Proteobacteriac__Gammaproteobacteriao__Burkholderialesf__Comamonadaceae__	0	0	27	18	0	0	7	0	0	0	11	0
d__Bacteriap__Proteobacteriac__Alphaproteobacteriao__Caulobacteralesf__Caulobacteraceaeg__uncultured	0	0	16	0	0	0	0	0	0	0	3	0
d__Bacteriap__Firmicutesc__Clostridiao__Clostridialesf__Clostridiaceaeg__Clostridium_sensu_stricto_12	3	0	0	16	4	0	0	0	0	0	0	0
d__Bacteriap__Proteobacteriac__Alphaproteobacteriao__Elsteralesf__unculturedg__uncultured	0	15	0	15	0	0	0	0	0	0	0	6
d__Bacteriap__Firmicutesc__Clostridiao__Peptostreptococcales-Tissierellalesf__Peptostreptococcales-Tissierellalesg__Anaerococcus	0	0	15	0	0	0	3	0	0	0	0	0
d__Bacteriap__Firmicutesc__Bacillio__Alicyclobacillalesf__Alicyclobacillaceaeg__Alicyclobacillus	0	0	0	15	0	0	0	0	0	0	0	0
d__Bacteriap__Proteobacteriac__Alphaproteobacteriao__Rhizobialesf__Beijerinckiaceaeg__1174-901-12	0	0	0	0	0	0	0	0	0	15	0	0

Bacteria	L1_ASR5ii	L2__ASR5	L3__ASR5i	L4__ASR5i	L5__ASR5i	L6__ASR5i	L1_WT	L2_WT	L3_WT	L4_WT	L5_WT	L6_WT
d__Bacteriap__Acidobacteriotac__Holophagaeo__Holophagalesf__Holophagaceae__	14	0	5	0	0	2	0	0	0	0	0	0
d__Bacteriap__Gemmatimonadotac__Gemmatimonadeteso__Gemmatimonadalesf__Gemmatimonadaceaeg__Gemmatimonas	14	0	6	0	0	0	0	0	0	0	0	0
d__Bacteriap__Acidobacteriotac__Acidobacteriaeo__Acidobacterialesf__Acidobacteriaceae_(Subgroup_1)__	13	2	14	15	0	8	0	0	0	0	0	0
d__Bacteriap__Firmicutesc__Clostridiao__Clostridiales____	3	0	14	0	0	0	0	0	0	0	0	0
d__Bacteriap__Proteobacteriac__Gammaproteobacteriao__Xanthomonadalesf__Rhodanobacteraceae__	3	0	0	14	0	0	0	0	0	0	0	0
d__Bacteriap__Proteobacteriac__Gammaproteobacteriao__Burkholderialesf__Alcaligenaceaeg__Achromobacter	0	0	0	0	0	0	0	0	0	14	0	0
d__Bacteriap__Actinobacteriotac__Thermoleophiliao__Gaiellalesf__unculturedg__uncultured	3	0	13	0	0	0	0	0	0	0	0	0
d__Bacteriap__Actinobacteriotac__Actinobacteriao__Micrococcalesf__Microbacteriaceaeg__Curtobacterium	0	0	13	0	0	0	0	0	0	5	0	0
d__Bacteriap__Bacteroidotac__Bacteroidiao__Chitinophagalesf__Chitinophagaceaeg__Puia	0	0	0	12	0	0	0	0	13	4	0	0
d__Bacteriap__Bacteroidotac__Bacteroidiao__Cytophagalesf__Amoebophilaceaeg__Candidatus_Amoebophilus	2	0	0	13	0	0	0	0	0	0	0	0
d__Bacteriap__Proteobacteriac__Alphaproteobacteriao__Acetobacteralesf__Acetobacteraceaeg__Craurococcus-Caldovatus	0	0	0	0	0	0	0	0	0	13	0	0
d__Bacteriap__Firmicutesc__Bacillio__Bacillalesf__Planococcaceaeg__Lysinibacillus	8	0	0	0	0	0	0	0	0	13	0	0
d__Bacteriap__Actinobacteriotac__Actinobacteriao__Streptomycetalesf__Streptomycetaceaeg__Streptomyces	16	0	11	0	0	0	0	0	0	0	0	0

**Bacteria**	**L1_ASR5ii**	**L2__ASR5**	**L3__ASR5i**	**L4__ASR5i**	**L5__ASR5i**	**L6__ASR5i**	**L1_WT**	**L2_WT**	**L3_WT**	**L4_WT**	**L5_WT**	**L6_WT**
d__Bacteriap__Proteobacteriac__Gammaproteobacteriao__Burkholderialesf__Neisseriaceaeg__Neisseria	12	0	0	0	0	0	0	0	0	0	0	0
d__Bacteriap__Firmicutes________	12	0	0	0	0	0	0	0	0	0	0	0
d__Bacteriap__Actinobacteriotac__Actinobacteriao__Frankialesf__Geodermatophilaceae__	0	12	0	0	0	0	0	0	0	0	0	0
d__Bacteriap__Acidobacteriotac__Acidobacteriaeo__Acidobacterialesf__unculturedg__uncultured	0	24	0	0	0	5	0	0	0	0	0	0
d__Bacteriap__Bacteroidotac__Bacteroidiao__Bacteroidales____	16	0	8	0	0	4	0	0	0	0	0	0
d__Bacteriap__Bacteroidotac__Bacteroidiao__Sphingobacterialesf__Sphingobacteriaceaeg__Mucilaginibacter	0	5	0	0	0	0	0	0	12	0	0	0
d__Bacteriap__Bdellovibrionotac__Bdellovibrioniao__Bdellovibrionalesf__Bdellovibrionaceaeg__OM27_clade	0	0	0	12	0	0	2	0	0	0	0	0
d__Bacteriap__Myxococcotac__Myxococciao__Myxococcalesf__Anaeromyxobacteraceaeg__Anaeromyxobacter	0	0	0	0	0	0	0	0	0	12	0	0
d__Bacteriap__Firmicutesc__Clostridiao__Peptostreptococcales-Tissierellalesf__Peptostreptococcales-Tissierellalesg__Tissierella	11	6	0	0	0	0	0	0	0	0	0	0
d__Bacteriap__Proteobacteriac__Gammaproteobacteriao__Burkholderialesf__SC-I-84g__SC-I-84	11	0	10	0	0	5	0	0	0	0	0	0
d__Bacteriap__Firmicutesc__Clostridiao__Clostridialesf__Clostridiaceaeg__Clostridium_sensu_stricto_5	11	0	8	0	0	0	0	0	0	0	0	0
d__Bacteriap__Firmicutesc__Bacillio__Bacillalesf__Planococcaceae__	0	11	0	0	0	0	0	0	0	0	0	0
d__Bacteriap__Proteobacteriac__Gammaproteobacteriao__Burkholderialesf__Comamonadaceaeg__Azohydromonas	0	11	0	0	0	0	0	0	0	0	0	0
d__Bacteriap__Actinobacteriotac__Actinobacteriao__Corynebacterialesf__Corynebacteriaceaeg__Turicella	0	0	0	0	0	0	0	11	0	0	0	0

**Bacteria**	**L1_ASR5ii**	**L2__ASR5**	**L3__ASR5i**	**L4__ASR5i**	**L5__ASR5i**	**L6__ASR5i**	**L1_WT**	**L2_WT**	**L3_WT**	**L4_WT**	**L5_WT**	**L6_WT**
d__Bacteriap__Firmicutesc__Clostridiao__Peptostreptococcales-Tissierellalesf__Peptostreptococcaceaeg__Romboutsia	8	0	11	0	0	0	3	0	0	0	0	0
d__Bacteriap__Deinococcotac__Deinococcio__Deinococcalesf__Trueperaceaeg__Truepera	0	0	11	8	0	0	0	0	0	0	0	0
d__Bacteriap__Acidobacteriotac__Holophagaeo__Holophagalesf__Holophagaceaeg__Geothrix	0	0	11	0	0	2	0	0	0	0	0	0
d__Bacteriap__Proteobacteriac__Alphaproteobacteriao__Rhizobialesf__Beijerinckiaceaeg__uncultured	0	0	11	0	0	0	0	0	0	0	0	0
d__Bacteriap__Proteobacteriac__Gammaproteobacteriao__Burkholderialesf__Rhodocyclaceaeg__Methyloversatilis	0	5	11	0	0	0	0	0	0	0	0	0
d__Bacteriap__Desulfobacterotac__Desulfuromonadiao__Geobacterales____	0	0	11	4	0	0	0	0	0	0	0	0
Unassigned__________	3	6	9	0	0	0	10	6	11	0	0	0
d__Bacteriap__Bacteroidotac__Bacteroidiao__Chitinophagalesf__Chitinophagaceaeg__Sediminibacterium	2	0	0	0	4	0	0	0	11	4	0	0
d__Bacteriap__Actinobacteriotac__Actinobacteriao__Frankialesf__Frankiaceaeg__Jatrophihabitans	0	0	0	11	0	0	0	0	0	0	0	0
d__Bacteriap__Myxococcotac__Polyangiao__Polyangiales____	0	0	0	0	7	0	0	0	9	0	7	11
d__Bacteriap__Firmicutesc__Bacillio__Bacillales____	10	0	0	0	0	0	0	0	0	0	0	0
d__Bacteriap__Proteobacteriac__Alphaproteobacteriao__Rhizobialesf__Beijerinckiaceaeg__Microvirga	0	0	10	0	0	0	0	0	0	7	0	0
d__Bacteriap__Actinobacteriotac__Actinobacteriao__Frankiales____	0	0	10	0	0	0	0	0	0	0	0	0
d__Bacteriap__Deinococcotac__Deinococcio__Deinococcalesf__Deinococcaceaeg__Deinococcus	0	0	0	0	0	0	2	0	10	10	0	0
d__Bacteriap__Actinobacteriotac__Actinobacteriao__Catenulisporalesf__Actinospicaceaeg__Actinospica	0	0	0	10	0	0	0	0	0	0	0	0

Bacteria	L1_ASR5ii	L2__ASR5	L3__ASR5i	L4__ASR5i	L5__ASR5i	L6__ASR5i	L1_WT	L2_WT	L3_WT	L4_WT	L5_WT	L6_WT
d__Bacteriap__Proteobacteriac__Alphaproteobacteriao__Rhizobialesf__Beijerinckiaceaeg__Roseiarcus	9	0	0	10	4	0	0	0	0	0	0	0
d__Bacteriap__Actinobacteriotac__Actinobacteriao__Frankialesf__Acidothermaceaeg__Acidothermus	0	3	4	10	3	0	0	0	0	0	0	0
d__Bacteriap__Actinobacteriotac__Actinobacteriao__Micrococcalesf__Dermacoccaceae__	0	0	0	0	0	0	0	0	0	10	0	0
d__Bacteriap__Acidobacteriotac__Acidobacteriaeo__Bryobacteralesf__Bryobacteraceaeg__Bryobacter	9	0	0	0	6	0	0	0	0	0	0	0
d__Bacteriap__Firmicutesc__Clostridiao__Clostridialesf__Clostridiaceaeg__Clostridium_sensu_stricto_13	9	0	0	0	0	0	0	0	0	0	0	0
d__Bacteriap__Proteobacteriac__Alphaproteobacteriao__Rhodobacteralesf__Rhodobacteraceaeg__Wenxinia	0	9	0	0	0	0	0	0	0	0	0	0
d__Bacteriap__Proteobacteriac__Gammaproteobacteriao__Burkholderialesf__Oxalobacteraceae__	0	9	9	0	0	0	0	0	0	0	0	0
d__Bacteriap__Firmicutesc__Clostridiao__Oscillospiralesf__Oscillospiraceaeg__Sporobacter	0	0	9	0	0	0	0	0	0	0	0	0
d__Bacteriap__Proteobacteriac__Gammaproteobacteriao__Gammaproteobacteria_Incertae_Sedisf__Unknown_Familyg__Acidibacter	0	4	9	0	2	0	0	0	0	0	0	0
d__Bacteriap__Firmicutesc__Clostridiao__Clostridialesf__Oxobacteraceaeg__Oxobacter	3	0	9	4	4	0	0	0	0	0	0	0
d__Bacteriap__Proteobacteriac__Gammaproteobacteriao__Xanthomonadalesf__Rhodanobacteraceaeg__Rudaea	0	0	9	0	0	0	0	0	0	0	0	0
d__Bacteriap__Chloroflexic__AD3o__AD3f__AD3g__AD3	0	0	0	9	0	0	0	0	0	0	0	0
d__Bacteriap__Actinobacteriotac__Actinobacteriao__Corynebacterialesf__Nocardiaceaeg__Smaragdicoccus	0	0	0	9	0	0	0	0	0	0	0	0

Bacteria	L1_ASR5ii	L2__ASR5	L3__ASR5i	L4__ASR5i	L5__ASR5i	L6__ASR5i	L1_WT	L2_WT	L3_WT	L4_WT	L5_WT	L6_WT
d__Bacteriap__Actinobacteriotac__Actinobacteriao__Micrococcalesf__Micrococcaceaeg__Tersicoccus	0	0	0	0	0	0	0	0	0	9	0	0
d__Bacteriap__Cyanobacteriac__Cyanobacteriiao__Cyanobacterialesf__Nostocaceaeg__Scytonema_VB-61278	0	0	5	0	0	0	0	0	0	0	9	0
d__Bacteriap__Firmicutesc__Clostridiao__Clostridialesf__Clostridiaceaeg__Clostridium_sensu_stricto_8	8	0	0	0	0	0	0	0	0	0	0	0
d__Bacteriap__FCPU426c__FCPU426o__FCPU426f__FCPU426g__FCPU426	8	0	0	0	0	0	0	0	0	0	0	0
d__Bacteriap__Acidobacteriotac__Acidobacteriaeo__Acidobacterialesf__Koribacteraceaeg__Candidatus_Koribacter	8	0	0	0	0	7	0	0	0	0	0	0
d__Bacteriap__Firmicutesc__Clostridiao__Clostridialesf__Clostridiaceaeg__Clostridium_sensu_stricto_11	8	0	0	0	0	0	0	0	0	0	0	0
d__Bacteriap__Actinobacteriotac__Actinobacteriao__Corynebacterialesf__Corynebacteriaceaeg__Corynebacterium	12	5	0	0	6	2	4	0	3	0	0	0
d__Bacteriap__Proteobacteriac__Alphaproteobacteriao__Rickettsialesf__Rickettsiaceae__	0	0	0	0	0	0	8	7	0	0	0	0
d__Bacteriap__Actinobacteriotac__Actinobacteriao__Micrococcalesf__Cellulomonadaceaeg__Cellulomonas	0	0	0	0	0	0	8	0	0	0	8	0
d__Bacteriap__Actinobacteriotac__Actinobacteriao__Pseudonocardialesf__Pseudonocardiaceaeg__Actinomycetospora	3	0	2	0	0	0	8	0	0	0	5	0
d__Bacteriap__Chloroflexic__Ktedonobacteriao__Ktedonobacteralesf__JG30-KF-AS9g__JG30-KF-AS9	0	8	0	0	0	0	0	0	0	0	0	0
d__Bacteriap__Firmicutesc__Bacillio__Lactobacillalesf__Streptococcaceaeg__Streptococcus	0	8	7	0	5	0	0	0	0	0	0	0
d__Bacteriap__Actinobacteriotac__Acidimicrobiiao__IMCC26256f__IMCC26256g__IMCC26256	0	11	0	5	0	0	0	0	0	0	0	0

Bacteria	L1_A SR5ii	L2__ ASR5	L3__ ASR5i	L4__ ASR5i	L5__ ASR5i	L6__ ASR5i	L1_ WT	L2_ WT	L3_ WT	L4_ WT	L5_ WT	L6_ WT
d__Bacteriap__Myxococcotac__Myxococciao__Myxococcalesf__Myxococcaceae__	0	8	0	0	0	0	0	0	0	0	0	0
d__Bacteriap__Proteobacteriac__Alphaproteobacteriao__Micropepsalesf__Micropepsaceaeg__Rhizomicrobium	0	8	0	0	0	0	0	0	0	0	0	0
d__Bacteriap__Proteobacteriac__Alphaproteobacteriao__Paracaedibacteralesf__Paracaedibacteraceaeg__Candidatus_Paracaedibacter	0	0	0	0	0	0	0	8	0	0	0	0
d__Bacteriap__Acidobacteriotac__Thermoanaerobaculiao__Thermoanaerobaculalesf__Thermoanaerobaculaceaeg__Subgroup_10	0	0	8	0	0	0	0	0	0	0	0	0
d__Bacteriap__Myxococcotac__Polyangiao__Polyangialesf__Polyangiaceae__	0	0	0	8	0	0	0	0	0	0	0	0
d__Bacteriap__Cyanobacteriac__Cyanobacteriiao__Cyanobacterialesf__Nostocaceaeg__Mastigocladopsis_PCC-10914	0	0	0	0	0	0	0	0	0	8	0	0
d__Bacteriap__Proteobacteriac__Gammaproteobacteriao__Diplorickettsialesf__Diplorickettsiaceaeg__uncultured	0	0	0	0	0	0	0	0	0	8	0	0
d__Bacteriap__Proteobacteriac__Gammaproteobacteriao__Xanthomonadalesf__Xanthomonadaceaeg__Pseudoxanthomonas	0	0	0	0	0	0	0	0	0	0	8	0
d__Bacteriap__Bacteroidotac__Bacteroidiao__Chitinophagalesf__Chitinophagaceaeg__Flavisolibacter	0	0	0	0	0	0	0	0	0	0	8	0
d__Bacteriap__Firmicutesc__Desulfitobacteriiao__Desulfitobacterialesf__Desulfitobacteriaceaeg__Desulfosporosinus	7	0	0	0	0	2	0	0	0	0	0	0
d__Bacteriap__Firmicutesc__Clostridiao__Lachnospiralesf__Lachnospiraceae__	7	7	0	0	0	0	0	0	0	0	0	0
d__Bacteriap__Firmicutesc__Bacillio__Paenibacillalesf__Paenibacillaceaeg__Paenibacillus	19	11	2	0	0	0	5	0	0	5	0	0
d__Bacteriap__Firmicutesc__Clostridiao__Clostridialesf__Caloramatoraceaeg__Fonticella	7	0	0	0	0	0	0	0	0	0	0	0
d__Bacteriap__Firmicutesc__Clostridiao__Clostridialesf__Clostridiaceaeg__Clostridium_sensu_stricto_10	7	0	0	0	0	0	0	0	0	0	0	0

Bacteria	L1_ASR5ii	L2__ASR5	L3__ASR5i	L4__ASR5i	L5__ASR5i	L6__ASR5i	L1_WT	L2_WT	L3_WT	L4_WT	L5_WT	L6_WT
d__Bacteriap__Actinobacteriotac__Actinobacteriao__Micrococcalesf__Dermatophilaceaeg__uncultured	0	0	0	0	0	0	7	0	0	0	0	0
d__Bacteriap__Firmicutesc__Bacillio__Thermoactinomycetalesf__Thermoactinomycetaceaeg__Thermoflavimicrobium	0	7	0	0	0	0	0	0	0	0	0	0
d__Bacteriap__Myxococcotac__Polyangiao__Polyangialesf__Phaselicystidaceaeg__Phaselicystis	0	0	7	0	0	0	0	3	0	0	0	0
d__Bacteriap__Proteobacteriac__Alphaproteobacteriao__Acetobacteralesf__Acetobacteraceaeg__Roseomonas	0	0	7	0	0	0	0	0	4	0	0	0
d__Bacteriap__Chloroflexic__TK10o__TK10f__TK10g__TK10	0	0	0	5	0	0	0	0	7	0	0	0
d__Bacteriap__Bacteroidotac__Kryptoniao__Kryptonialesf__Kryptoniaceaeg__Kryptoniaceae	0	0	0	7	0	0	0	0	0	0	0	0
d__Bacteriap__Patescibacteriac__Parcubacteriao__Candidatus_Zambryskibacteriaf__Candidatus_Zambryskibacteriag__Candidatus_Zambryskibacteria	0	0	0	7	0	0	0	0	0	0	0	0
d__Eukaryota__________	0	0	0	7	0	0	0	0	0	0	0	0
d__Bacteriap__Proteobacteriac__Alphaproteobacteriao__Acetobacteralesf__Acetobacteraceae__	9	0	0	7	0	0	6	8	0	0	0	0
d__Bacteriap__Proteobacteriac__Alphaproteobacteriao__Elsterales____	6	0	0	7	0	0	0	0	0	0	0	0
d__Bacteriap__Acidobacteriotac__Acidobacteriaeo__Acidobacterialesf__Acidobacteriaceae_(Subgroup_1)g__Terriglobus	0	0	0	0	0	0	0	0	0	7	0	0
d__Bacteriap__Proteobacteriac__Gammaproteobacteriao__Methylococcalesf__Methylomonadaceaeg__Methylomonas	4	0	0	0	7	0	0	0	0	0	0	0
d__Bacteriap__Bacteroidotac__Bacteroidiao__Chitinophagalesf__Chitinophagaceae__	0	0	0	0	0	2	0	0	0	0	7	0
d__Bacteriap__Bacteroidotac__Bacteroidiao__Cytophagalesf__Hymenobacteraceaeg__Hymenobacter	0	0	4	3	0	0	4	3	0	0	7	0

Bacteria	L1_ASR5ii	L2__ASR5	L3__ASR5i	L4__ASR5i	L5__ASR5i	L6__ASR5i	L1_WT	L2_WT	L3_WT	L4_WT	L5_WT	L6_WT
d__Bacteriap__Chloroflexic__Anaerolineaeo__SJA-15f__SJA-15g__SJA-15	6	0	0	0	0	0	0	0	0	0	0	0
d__Bacteriap__Firmicutesc__Desulfotomaculiao__Desulfotomaculalesf__Desulfotomaculalesg__Desulfofarcimen	6	0	0	0	0	0	0	0	0	0	0	0
d__Bacteriap__Firmicutesc__Clostridia______	0	6	0	0	0	0	0	0	0	0	0	0
d__Bacteriap__Proteobacteriac__Alphaproteobacteriao__Rhizobialesf__Xanthobacteraceaeg__Pseudolabrys	0	6	0	0	0	0	0	0	0	0	0	0
d__Bacteriap__Firmicutesc__Clostridiao__Clostridiaf__Hungateiclostridiaceae__	8	6	0	0	3	2	0	0	0	0	0	0
d__Bacteriap__Actinobacteriotac__Actinobacteriao__Streptomycetalesf__Streptomycetaceae__	0	6	0	0	0	0	0	0	0	0	0	0
d__Bacteriap__Actinobacteriotac__Actinobacteriao__Frankialesf__Geodermatophilaceaeg__Modestobacter	0	6	0	0	0	0	0	0	0	0	0	0
d__Bacteriap__Verrucomicrobiotac__Verrucomicrobiaeo__Pedosphaeralesf__Pedosphaeraceaeg__Ellin516	0	0	6	0	0	3	0	0	0	0	0	0
d__Bacteriap__Proteobacteriac__Gammaproteobacteriao__Methylococcalesf__Methylomonadaceaeg__Methylobacter	0	0	6	0	0	0	0	0	0	0	0	0
d__Bacteriap__Proteobacteriac__Gammaproteobacteriao__Burkholderialesf__Burkholderiaceae__	0	0	6	0	0	0	0	3	0	0	0	0
d__Bacteriap__Proteobacteriac__Alphaproteobacteriao__Caulobacteralesf__Caulobacteraceae__	0	0	0	6	0	0	0	0	0	0	0	0
d__Bacteriap__Actinobacteriotac__Actinobacteriao__Pseudonocardialesf__Pseudonocardiaceaeg__Pseudonocardia	0	0	0	0	0	0	0	0	0	0	6	0
d__Bacteriap__Firmicutesc__Bacillio__Entomoplasmatalesf__Entomoplasmatalesg__Candidatus_Spiroplasma	0	0	0	0	0	0	0	0	0	0	2	6
d__Bacteriap__Bacteroidotac__Bacteroidiao__Bacteroidalesf__Bacteroidetes_vadinHA17g__Bacteroidetes_vadinHA17	5	0	0	0	0	0	0	0	0	0	0	0

Bacteria	L1_ASR5ii	L2__ASR5	L3__ASR5i	L4__ASR5i	L5__ASR5i	L6__ASR5i	L1_WT	L2_WT	L3_WT	L4_WT	L5_WT	L6_WT
d__Bacteriap__Firmicutesc__Desulfitobacteriiao__Desulfitobacterialesf__Desulfitobacteriaceaeg__Desulfitobacterium	5	0	5	0	2	0	0	0	0	0	0	0
d__Bacteriap__Proteobacteriac__Alphaproteobacteria______	10	0	0	0	0	0	4	0	0	0	0	0
d__Bacteriap__Actinobacteriotac__Actinobacteriao__Corynebacterialesf__Dietziaceaeg__Dietzia	0	2	0	0	0	0	5	0	0	0	0	0
d__Bacteriap__Bacteroidotac__Bacteroidiao__Cytophagalesf__Microscillaceaeg__uncultured	0	5	0	0	0	0	0	0	0	0	0	0
d__Bacteriap__Cyanobacteriac__Sericytochromatiao__Sericytochromatiaf__Sericytochromatiag__Sericytochromatia	0	5	0	0	0	0	0	0	0	0	0	0
d__Bacteriap__Proteobacteriac__Alphaproteobacteriao__Rhodobacteralesf__Rhodobacteraceaeg__Rubellimicrobium	0	5	0	0	0	0	0	0	0	4	0	0
d__Bacteriap__Proteobacteriac__Gammaproteobacteriao__Xanthomonadalesf__Rhodanobacteraceaeg__Chujaibacter	0	5	0	0	0	0	0	0	0	0	0	0
d__Bacteriap__Acidobacteriotac__Acidobacteriaeo__Acidobacteriales____	6	0	5	0	0	0	0	0	0	0	0	0
d__Bacteriap__Actinobacteriotac__Actinobacteriao__Pseudonocardialesf__Pseudonocardiaceaeg__Saccharopolyspora	0	0	0	0	0	0	0	0	5	0	0	0
d__Bacteriap__Firmicutesc__Clostridiao__Oscillospiralesf__Ruminococcaceaeg__Faecalibacterium	0	0	0	5	0	0	0	0	0	0	0	0
d__Bacteriap__Chloroflexic__Ktedonobacteriao__Ktedonobacteralesf__Ktedonobacteraceaeg__uncultured	0	0	0	5	0	0	0	0	0	0	0	0
d__Bacteriap__Bdellovibrionotac__Bdellovibrioniao__Bdellovibrionalesf__Bdellovibrionaceaeg__Bdellovibrio	0	0	0	5	0	0	0	0	0	0	0	0
d__Bacteriap__Actinobacteriotac__Actinobacteriao__Corynebacterialesf__Nocardiaceaeg__Nocardia	0	0	0	5	0	0	0	0	0	0	0	0

**Bacteria**	**L1_ASR5ii**	**L2__ASR5**	**L3__ASR5i**	**L4__ASR5i**	**L5__ASR5i**	**L6__ASR5i**	**L1_WT**	**L2_WT**	**L3_WT**	**L4_WT**	**L5_WT**	**L6_WT**
d__Bacteriap__Bacteroidotac__Bacteroidiao__Bacteroidalesf__Porphyromonadaceaeg__Porphyromonas	0	0	0	0	0	0	0	0	0	5	0	0
d__Bacteriap__Firmicutesc__Clostridiao__Peptostreptococcales-Tissierellalesf__Peptostreptococcaceae__	0	0	0	0	0	0	0	0	0	5	0	0
d__Bacteriap__Desulfobacterotac__Desulfuromonadiao__Geobacteralesf__Geobacteraceaeg__Geotalea	0	0	0	0	5	0	0	0	0	0	0	0
d__Bacteriap__Firmicutesc__Bacillio__Lactobacillalesf__Lactobacillaceaeg__Lactobacillus	0	0	0	0	0	0	0	0	0	0	0	5
d__Bacteriap__Desulfobacterotac__Desulfuromonadiao__Geobacteralesf__Geobacteraceae__	4	0	0	0	0	0	0	0	0	0	0	0
d__Bacteriap__Bacteroidotac__Bacteroidiao__Chitinophagalesf__Chitinophagaceaeg__uncultured	4	0	0	0	0	0	0	0	0	0	0	0
d__Bacteriap__Patescibacteriac__Parcubacteria______	4	0	0	0	0	0	0	0	0	0	0	0
d__Bacteriap__Actinobacteriotac__Actinobacteriao__Actinomycetalesf__Actinomycetaceaeg__Actinomyces	4	0	0	0	0	0	0	0	0	0	0	0
d__Bacteriap__Firmicutesc__Bacilli______	4	0	0	0	0	0	0	4	0	0	0	0
d__Bacteriap__Desulfobacterotac__Syntrophobacteriao__Syntrophobacteralesf__Syntrophobacteraceae__	4	0	0	0	0	0	0	0	0	0	0	0
d__Bacteriap__Proteobacteriac__Gammaproteobacteriao__Burkholderialesf__Comamonadaceaeg__Tepidimonas	4	0	0	0	0	0	0	0	0	0	0	0
d__Bacteriap__Firmicutesc__Clostridiao__Clostridiaf__Gracilibacteraceaeg__Lutispora	4	0	0	0	0	0	0	0	0	0	0	0
d__Bacteriap__Bacteroidotac__Bacteroidiao__Cytophagalesf__Hymenobacteraceaeg__Adhaeribacter	0	0	0	0	0	0	4	0	0	0	0	0

**Bacteria**	**L1_ASR5ii**	**L2__ASR5**	**L3__ASR5i**	**L4__ASR5i**	**L5__ASR5i**	**L6__ASR5i**	**L1_WT**	**L2_WT**	**L3_WT**	**L4_WT**	**L5_WT**	**L6_WT**
d__Bacteriap__Actinobacteriotac__Actinobacteriao__Micrococcalesf__Microbacteriaceae__	0	0	0	0	0	0	4	0	0	0	0	0
d__Bacteriap__Bacteroidotac__Bacteroidiao__Sphingobacterialesf__Sphingobacteriaceaeg__Sphingobacterium	0	4	0	0	0	0	0	0	0	0	0	0
d__Bacteriap__Verrucomicrobiotac__Verrucomicrobiaeo__Pedosphaeralesf__Pedosphaeraceaeg__ADurb.Bin063-1	0	4	0	0	0	0	0	0	0	0	0	0
d__Bacteriap__Firmicutesc__Clostridiao__Peptostreptococcales-Tissierellalesf__Peptostreptococcaceaeg__Sporacetigenium	0	4	0	0	0	0	0	0	0	0	0	0
d__Bacteriap__Proteobacteriac__Gammaproteobacteriao__KF-JG30-C25f__KF-JG30-C25g__KF-JG30-C25	0	4	0	0	0	0	0	0	0	0	0	0
d__Bacteriap__Proteobacteriac__Alphaproteobacteriao__Acetobacteralesf__Acetobacteraceaeg__uncultured	0	4	0	0	0	0	0	0	0	0	0	0
d__Bacteriap__Desulfobacterotac__Desulfovibrioniao__Desulfovibrionalesf__Desulfovibrionaceaeg__Desulfovibrio	3	0	4	0	0	0	0	0	0	0	0	0
d__Bacteriap__Desulfobacterotac__Syntrophiao__Syntrophalesf__Syntrophaceaeg__Syntrophus	0	0	4	0	0	0	0	0	0	0	0	0
d__Bacteriap__Proteobacteriac__Alphaproteobacteriao__Rhizobialesf__Rhizobiales_Incertae_Sedisg__uncultured	0	0	4	0	0	0	0	0	0	0	0	0
d__Bacteriap__Cyanobacteriac__Cyanobacteriiao__Cyanobacterialesf__Chroococcidiopsaceaeg__Chroococcidiopsis_PCC_7203	0	2	4	0	0	0	0	0	0	0	0	0
d__Bacteriap__Firmicutesc__Clostridiao__Lachnospiralesf__Lachnospiraceaeg__Roseburia	0	0	0	0	0	0	0	0	4	0	0	0
d__Bacteriap__Proteobacteriac__Gammaproteobacteriao__Pseudomonadalesf__Moraxellaceaeg__Enhydrobacter	0	0	0	0	0	0	0	0	4	0	3	0

Bacteria	L1_ASR5ii	L2__ASR5	L3__ASR5i	L4__ASR5i	L5__ASR5i	L6__ASR5i	L1_WT	L2_WT	L3_WT	L4_WT	L5_WT	L6_WT
d__Bacteriap__Proteobacteriac__Gammaproteobacteriao__Diplorickettsialesf__Diplorickettsiaceaeg__Rickettsiella	0	0	0	4	0	0	0	0	0	0	0	0
d__Bacteriap__Proteobacteriac__Alphaproteobacteriao__unculturedf__unculturedg__uncultured	0	0	0	4	0	0	0	0	0	0	0	0
d__Bacteriap__Proteobacteriac__Gammaproteobacteriao__Burkholderialesf__Burkholderiaceaeg__Ralstonia	0	0	0	0	0	4	0	0	0	4	0	0
d__Bacteriap__Cyanobacteriac__Cyanobacteriiao__Cyanobacterialesf__Nostocaceaeg__Nostoc_PCC-8976	0	0	0	0	0	0	0	0	0	4	0	0
d__Bacteriap__Actinobacteriotac__Actinobacteriao__Micromonosporalesf__Micromonosporaceaeg__Planosporangium	0	0	0	0	4	0	0	0	0	0	0	0
d__Bacteriap__Firmicutesc__Clostridiao__Clostridialesf__Clostridiaceaeg__Clostridium_sensu_stricto_9	0	0	0	0	0	0	0	0	0	0	4	0
d__Bacteriap__Desulfobacterotac__Desulfuromonadiao__Geobacteralesf__Geobacteraceaeg__Citrifermentans	0	0	0	0	0	6	0	0	0	0	0	0
d__Bacteriap__Patescibacteriac__Parcubacteriao__Candidatus_Moranbacteriaf__Candidatus_Moranbacteriag__Candidatus_Moranbacteria	3	0	0	0	0	0	0	0	0	0	0	0
d__Bacteriap__Proteobacteriac__Gammaproteobacteriao__Burkholderialesf__Methylophilaceae__	3	0	0	0	0	0	0	0	0	0	0	0
d__Bacteriap__Proteobacteriac__Gammaproteobacteriao__Burkholderialesf__Methylophilaceaeg__uncultured	3	0	0	0	0	0	0	0	0	0	0	0
d__Bacteriap__Proteobacteriac__Gammaproteobacteriao__Burkholderialesf__Burkholderiaceaeg__Cupriavidus	3	0	0	0	0	0	0	0	0	0	0	0
d__Bacteriap__Proteobacteriac__Alphaproteobacteriao__Azospirillalesf__Azospirillaceaeg__Skermanella	0	0	0	0	0	0	3	0	0	0	0	0

Bacteria	L1_ASR5ii	L2__ASR5	L3__ASR5i	L4__ASR5i	L5__ASR5i	L6__ASR5i	L1_WT	L2_WT	L3_WT	L4_WT	L5_WT	L6_WT
d__Bacteriap__Bacteroidotac__Bacteroidiao__Chitinophagalesf__Chitinophagaceaeg__Parafilimonas	0	0	0	0	0	0	3	0	0	0	0	0
d__Bacteriap__Actinobacteriotac__Actinobacteriao__Micrococcalesf__Micrococcaceaeg__Rothia	0	3	0	0	0	0	0	0	0	0	0	0
d__Bacteriap__Myxococcotac__Polyangiao__Haliangialesf__Haliangiaceaeg__Haliangium	0	0	0	0	0	0	0	3	0	0	0	0
d__Bacteriap__Proteobacteriac__Alphaproteobacteriao__Rhizobialesf__Beijerinckiaceae__	0	0	0	0	0	0	0	3	0	0	0	0
d__Bacteriap__Acidobacteriotac__Acidobacteriaeo__AKIW659f__AKIW659g__AKIW659	0	0	3	0	0	0	0	0	0	0	0	0
d__Bacteriap__Actinobacteriotac__Acidimicrobiiao__unculturedf__unculturedg__uncultured	0	0	3	0	0	0	0	0	0	0	0	0
d__Bacteriap__Verrucomicrobiotac__Verrucomicrobiaeo__Pedosphaeralesf__Pedosphaeraceae__	0	0	3	0	0	0	0	0	0	0	0	0
d__Bacteriap__Acidobacteriotac__Acidobacteriaeo__Acidobacterialesf__Acidobacteriaceae_(Subgroup_1)g__Occallatibacter	0	0	3	0	0	0	0	0	0	0	0	0
d__Bacteriap__Fusobacteriotac__Fusobacteriiao__Fusobacterialesf__Leptotrichiaceaeg__Sneathia	0	0	0	0	0	0	0	0	3	0	0	0
d__Bacteriap__Myxococcotac__Polyangiao__Polyangialesf__Polyangiaceaeg__Pajaroellobacter	0	0	0	3	0	0	0	0	0	0	0	0
d__Bacteriap__Firmicutesc__Clostridiao__Peptostreptococcales-Tissierellalesf__Peptostreptococcaceaeg__Peptostreptococcus	0	0	0	0	0	0	0	0	0	3	0	0
d__Bacteriap__Actinobacteriotac__Coriobacteriiao__OPB41f__OPB41g__OPB41	0	0	0	0	0	3	0	0	0	0	0	0
d__Bacteriap__Firmicutesc__Thermoanaerobacteriao__Thermoanaerobacteralesf__SRB2g__SRB2	0	0	0	0	0	3	0	0	0	0	0	0

Bacteria	L1_ASR5ii	L2__ASR5	L3__ASR5i	L4__ASR5i	L5__ASR5i	L6__ASR5i	L1_WT	L2_WT	L3_WT	L4_WT	L5_WT	L6_WT
d__Bacteriap__Myxococcotac__bacteriap25o__bacteriap25f__bacteriap25g__bacteriap25	0	0	0	0	0	0	0	0	0	0	0	3
d__Archaeap__Halobacterotac__Methanocelliao__Methanocellalesf__Methanocellaceaeg__Methanocella	2	0	0	0	0	0	0	0	0	0	0	0
d__Bacteriap__Verrucomicrobiotac__Verrucomicrobiaeo__Chthoniobacteralesf__Chthoniobacteraceae__	2	0	0	0	0	0	0	0	0	0	0	0
d__Bacteriap__Chloroflexic__Ktedonobacteriao__Ktedonobacteralesf__Ktedonobacteraceae__	2	0	0	0	0	0	0	0	0	0	0	0
d__Bacteriap__Firmicutesc__Clostridiao__Christensenellalesf__Christensenellaceaeg__Christensenellaceae_R-7_group	2	0	0	0	0	0	0	0	0	0	0	0
d__Bacteriap__Firmicutesc__Clostridiao__Peptostreptococcales-Tissierellalesf__Anaerovoracaceaeg__Amnipila	2	0	0	0	0	0	0	0	0	0	0	0
d__Bacteriap__Firmicutesc__Bacillio__Lactobacillalesf__Carnobacteriaceaeg__Atopostipes	2	0	0	0	0	0	0	0	0	0	0	0
d__Bacteriap__Bacteroidotac__Bacteroidiao__Chitinophagalesf__Chitinophagaceaeg__Edaphobaculum	2	0	0	0	0	0	0	0	0	0	0	0
d__Bacteriap__Verrucomicrobiotac__Verrucomicrobiaeo__Chthoniobacteralesf__Chthoniobacteraceaeg__Candidatus_Udaeobacter	2	0	0	0	0	0	0	0	0	0	0	0
d__Bacteriap__Actinobacteriotac__Actinobacteriao__Catenulisporalesf__Catenulisporaceaeg__Catenulispora	2	0	0	0	0	0	0	0	0	0	0	0
d__Bacteriap__Acidobacteriotac__Acidobacteriae______	2	0	0	0	0	0	0	0	0	0	0	0
d__Bacteriap__Chloroflexic__Chloroflexiao__Thermomicrobialesf__JG30-KF-CM45g__JG30-KF-CM45	0	0	0	0	0	0	2	0	0	0	0	0
d__Bacteriap__Proteobacteriac__Alphaproteobacteriao__Paracaedibacteralesf__Paracaedibacteraceaeg__Candidatus_Finniella	0	0	0	0	0	0	2	0	0	0	0	0

**Bacteria**	**L1_ASR5ii**	**L2__ASR5**	**L3__ASR5i**	**L4__ASR5i**	**L5__ASR5i**	**L6__ASR5i**	**L1_WT**	**L2_WT**	**L3_WT**	**L4_WT**	**L5_WT**	**L6_WT**
d__Bacteriap__Proteobacteriac__Alphaproteobacteriao__Defluviicoccalesf__Defluviicoccaceaeg__Defluviicoccus	0	0	0	0	0	0	2	0	0	0	0	0
d__Bacteriap__Proteobacteriac__Alphaproteobacteriao__Sphingomonadalesf__Sphingomonadaceaeg__Sphingobium	0	0	0	0	0	0	2	0	0	0	0	0
d__Bacteriap__Proteobacteriac__Alphaproteobacteriao__Rhizobialesf__Beijerinckiaceaeg__Methylocella	0	0	0	0	0	0	2	0	0	0	0	0
d__Bacteriap__Chloroflexic__Anaerolineaeo__Anaerolinealesf__Anaerolineaceaeg__Anaerolinea	0	2	0	0	0	0	0	0	0	0	0	0
d__Bacteriap__Verrucomicrobiotac__Verrucomicrobiaeo__Pedosphaeralesf__Pedosphaeraceaeg__Pedosphaera	0	2	0	0	0	0	0	0	0	0	0	0
d__Bacteriap__Actinobacteriotac__Actinobacteriao__Propionibacterialesf__Propionibacteriaceaeg__Cutibacterium	0	0	0	2	0	0	0	2	0	0	0	0
d__Bacteriap__Actinobacteriotac__Actinobacteriao__Corynebacterialesf__Mycobacteriaceaeg__Mycobacterium	0	0	2	0	0	0	0	0	0	0	0	0
d__Bacteriap__Firmicutesc__Bacillio__Lactobacillalesf__Aerococcaceaeg__Aerococcus	0	0	2	0	0	0	0	0	0	0	0	0
d__Bacteriap__Proteobacteriac__Gammaproteobacteriao__Burkholderialesf__Oxalobacteraceaeg__Herbaspirillum	0	0	0	0	0	0	0	0	0	2	0	0
d__Eukaryotap__Protalveolatac__Syndinialeso__Syndinialesf__Syndinialesg__Amoebophrya	0	0	0	0	2	0	0	0	0	0	0	0
d__Bacteriap__Proteobacteriac__Alphaproteobacteriao__Rhizobialesf__Beijerinckiaceaeg__Rhodoblastus	0	0	0	0	2	0	0	0	0	0	0	0
d__Bacteriap__Actinobacteriotac__Acidimicrobiiao__Microtrichalesf__Ilumatobacteraceaeg__uncultured	0	0	0	0	0	2	0	0	0	0	2	0

**Bacteria**	**L1_ASR5ii**	**L2__ASR5**	**L3__ASR5i**	**L4__ASR5i**	**L5__ASR5i**	**L6__ASR5i**	**L1_WT**	**L2_WT**	**L3_WT**	**L4_WT**	**L5_WT**	**L6_WT**
d__Bacteriap__Actinobacteriotac__Actinobacteriao__Propionibacterialesf__Nocardioidaceae__	0	0	0	0	0	0	0	0	0	0	2	0
d__Bacteriap__Bacteroidotac__Bacteroidiao__Bacteroidalesf__SB-5g__SB-5	0	0	0	0	0	2	0	0	0	0	0	0
d__Bacteriap__Gemmatimonadotac__Gemmatimonadeteso__Gemmatimonadalesf__Gemmatimonadaceae__	0	0	0	0	0	2	0	0	0	0	0	0
d__Bacteriap__Proteobacteriac__Gammaproteobacteriao__Diplorickettsialesf__Diplorickettsiaceaeg__Aquicella	0	0	0	0	0	2	0	0	0	0	0	0
d__Bacteriap__Proteobacteriac__Alphaproteobacteriao__Rhizobiales____	0	0	0	0	0	2	0	0	0	0	0	0
d__Bacteriap__Actinobacteriotac__Thermoleophiliao__Solirubrobacteralesf__Solirubrobacteraceaeg__Conexibacter	0	0	0	0	0	0	0	0	0	0	0	2
	1425	2003	1763	1554	284	273	651	364	658	1019	534	213
