# Supplementary material for: The impact of OsASR5 on rice microbiome: Implications for nutrient cycling and stress response
Source: Genet Mol Biol. 2026 Oct 26;49(Suppl 3):e20250064. doi: 10.1590/1678-4685-GMB-2025-0064 (PMC13720868; doi:10.1590/1678-4685-GMB-2025-0064)
Supplement: Supplementary file 9 [file 1415-4757-GMB-49-s3-e20250064-s9.pdf]

Genetics and Molecular Biology

https://doi.org/10.1590/1678-4685-GMB-2025-0064

Supplementary Material to "The impact of OsASR5 on rice microbiome: Implications for nutrient cycling and stress response"

Table S9 - Bacterial genera abundance per sample from root microbiome.

**Bacteria**	**R1__ASR5i**	**R2__ASR5i**	**R3__ASR5i**	**R4__ASR5i**	**R5__ASR5i**	**R6__ASR5i**	**R1_WT**	**R2_WT**	**R3_WT**	**R4_WT**	**R5_WT**	**R6_WT**
d__Bacteriap__Proteobacteria________	46	175	62	181	104	227	41	383	548	265	159	526
d__Bacteriap__Proteobacteriac__Alphaproteobacteriao__Caulobacteralesf__Caulobacteraceae__	14	21	12	28	21	109	5	12	36	43	28	21
d__Bacteriap__Desulfobacterotac__Desulfuromonadiao__Geobacteralesf__Geobacteraceaeg__Geotalea	53	71	5	47	84	111	0	142	53	251	88	197
d__Bacteriap__Bacteroidotac__Bacteroidiao__Sphingobacterialesf__Sphingobacteriaceae__	0	2	0	0	2	2	0	3	8	0	2	2
d__Bacteriap__Proteobacteriac__Gammaproteobacteria______	86	174	185	114	126	479	173	377	525	372	418	547
d__Bacteriap__Acidobacteriotac__Acidobacteriaeo__Acidobacterialesf__Acidobacteriaceae_(Subgroup_1)__	905	1112	1305	959	1374	3553	768	821	1608	1454	1473	1924
d__Bacteriap__Actinobacteriotac__Thermoleophiliao__Gaiellalesf__unculturedg__uncultured	17	6	69	26	32	147	51	28	35	35	62	59
d__Bacteriap__Proteobacteriac__Gammaproteobacteriao__Xanthomonadalesf__Rhodanobacteraceaeg__Rhodanobacter	316	291	592	233	232	1142	466	303	568	443	654	566
d__Bacteriap__Bacteroidotac__Bacteroidiao__Sphingobacterialesf__CWT_CU03-E12g__CWT_CU03-E12	6	0	7	10	2	0	5	7	8	18	16	25
d__Bacteria__________	492	1004	709	855	875	2442	299	1068	1604	1090	1009	1567
d__Bacteriap__Firmicutes________	22	34	34	21	30	183	0	109	123	30	59	77
d__Bacteriap__Desulfobacterota________	13	28	28	37	43	88	0	23	37	54	26	73

Bacteria	R1__ASR5i	R2__ASR5i	R3__ASR5i	R4__ASR5i	R5__ASR5i	R6__ASR5i	R1_WT	R2_WT	R3_WT	R4_WT	R5_WT	R6_WT
d__Bacteriap__Actinobacteriotac__Actinobacteria______	39	76	118	77	172	355	126	84	172	198	196	89
d__Bacteriap__Proteobacteriac__Alphaproteobacteriao__Rhizobiales____	88	124	181	90	159	540	80	140	119	170	152	198
d__Bacteriap__Firmicutesc__Clostridia______	124	170	151	249	231	355	143	226	372	276	478	395
d__Bacteriap__Myxococcotac__Polyangiao__mle1-27f__mle1-27g__mle1-27	7	24	48	34	13	94	0	34	0	30	14	15
d__Bacteriap__Proteobacteriac__Alphaproteobacteriao__Elsteralesf__unculturedg__uncultured	261	135	266	171	136	655	178	139	453	340	460	391
d__Bacteriap__Chloroflexic__Anaerolineaeo__Anaerolinealesf__Anaerolineaceae__	11	52	34	32	31	127	34	19	18	44	73	54
d__Bacteriap__Proteobacteriac__Alphaproteobacteria______	357	439	450	294	392	1268	318	354	772	572	486	734
d__Bacteriap__Planctomycetotac__Planctomyceteso__Gemmatalesf__Gemmataceaeg__Gemmata	0	0	0	0	0	0	0	0	3	0	0	0
d__Bacteriap__Chloroflexic__Anaerolineaeo__Anaerolinealesf__Anaerolineaceaeg__Anaerolinea	23	88	53	53	68	208	11	106	94	57	106	102
d__Bacteriap__Proteobacteriac__Alphaproteobacteriao__Sphingomonadalesf__Sphingomonadaceae__	73	94	131	101	88	214	82	39	88	54	109	49
d__Bacteriap__Bacteroidotac__Bacteroidiao__Bacteroidalesf__Bacteroidetes_vadinHA17g__Bacteroidetes_vadinHA17	27	73	39	27	66	100	6	30	112	27	78	121
d__Bacteriap__Proteobacteriac__Gammaproteobacteriao__Burkholderialesf__Rhodocyclaceae__	1546	5439	2496	4058	1461	4069	2072	3885	7080	4390	4239	5720
d__Bacteriap__Firmicutesc__Desulfitobacteriiao__Desulfitobacterialesf__Desulfitobacteriaceaeg__Desulfitobacterium	5	0	21	6	0	11	4	4	19	14	27	11
d__Bacteriap__Acidobacteriota________	13	4	12	6	22	43	6	14	11	0	9	26
d__Bacteriap__Bacteroidotac__Bacteroidiao__Bacteroidales____	288	632	307	483	379	737	246	882	845	714	771	892
d__Bacteriap__Proteobacteriac__Alphaproteobacteriao__Rhodospirillalesf__Magnetospirillaceae__	102	165	243	72	116	502	23	108	77	83	142	115
d__Bacteriap__Chloroflexic__Ktedonobacteriao__Ktedonobacteralesf__JG30-KF-AS9g__JG30-KF-AS9	347	88	486	134	341	1109	451	142	807	721	816	368
d__Bacteriap__Firmicutesc__Negativicuteso__Veillonellales-Selenomonadales____	2	0	0	6	5	22	0	0	6	0	19	2
d__Bacteriap__Spirochaetotac__Spirochaetiao__Spirochaetalesf__Spirochaetaceae__	2	10	0	7	57	35	13	12	6	13	13	31
d__Bacteriap__Desulfobacterotac__Desulfuromonadiao__Geobacteralesf__Geobacteraceaeg__Citrifermentans	110	288	306	132	308	484	254	350	319	320	217	245
d__Bacteriap__Myxococcotac__Polyangiao__Haliangialesf__Haliangiaceaeg__Haliangium	193	511	393	498	435	698	332	430	450	369	626	615
d__Bacteriap__Desulfobacterotac__Syntrophobacteriao__Syntrophobacteralesf__Syntrophobacteraceaeg__Syntrophobacter	18	26	76	39	18	85	65	72	20	90	29	102

Bacteria	R1__ASR5i	R2__ASR5i	R3__ASR5i	R4__ASR5i	R5__ASR5i	R6__ASR5i	R1_WT	R2_WT	R3_WT	R4_WT	R5_WT	R6_WT
d__Bacteriap__Desulfobacterotac__Syntrophiao__Syntrophalesf__unculturedg__uncultured	4	0	0	2	0	10	4	0	0	11	0	8
d__Bacteriap__Acidobacteriotac__Holophagaeo__Holophagalesf__Holophagaceae__	413	703	537	560	595	1095	646	440	551	525	665	620
d__Bacteriap__Verrucomicrobiotac__Verrucomicrobiaeo__Pedosphaeralesf__Pedosphaeraceae__	58	40	16	19	19	70	22	39	23	28	33	72
d__Bacteriap__Bacteroidotac__Kryptoniao__Kryptonialesf__BSV26g__BSV26	70	24	69	33	144	216	7	36	61	92	145	113
d__Bacteriap__Myxococcotac__Myxococciao__Myxococcalesf__Anaeromyxobacteraceaeg__Anaeromyxobacter	280	405	606	255	1019	1367	282	168	498	205	566	447
d__Bacteriap__Proteobacteriac__Gammaproteobacteriao__Burkholderialesf__Comamonadaceae__	208	803	795	542	572	1919	376	415	268	873	496	355
d__Bacteriap__Proteobacteriac__Alphaproteobacteriao__Micropepsalesf__Micropepsaceaeg__uncultured	386	204	474	249	265	1186	362	183	1164	634	1122	707
d__Bacteriap__Firmicutesc__Clostridiao__Clostridialesf__Clostridiaceaeg__Clostridium_sensu_stricto_1	228	353	554	265	430	780	469	145	1113	457	606	444
d__Bacteriap__Actinobacteriotac__Actinobacteriao__Kineosporialesf__Kineosporiaceae__	123	486	325	251	393	838	167	313	122	236	159	283
d__Bacteriap__Desulfobacterotac__Syntrophorhabdiao__Syntrophorhabdalesf__Syntrophorhabdaceaeg__Syntrophorhabdus	5	9	15	4	13	25	12	4	17	28	6	19
d__Bacteriap__Actinobacteriotac__Coriobacteriiao__OPB41f__OPB41g__OPB41	50	70	91	61	68	257	148	55	143	52	173	53
d__Bacteriap__Planctomycetotac__Phycisphaeraeo__Tepidisphaeralesf__WD2101_soil_groupg__WD2101_soil_group	2	0	3	7	2	18	3	12	2	7	16	18
d__Bacteriap__Chloroflexic__Ktedonobacteriao__Ktedonobacteralesf__Ktedonobacteraceae__	41	29	61	21	44	92	78	25	26	106	152	56
d__Bacteriap__Acidobacteriotac__Acidobacteriaeo__Subgroup_13f__Subgroup_13g__Subgroup_13	10	14	28	13	0	54	10	18	20	13	8	36
d__Bacteriap__Desulfobacterotac__Desulfobulbiao__Desulfobulbalesf__unculturedg__uncultured	0	0	9	4	0	37	8	33	5	9	0	10
d__Bacteriap__Firmicutesc__Bacillio__Paenibacillalesf__Paenibacillaceaeg__Paenibacillus	12	133	28	5	12	55	34	13	65	21	49	18
d__Bacteriap__Firmicutesc__Bacillio__Bacillales____	5	2	0	6	0	11	5	4	0	16	10	15
d__Bacteriap__Firmicutesc__Negativicutes______	0	0	0	0	0	0	0	0	2	0	0	0
d__Bacteriap__Nitrospirotac__Thermodesulfovibrioniao__unculturedf__unculturedg__uncultured	27	27	110	17	30	256	26	125	30	81	12	84
d__Bacteriap__Bacteroidotac__Ignavibacteriao__Ignavibacteriales____	50	3	74	84	63	150	33	58	113	130	106	110
d__Bacteriap__Acidobacteriotac__Holophagaeo__Subgroup_7f__Subgroup_7g__Subgroup_7	9	4	14	0	2	30	0	4	0	12	0	9
d__Bacteriap__Firmicutesc__Bacillio__Bacillalesf__Bacillaceaeg__Bacillus	246	362	509	293	174	1278	353	163	388	302	352	567

Bacteria	R1__ASR5i	R2__ASR5i	R3__ASR5i	R4__ASR5i	R5__ASR5i	R6__ASR5i	R1_WT	R2_WT	R3_WT	R4_WT	R5_WT	R6_WT
d__Bacteriap__Chloroflexic__TK10o__TK10f__TK10g__TK10	13	0	5	8	0	20	0	7	0	0	4	2
d__Bacteriap__Firmicutesc__Clostridiao__Clostridiales____	4	16	4	8	22	36	29	13	78	7	44	23
d__Bacteriap__Proteobacteriac__Alphaproteobacteriao__Rhizobialesf__Xanthobacteraceae__	434	301	868	380	597	1408	828	220	519	745	907	695
d__Bacteriap__Acidobacteriotac__Acidobacteriaeo__Acidobacterialesf__unculturedg__uncultured	293	167	486	229	329	1166	182	367	402	462	332	845
d__Bacteriap__Proteobacteriac__Alphaproteobacteriao__Rickettsiales____	0	0	4	0	0	0	0	14	0	49	0	20
d__Bacteriap__Verrucomicrobiotac__Verrucomicrobiaeo__Pedosphaeralesf__Pedosphaeraceaeg__uncultured	12	135	135	67	74	410	44	120	78	132	85	200
d__Bacteriap__Proteobacteriac__Alphaproteobacteriao__Rhizobialesf__Xanthobacteraceaeg__Pseudolabrys	55	110	145	119	98	320	201	86	163	107	232	146
d__Bacteriap__Proteobacteriac__Gammaproteobacteriao__Xanthomonadales____	22	4	0	0	4	2	0	4	54	4	0	0
d__Bacteriap__Armatimonadotac__Fimbriimonadiao__Fimbriimonadalesf__Fimbriimonadaceaeg__Fimbriimonadaceae	0	6	14	6	5	81	14	10	9	22	7	27
d__Bacteriap__Proteobacteriac__Gammaproteobacteriao__Burkholderiales____	2121	5016	3889	3619	2885	6557	3755	7764	7645	6860	5063	6060
d__Bacteriap__Firmicutesc__Clostridiao__Clostridiaf__Hungateiclostridiaceae__	169	334	266	266	251	663	156	408	728	523	710	1171
d__Bacteriap__Nitrospirotac__4-29-1o__4-29-1f__4-29-1g__4-29-1	6	0	38	4	2	30	4	44	4	38	0	15
d__Bacteriap__Acidobacteriotac__Acidobacteriaeo__Bryobacteralesf__Bryobacteraceaeg__Bryobacter	140	95	114	66	111	364	70	55	121	116	177	259
d__Bacteriap__Desulfobacterotac__Desulfuromonadia______	10	15	5	7	24	51	0	0	13	0	30	17
d__Bacteriap__Bacteroidotac__Kapabacteriao__Kapabacterialesf__Kapabacterialesg__Kapabacteriales	22	8	13	16	21	44	10	14	3	13	19	6
d__Bacteriap__Desulfobacterotac__Desulfobacciao__Desulfobaccalesf__Desulfobaccaceaeg__Desulfobacca	16	0	41	17	12	104	17	19	20	37	44	45
d__Bacteriap__Nitrospirotac__Nitrospiriao__Nitrospiralesf__Nitrospiraceaeg__Nitrospira	9	6	9	2	0	45	5	20	10	18	10	40
d__Bacteriap__Desulfobacterotac__Desulfuromonadiao__Geobacterales____	16	78	112	76	11	22	52	145	13	197	98	4
d__Bacteriap__Chloroflexic__Ktedonobacteriao__Ktedonobacteralesf__Ktedonobacteraceaeg__FCPS473	6	0	10	3	5	10	8	7	11	31	5	6
d__Bacteriap__Acidobacteriotac__Acidobacteriaeo__Solibacteralesf__Solibacteraceaeg__Candidatus_Solibacter	402	396	533	491	619	1610	303	461	770	716	747	947
d__Bacteriap__Proteobacteriac__Gammaproteobacteriao__Coxiellalesf__Coxiellaceaeg__Coxiella	3	9	10	7	4	8	11	2	0	0	11	12
d__Bacteriap__Desulfobacterotac__Desulfuromonadiao__Geobacteralesf__Geobacteraceae__	285	403	725	202	495	1136	415	682	337	545	595	566

Bacteria	R1__ASR5i	R2__ASR5i	R3__ASR5i	R4__ASR5i	R5__ASR5i	R6__ASR5i	R1_WT	R2_WT	R3_WT	R4_WT	R5_WT	R6_WT
d__Bacteriap__Bacteroidotac__Bacteroidiao__Sphingobacterialesf__env,OPS_17g__env,OPS_17	0	0	0	0	0	0	0	4	0	6	0	0
d__Bacteriap__Bacteroidotac__Bacteroidiao__Chitinophagalesf__Chitinophagaceae__	34	49	130	24	19	166	43	18	84	32	84	19
d__Bacteriap__Verrucomicrobiotac__Verrucomicrobiaeo__Opitutalesf__Opitutaceaeg__Lacunisphaera	5	37	39	38	28	88	11	68	29	102	37	104
d__Bacteriap__Proteobacteriac__Alphaproteobacteriao__Acetobacteralesf__Acetobacteraceae__	221	258	340	180	233	764	376	96	360	250	454	256
d__Bacteriap__Proteobacteriac__Gammaproteobacteriao__Xanthomonadalesf__Xanthomonadaceae__	3	0	0	0	0	0	0	0	31	0	4	2
d__Bacteriap__Firmicutesc__Desulfitobacteriiao__Desulfitobacterialesf__Desulfitobacteriaceaeg__Desulfosporosinus	42	158	117	81	71	152	96	117	113	91	67	139
d__Bacteriap__Myxococcota________	7	15	4	9	10	13	3	6	9	0	4	13
d__Bacteriap__Bacteroidotac__Bacteroidia______	11	37	4	25	18	42	2	25	28	18	26	27
d__Bacteriap__Proteobacteriac__Alphaproteobacteriao__Azospirillales____	0	0	0	0	0	0	0	0	0	0	5	0
d__Bacteriap__Firmicutesc__Bacillio__Bacillalesf__Bacillaceae__	34	21	63	46	15	104	13	23	35	11	51	66
d__Bacteriap__Firmicutesc__Clostridiao__Christensenellalesf__Christensenellaceaeg__uncultured	41	50	83	70	72	106	29	54	84	45	124	93
d__Bacteriap__Acidobacteriotac__Acidobacteriaeo__Subgroup_2f__Subgroup_2g__Subgroup_2	106	87	134	24	39	270	60	102	136	150	124	154
d__Bacteriap__Proteobacteriac__Gammaproteobacteriao__Enterobacteralesf__Enterobacteriaceae__	9	26	54	0	54	19	0	0	92	0	0	0
d__Bacteriap__Proteobacteriac__Gammaproteobacteriao__Burkholderialesf__SC-I-84g__SC-I-84	190	67	313	82	120	598	245	0	297	173	383	195
d__Bacteriap__Proteobacteriac__Alphaproteobacteriao__Caulobacteralesf__Caulobacteraceaeg__uncultured	104	144	304	112	180	541	142	104	217	235	265	256
d__Bacteriap__Actinobacteriotac__Acidimicrobiiao__unculturedf__unculturedg__uncultured	39	46	77	19	45	148	77	69	53	34	89	85
d__Bacteriap__Firmicutesc__Clostridiao__Clostridiaf__Hungateiclostridiaceaeg__HN-HF0106	51	158	25	100	80	90	50	127	204	128	190	255
d__Bacteriap__Bacteroidotac__Bacteroidiao__Chitinophagales____	3	0	0	0	0	8	5	0	2	3	0	5
d__Bacteriap__Proteobacteriac__Gammaproteobacteriao__Burkholderialesf__Nitrosomonadaceaeg__Ellin6067	40	24	70	0	0	178	23	43	42	37	77	53
d__Bacteriap__Firmicutesc__Clostridiao__Lachnospiralesf__Lachnospiraceae__	34	104	85	104	91	196	61	137	388	173	210	371
d__Bacteriap__Proteobacteriac__Alphaproteobacteriao__Defluviicoccalesf__unculturedg__uncultured	0	0	0	0	0	0	0	0	0	0	12	5

Bacteria	R1__ASR5i	R2__ASR5i	R3__ASR5i	R4__ASR5i	R5__ASR5i	R6__ASR5i	R1_WT	R2_WT	R3_WT	R4_WT	R5_WT	R6_WT
d__Bacteriap__Desulfobacterotac__Syntrophobacteriao__Syntrophobacteralesf__Syntrophobacteraceae__	33	127	106	50	21	138	46	134	24	133	86	226
d__Bacteriap__Fibrobacterotac__Fibrobacteriao__Fibrobacterales____	0	3	0	4	0	4	2	5	0	2	2	10
d__Bacteriap__Gemmatimonadotac__Gemmatimonadeteso__Gemmatimonadalesf__Gemmatimonadaceae__	14	10	14	3	14	187	44	27	11	68	140	39
d__Bacteriap__Bacteroidotac__Bacteroidiao__Chitinophagalesf__Chitinophagaceaeg__uncultured	75	27	74	17	31	275	70	6	85	112	147	33
d__Bacteriap__Proteobacteriac__Alphaproteobacteriao__Rhizobialesf__Rhizobiales_Incertae_Sedisg__Bauldia	0	0	5	0	0	4	0	16	6	0	16	17
d__Bacteriap__Proteobacteriac__Gammaproteobacteriao__Diplorickettsialesf__Diplorickettsiaceaeg__uncultured	17	21	29	8	3	40	35	4	35	35	62	28
d__Bacteriap__Proteobacteriac__Alphaproteobacteriao__Rickettsialesf__SM2D12g__SM2D12	5	4	12	3	2	18	15	11	7	8	9	9
d__Bacteriap__Actinobacteriotac__Actinobacteriao__Micrococcalesf__Microbacteriaceae__	0	0	6	2	5	27	28	3	60	36	38	26
d__Bacteriap__Proteobacteriac__Gammaproteobacteriao__Methylococcalesf__Methylomonadaceaeg__Methylomonas	0	323	896	78	602	452	320	20	644	115	257	12
d__Bacteriap__Proteobacteriac__Alphaproteobacteriao__Rhodospirillalesf__Magnetospirillaceaeg__Telmatospirillum	35	139	82	54	58	129	60	75	92	78	98	69
d__Bacteriap__Acidobacteriotac__Acidobacteriaeo__Acidobacteriales____	136	50	96	40	141	362	51	61	149	81	133	108
d__Bacteriap__Proteobacteriac__Gammaproteobacteriao__Methylococcalesf__Methylomonadaceae__	2	59	21	14	9	47	2	5	40	19	3	0
d__Bacteriap__Firmicutesc__Clostridiao__Christensenellalesf__Christensenellaceaeg__Christensenellaceae_R-7_group	6	77	29	57	38	39	28	55	45	28	61	58
d__Bacteriap__Chloroflexic__Anaerolineae______	22	13	3	4	27	56	3	24	22	33	36	37
d__Bacteriap__Actinobacteriotac__Acidimicrobiiao__IMCC26256f__IMCC26256g__IMCC26256	22	0	16	0	39	109	42	0	28	25	85	40
d__Bacteriap__Verrucomicrobiotac__Chlamydiaeo__Chlamydialesf__Simkaniaceae__	3	0	0	0	0	3	0	3	0	0	0	0
d__Bacteriap__Verrucomicrobiotac__Verrucomicrobiaeo__Pedosphaeralesf__Pedosphaeraceaeg__Pedosphaeraceae	7	6	31	9	47	111	16	27	40	24	53	37
d__Bacteriap__Proteobacteriac__Gammaproteobacteriao__Gammaproteobacteria_Incertae_Sedisf__Unknown_Familyg__Acidibacter	324	287	338	250	189	776	307	130	513	337	601	672
d__Bacteriap__Patescibacteriac__Gracilibacteriao__Gracilibacteriaf__Gracilibacteriag__Gracilibacteria	30	24	36	16	21	129	14	51	7	97	15	31
d__Bacteriap__Planctomycetotac__Phycisphaeraeo__Tepidisphaeralesf__CPla-3_termite_groupg__CPla-3_termite_group	0	0	0	0	5	14	0	0	2	0	0	5

Bacteria	R1__ ASR 5i	R2__ ASR 5i	R3__ ASR 5i	R4__ ASR 5i	R5__ ASR 5i	R6__ ASR 5i	R1 _W T	R2 _W T	R3 _W T	R4 _W T	R5 _W T	R6 _W T
d__Bacteriap__Firmicutesc__Clostridiao__Christensenellalesf__Christensenellaceae__	23	35	8	46	34	73	15	64	76	41	73	33
d__Bacteriap__Actinobacteriotac__Actinobacteriao__Catenulisporalesf__Actinospicaceaeg__Actinospica	0	10	10	19	6	11	20	9	14	11	22	26
d__Bacteriap__Bacteroidotac__Bacteroidiao__Sphingobacterialesf__Sphingobacteriaceaeg__Mucilaginibacter	37	55	50	49	49	109	72	61	63	131	106	91
d__Bacteriap__Firmicutesc__Clostridiao__Clostridialesf__Caloramatoraceaeg__Fonticella	139	312	217	276	158	445	319	322	642	384	716	443
d__Bacteriap__Chloroflexic__Ktedonobacteriao__Ktedonobacteralesf__Ktedonobacteraceaeg__uncultured	14	13	35	5	0	19	10	0	35	45	26	15
d__Bacteriap__Actinobacteriotac__Actinobacteriao__Frankialesf__Acidothermaceaeg__Acidothermus	225	133	417	161	250	726	449	137	389	573	622	364
d__Bacteriap__Elusimicrobiotac__Elusimicrobiao__Lineage_IVf__Lineage_IVg__Lineage_IV	8	11	19	11	12	42	11	16	17	22	28	15
d__Bacteriap__Cyanobacteriac__Vampirivibrioniao__Gastranaerophilalesf__Gastranaerophilalesg__Gastranaerophilales	0	17	8	15	14	25	17	10	19	0	22	20
d__Bacteriap__Acidobacteriotac__Acidobacteriaeo__Acidobacterialesf__Koribacteraceaeg__Candidatus_Koribacter	406	283	553	363	689	1206	269	293	707	725	948	794
d__Bacteriap__Bacteroidotac__Bacteroidiao__Bacteroidalesf__Paludibacteraceaeg__uncultured	22	107	35	41	119	145	77	492	235	167	244	234
d__Bacteriap__Proteobacteriac__Gammaproteobacteriao__Methylococcalesf__Methylomonadaceaeg__Methylobacter	135	375	285	57	241	424	98	38	180	8	108	37
d__Bacteriap__Verrucomicrobiotac__Chlamydiaeo__Chlamydialesf__Parachlamydiaceaeg__uncultured	0	0	2	0	0	0	0	2	0	0	0	0
d__Bacteriap__Actinobacteriotac__MB-A2-108o__MB-A2-108f__MB-A2-108g__MB-A2-108	0	0	0	0	0	0	0	0	0	3	0	5
d__Bacteriap__Firmicutesc__Clostridiao__Clostridialesf__Clostridiaceaeg__Clostridium_sensu_stricto_10	53	17	67	50	109	149	91	0	50	21	95	14
d__Bacteriap__Proteobacteriac__Gammaproteobacteriao__Xanthomonadalesf__Rhodanobacteraceae__	39	36	26	22	22	129	6	25	58	13	22	119
d__Bacteriap__Actinobacteriotac__Thermoleophiliao__Solirubrobacteralesf__Solirubrobacteraceaeg__Conexibacter	15	24	25	0	30	86	38	22	32	0	65	24
d__Bacteriap__Acidobacteriotac__Acidobacteriaeo__Acidobacterialesf__Acidobacteriaceae_(Subgroup_1)g__uncultured	107	101	106	62	192	391	84	58	194	107	335	215
d__Bacteriap__Verrucomicrobiotac__Verrucomicrobiaeo__Opitutalesf__Opitutaceae__	21	16	29	25	0	52	17	44	16	78	49	69
d__Bacteriap__Fibrobacterotac__Fibrobacteriao__Fibrobacteralesf__Fibrobacteralesg__BBMC-4	100	366	153	308	112	220	104	357	313	323	234	520
d__Bacteriap__Verrucomicrobiotac__Verrucomicrobiaeo__Opitutalesf__Opitutaceaeg__Opitutus	0	0	0	0	20	30	13	0	0	0	2	10

Bacteria	R1__ ASR 5i	R2__ ASR 5i	R3__ ASR 5i	R4__ ASR 5i	R5__ ASR 5i	R6__ ASR 5i	R1 _W T	R2 _W T	R3 _W T	R4 _W T	R5 _W T	R6 _W T
d__Bacteriap__Actinobacteriotac__Actinobacteriao__Frankialesf__Geodermatophilaceaeg__Geodermatophilus	0	0	0	0	0	0	0	0	0	0	0	25
d__Bacteriap__Firmicutesc__Limnochordiao__Limnochordiaf__Limnochordiag__Hydrogenispora	63	152	68	83	56	128	55	84	204	94	193	260
d__Bacteriap__Dependentiaec__Babeliaeo__Babeliales____	0	4	0	2	0	4	5	4	2	2	0	2
d__Bacteriap__Patescibacteriac__Berkelbacteriao__Berkelbacteriaf__Berkelbacteriag__Berkelbacteria	8	0	6	6	11	21	2	5	23	11	5	20
d__Bacteriap__Sva0485c__Sva0485o__Sva0485f__Sva0485g__Sva0485	2	3	2	2	0	14	2	4	2	18	9	0
d__Bacteriap__Firmicutesc__Clostridiao__Clostridialesf__Clostridiaceae__	19	235	199	84	126	210	16	85	259	107	153	82
d__Bacteriap__Actinobacteriotac__Actinobacteriao__Propionibacterialesf__Nocardioidaceaeg__Kribbella	0	0	0	0	0	3	6	0	0	6	0	11
d__Bacteriap__Firmicutesc__Clostridiao__Oscillospiralesf__Oscillospiraceae__	21	8	14	0	4	0	0	0	30	5	203	39
d__Bacteriap__Actinobacteriotac__Thermoleophiliao__Solirubrobacteralesf__Solirubrobacteraceae__	0	0	0	2	10	22	3	10	24	2	7	6
d__Bacteriap__Firmicutesc__Clostridiao__Oscillospiralesf__Ruminococcaceae__	0	0	0	0	0	6	5	14	0	0	0	3
d__Bacteriap__Proteobacteriac__Gammaproteobacteriao__Burkholderialesf__Chitinimonadaceaeg__Chitinimonas	0	16	43	10	0	18	15	33	0	67	0	29
d__Bacteriap__Proteobacteriac__Gammaproteobacteriao__Burkholderialesf__Burkholderiaceaeg__Burkholderia-Caballeronia-Paraburkholderia	110	253	231	95	141	209	188	150	166	57	318	199
d__Bacteriap__Verrucomicrobiotac__Chlamydiaeo__Chlamydialesf__cvE6g__cvE6	0	0	3	0	7	11	0	7	2	0	9	0
d__Bacteriap__Halanaerobiaeotac__Halanaerobiiao__Halanaerobialesf__Halobacteroidaceaeg__uncultured	17	7	22	0	9	0	4	50	42	10	70	13
d__Bacteriap__Firmicutesc__Bacilli______	7	2	3	5	3	28	3	6	2	2	0	14
d__Bacteriap__Patescibacteriac__Saccharimonadiao__Saccharimonadalesf__Saccharimonadalesg__Saccharimonadales	16	25	37	6	24	49	11	15	50	7	14	9
d__Bacteriap__Proteobacteriac__Alphaproteobacteriao__Rhizobialesf__Beijerinckiaceae__	10	67	26	7	44	56	3	0	30	6	26	28
d__Bacteriap__Firmicutesc__Clostridiao__Lachnospiralesf__Defluviitaleaceaeg__Defluviitaleaceae_UCG-011	0	0	0	9	5	2	4	0	2	10	8	8
d__Bacteriap__Patescibacteriac__CPR2o__CPR2f__CPR2g__CPR2	7	4	15	0	5	0	15	3	4	7	27	0
d__Bacteriap__Firmicutesc__Clostridiao__Clostridia____	6	0	5	0	11	48	0	0	34	19	116	15
d__Archaeap__Halobacterotac__Methanocelliao__Methanocellalesf__Methanocellaceaeg__Methanocella	8	27	11	18	34	22	0	14	20	10	60	55
d__Bacteriap__Proteobacteriac__Gammaproteobacteriao__Diplorickettsialesf__Diplorickettsiaceaeg__Rickettsiella	0	0	0	0	0	0	30	0	0	0	12	3

Bacteria	R1__ASR5i	R2__ASR5i	R3__ASR5i	R4__ASR5i	R5__ASR5i	R6__ASR5i	R1_WT	R2_WT	R3_WT	R4_WT	R5_WT	R6_WT
d__Bacteriap__Myxococcotac__Polyangia______	0	8	7	10	4	44	0	9	4	11	8	14
d__Bacteriap__Proteobacteriac__Gammaproteobacteriao__Gammaproteobacteria_Incertae_Sedisf__Unknown_Familyg__Candidatus_Ovatusbacter	0	0	2	0	3	12	0	0	0	5	3	3
d__Bacteriap__Acidobacteriotac__Holophagaeo__Holophagalesf__Holophagaceaeg__Geothrix	321	506	561	350	955	1269	267	346	137	653	205	223
d__Bacteriap__Gemmatimonadotac__Gemmatimonadeteso__Gemmatimonadalesf__Gemmatimonadaceaeg__Gemmatimonas	169	67	228	72	134	481	83	21	271	264	365	211
d__Bacteriap__Proteobacteriac__Gammaproteobacteriao__KF-JG30-C25f__KF-JG30-C25g__KF-JG30-C25	124	34	182	77	49	255	58	122	296	311	287	344
d__Bacteriap__Firmicutesc__Clostridiao__Clostridialesf__Clostridiaceaeg__Clostridium_sensu_stricto_8	27	15	61	0	37	3	0	0	131	10	0	24
d__Bacteriap__Acidobacteriotac__Acidobacteriaeo__Acidobacteriaef__Acidobacteriaeg__Paludibaculum	0	0	0	0	0	31	12	0	0	0	0	0
d__Bacteriap__Proteobacteriac__Gammaproteobacteriao__Burkholderialesf__Comamonadaceaeg__Aquabacterium	43	13	51	9	55	0	0	3	75	84	3	12
d__Bacteriap__Proteobacteriac__Alphaproteobacteriao__Rhizobialesf__Kaistiaceaeg__Kaistia	0	0	0	0	0	0	25	0	0	4	0	0
d__Bacteriap__Bacteroidotac__Bacteroidiao__Chitinophagalesf__Chitinophagaceaeg__Edaphobaculum	25	24	18	14	12	94	22	10	23	31	14	34
d__Bacteriap__Chloroflexic__KD4-96o__KD4-96f__KD4-96g__KD4-96	66	100	70	96	104	472	66	56	143	111	171	146
d__Bacteriap__Bacteroidotac__Bacteroidiao__Cytophagalesf__Amoebophilaceaeg__Candidatus_Amoebophilus	7	3	5	0	0	8	0	4	3	0	0	6
d__Bacteriap__Firmicutesc__Desulfitobacteriiao__Desulfitobacterialesf__Desulfitobacteriaceae__	0	0	0	0	0	0	0	0	0	3	0	0
d__Bacteriap__Proteobacteriac__Alphaproteobacteriao__Sphingomonadalesf__Sphingomonadaceaeg__Sphingomonas	36	26	44	0	27	132	50	22	67	76	80	57
d__Bacteriap__Firmicutesc__Negativicuteso__Veillonellales-Selenomonadalesf__Sporomusaceaeg__uncultured	88	208	214	187	184	885	257	206	397	112	558	418
d__Bacteriap__Desulfobacterotac__Desulfovibrioniao__Desulfovibrionalesf__Desulfovibrionaceaeg__Desulfovibrio	89	236	261	172	200	501	274	357	131	289	222	575
d__Bacteriap__Actinobacteriota________	13	5	11	7	33	79	19	23	36	25	20	39
d__Bacteriap__Patescibacteriac__ABY1o__Candidatus_Falkowbacteriaf__Candidatus_Falkowbacteriag__Candidatus_Falkowbacteria	0	0	0	0	0	5	4	0	4	0	4	10
d__Bacteriap__Bacteroidotac__Bacteroidiao__Cytophagalesf__Microscillaceaeg__uncultured	0	6	0	5	9	20	7	0	2	36	0	12
d__Bacteriap__Actinobacteriotac__Actinobacteriao__Corynebacterialesf__Mycobacteriaceaeg__Mycobacterium	11	10	22	18	32	45	21	10	21	13	79	36

Bacteria	R1__ASR5i	R2__ASR5i	R3__ASR5i	R4__ASR5i	R5__ASR5i	R6__ASR5i	R1_WT	R2_WT	R3_WT	R4_WT	R5_WT	R6_WT
d__Bacteriap__Spirochaetotac__Spirochaetiao__Spirochaetalesf__Spirochaetaceaeg__Spirochaeta	7	0	12	0	12	42	10	16	5	12	7	39
d__Bacteriap__Acidobacteriotac__Vicinamibacteriao__Vicinamibacteralesf__unculturedg__uncultured	13	11	15	14	38	47	17	0	28	32	42	24
d__Bacteriap__Spirochaetotac__Spirochaetiao__Spirochaetalesf__Spirochaetaceaeg__Salinispira	8	0	8	0	22	0	3	0	6	0	4	2
d__Bacteriap__Acidobacteriotac__Acidobacteriae______	34	43	55	64	34	247	3	49	36	60	79	108
d__Bacteriap__Firmicutesc__Negativicuteso__Veillonellales-Selenomonadalesf__Sporomusaceaeg__Sporomusa	0	3	0	0	6	4	0	2	33	0	11	2
d__Bacteriap__Proteobacteriac__Alphaproteobacteriao__Rhizobialesf__Xanthobacteraceaeg__uncultured	64	39	87	66	24	383	116	59	118	50	100	25
d__Bacteriap__Verrucomicrobiotac__Verrucomicrobiaeo__Pedosphaeralesf__Pedosphaeraceaeg__Ellin516	11	8	15	15	14	85	13	26	15	52	30	66
d__Bacteriap__Firmicutesc__Clostridiao__Clostridialesf__Clostridiaceaeg__Clostridium_sensu_stricto_3	11	27	0	0	0	0	16	0	145	8	0	0
d__Bacteriap__Firmicutesc__Bacillio__Paenibacillalesf__Paenibacillaceaeg__Ammoniphilus	8	8	10	8	0	10	7	0	18	0	30	24
d__Bacteriap__Actinobacteriotac__Thermoleophiliao__Solirubrobacteralesf__Solirubrobacteraceaeg__uncultured	18	15	14	10	37	49	49	0	0	15	0	2
d__Bacteriap__Chloroflexic__AD3o__AD3f__AD3g__AD3	0	0	0	2	3	15	6	10	0	2	10	2
d__Bacteriap__Verrucomicrobiotac__Verrucomicrobiaeo__Chthoniobacteralesf__Xiphinematobacteraceaeg__Candidatus_Xiphinematobacter	0	0	4	0	14	34	10	5	0	0	8	0
d__Bacteriap__Proteobacteriac__Gammaproteobacteriao__WD260f__WD260g__WD260	79	71	136	63	85	267	155	49	213	118	207	167
d__Bacteriap__Proteobacteriac__Alphaproteobacteriao__Rhizobialesf__Rhizobiaceae__	48	276	407	61	219	354	212	69	178	872	254	61
d__Bacteriap__Actinobacteriotac__Actinobacteriao__Micromonosporalesf__Micromonosporaceaeg__Dactylosporangium	0	0	0	0	0	0	7	0	0	0	0	0
d__Archaeap__Crenarchaeotac__Bathyarchaeiao__Bathyarchaeiaf__Bathyarchaeiag__Bathyarchaeia	8	13	9	0	12	34	6	0	21	4	0	19
d__Bacteriap__Firmicutesc__Bacillio__Alicyclobacillalesf__Alicyclobacillaceaeg__Tumebacillus	77	6	21	15	11	57	68	0	18	22	57	34
d__Bacteriap__Firmicutesc__Bacillio__RF39f__RF39g__RF39	5	11	21	10	13	14	11	17	17	3	54	17
d__Bacteriap__Bacteroidotac__Ignavibacteriao__Ignavibacterialesf__LD-RB-34g__LD-RB-34	28	78	33	42	55	36	14	103	73	105	83	42
d__Bacteriap__Myxococcotac__Myxococciao__Myxococcalesf__Myxococcaceae__	27	16	13	7	10	90	20	9	5	24	4	7
d__Bacteriap__Proteobacteriac__Gammaproteobacteriao__Burkholderialesf__TRA3-20g__TRA3-20	0	3	5	0	0	26	5	0	0	19	17	10

Bacteria	R1__ ASR 5i	R2__ ASR 5i	R3__ ASR 5i	R4__ ASR 5i	R5__ ASR 5i	R6__ ASR 5i	R1 _W T	R2 _W T	R3 _W T	R4 _W T	R5 _W T	R6 _W T
d__Bacteriap__Proteobacteriac__Gammaproteobacteriao__Xanthomonadalesf__Rhodanobacteraceaeg__Rudaea	0	2	0	0	0	0	12	0	0	0	20	0
d__Bacteriap__Cyanobacteria________	0	0	0	0	0	4	0	0	0	0	0	2
d__Bacteriap__Proteobacteriac__Gammaproteobacteriao__Burkholderialesf__Hydrogenophilaceaeg__uncultured	7	12	0	0	0	8	0	29	0	20	0	11
d__Bacteriap__Planctomycetotac__Phycisphaeraeo__Phycisphaeralesf__Phycisphaeraceaeg__uncultured	0	0	0	0	0	2	4	0	0	0	0	0
d__Bacteriap__Acidobacteriotac__Holophagaeo__Holophagalesf__Holophagaceaeg__uncultured	9	69	7	58	42	88	22	28	78	41	10	52
d__Bacteriap__Proteobacteriac__Alphaproteobacteriao__Micropepsalesf__Micropepsaceaeg__Rhizomicrobium	119	396	315	216	234	617	145	416	560	462	480	631
d__Bacteriap__Chloroflexic__JG30-KF-CM66o__JG30-KF-CM66f__JG30-KF-CM66g__JG30-KF-CM66	0	0	5	0	0	11	4	0	0	0	2	0
d__Bacteriap__Proteobacteriac__Gammaproteobacteriao__Burkholderialesf__Burkholderiaceaeg__Ralstonia	2	13	2	51	32	50	24	3	0	31	3	0
d__Bacteriap__Spirochaetotac__Spirochaetiao__Spirochaetalesf__Spirochaetaceaeg__uncultured	140	215	192	105	225	364	96	207	184	246	199	193
d__Bacteriap__Actinobacteriotac__Thermoleophilia______	9	0	0	0	0	3	2	0	0	5	0	0
d__Bacteriap__Desulfobacterotac__Desulfuromonadiao__Geobacteralesf__Geobacteraceaeg__Geobacter	386	694	642	258	610	1132	379	306	360	472	668	380
d__Bacteriap__Proteobacteriac__Gammaproteobacteriao__Diplorickettsialesf__Diplorickettsiaceaeg__Aquicella	0	9	7	5	0	37	12	0	14	15	18	20
d__Bacteriap__Fibrobacterotac__Fibrobacteriao__Fibrobacteralesf__B122g__B122	2	2	6	6	0	15	3	6	12	0	13	13
d__Bacteriap__Chloroflexi________	14	0	30	5	3	24	18	6	16	26	35	12
d__Bacteriap__Patescibacteriac__WWE3o__WWE3f__WWE3g__WWE3	0	2	0	0	0	0	0	0	3	0	0	0
d__Bacteriap__Proteobacteriac__Gammaproteobacteriao__Pseudomonadalesf__Pseudomonadaceaeg__Pseudomonas	0	0	0	0	3	0	0	0	38	0	3	6
d__Bacteriap__Bacteroidotac__Bacteroidiao__Chitinophagalesf__Chitinophagaceaeg__Taibaiella	0	0	0	0	0	0	0	0	4	0	0	0
d__Bacteriap__Armatimonadotac__Armatimonadiao__Armatimonadalesf__Armatimonadalesg__Armatimonadales	0	0	3	0	0	3	4	2	0	0	15	4
d__Bacteriap__Myxococcotac__Polyangiao__Polyangialesf__Polyangiaceaeg__Pajaroellobacter	61	51	52	44	68	191	91	46	72	39	126	132
d__Bacteriap__Proteobacteriac__Gammaproteobacteriao__Burkholderialesf__Rhodocyclaceaeg__Methyloversatilis	1348	2352	1753	4113	1020	2026	2326	4128	5114	3632	3389	5572

Bacteria	R1__ ASR 5i	R2__ ASR 5i	R3__ ASR 5i	R4__ ASR 5i	R5__ ASR 5i	R6__ ASR 5i	R1 _W T	R2 _W T	R3 _W T	R4 _W T	R5 _W T	R6 _W T
d__Bacteriap__Bdellovibrionotac__Oligoflexiao__0319-6G20f__0319-6G20g__0319-6G20	9	17	54	12	18	81	37	11	29	46	54	26
d__Bacteriap__Firmicutesc__Clostridiao__Clostridialesf__Caloramatoraceae__	3	0	0	26	0	9	4	23	4	11	8	0
d__Bacteriap__Firmicutesc__Syntrophomonadiao__Syntrophomonadalesf__Syntrophomonadaceaeg__uncultured	0	0	0	0	4	0	0	0	0	4	8	3
d__Bacteriap__Firmicutesc__Clostridiao__Monoglobalesf__Monoglobaceaeg__Monoglobus	0	0	0	0	10	0	2	0	4	0	0	11
d__Bacteriap__Verrucomicrobiotac__Verrucomicrobiaeo__Pedosphaeralesf__Pedosphaeraceaeg__ADurb,Bin063-1	66	67	88	56	110	311	74	93	122	166	224	168
d__Bacteriap__Chloroflexic__Dehalococcoidiao__FS117-23B-02f__FS117-23B-02g__FS117-23B-02	0	0	0	0	0	0	0	0	0	0	0	2
d__Bacteriap__Proteobacteriac__Gammaproteobacteriao__Pseudomonadalesf__Moraxellaceaeg__Cavicella	0	4	0	0	0	0	0	0	0	9	0	6
d__Bacteriap__Actinobacteriotac__Actinobacteriao__Micromonosporalesf__Micromonosporaceae__	11	10	21	10	19	69	7	12	20	100	58	40
d__Bacteriap__Chloroflexic__SHA-26o__SHA-26f__SHA-26g__SHA-26	5	0	0	0	7	7	5	0	0	4	4	0
d__Bacteriap__Firmicutesc__Clostridiao__Clostridialesf__Oxobacteraceaeg__Oxobacter	19	2	13	16	40	19	0	39	16	19	44	38
d__Bacteriap__Proteobacteriac__Alphaproteobacteriao__Micropepsalesf__Micropepsaceae__	7	11	10	5	0	62	0	51	8	0	37	60
d__Bacteriap__Gemmatimonadotac__Gemmatimonadeteso__Gemmatimonadalesf__Gemmatimonadaceaeg__uncultured	27	2	28	2	17	98	17	13	31	68	66	63
d__Bacteriap__Elusimicrobiotac__Endomicrobiao__Endomicrobialesf__Endomicrobiaceaeg__Endomicrobium	10	16	10	8	18	46	12	19	9	13	19	18
d__Bacteriap__Firmicutesc__Clostridiao__Clostridialesf__Clostridiaceaeg__Clostridium_sensu_stricto_13	0	0	14	10	3	21	0	0	5	14	3	17
d__Bacteriap__Desulfobacterotac__Syntrophobacteriao__Syntrophobacteralesf__Syntrophobacteraceaeg__uncultured	0	0	0	0	0	3	3	0	0	3	3	0
d__Bacteriap__Verrucomicrobiotac__Chlamydiaeo__Chlamydialesf__Simkaniaceaeg__uncultured	0	2	0	0	2	10	0	0	2	5	7	0
d__Bacteriap__Firmicutesc__Clostridiao__Clostridiaf__Gracilibacteraceaeg__Lutispora	63	69	22	67	156	220	0	199	370	391	468	670
d__Bacteriap__Proteobacteriac__Gammaproteobacteriao__Burkholderialesf__Alcaligenaceae__	0	6	3	0	0	0	6	3	30	0	0	0
d__Bacteriap__Firmicutesc__Clostridiao__Clostridialesf__Clostridiaceaeg__Clostridium_sensu_stricto_19	0	4	0	0	9	32	0	5	0	0	0	6
d__Bacteriap__Myxococcotac__Polyangiao__Polyangiales____	3	20	15	4	15	36	5	4	10	23	7	32
d__Bacteriap__Bdellovibrionotac__Bdellovibrioniao__Bdellovibrionalesf__Bdellovibrionaceaeg__Bdellovibrio	8	25	41	8	7	80	4	7	34	108	17	22

Bacteria	R1__ ASR 5i	R2__ ASR 5i	R3__ ASR 5i	R4__ ASR 5i	R5__ ASR 5i	R6__ ASR 5i	R1 _W T	R2 _W T	R3 _W T	R4 _W T	R5 _W T	R6 _W T
d__Bacteriap__Firmicutesc__Bacillio__Alicyclobacillalesf__Alicyclobacillaceaeg__Alicyclobacillus	0	0	6	2	0	3	0	16	8	6	20	0
d__Bacteriap__Proteobacteriac__Alphaproteobacteriao__Caulobacteralesf__Caulobacteraceaeg__Phenylobacterium	62	31	94	55	52	129	70	26	66	151	203	77
d__Bacteriap__Myxococcotac__Myxococciao__Myxococcales___	3	5	4	7	39	12	0	0	2	4	0	7
d__Bacteriap__Spirochaetotac__Spirochaetiao__Spirochaetalesf__Spirochaetaceaeg__Spirochaeta_2	15	50	28	33	36	59	18	92	39	79	43	78
d__Bacteriap__Entotheonellaeotac__Entotheonelliao__Entotheonellalesf__Entotheonellaceaeg__Entotheonellaceae	0	0	0	0	0	0	0	2	0	2	0	0
d__Bacteriap__Actinobacteriotac__Actinobacteriao__Pseudonocardialesf__Pseudonocardiaceaeg__Amycolatopsis	0	0	22	4	2	18	0	0	0	0	4	6
d__Bacteriap__Proteobacteriac__Gammaproteobacteriao__Burkholderialesf__Burkholderiaceae__	0	23	5	23	2	16	8	8	22	32	59	53
d__Bacteriap__Firmicutesc__Bacillio__Bacillalesf__Planococcaceaeg__Solibacillus	0	0	0	0	0	5	0	0	0	0	0	8
d__Bacteriap__Proteobacteriac__Alphaproteobacteriao__Caulobacteralesf__Hyphomonadaceaeg__SWB02	0	0	0	0	0	0	0	0	13	10	0	3
d__Bacteriap__Actinobacteriotac__Actinobacteriao__Micrococcalesf__Micrococcaceae__	0	0	0	0	0	0	0	0	4	0	0	0
d__Bacteriap__Verrucomicrobiotac__Verrucomicrobiaeo__Chthoniobacteralesf__Chthoniobacteraceaeg__Candidatus_Udaeobacter	18	4	14	10	0	65	9	0	9	0	0	20
d__Bacteriap__Firmicutesc__Clostridiao__Clostridialesf__Clostridiaceaeg__Clostridium_sensu_stricto_12	179	243	447	198	239	819	369	215	687	439	443	529
d__Bacteriap__Proteobacteriac__Alphaproteobacteriao__Rhodospirillalesf__unculturedg__uncultured	27	39	65	18	23	107	11	49	51	84	70	37
d__Bacteriap__Firmicutesc__Desulfotomaculiao__Desulfotomaculalesf__Desulfotomaculalesg__Desulfofarcimen	0	0	21	6	0	11	0	6	5	10	0	4
d__Bacteriap__Bacteroidotac__Bacteroidiao__Bacteroidalesf__Prolixibacteraceaeg__BSV13	21	0	3	8	12	8	9	7	8	0	21	13
d__Bacteriap__Firmicutesc__Clostridiao__Clostridialesf__Clostridiaceaeg__Clostridium_sensu_stricto_5	15	0	0	0	0	40	0	0	60	36	0	0
d__Bacteriap__Proteobacteriac__Alphaproteobacteriao__Sphingomonadalesf__Sphingomonadaceaeg__Novosphingobium	0	59	67	0	17	65	49	0	130	216	53	32
d__Bacteriap__Proteobacteriac__Alphaproteobacteriao__Rhizobialesf__Rhizobiaceaeg__Allorhizobium-Neorhizobium-Pararhizobium-Rhizobium	0	143	165	76	89	200	17	0	79	54	9	3
d__Bacteriap__Firmicutesc__Clostridiao__Peptostreptococcales-Tissierellalesf__Peptostreptococcaceaeg__Terrisporobacter	0	0	2	0	0	0	0	5	0	0	0	5
d__Bacteriap__Proteobacteriac__Alphaproteobacteriao__Rhizobialesf__Xanthobacteraceaeg__Bradyrhizobium	5	5	13	43	11	124	10	51	38	60	47	30

Bacteria	R1__ ASR 5i	R2__ ASR 5i	R3__ ASR 5i	R4__ ASR 5i	R5__ ASR 5i	R6__ ASR 5i	R1 _W T	R2 _W T	R3 _W T	R4 _W T	R5 _W T	R6 _W T
d__Archaeap__Euryarchaeotac__Methanobacteriao__Methanobacterialesf__Methanobacteriaceaeg__Methanobacterium	0	0	7	0	6	26	11	3	30	7	19	3
d__Bacteriap__Chloroflexic__Anaerolineaeo__SBR1031____	0	0	0	0	0	0	0	0	0	3	0	3
d__Bacteriap__Firmicutesc__Negativicuteso__Veillonellales-Selenomonadalesf__Sporomusaceae__	5	6	0	0	0	28	4	0	5	0	24	5
d__Bacteriap__MBNT15c__MBNT15o__MBNT15f__MBNT15g__MBNT15	0	0	0	0	0	3	2	3	0	0	0	0
d__Bacteriap__Proteobacteriac__Gammaproteobacteriao__Burkholderialesf__Rhodocyclaceaeg__Azospira	11	24	29	12	12	32	0	0	0	0	14	0
d__Bacteriap__Firmicutesc__Clostridiao__Clostridialesf__unculturedg__uncultured	13	15	19	15	18	21	18	2	40	0	17	0
d__Bacteriap__Campilobacterotac__Campylobacteriao__Campylobacteralesf__Sulfurimonadaceaeg__Sulfuricurvum	0	0	0	21	0	0	15	4	8	16	31	0
d__Bacteriap__Firmicutesc__Clostridiao__Oscillospiralesf__Oscillospiraceaeg__Sporobacter	55	99	72	83	53	159	73	67	170	68	116	135
d__Bacteriap__Patescibacteriac__Saccharimonadiao__Saccharimonadales____	92	40	160	7	164	374	54	43	64	59	121	39
d__Bacteriap__Proteobacteriac__Gammaproteobacteriao__Xanthomonadalesf__Rhodanobacteraceaeg__Dyella	73	238	145	124	75	269	173	93	131	166	200	250
d__Bacteriap__Proteobacteriac__Alphaproteobacteriao__Acetobacteralesf__Acetobacteraceaeg__Acidocella	0	0	15	0	7	22	0	0	0	0	14	11
d__Bacteriap__FCPU426c__FCPU426o__FCPU426f__FCPU426g__FCPU426	0	6	3	0	2	30	14	27	4	43	6	22
d__Bacteriap__Bacteroidotac__Ignavibacteriao__Ignavibacterialesf__BSV40g__BSV40	30	30	17	13	33	43	15	22	64	22	62	62
d__Bacteriap__Planctomycetotac__Planctomyceteso__Isosphaeralesf__Isosphaeraceaeg__Tundrisphaera	0	0	0	0	5	4	3	0	0	0	0	0
d__Bacteriap__Proteobacteriac__Alphaproteobacteriao__Rhodospirillalesf__Magnetospirillaceaeg__Magnetospirillum	0	134	18	164	35	5	87	565	462	363	277	766
d__Bacteriap__Proteobacteriac__Gammaproteobacteriao__Methylococcalesf__Methylococcaceaeg__Methylocaldum	0	0	0	0	0	0	0	0	0	5	0	0
d__Bacteriap__Dependentiaec__Babeliaeo__Babelialesf__Vermiphilaceaeg__Vermiphilaceae	6	7	4	0	6	0	0	4	4	4	10	5
d__Bacteriap__Firmicutesc__Clostridiao__Oscillospiralesf__Ruminococcaceaeg__Caproiciproducens	8	29	9	4	9	24	8	12	74	4	7	6
d__Bacteriap__Firmicutesc__Desulfitobacteriiao__Desulfitobacterialesf__Heliobacteriaceaeg__uncultured	2	0	3	0	0	0	0	0	0	0	0	7
d__Bacteriap__Sumerlaeotac__Sumerlaeiao__Sumerlaealesf__Sumerlaeaceaeg__Sumerlaea	8	3	9	0	6	0	0	0	0	3	3	9
d__Bacteriap__Cyanobacteriac__Sericytochromatiao__Sericytochromatiaf__Sericytochromatiag__Sericytochromatia	2	0	3	0	7	16	4	13	2	5	2	3

Bacteria	R1__ ASR 5i	R2__ ASR 5i	R3__ ASR 5i	R4__ ASR 5i	R5__ ASR 5i	R6__ ASR 5i	R1 _W T	R2 _W T	R3 _W T	R4 _W T	R5 _W T	R6 _W T
d__Bacteriap__Desulfobacterotac__Desulfomoniliao__Desulfomonilalesf__Desulfomonilaceaeg__Desulfomonile	0	3	11	8	0	18	5	0	0	6	2	4
d__Bacteriap__Proteobacteriac__Alphaproteobacteriao__Reyranellalesf__Reyranellaceaeg__uncultured	0	0	0	0	0	0	0	0	5	0	0	0
d__Bacteriap__Desulfobacterotac__Syntrophobacteriao__Syntrophobacteralesf__unculturedg__uncultured	4	0	0	0	0	3	3	0	0	2	0	0
d__Bacteriap__Patescibacteriac__Parcubacteriao__Parcubacteriaf__Parcubacteriag__Parcubacteria	39	15	40	9	37	122	18	58	26	83	62	46
d__Bacteriap__Firmicutesc__Clostridiao__Lachnospiralesf__Lachnospiraceaeg__Epulopiscium	0	0	10	0	0	0	0	0	0	0	5	0
d__Bacteriap__Desulfobacterotac__Desulfobulbiao__Desulfobulbalesf__Desulfobulbaceaeg__Desulfobulbus	32	67	41	26	32	66	12	18	21	0	37	11
d__Bacteriap__Firmicutesc__Desulfotomaculiao__Desulfotomaculalesf__Desulfotomaculalesg__Desulfohalotomaculum	0	0	0	0	0	0	0	0	0	0	2	0
d__Bacteriap__Actinobacteriotac__Actinobacteriao__Catenulisporalesf__Catenulisporaceaeg__Catenulispora	0	7	0	7	0	12	12	7	0	0	9	23
d__Bacteriap__Verrucomicrobiotac__Verrucomicrobiaeo__Chthoniobacteralesf__Chthoniobacteraceae__	8	13	30	10	16	47	11	12	21	23	0	11
d__Bacteriap__Actinobacteriotac__Actinobacteriao__Corynebacterialesf__Nocardiaceaeg__Nocardia	0	4	9	10	19	14	11	0	3	8	7	11
d__Bacteriap__Myxococcotac__Myxococciao__Myxococcalesf__Myxococcaceaeg__P3OB-42	123	344	257	141	349	396	179	124	350	183	335	379
d__Bacteriap__Verrucomicrobiotac__Chlamydiaeo__Chlamydiales____	0	3	0	5	0	2	0	0	4	3	0	0
d__Bacteriap__Firmicutesc__Clostridiao__Clostridiaf__Gracilibacteraceae__	0	0	2	0	2	0	38	2	9	5	8	7
d__Bacteriap__Verrucomicrobiotac__Kiritimatiellaeo__WCHB1-41f__WCHB1-41g__WCHB1-41	2	0	0	0	0	0	0	8	0	0	2	0
d__Bacteriap__Desulfobacterotac__Desulfuromonadiao__Geobacteralesf__Geobacteraceaeg__uncultured	0	0	0	34	0	19	0	47	0	0	3	0
d__Bacteriap__Proteobacteriac__Gammaproteobacteriao__Burkholderialesf__Alcaligenaceaeg__Bordetella	9	41	29	21	16	14	6	0	0	10	12	20
d__Bacteriap__Proteobacteriac__Alphaproteobacteriao__Rickettsialesf__Rickettsiaceaeg__uncultured	0	0	0	0	0	2	3	0	0	0	0	0
d__Bacteriap__Cyanobacteriac__Vampirivibrioniao__Obscuribacteralesf__Obscuribacteraceaeg__Obscuribacteraceae	3	0	6	0	0	8	0	0	8	3	8	10
d__Bacteriap__Patescibacteria________	19	11	21	15	10	120	23	32	16	56	26	28
d__Bacteriap__Acidobacteriotac__Blastocatelliao__Pyrinomonadalesf__Pyrinomonadaceaeg__RB41	0	0	0	0	0	0	3	0	0	0	0	0

Bacteria	R1__ ASR 5i	R2__ ASR 5i	R3__ ASR 5i	R4__ ASR 5i	R5__ ASR 5i	R6__ ASR 5i	R1 _W T	R2 _W T	R3 _W T	R4 _W T	R5 _W T	R6 _W T
d__Bacteriap__Planctomycetotac__Planctomyceteso__Gemmatalesf__Gemmataceaeg__uncultured	4	0	3	0	8	9	4	0	5	3	6	4
d__Bacteriap__Acidobacteriotac__Acidobacteriaeo__AKIW659f__AKIW659g__AKIW659	0	0	0	0	0	0	0	10	0	0	0	0
d__Bacteriap__Patescibacteriac__Parcubacteriao__Candidatus_Kaiserbacteriaf__Candidatus_Kaiserbacteriag__Candidatus_Kaiserbacteria	10	32	21	10	8	27	25	13	0	33	9	31
d__Bacteriap__Bacteroidotac__Bacteroidiao__Chitinophagalesf__Chitinophagaceaeg__Sediminibacterium	5	20	0	0	6	96	5	0	0	19	4	3
d__Bacteriap__Chloroflexic__Anaerolineaeo__Anaerolinealesf__Anaerolineaceaeg__RBG-16-58-14	2	0	0	0	0	8	0	0	6	5	6	0
d__Bacteriap__Bacteroidotac__Kryptoniao__Kryptonialesf__Kryptoniaceaeg__Kryptoniaceae	16	14	21	18	0	47	0	20	122	77	42	78
d__Bacteriap__Proteobacteriac__Alphaproteobacteriao__Rhizobialesf__Rhizobiales_Incertae_Sedis__	0	0	0	0	2	23	0	0	0	0	2	0
d__Bacteriap__Acidobacteriotac__Acidobacteriaeo__Acidobacterialesf__Acidobacteriaceae_(Subgroup_1)g__Granulicella	0	84	26	0	23	2	0	0	65	23	48	60
d__Bacteriap__Patescibacteriac__Parcubacteriao__Candidatus_Yanofskybacteriaf__Candidatus_Yanofskybacteriag__Candidatus_Yanofskybacteria	7	19	19	3	4	19	4	10	3	6	8	5
d__Bacteriap__Firmicutesc__Clostridiao__Clostridialesf__Clostridiaceaeg__Clostridium_sensu_stricto_9	0	0	0	0	18	0	73	0	64	0	65	0
d__Bacteriap__Firmicutesc__Clostridiao__Clostridialesf__Clostridiaceaeg__Clostridium_sensu_stricto_11	58	3	113	217	86	56	29	0	89	65	100	71
d__Bacteriap__Actinobacteriotac__Actinobacteriao__Micrococcalesf__Intrasporangiaceae__	0	0	0	0	0	0	11	0	0	3	2	4
d__Bacteriap__Firmicutesc__Bacillio__Paenibacillalesf__Paenibacillaceae__	0	2	0	0	0	7	0	0	0	0	3	2
d__Archaeap__Halobacterotac__Methanosarciniao__Methanosarcinialesf__Methanosarcinaceaeg__Methanosarcina	19	5	17	0	33	22	13	0	27	7	41	13
d__Bacteriap__Firmicutesc__Clostridiao__Peptostreptococcales-Tissierellalesf__Peptostreptococcaceae__	2	0	3	0	3	15	4	0	2	9	4	2
d__Bacteriap__Proteobacteriac__Alphaproteobacteriao__Rhizobialesf__Hyphomicrobiaceaeg__Hyphomicrobium	0	0	0	5	0	35	27	28	5	0	16	0
d__Bacteriap__Firmicutesc__Bacillio__Lactobacillalesf__Streptococcaceaeg__Streptococcus	0	0	0	0	0	0	3	0	0	0	0	0
d__Bacteriap__Patescibacteriac__Parcubacteriao__Candidatus_Jorgensenbacteriaf__Candidatus_Jorgensenbacteriag__Candidatus_Jorgensenbacteria	16	92	46	26	23	120	39	98	21	72	38	67
d__Bacteriap__Proteobacteriac__Gammaproteobacteriao__Enterobacteralesf__Yersiniaceaeg__Serratia	0	0	0	0	0	0	0	0	0	0	6	0
d__Bacteriap__Proteobacteriac__Alphaproteobacteriao__Acetobacteralesf__Acetobacteraceaeg__uncultured	53	0	83	8	65	113	59	34	40	33	224	97

Bacteria	R1__ ASR 5i	R2__ ASR 5i	R3__ ASR 5i	R4__ ASR 5i	R5__ ASR 5i	R6__ ASR 5i	R1 _W T	R2 _W T	R3 _W T	R4 _W T	R5 _W T	R6 _W T
d__Bacteriap__Actinobacteriotac__Thermoleophiliao__Gaiellales____	0	0	0	0	0	8	0	0	3	0	0	0
d__Bacteriap__Proteobacteriac__Alphaproteobacteriao__Rhizobialesf__Devosiaceaeg__Devosia	15	44	33	40	38	84	48	56	49	36	74	58
d__Bacteriap__Gemmatimonadota________	0	0	6	0	3	12	2	0	0	5	0	2
d__Bacteriap__Proteobacteriac__Gammaproteobacteriao__Xanthomonadalesf__Rhodanobacteraceaeg__Luteibacter	0	0	0	0	0	0	0	0	0	0	2	0
d__Archaeap__Crenarchaeotac__Nitrososphaeriao__Nitrosotalealesf__Nitrosotaleaceaeg__Candidatus_Nitrosotalea	0	0	0	0	0	9	0	4	0	7	7	11
Unassigned__________	3	3	0	0	0	0	6	4	0	0	0	7
d__Bacteriap__Firmicutesc__Clostridiao__Peptostreptococcales-Tissierellalesf__Anaerovoraceaceae__	0	5	0	2	4	9	0	0	4	0	3	11
d__Bacteriap__Chloroflexic__Anaerolineaeo__SBR1031f__A4bg__A4b	0	8	0	0	0	15	0	2	0	21	0	3
d__Bacteriap__Chloroflexic__Dehalococcoidiao__Dehalococcoidalesf__unculturedg__uncultured	0	3	0	0	3	0	0	0	0	5	4	4
d__Bacteriap__Proteobacteriac__Alphaproteobacteriao__Rhizobialesf__Devosiaceae__	0	0	0	0	0	24	0	0	0	0	0	6
d__Bacteriap__Proteobacteriac__Alphaproteobacteriao__Rhodospirillales____	0	5	0	0	0	0	0	20	0	12	0	5
d__Bacteriap__Proteobacteriac__Alphaproteobacteriao__Elsterales____	2	33	36	40	21	105	27	35	0	55	95	80
d__Bacteriap__Myxococcotac__Polyangiao__Polyangialesf__BIrii41g__BIrii41	6	12	7	4	10	34	0	15	5	4	20	21
d__Bacteriap__Actinobacteriotac__Actinobacteriao__Micrococcalesf__Cellulomonadaceaeg__Cellulomonas	0	99	42	56	45	106	70	0	62	42	78	58
d__Bacteriap__Firmicutesc__Syntrophomonadiao__Syntrophomonadalesf__Syntrophomonadaceaeg__Syntrophomonas	6	0	0	0	19	18	2	0	4	0	0	12
d__Bacteriap__Proteobacteriac__Gammaproteobacteriao__Burkholderialesf__Oxalobacteraceae__	21	128	132	34	104	175	50	102	56	243	34	74
d__Bacteriap__Proteobacteriac__Alphaproteobacteriao__Paracaedibacteralesf__Paracaedibacteraceaeg__Candidatus_Finniella	0	0	0	0	0	0	0	0	0	0	0	6
d__Archaeap__Thermoplasmatotac__Thermoplasmatao__Methanomassiliicoccalesf__unculturedg__uncultured	0	0	0	0	0	0	0	5	0	0	0	0
d__Bacteriap__Firmicutesc__Desulfitobacteriiao__Desulfitobacteriales____	0	0	0	0	3	3	0	0	0	0	2	6
d__Bacteriap__Verrucomicrobiotac__Chlamydiaeo__Chlamydialesf__Parachlamydiaceaeg__Neochlamydia	0	0	2	0	9	7	0	0	2	3	4	0
d__Bacteriap__Firmicutesc__Clostridiao__Oscillospiralesf__[Eubacterium]_coprostanoligenes_groupg__[Eubacterium]_coprostanoligenes_group	4	0	4	0	6	0	0	0	10	0	0	0

Bacteria	R1__ ASR 5i	R2__ ASR 5i	R3__ ASR 5i	R4__ ASR 5i	R5__ ASR 5i	R6__ ASR 5i	R1 _W T	R2 _W T	R3 _W T	R4 _W T	R5 _W T	R6 _W T
d__Bacteriap__Proteobacteriac__Gammaproteobacteriao__Steroidobacteralesf__Steroidobacteraceaeg__Steroidobacter	9	12	7	12	13	39	13	26	5	25	22	57
d__Bacteriap__Bacteroidotac__Bacteroidiao__Cytophagalesf__Cytophagaceaeg__Cytophaga	0	0	0	0	0	0	0	0	3	15	0	0
d__Bacteriap__Actinobacteriotac__Actinobacteriao__Micrococcalesf__Micrococcaceaeg__Sinomonas	0	0	4	0	0	0	8	0	0	0	4	5
d__Bacteriap__Armatimonadotac__DG-56o__DG-56f__DG-56g__DG-56	0	0	0	0	0	0	0	0	2	0	0	0
d__Bacteriap__Firmicutesc__Clostridiao__Clostridia_UCG-014f__Clostridia_UCG-014g__Clostridia_UCG-014	0	3	8	0	0	4	6	9	5	28	0	0
d__Bacteriap__Proteobacteriac__Gammaproteobacteriao__Burkholderialesf__Chromobacteriaceaeg__Vogesella	0	16	0	13	7	36	0	0	0	0	15	0
d__Bacteriap__Verrucomicrobiotac__Chlamydiaeo__Chlamydialesf__Parachlamydiaceae__	0	0	0	0	3	0	2	0	0	0	4	0
d__Bacteriap__Armatimonadotac__unculturedo__unculturedf__unculturedg__uncultured	0	0	2	5	0	5	0	2	0	0	2	0
d__Bacteriap__Actinobacteriotac__Acidimicrobiia______	0	0	0	18	8	40	0	3	0	17	0	0
d__Bacteriap__Chloroflexic__Ktedonobacteriao__Ktedonobacteralesf__Ktedonobacteraceaeg__JG30a-KF-32	0	2	0	0	0	0	0	0	3	10	0	0
d__Bacteriap__Proteobacteriac__Gammaproteobacteriao__Burkholderialesf__Burkholderiaceaeg__Lautropia	0	0	0	0	0	0	3	0	0	0	0	0
d__Bacteriap__Firmicutesc__D8A-2o__D8A-2f__D8A-2g__D8A-2	0	0	0	0	0	3	0	2	0	2	0	0
d__Bacteriap__Actinobacteriotac__Actinobacteriao__Frankialesf__Sporichthyaceaeg__Longivirga	15	10	31	7	32	60	0	11	31	11	0	20
d__Bacteriap__Acidobacteriotac__Acidobacteriaeo__Acidobacterialesf__Acidobacteriaceae_(Subgroup_1)g__Occallatibacter	139	75	82	132	89	0	0	3	72	0	47	189
d__Bacteriap__Firmicutesc__Clostridiao__Clostridiaf__Hungateiclostridiaceaeg__Ruminiclostridium	156	260	253	273	312	367	209	263	625	404	588	525
d__Bacteriap__Bacteroidotac__Bacteroidiao__Sphingobacterialesf__Lentimicrobiaceae__	0	0	0	0	19	10	0	0	0	0	12	8
d__Bacteriap__Patescibacteriac__ABY1o__Candidatus_Kerfeldbacteriaf__Candidatus_Kerfeldbacteriag__Candidatus_Kerfeldbacteria	3	0	0	0	0	7	2	0	0	0	0	0
d__Bacteriap__Acidobacteriotac__Acidobacteriaeo__Subgroup_12f__Subgroup_12g__Subgroup_12	0	2	0	0	0	0	0	0	0	2	0	0
d__Bacteriap__Proteobacteriac__Gammaproteobacteriao__Burkholderialesf__Chromobacteriaceaeg__Paludibacterium	0	12	3	0	0	0	0	0	0	17	0	0
d__Bacteriap__WPS-2c__WPS-2o__WPS-2f__WPS-2g__WPS-2	20	8	34	10	3	51	46	8	13	26	53	24
d__Bacteriap__Bacteroidotac__Bacteroidiao__Chitinophagalesf__Chitinophagaceaeg__Dinghuibacter	2	4	5	0	0	0	0	12	0	21	0	23

Bacteria	R1__ ASR 5i	R2__ ASR 5i	R3__ ASR 5i	R4__ ASR 5i	R5__ ASR 5i	R6__ ASR 5i	R1 _W T	R2 _W T	R3 _W T	R4 _W T	R5 _W T	R6 _W T
d__Bacteriap__Actinobacteriotac__Actinobacteriao__Frankialesf__Frankiaceaeg__Jatrophihabitans	9	0	0	0	0	49	0	0	0	27	0	0
d__Bacteriap__Proteobacteriac__Alphaproteobacteriao__Reyranellalesf__Reyranellaceaeg__Reyranella	4	6	0	4	10	34	5	9	15	8	25	22
d__Bacteriap__Bacteroidotac__Bacteroidiao__Chitinophagalesf__37-13g__37-13	4	0	0	0	6	9	0	0	0	0	2	2
d__Archaeap__Halobacterotac__Halobacteriao__Halobacteralesf__Haloadaptaceaeg__Haladaptatus	0	0	0	0	0	0	0	0	0	0	0	3
d__Bacteriap__Firmicutesc__Desulfotomaculiao__Desulfotomaculalesf__Desulfotomaculalesg__Desulfurispora	0	0	0	0	0	0	0	0	0	0	0	12
d__Bacteriap__Bacteroidotac__Bacteroidiao__Bacteroidalesf__Paludibacteraceaeg__Paludibacter	9	31	47	8	18	6	34	4	48	0	22	20
d__Bacteriap__Bacteroidotac__Bacteroidiao__Chitinophagalesf__Chitinophagaceaeg__Puia	51	57	42	53	27	108	86	69	80	145	201	141
d__Bacteriap__Proteobacteriac__Alphaproteobacteriao__Acetobacteralesf__Acetobacteraceaeg__Rhodovastum	0	9	20	0	0	19	21	0	0	0	22	11
d__Bacteriap__Actinobacteriotac__Actinobacteriao__Pseudonocardialesf__Pseudonocardiaceae__	2	0	0	2	0	0	0	0	0	0	2	0
d__Bacteriap__Firmicutesc__Clostridiao__Lachnospiralesf__Lachnospiraceaeg__Anaerocolumna	0	0	0	0	0	0	0	0	0	0	0	9
d__Bacteriap__Patescibacteriac__Parcubacteria______	22	8	51	16	21	83	22	37	30	81	64	25
d__Bacteriap__Proteobacteriac__Gammaproteobacteriao__Burkholderialesf__Methylophilaceaeg__Methylophilus	0	21	39	0	6	45	26	4	48	43	32	9
d__Bacteriap__Proteobacteriac__Alphaproteobacteriao__unculturedf__unculturedg__uncultured	28	7	27	18	8	84	24	35	20	25	67	57
d__Bacteriap__Chloroflexic__Anaerolineaeo__SJA-15f__SJA-15g__SJA-15	109	27	108	41	112	575	104	31	190	145	322	70
d__Bacteriap__Verrucomicrobiotac__Verrucomicrobiae______	0	0	0	0	0	4	0	3	0	0	0	0
d__Bacteriap__Firmicutesc__Clostridiao__Oscillospiralesf__UCG-010g__UCG-010	0	4	0	0	0	0	0	0	0	0	15	9
d__Bacteriap__Firmicutesc__Negativicuteso__Veillonellales-Selenomonadalesf__unculturedg__uncultured	0	4	0	0	6	15	4	0	5	3	7	0
d__Bacteriap__Proteobacteriac__Alphaproteobacteriao__Rhizobialesf__Rhizobiaceaeg__Phyllobacterium	24	0	0	0	0	0	0	0	0	0	0	21
d__Bacteriap__Myxococcotac__Myxococciao__Myxococcalesf__Myxococcaceaeg__uncultured	65	57	90	82	208	261	47	13	45	36	64	17
d__Bacteriap__Myxococcotac__bacteriap25o__bacteriap25f__bacteriap25g__bacteriap25	0	0	0	2	0	13	4	6	7	2	0	0
d__Bacteriap__Proteobacteriac__Alphaproteobacteriao__Dongialesf__Dongiaceaeg__Dongia	0	19	23	22	20	59	31	26	53	40	73	8

Bacteria	R1__ ASR 5i	R2__ ASR 5i	R3__ ASR 5i	R4__ ASR 5i	R5__ ASR 5i	R6__ ASR 5i	R1 _W T	R2 _W T	R3 _W T	R4 _W T	R5 _W T	R6 _W T
d__Bacteriap__Hydrogenedentesc__Hydrogenedentiao__Hydrogenedentialesf__Hydrogenedensaceaeg__Hydrogenedensaceae	2	3	8	0	0	9	0	4	0	0	3	3
d__Bacteriap__Proteobacteriac__Alphaproteobacteriao__Rhizobialesf__Methyloligellaceaeg__uncultured	0	8	18	0	0	0	10	0	0	0	7	16
d__Bacteriap__Patescibacteriac__Parcubacteriao__Candidatus_Nomurabacteriaf__Candidatus_Nomurabacteriag__Candidatus_Nomurabacteria	0	0	0	0	0	3	0	10	0	0	5	0
d__Bacteriap__Bacteroidotac__Bacteroidiao__Bacteroidalesf__Prolixibacteraceaeg__WCHB1-32	20	36	11	21	36	34	14	50	26	80	29	70
d__Bacteriap__Proteobacteriac__Alphaproteobacteriao__Azospirillalesf__Azospirillaceaeg__Azospirillum	14	39	21	17	0	22	0	11	0	0	39	9
d__Bacteriap__Proteobacteriac__Alphaproteobacteriao__Rhizobialesf__Hyphomicrobiaceae__	0	0	0	0	0	0	0	0	3	0	0	0
d__Bacteriap__Actinobacteriotac__Actinobacteriao__Streptomycetalesf__Streptomycetaceaeg__Streptomyces	22	28	33	19	18	136	33	20	24	2	41	36
d__Bacteriap__Margulisbacteriac__Margulisbacteriao__Margulisbacteriaf__Margulisbacteriag__Margulisbacteria	0	15	11	16	0	6	0	0	0	0	7	7
d__Bacteriap__Firmicutesc__Clostridiao__Clostridiaf__Hungateiclostridiaceaeg__uncultured	5	6	19	3	0	26	5	3	7	2	31	39
d__Bacteriap__Chloroflexic__Anaerolineaeo__RBG-13-54-9f__RBG-13-54-9g__RBG-13-54-9	11	0	8	0	3	26	4	11	11	3	11	0
d__Bacteriap__Proteobacteriac__Alphaproteobacteriao__Micropepsalesf__Micropepsaceaeg__Micropepsis	0	0	28	0	0	24	12	0	44	15	16	24
d__Bacteriap__Proteobacteriac__Alphaproteobacteriao__Rhizobialesf__Beijerinckiaceaeg__Roseiarcus	131	142	284	113	146	591	205	53	266	275	342	161
d__Bacteriap__Proteobacteriac__Gammaproteobacteriao__Burkholderialesf__Burkholderiaceaeg__uncultured	0	0	0	0	0	0	6	0	0	0	0	0
d__Bacteriap__Chloroflexic__Ktedonobacteriao__Ktedonobacteralesf__Ktedonobacteraceaeg__BacC-u-018	0	0	0	0	0	0	0	7	0	0	0	0
d__Bacteriap__RCP2-54c__RCP2-54o__RCP2-54f__RCP2-54g__RCP2-54	39	19	79	10	9	93	49	19	31	22	82	32
d__Bacteriap__Firmicutesc__Limnochordiao__Limnochordiaf__Limnochordiag__Limnochordia	0	0	0	0	0	0	0	6	0	0	2	0
d__Bacteriap__Proteobacteriac__Gammaproteobacteriao__Burkholderialesf__Methylophilaceae__	0	8	32	5	121	11	0	0	0	14	0	5
d__Bacteriap__Spirochaetotac__Brevinematiao__Brevinematalesf__Brevinemataceaeg__Brevinema	0	0	0	0	2	2	0	0	2	0	0	0
d__Bacteriap__Bacteroidotac__Ignavibacteriao__Ignavibacterialesf__SR-FBR-L83g__SR-FBR-L83	13	22	33	3	8	30	0	23	11	14	47	27

Bacteria	R1__ ASR 5i	R2__ ASR 5i	R3__ ASR 5i	R4__ ASR 5i	R5__ ASR 5i	R6__ ASR 5i	R1 _W T	R2 _W T	R3 _W T	R4 _W T	R5 _W T	R6 _W T
d__Bacteriap__Planctomycetotac__Phycisphaeraeo__Phycisphaeralesf__AKAU3564_sediment_groupg__AKAU3564_sediment_group	0	4	4	0	0	9	0	9	4	0	0	0
d__Bacteriap__Verrucomicrobiotac__Verrucomicrobiaeo__Pedosphaeralesf__Pedosphaeraceaeg__Pedosphaera	0	0	0	5	0	2	0	0	0	0	2	0
d__Bacteriap__Firmicutesc__Clostridiao__Oscillospirales____	0	0	0	3	0	0	0	4	6	0	3	10
d__Bacteriap__Proteobacteriac__Alphaproteobacteriao__Azospirillalesf__Azospirillaceae__	0	32	0	8	8	0	0	7	14	0	0	2
d__Bacteriap__Actinobacteriotac__Actinobacteriao__Streptomycetalesf__Streptomycetaceae___	0	2	0	0	0	47	17	4	10	4	0	0
d__Bacteriap__Proteobacteriac__Gammaproteobacteriao__Burkholderialesf__Oxalobacteraceaeg__uncultured	6	0	0	0	0	0	4	0	3	0	0	0
d__Bacteriap__Bacteroidotac__Bacteroidiao__Flavobacterialesf__Flavobacteriaceae__	0	0	0	0	0	0	0	0	0	0	6	0
d__Bacteriap__Proteobacteriac__Gammaproteobacteriao__Diplorickettsialesf__Diplorickettsiaceae__	0	0	0	0	0	2	0	0	2	2	0	3
d__Bacteriap__Actinobacteriotac__Actinobacteriao__Micrococcales____	3	6	7	3	2	23	0	3	0	9	3	0
d__Bacteriap__Proteobacteriac__Gammaproteobacteriao__Burkholderialesf__A21bg__A21b	8	0	2	0	4	13	0	3	0	0	0	0
d__Bacteriap__Proteobacteriac__Gammaproteobacteriao__Steroidobacteralesf__Steroidobacteraceaeg__uncultured	0	15	14	0	16	39	0	0	0	8	0	0
d__Bacteriap__Proteobacteriac__Alphaproteobacteriao__Azospirillalesf__Inquilinaceaeg__Inquilinus	8	12	16	30	5	35	14	14	16	21	32	19
d__Bacteriap__Bdellovibrionotac__Oligoflexiao__Oligoflexalesf__unculturedg__uncultured	0	4	0	3	6	5	0	0	0	0	12	2
d__Bacteriap__Actinobacteriotac__Actinobacteriao__Micromonosporalesf__Micromonosporaceaeg__Micromonospora	4	0	7	0	0	0	25	0	0	0	12	0
d__Bacteriap__Proteobacteriac__Gammaproteobacteriao__Burkholderialesf__Gallionellaceae__	0	8	12	0	19	0	0	3	0	0	0	43
d__Bacteriap__Proteobacteriac__Alphaproteobacteriao__Rhizobialesf__Rhizobiaceaeg__Mesorhizobium	0	0	0	0	0	26	0	16	0	0	32	0
d__Bacteriap__Actinobacteriotac__Actinobacteriao__Micrococcalesf__Microbacteriaceaeg__Humibacter	0	0	0	0	0	10	0	0	0	0	0	5
d__Bacteriap__Bacteroidotac__Bacteroidiao__Chitinophagalesf__Chitinophagaceaeg__Ferruginibacter	0	2	0	0	4	2	0	0	0	0	0	4
d__Bacteriap__Firmicutesc__Desulfotomaculiao__Desulfotomaculalesf__Desulfotomaculalesg__Pelotomaculum	0	0	8	2	0	12	0	19	0	0	0	14
d__Bacteriap__Proteobacteriac__Alphaproteobacteriao__Caulobacteralesf__Caulobacteraceaeg__Asticcacaulis	9	113	0	16	57	39	22	12	25	56	12	28

Bacteria	R1__ ASR 5i	R2__ ASR 5i	R3__ ASR 5i	R4__ ASR 5i	R5__ ASR 5i	R6__ ASR 5i	R1 _W T	R2 _W T	R3 _W T	R4 _W T	R5 _W T	R6 _W T
d__Bacteriap__Fibrobacterotac__Fibrobacteriao__Fibrobacteralesf__Fibrobacteraceaeg__uncultured	5	0	12	11	0	16	6	37	0	8	0	3
d__Bacteriap__Patescibacteriac__Parcubacteriao__Candidatus_Moranbacteriaf__Candidatus_Moranbacteriag__Candidatus_Moranbacteria	0	15	5	3	10	50	13	15	11	13	8	16
d__Bacteriap__Chloroflexic__Ktedonobacteria______	9	0	11	0	0	0	0	0	6	0	0	0
d__Bacteriap__Firmicutesc__Clostridiao__Lachnospiralesf__Lachnospiraceaeg__Cellulosilyticum	0	0	0	0	0	11	16	0	7	5	19	0
d__Bacteriap__Firmicutesc__Desulfotomaculiao__Desulfotomaculalesf__Desulfotomaculalesg__Desulfotomaculum	0	0	0	0	0	0	0	0	0	2	0	2
d__Bacteriap__Proteobacteriac__Gammaproteobacteriao__Legionellalesf__Legionellaceaeg__Legionella	0	6	9	0	0	5	7	0	0	8	11	0
d__Bacteriap__Chloroflexic__Anaerolineaeo__SBR1031f__SBR1031g__SBR1031	0	11	3	5	0	15	3	23	4	8	6	19
d__Bacteriap__Bacteroidotac__Bacteroidiao__Bacteroidalesf__Bacteroidetes_BD2-2g__Bacteroidetes_BD2-2	2	0	3	0	3	0	0	0	0	0	0	3
d__Bacteriap__Chloroflexic__Ktedonobacteriao__C0119f__C0119g__C0119	0	0	3	0	7	13	6	0	2	7	3	3
d__Bacteriap__Planctomycetotac__Planctomyceteso__Isosphaeralesf__Isosphaeraceaeg__uncultured	0	0	0	0	0	0	0	0	0	0	0	3
d__Bacteriap__Proteobacteriac__Alphaproteobacteriao__Rhizobialesf__KF-JG30-B3g__KF-JG30-B3	0	0	0	4	2	0	0	0	16	19	2	8
d__Bacteriap__Desulfobacterotac__unculturedo__unculturedf__unculturedg__uncultured	0	0	4	0	0	5	4	3	0	0	0	14
d__Bacteriap__WS4c__WS4o__WS4f__WS4g__WS4	0	7	7	0	0	7	0	4	0	0	8	8
d__Bacteriap__Bacteroidotac__Bacteroidiao__Chitinophagalesf__Chitinophagaceaeg__Filimonas	0	0	0	0	0	0	0	0	0	0	6	0
d__Bacteriap__Proteobacteriac__Alphaproteobacteriao__Rhodospirillalesf__Magnetospirillaceaeg__uncultured	0	16	28	0	0	0	0	0	0	0	0	5
d__Bacteriap__Firmicutesc__Clostridiao__Peptostreptococcales-Tissierellalesf__Sedimentibacteraceaeg__Sedimentibacter	0	0	0	0	0	6	0	0	0	3	0	0
d__Bacteriap__Bacteroidota________	0	10	0	10	7	41	15	6	0	14	17	37
d__Bacteriap__Proteobacteriac__Gammaproteobacteriao__Pseudomonadalesf__Moraxellaceaeg__uncultured	0	0	2	10	0	14	0	0	12	0	16	8
d__Bacteriap__Firmicutesc__Clostridiao__Clostridialesf__Caloramatoraceaeg__Fervidicella	0	0	0	0	0	0	7	0	0	0	0	0
d__Bacteriap__Chloroflexic__Ktedonobacteriao__Ktedonobacteralesf__Ktedonobacteraceaeg__HSB_OF53-F07	2	0	2	4	0	4	0	7	2	0	0	0
d__Bacteriap__Proteobacteriac__Gammaproteobacteriao__Burkholderialesf__Neisseriaceaeg__uncultured	2	0	0	0	0	0	0	0	2	2	0	0

Bacteria	R1__ASR5i	R2__ASR5i	R3__ASR5i	R4__ASR5i	R5__ASR5i	R6__ASR5i	R1_WT	R2_WT	R3_WT	R4_WT	R5_WT	R6_WT
d__Bacteriap__Planctomycetotac__Planctomyceteso__Isosphaeralesf__Isosphaeraceae__	8	4	7	0	0	36	21	0	0	7	3	7
d__Bacteriap__Proteobacteriac__Alphaproteobacteriao__Acetobacteralesf__Acetobacteraceaeg__Rhodopila	8	0	4	0	0	8	0	0	22	0	0	0
d__Bacteriap__Proteobacteriac__Gammaproteobacteriao__Burkholderialesf__Nitrosomonadaceaeg__GOUTA6	0	0	0	0	0	22	0	0	0	31	0	17
d__Bacteriap__Acidobacteriotac__Subgroup_18o__Subgroup_18f__Subgroup_18g__Subgroup_18	0	8	7	0	18	18	0	0	3	6	20	18
d__Bacteriap__Firmicutesc__Clostridiao__Peptococcalesf__Peptococcaceaeg__uncultured	0	0	0	0	0	0	0	0	2	0	0	0
d__Bacteriap__Proteobacteriac__Alphaproteobacteriao__Rhizobialesf__Beijerinckiaceaeg__alphaI_cluster	0	0	49	0	0	43	0	0	23	0	30	6
d__Bacteriap__Proteobacteriac__Alphaproteobacteriao__Rhizobialesf__Rhizobiales_Incertae_Sedisg__Phreatobacter	0	0	0	6	0	0	0	0	0	0	0	11
d__Bacteriap__Spirochaetotac__Spirochaetiao__Spirochaetalesf__Spirochaetaceaeg__Treponema	0	0	0	0	0	0	0	0	5	0	0	19
d__Bacteriap__Acidobacteriotac__Acidobacteriaeo__Acidobacterialesf__Acidobacteriaceae_(Subgroup_1)g__Acidipila	0	16	0	37	0	14	0	9	0	0	0	0
d__Bacteriap__Proteobacteriac__Gammaproteobacteriao__Burkholderialesf__Comamonadaceaeg__Variovorax	0	0	0	0	0	0	0	0	0	0	13	0
d__Archaeap__Halobacterotac__Methanocelliao__Methanocellalesf__Methanocellaceaeg__Rice_Cluster_I	24	0	16	9	39	29	14	0	48	8	57	0
d__Bacteriap__Bacteroidotac__Bacteroidiao__Bacteroidalesf__Paludibacteraceae__	55	15	0	0	57	22	46	15	55	0	105	40
d__Bacteriap__Verrucomicrobiotac__Verrucomicrobiaeo__S-BQ2-57_soil_groupf__S-BQ2-57_soil_groupg__S-BQ2-57_soil_group	0	0	0	0	0	0	0	0	0	4	0	0
d__Bacteriap__Proteobacteriac__Gammaproteobacteriao__Xanthomonadalesf__Rhodanobacteraceaeg__Metallibacterium	0	0	0	0	0	0	0	0	2	0	0	0
d__Bacteriap__Desulfobacterotac__Desulfuromonadiao__Geobacteralesf__Geobacteraceaeg__Geobacteraceae	0	0	0	0	0	0	0	0	0	0	4	0
d__Bacteriap__Firmicutesc__unculturedo__unculturedf__unculturedg__uncultured	0	0	0	0	2	0	0	0	2	0	0	0
d__Bacteriap__Proteobacteriac__Gammaproteobacteriao__Burkholderialesf__Chromobacteriaceae__	3	49	10	0	0	28	0	0	0	0	6	7
d__Bacteriap__Actinobacteriotac__Acidimicrobiiao__Microtrichales____	11	4	5	0	0	0	0	0	7	0	0	5
d__Bacteriap__Actinobacteriotac__Thermoleophiliao__Solirubrobacteralesf__67-14g__67-14	4	7	9	5	7	12	19	0	0	0	27	0
d__Bacteriap__Chloroflexic__Dehalococcoidiao__S085f__S085g__S085	3	0	0	0	0	2	2	3	0	0	0	5
d__Bacteriap__Firmicutesc__Clostridiao__Clostridialesf__Clostridiaceaeg__Clostridium_sensu_stricto_18	0	0	0	0	0	0	0	0	0	4	0	0

Bacteria	R1__ ASR 5i	R2__ ASR 5i	R3__ ASR 5i	R4__ ASR 5i	R5__ ASR 5i	R6__ ASR 5i	R1 _W T	R2 _W T	R3 _W T	R4 _W T	R5 _W T	R6 _W T
d__Bacteriap__Gemmatimonadotac__Gemmatimonadeteso__Gemmatimonadalesf__Gemmatimonadaceaeg__Roseisolibacter	0	0	0	0	0	0	0	0	0	3	0	0
d__Bacteriap__Firmicutesc__Clostridiao__Thermincolalesf__Thermincolaceaeg__Thermincola	0	3	3	0	6	17	5	7	6	12	9	0
d__Bacteriap__Chloroflexic__Anaerolineaeo__Anaerolinealesf__Anaerolineaceaeg__Leptolinea	0	0	0	0	10	0	11	7	0	0	17	0
d__Bacteriap__Proteobacteriac__Gammaproteobacteriao__Burkholderialesf__Oxalobacteraceaeg__Herbaspirillum	0	0	0	0	26	44	33	5	33	0	0	5
d__Bacteriap__Myxococcotac__Polyangiao__Polyangialesf__Sandaracinaceaeg__uncultured	4	0	7	5	0	0	15	2	3	9	17	11
d__Bacteriap__Actinobacteriotac__Actinobacteriao__Streptosporangialesf__Thermomonosporaceaeg__Actinomadura	0	0	0	0	0	0	0	3	0	0	0	0
d__Bacteriap__Bacteroidotac__Bacteroidiao__Bacteroidalesf__SB-5g__SB-5	3	0	0	0	3	39	9	0	0	0	0	0
d__Bacteriap__Chloroflexic__Ktedonobacteriao__Ktedonobacteralesf__Ktedonobacteraceaeg__Ktedonobacter	9	6	5	17	0	7	13	4	20	5	0	9
d__Bacteriap__Desulfobacterotac__Desulfobacteriao__Desulfatiglandalesf__Desulfatiglandaceaeg__Desulfatiglans	5	7	0	3	5	0	0	4	0	11	6	4
d__Bacteriap__Firmicutesc__Clostridiao__Oscillospiralesf__Ethanoligenenaceaeg__Incertae_Sedis	0	0	10	0	3	5	4	5	14	0	11	8
d__Bacteriap__Actinobacteriotac__Thermoleophiliao__Solirubrobacterales____	0	0	0	0	0	3	7	5	0	0	0	0
d__Bacteriap__Myxococcotac__Polyangiao__Polyangialesf__Polyangiaceae__	28	44	27	23	48	220	10	23	8	3	34	9
d__Bacteriap__Firmicutesc__Bacillio__Paenibacillalesf__Paenibacillaceaeg__Cohnella	0	2	0	0	5	0	0	0	0	0	0	6
d__Bacteriap__Proteobacteriac__Alphaproteobacteriao__Rhodospirillalesf__Rhodospirillaceaeg__uncultured	10	8	5	0	3	26	10	4	16	15	28	10
d__Bacteriap__Gemmatimonadotac__S0134_terrestrial_groupo__S0134_terrestrial_groupf__S0134_terrestrial_groupg__S0134_terrestrial_group	0	0	6	0	0	15	0	0	6	8	5	2
d__Bacteriap__Latescibacterotac__Latescibacteriao__Latescibacteralesf__Latescibacteraceaeg__Candidatus_Latescibacter	0	0	0	0	0	4	0	4	0	2	0	5
d__Bacteriap__Proteobacteriac__Alphaproteobacteriao__Micropepsalesf__Micropepsaceaeg__Micropepsaceae	0	37	0	0	4	18	8	0	17	16	0	0
d__Bacteriap__Proteobacteriac__Gammaproteobacteriao__Burkholderialesf__Rhodocyclaceaeg__Zoogloea	0	0	0	0	0	0	0	0	0	0	6	0
d__Bacteriap__Firmicutesc__Desulfotomaculiao__Desulfotomaculalesf__Desulfallas-Sporotomaculumg__Desulfallas-Sporotomaculum	0	0	0	0	0	0	0	0	0	0	0	8
d__Bacteriap__SAR324_clade(Marine_group_B)c__SAR324_clade(Marine_group_B)o__SAR324_clade(Marine_group_B)f__SAR324_clade(Marine_group_B)g__SAR324_clade(Marine_group_B)	0	0	4	0	0	3	9	0	0	0	2	2

Bacteria	R1__ ASR 5i	R2__ ASR 5i	R3__ ASR 5i	R4__ ASR 5i	R5__ ASR 5i	R6__ ASR 5i	R1 _W T	R2 _W T	R3 _W T	R4 _W T	R5 _W T	R6 _W T
d__Bacteriap__Proteobacteriac__Alphaproteobacteriao__Rhizobialesf__Methyloligellaceaeg__Methyloceanibacter	17	0	0	10	0	40	0	5	18	0	0	0
d__Bacteriap__Actinobacteriotac__Actinobacteriao__Streptosporangialesf__Thermomonosporaceaeg__Actinoallomurus	0	0	0	0	5	11	3	0	0	3	4	0
d__Bacteriap__Proteobacteriac__Alphaproteobacteriao__Caulobacteralesf__Caulobacteraceaeg__Brevundimonas	0	0	0	0	0	0	0	0	3	0	0	0
d__Bacteriap__Chloroflexic__Ktedonobacteriao__Ktedonobacteralesf__Ktedonobacteraceaeg__Thermosporothrix	0	0	0	0	0	0	0	7	0	0	0	0
d__Bacteriap__Firmicutesc__Bacillio__Bacillalesf__Planococcaceaeg__Lysinibacillus	2	0	0	0	0	0	0	0	0	2	10	0
d__Bacteriap__Bacteroidotac__Bacteroidiao__Chitinophagalesf__Chitinophagaceaeg__Heliimonas	0	28	14	0	26	84	0	5	0	0	14	7
d__Bacteriap__Firmicutesc__Clostridiao__Peptostreptococcales-Tissierellalesf__Peptostreptococcaceaeg__Paraclostridium	36	4	39	12	34	87	93	0	56	61	77	26
d__Bacteriap__Bacteroidotac__Bacteroidiao__Sphingobacteriales____	17	30	23	0	8	26	9	20	49	15	18	11
d__Bacteriap__Elusimicrobiotac__Lineage_IIao__Lineage_IIaf__Lineage_IIag__Lineage_IIa	0	0	0	0	0	21	0	6	0	10	0	12
d__Bacteriap__Spirochaetota________	0	0	2	0	0	5	0	3	0	0	3	0
d__Bacteriap__Firmicutesc__Clostridiao__Peptostreptococcales-Tissierellalesf__Anaerovoracaceaeg__Anaerovorax	27	59	30	37	68	36	46	52	133	39	127	71
d__Bacteriap__Planctomycetotac__Phycisphaeraeo__Tepidisphaeralesf__Tepidisphaeraceaeg__Tepidisphaera	0	0	0	0	0	0	0	0	0	0	2	0
d__Bacteriap__Fibrobacterotac__Chitinivibrioniao__unculturedf__unculturedg__uncultured	3	0	0	0	0	0	0	2	0	0	0	0
d__Bacteriap__Proteobacteriac__Gammaproteobacteriao__Burkholderialesf__Nitrosomonadaceaeg__Nitrosospira	0	0	0	0	0	0	7	0	0	0	0	0
d__Bacteriap__Desulfobacterotac__Desulfobulbiao__Desulfobulbalesf__Desulfurivibrionaceaeg__MSBL7	0	0	0	3	0	3	0	0	0	11	0	3
d__Bacteriap__Bacteroidotac__Bacteroidiao__Chitinophagalesf__Chitinophagaceaeg__Chitinophaga	0	0	5	0	0	0	0	0	0	0	3	0
d__Bacteriap__Patescibacteriac__Saccharimonadiao__Saccharimonadalesf__LWQ8g__LWQ8	31	92	70	15	0	315	8	0	4	10	47	0
d__Bacteriap__Chloroflexic__Ktedonobacteriao__Ktedonobacteralesf__Ktedonobacteraceaeg__1921-3	0	0	0	0	0	2	0	8	0	0	0	2
d__Bacteriap__Firmicutesc__Clostridiao__Clostridialesf__Clostridiaceaeg__Clostridium_sensu_stricto_6	0	2	0	0	0	9	14	0	8	20	14	5
d__Bacteriap__Proteobacteriac__Alphaproteobacteriao__Rhizobialesf__Rhizobiales_Incertae_Sedisg__uncultured	17	100	128	27	17	325	45	0	59	13	81	0

Bacteria	R1__ ASR 5i	R2__ ASR 5i	R3__ ASR 5i	R4__ ASR 5i	R5__ ASR 5i	R6__ ASR 5i	R1 _W T	R2 _W T	R3 _W T	R4 _W T	R5 _W T	R6 _W T
d__Bacteriap__Patescibacteriac__Parcubacteriao__Candidatus_Wolfebacteriaf__Candidatus_Wolfebacteriag__Candidatus_Wolfebacteria	9	2	10	3	8	20	9	13	12	17	12	12
d__Bacteriap__Desulfobacterotac__Desulfobulbiao__Desulfobulbalesf__Desulfobulbaceae__	0	0	0	0	0	0	0	3	0	0	0	0
d__Bacteriap__Actinobacteriotac__Actinobacteriao__Streptosporangialesf__Streptosporangiaceae__	2	0	0	0	0	0	0	0	0	0	6	0
d__Bacteriap__Actinobacteriotac__Actinobacteriao__Corynebacterialesf__Corynebacteriaceaeg__Turicella	0	0	0	0	0	0	0	0	0	2	0	0
d__Bacteriap__Actinobacteriotac__Acidimicrobiiao__Microtrichalesf__Ilumatobacteraceaeg__uncultured	0	0	0	0	2	0	0	0	2	0	3	0
d__Bacteriap__Desulfobacterotac__Syntrophiao__Syntrophalesf__Syntrophaceaeg__Syntrophus	0	3	2	0	0	0	0	3	10	16	5	0
d__Bacteriap__Proteobacteriac__Alphaproteobacteriao__Holosporalesf__Holosporaceaeg__uncultured	0	0	0	0	4	10	0	0	0	0	10	0
d__Bacteriap__Proteobacteriac__Gammaproteobacteriao__Acidiferrobacteralesf__Acidiferrobacteraceaeg__Sulfurifustis	0	0	3	0	0	0	0	0	0	12	0	0
d__Bacteriap__Verrucomicrobiotac__Verrucomicrobiaeo__Chthoniobacteralesf__Chthoniobacteraceaeg__Chthoniobacter	3	4	8	6	3	0	0	4	7	0	14	0
d__Bacteriap__Actinobacteriotac__Actinobacteriao__Pseudonocardialesf__Pseudonocardiaceaeg__Pseudonocardia	8	0	0	2	3	8	0	0	6	0	0	0
d__Bacteriap__Proteobacteriac__Alphaproteobacteriao__Rhizobialesf__Rhodomicrobiaceaeg__Rhodomicrobium	9	0	0	15	28	34	20	0	34	12	53	5
d__Bacteriap__Bdellovibrionota________	0	0	3	0	0	2	0	0	0	0	0	2
d__Bacteriap__Firmicutesc__Clostridiao__Eubacterialesf__Anaerofustaceaeg__Anaerofustis	0	0	0	0	0	0	0	0	4	0	2	0
d__Bacteriap__Bacteroidotac__Bacteroidiao__Chitinophagalesf__unculturedg__uncultured	0	0	0	5	4	12	16	0	4	9	16	8
d__Bacteriap__Patescibacteriac__Saccharimonadiao__Saccharimonadalesf__Saccharimonadaceaeg__TM7a	9	0	27	5	4	10	0	0	4	7	17	0
d__Bacteriap__Proteobacteriac__Gammaproteobacteriao__Burkholderialesf__Comamonadaceaeg__Thiomonas	0	53	9	10	0	0	9	8	4	7	4	0
d__Bacteriap__Proteobacteriac__Gammaproteobacteriao__Burkholderialesf__Burkholderiaceaeg__Limnobacter	0	0	0	0	0	0	0	0	0	4	0	0
d__Bacteriap__Proteobacteriac__Alphaproteobacteriao__Rickettsialesf__Rickettsiaceaeg__Rickettsia	0	0	0	0	0	0	0	0	0	0	0	4
d__Bacteriap__Methylomirabilotac__Methylomirabiliao__Methylomirabilalesf__Methylomirabilaceaeg__Sh765B-TzT-35	0	0	0	0	0	0	0	2	0	0	0	6
d__Bacteriap__Myxococcotac__Myxococciao__Myxococcalesf__Myxococcaceaeg__Myxococcus	0	0	25	0	0	0	0	0	0	0	9	0

Bacteria	R1__ ASR 5i	R2__ ASR 5i	R3__ ASR 5i	R4__ ASR 5i	R5__ ASR 5i	R6__ ASR 5i	R1 _W T	R2 _W T	R3 _W T	R4 _W T	R5 _W T	R6 _W T
d__Bacteriap__Elusimicrobiotac__Lineage_IIbo__Lineage_IIbf__Lineage_IIbg__Lineage_IIb	0	0	0	0	4	5	0	0	0	0	3	0
d__Bacteriap__Chloroflexic__Dehalococcoidiao__vadinBA26f__vadinBA26g__vadinBA26	0	0	0	0	0	0	0	0	0	0	0	2
d__Bacteriap__Actinobacteriotac__Actinobacteriao__Propionibacterialesf__Nocardioidaceaeg__Nocardioides	0	0	0	0	0	0	0	0	0	4	2	0
d__Bacteriap__Firmicutesc__Clostridiao__Lachnospiralesf__Lachnospiraceaeg__Anaerosporobacter	0	0	0	0	0	0	0	0	0	0	0	9
d__Bacteriap__Chloroflexic__Anaerolineaeo__ADurb,Bin180f__ADurb,Bin180g__ADurb,Bin180	0	0	0	0	0	0	0	0	0	6	3	0
d__Bacteriap__Patescibacteriac__Parcubacteriao__Candidatus_Azambacteriaf__Candidatus_Azambacteriag__Candidatus_Azambacteria	0	0	0	0	0	0	0	0	0	2	0	0
d__Bacteriap__Firmicutesc__Bacillio__Staphylococcalesf__Staphylococcaceaeg__Staphylococcus	0	0	0	3	5	0	0	0	0	5	0	0
d__Bacteriap__Proteobacteriac__Gammaproteobacteriao__Enterobacteralesf__Enterobacteriaceaeg__Escherichia-Shigella	0	3	6	20	0	7	27	0	0	7	5	10
d__Bacteriap__Actinobacteriotac__Actinobacteriao__Propionibacterialesf__Propionibacteriaceae__	0	0	3	3	6	0	5	4	16	0	0	0
d__Bacteriap__Firmicutesc__Symbiobacteriiao__Symbiobacterialesf__Symbiobacteraceaeg__uncultured	0	0	0	0	0	0	0	0	0	2	0	3
d__Bacteriap__Methylomirabilotac__Methylomirabiliao__Methylomirabilalesf__Methylomirabilaceaeg__Z114MB74	0	0	0	0	0	0	0	0	0	3	0	0
d__Bacteriap__Chloroflexic__Anaerolineaeo__Anaerolinealesf__Anaerolineaceaeg__uncultured	18	9	8	7	2	24	2	13	3	5	12	25
d__Bacteriap__Planctomycetotac__Planctomyceteso__Pirellulalesf__Pirellulaceaeg__uncultured	0	0	0	0	2	0	0	0	0	4	5	3
d__Bacteriap__Firmicutesc__Clostridiao__Clostridialesf__Clostridiaceaeg__uncultured	0	7	11	0	0	0	7	0	18	0	11	11
d__Bacteriap__Myxococcotac__Polyangiao__Polyangialesf__Polyangiaceaeg__Labilithrix	0	0	0	0	0	0	0	0	0	16	0	0
d__Bacteriap__Proteobacteriac__Gammaproteobacteriao__R7C24f__R7C24g__R7C24	0	0	0	0	0	19	0	0	0	0	4	0
d__Archaeap__Nanoarchaeotac__Nanoarchaeiao__Woesearchaealesf__Woesearchaealesg__Woesearchaeales	7	0	0	2	0	6	3	8	2	5	0	3
d__Bacteriap__Proteobacteriac__Gammaproteobacteriao__Burkholderialesf__Gallionellaceaeg__Sideroxydans	0	31	32	20	4	46	32	57	31	45	25	28
d__Bacteriap__GAL15c__GAL15o__GAL15f__GAL15g__GAL15	5	0	0	0	3	0	0	4	0	0	0	0
d__Bacteriap__Latescibacterotac__Latescibacteriao__Latescibacteralesf__Latescibacteraceae__	0	0	0	0	0	0	0	2	0	0	0	0

Bacteria	R1__ ASR 5i	R2__ ASR 5i	R3__ ASR 5i	R4__ ASR 5i	R5__ ASR 5i	R6__ ASR 5i	R1 _W T	R2 _W T	R3 _W T	R4 _W T	R5 _W T	R6 _W T
d__Bacteriap__Patescibacteriac__ABY1o__Candidatus_Magasanikbacteriaf__Candidatus_Magasanikbacteriag__Candidatus_Magasanikbacteria	0	0	0	0	0	2	0	0	0	0	0	2
d__Bacteriap__Desulfobacterotac__Desulfobulbiao__Desulfobulbalesf__Desulfocapsaceaeg__[Desulfobacterium]_catecholicum_group	0	8	18	22	4	66	18	17	0	0	0	30
d__Bacteriap__Firmicutesc__Bacillio__Erysipelotrichalesf__Erysipelotrichaceaeg__Turicibacter	0	0	0	0	0	3	0	0	8	0	0	0
d__Bacteriap__Bacteroidotac__Bacteroidiao__Chitinophagalesf__Chitinophagaceaeg__Flavisolibacter	14	6	0	0	0	132	0	0	10	0	0	0
d__Bacteriap__Acidobacteriotac__Blastocatelliao__11-24f__11-24g__11-24	0	0	0	0	0	0	0	0	0	0	0	2
d__Bacteriap__Firmicutesc__Clostridiao__Peptostreptococcales-Tissierellales____	0	0	0	0	0	0	0	0	0	0	0	4
d__Bacteriap__Cyanobacteriac__Vampirivibrioniao__Vampirovibrionalesf__Vampirovibrionalesg__Vampirovibrionales	0	0	0	0	0	0	0	0	0	3	0	0
d__Bacteriap__Proteobacteriac__Alphaproteobacteriao__Rhizobialesf__Pleomorphomonadaceaeg__uncultured	9	271	64	13	13	15	0	12	0	295	0	0
d__Bacteriap__Proteobacteriac__Alphaproteobacteriao__Paracaedibacteralesf__Paracaedibacteraceaeg__Candidatus_Paracaedibacter	0	0	2	2	3	8	5	4	0	0	6	6
d__Bacteriap__Myxococcotac__Polyangiao__Blfdi19f__Blfdi19g__Blfdi19	0	0	0	0	0	0	0	0	2	0	0	0
d__Archaeap__Nanoarchaeotac__Nanoarchaeiao__Woesearchaealesf__GW2011_GWC1_47_15g__GW2011_GWC1_47_15	0	0	0	0	3	5	0	10	4	12	4	3
d__Bacteriap__Firmicutesc__Bacillio__Bacillalesf__Planococcaceae__	0	2	2	3	0	2	0	0	0	0	0	4
d__Bacteriap__Firmicutesc__Bacillio__Entomoplasmatalesf__type_IIIg__type_III	0	0	0	0	0	0	3	0	0	0	0	0
d__Bacteriap__Patescibacteriac__Microgenomatiao__Candidatus_Shapirobacteriaf__Candidatus_Shapirobacteriag__Candidatus_Shapirobacteria	0	0	5	0	0	4	0	0	0	3	9	0
d__Bacteriap__Bacteroidotac__Bacteroidiao__Cytophagalesf__Microscillaceae__	0	0	0	0	0	2	0	0	3	5	0	0
d__Bacteriap__Firmicutesc__Negativicuteso__unculturedf__unculturedg__uncultured	5	7	7	0	5	17	7	6	9	8	22	10
d__Bacteriap__Acidobacteriotac__Thermoanaerobaculiao__Thermoanaerobaculalesf__Thermoanaerobaculaceaeg__Subgroup_10	0	0	0	0	0	0	2	0	0	0	4	0
d__Bacteriap__Proteobacteriac__Gammaproteobacteriao__Diplorickettsialesf__Diplorickettsiaceaeg__Diplorickettsia	0	0	0	0	0	0	0	0	0	0	2	0
d__Bacteriap__Firmicutesc__Clostridiao__Lachnospirales____	0	0	0	0	4	3	0	0	2	0	0	0
d__Bacteriap__Firmicutesc__Moorelliao__Desulfitibacteralesf__Desulfitibacteraceaeg__Desulfitibacter	0	0	0	0	0	0	0	0	0	7	0	0
d__Bacteriap__Firmicutesc__Syntrophomonadiao__Syntrophomonadalesf__Syntrophomonadaceae__	0	0	8	0	0	0	0	0	12	0	0	0

Bacteria	R1__ASR5i	R2__ASR5i	R3__ASR5i	R4__ASR5i	R5__ASR5i	R6__ASR5i	R1_WT	R2_WT	R3_WT	R4_WT	R5_WT	R6_WT
d__Bacteriap__Firmicutesc__BRH-c20ao__BRH-c20af__BRH-c20ag__BRH-c20a	3	0	0	0	0	0	0	0	0	0	0	2
d__Bacteriap__Dependentiaec__Babeliaeo__Babelialesf__Babelialesg__Babeliales	0	2	0	0	3	0	0	0	0	2	2	0
d__Archaeap__Halobacterotac__Methanosarciniao__Methanosarcinialesf__Methanoperedenaceaeg__Candidatus_Methanoperedens	0	0	0	0	0	0	3	0	0	4	0	0
d__Bacteriap__Firmicutesc__Clostridiao__Oscillospiralesf__Ruminococcaceaeg__uncultured	0	2	0	0	6	0	0	0	0	3	0	10
d__Bacteriap__Firmicutesc__Clostridiao__Oscillospiralesf__Oscillospiraceaeg__NK4A214_group	0	10	0	0	15	0	0	0	16	0	0	0
d__Bacteriap__Proteobacteriac__Gammaproteobacteriao__Methylococcalesf__Methylococcaceae__	0	0	5	0	0	0	0	0	0	0	0	3
d__Bacteriap__Firmicutesc__Clostridiao__Clostridiaf__Hungateiclostridiaceaeg__Anaerobacterium	0	106	0	18	0	41	49	0	27	0	0	0
d__Bacteriap__Proteobacteriac__Gammaproteobacteriao__Burkholderialesf__Burkholderiaceaeg__Pandoraea	0	0	0	19	15	23	32	6	0	0	11	0
d__Bacteriap__Proteobacteriac__Gammaproteobacteriao__Pseudomonadalesf__Moraxellaceaeg__Acinetobacter	0	0	0	0	0	0	0	0	0	0	0	7
d__Bacteriap__Proteobacteriac__Alphaproteobacteriao__Rhizobialesf__Pleomorphomonadaceaeg__Pleomorphomonas	0	0	73	0	0	127	0	0	33	0	0	50
d__Bacteriap__Proteobacteriac__Gammaproteobacteriao__Burkholderialesf__Nitrosomonadaceaeg__mle1-7	3	0	4	0	0	5	0	0	0	0	3	0
d__Archaeap__Halobacterotac__Methanosarciniao__Methanosarcinialesf__Methanosaetaceaeg__Methanosaeta	0	0	2	0	0	0	0	3	0	2	0	0
d__Bacteriap__Proteobacteriac__Gammaproteobacteriao__Xanthomonadalesf__Xanthomonadaceaeg__Stenotrophomonas	0	2	0	0	0	0	0	0	0	0	7	0
d__Bacteriap__Proteobacteriac__Gammaproteobacteriao__Burkholderialesf__Oxalobacteraceaeg__Massilia	0	0	0	0	11	0	0	0	0	0	0	13
d__Bacteriap__Planctomycetotac__Planctomyceteso__Isosphaeralesf__Isosphaeraceaeg__Aquisphaera	0	0	9	0	0	0	13	0	8	0	11	0
d__Bacteriap__Chloroflexic__Ktedonobacteriao__Ktedonobacteralesf__Ktedonobacteraceaeg__1959-1	0	0	10	0	0	14	6	0	4	0	15	7
d__Bacteriap__Verrucomicrobiotac__Verrucomicrobiaeo__Pedosphaeralesf__Pedosphaeraceaeg__SH3-11	0	0	0	0	0	0	0	0	0	0	0	3
d__Bacteriap__Proteobacteriac__Gammaproteobacteriao__Burkholderialesf__Gallionellaceaeg__Gallionella	0	115	30	0	61	36	33	88	31	86	74	0
d__Bacteriap__Proteobacteriac__Gammaproteobacteriao__CCD24f__CCD24g__CCD24	0	0	0	0	0	0	0	0	0	4	4	0
d__Bacteriap__Bacteroidotac__Bacteroidiao__SM1A07f__SM1A07g__SM1A07	0	0	0	0	0	0	0	0	0	0	0	2

Bacteria	R1__ASR5i	R2__ASR5i	R3__ASR5i	R4__ASR5i	R5__ASR5i	R6__ASR5i	R1_WT	R2_WT	R3_WT	R4_WT	R5_WT	R6_WT
d__Bacteriap__Bacteroidotac__Bacteroidiao__Sphingobacterialesf__AKYH767g__AKYH767	0	0	0	0	0	3	0	0	0	2	0	0
d__Bacteriap__Firmicutesc__Bacillio__Lactobacillalesf__Lactobacillaceaeg__Lactobacillus	0	0	0	0	0	0	0	0	0	0	6	0
d__Bacteriap__Proteobacteriac__Gammaproteobacteriao__Enterobacterales____	0	0	0	0	0	0	0	0	7	0	0	0
d__Bacteriap__Proteobacteriac__Gammaproteobacteriao__CHAB-XI-27f__CHAB-XI-27g__CHAB-XI-27	0	0	0	0	0	0	0	0	0	0	2	0
d__Bacteriap__Chloroflexic__OLB14o__OLB14f__OLB14g__OLB14	4	3	0	0	3	6	9	2	9	4	0	3
d__Bacteriap__Firmicutesc__Bacillio__Brevibacillalesf__Brevibacillaceaeg__Brevibacillus	0	4	0	0	0	5	4	2	3	3	3	3
d__Archaeap__Halobacterotac__Methanomicrobiao__Methanomicrobialesf__Methanoregulaceaeg__Methanoregula	0	0	0	0	0	0	0	0	6	3	0	0
d__Bacteriap__Proteobacteriac__Gammaproteobacteriao__Burkholderialesf__Comamonadaceaeg__Curvibacter	0	0	0	0	0	0	0	0	0	0	30	0
d__Bacteriap__Planctomycetotac__Planctomyceteso__Gemmatalesf__Gemmataceaeg__Fimbriiglobus	0	0	0	0	0	0	0	0	0	3	0	0
d__Bacteriap__Bacteroidotac__Bacteroidiao__Sphingobacterialesf__Sphingobacteriaceaeg__Sphingobacterium	0	0	0	0	0	0	0	0	4	0	0	0
d__Bacteriap__Proteobacteriac__Gammaproteobacteriao__Xanthomonadalesf__Rhodanobacteraceaeg__Chujaibacter	30	77	66	54	20	124	73	34	66	38	76	85
d__Bacteriap__Firmicutesc__Bacillio__Bacillalesf__Sporolactobacillaceaeg__Pullulanibacillus	0	0	4	0	0	9	6	0	0	0	7	0
d__Bacteriap__Proteobacteriac__Gammaproteobacteriao__Steroidobacteralesf__Steroidobacteraceae__	0	0	0	0	0	0	0	0	0	2	0	0
d__Bacteriap__Myxococcotac__Polyangiao__Polyangialesf__Sandaracinaceaeg__Sandaracinus	0	0	0	0	0	0	0	4	0	0	2	0
d__Bacteriap__Firmicutesc__Clostridiao__Oscillospiralesf__Oscillospiraceaeg__Papillibacter	0	0	7	0	0	0	0	4	0	0	0	0
d__Bacteriap__Proteobacteriac__Alphaproteobacteriao__Rhizobialesf__unculturedg__uncultured	0	0	0	0	0	0	0	0	7	0	0	0
d__Bacteriap__Bdellovibrionotac__Oligoflexiao__Silvanigrellalesf__Silvanigrellaceaeg__Silvanigrella	0	0	0	0	0	0	0	0	0	3	0	0
d__Bacteriap__Firmicutesc__Symbiobacteriiao__Symbiobacteriales____	0	0	0	0	0	0	0	0	3	0	0	0
d__Bacteriap__Verrucomicrobiotac__Lentisphaeriao__Victivallalesf__Victivallalesg__Victivallales	0	2	0	0	0	0	0	3	4	0	0	0
d__Bacteriap__Actinobacteriotac__Actinobacteriao__Frankialesf__unculturedg__uncultured	22	0	0	5	0	0	53	11	0	0	0	0
d__Bacteriap__Chloroflexic__Dehalococcoidiao__GIF3f__GIF3g__GIF3	0	2	0	0	0	0	2	0	0	0	0	0

Bacteria	R1__ ASR 5i	R2__ ASR 5i	R3__ ASR 5i	R4__ ASR 5i	R5__ ASR 5i	R6__ ASR 5i	R1 _WT	R2 _WT	R3 _WT	R4 _WT	R5 _WT	R6 _WT
d__Bacteriap__Proteobacteriac__Gammaproteobacteriao__Burkholderialesf__Oxalobacteraceaeg__Paucimonas	0	0	0	0	0	0	0	0	4	0	0	0
d__Bacteriap__Bacteroidotac__Bacteroidiao__Cytophagalesf__Cytophagaceaeg__Sporocytophaga	0	0	0	0	0	0	4	0	0	0	0	0
d__Bacteriap__Proteobacteriac__Alphaproteobacteriao__Caulobacteralesf__Hyphomonadaceaeg__Hirschia	0	0	0	0	0	0	0	0	7	9	0	0
d__Bacteriap__Chloroflexic__Ktedonobacteriao__Ktedonobacteralesf__Ktedonobacteraceaeg__1921-2	0	0	0	0	0	0	0	0	4	0	0	0
d__Bacteriap__Acidobacteriotac__Acidobacteriaeo__Acidobacterialesf__Acidobacteriaceae_(Subgroup_1)g__Edaphobacter	0	0	18	0	0	0	0	0	0	0	42	2
d__Bacteriap__Proteobacteriac__Gammaproteobacteriao__Salinisphaeralesf__Solimonadaceaeg__Nevskia	0	3	5	0	0	10	2	0	0	20	0	0
d__Bacteriap__Acidobacteriotac__Acidobacteriaeo__Acidobacterialesf__Acidobacteriaceae_(Subgroup_1)g__Terriglobus	0	0	0	0	0	0	0	0	0	10	4	0
d__Bacteriap__Verrucomicrobiotac__Verrucomicrobiaeo__Chthoniobacteralesf__Terrimicrobiaceaeg__FukuN18_freshwater_group	3	21	22	0	6	61	0	6	5	0	0	9
d__Bacteriap__Firmicutesc__Bacillio__Bacillalesf__Planococcaceaeg__Rummeliibacillus	0	12	12	3	0	16	7	0	0	0	0	0
d__Bacteriap__Proteobacteriac__Alphaproteobacteriao__Parvibaculalesf__Parvibaculaceaeg__Parvibaculum	0	0	2	0	0	0	0	3	0	0	3	0
d__Bacteriap__Actinobacteriotac__Actinobacteriao__Micrococcalesf__Intrasporangiaceaeg__Terrabacter	28	0	0	0	0	0	0	0	0	0	19	0
d__Bacteriap__Proteobacteriac__Alphaproteobacteriao__Rhodobacteralesf__Rhodobacteraceaeg__Amaricoccus	0	0	0	0	0	0	0	0	0	0	5	0
d__Bacteriap__Firmicutesc__Clostridiao__unculturedf__unculturedg__uncultured	0	5	0	4	0	0	10	0	0	0	0	2
d__Bacteriap__Proteobacteriac__Alphaproteobacteriao__Rhizobialesf__Beijerinckiaceaeg__Methylocella	15	0	0	0	0	0	23	0	0	59	0	0
d__Bacteriap__Acidobacteriotac__Holophagaeo__Holophagalesf__Holophagaceaeg__Holophaga	0	0	3	0	0	0	0	0	0	0	3	0
d__Bacteriap__Desulfobacterotac__Desulfovibrioniao__Desulfovibrionalesf__Desulfovibrionaceae__	0	0	0	0	0	0	0	0	3	0	0	0
d__Bacteriap__Patescibacteriac__Gracilibacteriao__Candidatus_Peregrinibacteriaf__Candidatus_Peregrinibacteriag__Candidatus_Peregrinibacteria	0	0	0	0	0	0	0	0	2	0	0	0
d__Bacteriap__Proteobacteriac__Alphaproteobacteriao__Rhodobacteralesf__Rhodobacteraceaeg__Rubellimicrobium	0	0	0	0	0	0	2	0	0	0	0	0
d__Bacteriap__Proteobacteriac__Alphaproteobacteriao__Rhodobacteralesf__Rhodobacteraceae__	0	0	8	0	0	8	27	0	16	27	12	0

Bacteria	R1__ASR5i	R2__ASR5i	R3__ASR5i	R4__ASR5i	R5__ASR5i	R6__ASR5i	R1_WT	R2_WT	R3_WT	R4_WT	R5_WT	R6_WT
d__Bacteriap__Actinobacteriotac__Acidimicrobiiao__Microtrichalesf__Microtrichaceaeg__uncultured	0	0	0	0	0	0	0	0	0	5	0	0
d__Bacteriap__Actinobacteriotac__Actinobacteriao__Micromonosporalesf__Micromonosporaceaeg__Actinocatenispora	0	0	0	0	0	0	0	0	0	2	0	0
d__Bacteriap__Firmicutesc__Clostridiao__Oscillospiralesf__Ethanoligenenaceaeg__Ethanoligenenaceae	0	0	0	0	0	0	0	0	4	0	0	0
d__Bacteriap__Bacteroidotac__Bacteroidiao__Chitinophagalesf__Chitinophagaceaeg__Flavihumibacter	0	0	0	0	0	0	0	0	2	0	0	0
d__Bacteriap__Proteobacteriac__Gammaproteobacteriao__PLTA13f__PLTA13g__PLTA13	0	0	0	0	0	0	0	2	0	0	0	0
d__Archaeap__Halobacterotac__Methanomicrobiao__Methanomicrobialesf__Rice_Cluster_IIg__Rice_Cluster_II	0	0	0	0	0	0	5	0	0	0	0	0
d__Bacteriap__Proteobacteriac__Alphaproteobacteriao__Rhizobialesf__Beijerinckiaceaeg__Rhodoblastus	0	9	0	0	0	0	0	0	10	9	0	0
d__Bacteriap__Firmicutesc__Bacillio__Thermoactinomycetalesf__Thermoactinomycetaceaeg__Pasteuria	0	0	0	0	0	0	0	0	0	2	0	0
d__Bacteriap__Chloroflexic__Ktedonobacteriao__B10-SB3Af__B10-SB3Ag__B10-SB3A	4	0	0	0	0	4	6	0	0	0	0	0
d__Bacteriap__Proteobacteriac__Gammaproteobacteriao__Burkholderialesf__Nitrosomonadaceaeg__MND1	0	0	0	0	0	0	0	0	0	0	0	3
d__Bacteriap__Bacteroidotac__Bacteroidiao__Chitinophagalesf__Chitinophagaceaeg__Niastella	0	0	0	0	0	0	6	0	0	0	0	0
d__Bacteriap__Proteobacteriac__Gammaproteobacteriao__Burkholderialesf__Alcaligenaceaeg__Achromobacter	0	0	0	0	0	0	0	0	51	0	4	0
d__Bacteriap__Bacteroidotac__Bacteroidiao__Chitinophagalesf__Chitinophagaceaeg__Niabella	0	0	0	0	0	0	0	0	3	0	0	0
d__Bacteriap__Patescibacteriac__Parcubacteriao__Candidatus_Staskawiczbacteriaf__Candidatus_Staskawiczbacteriag__Candidatus_Staskawiczbacteria	0	0	0	0	0	0	0	3	0	0	0	0
d__Bacteriap__Patescibacteriac__Dojkabacteriao__Dojkabacteriaf__Dojkabacteriag__Dojkabacteria	0	0	0	0	0	0	0	0	3	0	0	0
d__Bacteriap__Bacteroidotac__Ignavibacteriao__Ignavibacterialesf__Ignavibacteriaceaeg__Ignavibacterium	0	0	0	0	0	0	0	0	11	0	21	0
d__Bacteriap__Proteobacteriac__Gammaproteobacteriao__Xanthomonadalesf__Rhodanobacteraceaeg__Dokdonella	0	0	5	7	0	0	0	0	0	0	27	9
d__Bacteriap__Proteobacteriac__Alphaproteobacteriao__Acetobacteralesf__Acetobacteraceaeg__Acetobacteraceae	0	0	16	0	0	0	0	0	13	0	0	12
d__Bacteriap__Patescibacteriac__Parcubacteriao__Candidatus_Liptonbacteriaf__Candidatus_Liptonbacteriag__Candidatus_Liptonbacteria	0	0	0	0	0	0	0	0	0	5	0	0

Bacteria	R1__ASR5i	R2__ASR5i	R3__ASR5i	R4__ASR5i	R5__ASR5i	R6__ASR5i	R1_WT	R2_WT	R3_WT	R4_WT	R5_WT	R6_WT
d__Archaeap__Thermoplasmatotac__Thermoplasmatao__Methanomassiliicoccalesf__Methanomassiliicoccaceaeg__Methanomassiliicoccus	0	7	0	0	0	2	0	3	5	0	0	0
d__Bacteriap__Bacteroidotac__Bacteroidiao__Bacteroidalesf__Williamwhitmaniaceaeg__Blvii28_wastewater-sludge_group	0	0	0	0	0	0	0	0	0	0	5	0
d__Bacteriap__Verrucomicrobiotac__Verrucomicrobiaeo__Methylacidiphilalesf__Methylacidiphilaceaeg__uncultured	0	0	0	0	3	0	0	0	0	0	0	4
d__Bacteriap__Proteobacteriac__Alphaproteobacteriao__Rhizobialesf__Amb-16S-1323g__Amb-16S-1323	0	0	0	0	0	0	8	0	0	0	0	0
d__Bacteriap__Desulfobacterotac__Desulfobulbiao__Desulfobulbales____	0	21	10	0	2	0	0	0	0	5	0	0
d__Bacteriap__Chloroflexic__Chloroflexiao__Thermomicrobialesf__Thermomicrobiaceaeg__Nitrolancea	0	0	0	0	0	0	0	0	3	0	0	0
d__Bacteriap__Actinobacteriotac__Actinobacteriao__Micromonosporalesf__Micromonosporaceaeg__Actinoplanes	0	0	0	0	0	0	0	0	12	0	0	0
d__Bacteriap__Firmicutesc__Clostridiao__Clostridia_vadinBB60_groupf__Clostridia_vadinBB60_groupg__Clostridia_vadinBB60_group	0	0	0	0	2	0	0	0	0	0	0	3
d__Bacteriap__Proteobacteriac__Gammaproteobacteriao__Burkholderialesf__Comamonadaceaeg__uncultured	9	0	0	0	0	0	18	15	0	99	26	0
d__Bacteriap__Proteobacteriac__Gammaproteobacteriao__Xanthomonadalesf__Xanthomonadaceaeg__Arenimonas	0	0	0	0	0	0	0	2	0	0	0	0
d__Bacteriap__Proteobacteriac__Alphaproteobacteriao__Rhizobialesf__Beijerinckiaceaeg__Methylocystis	45	137	333	0	149	513	114	0	41	0	94	34
d__Bacteriap__Proteobacteriac__Gammaproteobacteriao__Burkholderialesf__Comamonadaceaeg__Rhodoferax	0	0	0	0	14	0	0	0	0	0	0	6
d__Bacteriap__Proteobacteriac__Gammaproteobacteriao__Xanthomonadalesf__Rhodanobacteraceaeg__Mizugakiibacter	0	0	0	0	0	0	0	0	0	0	8	0
d__Bacteriap__Firmicutesc__Negativicuteso__Veillonellales-Selenomonadalesf__Sporomusaceaeg__Pelosinus	0	0	4	0	0	0	0	0	0	0	4	0
	21804	38466	38772	30479	31285	79558	29239	38054	56903	48349	52415	55830
